# Hormonal regulation of miRNA during mammary gland development

**DOI:** 10.1242/bio.060308

**Published:** 2024-06-10

**Authors:** Cameron Confuorti, Maritza Jaramillo, Isabelle Plante

**Affiliations:** INRS, Centre Armand-Frappier Santé Biotechnologie, 531 boul. des Prairies, Laval, QC, H7V 1B7, Canada

**Keywords:** Development, Hormones, Mammary gland, miRNA

## Abstract

The mammary gland is a unique organ as most of its development occurs after birth through stages of proliferation, differentiation and apoptosis that are tightly regulated by circulating hormones and growth factors. Throughout development, hormonal cues induce the regulation of different pathways, ultimately leading to differential transcription and expression of genes involved in this process, but also in the activation or inhibition of post-transcriptional mechanisms of regulation. However, the role of microRNAs (miRNAs) in the different phases of mammary gland remodeling is still poorly understood. The objectives of this study were to analyze the expression of miRNA in key stages of mammary gland development in mice and to determine whether it could be associated with hormonal variation between stages. To do so, miRNAs were isolated from mouse mammary glands at stages of adulthood, pregnancy, lactation and involution, and sequenced. Results showed that 490, 473, 419, and 460 miRNAs are detected in adult, pregnant, lactating and involuting mice, respectively, most of them being common to all four groups, and 58 unique to one stage. Most genes could be divided into six clusters of expression, including two encompassing the highest number of miRNA (clusters 1 and 3) and showing opposite profiles of expression, reaching a peak at adulthood and valley at lactation, or showing the lowest expression at adulthood and peaking at lactation. GO and KEGG analyses suggest that the miRNAs differentially expressed between stages influence the expression of targets associated with mammary gland homeostasis and hormone regulation. To further understand the links between miRNA expression and hormones involved in mammary gland development, miRNAs were then sequenced in breast cells exposed to estradiol, progesterone, prolactin and oxytocin. Four, 38, 24 and 66 miRNAs were associated with progesterone, estradiol, prolactin, and oxytocin exposure, respectively. Finally, when looking at miRNAs modulated by the hormones, differentially expressed during mammary gland development, and having a pattern of expression that could be correlated with the relative levels of hormones known to be found *in vivo,* 16 miRNAs were identified as likely regulated by circulating hormones. Overall, our study brings a better understanding of the regulation of miRNAs throughout mammary gland development and suggests that there is a relationship between their expression and the main hormones involved in mammary gland development. Future studies will examine this role more in detail.

## Teaser

**Summary:** By combining the patterns of expression of miRNA during mammary development and the miRNAs with expression shown to be regulated by hormones, we identified 16 mammary gland miRNAs whose expression is likely regulated by circulating hormones.

## INTRODUCTION

**Fig. 1.**
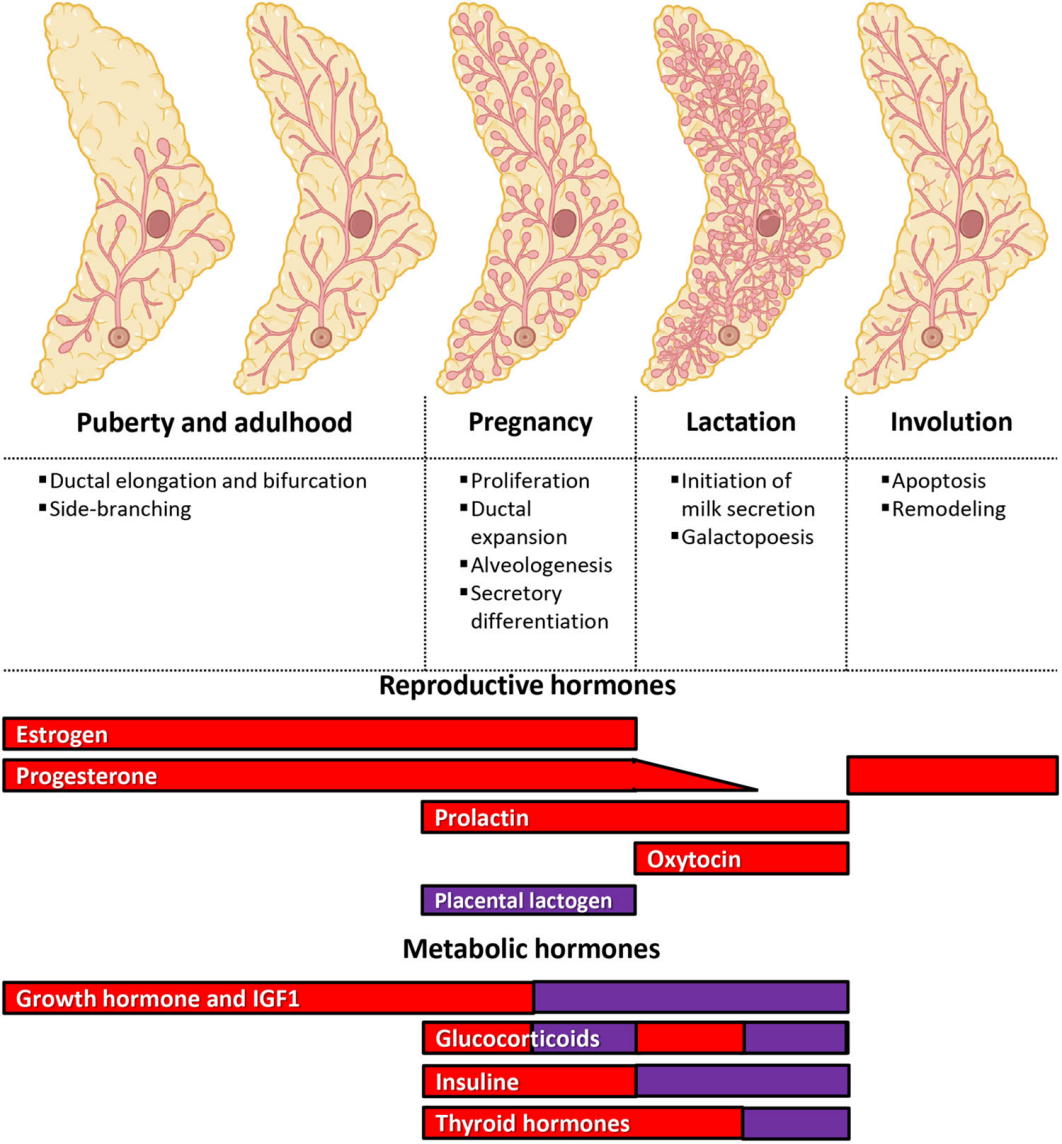
**Main hormones involved in mammary gland development.** The development of the mammary gland epithelium occurs mainly after birth. At puberty, a surge of hormones induces ductal elongation and bifurcation until the epithelium reaches the edge of the fat pad (i.e, stroma). During adulthood, the epithelium undergoes cycles of development and regression, following the menstrual cycles. A second wave of major remodeling will happen during pregnancy to form the alveoli that will produce milk during lactation. At weaning, the accumulation of milk will induce the involution of the epithelium to its pre-pregnancy stage. Reproductive and metabolic hormones have stage-specific roles during those processes, some having well-documented essential roles (red), and some having either complementary or less-documented roles (purple).

The mammary gland is a unique organ as most of its development occurs after birth through stages of proliferation, differentiation and apoptosis that are tightly regulated by circulating hormones and growth factors (Fig. 1). Studies mainly using transplants and genetically engineered mice have demonstrated that some of these hormones play a crucial role in mammary gland development and function in a stage-specific manner, while others play accessory or less-understood roles (Hannan et al., 2023). It is composed of two main compartments: the epithelium, a ramified tree-like structure that produces milk during lactation, and the stroma (also called fat pad), mainly composed of adipocytes, fibroblasts, immune cells and extracellular matrix (Hovey and Aimo, 2010; Sakakura et al., 2013). At birth, the rudimentary epithelial tree is composed of only a few branches that grow at the same pace as the body until puberty (Fig. 1). Then, a surge of hormones, particularly estrogens, induces the proliferation of epithelial cells at the tips of ducts, in structures named terminal-end buds (TEBs) (Hinck and Silberstein, 2005). Between puberty and adulthood, the epithelium continues to elongate and ramify, mainly under the combined action of estrogens and progesterone, until it reaches the edge of the fat pad (Hannan et al., 2023; McNally and Stein, 2017). The mammary gland then undergoes cycles of proliferation and apoptosis, in accordance with the hormonal variation induced by each menstrual cycle. Another intense phase of remodeling occurs during pregnancy with the rising levels of prolactin and progesterone (Berryhill et al., 2016; Brisken and Rajaram, 2006; Hannan et al., 2023; Lollivier et al., 2006; McNally and Stein, 2017; Rudolph et al., 2007), whereby the number of secondary and tertiary branches increases drastically, providing a ductal arbor for the formation of alveoli, the units that secrete milk. After parturition, the withdrawal of progesterone triggers secretory activation. Prolactin stimulates the production of milk, while oxytocin stimulates milk ejection (Hannan et al., 2023; McNally and Stein, 2017). At weaning, milk accumulation and change in hormonal stimulation, notably a decrease in prolactin and an increase in estrogens, induces involution of the mammary gland epithelium (Jena et al., 2019), whereby the mammary gland gradually returns to a structure similar to its pre-pregnancy stage. Of note, during all these phases, the stroma is also remodeled and actively contributes to the development of the epithelium by providing chemical, physical, and nutritional support, although mechanisms involved in this process are less known. Throughout these phases of remodeling, hormonal cues induce the regulation of different pathways, ultimately leading to differential transcription and expression of genes involved in this process, but also in the activation or inhibition of post-transcriptional mechanisms of regulation.

MicroRNAs (miRNAs) are 19 to 25 nucleotides long non-coding RNA molecules that post-transcriptionally regulate the expression of messenger RNA (mRNA), typically by binding to consensus sequences on their 3′-UTR (Cai et al., 2009). Over the last decades, thousands of miRNAs have been identified and classified into families, based on their seed sequences, i.e. primary determinant of recognition for miRNAs (Cai et al., 2009). An increasing number of articles are reporting the crucial role that miRNAs play in regulating the expression of genes within tissues, which regulates important tissue functions such as development, differentiation, proliferation, neoplastic transformation, and apoptosis (Cai et al., 2009). As a result, it is generally assumed that high levels of a specific miRNA within a cell or a tissue likely reflect a regulatory role, and that increased expression upon change in conditions is likely to have a biological impact on that tissue (Kilikevicius et al., 2022). Thus, evaluating the expression of miRNA during different stages of mammary gland development can bring important insights on their stage-specific role, and also on the impact of hormones on their regulation or on their role in breast cancer.

Compared to increasing literature reporting the differential expression of specific miRNAs in breast cancer tissues compared to normal tissues, only a few studies have evaluated the role of miRNA in mammary gland development. Using a bead-based flow-cytometric microarray platform, 102 miRNAs were detected in mouse mammary gland tissues from 18 different time points (Avril-Sassen et al., 2009). The expression of miRNA was found to follow seven clusters of expression, and the mean miRNA expression was lower at lactation (Avril-Sassen et al., 2009). Although this study provided an initial screen of miRNA expression in the mammary gland, it did not allow for a global-scale analysis of the miRNAome. Similarly, the expression of miRNA was analyzed in breast tissues from human, cow, goat and other animals but mainly during lactation (Bockmeyer et al., 2011; Dysin et al., 2021; Galio et al., 2013; Ji et al., 2012; Le Guillou et al., 2014; Li et al., 2012; Liu et al., 2004; Nagaoka et al., 2013; Wang et al., 2021; Xuan et al., 2020). Other studies have focused on specific miRNA or a small number of miRNAs (Feuermann et al., 2012; Roth and Moorehead, 2021; Ucar et al., 2010). Interestingly, in a recent literature review, 32 miRNAs were identified as implicated in both mammary gland development and breast cancer development across species, and were associated with specific stages of development or tumorigenic processes (Wu et al., 2022).

Given the crucial role of miRNA in the development and function of tissues, a deeper analysis of miRNA expression during mammary gland development is required to further understand their role. In addition, although the development of the mammary gland is tightly orchestrated by hormones, little is known regarding the relationship between miRNA and hormones during mammary gland development. The objectives of this study were to analyze transcriptome-wide changes in the expression of miRNAs in key stages of mammary gland development in mice and to determine whether regulation of specific miRNA subsets could be associated with hormonal variation between stages.

## RESULTS

### miRNA expression follows six clusters of expression during mammary gland development

**Fig. 2.**
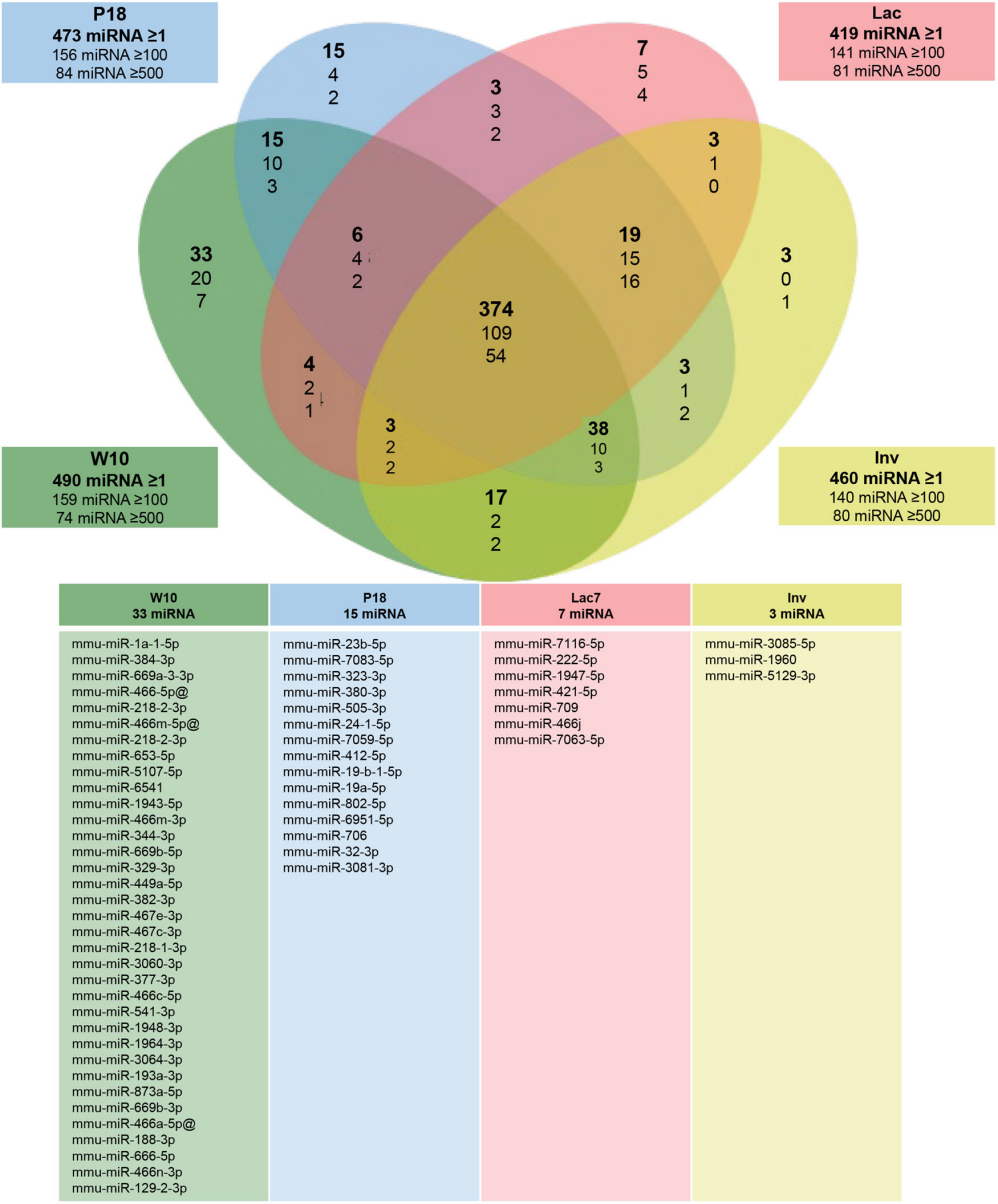
**miRNA expressed at 10 weeks of age (W10), day 18 of gestation (P18), day 7 of lactation (Lac7) and day 3 of involution (Inv3) in the mammary gland of mice.** Small RNA were extracted from mammary glands using the mirVana™ miRNA Isolation kit (ThermoFisher) and sequenced (Illumina NovaSeq6000). Six animals were used per group (*N*=6). Between 74 and 490 miRNAs were identified per group, depending of the threshold of average reads per million per group (>1, upper number; >100 middle number; >500 lower number, within the text boxes). The table lists the miRNA that are unique for each stage.

**Fig. 3.**
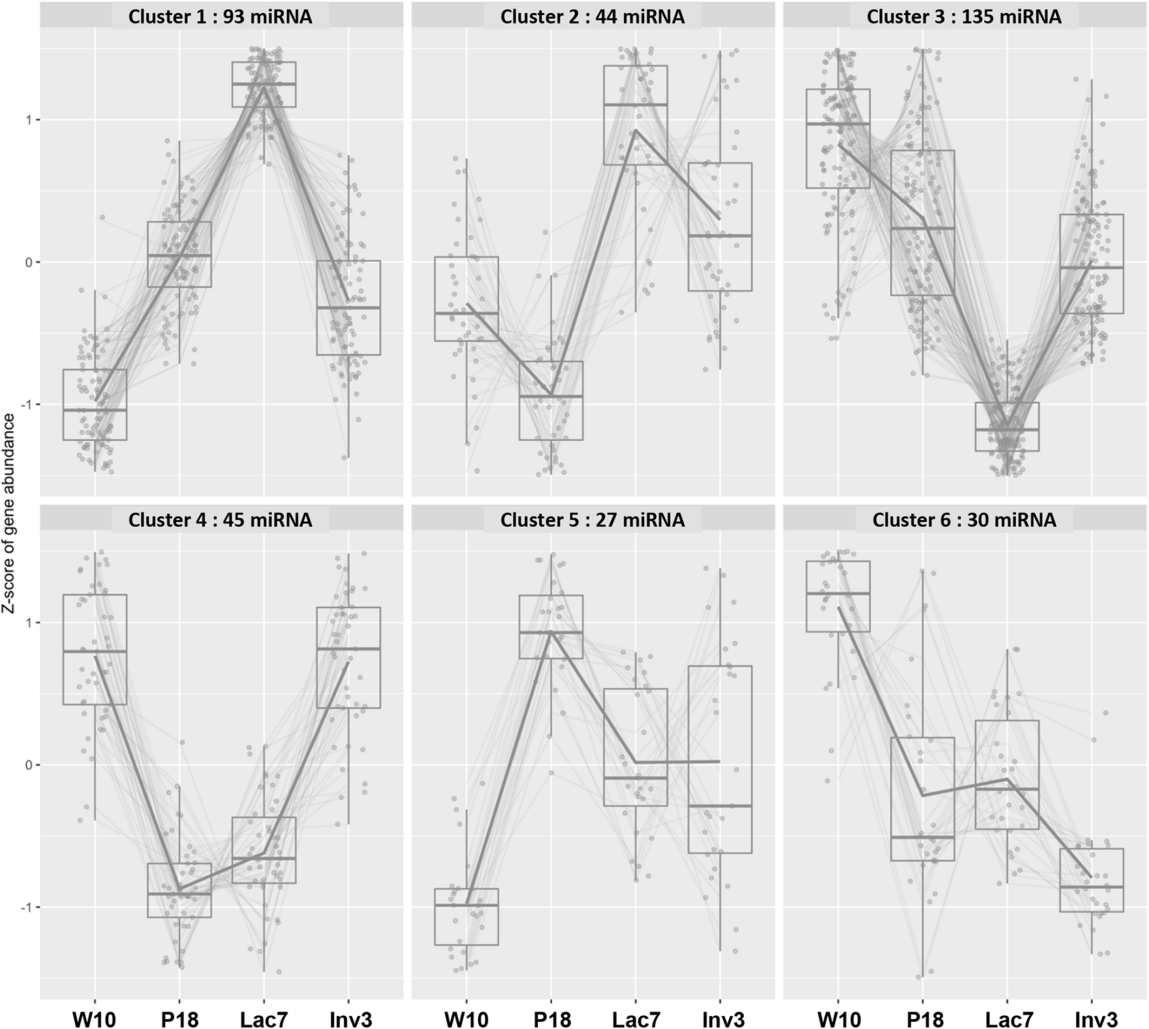
**miRNA expressed in the mammary gland can be clustered in six patterns of expression.** Using bigPint library (Rutter and Cook, 2020; Stephens, 2017) six clusters of expression were identified, comprising 93, 44, 135, 45, 27 and 30 miRNA, respectively.

miRNAs were extracted and sequenced from mammary glands of adult, pregnant, lactating and involuting mice (Fig. 2). Results showed that 490, 473, 419 and 460 miRNAs are detected in adult, pregnant, lactating and involuting mice (Tables S1-S4) respectively, including 374, 66 and 45 expressed at four (all), three and two stages, respectively, and 58 unique to one stage (Fig. 2 and Table S5). When analyzing the expression of individual miRNAs between the stages, most genes could be divided in six clusters of expression (Fig. 3 and Table S6). Interestingly, the two clusters containing the highest number of miRNA (clusters 1 and 3) showed opposite profiles of expression. Cluster 1 showed an increase in miRNAs expression between adult and lactation, followed by a decrease at involution, while cluster 3 showed a decrease in miRNAs expression between adult and lactation, followed by an increase at involution (Fig. 3). Cluster 4 also showed a decreased in miRNA expression specifically during pregnancy and lactation (Fig. 3).

### miRNAs vary in expression between different stages of mammary gland development

**Fig. 4.**
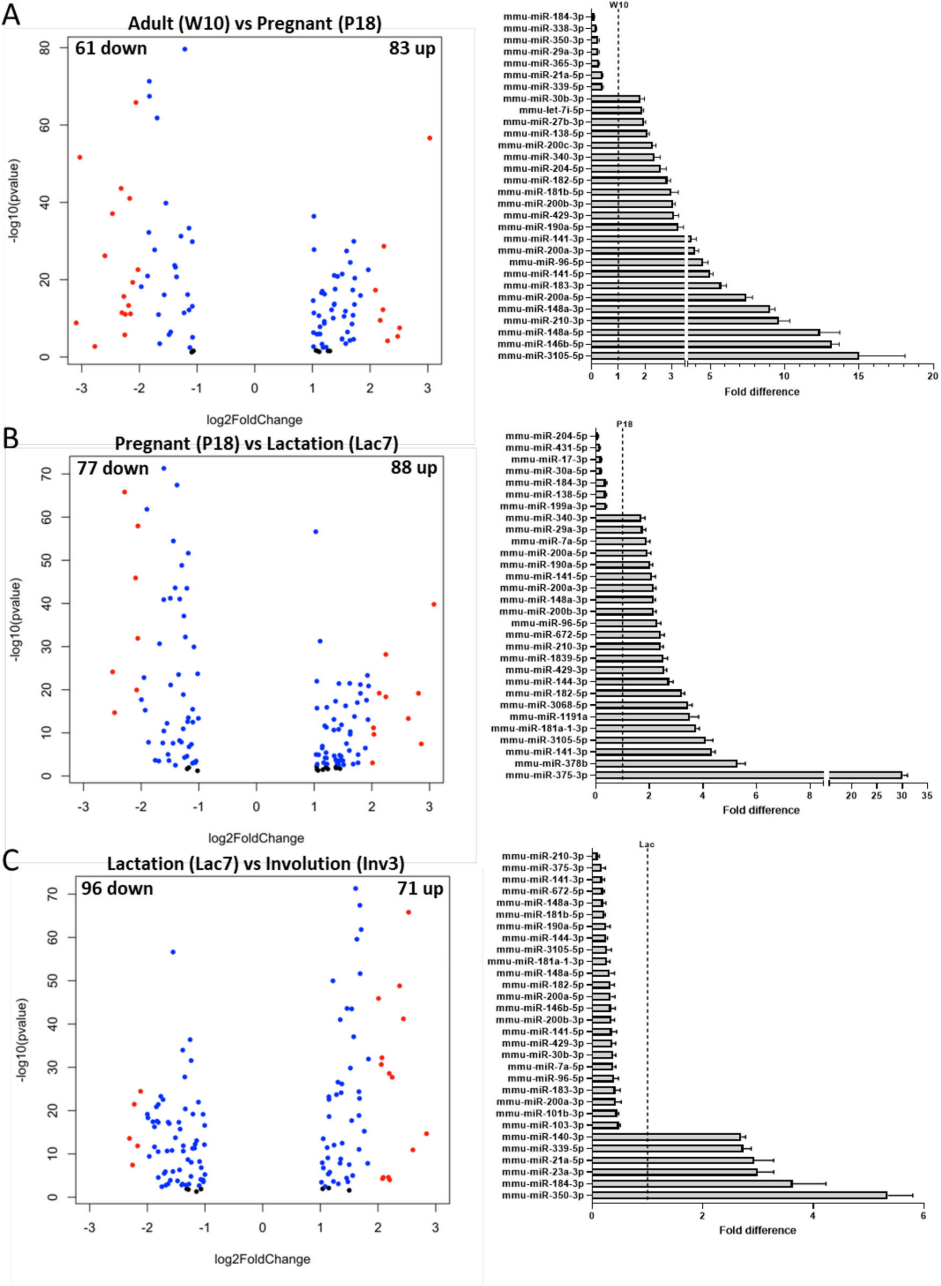
**miRNA differentially expressed between two consecutive stages of mammary gland development.** miRNA differentially expressed between (A) adult and pregnant, (B) pregnant and lactating, and (C) lactating and involuting mice were identified using the *DESeq2/1.26.0* application (Log 2 fold change cutoff=1; FDR cutoff: 1). The Volcano plots (left) show up- and downregulated miRNA for each comparison; the 30 miRNA that showed the biggest fold change are showed on the right. On volcano plots, red dots showed miRNA with Log 2 fold change (- or +) ≥2, while blue dots represent miRNA with Log 2 fold change (- or +) between 1 and 2. Black dots represent miRNA with *P* values between 0.05 and 0.001.

We next asked more precisely how miRNA expression fluctuates during mammary gland development by comparing consecutive stages of development. To do so, we identified miRNA differentially expressed in mammary glands from adult versus pregnant mice (Fig. 4A and Table S7), between pregnant and lactating mice (Fig. 4B and Table S8) and between lactating and involuting mice (Fig. 4C and Table S9). 144, 165 and 167 miRNAs were differentially expressed between adult and pregnant glands, between pregnant and lactating glands, and between lactating and involuting glands, respectively (Fig. 4 and Tables S7-S9). For the first two comparisons, about 55% of those miRNAs were upregulated, and 23 out of the 30 miRNAs that showed the highest fold change were also upregulated (Fig. 4A, B). However, an opposite trend was observed between lactating and involuting glands, as 57% of total differentially expressed miRNAs, and 24 out of the 30 miRNAs with the highest fold change, were downregulated (Fig. 4C and Table S9).

### Differentially expressed miRNAs are predicted to target various biological processes and pathways

**Fig. 5.**
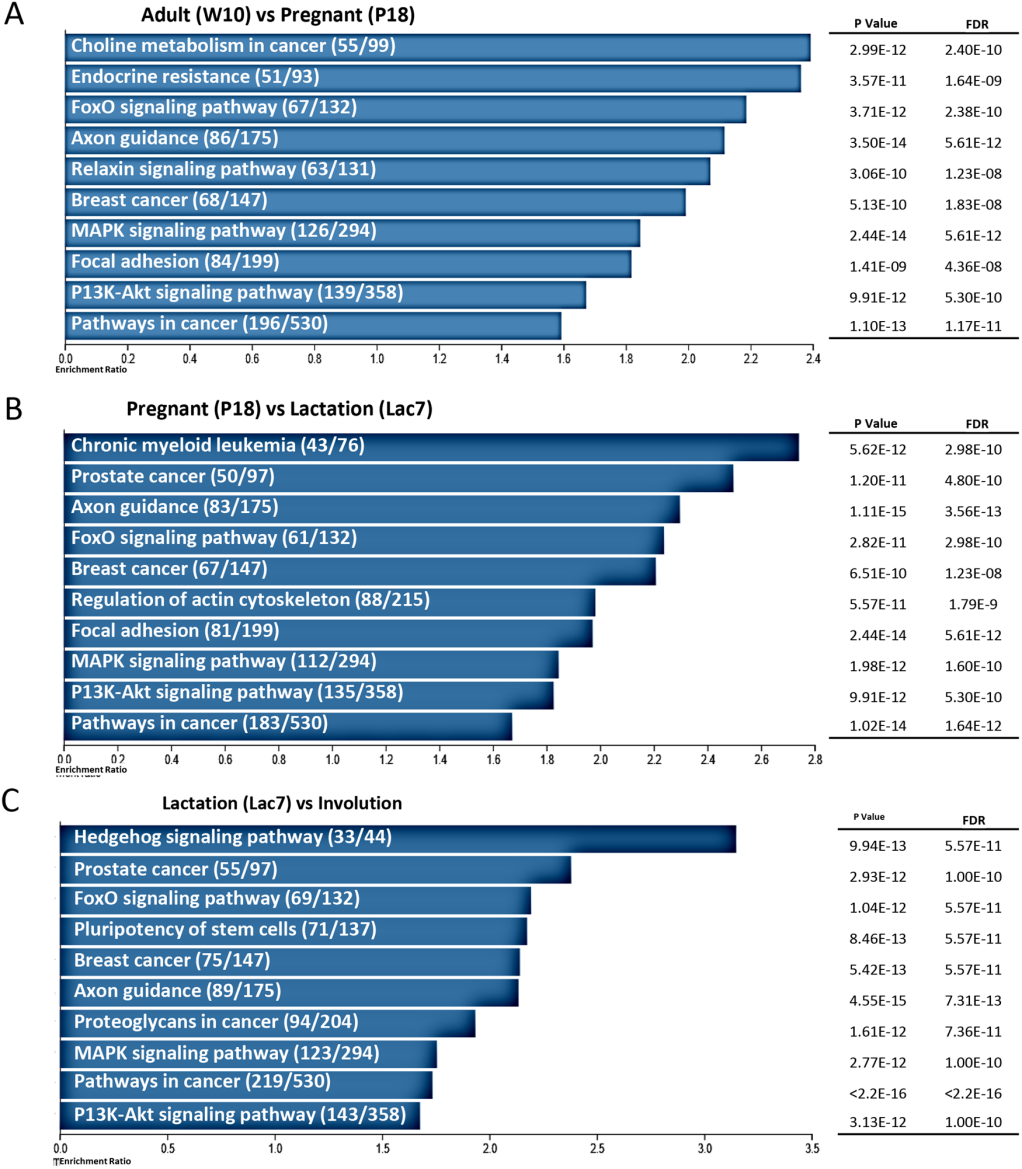
**Predicted biological processes and pathways targeted by the differentially expressed miRNAs.** The list of predicted mRNA target was generated for each set of differentially expressed miRNA: (A) adult versus pregnant; (B) pregnant versus lactating; (C) lactating versus involuting mice, using miRDB version 6.0. These lists of predicted targets were analyzed using a KEGG pathway enrichment analysis. The top 10 pathways predicted to be modified are showed for each comparison. Numbers in brackets indicate the number of overlaps/gene set size.

To better understand how the change in miRNAs expression could be linked with biological processes, genes predicted to be targeted by those miRNAs were identified using miRDBv6.0 and used in a Gene Ontology (GO) enrichment (Fig. S1 and Tables S10-S12) and Kyoto Encyclopedia of Genes and Genomes (KEGG) pathway enrichment analysis (Fig. 5). A similar number of predicted genes were identified for each comparison, with 4999, 4414 and 5046 genes predicted to be targeted by the miRNAs between adult and pregnant mice, pregnant and lactating mice, and lactating and involuting mice, respectively (Fig. S1 and Tables S10-S12).

Using KEGG pathway enrichment analysis, we identified the 10 most enriched pathways involving the predicted targets. Six were common to the three comparisons: “breast cancer”, “FoxO signaling pathway”, “axon guidance”, “pathways in cancer”, “MAPK signaling pathway”, and “PI3K-Akt signaling pathway” (Fig. 5). “Focal adhesion” was found when comparing adult and pregnant mice, and pregnant and lactating mice, while “prostate cancer” was common to the comparisons between adult and pregnant mice, and pregnant and lactating mice (Fig. 5). “Endocrine resistance”, “Choline metabolism in cancer” and “Relaxin signaling pathway” were only found between adult and pregnant mice; “Regulation of actin cytoskeleton” and “Chronic myeloid leukemia” were only found between pregnant and lactating mice; “signaling pathways regulating pluripotency of stem cells”, “hedgehog pathway” and “proteoglycans in cancer” were found only when comparing lactating and involuting mice (Fig. 5).

### Various genes predicted to be targeted by expressed miRNA can be associated with hormonal regulation

**Fig. 6.**
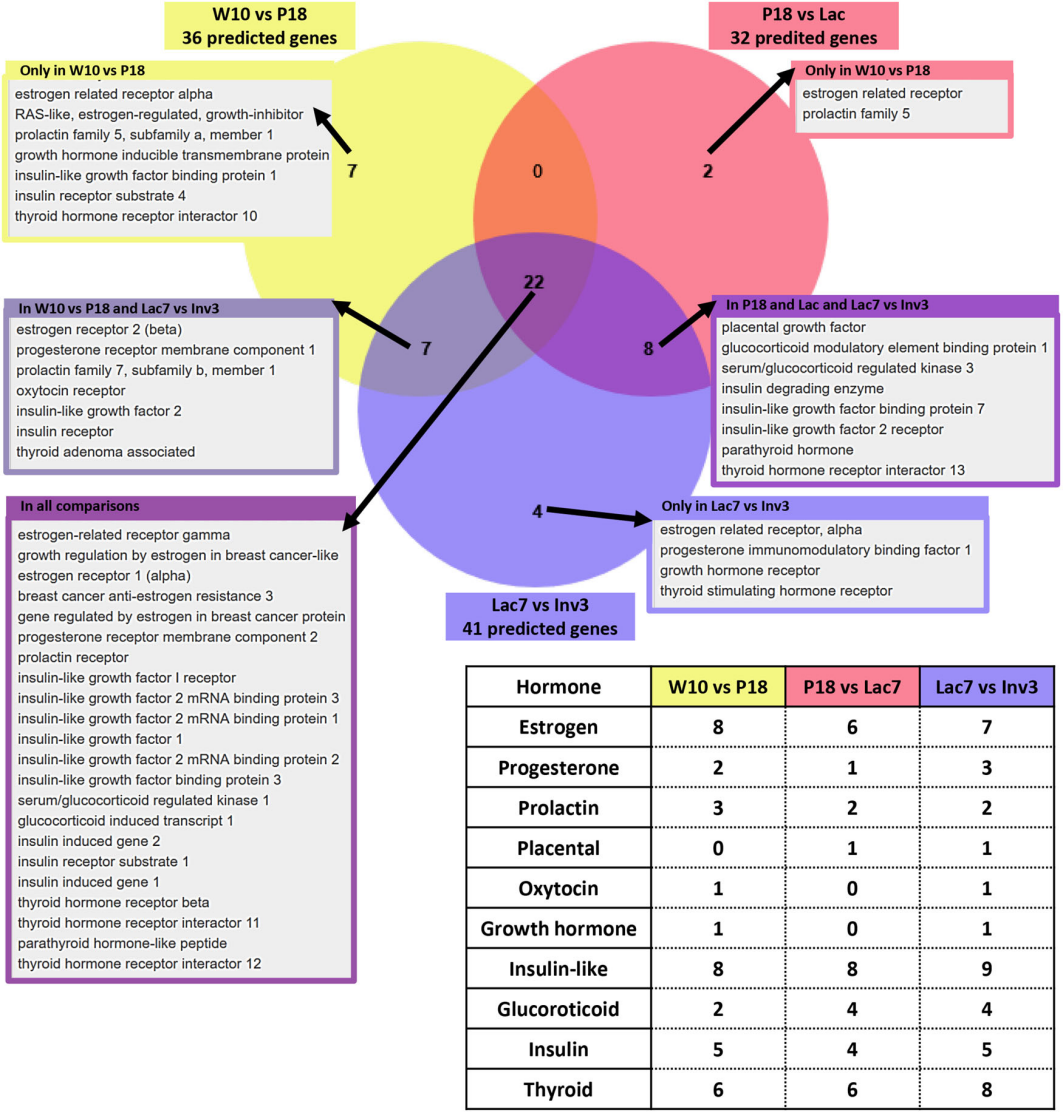
**Genes associated with hormonal regulation predicted to be modulated by the differentially expressed miRNA for each comparison.** The list of predicted mRNA target was generated for each set of differentially expressed miRNA: (A) adult versus pregnant; (B) pregnant versus lactating; (C) lactating versus involuting mice using miRDB version 6.0. and search for the terms: estrogen, progesterone, prolactin, placental, oxytocin, growth hormone, insulin-like, glucocorticoid, insulin and thyroid in reference to the main hormones known to be implicated in mammary gland development (Fig. 1). The Venn diagram shows results for each comparison and common to 2-3 comparisons. The table shows the number of terms associated with each hormone for each comparison.

From the KEGG pathway enrichment analysis, a few pathways could be linked with mammary gland homeostasis and/or hormone regulation, such as “Breast cancer”, and “Endocrine resistance” (Fig. 5). Other pathways, such as the FoxO, PI3K-Akt and MAPK pathways are known to crosstalk with the hormonal pathways (Atanaskova et al., 2002; Bullock, 2016; Ciruelos Gil, 2014; Dwyer et al., 2020; McGlynn et al., 2013). Knowing the importance of hormonal signaling in mammary gland development (Fig. 1), we then asked whether changes in miRNAs expression can be correlated with activation or inhibition of hormonal signaling pathways. To do so, we first searched the lists of predicted genes obtained by miRDB version 6.0 for each of the comparison groups using the terms “estrogen, progesterone, prolactin, placental, oxytocin, growth hormone, insulin-like, glucocorticoid, insulin and thyroid” in reference with the main hormones shown to be involved in mammary gland development (Fig. 1). Thirty-six, 32 and 41 genes predicted to be targeted by miRNA were found when analyzing results from the three comparison groups, including 22 that were common to all, 15 shared by two groups of comparison and 13 that were unique to a specific group of comparison (Fig. 6). For each comparison, most genes found were related to estrogen, insulin-like or insulin, and thyroid (Fig. 6, table).

### Distinct miRNAs are regulated by hormones *in vitro*

**Fig. 7.**
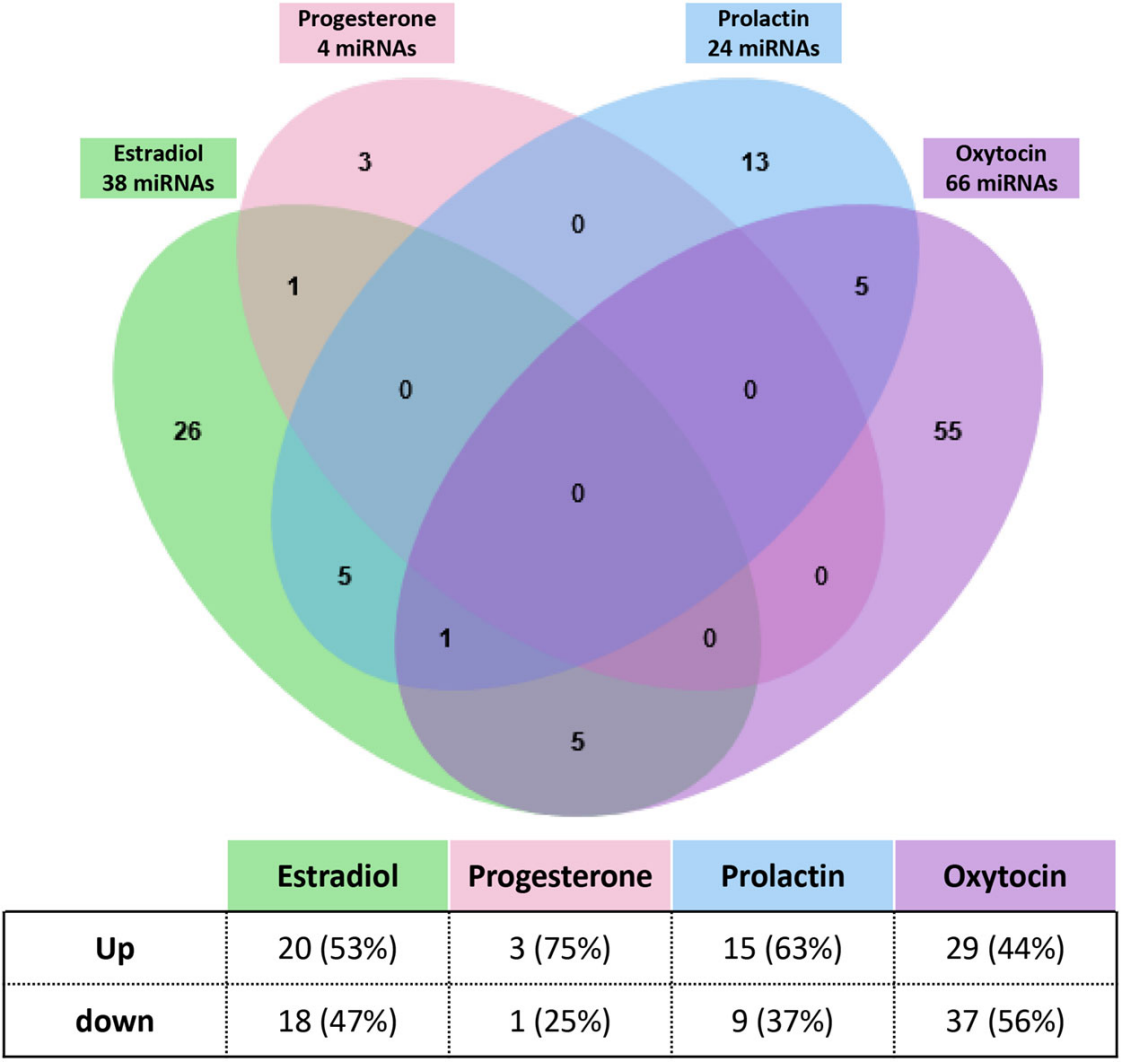
**miRNA modulated by estradiol, progesterone, prolactin and oxytocin in human breast T47D cells.** T47D cells were exposed to estradiol, progesterone, prolactin and oxytocin. Small RNA were extracted from cells using the mirVana™ miRNA Isolation kit (ThermoFisher) and sequenced (Illumina NovaSeq6000). 38 (estradiol), 4 (progesterone), 24 (prolactin) and 66 (oxytocin) miRNAs were differentially expressed in treated cells compared to vehicle treated cells. The Venn diagram shows that most miRNA are unique to one treatment, and none are shared by all treatments.

To further understand the links between hormones and miRNAs, we next exposed human breast cells to the four reproductive hormones that are well known to have a crucial role in mammary gland development and function, namely estradiol (estrogen), progesterone, prolactin, and oxytocin (Fig. 1). miRNAs were extracted and sequenced to evaluate the effects of hormonal treatment on miRNA expression. Four, 38, 24 and 66 miRNAs were associated with progesterone, estradiol, prolactin, and oxytocin, respectively (Fig. 7 and Table S13). Interestingly, no miRNA was common to all hormones, and most of them were unique to a specific treatment.

### Distinct miRNAs expressed in mice are hormonally regulated in breast cells

**Fig. 8.**
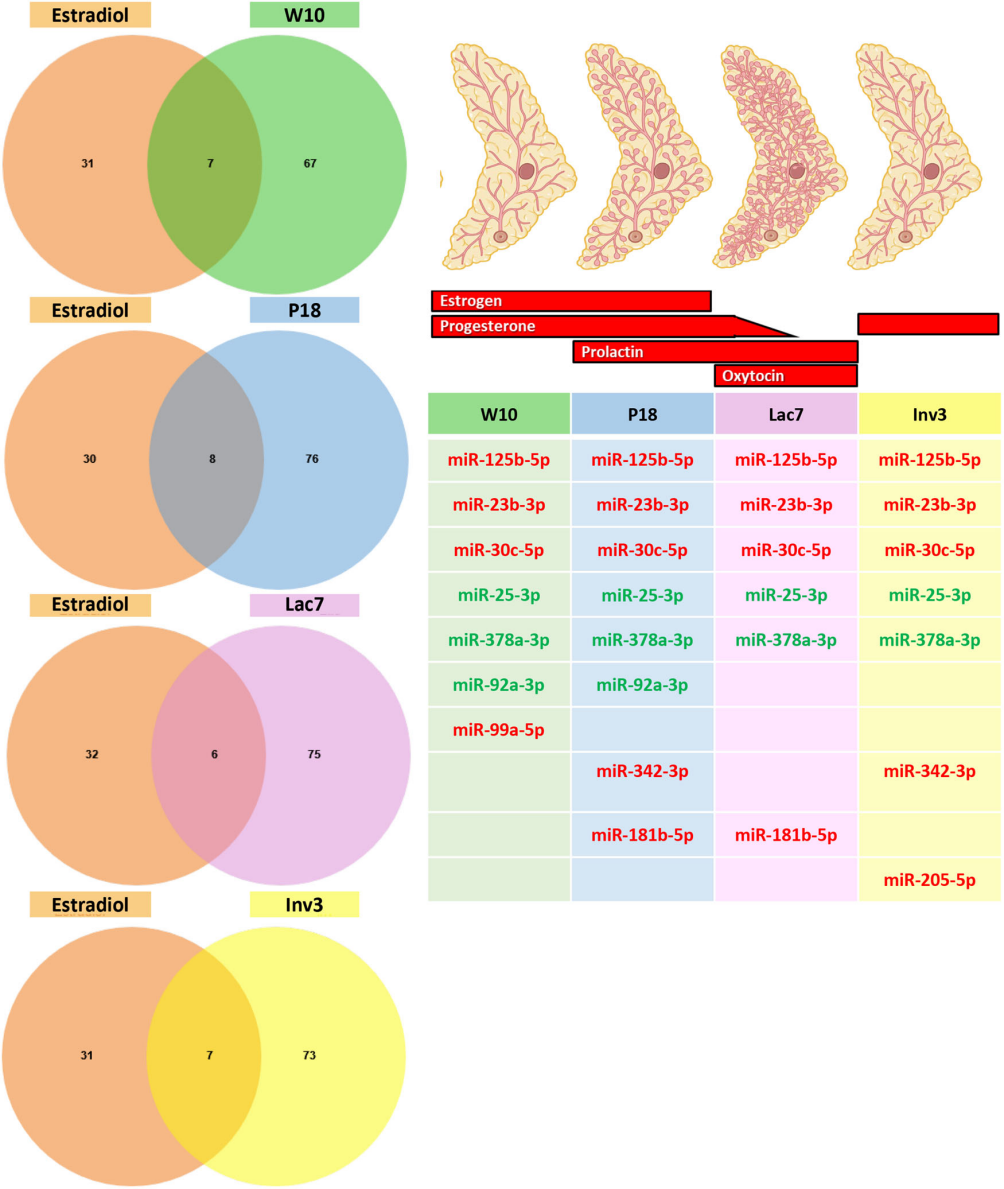
**Comparison between miRNA expressed at each stage of development and miRNA modulated by estradiol.** The Venn diagram shows that between 6-8 miRNAs that are modulated by estradiol are expressed at the four stages of development studied (≥500 reads). The table shows the list of miRNA that were upregulated (green) and downregulated (red) by estradiol at each stage of development.

**Fig. 9.**
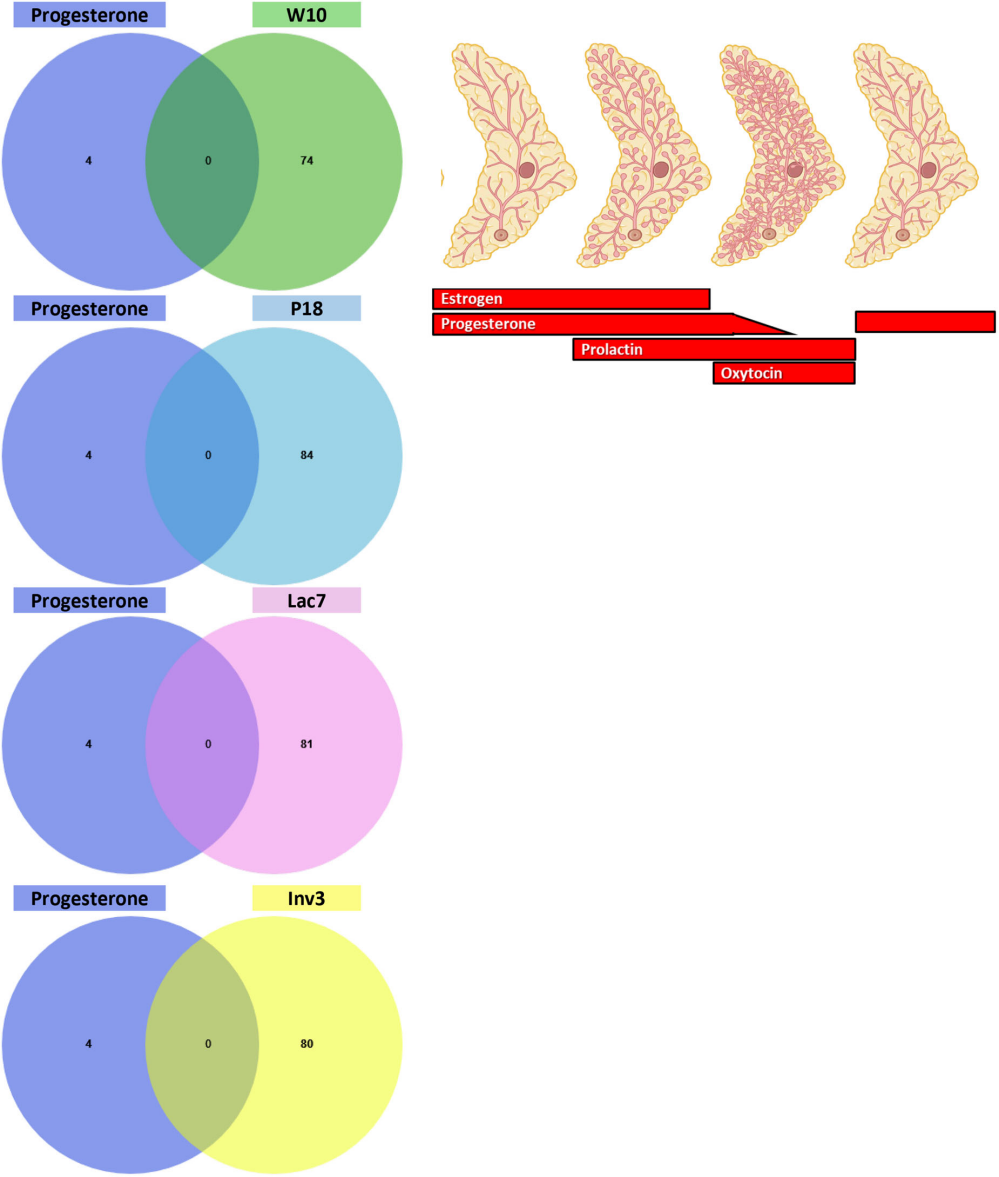
**Comparison between miRNA expressed at each stage of development and miRNA modulated by progesterone.** The Venn diagram shows that none of miRNAs that are modulated by progesterone are expressed at the four stages of development studied (≥500 reads).

**Fig. 10.**
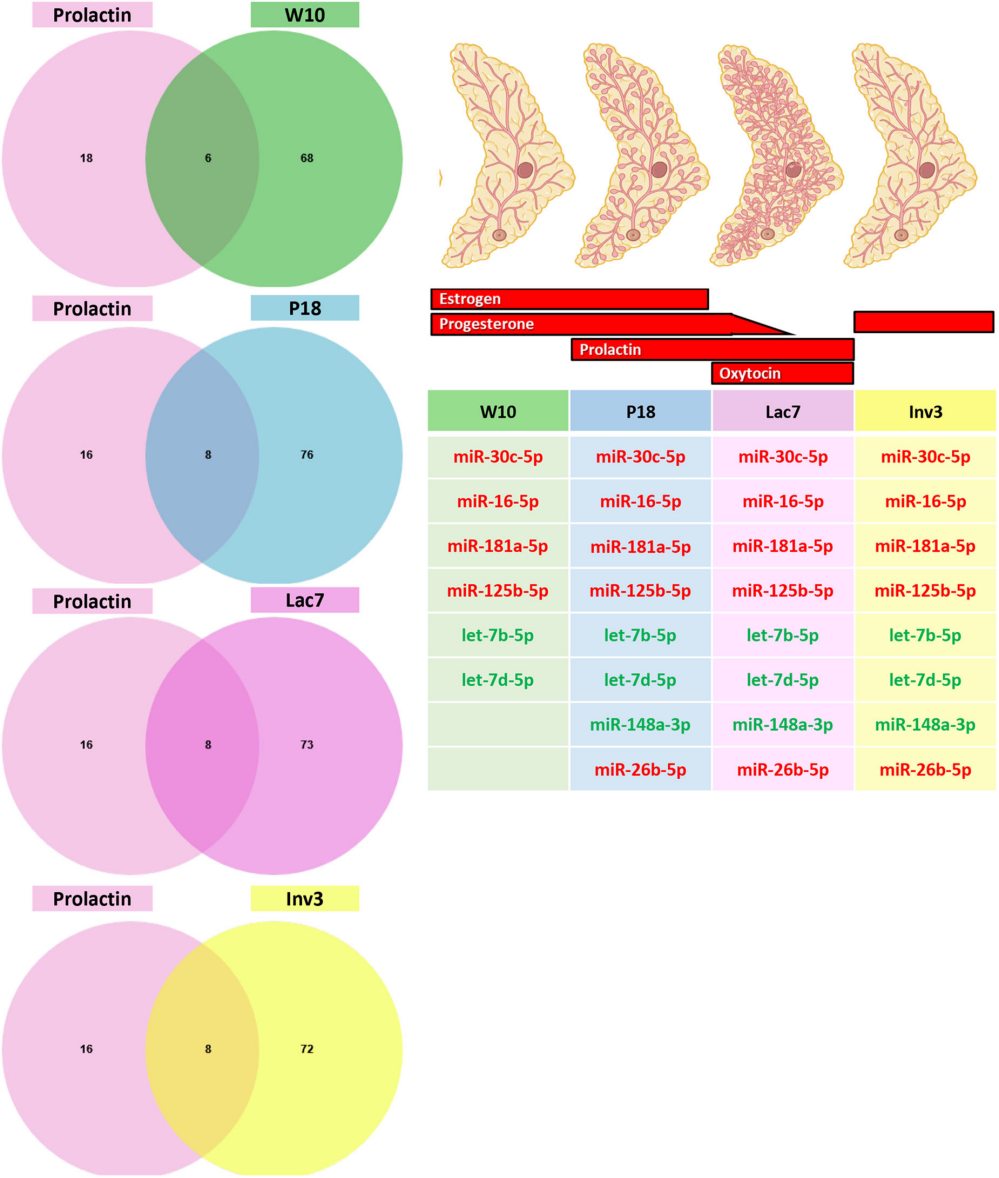
**Comparison between miRNA expressed at each stage of development and miRNA modulated by prolactin.** The Venn diagram shows that between 6-8 miRNAs that are modulated by prolactin are expressed at the four stages of development studied (≥0.500 reads). The table shows the list of miRNA that were upregulated (green) and downregulated (red) by prolactin at each stage of development.

**Fig. 11.**
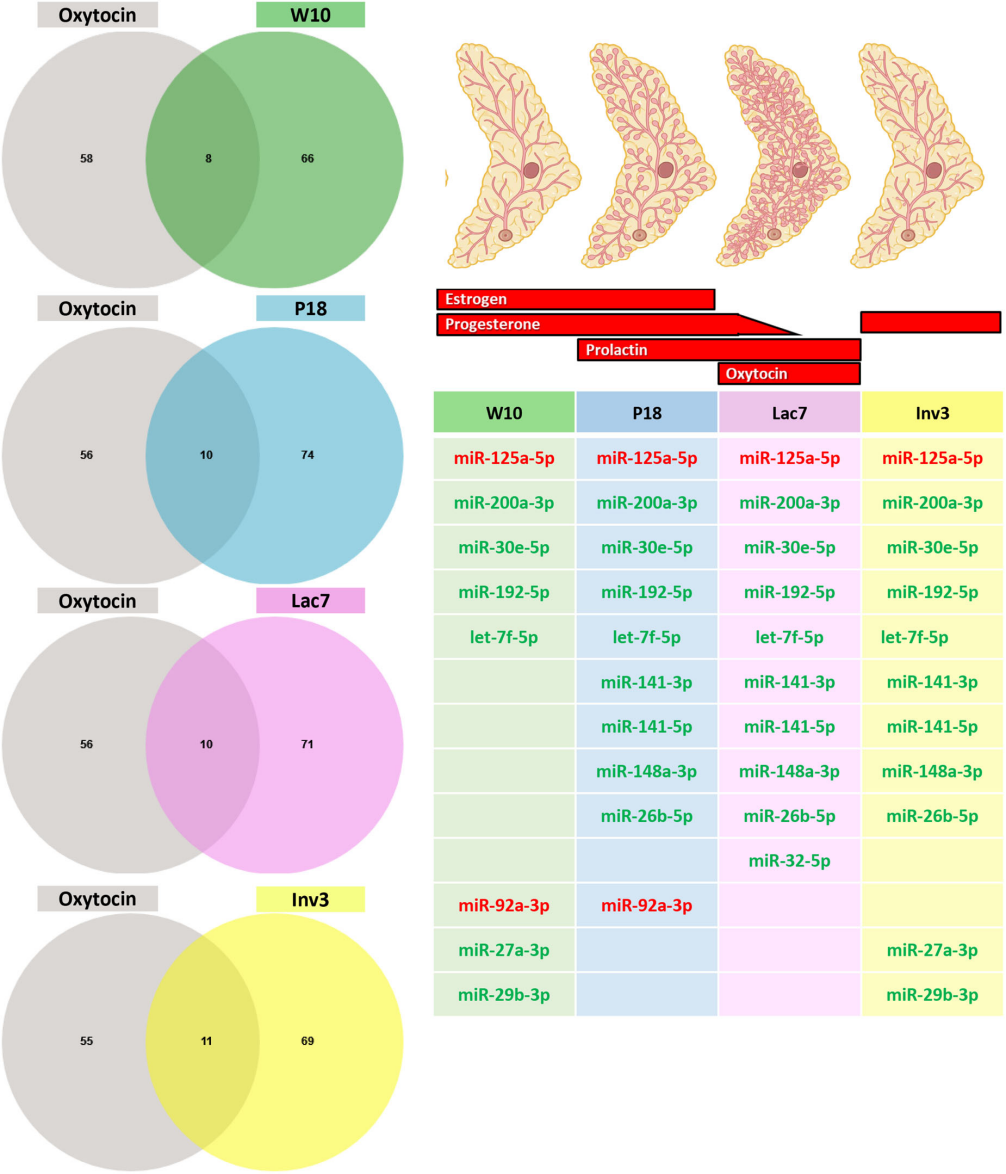
**Comparison between miRNA expressed at each stage of development and miRNA modulated by oxytocin.** The Venn diagram shows that between 8-11 miRNAs that are modulated by oxytocine are expressed at the four stages of development studied (≥0.500 reads). The table shows the list of miRNA that were upregulated (green) and downregulated (red) by oxytocin at each stage of development.

We next wanted to determine whether some miRNAs found *in vivo* could be linked with the four reproductive hormones tested *in vitro.* We thus compared the differentially expressed miRNAs upon each treatment (Fig. 7) with the list of miRNAs found at each stage of development (Fig. 2). Since it has been suggested that miRNA representing less than 100 reads per million are unlikely to be functionally relevant (Mullokandov et al., 2012), only miRNA with reads above 500 were used for each developmental stage. For estradiol, out of the 38 miRNAs that were statistically differentially expressed by the treatment *in vitro*, five were expressed at the four developmental stages (Fig. 8) while miR-92a-3p was expressed only in adult and pregnant mice, miR-342-3p was expressed in pregnant and involuting mice, miR-181b-5p was expressed in pregnant and lactating mice, and miR-205-5p in involuting mice, although its expression barely reached the cut-off of 500 RPM. For progesterone, only four miRNAs were found to be modulated by progesterone *in vitro,* and none were found to be expressed in the mammary gland at the stages studied (Fig. 9). For prolactin, six out of 24 miRNAs were expressed at the four developmental stages, and two were found in all stages, except in adult mice (Fig. 10). Surprisingly, miRNAs linked with oxytocin, which is thought to be crucial only at lactation (Fig. 1), showed the most variations. Out of the 66 miRNAs differentially expressed *in vitro*, five were common to all stages of development and four were found in all stages, except adult (Fig. 11). miR-92a-3p was expressed only in adult and pregnant mice (Fig. 11). miR-27a-3p and miR-29b-3p were expressed in adult and involuting mice only, although their expression was low, barely reaching the 500 RPM cut-off (data not shown).

**Fig. 12.**
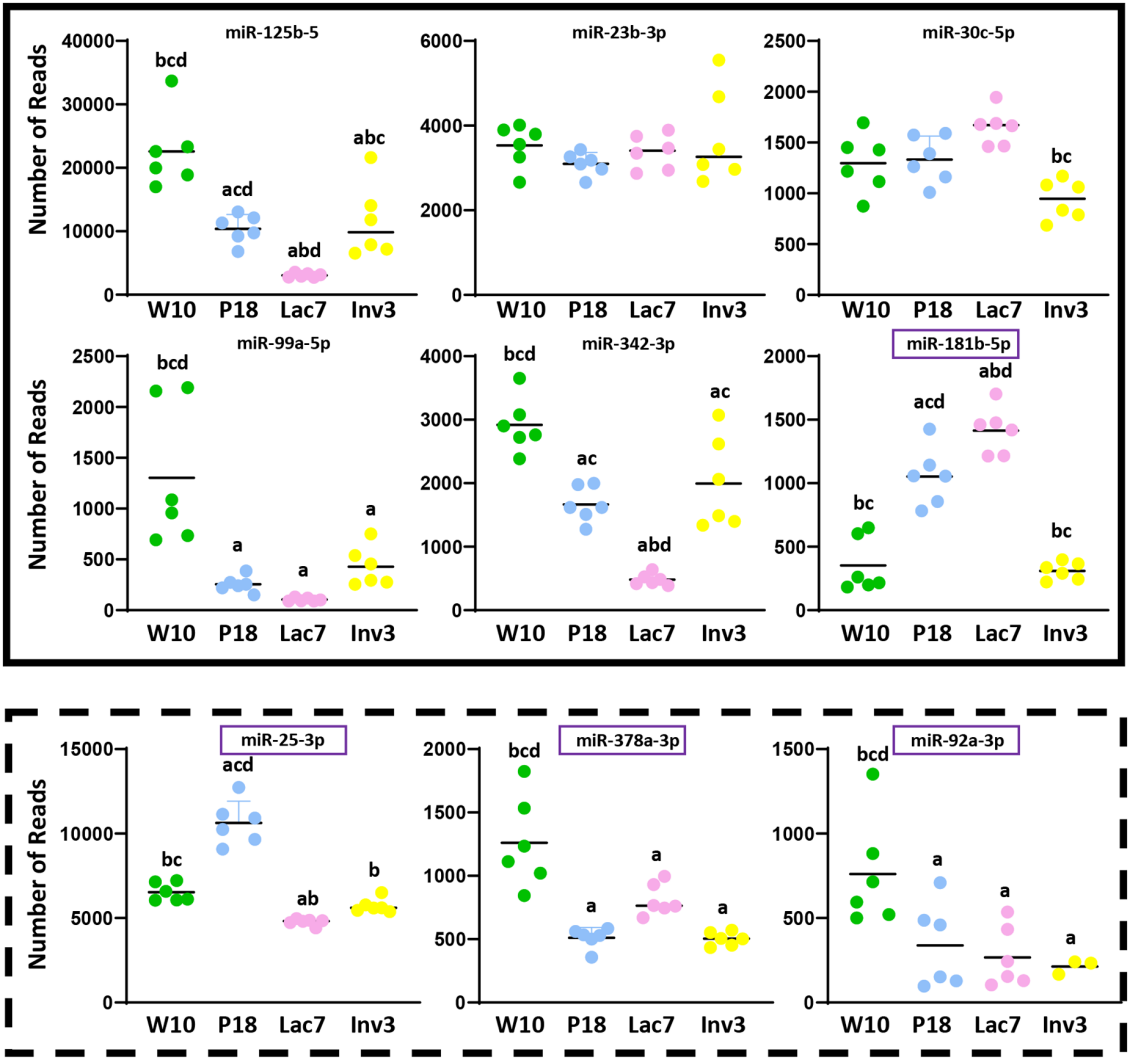
**Levels of expression of miRNA potentially regulated by estradiol during mammary gland development.** Small RNA were extracted from mammary glands using the mirVana™ miRNA Isolation (ThermoFisher) and sequenced (Illumina NovaSeq6000). Six animals were used per group (*N*=6). The red and the green squares indicate miRNA that were down- and upregulated by estradiol *in vitro*, respectively. Purple squares indicate miRNAs whose expression correlates with estradiol levels *in vivo.* For all graphs, a indicates different from W10; b indicates different from P18; c indicates different from Lac7; and d indicates different from Inv3 (*P*≤0,05) as analyzed by an one-way ANOVA followed by a Tukey's multiple comparisons test.

**Fig. 13.**
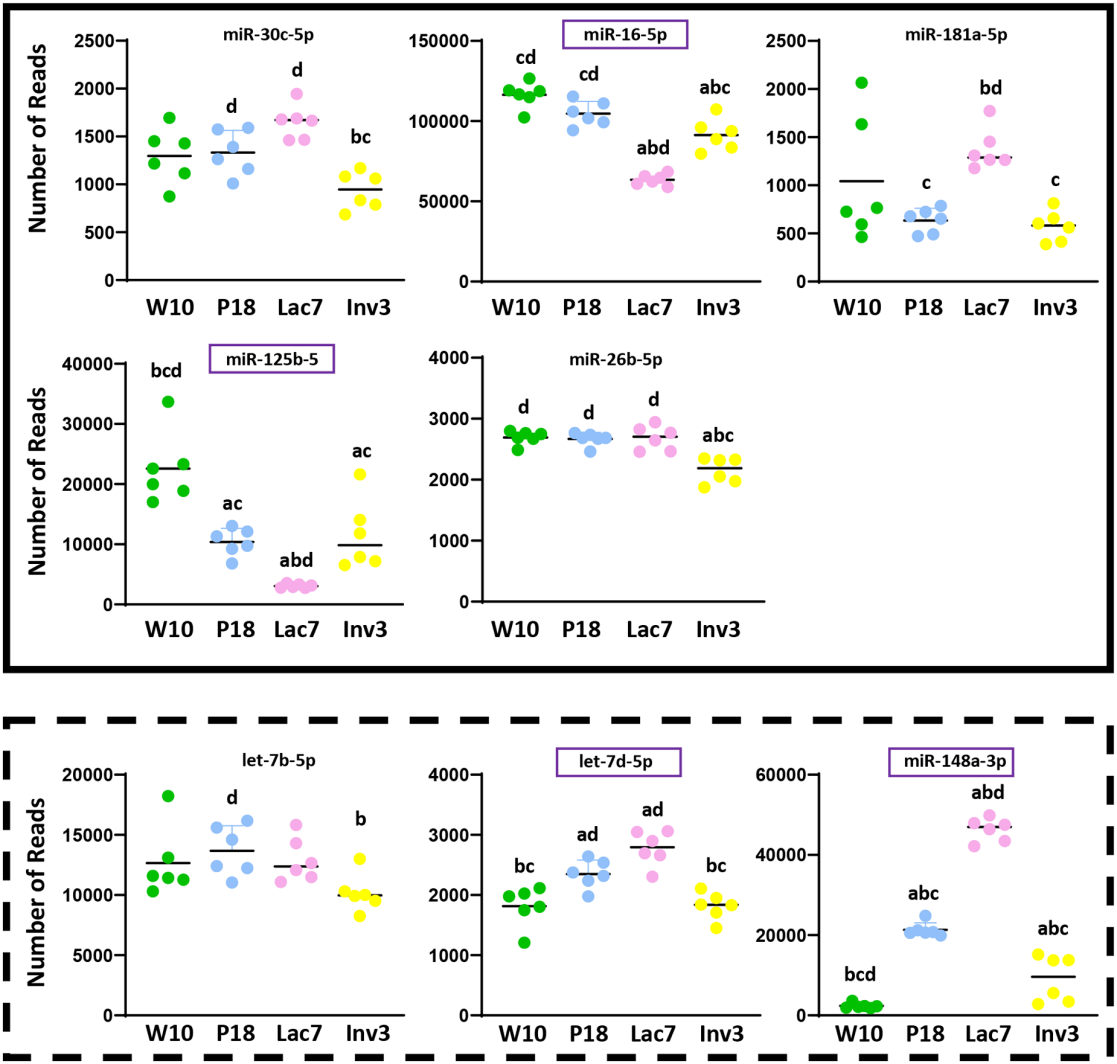
**Levels of expression of miRNA potentially regulated by prolactin during mammary gland development.** Small RNA were extracted from mammary glands using the mirVana™ miRNA Isolation kit (ThermoFisher) and sequenced (Illumina NovaSeq6000). Six animals were used per group (*N*=6). The red and the green squares indicate miRNA that were down- and upregulated by prolactine *in vitro*, respectively. Purple squares indicate miRNAs whose expression correlates with prolactin levels *in vivo.* For all graphs, a indicates different from W10; b indicates different from P18; c indicates different from Lac7; d indicates different from Inv3 (*P*≤0,05) as analyzed by an one-way ANOVA followed by a Tukey's multiple comparisons test.

**Fig. 14.**
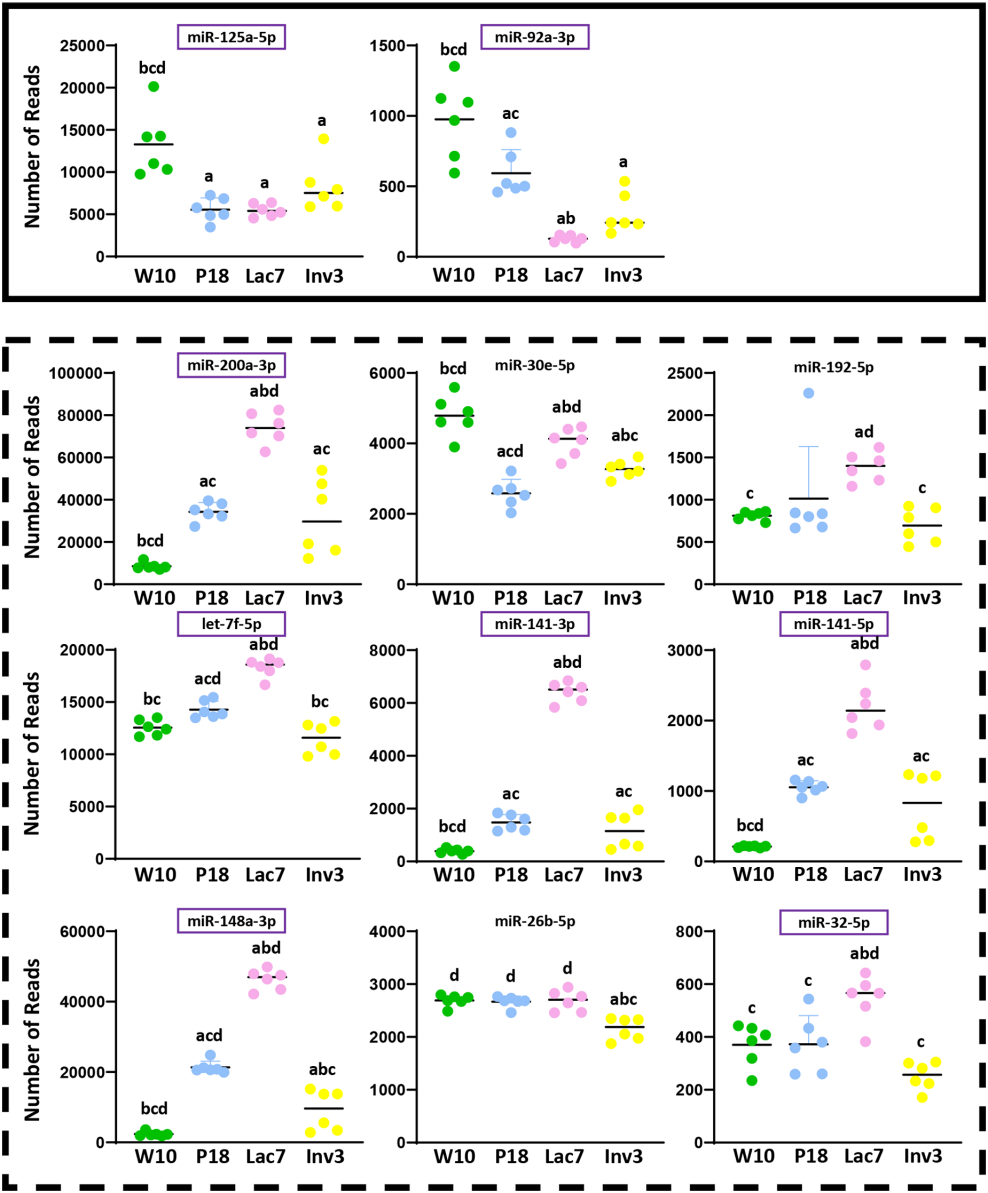
**Levels of expression of miRNA potentially regulated by oxytocin during mammary gland development.** Small RNA were extracted from mammary glands using the mirVana™ miRNA Isolation kit (ThermoFisher) and sequenced (Illumina NovaSeq6000). Six animals were used per group (*N*=6). The red and the green squares indicate miRNA that were down- and upregulated by oxytocin *in vitro*, respectively. Purple squares indicate miRNAs whose expression correlates with prolactin levels *in vivo.* For all graphs, a indicates different from W10; b indicates different from P18; c indicates different from Lac7; d indicates different from Inv3 (*P*≤0,05) as analyzed by an one-way ANOVA followed by a Tukey's multiple comparisons test.

**Fig. 15.**
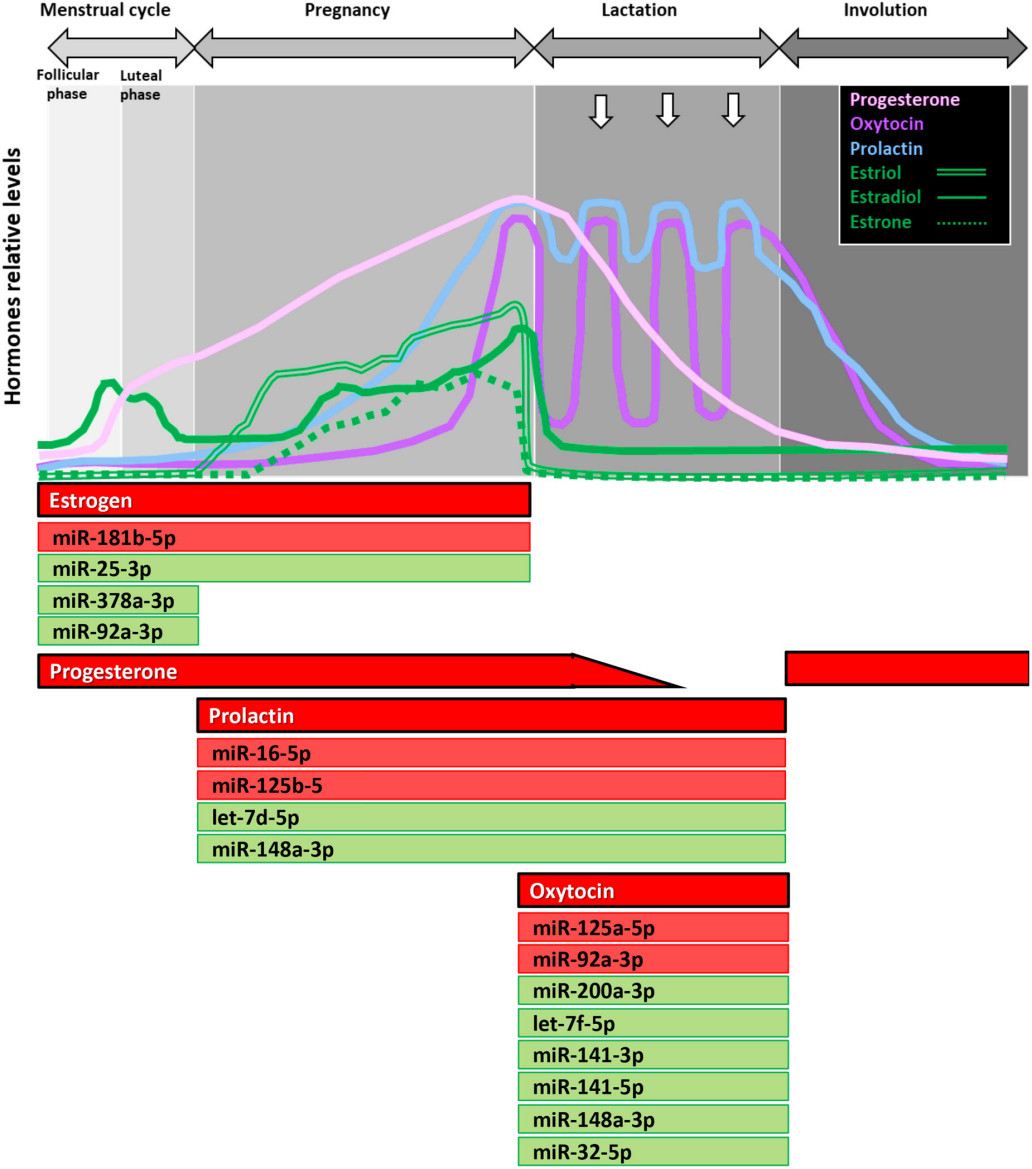
**miRNA likely regulated by hormones at specific stages of mammary gland development.** Top panel: schematic illustration of the relative levels of key hormones involved in mammary gland development at the different stages. Bottom panel: miRNAs likely to be regulated by hormones at each stage of development, based on *in vivo* and *in vitro* results of the current study.

Using the miRNAs identified from the Venn diagrams (Figs 8–11), we then evaluated whether their patterns of expression could be correlated with the relative levels of hormones known to be found *in vivo* (Figs 12–15). Based on these analyses, our results suggest that estradiol and prolactin are likely to influence the expression of four miRNAs each during mammary gland development (miR-181b-5p, miR-25-3p, miR-378a-3p, miR-92a-3p; miR-16-5p, miR-125b-5, let-7d-5p, miR-148a-3p), while oxytocin seems to regulate eight miRNAs (miR-125a-5p, miR-92a-3p, miR-200a-3p, let-7f-5p, miR-141-3p, miR-141-5p, miR-148a-3p, miR-32-5p) during lactation (Fig. 15).

## DISCUSSION

In the current study, we showed that although many miRNAs are expressed at the four key stages of mammary gland development studied and a few are uniquely expressed at one stage, their levels of expression vary greatly between stages, especially between adult and lactating mice. As expected, differentially expressed miRNA are linked to processes and pathways related with remodeling and hormonal regulation. Surprisingly, a limited number of miRNAs were regulated by exposure to hormones *in vitro*, especially for progesterone. Nevertheless, by comparing *in vivo* and *in vitro* results, and referring to relative hormonal levels at the different stages, we were able to identify 16 miRNAs likely regulated by hormones in a stage-specific manner.

### Limitations to take into consideration regarding the experimental design

Although our results bring interesting insight regarding the role of miRNA in mammary gland development, there are some limitations that should be considered when analyzing the data. First, our results represent the change in miRNA in the entire gland, as whole homogenates were done. Thereby, changes observed can be associated with change in miRNA expression in the epithelium, in the stroma, or in both compartments. Interestingly, data from Avril-Sassen and co-workers suggests that most miRNA differentially expressed in mammary gland homogenates are associated with mammary epithelium-driven events (Avril-Sassen et al., 2009). Thus, changes in most miRNAs detected in our analysis likely originate from the epithelium, even though the relative proportion of the stroma and epithelium varies between stages. Further analyses are needed to better understand the compartment-specific role and regulation of miRNAs in the mammary gland. Second, we limited ourselves to four developmental stages. The same above-mentioned study demonstrated that miRNA expression is similar from 6 weeks of age to early pregnancy, as for lactation and early stages of involution (between 12-48 h after weaning) (Avril-Sassen et al., 2009). Based on these results, we chose to use adult (W10), pregnancy day 18 (P18), lactation day 7 (Lac), and involution day 3 (Inv) in our study to optimize the distinction between miRNAs expressed at each stage, and to get different hormonal and stage profiles. Finally, we used mouse tissues to evaluate stage-specific miRNA expression, but a human breast cancer cell line for the hormonal treatments. Tissues are composed of many different types of cells, each potentially expressing different miRNAs, while the *in vitro* study reports the response of only one cell type to the treatment. This could explain, in part, why a fewer number of miRNAs were identified upon the cell treatments. Part of the discrepancy can also come from the fact that T47-D cells are cancerous cells. However, they represent the luminal subtype of breast cancer and are not considered are aggressive. More importantly, they express the hormonal receptors, as oppose of many non-tumorigenic cell lines, which was crucial for our experiment. In addition, some interspecies differences could be present in miRNAs regulation. However, it has been demonstrated that there is a high level of conservation between human and mice miRNAs. In a study looking at conserved miRNAs between 51 vertebrates using BLAST, bovine and murine miRNAs showed similar levels of conservation, and were the closest to humans (Ji et al., 2017; Xuan et al., 2020). Similarly, comparing mammospheres made from tissues from six mammals (cow, human, pig, horse, dog and rat), out of the 101 that were annotated, 86 were common to all six species (Miller et al., 2022), suggesting a good level of interspecies conservation. For this reason, in our study, the miRNAs commonly identified in mice and humans are likely to play a similar role within the mammary gland.

### The number of miRNAs expressed and their expression levels vary between stages in a pattern consistent with mammary gland development

When miRNAs were sequenced from mammary glands of mice at key stages of development, we found that 70-80% of miRNA were commonly expressed at all stages analyzed, while 12-18% were expressed at only one stage. These percentages were similar whether we include all miRNAs detected (>1 read per million) or the ones though to be functionally relevant (>500 reads per million) (Mullokandov et al., 2012). Interestingly, when analyzing the miRNAs expressed, especially those specific to only one stage, there was an apparent inverse correlation between the number of miRNAs expressed and the remodeling and functional activity thought to happen at that stage specifically. Indeed, the highest number of total and uniquely expressed miRNA was found in adult mice, when the mammary gland is relatively quiescent, followed by pregnancy, when the gland is undergoing alveologenesis, then lactation, the functional stage, and involution. The lowest number of uniquely expressed miRNA were found during involution, when the gland experiences an intensive phase of apoptosis and remodeling, including regression of the alveoli and the re-expansion of the fat pad. A similar pattern was found in dairy goats, with about 80% of miRNA being commonly expressed between dry period (adult), late pregnancy and late lactation, with the highest number of stage-specific, highly expressed miRNA found in the dry period (Xuan et al., 2020). In cows, the lowest number of miRNA expressed in the milk fat layer, which is thought to be the closest representation of mammary gland tissue (Li et al., 2016), was found in involution compared to lactation or galactopoiesis (established lactation) (Do et al., 2017). Considering that the main role of miRNAs is thought to be down regulation of mRNA levels, it suggests that many genes are repressed in adult mouse mammary glands but expressed at the other more active stages of development.

However, when analyzing expression levels between stages, the patterns were slightly different. Six main clusters of expression could be found, with most of the miRNAs following clusters 1 and 3, which showed opposite tendencies. In cluster 1, an increased expression of the 93 miRNAs could be observed from adult mice up to lactation, followed by a decrease in involution. This pattern was confirmed when analyzing the levels of expression of miRNAs differentially expressed (DE) between stages, as out of the 30 DE miRNA, most were upregulated between adult and pregnant mice, and between pregnant and lactating mice, while the majority were downregulated between lactation and involution. Similarly, a decreased number of miRNA and twofold reduction of miRNA mean expression was observed between lactation and early involution in mice in a study using a bead-based flow-cytometric microarray analysis (Avril-Sassen et al., 2009). In contrast, the 135 miRNAs in cluster 3 showed a significant decrease in expression from adult to lactation, followed by an increased expression at involution. For both clusters, most miRNA showed similar levels of expression between pregnancy and involution, although the expression was generally slightly lower at involution. Avril-Sassen and co-workers showed a decrease of expression at lactation, similar to our cluster 3, in their three largest clusters of expression, as well as an inverted cluster of expression with a peak of expression at lactation, similar to our cluster 1 (Avril-Sassen et al., 2009). These patterns of expression are consistent with the development of the mammary gland, as going from a relatively inactive stage (adult) gradually to a functional stage (lactation), and then returning to a more quiescent stage through the involution process requires activation and inhibition of specific signaling pathways.

### miRNAs are differentially expressed especially during lactation

Lactation is a particularly important stage for the mammary gland as it is the functional stage of the gland, involving an important remodeling and differential expression of thousands of genes (Lemay et al., 2009; Wickramasinghe et al., 2012). In addition, it is a stage of importance for the offspring of all mammals, but especially for the cattle. As a result, many studies on miRNA have been conducted at this stage in this species raised in milk industries (Do et al., 2017; Dysin et al., 2021; Galio et al., 2013; Ji et al., 2012; Le Guillou et al., 2014; Li et al., 2016, 2012; Wang et al., 2021; Xuan et al., 2020). Le Guillou and collaborators showed that 123 miRNA are present in mouse and bovine mammary glands analyzed at lactation, and 24 out of the 30 expressed at high levels were common to both species, suggesting conservation of miRNA between these two species (Le Guillou et al., 2014). Among those miRNAs, they found six miRNAs (miR-126-5p, miR-16-5p), and members of the miR-200 family (miR-141-3p, miR-200a-3p, miR-200b-3p, miR-200c-3p) that were present in both species at lactation, but not reported in other stages. In our study, although they were detectable at all stages, miR-141-3p and miR-200a-3p showed their highest expression level in lactation *in vivo* and were upregulated by oxytocin *in vitro.* miR-200b-3p and miR-200c-3p expression was also detectable at all stages and peaked at lactation. In contrast, miR-16-5p was not only present in all stages but was downregulated at lactation and by prolactin, and miR-126-5p was not detected. Although these discrepancies could be explained by a different sampling time (lactation day 7 versus 12), a strain difference (C57BL/6 versus FVB/N) or parameters used for sequencing, miR-126-5p and miR-16-5p were not reported either to be specific or peaking at lactation in other studies, including in mice (Avril-Sassen et al., 2009; Do et al., 2017; Dysin et al., 2021; Galio et al., 2013). Interestingly, using KEGG function annotations, Le Guillou and co-workers identified 83 biological processes targeted by miRNA highly expressed in lactation. Most of the pathways identified in the current study using the differentially expressed miRNA between pregnant and lactating mice, or between lactating and involuting mice, were present on this list (Le Guillou et al., 2014), and also in studies in the dairy goat (Dong et al., 2013; Xuan et al., 2020).

### Several differentially expressed miRNA are hormonally regulated *in vitro*

Since mammary gland development is tightly regulated by hormones, we aimed to determine the relationship between changes in miRNA between stages of development and hormones associated with those stages. Using miRDB version 6.0, we first determined genes predicted to be targeted by DE miRNA between stages. As expected, many potential targets were associated with reproductive and metabolic hormones known to play a role in mammary gland development (Fig. 1), including receptors for estrogen, prolactin, oxytocin, insulin, thyroid and growth hormone, and many related factors. The number of predicted targets was the highest for estrogen, insulin-like and thyroid signaling. These results suggest that miRNA can regulate hormonal signaling within the mammary gland, which is in concordance with studies demonstrating that overexpression of specific miRNAs results in a decrease in transcript or protein levels of estrogen receptor (ER)α and progesterone receptor, as well as signaling and the expression of genes regulated by these receptors (Cochrane et al., 2011; Fletcher et al., 2014; Godbole et al., 2017; Wang and Yang, 2021). However, using this approach, we could only identify genes likely targeted by miRNAs, but not miRNAs whose expression could be modulated by hormones. Thus, using an *in vitro* approach, we analyzed the four reproductive hormones that are known to play crucial roles in mammary gland development, and found that the expression of 38, 4, 24 and 66 miRNAs was significantly changed by estradiol, progesterone, prolactin and oxytocin, respectively. Within this list, a few miRNAs were already identified as estradiol regulated, such as miR-181a, miR-92a, miR-30b, and miR-23b, which were also regulated by estradiol in our experiments using T47-D cells (Cochrane et al., 2011; Fletcher et al., 2014), or expressed in ERα-positive T47-D cells, such as miR-23, miR125b and miR-30c (Cochrane et al., 2010).

The effects of progesterone on miRNA expression in breast cells have not been examined by many studies. One study in mouse mammary epithelial cells demonstrated that miR-129-2 targets the progesterone receptor (PR) and is upregulated in response to progesterone (Godbole et al., 2017). In T47-D cells treated for 6 h with medroxyprogesterone acetate (MPA), a synthetic progesterone molecule, 28 miRNAs were differentially expressed, 20 being downregulated and eight upregulated (Cochrane et al., 2012). Although for many miRNAs the trends were similar after 24 h of treatment, none reached statistical significance, suggesting a transient effect. We found only four miRNAs that were significantly changed (*P*≤0.05) upon progesterone treatment, although 179 showed a log2 fold change above 1.5, including some that were identified by Cochrane and collaborators (Cochrane et al., 2012). Whether the discrepancies between our results and their study is due to the difference in molecules used or time of exposure remains to be determined.

Similarly, only a few studies have analyzed the links between prolactin or oxytocin with miRNAs. In a recent article, miR-148a and miR-26a were upregulated, while miR-320 was downregulated in MCF10A cells, a non-tumorigenic human breast cell line, upon treatment with oxytocin (Gutman-Ido et al., 2022). In our study, 66 miRNAs were affected by oxytocin, including miR-148a and miR-26a that were also upregulated, and miR-320 (320a-3p, 320b, 320c), which was downregulated. Interestingly, the authors showed that the expression of miR-148a and miR-320 was also modified in extracellular vesicles secreted by MCF10A cells upon the treatment, as well as in human milk from mothers who received oxytocin during delivery, further supporting an important role of these miRNAs in oxytocin-induced signaling in the mammary gland. In addition, a few other miRNAs that were significantly expressed upon oxytocin treatment in our study, were also affected by an oxytocin treatment in pregnant women (Gutman-Ido et al., 2022). In another study, bovine mammary epithelial cells (BMECs) were exposed to a mixture of lactogenic hormones (dexamethasone, insulin and prolactin) for 6 days, and miRNA expression was analyzed in both the media and the cells (Muroya et al., 2016). The treatment induced a downregulation of miR-21-5p, miR-25, miR-26a, miR-223, and miR-320a in the cells, a downregulation of miR-155, miR-182, miR-200c, and miR-339a in the BMEC culture medium, and an upregulation of miR-148a in the BMEC culture medium (Muroya et al., 2016). Finally, miR-135b expression was shown to be expressed at early lactation in goats, gradually increasing until the end of lactation; its overexpression in epithelial cells was associated with decreased expression of β-casein and fat droplet formulation, two events associated with milk production (Chen et al., 2018). In addition, exposure to prolactin reduces its expression, supporting a role of miRNA in prolactin-induced milk production (Chen et al., 2018). In our study, miR-135b expression was low at all stages of development studied *in vivo*, and was only slightly decreased (not significant) upon prolactin treatment. This could be related to a species discrepancy, or a difference in the treatment as we used a lower dose *in vitro*.

### Conclusion

By combining the patterns of expression of miRNA during mammary development and the miRNAs with expression shown to be regulated by hormones, we identified 16 mammary gland miRNAs whose expression is likely to be regulated by circulating hormones. While some of them had already been identified to be important in mammary gland development, in breast cancer, or regulated by steroid hormones, our study suggests that there is a relationship with their expression and main hormones involved in mammary gland development (Fig. 15). Future studies will further examine this role more in detail.

## MATERIAL AND METHODS

### Animals

Female C57BL/6 mice were purchased from Charles River Canada (St. Constant, Quebec, Canada). Mice were maintained under a constant photoperiod of 12 h light:12 h dark and received food and water *ad libitum*. All animal protocols used in this study were approved by the University Animal Care Committee (INRS-Armand-Frappier Santé Biotechnologie, Laval, Canada). Female mice were euthanized using CO_2_ followed by cardiac puncture, and the mammary glands were collected at the following time points: adult (W10), pregnancy day 18 (P18), lactation day 7 (Lac7), and involution day 3 (Inv3). These stages were chosen based on a previous study that showed that miRNA expression is similar from 6 weeks of age to early pregnancy, as for lactation and early stages of involution (between 12-48 h after weaning) (Avril-Sassen et al., 2009). We thus used four distinct stages to optimize the distinction between miRNAs expressed at each stage, and to get different hormonal and stage profiles. For each developmental stage, six mice were sampled (*N*=6). The mammary gland pairs 4 and 5 (abdominal and inguinal) were flash-frozen in liquid nitrogen immediately after dissection.

### Cell culture and treatments

T47D cells, a luminal breast cancer cell line originating from a 54-year-old female patient, were obtained from American Type Culture Collection (ATCC). Cells were maintained in Roswell Park Memorial Institute (RPMI-1640) media without phenol red (ThermoFisher Scientific, USA), supplemented with fetal bovine serum (FBS) (Wisent Bio Products, Canada) at 10% final concentration, and incubated at 37°C and 5% CO_2_. Cells were plated at normal seeding density and passaged before 90% confluency to maintain log-phase growth. Doubling time was calculated and the cells were not kept for more than 10 passages. At 60 to 70% confluency and 16 h prior to hormonal exposure, media was replaced with media containing hormone-stripped FBS (Wisent Bio Products, Canada) to eliminate effects of background hormone signaling. Then, cells were exposed to estrogen (E8875, Sigma-Aldrich, USA), progesterone (P0130, Sigma-Aldrich, USA), prolactin (L4021, Sigma-Aldrich, USA) and oxytocin (O6379, Sigma-Aldrich, USA) or the appropriate vehicle. Based on literature, treatments were as follows: 8.7 nM (200 ng/ml) and 8 h for prolactin, 10 nM and 24 h for estrogen, 10 nM and 6 h for progesterone, and 100 nM and 24 h for oxytocin (Bhat-Nakshatri et al., 2009; Cochrane et al., 2012; Cook et al., 2015; Ferraro et al., 2012). Vehicles were 0.1% ethanol for estrogen and progesterone, 4 mM hydrochloric acid for prolactin and nuclease-free water for oxytocin.

### *In vitro* miRNA isolation and sequencing

Upon termination of exposure time, media was removed, cells were washed with Dulbecco's phosphate buffered saline (ThermoFisher Scientific, USA) and detached by incubating at 37°C with trypsin-EDTA 0.25% (ThermoFisher Scientific, USA) for 7 to 10 min. Then, cells were pelleted by centrifuging for 5 min at 125 ***g***, and the supernatant was discarded. The *mir*Vana™ miRNA isolation kit (ThermoFisher Scientific, Mississauga, ON, Canada) was used according to instructions to isolate small RNA samples from cells. miRNAs were quantified using a Nanodrop 1000 (ThermoFisher Scientific, USA), while purity and integrity were assessed using the Agilent Small RNA kit and an Agilent 2100 bioanalyzer (Agilent, USA). Data were analyzed using the Bioanalyzer 2100 Expert (version B.02.11.S1824 SR1) software; digital gel images and electropherograms of samples were validated prior to sequencing. Extracts were delivered to the Institute for Research in Cancer and Immunology (IRIC) for miRNA-seq analysis (Illumina platform). FASTQC version 0.11.8 was used for read-quality assessment, while Cutadept was used for reads trimming. miRNA amount was calculated using MIRDEEP and COMPSRA software, and DESeq2 software was applied to calculate differential expression within each separate hormone experiment. Where applicable, the removeBatchEffect software from limma was used to correct miRNA quantity. The datasets can be found in the NCBI GEO database under the accession number GSE263253.

### *In vivo* miRNA isolation and sequencing

Frozen tissues were ground in liquid nitrogen. miRNAs were extracted from the resulting powder using the *mir*Vana™ miRNA isolation kit following the manufacturer's instructions (ThermoFisher Scientific, Mississauga, ON, Canada). The miRNA concentration was quantified with a Nanodrop 1000 (ThermoFisher Scientific, Canada), while purity and integrity were assessed using the Agilent Small RNA kit (Agilent, Wilmington, DE, USA) and an Agilent bioanalyzer 2100. Data were analyzed using the Bioanalyzer 2100 Expert (version B.02.11.S1824 SR1) software; digital gel images and electropherograms of samples were validated prior to sequencing. miRNA libraries were prepared using a QIAseq miRNA stranded kit (Quiagen, Toronto, ON, Canada) and sequenced using an Illumina NextSeq 500 apparatus. The mapping against reference genome was done using QuickMirSEq and miRbase/22 (Kozomara et al., 2019; Zhao et al., 2017). Analyses of differently expressed miRNAs and of clustering were performed using DESeq2/1.26.0, DEGReport and bigPint applications (Rutter and Cook, 2020; Stephens, 2017). The datasets can be found in the NCBI GEO database under the accession number GSE264358.

## Supplementary Material

10.1242/biolopen.060308_sup1 Supplementary information
