# Supplementary material for: Hormonal regulation of miRNA during mammary gland development
Source: Biol Open. 2024 Jun 10;13(6):bio060308. doi: 10.1242/bio.060308 (PMC11190577; doi:10.1242/bio.060308)
Supplement: Supplementary information [file biolopen-13-060308-s1.pdf]

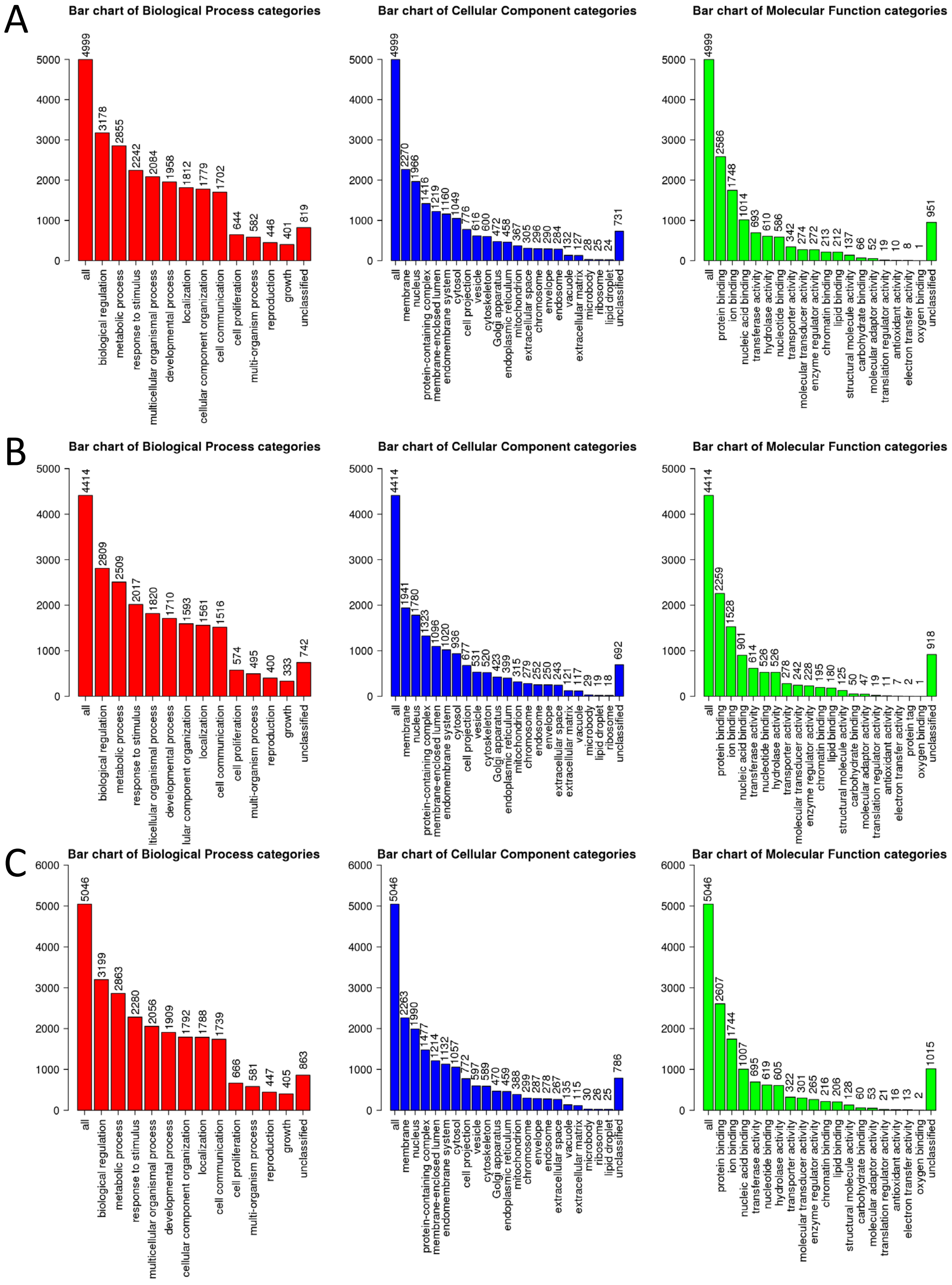

Fig. S1. Biological processes, cellular component and molecular functions predicted to be modified by miRNA between the different stages of mammary gland development. Using the WEB-based GEne SeT AnaLysis Toolkit (Wang, 2013), functional predictions were made based on change in mammary gland miRNA levels between virgin adult (W10) and pregnant mice (P18) (**A**), P18 and lactating mice (Lac7) (**B**) and Lac7 and involuting mice (Inv3) (**C**). To do so, a list of target genes was retrieved (containing 4999, 4414 and 5046 unique entrezgene ID, respectively), and submitted to a GO enrichment analysis. Target genes were collected from miRDB/v6.0 (Chen, 2020), that is using MirTarget/V4 (Liu, 2019) for predictions and miRBase/22 for miRNAs (Kozomara, 2019). Functional analyses were clustered by biological processes (red charts), cellular components (blue charts) and molecular function (green charts) categories. The number of unique entrezgene ID in each category is indicated on top of each bar.

Biology Open • Supplementary information

Table S1. miRNA expressed in the mammary gland at 10 weeks of age. The number of reads determined by the sequencing is indicated for each of the animal, as well as the average of the group (N=6). Only miRNA with an average read for the group above 1 are listed.

miRNA	W10_1	W10_2	W10_3	W10_4	W10_5	W10_6	Average expression
**mmu-miR-16-5p**	126502.07	119135.45	118705.94	102295.40	114919.88	116735.36	**116382.35**
**mmu-let-7c-5p**	98932.93	86198.05	121138.64	81195.43	98389.56	93363.62	**96536.37**
**mmu-miR-143-3p**	103515.02	92640.21	86609.11	96338.47	84720.84	90271.59	**92349.21**
**mmu-miR-199a-3p**	101843.51	69335.46	82322.00	76148.83	87954.14	76142.13	**82291.01**
**mmu-miR-142b**	45639.78	97586.37	51252.23	81665.66	78377.56	86919.98	**73573.60**
**mmu-let-7a-5p**	45186.86	48393.17	62991.04	40754.82	57668.53	53432.76	**51404.53**
**mmu-miR-29a-3p**	52788.46	52878.56	36837.13	45352.71	39818.47	60474.12	**48024.91**
**mmu-miR-103-3p**	34277.83	30107.15	43128.99	31803.28	30939.80	34157.54	**34069.10**
**mmu-miR-21a-5p**	30544.43	33761.03	27889.82	41060.41	30619.71	33298.26	**32862.28**
**mmu-miR-142a-5p**	20656.63	39125.13	21751.58	38166.95	38327.33	33897.97	**31987.60**
**mmu-miR-125b-5p**	23332.13	18888.45	33698.88	19992.73	22578.87	17030.54	**22586.93**
**mmu-miR-23a-3p**	12041.35	16599.42	17261.47	17298.33	14694.59	15949.47	**15640.77**
**mmu-miR-126a-3p**	15111.53	15220.58	15766.96	15105.38	15423.98	16424.86	**15508.88**
**mmu-miR-27b-3p**	12676.52	13403.69	13485.70	14918.15	14888.75	12791.80	**13694.10**
**mmu-miR-125a-5p**	14264.99	11007.11	20140.76	10324.55	14182.96	9769.39	**13281.63**
**mmu-miR-146a-5p**	14724.39	14306.89	13423.15	11427.49	12606.11	12334.31	**13137.06**
**mmu-let-7b-5p**	11588.42	11272.91	18218.31	10311.64	11433.30	13111.05	**12655.94**
**mmu-let-7f-5p**	12405.84	11831.62	12644.07	11696.50	13300.52	13500.91	**12563.24**
**mmu-miR-1b-5p**	11955.07	7963.52	1811.63	18851.05	15400.37	2537.08	**9753.12**
**mmu-miR-101a-3p@**	6499.48	8671.74	8236.26	12826.33	12018.39	10122.45	**9729.11**
**mmu-miR-133a-3p**	8972.24	10082.06	2066.20	20488.78	12930.57	3166.62	**9617.75**
**mmu-miR-200a-3p**	7072.11	8082.43	7832.46	11694.35	8507.85	8095.57	**8547.46**
**mmu-miR-10a-5p**	10924.13	7380.34	9949.13	6943.65	6826.05	7040.36	**8177.28**
**mmu-miR-30a-5p**	8059.92	6798.90	6418.05	6009.65	5599.02	7687.81	**6762.23**
**mmu-miR-26a-5p**	6425.07	6170.24	7584.47	7277.22	6324.04	6443.64	**6704.11**
**mmu-miR-25-3p**	7144.36	6576.81	7215.78	6122.64	6050.30	6069.69	**6529.93**
**mmu-miR-30d-5p**	8091.19	6749.06	7423.17	5142.37	4979.82	6235.78	**6436.90**
**mmu-miR-195a-5p**	5451.28	5912.31	7490.10	6072.06	5724.96	6296.45	**6157.86**
**mmu-miR-203-3p**	8552.74	5300.27	5724.56	3943.67	4621.25	5777.29	**5653.30**

Biology Open • Supplementary information

**mmu-miR-196a-5p**	8671.36	4961.03	5406.35	3512.18	5036.67	5268.09	**5475.95**
**mmu-miR-199a-5p**	4288.77	5019.61	4392.45	5246.75	4566.15	5866.80	**4896.76**
**mmu-miR-30e-5p**	4909.93	5112.29	3899.77	4608.66	4595.01	5593.30	**4786.49**
**mmu-miR-142a-3p**	2913.82	6154.51	3309.43	5337.13	5009.56	5810.11	**4755.76**
**mmu-miR-200b-3p**	3984.67	4186.36	5713.59	5785.84	4599.38	3782.24	**4675.35**
**mmu-miR-3074-5p**	3279.40	4431.17	5332.83	5847.17	4663.23	4472.46	**4671.04**
**mmu-miR-15b-5p**	1879.64	4680.36	3188.73	3645.61	4061.52	4027.89	**3580.63**
**mmu-miR-93-5p**	3898.39	3520.98	3790.04	3473.44	3146.72	3447.08	**3546.11**
**mmu-miR-23b-3p**	2664.71	3798.15	3899.77	4010.38	3559.52	3257.12	**3531.61**
**mmu-miR-140-3p**	4356.71	3370.59	3578.26	3337.86	3065.38	3071.15	**3463.33**
**mmu-miR-16-5p**	2846.96	2594.18	3938.17	3020.43	3137.10	3244.20	**3130.17**
**mmu-let-7c-5p**	2722.94	3079.44	3651.78	2761.11	2900.96	2384.91	**2916.86**
**mmu-miR-143-3p**	4455.92	2656.26	3062.54	2398.48	2485.54	2293.41	**2892.03**
**mmu-miR-199a-3p**	2802.75	3088.18	2291.14	2910.67	2294.88	3643.01	**2838.44**
**mmu-miR-142b**	2687.36	2670.25	2761.88	2487.79	2796.89	2749.91	**2692.35**
**mmu-let-7a-5p**	2857.74	2914.19	1796.26	2351.14	2335.99	3377.46	**2605.46**
**mmu-miR-29a-3p**	3404.49	2087.06	2060.71	2184.35	2385.84	2310.32	**2405.46**
**mmu-miR-103-3p**	2815.69	1948.04	1879.66	2478.11	2261.65	2767.81	**2358.49**
**mmu-miR-21a-5p**	3632.03	1914.81	1809.43	2294.11	2146.20	2339.16	**2355.96**
**mmu-miR-142a-5p**	2003.66	2219.08	1600.95	2526.53	2412.95	2480.39	**2207.26**
**mmu-miR-125b-5p**	2817.84	2035.47	2434.89	1555.95	2134.83	2263.58	**2207.09**
**mmu-miR-23a-3p**	1807.39	2387.83	1848.93	2555.58	1853.22	2696.20	**2191.53**
**mmu-let-7g-5p**	1550.73	2197.23	1803.94	1741.02	2256.40	2547.02	**2016.06**
**mmu-miR-221-3p**	1731.90	1819.51	2423.91	2333.92	1662.56	1703.65	**1945.91**
**mmu-miR-223-3p**	1150.65	1988.26	1664.59	2054.15	2055.25	2390.88	**1883.96**
**mmu-miR-19b-3p**	1395.44	2333.62	1270.66	2196.19	1552.37	2544.04	**1882.05**
**mmu-let-7d-5p**	1207.80	1749.56	2113.38	1806.66	1979.16	2023.89	**1813.41**
**mmu-miR-497a-5p**	1055.75	1750.44	1640.45	2156.37	1507.77	2295.40	**1734.36**
**mmu-miR-107-3p**	991.04	1448.79	1585.58	1448.34	1345.97	1640.99	**1410.12**
**mmu-miR-99a-5p**	2157.87	957.41	1086.32	2189.73	693.54	733.97	**1303.14**
**mmu-miR-30c-5p**	873.50	1428.68	1115.94	1450.49	1217.41	1694.70	**1296.79**

Biology Open • Supplementary information

**mmu-miR-378a-3p**	1824.64	1112.17	1234.45	1533.35	844.84	1020.40	**1261.64**
**mmu-miR-191-5p**	1326.42	1154.13	1439.64	1148.13	1316.23	1059.19	**1240.62**
**mmu-miR-1a-3p**	1502.20	988.88	213.97	2325.31	1792.88	350.08	**1195.55**
**mmu-miR-145a-5p**	780.76	1225.83	1211.41	1453.72	815.10	1417.22	**1150.67**
**mmu-miR-214-3p**	876.73	783.41	1179.59	1979.90	1222.65	602.69	**1107.50**
**mmu-miR-320-3p**	992.12	954.78	1684.34	1219.15	955.03	833.43	**1106.48**
**mmu-miR-150-5p**	637.33	1413.81	1132.40	941.53	1229.65	1098.97	**1075.62**
**mmu-miR-872-5p**	1524.85	919.81	853.69	1352.58	904.31	833.43	**1064.78**
**mmu-miR-425-5p**	957.61	1130.53	1005.12	1367.64	1011.01	806.57	**1046.41**
**mmu-miR-181a-5p**	1634.85	725.71	764.81	2065.99	594.71	464.45	**1041.75**
**mmu-miR-451a**	998.59	1139.27	539.87	1761.47	549.23	1002.50	**998.49**
**mmu-miR-92a-3p**	1124.76	967.90	1097.29	1351.50	714.53	594.74	**975.12**
**mmu-miR-199b-5p**	750.56	1086.81	580.47	1028.69	730.27	1589.28	**961.01**
**mmu-miR-15a-5p**	657.82	1077.19	751.64	1002.86	864.08	1155.66	**918.21**
**mmu-miR-194-5p**	714.98	927.68	732.99	709.11	803.73	1026.37	**819.14**
**mmu-miR-192-5p**	851.93	838.50	774.69	729.55	812.48	861.27	**811.40**
**mmu-miR-541-5p**	968.40	659.26	826.26	794.11	586.84	648.44	**747.22**
**mmu-miR-10b-5p**	923.11	732.70	865.76	810.25	488.01	656.40	**746.04**
**mmu-miR-182-5p**	857.32	625.16	776.88	716.64	734.64	577.83	**714.75**
**mmu-miR-1843a-5p@**	740.86	661.00	711.04	697.27	583.34	606.67	**666.70**
**mmu-miR-3065-5p**	466.94	662.75	549.74	632.71	690.91	662.36	**610.90**
**mmu-miR-365-3p**	504.69	796.53	539.87	489.60	418.92	864.26	**602.31**
**mmu-miR-29b-3p**	397.93	631.28	454.28	565.99	645.44	778.73	**578.94**
**mmu-miR-27a-3p**	353.71	634.77	502.56	640.24	571.10	770.77	**578.86**
**mmu-miR-338-3p**	519.79	688.98	474.03	676.83	442.53	618.61	**570.13**
**mmu-miR-184-3p**	306.26	566.57	542.06	262.55	661.18	715.08	**508.95**
**mmu-miR-152-3p**	515.47	452.04	502.56	515.42	445.16	528.10	**493.13**
**mmu-miR-339-5p**	526.26	530.73	514.63	416.43	419.80	507.22	**485.85**
**mmu-miR-133b-3p**	411.95	575.32	70.23	1124.46	510.75	179.02	**478.62**
**mmu-miR-151-5p**	432.44	436.30	361.01	649.93	557.98	389.86	**471.25**
**mmu-miR-151-3p**	533.80	362.85	692.39	389.52	495.88	274.49	**458.16**

**mmu-miR-205-5p**	553.22	330.50	511.34	690.81	278.11	258.58	**437.09**
**mmu-miR-24-3p**	350.48	383.84	497.07	521.88	404.93	424.67	**430.48**
**mmu-miR-22-3p**	254.50	488.76	295.17	660.69	363.82	508.21	**428.53**
**mmu-miR-3964**	382.83	497.50	219.46	440.10	438.16	504.23	**413.71**
**mmu-miR-141-3p**	270.68	398.70	328.09	528.33	436.41	393.84	**392.68**
**mmu-miR-32-5p**	319.20	432.80	234.82	386.30	442.53	407.76	**370.57**
**mmu-miR-125b-2-3p**	341.85	313.02	409.29	386.30	347.21	384.89	**363.76**
**mmu-miR-301a-3p**	250.19	403.07	256.77	477.76	341.08	421.69	**358.43**
**mmu-miR-708-5p**	432.44	331.38	323.70	312.05	296.48	414.72	**351.80**
**mmu-miR-181b-5p**	602.82	215.96	260.06	649.93	198.53	183.00	**351.72**
**mmu-miR-30b-5p**	227.54	422.31	241.40	388.45	327.09	481.36	**348.03**
**mmu-miR-3068-5p**	298.72	338.37	320.41	380.92	354.20	391.85	**347.41**
**mmu-miR-224-5p**	326.75	298.15	446.60	259.32	341.96	334.17	**334.49**
**mmu-miR-20a-5p**	211.37	397.83	291.88	384.14	332.34	358.04	**329.27**
**mmu-miR-532-5p**	463.71	278.04	343.45	242.11	291.23	280.46	**316.50**
**mmu-miR-126a-5p**	256.66	355.86	260.06	363.70	251.00	354.06	**306.89**
**mmu-miR-669o-3p**	193.03	364.60	210.68	334.65	383.94	346.10	**305.50**
**mmu-miR-708-3p**	322.44	296.40	330.28	284.07	223.89	357.04	**302.35**
**mmu-miR-542-3p**	350.48	294.65	261.16	293.76	315.72	290.41	**301.03**
**mmu-miR-17-3p**	322.44	306.02	374.18	370.16	221.27	206.86	**300.16**
**mmu-miR-133a-5p**	246.95	258.81	52.67	680.05	382.19	72.60	**282.21**
**mmu-miR-34c-5p**	216.76	232.58	323.70	366.93	263.25	270.52	**278.96**
**mmu-miR-145a-3p**	160.68	302.52	247.99	334.65	269.37	346.10	**276.89**
**mmu-miR-10b-3p**	286.85	277.17	290.78	224.89	227.39	277.48	**264.09**
**mmu-miR-429-3p**	232.93	272.80	268.84	289.45	248.38	259.58	**262.00**
**mmu-miR-10a-3p**	304.11	246.56	274.32	225.97	270.24	237.70	**259.82**
**mmu-miR-350-3p**	198.42	319.14	141.55	230.27	171.42	455.50	**252.72**
**mmu-miR-434-5p**	323.52	256.18	178.86	269.01	190.66	272.50	**248.46**
**mmu-miR-183-5p**	410.87	184.49	215.07	174.32	248.38	222.78	**242.65**
**mmu-miR-674-5p**	141.27	210.72	296.27	285.15	240.51	223.77	**232.95**
**mmu-miR-185-5p**	182.25	215.09	251.28	229.20	230.89	225.76	**222.41**

Biology Open • Supplementary information

**mmu-miR-130a-3p**	216.76	227.33	210.68	219.51	192.41	266.54	**222.21**
**mmu-miR-155-5p**	231.85	275.42	222.75	181.85	198.53	219.79	**221.70**
**mmu-miR-99b-5p**	197.35	239.57	287.49	166.79	206.40	183.99	**213.60**
**mmu-miR-141-5p**	222.15	190.61	224.94	214.13	215.15	195.92	**210.48**
**mmu-miR-3535**	292.24	180.99	226.04	174.32	191.53	172.06	**206.20**
**mmu-miR-19a-3p**	119.70	248.31	133.87	230.27	189.78	237.70	**193.27**
**mmu-miR-34a-5p**	111.07	227.33	205.19	168.94	182.79	254.60	**191.65**
**mmu-miR-361-5p**	183.33	178.37	249.08	188.31	160.05	128.30	**181.24**
**mmu-miR-484**	125.09	154.76	203.00	294.83	160.05	100.45	**173.03**
**mmu-miR-362-3p**	167.15	154.76	80.10	342.18	118.94	171.06	**172.37**
**mmu-miR-204-5p**	91.66	143.39	203.00	271.16	121.57	132.27	**160.51**
**mmu-miR-9-5p**	198.42	150.39	122.90	142.04	168.79	174.04	**159.43**
**mmu-miR-3071-3p**	110.00	187.11	109.73	171.09	146.05	198.91	**153.82**
**mmu-miR-381-3p**	163.92	172.25	116.31	188.31	116.32	165.09	**153.70**
**mmu-miR-362-5p**	142.35	152.14	119.60	259.32	104.95	125.31	**150.61**
**mmu-miR-126b-5p**	139.11	147.76	139.36	167.86	126.81	182.00	**150.48**
**mmu-miR-1839-5p**	173.62	151.26	163.50	170.01	118.07	124.32	**150.13**
**mmu-miR-466c-3p@**	107.84	187.11	108.63	157.10	179.29	159.13	**149.85**
**mmu-miR-7a-5p**	147.74	141.64	159.11	116.21	160.92	156.14	**146.96**
**mmu-miR-455-5p**	104.60	127.65	123.99	220.59	160.92	129.29	**144.51**
**mmu-miR-30a-3p**	108.92	151.26	132.77	151.72	149.55	172.06	**144.38**
**mmu-miR-500-3p**	85.19	157.38	125.09	234.58	131.19	132.27	**144.28**
**mmu-miR-378c**	187.64	140.77	123.99	152.80	107.57	110.39	**137.19**
**mmu-miR-669c-5p**	118.62	133.77	128.38	114.06	141.68	179.02	**135.92**
**mmu-miR-379-5p**	175.78	128.53	127.29	137.73	103.20	131.28	**133.97**
**mmu-miR-1198-5p**	143.43	118.04	176.66	114.06	132.06	116.36	**133.44**
**mmu-miR-411-5p**	143.43	138.15	103.15	122.67	113.69	179.02	**133.35**
**mmu-miR-872-3p**	61.47	138.15	159.11	146.34	137.31	132.27	**129.11**
**mmu-miR-100-5p**	195.19	104.92	126.19	175.39	75.21	80.56	**126.24**
**mmu-miR-324-5p**	128.33	133.77	92.17	143.11	125.94	130.28	**125.60**
**mmu-miR-203-5p**	211.37	84.81	128.38	85.01	114.57	125.31	**124.91**

Biology Open • Supplementary information

**mmu-miR-431-5p**	153.13	105.80	137.16	117.29	111.95	117.36	**123.78**
**mmu-let-7d-3p**	100.29	111.92	159.11	114.06	141.68	106.42	**122.25**
**mmu-miR-27a-5p**	84.11	101.42	165.69	145.26	128.56	103.43	**121.41**
**mmu-miR-143-5p**	59.31	132.03	126.19	139.88	118.07	143.21	**119.78**
**mmu-miR-28a-3p**	142.35	90.93	128.38	108.68	106.70	134.26	**118.55**
**mmu-miR-138-5p**	89.51	118.04	148.13	102.22	118.94	131.28	**118.02**
**mmu-miR-22-5p**	97.06	129.40	96.56	166.79	90.08	107.41	**114.55**
**mmu-miR-300-3p**	158.52	131.15	84.49	122.67	70.84	110.39	**113.01**
**mmu-miR-15b-3p**	72.25	144.27	81.20	102.22	127.69	138.24	**110.98**
**mmu-miR-31-5p**	67.94	81.31	155.82	139.88	104.07	108.41	**109.57**
**mmu-miR-140-5p**	84.11	115.41	87.78	121.59	104.07	121.33	**105.72**
**mmu-miR-345-3p**	129.41	97.93	113.02	117.29	90.08	76.58	**104.05**
**mmu-miR-127-5p**	138.03	122.41	73.52	109.76	76.09	104.43	**104.04**
**mmu-miR-674-3p**	102.45	98.80	134.97	109.76	107.57	69.62	**103.86**
**mmu-miR-574-3p**	56.08	92.68	140.45	115.14	108.45	107.41	**103.37**
**mmu-let-7i-3p**	59.31	128.53	55.96	131.28	91.83	149.18	**102.68**
**mmu-miR-214-5p**	71.17	94.43	96.56	149.57	92.70	111.39	**102.64**
**mmu-miR-106b-5p**	71.17	95.30	73.52	145.26	108.45	121.33	**102.51**
**mmu-miR-340-5p**	122.94	100.55	66.93	112.98	101.45	70.61	**95.91**
**mmu-miR-652-3p**	91.66	76.94	88.88	159.25	81.34	75.59	**95.61**
**mmu-miR-193a-5p**	62.55	93.55	137.16	74.25	83.08	122.33	**95.49**
**mmu-miR-144-3p**	47.45	114.54	52.67	112.98	78.71	120.34	**87.78**
**mmu-let-7a-1-3p**	72.25	98.80	54.86	97.92	83.08	93.49	**83.40**
**mmu-miR-20b-5p**	64.70	104.92	69.13	77.47	95.33	87.52	**83.18**
**mmu-miR-17-5p**	97.06	90.06	57.06	75.32	80.46	93.49	**82.24**
**mmu-miR-1843a-3p**	76.57	93.55	79.00	68.87	76.96	88.51	**80.58**
**mmu-miR-29a-5p**	50.68	69.07	74.62	123.74	70.84	80.56	**78.25**
**mmu-miR-186-5p**	75.49	80.44	72.42	80.70	71.72	86.53	**77.88**
**mmu-miR-376b-5p**	57.15	80.44	69.13	60.26	84.83	115.37	**77.86**
**mmu-miR-218-5p**	78.72	80.44	53.77	94.69	83.08	72.60	**77.22**
**mmu-miR-148b-3p**	76.57	78.69	50.48	91.46	70.84	90.50	**76.42**

Biology Open • Supplementary information

**mmu-miR-671-5p**	112.15	48.96	69.13	88.23	64.72	58.68	**73.65**
**mmu-miR-139-5p**	77.64	82.19	41.70	55.95	80.46	99.45	**72.90**
**mmu-miR-195a-3p**	67.94	55.08	92.17	88.23	59.47	65.64	**71.42**
**mmu-miR-29c-5p**	51.76	62.08	72.42	60.26	86.58	80.56	**68.94**
**mmu-miR-206-3p**	112.15	74.32	27.43	92.54	80.46	18.90	**67.63**
**mmu-miR-664-3p**	84.11	69.95	59.25	99.00	43.73	44.75	**66.80**
**mmu-let-7j**	71.17	66.45	60.35	65.64	61.22	73.60	**66.41**
**mmu-miR-351-5p**	69.02	61.20	81.20	49.50	62.97	59.67	**63.93**
**mmu-miR-486a-5p**	86.27	72.57	50.48	78.55	53.35	33.81	**62.51**
**mmu-miR-200b-5p**	57.15	61.20	71.32	57.03	68.22	56.69	**61.94**
**mmu-miR-378a-5p**	57.15	83.94	69.13	46.27	47.23	66.63	**61.73**
**mmu-miR-127-3p**	84.11	61.20	57.06	54.88	43.73	64.65	**60.94**
**mmu-miR-503-5p**	57.15	59.46	69.13	62.41	57.72	57.68	**60.59**
**mmu-miR-676-5p**	80.88	57.71	57.06	59.18	52.47	51.72	**59.84**
**mmu-miR-144-5p**	86.27	65.58	35.11	61.33	50.73	50.72	**58.29**
**mmu-miR-450b-3p**	30.20	69.95	73.52	53.80	56.85	61.66	**57.66**
**mmu-miR-425-3p**	29.12	46.34	82.30	72.09	62.97	39.78	**55.43**
**mmu-miR-98-5p**	34.51	62.95	51.57	47.35	57.72	76.58	**55.11**
**mmu-miR-30d-3p**	51.76	48.09	60.35	51.65	55.10	63.65	**55.10**
**mmu-miR-322-3p**	48.53	55.96	46.09	76.40	48.98	49.73	**54.28**
**mmu-miR-486a-3p@**	65.78	55.08	57.06	68.87	35.86	38.79	**53.57**
**mmu-miR-30b-3p**	48.53	55.08	53.77	49.50	45.48	50.72	**50.51**
**mmu-miR-128-3p**	44.21	58.58	48.28	53.80	51.60	45.75	**50.37**
**mmu-miR-466d-3p**	35.59	53.33	34.02	53.80	71.72	52.71	**50.20**
**mmu-miR-542-5p**	48.53	46.34	50.48	47.35	38.48	61.66	**48.81**
**mmu-miR-676-3p**	49.61	41.09	73.52	41.97	32.36	49.73	**48.05**
**mmu-miR-96-5p**	28.04	46.34	30.72	60.26	62.97	48.73	**46.18**
**mmu-miR-467a-3p@**	34.51	64.70	19.75	50.57	44.60	53.71	**44.64**
**mmu-miR-363-3p**	32.35	55.08	40.60	80.70	36.73	16.91	**43.73**
**mmu-miR-382-5p**	61.47	41.09	41.70	26.90	42.85	44.75	**43.13**
**mmu-miR-150-3p**	40.98	45.47	44.99	34.43	42.85	37.79	**41.09**

**mmu-miR-450b-5p**	44.21	42.84	37.31	41.97	34.98	37.79	**39.85**
**mmu-miR-511-3p**	36.67	35.85	38.41	64.56	27.99	31.83	**39.22**
**mmu-miR-299a-5p@**	40.98	45.47	32.92	34.43	40.23	40.78	**39.14**
**mmu-miR-18a-5p**	19.41	50.71	23.04	54.88	50.73	34.81	**38.93**
**mmu-miR-342-5p**	60.39	42.84	30.72	25.82	34.98	36.80	**38.59**
**mmu-miR-190a-5p**	25.88	34.97	31.82	49.50	42.85	45.75	**38.46**
**mmu-miR-423-3p**	52.84	35.85	43.89	35.51	33.23	27.85	**38.20**
**mmu-miR-30f**	43.14	34.97	31.82	44.12	36.73	32.82	**37.27**
**mmu-miR-335-5p**	38.82	47.21	36.21	33.36	23.61	42.77	**37.00**
**mmu-miR-106b-3p**	42.06	41.09	32.92	40.89	27.99	31.83	**36.13**
**mmu-miR-154-5p**	21.57	50.71	31.82	34.43	27.99	39.78	**34.38**
**mmu-miR-434-3p**	43.14	40.22	25.24	48.42	25.36	23.87	**34.38**
**mmu-miR-690**	25.88	23.61	34.02	40.89	45.48	31.83	**33.62**
**mmu-miR-223-5p**	42.06	21.86	47.18	33.36	35.86	18.90	**33.20**
**mmu-miR-9-3p**	18.33	30.60	34.02	27.98	40.23	46.74	**32.98**
**mmu-miR-219a-5p**	33.43	41.97	19.75	33.36	26.24	42.77	**32.92**
**mmu-miR-532-3p**	33.43	33.23	41.70	20.44	30.61	34.81	**32.37**
**mmu-miR-210-3p**	12.94	25.36	35.11	35.51	44.60	35.80	**31.55**
**mmu-miR-181c-5p**	44.21	17.49	23.04	69.94	16.62	14.92	**31.04**
**mmu-miR-672-5p**	23.72	20.98	47.18	19.37	37.61	31.83	**30.12**
**mmu-miR-345-5p**	14.02	37.60	26.33	37.66	23.61	39.78	**29.83**
**mmu-miR-331-3p**	12.94	35.85	36.21	35.51	18.37	35.80	**29.11**
**mmu-miR-378b**	23.72	27.98	31.82	30.13	26.24	29.84	**28.29**
**mmu-miR-34b-5p**	15.10	20.98	20.85	41.97	38.48	28.84	**27.70**
**mmu-miR-374c-3p**	19.41	27.10	18.65	45.19	25.36	27.85	**27.26**
**mmu-miR-34b-3p**	33.43	13.99	34.02	45.19	15.74	20.89	**27.21**
**mmu-miR-93-3p**	22.65	40.22	29.63	34.43	20.12	14.92	**27.00**
**mmu-miR-582-3p**	36.67	27.10	18.65	39.81	19.24	17.90	**26.56**
**mmu-miR-744-5p**	17.25	18.36	29.63	24.75	37.61	30.83	**26.41**
**mmu-miR-375-3p**	76.57	21.86	10.97	27.98	10.49	8.95	**26.14**
**mmu-miR-222-3p**	26.96	27.10	23.04	44.12	15.74	15.91	**25.48**

Biology Open • Supplementary information

**mmu-miR-211-5p**	14.02	39.35	16.46	33.36	23.61	23.87	**25.11**
**mmu-miR-30c-2-3p**	26.96	25.36	29.63	26.90	13.99	25.86	**24.78**
**mmu-miR-423-5p**	25.88	13.99	39.50	17.22	29.74	21.88	**24.70**
**mmu-miR-7a-1-3p**	15.10	22.73	24.14	33.36	27.11	24.86	**24.55**
**mmu-miR-33-5p**	16.18	29.73	14.26	39.81	17.49	28.84	**24.39**
**mmu-miR-186-3p**	20.49	33.23	24.14	15.06	20.12	32.82	**24.31**
**mmu-miR-409-5p**	28.04	18.36	23.04	27.98	17.49	29.84	**24.13**
**mmu-miR-5099**	47.45	13.99	16.46	32.28	19.24	11.93	**23.56**
**mmu-miR-139-3p**	12.94	15.74	43.89	18.29	23.61	20.89	**22.56**
**mmu-miR-491-5p**	36.67	23.61	26.33	17.22	8.75	19.89	**22.08**
**mmu-miR-28a-5p@**	17.25	13.99	20.85	32.28	23.61	20.89	**21.48**
**mmu-miR-467e-5p**	8.63	22.73	24.14	23.67	24.49	22.87	**21.09**
**mmu-miR-297c-3p**	18.33	23.61	10.97	23.67	22.74	26.85	**21.03**
**mmu-miR-181a-1-3p**	21.57	20.11	25.24	23.67	11.37	20.89	**20.48**
**mmu-miR-132-3p**	18.33	14.86	17.56	30.13	21.86	17.90	**20.11**
**mmu-miR-326-3p**	24.80	14.86	25.24	27.98	13.12	10.94	**19.49**
**mmu-miR-455-3p**	15.10	19.24	24.14	22.60	19.24	13.92	**19.04**
**mmu-miR-21a-3p**	18.33	22.73	19.75	24.75	14.87	9.95	**18.40**
**mmu-miR-181d-5p**	23.72	14.86	17.56	45.19	3.50	4.97	**18.30**
**mmu-miR-361-3p**	18.33	18.36	26.33	17.22	18.37	10.94	**18.26**
**mmu-miR-350-5p**	19.41	19.24	9.88	21.52	17.49	20.89	**18.07**
**mmu-miR-190b-5p**	14.02	18.36	14.26	12.91	18.37	25.86	**17.30**
**mmu-miR-497a-3p**	17.25	16.61	13.17	18.29	19.24	16.91	**16.91**
**mmu-miR-16-1-3p**	6.47	21.86	8.78	20.44	20.12	21.88	**16.59**
**mmu-miR-134-5p**	20.49	6.99	31.82	13.99	8.75	13.92	**15.99**
**mmu-miR-669f-3p**	6.47	16.61	14.26	20.44	20.12	17.90	**15.97**
**mmu-miR-3473b**	6.47	17.49	15.36	12.91	13.12	27.85	**15.53**
**mmu-miR-30e-3p**	8.63	15.74	15.36	16.14	14.87	20.89	**15.27**
**mmu-miR-1843b-3p**	15.10	14.86	14.26	16.14	17.49	11.93	**14.96**
**mmu-miR-664-5p**	25.88	13.99	19.75	4.30	11.37	12.93	**14.70**
**mmu-miR-669a-5p**	9.71	13.99	4.39	17.22	16.62	18.90	**13.47**

**mmu-miR-106a-5p**	4.31	16.61	4.39	23.67	17.49	11.93	**13.07**
**mmu-miR-200a-5p**	15.10	10.49	16.46	15.06	8.75	11.93	**12.97**
**mmu-miR-125b-1-3p**	19.41	8.74	18.65	12.91	7.00	10.94	**12.94**
**mmu-miR-340-3p**	9.71	13.12	18.65	11.84	9.62	13.92	**12.81**
**mmu-miR-1191a**	10.78	16.61	13.17	11.84	13.12	9.95	**12.58**
**mmu-miR-1291**	8.63	13.12	16.46	11.84	14.87	9.95	**12.48**
**mmu-miR-301b-3p**	14.02	16.61	8.78	7.53	9.62	16.91	**12.25**
**mmu-miR-574-5p**	10.78	12.24	13.17	8.61	17.49	8.95	**11.87**
**mmu-miR-135a-5p**	14.02	14.86	8.78	16.14	7.00	9.95	**11.79**
**mmu-miR-598-3p**	19.41	11.37	4.39	10.76	11.37	11.93	**11.54**
**mmu-miR-130b-3p**	10.78	10.49	17.56	6.46	9.62	13.92	**11.47**
**mmu-miR-136-5p**	7.55	11.37	2.19	12.91	12.24	15.91	**10.36**
**mmu-miR-665-3p**	8.63	7.87	8.78	11.84	7.87	14.92	**9.99**
**mmu-miR-34c-3p**	7.55	2.62	19.75	13.99	7.87	7.96	**9.96**
**mmu-miR-700-5p**	4.31	9.62	8.78	13.99	6.12	12.93	**9.29**
**mmu-miR-20a-3p**	15.10	6.99	2.19	12.91	6.12	11.93	**9.21**
**mmu-miR-6240**	26.96	6.99	9.88	7.53	2.62	0.99	**9.16**
**mmu-miR-1193-3p**	7.55	12.24	3.29	16.14	8.75	6.96	**9.16**
**mmu-miR-466f-3p**	4.31	8.74	12.07	11.84	12.24	4.97	**9.03**
**mmu-miR-328-3p**	6.47	13.12	10.97	1.08	11.37	8.95	**8.66**
**mmu-miR-701-5p**	12.94	4.37	3.29	8.61	10.49	11.93	**8.61**
**mmu-miR-409-3p**	9.71	6.12	13.17	4.30	9.62	7.96	**8.48**
**mmu-miR-3071-5p**	9.71	13.99	1.10	9.68	5.25	10.94	**8.45**
**mmu-miR-672-3p**	4.31	11.37	12.07	10.76	7.00	4.97	**8.41**
**mmu-miR-503-3p**	5.39	12.24	9.88	4.30	8.75	8.95	**8.25**
**mmu-miR-450a-5p**	2.16	11.37	7.68	10.76	7.00	8.95	**7.99**
**mmu-miR-450a-1-3p**	9.71	4.37	4.39	5.38	5.25	17.90	**7.83**
**mmu-miR-1981-5p**	12.94	4.37	8.78	2.15	8.75	9.95	**7.82**
**mmu-miR-3068-3p**	4.31	6.12	4.39	11.84	7.00	12.93	**7.77**
**mmu-miR-181c-3p**	7.55	4.37	10.97	15.06	6.12	1.99	**7.68**
**mmu-miR-376c-3p**	6.47	10.49	3.29	5.38	8.75	9.95	**7.39**

Biology Open • Supplementary information

**mmu-miR-374b-5p@**	6.47	9.62	3.29	17.22	2.62	4.97	**7.37**
**mmu-miR-1306-5p**	10.78	9.62	4.39	5.38	7.87	5.97	**7.34**
**mmu-miR-5121**	11.86	5.25	5.49	3.23	7.87	9.95	**7.28**
**mmu-miR-15a-3p**	7.55	10.49	6.58	7.53	4.37	6.96	**7.25**
**mmu-miR-421-3p**	8.63	6.12	5.49	6.46	9.62	6.96	**7.21**
**mmu-miR-16-2-3p**	4.31	14.86	2.19	7.53	5.25	8.95	**7.18**
**mmu-miR-187-3p**	4.31	7.87	7.68	8.61	6.12	6.96	**6.93**
**mmu-miR-181a-2-3p**	7.55	6.99	4.39	9.68	4.37	6.96	**6.66**
**mmu-miR-668-3p**	7.55	6.12	8.78	5.38	6.12	5.97	**6.65**
**mmu-miR-467c-5p**	1.08	8.74	10.97	2.15	5.25	10.94	**6.52**
**mmu-let-7g-3p**	5.39	4.37	0.00	11.84	3.50	12.93	**6.34**
**mmu-miR-124-3p**	2.16	4.37	2.19	21.52	1.75	5.97	**6.33**
**mmu-miR-485-5p**	4.31	3.50	9.88	5.38	7.87	6.96	**6.32**
**mmu-miR-29b-2-5p**	10.78	5.25	9.88	4.30	2.62	4.97	**6.30**
**mmu-miR-369-3p**	5.39	8.74	1.10	10.76	1.75	9.95	**6.28**
**mmu-miR-1a-1-5p**	7.55	9.62	1.10	13.99	4.37	0.99	**6.27**
**mmu-miR-221-5p**	6.47	6.12	5.49	5.38	8.75	4.97	**6.20**
**mmu-miR-3473a**	5.39	6.12	4.39	7.53	9.62	3.98	**6.17**
**mmu-miR-337-5p**	1.08	7.87	3.29	4.30	10.49	9.95	**6.16**
**mmu-miR-34a-3p**	1.08	8.74	4.39	7.53	6.12	8.95	**6.14**
**mmu-miR-99b-3p**	5.39	6.12	3.29	6.46	7.00	7.96	**6.04**
**mmu-miR-1981-3p**	6.47	4.37	7.68	3.23	6.12	7.96	**5.97**
**mmu-miR-501-5p**	6.47	4.37	4.39	15.06	2.62	1.99	**5.82**
**mmu-miR-3544-3p**	6.47	4.37	6.58	3.23	6.12	7.96	**5.79**
**mmu-miR-369-5p**	2.16	2.62	13.17	4.30	7.87	3.98	**5.68**
**mmu-miR-700-3p**	7.55	1.75	8.78	5.38	7.00	2.98	**5.57**
**mmu-miR-376b-3p**	4.31	6.12	4.39	5.38	6.12	6.96	**5.55**
**mmu-miR-27b-5p**	1.08	4.37	3.29	15.06	6.12	2.98	**5.48**
**mmu-miR-511-5p**	3.24	5.25	6.58	6.46	5.25	5.97	**5.46**
**mmu-miR-125a-3p**	6.47	6.12	2.19	3.23	8.75	5.97	**5.46**
**mmu-miR-149-5p**	2.16	7.87	3.29	3.23	8.75	6.96	**5.38**

Biology Open • Supplementary information

**mmu-miR-152-5p**	4.31	4.37	7.68	2.15	5.25	7.96	**5.29**
**mmu-miR-135b-5p**	5.39	6.99	7.68	5.38	3.50	1.99	**5.16**
**mmu-miR-466h-3p**	10.78	1.75	4.39	6.46	4.37	2.98	**5.12**
**mmu-miR-341-3p**	4.31	6.12	4.39	6.46	4.37	4.97	**5.10**
**mmu-miR-29b-1-5p**	1.08	4.37	5.49	3.23	11.37	4.97	**5.09**
**mmu-miR-299a-3p@**	8.63	1.75	4.39	5.38	4.37	5.97	**5.08**
**mmu-miR-137-3p**	4.31	5.25	5.49	2.15	5.25	7.96	**5.07**
**mmu-miR-216a-5p**	3.24	6.99	2.19	6.46	6.12	4.97	**5.00**
**mmu-miR-183-3p**	4.31	5.25	9.88	3.23	6.12	0.99	**4.96**
**mmu-miR-18a-3p**	5.39	2.62	4.39	8.61	3.50	4.97	**4.91**
**mmu-miR-324-3p**	3.24	3.50	5.49	7.53	5.25	3.98	**4.83**
**mmu-miR-92a-1-5p**	2.16	5.25	7.68	4.30	4.37	4.97	**4.79**
**mmu-miR-450a-2-3p**	2.16	5.25	4.39	6.46	6.12	3.98	**4.73**
**mmu-miR-330-5p**	2.16	8.74	6.58	0.00	7.87	2.98	**4.72**
**mmu-miR-193b-3p**	7.55	2.62	5.49	9.68	0.87	1.99	**4.70**
**mmu-miR-452-3p**	4.31	4.37	3.29	3.23	7.00	5.97	**4.70**
**mmu-miR-132-5p**	3.24	2.62	5.49	7.53	5.25	3.98	**4.69**
**mmu-miR-193b-5p**	6.47	1.75	4.39	1.08	6.12	7.96	**4.63**
**mmu-miR-148b-5p**	7.55	4.37	2.19	7.53	1.75	3.98	**4.56**
**mmu-miR-188-5p**	1.08	1.75	4.39	10.76	4.37	4.97	**4.55**
**mmu-miR-376a-5p**	2.16	6.12	2.19	7.53	1.75	6.96	**4.45**
**mmu-miR-485-3p**	6.47	1.75	5.49	2.15	7.00	2.98	**4.31**
**mmu-miR-3084-3p**	5.39	5.25	1.10	7.53	3.50	2.98	**4.29**
**mmu-miR-466i-3p**	2.16	6.99	3.29	2.15	7.00	3.98	**4.26**
**mmu-let-7b-3p**	4.31	4.37	5.49	2.15	5.25	3.98	**4.26**
**mmu-miR-467d-5p**	1.08	3.50	4.39	10.76	1.75	3.98	**4.24**
**mmu-miR-1941-3p**	6.47	3.50	4.39	3.23	5.25	1.99	**4.14**
**mmu-miR-344b-3p**	2.16	4.37	4.39	4.30	2.62	6.96	**4.13**
**mmu-let-7k**	3.24	2.62	4.39	4.30	5.25	4.97	**4.13**
**mmu-miR-339-3p**	1.08	4.37	5.49	5.38	5.25	2.98	**4.09**
**mmu-miR-6238**	11.86	4.37	3.29	3.23	1.75	0.00	**4.08**

Biology Open • Supplementary information

**mmu-miR-1839-3p**	1.08	5.25	0.00	8.61	5.25	3.98	**4.03**
**mmu-miR-181b-1-3p**	2.16	5.25	3.29	2.15	5.25	5.97	**4.01**
**mmu-miR-215-5p**	3.24	2.62	1.10	8.61	3.50	4.97	**4.01**
**mmu-miR-615-3p**	3.24	3.50	4.39	3.23	5.25	2.98	**3.77**
**mmu-miR-335-3p**	7.55	0.87	4.39	3.23	3.50	2.98	**3.75**
**mmu-miR-3064-5p**	1.08	8.74	3.29	1.08	5.25	2.98	**3.74**
**mmu-miR-384-3p**	0.00	4.37	2.19	3.23	3.50	8.95	**3.71**
**mmu-miR-181b-2-3p**	1.08	1.75	5.49	2.15	6.12	4.97	**3.59**
**mmu-miR-147-3p**	2.16	6.12	1.10	4.30	3.50	3.98	**3.53**
**mmu-miR-3058-5p**	2.16	1.75	6.58	3.23	4.37	2.98	**3.51**
**mmu-miR-673-5p**	3.24	4.37	5.49	3.23	1.75	2.98	**3.51**
**mmu-miR-3544-5p**	3.24	4.37	5.49	1.08	2.62	3.98	**3.46**
**mmu-miR-329-5p**	2.16	6.12	3.29	4.30	0.87	3.98	**3.45**
**mmu-miR-296-3p**	1.08	2.62	4.39	5.38	3.50	2.98	**3.33**
**mmu-miR-298-5p**	4.31	6.12	2.19	4.30	0.00	2.98	**3.32**
**mmu-miR-669a-3-3p**	3.24	3.50	2.19	3.23	1.75	5.97	**3.31**
**mmu-miR-494-3p**	4.31	3.50	2.19	2.15	4.37	2.98	**3.25**
**mmu-miR-488-3p**	5.39	6.12	2.19	2.15	2.62	0.99	**3.24**
**mmu-miR-671-3p**	6.47	1.75	3.29	1.08	2.62	3.98	**3.20**
**mmu-miR-493-5p**	3.24	2.62	0.00	6.46	0.87	5.97	**3.19**
**mmu-miR-190a-3p**	2.16	4.37	1.10	5.38	0.87	4.97	**3.14**
**mmu-miR-5126**	3.24	4.37	2.19	5.38	2.62	0.99	**3.13**
**mmu-miR-874-5p**	4.31	5.25	4.39	0.00	3.50	0.99	**3.07**
**mmu-miR-30c-1-3p**	3.24	2.62	2.19	2.15	5.25	2.98	**3.07**
**mmu-miR-501-3p**	5.39	4.37	2.19	3.23	0.87	1.99	**3.01**
**mmu-miR-20b-3p**	4.31	1.75	1.10	3.23	3.50	3.98	**2.98**
**mmu-miR-3057-5p**	5.39	0.00	5.49	1.08	1.75	3.98	**2.95**
**mmu-miR-1249-3p**	2.16	5.25	2.19	3.23	2.62	1.99	**2.91**
**mmu-miR-466h-5p**	0.00	3.50	5.49	1.08	4.37	2.98	**2.90**
**mmu-miR-5107-3p**	7.55	3.50	3.29	1.08	0.00	1.99	**2.90**
**mmu-miR-499-5p**	1.08	4.37	3.29	1.08	3.50	3.98	**2.88**

**mmu-miR-26a-2-3p**	4.31	1.75	2.19	4.30	2.62	1.99	**2.86**
**mmu-miR-466i-5p**	1.08	2.62	2.19	5.38	1.75	3.98	**2.83**
**mmu-miR-466m-5p@**	2.16	2.62	3.29	2.15	3.50	2.98	**2.78**
**mmu-miR-467a-5p@**	1.08	4.37	2.19	3.23	2.62	2.98	**2.75**
**mmu-miR-99a-3p**	0.00	5.25	0.00	5.38	0.87	4.97	**2.75**
**mmu-miR-3065-3p**	1.08	3.50	5.49	0.00	4.37	1.99	**2.74**
**mmu-miR-3066-5p**	2.16	4.37	1.10	2.15	2.62	3.98	**2.73**
**mmu-miR-218-2-3p**	2.16	2.62	2.19	6.46	0.87	1.99	**2.72**
**mmu-miR-653-5p**	5.39	0.87	2.19	4.30	3.50	0.00	**2.71**
**mmu-miR-466k**	0.00	4.37	0.00	4.30	4.37	2.98	**2.67**
**mmu-miR-379-3p**	4.31	1.75	0.00	2.15	3.50	3.98	**2.62**
**mmu-miR-136-3p**	2.16	1.75	1.10	1.08	0.87	7.96	**2.49**
**mmu-miR-5107-5p**	4.31	3.50	0.00	3.23	0.87	2.98	**2.48**
**mmu-miR-33-3p**	2.16	2.62	1.10	3.23	1.75	3.98	**2.47**
**mmu-miR-6541**	2.16	0.87	2.19	1.08	4.37	3.98	**2.44**
**mmu-let-7e-3p**	2.16	4.37	2.19	1.08	1.75	2.98	**2.42**
**mmu-miR-217-5p**	7.55	1.75	0.00	1.08	2.62	0.99	**2.33**
**mmu-miR-330-3p**	4.31	0.87	1.10	2.15	3.50	1.99	**2.32**
**mmu-miR-1968-5p**	3.24	1.75	2.19	2.15	3.50	0.99	**2.30**
**mmu-miR-744-3p**	3.24	3.50	2.19	0.00	0.87	3.98	**2.30**
**mmu-miR-2137**	3.24	2.62	2.19	2.15	3.50	0.00	**2.28**
**mmu-miR-130b-5p**	0.00	3.50	3.29	3.23	2.62	0.99	**2.27**
**mmu-miR-26b-3p**	1.08	3.50	3.29	2.15	3.50	0.00	**2.25**
**mmu-miR-666-3p**	1.08	4.37	2.19	2.15	2.62	0.99	**2.23**
**mmu-miR-205-3p**	0.00	1.75	2.19	4.30	1.75	2.98	**2.16**
**mmu-miR-3108-5p**	2.16	2.62	2.19	2.15	0.87	2.98	**2.16**
**mmu-miR-376a-3p**	0.00	1.75	0.00	3.23	1.75	5.97	**2.12**
**mmu-miR-146b-3p**	3.24	0.00	4.39	2.15	0.87	1.99	**2.11**
**mmu-miR-6236**	2.16	0.87	5.49	3.23	0.87	0.00	**2.10**
**mmu-miR-1943-5p**	4.31	0.00	2.19	2.15	3.50	0.00	**2.03**
**mmu-miR-466q**	1.08	3.50	0.00	1.08	3.50	2.98	**2.02**

Biology Open • Supplementary information

**mmu-miR-874-3p**	3.24	1.75	2.19	1.08	0.87	2.98	**2.02**
**mmu-miR-466m-3p**	1.08	3.50	0.00	1.08	4.37	1.99	**2.00**
**mmu-miR-5709-5p**	1.08	2.62	1.10	3.23	1.75	1.99	**1.96**
**mmu-miR-148a-5p**	1.08	0.00	2.19	5.38	0.00	2.98	**1.94**
**mmu-miR-344-3p**	3.24	4.37	0.00	2.15	0.87	0.99	**1.94**
**mmu-miR-3076-3p**	2.16	1.75	1.10	0.00	4.37	1.99	**1.90**
**mmu-miR-877-5p**	4.31	0.00	1.10	1.08	0.87	3.98	**1.89**
**mmu-let-7f-1-3p**	1.08	4.37	0.00	3.23	2.62	0.00	**1.88**
**mmu-miR-191-3p**	1.08	2.62	1.10	1.08	4.37	0.99	**1.87**
**mmu-miR-1955-3p**	0.00	3.50	1.10	1.08	3.50	1.99	**1.86**
**mmu-miR-5114**	3.24	0.87	1.10	1.08	0.87	3.98	**1.86**
**mmu-miR-669b-5p**	1.08	1.75	0.00	4.30	1.75	1.99	**1.81**
**mmu-miR-669p-3p**	3.24	2.62	1.10	2.15	1.75	0.00	**1.81**
**mmu-miR-101b-5p**	0.00	2.62	2.19	1.08	0.87	3.98	**1.79**
**mmu-miR-582-5p**	0.00	2.62	1.10	3.23	1.75	1.99	**1.78**
**mmu-miR-329-3p**	2.16	3.50	0.00	2.15	0.87	1.99	**1.78**
**mmu-miR-1198-3p**	1.08	0.87	3.29	4.30	0.87	0.00	**1.74**
**mmu-miR-3083-5p**	4.31	0.87	2.19	2.15	0.87	0.00	**1.73**
**mmu-miR-449a-5p**	1.08	1.75	1.10	1.08	4.37	0.99	**1.73**
**mmu-miR-6516-5p**	1.08	3.50	0.00	2.15	3.50	0.00	**1.71**
**mmu-miR-382-3p**	1.08	2.62	3.29	2.15	0.00	0.99	**1.69**
**mmu-miR-652-5p**	1.08	0.00	0.00	5.38	2.62	0.99	**1.68**
**mmu-miR-378d**	2.16	1.75	0.00	3.23	0.87	1.99	**1.67**
**mmu-miR-540-3p**	2.16	0.87	2.19	1.08	3.50	0.00	**1.63**
**mmu-miR-677-5p**	3.24	0.00	2.19	3.23	0.87	0.00	**1.59**
**mmu-miR-3968**	2.16	0.00	2.19	2.15	1.75	0.99	**1.54**
**mmu-miR-1306-3p**	1.08	1.75	1.10	3.23	0.87	0.99	**1.50**
**mmu-miR-219a-1-3p**	2.16	1.75	1.10	2.15	0.87	0.99	**1.50**
**mmu-miR-497b**	2.16	1.75	0.00	3.23	0.87	0.99	**1.50**
**mmu-miR-3069-5p**	2.16	0.87	1.10	0.00	0.87	3.98	**1.50**
**mmu-miR-411-3p**	2.16	1.75	1.10	2.15	1.75	0.00	**1.49**

Biology Open • Supplementary information

**mmu-miR-467e-3p**	1.08	1.75	0.00	2.15	0.87	2.98	**1.47**
**mmu-miR-138-1-3p**	2.16	0.87	1.10	1.08	2.62	0.99	**1.47**
**mmu-miR-196a-2-3p**	1.08	0.00	1.10	0.00	3.50	2.98	**1.44**
**mmu-miR-3084-5p**	0.00	0.00	1.10	1.08	3.50	2.98	**1.44**
**mmu-miR-467c-3p**	0.00	0.00	1.10	1.08	3.50	2.98	**1.44**
**mmu-miR-31-3p**	2.16	3.50	0.00	0.00	0.00	2.98	**1.44**
**mmu-miR-5113**	2.16	1.75	0.00	0.00	1.75	2.98	**1.44**
**mmu-let-7c-1-3p**	2.16	2.62	0.00	1.08	1.75	0.99	**1.43**
**mmu-miR-101a-5p**	0.00	3.50	0.00	1.08	0.00	3.98	**1.43**
**mmu-let-7f-2-3p**	1.08	0.00	3.29	1.08	0.00	2.98	**1.41**
**mmu-miR-25-5p**	3.24	0.87	2.19	0.00	0.87	0.99	**1.36**
**mmu-miR-7010-5p**	0.00	0.87	3.29	1.08	0.87	1.99	**1.35**
**mmu-miR-195b**	1.08	0.87	2.19	1.08	0.87	1.99	**1.35**
**mmu-miR-218-1-3p**	1.08	1.75	1.10	2.15	0.00	1.99	**1.35**
**mmu-miR-18b-5p**	3.24	1.75	0.00	1.08	0.00	1.99	**1.34**
**mmu-miR-3060-3p**	2.16	0.87	1.10	1.08	1.75	0.99	**1.33**
**mmu-miR-377-3p**	1.08	3.50	1.10	1.08	0.00	0.99	**1.29**
**mmu-miR-466c-5p**	1.08	1.75	1.10	0.00	1.75	1.99	**1.28**
**mmu-miR-541-3p**	1.08	2.62	0.00	2.15	1.75	0.00	**1.27**
**mmu-miR-129b-3p**	6.47	0.00	1.10	0.00	0.00	0.00	**1.26**
**mmu-miR-5123**	0.00	1.75	0.00	1.08	1.75	2.98	**1.26**
**mmu-miR-3105-5p**	1.08	0.00	1.10	4.30	0.00	0.99	**1.25**
**mmu-miR-1948-3p**	3.24	0.00	0.00	3.23	0.87	0.00	**1.22**
**mmu-miR-1964-3p**	1.08	0.00	3.29	1.08	0.87	0.99	**1.22**
**mmu-miR-26a-1-3p**	2.16	0.00	1.10	2.15	0.87	0.99	**1.21**
**mmu-miR-3064-3p**	1.08	0.87	1.10	2.15	0.00	1.99	**1.20**
**mmu-miR-193a-3p**	2.16	0.00	0.00	2.15	0.87	1.99	**1.20**
**mmu-miR-98-3p**	1.08	0.87	1.10	3.23	0.87	0.00	**1.19**
**mmu-miR-873a-5p**	0.00	1.75	1.10	3.23	0.00	0.99	**1.18**
**mmu-miR-669b-3p**	0.00	3.50	1.10	0.00	0.00	1.99	**1.10**
**mmu-miR-147-5p**	0.00	0.87	0.00	1.08	2.62	1.99	**1.09**

Biology Open • Supplementary information

**mmu-miR-466a-5p@**	0.00	4.37	0.00	1.08	0.00	0.99	**1.07**
**mmu-miR-380-5p**	3.24	0.00	2.19	0.00	0.00	0.99	**1.07**
**mmu-miR-212-5p**	0.00	0.00	2.19	3.23	0.00	0.99	**1.07**
**mmu-miR-188-3p**	2.16	0.00	1.10	1.08	0.87	0.99	**1.03**
**mmu-miR-666-5p**	1.08	0.87	2.19	1.08	0.87	0.00	**1.02**
**mmu-miR-466n-3p**	1.08	0.87	0.00	2.15	0.00	1.99	**1.02**
**mmu-miR-129-2-3p**	0.00	0.00	3.29	0.00	1.75	0.99	**1.01**
**mmu-miR-187-5p**	1.08	0.00	2.19	0.00	1.75	0.99	**1.00**
**mmu-miR-496a-3p**	1.08	0.87	2.19	0.00	0.87	0.99	**1.00**
**mmu-miR-7043-3p**	1.08	0.87	1.10	1.08	0.87	0.99	**1.00**
**mmu-miR-200c-5p**	1.08	0.00	0.00	2.15	1.75	0.99	**1.00**

Table S2. miRNA expressed in the mammary gland at day 18 of gestation. The number of reads determined by the sequencing is indi[illegible]d for each of the animal, as well as the average of the group (N=6). Only miRNA with an average read for the group above 1 are listed.

miRNA	P18_1	P18_2	P18_3	P18_4	P18_5	P18_6	Average expression
**mmu-let-7c-5p**	144548.96	146169.20	154081.31	173046.09	146981.33	129438.07	**149044.16**
**mmu-miR-143-3p**	142024.92	147649.04	138816.70	137782.63	152316.82	154585.47	**145529.26**
**mmu-miR-16-5p**	115300.86	99334.33	111016.28	94342.14	105950.31	101826.37	**104628.38**
**mmu-let-7a-5p**	81036.27	77212.40	78113.10	88267.13	78477.30	67213.77	**78386.66**
**mmu-miR-199a-3p**	54938.90	45433.74	53606.19	49015.48	57853.65	45092.70	**50990.11**
**mmu-miR-103-3p**	37254.31	42573.48	35183.39	35282.99	34848.47	35464.29	**36767.82**
**mmu-miR-200a-3p**	33273.11	38142.26	27361.43	35199.47	32248.10	39510.13	**34289.08**
**mmu-miR-146b-5p**	30840.78	33488.39	33963.37	32692.90	27348.58	28148.45	**31080.41**
**mmu-miR-142b**	24514.88	30577.39	31570.22	13600.17	31165.04	38450.40	**28313.02**
**mmu-miR-27b-3p**	24882.74	27695.39	24862.79	27084.28	28775.78	28681.77	**26997.13**
**mmu-miR-148a-3p**	19935.54	20584.11	20799.97	24827.16	21215.81	20613.08	**21329.28**
**mmu-miR-126a-3p**	14704.04	15572.96	15090.93	15534.06	15468.38	16133.93	**15417.38**
**mmu-miR-200b-3p**	13596.40	15439.37	13753.70	15191.32	13934.76	14275.37	**14365.15**
**mmu-let-7f-5p**	14058.00	13489.39	15454.27	15150.10	13609.49	13863.89	**14270.86**
**mmu-miR-142a-5p**	10111.45	15383.45	17735.54	4771.28	16136.06	19411.97	**13924.96**
**mmu-let-7b-5p**	15615.02	12236.34	14607.18	16177.25	12403.71	11042.15	**13680.28**
**mmu-miR-21a-5p**	13499.59	11165.56	12618.93	14116.45	11799.53	15979.91	**13196.66**
**mmu-miR-29a-3p**	9761.93	12980.92	14812.83	8079.40	10014.45	16200.59	**11975.02**
**mmu-miR-25-3p**	12731.27	10242.86	9085.67	11148.90	10923.30	9661.74	**10632.29**
**mmu-miR-125b-5p**	13047.16	9257.00	11330.71	12114.22	9745.83	6822.75	**10386.28**
**mmu-miR-23a-3p**	8833.63	10093.74	7313.71	8989.41	8361.54	9289.33	**8813.56**
**mmu-miR-146a-5p**	8393.43	6474.41	8295.05	7313.66	8030.27	9162.90	**7944.95**
**mmu-miR-101a-3p@**	6039.56	7878.65	8412.26	6645.52	8293.74	9127.27	**7732.83**
**mmu-miR-200c-3p**	6864.94	7251.09	5924.28	7760.52	5506.28	6598.62	**6650.96**
**mmu-miR-203-3p**	7398.89	6177.20	6243.93	6611.90	6854.52	6113.58	**6566.67**
**mmu-miR-10a-5p**	6710.06	5783.68	7052.66	7219.29	4939.86	5452.69	**6193.04**
**mmu-miR-93-5p**	6313.67	5669.77	4965.31	5319.02	5827.25	6012.44	**5684.58**
**mmu-miR-3074-5p**	5000.19	6253.83	5061.21	5864.59	5812.66	5884.85	**5646.22**
**mmu-miR-125a-5p**	7269.48	4870.30	5783.63	6873.30	4998.21	3489.53	**5547.41**

**mmu-miR-195a-5p**	6049.75	4960.40	5429.88	5371.08	5500.27	4984.89	**5382.71**
**mmu-miR-26a-5p**	5083.75	4464.36	4687.21	4920.96	4491.01	4156.18	**4633.91**
**mmu-let-7i-5p**	4693.47	4263.46	4785.24	4846.12	4670.38	4375.71	**4605.73**
**mmu-miR-15b-5p**	4032.15	5416.05	4159.78	3717.02	5120.08	4872.25	**4552.89**
**mmu-miR-30a-5p**	4056.60	4231.36	3967.99	4077.12	4222.39	4575.70	**4188.53**
**mmu-let-7e-5p**	4365.36	4096.73	4324.94	4762.61	3946.91	3430.92	**4154.58**
**mmu-miR-23b-3p**	2972.40	3431.89	2656.34	3262.56	3090.41	3183.80	**3099.57**
**mmu-miR-199a-5p**	2656.51	3092.22	2468.80	2436.08	3191.68	3494.13	**2889.90**
**mmu-miR-101b-3p**	2132.75	2815.73	2550.85	2215.90	3474.03	3276.90	**2744.36**
**mmu-miR-26b-5p**	2672.81	2684.21	2736.25	2683.37	2766.01	2460.83	**2667.25**
**mmu-miR-30d-5p**	2957.11	2464.67	2730.92	2662.76	2359.22	2435.55	**2601.71**
**mmu-miR-30e-5p**	2339.60	2720.45	2676.58	2028.26	2526.57	3217.13	**2584.77**
**mmu-let-7d-5p**	2237.70	2642.78	1979.73	2536.95	2376.38	2318.31	**2348.64**
**mmu-miR-196a-5p**	2487.36	1952.06	2705.35	2582.50	2205.60	2111.42	**2340.72**
**mmu-miR-19b-3p**	2518.95	2097.04	1309.52	2152.99	2120.64	3471.14	**2278.38**
**mmu-miR-182-5p**	2166.37	1888.89	2109.72	2324.36	1964.44	1774.65	**2038.07**
**mmu-miR-322-5p**	1955.44	2100.14	1894.49	1612.84	1910.38	2333.25	**1967.76**
**mmu-let-7g-5p**	1759.80	2197.49	1500.25	1875.32	1912.95	2116.02	**1893.64**
**mmu-miR-142a-3p**	1501.99	1980.02	1989.32	881.80	2042.54	2471.18	**1811.14**
**mmu-miR-221-3p**	1994.17	1943.77	1885.97	1816.75	1534.48	1575.81	**1791.83**
**mmu-miR-342-3p**	1978.88	1618.60	1999.98	1619.35	1508.73	1273.52	**1666.51**
**mmu-miR-141-3p**	1151.46	1836.07	1185.92	1297.22	1610.00	1765.46	**1474.36**
**mmu-miR-140-3p**	1511.16	1508.83	1628.11	1189.84	1385.15	1576.96	**1466.68**
**mmu-miR-126b-3p**	1255.40	1400.10	950.44	1337.35	1266.72	1933.27	**1357.21**
**mmu-miR-30c-5p**	1160.63	1574.07	1009.05	1390.49	1263.29	1590.75	**1331.38**
**mmu-miR-1b-5p**	226.22	143.94	246.13	3320.05	974.93	2744.73	**1276.00**
**mmu-miR-223-3p**	977.21	1286.18	1242.39	1249.49	1125.97	1303.40	**1197.44**
**mmu-miR-320-3p**	1526.45	936.16	908.89	1562.95	915.71	1173.52	**1170.61**
**mmu-miR-107-3p**	1089.30	1377.31	1023.96	1047.75	1076.20	1291.91	**1151.07**
**mmu-miR-145a-5p**	1048.54	1212.66	679.80	1188.75	976.64	1571.21	**1112.93**
**mmu-miR-191-5p**	1156.55	1039.72	1249.85	1041.24	1032.43	928.70	**1074.75**

Biology Open • Supplementary information

**mmu-miR-141-5p**	1155.54	1014.86	1052.73	1065.11	1137.13	901.12	**1054.42**
**mmu-miR-181b-5p**	1425.57	1055.25	1142.23	1057.51	781.83	855.14	**1052.92**
**mmu-miR-192-5p**	678.65	801.53	834.30	665.96	844.48	2260.84	**1014.29**
**mmu-miR-872-5p**	1055.67	977.58	1127.32	876.38	829.03	874.68	**956.78**
**mmu-miR-183-5p**	1054.66	910.27	1025.03	1030.40	960.34	743.65	**954.06**
**mmu-miR-196b-5p**	1003.71	773.57	1067.65	995.69	919.14	890.77	**941.76**
**mmu-miR-497a-5p**	836.59	1006.58	659.56	783.10	779.25	1386.16	**908.54**
**mmu-miR-29c-3p**	623.62	893.70	1351.08	584.62	728.62	1118.35	**883.33**
**mmu-miR-429-3p**	838.63	864.70	598.82	822.15	827.31	918.36	**811.66**
**mmu-miR-15a-5p**	710.24	934.09	676.60	741.89	712.31	1040.19	**802.55**
**mmu-miR-133a-3p**	193.61	167.76	176.88	1912.20	492.61	1704.54	**774.60**
**mmu-miR-301a-3p**	679.67	946.51	509.32	621.49	736.34	903.42	**732.79**
**mmu-miR-20a-5p**	757.11	883.34	500.79	642.10	726.05	855.14	**727.42**
**mmu-miR-425-5p**	718.39	685.55	797.01	604.14	585.30	685.03	**679.24**
**mmu-miR-541-5p**	746.92	544.71	776.76	662.71	647.95	526.42	**650.91**
**mmu-miR-181a-5p**	786.66	653.45	723.49	677.89	471.16	490.79	**633.91**
**mmu-miR-152-3p**	592.03	632.74	588.17	556.41	618.77	629.86	**603.00**
**mmu-miR-92a-3p**	881.43	520.89	500.79	709.35	459.14	487.34	**593.16**
**mmu-miR-194-5p**	452.43	545.75	449.65	439.27	496.90	1072.38	**576.06**
**mmu-miR-10b-5p**	674.57	540.57	633.98	635.59	450.56	491.94	**571.20**
**mmu-miR-24-3p**	467.72	581.99	510.38	592.21	554.40	570.10	**546.13**
**mmu-miR-151-5p**	363.78	606.85	320.72	638.85	505.49	711.47	**524.53**
**mmu-miR-378a-3p**	584.90	500.18	560.46	529.30	357.02	534.46	**511.05**
**mmu-miR-214-3p**	565.54	537.46	404.90	588.95	457.43	482.74	**506.17**
**mmu-miR-542-3p**	451.41	469.12	558.33	532.55	472.02	474.70	**493.02**
**mmu-miR-27a-3p**	427.98	603.74	350.56	460.97	509.78	603.43	**492.74**
**mmu-miR-451a**	359.70	700.05	316.46	277.67	387.91	805.72	**474.59**
**mmu-miR-205-5p**	569.62	497.08	392.11	460.97	337.28	506.88	**460.66**
**mmu-miR-151-3p**	611.39	370.74	555.13	548.82	358.73	294.24	**456.51**
**mmu-miR-204-5p**	380.08	448.40	309.00	519.54	356.16	487.34	**416.75**
**mmu-miR-1843a-5p@**	384.16	403.87	382.52	465.31	303.81	333.32	**378.83**

Biology Open • Supplementary information

**mmu-miR-32-5p**	258.82	432.87	358.01	260.31	380.19	543.66	**372.31**
**mmu-miR-122-5p**	2.04	2.07	1.07	1.08	0.00	2062.00	**344.71**
**mmu-miR-10b-3p**	291.43	327.24	310.07	362.27	330.41	339.07	**326.75**
**mmu-miR-145a-3p**	273.09	415.27	279.17	306.95	297.80	368.95	**323.54**
**mmu-miR-34c-5p**	320.98	358.31	255.72	265.73	306.38	335.62	**307.12**
**mmu-miR-210-3p**	228.25	397.66	273.84	321.05	318.40	280.45	**303.28**
**mmu-miR-150-5p**	328.12	318.96	392.11	126.90	314.10	267.81	**291.33**
**mmu-miR-31-5p**	264.94	350.02	255.72	249.46	303.81	275.85	**283.30**
**mmu-miR-434-5p**	257.80	285.82	279.17	258.14	289.22	296.54	**277.78**
**mmu-miR-17-3p**	365.82	300.32	289.82	244.04	195.67	265.51	**276.86**
**mmu-miR-674-5p**	195.65	306.53	194.99	276.58	309.81	294.24	**262.97**
**mmu-miR-34a-5p**	269.01	284.78	210.97	271.16	284.93	245.97	**261.14**
**mmu-miR-29b-3p**	199.72	310.67	232.28	181.13	288.36	342.52	**259.11**
**mmu-miR-30b-5p**	230.29	304.46	165.16	237.53	233.43	380.45	**258.55**
**mmu-miR-99a-5p**	386.20	254.75	273.84	239.70	151.90	219.53	**254.32**
**mmu-miR-138-5p**	266.98	249.57	220.56	260.31	217.13	265.51	**246.68**
**mmu-miR-224-5p**	273.09	235.08	254.66	194.15	240.30	216.08	**235.56**
**mmu-miR-143-5p**	196.67	280.64	153.43	240.79	236.01	295.39	**233.82**
**mmu-miR-125b-2-3p**	204.82	246.47	226.96	231.03	238.58	242.52	**231.73**
**mmu-miR-199b-5p**	147.75	248.54	174.74	189.81	229.14	375.85	**227.64**
**mmu-miR-19a-3p**	216.03	238.18	193.92	211.50	210.26	288.50	**226.40**
**mmu-miR-3068-5p**	169.15	242.32	193.92	242.96	213.69	263.21	**220.88**
**mmu-miR-200b-5p**	296.53	167.76	265.31	202.83	238.58	140.23	**218.54**
**mmu-miR-96-5p**	197.68	271.32	171.55	201.74	175.93	232.18	**208.40**
**mmu-miR-7a-5p**	196.67	193.65	222.69	162.69	228.28	234.47	**206.41**
**mmu-miR-708-5p**	151.83	225.76	214.17	221.26	230.86	191.95	**205.97**
**mmu-miR-532-5p**	206.86	167.76	258.92	228.86	181.94	177.01	**203.56**
**mmu-miR-339-5p**	178.32	223.68	192.86	199.57	156.19	237.92	**198.09**
**mmu-miR-106b-5p**	140.62	264.07	167.29	161.61	197.39	255.16	**197.69**
**mmu-miR-361-5p**	220.10	185.37	221.63	242.96	152.76	145.97	**194.80**
**mmu-miR-126a-5p**	180.36	202.97	128.93	186.56	155.34	287.35	**190.25**

Biology Open • Supplementary information

**mmu-miR-185-5p**	187.49	173.98	210.97	189.81	192.24	174.71	**188.20**
**mmu-miR-3065-5p**	118.20	156.37	156.63	312.37	172.50	210.34	**187.74**
**mmu-miR-22-3p**	148.77	205.04	111.88	206.08	148.47	262.06	**180.38**
**mmu-miR-3071-3p**	143.68	191.58	151.30	160.53	171.64	245.97	**177.45**
**mmu-miR-28a-3p**	182.40	143.94	186.47	183.30	175.93	165.51	**172.93**
**mmu-miR-379-5p**	151.83	134.62	194.99	165.95	189.66	165.51	**167.09**
**mmu-miR-365-3p**	128.39	202.97	128.93	175.71	125.30	202.29	**160.60**
**mmu-miR-30a-3p**	147.75	150.16	164.09	171.37	149.33	162.06	**157.46**
**mmu-miR-484**	186.48	160.51	147.04	174.63	132.16	135.63	**156.08**
**mmu-miR-10a-3p**	136.54	160.51	128.93	169.20	148.47	186.20	**154.98**
**mmu-miR-1a-3p**	33.63	19.68	43.69	408.91	115.00	295.39	**152.72**
**mmu-miR-431-5p**	157.94	120.13	170.48	146.42	134.74	164.36	**149.01**
**mmu-miR-27a-5p**	140.62	125.30	212.04	167.03	108.99	102.30	**142.71**
**mmu-miR-381-3p**	139.60	128.41	149.17	97.62	131.31	183.90	**138.34**
**mmu-miR-335-5p**	104.96	128.41	122.53	155.10	134.74	156.32	**133.68**
**mmu-miR-340-5p**	114.13	129.45	135.32	112.80	100.41	164.36	**126.08**
**mmu-miR-18a-5p**	97.82	158.44	80.98	110.63	145.90	158.62	**125.40**
**mmu-miR-190a-5p**	117.18	140.84	91.63	133.41	124.44	143.67	**125.20**
**mmu-miR-17-5p**	109.03	134.62	93.77	125.82	140.75	126.43	**121.74**
**mmu-miR-15b-3p**	95.79	147.05	129.99	70.50	120.15	152.87	**119.39**
**mmu-miR-144-3p**	70.31	130.48	82.04	74.84	133.02	216.08	**117.80**
**mmu-miR-3535**	141.64	98.38	148.11	88.94	117.57	103.44	**116.35**
**mmu-miR-130a-3p**	96.80	106.66	89.50	141.00	113.28	149.42	**116.11**
**mmu-miR-1198-5p**	147.75	93.20	133.19	134.49	106.42	73.56	**114.77**
**mmu-miR-455-5p**	122.28	111.84	114.01	95.45	105.56	118.39	**111.26**
**mmu-miR-126b-5p**	115.15	95.27	98.03	92.19	115.86	142.52	**109.84**
**mmu-miR-3964**	73.37	104.59	140.65	45.55	122.72	171.26	**109.69**
**mmu-miR-669o-3p**	86.61	134.62	120.40	58.57	103.84	148.27	**108.72**
**mmu-miR-674-3p**	118.20	117.02	109.75	94.36	104.70	86.20	**105.04**
**mmu-miR-99b-5p**	138.58	112.88	111.88	113.89	78.10	60.92	**102.71**
**mmu-miR-1839-5p**	110.05	96.31	103.36	112.80	87.54	101.15	**101.87**

Biology Open • Supplementary information

**mmu-miR-98-5p**	111.07	97.34	78.85	106.29	115.86	101.15	**101.76**
**mmu-miR-155-5p**	91.71	117.02	137.45	65.08	102.13	95.40	**101.47**
**mmu-miR-345-3p**	98.84	92.17	87.37	111.72	108.99	106.89	**101.00**
**mmu-miR-872-3p**	87.63	108.74	78.85	97.62	117.57	103.44	**98.98**
**mmu-miR-200a-5p**	84.58	110.81	114.01	81.35	96.98	88.50	**96.04**
**mmu-miR-338-3p**	79.48	76.63	86.31	138.83	78.96	111.49	**95.28**
**mmu-miR-30b-3p**	83.56	91.13	87.37	119.31	73.81	104.59	**93.30**
**mmu-miR-127-5p**	76.42	83.88	88.44	90.02	97.84	114.94	**91.92**
**mmu-miR-411-5p**	85.60	86.99	80.98	83.52	81.53	116.09	**89.12**
**mmu-miR-708-3p**	88.65	72.49	80.98	98.70	95.26	96.55	**88.77**
**mmu-miR-203-5p**	70.31	97.34	114.01	91.11	85.82	72.41	**88.50**
**mmu-miR-324-5p**	82.54	107.70	62.87	78.09	66.94	106.89	**84.17**
**mmu-let-7i-3p**	95.79	80.77	38.36	77.01	76.38	128.73	**82.84**
**mmu-let-7j**	87.63	75.60	73.52	80.26	73.81	103.44	**82.38**
**mmu-miR-503-5p**	74.39	88.02	104.42	69.42	66.94	77.01	**80.03**
**mmu-miR-148b-3p**	85.60	80.77	72.46	62.91	80.67	97.70	**80.02**
**mmu-miR-376b-5p**	62.16	84.92	63.93	78.09	96.12	82.76	**78.00**
**mmu-miR-30d-3p**	98.84	75.60	90.57	66.16	63.51	54.02	**74.78**
**mmu-miR-300-3p**	65.22	85.95	73.52	61.82	65.22	96.55	**74.71**
**mmu-miR-193a-5p**	82.54	62.13	79.91	109.55	56.64	57.47	**74.71**
**mmu-miR-450b-3p**	59.10	83.88	69.26	66.16	78.96	78.16	**72.59**
**mmu-miR-500-3p**	67.25	88.02	53.28	67.25	37.76	114.94	**71.42**
**mmu-miR-669c-5p**	82.54	54.89	82.04	65.08	69.52	71.26	**70.89**
**mmu-miR-140-5p**	63.18	70.42	55.41	49.89	64.37	87.35	**65.10**
**mmu-miR-350-3p**	35.66	89.06	59.67	49.89	58.36	85.05	**62.95**
**mmu-miR-466c-3p@**	57.06	85.95	53.28	28.20	69.52	59.77	**58.96**
**mmu-miR-9-5p**	39.74	62.13	62.87	60.74	60.07	64.37	**58.32**
**mmu-miR-362-3p**	37.70	92.17	47.95	36.88	38.62	96.55	**58.31**
**mmu-miR-652-3p**	66.23	66.28	44.75	61.82	42.91	67.81	**58.30**
**mmu-miR-351-5p**	78.46	36.25	52.21	67.25	64.37	47.12	**57.61**
**mmu-miR-690**	39.74	45.57	49.01	59.65	48.06	93.10	**55.86**

**mmu-miR-214-5p**	60.12	73.53	33.03	55.32	51.49	58.62	**55.35**
**mmu-miR-322-3p**	55.03	52.81	31.97	59.65	65.22	66.66	**55.22**
**mmu-let-7a-1-3p**	60.12	66.28	39.42	46.64	36.04	81.61	**55.02**
**mmu-miR-382-5p**	72.35	34.17	59.67	62.91	63.51	35.63	**54.71**
**mmu-miR-671-5p**	61.14	57.99	77.78	48.81	43.77	34.48	**54.00**
**mmu-miR-22-5p**	47.89	68.35	49.01	46.64	60.93	50.57	**53.90**
**mmu-miR-186-5p**	56.04	56.96	41.56	41.22	60.93	57.47	**52.36**
**mmu-miR-362-5p**	55.03	61.10	50.08	52.06	30.04	64.37	**52.11**
**mmu-miR-425-3p**	42.80	54.89	43.69	50.98	57.50	62.07	**51.99**
**mmu-miR-195a-3p**	45.85	48.67	31.97	54.23	57.50	67.81	**51.01**
**mmu-miR-672-5p**	52.99	46.60	53.28	70.50	42.05	31.03	**49.41**
**mmu-miR-127-3p**	60.12	42.46	58.60	39.05	37.76	50.57	**48.09**
**mmu-miR-574-3p**	42.80	44.53	53.28	68.33	42.91	34.48	**47.72**
**mmu-miR-184-3p**	46.87	59.03	62.87	42.30	42.91	32.18	**47.69**
**mmu-miR-450b-5p**	46.87	57.99	41.56	41.22	44.63	50.57	**47.14**
**mmu-miR-128-3p**	48.91	43.49	52.21	39.05	45.49	39.08	**44.71**
**mmu-miR-378c**	48.91	37.28	43.69	46.64	29.18	54.02	**43.29**
**mmu-miR-1843a-3p**	32.61	49.71	53.28	46.64	39.48	37.93	**43.28**
**mmu-miR-30c-2-3p**	44.84	38.32	54.34	42.30	38.62	33.33	**41.96**
**mmu-miR-542-5p**	47.89	33.14	51.14	46.64	36.90	33.33	**41.51**
**mmu-miR-144-5p**	34.65	37.28	28.77	29.28	46.34	65.51	**40.31**
**mmu-miR-133a-5p**	15.28	4.14	9.59	92.19	20.60	97.70	**39.92**
**mmu-miR-29c-5p**	27.51	54.89	25.57	37.96	38.62	42.53	**37.85**
**mmu-miR-100-5p**	52.99	33.14	45.82	44.47	24.03	20.69	**36.86**
**mmu-miR-5099**	44.84	20.71	33.03	41.22	32.61	40.23	**35.44**
**mmu-miR-218-5p**	31.59	27.96	34.10	41.22	35.19	36.78	**34.47**
**mmu-miR-342-5p**	40.76	30.03	25.57	35.79	30.04	43.68	**34.31**
**mmu-miR-181a-1-3p**	42.80	34.17	36.23	27.12	32.61	32.18	**34.19**
**mmu-miR-375-3p**	35.66	42.46	17.05	29.28	32.61	35.63	**32.12**
**mmu-miR-187-3p**	37.70	22.78	28.77	28.20	33.47	34.48	**30.90**
**mmu-miR-340-3p**	24.46	40.39	21.31	34.71	29.18	31.03	**30.18**

Biology Open • Supplementary information

**mmu-miR-181d-5p**	43.82	31.07	26.64	28.20	19.74	31.03	**30.08**
**mmu-miR-106b-3p**	41.78	25.89	35.16	24.95	30.04	21.84	**29.94**
**mmu-miR-29a-5p**	29.55	37.28	19.18	23.86	24.03	40.23	**29.02**
**mmu-miR-93-3p**	37.70	22.78	29.83	37.96	24.03	20.69	**28.83**
**mmu-miR-139-5p**	15.28	33.14	21.31	36.88	28.32	36.78	**28.62**
**mmu-miR-183-3p**	27.51	26.92	22.38	34.71	30.90	28.73	**28.53**
**mmu-miR-133b-3p**	6.11	4.14	1.07	49.89	10.30	97.70	**28.20**
**mmu-miR-378a-5p**	33.63	35.21	18.11	32.54	20.60	27.59	**27.95**
**mmu-miR-28a-5p@**	23.44	25.89	20.24	37.96	26.60	31.03	**27.53**
**mmu-miR-20b-5p**	21.40	26.92	33.03	14.10	37.76	31.03	**27.37**
**mmu-miR-34b-3p**	36.68	32.10	24.51	23.86	24.89	19.54	**26.93**
**mmu-miR-30f**	21.40	29.00	29.83	26.03	28.32	25.29	**26.65**
**mmu-miR-206-3p**	13.25	2.07	5.33	48.81	23.17	66.66	**26.55**
**mmu-miR-7a-1-3p**	20.38	32.10	17.05	17.35	29.18	37.93	**25.67**
**mmu-miR-434-3p**	27.51	19.68	23.44	34.71	17.16	25.29	**24.63**
**mmu-miR-423-5p**	33.63	21.75	20.24	24.95	22.31	22.99	**24.31**
**mmu-miR-148a-5p**	28.53	31.07	28.77	19.52	15.45	20.69	**24.01**
**mmu-miR-744-5p**	21.40	29.00	14.92	27.12	21.46	27.59	**23.58**
**mmu-miR-676-3p**	39.74	18.64	21.31	28.20	19.74	13.79	**23.57**
**mmu-let-7d-3p**	20.38	16.57	12.79	30.37	16.31	35.63	**22.01**
**mmu-miR-34b-5p**	20.38	25.89	12.79	17.35	18.02	33.33	**21.29**
**mmu-miR-181b-1-3p**	21.40	20.71	21.31	14.10	16.31	27.59	**20.24**
**mmu-miR-223-5p**	17.32	15.53	21.31	27.12	14.59	22.99	**19.81**
**mmu-miR-345-5p**	17.32	25.89	13.85	17.35	19.74	24.14	**19.72**
**mmu-miR-331-3p**	11.21	23.82	7.46	21.69	17.16	36.78	**19.69**
**mmu-miR-676-5p**	18.34	20.71	28.77	21.69	15.45	8.05	**18.84**
**mmu-miR-3105-5p**	15.28	18.64	9.59	17.35	14.59	36.78	**18.71**
**mmu-miR-33-5p**	11.21	20.71	17.05	8.68	23.17	31.03	**18.64**
**mmu-miR-9-3p**	20.38	21.75	17.05	15.18	18.02	18.39	**18.46**
**mmu-miR-139-3p**	27.51	14.50	14.92	22.78	13.73	17.24	**18.45**
**mmu-miR-299a-5p@**	15.28	21.75	18.11	16.27	16.31	16.09	**17.30**

Biology Open • Supplementary information

**mmu-miR-181c-5p**	13.25	27.96	18.11	15.18	8.58	20.69	**17.30**
**mmu-miR-335-3p**	9.17	22.78	19.18	19.52	15.45	14.94	**16.84**
**mmu-miR-466d-3p**	14.27	17.60	20.24	9.76	17.16	21.84	**16.81**
**mmu-miR-27b-5p**	13.25	22.78	12.79	11.93	15.45	24.14	**16.72**
**mmu-miR-154-5p**	11.21	11.39	14.92	23.86	13.73	24.14	**16.54**
**mmu-miR-374c-3p**	21.40	20.71	8.52	15.18	6.01	25.29	**16.19**
**mmu-miR-3473b**	13.25	16.57	14.92	10.85	13.73	27.59	**16.15**
**mmu-miR-298-5p**	11.21	13.46	27.70	14.10	15.45	13.79	**15.95**
**mmu-miR-664-3p**	19.36	19.68	12.79	13.02	11.16	19.54	**15.93**
**mmu-miR-486a-5p**	22.42	14.50	12.79	18.44	7.72	13.79	**14.94**
**mmu-miR-34c-3p**	16.30	12.43	11.72	11.93	18.02	16.09	**14.42**
**mmu-miR-186-3p**	12.23	20.71	12.79	13.02	15.45	11.49	**14.28**
**mmu-miR-181c-3p**	16.30	17.60	10.66	11.93	16.31	12.64	**14.24**
**mmu-miR-150-3p**	20.38	15.53	14.92	5.42	15.45	11.49	**13.87**
**mmu-miR-423-3p**	18.34	12.43	17.05	15.18	5.15	14.94	**13.85**
**mmu-miR-582-3p**	4.08	10.36	15.98	21.69	15.45	12.64	**13.37**
**mmu-miR-3473a**	12.23	13.46	14.92	11.93	13.73	12.64	**13.15**
**mmu-miR-219a-5p**	13.25	12.43	10.66	7.59	7.72	26.44	**13.02**
**mmu-miR-301b-3p**	19.36	10.36	6.39	10.85	17.16	13.79	**12.99**
**mmu-miR-30e-3p**	15.28	8.28	18.11	14.10	6.87	14.94	**12.93**
**mmu-miR-486a-3p@**	16.30	8.28	11.72	16.27	11.16	13.79	**12.92**
**mmu-miR-136-5p**	8.15	13.46	14.92	8.68	12.01	19.54	**12.79**
**mmu-miR-532-3p**	18.34	11.39	11.72	8.68	11.16	14.94	**12.71**
**mmu-miR-15a-3p**	10.19	12.43	9.59	15.18	12.87	14.94	**12.53**
**mmu-miR-378b**	12.23	16.57	14.92	8.68	9.44	12.64	**12.41**
**mmu-miR-222-3p**	15.28	12.43	11.72	7.59	15.45	10.34	**12.14**
**mmu-miR-574-5p**	8.15	14.50	13.85	13.02	12.87	10.34	**12.12**
**mmu-miR-1191a**	16.30	9.32	13.85	6.51	15.45	10.34	**11.96**
**mmu-miR-1193-3p**	14.27	8.28	7.46	11.93	10.30	18.39	**11.77**
**mmu-miR-134-5p**	10.19	5.18	21.31	14.10	6.87	12.64	**11.72**
**mmu-miR-326-3p**	9.17	14.50	2.13	6.51	6.87	28.73	**11.32**

Biology Open • Supplementary information

**mmu-miR-491-5p**	12.23	9.32	13.85	13.02	5.15	13.79	**11.23**
**mmu-miR-503-3p**	11.21	11.39	18.11	8.68	7.72	9.20	**11.05**
**mmu-miR-467e-5p**	10.19	9.32	10.66	6.51	11.16	17.24	**10.85**
**mmu-miR-409-3p**	11.21	9.32	14.92	7.59	10.30	11.49	**10.81**
**mmu-miR-190b-5p**	11.21	10.36	6.39	15.18	12.87	6.90	**10.49**
**mmu-miR-363-3p**	10.19	8.28	9.59	8.68	6.01	19.54	**10.38**
**mmu-miR-1291**	10.19	8.28	12.79	7.59	6.01	16.09	**10.16**
**mmu-miR-664-5p**	15.28	10.36	10.66	5.42	7.72	11.49	**10.16**
**mmu-miR-20a-3p**	6.11	10.36	9.59	13.02	5.15	16.09	**10.05**
**mmu-miR-296-3p**	16.30	5.18	11.72	8.68	7.72	9.20	**9.80**
**mmu-miR-511-3p**	7.13	8.28	11.72	9.76	11.16	10.34	**9.73**
**mmu-miR-672-3p**	3.06	10.36	12.79	9.76	16.31	4.60	**9.48**
**mmu-miR-665-3p**	10.19	11.39	8.52	8.68	7.72	10.34	**9.47**
**mmu-miR-337-5p**	4.08	12.43	8.52	9.76	10.30	10.34	**9.24**
**mmu-miR-376c-3p**	5.09	11.39	6.39	13.02	5.15	13.79	**9.14**
**mmu-miR-92a-1-5p**	11.21	12.43	5.33	7.59	6.01	11.49	**9.01**
**mmu-miR-16-1-3p**	9.17	17.60	5.33	5.42	9.44	6.90	**8.98**
**mmu-miR-497a-3p**	6.11	8.28	10.66	2.17	10.30	16.09	**8.94**
**mmu-miR-450a-1-3p**	7.13	14.50	10.66	8.68	7.72	4.60	**8.88**
**mmu-miR-361-3p**	11.21	12.43	3.20	11.93	9.44	4.60	**8.80**
**mmu-miR-6240**	2.04	3.11	3.20	3.25	0.86	40.23	**8.78**
**mmu-miR-466i-5p**	4.08	5.18	9.59	13.02	6.01	13.79	**8.61**
**mmu-miR-132-3p**	13.25	8.28	8.52	9.76	4.29	6.90	**8.50**
**mmu-miR-1843b-3p**	14.27	6.21	6.39	7.59	8.58	6.90	**8.32**
**mmu-miR-5123**	8.15	12.43	4.26	6.51	6.01	11.49	**8.14**
**mmu-miR-409-5p**	10.19	10.36	8.52	9.76	6.01	2.30	**7.86**
**mmu-miR-350-5p**	7.13	10.36	7.46	9.76	6.01	5.75	**7.75**
**mmu-miR-450a-5p**	6.11	8.28	5.33	9.76	7.72	9.20	**7.73**
**mmu-miR-1981-5p**	6.11	6.21	6.39	8.68	6.01	12.64	**7.67**
**mmu-miR-205-3p**	9.17	10.36	8.52	4.34	7.72	4.60	**7.45**
**mmu-miR-130b-3p**	8.15	7.25	8.52	4.34	6.87	9.20	**7.39**

**mmu-miR-701-5p**	10.19	9.32	9.59	2.17	6.87	5.75	**7.32**
**mmu-miR-200c-5p**	6.11	7.25	5.33	5.42	6.87	10.34	**6.89**
**mmu-miR-339-3p**	7.13	9.32	3.20	10.85	6.01	4.60	**6.85**
**mmu-miR-421-3p**	11.21	6.21	7.46	4.34	6.01	5.75	**6.83**
**mmu-miR-485-5p**	8.15	6.21	6.39	7.59	4.29	8.05	**6.78**
**mmu-miR-669a-5p**	9.17	13.46	3.20	1.08	7.72	5.75	**6.73**
**mmu-miR-3105-3p**	5.09	10.36	5.33	5.42	6.87	6.90	**6.66**
**mmu-miR-125b-1-3p**	9.17	6.21	5.33	4.34	7.72	6.90	**6.61**
**mmu-miR-29b-1-5p**	3.06	12.43	5.33	5.42	6.01	6.90	**6.53**
**mmu-miR-467a-3p@**	7.13	6.21	9.59	1.08	4.29	10.34	**6.44**
**mmu-miR-3064-5p**	7.13	7.25	1.07	7.59	5.15	9.20	**6.23**
**mmu-miR-124-3p**	8.15	8.28	3.20	2.17	10.30	4.60	**6.12**
**mmu-miR-152-5p**	6.11	7.25	4.26	3.25	5.15	10.34	**6.06**
**mmu-miR-455-3p**	10.19	6.21	3.20	6.51	7.72	2.30	**6.02**
**mmu-miR-330-5p**	4.08	12.43	6.39	3.25	2.57	5.75	**5.75**
**mmu-miR-3071-5p**	7.13	4.14	8.52	1.08	4.29	8.05	**5.54**
**mmu-miR-106a-5p**	7.13	10.36	2.13	3.25	3.43	4.60	**5.15**
**mmu-miR-125a-3p**	7.13	6.21	0.00	5.42	5.15	6.90	**5.14**
**mmu-miR-376b-3p**	4.08	4.14	6.39	5.42	6.87	3.45	**5.06**
**mmu-miR-6238**	4.08	2.07	3.20	2.17	2.57	16.09	**5.03**
**mmu-miR-211-5p**	7.13	6.21	4.26	0.00	4.29	8.05	**4.99**
**mmu-miR-582-5p**	4.08	4.14	0.00	4.34	4.29	12.64	**4.92**
**mmu-miR-18a-3p**	6.11	2.07	9.59	4.34	4.29	2.30	**4.78**
**mmu-miR-190a-3p**	5.09	5.18	5.33	3.25	5.15	4.60	**4.77**
**mmu-miR-5121**	4.08	5.18	4.26	3.25	4.29	6.90	**4.66**
**mmu-miR-700-3p**	8.15	3.11	4.26	4.34	4.29	3.45	**4.60**
**mmu-let-7g-3p**	7.13	4.14	6.39	4.34	1.72	3.45	**4.53**
**mmu-miR-1968-5p**	5.09	5.18	4.26	3.25	3.43	5.75	**4.49**
**mmu-miR-221-5p**	8.15	7.25	4.26	4.34	1.72	1.15	**4.48**
**mmu-miR-188-5p**	3.06	7.25	3.20	6.51	4.29	2.30	**4.44**
**mmu-miR-299a-3p@**	3.06	3.11	3.20	5.42	3.43	8.05	**4.38**

Biology Open • Supplementary information

**mmu-miR-99b-3p**	2.04	6.21	7.46	3.25	2.57	4.60	**4.36**
**mmu-miR-3066-5p**	0.00	6.21	4.26	3.25	7.72	4.60	**4.34**
**mmu-miR-149-5p**	4.08	6.21	3.20	6.51	2.57	3.45	**4.34**
**mmu-miR-493-5p**	3.06	3.11	3.20	5.42	4.29	6.90	**4.33**
**mmu-miR-329-5p**	4.08	7.25	4.26	4.34	3.43	2.30	**4.28**
**mmu-miR-376a-5p**	1.02	6.21	2.13	2.17	2.57	11.49	**4.27**
**mmu-miR-668-3p**	7.13	1.04	4.26	3.25	5.15	4.60	**4.24**
**mmu-let-7k**	2.04	4.14	4.26	6.51	3.43	4.60	**4.16**
**mmu-miR-341-3p**	3.06	4.14	7.46	2.17	1.72	5.75	**4.05**
**mmu-miR-21a-3p**	1.02	2.07	7.46	6.51	2.57	4.60	**4.04**
**mmu-miR-1981-3p**	2.04	6.21	6.39	0.00	4.29	4.60	**3.92**
**mmu-miR-146b-3p**	2.04	3.11	2.13	7.59	5.15	3.45	**3.91**
**mmu-miR-700-5p**	8.15	1.04	4.26	4.34	4.29	1.15	**3.87**
**mmu-miR-466f-3p**	5.09	9.32	2.13	4.34	1.72	0.00	**3.77**
**mmu-miR-16-2-3p**	5.09	3.11	4.26	3.25	3.43	3.45	**3.77**
**mmu-miR-3058-5p**	4.08	3.11	4.26	3.25	5.15	2.30	**3.69**
**mmu-miR-467c-5p**	4.08	1.04	6.39	3.25	2.57	4.60	**3.66**
**mmu-miR-31-3p**	2.04	4.14	2.13	1.08	5.15	6.90	**3.57**
**mmu-let-7b-3p**	4.08	5.18	4.26	0.00	4.29	3.45	**3.54**
**mmu-miR-669f-3p**	1.02	3.11	6.39	3.25	5.15	2.30	**3.54**
**mmu-miR-148b-5p**	1.02	3.11	3.20	3.25	2.57	8.05	**3.53**
**mmu-miR-132-5p**	4.08	4.14	1.07	6.51	1.72	3.45	**3.50**
**mmu-miR-147-3p**	4.08	3.11	0.00	3.25	3.43	6.90	**3.46**
**mmu-miR-3069-5p**	9.17	2.07	0.00	2.17	2.57	4.60	**3.43**
**mmu-miR-135b-5p**	2.04	2.07	3.20	4.34	4.29	4.60	**3.42**
**mmu-miR-494-3p**	2.04	5.18	2.13	5.42	4.29	1.15	**3.37**
**mmu-miR-450a-2-3p**	2.04	1.04	4.26	2.17	6.01	4.60	**3.35**
**mmu-miR-181b-2-3p**	2.04	1.04	7.46	1.08	3.43	4.60	**3.28**
**mmu-miR-30c-1-3p**	4.08	2.07	5.33	5.42	2.57	0.00	**3.25**
**mmu-miR-297c-3p**	1.02	4.14	2.13	3.25	0.86	8.05	**3.24**
**mmu-miR-34a-3p**	3.06	5.18	2.13	4.34	3.43	1.15	**3.22**

**mmu-miR-3544-3p**	3.06	1.04	4.26	2.17	5.15	3.45	**3.19**
**mmu-miR-379-3p**	1.02	4.14	2.13	8.68	0.86	2.30	**3.19**
**mmu-miR-3068-3p**	0.00	5.18	1.07	5.42	2.57	4.60	**3.14**
**mmu-miR-598-3p**	4.08	7.25	2.13	1.08	1.72	2.30	**3.09**
**mmu-miR-1839-3p**	1.02	3.11	5.33	2.17	1.72	4.60	**2.99**
**mmu-miR-501-5p**	6.11	2.07	3.20	5.42	0.00	1.15	**2.99**
**mmu-miR-5126**	1.02	6.21	1.07	3.25	1.72	4.60	**2.98**
**mmu-miR-6236**	2.04	3.11	3.20	4.34	1.72	3.45	**2.98**
**mmu-miR-1306-5p**	3.06	2.07	4.26	2.17	2.57	3.45	**2.93**
**mmu-miR-369-5p**	2.04	5.18	2.13	0.00	3.43	4.60	**2.90**
**mmu-miR-467d-5p**	3.06	2.07	1.07	1.08	4.29	5.75	**2.89**
**mmu-miR-3084-3p**	1.02	5.18	0.00	1.08	8.58	1.15	**2.84**
**mmu-miR-877-5p**	3.06	2.07	2.13	1.08	2.57	5.75	**2.78**
**mmu-miR-328-3p**	8.15	1.04	3.20	1.08	0.86	2.30	**2.77**
**mmu-miR-122-3p**	0.00	0.00	0.00	0.00	0.00	16.09	**2.68**
**mmu-miR-3544-5p**	1.02	4.14	2.13	3.25	0.86	4.60	**2.67**
**mmu-miR-369-3p**	2.04	3.11	3.20	2.17	0.86	4.60	**2.66**
**mmu-miR-135a-5p**	2.04	1.04	3.20	2.17	1.72	5.75	**2.65**
**mmu-miR-26a-2-3p**	2.04	5.18	0.00	1.08	1.72	5.75	**2.63**
**mmu-miR-511-5p**	3.06	1.04	6.39	3.25	0.86	1.15	**2.63**
**mmu-miR-25-5p**	3.06	3.11	2.13	2.17	2.57	2.30	**2.56**
**mmu-miR-3057-5p**	1.02	3.11	2.13	2.17	1.72	4.60	**2.46**
**mmu-miR-216a-5p**	3.06	3.11	0.00	3.25	1.72	3.45	**2.43**
**mmu-miR-193b-5p**	3.06	2.07	1.07	4.34	1.72	2.30	**2.43**
**mmu-miR-324-3p**	0.00	1.04	3.20	3.25	3.43	3.45	**2.40**
**mmu-miR-652-5p**	1.02	4.14	1.07	0.00	3.43	4.60	**2.38**
**mmu-miR-501-3p**	1.02	1.04	6.39	1.08	0.86	3.45	**2.31**
**mmu-miR-29b-2-5p**	0.00	4.14	2.13	2.17	2.57	2.30	**2.22**
**mmu-miR-26b-3p**	4.08	3.11	0.00	1.08	2.57	2.30	**2.19**
**mmu-miR-18b-5p**	2.04	2.07	3.20	2.17	3.43	0.00	**2.15**
**mmu-miR-3968**	3.06	2.07	2.13	2.17	0.86	2.30	**2.10**

Biology Open • Supplementary information

**mmu-miR-2137**	2.04	2.07	1.07	2.17	1.72	3.45	**2.09**
**mmu-miR-138-1-3p**	2.04	1.04	2.13	3.25	1.72	2.30	**2.08**
**mmu-miR-374b-5p@**	2.04	4.14	2.13	0.00	1.72	2.30	**2.06**
**mmu-miR-540-3p**	1.02	1.04	0.00	4.34	3.43	2.30	**2.02**
**mmu-miR-1306-3p**	3.06	2.07	1.07	2.17	0.00	3.45	**1.97**
**mmu-miR-3065-3p**	0.00	4.14	3.20	3.25	0.00	1.15	**1.96**
**mmu-miR-195b**	1.02	3.11	2.13	1.08	0.86	3.45	**1.94**
**mmu-miR-191-3p**	3.06	1.04	1.07	4.34	1.72	0.00	**1.87**
**mmu-miR-380-5p**	5.09	2.07	3.20	0.00	0.86	0.00	**1.87**
**mmu-miR-466k**	1.02	5.18	0.00	1.08	2.57	1.15	**1.83**
**mmu-let-7f-1-3p**	4.08	0.00	0.00	1.08	3.43	2.30	**1.82**
**mmu-miR-23b-5p**	4.08	0.00	0.00	2.17	3.43	1.15	**1.81**
**mmu-miR-1955-3p**	2.04	3.11	1.07	1.08	0.86	2.30	**1.74**
**mmu-miR-3473e**	2.04	1.04	0.00	3.25	1.72	2.30	**1.73**
**mmu-miR-101b-5p**	2.04	4.14	1.07	1.08	0.86	1.15	**1.72**
**mmu-miR-182-3p**	0.00	4.14	1.07	1.08	1.72	2.30	**1.72**
**mmu-miR-6538**	0.00	3.11	1.07	1.08	2.57	2.30	**1.69**
**mmu-miR-3108-5p**	3.06	2.07	0.00	3.25	1.72	0.00	**1.68**
**mmu-miR-376a-3p**	0.00	5.18	1.07	0.00	2.57	1.15	**1.66**
**mmu-miR-7083-5p**	0.00	1.04	2.13	1.08	1.72	3.45	**1.57**
**mmu-miR-33-3p**	0.00	4.14	2.13	1.08	0.86	1.15	**1.56**
**mmu-miR-20b-3p**	1.02	4.14	0.00	1.08	1.72	1.15	**1.52**
**mmu-miR-323-3p**	2.04	1.04	2.13	2.17	1.72	0.00	**1.52**
**mmu-miR-5113**	2.04	2.07	3.20	0.00	1.72	0.00	**1.51**
**mmu-miR-3470a**	0.00	2.07	0.00	3.25	0.86	2.30	**1.41**
**mmu-miR-26a-1-3p**	3.06	1.04	2.13	1.08	0.00	1.15	**1.41**
**mmu-miR-673-5p**	2.04	1.04	1.07	1.08	0.86	2.30	**1.40**
**mmu-miR-499-5p**	1.02	1.04	0.00	1.08	1.72	3.45	**1.39**
**mmu-miR-7010-5p**	3.06	0.00	2.13	1.08	0.86	1.15	**1.38**
**mmu-miR-874-3p**	2.04	3.11	1.07	0.00	0.86	1.15	**1.37**
**mmu-miR-380-3p**	2.04	1.04	3.20	1.08	0.86	0.00	**1.37**

**mmu-miR-485-3p**	0.00	1.04	0.00	5.42	1.72	0.00	**1.36**
**mmu-miR-466h-3p**	1.02	1.04	1.07	2.17	1.72	1.15	**1.36**
**mmu-miR-5615-5p**	1.02	1.04	1.07	2.17	1.72	1.15	**1.36**
**mmu-miR-452-3p**	4.08	2.07	0.00	0.00	0.86	1.15	**1.36**
**mmu-miR-497b**	1.02	3.11	0.00	0.00	1.72	2.30	**1.36**
**mmu-miR-5709-5p**	1.02	5.18	0.00	1.08	0.86	0.00	**1.36**
**mmu-miR-217-5p**	0.00	1.04	4.26	1.08	1.72	0.00	**1.35**
**mmu-miR-744-3p**	0.00	3.11	1.07	2.17	1.72	0.00	**1.35**
**mmu-miR-219a-1-3p**	1.02	0.00	1.07	2.17	2.57	1.15	**1.33**
**mmu-miR-6946-5p**	2.04	0.00	2.13	0.00	2.57	1.15	**1.32**
**mmu-let-7c-1-3p**	1.02	1.04	0.00	3.25	2.57	0.00	**1.31**
**mmu-miR-467a-5p@**	0.00	0.00	1.07	2.17	0.00	4.60	**1.31**
**mmu-miR-466h-5p**	1.02	0.00	0.00	2.17	3.43	1.15	**1.30**
**mmu-miR-466i-3p**	0.00	0.00	2.13	0.00	0.86	4.60	**1.27**
**mmu-miR-101a-5p**	3.06	1.04	0.00	2.17	0.00	1.15	**1.24**
**mmu-miR-1983**	0.00	2.07	1.07	1.08	0.86	2.30	**1.23**
**mmu-miR-505-3p**	3.06	2.07	0.00	1.08	0.00	1.15	**1.23**
**mmu-miR-130b-5p**	2.04	0.00	1.07	1.08	0.86	2.30	**1.23**
**mmu-miR-187-5p**	1.02	0.00	1.07	3.25	0.86	1.15	**1.23**
**mmu-miR-212-5p**	1.02	0.00	3.20	1.08	0.86	1.15	**1.22**
**mmu-miR-1941-3p**	1.02	2.07	0.00	2.17	0.86	1.15	**1.21**
**mmu-miR-24-1-5p**	0.00	2.07	1.07	3.25	0.86	0.00	**1.21**
**mmu-miR-671-3p**	0.00	0.00	2.13	1.08	1.72	2.30	**1.21**
**mmu-miR-137-3p**	1.02	0.00	0.00	2.17	1.72	2.30	**1.20**
**mmu-miR-7059-5p**	2.04	2.07	0.00	2.17	0.86	0.00	**1.19**
**mmu-miR-181a-2-3p**	2.04	2.07	2.13	0.00	0.86	0.00	**1.18**
**mmu-miR-488-3p**	1.02	2.07	1.07	0.00	1.72	1.15	**1.17**
**mmu-miR-466q**	0.00	2.07	2.13	1.08	1.72	0.00	**1.17**
**mmu-miR-1198-3p**	1.02	1.04	3.20	0.00	1.72	0.00	**1.16**
**mmu-miR-412-5p**	3.06	1.04	0.00	0.00	1.72	1.15	**1.16**
**mmu-miR-19b-1-5p**	2.04	1.04	1.07	1.08	1.72	0.00	**1.16**

**mmu-miR-19a-5p**	1.02	1.04	1.07	0.00	2.57	1.15	**1.14**
**mmu-miR-802-5p**	2.04	0.00	0.00	0.00	0.00	4.60	**1.11**
**mmu-miR-6951-5p**	1.02	0.00	2.13	1.08	0.00	2.30	**1.09**
**mmu-miR-666-3p**	0.00	0.00	0.00	0.00	1.72	4.60	**1.05**
**mmu-miR-3076-5p**	3.06	1.04	1.07	1.08	0.00	0.00	**1.04**
**mmu-miR-706**	1.02	0.00	0.00	0.00	1.72	3.45	**1.03**
**mmu-miR-32-3p**	0.00	2.07	0.00	0.00	1.72	2.30	**1.02**
**mmu-miR-3079-5p**	0.00	1.04	2.13	0.00	1.72	1.15	**1.01**
**mmu-miR-3081-3p**	2.04	3.11	0.00	0.00	0.86	0.00	**1.00**
**mmu-miR-330-3p**	0.00	1.04	1.07	2.17	1.72	0.00	**1.00**

Table S3. miRNA expressed in the mammary gland at day 7 of lactation. The number of reads determined by the sequencing is indicated for each of the animal, as well as the average of the group (N=6). Only miRNA with an average read for the group above 1 are listed.

miRNA	Lac7_1	Lac7_2	Lac7_3	Lac7_4	Lac7_5	Lac7_6	Average expression
**mmu-miR-143-3p**	132142.61	148114.39	138759.09	139249.09	144154.73	128703.97	**138520.65**
**mmu-let-7c-5p**	122462.25	109964.67	99247.10	113318.44	106383.62	139372.42	**115124.75**
**mmu-let-7a-5p**	84710.65	68566.99	79126.30	83642.55	79591.61	98346.08	**82330.70**
**mmu-miR-200a-3p**	70176.60	80726.41	82433.30	71660.75	76179.76	62672.22	**73974.84**
**mmu-miR-16-5p**	62261.76	58955.62	64661.51	65691.79	68485.31	60911.00	**63494.50**
**mmu-miR-103-3p**	51177.69	61037.95	56911.56	46392.42	52520.95	52985.51	**53504.35**
**mmu-miR-146b-5p**	54079.70	50499.17	47979.69	51592.65	49657.30	47442.02	**50208.42**
**mmu-miR-148a-3p**	46406.57	47533.59	49877.88	47936.53	43452.98	42158.35	**46227.65**
**mmu-miR-27b-3p**	38107.41	40506.40	36408.15	34256.34	36636.85	37613.45	**37254.77**
**mmu-miR-200b-3p**	31979.11	34464.16	31502.52	29189.11	26941.94	33433.71	**31251.76**
**mmu-miR-29a-3p**	18473.45	22168.25	23614.66	21862.24	24362.99	15600.99	**21013.76**
**mmu-miR-199a-3p**	22734.13	20670.73	21347.21	16898.75	21067.74	15992.84	**19785.23**
**mmu-let-7f-5p**	19141.53	16662.65	18766.86	18414.93	17993.60	18806.02	**18297.60**
**mmu-miR-126a-3p**	16937.63	17686.42	17590.87	18986.82	18738.66	19311.64	**18208.67**
**mmu-let-7b-5p**	14301.35	12080.46	11096.99	12657.44	11484.89	15821.50	**12907.11**
**mmu-miR-101a-3p@**	12944.18	14628.50	12563.64	10964.37	13126.45	10987.27	**12535.74**
**mmu-miR-142b**	6090.76	7028.53	8123.66	22539.20	19927.43	6667.08	**11729.44**
**mmu-miR-21a-5p**	10067.69	9101.49	11266.05	13037.81	10371.84	10044.86	**10648.29**
**mmu-miR-200c-3p**	10154.77	9057.33	9354.51	7658.04	6835.81	9886.15	**8824.44**
**mmu-miR-30a-5p**	7284.29	9580.59	9729.70	8942.80	8842.33	8535.04	**8819.13**
**mmu-let-7i-5p**	7119.15	6849.21	6791.96	7302.93	6866.10	7602.46	**7088.64**
**mmu-miR-3074-5p**	6066.74	6309.89	6419.73	7310.91	7190.17	6894.60	**6698.67**
**mmu-miR-182-5p**	7101.13	6912.10	6597.69	5808.03	6254.30	6713.43	**6564.45**
**mmu-miR-141-3p**	6593.69	6843.85	6679.25	6087.33	6425.42	5838.43	**6411.33**
**mmu-miR-125a-5p**	4838.68	4562.12	6378.21	5588.58	5233.62	6297.70	**5483.15**
**mmu-miR-26a-5p**	5195.99	5256.68	5707.91	5689.66	5003.44	5424.11	**5379.63**
**mmu-miR-142a-5p**	2804.42	3455.38	3351.49	9582.52	9787.29	3143.23	**5354.06**

**mmu-miR-203-3p**	4095.54	5531.02	5076.17	5048.61	4683.91	5221.87	**4942.85**
**mmu-miR-25-3p**	4843.18	4418.93	4862.63	4972.80	4726.31	4803.33	**4771.20**
**mmu-let-7e-5p**	5176.47	4548.74	4315.41	4314.46	4565.79	5525.23	**4741.02**
**mmu-miR-1b-5p**	12349.66	5027.84	4475.57	155.61	221.10	6106.69	**4722.75**
**mmu-miR-10a-5p**	4901.73	3744.45	4714.33	4064.42	4275.03	3727.50	**4237.91**
**mmu-miR-30e-5p**	3714.21	4471.12	4152.29	4108.31	4403.75	3425.53	**4045.87**
**mmu-miR-23a-3p**	4425.82	3808.68	4407.36	3818.38	3628.40	4061.76	**4025.07**
**mmu-miR-101b-3p**	2944.04	4515.28	3562.07	3830.35	4378.01	3612.33	**3807.01**
**mmu-miR-23b-3p**	3891.36	3345.65	3467.16	2948.57	2871.22	3747.16	**3378.52**
**mmu-miR-146a-5p**	1828.58	2755.47	2455.78	4383.62	3401.25	4778.05	**3267.13**
**mmu-miR-125b-5p**	2793.91	2734.06	3562.07	3328.94	2930.28	3158.68	**3084.66**
**mmu-miR-30d-5p**	2645.28	3253.31	3234.34	2878.08	2636.50	3352.50	**3000.00**
**mmu-miR-195a-5p**	3331.38	2809.00	3256.58	2927.29	3048.40	2563.18	**2989.31**
**mmu-let-7d-5p**	2663.30	2304.48	2897.70	3062.95	2695.56	3047.73	**2778.62**
**mmu-miR-26b-5p**	2466.63	2459.72	2826.52	2943.25	2766.73	2646.05	**2684.82**
**mmu-miR-221-3p**	2259.45	2585.51	2529.93	2526.97	2474.46	2738.74	**2519.18**
**mmu-miR-93-5p**	2047.77	2343.29	2687.12	2346.09	2348.77	2285.09	**2343.02**
**mmu-miR-15b-5p**	2454.62	1987.31	2079.11	2362.05	2533.52	2362.34	**2296.49**
**mmu-miR-141-5p**	2790.91	2044.86	2390.53	1937.78	2239.73	1817.40	**2203.54**
**mmu-let-7g-5p**	2287.98	1801.30	2311.93	2340.77	2139.79	2199.42	**2180.20**
**mmu-miR-429-3p**	2175.38	1976.61	2295.62	2133.29	2065.58	1839.87	**2081.06**
**mmu-miR-107-3p**	1489.29	1923.08	1984.20	1723.66	2011.07	1639.03	**1795.06**
**mmu-miR-126b-3p**	1522.31	1729.03	1944.16	1860.64	1864.17	1575.83	**1749.36**
**mmu-miR-29c-3p**	1295.62	1840.10	1782.52	1641.20	1847.52	1634.82	**1673.63**
**mmu-miR-30c-5p**	1465.27	1664.79	1944.16	1677.11	1688.51	1460.66	**1650.08**
**mmu-miR-196a-5p**	1496.79	1563.09	1291.66	1389.83	1347.78	1691.00	**1463.36**
**mmu-miR-133a-3p**	3416.95	1662.12	1654.98	87.78	87.83	1759.82	**1444.91**
**mmu-miR-181b-5p**	1216.05	1461.38	1702.44	1419.09	1214.51	1476.11	**1414.93**
**mmu-miR-192-5p**	1460.76	1620.63	1505.20	1232.89	1341.72	1158.70	**1386.65**
**mmu-miR-199a-5p**	1345.16	1354.32	1453.30	1412.44	1582.50	1162.91	**1385.11**
**mmu-miR-181a-5p**	1178.52	1310.15	1772.14	1266.14	1262.97	1452.23	**1373.69**

Biology Open • Supplementary information

**mmu-miR-140-3p**	918.79	1227.18	1327.25	1381.85	1529.50	1032.29	**1236.14**
**mmu-miR-183-5p**	1148.49	1112.09	1032.14	960.25	1093.37	1133.42	**1079.96**
**mmu-miR-151-5p**	1056.91	1164.28	1052.90	1020.10	1041.88	1047.74	**1063.97**
**mmu-miR-19b-3p**	924.80	810.98	1164.12	1178.36	1096.39	964.88	**1023.26**
**mmu-miR-375-3p**	956.33	1027.78	1008.41	919.02	843.50	1028.08	**963.85**
**mmu-miR-145a-5p**	780.67	841.76	1069.21	1107.87	929.82	988.76	**953.02**
**mmu-miR-322-5p**	743.14	788.23	794.87	788.68	1031.28	814.60	**826.80**
**mmu-miR-378a-3p**	761.16	931.43	995.07	766.07	745.06	668.53	**811.22**
**mmu-miR-223-3p**	705.61	896.63	831.94	782.03	884.38	706.45	**801.17**
**mmu-miR-142a-3p**	429.37	444.30	541.28	1595.98	1320.52	449.43	**796.81**
**mmu-miR-3068-5p**	699.60	788.23	851.22	740.80	837.44	662.92	**763.37**
**mmu-miR-152-3p**	759.66	782.88	846.77	752.77	788.98	560.39	**748.58**
**mmu-miR-210-3p**	659.07	835.07	765.21	688.93	695.09	780.89	**737.38**
**mmu-miR-320-3p**	974.34	661.10	559.08	610.46	470.97	1068.81	**724.13**
**mmu-miR-191-5p**	593.01	701.25	652.50	691.59	649.66	717.69	**667.62**
**mmu-miR-24-3p**	587.01	599.54	656.95	715.53	645.12	655.89	**643.34**
**mmu-miR-451a**	659.07	543.33	656.95	630.41	651.17	551.96	**615.48**
**mmu-miR-15a-5p**	517.95	568.76	616.91	574.55	616.34	466.29	**560.13**
**mmu-miR-1843a-5p@**	531.46	608.91	636.19	504.06	549.71	529.49	**559.97**
**mmu-miR-425-5p**	498.43	519.24	701.44	549.28	536.08	514.04	**553.09**
**mmu-miR-32-5p**	565.99	566.08	594.67	516.03	642.09	382.02	**544.48**
**mmu-miR-1a-3p**	1357.17	584.82	523.48	15.96	24.23	654.49	**526.69**
**mmu-miR-194-5p**	466.90	543.33	542.76	539.97	586.06	445.22	**520.71**
**mmu-miR-10b-5p**	456.39	436.27	600.60	506.72	490.65	505.61	**499.37**
**mmu-miR-196b-5p**	523.95	536.64	474.55	445.54	430.08	582.86	**498.94**
**mmu-miR-29b-3p**	382.83	501.85	470.10	520.02	605.74	456.46	**489.50**
**mmu-miR-872-5p**	516.45	511.21	538.31	429.58	463.39	462.07	**486.84**
**mmu-miR-342-3p**	486.42	386.76	434.51	637.06	525.48	417.13	**481.23**
**mmu-miR-96-5p**	507.44	463.04	570.94	469.48	493.68	363.76	**478.06**
**mmu-miR-151-3p**	400.85	357.31	511.62	508.05	360.42	466.29	**434.09**
**mmu-miR-27a-3p**	448.89	409.51	424.13	454.85	449.76	391.85	**429.83**

**mmu-miR-497a-5p**	400.85	366.68	496.79	393.67	463.39	261.23	**397.10**
**mmu-miR-7a-5p**	409.85	460.36	339.60	445.54	331.64	376.40	**393.90**
**mmu-miR-542-3p**	396.34	380.07	326.25	347.13	442.19	398.87	**381.81**
**mmu-miR-30b-5p**	295.76	318.51	430.06	345.80	334.67	307.58	**338.73**
**mmu-miR-144-3p**	264.23	333.23	284.73	356.44	371.02	328.65	**323.05**
**mmu-miR-20a-5p**	273.24	302.45	404.85	352.45	336.19	252.81	**320.33**
**mmu-miR-10b-3p**	312.27	319.84	372.22	256.69	305.90	303.37	**311.72**
**mmu-miR-301a-3p**	271.73	232.86	344.05	344.47	334.67	273.87	**300.28**
**mmu-miR-185-5p**	262.73	307.80	259.52	307.23	274.10	313.20	**287.43**
**mmu-miR-200b-5p**	354.31	247.58	357.39	268.66	301.36	179.77	**284.85**
**mmu-miR-674-5p**	243.21	267.65	265.45	313.88	254.41	261.23	**267.64**
**mmu-miR-126a-5p**	219.19	248.92	295.11	279.30	266.53	237.36	**257.74**
**mmu-miR-1839-5p**	202.68	230.18	296.59	301.91	236.24	272.47	**256.68**
**mmu-miR-190a-5p**	259.72	291.74	266.93	255.36	239.27	209.27	**253.72**
**mmu-miR-340-5p**	286.75	202.08	212.06	232.75	248.35	183.99	**227.66**
**mmu-miR-34a-5p**	165.14	192.71	268.42	268.66	245.33	193.82	**222.35**
**mmu-miR-22-3p**	238.71	211.44	238.76	190.19	169.61	202.25	**208.49**
**mmu-miR-30a-3p**	154.63	218.14	206.13	196.84	201.41	226.12	**200.55**
**mmu-miR-34c-5p**	190.66	191.37	268.42	216.79	190.81	137.64	**199.28**
**mmu-miR-145a-3p**	180.16	177.99	246.17	203.49	190.81	174.16	**195.46**
**mmu-miR-532-5p**	168.15	196.72	180.92	194.18	202.92	213.48	**192.73**
**mmu-miR-3065-5p**	268.73	222.15	275.83	119.70	139.32	108.15	**188.98**
**mmu-miR-200a-5p**	177.15	219.47	197.23	151.62	165.06	207.86	**186.40**
**mmu-miR-484**	204.18	230.18	231.34	148.96	133.26	154.49	**183.74**
**mmu-miR-143-5p**	148.63	156.58	149.78	224.77	193.84	183.99	**176.27**
**mmu-miR-339-5p**	142.62	175.31	212.06	214.13	181.72	126.40	**175.37**
**mmu-miR-31-5p**	148.63	141.86	139.40	210.14	199.90	158.71	**166.44**
**mmu-miR-214-3p**	183.16	145.87	179.44	139.65	160.52	176.96	**164.27**
**mmu-miR-28a-3p**	123.11	163.27	176.47	172.90	165.06	176.96	**162.96**
**mmu-miR-205-5p**	127.61	129.81	207.61	178.22	160.52	125.00	**154.80**
**mmu-let-7i-3p**	151.63	156.58	163.13	168.91	146.89	130.62	**152.96**

Biology Open • Supplementary information

**mmu-miR-541-5p**	259.72	115.09	140.88	142.31	143.86	112.36	**152.37**
**mmu-miR-500-3p**	175.65	157.91	189.82	148.96	112.06	125.00	**151.57**
**mmu-miR-126b-5p**	151.63	129.81	145.33	147.63	139.32	148.88	**143.77**
**mmu-miR-345-3p**	174.15	131.15	145.33	155.61	118.12	136.23	**143.43**
**mmu-miR-148b-3p**	136.62	119.10	134.95	174.23	116.61	119.38	**133.48**
**mmu-miR-10a-3p**	138.12	116.43	145.33	130.34	146.89	105.34	**130.41**
**mmu-miR-199b-5p**	106.59	206.09	176.47	97.09	119.63	66.01	**128.65**
**mmu-miR-181a-1-3p**	123.11	141.86	118.64	134.33	133.26	117.98	**128.20**
**mmu-miR-92a-3p**	127.61	96.35	151.26	129.01	104.49	154.49	**127.20**
**mmu-miR-361-5p**	138.12	111.08	121.60	133.00	102.98	143.26	**125.01**
**mmu-miR-106b-5p**	118.60	107.06	143.85	106.40	159.01	94.10	**121.50**
**mmu-miR-19a-3p**	126.11	92.34	124.57	146.30	131.75	101.12	**120.37**
**mmu-miR-125b-2-3p**	156.13	128.47	106.77	98.42	99.95	125.00	**119.12**
**mmu-miR-672-5p**	102.09	129.81	127.53	111.72	101.46	140.45	**118.84**
**mmu-miR-3535**	76.57	112.41	120.12	150.29	152.95	98.31	**118.44**
**mmu-miR-30b-3p**	132.11	111.08	94.91	129.01	109.03	122.19	**116.39**
**mmu-miR-708-5p**	88.58	137.84	136.43	106.40	74.20	130.62	**112.35**
**mmu-miR-133a-5p**	294.25	113.75	100.84	2.66	4.54	117.98	**105.67**
**mmu-miR-99a-5p**	120.10	93.68	130.50	101.08	90.86	89.89	**104.35**
**mmu-miR-669o-3p**	69.06	85.65	96.39	127.68	165.06	81.46	**104.22**
**mmu-miR-378c**	79.57	92.34	136.43	115.71	101.46	84.27	**101.63**
**mmu-miR-98-5p**	93.08	62.90	108.26	83.79	107.52	94.10	**91.61**
**mmu-miR-138-5p**	91.58	82.97	130.50	75.81	80.26	88.48	**91.60**
**mmu-miR-365-3p**	97.58	96.35	117.15	63.84	74.20	98.31	**91.24**
**mmu-miR-224-5p**	111.10	78.96	78.60	90.44	86.32	95.50	**90.15**
**mmu-miR-130a-3p**	67.56	91.00	100.84	90.44	89.35	91.29	**88.41**
**mmu-miR-676-3p**	91.58	69.59	80.08	93.10	78.75	112.36	**87.58**
**mmu-let-7j**	85.57	74.94	84.53	99.75	90.86	80.06	**85.95**
**mmu-miR-99b-5p**	60.05	81.63	96.39	90.44	92.38	88.48	**84.90**
**mmu-miR-27a-5p**	94.58	76.28	66.73	73.15	106.01	85.67	**83.74**
**mmu-miR-335-5p**	49.54	54.87	105.29	114.38	90.86	81.46	**82.73**

Biology Open • Supplementary information

**mmu-miR-140-5p**	70.56	70.93	81.56	81.13	92.38	96.91	**82.25**
**mmu-miR-3105-5p**	67.56	93.68	87.49	82.46	60.57	75.84	**77.93**
**mmu-miR-193a-5p**	73.56	69.59	75.63	58.52	74.20	99.72	**75.20**
**mmu-miR-1198-5p**	78.07	53.53	83.05	87.78	69.66	75.84	**74.66**
**mmu-miR-338-3p**	109.59	84.31	109.74	41.23	53.00	46.35	**74.04**
**mmu-miR-362-3p**	87.08	104.38	84.53	54.53	83.29	28.09	**73.65**
**mmu-miR-18a-5p**	76.57	64.24	77.11	77.14	72.69	60.39	**71.36**
**mmu-miR-33-5p**	97.58	81.63	56.35	41.23	59.06	67.42	**67.21**
**mmu-miR-30d-3p**	58.55	85.65	68.22	63.84	43.92	61.80	**63.66**
**mmu-miR-378b**	63.05	77.62	65.25	50.54	62.09	61.80	**63.39**
**mmu-miR-22-5p**	63.05	64.24	68.22	58.52	60.57	61.80	**62.73**
**mmu-miR-133b-3p**	157.64	78.96	83.05	0.00	1.51	54.77	**62.66**
**mmu-miR-324-5p**	46.54	61.56	62.28	73.15	56.03	60.39	**59.99**
**mmu-miR-450b-3p**	73.56	53.53	56.35	63.84	48.46	60.39	**59.36**
**mmu-miR-17-5p**	60.05	49.52	56.35	77.14	65.12	44.94	**58.85**
**mmu-miR-17-3p**	48.04	48.18	84.53	62.51	48.46	46.35	**56.35**
**mmu-miR-186-5p**	31.53	46.84	59.32	66.50	63.60	46.35	**52.36**
**mmu-miR-340-3p**	52.55	52.19	35.59	63.84	57.55	44.94	**51.11**
**mmu-miR-455-5p**	43.54	46.84	54.87	51.87	56.03	53.37	**51.09**
**mmu-miR-150-5p**	24.02	13.38	31.14	123.69	86.32	26.69	**50.87**
**mmu-miR-362-5p**	49.54	52.19	57.84	38.57	56.03	46.35	**50.09**
**mmu-miR-9-5p**	57.05	54.87	57.84	27.93	48.46	49.16	**49.22**
**mmu-miR-708-3p**	52.55	52.19	40.04	57.19	33.32	57.58	**48.81**
**mmu-miR-3473b**	57.05	26.77	35.59	54.53	86.32	28.09	**48.06**
**mmu-miR-676-5p**	39.03	54.87	51.90	53.20	40.89	46.35	**47.71**
**mmu-miR-434-5p**	70.56	56.21	45.97	46.55	39.37	26.69	**47.56**
**mmu-miR-7a-1-3p**	52.55	40.15	48.94	38.57	48.46	50.56	**46.54**
**mmu-miR-15b-3p**	43.54	33.46	34.11	55.86	56.03	47.75	**45.13**
**mmu-miR-3071-3p**	84.07	25.43	47.45	41.23	43.92	22.47	**44.10**
**mmu-miR-872-3p**	57.05	37.47	37.07	54.53	43.92	33.71	**43.96**
**mmu-miR-669c-5p**	43.54	34.79	54.87	46.55	43.92	39.33	**43.83**

Biology Open • Supplementary information

**mmu-miR-30c-2-3p**	37.53	52.19	45.97	33.25	48.46	43.54	**43.49**
**mmu-miR-1191a**	45.04	56.21	48.94	31.92	33.32	36.52	**41.99**
**mmu-miR-144-5p**	49.54	42.82	26.69	35.91	49.97	42.13	**41.18**
**mmu-miR-155-5p**	30.03	24.09	31.14	77.14	54.52	29.49	**41.07**
**mmu-miR-378a-5p**	24.02	40.15	47.45	39.90	45.43	47.75	**40.78**
**mmu-miR-29c-5p**	31.53	41.49	50.42	41.23	31.80	47.75	**40.70**
**mmu-miR-379-5p**	61.55	45.50	34.11	47.88	37.86	16.85	**40.63**
**mmu-miR-345-5p**	36.03	38.81	59.32	38.57	22.72	32.30	**37.96**
**mmu-miR-674-3p**	36.03	38.81	48.94	29.26	25.74	44.94	**37.29**
**mmu-miR-652-3p**	36.03	44.16	31.14	27.93	33.32	46.35	**36.49**
**mmu-miR-204-5p**	57.05	29.44	26.69	49.21	30.29	21.07	**35.63**
**mmu-miR-183-3p**	43.54	32.12	41.52	23.94	37.86	32.30	**35.21**
**mmu-let-7a-1-3p**	39.03	36.13	37.07	27.93	42.40	26.69	**34.88**
**mmu-miR-450b-5p**	31.53	33.46	54.87	14.63	36.34	33.71	**34.09**
**mmu-miR-690**	25.52	34.79	29.66	19.95	56.03	36.52	**33.75**
**mmu-miR-381-3p**	58.55	34.79	25.21	29.26	25.74	26.69	**33.37**
**mmu-miR-222-3p**	40.54	37.47	31.14	27.93	15.14	47.75	**33.33**
**mmu-miR-28a-5p@**	43.54	30.78	35.59	35.91	24.23	25.28	**32.56**
**mmu-miR-542-5p**	28.52	38.81	22.24	33.25	28.77	32.30	**30.65**
**mmu-miR-148a-5p**	42.04	34.79	31.14	31.92	24.23	15.45	**29.93**
**mmu-miR-466c-3p@**	28.52	20.07	38.56	34.58	34.83	19.66	**29.37**
**mmu-miR-574-3p**	25.52	32.12	25.21	29.26	28.77	32.30	**28.86**
**mmu-miR-423-5p**	36.03	18.74	29.66	25.27	10.60	47.75	**28.01**
**mmu-miR-128-3p**	40.54	17.40	26.69	25.27	21.20	35.11	**27.70**
**mmu-miR-376b-5p**	51.04	30.78	25.21	21.28	22.72	12.64	**27.28**
**mmu-miR-1843a-3p**	18.02	28.10	37.07	18.62	33.32	25.28	**26.74**
**mmu-miR-5099**	30.03	25.43	20.76	46.55	13.63	22.47	**26.48**
**mmu-miR-203-5p**	22.52	36.13	26.69	17.29	28.77	25.28	**26.11**
**mmu-miR-29a-5p**	21.02	26.77	28.18	27.93	25.74	26.69	**26.06**
**mmu-miR-350-3p**	21.02	33.46	32.63	27.93	19.69	21.07	**25.97**
**mmu-miR-671-5p**	30.03	30.78	25.21	29.26	21.20	16.85	**25.56**

Biology Open • Supplementary information

**mmu-miR-30f**	28.52	26.77	37.07	23.94	21.20	14.04	**25.26**
**mmu-miR-3964**	18.02	20.07	22.24	38.57	40.89	11.24	**25.17**
**mmu-miR-214-5p**	27.02	22.75	23.73	26.60	24.23	23.88	**24.70**
**mmu-miR-206-3p**	69.06	22.75	14.83	0.00	3.03	36.52	**24.37**
**mmu-miR-187-3p**	24.02	20.07	34.11	19.95	22.72	25.28	**24.36**
**mmu-miR-503-5p**	27.02	18.74	14.83	25.27	18.17	40.73	**24.13**
**mmu-miR-29b-1-5p**	24.02	16.06	19.28	26.60	21.20	36.52	**23.95**
**mmu-miR-218-5p**	27.02	25.43	25.21	25.27	22.72	16.85	**23.75**
**mmu-miR-431-5p**	30.03	24.09	19.28	21.28	22.72	23.88	**23.55**
**mmu-miR-195a-3p**	13.51	20.07	26.69	27.93	28.77	18.26	**22.54**
**mmu-miR-425-3p**	18.02	34.79	16.31	23.94	18.17	22.47	**22.28**
**mmu-miR-139-5p**	12.01	21.41	22.24	25.27	16.66	26.69	**20.71**
**mmu-miR-27b-5p**	28.52	17.40	16.31	22.61	19.69	19.66	**20.70**
**mmu-miR-664-5p**	22.52	17.40	17.80	10.64	24.23	25.28	**19.65**
**mmu-miR-34c-3p**	15.01	21.41	35.59	11.97	22.72	9.83	**19.42**
**mmu-miR-190b-5p**	24.02	12.04	16.31	34.58	16.66	12.64	**19.38**
**mmu-miR-34b-5p**	21.02	8.03	34.11	18.62	19.69	11.24	**18.79**
**mmu-miR-322-3p**	10.51	8.03	20.76	15.96	22.72	30.90	**18.15**
**mmu-miR-30e-3p**	21.02	20.07	16.31	21.28	10.60	18.26	**17.92**
**mmu-miR-382-5p**	30.03	10.71	10.38	18.62	9.09	28.09	**17.82**
**mmu-miR-184-3p**	12.01	18.74	26.69	11.97	18.17	18.26	**17.64**
**mmu-miR-411-5p**	28.52	21.41	17.80	13.30	13.63	8.43	**17.18**
**mmu-miR-3473a**	21.02	8.03	17.80	13.30	27.26	15.45	**17.14**
**mmu-miR-486a-5p**	28.52	16.06	19.28	7.98	15.14	15.45	**17.07**
**mmu-miR-664-3p**	15.01	16.06	32.63	14.63	12.11	9.83	**16.71**
**mmu-miR-351-5p**	15.01	20.07	16.31	17.29	18.17	11.24	**16.35**
**mmu-miR-1291**	4.50	18.74	23.73	22.61	21.20	7.02	**16.30**
**mmu-miR-34b-3p**	19.52	10.71	22.24	15.96	18.17	9.83	**16.07**
**mmu-miR-181b-1-3p**	12.01	16.06	11.86	6.65	25.74	23.88	**16.03**
**mmu-miR-672-3p**	9.01	16.06	19.28	15.96	19.69	15.45	**15.91**
**mmu-miR-374c-3p**	22.52	16.06	11.86	14.63	12.11	16.85	**15.67**

Biology Open • Supplementary information

**mmu-let-7d-3p**	13.51	20.07	16.31	10.64	13.63	16.85	**15.17**
**mmu-miR-9-3p**	21.02	12.04	17.80	19.95	9.09	8.43	**14.72**
**mmu-miR-100-5p**	12.01	21.41	13.35	14.63	13.63	12.64	**14.61**
**mmu-miR-574-5p**	12.01	17.40	11.86	11.97	16.66	16.85	**14.46**
**mmu-miR-181b-2-3p**	10.51	16.06	10.38	9.31	13.63	22.47	**13.73**
**mmu-miR-5123**	12.01	25.43	13.35	9.31	12.11	9.83	**13.67**
**mmu-miR-532-3p**	3.00	13.38	14.83	22.61	13.63	14.04	**13.58**
**mmu-miR-342-5p**	10.51	9.37	17.80	14.63	19.69	7.02	**13.17**
**mmu-miR-106b-3p**	7.51	13.38	8.90	23.94	16.66	8.43	**13.14**
**mmu-miR-421-3p**	15.01	12.04	7.41	19.95	7.57	14.04	**12.67**
**mmu-miR-186-3p**	12.01	13.38	17.80	13.30	4.54	12.64	**12.28**
**mmu-miR-16-1-3p**	7.51	17.40	13.35	9.31	12.11	8.43	**11.35**
**mmu-let-7g-3p**	9.01	8.03	23.73	6.65	16.66	2.81	**11.15**
**mmu-miR-3064-5p**	10.51	13.38	11.86	6.65	7.57	16.85	**11.14**
**mmu-miR-300-3p**	12.01	10.71	8.90	9.31	16.66	8.43	**11.00**
**mmu-miR-146b-3p**	15.01	6.69	7.41	13.30	13.63	9.83	**10.98**
**mmu-miR-582-3p**	7.51	17.40	13.35	6.65	9.09	11.24	**10.87**
**mmu-miR-148b-5p**	12.01	8.03	7.41	7.98	19.69	8.43	**10.59**
**mmu-miR-744-5p**	9.01	6.69	14.83	10.64	9.09	12.64	**10.48**
**mmu-miR-1968-5p**	10.51	14.72	8.90	9.31	12.11	7.02	**10.43**
**mmu-miR-125a-3p**	9.01	8.03	7.41	13.30	9.09	15.45	**10.38**
**mmu-miR-466d-3p**	4.50	8.03	10.38	14.63	19.69	4.21	**10.24**
**mmu-miR-122-5p**	0.00	0.00	1.48	2.66	1.51	54.77	**10.07**
**mmu-miR-466i-5p**	12.01	10.71	7.41	7.98	12.11	9.83	**10.01**
**mmu-miR-223-5p**	16.51	6.69	4.45	7.98	12.11	8.43	**9.36**
**mmu-miR-486a-3p@**	9.01	6.69	7.41	10.64	7.57	12.64	**8.99**
**mmu-miR-7116-5p**	6.01	8.03	11.86	6.65	13.63	7.02	**8.87**
**mmu-miR-127-3p**	10.51	4.01	4.45	13.30	13.63	7.02	**8.82**
**mmu-miR-132-3p**	4.50	13.38	8.90	3.99	9.09	12.64	**8.75**
**mmu-miR-127-5p**	7.51	18.74	8.90	3.99	4.54	8.43	**8.69**
**mmu-miR-3068-3p**	6.01	8.03	13.35	7.98	10.60	5.62	**8.60**

Biology Open • Supplementary information

**mmu-miR-339-3p**	4.50	10.71	8.90	5.32	10.60	9.83	**8.31**
**mmu-miR-181c-5p**	7.51	4.01	14.83	9.31	7.57	5.62	**8.14**
**mmu-miR-193b-5p**	6.01	4.01	7.41	9.31	6.06	14.04	**7.81**
**mmu-miR-221-5p**	12.01	5.35	16.31	6.65	3.03	2.81	**7.69**
**mmu-miR-1843b-3p**	4.50	6.69	13.35	5.32	9.09	7.02	**7.66**
**mmu-miR-361-3p**	6.01	13.38	8.90	5.32	9.09	2.81	**7.59**
**mmu-miR-93-3p**	3.00	9.37	7.41	13.30	6.06	5.62	**7.46**
**mmu-miR-1981-5p**	10.51	14.72	4.45	5.32	6.06	2.81	**7.31**
**mmu-miR-152-5p**	4.50	13.38	10.38	3.99	1.51	9.83	**7.27**
**mmu-miR-503-3p**	7.51	4.01	8.90	2.66	6.06	14.04	**7.20**
**mmu-miR-139-3p**	9.01	1.34	4.45	6.65	7.57	14.04	**7.18**
**mmu-miR-181d-5p**	18.02	1.34	4.45	6.65	10.60	1.40	**7.08**
**mmu-miR-466q**	7.51	2.68	13.35	10.64	1.51	5.62	**6.89**
**mmu-miR-200c-5p**	7.51	6.69	13.35	3.99	4.54	4.21	**6.72**
**mmu-miR-450a-1-3p**	6.01	6.69	5.93	7.98	6.06	5.62	**6.38**
**mmu-miR-20b-5p**	3.00	1.34	1.48	21.28	7.57	2.81	**6.25**
**mmu-miR-423-3p**	6.01	4.01	8.90	3.99	6.06	8.43	**6.23**
**mmu-miR-219a-5p**	10.51	0.00	16.31	3.99	4.54	1.40	**6.13**
**mmu-let-7b-3p**	3.00	5.35	11.86	3.99	7.57	4.21	**6.00**
**mmu-miR-301b-3p**	7.51	1.34	5.93	11.97	4.54	4.21	**5.92**
**mmu-miR-6240**	7.51	6.69	11.86	3.99	1.51	2.81	**5.73**
**mmu-miR-331-3p**	3.00	8.03	8.90	7.98	4.54	1.40	**5.64**
**mmu-miR-467e-5p**	7.51	5.35	2.97	2.66	7.57	7.02	**5.51**
**mmu-miR-3058-5p**	4.50	5.35	5.93	5.32	9.09	2.81	**5.50**
**mmu-miR-181a-2-3p**	4.50	4.01	8.90	3.99	4.54	7.02	**5.49**
**mmu-miR-130b-3p**	6.01	2.68	5.93	5.32	4.54	8.43	**5.49**
**mmu-miR-582-5p**	3.00	5.35	5.93	5.32	9.09	4.21	**5.48**
**mmu-miR-491-5p**	4.50	2.68	5.93	7.98	3.03	8.43	**5.43**
**mmu-miR-374b-5p@**	9.01	4.01	0.00	7.98	3.03	8.43	**5.41**
**mmu-miR-154-5p**	10.51	1.34	1.48	9.31	4.54	4.21	**5.23**
**mmu-miR-15a-3p**	9.01	5.35	1.48	2.66	6.06	5.62	**5.03**

Biology Open • Supplementary information

**mmu-miR-450a-5p**	3.00	2.68	7.41	7.98	6.06	2.81	**4.99**
**mmu-miR-877-5p**	6.01	5.35	4.45	6.65	3.03	4.21	**4.95**
**mmu-miR-5121**	1.50	5.35	8.90	2.66	6.06	4.21	**4.78**
**mmu-let-7k**	6.01	9.37	0.00	5.32	3.03	4.21	**4.66**
**mmu-miR-188-5p**	0.00	1.34	11.86	1.33	7.57	5.62	**4.62**
**mmu-miR-511-3p**	10.51	4.01	7.41	2.66	0.00	2.81	**4.57**
**mmu-miR-497a-3p**	6.01	5.35	4.45	3.99	4.54	2.81	**4.53**
**mmu-miR-99b-3p**	1.50	5.35	7.41	6.65	3.03	2.81	**4.46**
**mmu-miR-6538**	10.51	4.01	1.48	6.65	0.00	2.81	**4.24**
**mmu-miR-29b-2-5p**	6.01	1.34	4.45	9.31	1.51	2.81	**4.24**
**mmu-miR-181c-3p**	1.50	12.04	2.97	2.66	4.54	1.40	**4.19**
**mmu-miR-3084-3p**	4.50	0.00	7.41	3.99	4.54	4.21	**4.11**
**mmu-miR-92a-1-5p**	4.50	5.35	4.45	2.66	4.54	2.81	**4.05**
**mmu-miR-134-5p**	12.01	2.68	0.00	1.33	6.06	1.40	**3.91**
**mmu-miR-3105-3p**	4.50	2.68	5.93	3.99	1.51	4.21	**3.80**
**mmu-miR-5126**	6.01	6.69	4.45	3.99	1.51	0.00	**3.78**
**mmu-miR-20a-3p**	3.00	5.35	2.97	3.99	3.03	4.21	**3.76**
**mmu-miR-31-3p**	3.00	2.68	5.93	3.99	4.54	1.40	**3.59**
**mmu-miR-3066-5p**	4.50	2.68	2.97	5.32	3.03	2.81	**3.55**
**mmu-miR-434-3p**	10.51	2.68	1.48	0.00	3.03	2.81	**3.42**
**mmu-miR-452-3p**	3.00	2.68	4.45	2.66	4.54	2.81	**3.36**
**mmu-miR-466f-3p**	1.50	2.68	4.45	6.65	4.54	0.00	**3.30**
**mmu-miR-205-3p**	3.00	1.34	4.45	1.33	6.06	2.81	**3.17**
**mmu-miR-299a-5p@**	7.51	1.34	2.97	3.99	3.03	0.00	**3.14**
**mmu-miR-124-3p**	6.01	4.01	4.45	1.33	1.51	1.40	**3.12**
**mmu-miR-669f-3p**	3.00	2.68	2.97	3.99	6.06	0.00	**3.12**
**mmu-miR-191-3p**	1.50	0.00	1.48	5.32	4.54	5.62	**3.08**
**mmu-miR-669a-5p**	0.00	4.01	5.93	5.32	1.51	1.40	**3.03**
**mmu-miR-5113**	3.00	6.69	4.45	3.99	0.00	0.00	**3.02**
**mmu-miR-190a-3p**	4.50	2.68	2.97	0.00	3.03	4.21	**2.90**
**mmu-miR-467d-5p**	4.50	2.68	5.93	1.33	0.00	2.81	**2.88**

Biology Open • Supplementary information

**mmu-miR-16-2-3p**	3.00	2.68	1.48	1.33	4.54	4.21	**2.87**
**mmu-miR-26b-3p**	3.00	2.68	1.48	2.66	4.54	2.81	**2.86**
**mmu-miR-3473e**	1.50	4.01	1.48	3.99	4.54	1.40	**2.82**
**mmu-miR-378d**	3.00	2.68	4.45	5.32	0.00	1.40	**2.81**
**mmu-miR-132-5p**	4.50	0.00	2.97	1.33	3.03	4.21	**2.67**
**mmu-miR-450a-2-3p**	3.00	2.68	2.97	2.66	4.54	0.00	**2.64**
**mmu-miR-467a-3p@**	4.50	1.34	0.00	3.99	4.54	1.40	**2.63**
**mmu-miR-3968**	3.00	1.34	2.97	3.99	0.00	4.21	**2.59**
**mmu-miR-1839-3p**	1.50	5.35	1.48	1.33	3.03	2.81	**2.58**
**mmu-miR-467c-5p**	1.50	2.68	1.48	3.99	3.03	2.81	**2.58**
**mmu-miR-1955-3p**	3.00	4.01	2.97	3.99	1.51	0.00	**2.58**
**mmu-miR-297c-3p**	1.50	0.00	2.97	7.98	3.03	0.00	**2.58**
**mmu-miR-700-3p**	3.00	1.34	1.48	5.32	1.51	2.81	**2.58**
**mmu-miR-350-5p**	1.50	4.01	0.00	3.99	3.03	2.81	**2.56**
**mmu-miR-1193-3p**	3.00	2.68	1.48	6.65	1.51	0.00	**2.55**
**mmu-miR-598-3p**	0.00	4.01	1.48	3.99	3.03	2.81	**2.55**
**mmu-miR-409-5p**	0.00	0.00	2.97	9.31	1.51	1.40	**2.53**
**mmu-miR-136-5p**	4.50	2.68	1.48	0.00	6.06	0.00	**2.45**
**mmu-miR-499-5p**	4.50	0.00	4.45	1.33	1.51	2.81	**2.43**
**mmu-miR-7033-5p**	4.50	0.00	0.00	3.99	6.06	0.00	**2.43**
**mmu-miR-150-3p**	4.50	1.34	1.48	2.66	4.54	0.00	**2.42**
**mmu-miR-6238**	3.00	1.34	2.97	2.66	3.03	1.40	**2.40**
**mmu-miR-2137**	4.50	2.68	2.97	1.33	1.51	1.40	**2.40**
**mmu-miR-337-5p**	1.50	2.68	2.97	1.33	3.03	2.81	**2.39**
**mmu-miR-330-5p**	1.50	5.35	1.48	0.00	3.03	2.81	**2.36**
**mmu-miR-326-3p**	3.00	1.34	4.45	5.32	0.00	0.00	**2.35**
**mmu-miR-147-3p**	3.00	4.01	2.97	2.66	0.00	1.40	**2.34**
**mmu-miR-219a-1-3p**	1.50	0.00	2.97	1.33	4.54	2.81	**2.19**
**mmu-miR-328-3p**	0.00	1.34	2.97	2.66	6.06	0.00	**2.17**
**mmu-miR-18a-3p**	4.50	0.00	2.97	3.99	1.51	0.00	**2.16**
**mmu-miR-193b-3p**	4.50	1.34	1.48	1.33	1.51	2.81	**2.16**

Biology Open • Supplementary information

**mmu-miR-409-3p**	4.50	2.68	0.00	0.00	1.51	4.21	**2.15**
**mmu-miR-5615-5p**	1.50	2.68	0.00	2.66	6.06	0.00	**2.15**
**mmu-miR-455-3p**	3.00	1.34	1.48	2.66	1.51	2.81	**2.13**
**mmu-miR-701-5p**	3.00	2.68	1.48	0.00	0.00	5.62	**2.13**
**mmu-miR-485-5p**	1.50	1.34	1.48	2.66	1.51	4.21	**2.12**
**mmu-miR-3084-5p**	0.00	1.34	1.48	1.33	1.51	7.02	**2.11**
**mmu-miR-1306-5p**	1.50	5.35	1.48	1.33	1.51	1.40	**2.10**
**mmu-miR-335-3p**	0.00	4.01	1.48	2.66	1.51	2.81	**2.08**
**mmu-miR-501-3p**	1.50	1.34	2.97	0.00	6.06	0.00	**1.98**
**mmu-miR-182-3p**	1.50	1.34	5.93	0.00	3.03	0.00	**1.97**
**mmu-miR-3108-5p**	3.00	1.34	1.48	0.00	4.54	1.40	**1.96**
**mmu-miR-3076-3p**	3.00	0.00	2.97	1.33	3.03	1.40	**1.96**
**mmu-miR-3069-5p**	0.00	1.34	0.00	1.33	6.06	2.81	**1.92**
**mmu-miR-501-5p**	3.00	2.68	2.97	1.33	1.51	0.00	**1.92**
**mmu-miR-363-3p**	1.50	4.01	2.97	0.00	1.51	1.40	**1.90**
**mmu-miR-101a-5p**	1.50	2.68	1.48	0.00	1.51	4.21	**1.90**
**mmu-miR-376c-3p**	3.00	2.68	0.00	1.33	1.51	2.81	**1.89**
**mmu-miR-34a-3p**	0.00	5.35	1.48	1.33	1.51	1.40	**1.85**
**mmu-miR-21a-3p**	3.00	1.34	1.48	1.33	3.03	0.00	**1.70**
**mmu-miR-3470a**	0.00	1.34	4.45	1.33	1.51	1.40	**1.67**
**mmu-miR-5114**	1.50	0.00	2.97	3.99	1.51	0.00	**1.66**
**mmu-miR-3057-5p**	1.50	1.34	4.45	2.66	0.00	0.00	**1.66**
**mmu-miR-466h-5p**	0.00	1.34	1.48	2.66	1.51	2.81	**1.63**
**mmu-miR-3470b**	1.50	1.34	0.00	2.66	0.00	4.21	**1.62**
**mmu-miR-3076-5p**	0.00	4.01	0.00	1.33	1.51	2.81	**1.61**
**mmu-miR-669p-3p**	1.50	0.00	1.48	0.00	4.54	1.40	**1.49**
**mmu-miR-6516-5p**	1.50	0.00	2.97	0.00	3.03	1.40	**1.48**
**mmu-miR-6236**	4.50	0.00	1.48	1.33	1.51	0.00	**1.47**
**mmu-miR-25-5p**	3.00	0.00	1.48	0.00	1.51	2.81	**1.47**
**mmu-miR-135a-5p**	1.50	0.00	1.48	1.33	3.03	1.40	**1.46**
**mmu-miR-26a-2-3p**	0.00	0.00	1.48	2.66	4.54	0.00	**1.45**

Biology Open • Supplementary information

**mmu-miR-30c-1-3p**	3.00	0.00	0.00	1.33	1.51	2.81	**1.44**
**mmu-miR-3071-5p**	1.50	1.34	1.48	0.00	1.51	2.81	**1.44**
**mmu-miR-33-3p**	0.00	1.34	1.48	1.33	1.51	2.81	**1.41**
**mmu-miR-6946-5p**	0.00	1.34	2.97	1.33	0.00	2.81	**1.41**
**mmu-miR-137-3p**	1.50	1.34	0.00	1.33	0.00	4.21	**1.40**
**mmu-miR-222-5p**	0.00	1.34	0.00	2.66	1.51	2.81	**1.39**
**mmu-miR-1947-5p**	1.50	6.69	0.00	0.00	0.00	0.00	**1.37**
**mmu-miR-1981-3p**	0.00	1.34	0.00	5.32	0.00	1.40	**1.34**
**mmu-let-7f-1-3p**	1.50	1.34	2.97	0.00	1.51	0.00	**1.22**
**mmu-miR-6996-5p**	1.50	1.34	2.97	0.00	1.51	0.00	**1.22**
**mmu-miR-3544-3p**	1.50	0.00	4.45	1.33	0.00	0.00	**1.21**
**mmu-miR-421-5p**	0.00	1.34	1.48	0.00	3.03	1.40	**1.21**
**mmu-miR-376a-3p**	1.50	1.34	1.48	0.00	1.51	1.40	**1.21**
**mmu-miR-324-3p**	0.00	1.34	1.48	1.33	3.03	0.00	**1.20**
**mmu-miR-709**	1.50	1.34	1.48	1.33	1.51	0.00	**1.19**
**mmu-miR-369-5p**	0.00	1.34	4.45	1.33	0.00	0.00	**1.19**
**mmu-miR-466j**	0.00	1.34	1.48	1.33	0.00	2.81	**1.16**
**mmu-miR-7063-5p**	0.00	0.00	0.00	3.99	1.51	1.40	**1.15**
**mmu-miR-101b-5p**	0.00	2.68	1.48	1.33	0.00	1.40	**1.15**
**mmu-miR-376a-5p**	0.00	2.68	1.48	1.33	0.00	1.40	**1.15**
**mmu-miR-5107-3p**	1.50	0.00	2.97	0.00	1.51	0.00	**1.00**

Biology Open • Supplementary information

Table S4. miRNA expression in the mammary gland at involution day 3. The number of reads determined by the sequencing is indicated for each of the animal, as well as the average of the group (N=6). Only miRNA with an average read for the group above 1 are listed.

miRNA	Inv_1	Inv_2	Inv_3	Inv_4	Inv_5	Inv_6	Average expression
**mmu-miR-143-3p**	265177.58	237485.10	231982.93	234466.07	295262.95	212943.93	**246219.76**
**mmu-miR-16-5p**	79632.14	96055.25	83541.16	88765.60	107347.74	93834.65	**91529.42**
**mmu-let-7c-5p**	75926.72	96431.49	94518.99	86763.70	76908.13	88418.27	**86494.55**
**mmu-let-7a-5p**	46342.60	52374.63	63439.19	63586.71	50916.50	62693.54	**56558.86**
**mmu-miR-199a-3p**	61682.58	58659.08	39668.39	45131.34	49049.68	39371.64	**48927.12**
**mmu-miR-29a-3p**	35584.19	34513.18	36926.41	35109.55	41244.29	41882.48	**37543.35**
**mmu-miR-142b**	51558.33	43266.58	25944.63	29080.29	34957.12	29455.67	**35710.44**
**mmu-miR-200a-3p**	16144.91	12214.27	47570.25	40262.26	19126.31	53996.30	**31552.38**
**mmu-miR-21a-5p**	49304.87	30619.37	26432.75	26941.18	29801.46	23914.65	**31169.05**
**mmu-miR-103-3p**	20547.99	28217.02	27406.03	29398.75	22798.04	26727.40	**25849.21**
**mmu-miR-27b-3p**	24621.54	20657.14	30478.04	30492.38	17959.55	29023.58	**25538.71**
**mmu-miR-146b-5p**	5787.25	7147.64	23926.92	24907.53	15062.17	26278.69	**17185.03**
**mmu-miR-126a-3p**	12714.95	12293.47	16462.79	17665.88	15374.58	17347.40	**15309.85**
**mmu-miR-146a-5p**	23197.90	16392.50	10577.65	14155.64	12458.15	11230.24	**14668.68**
**mmu-miR-142a-5p**	18118.61	18590.53	9684.40	11344.79	12287.66	13422.56	**13908.09**
**mmu-miR-23a-3p**	15751.02	15493.31	11520.30	9614.24	10437.04	9439.55	**12042.58**
**mmu-miR-125b-5p**	14034.76	21609.44	7878.15	7172.03	11815.24	6557.55	**11511.20**
**mmu-let-7f-5p**	9803.31	9991.94	12470.85	12801.92	10717.06	13155.27	**11490.06**
**mmu-miR-200b-3p**	6867.64	6767.80	16314.57	12811.14	6821.50	14873.95	**10742.77**
**mmu-let-7b-5p**	8264.68	13008.15	10306.90	10015.65	9923.66	9522.65	**10173.62**
**mmu-miR-148a-3p**	3405.07	2810.09	13758.35	13717.37	5579.50	15195.24	**9077.60**
**mmu-miR-101a-3p@**	8825.89	8623.79	9010.51	9713.57	6488.14	10526.71	**8864.77**
**mmu-miR-125a-5p**	8789.85	13952.35	7132.13	5977.03	7946.36	5898.34	**8282.68**
**mmu-miR-10a-5p**	6499.50	6596.78	6506.66	6174.66	7156.77	7623.94	**6759.72**
**mmu-miR-3074-5p**	6774.96	6443.77	6983.91	6841.28	4628.95	5697.52	**6228.40**
**mmu-miR-30a-5p**	3597.29	4774.99	7101.50	6744.00	6073.83	6945.33	**5872.82**
**mmu-miR-25-3p**	5458.58	5594.08	5606.50	5776.33	5381.39	6500.77	**5719.61**
**mmu-miR-195a-5p**	4828.71	6476.17	3747.88	4267.99	4764.20	3255.93	**4556.81**
**mmu-miR-200c-3p**	2504.03	3323.14	6855.46	4164.57	3230.74	6335.97	**4402.32**
**mmu-miR-26a-5p**	4247.76	5476.17	3921.78	4406.23	4124.14	3719.87	**4315.99**
**mmu-let-7i-5p**	2968.28	2595.87	4846.65	4932.56	3638.39	5642.13	**4103.98**
**mmu-let-7e-5p**	2828.41	3862.30	4036.40	4501.46	3364.08	3967.77	**3760.07**
**mmu-miR-23b-3p**	4682.83	5549.97	3442.55	2965.48	3083.11	2683.96	**3734.65**
**mmu-miR-30d-5p**	2706.55	4666.98	3780.48	3218.40	3991.75	3442.89	**3634.51**

Biology Open • Supplementary information

**mmu-miR-140-3p**	3484.88	3283.54	3330.90	3271.65	3666.01	2980.33	**3336.22**
**mmu-miR-30e-5p**	2921.08	3212.43	3123.39	3410.91	3332.65	3617.39	**3269.64**
**mmu-miR-199a-5p**	4599.59	3659.78	2525.59	2670.57	3096.44	2487.30	**3173.21**
**mmu-miR-203-3p**	3677.96	3585.97	3116.48	2818.02	2302.09	2764.28	**3044.13**
**mmu-miR-93-5p**	2878.18	3186.33	2842.77	3047.39	2803.08	2778.13	**2922.65**
**mmu-miR-221-3p**	2764.90	2813.69	2724.20	2752.49	2434.48	2458.22	**2658.00**
**mmu-miR-142a-3p**	3388.77	2684.98	1558.24	2035.69	2202.08	2082.91	**2325.45**
**mmu-miR-182-5p**	1121.58	1285.33	3100.67	2841.57	1304.87	3498.29	**2192.05**
**mmu-miR-26b-5p**	1875.02	2327.64	2056.24	2319.34	1973.49	2347.42	**2149.86**
**mmu-miR-196a-5p**	2550.37	2538.26	1773.65	1855.47	2394.48	1659.12	**2128.56**
**mmu-miR-15b-5p**	2558.95	2895.60	1667.92	1686.51	1690.61	1732.52	**2038.69**
**mmu-miR-342-3p**	2618.16	3070.22	1336.90	1397.75	2061.12	1487.39	**1995.26**
**mmu-miR-126b-3p**	1895.62	1495.95	1970.28	2201.58	1841.10	2364.04	**1961.43**
**mmu-miR-145a-5p**	2187.38	1747.98	1840.84	1726.45	1831.58	1695.13	**1838.23**
**mmu-miR-223-3p**	2032.92	1815.49	1641.24	1704.94	2226.85	1598.19	**1836.61**
**mmu-let-7d-5p**	1706.83	1832.59	1952.49	2109.42	1452.50	1841.93	**1815.96**
**mmu-miR-101b-3p**	1657.05	1550.86	1701.51	1866.73	1420.11	2121.68	**1719.66**
**mmu-miR-19b-3p**	1860.43	1062.11	1710.41	2003.95	1505.84	2171.54	**1719.05**
**mmu-let-7g-5p**	1502.59	1459.05	1910.99	1819.63	1440.12	1792.07	**1654.08**
**mmu-miR-29c-3p**	1789.21	1657.97	1428.80	1587.18	1869.68	1573.26	**1651.02**
**mmu-miR-322-5p**	1583.25	1548.16	1407.06	1600.50	1982.07	1618.96	**1623.33**
**mmu-miR-141-3p**	580.96	453.65	1661.00	1646.58	654.34	1954.11	**1158.44**
**mmu-miR-107-3p**	1133.59	1240.33	1245.01	1224.69	1007.70	1085.77	**1156.18**
**mmu-miR-497a-5p**	1495.73	1321.34	896.21	732.15	1149.62	670.30	**1044.23**
**mmu-miR-30c-5p**	787.77	834.39	1061.22	1082.36	685.77	1170.25	**936.96**
**mmu-miR-191-5p**	773.18	1127.82	705.51	780.28	866.74	857.26	**851.80**
**mmu-miR-15a-5p**	967.97	713.77	832.97	787.45	820.07	854.49	**829.45**
**mmu-miR-320-3p**	842.69	1062.11	885.34	629.75	551.47	819.87	**798.54**
**mmu-miR-141-5p**	295.20	279.03	1215.37	1232.88	481.94	1181.33	**780.96**
**mmu-miR-183-5p**	305.50	411.34	1157.07	1007.61	543.85	1227.03	**775.40**
**mmu-miR-429-3p**	476.26	392.44	1058.26	980.98	497.18	1091.31	**749.41**
**mmu-miR-196b-5p**	977.41	991.00	548.40	673.79	741.01	490.26	**736.98**
**mmu-miR-10b-5p**	653.04	657.97	737.13	710.65	794.35	854.49	**734.61**
**mmu-miR-425-5p**	967.12	765.08	658.08	594.94	514.33	688.30	**697.98**
**mmu-miR-192-5p**	444.51	924.40	790.48	598.01	501.95	904.35	**693.95**
**mmu-miR-872-5p**	829.81	610.26	720.33	691.19	610.53	688.30	**691.74**
**mmu-miR-205-5p**	1073.52	1042.31	482.19	406.52	682.91	443.17	**688.44**

Biology Open • Supplementary information

**mmu-miR-451a**	768.89	402.34	768.74	582.65	660.05	938.97	**686.94**
**mmu-miR-27a-3p**	885.59	530.15	801.35	775.16	463.85	660.60	**686.12**
**mmu-miR-194-5p**	484.84	759.68	632.39	599.03	522.90	616.29	**602.52**
**mmu-miR-152-3p**	432.50	441.05	633.37	711.67	463.85	800.48	**580.49**
**mmu-miR-24-3p**	584.39	585.06	671.91	678.91	444.80	512.42	**579.58**
**mmu-miR-181a-5p**	562.08	387.94	813.21	603.13	414.32	656.45	**572.86**
**mmu-miR-29b-3p**	561.22	505.85	459.47	582.65	418.13	616.29	**523.94**
**mmu-miR-1843a-5p@**	519.17	556.26	530.61	523.26	440.99	509.65	**513.32**
**mmu-miR-378a-3p**	452.24	434.75	570.14	552.95	501.95	505.49	**502.92**
**mmu-miR-301a-3p**	737.14	342.94	498.99	539.64	381.94	441.79	**490.41**
**mmu-miR-339-5p**	509.73	531.96	533.58	428.03	484.80	382.24	**478.39**
**mmu-miR-1b-5p**	305.50	292.53	225.29	270.33	1420.11	332.38	**474.36**
**mmu-miR-34a-5p**	283.18	276.33	644.24	735.22	335.27	540.12	**469.06**
**mmu-miR-214-3p**	816.08	770.48	323.11	267.26	360.03	227.13	**460.68**
**mmu-miR-3068-5p**	374.15	299.73	555.31	500.73	319.07	612.13	**443.52**
**mmu-miR-99a-5p**	751.72	538.26	295.44	254.97	455.27	275.60	**428.54**
**mmu-miR-199b-5p**	442.80	438.35	308.29	315.39	492.42	315.76	**385.50**
**mmu-miR-34c-5p**	572.37	441.95	284.57	293.89	349.55	357.31	**383.27**
**mmu-miR-151-3p**	356.13	448.25	494.05	371.71	275.26	337.92	**380.55**
**mmu-miR-151-5p**	229.98	237.62	547.41	382.97	197.16	589.97	**364.19**
**mmu-miR-145a-3p**	412.76	379.84	294.45	327.68	354.31	270.06	**339.85**
**mmu-miR-20a-5p**	344.97	259.23	374.49	350.20	222.88	401.62	**325.57**
**mmu-miR-541-5p**	599.83	204.32	256.91	271.36	309.55	292.22	**322.37**
**mmu-miR-10b-3p**	254.01	248.43	339.91	365.56	343.84	335.15	**314.48**
**mmu-miR-133a-3p**	250.57	284.43	205.53	180.22	742.92	195.27	**309.82**
**mmu-miR-181b-5p**	290.05	243.93	397.22	335.87	222.88	365.62	**309.26**
**mmu-miR-92a-3p**	433.36	535.56	243.07	166.91	233.35	240.97	**308.87**
**mmu-miR-22-3p**	405.04	303.33	317.18	282.62	229.54	279.75	**302.91**
**mmu-miR-126a-5p**	272.03	238.52	261.85	292.86	280.98	355.92	**283.69**
**mmu-miR-224-5p**	371.57	344.74	238.13	198.65	288.59	253.44	**282.52**
**mmu-miR-30b-5p**	242.85	207.92	332.99	302.08	197.16	342.07	**270.85**
**mmu-miR-31-5p**	181.07	284.43	319.16	411.64	162.87	257.59	**269.46**
**mmu-miR-143-5p**	321.80	246.63	273.70	276.48	232.40	218.82	**261.64**
**mmu-miR-542-3p**	242.85	218.72	250.98	252.93	248.59	311.61	**254.28**
**mmu-miR-32-5p**	281.47	171.02	233.19	301.05	223.83	304.68	**252.54**
**mmu-miR-532-5p**	249.72	252.03	216.39	224.25	335.27	224.36	**250.34**
**mmu-miR-150-5p**	301.20	481.55	147.23	176.13	223.83	153.73	**247.28**

Biology Open • Supplementary information

**mmu-miR-17-3p**	237.70	261.93	217.38	203.77	259.07	160.65	**223.42**
**mmu-miR-3535**	114.99	164.72	202.56	322.56	211.45	275.60	**215.31**
**mmu-miR-200b-5p**	90.10	90.91	354.73	280.57	156.20	264.52	**206.17**
**mmu-miR-125b-2-3p**	227.41	238.52	161.06	163.84	182.87	168.96	**190.44**
**mmu-miR-96-5p**	133.01	86.41	274.69	240.64	85.72	303.30	**187.30**
**mmu-miR-3065-5p**	229.98	247.53	162.05	150.53	212.40	98.33	**183.47**
**mmu-miR-674-5p**	191.36	215.12	190.70	206.85	142.87	139.88	**181.13**
**mmu-miR-185-5p**	173.34	216.92	169.95	180.22	196.21	135.72	**178.73**
**mmu-miR-340-5p**	134.73	87.31	172.92	217.09	173.35	281.14	**177.76**
**mmu-miR-99b-5p**	145.02	230.42	179.83	133.12	191.44	141.26	**170.18**
**mmu-miR-375-3p**	39.47	35.10	392.28	185.34	60.96	306.07	**169.87**
**mmu-miR-122-5p**	0.00	625.56	111.66	35.84	34.29	195.27	**167.10**
**mmu-miR-10a-3p**	165.62	179.12	173.91	140.29	172.39	157.88	**164.87**
**mmu-miR-1839-5p**	129.58	161.12	178.85	187.39	132.39	173.11	**160.41**
**mmu-miR-3964**	212.82	214.22	70.16	108.54	184.78	130.18	**153.45**
**mmu-miR-7a-5p**	126.15	112.51	153.16	166.91	119.06	229.90	**151.28**
**mmu-miR-27a-5p**	127.00	172.82	205.53	169.98	67.62	153.73	**149.45**
**mmu-miR-365-3p**	144.17	240.32	121.54	93.18	168.59	117.72	**147.59**
**mmu-miR-350-3p**	138.16	144.92	133.39	120.83	190.49	106.64	**139.07**
**mmu-miR-19a-3p**	159.61	126.01	96.83	106.49	157.16	186.96	**138.84**
**mmu-let-7i-3p**	162.19	79.21	161.06	168.96	100.01	160.65	**138.68**
**mmu-miR-338-3p**	152.75	181.82	104.74	89.09	187.63	72.02	**131.34**
**mmu-miR-126b-5p**	119.28	101.71	126.48	155.65	122.87	159.26	**130.88**
**mmu-miR-434-5p**	220.54	72.01	114.62	98.30	108.58	167.57	**130.27**
**mmu-miR-155-5p**	149.32	191.72	90.91	102.40	120.96	114.95	**128.38**
**mmu-miR-484**	118.42	122.41	148.22	116.73	74.29	138.49	**119.76**
**mmu-miR-28a-3p**	105.55	110.71	146.24	125.95	102.87	124.64	**119.33**
**mmu-miR-708-5p**	110.70	223.22	68.18	103.42	133.34	67.86	**117.79**
**mmu-miR-361-5p**	126.15	169.22	107.70	98.30	86.67	109.41	**116.24**
**mmu-miR-130a-3p**	126.15	151.22	99.80	100.35	95.25	83.09	**109.31**
**mmu-miR-30a-3p**	63.50	89.11	125.49	133.12	91.44	152.34	**109.17**
**mmu-miR-500-3p**	134.73	81.01	117.58	97.28	100.96	98.33	**104.98**
**mmu-miR-362-3p**	141.59	77.41	82.01	79.87	99.06	114.95	**99.15**
**mmu-miR-324-5p**	90.10	99.01	103.75	94.21	88.58	98.33	**95.66**
**mmu-miR-140-5p**	124.43	84.61	83.00	95.23	86.67	84.48	**93.07**
**mmu-miR-1198-5p**	84.96	123.31	82.01	106.49	81.91	77.56	**92.71**
**mmu-miR-669o-3p**	113.27	180.92	50.39	66.56	84.77	56.78	**92.12**

Biology Open • Supplementary information

**mmu-miR-22-5p**	139.02	84.61	99.80	74.75	62.86	87.25	**91.38**
**mmu-let-7j**	89.25	87.31	81.02	68.61	84.77	120.49	**88.58**
**mmu-miR-362-5p**	135.58	69.31	90.91	92.16	69.53	72.02	**88.25**
**mmu-miR-708-3p**	91.82	160.22	44.46	65.54	97.15	56.78	**86.00**
**mmu-miR-148b-3p**	62.64	52.21	86.95	99.33	77.15	128.80	**84.51**
**mmu-miR-144-3p**	78.09	67.51	100.79	84.99	62.86	101.10	**82.56**
**mmu-miR-106b-5p**	90.96	63.01	102.76	86.02	51.43	91.40	**80.93**
**mmu-miR-455-5p**	127.00	92.71	64.23	74.75	65.72	48.47	**78.81**
**mmu-miR-872-3p**	101.26	94.51	84.98	72.70	45.72	70.63	**78.30**
**mmu-miR-381-3p**	145.02	47.70	58.30	57.34	78.10	83.09	**78.26**
**mmu-miR-503-5p**	60.93	46.80	97.82	101.38	60.96	85.86	**75.63**
**mmu-miR-411-5p**	106.41	45.90	55.33	69.63	72.39	99.71	**74.90**
**mmu-miR-3071-3p**	139.88	35.10	47.43	67.58	54.29	103.87	**74.69**
**mmu-miR-210-3p**	26.60	22.50	137.35	116.73	38.10	103.87	**74.19**
**mmu-miR-379-5p**	109.84	30.60	57.31	60.42	74.29	108.02	**73.41**
**mmu-miR-138-5p**	63.50	66.61	82.01	94.21	85.72	42.93	**72.50**
**mmu-miR-345-3p**	68.65	72.91	67.19	79.87	60.00	84.48	**72.18**
**mmu-miR-652-3p**	104.69	72.91	73.12	53.25	65.72	60.94	**71.77**
**mmu-let-7d-3p**	55.78	173.72	51.38	58.37	28.57	51.24	**69.84**
**mmu-miR-15b-3p**	90.96	73.81	54.35	59.39	66.67	70.63	**69.30**
**mmu-miR-186-5p**	67.79	66.61	71.14	57.34	53.34	76.17	**65.40**
**mmu-miR-190a-5p**	32.61	25.20	93.87	68.61	28.57	137.11	**64.33**
**mmu-miR-184-3p**	61.79	102.61	43.48	38.91	88.58	48.47	**63.97**
**mmu-let-7a-1-3p**	52.35	65.71	77.07	53.25	61.91	73.40	**63.95**
**mmu-miR-204-5p**	102.98	82.81	48.42	44.03	58.10	44.32	**63.44**
**mmu-miR-200a-5p**	30.03	36.00	115.61	76.80	33.34	83.09	**62.48**
**mmu-miR-674-3p**	57.49	100.81	44.46	54.27	66.67	49.86	**62.26**
**mmu-miR-214-5p**	108.12	58.51	46.44	60.42	43.81	55.40	**62.12**
**mmu-miR-378c**	58.35	54.91	59.29	59.39	55.24	72.02	**59.87**
**mmu-miR-351-5p**	39.47	59.41	74.11	68.61	55.24	56.78	**58.94**
**mmu-miR-98-5p**	54.06	42.30	57.31	77.82	40.00	76.17	**57.94**
**mmu-miR-300-3p**	77.23	20.70	55.33	63.49	44.77	74.79	**56.05**
**mmu-miR-425-3p**	48.91	46.80	67.19	81.92	37.15	45.70	**54.61**
**mmu-miR-144-5p**	42.05	28.80	67.19	65.54	48.58	73.40	**54.26**
**mmu-miR-1a-3p**	30.89	30.60	24.70	29.70	160.01	48.47	**54.06**
**mmu-miR-100-5p**	75.52	79.21	38.54	33.79	54.29	34.62	**52.66**
**mmu-miR-676-3p**	32.61	46.80	68.18	63.49	48.58	55.40	**52.51**

**mmu-miR-9-5p**	52.35	63.91	54.35	45.06	60.00	36.01	**51.95**
**mmu-miR-17-5p**	52.35	41.40	58.30	49.15	36.19	70.63	**51.34**
**mmu-miR-690**	16.30	34.20	57.31	71.68	43.81	84.48	**51.30**
**mmu-miR-218-5p**	82.38	57.61	48.42	37.89	28.57	52.63	**51.25**
**mmu-miR-669c-5p**	58.35	72.01	27.67	46.08	57.15	44.32	**50.93**
**mmu-miR-29a-5p**	90.10	58.51	54.35	34.82	22.86	44.32	**50.83**
**mmu-miR-29c-5p**	50.63	68.41	48.42	55.30	28.57	44.32	**49.28**
**mmu-miR-466c-3p@**	66.08	89.11	25.69	36.86	44.77	31.85	**49.06**
**mmu-miR-30d-3p**	36.04	40.50	72.13	40.96	38.10	62.32	**48.34**
**mmu-miR-574-3p**	63.50	80.11	35.57	31.74	38.10	38.78	**47.97**
**mmu-miR-431-5p**	84.10	36.90	44.46	31.74	29.53	56.78	**47.25**
**mmu-miR-223-5p**	50.63	42.30	34.58	31.74	83.82	40.16	**47.21**
**mmu-miR-193a-5p**	42.91	63.91	51.38	37.89	46.67	38.78	**46.92**
**mmu-miR-195a-3p**	66.08	63.01	34.58	41.98	36.19	38.78	**46.77**
**mmu-miR-127-5p**	83.24	19.80	36.56	29.70	40.96	67.86	**46.35**
**mmu-miR-676-5p**	34.33	36.90	59.29	36.86	45.72	58.17	**45.21**
**mmu-miR-18a-5p**	40.33	38.70	50.39	53.25	31.43	54.01	**44.69**
**mmu-miR-30b-3p**	25.74	41.40	61.26	50.18	53.34	34.62	**44.42**
**mmu-miR-450b-5p**	35.18	35.10	53.36	58.37	40.96	41.55	**44.09**
**mmu-miR-1843a-3p**	44.62	54.91	41.50	32.77	53.34	36.01	**43.86**
**mmu-miR-671-5p**	41.19	49.51	40.51	43.01	42.86	44.32	**43.57**
**mmu-miR-450b-3p**	32.61	40.50	61.26	56.32	30.48	30.47	**41.94**
**mmu-miR-335-5p**	44.62	40.50	17.79	47.10	27.62	66.48	**40.69**
**mmu-miR-376b-5p**	55.78	29.70	40.51	34.82	20.00	40.16	**36.83**
**mmu-miR-222-3p**	41.19	28.80	40.51	34.82	33.34	34.62	**35.55**
**mmu-miR-181a-1-3p**	18.02	12.60	47.43	50.18	30.48	47.09	**34.30**
**mmu-miR-542-5p**	30.89	37.80	33.60	37.89	31.43	29.08	**33.45**
**mmu-miR-128-3p**	32.61	32.40	38.54	26.62	35.24	27.70	**32.19**
**mmu-miR-342-5p**	29.18	31.50	32.61	33.79	35.24	29.08	**31.90**
**mmu-miR-20b-5p**	46.34	41.40	20.75	30.72	25.72	24.93	**31.64**
**mmu-miR-139-5p**	26.60	41.40	43.48	34.82	27.62	15.23	**31.53**
**mmu-miR-486a-5p**	21.45	27.90	30.63	24.58	52.39	31.85	**31.47**
**mmu-miR-486a-3p@**	29.18	39.60	28.65	19.46	36.19	30.47	**30.59**
**mmu-miR-322-3p**	48.91	21.60	22.73	29.70	27.62	30.47	**30.17**
**mmu-miR-203-5p**	33.47	55.81	26.68	13.31	26.67	24.93	**30.15**
**mmu-miR-34b-5p**	54.06	40.50	15.81	18.43	24.76	20.77	**29.06**
**mmu-miR-3473b**	11.16	7.20	34.58	56.32	20.00	34.62	**27.31**

**mmu-miR-378b**	15.45	9.90	36.56	25.60	25.72	49.86	**27.18**
**mmu-miR-378a-5p**	20.60	16.20	31.62	27.65	21.91	42.93	**26.82**
**mmu-miR-106b-3p**	31.75	27.00	31.62	19.46	33.34	13.85	**26.17**
**mmu-miR-423-3p**	30.03	27.90	33.60	25.60	11.43	24.93	**25.58**
**mmu-miR-382-5p**	25.74	13.50	24.70	18.43	23.81	42.93	**24.85**
**mmu-miR-664-3p**	17.16	37.80	36.56	23.55	15.24	18.00	**24.72**
**mmu-miR-33-5p**	22.31	15.30	23.71	23.55	22.86	38.78	**24.42**
**mmu-miR-127-3p**	30.03	13.50	16.80	18.43	34.29	31.85	**24.15**
**mmu-miR-34b-3p**	49.77	31.50	13.83	14.34	18.10	12.46	**23.33**
**mmu-miR-30c-2-3p**	7.72	21.60	35.57	34.82	16.19	23.54	**23.24**
**mmu-miR-672-5p**	15.45	24.30	37.55	22.53	18.10	20.77	**23.12**
**mmu-miR-340-3p**	13.73	15.30	31.62	24.58	26.67	26.31	**23.04**
**mmu-miR-30f**	21.45	18.90	28.65	22.53	22.86	23.54	**22.99**
**mmu-miR-582-3p**	30.89	19.80	22.73	12.29	25.72	26.31	**22.96**
**mmu-miR-93-3p**	25.74	31.50	22.73	23.55	11.43	20.77	**22.62**
**mmu-miR-139-3p**	16.30	28.80	25.69	20.48	22.86	19.39	**22.25**
**mmu-miR-5099**	11.16	12.60	25.69	20.48	10.48	52.63	**22.17**
**mmu-miR-511-3p**	38.62	33.30	12.85	11.26	25.72	11.08	**22.14**
**mmu-miR-532-3p**	26.60	28.80	17.79	18.43	19.05	19.39	**21.68**
**mmu-miR-132-3p**	24.89	32.40	21.74	13.31	18.10	18.00	**21.41**
**mmu-miR-3105-5p**	4.29	4.50	34.58	38.91	15.24	27.70	**20.87**
**mmu-miR-16-1-3p**	27.46	16.20	20.75	23.55	26.67	8.31	**20.49**
**mmu-miR-186-3p**	15.45	16.20	28.65	19.46	21.91	19.39	**20.18**
**mmu-miR-345-5p**	18.02	20.70	20.75	17.41	15.24	27.70	**19.97**
**mmu-miR-7a-1-3p**	16.30	15.30	28.65	18.43	11.43	27.70	**19.64**
**mmu-miR-28a-5p@**	21.45	12.60	25.69	14.34	14.29	26.31	**19.11**
**mmu-miR-5121**	6.87	7.20	18.77	30.72	16.19	31.85	**18.60**
**mmu-miR-744-5p**	23.17	26.10	29.64	18.43	9.52	4.15	**18.50**
**mmu-miR-374c-3p**	24.03	12.60	23.71	16.38	15.24	18.00	**18.33**
**mmu-miR-187-3p**	13.73	5.40	27.67	19.46	14.29	27.70	**18.04**
**mmu-miR-423-5p**	14.59	33.30	17.79	17.41	12.38	9.69	**17.53**
**mmu-miR-219a-5p**	24.89	9.00	7.90	12.29	18.10	27.70	**16.65**
**mmu-miR-664-5p**	11.16	15.30	21.74	12.29	21.91	15.23	**16.27**
**mmu-miR-363-3p**	30.89	22.50	5.93	10.24	19.05	6.92	**15.92**
**mmu-miR-301b-3p**	29.18	10.80	16.80	15.36	14.29	8.31	**15.79**
**mmu-miR-3473a**	12.01	4.50	16.80	36.86	9.52	13.85	**15.59**
**mmu-miR-21a-3p**	17.16	15.30	17.79	14.34	13.33	12.46	**15.06**

**mmu-miR-133b-3p**	7.72	14.40	12.85	5.12	39.05	11.08	**15.04**
**mmu-miR-183-3p**	10.30	7.20	24.70	17.41	7.62	20.77	**14.67**
**mmu-miR-466d-3p**	18.02	19.80	13.83	5.12	17.14	13.85	**14.63**
**mmu-miR-1191a**	11.16	7.20	14.82	21.50	13.33	15.23	**13.87**
**mmu-miR-27b-5p**	12.01	16.20	18.77	9.22	11.43	15.23	**13.81**
**mmu-miR-34c-3p**	12.87	21.60	7.90	17.41	10.48	12.46	**13.79**
**mmu-miR-350-5p**	28.32	13.50	7.90	9.22	6.67	12.46	**13.01**
**mmu-miR-130b-3p**	11.16	15.30	14.82	11.26	16.19	8.31	**12.84**
**mmu-miR-154-5p**	24.03	0.90	11.86	10.24	14.29	11.08	**12.07**
**mmu-miR-9-3p**	6.87	16.20	4.94	14.34	15.24	13.85	**11.91**
**mmu-miR-30e-3p**	6.87	9.00	19.76	10.24	11.43	13.85	**11.86**
**mmu-miR-6240**	5.15	5.40	28.65	9.22	4.76	16.62	**11.63**
**mmu-miR-221-5p**	8.58	12.60	12.85	9.22	8.57	15.23	**11.18**
**mmu-miR-361-3p**	12.01	16.20	8.89	7.17	9.52	12.46	**11.04**
**mmu-miR-150-3p**	12.01	23.40	6.92	7.17	9.52	6.92	**10.99**
**mmu-miR-190b-5p**	10.30	14.40	8.89	12.29	5.71	13.85	**10.91**
**mmu-miR-331-3p**	9.44	23.40	9.88	7.17	6.67	8.31	**10.81**
**mmu-miR-497a-3p**	21.45	15.30	1.98	9.22	7.62	8.31	**10.65**
**mmu-miR-326-3p**	12.87	12.60	13.83	6.14	13.33	4.15	**10.49**
**mmu-miR-34a-3p**	6.87	9.00	16.80	11.26	2.86	13.85	**10.11**
**mmu-miR-455-3p**	18.88	18.90	7.90	4.10	5.71	4.15	**9.94**
**mmu-miR-15a-3p**	12.01	9.90	7.90	13.31	6.67	9.69	**9.91**
**mmu-miR-148a-5p**	4.29	3.60	8.89	17.41	6.67	16.62	**9.58**
**mmu-miR-1291**	8.58	7.20	9.88	5.12	9.52	16.62	**9.49**
**mmu-miR-467a-3p@**	8.58	24.30	4.94	1.02	9.52	5.54	**8.98**
**mmu-miR-574-5p**	8.58	9.00	6.92	15.36	7.62	5.54	**8.84**
**mmu-miR-181c-5p**	12.01	9.00	8.89	8.19	3.81	11.08	**8.83**
**mmu-miR-133a-5p**	6.87	7.20	2.96	3.07	28.57	4.15	**8.80**
**mmu-miR-1843b-3p**	5.15	10.80	12.85	6.14	4.76	12.46	**8.69**
**mmu-miR-99b-3p**	9.44	9.90	7.90	7.17	10.48	5.54	**8.41**
**mmu-miR-1981-5p**	4.29	9.00	6.92	10.24	11.43	8.31	**8.37**
**mmu-miR-181b-1-3p**	4.29	3.60	9.88	8.19	4.76	16.62	**7.89**
**mmu-miR-125b-1-3p**	18.02	12.60	6.92	3.07	3.81	2.77	**7.87**
**mmu-miR-491-5p**	7.72	11.70	8.89	5.12	9.52	4.15	**7.85**
**mmu-miR-450a-1-3p**	6.01	8.10	7.90	13.31	2.86	8.31	**7.75**
**mmu-miR-299a-5p@**	14.59	9.90	5.93	7.17	5.71	2.77	**7.68**
**mmu-miR-434-3p**	20.60	9.00	4.94	1.02	4.76	5.54	**7.64**

Biology Open • Supplementary information

**mmu-miR-328-3p**	1.72	11.70	9.88	7.17	2.86	8.31	**6.94**
**mmu-miR-29b-1-5p**	8.58	8.10	6.92	8.19	3.81	5.54	**6.86**
**mmu-miR-152-5p**	6.01	0.90	7.90	9.22	3.81	12.46	**6.72**
**mmu-miR-503-3p**	3.43	4.50	13.83	9.22	4.76	4.15	**6.65**
**mmu-miR-467e-5p**	6.01	11.70	3.95	3.07	4.76	9.69	**6.53**
**mmu-miR-3068-3p**	8.58	6.30	2.96	10.24	4.76	5.54	**6.40**
**mmu-miR-421-3p**	7.72	4.50	9.88	6.14	5.71	4.15	**6.35**
**mmu-miR-29b-2-5p**	5.15	6.30	8.89	8.19	6.67	2.77	**6.33**
**mmu-miR-598-3p**	4.29	9.90	0.00	9.22	8.57	5.54	**6.25**
**mmu-miR-669a-5p**	6.01	8.10	5.93	2.05	5.71	9.69	**6.25**
**mmu-miR-134-5p**	9.44	3.60	4.94	5.12	2.86	11.08	**6.17**
**mmu-miR-409-5p**	12.01	3.60	3.95	2.05	6.67	8.31	**6.10**
**mmu-miR-665-3p**	6.87	2.70	2.96	8.19	2.86	12.46	**6.01**
**mmu-miR-146b-3p**	2.57	3.60	7.90	9.22	2.86	9.69	**5.97**
**mmu-miR-136-5p**	12.01	3.60	0.00	4.10	4.76	9.69	**5.69**
**mmu-miR-3064-5p**	3.43	7.20	8.89	7.17	3.81	2.77	**5.55**
**mmu-miR-200c-5p**	0.00	2.70	6.92	8.19	2.86	12.46	**5.52**
**mmu-miR-147-3p**	7.72	3.60	3.95	3.07	10.48	4.15	**5.50**
**mmu-miR-700-5p**	12.01	7.20	6.92	3.07	0.95	2.77	**5.49**
**mmu-miR-672-3p**	6.87	8.10	8.89	1.02	3.81	4.15	**5.47**
**mmu-miR-181c-3p**	5.15	4.50	4.94	9.22	4.76	4.15	**5.45**
**mmu-miR-376c-3p**	9.44	2.70	4.94	6.14	1.90	6.92	**5.34**
**mmu-miR-20a-3p**	6.87	4.50	6.92	6.14	3.81	2.77	**5.17**
**mmu-miR-450a-5p**	11.16	3.60	6.92	3.07	1.90	4.15	**5.13**
**mmu-miR-374b-5p@**	7.72	5.40	3.95	5.12	2.86	5.54	**5.10**
**mmu-miR-205-3p**	5.15	4.50	6.92	6.14	4.76	2.77	**5.04**
**mmu-miR-297c-3p**	6.01	7.20	4.94	6.14	5.71	0.00	**5.00**
**mmu-let-7g-3p**	7.72	1.80	0.99	5.12	3.81	9.69	**4.86**
**mmu-miR-106a-5p**	4.29	7.20	2.96	4.10	4.76	5.54	**4.81**
**mmu-miR-330-5p**	8.58	5.40	3.95	4.10	3.81	2.77	**4.77**
**mmu-miR-124-3p**	6.87	6.30	3.95	6.14	2.86	1.38	**4.58**
**mmu-miR-92a-1-5p**	4.29	2.70	6.92	6.14	2.86	4.15	**4.51**
**mmu-miR-181d-5p**	12.01	4.50	3.95	4.10	0.95	1.38	**4.48**
**mmu-miR-132-5p**	6.87	8.10	2.96	5.12	3.81	0.00	**4.48**
**mmu-miR-339-3p**	5.15	2.70	6.92	3.07	4.76	4.15	**4.46**
**mmu-miR-2137**	0.86	0.90	6.92	6.14	2.86	8.31	**4.33**
**mmu-miR-466i-5p**	2.57	9.90	3.95	2.05	2.86	4.15	**4.25**

Biology Open • Supplementary information

**mmu-miR-3084-3p**	3.43	3.60	4.94	3.07	1.90	8.31	**4.21**
**mmu-miR-582-5p**	6.01	6.30	3.95	5.12	3.81	0.00	**4.20**
**mmu-miR-511-5p**	6.01	10.80	0.99	4.10	1.90	1.38	**4.20**
**mmu-miR-125a-3p**	1.72	4.50	4.94	4.10	0.00	9.69	**4.16**
**mmu-miR-299a-3p@**	6.87	0.90	3.95	5.12	3.81	4.15	**4.13**
**mmu-miR-135a-5p**	4.29	10.80	1.98	1.02	3.81	2.77	**4.11**
**mmu-miR-1193-3p**	9.44	2.70	1.98	2.05	2.86	5.54	**4.10**
**mmu-miR-5123**	0.86	0.00	6.92	6.14	1.90	8.31	**4.02**
**mmu-miR-701-5p**	6.01	2.70	3.95	2.05	3.81	5.54	**4.01**
**mmu-miR-1981-3p**	0.86	9.90	2.96	6.14	1.90	1.38	**3.86**
**mmu-miR-6538**	0.00	2.70	7.90	7.17	0.95	4.15	**3.81**
**mmu-miR-337-5p**	3.43	1.80	3.95	2.05	1.90	9.69	**3.80**
**mmu-miR-26a-2-3p**	1.72	2.70	3.95	3.07	5.71	5.54	**3.78**
**mmu-miR-669f-3p**	2.57	7.20	3.95	3.07	2.86	2.77	**3.74**
**mmu-miR-501-5p**	6.01	4.50	0.99	2.05	2.86	5.54	**3.66**
**mmu-miR-450a-2-3p**	4.29	6.30	3.95	1.02	4.76	1.38	**3.62**
**mmu-miR-31-3p**	3.43	1.80	3.95	4.10	2.86	5.54	**3.61**
**mmu-miR-376b-3p**	2.57	0.90	6.92	1.02	1.90	8.31	**3.60**
**mmu-miR-190a-3p**	2.57	0.00	3.95	6.14	1.90	6.92	**3.58**
**mmu-miR-409-3p**	6.87	1.80	6.92	2.05	3.81	0.00	**3.58**
**mmu-miR-181a-2-3p**	0.86	3.60	3.95	6.14	0.95	5.54	**3.51**
**mmu-miR-181b-2-3p**	0.00	2.70	3.95	5.12	0.95	8.31	**3.51**
**mmu-miR-211-5p**	4.29	3.60	1.98	3.07	3.81	4.15	**3.48**
**mmu-miR-18a-3p**	6.01	3.60	3.95	3.07	2.86	1.38	**3.48**
**mmu-miR-341-3p**	7.72	0.90	2.96	3.07	1.90	4.15	**3.45**
**mmu-miR-335-3p**	2.57	3.60	0.99	4.10	0.95	8.31	**3.42**
**mmu-miR-122-3p**	0.00	18.00	1.98	0.00	0.00	0.00	**3.33**
**mmu-miR-149-5p**	2.57	5.40	3.95	1.02	2.86	4.15	**3.33**
**mmu-miR-501-3p**	1.72	3.60	3.95	1.02	3.81	5.54	**3.27**
**mmu-miR-700-3p**	5.15	1.80	2.96	2.05	1.90	5.54	**3.23**
**mmu-miR-330-3p**	0.86	1.80	4.94	3.07	2.86	5.54	**3.18**
**mmu-miR-148b-5p**	5.15	1.80	3.95	4.10	0.95	2.77	**3.12**
**mmu-let-7b-3p**	1.72	4.50	4.94	1.02	0.95	5.54	**3.11**
**mmu-miR-324-3p**	5.15	0.90	3.95	2.05	3.81	2.77	**3.11**
**mmu-let-7k**	0.86	5.40	5.93	2.05	2.86	1.38	**3.08**
**mmu-miR-452-3p**	5.15	4.50	2.96	2.05	3.81	0.00	**3.08**
**mmu-miR-466f-3p**	3.43	10.80	0.00	1.02	2.86	0.00	**3.02**

Biology Open • Supplementary information

**mmu-miR-6238**	0.86	7.20	5.93	3.07	0.95	0.00	**3.00**
**mmu-miR-7033-5p**	0.00	0.00	8.89	4.10	2.86	1.38	**2.87**
**mmu-miR-3058-5p**	1.72	0.00	2.96	2.05	4.76	5.54	**2.84**
**mmu-miR-188-5p**	3.43	2.70	4.94	3.07	2.86	0.00	**2.83**
**mmu-miR-3473e**	0.00	0.90	1.98	7.17	0.95	5.54	**2.76**
**mmu-miR-3071-5p**	4.29	2.70	3.95	2.05	1.90	1.38	**2.71**
**mmu-miR-344b-3p**	9.44	3.60	1.98	1.02	0.00	0.00	**2.67**
**mmu-miR-187-5p**	2.57	0.90	1.98	2.05	0.00	8.31	**2.64**
**mmu-miR-147-5p**	6.01	2.70	2.96	1.02	2.86	0.00	**2.59**
**mmu-miR-369-3p**	2.57	0.90	0.99	2.05	4.76	4.15	**2.57**
**mmu-miR-666-3p**	6.01	2.70	1.98	0.00	1.90	2.77	**2.56**
**mmu-miR-30c-1-3p**	1.72	2.70	2.96	2.05	0.95	4.15	**2.42**
**mmu-miR-193b-5p**	1.72	0.90	1.98	4.10	2.86	2.77	**2.39**
**mmu-miR-466q**	1.72	0.00	0.99	7.17	0.95	2.77	**2.27**
**mmu-miR-3083-5p**	0.86	1.80	2.96	3.07	4.76	0.00	**2.24**
**mmu-miR-298-5p**	2.57	0.00	2.96	3.07	1.90	2.77	**2.21**
**mmu-miR-488-3p**	4.29	2.70	1.98	1.02	1.90	1.38	**2.21**
**mmu-miR-182-3p**	1.72	0.00	5.93	2.05	1.90	1.38	**2.16**
**mmu-miR-3108-5p**	1.72	0.90	3.95	3.07	0.95	1.38	**2.00**
**mmu-miR-329-5p**	4.29	0.00	1.98	0.00	2.86	2.77	**1.98**
**mmu-miR-467d-5p**	2.57	3.60	1.98	1.02	0.95	1.38	**1.92**
**mmu-miR-485-5p**	0.86	0.90	1.98	3.07	1.90	2.77	**1.91**
**mmu-miR-296-3p**	3.43	3.60	1.98	0.00	0.95	1.38	**1.89**
**mmu-miR-137-3p**	0.86	1.80	0.99	0.00	4.76	2.77	**1.86**
**mmu-miR-877-5p**	0.86	1.80	2.96	4.10	0.00	1.38	**1.85**
**mmu-miR-3084-5p**	1.72	0.90	2.96	4.10	0.00	1.38	**1.84**
**mmu-miR-1306-5p**	3.43	4.50	0.99	2.05	0.00	0.00	**1.83**
**mmu-miR-1955-3p**	1.72	1.80	2.96	3.07	0.00	1.38	**1.82**
**mmu-miR-206-3p**	0.00	0.90	0.99	0.00	4.76	4.15	**1.80**
**mmu-miR-101b-5p**	0.00	0.90	4.94	2.05	0.00	2.77	**1.78**
**mmu-miR-5126**	0.86	0.00	2.96	2.05	1.90	2.77	**1.76**
**mmu-let-7e-3p**	2.57	3.60	0.99	1.02	0.95	1.38	**1.75**
**mmu-miR-3066-5p**	0.86	0.90	0.99	2.05	2.86	2.77	**1.74**
**mmu-miR-3544-3p**	1.72	1.80	0.00	1.02	2.86	2.77	**1.70**
**mmu-miR-1249-3p**	2.57	2.70	0.99	0.00	3.81	0.00	**1.68**
**mmu-miR-195b**	0.86	1.80	0.99	3.07	1.90	1.38	**1.67**
**mmu-miR-376a-5p**	6.01	0.00	2.96	1.02	0.00	0.00	**1.67**

Biology Open • Supplementary information

**mmu-miR-191-3p**	1.72	0.90	2.96	2.05	0.95	1.38	**1.66**
**mmu-miR-494-3p**	5.15	0.00	0.99	1.02	0.00	2.77	**1.66**
**mmu-miR-497b**	3.43	3.60	0.99	0.00	1.90	0.00	**1.65**
**mmu-miR-369-5p**	0.86	2.70	2.96	1.02	0.95	1.38	**1.65**
**mmu-miR-874-5p**	1.72	0.90	1.98	1.02	2.86	1.38	**1.64**
**mmu-miR-677-5p**	0.00	0.00	1.98	2.05	0.00	5.54	**1.60**
**mmu-miR-1198-3p**	3.43	0.90	0.99	0.00	2.86	1.38	**1.59**
**mmu-miR-5615-5p**	0.00	2.70	1.98	2.05	0.00	2.77	**1.58**
**mmu-miR-6516-5p**	4.29	0.90	0.99	0.00	1.90	1.38	**1.58**
**mmu-miR-6236**	2.57	1.80	2.96	1.02	0.95	0.00	**1.55**
**mmu-miR-467c-5p**	1.72	2.70	0.99	1.02	2.86	0.00	**1.55**
**mmu-miR-5107-3p**	3.43	1.80	1.98	1.02	0.95	0.00	**1.53**
**mmu-miR-466k**	1.72	0.90	0.00	2.05	2.86	1.38	**1.49**
**mmu-miR-25-5p**	0.86	0.00	0.00	0.00	3.81	4.15	**1.47**
**mmu-miR-378d**	0.86	2.70	0.99	0.00	0.00	4.15	**1.45**
**mmu-miR-1839-3p**	0.00	0.90	2.96	2.05	0.00	2.77	**1.45**
**mmu-miR-615-3p**	2.57	1.80	1.98	0.00	0.95	1.38	**1.45**
**mmu-miR-3065-3p**	3.43	1.80	0.99	1.02	0.00	1.38	**1.44**
**mmu-let-7c-1-3p**	1.72	0.00	3.95	1.02	1.90	0.00	**1.43**
**mmu-miR-3470a**	0.00	0.00	0.99	6.14	0.00	1.38	**1.42**
**mmu-miR-219a-1-3p**	2.57	0.00	0.99	2.05	2.86	0.00	**1.41**
**mmu-miR-3082-5p**	0.86	2.70	0.99	1.02	2.86	0.00	**1.41**
**mmu-miR-26a-1-3p**	1.72	0.90	1.98	1.02	0.00	2.77	**1.40**
**mmu-miR-493-5p**	2.57	0.90	0.00	0.00	1.90	2.77	**1.36**
**mmu-miR-215-5p**	0.00	0.90	0.99	2.05	0.00	4.15	**1.35**
**mmu-miR-101a-5p**	0.00	0.90	0.00	2.05	0.95	4.15	**1.34**
**mmu-miR-16-2-3p**	1.72	0.90	0.00	2.05	1.90	1.38	**1.33**
**mmu-let-7f-2-3p**	0.86	2.70	1.98	1.02	0.00	1.38	**1.32**
**mmu-miR-671-3p**	1.72	0.90	0.99	1.02	1.90	1.38	**1.32**
**mmu-miR-3057-5p**	1.72	1.80	0.99	1.02	0.95	1.38	**1.31**
**mmu-miR-3069-5p**	0.86	3.60	0.00	1.02	0.95	1.38	**1.30**
**mmu-miR-216a-5p**	1.72	2.70	0.99	0.00	0.95	1.38	**1.29**
**mmu-miR-874-3p**	1.72	3.60	0.00	1.02	0.00	1.38	**1.29**
**mmu-miR-99a-3p**	2.57	0.90	1.98	1.02	0.95	0.00	**1.24**
**mmu-miR-1983**	0.00	0.90	0.00	4.10	0.95	1.38	**1.22**
**mmu-miR-135b-5p**	0.00	6.30	0.00	1.02	0.00	0.00	**1.22**
**mmu-miR-668-3p**	2.57	1.80	0.99	0.00	1.90	0.00	**1.21**

Biology Open • Supplementary information

**mmu-miR-3079-5p**	2.57	2.70	0.00	1.02	0.95	0.00	**1.21**
**mmu-miR-1960**	0.86	0.00	2.96	1.02	0.95	1.38	**1.20**
**mmu-miR-196a-2-3p**	3.43	2.70	0.99	0.00	0.00	0.00	**1.19**
**mmu-miR-6996-5p**	0.00	1.80	0.00	1.02	2.86	1.38	**1.18**
**mmu-miR-3470b**	0.86	0.90	2.96	0.00	0.95	1.38	**1.18**
**mmu-miR-1968-5p**	0.00	0.90	0.00	1.02	0.95	4.15	**1.17**
**mmu-miR-652-5p**	1.72	0.00	0.99	1.02	1.90	1.38	**1.17**
**mmu-miR-20b-3p**	2.57	1.80	0.00	1.02	0.00	1.38	**1.13**
**mmu-miR-1941-3p**	0.00	0.90	0.00	2.05	3.81	0.00	**1.13**
**mmu-miR-129b-3p**	0.86	0.90	0.99	1.02	2.86	0.00	**1.11**
**mmu-miR-98-3p**	0.86	0.00	0.99	1.02	0.95	2.77	**1.10**
**mmu-miR-18b-5p**	1.72	0.00	0.00	0.00	1.90	2.77	**1.07**
**mmu-miR-1306-3p**	3.43	0.90	0.00	0.00	1.90	0.00	**1.04**
**mmu-miR-411-3p**	4.29	0.90	0.99	0.00	0.00	0.00	**1.03**
**mmu-miR-3544-5p**	0.00	0.00	0.00	0.00	4.76	1.38	**1.02**
**mmu-miR-5129-3p**	0.00	1.80	0.00	1.02	1.90	1.38	**1.02**
**mmu-miR-136-3p**	0.86	0.90	0.99	1.02	0.95	1.38	**1.02**
**mmu-miR-33-3p**	0.86	1.80	0.99	1.02	0.00	1.38	**1.01**
**mmu-let-7f-1-3p**	0.86	0.90	0.99	0.00	1.90	1.38	**1.01**

Biology Open • Supplementary information

Table S5. miRNA common between 2, 3 or 4 stages of the mammary gland development.

W10|P18	W10|Lac	W10|Inv	P18|Lac	P18|Inv	Lac|Inv
mmu-miR-466h-3p	mmu-miR-193b-3p	mmu-miR-344b-3p	mmu-miR-3105-3p	mmu-miR-122-3p	mmu-miR-7033-5p
mmu-miR-485-3p	mmu-miR-3076-3p	mmu-miR-215-5p	mmu-miR-6946-5p	mmu-miR-1983	mmu-miR-3470b
mmu-miR-466i-3p	mmu-miR-5114	mmu-miR-615-3p	mmu-miR-3076-5p	mmu-miR-3079-5p	mmu-miR-6996-5p
mmu-miR-673-5p	mmu-miR-669p-3p	mmu-miR-874-5p			
mmu-miR-467a-5p@		mmu-miR-1249-3p			
mmu-miR-379-3p		mmu-miR-5107-3p			
mmu-miR-217-5p		mmu-miR-99a-3p			
mmu-miR-744-3p		mmu-miR-136-3p			
mmu-miR-130b-5p		mmu-let-7e-3p			
mmu-miR-5709-5p		mmu-miR-3083-5p			
mmu-miR-540-3p		mmu-miR-677-5p			
mmu-miR-138-1-3p		mmu-miR-411-3p			
mmu-miR-7010-5p		mmu-miR-196a-2-3p			
mmu-miR-380-5p		mmu-let-7f-2-3p			
mmu-miR-212-5p		mmu-miR-129b-3p			
		mmu-miR-98-3p			
		mmu-miR-147-5p			

W10|P18|Lac	W10|P18|Inv			W10|Lac|Inv	P18|Lac|Inv
mmu-miR-466h-5p	mmu-miR-211-5p	mmu-miR-216a-5p	mmu-miR-330-3p	mmu-miR-6516-5p	mmu-miR-146b-5p
mmu-miR-499-5p	mmu-miR-106a-5p	mmu-miR-1941-3p	mmu-miR-666-3p	mmu-miR-378d	mmu-miR-148a-3p
mmu-miR-26b-3p	mmu-miR-125b-1-3p	mmu-miR-3544-5p	mmu-miR-874-3p	mmu-miR-3084-5p	mmu-miR-200c-3p
mmu-miR-376a-3p	mmu-miR-665-3p	mmu-miR-329-5p	mmu-miR-1198-3p		mmu-let-7i-5p
mmu-miR-3968	mmu-miR-700-5p	mmu-miR-296-3p	mmu-miR-652-5p		mmu-let-7e-5p
mmu-miR-5113	mmu-miR-668-3p	mmu-miR-298-5p	mmu-miR-1306-3p		mmu-miR-101b-3p
	mmu-miR-369-3p	mmu-miR-494-3p	mmu-miR-497b		mmu-miR-26b-5p
	mmu-miR-376b-3p	mmu-miR-488-3p	mmu-let-7c-1-3p		mmu-miR-322-5p
	mmu-miR-511-5p	mmu-miR-671-3p	mmu-miR-195b		mmu-miR-342-3p
	mmu-miR-149-5p	mmu-miR-493-5p	mmu-miR-18b-5p		mmu-miR-126b-3p

	mmu-miR-135b-5p	mmu-miR-20b-3p	mmu-miR-26a-1-3p		mmu-miR-196b-5p
	mmu-miR-341-3p	mmu-miR-3065-3p	mmu-miR-187-5p		mmu-miR-29c-3p
	mmu-miR-299a-3p@	mmu-miR-466k			mmu-miR-122-5p
					mmu-miR-200c-5p
					mmu-miR-3473e
					mmu-miR-182-3p
					mmu-miR-6538
					mmu-miR-3470a
					mmu-miR-5615-5p
**W10|Inv|P18|Lac**					
mmu-miR-16-5p	mmu-miR-192-5p	mmu-miR-466c-3p@	mmu-miR-30d-3p	mmu-miR-350-5p	mmu-miR-152-5p
mmu-let-7c-5p	mmu-miR-541-5p	mmu-miR-7a-5p	mmu-miR-322-3p	mmu-miR-190b-5p	mmu-miR-29b-1-5p
mmu-miR-143-3p	mmu-miR-10b-5p	mmu-miR-455-5p	mmu-miR-486a-3p@	mmu-miR-497a-3p	mmu-miR-137-3p
mmu-miR-199a-3p	mmu-miR-182-5p	mmu-miR-30a-3p	mmu-miR-30b-3p	mmu-miR-16-1-3p	mmu-miR-183-3p
mmu-miR-142b	mmu-miR-1843a-5p@	mmu-miR-500-3p	mmu-miR-128-3p	mmu-miR-134-5p	mmu-miR-18a-3p
mmu-let-7a-5p	mmu-miR-3065-5p	mmu-miR-378c	mmu-miR-466d-3p	mmu-miR-669f-3p	mmu-miR-324-3p
mmu-miR-29a-3p	mmu-miR-365-3p	mmu-miR-669c-5p	mmu-miR-542-5p	mmu-miR-3473b	mmu-miR-92a-1-5p
mmu-miR-103-3p	mmu-miR-29b-3p	mmu-miR-379-5p	mmu-miR-676-3p	mmu-miR-30e-3p	mmu-miR-450a-2-3p
mmu-miR-21a-5p	mmu-miR-27a-3p	mmu-miR-1198-5p	mmu-miR-96-5p	mmu-miR-1843b-3p	mmu-miR-330-5p
mmu-miR-142a-5p	mmu-miR-338-3p	mmu-miR-411-5p	mmu-miR-467a-3p@	mmu-miR-664-5p	mmu-miR-452-3p
mmu-miR-125b-5p	mmu-miR-184-3p	mmu-miR-872-3p	mmu-miR-363-3p	mmu-miR-669a-5p	mmu-miR-132-5p
mmu-miR-23a-3p	mmu-miR-152-3p	mmu-miR-100-5p	mmu-miR-382-5p	mmu-miR-200a-5p	mmu-miR-193b-5p
mmu-miR-126a-3p	mmu-miR-339-5p	mmu-miR-324-5p	mmu-miR-150-3p	mmu-miR-340-3p	mmu-miR-148b-5p
mmu-miR-27b-3p	mmu-miR-133b-3p	mmu-miR-203-5p	mmu-miR-450b-5p	mmu-miR-1191a	mmu-miR-188-5p
mmu-miR-125a-5p	mmu-miR-151-5p	mmu-miR-431-5p	mmu-miR-511-3p	mmu-miR-1291	mmu-miR-376a-5p
mmu-miR-146a-5p	mmu-miR-151-3p	mmu-let-7d-3p	mmu-miR-299a-5p@	mmu-miR-301b-3p	mmu-miR-3084-3p
mmu-let-7b-5p	mmu-miR-205-5p	mmu-miR-27a-5p	mmu-miR-18a-5p	mmu-miR-574-5p	mmu-let-7b-3p
mmu-let-7f-5p	mmu-miR-24-3p	mmu-miR-143-5p	mmu-miR-342-5p	mmu-miR-135a-5p	mmu-miR-467d-5p
mmu-miR-1b-5p	mmu-miR-22-3p	mmu-miR-28a-3p	mmu-miR-190a-5p	mmu-miR-598-3p	mmu-let-7k
mmu-miR-101a-3p@	mmu-miR-3964		mmu-miR-423-3p	mmu-miR-130b-3p	mmu-miR-339-3p

Biology Open • Supplementary information

mmu-miR-133a-3p	mmu-miR-141-3p	mmu-miR-138-5p	mmu-miR-30f	mmu-miR-136-5p	mmu-miR-6238
mmu-miR-200a-3p	mmu-miR-32-5p	mmu-miR-22-5p	mmu-miR-335-5p	mmu-miR-34c-3p	mmu-miR-1839-3p
mmu-miR-10a-5p	mmu-miR-125b-2-3p	mmu-miR-300-3p	mmu-miR-106b-3p	mmu-miR-20a-3p	mmu-miR-181b-1-3p
mmu-miR-30a-5p	mmu-miR-301a-3p	mmu-miR-15b-3p	mmu-miR-154-5p	mmu-miR-6240	mmu-miR-335-3p
mmu-miR-26a-5p	mmu-miR-708-5p	mmu-miR-31-5p	mmu-miR-434-3p	mmu-miR-1193-3p	mmu-miR-3064-5p
mmu-miR-25-3p	mmu-miR-181b-5p	mmu-miR-140-5p	mmu-miR-690	mmu-miR-466f-3p	mmu-miR-181b-2-3p
mmu-miR-30d-5p	mmu-miR-30b-5p	mmu-miR-345-3p	mmu-miR-223-5p	mmu-miR-328-3p	mmu-miR-147-3p
mmu-miR-195a-5p	mmu-miR-3068-5p	mmu-miR-127-5p	mmu-miR-9-3p	mmu-miR-701-5p	mmu-miR-3058-5p
mmu-miR-203-3p	mmu-miR-224-5p	mmu-miR-674-3p	mmu-miR-219a-5p	mmu-miR-409-3p	mmu-miR-190a-3p
mmu-miR-196a-5p	mmu-miR-20a-5p	mmu-miR-574-3p	mmu-miR-532-3p	mmu-miR-3071-5p	mmu-miR-5126
mmu-miR-199a-5p	mmu-miR-532-5p	mmu-let-7i-3p	mmu-miR-210-3p	mmu-miR-672-3p	mmu-miR-30c-1-3p
mmu-miR-30e-5p	mmu-miR-126a-5p	mmu-miR-214-5p	mmu-miR-181c-5p	mmu-miR-503-3p	mmu-miR-501-3p
mmu-miR-142a-3p	mmu-miR-669o-3p	mmu-miR-106b-5p	mmu-miR-672-5p	mmu-miR-450a-5p	mmu-miR-3057-5p
mmu-miR-200b-3p	mmu-miR-708-3p	mmu-miR-340-5p	mmu-miR-345-5p	mmu-miR-450a-1-3p	mmu-miR-26a-2-3p
mmu-miR-3074-5p	mmu-miR-542-3p	mmu-miR-652-3p	mmu-miR-331-3p	mmu-miR-1981-5p	mmu-miR-466i-5p
mmu-miR-15b-5p	mmu-miR-17-3p	mmu-miR-193a-5p	mmu-miR-378b	mmu-miR-3068-3p	mmu-miR-3066-5p
mmu-miR-93-5p	mmu-miR-133a-5p	mmu-miR-144-3p	mmu-miR-34b-5p	mmu-miR-181c-3p	mmu-miR-33-3p
mmu-miR-23b-3p	mmu-miR-34c-5p	mmu-let-7a-1-3p	mmu-miR-374c-3p	mmu-miR-376c-3p	mmu-miR-1968-5p
mmu-miR-140-3p	mmu-miR-145a-3p	mmu-miR-20b-5p	mmu-miR-34b-3p	mmu-miR-374b-5p@	mmu-miR-2137
mmu-let-7g-5p	mmu-miR-10b-3p	mmu-miR-17-5p	mmu-miR-93-3p	mmu-miR-1306-5p	mmu-miR-205-3p
mmu-miR-221-3p	mmu-miR-429-3p	mmu-miR-1843a-3p	mmu-miR-582-3p	mmu-miR-5121	mmu-miR-3108-5p
mmu-miR-223-3p	mmu-miR-10a-3p	mmu-miR-29a-5p	mmu-miR-744-5p	mmu-miR-15a-3p	mmu-miR-146b-3p
mmu-miR-19b-3p	mmu-miR-350-3p	mmu-miR-186-5p	mmu-miR-375-3p	mmu-miR-421-3p	mmu-miR-6236
mmu-let-7d-5p	mmu-miR-434-5p	mmu-miR-376b-5p	mmu-miR-222-3p	mmu-miR-16-2-3p	mmu-miR-466q
mmu-miR-497a-5p	mmu-miR-183-5p	mmu-miR-218-5p	mmu-miR-30c-2-3p	mmu-miR-187-3p	mmu-miR-148a-5p
mmu-miR-107-3p	mmu-miR-674-5p	mmu-miR-148b-3p	mmu-miR-423-5p	mmu-miR-181a-2-3p	mmu-miR-877-5p
mmu-miR-99a-5p	mmu-miR-185-5p	mmu-miR-671-5p	mmu-miR-7a-1-3p	mmu-miR-467c-5p	mmu-let-7f-1-3p
mmu-miR-30c-5p	mmu-miR-130a-3p	mmu-miR-139-5p	mmu-miR-33-5p	mmu-let-7g-3p	mmu-miR-191-3p
mmu-miR-378a-3p	mmu-miR-155-5p	mmu-miR-195a-3p	mmu-miR-186-3p	mmu-miR-124-3p	mmu-miR-1955-3p
mmu-miR-191-5p	mmu-miR-99b-5p	mmu-miR-29c-5p	mmu-miR-409-5p	mmu-miR-485-5p	mmu-miR-101b-5p
mmu-miR-1a-3p	mmu-miR-141-5p	mmu-miR-206-3p	mmu-miR-5099	mmu-miR-29b-2-5p	mmu-miR-582-5p

mmu-miR-145a-5p	mmu-miR-3535	mmu-miR-664-3p	mmu-miR-139-3p	mmu-miR-221-5p	mmu-miR-219a-1-3p
mmu-miR-214-3p	mmu-miR-19a-3p	mmu-let-7j	mmu-miR-491-5p	mmu-miR-3473a	mmu-miR-3069-5p
mmu-miR-320-3p	mmu-miR-34a-5p	mmu-miR-351-5p	mmu-miR-28a-5p@	mmu-miR-337-5p	mmu-miR-31-3p
mmu-miR-150-5p	mmu-miR-361-5p	mmu-miR-486a-5p	mmu-miR-467e-5p	mmu-miR-34a-3p	mmu-miR-101a-5p
mmu-miR-872-5p	mmu-miR-484	mmu-miR-200b-5p	mmu-miR-297c-3p	mmu-miR-99b-3p	mmu-miR-25-5p
mmu-miR-425-5p	mmu-miR-362-3p	mmu-miR-378a-5p	mmu-miR-181a-1-3p	mmu-miR-1981-3p	mmu-miR-5123
mmu-miR-181a-5p	mmu-miR-204-5p	mmu-miR-127-3p	mmu-miR-132-3p	mmu-miR-501-5p	mmu-miR-3105-5p
mmu-miR-451a	mmu-miR-9-5p	mmu-miR-503-5p	mmu-miR-326-3p	mmu-miR-3544-3p	mmu-miR-1839-5p
mmu-miR-92a-3p	mmu-miR-3071-3p	mmu-miR-676-5p	mmu-miR-455-3p	mmu-miR-369-5p	mmu-miR-98-5p
mmu-miR-199b-5p	mmu-miR-381-3p	mmu-miR-144-5p	mmu-miR-21a-3p	mmu-miR-700-3p	
mmu-miR-15a-5p	mmu-miR-362-5p	mmu-miR-450b-3p	mmu-miR-181d-5p	mmu-miR-27b-5p	
mmu-miR-194-5p	mmu-miR-126b-5p	mmu-miR-425-3p	mmu-miR-361-3p	mmu-miR-125a-3p	

Table S6. miRNA identified in each cluster of expression.

Cluster 1: 93 miRNA					
mmu-miR-27b-3p	mmu-miR-141-5p	mmu-let-7a-5p	mmu-miR-30a-3p	mmu-miR-3473b	mmu-miR-6538
mmu-miR-429-3p	mmu-let-7i-5p	mmu-miR-1191a	mmu-miR-26b-5p	mmu-miR-146b-3p	mmu-miR-125a-3p
mmu-miR-200b-3p	mmu-miR-190a-5p	mmu-miR-3074-5p	mmu-miR-148b-3p	mmu-miR-674-5p	mmu-miR-193a-5p
mmu-miR-141-3p	mmu-miR-3105-5p	mmu-miR-183-5p	mmu-miR-98-5p	mmu-miR-466i-5p	mmu-miR-339-3p
mmu-miR-182-5p	mmu-miR-144-3p	mmu-miR-151-5p	mmu-miR-7a-1-3p	mmu-miR-200c-5p	mmu-miR-582-5p
mmu-miR-146b-5p	mmu-miR-30b-3p	mmu-miR-345-3p	mmu-miR-32-5p	mmu-miR-28a-5p-	mmu-miR-877-5p
mmu-miR-210-3p	mmu-miR-375-3p	mmu-let-7e-5p	mmu-miR-5123	mmu-miR-3473a	mmu-miR-30e-3p
mmu-miR-200a-5p	mmu-miR-24-3p	mmu-miR-185-5p	mmu-miR-27b-5p	mmu-miR-30d-3p	mmu-miR-574-5p
mmu-miR-103-3p	mmu-miR-181b-5p	mmu-miR-200b-5p	mmu-miR-1968-5p	mmu-miR-672-3p	mmu-miR-3473e
mmu-miR-148a-3p	mmu-miR-221-3p	mmu-miR-126a-3p	mmu-let-7j	mmu-miR-3064-5p	mmu-miR-5113
mmu-miR-181a-1-3p	mmu-miR-101b-3p	mmu-miR-152-3p	mmu-miR-10b-3p	mmu-miR-450b-3p	mmu-miR-31-3p
mmu-miR-96-5p	mmu-let-7d-5p	mmu-miR-340-5p	mmu-miR-192-5p	mmu-let-7b-5p	mmu-miR-3470b
mmu-miR-7a-5p	mmu-miR-183-3p	mmu-miR-29b-1-5p	mmu-let-7g-5p	mmu-miR-34c-3p	mmu-miR-423-5p
mmu-let-7f-5p	mmu-miR-148a-5p	mmu-miR-30c-5p	mmu-miR-30c-2-3p	mmu-miR-484	
mmu-miR-672-5p	mmu-miR-340-3p	mmu-miR-7116-5p	mmu-miR-181a-5p	mmu-miR-23b-3p	
mmu-miR-200a-3p	mmu-miR-200c-3p	mmu-miR-28a-3p	mmu-miR-181b-2-3p	mmu-miR-421-3p	
**Cluster 2: 44 miRNA**					
mmu-miR-143-3p	mmu-miR-33-5p	mmu-miR-222-3p	mmu-miR-148b-5p	mmu-miR-190b-5p	mmu-miR-182-3p
mmu-miR-3068-5p	mmu-miR-1843a-5p-	mmu-miR-26a-5p	mmu-miR-140-5p	mmu-miR-664-5p	mmu-miR-99b-3p
mmu-miR-1839-5p	mmu-miR-30e-5p	mmu-miR-500-3p	mmu-miR-27a-3p	mmu-miR-16-1-3p	mmu-miR-147-5p
mmu-miR-107-3p	mmu-miR-101a-3p-	mmu-let-7i-3p	mmu-miR-466q	mmu-miR-3068-3p	mmu-miR-452-3p
mmu-miR-378b	mmu-miR-29b-3p	mmu-miR-345-5p	mmu-miR-193b-5p	mmu-miR-1291	
mmu-miR-30a-5p	mmu-miR-34a-5p	mmu-miR-126a-5p	mmu-miR-181a-2-3p	mmu-let-7g-3p	
mmu-miR-378c	mmu-miR-676-5p	mmu-miR-126b-3p	mmu-miR-30b-5p	mmu-miR-7033-5p	
mmu-miR-676-3p	mmu-miR-145a-5p	mmu-miR-126b-5p	mmu-miR-221-5p	mmu-miR-374b-5p-	
**Cluster 3: 135 miRNA**					
mmu-miR-184-3p	mmu-miR-125b-5p	mmu-miR-503-5p	mmu-miR-106a-5p	mmu-miR-409-3p	mmu-miR-301b-3p

mmu-miR-93-5p	mmu-miR-486a-3p-	mmu-miR-511-3p	mmu-miR-350-5p	mmu-miR-324-5p	mmu-miR-493-5p
mmu-miR-204-5p	mmu-let-7d-3p	mmu-miR-381-3p	mmu-miR-322-3p	mmu-miR-671-5p	mmu-miR-497a-3p
mmu-miR-23a-3p	mmu-miR-150-3p	mmu-miR-379-5p	mmu-miR-15b-3p	mmu-miR-491-5p	mmu-miR-671-3p
mmu-miR-338-3p	mmu-miR-199a-5p	mmu-miR-434-3p	mmu-miR-668-3p	mmu-miR-1193-3p	mmu-miR-669a-5p
mmu-miR-431-5p	mmu-miR-20b-5p	mmu-miR-466c-3p-	mmu-miR-154-5p	mmu-miR-181c-3p	mmu-miR-134-5p
mmu-miR-365-3p	mmu-miR-211-5p	mmu-miR-669c-5p	mmu-miR-872-3p	mmu-miR-149-5p	mmu-miR-511-5p
mmu-miR-350-3p	mmu-miR-125b-2-3p	mmu-miR-466d-3p	mmu-miR-93-3p	mmu-miR-329-5p	mmu-miR-15a-5p
mmu-miR-17-3p	mmu-miR-541-5p	mmu-miR-142b	mmu-miR-135a-5p	mmu-miR-139-3p	mmu-miR-494-3p
mmu-miR-99a-5p	mmu-miR-100-5p	mmu-miR-214-5p	mmu-miR-455-5p	mmu-miR-700-5p	mmu-miR-466i-3p
mmu-miR-199a-3p	mmu-miR-298-5p	mmu-miR-130a-3p	mmu-miR-195a-3p	mmu-miR-369-3p	mmu-miR-376b-3p
mmu-miR-3964	mmu-miR-127-3p	mmu-miR-142a-3p	mmu-miR-125b-1-3p	mmu-miR-341-3p	mmu-miR-3544-3p
mmu-miR-342-3p	mmu-miR-196a-5p	mmu-miR-409-5p	mmu-miR-872-5p	mmu-miR-615-3p	mmu-miR-701-5p
mmu-miR-299a-5p-	mmu-miR-322-5p	mmu-miR-218-5p	mmu-miR-382-5p	mmu-miR-135b-5p	mmu-miR-3544-5p
mmu-miR-411-5p	mmu-miR-467a-3p-	mmu-miR-3071-3p	mmu-miR-331-3p	mmu-miR-136-5p	mmu-miR-874-5p
mmu-miR-708-3p	mmu-miR-155-5p	mmu-miR-665-3p	mmu-miR-219a-5p	mmu-miR-19a-3p	mmu-miR-299a-3p-
mmu-miR-199b-5p	mmu-miR-363-3p	mmu-miR-674-3p	mmu-miR-342-5p	mmu-miR-466h-3p	mmu-miR-744-5p
mmu-miR-434-5p	mmu-miR-205-5p	mmu-miR-142a-5p	mmu-miR-326-3p	mmu-miR-3071-5p	
mmu-miR-127-5p	mmu-miR-497a-5p	mmu-miR-376b-5p	mmu-miR-195a-5p	mmu-miR-106b-3p	
mmu-miR-92a-3p	mmu-miR-214-3p	mmu-miR-296-3p	mmu-miR-19b-3p	mmu-miR-376c-3p	
mmu-miR-196b-5p	mmu-miR-335-3p	mmu-miR-139-5p	mmu-miR-425-3p	mmu-miR-337-5p	
mmu-miR-300-3p	mmu-miR-351-5p	mmu-miR-1843a-3p	mmu-miR-16-5p	mmu-miR-485-3p	
mmu-miR-150-5p	mmu-miR-423-3p	mmu-miR-455-3p	mmu-miR-652-3p	mmu-miR-191-5p	
		mmu-miR-574-3p	mmu-miR-216a-5p	mmu-miR-1941-3p	
**Cluster 4: 45 miRNA**					
mmu-miR-339-5p	mmu-miR-30d-5p	mmu-miR-5121	mmu-miR-34a-3p	mmu-miR-582-3p	mmu-miR-186-5p
mmu-miR-140-3p	mmu-miR-664-3p	mmu-miR-22-3p	mmu-miR-3535	mmu-miR-130b-3p	mmu-miR-137-3p
mmu-miR-29a-3p	mmu-miR-297c-3p	mmu-miR-29a-5p	mmu-miR-362-3p	mmu-miR-29c-5p	mmu-miR-344b-3p
mmu-miR-21a-5p	mmu-miR-223-5p	mmu-miR-22-5p	mmu-let-7a-1-3p	mmu-miR-3083-5p	mmu-miR-186-3p
mmu-miR-146a-5p	mmu-miR-362-5p	mmu-miR-132-3p	mmu-miR-10a-5p	mmu-miR-425-5p	mmu-miR-361-3p
mmu-miR-224-5p	mmu-miR-486a-5p	mmu-miR-125a-5p	mmu-miR-10b-5p	mmu-miR-328-3p	

Biology Open • Supplementary information

mmu-miR-21a-3p	mmu-miR-99b-5p	mmu-miR-10a-3p	mmu-miR-598-3p	mmu-miR-29b-2-5p	
mmu-miR-223-3p	mmu-miR-669f-3p	mmu-miR-532-3p	mmu-miR-330-3p	mmu-miR-215-5p	
**Cluster 5: 27 miRNA**					
mmu-miR-25-3p	mmu-miR-20a-5p	mmu-miR-106b-5p	mmu-miR-3105-3p	mmu-miR-15a-3p	mmu-miR-92a-1-5p
mmu-miR-138-5p	mmu-let-7c-5p	mmu-miR-181b-1-3p	mmu-miR-34c-5p	mmu-miR-27a-5p	mmu-miR-1983
mmu-miR-335-5p	mmu-miR-143-5p	mmu-miR-17-5p	mmu-miR-145a-3p	mmu-miR-690	
mmu-miR-542-3p	mmu-miR-31-5p	mmu-miR-301a-3p	mmu-miR-205-3p	mmu-miR-361-5p	
mmu-miR-18a-5p	mmu-miR-187-3p	mmu-miR-203-3p	mmu-miR-122-5p	mmu-miR-450b-5p	
**Cluster 6: 30 miRNA**					
mmu-miR-9-5p	mmu-miR-133a-3p	mmu-miR-133b-3p	mmu-miR-467e-5p	mmu-miR-485-5p	mmu-miR-384-3p
mmu-miR-378a-3p	mmu-miR-708-5p	mmu-miR-181d-5p	mmu-miR-1a-1-5p	mmu-miR-193b-3p	mmu-miR-669a-3-3p
mmu-miR-29c-3p	mmu-miR-15b-5p	mmu-miR-133a-5p	mmu-miR-206-3p	mmu-miR-16-2-3p	mmu-miR-466m-5p-
mmu-miR-203-5p	mmu-miR-669o-3p	mmu-miR-1a-3p	mmu-miR-181c-5p	mmu-miR-451a	mmu-miR-653-5p
mmu-miR-3065-5p	mmu-miR-378a-5p	mmu-miR-1b-5p	mmu-miR-9-3p	mmu-miR-1306-5p	mmu-miR-379-3p

Table S7. miRNA differentially expressed between adult (W10) and pregnant (P18) mice.

miRNA	baseMean	log2FoldChange	lfcSE	stat	pvalue	[illegible]
**Up-regulated**						
**mmu-miR-1a-1-5p**	1.358764567	4.794090713	1.287588876	21.910641	6.81E-05	[illegible]
**mmu-miR-133b-3p**	112.5629847	3.886625976	0.957246755	27.51563631	4.59E-06	[illegible]
**mmu-miR-653-5p**	0.498672012	3.600514932	1.862173294	8.556855278	0.035801574	[illegible]
**mmu-miR-133a-3p**	2368.674849	3.418111542	0.769780876	37.89554622	2.97E-08	[illegible]
**mmu-miR-184-3p**	122.6865433	3.157912061	0.280313876	265.9586807	2.31E-57	[illegible]
**mmu-miR-1a-3p**	404.1350897	2.755270678	0.847401622	23.9040173	2.62E-05	[illegible]
**mmu-miR-1b-5p**	3426.674947	2.72615686	0.866109663	22.47396601	5.20E-05	[illegible]
**mmu-miR-215-5p**	1.393093243	2.68736291	1.036154905	9.100685617	0.02798174	[illegible]
**mmu-miR-466m-5p@**	0.934441196	2.668990364	1.048288074	9.211915639	0.026602087	[illegible]
**mmu-miR-133a-5p**	89.82058384	2.605949005	0.882493513	23.98291669	2.52E-05	[illegible]
**mmu-miR-384-3p**	1.027180287	2.574986736	1.151199966	9.74192431	0.0208922	[illegible]
**mmu-miR-467a-3p@**	12.31254233	2.551904543	0.470329136	60.04724051	5.74E-13	[illegible]
**mmu-miR-297c-3p**	6.370833167	2.511196356	0.475542154	47.10516505	3.30E-10	[illegible]
**mmu-miR-338-3p**	174.5856256	2.331226775	0.250800752	136.3948523	2.26E-29	[illegible]
**mmu-let-7d-3p**	47.16435266	2.242518106	0.319644409	83.61669533	5.14E-18	[illegible]
**mmu-miR-181a-2-3p**	3.726180378	2.211549525	0.655629825	18.51713506	0.000344015	[illegible]
**mmu-miR-193b-3p**	1.732913591	2.153952334	0.864087455	13.62903891	0.003456151	[illegible]
**mmu-miR-99a-5p**	421.4757133	2.103531356	0.303230249	108.1145526	2.79E-23	[illegible]
**mmu-miR-211-5p**	6.693361953	2.088396351	0.4128718	77.16712744	1.24E-16	[illegible]
**mmu-miR-669a-3-3p**	1.008247146	2.021842462	0.954856179	9.229838774	0.026386103	[illegible]
**mmu-miR-344b-3p**	1.748094698	1.999519312	0.939555134	8.01806885	0.045639749	[illegible]
**mmu-miR-21a-3p**	8.218180028	1.959648256	0.391707073	66.29601476	2.65E-14	[illegible]
**mmu-miR-3083-5p**	0.940606947	1.958269859	1.179194428	10.95054786	0.01199631	[illegible]
**mmu-miR-135a-5p**	4.062312633	1.932413044	0.527543875	24.59528059	1.88E-05	[illegible]
**mmu-miR-669f-3p**	5.362550339	1.903178546	0.441221841	32.84890588	3.47E-07	[illegible]
**mmu-miR-615-3p**	1.208631326	1.894543708	0.847154433	14.71567934	0.002076469	[illegible]
**mmu-miR-363-3p**	14.43168287	1.838703015	0.41861996	58.24859941	1.39E-12	[illegible]
**mmu-miR-137-3p**	2.01145281	1.838488196	0.738799773	8.157609233	0.042864262	[illegible]
**mmu-miR-199b-5p**	347.8513637	1.831305682	0.254919469	98.068008	4.04E-21	[illegible]
**mmu-miR-486a-5p**	26.69966123	1.826827623	0.30526773	42.62338793	2.96E-09	[illegible]
**mmu-miR-664-3p**	25.65702241	1.823227429	0.281434211	53.26341865	1.61E-11	[illegible]

Biology Open • Supplementary information

**mmu-miR-486a-3p@**	22.16840226	1.798403019	0.256630735	83.75700858	4.80E-18	[illegible]
**mmu-miR-350-3p**	99.13806399	1.755385023	0.25593424	116.9704976	3.47E-25	[illegible]
**mmu-miR-29a-3p**	25777.64838	1.755103171	0.159216837	142.3109259	1.20E-30	[illegible]
**mmu-miR-511-3p**	15.66389615	1.743780849	0.357806654	51.97954009	3.03E-11	[illegible]
**mmu-miR-3964**	142.0915454	1.666935276	0.312965732	103.0776678	3.39E-22	[illegible]
**mmu-miR-598-3p**	4.964432544	1.663052237	0.539443112	13.96107022	0.002958625	[illegible]
**mmu-miR-365-3p**	202.4822814	1.661493918	0.213288071	130.7174844	3.79E-28	[illegible]
**mmu-miR-466h-3p**	1.56793887	1.643809251	0.736823155	13.94654374	0.002978826	[illegible]
**mmu-miR-874-5p**	1.200022433	1.63179847	0.899012406	8.273798483	0.040679389	[illegible]
**mmu-miR-150-5p**	330.436374	1.622215018	0.385790882	84.92170681	2.70E-18	[illegible]
**mmu-miR-466i-3p**	1.377920705	1.586385743	0.854523616	9.657551464	0.021713018	[illegible]
**mmu-miR-374b-5p@**	4.430587298	1.57294479	0.601605743	9.536047798	0.022950948	[illegible]
**mmu-miR-452-3p**	2.777995905	1.546956521	0.660810031	7.337653766	0.061879878	[illegible]
**mmu-miR-1941-3p**	1.366980064	1.546235726	0.800776517	11.17500682	0.010816227	[illegible]
**mmu-miR-100-5p**	47.36730637	1.517208864	0.296127409	67.00662106	1.87E-14	[illegible]
**mmu-miR-708-3p**	107.4330313	1.513389676	0.222703463	100.1438342	1.45E-21	[illegible]
**mmu-miR-3065-5p**	244.6251794	1.456432063	0.246638459	49.78507333	8.88E-11	[illegible]
**mmu-miR-29c-3p**	1543.69309	1.427139381	0.179348089	65.95877196	3.13E-14	[illegible]
**mmu-miR-378c**	75.34400739	1.425002566	0.17271063	101.3258314	8.06E-22	[illegible]
**mmu-miR-485-3p**	1.296062063	1.420843141	0.872069988	11.60929352	0.008848739	[illegible]
**mmu-miR-328-3p**	4.365593314	1.411520694	0.619199419	10.05444405	0.018108965	[illegible]
**mmu-miR-676-5p**	38.35286563	1.410248336	0.222456958	59.88512628	6.22E-13	[illegible]
**mmu-miR-455-3p**	7.668250687	1.383001052	0.409509069	30.39922067	1.14E-06	[illegible]
**mmu-miR-409-5p**	8.195583015	1.351269322	0.381165326	39.22530328	1.56E-08	[illegible]
**mmu-miR-20b-5p**	30.21191906	1.346531262	0.295198277	78.83356523	5.46E-17	[illegible]
**mmu-miR-466d-3p**	18.95782071	1.335386448	0.286605894	45.85170611	6.10E-10	[illegible]
**mmu-miR-362-3p**	86.49870329	1.320190447	0.314148481	17.91799096	0.000457316	[illegible]
**mmu-miR-150-3p**	13.72767638	1.306307983	0.293185178	82.30478517	9.83E-18	[illegible]
**mmu-miR-362-5p**	72.55219448	1.281622929	0.217865565	43.35230577	2.07E-09	[illegible]
**mmu-miR-669o-3p**	128.4788827	1.243253882	0.286231757	30.52344653	1.07E-06	[illegible]
**mmu-miR-29b-2-5p**	4.247138664	1.231012665	0.559974015	9.783679066	0.02049734	[illegible]
**mmu-miR-423-3p**	17.57200617	1.230028312	0.274040013	52.74453924	2.08E-11	[illegible]
**mmu-miR-9-5p**	67.06992983	1.20669258	0.173518683	80.45079778	2.46E-17	[illegible]
**mmu-miR-29a-5p**	39.13929738	1.180589987	0.254528605	30.21740247	1.24E-06	[illegible]

Biology Open • Supplementary information

**mmu-miR-671-3p**	1.164669384	1.156620965	0.805318809	10.30910594	0.016113362	[illegible]
**mmu-miR-206-3p**	24.99298637	1.14911114	0.86612108	21.15501273	9.78E-05	[illegible]
**mmu-miR-142a-3p**	2016.056019	1.138381154	0.316477323	40.36847953	8.90E-09	[illegible]
**mmu-miR-142b**	31041.08194	1.123698837	0.3137905	43.38452519	2.04E-09	[illegible]
**mmu-miR-139-5p**	32.35458436	1.11528643	0.249696765	32.11912903	4.94E-07	[illegible]
**mmu-miR-219a-5p**	14.26926358	1.112975164	0.385001901	21.61667646	7.84E-05	[illegible]
**mmu-miR-106a-5p**	4.724260817	1.102326046	0.478342886	30.18749125	1.26E-06	[illegible]
**mmu-miR-532-3p**	17.36888037	1.100988287	0.265718395	20.84373858	0.000113443	[illegible]
**mmu-miR-466c-3p@**	59.32097993	1.094392881	0.268237293	47.43430475	2.81E-10	[illegible]
**mmu-miR-1306-5p**	2.968437413	1.092300304	0.524709936	10.51870393	0.014634553	[illegible]
**mmu-miR-3068-3p**	5.844458955	1.073719113	0.482282435	13.78719642	0.003209601	[illegible]
**mmu-miR-21a-5p**	19034.2627	1.06462141	0.151716526	132.3706964	1.67E-28	[illegible]
**mmu-miR-30d-5p**	3392.881304	1.056278548	0.14076363	56.07081272	4.06E-12	[illegible]
**mmu-miR-378a-3p**	673.8714094	1.054821076	0.159712487	71.01251692	2.59E-15	[illegible]
**mmu-miR-339-5p**	290.5954939	1.051357953	0.127747456	172.3237964	4.01E-37	[illegible]
**mmu-miR-369-3p**	2.382490077	1.009402287	0.630940286	15.17650078	0.001671863	[illegible]
**mmu-miR-125a-5p**	7039.732718	1.004851077	0.220044339	24.39534965	2.07E-05	[illegible]
**mmu-miR-22-3p**	242.4145584	1.000537549	0.194818749	30.36202696	1.16E-06	[illegible]
**Down-regulated**						
**mmu-miR-181a-1-3p**	53.42133543	-1.009365075	0.26073867	216.3452141	1.24E-46	[illegible]
**mmu-miR-30c-2-3p**	30.6319151	-1.029125276	0.244238194	39.9450301	1.09E-08	[illegible]
**mmu-miR-15a-3p**	7.705565683	-1.03576001	0.33329274	14.00615076	0.002896792	[illegible]
**mmu-miR-7116-5p**	2.724939119	-1.049943863	1.098221369	71.44037146	2.10E-15	[illegible]
**mmu-miR-30b-3p**	70.94130362	-1.129683291	0.169917681	141.8776689	1.49E-30	[illegible]
**mmu-miR-98-5p**	69.93813827	-1.134665649	0.179915315	64.1048034	7.80E-14	[illegible]
**mmu-miR-92a-1-5p**	4.926846957	-1.151495821	0.405906799	9.519877813	0.023120831	[illegible]
**mmu-miR-181c-3p**	6.872165404	-1.177129886	0.389710782	16.67052598	0.000826015	[illegible]
**mmu-let-7i-5p**	4294.865446	-1.183280764	0.163686188	158.1057024	4.70E-34	[illegible]
**mmu-miR-106b-5p**	112.2268627	-1.195751572	0.186665848	59.65925232	6.95E-13	[illegible]
**mmu-miR-1968-5p**	4.450988056	-1.198276325	0.540254123	53.90306352	1.18E-11	[illegible]
**mmu-miR-143-5p**	180.7936393	-1.21322521	0.174304326	78.25360244	7.27E-17	[illegible]
**mmu-miR-146b-3p**	5.559283647	-1.221117811	0.590913598	38.18609841	2.58E-08	[illegible]
**mmu-miR-27b-3p**	24251.91576	-1.228981631	0.100160979	372.1928102	2.33E-80	[illegible]
**mmu-miR-3470b**	0.862074046	-1.275205535	1.345036012	8.792230749	0.03218472	[illegible]

Biology Open • Supplementary information

**mmu-miR-301a-3p**	417.8257098	-1.281022991	0.185625484	56.19021055	3.83E-12	[illegible]
**mmu-miR-138-5p**	116.0548328	-1.313618938	0.146182524	148.4969584	5.56E-32	[illegible]
**mmu-miR-3473a**	12.2329707	-1.330205391	0.412486961	26.39516988	7.88E-06	[illegible]
**mmu-miR-20a-5p**	377.170498	-1.391858206	0.150221821	99.605252	1.89E-21	[illegible]
**mmu-miR-200c-3p**	5340.032311	-1.440199354	0.20349254	111.1798018	6.12E-24	[illegible]
**mmu-miR-340-3p**	27.95687978	-1.49299695	0.258025935	113.3865196	2.05E-24	[illegible]
**mmu-miR-31-3p**	2.818438828	-1.525878232	0.672604743	9.235210909	0.026321701	[illegible]
**mmu-miR-31-5p**	188.5229683	-1.632443088	0.202169551	77.99246187	8.27E-17	[illegible]
**mmu-miR-204-5p**	142.8778395	-1.635484944	0.247679495	188.0616637	1.60E-40	[illegible]
**mmu-miR-3473e**	1.875782239	-1.658055286	1.024511001	9.518313147	0.023137335	[illegible]
**mmu-miR-582-5p**	3.815607751	-1.662881642	0.62485803	13.51275444	0.003649306	[illegible]
**mmu-miR-182-5p**	2833.429736	-1.761993768	0.214493612	289.9560869	1.48E-62	[illegible]
**mmu-miR-296-3p**	3.425533797	-1.825962855	0.494190568	32.88449935	3.41E-07	[illegible]
**mmu-miR-181b-5p**	740.6846825	-1.838446292	0.261983415	132.0584641	1.95E-28	[illegible]
**mmu-miR-466i-5p**	6.027872382	-1.846727767	0.488413439	29.62765525	1.65E-06	[illegible]
**mmu-miR-27b-5p**	13.33628312	-1.861315122	0.357558716	54.08958144	1.07E-11	[illegible]
**mmu-miR-200b-3p**	14790.12574	-1.873663061	0.179958896	315.8843014	3.63E-68	[illegible]
**mmu-miR-429-3p**	951.1698198	-1.880028006	0.18775555	333.7978101	4.81E-72	[illegible]
**mmu-miR-18a-5p**	63.43149218	-1.922783769	0.210003822	100.639488	1.13E-21	[illegible]
**mmu-miR-190a-5p**	116.293464	-1.951403707	0.278149728	152.9896728	5.97E-33	[illegible]
**mmu-miR-6538**	2.465253541	-2.016393336	1.107762341	16.79837945	0.000777525	[illegible]
**mmu-miR-205-3p**	4.03799113	-2.063245453	0.525681092	18.45165155	0.000354895	[illegible]
**mmu-miR-200b-5p**	182.5956871	-2.082202512	0.279643935	87.79360625	6.52E-19	[illegible]
**mmu-miR-335-5p**	67.0422257	-2.102794819	0.230627974	108.2658615	2.59E-23	[illegible]
**mmu-miR-141-3p**	2366.759146	-2.1583812	0.259559441	308.4345092	1.49E-66	[illegible]
**mmu-miR-183-5p**	720.7181715	-2.219183044	0.266436785	93.00896644	4.94E-20	[illegible]
**mmu-miR-200a-3p**	35951.03325	-2.254674067	0.243289941	193.7261763	9.56E-42	[illegible]
**mmu-miR-187-3p**	18.60817487	-2.413075582	0.313356138	76.0498347	2.16E-16	[illegible]
**mmu-miR-96-5p**	223.6361993	-2.415981428	0.259585511	205.6995888	2.47E-44	[illegible]
**mmu-miR-335-3p**	5.673791222	-2.443732197	0.442743147	54.97879249	6.94E-12	[illegible]
**mmu-miR-182-3p**	1.431453785	-2.508282922	1.121110667	9.222229305	0.026477591	[illegible]
**mmu-miR-298-5p**	4.789563109	-2.532942094	0.482630258	65.07572677	4.83E-14	[illegible]
**mmu-miR-181b-1-3p**	11.2446293	-2.548586307	0.407173904	56.37344252	3.50E-12	[illegible]
**mmu-miR-141-5p**	1035.269983	-2.579055641	0.274830581	175.4863061	8.32E-38	[illegible]

Biology Open • Supplementary information

**mmu-miR-183-3p**	19.83412676	-2.775823286	0.344112498	124.9091537	6.76E-27	[illegible]
**mmu-miR-5123**	6.536994105	-2.872546439	0.64670067	54.42264323	9.12E-12	[illegible]
**mmu-miR-1983**	0.686234063	-2.969930911	1.294362792	7.527185025	0.056864051	[illegible]
**mmu-miR-200c-5p**	4.686753262	-3.038550141	0.721568269	29.30224192	1.93E-06	[illegible]
**mmu-miR-200a-5p**	87.35168412	-3.160398873	0.285801677	242.9168492	2.23E-52	[illegible]
**mmu-miR-148a-3p**	19463.75931	-3.422145772	0.294206076	229.8395854	1.50E-49	[illegible]
**mmu-miR-210-3p**	285.0656017	-3.51570342	0.325077485	255.9948338	3.30E-55	[illegible]
**mmu-miR-148a-5p**	15.72949366	-3.965626241	0.526042522	116.5971667	4.17E-25	[illegible]
**mmu-miR-146b-5p**	24587.01652	-3.972043668	0.257093555	279.6402696	2.53E-60	[illegible]
**mmu-miR-3105-5p**	29.8045294	-4.229735917	0.671263133	151.5600771	1.21E-32	[illegible]
**mmu-miR-3105-3p**	2.723923826	-4.475036078	0.922146686	44.02328127	1.49E-09	[illegible]
**mmu-miR-122-5p**	110.62168	-8.901588898	1.967166378	14.95475376	0.001855726	[illegible]

Table S8. miRNA differentially expressed between pregnant (P18) and lactating (Lac) mice.

miRNA	baseMean	log2FoldChange	lfcSE	stat	pvalue	[illegible]
**Up-regulated**						
**mmu-miR-122-5p**	110.62168	4.639549553	1.867012523	14.95475376	0.001855726	[illegible]
**mmu-miR-665-3p**	5.308828161	4.383402469	0.888976354	37.45891604	3.68E-08	[illegible]
**mmu-miR-298-5p**	4.789563109	4.190607218	0.895370822	65.07572677	4.83E-14	[illegible]
**mmu-miR-329-5p**	2.020057522	3.724747241	0.978919367	16.42227064	0.000928911	[illegible]
**mmu-miR-149-5p**	2.752609418	3.248716371	0.94241611	16.54228631	0.000877669	[illegible]
**mmu-miR-668-3p**	2.494813423	3.241901474	0.939648678	25.6550206	1.13E-05	[illegible]
**mmu-miR-204-5p**	142.8778395	3.234563317	0.270281119	188.0616637	1.60E-40	[illegible]
**mmu-miR-341-3p**	2.680940249	3.140686387	0.950575716	15.08518527	0.00174528	[illegible]
**mmu-miR-127-5p**	52.01520914	3.087900308	0.355987554	92.34797671	6.86E-20	[illegible]
**mmu-miR-296-3p**	3.425533797	3.066632877	0.812289215	32.88449935	3.41E-07	[illegible]
**mmu-miR-106a-5p**	4.724260817	3.022079764	0.946688098	30.18749125	1.26E-06	[illegible]
**mmu-miR-135b-5p**	2.013481273	2.918947685	1.024831751	14.48420156	0.002314957	[illegible]
**mmu-miR-494-3p**	1.778713296	2.91000207	1.005482438	9.88918672	0.01953196	[illegible]
**mmu-miR-216a-5p**	1.723713615	2.887274041	0.999701426	19.05686152	0.000266096	[illegible]
**mmu-miR-493-5p**	1.956600028	2.764756129	0.997455569	10.48847376	0.01483929	[illegible]
**mmu-miR-335-3p**	5.673791222	2.677309482	0.578309786	54.97879249	6.94E-12	[illegible]
**mmu-miR-434-3p**	14.28393976	2.566931983	0.52025787	47.88527996	2.25E-10	[illegible]
**mmu-miR-369-3p**	2.382490077	2.523098098	1.018041772	15.17650078	0.001671863	[illegible]
**mmu-miR-3544-5p**	1.527235619	2.522788682	1.075317891	8.665529076	0.034084812	[illegible]
**mmu-miR-125b-1-3p**	5.892025718	2.500599302	0.832647688	23.75661236	2.81E-05	[illegible]
**mmu-miR-300-3p**	52.79264464	2.457524384	0.318019741	88.54115228	4.51E-19	[illegible]
**mmu-miR-431-5p**	72.16523507	2.35861296	0.233309861	134.1941855	6.75E-29	[illegible]
**mmu-miR-150-3p**	13.72767638	2.252496306	0.534047759	82.30478517	9.83E-18	[illegible]
**mmu-miR-434-5p**	148.587505	2.245369687	0.23749898	92.48568734	6.41E-20	[illegible]
**mmu-miR-150-5p**	330.436374	2.210847322	0.397709737	84.92170681	2.70E-18	[illegible]
**mmu-miR-299a-5p@**	13.51145799	2.182748202	0.457209083	101.7567615	6.51E-22	[illegible]
**mmu-miR-127-3p**	29.66110405	2.144325976	0.329446794	63.90263594	8.61E-14	[illegible]
**mmu-miR-363-3p**	14.43168287	2.127251723	0.654364717	58.24859941	1.39E-12	[illegible]
**mmu-miR-376b-3p**	3.200933073	2.123282063	0.828625811	9.435533404	0.024027159	[illegible]
**mmu-miR-136-5p**	6.639621401	2.102591301	0.615060326	14.31732764	0.002503544	[illegible]
**mmu-miR-211-5p**	6.693361953	2.081352424	0.82242764	77.16712744	1.24E-16	[illegible]

Biology Open • Supplementary information

**mmu-miR-411-5p**	65.66944853	2.072042283	0.256692773	100.2883903	1.35E-21	[illegible]
**mmu-miR-409-3p**	5.32959814	2.031555651	0.58579314	18.97339259	0.000276884	[illegible]
**mmu-miR-17-3p**	182.2035116	2.003163209	0.201271816	111.6446379	4.86E-24	[illegible]
**mmu-miR-376c-3p**	5.056501427	1.932833972	0.618932227	11.83144382	0.007983442	[illegible]
**mmu-miR-92a-3p**	411.5862409	1.922184567	0.253890771	92.28859092	7.06E-20	[illegible]
**mmu-miR-326-3p**	9.016788561	1.921179118	0.615350579	20.01632857	0.000168425	[illegible]
**mmu-miR-671-3p**	1.164669384	1.890738392	1.110893085	10.30910594	0.016113362	[illegible]
**mmu-miR-1193-3p**	5.828094126	1.863167052	0.5640625	16.69501525	0.0008165	[illegible]
**mmu-miR-379-3p**	1.450902841	1.837580518	1.066705447	8.313310259	0.039961446	[illegible]
**mmu-miR-3964**	142.0915454	1.814567837	0.341689221	103.0776678	3.39E-22	[illegible]
**mmu-miR-20b-5p**	30.21191906	1.806206681	0.400622689	78.83356523	5.46E-17	[illegible]
**mmu-miR-541-5p**	394.5785517	1.800529644	0.240042507	67.28882543	1.62E-14	[illegible]
**mmu-miR-181d-5p**	12.72706095	1.792273655	0.5598952	24.32555868	2.14E-05	[illegible]
**mmu-miR-381-3p**	85.21617944	1.74477171	0.263586256	50.82325545	5.34E-11	[illegible]
**mmu-miR-700-5p**	4.052344936	1.744670819	0.873469533	15.41006655	0.001497727	[illegible]
**mmu-miR-379-5p**	88.82524526	1.742681495	0.263410924	47.97297091	2.16E-10	[illegible]
**mmu-miR-3071-3p**	95.27009915	1.705488572	0.296834405	37.8126863	3.10E-08	[illegible]
**mmu-miR-330-3p**	1.386734368	1.658486062	1.127649477	13.46145738	0.003737876	[illegible]
**mmu-miR-337-5p**	4.620031886	1.653131447	0.589210524	11.76579076	0.00823008	[illegible]
**mmu-miR-3071-5p**	3.757106464	1.646700194	0.73506882	12.37465325	0.006203992	[illegible]
**mmu-miR-93-3p**	18.41715709	1.631714876	0.352139926	24.96735054	1.57E-05	[illegible]
**mmu-miR-485-3p**	1.296062063	1.59332447	1.172520003	11.60929352	0.008848739	[illegible]
**mmu-miR-511-5p**	2.743335476	1.567913347	0.990571184	10.25921995	0.016486444	[illegible]
**mmu-miR-351-5p**	42.36806337	1.520374221	0.250458327	53.33924599	1.55E-11	[illegible]
**mmu-miR-342-3p**	1493.620355	1.49253993	0.20393853	102.8937072	3.71E-22	[illegible]
**mmu-miR-701-5p**	4.683618434	1.47975277	0.617919355	9.01956405	0.0290319	[illegible]
**mmu-miR-331-3p**	13.40286356	1.471872441	0.4452835	21.85677666	6.99E-05	[illegible]
**mmu-miR-203-5p**	56.11999962	1.461509305	0.286825185	64.11737031	7.75E-14	[illegible]
**mmu-miR-125b-5p**	9910.934803	1.458649704	0.243562722	83.83561846	4.61E-18	[illegible]
**mmu-miR-181c-3p**	6.872165404	1.44514677	0.471474945	16.67052598	0.000826015	[illegible]
**mmu-miR-503-5p**	52.59856417	1.418315582	0.231765828	52.60728497	2.22E-11	[illegible]
**mmu-miR-1941-3p**	1.366980064	1.405504009	1.118973598	11.17500682	0.010816227	[illegible]
**mmu-miR-615-3p**	1.208631326	1.401535011	1.149065921	14.71567934	0.002076469	[illegible]
**mmu-miR-485-5p**	3.626299386	1.348113548	0.592652652	14.16232311	0.002692326	[illegible]

Biology Open • Supplementary information

**mmu-miR-154-5p**	14.06057049	1.339441292	0.473976414	25.43829418	1.25E-05	[illegible]
**mmu-miR-382-5p**	30.44854108	1.320952997	0.317939391	22.02791483	6.44E-05	[illegible]
**mmu-miR-214-3p**	462.0085635	1.318026398	0.285364656	55.55352296	5.23E-12	[illegible]
**mmu-miR-409-5p**	8.195583015	1.303107582	0.56702257	39.22530328	1.56E-08	[illegible]
**mmu-miR-322-3p**	33.7094442	1.30104508	0.269387761	27.82918042	3.94E-06	[illegible]
**mmu-miR-134-5p**	7.947980037	1.295595144	0.560550905	10.27640052	0.016357006	[illegible]
**mmu-miR-205-5p**	382.7489282	1.272895114	0.26291117	57.13912119	2.40E-12	[illegible]
**mmu-miR-350-5p**	8.714247927	1.261423567	0.553030554	28.24658548	3.22E-06	[illegible]
**mmu-miR-376b-5p**	46.81228445	1.219078058	0.253634759	32.91798078	3.35E-07	[illegible]
**mmu-miR-455-3p**	7.668250687	1.213376093	0.616987922	30.39922067	1.14E-06	[illegible]
**mmu-miR-674-3p**	66.27260057	1.199280191	0.214186874	35.04425633	1.19E-07	[illegible]
**mmu-miR-3544-3p**	2.460864555	1.15250911	0.801590189	9.238071919	0.026287465	[illegible]
**mmu-miR-184-3p**	122.6865433	1.147557578	0.325778175	265.9586807	2.31E-57	[illegible]
**mmu-miR-138-5p**	116.0548328	1.131735615	0.154524068	148.4969584	5.56E-32	[illegible]
**mmu-miR-466h-3p**	1.56793887	1.115867963	1.081116625	13.94654374	0.002978826	[illegible]
**mmu-miR-15b-3p**	74.36748757	1.090658005	0.224876533	25.77318355	1.06E-05	[illegible]
**mmu-miR-224-5p**	202.8814491	1.084303166	0.177836967	76.35456931	1.86E-16	[illegible]
**mmu-miR-342-5p**	25.49821991	1.082172311	0.275962275	21.123541	9.92E-05	[illegible]
**mmu-miR-199a-3p**	42927.94576	1.069258378	0.145682478	105.3947673	1.07E-22	[illegible]
**mmu-miR-142a-5p**	13529.79614	1.060765156	0.333223428	34.56137459	1.51E-07	[illegible]
**mmu-miR-139-3p**	15.2495545	1.057349151	0.38057969	16.39173083	0.000942418	[illegible]
**mmu-miR-100-5p**	47.36730637	1.032124996	0.344501203	67.00662106	1.87E-14	[illegible]
**mmu-miR-15a-3p**	7.705565683	1.017122328	0.403020358	14.00615076	0.002896792	[illegible]
**Down-regulated**						
**mmu-miR-101a-3p@**	8855.379703	-1.002622304	0.140487677	67.81557628	1.25E-14	[illegible]
**mmu-miR-1291**	10.98775681	-1.003005619	0.350097198	12.21456112	0.006683167	[illegible]
**mmu-miR-672-3p**	9.101251328	-1.020812879	0.375532644	23.12643931	3.80E-05	[illegible]
**mmu-miR-3473e**	1.875782239	-1.040866506	0.830312611	9.518313147	0.023137335	[illegible]
**mmu-miR-148b-3p**	86.55182962	-1.044779592	0.180831818	65.26361631	4.41E-14	[illegible]
**mmu-miR-221-5p**	6.713831631	-1.062240358	0.456003805	16.84736397	0.000759706	[illegible]
**mmu-miR-5123**	6.536994105	-1.07165406	0.437460915	54.42264323	9.12E-12	[illegible]
**mmu-miR-340-3p**	27.95687978	-1.075614737	0.221668119	113.3865196	2.05E-24	[illegible]
**mmu-miR-29a-3p**	25777.64838	-1.115784792	0.159268416	142.3109259	1.20E-30	[illegible]
**mmu-miR-3064-5p**	6.280270231	-1.161005228	0.428438961	22.12958127	6.13E-05	[illegible]

Biology Open • Supplementary information

**mmu-miR-877-5p**	2.684356842	-1.161187118	0.597959326	11.88684236	0.007781016	[illegible]
**mmu-miR-340-5p**	147.3454342	-1.163848778	0.215499499	75.09296962	3.46E-16	[illegible]
**mmu-miR-7a-1-3p**	26.91930029	-1.16409829	0.221945347	61.16401665	3.32E-13	[illegible]
**mmu-miR-215-5p**	1.393093243	-1.175527471	1.228435674	9.100685617	0.02798174	[illegible]
**mmu-miR-190b-5p**	13.11926493	-1.181559181	0.320120456	16.70354662	0.000813211	[illegible]
**mmu-let-7i-3p**	109.5463489	-1.201693226	0.241803158	36.98236567	4.64E-08	[illegible]
**mmu-miR-421-3p**	7.702981089	-1.20350286	0.368848976	18.46078392	0.000353357	[illegible]
**mmu-miR-29c-3p**	1543.69309	-1.224507796	0.179832002	65.95877196	3.13E-14	[illegible]
**mmu-miR-133a-3p**	2368.674849	-1.229650689	0.769982229	37.89554622	2.97E-08	[illegible]
**mmu-miR-29b-3p**	414.7676513	-1.230964265	0.167138697	61.72457278	2.52E-13	[illegible]
**mmu-miR-7a-5p**	212.1450193	-1.238229476	0.150711789	205.2393457	3.11E-44	[illegible]
**mmu-miR-29b-2-5p**	4.247138664	-1.240994959	0.611757858	9.783679066	0.02049734	[illegible]
**mmu-miR-345-5p**	24.269991	-1.244768081	0.24162547	34.29186603	1.72E-07	[illegible]
**mmu-miR-664-5p**	14.02408157	-1.256912429	0.349641441	16.08140091	0.00109123	[illegible]
**mmu-miR-200a-5p**	87.35168412	-1.257458973	0.239654017	242.9168492	2.23E-52	[illegible]
**mmu-miR-5113**	1.472388213	-1.274186345	0.896848752	9.297933464	0.025581079	[illegible]
**mmu-miR-190a-5p**	116.293464	-1.327704098	0.265163036	152.9896728	5.97E-33	[illegible]
**mmu-miR-125a-3p**	5.835420597	-1.335955212	0.457134183	16.70392412	0.000813066	[illegible]
**mmu-miR-151-5p**	566.287775	-1.339614409	0.225145781	90.97409583	1.35E-19	[illegible]
**mmu-miR-141-5p**	1035.269983	-1.36064078	0.272238792	175.4863061	8.32E-38	[illegible]
**mmu-miR-30a-5p**	5866.960062	-1.378370201	0.135337832	112.5553632	3.09E-24	[illegible]
**mmu-miR-500-3p**	106.2269419	-1.402895466	0.21799832	41.09889966	6.23E-09	[illegible]
**mmu-miR-181a-5p**	825.2492268	-1.408824895	0.273751608	39.41505446	1.42E-08	[illegible]
**mmu-miR-200a-3p**	35951.03325	-1.416775729	0.24322018	193.7261763	9.56E-42	[illegible]
**mmu-miR-148a-3p**	19463.75931	-1.417662929	0.293975848	229.8395854	1.50E-49	[illegible]
**mmu-miR-200b-3p**	14790.12574	-1.427088362	0.179796545	315.8843014	3.63E-68	[illegible]
**mmu-miR-133b-3p**	112.5629847	-1.497870538	0.961041805	27.51563631	4.59E-06	[illegible]
**mmu-miR-96-5p**	223.6361993	-1.503409589	0.24688175	205.6995888	2.47E-44	[illegible]
**mmu-miR-1968-5p**	4.450988056	-1.531284008	0.436543049	53.90306352	1.18E-11	[illegible]
**mmu-miR-378c**	75.34400739	-1.54070024	0.179451638	101.3258314	8.06E-22	[illegible]
**mmu-miR-672-5p**	53.29376199	-1.567234085	0.212582067	194.5663199	6.30E-42	[illegible]
**mmu-let-7g-3p**	6.175412608	-1.577593548	0.565418813	11.09111289	0.011243259	[illegible]
**mmu-miR-210-3p**	285.0656017	-1.588809675	0.308827042	255.9948338	3.30E-55	[illegible]
**mmu-miR-193b-3p**	1.732913591	-1.614580772	0.949430917	13.62903891	0.003456151	[illegible]

Biology Open • Supplementary information

**mmu-miR-6538**	2.465253541	-1.637598154	0.82342853	16.79837945	0.000777525	[illegible]
**mmu-miR-1839-5p**	155.2904071	-1.638018585	0.130706212	193.1017306	1.30E-41	[illegible]
**mmu-miR-676-5p**	38.35286563	-1.640346613	0.232540654	59.88512628	6.22E-13	[illegible]
**mmu-miR-466m-5p@**	0.934441196	-1.659195903	1.224130911	9.211915639	0.026602087	[illegible]
**mmu-miR-429-3p**	951.1698198	-1.665145176	0.184844343	333.7978101	4.81E-72	[illegible]
**mmu-miR-374b-5p@**	4.430587298	-1.721238665	0.636131778	9.536047798	0.022950948	[illegible]
**mmu-miR-133a-5p**	89.82058384	-1.741684634	0.884463128	23.98291669	2.52E-05	[illegible]
**mmu-miR-222-3p**	24.53746285	-1.749162714	0.289199699	51.54045632	3.75E-11	[illegible]
**mmu-miR-3068-3p**	5.844458955	-1.772125168	0.495925426	13.78719642	0.003209601	[illegible]
**mmu-miR-144-3p**	146.8244187	-1.781590305	0.237706784	145.7667998	2.16E-31	[illegible]
**mmu-miR-146b-3p**	5.559283647	-1.78827225	0.471767595	38.18609841	2.58E-08	[illegible]
**mmu-miR-3473b**	25.57216464	-1.898010578	0.401031496	38.43694948	2.28E-08	[illegible]
**mmu-miR-148b-5p**	5.158771695	-1.922130746	0.49124937	25.88687776	1.01E-05	[illegible]
**mmu-miR-3470b**	0.862074046	-1.980955821	1.188860193	8.792230749	0.03218472	[illegible]
**mmu-miR-182-5p**	2833.429736	-1.989187347	0.213541644	289.9560869	1.48E-62	[illegible]
**mmu-miR-193b-5p**	4.029366624	-2.010557622	0.558343669	19.05459976	0.000266383	[illegible]
**mmu-miR-1191a**	19.32929526	-2.093021836	0.268398113	109.3067535	1.55E-23	[illegible]
**mmu-miR-3068-5p**	417.5726768	-2.097412297	0.140346726	272.0517069	1.11E-58	[illegible]
**mmu-miR-1a-3p**	404.1350897	-2.114273486	0.847789648	23.9040173	2.62E-05	[illegible]
**mmu-miR-33-5p**	32.11388508	-2.161889818	0.28812837	85.51408907	2.01E-18	[illegible]
**mmu-miR-29b-1-5p**	10.36523807	-2.192495014	0.367874548	74.08056508	5.70E-16	[illegible]
**mmu-miR-676-3p**	49.60781438	-2.194578855	0.23817691	95.96128113	1.15E-20	[illegible]
**mmu-miR-1a-1-5p**	1.358764567	-2.203405613	1.509331509	21.910641	6.81E-05	[illegible]
**mmu-miR-181a-1-3p**	53.42133543	-2.210023971	0.234129218	216.3452141	1.24E-46	[illegible]
**mmu-miR-1b-5p**	3426.674947	-2.222329578	0.866150846	22.47396601	5.20E-05	[illegible]
**mmu-miR-181b-2-3p**	5.867149593	-2.381037753	0.522418983	39.24909583	1.54E-08	[illegible]
**mmu-miR-3105-5p**	29.8045294	-2.384993477	0.402511629	151.5600771	1.21E-32	[illegible]
**mmu-miR-141-3p**	2366.759146	-2.430911171	0.257999175	308.4345092	1.49E-66	[illegible]
**mmu-miR-181a-2-3p**	3.726180378	-2.488033095	0.684401365	18.51713506	0.000344015	[illegible]
**mmu-miR-7033-5p**	1.52872816	-2.558090834	1.256699604	10.41881239	0.015321875	[illegible]
**mmu-miR-378b**	31.07898871	-2.661778817	0.274170254	115.4646024	7.31E-25	[illegible]
**mmu-miR-466q**	2.956602727	-2.811024158	0.784066214	19.36045768	0.000230269	[illegible]
**mmu-miR-7116-5p**	2.724939119	-3.654598717	0.772130762	71.44037146	2.10E-15	[illegible]
**mmu-miR-375-3p**	307.7228915	-5.219530867	0.53979291	136.1307695	2.58E-29	[illegible]

Table S9. miRNA differentially expressed between lactating (P18) and involuting (Lac) mice.

miRNA	baseMean	log2FoldChange	lfcSE	stat	pvalue	[illegible]
Up-regulated						
**mmu-miR-7116-5p**	2.724939119	4.788850923	0.93631329	71.4403715	2.10E-15	[illegible]
**mmu-miR-206-3p**	24.99298637	3.957483426	0.97361186	21.1550127	9.78E-05	[illegible]
**mmu-miR-133a-5p**	89.82058384	3.675021282	0.90043993	23.9829167	2.52E-05	[illegible]
**mmu-miR-1968-5p**	4.450988056	3.551762983	0.6738222	53.9030635	1.18E-11	[illegible]
**mmu-miR-210-3p**	285.0656017	3.519936377	0.31468663	255.994834	3.30E-55	[illegible]
**mmu-miR-1b-5p**	3426.674947	3.416024618	0.86639237	22.473966	5.20E-05	[illegible]
**mmu-miR-1a-3p**	404.1350897	3.386953828	0.85003618	23.9040173	2.62E-05	[illegible]
**mmu-miR-5113**	1.472388213	3.243877033	1.12518688	9.29793346	0.02558108	[illegible]
**mmu-miR-375-3p**	307.7228915	2.723336293	0.53130592	136.13077	2.58E-29	[illegible]
**mmu-miR-141-3p**	2366.759146	2.666753765	0.25812402	308.434509	1.49E-66	[illegible]
**mmu-miR-672-5p**	53.29376199	2.547209382	0.23050826	194.56632	6.30E-42	[illegible]
**mmu-miR-148a-3p**	19463.75931	2.543789792	0.29401423	229.839585	1.50E-49	[illegible]
**mmu-miR-181b-5p**	740.6846825	2.376612848	0.26231508	132.058464	1.95E-28	[illegible]
**mmu-miR-133a-3p**	2368.674849	2.336992795	0.77038621	37.8955462	2.97E-08	[illegible]
**mmu-miR-193b-3p**	1.732913591	2.285772807	1.07005519	13.6290389	0.00345615	[illegible]
**mmu-miR-181b-2-3p**	5.867149593	2.269843966	0.52074986	39.2490958	1.54E-08	[illegible]
**mmu-miR-190a-5p**	116.293464	2.20035139	0.27031727	152.989673	5.97E-33	[illegible]
**mmu-miR-133b-3p**	112.5629847	2.167232873	0.96588971	27.5156363	4.59E-06	[illegible]
**mmu-miR-144-3p**	146.8244187	2.161634697	0.24023824	145.7668	2.16E-31	[illegible]
**mmu-miR-3105-3p**	2.723923826	2.109171874	0.76313203	44.0232813	1.49E-09	[illegible]
**mmu-miR-181a-1-3p**	53.42133543	2.106788314	0.23398744	216.345214	1.24E-46	[illegible]
**mmu-miR-3105-5p**	29.8045294	2.101449241	0.3995704	151.560077	1.21E-32	[illegible]
**mmu-miR-5123**	6.536994105	2.045298269	0.48949218	54.4226432	9.12E-12	[illegible]
**mmu-miR-29b-1-5p**	10.36523807	1.975357129	0.36142195	74.0805651	5.70E-16	[illegible]
**mmu-miR-148b-5p**	5.158771695	1.928852963	0.49822982	25.8868778	1.01E-05	[illegible]
**mmu-miR-193b-5p**	4.029366624	1.91775252	0.55767868	19.0545998	0.00026638	[illegible]
**mmu-miR-148a-5p**	15.72949366	1.870687998	0.35766479	116.597167	4.17E-25	[illegible]
**mmu-miR-466q**	2.956602727	1.798840306	0.68817169	19.3604577	0.00023027	[illegible]
**mmu-miR-1191a**	19.32929526	1.786960578	0.26059412	109.306754	1.55E-23	[illegible]
**mmu-miR-200a-5p**	87.35168412	1.785214046	0.2438279	242.916849	2.23E-52	[illegible]
**mmu-miR-182-5p**	2833.429736	1.781454314	0.21351255	289.956087	1.48E-62	[illegible]

Biology Open • Supplementary information

**mmu-miR-151-5p**	566.287775	1.75109691	0.22579857	90.9740958	1.35E-19	[illegible]
**mmu-miR-200b-3p**	14790.12574	1.736413083	0.17981946	315.884301	3.63E-68	[illegible]
**mmu-miR-146b-5p**	24587.01652	1.735635272	0.25684508	279.64027	2.53E-60	[illegible]
**mmu-miR-672-3p**	9.101251328	1.691790171	0.40814724	23.1264393	3.80E-05	[illegible]
**mmu-miR-141-5p**	1035.269983	1.690847216	0.27245606	175.486306	8.32E-38	[illegible]
**mmu-miR-429-3p**	951.1698198	1.664912789	0.18493137	333.79781	4.81E-72	[illegible]
**mmu-miR-33-5p**	32.11388508	1.66069325	0.27979512	85.5140891	2.01E-18	[illegible]
**mmu-miR-1a-1-5p**	1.358764567	1.631647657	1.50933151	21.910641	6.81E-05	[illegible]
**mmu-miR-877-5p**	2.684356842	1.623800594	0.64627603	11.8868424	0.00778102	[illegible]
**mmu-miR-125a-3p**	5.835420597	1.609609567	0.47834282	16.7039241	0.00081307	[illegible]
**mmu-miR-7a-5p**	212.1450193	1.578384946	0.15273433	205.239346	3.11E-44	[illegible]
**mmu-miR-30b-3p**	70.94130362	1.565763403	0.17391409	141.877669	1.49E-30	[illegible]
**mmu-miR-96-5p**	223.6361993	1.550445721	0.24725835	205.699589	2.47E-44	[illegible]
**mmu-miR-183-3p**	19.83412676	1.474931215	0.27398325	124.909154	6.76E-27	[illegible]
**mmu-miR-7a-1-3p**	26.91930029	1.451957645	0.23112925	61.1640167	3.32E-13	[illegible]
**mmu-miR-181a-5p**	825.2492268	1.442829177	0.27385308	39.4150545	1.42E-08	[illegible]
**mmu-miR-378b**	31.07898871	1.442400263	0.24396028	115.464602	7.31E-25	[illegible]
**mmu-miR-200a-3p**	35951.03325	1.4249427	0.24322189	193.726176	9.56E-42	[illegible]
**mmu-miR-466i-5p**	6.027872382	1.404930388	0.45186289	29.6276552	1.65E-06	[illegible]
**mmu-let-7g-3p**	6.175412608	1.373338264	0.56015544	11.0911129	0.01124326	[illegible]
**mmu-miR-340-3p**	27.95687978	1.347600649	0.2291788	113.38652	2.05E-24	[illegible]
**mmu-miR-101b-3p**	2415.133268	1.332029957	0.14231522	126.837423	2.60E-27	[illegible]
**mmu-miR-32-5p**	351.9228231	1.28283427	0.18854803	59.0617395	9.33E-13	[illegible]
**mmu-miR-181b-1-3p**	11.2446293	1.280908326	0.37589539	56.3734425	3.50E-12	[illegible]
**mmu-miR-16-2-3p**	3.214709022	1.280782828	0.73672349	12.0914449	0.00707638	[illegible]
**mmu-miR-103-3p**	34469.00084	1.2293933	0.09564329	235.234037	1.02E-50	[illegible]
**mmu-miR-335-5p**	67.0422257	1.216246338	0.23637112	108.265862	2.59E-23	[illegible]
**mmu-miR-200c-3p**	5340.032311	1.197380509	0.20340481	111.179802	6.12E-24	[illegible]
**mmu-miR-192-5p**	894.4673823	1.189171194	0.22427018	45.1702685	8.51E-10	[illegible]
**mmu-miR-345-3p**	95.9386559	1.17483844	0.14010773	89.9144551	2.29E-19	[illegible]
**mmu-miR-3064-5p**	6.280270231	1.172747037	0.43327277	22.1295813	6.13E-05	[illegible]
**mmu-miR-421-3p**	7.702981089	1.166563777	0.37130128	18.4607839	0.00035336	[illegible]
**mmu-miR-345-5p**	24.269991	1.116399504	0.24061557	34.291866	1.72E-07	[illegible]
**mmu-miR-146b-3p**	5.559283647	1.114271844	0.43846715	38.1860984	2.58E-08	[illegible]

Biology Open • Supplementary information

**mmu-miR-466m-5p@**	0.934441196	1.109143373	1.20934405	9.21191564	0.02660209	[illegible]
**mmu-miR-30c-2-3p**	30.6319151	1.102488701	0.25247516	39.9450301	1.09E-08	[illegible]
**mmu-miR-30a-3p**	139.3109028	1.076373747	0.15467706	65.9729031	3.11E-14	[illegible]
**mmu-miR-339-3p**	5.486896096	1.06931793	0.44005329	14.2016047	0.00264319	[illegible]
**mmu-miR-190b-5p**	13.11926493	1.026097205	0.31771964	16.7035466	0.00081321	[illegible]
**mmu-miR-3473b**	25.57216464	1.015031997	0.38914311	38.4369495	2.28E-08	[illegible]
**Down-regulated**						
**mmu-miR-199a-5p**	2631.055333	-1.027924782	0.14380582	80.3526042	2.58E-17	[illegible]
**mmu-miR-223-3p**	1249.657316	-1.03282413	0.1443045	59.5059076	7.50E-13	[illegible]
**mmu-miR-154-5p**	14.06057049	-1.048486527	0.48096258	25.4382942	1.25E-05	[illegible]
**mmu-miR-434-3p**	14.28393976	-1.053719526	0.54809279	47.88528	2.25E-10	[illegible]
**mmu-miR-381-3p**	85.21617944	-1.066475838	0.26707328	50.8232554	5.34E-11	[illegible]
**mmu-miR-136-5p**	6.639621401	-1.06704218	0.64153881	14.3173276	0.00250354	[illegible]
**mmu-miR-409-5p**	8.195583015	-1.067090104	0.57701283	39.2253033	1.56E-08	[illegible]
**mmu-miR-130b-3p**	8.19347228	-1.079498357	0.39267781	11.8698218	0.00784266	[illegible]
**mmu-miR-497a-3p**	8.728692242	-1.093608366	0.47784132	10.3558872	0.01577105	[illegible]
**mmu-miR-425-3p**	40.00120665	-1.100179417	0.2602235	19.3659633	0.00022967	[illegible]
**mmu-miR-92a-3p**	411.5862409	-1.104188511	0.25494776	92.2885909	7.06E-20	[illegible]
**mmu-miR-598-3p**	4.964432544	-1.107098091	0.62478715	13.9610702	0.00295862	[illegible]
**mmu-miR-342-5p**	25.49821991	-1.118635945	0.27670331	21.123541	9.92E-05	[illegible]
**mmu-miR-132-3p**	12.85775041	-1.121325129	0.33737656	27.0396944	5.78E-06	[illegible]
**mmu-miR-199a-3p**	42927.94576	-1.138653365	0.14568303	105.394767	1.07E-22	[illegible]
**mmu-miR-214-5p**	51.65672992	-1.163670178	0.24596207	41.2948706	5.66E-09	[illegible]
**mmu-miR-142a-5p**	13529.79614	-1.188715836	0.33322274	34.5613746	1.51E-07	[illegible]
**mmu-miR-299a-5p@**	13.51145799	-1.203211857	0.48120956	101.756762	6.51E-22	[illegible]
**mmu-miR-493-5p**	1.956600028	-1.213164916	1.082023	10.4884738	0.01483929	[illegible]
**mmu-miR-497a-5p**	860.4505093	-1.237334531	0.21185962	55.7058408	4.85E-12	[illegible]
**mmu-miR-140-3p**	2068.444009	-1.263272072	0.13283291	149.959879	2.69E-32	[illegible]
**mmu-miR-301b-3p**	10.35940794	-1.267935946	0.42404304	10.790159	0.0129164	[illegible]
**mmu-miR-339-5p**	290.5954939	-1.280580043	0.13173318	172.323796	4.01E-37	[illegible]
**mmu-miR-434-5p**	148.587505	-1.281916568	0.2404386	92.4856873	6.41E-20	[illegible]
**mmu-miR-127-3p**	29.66110405	-1.282614272	0.33986073	63.9026359	8.61E-14	[illegible]
**mmu-miR-376c-3p**	5.056501427	-1.30222522	0.63937169	11.8314438	0.00798344	[illegible]
**mmu-miR-219a-5p**	14.26926358	-1.310596873	0.46296792	21.6166765	7.84E-05	[illegible]

Biology Open • Supplementary information

**mmu-miR-214-3p**	462.0085635	-1.317325083	0.28545596	55.553523	5.23E-12	[illegible]
**mmu-miR-3544-5p**	1.527235619	-1.321390321	1.16384437	8.66552908	0.03408481	[illegible]
**mmu-miR-142a-3p**	2016.056019	-1.365274809	0.31726135	40.3684795	8.90E-09	[illegible]
**mmu-miR-21a-5p**	19034.2627	-1.38273032	0.15178252	132.370696	1.67E-28	[illegible]
**mmu-miR-135a-5p**	4.062312633	-1.388183605	0.73400769	24.5952806	1.88E-05	[illegible]
**mmu-miR-93-3p**	18.41715709	-1.40426512	0.35647556	24.9673505	1.57E-05	[illegible]
**mmu-miR-23a-3p**	8642.634214	-1.407097388	0.12169509	161.124285	1.05E-34	[illegible]
**mmu-miR-142b**	31041.08194	-1.426565864	0.31384528	43.3845252	2.04E-09	[illegible]
**mmu-miR-298-5p**	4.789563109	-1.432272732	0.96908938	65.0757268	4.83E-14	[illegible]
**mmu-miR-199b-5p**	347.8513637	-1.433591103	0.26041504	98.068008	4.04E-21	[illegible]
**mmu-miR-503-5p**	52.59856417	-1.451484271	0.23219547	52.607285	2.22E-11	[illegible]
**mmu-miR-139-3p**	15.2495545	-1.462203834	0.3755962	16.3917308	0.00094242	[illegible]
**mmu-miR-155-5p**	103.676912	-1.46541677	0.25343783	58.3572391	1.32E-12	[illegible]
**mmu-miR-224-5p**	202.8814491	-1.477280191	0.17705137	76.3545693	1.86E-16	[illegible]
**mmu-miR-328-3p**	4.365593314	-1.485794616	0.70502566	10.0544441	0.01810897	[illegible]
**mmu-miR-1941-3p**	1.366980064	-1.508635543	1.11745665	11.1750068	0.01081623	[illegible]
**mmu-miR-486a-3p@**	22.16840226	-1.593679155	0.31265446	83.7570086	4.80E-18	[illegible]
**mmu-miR-467a-3p@**	12.31254233	-1.619875649	0.61949365	60.0472405	5.74E-13	[illegible]
**mmu-miR-135b-5p**	2.013481273	-1.631956864	1.10293327	14.4842016	0.00231496	[illegible]
**mmu-miR-376b-3p**	3.200933073	-1.648231028	0.84841456	9.4355334	0.02402716	[illegible]
**mmu-miR-668-3p**	2.494813423	-1.666525216	1.03102325	25.6550206	1.13E-05	[illegible]
**mmu-miR-351-5p**	42.36806337	-1.66763078	0.25001667	53.339246	1.55E-11	[illegible]
**mmu-miR-100-5p**	47.36730637	-1.683537882	0.34000456	67.0066211	1.87E-14	[illegible]
**mmu-miR-211-5p**	6.693361953	-1.699725597	0.83900504	77.1671274	1.24E-16	[illegible]
**mmu-miR-184-3p**	122.6865433	-1.71039471	0.32260923	265.958681	2.31E-57	[illegible]
**mmu-miR-125b-5p**	9910.934803	-1.732191063	0.24355482	83.8356185	4.61E-18	[illegible]
**mmu-miR-5121**	7.95918194	-1.767183496	0.48208948	32.1030359	4.98E-07	[illegible]
**mmu-miR-344b-3p**	1.748094698	-1.791789604	1.08666147	8.01806885	0.04563975	[illegible]
**mmu-miR-17-3p**	182.2035116	-1.833364144	0.20193109	111.644638	4.86E-24	[illegible]
**mmu-miR-423-3p**	17.57200617	-1.866481476	0.36559794	52.7445392	2.08E-11	[illegible]
**mmu-miR-342-3p**	1493.620355	-1.880113253	0.20384421	102.893707	3.71E-22	[illegible]
**mmu-miR-99a-5p**	421.4757133	-1.883554046	0.30961737	108.114553	2.79E-23	[illegible]
**mmu-miR-299a-3p@**	3.09667361	-1.953382306	0.84580987	7.81094328	0.05008488	[illegible]
**mmu-miR-411-5p**	65.66944853	-1.956061157	0.25793782	100.28839	1.35E-21	[illegible]

Biology Open • Supplementary information

**mmu-miR-146a-5p**	8463.671814	-1.984109307	0.21525756	84.2760552	3.71E-18	[illegible]
**mmu-miR-205-5p**	382.7489282	-1.994141771	0.26239208	57.1391212	2.40E-12	[illegible]
**mmu-miR-494-3p**	1.778713296	-1.995003115	1.05658494	9.88918672	0.01953196	[illegible]
**mmu-miR-874-5p**	1.200022433	-2.009783493	1.08592659	8.27379848	0.04067939	[illegible]
**mmu-let-7d-3p**	47.16435266	-2.010715324	0.3471939	83.6166953	5.14E-18	[illegible]
**mmu-miR-326-3p**	9.016788561	-2.011338493	0.61542736	20.0163286	0.00016842	[illegible]
**mmu-miR-150-3p**	13.72767638	-2.063217776	0.53911851	82.3047852	9.83E-18	[illegible]
**mmu-miR-455-3p**	7.668250687	-2.080581357	0.59700277	30.3992207	1.14E-06	[illegible]
**mmu-miR-150-5p**	330.436374	-2.096206665	0.39795823	84.9217068	2.70E-18	[illegible]
**mmu-miR-20b-5p**	30.21191906	-2.146556509	0.3980913	78.8335652	5.46E-17	[illegible]
**mmu-miR-216a-5p**	1.723713615	-2.14918119	1.05543101	19.0568615	0.0002661	[illegible]
**mmu-miR-511-3p**	15.66389615	-2.157031905	0.45577626	51.9795401	3.03E-11	[illegible]
**mmu-miR-350-5p**	8.714247927	-2.159889287	0.53483026	28.2465855	3.22E-06	[illegible]
**mmu-miR-671-3p**	1.164669384	-2.166780003	1.09433703	10.3091059	0.01611336	[illegible]
**mmu-miR-300-3p**	52.79264464	-2.171673091	0.32031371	88.5411523	4.51E-19	[illegible]
**mmu-miR-223-5p**	24.23993631	-2.192652559	0.35865703	46.8702137	3.70E-10	[illegible]
**mmu-miR-34a-3p**	4.621206314	-2.202495226	0.6329629	20.6988184	0.00012158	[illegible]
**mmu-miR-127-5p**	52.01520914	-2.230813272	0.3610949	92.3479767	6.86E-20	[illegible]
**mmu-miR-350-3p**	99.13806399	-2.264594101	0.28281516	116.970498	3.47E-25	[illegible]
**mmu-miR-615-3p**	1.208631326	-2.314475195	1.06704482	14.7156793	0.00207647	[illegible]
**mmu-miR-700-5p**	4.052344936	-2.379210345	0.85703282	15.4100666	0.00149773	[illegible]
**mmu-miR-511-5p**	2.743335476	-2.423060355	0.96178046	10.25922	0.01648644	[illegible]
**mmu-miR-3964**	142.0915454	-2.446085412	0.34021176	103.077668	3.39E-22	[illegible]
**mmu-miR-369-3p**	2.382490077	-2.62217243	1.01757511	15.1765008	0.00167186	[illegible]
**mmu-miR-329-5p**	2.020057522	-2.751859617	1.02408493	16.4222706	0.00092891	[illegible]
**mmu-miR-125b-1-3p**	5.892025718	-2.914322728	0.82617781	23.7566124	2.81E-05	[illegible]
**mmu-miR-363-3p**	14.43168287	-2.935834845	0.64385542	58.2485994	1.39E-12	[illegible]
**mmu-miR-3083-5p**	0.940606947	-2.96974379	1.15714807	10.9505479	0.01199631	[illegible]
**mmu-miR-149-5p**	2.752609418	-2.99845762	0.95396614	16.5422863	0.00087767	[illegible]
**mmu-miR-21a-3p**	8.218180028	-3.012611996	0.60373778	66.2960148	2.65E-14	[illegible]
**mmu-miR-106a-5p**	4.724260817	-3.057664883	0.94810374	30.1874912	1.26E-06	[illegible]
**mmu-miR-341-3p**	2.680940249	-3.0681176	0.95610252	15.0851853	0.00174528	[illegible]
**mmu-miR-330-3p**	1.386734368	-3.367575171	1.01796429	13.4614574	0.00373788	[illegible]
**mmu-miR-122-5p**	110.62168	-3.771967907	1.86729835	14.9547538	0.00185573	[illegible]

Biology Open • Supplementary information

**mmu-miR-665-3p**	5.308828161	-3.791269996	0.90167683	37.458916	3.68E-08	[illegible]

Table S10. Genes predicted to be modulated by miRNAs differentially expressed between W10 and P18.

userId	geneSymbol	geneName	entrezgene	gLink
Ppp1r1c	Ppp1r1c	protein phosphatase 1, regulatory inhibitor subunit 1C	75276	https://www.ncbi.nlm.nih.gov/gene/?term=75276
Kcnk5	Kcnk5	potassium channel, subfamily K, member 5	16529	https://www.ncbi.nlm.nih.gov/gene/?term=16529
Tmbim6	Tmbim6	transmembrane BAX inhibitor motif containing 6	110213	https://www.ncbi.nlm.nih.gov/gene/?term=110213
Gosr2	Gosr2	golgi SNAP receptor complex member 2	56494	https://www.ncbi.nlm.nih.gov/gene/?term=56494
Tmem170b	Tmem170b	transmembrane protein 170B	621976	https://www.ncbi.nlm.nih.gov/gene/?term=621976
Kmt2c	Kmt2c	lysine (K)-specific methyltransferase 2C	231051	https://www.ncbi.nlm.nih.gov/gene/?term=231051
Slc25a25	Slc25a25	solute carrier family 25 (mitochondrial carrier, phosphate carrier), member 25	227731	https://www.ncbi.nlm.nih.gov/gene/?term=227731
Anks1	Anks1	ankyrin repeat and SAM domain containing 1	224650	https://www.ncbi.nlm.nih.gov/gene/?term=224650
Pparg	Pparg	peroxisome proliferator activated receptor gamma	19016	https://www.ncbi.nlm.nih.gov/gene/?term=19016
Dram2	Dram2	DNA-damage regulated autophagy modulator 2	67171	https://www.ncbi.nlm.nih.gov/gene/?term=67171
Nipal4	Nipal4	NIPA-like domain containing 4	214112	https://www.ncbi.nlm.nih.gov/gene/?term=214112
Omt2a	Omt2a	oocyte maturation, alpha	18379	https://www.ncbi.nlm.nih.gov/gene/?term=18379
Lonrf1	Lonrf1	LON peptidase N-terminal domain and ring finger 1	244421	https://www.ncbi.nlm.nih.gov/gene/?term=244421
Usf3	Usf3	upstream transcription factor family member 3	207806	https://www.ncbi.nlm.nih.gov/gene/?term=207806
Gxylt1	Gxylt1	glucoside xylosyltransferase 1	223827	https://www.ncbi.nlm.nih.gov/gene/?term=223827

Biology Open • Supplementary information

Nr5a2	Nr5a2	nuclear receptor subfamily 5, group A, member 2	26424	https://www.ncbi.nlm.nih.gov/gene/?term=26424
Peli2	Peli2	pellino 2	93834	https://www.ncbi.nlm.nih.gov/gene/?term=93834
Tnpo1	Tnpo1	transportin 1	238799	https://www.ncbi.nlm.nih.gov/gene/?term=238799
Wnk3	Wnk3	WNK lysine deficient protein kinase 3	279561	https://www.ncbi.nlm.nih.gov/gene/?term=279561
Mknk2	Mknk2	MAP kinase-interacting serine/threonine kinase 2	17347	https://www.ncbi.nlm.nih.gov/gene/?term=17347
Rpgrip1l	Rpgrip1l	Rpgrip1-like	244585	https://www.ncbi.nlm.nih.gov/gene/?term=244585
Clcn3	Clcn3	chloride channel, voltage-sensitive 3	12725	https://www.ncbi.nlm.nih.gov/gene/?term=12725
Sval3	Sval3	seminal vesicle antigen-like 3	387564	https://www.ncbi.nlm.nih.gov/gene/?term=387564
Rbpms2	Rbpms2	RNA binding protein with multiple splicing 2	71973	https://www.ncbi.nlm.nih.gov/gene/?term=71973
Map2k4	Map2k4	mitogen-activated protein kinase kinase 4	26398	https://www.ncbi.nlm.nih.gov/gene/?term=26398
Cep135	Cep135	centrosomal protein 135	381644	https://www.ncbi.nlm.nih.gov/gene/?term=381644
Pnkd	Pnkd	paroxysmal nonkinesiogenic dyskinesia	56695	https://www.ncbi.nlm.nih.gov/gene/?term=56695
Pds5b	Pds5b	PDS5 cohesin associated factor B	100710	https://www.ncbi.nlm.nih.gov/gene/?term=100710
Onecut2	Onecut2	one cut domain, family member 2	225631	https://www.ncbi.nlm.nih.gov/gene/?term=225631
Pigc	Pigc	phosphatidylinositol glycan anchor biosynthesis, class C	67292	https://www.ncbi.nlm.nih.gov/gene/?term=67292
Nr2f6	Nr2f6	nuclear receptor subfamily 2, group F, member 6	13864	https://www.ncbi.nlm.nih.gov/gene/?term=13864
Csrp2	Csrp2	cysteine and glycine-rich protein 2	13008	https://www.ncbi.nlm.nih.gov/gene/?term=13008
Add1	Add1	adducin 1 (alpha)	11518	https://www.ncbi.nlm.nih.gov/gene/?term=11518
Tlk2	Tlk2	tousled-like kinase 2 (Arabidopsis)	24086	https://www.ncbi.nlm.nih.gov/gene/?term=24086
Ugcg	Ugcg	UDP-glucose ceramide glucosyltransferase	22234	https://www.ncbi.nlm.nih.gov/gene/?term=22234
Pard6b	Pard6b	par-6 family cell polarity regulator	58220	https://www.ncbi.nlm.nih.gov/gene/?term=58220

Biology Open • Supplementary information

		beta		
Zcchc24	Zcchc24	zinc finger, CCHC domain containing 24	71918	https://www.ncbi.nlm.nih.gov/gene/?term=71918
Apaf1	Apaf1	apoptotic peptidase activating factor 1	11783	https://www.ncbi.nlm.nih.gov/gene/?term=11783
Smad9	Smad9	SMAD family member 9	55994	https://www.ncbi.nlm.nih.gov/gene/?term=55994
Hoxa5	Hoxa5	homeobox A5	15402	https://www.ncbi.nlm.nih.gov/gene/?term=15402
Fam193b	Fam193b	family with sequence similarity 193, member B	212483	https://www.ncbi.nlm.nih.gov/gene/?term=212483
Herc3	Herc3	hect domain and RLD 3	73998	https://www.ncbi.nlm.nih.gov/gene/?term=73998
Ino80d	Ino80d	INO80 complex subunit D	227195	https://www.ncbi.nlm.nih.gov/gene/?term=227195
Tsc22d2	Tsc22d2	TSC22 domain family, member 2	72033	https://www.ncbi.nlm.nih.gov/gene/?term=72033
Plppr1	Plppr1	phospholipid phosphatase related 1	272031	https://www.ncbi.nlm.nih.gov/gene/?term=272031
Car10	Car10	carbonic anhydrase 10	72605	https://www.ncbi.nlm.nih.gov/gene/?term=72605
Adcy6	Adcy6	adenylate cyclase 6	11512	https://www.ncbi.nlm.nih.gov/gene/?term=11512
Slitrk1	Slitrk1	SLIT and NTRK-like family, member 1	76965	https://www.ncbi.nlm.nih.gov/gene/?term=76965
Itsn2	Itsn2	intersectin 2	20403	https://www.ncbi.nlm.nih.gov/gene/?term=20403
Mier3	Mier3	MIER family member 3	218613	https://www.ncbi.nlm.nih.gov/gene/?term=218613
Asph	Asph	aspartate-beta-hydroxylase	65973	https://www.ncbi.nlm.nih.gov/gene/?term=65973
Hsdl1	Hsdl1	hydroxysteroid dehydrogenase like 1	72552	https://www.ncbi.nlm.nih.gov/gene/?term=72552
Rara	Rara	retinoic acid receptor, alpha	19401	https://www.ncbi.nlm.nih.gov/gene/?term=19401
Abca1	Abca1	ATP-binding cassette, sub-family A (ABC1), member 1	11303	https://www.ncbi.nlm.nih.gov/gene/?term=11303
Sema7a	Sema7a	sema domain, immunoglobulin domain (Ig), and GPI membrane anchor, (semaphorin) 7A	20361	https://www.ncbi.nlm.nih.gov/gene/?term=20361
Klhl31	Klhl31	kelch-like 31	244923	https://www.ncbi.nlm.nih.gov/gene/?term=244923
2410004B18Rik	2410004B18Rik	RIKEN cDNA 2410004B18 gene	66421	https://www.ncbi.nlm.nih.gov/gene/?term=66421
Usp42	Usp42	ubiquitin specific peptidase 42	76800	https://www.ncbi.nlm.nih.gov/gene/?term=76800

Biology Open • Supplementary information

Pikfyve	Pikfyve	phosphoinositide kinase, FYVE type zinc finger containing	18711	https://www.ncbi.nlm.nih.gov/gene/?term=18711
St6galnac3	St6galnac3	ST6 (alpha-N-acetyl-neuraminyl-2,3-beta-galactosyl-1,3)-N-acetylgalactosaminide alpha-2,6-sialyltransferase 3	20447	https://www.ncbi.nlm.nih.gov/gene/?term=20447
Utp23	Utp23	UTP23 small subunit processome component	78581	https://www.ncbi.nlm.nih.gov/gene/?term=78581
Slc6a1	Slc6a1	solute carrier family 6 (neurotransmitter transporter, GABA), member 1	232333	https://www.ncbi.nlm.nih.gov/gene/?term=232333
Crebrf	Crebrf	CREB3 regulatory factor	77128	https://www.ncbi.nlm.nih.gov/gene/?term=77128
Cds1	Cds1	CDP-diacylglycerol synthase 1	74596	https://www.ncbi.nlm.nih.gov/gene/?term=74596
Zfhx3	Zfhx3	zinc finger homeobox 3	11906	https://www.ncbi.nlm.nih.gov/gene/?term=11906
Szrd1	Szrd1	SUZ RNA binding domain containing 1	213491	https://www.ncbi.nlm.nih.gov/gene/?term=213491
Nrk	Nrk	Nik related kinase	27206	https://www.ncbi.nlm.nih.gov/gene/?term=27206
Kmt5b	Kmt5b	lysine methyltransferase 5B	225888	https://www.ncbi.nlm.nih.gov/gene/?term=225888
Plxnd1	Plxnd1	plexin D1	67784	https://www.ncbi.nlm.nih.gov/gene/?term=67784
Ncald	Ncald	neurocalcin delta	52589	https://www.ncbi.nlm.nih.gov/gene/?term=52589
Kcnn3	Kcnn3	potassium intermediate/small conductance calcium-activated channel, subfamily N, member 3	140493	https://www.ncbi.nlm.nih.gov/gene/?term=140493
Kcnk2	Kcnk2	potassium channel, subfamily K, member 2	16526	https://www.ncbi.nlm.nih.gov/gene/?term=16526
Gns	Gns	glucosamine (N-acetyl)-6-sulfatase	75612	https://www.ncbi.nlm.nih.gov/gene/?term=75612
Ckap4	Ckap4	cytoskeleton-associated protein 4	216197	https://www.ncbi.nlm.nih.gov/gene/?term=216197
Gata3	Gata3	GATA binding protein 3	14462	https://www.ncbi.nlm.nih.gov/gene/?term=14462
Pgm2l1	Pgm2l1	phosphoglucomutase 2-like 1	70974	https://www.ncbi.nlm.nih.gov/gene/?term=70974

Arhgef37	Arhgef37	Rho guanine nucleotide exchange factor (GEF) 37	328967	https://www.ncbi.nlm.nih.gov/gene/?term=328967
Pdia5	Pdia5	protein disulfide isomerase associated 5	72599	https://www.ncbi.nlm.nih.gov/gene/?term=72599
Zbtb33	Zbtb33	zinc finger and BTB domain containing 33	56805	https://www.ncbi.nlm.nih.gov/gene/?term=56805
Sema6d	Sema6d	sema domain, transmembrane domain (TM), and cytoplasmic domain, (semaphorin) 6D	214968	https://www.ncbi.nlm.nih.gov/gene/?term=214968
Stk39	Stk39	serine/threonine kinase 39	53416	https://www.ncbi.nlm.nih.gov/gene/?term=53416
Retreg3	Retreg3	reticulophagy regulator family member 3	67998	https://www.ncbi.nlm.nih.gov/gene/?term=67998
Appbp2	Appbp2	amyloid beta precursor protein (cytoplasmic tail) binding protein 2	66884	https://www.ncbi.nlm.nih.gov/gene/?term=66884
Ercc6l	Ercc6l	excision repair cross-complementing rodent repair deficiency complementation group 6 like	236930	https://www.ncbi.nlm.nih.gov/gene/?term=236930
Slc35f4	Slc35f4	solute carrier family 35, member F4	75288	https://www.ncbi.nlm.nih.gov/gene/?term=75288
Zhx1	Zhx1	zinc fingers and homeoboxes 1	22770	https://www.ncbi.nlm.nih.gov/gene/?term=22770
Dot1l	Dot1l	DOT1-like, histone H3 methyltransferase (S. cerevisiae)	208266	https://www.ncbi.nlm.nih.gov/gene/?term=208266
Wnk2	Wnk2	WNK lysine deficient protein kinase 2	75607	https://www.ncbi.nlm.nih.gov/gene/?term=75607
Reps1	Reps1	RalBP1 associated Eps domain containing protein	19707	https://www.ncbi.nlm.nih.gov/gene/?term=19707
Ehf	Ehf	ets homologous factor	13661	https://www.ncbi.nlm.nih.gov/gene/?term=13661
Fbxw7	Fbxw7	F-box and WD-40 domain protein 7	50754	https://www.ncbi.nlm.nih.gov/gene/?term=50754
Spty2d1	Spty2d1	SPT2, Suppressor of Ty, domain containing 1 (S. cerevisiae)	101685	https://www.ncbi.nlm.nih.gov/gene/?term=101685
Zfp800	Zfp800	zinc finger protein 800	627049	https://www.ncbi.nlm.nih.gov/gene/?term=627049

Cipc	Cipc	CLOCK interacting protein, circadian	217732	https://www.ncbi.nlm.nih.gov/gene/?term=217732
Usp46	Usp46	ubiquitin specific peptidase 46	69727	https://www.ncbi.nlm.nih.gov/gene/?term=69727
Epb41l4a	Epb41l4a	erythrocyte membrane protein band 4.1 like 4a	13824	https://www.ncbi.nlm.nih.gov/gene/?term=13824
Gria3	Gria3	glutamate receptor, ionotropic, AMPA3 (alpha 3)	53623	https://www.ncbi.nlm.nih.gov/gene/?term=53623
Brpf3	Brpf3	bromodomain and PHD finger containing, 3	268936	https://www.ncbi.nlm.nih.gov/gene/?term=268936
Rnf139	Rnf139	ring finger protein 139	75841	https://www.ncbi.nlm.nih.gov/gene/?term=75841
Cog6	Cog6	component of oligomeric golgi complex 6	67542	https://www.ncbi.nlm.nih.gov/gene/?term=67542
Dnajc27	Dnajc27	DnaJ heat shock protein family (Hsp40) member C27	217378	https://www.ncbi.nlm.nih.gov/gene/?term=217378
Lpar6	Lpar6	lysophosphatidic acid receptor 6	67168	https://www.ncbi.nlm.nih.gov/gene/?term=67168
Fzd4	Fzd4	frizzled class receptor 4	14366	https://www.ncbi.nlm.nih.gov/gene/?term=14366
Omt2b	Omt2b	oocyte maturation, beta	382088	https://www.ncbi.nlm.nih.gov/gene/?term=382088
Pakap	Pakap	paralemmin A kinase anchor protein	677884	https://www.ncbi.nlm.nih.gov/gene/?term=677884
Twistnb	Twistnb	twist basic helix-loop-helix transcription factor 1 neighbor	28071	https://www.ncbi.nlm.nih.gov/gene/?term=28071
Cth	Cth	cystathionase (cystathionine gamma-lyase)	107869	https://www.ncbi.nlm.nih.gov/gene/?term=107869
Kdm3a	Kdm3a	lysine (K)-specific demethylase 3A	104263	https://www.ncbi.nlm.nih.gov/gene/?term=104263
Hbegf	Hbegf	heparin-binding EGF-like growth factor	15200	https://www.ncbi.nlm.nih.gov/gene/?term=15200
Fzd7	Fzd7	frizzled class receptor 7	14369	https://www.ncbi.nlm.nih.gov/gene/?term=14369
Gspt1	Gspt1	G1 to S phase transition 1	14852	https://www.ncbi.nlm.nih.gov/gene/?term=14852
Gata2	Gata2	GATA binding protein 2	14461	https://www.ncbi.nlm.nih.gov/gene/?term=14461
Hoxa10	Hoxa10	homeobox A10	15395	https://www.ncbi.nlm.nih.gov/gene/?term=15395

Biology Open • Supplementary information

Pdgfra	Pdgfra	platelet derived growth factor receptor, alpha polypeptide	18595	https://www.ncbi.nlm.nih.gov/gene/?term=18595
Pdzk1ip1	Pdzk1ip1	PDZK1 interacting protein 1	67182	https://www.ncbi.nlm.nih.gov/gene/?term=67182
Rps6kb1	Rps6kb1	ribosomal protein S6 kinase, polypeptide 1	72508	https://www.ncbi.nlm.nih.gov/gene/?term=72508
Fut9	Fut9	fucosyltransferase 9	14348	https://www.ncbi.nlm.nih.gov/gene/?term=14348
Csf1	Csf1	colony stimulating factor 1 (macrophage)	12977	https://www.ncbi.nlm.nih.gov/gene/?term=12977
Lpin1	Lpin1	lipin 1	14245	https://www.ncbi.nlm.nih.gov/gene/?term=14245
Matn3	Matn3	matrilin 3	17182	https://www.ncbi.nlm.nih.gov/gene/?term=17182
Eya1	Eya1	EYA transcriptional coactivator and phosphatase 1	14048	https://www.ncbi.nlm.nih.gov/gene/?term=14048
Nrbf2	Nrbf2	nuclear receptor binding factor 2	641340	https://www.ncbi.nlm.nih.gov/gene/?term=641340
Kat2b	Kat2b	K(lysine) acetyltransferase 2B	18519	https://www.ncbi.nlm.nih.gov/gene/?term=18519
Slc7a11	Slc7a11	solute carrier family 7 (cationic amino acid transporter, y+ system), member 11	26570	https://www.ncbi.nlm.nih.gov/gene/?term=26570
Pla2g6	Pla2g6	phospholipase A2, group VI	53357	https://www.ncbi.nlm.nih.gov/gene/?term=53357
Serpini1	Serpini1	serine (or cysteine) peptidase inhibitor, clade I, member 1	20713	https://www.ncbi.nlm.nih.gov/gene/?term=20713
Aqp11	Aqp11	aquaporin 11	66333	https://www.ncbi.nlm.nih.gov/gene/?term=66333
Golm1	Golm1	golgi membrane protein 1	105348	https://www.ncbi.nlm.nih.gov/gene/?term=105348
Paqr9	Paqr9	progestin and adipoQ receptor family member IX	75552	https://www.ncbi.nlm.nih.gov/gene/?term=75552
Galnt3	Galnt3	polypeptide N-acetylgalactosaminyltransferase 3	14425	https://www.ncbi.nlm.nih.gov/gene/?term=14425
Ccnc	Ccnc	cyclin C	51813	https://www.ncbi.nlm.nih.gov/gene/?term=51813
B4galt3	B4galt3	UDP-Gal:betaGlcNAc beta 1,4-galactosyltransferase, polypeptide 3	57370	https://www.ncbi.nlm.nih.gov/gene/?term=57370

Biology Open • Supplementary information

Ing5	Ing5	inhibitor of growth family, member 5	66262	https://www.ncbi.nlm.nih.gov/gene/?term=66262
Ube2n	Ube2n	ubiquitin-conjugating enzyme E2N	93765	https://www.ncbi.nlm.nih.gov/gene/?term=93765
Creb1	Creb1	cAMP responsive element binding protein 1	12912	https://www.ncbi.nlm.nih.gov/gene/?term=12912
Chka	Chka	choline kinase alpha	12660	https://www.ncbi.nlm.nih.gov/gene/?term=12660
Dcun1d4	Dcun1d4	DCN1, defective in cullin neddylation 1, domain containing 4 (S. cerevisiae)	100737	https://www.ncbi.nlm.nih.gov/gene/?term=100737
Aldh5a1	Aldh5a1	aldhehyde dehydrogenase family 5, subfamily A1	214579	https://www.ncbi.nlm.nih.gov/gene/?term=214579
Bmi1	Bmi1	Bmi1 polycomb ring finger oncogene	12151	https://www.ncbi.nlm.nih.gov/gene/?term=12151
Tnrc6b	Tnrc6b	trinucleotide repeat containing 6b	213988	https://www.ncbi.nlm.nih.gov/gene/?term=213988
Erlec1	Erlec1	endoplasmic reticulum lectin 1	66753	https://www.ncbi.nlm.nih.gov/gene/?term=66753
B3gnt7	B3gnt7	UDP-GlcNAc:betaGal beta-1,3-N-acetylglucosaminyltransferase 7	227327	https://www.ncbi.nlm.nih.gov/gene/?term=227327
Nabp1	Nabp1	nucleic acid binding protein 1	109019	https://www.ncbi.nlm.nih.gov/gene/?term=109019
Zkscan4	Zkscan4	zinc finger with KRAB and SCAN domains 4	544922	https://www.ncbi.nlm.nih.gov/gene/?term=544922
Phb	Phb	prohibitin	18673	https://www.ncbi.nlm.nih.gov/gene/?term=18673
Myt1	Myt1	myelin transcription factor 1	17932	https://www.ncbi.nlm.nih.gov/gene/?term=17932
Seh1l	Seh1l	SEH1-like (S. cerevisiae	72124	https://www.ncbi.nlm.nih.gov/gene/?term=72124
Gcc2	Gcc2	GRIP and coiled-coil domain containing 2	70297	https://www.ncbi.nlm.nih.gov/gene/?term=70297
Suco	Suco	SUN domain containing ossification factor	226551	https://www.ncbi.nlm.nih.gov/gene/?term=226551
4932438A13Rik	4932438A13Rik	RIKEN cDNA 4932438A13 gene	229227	https://www.ncbi.nlm.nih.gov/gene/?term=229227
Arhgap1	Arhgap1	Rho GTPase activating protein 1	228359	https://www.ncbi.nlm.nih.gov/gene/?term=228359
Ccdc28b	Ccdc28b	coiled coil domain containing 28B	66264	https://www.ncbi.nlm.nih.gov/gene/?term=66264
Tmem240	Tmem240	transmembrane protein 240	381582	https://www.ncbi.nlm.nih.gov/gene/?term=381582

Slc26a2	Slc26a2	solute carrier family 26 (sulfate transporter), member 2	13521	https://www.ncbi.nlm.nih.gov/gene/?term=13521
Gab1	Gab1	growth factor receptor bound protein 2-associated protein 1	14388	https://www.ncbi.nlm.nih.gov/gene/?term=14388
Nav2	Nav2	neuron navigator 2	78286	https://www.ncbi.nlm.nih.gov/gene/?term=78286
Tab3	Tab3	TGF-beta activated kinase 1/MAP3K7 binding protein 3	66724	https://www.ncbi.nlm.nih.gov/gene/?term=66724
Kcnj13	Kcnj13	potassium inwardly-rectifying channel, subfamily J, member 13	100040591	https://www.ncbi.nlm.nih.gov/gene/?term=100040
Wsb1	Wsb1	WD repeat and SOCS box-containing 1	78889	https://www.ncbi.nlm.nih.gov/gene/?term=78889
Grm5	Grm5	glutamate receptor, metabotropic 5	108071	https://www.ncbi.nlm.nih.gov/gene/?term=108071
Klhdc3	Klhdc3	kelch domain containing 3	71765	https://www.ncbi.nlm.nih.gov/gene/?term=71765
Kctd8	Kctd8	potassium channel tetramerisation domain containing 8	243043	https://www.ncbi.nlm.nih.gov/gene/?term=243043
Adora2b	Adora2b	adenosine A2b receptor	11541	https://www.ncbi.nlm.nih.gov/gene/?term=11541
Fam184a	Fam184a	family with sequence similarity 184, member A	75906	https://www.ncbi.nlm.nih.gov/gene/?term=75906
Cdh11	Cdh11	cadherin 11	12552	https://www.ncbi.nlm.nih.gov/gene/?term=12552
Mal2	Mal2	mal, T cell differentiation protein 2	105853	https://www.ncbi.nlm.nih.gov/gene/?term=105853
Ppif	Ppif	peptidylprolyl isomerase F (cyclophilin F)	105675	https://www.ncbi.nlm.nih.gov/gene/?term=105675
Ptgdr	Ptgdr	prostaglandin D receptor	19214	https://www.ncbi.nlm.nih.gov/gene/?term=19214
Vav2	Vav2	vav 2 oncogene	22325	https://www.ncbi.nlm.nih.gov/gene/?term=22325
Clcn5	Clcn5	chloride channel, voltage-sensitive 5	12728	https://www.ncbi.nlm.nih.gov/gene/?term=12728
Abhd17c	Abhd17c	abhydrolase domain containing 17C	70178	https://www.ncbi.nlm.nih.gov/gene/?term=70178
C2cd2	C2cd2	C2 calcium-dependent domain containing 2	207781	https://www.ncbi.nlm.nih.gov/gene/?term=207781
Acer2	Acer2	alkaline ceramidase 2	230379	https://www.ncbi.nlm.nih.gov/gene/?term=230379

Biology Open • Supplementary information

Alkal2	Alkal2	ALK and LTK ligand 2	100294583	https://www.ncbi.nlm.nih.gov/gene/?term=100294[illegible]
Limk1	Limk1	LIM-domain containing, protein kinase	16885	https://www.ncbi.nlm.nih.gov/gene/?term=16885
Cdip1	Cdip1	cell death inducing Trp53 target 1	66626	https://www.ncbi.nlm.nih.gov/gene/?term=66626
Larp4	Larp4	La ribonucleoprotein domain family, member 4	207214	https://www.ncbi.nlm.nih.gov/gene/?term=207214
Tmcc1	Tmcc1	transmembrane and coiled coil domains 1	330401	https://www.ncbi.nlm.nih.gov/gene/?term=330401
Runx1	Runx1	runt related transcription factor 1	12394	https://www.ncbi.nlm.nih.gov/gene/?term=12394
Plcl2	Plcl2	phospholipase C-like 2	224860	https://www.ncbi.nlm.nih.gov/gene/?term=224860
Mmd	Mmd	monocyte to macrophage differentiation-associated	67468	https://www.ncbi.nlm.nih.gov/gene/?term=67468
Unkl	Unkl	unkempt family like zinc finger	74154	https://www.ncbi.nlm.nih.gov/gene/?term=74154
Nemp2	Nemp2	nuclear envelope integral membrane protein 2	227094	https://www.ncbi.nlm.nih.gov/gene/?term=227094
Edrf1	Edrf1	erythroid differentiation regulatory factor 1	214764	https://www.ncbi.nlm.nih.gov/gene/?term=214764
Raph1	Raph1	Ras association (RalGDS/AF-6) and pleckstrin homology domains 1	77300	https://www.ncbi.nlm.nih.gov/gene/?term=77300
Lifr	Lifr	LIF receptor alpha	16880	https://www.ncbi.nlm.nih.gov/gene/?term=16880
Ppp1cc	Ppp1cc	protein phosphatase 1 catalytic subunit gamma	19047	https://www.ncbi.nlm.nih.gov/gene/?term=19047
Sema6a	Sema6a	sema domain, transmembrane domain (TM), and cytoplasmic domain, (semaphorin) 6A	20358	https://www.ncbi.nlm.nih.gov/gene/?term=20358
Mstn	Mstn	myostatin	17700	https://www.ncbi.nlm.nih.gov/gene/?term=17700
Mbtd1	Mbtd1	mbt domain containing 1	103537	https://www.ncbi.nlm.nih.gov/gene/?term=103537
E2f7	E2f7	E2F transcription factor 7	52679	https://www.ncbi.nlm.nih.gov/gene/?term=52679
Tnrc18	Tnrc18	trinucleotide repeat containing 18	231861	https://www.ncbi.nlm.nih.gov/gene/?term=231861

Biology Open • Supplementary information

Agrn	Agrn	agrin	11603	https://www.ncbi.nlm.nih.gov/gene/?term=11603
En2	En2	engrailed 2	13799	https://www.ncbi.nlm.nih.gov/gene/?term=13799
Edem3	Edem3	ER degradation enhancer, mannosidase alpha-like 3	66967	https://www.ncbi.nlm.nih.gov/gene/?term=66967
Nrp2	Nrp2	neuropilin 2	18187	https://www.ncbi.nlm.nih.gov/gene/?term=18187
Tor1b	Tor1b	torsin family 1, member B	30934	https://www.ncbi.nlm.nih.gov/gene/?term=30934
Btg2	Btg2	B cell translocation gene 2, anti-proliferative	12227	https://www.ncbi.nlm.nih.gov/gene/?term=12227
Mdm4	Mdm4	transformed mouse 3T3 cell double minute 4	17248	https://www.ncbi.nlm.nih.gov/gene/?term=17248
Sowaha	Sowaha	sosondowah ankyrin repeat domain family member A	237761	https://www.ncbi.nlm.nih.gov/gene/?term=237761
Arfgef1	Arfgef1	ADP-ribosylation factor guanine nucleotide-exchange factor 1(brefeldin A-inhibited)	211673	https://www.ncbi.nlm.nih.gov/gene/?term=211673
Insm2	Insm2	insulinoma-associated 2	56856	https://www.ncbi.nlm.nih.gov/gene/?term=56856
Ogn	Ogn	osteoglycin	18295	https://www.ncbi.nlm.nih.gov/gene/?term=18295
Slc35f1	Slc35f1	solute carrier family 35, member F1	215085	https://www.ncbi.nlm.nih.gov/gene/?term=215085
Zfp143	Zfp143	zinc finger protein 143	20841	https://www.ncbi.nlm.nih.gov/gene/?term=20841
Plekhh1	Plekhh1	pleckstrin homology domain containing, family H (with MyTH4 domain) member 1	211945	https://www.ncbi.nlm.nih.gov/gene/?term=211945
Trappc8	Trappc8	trafficking protein particle complex 8	75964	https://www.ncbi.nlm.nih.gov/gene/?term=75964
Ppara	Ppara	peroxisome proliferator activated receptor alpha	19013	https://www.ncbi.nlm.nih.gov/gene/?term=19013
Colgalt2	Colgalt2	collagen beta(1-O)galactosyltransferase 2	269132	https://www.ncbi.nlm.nih.gov/gene/?term=269132
Ccnk	Ccnk	cyclin K	12454	https://www.ncbi.nlm.nih.gov/gene/?term=12454
Cecr2	Cecr2	CECR2, histone acetyl-lysine reader	330409	https://www.ncbi.nlm.nih.gov/gene/?term=330409

Aff4	Aff4	AF4/FMR2 family, member 4	93736	https://www.ncbi.nlm.nih.gov/gene/?term=93736
Dnajc13	Dnajc13	DnaJ heat shock protein family (Hsp40) member C13	235567	https://www.ncbi.nlm.nih.gov/gene/?term=235567
Plk2	Plk2	polo like kinase 2	20620	https://www.ncbi.nlm.nih.gov/gene/?term=20620
Sox11	Sox11	SRY (sex determining region Y)-box 11	20666	https://www.ncbi.nlm.nih.gov/gene/?term=20666
Ankrd42	Ankrd42	ankyrin repeat domain 42	73845	https://www.ncbi.nlm.nih.gov/gene/?term=73845
Ampd3	Ampd3	adenosine monophosphate deaminase 3	11717	https://www.ncbi.nlm.nih.gov/gene/?term=11717
Pdik1l	Pdik1l	PDLIM1 interacting kinase 1 like	230809	https://www.ncbi.nlm.nih.gov/gene/?term=230809
Smim4	Smim4	small integral membrane protein 4	66487	https://www.ncbi.nlm.nih.gov/gene/?term=66487
Strap	Strap	serine/threonine kinase receptor associated protein	20901	https://www.ncbi.nlm.nih.gov/gene/?term=20901
Mospd2	Mospd2	motile sperm domain containing 2	76763	https://www.ncbi.nlm.nih.gov/gene/?term=76763
Rdh10	Rdh10	retinol dehydrogenase 10 (all-trans)	98711	https://www.ncbi.nlm.nih.gov/gene/?term=98711
Zfand6	Zfand6	zinc finger, AN1-type domain 6	65098	https://www.ncbi.nlm.nih.gov/gene/?term=65098
Rusc2	Rusc2	RUN and SH3 domain containing 2	100213	https://www.ncbi.nlm.nih.gov/gene/?term=100213
Nbr1	Nbr1	NBR1, autophagy cargo receptor	17966	https://www.ncbi.nlm.nih.gov/gene/?term=17966
Dek	Dek	DEK oncogene (DNA binding)	110052	https://www.ncbi.nlm.nih.gov/gene/?term=110052
Fli1	Fli1	Friend leukemia integration 1	14247	https://www.ncbi.nlm.nih.gov/gene/?term=14247
Cdk17	Cdk17	cyclin-dependent kinase 17	237459	https://www.ncbi.nlm.nih.gov/gene/?term=237459
Slc30a4	Slc30a4	solute carrier family 30 (zinc transporter), member 4	22785	https://www.ncbi.nlm.nih.gov/gene/?term=22785
Eloc	Eloc	elongin C	67923	https://www.ncbi.nlm.nih.gov/gene/?term=67923
Rims2	Rims2	regulating synaptic membrane exocytosis 2	116838	https://www.ncbi.nlm.nih.gov/gene/?term=116838
Xkr4	Xkr4	X-linked Kx blood group related 4	497097	https://www.ncbi.nlm.nih.gov/gene/?term=497097
Wapl	Wapl	WAPL cohesin release factor	218914	https://www.ncbi.nlm.nih.gov/gene/?term=218914
Mosmo	Mosmo	modulator of smoothened	233812	https://www.ncbi.nlm.nih.gov/gene/?term=233812

Biology Open • Supplementary information

Cntn1	Cntn1	contactin 1	12805	https://www.ncbi.nlm.nih.gov/gene/?term=12805
Pfpl	Pfpl	pore forming protein-like	56093	https://www.ncbi.nlm.nih.gov/gene/?term=56093
Lhfp	Lhfp	lipoma HMGIC fusion partner	108927	https://www.ncbi.nlm.nih.gov/gene/?term=108927
Slit2	Slit2	slit guidance ligand 2	20563	https://www.ncbi.nlm.nih.gov/gene/?term=20563
Rab11fip2	Rab11fip2	RAB11 family interacting protein 2 (class I)	74998	https://www.ncbi.nlm.nih.gov/gene/?term=74998
Pom121l12	Pom121l12	POM121 membrane glycoprotein-like 12	432536	https://www.ncbi.nlm.nih.gov/gene/?term=432536
Pcmtd1	Pcmtd1	protein-L-isoaspartate (D-aspartate) O-methyltransferase domain containing 1	319263	https://www.ncbi.nlm.nih.gov/gene/?term=319263
Dlc1	Dlc1	deleted in liver cancer 1	50768	https://www.ncbi.nlm.nih.gov/gene/?term=50768
Arhgap20	Arhgap20	Rho GTPase activating protein 20	244867	https://www.ncbi.nlm.nih.gov/gene/?term=244867
Hmbox1	Hmbox1	homeobox containing 1	219150	https://www.ncbi.nlm.nih.gov/gene/?term=219150
Thap1	Thap1	THAP domain containing, apoptosis associated protein 1	73754	https://www.ncbi.nlm.nih.gov/gene/?term=73754
Qk	Qk	quaking	19317	https://www.ncbi.nlm.nih.gov/gene/?term=19317
Atp11c	Atp11c	ATPase, class VI, type 11C	320940	https://www.ncbi.nlm.nih.gov/gene/?term=320940
Zfp131	Zfp131	zinc finger protein 131	72465	https://www.ncbi.nlm.nih.gov/gene/?term=72465
Fez2	Fez2	fasciculation and elongation protein zeta 2 (zygin II)	225020	https://www.ncbi.nlm.nih.gov/gene/?term=225020
Sulf1	Sulf1	sulfatase 1	240725	https://www.ncbi.nlm.nih.gov/gene/?term=240725
Cnot6	Cnot6	CCR4-NOT transcription complex, subunit 6	104625	https://www.ncbi.nlm.nih.gov/gene/?term=104625
Golga1	Golga1	golgi autoantigen, golgin subfamily a, 1	76899	https://www.ncbi.nlm.nih.gov/gene/?term=76899
Elmod2	Elmod2	ELMO/CED-12 domain containing 2	244548	https://www.ncbi.nlm.nih.gov/gene/?term=244548
Rap1b	Rap1b	RAS related protein 1b	215449	https://www.ncbi.nlm.nih.gov/gene/?term=215449
Rab21	Rab21	RAB21, member RAS oncogene	216344	https://www.ncbi.nlm.nih.gov/gene/?term=216344

		family		
Phf11d	Phf11d	PHD finger protein 11D	219132	https://www.ncbi.nlm.nih.gov/gene/?term=219132
Khdrbs1	Khdrbs1	KH domain containing, RNA binding, signal transduction associated 1	20218	https://www.ncbi.nlm.nih.gov/gene/?term=20218
Chrdl1	Chrdl1	chordin-like 1	83453	https://www.ncbi.nlm.nih.gov/gene/?term=83453
Zfp711	Zfp711	zinc finger protein 711	245595	https://www.ncbi.nlm.nih.gov/gene/?term=245595
Rnd3	Rnd3	Rho family GTPase 3	74194	https://www.ncbi.nlm.nih.gov/gene/?term=74194
Immp2l	Immp2l	IMP2 inner mitochondrial membrane peptidase-like (S. cerevisiae)	93757	https://www.ncbi.nlm.nih.gov/gene/?term=93757
Fbxo33	Fbxo33	F-box protein 33	70611	https://www.ncbi.nlm.nih.gov/gene/?term=70611
Tfap2a	Tfap2a	transcription factor AP-2, alpha	21418	https://www.ncbi.nlm.nih.gov/gene/?term=21418
Ap1s2	Ap1s2	adaptor-related protein complex 1, sigma 2 subunit	108012	https://www.ncbi.nlm.nih.gov/gene/?term=108012
Spryd7	Spryd7	SPRY domain containing 7	66674	https://www.ncbi.nlm.nih.gov/gene/?term=66674
Tjp1	Tjp1	tight junction protein 1	21872	https://www.ncbi.nlm.nih.gov/gene/?term=21872
Ppp1r9b	Ppp1r9b	protein phosphatase 1, regulatory subunit 9B	217124	https://www.ncbi.nlm.nih.gov/gene/?term=217124
Gpr161	Gpr161	G protein-coupled receptor 161	240888	https://www.ncbi.nlm.nih.gov/gene/?term=240888
Fndc3b	Fndc3b	fibronectin type III domain containing 3B	72007	https://www.ncbi.nlm.nih.gov/gene/?term=72007
Hdhd2	Hdhd2	haloacid dehalogenase-like hydrolase domain containing 2	76987	https://www.ncbi.nlm.nih.gov/gene/?term=76987
Cfl2	Cfl2	cofilin 2, muscle	12632	https://www.ncbi.nlm.nih.gov/gene/?term=12632
Ywhag	Ywhag	tyrosine 3-monooxygenase/tryptophan 5-monooxygenase activation protein, gamma polypeptide	22628	https://www.ncbi.nlm.nih.gov/gene/?term=22628
Desi2	Desi2	desumoylating isopeptidase 2	78825	https://www.ncbi.nlm.nih.gov/gene/?term=78825
Dennd5a	Dennd5a	DENN/MADD domain containing 5A	19347	https://www.ncbi.nlm.nih.gov/gene/?term=19347

Gpm6a	Gpm6a	glycoprotein m6a	234267	https://www.ncbi.nlm.nih.gov/gene/?term=234267
Adamts6	Adamts6	a disintegrin-like and metallopeptidase (reprolysin type) with thrombospondin type 1 motif, 6	108154	https://www.ncbi.nlm.nih.gov/gene/?term=108154
Atxn1	Atxn1	ataxin 1	20238	https://www.ncbi.nlm.nih.gov/gene/?term=20238
Osbpl11	Osbpl11	oxysterol binding protein-like 11	106326	https://www.ncbi.nlm.nih.gov/gene/?term=106326
Sec23a	Sec23a	SEC23 homolog A, COPII coat complex component	20334	https://www.ncbi.nlm.nih.gov/gene/?term=20334
Ptpn21	Ptpn21	protein tyrosine phosphatase, non-receptor type 21	24000	https://www.ncbi.nlm.nih.gov/gene/?term=24000
Hipk3	Hipk3	homeodomain interacting protein kinase 3	15259	https://www.ncbi.nlm.nih.gov/gene/?term=15259
Fignl2	Fignl2	fidgetin-like 2	668225	https://www.ncbi.nlm.nih.gov/gene/?term=668225
Pi4kb	Pi4kb	phosphatidylinositol 4-kinase beta	107650	https://www.ncbi.nlm.nih.gov/gene/?term=107650
Pik3cb	Pik3cb	phosphatidylinositol-4,5-bisphosphate 3-kinase catalytic subunit beta	74769	https://www.ncbi.nlm.nih.gov/gene/?term=74769
Lrp1b	Lrp1b	low density lipoprotein-related protein 1B (deleted in tumors)	94217	https://www.ncbi.nlm.nih.gov/gene/?term=94217
Gpatch8	Gpatch8	G patch domain containing 8	237943	https://www.ncbi.nlm.nih.gov/gene/?term=237943
Nr3c1	Nr3c1	nuclear receptor subfamily 3, group C, member 1	14815	https://www.ncbi.nlm.nih.gov/gene/?term=14815
Erg	Erg	ETS transcription factor	13876	https://www.ncbi.nlm.nih.gov/gene/?term=13876
Vash2	Vash2	vasohibin 2	226841	https://www.ncbi.nlm.nih.gov/gene/?term=226841
Synj1	Synj1	synaptojanin 1	104015	https://www.ncbi.nlm.nih.gov/gene/?term=104015
Evi5	Evi5	ecotropic viral integration site 5	14020	https://www.ncbi.nlm.nih.gov/gene/?term=14020
Elmod1	Elmod1	ELMO/CED-12 domain containing 1	270162	https://www.ncbi.nlm.nih.gov/gene/?term=270162
Syncrip	Syncrip	synaptotagmin binding, cytoplasmic RNA interacting protein	56403	https://www.ncbi.nlm.nih.gov/gene/?term=56403

Biology Open • Supplementary information

Tubb5	Tubb5	tubulin, beta 5 class I	22154	https://www.ncbi.nlm.nih.gov/gene/?term=22154
Ppm1f	Ppm1f	protein phosphatase 1F (PP2C domain containing)	68606	https://www.ncbi.nlm.nih.gov/gene/?term=68606
Dpy19l1	Dpy19l1	dpy-19-like 1 (C. elegans)	244745	https://www.ncbi.nlm.nih.gov/gene/?term=244745
Mprip	Mprip	myosin phosphatase Rho interacting protein	26936	https://www.ncbi.nlm.nih.gov/gene/?term=26936
Ankrd45	Ankrd45	ankyrin repeat domain 45	73844	https://www.ncbi.nlm.nih.gov/gene/?term=73844
Plcl1	Plcl1	phospholipase C-like 1	227120	https://www.ncbi.nlm.nih.gov/gene/?term=227120
Dennd5b	Dennd5b	DENN/MADD domain containing 5B	320560	https://www.ncbi.nlm.nih.gov/gene/?term=320560
Bag5	Bag5	BCL2-associated athanogene 5	70369	https://www.ncbi.nlm.nih.gov/gene/?term=70369
Med13	Med13	mediator complex subunit 13	327987	https://www.ncbi.nlm.nih.gov/gene/?term=327987
Rbsn	Rbsn	rabenosyn, RAB effector	78287	https://www.ncbi.nlm.nih.gov/gene/?term=78287
Prr11	Prr11	proline rich 11	270906	https://www.ncbi.nlm.nih.gov/gene/?term=270906
Foxp1	Foxp1	forkhead box P1	108655	https://www.ncbi.nlm.nih.gov/gene/?term=108655
Sox5	Sox5	SRY (sex determining region Y)-box 5	20678	https://www.ncbi.nlm.nih.gov/gene/?term=20678
Wipf1	Wipf1	WAS/WASL interacting protein family, member 1	215280	https://www.ncbi.nlm.nih.gov/gene/?term=215280
Vash1	Vash1	vasohibin 1	238328	https://www.ncbi.nlm.nih.gov/gene/?term=238328
Nrf1	Nrf1	nuclear respiratory factor 1	18181	https://www.ncbi.nlm.nih.gov/gene/?term=18181
Cdh20	Cdh20	cadherin 20	23836	https://www.ncbi.nlm.nih.gov/gene/?term=23836
Ago2	Ago2	argonaute RISC catalytic subunit 2	239528	https://www.ncbi.nlm.nih.gov/gene/?term=239528
St6galnac5	St6galnac5	ST6 (alpha-N-acetyl-neuraminyl-2,3-beta-galactosyl-1,3)-N-acetylgalactosaminide alpha-2,6-sialyltransferase 5	26938	https://www.ncbi.nlm.nih.gov/gene/?term=26938
Jun	Jun	jun proto-oncogene	16476	https://www.ncbi.nlm.nih.gov/gene/?term=16476
Ypel2	Ypel2	yippee like 2	77864	https://www.ncbi.nlm.nih.gov/gene/?term=77864
Nfia	Nfia	nuclear factor I/A	18027	https://www.ncbi.nlm.nih.gov/gene/?term=18027
Zeb2	Zeb2	zinc finger E-box binding homeobox	24136	https://www.ncbi.nlm.nih.gov/gene/?term=24136

		2		
Rasa2	Rasa2	RAS p21 protein activator 2	114713	https://www.ncbi.nlm.nih.gov/gene/?term=114713
Cd302	Cd302	CD302 antigen	66205	https://www.ncbi.nlm.nih.gov/gene/?term=66205
Phf21b	Phf21b	PHD finger protein 21B	271305	https://www.ncbi.nlm.nih.gov/gene/?term=271305
Lats2	Lats2	large tumor suppressor 2	50523	https://www.ncbi.nlm.nih.gov/gene/?term=50523
Slc14a1	Slc14a1	solute carrier family 14 (urea transporter), member 1	108052	https://www.ncbi.nlm.nih.gov/gene/?term=108052
Esrrg	Esrrg	estrogen-related receptor gamma	26381	https://www.ncbi.nlm.nih.gov/gene/?term=26381
Map2	Map2	microtubule-associated protein 2	17756	https://www.ncbi.nlm.nih.gov/gene/?term=17756
Ric1	Ric1	RAB6A GEF complex partner 1	226089	https://www.ncbi.nlm.nih.gov/gene/?term=226089
Tbc1d12	Tbc1d12	TBC1D12: TBC1 domain family, member 12	209478	https://www.ncbi.nlm.nih.gov/gene/?term=209478
Egln1	Egln1	egl-9 family hypoxia-inducible factor 1	112405	https://www.ncbi.nlm.nih.gov/gene/?term=112405
Crtap	Crtap	cartilage associated protein	56693	https://www.ncbi.nlm.nih.gov/gene/?term=56693
Ddx3y	Ddx3y	DEAD (Asp-Glu-Ala-Asp) box polypeptide 3, Y-linked	26900	https://www.ncbi.nlm.nih.gov/gene/?term=26900
Uba6	Uba6	ubiquitin-like modifier activating enzyme 6	231380	https://www.ncbi.nlm.nih.gov/gene/?term=231380
Nova1	Nova1	neuro-oncological ventral antigen 1	664883	https://www.ncbi.nlm.nih.gov/gene/?term=664883
Fhl1	Fhl1	four and a half LIM domains 1	14199	https://www.ncbi.nlm.nih.gov/gene/?term=14199
Crkl	Crkl	v-crk avian sarcoma virus CT10 oncogene homolog-like	12929	https://www.ncbi.nlm.nih.gov/gene/?term=12929
Ptpn12	Ptpn12	protein tyrosine phosphatase, non-receptor type 12	19248	https://www.ncbi.nlm.nih.gov/gene/?term=19248
Asap1	Asap1	ArfGAP with SH3 domain, ankyrin repeat and PH domain1	13196	https://www.ncbi.nlm.nih.gov/gene/?term=13196
Tbk1	Tbk1	TANK-binding kinase 1	56480	https://www.ncbi.nlm.nih.gov/gene/?term=56480
Hook3	Hook3	hook microtubule tethering protein 3	320191	https://www.ncbi.nlm.nih.gov/gene/?term=320191

Tob1	Tob1	transducer of ErbB-2.1	22057	https://www.ncbi.nlm.nih.gov/gene/?term=22057
Slc38a2	Slc38a2	solute carrier family 38, member 2	67760	https://www.ncbi.nlm.nih.gov/gene/?term=67760
Amfr	Amfr	autocrine motility factor receptor	23802	https://www.ncbi.nlm.nih.gov/gene/?term=23802
Golga7	Golga7	golgi autoantigen, golgin subfamily a, 7	57437	https://www.ncbi.nlm.nih.gov/gene/?term=57437
Aff3	Aff3	AF4/FMR2 family, member 3	16764	https://www.ncbi.nlm.nih.gov/gene/?term=16764
Slc35a2	Slc35a2	solute carrier family 35 (UDP-galactose transporter), member A2	22232	https://www.ncbi.nlm.nih.gov/gene/?term=22232
Trim33	Trim33	tripartite motif-containing 33	94093	https://www.ncbi.nlm.nih.gov/gene/?term=94093
Errfi1	Errfi1	ERBB receptor feedback inhibitor 1	74155	https://www.ncbi.nlm.nih.gov/gene/?term=74155
Msn	Msn	moesin	17698	https://www.ncbi.nlm.nih.gov/gene/?term=17698
Zeb1	Zeb1	zinc finger E-box binding homeobox 1	21417	https://www.ncbi.nlm.nih.gov/gene/?term=21417
Jazf1	Jazf1	JAZF zinc finger 1	231986	https://www.ncbi.nlm.nih.gov/gene/?term=231986
Rap2c	Rap2c	RAP2C, member of RAS oncogene family	72065	https://www.ncbi.nlm.nih.gov/gene/?term=72065
Rnf169	Rnf169	ring finger protein 169	108937	https://www.ncbi.nlm.nih.gov/gene/?term=108937
Phf11b	Phf11b	PHD finger protein 11B	236451	https://www.ncbi.nlm.nih.gov/gene/?term=236451
Tent4b	Tent4b	terminal nucleotidyltransferase 4B	214627	https://www.ncbi.nlm.nih.gov/gene/?term=214627
Spag9	Spag9	sperm associated antigen 9	70834	https://www.ncbi.nlm.nih.gov/gene/?term=70834
Unc5d	Unc5d	unc-5 netrin receptor D	210801	https://www.ncbi.nlm.nih.gov/gene/?term=210801
2700081O15Rik	2700081O15Rik	RIKEN cDNA 2700081O15 gene	108899	https://www.ncbi.nlm.nih.gov/gene/?term=108899
Agfg1	Agfg1	ArfGAP with FG repeats 1	15463	https://www.ncbi.nlm.nih.gov/gene/?term=15463
Ccnyl1	Ccnyl1	cyclin Y-like 1	227210	https://www.ncbi.nlm.nih.gov/gene/?term=227210
Bap1	Bap1	Brca1 associated protein 1	104416	https://www.ncbi.nlm.nih.gov/gene/?term=104416
Eps8	Eps8	epidermal growth factor receptor pathway substrate 8	13860	https://www.ncbi.nlm.nih.gov/gene/?term=13860
Igsf10	Igsf10	immunoglobulin superfamily, member 10	242050	https://www.ncbi.nlm.nih.gov/gene/?term=242050

Rbfox2	Rbfox2	RNA binding protein, fox-1 homolog (C. elegans) 2	93686	https://www.ncbi.nlm.nih.gov/gene/?term=93686
Scd1	Scd1	stearoyl-Coenzyme A desaturase 1	20249	https://www.ncbi.nlm.nih.gov/gene/?term=20249
Pkd1	Pkd1	polycystin 1, transient receptor poteintial channel interacting	18763	https://www.ncbi.nlm.nih.gov/gene/?term=18763
Mboat2	Mboat2	membrane bound O-acyltransferase domain containing 2	67216	https://www.ncbi.nlm.nih.gov/gene/?term=67216
Ano5	Ano5	anoctamin 5	233246	https://www.ncbi.nlm.nih.gov/gene/?term=233246
Ranbp9	Ranbp9	RAN binding protein 9	56705	https://www.ncbi.nlm.nih.gov/gene/?term=56705
Arih1	Arih1	ariadne RBR E3 ubiquitin protein ligase 1	23806	https://www.ncbi.nlm.nih.gov/gene/?term=23806
Clasp1	Clasp1	CLIP associating protein 1	76707	https://www.ncbi.nlm.nih.gov/gene/?term=76707
Mindy2	Mindy2	MINDY lysine 48 deubiquitinase 2	235461	https://www.ncbi.nlm.nih.gov/gene/?term=235461
Mgat2	Mgat2	mannoside acetylglucosaminyltransferase 2	217664	https://www.ncbi.nlm.nih.gov/gene/?term=217664
1810013L24Rik	1810013L24Rik	RIKEN cDNA 1810013L24 gene	69053	https://www.ncbi.nlm.nih.gov/gene/?term=69053
Hnrnpf	Hnrnpf	heterogeneous nuclear ribonucleoprotein F	98758	https://www.ncbi.nlm.nih.gov/gene/?term=98758
Lpar1	Lpar1	lysophosphatidic acid receptor 1	14745	https://www.ncbi.nlm.nih.gov/gene/?term=14745
Oosp3	Oosp3	oocyte secreted protein 3	225923	https://www.ncbi.nlm.nih.gov/gene/?term=225923
Gucy1a1	Gucy1a1	guanylate cyclase 1, soluble, alpha 1	60596	https://www.ncbi.nlm.nih.gov/gene/?term=60596
Mybl1	Mybl1	myeloblastosis oncogene-like 1	17864	https://www.ncbi.nlm.nih.gov/gene/?term=17864
Ntng1	Ntng1	netrin G1	80883	https://www.ncbi.nlm.nih.gov/gene/?term=80883
Dusp3	Dusp3	dual specificity phosphatase 3 (vaccinia virus phosphatase VH1-related)	72349	https://www.ncbi.nlm.nih.gov/gene/?term=72349
Mbnl1	Mbnl1	muscleblind like splicing factor 1	56758	https://www.ncbi.nlm.nih.gov/gene/?term=56758
Arfgef3	Arfgef3	ARFGEF family member 3	215821	https://www.ncbi.nlm.nih.gov/gene/?term=215821
Rnf145	Rnf145	ring finger protein 145	74315	https://www.ncbi.nlm.nih.gov/gene/?term=74315

Biology Open • Supplementary information

Myt1l	Myt1l	myelin transcription factor 1-like	17933	https://www.ncbi.nlm.nih.gov/gene/?term=17933
Kif3a	Kif3a	kinesin family member 3A	16568	https://www.ncbi.nlm.nih.gov/gene/?term=16568
Dr1	Dr1	down-regulator of transcription 1	13486	https://www.ncbi.nlm.nih.gov/gene/?term=13486
Cyth3	Cyth3	cytohesin 3	19159	https://www.ncbi.nlm.nih.gov/gene/?term=19159
Pcdh9	Pcdh9	protocadherin 9	211712	https://www.ncbi.nlm.nih.gov/gene/?term=211712
Dmwd	Dmwd	dystrophia myotonica-containing WD repeat motif	13401	https://www.ncbi.nlm.nih.gov/gene/?term=13401
Fbxl2	Fbxl2	F-box and leucine-rich repeat protein 2	72179	https://www.ncbi.nlm.nih.gov/gene/?term=72179
Ulk2	Ulk2	unc-51 like kinase 2	29869	https://www.ncbi.nlm.nih.gov/gene/?term=29869
Cyp26b1	Cyp26b1	cytochrome P450, family 26, subfamily b, polypeptide 1	232174	https://www.ncbi.nlm.nih.gov/gene/?term=232174
Ccne2	Ccne2	cyclin E2	12448	https://www.ncbi.nlm.nih.gov/gene/?term=12448
Myh10	Myh10	myosin, heavy polypeptide 10, non-muscle	77579	https://www.ncbi.nlm.nih.gov/gene/?term=77579
Gpc2	Gpc2	glypican 2 (cerebroglycan)	71951	https://www.ncbi.nlm.nih.gov/gene/?term=71951
Simc1	Simc1	SUMO-interacting motifs containing 1	319719	https://www.ncbi.nlm.nih.gov/gene/?term=319719
Sacm1l	Sacm1l	SAC1 suppressor of actin mutations 1-like (yeast)	83493	https://www.ncbi.nlm.nih.gov/gene/?term=83493
Klhl28	Klhl28	kelch-like 28	66689	https://www.ncbi.nlm.nih.gov/gene/?term=66689
Osbpl6	Osbpl6	oxysterol binding protein-like 6	99031	https://www.ncbi.nlm.nih.gov/gene/?term=99031
Yaf2	Yaf2	YY1 associated factor 2	67057	https://www.ncbi.nlm.nih.gov/gene/?term=67057
Hs2st1	Hs2st1	heparan sulfate 2-O-sulfotransferase 1	23908	https://www.ncbi.nlm.nih.gov/gene/?term=23908
Myadm	Myadm	myeloid-associated differentiation marker	50918	https://www.ncbi.nlm.nih.gov/gene/?term=50918
Zfr	Zfr	zinc finger RNA binding protein	22763	https://www.ncbi.nlm.nih.gov/gene/?term=22763
Epn1	Epn1	epsin 1	13854	https://www.ncbi.nlm.nih.gov/gene/?term=13854

Biology Open • Supplementary information

Mindy3	Mindy3	MINDY lysine 48 deubiquitinase 3	66960	https://www.ncbi.nlm.nih.gov/gene/?term=66960
Rsad2	Rsad2	radical S-adenosyl methionine domain containing 2	58185	https://www.ncbi.nlm.nih.gov/gene/?term=58185
Cryz	Cryz	crystallin, zeta	12972	https://www.ncbi.nlm.nih.gov/gene/?term=12972
Ikzf2	Ikzf2	IKAROS family zinc finger 2	22779	https://www.ncbi.nlm.nih.gov/gene/?term=22779
Dcp2	Dcp2	decapping mRNA 2	70640	https://www.ncbi.nlm.nih.gov/gene/?term=70640
Crlf3	Crlf3	cytokine receptor-like factor 3	54394	https://www.ncbi.nlm.nih.gov/gene/?term=54394
Rnft1	Rnft1	ring finger protein, transmembrane 1	76892	https://www.ncbi.nlm.nih.gov/gene/?term=76892
Dstyk	Dstyk	dual serine/threonine and tyrosine protein kinase	213452	https://www.ncbi.nlm.nih.gov/gene/?term=213452
Tcf12	Tcf12	transcription factor 12	21406	https://www.ncbi.nlm.nih.gov/gene/?term=21406
Ube3a	Ube3a	ubiquitin protein ligase E3A	22215	https://www.ncbi.nlm.nih.gov/gene/?term=22215
Ythdf2	Ythdf2	YTH N6-methyladenosine RNA binding protein 2	213541	https://www.ncbi.nlm.nih.gov/gene/?term=213541
St3gal3	St3gal3	ST3 beta-galactoside alpha-2,3-sialyltransferase 3	20441	https://www.ncbi.nlm.nih.gov/gene/?term=20441
Iqschfp	Iqschfp	Iqcj and Schip1 fusion protein	100505386	https://www.ncbi.nlm.nih.gov/gene/?term=100505
4921524J17Rik	4921524J17Rik	RIKEN cDNA 4921524J17 gene	66714	https://www.ncbi.nlm.nih.gov/gene/?term=66714
Supt6	Supt6	suppressor of Ty 6	20926	https://www.ncbi.nlm.nih.gov/gene/?term=20926
Tgfb2	Tgfb2	transforming growth factor, beta 2	21808	https://www.ncbi.nlm.nih.gov/gene/?term=21808
Mrpl17	Mrpl17	mitochondrial ribosomal protein L17	27397	https://www.ncbi.nlm.nih.gov/gene/?term=27397
Pgrmc2	Pgrmc2	progesterone receptor membrane component 2	70804	https://www.ncbi.nlm.nih.gov/gene/?term=70804
Prkacb	Prkacb	protein kinase, cAMP dependent, catalytic, beta	18749	https://www.ncbi.nlm.nih.gov/gene/?term=18749
Chd2	Chd2	chromodomain helicase DNA binding protein 2	244059	https://www.ncbi.nlm.nih.gov/gene/?term=244059
Nkd1	Nkd1	naked cuticle 1	93960	https://www.ncbi.nlm.nih.gov/gene/?term=93960
Sobp	Sobp	sine oculis binding protein	109205	https://www.ncbi.nlm.nih.gov/gene/?term=109205

Biology Open • Supplementary information

Wdfy3	Wdfy3	WD repeat and FYVE domain containing 3	72145	https://www.ncbi.nlm.nih.gov/gene/?term=72145
Trhde	Trhde	TRH-degrading enzyme	237553	https://www.ncbi.nlm.nih.gov/gene/?term=237553
Ccdc80	Ccdc80	coiled-coil domain containing 80	67896	https://www.ncbi.nlm.nih.gov/gene/?term=67896
Zbtb34	Zbtb34	zinc finger and BTB domain containing 34	241311	https://www.ncbi.nlm.nih.gov/gene/?term=241311
Lysmd3	Lysmd3	LysM, putative peptidoglycan-binding, domain containing 3	80289	https://www.ncbi.nlm.nih.gov/gene/?term=80289
Atxn7	Atxn7	ataxin 7	246103	https://www.ncbi.nlm.nih.gov/gene/?term=246103
Astn1	Astn1	astrotactin 1	11899	https://www.ncbi.nlm.nih.gov/gene/?term=11899
Stx2	Stx2	syntaxin 2	13852	https://www.ncbi.nlm.nih.gov/gene/?term=13852
Brd3	Brd3	bromodomain containing 3	67382	https://www.ncbi.nlm.nih.gov/gene/?term=67382
Map7d1	Map7d1	MAP7 domain containing 1	245877	https://www.ncbi.nlm.nih.gov/gene/?term=245877
Cdk8	Cdk8	cyclin-dependent kinase 8	264064	https://www.ncbi.nlm.nih.gov/gene/?term=264064
Npas3	Npas3	neuronal PAS domain protein 3	27386	https://www.ncbi.nlm.nih.gov/gene/?term=27386
Tada1	Tada1	transcriptional adaptor 1	27878	https://www.ncbi.nlm.nih.gov/gene/?term=27878
Nfasc	Nfasc	neurofascin	269116	https://www.ncbi.nlm.nih.gov/gene/?term=269116
Lbr	Lbr	lamin B receptor	98386	https://www.ncbi.nlm.nih.gov/gene/?term=98386
Rheb	Rheb	Ras homolog enriched in brain	19744	https://www.ncbi.nlm.nih.gov/gene/?term=19744
Lrrc8a	Lrrc8a	leucine rich repeat containing 8A	241296	https://www.ncbi.nlm.nih.gov/gene/?term=241296
Zfp644	Zfp644	zinc finger protein 644	52397	https://www.ncbi.nlm.nih.gov/gene/?term=52397
Ccdc177	Ccdc177	coiled-coil domain containing 177	380768	https://www.ncbi.nlm.nih.gov/gene/?term=380768
Mbnl3	Mbnl3	muscleblind like splicing factor 3	171170	https://www.ncbi.nlm.nih.gov/gene/?term=171170
Cdc25a	Cdc25a	cell division cycle 25A	12530	https://www.ncbi.nlm.nih.gov/gene/?term=12530
C3ar1	C3ar1	complement component 3a receptor 1	12267	https://www.ncbi.nlm.nih.gov/gene/?term=12267
E2f3	E2f3	E2F transcription factor 3	13557	https://www.ncbi.nlm.nih.gov/gene/?term=13557
9330159F19Rik	9330159F19Rik	RIKEN cDNA 9330159F19 gene	212448	https://www.ncbi.nlm.nih.gov/gene/?term=212448

Biology Open • Supplementary information

Tcerg1	Tcerg1	transcription elongation regulator 1 (CA150)	56070	https://www.ncbi.nlm.nih.gov/gene/?term=56070
Zfp597	Zfp597	zinc finger protein 597	71063	https://www.ncbi.nlm.nih.gov/gene/?term=71063
Kcng3	Kcng3	potassium voltage-gated channel, subfamily G, member 3	225030	https://www.ncbi.nlm.nih.gov/gene/?term=225030
Stk17b	Stk17b	serine/threonine kinase 17b (apoptosis-inducing)	98267	https://www.ncbi.nlm.nih.gov/gene/?term=98267
Stxbp5	Stxbp5	syntaxin binding protein 5 (tomosyn)	78808	https://www.ncbi.nlm.nih.gov/gene/?term=78808
Cpeb3	Cpeb3	cytoplasmic polyadenylation element binding protein 3	208922	https://www.ncbi.nlm.nih.gov/gene/?term=208922
Fam227a	Fam227a	family with sequence similarity 227, member A	75729	https://www.ncbi.nlm.nih.gov/gene/?term=75729
Dcun1d3	Dcun1d3	DCN1, defective in cullin neddylation 1, domain containing 3 (S. cerevisiae)	233805	https://www.ncbi.nlm.nih.gov/gene/?term=233805
Tiam1	Tiam1	T cell lymphoma invasion and metastasis 1	21844	https://www.ncbi.nlm.nih.gov/gene/?term=21844
Tmem135	Tmem135	transmembrane protein 135	72759	https://www.ncbi.nlm.nih.gov/gene/?term=72759
Taf12	Taf12	TATA-box binding protein associated factor 12	66464	https://www.ncbi.nlm.nih.gov/gene/?term=66464
Tmem221	Tmem221	transmembrane protein 221	434325	https://www.ncbi.nlm.nih.gov/gene/?term=434325
Fnip2	Fnip2	folliculin interacting protein 2	329679	https://www.ncbi.nlm.nih.gov/gene/?term=329679
Atrnl1	Atrnl1	attractin like 1	226255	https://www.ncbi.nlm.nih.gov/gene/?term=226255
Tmeff1	Tmeff1	transmembrane protein with EGF-like and two follistatin-like domains 1	230157	https://www.ncbi.nlm.nih.gov/gene/?term=230157
Arpc5	Arpc5	actin related protein 2/3 complex, subunit 5	67771	https://www.ncbi.nlm.nih.gov/gene/?term=67771
Fam91a1	Fam91a1	family with sequence similarity 91, member A1	210998	https://www.ncbi.nlm.nih.gov/gene/?term=210998
Ranbp6	Ranbp6	RAN binding protein 6	240614	https://www.ncbi.nlm.nih.gov/gene/?term=240614

Biology Open • Supplementary information

Nrp1	Nrp1	neuropilin 1	18186	https://www.ncbi.nlm.nih.gov/gene/?term=18186
Adck2	Adck2	aarF domain containing kinase 2	57869	https://www.ncbi.nlm.nih.gov/gene/?term=57869
Jag1	Jag1	jagged 1	16449	https://www.ncbi.nlm.nih.gov/gene/?term=16449
Tfap2c	Tfap2c	transcription factor AP-2, gamma	21420	https://www.ncbi.nlm.nih.gov/gene/?term=21420
Hcn1	Hcn1	hyperpolarization-activated, cyclic nucleotide-gated K+ 1	15165	https://www.ncbi.nlm.nih.gov/gene/?term=15165
Msantd4	Msantd4	Myb/SANT-like DNA-binding domain containing 4 with coiled-coils	78100	https://www.ncbi.nlm.nih.gov/gene/?term=78100
Tmem237	Tmem237	transmembrane protein 237	381259	https://www.ncbi.nlm.nih.gov/gene/?term=381259
Abhd18	Abhd18	abhydrolase domain containing 18	269423	https://www.ncbi.nlm.nih.gov/gene/?term=269423
Qser1	Qser1	glutamine and serine rich 1	99003	https://www.ncbi.nlm.nih.gov/gene/?term=99003
Hmg20a	Hmg20a	high mobility group 20A	66867	https://www.ncbi.nlm.nih.gov/gene/?term=66867
Adgrb3	Adgrb3	adhesion G protein-coupled receptor B3	210933	https://www.ncbi.nlm.nih.gov/gene/?term=210933
Stag1	Stag1	stromal antigen 1	20842	https://www.ncbi.nlm.nih.gov/gene/?term=20842
Slc6a9	Slc6a9	solute carrier family 6 (neurotransmitter transporter, glycine), member 9	14664	https://www.ncbi.nlm.nih.gov/gene/?term=14664
Hephl1	Hephl1	hephaestin-like 1	244698	https://www.ncbi.nlm.nih.gov/gene/?term=244698
Zyg11b	Zyg11b	zyg-ll family member B, cell cycle regulator	414872	https://www.ncbi.nlm.nih.gov/gene/?term=414872
Cfap97	Cfap97	cilia and flagella associated protein 97	66756	https://www.ncbi.nlm.nih.gov/gene/?term=66756
Ppm1e	Ppm1e	protein phosphatase 1E (PP2C domain containing)	320472	https://www.ncbi.nlm.nih.gov/gene/?term=320472
Il13ra2	Il13ra2	interleukin 13 receptor, alpha 2	16165	https://www.ncbi.nlm.nih.gov/gene/?term=16165
Adpgk	Adpgk	ADP-dependent glucokinase	72141	https://www.ncbi.nlm.nih.gov/gene/?term=72141
Mtmr6	Mtmr6	myotubularin related protein 6	219135	https://www.ncbi.nlm.nih.gov/gene/?term=219135
Ttpal	Ttpal	tocopherol (alpha) transfer protein-	76080	https://www.ncbi.nlm.nih.gov/gene/?term=76080

		like		
Ttbk2	Ttbk2	tau tubulin kinase 2	140810	https://www.ncbi.nlm.nih.gov/gene/?term=140810
Arhgef18	Arhgef18	rho/rac guanine nucleotide exchange factor (GEF) 18	102098	https://www.ncbi.nlm.nih.gov/gene/?term=102098
Klf6	Klf6	Kruppel-like factor 6	23849	https://www.ncbi.nlm.nih.gov/gene/?term=23849
Sipa1l2	Sipa1l2	signal-induced proliferation-associated 1 like 2	244668	https://www.ncbi.nlm.nih.gov/gene/?term=244668
Klf12	Klf12	Kruppel-like factor 12	16597	https://www.ncbi.nlm.nih.gov/gene/?term=16597
Epha7	Epha7	Eph receptor A7	13841	https://www.ncbi.nlm.nih.gov/gene/?term=13841
Nufip2	Nufip2	nuclear fragile X mental retardation protein interacting protein 2	68564	https://www.ncbi.nlm.nih.gov/gene/?term=68564
Reck	Reck	reversion-inducing-cysteine-rich protein with kazal motifs	53614	https://www.ncbi.nlm.nih.gov/gene/?term=53614
Kif5b	Kif5b	kinesin family member 5B	16573	https://www.ncbi.nlm.nih.gov/gene/?term=16573
Ankrd44	Ankrd44	ankyrin repeat domain 44	329154	https://www.ncbi.nlm.nih.gov/gene/?term=329154
Ptprg	Ptprg	protein tyrosine phosphatase, receptor type, G	19270	https://www.ncbi.nlm.nih.gov/gene/?term=19270
Greb1l	Greb1l	growth regulation by estrogen in breast cancer-like	381157	https://www.ncbi.nlm.nih.gov/gene/?term=381157
Pdcd10	Pdcd10	programmed cell death 10	56426	https://www.ncbi.nlm.nih.gov/gene/?term=56426
Mbnl2	Mbnl2	muscleblind like splicing factor 2	105559	https://www.ncbi.nlm.nih.gov/gene/?term=105559
Foxf2	Foxf2	forkhead box F2	14238	https://www.ncbi.nlm.nih.gov/gene/?term=14238
Rac1	Rac1	Rac family small GTPase 1	19353	https://www.ncbi.nlm.nih.gov/gene/?term=19353
Tectb	Tectb	tectorin beta	21684	https://www.ncbi.nlm.nih.gov/gene/?term=21684
Gpr146	Gpr146	G protein-coupled receptor 146	80290	https://www.ncbi.nlm.nih.gov/gene/?term=80290
Clock	Clock	circadian locomotor output cycles kaput	12753	https://www.ncbi.nlm.nih.gov/gene/?term=12753
Zbtb37	Zbtb37	zinc finger and BTB domain containing 37	240869	https://www.ncbi.nlm.nih.gov/gene/?term=240869

Hoxa9	Hoxa9	homeobox A9	15405	https://www.ncbi.nlm.nih.gov/gene/?term=15405
Prelid3b	Prelid3b	PRELI domain containing 3B	66390	https://www.ncbi.nlm.nih.gov/gene/?term=66390
Slc44a2	Slc44a2	solute carrier family 44, member 2	68682	https://www.ncbi.nlm.nih.gov/gene/?term=68682
Msra	Msra	methionine sulfoxide reductase A	110265	https://www.ncbi.nlm.nih.gov/gene/?term=110265
Egr3	Egr3	early growth response 3	13655	https://www.ncbi.nlm.nih.gov/gene/?term=13655
Sinhcaf	Sinhcaf	SIN3-HDAC complex associated factor	56306	https://www.ncbi.nlm.nih.gov/gene/?term=56306
Phyhipl	Phyhipl	phytanoyl-CoA hydroxylase interacting protein-like	70911	https://www.ncbi.nlm.nih.gov/gene/?term=70911
Vamp3	Vamp3	vesicle-associated membrane protein 3	22319	https://www.ncbi.nlm.nih.gov/gene/?term=22319
Grb2	Grb2	growth factor receptor bound protein 2	14784	https://www.ncbi.nlm.nih.gov/gene/?term=14784
Braf	Braf	Braf transforming gene	109880	https://www.ncbi.nlm.nih.gov/gene/?term=109880
Prrg3	Prrg3	proline rich Gla (G-carboxyglutamic acid) 3 (transmembrane)	208748	https://www.ncbi.nlm.nih.gov/gene/?term=208748
Palld	Palld	palladin, cytoskeletal associated protein	72333	https://www.ncbi.nlm.nih.gov/gene/?term=72333
Mtss1	Mtss1	metastasis suppressor 1	211401	https://www.ncbi.nlm.nih.gov/gene/?term=211401
Ankrd27	Ankrd27	ankyrin repeat domain 27 (VPS9 domain)	245886	https://www.ncbi.nlm.nih.gov/gene/?term=245886
Spin1	Spin1	spindlin 1	20729	https://www.ncbi.nlm.nih.gov/gene/?term=20729
Coro1c	Coro1c	coronin, actin binding protein 1C	23790	https://www.ncbi.nlm.nih.gov/gene/?term=23790
Tro	Tro	trophinin	56191	https://www.ncbi.nlm.nih.gov/gene/?term=56191
Fam126b	Fam126b	family with sequence similarity 126, member B	213056	https://www.ncbi.nlm.nih.gov/gene/?term=213056
Ednrb	Ednrb	endothelin receptor type B	13618	https://www.ncbi.nlm.nih.gov/gene/?term=13618
Vezt	Vezt	vezatin, adherens junctions transmembrane protein	215008	https://www.ncbi.nlm.nih.gov/gene/?term=215008

Add3	Add3	adducin 3 (gamma)	27360	https://www.ncbi.nlm.nih.gov/gene/?term=27360
Taf15	Taf15	TATA-box binding protein associated factor 15	70439	https://www.ncbi.nlm.nih.gov/gene/?term=70439
Etl4	Etl4	enhancer trap locus 4	208618	https://www.ncbi.nlm.nih.gov/gene/?term=208618
Phf20l1	Phf20l1	PHD finger protein 20-like 1	239510	https://www.ncbi.nlm.nih.gov/gene/?term=239510
Prkce	Prkce	protein kinase C, epsilon	18754	https://www.ncbi.nlm.nih.gov/gene/?term=18754
Mitf	Mitf	melanogenesis associated transcription factor	17342	https://www.ncbi.nlm.nih.gov/gene/?term=17342
Nudt13	Nudt13	nudix (nucleoside diphosphate linked moiety X)-type motif 13	67725	https://www.ncbi.nlm.nih.gov/gene/?term=67725
Gli2	Gli2	GLI-Kruppel family member GLI2	14633	https://www.ncbi.nlm.nih.gov/gene/?term=14633
Cd2ap	Cd2ap	CD2-associated protein	12488	https://www.ncbi.nlm.nih.gov/gene/?term=12488
Sp3	Sp3	trans-acting transcription factor 3	20687	https://www.ncbi.nlm.nih.gov/gene/?term=20687
Thbs2	Thbs2	thrombospondin 2	21826	https://www.ncbi.nlm.nih.gov/gene/?term=21826
Arhgef3	Arhgef3	Rho guanine nucleotide exchange factor (GEF) 3	71704	https://www.ncbi.nlm.nih.gov/gene/?term=71704
Inpp5a	Inpp5a	inositol polyphosphate-5-phosphatase A	212111	https://www.ncbi.nlm.nih.gov/gene/?term=212111
Shc4	Shc4	SHC (Src homology 2 domain containing) family, member 4	271849	https://www.ncbi.nlm.nih.gov/gene/?term=271849
Bnip3	Bnip3	NA	12176	https://www.ncbi.nlm.nih.gov/gene/?term=12176
Lmtk2	Lmtk2	lemur tyrosine kinase 2	231876	https://www.ncbi.nlm.nih.gov/gene/?term=231876
Magi1	Magi1	membrane associated guanylate kinase, WW and PDZ domain containing 1	14924	https://www.ncbi.nlm.nih.gov/gene/?term=14924
Stx5a	Stx5a	syntaxin 5A	56389	https://www.ncbi.nlm.nih.gov/gene/?term=56389
Nptx1	Nptx1	neuronal pentraxin 1	18164	https://www.ncbi.nlm.nih.gov/gene/?term=18164
Rasa1	Rasa1	RAS p21 protein activator 1	218397	https://www.ncbi.nlm.nih.gov/gene/?term=218397
Wasl	Wasl	Wiskott-Aldrich syndrome-like	73178	https://www.ncbi.nlm.nih.gov/gene/?term=73178

Biology Open • Supplementary information

		(human)		
Trabd2b	Trabd2b	TraB domain containing 2B	666048	https://www.ncbi.nlm.nih.gov/gene/?term=666048
Elavl4	Elavl4	ELAV like RNA binding protein 4	15572	https://www.ncbi.nlm.nih.gov/gene/?term=15572
Wdr76	Wdr76	WD repeat domain 76	241627	https://www.ncbi.nlm.nih.gov/gene/?term=241627
Fam43a	Fam43a	family with sequence similarity 43, member A	224093	https://www.ncbi.nlm.nih.gov/gene/?term=224093
Topbp1	Topbp1	topoisomerase (DNA) II binding protein 1	235559	https://www.ncbi.nlm.nih.gov/gene/?term=235559
Slc39a9	Slc39a9	solute carrier family 39 (zinc transporter), member 9	328133	https://www.ncbi.nlm.nih.gov/gene/?term=328133
Rnf152	Rnf152	ring finger protein 152	320311	https://www.ncbi.nlm.nih.gov/gene/?term=320311
Rapgef5	Rapgef5	Rap guanine nucleotide exchange factor (GEF) 5	217944	https://www.ncbi.nlm.nih.gov/gene/?term=217944
Kdm2b	Kdm2b	lysine (K)-specific demethylase 2B	30841	https://www.ncbi.nlm.nih.gov/gene/?term=30841
Slc1a1	Slc1a1	solute carrier family 1 (neuronal/epithelial high affinity glutamate transporter, system Xag), member 1	20510	https://www.ncbi.nlm.nih.gov/gene/?term=20510
Pcnx	Pcnx	pecanex homolog	54604	https://www.ncbi.nlm.nih.gov/gene/?term=54604
Myrip	Myrip	myosin VIIA and Rab interacting protein	245049	https://www.ncbi.nlm.nih.gov/gene/?term=245049
Phip	Phip	pleckstrin homology domain interacting protein	83946	https://www.ncbi.nlm.nih.gov/gene/?term=83946
Stox2	Stox2	storkhead box 2	71069	https://www.ncbi.nlm.nih.gov/gene/?term=71069
Wdr5b	Wdr5b	WD repeat domain 5B	69544	https://www.ncbi.nlm.nih.gov/gene/?term=69544
Ell	Ell	elongation factor RNA polymerase II	13716	https://www.ncbi.nlm.nih.gov/gene/?term=13716
Dcun1d1	Dcun1d1	DCN1, defective in cullin neddylation 1, domain containing 1 (S. cerevisiae)	114893	https://www.ncbi.nlm.nih.gov/gene/?term=114893
Tmem145	Tmem145	transmembrane protein 145	330485	https://www.ncbi.nlm.nih.gov/gene/?term=330485

Biology Open • Supplementary information

Pnpla2	Pnpla2	patatin-like phospholipase domain containing 2	66853	https://www.ncbi.nlm.nih.gov/gene/?term=66853
Kif19a	Kif19a	kinesin family member 19A	286942	https://www.ncbi.nlm.nih.gov/gene/?term=286942
Aldh6a1	Aldh6a1	aldehyde dehydrogenase family 6, subfamily A1	104776	https://www.ncbi.nlm.nih.gov/gene/?term=104776
Ell2	Ell2	elongation factor RNA polymerase II 2	192657	https://www.ncbi.nlm.nih.gov/gene/?term=192657
Lims1	Lims1	LIM and senescent cell antigen-like domains 1	110829	https://www.ncbi.nlm.nih.gov/gene/?term=110829
Cacnb4	Cacnb4	calcium channel, voltage-dependent, beta 4 subunit	12298	https://www.ncbi.nlm.nih.gov/gene/?term=12298
Gfm2	Gfm2	G elongation factor, mitochondrial 2	320806	https://www.ncbi.nlm.nih.gov/gene/?term=320806
Xpr1	Xpr1	xenotropic and polytropic retrovirus receptor 1	19775	https://www.ncbi.nlm.nih.gov/gene/?term=19775
Cbarp	Cbarp	calcium channel, voltage-dependent, beta subunit associated regulatory protein	100503659	https://www.ncbi.nlm.nih.gov/gene/?term=100503
Pcx	Pcx	pyruvate carboxylase	18563	https://www.ncbi.nlm.nih.gov/gene/?term=18563
Zcchc14	Zcchc14	zinc finger, CCHC domain containing 14	142682	https://www.ncbi.nlm.nih.gov/gene/?term=142682
Yipf4	Yipf4	Yip1 domain family, member 4	67864	https://www.ncbi.nlm.nih.gov/gene/?term=67864
Ints13	Ints13	integrator complex subunit 13	71177	https://www.ncbi.nlm.nih.gov/gene/?term=71177
Magel2	Magel2	melanoma antigen, family L, 2	27385	https://www.ncbi.nlm.nih.gov/gene/?term=27385
Dok4	Dok4	docking protein 4	114255	https://www.ncbi.nlm.nih.gov/gene/?term=114255
Chic1	Chic1	cysteine-rich hydrophobic domain 1	12212	https://www.ncbi.nlm.nih.gov/gene/?term=12212
Cdv3	Cdv3	carnitine deficiency-associated gene expressed in ventricle 3	321022	https://www.ncbi.nlm.nih.gov/gene/?term=321022
Gnaq	Gnaq	guanine nucleotide binding protein, alpha q polypeptide	14682	https://www.ncbi.nlm.nih.gov/gene/?term=14682

Biology Open • Supplementary information

Bcl11a	Bcl11a	B cell CLL/lymphoma 11A (zinc finger protein)	14025	https://www.ncbi.nlm.nih.gov/gene/?term=14025
Slitrk4	Slitrk4	SLIT and NTRK-like family, member 4	245446	https://www.ncbi.nlm.nih.gov/gene/?term=245446
Dab1	Dab1	disabled 1	13131	https://www.ncbi.nlm.nih.gov/gene/?term=13131
Ralgps2	Ralgps2	Ral GEF with PH domain and SH3 binding motif 2	78255	https://www.ncbi.nlm.nih.gov/gene/?term=78255
Prtg	Prtg	protogenin	235472	https://www.ncbi.nlm.nih.gov/gene/?term=235472
Rgs17	Rgs17	regulator of G-protein signaling 17	56533	https://www.ncbi.nlm.nih.gov/gene/?term=56533
Slc1a2	Slc1a2	solute carrier family 1 (glial high affinity glutamate transporter), member 2	20511	https://www.ncbi.nlm.nih.gov/gene/?term=20511
Retreg1	Retreg1	reticulophagy regulator 1	66270	https://www.ncbi.nlm.nih.gov/gene/?term=66270
Mrtfb	Mrtfb	myocardin related transcription factor B	239719	https://www.ncbi.nlm.nih.gov/gene/?term=239719
Arf4	Arf4	ADP-ribosylation factor 4	11843	https://www.ncbi.nlm.nih.gov/gene/?term=11843
Igsf3	Igsf3	immunoglobulin superfamily, member 3	78908	https://www.ncbi.nlm.nih.gov/gene/?term=78908
Zc3h15	Zc3h15	zinc finger CCCH-type containing 15	69082	https://www.ncbi.nlm.nih.gov/gene/?term=69082
Dock1	Dock1	dedicator of cytokinesis 1	330662	https://www.ncbi.nlm.nih.gov/gene/?term=330662
Rarg	Rarg	retinoic acid receptor, gamma	19411	https://www.ncbi.nlm.nih.gov/gene/?term=19411
Dcaf4	Dcaf4	DDB1 and CUL4 associated factor 4	73828	https://www.ncbi.nlm.nih.gov/gene/?term=73828
Mak	Mak	male germ cell-associated kinase	17152	https://www.ncbi.nlm.nih.gov/gene/?term=17152
Nrn1	Nrn1	neuritin 1	68404	https://www.ncbi.nlm.nih.gov/gene/?term=68404
Anxa11	Anxa11	annexin A11	11744	https://www.ncbi.nlm.nih.gov/gene/?term=11744
Gys2	Gys2	glycogen synthase 2	232493	https://www.ncbi.nlm.nih.gov/gene/?term=232493
Cep250	Cep250	centrosomal protein 250	16328	https://www.ncbi.nlm.nih.gov/gene/?term=16328
Foxo3	Foxo3	forkhead box O3	56484	https://www.ncbi.nlm.nih.gov/gene/?term=56484
Brms1l	Brms1l	breast cancer metastasis-suppressor 1-like	52592	https://www.ncbi.nlm.nih.gov/gene/?term=52592

Biology Open • Supplementary information

Plekha7	Plekha7	pleckstrin homology domain containing, family A member 7	233765	https://www.ncbi.nlm.nih.gov/gene/?term=233765
Baalc	Baalc	brain and acute leukemia, cytoplasmic	118452	https://www.ncbi.nlm.nih.gov/gene/?term=118452
Igf1r	Igf1r	insulin-like growth factor I receptor	16001	https://www.ncbi.nlm.nih.gov/gene/?term=16001
Snx30	Snx30	sorting nexin family member 30	209131	https://www.ncbi.nlm.nih.gov/gene/?term=209131
Fam171a1	Fam171a1	family with sequence similarity 171, member A1	269233	https://www.ncbi.nlm.nih.gov/gene/?term=269233
Frs2	Frs2	fibroblast growth factor receptor substrate 2	327826	https://www.ncbi.nlm.nih.gov/gene/?term=327826
Pbx2	Pbx2	pre B cell leukemia homeobox 2	18515	https://www.ncbi.nlm.nih.gov/gene/?term=18515
Slc39a1	Slc39a1	solute carrier family 39 (zinc transporter), member 1	30791	https://www.ncbi.nlm.nih.gov/gene/?term=30791
Fam168a	Fam168a	family with sequence similarity 168, member A	319604	https://www.ncbi.nlm.nih.gov/gene/?term=319604
Cnnm3	Cnnm3	cyclin M3	94218	https://www.ncbi.nlm.nih.gov/gene/?term=94218
Ccny	Ccny	cyclin Y	67974	https://www.ncbi.nlm.nih.gov/gene/?term=67974
Usp13	Usp13	ubiquitin specific peptidase 13 (isopeptidase T-3)	72607	https://www.ncbi.nlm.nih.gov/gene/?term=72607
L1cam	L1cam	L1 cell adhesion molecule	16728	https://www.ncbi.nlm.nih.gov/gene/?term=16728
Wwc2	Wwc2	WW, C2 and coiled-coil domain containing 2	52357	https://www.ncbi.nlm.nih.gov/gene/?term=52357
Chst1	Chst1	carbohydrate sulfotransferase 1	76969	https://www.ncbi.nlm.nih.gov/gene/?term=76969
N4bp1	N4bp1	NEDD4 binding protein 1	80750	https://www.ncbi.nlm.nih.gov/gene/?term=80750
Has2	Has2	hyaluronan synthase 2	15117	https://www.ncbi.nlm.nih.gov/gene/?term=15117
Nid1	Nid1	nidogen 1	18073	https://www.ncbi.nlm.nih.gov/gene/?term=18073
Mast4	Mast4	microtubule associated serine/threonine kinase family member 4	328329	https://www.ncbi.nlm.nih.gov/gene/?term=328329

Biology Open • Supplementary information

Dock4	Dock4	dedicator of cytokinesis 4	238130	https://www.ncbi.nlm.nih.gov/gene/?term=238130
Cbfa2t3	Cbfa2t3	CBFA2/RUNX1 translocation partner 3	12398	https://www.ncbi.nlm.nih.gov/gene/?term=12398
Pdzd8	Pdzd8	PDZ domain containing 8	107368	https://www.ncbi.nlm.nih.gov/gene/?term=107368
Rnf208	Rnf208	ring finger protein 208	68846	https://www.ncbi.nlm.nih.gov/gene/?term=68846
Smad1	Smad1	SMAD family member 1	17125	https://www.ncbi.nlm.nih.gov/gene/?term=17125
Rev1	Rev1	REV1, DNA directed polymerase	56210	https://www.ncbi.nlm.nih.gov/gene/?term=56210
Bcl2l12	Bcl2l12	BCL2-like 12 (proline rich)	75736	https://www.ncbi.nlm.nih.gov/gene/?term=75736
Kdelr2	Kdelr2	KDEL (Lys-Asp-Glu-Leu) endoplasmic reticulum protein retention receptor 2	66913	https://www.ncbi.nlm.nih.gov/gene/?term=66913
Ipcef1	Ipcef1	interaction protein for cytohesin exchange factors 1	320495	https://www.ncbi.nlm.nih.gov/gene/?term=320495
Adgrl2	Adgrl2	adhesion G protein-coupled receptor L2	99633	https://www.ncbi.nlm.nih.gov/gene/?term=99633
Cttn	Cttn	cortactin	13043	https://www.ncbi.nlm.nih.gov/gene/?term=13043
Nexmif	Nexmif	neurite extension and migration factor	245555	https://www.ncbi.nlm.nih.gov/gene/?term=245555
Zfc3h1	Zfc3h1	zinc finger, C3H1-type containing	216345	https://www.ncbi.nlm.nih.gov/gene/?term=216345
Cep83	Cep83	centrosomal protein 83	77048	https://www.ncbi.nlm.nih.gov/gene/?term=77048
Ube2q2	Ube2q2	ubiquitin-conjugating enzyme E2Q family member 2	109161	https://www.ncbi.nlm.nih.gov/gene/?term=109161
Rab2a	Rab2a	RAB2A, member RAS oncogene family	59021	https://www.ncbi.nlm.nih.gov/gene/?term=59021
Slco3a1	Slco3a1	solute carrier organic anion transporter family, member 3a1	108116	https://www.ncbi.nlm.nih.gov/gene/?term=108116
Zbtb41	Zbtb41	zinc finger and BTB domain containing 41	226470	https://www.ncbi.nlm.nih.gov/gene/?term=226470
Ahcyl1	Ahcyl1	S-adenosylhomocysteine hydrolase-like 1	229709	https://www.ncbi.nlm.nih.gov/gene/?term=229709

Satb2	Satb2	special AT-rich sequence binding protein 2	212712	https://www.ncbi.nlm.nih.gov/gene/?term=212712
Col25a1	Col25a1	collagen, type XXV, alpha 1	77018	https://www.ncbi.nlm.nih.gov/gene/?term=77018
Adra2c	Adra2c	adrenergic receptor, alpha 2c	11553	https://www.ncbi.nlm.nih.gov/gene/?term=11553
Bcl2l13	Bcl2l13	BCL2-like 13 (apoptosis facilitator)	94044	https://www.ncbi.nlm.nih.gov/gene/?term=94044
Bach2	Bach2	BTB and CNC homology, basic leucine zipper transcription factor 2	12014	https://www.ncbi.nlm.nih.gov/gene/?term=12014
Ppm1k	Ppm1k	protein phosphatase 1K (PP2C domain containing)	243382	https://www.ncbi.nlm.nih.gov/gene/?term=243382
Tapt1	Tapt1	transmembrane anterior posterior transformation 1	231225	https://www.ncbi.nlm.nih.gov/gene/?term=231225
Taf4b	Taf4b	TATA-box binding protein associated factor 4b	72504	https://www.ncbi.nlm.nih.gov/gene/?term=72504
Cadm2	Cadm2	cell adhesion molecule 2	239857	https://www.ncbi.nlm.nih.gov/gene/?term=239857
Fam118a	Fam118a	family with sequence similarity 118, member A	73225	https://www.ncbi.nlm.nih.gov/gene/?term=73225
Coa7	Coa7	cytochrome c oxidase assembly factor 7	69893	https://www.ncbi.nlm.nih.gov/gene/?term=69893
Htr1a	Htr1a	5-hydroxytryptamine (serotonin) receptor 1A	15550	https://www.ncbi.nlm.nih.gov/gene/?term=15550
Tns3	Tns3	tensin 3	319939	https://www.ncbi.nlm.nih.gov/gene/?term=319939
Atat1	Atat1	alpha tubulin acetyltransferase 1	73242	https://www.ncbi.nlm.nih.gov/gene/?term=73242
Zfp36l1	Zfp36l1	zinc finger protein 36, C3H type-like 1	12192	https://www.ncbi.nlm.nih.gov/gene/?term=12192
Tagln3	Tagln3	transgelin 3	56370	https://www.ncbi.nlm.nih.gov/gene/?term=56370
Ppil1	Ppil1	peptidylprolyl isomerase (cyclophilin)-like 1	68816	https://www.ncbi.nlm.nih.gov/gene/?term=68816
Mtm1	Mtm1	X-linked myotubular myopathy gene 1	17772	https://www.ncbi.nlm.nih.gov/gene/?term=17772

Biology Open • Supplementary information

Foxn2	Foxn2	forkhead box N2	14236	https://www.ncbi.nlm.nih.gov/gene/?term=14236
0610030E20Rik	0610030E20Rik	RIKEN cDNA 0610030E20 gene	68364	https://www.ncbi.nlm.nih.gov/gene/?term=68364
Wipi2	Wipi2	WD repeat domain, phosphoinositide interacting 2	74781	https://www.ncbi.nlm.nih.gov/gene/?term=74781
Irak1	Irak1	interleukin-1 receptor-associated kinase 1	16179	https://www.ncbi.nlm.nih.gov/gene/?term=16179
Fbxw2	Fbxw2	F-box and WD-40 domain protein 2	30050	https://www.ncbi.nlm.nih.gov/gene/?term=30050
Slc10a3	Slc10a3	solute carrier family 10 (sodium/bile acid cotransporter family), member 3	214601	https://www.ncbi.nlm.nih.gov/gene/?term=214601
Eif4g2	Eif4g2	eukaryotic translation initiation factor 4, gamma 2	13690	https://www.ncbi.nlm.nih.gov/gene/?term=13690
Numb	Numb	NUMB endocytic adaptor protein	18222	https://www.ncbi.nlm.nih.gov/gene/?term=18222
Kras	Kras	Kirsten rat sarcoma viral oncogene homolog	16653	https://www.ncbi.nlm.nih.gov/gene/?term=16653
Rbm18	Rbm18	RNA binding motif protein 18	67889	https://www.ncbi.nlm.nih.gov/gene/?term=67889
Tdrkh	Tdrkh	tudor and KH domain containing protein	72634	https://www.ncbi.nlm.nih.gov/gene/?term=72634
Phc3	Phc3	polyhomeotic 3	241915	https://www.ncbi.nlm.nih.gov/gene/?term=241915
Sec23ip	Sec23ip	Sec23 interacting protein	207352	https://www.ncbi.nlm.nih.gov/gene/?term=207352
Traf6	Traf6	TNF receptor-associated factor 6	22034	https://www.ncbi.nlm.nih.gov/gene/?term=22034
Med1	Med1	mediator complex subunit 1	19014	https://www.ncbi.nlm.nih.gov/gene/?term=19014
Paqr5	Paqr5	progestin and adipoQ receptor family member V	74090	https://www.ncbi.nlm.nih.gov/gene/?term=74090
Eif5a2	Eif5a2	eukaryotic translation initiation factor 5A2	208691	https://www.ncbi.nlm.nih.gov/gene/?term=208691
D030056L22Rik	D030056L22Rik	RIKEN cDNA D030056L22 gene	225995	https://www.ncbi.nlm.nih.gov/gene/?term=225995
Esyt2	Esyt2	extended synaptotagmin-like protein 2	52635	https://www.ncbi.nlm.nih.gov/gene/?term=52635

Sbspon	Sbspon	somatomedin B and thrombospondin, type 1 domain containing	226866	https://www.ncbi.nlm.nih.gov/gene/?term=226866
Appl1	Appl1	adaptor protein, phosphotyrosine interaction, PH domain and leucine zipper containing 1	72993	https://www.ncbi.nlm.nih.gov/gene/?term=72993
Mri1	Mri1	methylthioribose-1-phosphate isomerase 1	67873	https://www.ncbi.nlm.nih.gov/gene/?term=67873
Nus1	Nus1	NUS1 dehydrodolichyl diphosphate synthase subunit	52014	https://www.ncbi.nlm.nih.gov/gene/?term=52014
Ncam1	Ncam1	neural cell adhesion molecule 1	17967	https://www.ncbi.nlm.nih.gov/gene/?term=17967
Acvr1b	Acvr1b	activin A receptor, type 1B	11479	https://www.ncbi.nlm.nih.gov/gene/?term=11479
Ranbp3	Ranbp3	RAN binding protein 3	71810	https://www.ncbi.nlm.nih.gov/gene/?term=71810
Ripply3	Ripply3	ripply transcriptional repressor 3	170765	https://www.ncbi.nlm.nih.gov/gene/?term=170765
Tfpi2	Tfpi2	tissue factor pathway inhibitor 2	21789	https://www.ncbi.nlm.nih.gov/gene/?term=21789
A1bg	A1bg	alpha-1-B glycoprotein	117586	https://www.ncbi.nlm.nih.gov/gene/?term=117586
Plpp3	Plpp3	phospholipid phosphatase 3	67916	https://www.ncbi.nlm.nih.gov/gene/?term=67916
Insig2	Insig2	insulin induced gene 2	72999	https://www.ncbi.nlm.nih.gov/gene/?term=72999
Armc1	Armc1	armadillo repeat containing 1	74252	https://www.ncbi.nlm.nih.gov/gene/?term=74252
Foxc1	Foxc1	forkhead box C1	17300	https://www.ncbi.nlm.nih.gov/gene/?term=17300
Atad2	Atad2	ATPase family, AAA domain containing 2	70472	https://www.ncbi.nlm.nih.gov/gene/?term=70472
Wnt5a	Wnt5a	wingless-type MMTV integration site family, member 5A	22418	https://www.ncbi.nlm.nih.gov/gene/?term=22418
Tmprss11e	Tmprss11e	transmembrane protease, serine 11e	243084	https://www.ncbi.nlm.nih.gov/gene/?term=243084
Ubr3	Ubr3	ubiquitin protein ligase E3 component n-recognin 3	68795	https://www.ncbi.nlm.nih.gov/gene/?term=68795
Usp53	Usp53	ubiquitin specific peptidase 53	99526	https://www.ncbi.nlm.nih.gov/gene/?term=99526

Cyp2c50	Cyp2c50	cytochrome P450, family 2, subfamily c, polypeptide 50	107141	https://www.ncbi.nlm.nih.gov/gene/?term=107141
Jchain	Jchain	immunoglobulin joining chain	16069	https://www.ncbi.nlm.nih.gov/gene/?term=16069
Kif3c	Kif3c	kinesin family member 3C	16570	https://www.ncbi.nlm.nih.gov/gene/?term=16570
4930447C04Rik	4930447C04Rik	RIKEN cDNA 4930447C04 gene	75801	https://www.ncbi.nlm.nih.gov/gene/?term=75801
Usp22	Usp22	ubiquitin specific peptidase 22	216825	https://www.ncbi.nlm.nih.gov/gene/?term=216825
Syt6	Syt6	synaptotagmin VI	54524	https://www.ncbi.nlm.nih.gov/gene/?term=54524
Ism1	Ism1	isthmin 1, angiogenesis inhibitor	319909	https://www.ncbi.nlm.nih.gov/gene/?term=319909
Sgms1	Sgms1	sphingomyelin synthase 1	208449	https://www.ncbi.nlm.nih.gov/gene/?term=208449
Nptn	Nptn	neuroplastin	20320	https://www.ncbi.nlm.nih.gov/gene/?term=20320
Arpp19	Arpp19	cAMP-regulated phosphoprotein 19	59046	https://www.ncbi.nlm.nih.gov/gene/?term=59046
Sik1	Sik1	salt inducible kinase 1	17691	https://www.ncbi.nlm.nih.gov/gene/?term=17691
Smad2	Smad2	SMAD family member 2	17126	https://www.ncbi.nlm.nih.gov/gene/?term=17126
Rab34	Rab34	RAB34, member RAS oncogene family	19376	https://www.ncbi.nlm.nih.gov/gene/?term=19376
Barhl2	Barhl2	BarH like homeobox 2	104382	https://www.ncbi.nlm.nih.gov/gene/?term=104382
Arhgap21	Arhgap21	Rho GTPase activating protein 21	71435	https://www.ncbi.nlm.nih.gov/gene/?term=71435
Plaa	Plaa	phospholipase A2, activating protein	18786	https://www.ncbi.nlm.nih.gov/gene/?term=18786
Ucp3	Ucp3	uncoupling protein 3 (mitochondrial, proton carrier)	22229	https://www.ncbi.nlm.nih.gov/gene/?term=22229
Cand1	Cand1	cullin associated and neddylation disassociated 1	71902	https://www.ncbi.nlm.nih.gov/gene/?term=71902
Bcl2l11	Bcl2l11	BCL2-like 11 (apoptosis facilitator)	12125	https://www.ncbi.nlm.nih.gov/gene/?term=12125
Otud4	Otud4	OTU domain containing 4	73945	https://www.ncbi.nlm.nih.gov/gene/?term=73945
Oas1c	Oas1c	2'-5' oligoadenylate synthetase 1C	114643	https://www.ncbi.nlm.nih.gov/gene/?term=114643
Ptpn14	Ptpn14	protein tyrosine phosphatase, non-receptor type 14	19250	https://www.ncbi.nlm.nih.gov/gene/?term=19250
S1pr1	S1pr1	sphingosine-1-phosphate receptor 1	13609	https://www.ncbi.nlm.nih.gov/gene/?term=13609

Sos2	Sos2	SOS Ras/Rho guanine nucleotide exchange factor 2	20663	https://www.ncbi.nlm.nih.gov/gene/?term=20663
Npepl1	Npepl1	aminopeptidase-like 1	228961	https://www.ncbi.nlm.nih.gov/gene/?term=228961
Rtca	Rtca	RNA 3'-terminal phosphate cyclase	66368	https://www.ncbi.nlm.nih.gov/gene/?term=66368
Naaladl2	Naaladl2	N-acetylated alpha-linked acidic dipeptidase-like 2	635702	https://www.ncbi.nlm.nih.gov/gene/?term=635702
Maf1	Maf1	MAF1 homolog, negative regulator of RNA polymerase III	68877	https://www.ncbi.nlm.nih.gov/gene/?term=68877
Esr1	Esr1	estrogen receptor 1 (alpha)	13982	https://www.ncbi.nlm.nih.gov/gene/?term=13982
Atp8a1	Atp8a1	ATPase, aminophospholipid transporter (APLT), class I, type 8A, member 1	11980	https://www.ncbi.nlm.nih.gov/gene/?term=11980
Tmem9b	Tmem9b	TMEM9 domain family, member B	56786	https://www.ncbi.nlm.nih.gov/gene/?term=56786
Epn2	Epn2	epsin 2	13855	https://www.ncbi.nlm.nih.gov/gene/?term=13855
Btbd3	Btbd3	BTB (POZ) domain containing 3	228662	https://www.ncbi.nlm.nih.gov/gene/?term=228662
Dicer1	Dicer1	dicer 1, ribonuclease type III	192119	https://www.ncbi.nlm.nih.gov/gene/?term=192119
Stam	Stam	signal transducing adaptor molecule (SH3 domain and ITAM motif) 1	20844	https://www.ncbi.nlm.nih.gov/gene/?term=20844
Akap1	Akap1	A kinase (PRKA) anchor protein 1	11640	https://www.ncbi.nlm.nih.gov/gene/?term=11640
Fbn1	Fbn1	fibrillin 1	14118	https://www.ncbi.nlm.nih.gov/gene/?term=14118
Ube3b	Ube3b	ubiquitin protein ligase E3B	117146	https://www.ncbi.nlm.nih.gov/gene/?term=117146
Ctxn3	Ctxn3	cortexin 3	629147	https://www.ncbi.nlm.nih.gov/gene/?term=629147
Arl6ip1	Arl6ip1	ADP-ribosylation factor-like 6 interacting protein 1	54208	https://www.ncbi.nlm.nih.gov/gene/?term=54208
Lrp2	Lrp2	low density lipoprotein receptor-related protein 2	14725	https://www.ncbi.nlm.nih.gov/gene/?term=14725
Cdk19	Cdk19	cyclin-dependent kinase 19	78334	https://www.ncbi.nlm.nih.gov/gene/?term=78334
Mospd1	Mospd1	motile sperm domain containing 1	70380	https://www.ncbi.nlm.nih.gov/gene/?term=70380
Tgfa	Tgfa	transforming growth factor alpha	21802	https://www.ncbi.nlm.nih.gov/gene/?term=21802

Biology Open • Supplementary information

Ywhab	Ywhab	tyrosine 3-monooxygenase/tryptophan 5-monooxygenase activation protein, beta polypeptide	54401	https://www.ncbi.nlm.nih.gov/gene/?term=54401
Usp32	Usp32	ubiquitin specific peptidase 32	237898	https://www.ncbi.nlm.nih.gov/gene/?term=237898
Med12l	Med12l	mediator complex subunit 12-like	329650	https://www.ncbi.nlm.nih.gov/gene/?term=329650
Nrarp	Nrarp	Notch-regulated ankyrin repeat protein	67122	https://www.ncbi.nlm.nih.gov/gene/?term=67122
Tmem54	Tmem54	transmembrane protein 54	66260	https://www.ncbi.nlm.nih.gov/gene/?term=66260
Fam104a	Fam104a	family with sequence similarity 104, member A	28081	https://www.ncbi.nlm.nih.gov/gene/?term=28081
Col4a1	Col4a1	collagen, type IV, alpha 1	12826	https://www.ncbi.nlm.nih.gov/gene/?term=12826
Hepacam2	Hepacam2	HEPACAM family member 2	101202	https://www.ncbi.nlm.nih.gov/gene/?term=101202
B4galt5	B4galt5	UDP-Gal:betaGlcNAc beta 1,4-galactosyltransferase, polypeptide 5	56336	https://www.ncbi.nlm.nih.gov/gene/?term=56336
Jmy	Jmy	junction-mediating and regulatory protein	57748	https://www.ncbi.nlm.nih.gov/gene/?term=57748
Tnrc6c	Tnrc6c	trinucleotide repeat containing 6C	217351	https://www.ncbi.nlm.nih.gov/gene/?term=217351
Abcb7	Abcb7	ATP-binding cassette, sub-family B (MDR/TAP), member 7	11306	https://www.ncbi.nlm.nih.gov/gene/?term=11306
Arrdc3	Arrdc3	arrestin domain containing 3	105171	https://www.ncbi.nlm.nih.gov/gene/?term=105171
Btaf1	Btaf1	B-TFIID TATA-box binding protein associated factor 1	107182	https://www.ncbi.nlm.nih.gov/gene/?term=107182
Rictor	Rictor	RPTOR independent companion of MTOR, complex 2	78757	https://www.ncbi.nlm.nih.gov/gene/?term=78757
Canx	Canx	calnexin	12330	https://www.ncbi.nlm.nih.gov/gene/?term=12330
Manba	Manba	mannosidase, beta A, lysosomal	110173	https://www.ncbi.nlm.nih.gov/gene/?term=110173
Robo1	Robo1	roundabout guidance receptor 1	19876	https://www.ncbi.nlm.nih.gov/gene/?term=19876
Phactr2	Phactr2	phosphatase and actin regulator 2	215789	https://www.ncbi.nlm.nih.gov/gene/?term=215789

Biology Open • Supplementary information

Amacr	Amacr	alpha-methylacyl-CoA racemase	17117	https://www.ncbi.nlm.nih.gov/gene/?term=17117
Ncoa1	Ncoa1	nuclear receptor coactivator 1	17977	https://www.ncbi.nlm.nih.gov/gene/?term=17977
Rph3al	Rph3al	rabphilin 3A-like (without C2 domains)	380714	https://www.ncbi.nlm.nih.gov/gene/?term=380714
Sgcb	Sgcb	sarcoglycan, beta (dystrophin-associated glycoprotein)	24051	https://www.ncbi.nlm.nih.gov/gene/?term=24051
Lgalsl	Lgalsl	lectin, galactoside binding-like	216551	https://www.ncbi.nlm.nih.gov/gene/?term=216551
Ppp6r1	Ppp6r1	protein phosphatase 6, regulatory subunit 1	243819	https://www.ncbi.nlm.nih.gov/gene/?term=243819
Slco4a1	Slco4a1	solute carrier organic anion transporter family, member 4a1	108115	https://www.ncbi.nlm.nih.gov/gene/?term=108115
Robo2	Robo2	roundabout guidance receptor 2	268902	https://www.ncbi.nlm.nih.gov/gene/?term=268902
Adamts15	Adamts15	a disintegrin-like and metallopeptidase (reprolysin type) with thrombospondin type 1 motif, 15	235130	https://www.ncbi.nlm.nih.gov/gene/?term=235130
Jph3	Jph3	junctophilin 3	57340	https://www.ncbi.nlm.nih.gov/gene/?term=57340
Fam234a	Fam234a	family with sequence similarity 234, member A	106581	https://www.ncbi.nlm.nih.gov/gene/?term=106581
Hbs1l	Hbs1l	Hbs1-like (S. cerevisiae)	56422	https://www.ncbi.nlm.nih.gov/gene/?term=56422
Hs3st1	Hs3st1	heparan sulfate (glucosamine) 3-O-sulfotransferase 1	15476	https://www.ncbi.nlm.nih.gov/gene/?term=15476
Inhbb	Inhbb	inhibin beta-B	16324	https://www.ncbi.nlm.nih.gov/gene/?term=16324
Ssr1	Ssr1	signal sequence receptor, alpha	107513	https://www.ncbi.nlm.nih.gov/gene/?term=107513
Dgcr8	Dgcr8	DGCR8, microprocessor complex subunit	94223	https://www.ncbi.nlm.nih.gov/gene/?term=94223
Ube2d3	Ube2d3	ubiquitin-conjugating enzyme E2D 3	66105	https://www.ncbi.nlm.nih.gov/gene/?term=66105
Ino80	Ino80	INO80 complex subunit	68142	https://www.ncbi.nlm.nih.gov/gene/?term=68142

Biology Open • Supplementary information

C1galt1	C1galt1	core 1 synthase, glycoprotein-N-acetylgalactosamine 3-beta-galactosyltransferase, 1	94192	https://www.ncbi.nlm.nih.gov/gene/?term=94192
Ajuba	Ajuba	ajuba LIM protein	16475	https://www.ncbi.nlm.nih.gov/gene/?term=16475
D5Ertd579e	D5Ertd579e	DNA segment, Chr 5, ERATO Doi 579, expressed	320661	https://www.ncbi.nlm.nih.gov/gene/?term=320661
Tnrc6a	Tnrc6a	trinucleotide repeat containing 6a	233833	https://www.ncbi.nlm.nih.gov/gene/?term=233833
Meox2	Meox2	mesenchyme homeobox 2	17286	https://www.ncbi.nlm.nih.gov/gene/?term=17286
Mdfic	Mdfic	MyoD family inhibitor domain containing	16543	https://www.ncbi.nlm.nih.gov/gene/?term=16543
Itga9	Itga9	integrin alpha 9	104099	https://www.ncbi.nlm.nih.gov/gene/?term=104099
Rmnd5a	Rmnd5a	required for meiotic nuclear division 5 homolog A	68477	https://www.ncbi.nlm.nih.gov/gene/?term=68477
Eogt	Eogt	EGF domain-specific O-linked N-acetylglucosamine (GlcNAc) transferase	101351	https://www.ncbi.nlm.nih.gov/gene/?term=101351
Relch	Relch	RAB11 binding and LisH domain, coiled-coil and HEAT repeat containing	227446	https://www.ncbi.nlm.nih.gov/gene/?term=227446
Itga5	Itga5	integrin alpha 5 (fibronectin receptor alpha)	16402	https://www.ncbi.nlm.nih.gov/gene/?term=16402
Ppp1r10	Ppp1r10	protein phosphatase 1, regulatory subunit 10	52040	https://www.ncbi.nlm.nih.gov/gene/?term=52040
Phf3	Phf3	PHD finger protein 3	213109	https://www.ncbi.nlm.nih.gov/gene/?term=213109
Mpped2	Mpped2	metallophosphoesterase domain containing 2	77015	https://www.ncbi.nlm.nih.gov/gene/?term=77015
Psmb6	Psmb6	proteasome (prosome, macropain) subunit, beta type 6	19175	https://www.ncbi.nlm.nih.gov/gene/?term=19175
Cblb	Cblb	Casitas B-lineage lymphoma b	208650	https://www.ncbi.nlm.nih.gov/gene/?term=208650

Biology Open • Supplementary information

Gadd45a	Gadd45a	growth arrest and DNA-damage-inducible 45 alpha	13197	https://www.ncbi.nlm.nih.gov/gene/?term=13197
Pde1a	Pde1a	phosphodiesterase 1A, calmodulin-dependent	18573	https://www.ncbi.nlm.nih.gov/gene/?term=18573
Cmip	Cmip	c-Maf inducing protein	74440	https://www.ncbi.nlm.nih.gov/gene/?term=74440
Serpinb2	Serpinb2	serine (or cysteine) peptidase inhibitor, clade B, member 2	18788	https://www.ncbi.nlm.nih.gov/gene/?term=18788
Sh3pxd2b	Sh3pxd2b	SH3 and PX domains 2B	268396	https://www.ncbi.nlm.nih.gov/gene/?term=268396
Bcr	Bcr	breakpoint cluster region	110279	https://www.ncbi.nlm.nih.gov/gene/?term=110279
Ttyh3	Ttyh3	tweety family member 3	78339	https://www.ncbi.nlm.nih.gov/gene/?term=78339
Plod2	Plod2	procollagen lysine, 2-oxoglutarate 5-dioxygenase 2	26432	https://www.ncbi.nlm.nih.gov/gene/?term=26432
Tsku	Tsku	tsukushi, small leucine rich proteoglycan	244152	https://www.ncbi.nlm.nih.gov/gene/?term=244152
Cep170b	Cep170b	centrosomal protein 170B	217882	https://www.ncbi.nlm.nih.gov/gene/?term=217882
Sh3kbp1	Sh3kbp1	SH3-domain kinase binding protein 1	58194	https://www.ncbi.nlm.nih.gov/gene/?term=58194
Tgfbr1	Tgfbr1	transforming growth factor, beta receptor I	21812	https://www.ncbi.nlm.nih.gov/gene/?term=21812
Slc16a9	Slc16a9	solute carrier family 16 (monocarboxylic acid transporters), member 9	66859	https://www.ncbi.nlm.nih.gov/gene/?term=66859
Gulp1	Gulp1	GULP, engulfment adaptor PTB domain containing 1	70676	https://www.ncbi.nlm.nih.gov/gene/?term=70676
Rab23	Rab23	RAB23, member RAS oncogene family	19335	https://www.ncbi.nlm.nih.gov/gene/?term=19335
Eif4ebp2	Eif4ebp2	eukaryotic translation initiation factor 4E binding protein 2	13688	https://www.ncbi.nlm.nih.gov/gene/?term=13688
Gpc3	Gpc3	glypican 3	14734	https://www.ncbi.nlm.nih.gov/gene/?term=14734
Cnn3	Cnn3	calponin 3, acidic	71994	https://www.ncbi.nlm.nih.gov/gene/?term=71994

Biology Open • Supplementary information

Nsg1	Nsg1	neuron specific gene family member 1	18196	https://www.ncbi.nlm.nih.gov/gene/?term=18196
Celsr1	Celsr1	cadherin, EGF LAG seven-pass G-type receptor 1	12614	https://www.ncbi.nlm.nih.gov/gene/?term=12614
Hsd3b5	Hsd3b5	hydroxy-delta-5-steroid dehydrogenase, 3 beta- and steroid delta-isomerase 5	15496	https://www.ncbi.nlm.nih.gov/gene/?term=15496
Zkscan17	Zkscan17	zinc finger with KRAB and SCAN domains 17	268417	https://www.ncbi.nlm.nih.gov/gene/?term=268417
Morf4l1	Morf4l1	mortality factor 4 like 1	21761	https://www.ncbi.nlm.nih.gov/gene/?term=21761
Ogt	Ogt	O-linked N-acetylglucosamine (GlcNAc) transferase (UDP-N-acetylglucosamine:polypeptide-N-acetylglucosaminyl transferase)	108155	https://www.ncbi.nlm.nih.gov/gene/?term=108155
Klhl7	Klhl7	kelch-like 7	52323	https://www.ncbi.nlm.nih.gov/gene/?term=52323
Ralgps1	Ralgps1	Ral GEF with PH domain and SH3 binding motif 1	241308	https://www.ncbi.nlm.nih.gov/gene/?term=241308
Foxq1	Foxq1	forkhead box Q1	15220	https://www.ncbi.nlm.nih.gov/gene/?term=15220
Aqp5	Aqp5	aquaporin 5	11830	https://www.ncbi.nlm.nih.gov/gene/?term=11830
Mtor	Mtor	mechanistic target of rapamycin kinase	56717	https://www.ncbi.nlm.nih.gov/gene/?term=56717
Foxk2	Foxk2	forkhead box K2	68837	https://www.ncbi.nlm.nih.gov/gene/?term=68837
Sox6	Sox6	SRY (sex determining region Y)-box 6	20679	https://www.ncbi.nlm.nih.gov/gene/?term=20679
Pmepa1	Pmepa1	prostate transmembrane protein, androgen induced 1	65112	https://www.ncbi.nlm.nih.gov/gene/?term=65112
Cd164	Cd164	CD164 antigen	53599	https://www.ncbi.nlm.nih.gov/gene/?term=53599
Tcf7l2	Tcf7l2	transcription factor 7 like 2, T cell specific, HMG box	21416	https://www.ncbi.nlm.nih.gov/gene/?term=21416
Olfr613	Olfr613	olfactory receptor 613	259104	https://www.ncbi.nlm.nih.gov/gene/?term=259104

Fundc2	Fundc2	FUN14 domain containing 2	67391	https://www.ncbi.nlm.nih.gov/gene/?term=67391
Pde8b	Pde8b	phosphodiesterase 8B	218461	https://www.ncbi.nlm.nih.gov/gene/?term=218461
E2f5	E2f5	E2F transcription factor 5	13559	https://www.ncbi.nlm.nih.gov/gene/?term=13559
Ahr	Ahr	aryl-hydrocarbon receptor	11622	https://www.ncbi.nlm.nih.gov/gene/?term=11622
Trib3	Trib3	tribbles pseudokinase 3	228775	https://www.ncbi.nlm.nih.gov/gene/?term=228775
Sdc2	Sdc2	syndecan 2	15529	https://www.ncbi.nlm.nih.gov/gene/?term=15529
Slain2	Slain2	SLAIN motif family, member 2	75991	https://www.ncbi.nlm.nih.gov/gene/?term=75991
Casp2	Casp2	caspase 2	12366	https://www.ncbi.nlm.nih.gov/gene/?term=12366
Arhgap24	Arhgap24	Rho GTPase activating protein 24	231532	https://www.ncbi.nlm.nih.gov/gene/?term=231532
Ebf3	Ebf3	early B cell factor 3	13593	https://www.ncbi.nlm.nih.gov/gene/?term=13593
Dgkh	Dgkh	diacylglycerol kinase, eta	380921	https://www.ncbi.nlm.nih.gov/gene/?term=380921
Tes	Tes	testis derived transcript	21753	https://www.ncbi.nlm.nih.gov/gene/?term=21753
Ntn4	Ntn4	netrin 4	57764	https://www.ncbi.nlm.nih.gov/gene/?term=57764
Map2k1	Map2k1	mitogen-activated protein kinase kinase 1	26395	https://www.ncbi.nlm.nih.gov/gene/?term=26395
Hsf5	Hsf5	heat shock transcription factor family member 5	327992	https://www.ncbi.nlm.nih.gov/gene/?term=327992
Rassf8	Rassf8	Ras association (RalGDS/AF-6) domain family (N-terminal) member 8	71323	https://www.ncbi.nlm.nih.gov/gene/?term=71323
Plppr4	Plppr4	phospholipid phosphatase related 4	229791	https://www.ncbi.nlm.nih.gov/gene/?term=229791
Abcd1	Abcd1	ATP-binding cassette, sub-family D (ALD), member 1	11666	https://www.ncbi.nlm.nih.gov/gene/?term=11666
Tut4	Tut4	terminal uridylyl transferase 4	230594	https://www.ncbi.nlm.nih.gov/gene/?term=230594
Zcchc3	Zcchc3	zinc finger, CCHC domain containing 3	67917	https://www.ncbi.nlm.nih.gov/gene/?term=67917
B4galnt1	B4galnt1	beta-1,4-N-acetyl-galactosaminyl transferase 1	14421	https://www.ncbi.nlm.nih.gov/gene/?term=14421

Cpeb1	Cpeb1	cytoplasmic polyadenylation element binding protein 1	12877	https://www.ncbi.nlm.nih.gov/gene/?term=12877
Vat1l	Vat1l	vesicle amine transport protein 1 like	270097	https://www.ncbi.nlm.nih.gov/gene/?term=270097
Gad2	Gad2	glutamic acid decarboxylase 2	14417	https://www.ncbi.nlm.nih.gov/gene/?term=14417
Tac1	Tac1	tachykinin 1	21333	https://www.ncbi.nlm.nih.gov/gene/?term=21333
Ube2g1	Ube2g1	ubiquitin-conjugating enzyme E2G 1	67128	https://www.ncbi.nlm.nih.gov/gene/?term=67128
Galnt2	Galnt2	polypeptide N-acetylgalactosaminyltransferase 2	108148	https://www.ncbi.nlm.nih.gov/gene/?term=108148
Slc12a6	Slc12a6	solute carrier family 12, member 6	107723	https://www.ncbi.nlm.nih.gov/gene/?term=107723
Tmem198	Tmem198	transmembrane protein 198	319998	https://www.ncbi.nlm.nih.gov/gene/?term=319998
Farp1	Farp1	FERM, RhoGEF (Arhgef) and pleckstrin domain protein 1 (chondrocyte-derived)	223254	https://www.ncbi.nlm.nih.gov/gene/?term=223254
Arf2	Arf2	ADP-ribosylation factor 2	11841	https://www.ncbi.nlm.nih.gov/gene/?term=11841
Ankib1	Ankib1	ankyrin repeat and IBR domain containing 1	70797	https://www.ncbi.nlm.nih.gov/gene/?term=70797
Ezr	Ezr	ezrin	22350	https://www.ncbi.nlm.nih.gov/gene/?term=22350
Arpc5l	Arpc5l	actin related protein 2/3 complex, subunit 5-like	74192	https://www.ncbi.nlm.nih.gov/gene/?term=74192
Dlat	Dlat	dihydrolipoamide S-acetyltransferase (E2 component of pyruvate dehydrogenase complex)	235339	https://www.ncbi.nlm.nih.gov/gene/?term=235339
Tbr1	Tbr1	T-box brain gene 1	21375	https://www.ncbi.nlm.nih.gov/gene/?term=21375
Prrt3	Prrt3	proline-rich transmembrane protein 3	210673	https://www.ncbi.nlm.nih.gov/gene/?term=210673
Zfp449	Zfp449	zinc finger protein 449	78619	https://www.ncbi.nlm.nih.gov/gene/?term=78619
Nova2	Nova2	neuro-oncological ventral antigen 2	384569	https://www.ncbi.nlm.nih.gov/gene/?term=384569
Pnpla8	Pnpla8	patatin-like phospholipase domain containing 8	67452	https://www.ncbi.nlm.nih.gov/gene/?term=67452

Gpr21	Gpr21	G protein-coupled receptor 21	338346	https://www.ncbi.nlm.nih.gov/gene/?term=338346
Eif5	Eif5	NA	217869	https://www.ncbi.nlm.nih.gov/gene/?term=217869
Rapgef4	Rapgef4	Rap guanine nucleotide exchange factor (GEF) 4	56508	https://www.ncbi.nlm.nih.gov/gene/?term=56508
Spast	Spast	spastin	50850	https://www.ncbi.nlm.nih.gov/gene/?term=50850
Grhl2	Grhl2	grainyhead like transcription factor 2	252973	https://www.ncbi.nlm.nih.gov/gene/?term=252973
Stmnd1	Stmnd1	stathmin domain containing 1	380842	https://www.ncbi.nlm.nih.gov/gene/?term=380842
Uck2	Uck2	uridine-cytidine kinase 2	80914	https://www.ncbi.nlm.nih.gov/gene/?term=80914
Arsj	Arsj	arylsulfatase J	271970	https://www.ncbi.nlm.nih.gov/gene/?term=271970
Irs1	Irs1	insulin receptor substrate 1	16367	https://www.ncbi.nlm.nih.gov/gene/?term=16367
Jmjd1c	Jmjd1c	jumonji domain containing 1C	108829	https://www.ncbi.nlm.nih.gov/gene/?term=108829
Unc13c	Unc13c	unc-13 homolog C	208898	https://www.ncbi.nlm.nih.gov/gene/?term=208898
2610507B11Rik	2610507B11Rik	RIKEN cDNA 2610507B11 gene	72503	https://www.ncbi.nlm.nih.gov/gene/?term=72503
Adgra2	Adgra2	adhesion G protein-coupled receptor A2	78560	https://www.ncbi.nlm.nih.gov/gene/?term=78560
Bsdc1	Bsdc1	BSD domain containing 1	100383	https://www.ncbi.nlm.nih.gov/gene/?term=100383
Frmd5	Frmd5	FERM domain containing 5	228564	https://www.ncbi.nlm.nih.gov/gene/?term=228564
Epha3	Epha3	Eph receptor A3	13837	https://www.ncbi.nlm.nih.gov/gene/?term=13837
Snx16	Snx16	sorting nexin 16	74718	https://www.ncbi.nlm.nih.gov/gene/?term=74718
Ppp4r3a	Ppp4r3a	protein phosphatase 4 regulatory subunit 3A	68734	https://www.ncbi.nlm.nih.gov/gene/?term=68734
Rab3c	Rab3c	RAB3C, member RAS oncogene family	67295	https://www.ncbi.nlm.nih.gov/gene/?term=67295
Nlgn2	Nlgn2	neuroligin 2	216856	https://www.ncbi.nlm.nih.gov/gene/?term=216856
Nptx2	Nptx2	neuronal pentraxin 2	53324	https://www.ncbi.nlm.nih.gov/gene/?term=53324
Ctdsp1	Ctdsp1	CTD (carboxy-terminal domain, RNA polymerase II, polypeptide A) small phosphatase 1	227292	https://www.ncbi.nlm.nih.gov/gene/?term=227292
Spen	Spen	spen family transcription repressor	56381	https://www.ncbi.nlm.nih.gov/gene/?term=56381

Biology Open • Supplementary information

Alk	Alk	anaplastic lymphoma kinase	11682	https://www.ncbi.nlm.nih.gov/gene/?term=11682
Oxsr1	Oxsr1	oxidative-stress responsive 1	108737	https://www.ncbi.nlm.nih.gov/gene/?term=108737
Galr1	Galr1	galanin receptor 1	14427	https://www.ncbi.nlm.nih.gov/gene/?term=14427
Umad1	Umad1	UMAP1-MVP12 associated (UMA) domain containing 1	100036521	https://www.ncbi.nlm.nih.gov/gene/?term=100036
Rnf138	Rnf138	ring finger protein 138	56515	https://www.ncbi.nlm.nih.gov/gene/?term=56515
Mcoln1	Mcoln1	mucolipin 1	94178	https://www.ncbi.nlm.nih.gov/gene/?term=94178
Ucp1	Ucp1	uncoupling protein 1 (mitochondrial, proton carrier)	22227	https://www.ncbi.nlm.nih.gov/gene/?term=22227
Pde1c	Pde1c	phosphodiesterase 1C	18575	https://www.ncbi.nlm.nih.gov/gene/?term=18575
Dyrk1a	Dyrk1a	dual-specificity tyrosine-(Y)-phosphorylation regulated kinase 1a	13548	https://www.ncbi.nlm.nih.gov/gene/?term=13548
Hyal4	Hyal4	hyaluronoglucosaminidase 4	77042	https://www.ncbi.nlm.nih.gov/gene/?term=77042
Glis3	Glis3	GLIS family zinc finger 3	226075	https://www.ncbi.nlm.nih.gov/gene/?term=226075
Slc43a1	Slc43a1	solute carrier family 43, member 1	72401	https://www.ncbi.nlm.nih.gov/gene/?term=72401
Pdp1	Pdp1	pyruvate dehyrogenase phosphatase catalytic subunit 1	381511	https://www.ncbi.nlm.nih.gov/gene/?term=381511
Tnfaip8	Tnfaip8	tumor necrosis factor, alpha-induced protein 8	106869	https://www.ncbi.nlm.nih.gov/gene/?term=106869
Ccdc120	Ccdc120	coiled-coil domain containing 120	54648	https://www.ncbi.nlm.nih.gov/gene/?term=54648
Efemp2	Efemp2	epidermal growth factor-containing fibulin-like extracellular matrix protein 2	58859	https://www.ncbi.nlm.nih.gov/gene/?term=58859
Nrg3	Nrg3	neuregulin 3	18183	https://www.ncbi.nlm.nih.gov/gene/?term=18183
Creb5	Creb5	cAMP responsive element binding protein 5	231991	https://www.ncbi.nlm.nih.gov/gene/?term=231991
St7	St7	suppression of tumorigenicity 7	64213	https://www.ncbi.nlm.nih.gov/gene/?term=64213
Tgfbr2	Tgfbr2	transforming growth factor, beta receptor II	21813	https://www.ncbi.nlm.nih.gov/gene/?term=21813

Napg	Napg	N-ethylmaleimide sensitive fusion protein attachment protein gamma	108123	https://www.ncbi.nlm.nih.gov/gene/?term=108123
Trim12c	Trim12c	tripartite motif-containing 12C	319236	https://www.ncbi.nlm.nih.gov/gene/?term=319236
P2rx7	P2rx7	purinergic receptor P2X, ligand-gated ion channel, 7	18439	https://www.ncbi.nlm.nih.gov/gene/?term=18439
Ssrp1	Ssrp1	structure specific recognition protein 1	20833	https://www.ncbi.nlm.nih.gov/gene/?term=20833
Nbea	Nbea	neurobeachin	26422	https://www.ncbi.nlm.nih.gov/gene/?term=26422
Lin7a	Lin7a	lin-7 homolog A (C. elegans)	108030	https://www.ncbi.nlm.nih.gov/gene/?term=108030
Gcnt2	Gcnt2	glucosaminyl (N-acetyl) transferase 2, I-branching enzyme	14538	https://www.ncbi.nlm.nih.gov/gene/?term=14538
Ap2a2	Ap2a2	adaptor-related protein complex 2, alpha 2 subunit	11772	https://www.ncbi.nlm.nih.gov/gene/?term=11772
Lgals3	Lgals3	lectin, galactose binding, soluble 3	16854	https://www.ncbi.nlm.nih.gov/gene/?term=16854
Adamts5	Adamts5	a disintegrin-like and metallopeptidase (reprolysin type) with thrombospondin type 1 motif, 5 (aggrecanase-2)	23794	https://www.ncbi.nlm.nih.gov/gene/?term=23794
Trp53inp1	Trp53inp1	transformation related protein 53 inducible nuclear protein 1	60599	https://www.ncbi.nlm.nih.gov/gene/?term=60599
Dnajc14	Dnajc14	DnaJ heat shock protein family (Hsp40) member C14	74330	https://www.ncbi.nlm.nih.gov/gene/?term=74330
Crtac1	Crtac1	cartilage acidic protein 1	72832	https://www.ncbi.nlm.nih.gov/gene/?term=72832
Gpatch11	Gpatch11	G patch domain containing 11	53951	https://www.ncbi.nlm.nih.gov/gene/?term=53951
Ccdc117	Ccdc117	coiled-coil domain containing 117	104479	https://www.ncbi.nlm.nih.gov/gene/?term=104479
Dmtf1	Dmtf1	cyclin D binding myb-like transcription factor 1	23857	https://www.ncbi.nlm.nih.gov/gene/?term=23857
Prrx1	Prrx1	paired related homeobox 1	18933	https://www.ncbi.nlm.nih.gov/gene/?term=18933

Biology Open • Supplementary information

Mgat3	Mgat3	mannoside acetylglucosaminyltransferase 3	17309	https://www.ncbi.nlm.nih.gov/gene/?term=17309
Ncoa7	Ncoa7	nuclear receptor coactivator 7	211329	https://www.ncbi.nlm.nih.gov/gene/?term=211329
Cox5a	Cox5a	cytochrome c oxidase subunit 5A	12858	https://www.ncbi.nlm.nih.gov/gene/?term=12858
Chp1	Chp1	calcineurin-like EF hand protein 1	56398	https://www.ncbi.nlm.nih.gov/gene/?term=56398
Gba	Gba	glucosidase, beta, acid	14466	https://www.ncbi.nlm.nih.gov/gene/?term=14466
Ttyh1	Ttyh1	tweety family member 1	57776	https://www.ncbi.nlm.nih.gov/gene/?term=57776
Rnf170	Rnf170	ring finger protein 170	77733	https://www.ncbi.nlm.nih.gov/gene/?term=77733
Sos1	Sos1	SOS Ras/Rac guanine nucleotide exchange factor 1	20662	https://www.ncbi.nlm.nih.gov/gene/?term=20662
Pid1	Pid1	phosphotyrosine interaction domain containing 1	98496	https://www.ncbi.nlm.nih.gov/gene/?term=98496
Ppargc1a	Ppargc1a	peroxisome proliferative activated receptor, gamma, coactivator 1 alpha	19017	https://www.ncbi.nlm.nih.gov/gene/?term=19017
Lamp1	Lamp1	lysosomal-associated membrane protein 1	16783	https://www.ncbi.nlm.nih.gov/gene/?term=16783
Spred1	Spred1	sprouty protein with EVH-1 domain 1, related sequence	114715	https://www.ncbi.nlm.nih.gov/gene/?term=114715
4930402H24Rik	4930402H24Rik	RIKEN cDNA 4930402H24 gene	228602	https://www.ncbi.nlm.nih.gov/gene/?term=228602
Dtwd1	Dtwd1	DTW domain containing 1	69185	https://www.ncbi.nlm.nih.gov/gene/?term=69185
Tox3	Tox3	TOX high mobility group box family member 3	244579	https://www.ncbi.nlm.nih.gov/gene/?term=244579
Spop	Spop	speckle-type POZ protein	20747	https://www.ncbi.nlm.nih.gov/gene/?term=20747
Fras1	Fras1	Fraser extracellular matrix complex subunit 1	231470	https://www.ncbi.nlm.nih.gov/gene/?term=231470
Slc38a9	Slc38a9	solute carrier family 38, member 9	268706	https://www.ncbi.nlm.nih.gov/gene/?term=268706
Sirt1	Sirt1	sirtuin 1	93759	https://www.ncbi.nlm.nih.gov/gene/?term=93759
Dppa2	Dppa2	developmental pluripotency	73703	https://www.ncbi.nlm.nih.gov/gene/?term=73703

Biology Open • Supplementary information

		associated 2		
Abcb10	Abcb10	ATP-binding cassette, sub-family B (MDR/TAP), member 10	56199	https://www.ncbi.nlm.nih.gov/gene/?term=56199
Abraxas2	Abraxas2	BRISC complex subunit	109359	https://www.ncbi.nlm.nih.gov/gene/?term=109359
Wdr46	Wdr46	WD repeat domain 46	57315	https://www.ncbi.nlm.nih.gov/gene/?term=57315
Rhobtb3	Rhobtb3	Rho-related BTB domain containing 3	73296	https://www.ncbi.nlm.nih.gov/gene/?term=73296
Lsm5	Lsm5	LSM5 homolog, U6 small nuclear RNA and mRNA degradation associated	66373	https://www.ncbi.nlm.nih.gov/gene/?term=66373
Nr3c2	Nr3c2	nuclear receptor subfamily 3, group C, member 2	110784	https://www.ncbi.nlm.nih.gov/gene/?term=110784
Pcgf3	Pcgf3	polycomb group ring finger 3	69587	https://www.ncbi.nlm.nih.gov/gene/?term=69587
Zfp423	Zfp423	zinc finger protein 423	94187	https://www.ncbi.nlm.nih.gov/gene/?term=94187
Gpbp1l1	Gpbp1l1	GC-rich promoter binding protein 1-like 1	77110	https://www.ncbi.nlm.nih.gov/gene/?term=77110
Arhgap11a	Arhgap11a	Rho GTPase activating protein 11A	228482	https://www.ncbi.nlm.nih.gov/gene/?term=228482
Grk4	Grk4	G protein-coupled receptor kinase 4	14772	https://www.ncbi.nlm.nih.gov/gene/?term=14772
Fbn2	Fbn2	fibrillin 2	14119	https://www.ncbi.nlm.nih.gov/gene/?term=14119
Dpy19l4	Dpy19l4	dpy-19-like 4 (C. elegans)	381510	https://www.ncbi.nlm.nih.gov/gene/?term=381510
Rell1	Rell1	RELT-like 1	100532	https://www.ncbi.nlm.nih.gov/gene/?term=100532
Plcb1	Plcb1	phospholipase C, beta 1	18795	https://www.ncbi.nlm.nih.gov/gene/?term=18795
Rab22a	Rab22a	RAB22A, member RAS oncogene family	19334	https://www.ncbi.nlm.nih.gov/gene/?term=19334
Camk1	Camk1	calcium/calmodulin-dependent protein kinase I	52163	https://www.ncbi.nlm.nih.gov/gene/?term=52163
Mapre2	Mapre2	microtubule-associated protein, RP/EB family, member 2	212307	https://www.ncbi.nlm.nih.gov/gene/?term=212307
Slc39a11	Slc39a11	solute carrier family 39 (metal ion transporter), member 11	69806	https://www.ncbi.nlm.nih.gov/gene/?term=69806

Biology Open • Supplementary information

Larp4b	Larp4b	La ribonucleoprotein domain family, member 4B	217980	https://www.ncbi.nlm.nih.gov/gene/?term=217980
Dera	Dera	deoxyribose-phosphate aldolase (putative)	232449	https://www.ncbi.nlm.nih.gov/gene/?term=232449
Jarid2	Jarid2	jumonji, AT rich interactive domain 2	16468	https://www.ncbi.nlm.nih.gov/gene/?term=16468
Shc1	Shc1	src homology 2 domain-containing transforming protein C1	20416	https://www.ncbi.nlm.nih.gov/gene/?term=20416
Clmp	Clmp	CXADR-like membrane protein	71566	https://www.ncbi.nlm.nih.gov/gene/?term=71566
Fry	Fry	FRY microtubule binding protein	320365	https://www.ncbi.nlm.nih.gov/gene/?term=320365
Itpr1	Itpr1	inositol 1,4,5-trisphosphate receptor 1	16438	https://www.ncbi.nlm.nih.gov/gene/?term=16438
Ssr3	Ssr3	signal sequence receptor, gamma	67437	https://www.ncbi.nlm.nih.gov/gene/?term=67437
Dnm2	Dnm2	dynamin 2	13430	https://www.ncbi.nlm.nih.gov/gene/?term=13430
Ptprd	Ptprd	protein tyrosine phosphatase, receptor type, D	19266	https://www.ncbi.nlm.nih.gov/gene/?term=19266
Pde12	Pde12	phosphodiesterase 12	211948	https://www.ncbi.nlm.nih.gov/gene/?term=211948
Mlxip	Mlxip	MLX interacting protein	208104	https://www.ncbi.nlm.nih.gov/gene/?term=208104
Smim13	Smim13	small integral membrane protein 13	108934	https://www.ncbi.nlm.nih.gov/gene/?term=108934
Ephb6	Ephb6	Eph receptor B6	13848	https://www.ncbi.nlm.nih.gov/gene/?term=13848
Epha4	Epha4	Eph receptor A4	13838	https://www.ncbi.nlm.nih.gov/gene/?term=13838
Hapln1	Hapln1	hyaluronan and proteoglycan link protein 1	12950	https://www.ncbi.nlm.nih.gov/gene/?term=12950
Arl8b	Arl8b	ADP-ribosylation factor-like 8B	67166	https://www.ncbi.nlm.nih.gov/gene/?term=67166
Hoxc8	Hoxc8	homeobox C8	15426	https://www.ncbi.nlm.nih.gov/gene/?term=15426
Ankfy1	Ankfy1	ankyrin repeat and FYVE domain containing 1	11736	https://www.ncbi.nlm.nih.gov/gene/?term=11736
Lrrn4cl	Lrrn4cl	LRRN4 C-terminal like	68852	https://www.ncbi.nlm.nih.gov/gene/?term=68852
Fam126a	Fam126a	family with sequence similarity 126, member A	84652	https://www.ncbi.nlm.nih.gov/gene/?term=84652

Arid3b	Arid3b	AT rich interactive domain 3B (BRIGHT-like)	56380	https://www.ncbi.nlm.nih.gov/gene/?term=56380
Zfp629	Zfp629	zinc finger protein 629	320683	https://www.ncbi.nlm.nih.gov/gene/?term=320683
Tmem164	Tmem164	transmembrane protein 164	209497	https://www.ncbi.nlm.nih.gov/gene/?term=209497
Fam149a	Fam149a	family with sequence similarity 149, member A	212326	https://www.ncbi.nlm.nih.gov/gene/?term=212326
Rere	Rere	arginine glutamic acid dipeptide (RE) repeats	68703	https://www.ncbi.nlm.nih.gov/gene/?term=68703
Evc2	Evc2	EvC ciliary complex subunit 2	68525	https://www.ncbi.nlm.nih.gov/gene/?term=68525
Tmem263	Tmem263	transmembrane protein 263	103266	https://www.ncbi.nlm.nih.gov/gene/?term=103266
Elavl3	Elavl3	ELAV like RNA binding protein 3	15571	https://www.ncbi.nlm.nih.gov/gene/?term=15571
Pcdh12	Pcdh12	protocadherin 12	53601	https://www.ncbi.nlm.nih.gov/gene/?term=53601
Naa15	Naa15	N(alpha)-acetyltransferase 15, NatA auxiliary subunit	74838	https://www.ncbi.nlm.nih.gov/gene/?term=74838
Ankrd13a	Ankrd13a	ankyrin repeat domain 13a	68420	https://www.ncbi.nlm.nih.gov/gene/?term=68420
Mrps33	Mrps33	mitochondrial ribosomal protein S33	14548	https://www.ncbi.nlm.nih.gov/gene/?term=14548
Nanos1	Nanos1	nanos C2HC-type zinc finger 1	332397	https://www.ncbi.nlm.nih.gov/gene/?term=332397
Ccdc68	Ccdc68	coiled-coil domain containing 68	381175	https://www.ncbi.nlm.nih.gov/gene/?term=381175
Slc2a1	Slc2a1	solute carrier family 2 (facilitated glucose transporter), member 1	20525	https://www.ncbi.nlm.nih.gov/gene/?term=20525
Thy1	Thy1	thymus cell antigen 1, theta	21838	https://www.ncbi.nlm.nih.gov/gene/?term=21838
Sgk1	Sgk1	serum/glucocorticoid regulated kinase 1	20393	https://www.ncbi.nlm.nih.gov/gene/?term=20393
Tmem167	Tmem167	transmembrane protein 167	66074	https://www.ncbi.nlm.nih.gov/gene/?term=66074
Kdm7a	Kdm7a	lysine (K)-specific demethylase 7A	338523	https://www.ncbi.nlm.nih.gov/gene/?term=338523
Tfrc	Tfrc	transferrin receptor	22042	https://www.ncbi.nlm.nih.gov/gene/?term=22042
Fastkd2	Fastkd2	FAST kinase domains 2	75619	https://www.ncbi.nlm.nih.gov/gene/?term=75619
Arpc1a	Arpc1a	actin related protein 2/3 complex, subunit 1A	56443	https://www.ncbi.nlm.nih.gov/gene/?term=56443

Zfp106	Zfp106	zinc finger protein 106	20402	https://www.ncbi.nlm.nih.gov/gene/?term=20402
Pigk	Pigk	phosphatidylinositol glycan anchor biosynthesis, class K	329777	https://www.ncbi.nlm.nih.gov/gene/?term=329777
Nedd9	Nedd9	neural precursor cell expressed, developmentally down-regulated gene 9	18003	https://www.ncbi.nlm.nih.gov/gene/?term=18003
Zfp850	Zfp850	zinc finger protein 850	100043772	https://www.ncbi.nlm.nih.gov/gene/?term=100043
Rd3	Rd3	retinal degeneration 3	74023	https://www.ncbi.nlm.nih.gov/gene/?term=74023
Hmbs	Hmbs	hydroxymethylbilane synthase	15288	https://www.ncbi.nlm.nih.gov/gene/?term=15288
Mctp1	Mctp1	multiple C2 domains, transmembrane 1	78771	https://www.ncbi.nlm.nih.gov/gene/?term=78771
Ddx19b	Ddx19b	DEAD (Asp-Glu-Ala-Asp) box polypeptide 19b	234733	https://www.ncbi.nlm.nih.gov/gene/?term=234733
Naa50	Naa50	N(alpha)-acetyltransferase 50, NatE catalytic subunit	72117	https://www.ncbi.nlm.nih.gov/gene/?term=72117
Epdr1	Epdr1	ependymin related protein 1 (zebrafish)	105298	https://www.ncbi.nlm.nih.gov/gene/?term=105298
Slc36a4	Slc36a4	solute carrier family 36 (proton/amino acid symporter), member 4	234967	https://www.ncbi.nlm.nih.gov/gene/?term=234967
Cdr1	Cdr1	cerebellar degeneration related antigen 1	631990	https://www.ncbi.nlm.nih.gov/gene/?term=631990
Fbxo11	Fbxo11	F-box protein 11	225055	https://www.ncbi.nlm.nih.gov/gene/?term=225055
4932429P05Rik	4932429P05Rik	RIKEN cDNA 4932429P05 gene	245509	https://www.ncbi.nlm.nih.gov/gene/?term=245509
Cdkn1b	Cdkn1b	cyclin-dependent kinase inhibitor 1B	12576	https://www.ncbi.nlm.nih.gov/gene/?term=12576
Trib2	Trib2	tribbles pseudokinase 2	217410	https://www.ncbi.nlm.nih.gov/gene/?term=217410
Itprid2	Itprid2	ITPR interacting domain containing 2	70599	https://www.ncbi.nlm.nih.gov/gene/?term=70599
Fam135a	Fam135a	family with sequence similarity 135, member A	68187	https://www.ncbi.nlm.nih.gov/gene/?term=68187

Biology Open • Supplementary information

Daam2	Daam2	dishevelled associated activator of morphogenesis 2	76441	https://www.ncbi.nlm.nih.gov/gene/?term=76441
Gpatch2l	Gpatch2l	G patch domain containing 2 like	70373	https://www.ncbi.nlm.nih.gov/gene/?term=70373
Scarb2	Scarb2	scavenger receptor class B, member 2	12492	https://www.ncbi.nlm.nih.gov/gene/?term=12492
Myo1c	Myo1c	myosin IC	17913	https://www.ncbi.nlm.nih.gov/gene/?term=17913
Brdt	Brdt	bromodomain, testis-specific	114642	https://www.ncbi.nlm.nih.gov/gene/?term=114642
Pcyt1a	Pcyt1a	phosphate cytidylyltransferase 1, choline, alpha isoform	13026	https://www.ncbi.nlm.nih.gov/gene/?term=13026
Mfsd12	Mfsd12	major facilitator superfamily domain containing 12	73822	https://www.ncbi.nlm.nih.gov/gene/?term=73822
Unc5c	Unc5c	unc-5 netrin receptor C	22253	https://www.ncbi.nlm.nih.gov/gene/?term=22253
Ptpmt1	Ptpmt1	protein tyrosine phosphatase, mitochondrial 1	66461	https://www.ncbi.nlm.nih.gov/gene/?term=66461
6430571L13Rik	6430571L13Rik	RIKEN cDNA 6430571L13 gene	235599	https://www.ncbi.nlm.nih.gov/gene/?term=235599
Pak6	Pak6	p21 (RAC1) activated kinase 6	214230	https://www.ncbi.nlm.nih.gov/gene/?term=214230
Slc24a3	Slc24a3	solute carrier family 24 (sodium/potassium/calcium exchanger), member 3	94249	https://www.ncbi.nlm.nih.gov/gene/?term=94249
Erbb4	Erbb4	erb-b2 receptor tyrosine kinase 4	13869	https://www.ncbi.nlm.nih.gov/gene/?term=13869
Tbck	Tbck	TBC1 domain containing kinase	271981	https://www.ncbi.nlm.nih.gov/gene/?term=271981
Slc15a1	Slc15a1	solute carrier family 15 (oligopeptide transporter), member 1	56643	https://www.ncbi.nlm.nih.gov/gene/?term=56643
Tgfbr3	Tgfbr3	transforming growth factor, beta receptor III	21814	https://www.ncbi.nlm.nih.gov/gene/?term=21814
Dtx2	Dtx2	deltex 2, E3 ubiquitin ligase	74198	https://www.ncbi.nlm.nih.gov/gene/?term=74198
Masp1	Masp1	mannan-binding lectin serine peptidase 1	17174	https://www.ncbi.nlm.nih.gov/gene/?term=17174

Biology Open • Supplementary information

Arid3a	Arid3a	AT rich interactive domain 3A (BRIGHT-like)	13496	https://www.ncbi.nlm.nih.gov/gene/?term=13496
Wnt9a	Wnt9a	wingless-type MMTV integration site family, member 9A	216795	https://www.ncbi.nlm.nih.gov/gene/?term=216795
Senp2	Senp2	SUMO/sentrin specific peptidase 2	75826	https://www.ncbi.nlm.nih.gov/gene/?term=75826
Slc10a7	Slc10a7	solute carrier family 10 (sodium/bile acid cotransporter family), member 7	76775	https://www.ncbi.nlm.nih.gov/gene/?term=76775
Nme6	Nme6	NME/NM23 nucleoside diphosphate kinase 6	54369	https://www.ncbi.nlm.nih.gov/gene/?term=54369
Mapk6	Mapk6	mitogen-activated protein kinase 6	50772	https://www.ncbi.nlm.nih.gov/gene/?term=50772
Sall4	Sall4	spalt like transcription factor 4	99377	https://www.ncbi.nlm.nih.gov/gene/?term=99377
Gdf6	Gdf6	growth differentiation factor 6	242316	https://www.ncbi.nlm.nih.gov/gene/?term=242316
Fbxl12	Fbxl12	F-box and leucine-rich repeat protein 12	30843	https://www.ncbi.nlm.nih.gov/gene/?term=30843
Ddx19a	Ddx19a	DEAD (Asp-Glu-Ala-Asp) box polypeptide 19a	13680	https://www.ncbi.nlm.nih.gov/gene/?term=13680
Rgs6	Rgs6	regulator of G-protein signaling 6	50779	https://www.ncbi.nlm.nih.gov/gene/?term=50779
Fasl	Fasl	Fas ligand (TNF superfamily, member 6)	14103	https://www.ncbi.nlm.nih.gov/gene/?term=14103
Hip1	Hip1	huntingtin interacting protein 1	215114	https://www.ncbi.nlm.nih.gov/gene/?term=215114
Pappa	Pappa	pregnancy-associated plasma protein A	18491	https://www.ncbi.nlm.nih.gov/gene/?term=18491
Bach1	Bach1	BTB and CNC homology 1, basic leucine zipper transcription factor 1	12013	https://www.ncbi.nlm.nih.gov/gene/?term=12013
G6pc2	G6pc2	glucose-6-phosphatase, catalytic, 2	14378	https://www.ncbi.nlm.nih.gov/gene/?term=14378
Eef2k	Eef2k	eukaryotic elongation factor-2 kinase	13631	https://www.ncbi.nlm.nih.gov/gene/?term=13631
Lin28a	Lin28a	lin-28 homolog A (C. elegans)	83557	https://www.ncbi.nlm.nih.gov/gene/?term=83557
Gpatch3	Gpatch3	G patch domain containing 3	242691	https://www.ncbi.nlm.nih.gov/gene/?term=242691

Biology Open • Supplementary information

Klk10	Klk10	kallikrein related-peptidase 10	69540	https://www.ncbi.nlm.nih.gov/gene/?term=69540
Pbx3	Pbx3	pre B cell leukemia homeobox 3	18516	https://www.ncbi.nlm.nih.gov/gene/?term=18516
Edn1	Edn1	endothelin 1	13614	https://www.ncbi.nlm.nih.gov/gene/?term=13614
Bcat1	Bcat1	branched chain aminotransferase 1, cytosolic	12035	https://www.ncbi.nlm.nih.gov/gene/?term=12035
Zswim5	Zswim5	zinc finger SWIM-type containing 5	74464	https://www.ncbi.nlm.nih.gov/gene/?term=74464
Col4a2	Col4a2	collagen, type IV, alpha 2	12827	https://www.ncbi.nlm.nih.gov/gene/?term=12827
Hif1an	Hif1an	hypoxia-inducible factor 1, alpha subunit inhibitor	319594	https://www.ncbi.nlm.nih.gov/gene/?term=319594
Stard13	Stard13	StAR-related lipid transfer (START) domain containing 13	243362	https://www.ncbi.nlm.nih.gov/gene/?term=243362
Nr6a1	Nr6a1	nuclear receptor subfamily 6, group A, member 1	14536	https://www.ncbi.nlm.nih.gov/gene/?term=14536
Adamts8	Adamts8	a disintegrin-like and metallopeptidase (reprolysin type) with thrombospondin type 1 motif, 8	30806	https://www.ncbi.nlm.nih.gov/gene/?term=30806
Ahctf1	Ahctf1	AT hook containing transcription factor 1	226747	https://www.ncbi.nlm.nih.gov/gene/?term=226747
Fign	Fign	fidgetin	60344	https://www.ncbi.nlm.nih.gov/gene/?term=60344
Acvr1c	Acvr1c	activin A receptor, type IC	269275	https://www.ncbi.nlm.nih.gov/gene/?term=269275
Map3k1	Map3k1	mitogen-activated protein kinase kinase kinase 1	26401	https://www.ncbi.nlm.nih.gov/gene/?term=26401
Cdc34	Cdc34	cell division cycle 34	216150	https://www.ncbi.nlm.nih.gov/gene/?term=216150
Cldn12	Cldn12	claudin 12	64945	https://www.ncbi.nlm.nih.gov/gene/?term=64945
Pald1	Pald1	phosphatase domain containing, paladin 1	27355	https://www.ncbi.nlm.nih.gov/gene/?term=27355
Zc3hav1l	Zc3hav1l	zinc finger CCCH-type, antiviral 1-like	209032	https://www.ncbi.nlm.nih.gov/gene/?term=209032
Trim67	Trim67	tripartite motif-containing 67	330863	https://www.ncbi.nlm.nih.gov/gene/?term=330863
Skil	Skil	SKI-like	20482	https://www.ncbi.nlm.nih.gov/gene/?term=20482

Biology Open • Supplementary information

Vstm5	Vstm5	V-set and transmembrane domain containing 5	69137	https://www.ncbi.nlm.nih.gov/gene/?term=69137
Arid3c	Arid3c	AT rich interactive domain 3C (BRIGHT-like)	550619	https://www.ncbi.nlm.nih.gov/gene/?term=550619
Ptafr	Ptafr	platelet-activating factor receptor	19204	https://www.ncbi.nlm.nih.gov/gene/?term=19204
Igdcc3	Igdcc3	immunoglobulin superfamily, DCC subclass, member 3	19289	https://www.ncbi.nlm.nih.gov/gene/?term=19289
Plekha8	Plekha8	pleckstrin homology domain containing, family A (phosphoinositide binding specific) member 8	231999	https://www.ncbi.nlm.nih.gov/gene/?term=231999
Cpeb2	Cpeb2	cytoplasmic polyadenylation element binding protein 2	231207	https://www.ncbi.nlm.nih.gov/gene/?term=231207
Pla2g3	Pla2g3	phospholipase A2, group III	237625	https://www.ncbi.nlm.nih.gov/gene/?term=237625
Lrig2	Lrig2	leucine-rich repeats and immunoglobulin-like domains 2	269473	https://www.ncbi.nlm.nih.gov/gene/?term=269473
Prpf38b	Prpf38b	PRP38 pre-mRNA processing factor 38 (yeast) domain containing B	66921	https://www.ncbi.nlm.nih.gov/gene/?term=66921
Rufy3	Rufy3	RUN and FYVE domain containing 3	52822	https://www.ncbi.nlm.nih.gov/gene/?term=52822
Zfp583	Zfp583	zinc finger protein 583	213011	https://www.ncbi.nlm.nih.gov/gene/?term=213011
Fgd6	Fgd6	FYVE, RhoGEF and PH domain containing 6	13998	https://www.ncbi.nlm.nih.gov/gene/?term=13998
Arhgap12	Arhgap12	Rho GTPase activating protein 12	75415	https://www.ncbi.nlm.nih.gov/gene/?term=75415
Map4k3	Map4k3	mitogen-activated protein kinase kinase kinase kinase 3	225028	https://www.ncbi.nlm.nih.gov/gene/?term=225028
Adrb3	Adrb3	adrenergic receptor, beta 3	11556	https://www.ncbi.nlm.nih.gov/gene/?term=11556
Elp1	Elp1	elongator complex protein 1	230233	https://www.ncbi.nlm.nih.gov/gene/?term=230233
Rab11fip4	Rab11fip4	RAB11 family interacting protein 4 (class II)	268451	https://www.ncbi.nlm.nih.gov/gene/?term=268451

Pbx1	Pbx1	pre B cell leukemia homeobox 1	18514	https://www.ncbi.nlm.nih.gov/gene/?term=18514
Adamts12	Adamts12	a disintegrin-like and metallopeptidase (reprolysin type) with thrombospondin type 1 motif, 12	239337	https://www.ncbi.nlm.nih.gov/gene/?term=239337
Soat2	Soat2	sterol O-acyltransferase 2	223920	https://www.ncbi.nlm.nih.gov/gene/?term=223920
Thoc2	Thoc2	THO complex 2	331401	https://www.ncbi.nlm.nih.gov/gene/?term=331401
Gatm	Gatm	glycine amidinotransferase (L-arginine:glycine amidinotransferase)	67092	https://www.ncbi.nlm.nih.gov/gene/?term=67092
Hic2	Hic2	hypermethylated in cancer 2	58180	https://www.ncbi.nlm.nih.gov/gene/?term=58180
Pik3ip1	Pik3ip1	phosphoinositide-3-kinase interacting protein 1	216505	https://www.ncbi.nlm.nih.gov/gene/?term=216505
Tmem65	Tmem65	transmembrane protein 65	74868	https://www.ncbi.nlm.nih.gov/gene/?term=74868
Igf2bp3	Igf2bp3	insulin-like growth factor 2 mRNA binding protein 3	140488	https://www.ncbi.nlm.nih.gov/gene/?term=140488
Slc2a12	Slc2a12	solute carrier family 2 (facilitated glucose transporter), member 12	353169	https://www.ncbi.nlm.nih.gov/gene/?term=353169
Zfyve26	Zfyve26	zinc finger, FYVE domain containing 26	211978	https://www.ncbi.nlm.nih.gov/gene/?term=211978
Igf2bp1	Igf2bp1	insulin-like growth factor 2 mRNA binding protein 1	140486	https://www.ncbi.nlm.nih.gov/gene/?term=140486
Cemip2	Cemip2	cell migration inducing hyaluronidase 2	83921	https://www.ncbi.nlm.nih.gov/gene/?term=83921
Limd2	Limd2	LIM domain containing 2	67803	https://www.ncbi.nlm.nih.gov/gene/?term=67803
Col3a1	Col3a1	collagen, type III, alpha 1	12825	https://www.ncbi.nlm.nih.gov/gene/?term=12825
Zbtb5	Zbtb5	zinc finger and BTB domain containing 5	230119	https://www.ncbi.nlm.nih.gov/gene/?term=230119
9930012K11Rik	9930012K11Rik	RIKEN cDNA 9930012K11 gene	268759	https://www.ncbi.nlm.nih.gov/gene/?term=268759
Cd200r1	Cd200r1	CD200 receptor 1	57781	https://www.ncbi.nlm.nih.gov/gene/?term=57781

Biology Open • Supplementary information

Ints6l	Ints6l	integrator complex subunit 6 like	236790	https://www.ncbi.nlm.nih.gov/gene/?term=236790
Ppp2r2a	Ppp2r2a	protein phosphatase 2, regulatory subunit B, alpha	71978	https://www.ncbi.nlm.nih.gov/gene/?term=71978
Il6	Il6	interleukin 6	16193	https://www.ncbi.nlm.nih.gov/gene/?term=16193
Cntrl	Cntrl	centriolin	26920	https://www.ncbi.nlm.nih.gov/gene/?term=26920
Hand1	Hand1	heart and neural crest derivatives expressed 1	15110	https://www.ncbi.nlm.nih.gov/gene/?term=15110
Trim71	Trim71	tripartite motif-containing 71	636931	https://www.ncbi.nlm.nih.gov/gene/?term=636931
Ccr7	Ccr7	chemokine (C-C motif) receptor 7	12775	https://www.ncbi.nlm.nih.gov/gene/?term=12775
Zfp512b	Zfp512b	zinc finger protein 512B	269401	https://www.ncbi.nlm.nih.gov/gene/?term=269401
Stx3	Stx3	syntaxin 3	20908	https://www.ncbi.nlm.nih.gov/gene/?term=20908
Ercc6	Ercc6	excision repair cross-complementing rodent repair deficiency, complementation group 6	319955	https://www.ncbi.nlm.nih.gov/gene/?term=319955
Fam189a1	Fam189a1	family with sequence similarity 189, member A1	70638	https://www.ncbi.nlm.nih.gov/gene/?term=70638
Stk40	Stk40	serine/threonine kinase 40	74178	https://www.ncbi.nlm.nih.gov/gene/?term=74178
Trim6	Trim6	tripartite motif-containing 6	94088	https://www.ncbi.nlm.nih.gov/gene/?term=94088
Lin28b	Lin28b	lin-28 homolog B (C. elegans)	380669	https://www.ncbi.nlm.nih.gov/gene/?term=380669
Nras	Nras	neuroblastoma ras oncogene	18176	https://www.ncbi.nlm.nih.gov/gene/?term=18176
Coil	Coil	coilin	12812	https://www.ncbi.nlm.nih.gov/gene/?term=12812
Usp38	Usp38	ubiquitin specific peptidase 38	74841	https://www.ncbi.nlm.nih.gov/gene/?term=74841
Igdcc4	Igdcc4	immunoglobulin superfamily, DCC subclass, member 4	56741	https://www.ncbi.nlm.nih.gov/gene/?term=56741
Galnt1	Galnt1	polypeptide N-acetylgalactosaminyltransferase 1	14423	https://www.ncbi.nlm.nih.gov/gene/?term=14423
Gng5	Gng5	guanine nucleotide binding protein (G protein), gamma 5	14707	https://www.ncbi.nlm.nih.gov/gene/?term=14707
Dtx4	Dtx4	deltex 4, E3 ubiquitin ligase	207521	https://www.ncbi.nlm.nih.gov/gene/?term=207521

Biology Open • Supplementary information

Slc25a27	Slc25a27	solute carrier family 25, member 27	74011	https://www.ncbi.nlm.nih.gov/gene/?term=74011
Yod1	Yod1	YOD1 deubiquitinase	226418	https://www.ncbi.nlm.nih.gov/gene/?term=226418
Kif2b	Kif2b	kinesin family member 2B	73470	https://www.ncbi.nlm.nih.gov/gene/?term=73470
Cbx5	Cbx5	chromobox 5	12419	https://www.ncbi.nlm.nih.gov/gene/?term=12419
Bzw1	Bzw1	basic leucine zipper and W2 domains 1	66882	https://www.ncbi.nlm.nih.gov/gene/?term=66882
Utrn	Utrn	utrophin	22288	https://www.ncbi.nlm.nih.gov/gene/?term=22288
Il13	Il13	interleukin 13	16163	https://www.ncbi.nlm.nih.gov/gene/?term=16163
Hmga2	Hmga2	high mobility group AT-hook 2	15364	https://www.ncbi.nlm.nih.gov/gene/?term=15364
Smim3	Smim3	small integral membrane protein 3	106878	https://www.ncbi.nlm.nih.gov/gene/?term=106878
Zfp275	Zfp275	zinc finger protein 275	27081	https://www.ncbi.nlm.nih.gov/gene/?term=27081
Tmem198b	Tmem198b	transmembrane protein 198b	73827	https://www.ncbi.nlm.nih.gov/gene/?term=73827
Tnfaip8l3	Tnfaip8l3	tumor necrosis factor, alpha-induced protein 8-like 3	244882	https://www.ncbi.nlm.nih.gov/gene/?term=244882
Tspan5	Tspan5	tetraspanin 5	56224	https://www.ncbi.nlm.nih.gov/gene/?term=56224
Fndc3a	Fndc3a	fibronectin type III domain containing 3A	319448	https://www.ncbi.nlm.nih.gov/gene/?term=319448
Adrb2	Adrb2	adrenergic receptor, beta 2	11555	https://www.ncbi.nlm.nih.gov/gene/?term=11555
Tmprss2	Tmprss2	transmembrane protease, serine 2	50528	https://www.ncbi.nlm.nih.gov/gene/?term=50528
Limk2	Limk2	LIM motif-containing protein kinase 2	16886	https://www.ncbi.nlm.nih.gov/gene/?term=16886
Slc17a6	Slc17a6	solute carrier family 17 (sodium-dependent inorganic phosphate cotransporter), member 6	140919	https://www.ncbi.nlm.nih.gov/gene/?term=140919
Zfp292	Zfp292	zinc finger protein 292	30046	https://www.ncbi.nlm.nih.gov/gene/?term=30046
Celf4	Celf4	CUGBP, Elav-like family member 4	108013	https://www.ncbi.nlm.nih.gov/gene/?term=108013
Dpep1	Dpep1	dipeptidase 1	13479	https://www.ncbi.nlm.nih.gov/gene/?term=13479
Trim36	Trim36	tripartite motif-containing 36	28105	https://www.ncbi.nlm.nih.gov/gene/?term=28105
Trps1	Trps1	transcriptional repressor GATA	83925	https://www.ncbi.nlm.nih.gov/gene/?term=83925

		binding 1		
Cebpa	Cebpa	CCAAT/enhancer binding protein (C/EBP), alpha	12606	https://www.ncbi.nlm.nih.gov/gene/?term=12606
Slc20a2	Slc20a2	solute carrier family 20, member 2	20516	https://www.ncbi.nlm.nih.gov/gene/?term=20516
Nlgn1	Nlgn1	neuroligin 1	192167	https://www.ncbi.nlm.nih.gov/gene/?term=192167
Serpinb9e	Serpinb9e	serine (or cysteine) peptidase inhibitor, clade B, member 9e	20710	https://www.ncbi.nlm.nih.gov/gene/?term=20710
Alkbh8	Alkbh8	alkB homolog 8, tRNA methyltransferase	67667	https://www.ncbi.nlm.nih.gov/gene/?term=67667
Tor1aip1	Tor1aip1	torsin A interacting protein 1	208263	https://www.ncbi.nlm.nih.gov/gene/?term=208263
Eda2r	Eda2r	ectodysplasin A2 receptor	245527	https://www.ncbi.nlm.nih.gov/gene/?term=245527
Serpinb9f	Serpinb9f	serine (or cysteine) peptidase inhibitor, clade B, member 9f	20709	https://www.ncbi.nlm.nih.gov/gene/?term=20709
Chd7	Chd7	chromodomain helicase DNA binding protein 7	320790	https://www.ncbi.nlm.nih.gov/gene/?term=320790
Epc2	Epc2	enhancer of polycomb homolog 2	227867	https://www.ncbi.nlm.nih.gov/gene/?term=227867
Zfp322a	Zfp322a	zinc finger protein 322A	218100	https://www.ncbi.nlm.nih.gov/gene/?term=218100
Setdb2	Setdb2	SET domain, bifurcated 2	239122	https://www.ncbi.nlm.nih.gov/gene/?term=239122
Mr1	Mr1	major histocompatibility complex, class I-related	15064	https://www.ncbi.nlm.nih.gov/gene/?term=15064
Tdrd5	Tdrd5	tudor domain containing 5	214575	https://www.ncbi.nlm.nih.gov/gene/?term=214575
Pigw	Pigw	phosphatidylinositol glycan anchor biosynthesis, class W	70325	https://www.ncbi.nlm.nih.gov/gene/?term=70325
Neurod1	Neurod1	neurogenic differentiation 1	18012	https://www.ncbi.nlm.nih.gov/gene/?term=18012
Setbp1	Setbp1	SET binding protein 1	240427	https://www.ncbi.nlm.nih.gov/gene/?term=240427
Smpdl3b	Smpdl3b	sphingomyelin phosphodiesterase, acid-like 3B	100340	https://www.ncbi.nlm.nih.gov/gene/?term=100340
Myo5a	Myo5a	myosin VA	17918	https://www.ncbi.nlm.nih.gov/gene/?term=17918
Aak1	Aak1	AP2 associated kinase 1	269774	https://www.ncbi.nlm.nih.gov/gene/?term=269774

Biology Open • Supplementary information

Tigd2	Tigd2	tigger transposable element derived 2	68140	https://www.ncbi.nlm.nih.gov/gene/?term=68140
Dcaf10	Dcaf10	DDB1 and CUL4 associated factor 10	242418	https://www.ncbi.nlm.nih.gov/gene/?term=242418
Psg29	Psg29	pregnancy-specific glycoprotein 29	114872	https://www.ncbi.nlm.nih.gov/gene/?term=114872
Ktn1	Ktn1	kinectin 1	16709	https://www.ncbi.nlm.nih.gov/gene/?term=16709
Cpa3	Cpa3	carboxypeptidase A3, mast cell	12873	https://www.ncbi.nlm.nih.gov/gene/?term=12873
Ap3s1	Ap3s1	adaptor-related protein complex 3, sigma 1 subunit	11777	https://www.ncbi.nlm.nih.gov/gene/?term=11777
Usp36	Usp36	ubiquitin specific peptidase 36	72344	https://www.ncbi.nlm.nih.gov/gene/?term=72344
Thrb	Thrb	thyroid hormone receptor beta	21834	https://www.ncbi.nlm.nih.gov/gene/?term=21834
Nppc	Nppc	natriuretic peptide type C	18159	https://www.ncbi.nlm.nih.gov/gene/?term=18159
Mtf2	Mtf2	metal response element binding transcription factor 2	17765	https://www.ncbi.nlm.nih.gov/gene/?term=17765
Mief2	Mief2	mitochondrial elongation factor 2	237781	https://www.ncbi.nlm.nih.gov/gene/?term=237781
Ank1	Ank1	ankyrin 1, erythroid	11733	https://www.ncbi.nlm.nih.gov/gene/?term=11733
Fem1c	Fem1c	fem-1 homolog c (C.elegans)	240263	https://www.ncbi.nlm.nih.gov/gene/?term=240263
Epha8	Epha8	Eph receptor A8	13842	https://www.ncbi.nlm.nih.gov/gene/?term=13842
Upf2	Upf2	UPF2 regulator of nonsense transcripts homolog (yeast)	326622	https://www.ncbi.nlm.nih.gov/gene/?term=326622
Fabp9	Fabp9	fatty acid binding protein 9, testis	21884	https://www.ncbi.nlm.nih.gov/gene/?term=21884
Chd6	Chd6	chromodomain helicase DNA binding protein 6	71389	https://www.ncbi.nlm.nih.gov/gene/?term=71389
St6galnac4	St6galnac4	ST6 (alpha-N-acetyl-neuraminyl-2,3-beta-galactosyl-1,3)-N-acetylgalactosaminide alpha-2,6-sialyltransferase 4	20448	https://www.ncbi.nlm.nih.gov/gene/?term=20448
Sh3gl2	Sh3gl2	SH3-domain GRB2-like 2	20404	https://www.ncbi.nlm.nih.gov/gene/?term=20404
Afap1	Afap1	actin filament associated protein 1	70292	https://www.ncbi.nlm.nih.gov/gene/?term=70292
Caln1	Caln1	calneuron 1	140904	https://www.ncbi.nlm.nih.gov/gene/?term=140904

Psd	Psd	pleckstrin and Sec7 domain containing	73728	https://www.ncbi.nlm.nih.gov/gene/?term=73728
Plxnb2	Plxnb2	plexin B2	140570	https://www.ncbi.nlm.nih.gov/gene/?term=140570
Nkapd1	Nkapd1	NKAP domain containing 1	270156	https://www.ncbi.nlm.nih.gov/gene/?term=270156
Zfp148	Zfp148	zinc finger protein 148	22661	https://www.ncbi.nlm.nih.gov/gene/?term=22661
Lsm14a	Lsm14a	LSM14A mRNA processing body assembly factor	67070	https://www.ncbi.nlm.nih.gov/gene/?term=67070
D17H6S53E	D17H6S53E	DNA segment, Chr 17, human D6S53E	114585	https://www.ncbi.nlm.nih.gov/gene/?term=114585
Unc5a	Unc5a	unc-5 netrin receptor A	107448	https://www.ncbi.nlm.nih.gov/gene/?term=107448
Lamc1	Lamc1	laminin, gamma 1	226519	https://www.ncbi.nlm.nih.gov/gene/?term=226519
Sorbs2	Sorbs2	sorbin and SH3 domain containing 2	234214	https://www.ncbi.nlm.nih.gov/gene/?term=234214
Scn8a	Scn8a	sodium channel, voltage-gated, type VIII, alpha	20273	https://www.ncbi.nlm.nih.gov/gene/?term=20273
Cnot6l	Cnot6l	CCR4-NOT transcription complex, subunit 6-like	231464	https://www.ncbi.nlm.nih.gov/gene/?term=231464
Klf11	Klf11	Kruppel-like factor 11	194655	https://www.ncbi.nlm.nih.gov/gene/?term=194655
Cped1	Cped1	cadherin-like and PC-esterase domain containing 1	214642	https://www.ncbi.nlm.nih.gov/gene/?term=214642
Clec1a	Clec1a	C-type lectin domain family 1, member a	243653	https://www.ncbi.nlm.nih.gov/gene/?term=243653
Nsmf	Nsmf	NMDA receptor synaptonuclear signaling and neuronal migration factor	56876	https://www.ncbi.nlm.nih.gov/gene/?term=56876
Sys1	Sys1	SYS1 Golgi-localized integral membrane protein homolog (S. cerevisiae)	66460	https://www.ncbi.nlm.nih.gov/gene/?term=66460
Zfp36l2	Zfp36l2	zinc finger protein 36, C3H type-like 2	12193	https://www.ncbi.nlm.nih.gov/gene/?term=12193
Atcay	Atcay	ataxia, cerebellar, Cayman type	16467	https://www.ncbi.nlm.nih.gov/gene/?term=16467

Biology Open • Supplementary information

Nfix	Nfix	nuclear factor I/X	18032	https://www.ncbi.nlm.nih.gov/gene/?term=18032
Sin3a	Sin3a	transcriptional regulator, SIN3A (yeast)	20466	https://www.ncbi.nlm.nih.gov/gene/?term=20466
Gng2	Gng2	guanine nucleotide binding protein (G protein), gamma 2	14702	https://www.ncbi.nlm.nih.gov/gene/?term=14702
L3mbtl3	L3mbtl3	L3MBTL3 histone methyl-lysine binding protein	237339	https://www.ncbi.nlm.nih.gov/gene/?term=237339
Clvs1	Clvs1	clavesin 1	74438	https://www.ncbi.nlm.nih.gov/gene/?term=74438
Tmtc4	Tmtc4	transmembrane and tetratricopeptide repeat containing 4	70551	https://www.ncbi.nlm.nih.gov/gene/?term=70551
Celf5	Celf5	CUGBP, Elav-like family member 5	319586	https://www.ncbi.nlm.nih.gov/gene/?term=319586
Fermt2	Fermt2	fermitin family member 2	218952	https://www.ncbi.nlm.nih.gov/gene/?term=218952
Sh2b3	Sh2b3	SH2B adaptor protein 3	16923	https://www.ncbi.nlm.nih.gov/gene/?term=16923
Vezf1	Vezf1	vascular endothelial zinc finger 1	22344	https://www.ncbi.nlm.nih.gov/gene/?term=22344
Uhrf1bp1	Uhrf1bp1	UHRF1 (ICBP90) binding protein 1	224648	https://www.ncbi.nlm.nih.gov/gene/?term=224648
Has3	Has3	hyaluronan synthase 3	15118	https://www.ncbi.nlm.nih.gov/gene/?term=15118
Syn3	Syn3	synapsin III	27204	https://www.ncbi.nlm.nih.gov/gene/?term=27204
Arhgap6	Arhgap6	Rho GTPase activating protein 6	11856	https://www.ncbi.nlm.nih.gov/gene/?term=11856
Pxmp4	Pxmp4	peroxisomal membrane protein 4	59038	https://www.ncbi.nlm.nih.gov/gene/?term=59038
Slc6a8	Slc6a8	solute carrier family 6 (neurotransmitter transporter, creatine), member 8	102857	https://www.ncbi.nlm.nih.gov/gene/?term=102857
Corin	Corin	corin	53419	https://www.ncbi.nlm.nih.gov/gene/?term=53419
1700017B05Rik	1700017B05Rik	RIKEN cDNA 1700017B05 gene	74211	https://www.ncbi.nlm.nih.gov/gene/?term=74211
Dcp1a	Dcp1a	decapping mRNA 1A	75901	https://www.ncbi.nlm.nih.gov/gene/?term=75901
Sptb	Sptb	spectrin beta, erythrocytic	20741	https://www.ncbi.nlm.nih.gov/gene/?term=20741
Lzts3	Lzts3	leucine zipper, putative tumor suppressor family member 3	241638	https://www.ncbi.nlm.nih.gov/gene/?term=241638
Zfp385a	Zfp385a	zinc finger protein 385A	29813	https://www.ncbi.nlm.nih.gov/gene/?term=29813

Biology Open • Supplementary information

Mcu	Mcu	mitochondrial calcium uniporter	215999	https://www.ncbi.nlm.nih.gov/gene/?term=215999
Slx4	Slx4	SLX4 structure-specific endonuclease subunit homolog (S. cerevisiae)	52864	https://www.ncbi.nlm.nih.gov/gene/?term=52864
Rasl12	Rasl12	RAS-like, family 12	70784	https://www.ncbi.nlm.nih.gov/gene/?term=70784
Kmt5c	Kmt5c	lysine methyltransferase 5C	232811	https://www.ncbi.nlm.nih.gov/gene/?term=232811
Vegfa	Vegfa	vascular endothelial growth factor A	22339	https://www.ncbi.nlm.nih.gov/gene/?term=22339
Pcdha12	Pcdha12	protocadherin alpha 12	192164	https://www.ncbi.nlm.nih.gov/gene/?term=192164
Tll1	Tll1	tolloid-like	21892	https://www.ncbi.nlm.nih.gov/gene/?term=21892
Sestd1	Sestd1	SEC14 and spectrin domains 1	228071	https://www.ncbi.nlm.nih.gov/gene/?term=228071
Dgkd	Dgkd	diacylglycerol kinase, delta	227333	https://www.ncbi.nlm.nih.gov/gene/?term=227333
Timd2	Timd2	T cell immunoglobulin and mucin domain containing 2	171284	https://www.ncbi.nlm.nih.gov/gene/?term=171284
Col6a3	Col6a3	collagen, type VI, alpha 3	12835	https://www.ncbi.nlm.nih.gov/gene/?term=12835
Eomes	Eomes	eomesodermin	13813	https://www.ncbi.nlm.nih.gov/gene/?term=13813
Col4a3	Col4a3	collagen, type IV, alpha 3	12828	https://www.ncbi.nlm.nih.gov/gene/?term=12828
Foxj2	Foxj2	forkhead box J2	60611	https://www.ncbi.nlm.nih.gov/gene/?term=60611
Stmn2	Stmn2	stathmin-like 2	20257	https://www.ncbi.nlm.nih.gov/gene/?term=20257
Kif26a	Kif26a	kinesin family member 26A	668303	https://www.ncbi.nlm.nih.gov/gene/?term=668303
Igf1	Igf1	insulin-like growth factor 1	16000	https://www.ncbi.nlm.nih.gov/gene/?term=16000
Nup160	Nup160	nucleoporin 160	59015	https://www.ncbi.nlm.nih.gov/gene/?term=59015
Col1a1	Col1a1	collagen, type I, alpha 1	12842	https://www.ncbi.nlm.nih.gov/gene/?term=12842
Hormad1	Hormad1	HORMA domain containing 1	67981	https://www.ncbi.nlm.nih.gov/gene/?term=67981
Pcdha7	Pcdha7	protocadherin alpha 7	12939	https://www.ncbi.nlm.nih.gov/gene/?term=12939
Tet1	Tet1	tet methylcytosine dioxygenase 1	52463	https://www.ncbi.nlm.nih.gov/gene/?term=52463
Dio2	Dio2	deiodinase, iodothyronine, type II	13371	https://www.ncbi.nlm.nih.gov/gene/?term=13371
Rapgefl1	Rapgefl1	Rap guanine nucleotide exchange factor (GEF)-like 1	268480	https://www.ncbi.nlm.nih.gov/gene/?term=268480

Biology Open • Supplementary information

Frat2	Frat2	frequently rearranged in advanced T cell lymphomas 2	212398	https://www.ncbi.nlm.nih.gov/gene/?term=212398
Nasp	Nasp	nuclear autoantigenic sperm protein (histone-binding)	50927	https://www.ncbi.nlm.nih.gov/gene/?term=50927
Rnpepl1	Rnpepl1	arginyl aminopeptidase (aminopeptidase B)-like 1	108657	https://www.ncbi.nlm.nih.gov/gene/?term=108657
Kdm5b	Kdm5b	lysine (K)-specific demethylase 5B	75605	https://www.ncbi.nlm.nih.gov/gene/?term=75605
Kcna5	Kcna5	potassium voltage-gated channel, shaker-related subfamily, member 5	16493	https://www.ncbi.nlm.nih.gov/gene/?term=16493
Traf4	Traf4	TNF receptor associated factor 4	22032	https://www.ncbi.nlm.nih.gov/gene/?term=22032
Iffo1	Iffo1	intermediate filament family orphan 1	320678	https://www.ncbi.nlm.nih.gov/gene/?term=320678
Slc31a1	Slc31a1	solute carrier family 31, member 1	20529	https://www.ncbi.nlm.nih.gov/gene/?term=20529
Elf2	Elf2	E74-like factor 2	69257	https://www.ncbi.nlm.nih.gov/gene/?term=69257
Col19a1	Col19a1	collagen, type XIX, alpha 1	12823	https://www.ncbi.nlm.nih.gov/gene/?term=12823
Igsf9b	Igsf9b	immunoglobulin superfamily, member 9B	235086	https://www.ncbi.nlm.nih.gov/gene/?term=235086
Tfeb	Tfeb	transcription factor EB	21425	https://www.ncbi.nlm.nih.gov/gene/?term=21425
Rab30	Rab30	RAB30, member RAS oncogene family	75985	https://www.ncbi.nlm.nih.gov/gene/?term=75985
Tet2	Tet2	tet methylcytosine dioxygenase 2	214133	https://www.ncbi.nlm.nih.gov/gene/?term=214133
Col5a3	Col5a3	collagen, type V, alpha 3	53867	https://www.ncbi.nlm.nih.gov/gene/?term=53867
Gid8	Gid8	GID complex subunit 8	76425	https://www.ncbi.nlm.nih.gov/gene/?term=76425
Dnmt3b	Dnmt3b	DNA methyltransferase 3B	13436	https://www.ncbi.nlm.nih.gov/gene/?term=13436
Cldn1	Cldn1	claudin 1	12737	https://www.ncbi.nlm.nih.gov/gene/?term=12737
Tarbp1	Tarbp1	TAR RNA binding protein 1	212728	https://www.ncbi.nlm.nih.gov/gene/?term=212728
Rnf19a	Rnf19a	ring finger protein 19A	30945	https://www.ncbi.nlm.nih.gov/gene/?term=30945
Erlin2	Erlin2	ER lipid raft associated 2	244373	https://www.ncbi.nlm.nih.gov/gene/?term=244373
Narf	Narf	nuclear prelamin A recognition	67608	https://www.ncbi.nlm.nih.gov/gene/?term=67608

Biology Open • Supplementary information

		factor		
Eml4	Eml4	echinoderm microtubule associated protein like 4	78798	https://www.ncbi.nlm.nih.gov/gene/?term=78798
Adamts2	Adamts2	a disintegrin-like and metallopeptidase (reprolysin type) with thrombospondin type 1 motif, 2	216725	https://www.ncbi.nlm.nih.gov/gene/?term=216725
Dnmt3a	Dnmt3a	DNA methyltransferase 3A	13435	https://www.ncbi.nlm.nih.gov/gene/?term=13435
Zfp282	Zfp282	zinc finger protein 282	101095	https://www.ncbi.nlm.nih.gov/gene/?term=101095
Zdhhc21	Zdhhc21	zinc finger, DHHC domain containing 21	68268	https://www.ncbi.nlm.nih.gov/gene/?term=68268
Sh3pxd2a	Sh3pxd2a	SH3 and PX domains 2A	14218	https://www.ncbi.nlm.nih.gov/gene/?term=14218
Ireb2	Ireb2	iron responsive element binding protein 2	64602	https://www.ncbi.nlm.nih.gov/gene/?term=64602
Ttc9	Ttc9	tetratricopeptide repeat domain 9	69480	https://www.ncbi.nlm.nih.gov/gene/?term=69480
Pcdha10	Pcdha10	protocadherin alpha 10	12943	https://www.ncbi.nlm.nih.gov/gene/?term=12943
Pcdhac1	Pcdhac1	protocadherin alpha subfamily C, 1	353236	https://www.ncbi.nlm.nih.gov/gene/?term=353236
Col4a5	Col4a5	collagen, type IV, alpha 5	12830	https://www.ncbi.nlm.nih.gov/gene/?term=12830
Pxdn	Pxdn	peroxidasin	69675	https://www.ncbi.nlm.nih.gov/gene/?term=69675
Mycn	Mycn	v-myc avian myelocytomatosis viral related oncogene, neuroblastoma derived	18109	https://www.ncbi.nlm.nih.gov/gene/?term=18109
Eln	Eln	elastin	13717	https://www.ncbi.nlm.nih.gov/gene/?term=13717
Tpk1	Tpk1	thiamine pyrophosphokinase	29807	https://www.ncbi.nlm.nih.gov/gene/?term=29807
Slc25a3	Slc25a3	solute carrier family 25 (mitochondrial carrier, phosphate carrier), member 3	18674	https://www.ncbi.nlm.nih.gov/gene/?term=18674
Pcdha4	Pcdha4	protocadherin alpha 4	12936	https://www.ncbi.nlm.nih.gov/gene/?term=12936
Slc43a2	Slc43a2	solute carrier family 43, member 2	215113	https://www.ncbi.nlm.nih.gov/gene/?term=215113
Pcdha5	Pcdha5	protocadherin alpha 5	12941	https://www.ncbi.nlm.nih.gov/gene/?term=12941

Biology Open • Supplementary information

Brwd3	Brwd3	bromodomain and WD repeat domain containing 3	382236	https://www.ncbi.nlm.nih.gov/gene/?term=382236
Pcdha8	Pcdha8	protocadherin alpha 8	353235	https://www.ncbi.nlm.nih.gov/gene/?term=353235
Samd4	Samd4	sterile alpha motif domain containing 4	74480	https://www.ncbi.nlm.nih.gov/gene/?term=74480
Unc13b	Unc13b	unc-13 homolog B	22249	https://www.ncbi.nlm.nih.gov/gene/?term=22249
Nap1l3	Nap1l3	nucleosome assembly protein 1-like 3	54561	https://www.ncbi.nlm.nih.gov/gene/?term=54561
Serpinh1	Serpinh1	serine (or cysteine) peptidase inhibitor, clade H, member 1	12406	https://www.ncbi.nlm.nih.gov/gene/?term=12406
Tmem183a	Tmem183a	transmembrane protein 183A	57439	https://www.ncbi.nlm.nih.gov/gene/?term=57439
Adamts17	Adamts17	a disintegrin-like and metallopeptidase (reprolysin type) with thrombospondin type 1 motif, 17	233332	https://www.ncbi.nlm.nih.gov/gene/?term=233332
Map6	Map6	microtubule-associated protein 6	17760	https://www.ncbi.nlm.nih.gov/gene/?term=17760
Glis2	Glis2	GLIS family zinc finger 2	83396	https://www.ncbi.nlm.nih.gov/gene/?term=83396
Atp2b4	Atp2b4	ATPase, Ca++ transporting, plasma membrane 4	381290	https://www.ncbi.nlm.nih.gov/gene/?term=381290
Pmp22	Pmp22	peripheral myelin protein 22	18858	https://www.ncbi.nlm.nih.gov/gene/?term=18858
Chsy1	Chsy1	chondroitin sulfate synthase 1	269941	https://www.ncbi.nlm.nih.gov/gene/?term=269941
Cd276	Cd276	CD276 antigen	102657	https://www.ncbi.nlm.nih.gov/gene/?term=102657
Col22a1	Col22a1	collagen, type XXII, alpha 1	69700	https://www.ncbi.nlm.nih.gov/gene/?term=69700
Stard8	Stard8	START domain containing 8	236920	https://www.ncbi.nlm.nih.gov/gene/?term=236920
Tet3	Tet3	tet methylcytosine dioxygenase 3	194388	https://www.ncbi.nlm.nih.gov/gene/?term=194388
Pcdha6	Pcdha6	protocadherin alpha 6	12937	https://www.ncbi.nlm.nih.gov/gene/?term=12937
Zfp704	Zfp704	zinc finger protein 704	170753	https://www.ncbi.nlm.nih.gov/gene/?term=170753
Tcf4	Tcf4	transcription factor 4	21413	https://www.ncbi.nlm.nih.gov/gene/?term=21413
Col5a1	Col5a1	collagen, type V, alpha 1	12831	https://www.ncbi.nlm.nih.gov/gene/?term=12831

Biology Open • Supplementary information

Col7a1	Col7a1	collagen, type VII, alpha 1	12836	https://www.ncbi.nlm.nih.gov/gene/?term=12836
Pgap2	Pgap2	post-GPI attachment to proteins 2	233575	https://www.ncbi.nlm.nih.gov/gene/?term=233575
Ddx3x	Ddx3x	DEAD/H (Asp-Glu-Ala-Asp/His) box polypeptide 3, X-linked	13205	https://www.ncbi.nlm.nih.gov/gene/?term=13205
Col15a1	Col15a1	collagen, type XV, alpha 1	12819	https://www.ncbi.nlm.nih.gov/gene/?term=12819
Pcdha3	Pcdha3	protocadherin alpha 3	192163	https://www.ncbi.nlm.nih.gov/gene/?term=192163
Mapkbp1	Mapkbp1	mitogen-activated protein kinase binding protein 1	26390	https://www.ncbi.nlm.nih.gov/gene/?term=26390
Fer	Fer	fer (fms/fps related) protein kinase	14158	https://www.ncbi.nlm.nih.gov/gene/?term=14158
Mcl1	Mcl1	myeloid cell leukemia sequence 1	17210	https://www.ncbi.nlm.nih.gov/gene/?term=17210
Adamts9	Adamts9	a disintegrin-like and metallopeptidase (reprolysin type) with thrombospondin type 1 motif, 9	101401	https://www.ncbi.nlm.nih.gov/gene/?term=101401
Ifi30	Ifi30	interferon gamma inducible protein 30	65972	https://www.ncbi.nlm.nih.gov/gene/?term=65972
Adamts18	Adamts18	a disintegrin-like and metallopeptidase (reprolysin type) with thrombospondin type 1 motif, 18	208936	https://www.ncbi.nlm.nih.gov/gene/?term=208936
Nav1	Nav1	neuron navigator 1	215690	https://www.ncbi.nlm.nih.gov/gene/?term=215690
Pcdha2	Pcdha2	protocadherin alpha 2	353234	https://www.ncbi.nlm.nih.gov/gene/?term=353234
Ppic	Ppic	peptidylprolyl isomerase C	19038	https://www.ncbi.nlm.nih.gov/gene/?term=19038
Lif	Lif	leukemia inhibitory factor	16878	https://www.ncbi.nlm.nih.gov/gene/?term=16878
Amer1	Amer1	APC membrane recruitment 1	72345	https://www.ncbi.nlm.nih.gov/gene/?term=72345
Pcdhac2	Pcdhac2	protocadherin alpha subfamily C, 2	353237	https://www.ncbi.nlm.nih.gov/gene/?term=353237
Ankrd13b	Ankrd13b	ankyrin repeat domain 13b	268445	https://www.ncbi.nlm.nih.gov/gene/?term=268445
Col5a2	Col5a2	collagen, type V, alpha 2	12832	https://www.ncbi.nlm.nih.gov/gene/?term=12832
Taf5	Taf5	TATA-box binding protein associated factor 5	226182	https://www.ncbi.nlm.nih.gov/gene/?term=226182

Biology Open • Supplementary information

Dusp2	Dusp2	dual specificity phosphatase 2	13537	https://www.ncbi.nlm.nih.gov/gene/?term=13537
Eva1b	Eva1b	eva-1 homolog B (C. elegans)	230752	https://www.ncbi.nlm.nih.gov/gene/?term=230752
Mark3	Mark3	MAP/microtubule affinity regulating kinase 3	17169	https://www.ncbi.nlm.nih.gov/gene/?term=17169
Pcdha9	Pcdha9	protocadherin alpha 9	192161	https://www.ncbi.nlm.nih.gov/gene/?term=192161
Dpysl5	Dpysl5	dihydropyrimidinase-like 5	65254	https://www.ncbi.nlm.nih.gov/gene/?term=65254
Camk4	Camk4	calcium/calmodulin-dependent protein kinase IV	12326	https://www.ncbi.nlm.nih.gov/gene/?term=12326
Col2a1	Col2a1	collagen, type II, alpha 1	12824	https://www.ncbi.nlm.nih.gov/gene/?term=12824
Il1rap	Il1rap	interleukin 1 receptor accessory protein	16180	https://www.ncbi.nlm.nih.gov/gene/?term=16180
Lysmd1	Lysmd1	LysM, putative peptidoglycan-binding, domain containing 1	217779	https://www.ncbi.nlm.nih.gov/gene/?term=217779
Zfp346	Zfp346	zinc finger protein 346	26919	https://www.ncbi.nlm.nih.gov/gene/?term=26919
Dlg2	Dlg2	discs large MAGUK scaffold protein 2	23859	https://www.ncbi.nlm.nih.gov/gene/?term=23859
Fam167a	Fam167a	family with sequence similarity 167, member A	219148	https://www.ncbi.nlm.nih.gov/gene/?term=219148
Ppm1d	Ppm1d	protein phosphatase 1D magnesium-dependent, delta isoform	53892	https://www.ncbi.nlm.nih.gov/gene/?term=53892
N4bp2l1	N4bp2l1	NEDD4 binding protein 2-like 1	100637	https://www.ncbi.nlm.nih.gov/gene/?term=100637
Col4a6	Col4a6	collagen, type IV, alpha 6	94216	https://www.ncbi.nlm.nih.gov/gene/?term=94216
Col9a1	Col9a1	collagen, type IX, alpha 1	12839	https://www.ncbi.nlm.nih.gov/gene/?term=12839
Fem1b	Fem1b	feminization 1 homolog b (C. elegans)	14155	https://www.ncbi.nlm.nih.gov/gene/?term=14155
Smtnl2	Smtnl2	smoothelin-like 2	276829	https://www.ncbi.nlm.nih.gov/gene/?term=276829
Hdac4	Hdac4	histone deacetylase 4	208727	https://www.ncbi.nlm.nih.gov/gene/?term=208727
Atp1b4	Atp1b4	ATPase, (Na+)/K+ transporting, beta 4 polypeptide	67821	https://www.ncbi.nlm.nih.gov/gene/?term=67821
Zmiz1	Zmiz1	zinc finger, MIZ-type containing 1	328365	https://www.ncbi.nlm.nih.gov/gene/?term=328365

Biology Open • Supplementary information

Pcdha1	Pcdha1	protocadherin alpha 1	116731	https://www.ncbi.nlm.nih.gov/gene/?term=116731
D630045J12Rik	D630045J12Rik	RIKEN cDNA D630045J12 gene	330286	https://www.ncbi.nlm.nih.gov/gene/?term=330286
Proser1	Proser1	proline and serine rich 1	212127	https://www.ncbi.nlm.nih.gov/gene/?term=212127
Carmil1	Carmil1	capping protein regulator and myosin 1 linker 1	68732	https://www.ncbi.nlm.nih.gov/gene/?term=68732
Trim63	Trim63	tripartite motif-containing 63	433766	https://www.ncbi.nlm.nih.gov/gene/?term=433766
Rest	Rest	RE1-silencing transcription factor	19712	https://www.ncbi.nlm.nih.gov/gene/?term=19712
Bmf	Bmf	BCL2 modifying factor	171543	https://www.ncbi.nlm.nih.gov/gene/?term=171543
Col11a1	Col11a1	collagen, type XI, alpha 1	12814	https://www.ncbi.nlm.nih.gov/gene/?term=12814
Morf4l2	Morf4l2	mortality factor 4 like 2	56397	https://www.ncbi.nlm.nih.gov/gene/?term=56397
C1qtnf6	C1qtnf6	C1q and tumor necrosis factor related protein 6	72709	https://www.ncbi.nlm.nih.gov/gene/?term=72709
Adam19	Adam19	a disintegrin and metallopeptidase domain 19 (meltrin beta)	11492	https://www.ncbi.nlm.nih.gov/gene/?term=11492
Pan2	Pan2	PAN2 poly(A) specific ribonuclease subunit	103135	https://www.ncbi.nlm.nih.gov/gene/?term=103135
Cdk6	Cdk6	cyclin-dependent kinase 6	12571	https://www.ncbi.nlm.nih.gov/gene/?term=12571
Crispld1	Crispld1	cysteine-rich secretory protein LCCL domain containing 1	83691	https://www.ncbi.nlm.nih.gov/gene/?term=83691
Pten	Pten	phosphatase and tensin homolog	19211	https://www.ncbi.nlm.nih.gov/gene/?term=19211
Hbp1	Hbp1	high mobility group box transcription factor 1	73389	https://www.ncbi.nlm.nih.gov/gene/?term=73389
Fstl1	Fstl1	follistatin-like 1	14314	https://www.ncbi.nlm.nih.gov/gene/?term=14314
Pcdha11	Pcdha11	protocadherin alpha 11	12942	https://www.ncbi.nlm.nih.gov/gene/?term=12942
Zbtb10	Zbtb10	zinc finger and BTB domain containing 10	229055	https://www.ncbi.nlm.nih.gov/gene/?term=229055
Smurf2	Smurf2	SMAD specific E3 ubiquitin protein ligase 2	66313	https://www.ncbi.nlm.nih.gov/gene/?term=66313
Sparc	Sparc	secreted acidic cysteine rich	20692	https://www.ncbi.nlm.nih.gov/gene/?term=20692

Biology Open • Supplementary information

		glycoprotein		
Purg	Purg	purine-rich element binding protein G	75029	https://www.ncbi.nlm.nih.gov/gene/?term=75029
Nsd1	Nsd1	nuclear receptor-binding SET-domain protein 1	18193	https://www.ncbi.nlm.nih.gov/gene/?term=18193
Spc25	Spc25	SPC25, NDC80 kinetochore complex component, homolog (S. cerevisiae)	66442	https://www.ncbi.nlm.nih.gov/gene/?term=66442
Rgs9	Rgs9	regulator of G-protein signaling 9	19739	https://www.ncbi.nlm.nih.gov/gene/?term=19739
Trex2	Trex2	three prime repair exonuclease 2	24102	https://www.ncbi.nlm.nih.gov/gene/?term=24102
Cpsf6	Cpsf6	cleavage and polyadenylation specific factor 6	432508	https://www.ncbi.nlm.nih.gov/gene/?term=432508
Srsf1	Srsf1	serine/arginine-rich splicing factor 1	110809	https://www.ncbi.nlm.nih.gov/gene/?term=110809
Saa3	Saa3	serum amyloid A 3	20210	https://www.ncbi.nlm.nih.gov/gene/?term=20210
Mogs	Mogs	mannosyl-oligosaccharide glucosidase	57377	https://www.ncbi.nlm.nih.gov/gene/?term=57377
Aplp1	Aplp1	amyloid beta (A4) precursor-like protein 1	11803	https://www.ncbi.nlm.nih.gov/gene/?term=11803
Celsr2	Celsr2	cadherin, EGF LAG seven-pass G-type receptor 2	53883	https://www.ncbi.nlm.nih.gov/gene/?term=53883
Hspa9	Hspa9	heat shock protein 9	15526	https://www.ncbi.nlm.nih.gov/gene/?term=15526
Fut1	Fut1	fucosyltransferase 1	14343	https://www.ncbi.nlm.nih.gov/gene/?term=14343
Ets1	Ets1	E26 avian leukemia oncogene 1, 5' domain	23871	https://www.ncbi.nlm.nih.gov/gene/?term=23871
Spag11b	Spag11b	sperm associated antigen 11B	546038	https://www.ncbi.nlm.nih.gov/gene/?term=546038
Nf2	Nf2	neurofibromin 2	18016	https://www.ncbi.nlm.nih.gov/gene/?term=18016
Zbtb18	Zbtb18	zinc finger and BTB domain containing 18	30928	https://www.ncbi.nlm.nih.gov/gene/?term=30928
Actr2	Actr2	ARP2 actin-related protein 2	66713	https://www.ncbi.nlm.nih.gov/gene/?term=66713
Dusp16	Dusp16	dual specificity phosphatase 16	70686	https://www.ncbi.nlm.nih.gov/gene/?term=70686

Biology Open • Supplementary information

B4galt7	B4galt7	xylosylprotein beta1,4-galactosyltransferase, polypeptide 7 (galactosyltransferase I)	218271	https://www.ncbi.nlm.nih.gov/gene/?term=218271
Pcmtd2	Pcmtd2	protein-L-isoaspartate (D-aspartate) O-methyltransferase domain containing 2	245867	https://www.ncbi.nlm.nih.gov/gene/?term=245867
1110038F14Rik	1110038F14Rik	RIKEN cDNA 1110038F14 gene	117171	https://www.ncbi.nlm.nih.gov/gene/?term=117171
Prrc2c	Prrc2c	proline-rich coiled-coil 2C	226562	https://www.ncbi.nlm.nih.gov/gene/?term=226562
Herpud2	Herpud2	HERPUD family member 2	80517	https://www.ncbi.nlm.nih.gov/gene/?term=80517
Tdrd3	Tdrd3	tudor domain containing 3	219249	https://www.ncbi.nlm.nih.gov/gene/?term=219249
Crybg1	Crybg1	crystallin beta-gamma domain containing 1	11630	https://www.ncbi.nlm.nih.gov/gene/?term=11630
Zfp984	Zfp984	zinc finger protein 984	100041677	https://www.ncbi.nlm.nih.gov/gene/?term=100041
Armcx1	Armcx1	armadillo repeat containing, X-linked 1	78248	https://www.ncbi.nlm.nih.gov/gene/?term=78248
Fgf18	Fgf18	fibroblast growth factor 18	14172	https://www.ncbi.nlm.nih.gov/gene/?term=14172
Gramd3	Gramd3	GRAM domain containing 3	107022	https://www.ncbi.nlm.nih.gov/gene/?term=107022
Spry1	Spry1	sprouty RTK signaling antagonist 1	24063	https://www.ncbi.nlm.nih.gov/gene/?term=24063
Tipin	Tipin	timeless interacting protein	66131	https://www.ncbi.nlm.nih.gov/gene/?term=66131
Ccl20	Ccl20	chemokine (C-C motif) ligand 20	20297	https://www.ncbi.nlm.nih.gov/gene/?term=20297
Ifrd1	Ifrd1	interferon-related developmental regulator 1	15982	https://www.ncbi.nlm.nih.gov/gene/?term=15982
Nkiras1	Nkiras1	NFKB inhibitor interacting Ras-like protein 1	69721	https://www.ncbi.nlm.nih.gov/gene/?term=69721
Tgfbi	Tgfbi	transforming growth factor, beta induced	21810	https://www.ncbi.nlm.nih.gov/gene/?term=21810
Ski	Ski	ski sarcoma viral oncogene homolog (avian)	20481	https://www.ncbi.nlm.nih.gov/gene/?term=20481
Ankrd49	Ankrd49	ankyrin repeat domain 49	56503	https://www.ncbi.nlm.nih.gov/gene/?term=56503

Caskin1	Caskin1	CASK interacting protein 1	268932	https://www.ncbi.nlm.nih.gov/gene/?term=268932
Adgrg2	Adgrg2	adhesion G protein-coupled receptor G2	237175	https://www.ncbi.nlm.nih.gov/gene/?term=237175
Il21	Il21	interleukin 21	60505	https://www.ncbi.nlm.nih.gov/gene/?term=60505
Zfp367	Zfp367	zinc finger protein 367	238673	https://www.ncbi.nlm.nih.gov/gene/?term=238673
Scml2	Scml2	Scm polycomb group protein like 2	107815	https://www.ncbi.nlm.nih.gov/gene/?term=107815
Ntf3	Ntf3	neurotrophin 3	18205	https://www.ncbi.nlm.nih.gov/gene/?term=18205
Pnpt1	Pnpt1	polyribonucleotide nucleotidyltransferase 1	71701	https://www.ncbi.nlm.nih.gov/gene/?term=71701
Ccl1	Ccl1	chemokine (C-C motif) ligand 1	20290	https://www.ncbi.nlm.nih.gov/gene/?term=20290
Spink14	Spink14	serine peptidase inhibitor, Kazal type 14	433178	https://www.ncbi.nlm.nih.gov/gene/?term=433178
Bcl11b	Bcl11b	B cell leukemia/lymphoma 11B	58208	https://www.ncbi.nlm.nih.gov/gene/?term=58208
Tmem236	Tmem236	transmembrane protein 236	625286	https://www.ncbi.nlm.nih.gov/gene/?term=625286
Tent5a	Tent5a	terminal nucleotidyltransferase 5A	212943	https://www.ncbi.nlm.nih.gov/gene/?term=212943
Bcl7a	Bcl7a	B cell CLL/lymphoma 7A	77045	https://www.ncbi.nlm.nih.gov/gene/?term=77045
Tsga10	Tsga10	testis specific 10	211484	https://www.ncbi.nlm.nih.gov/gene/?term=211484
Dusp8	Dusp8	dual specificity phosphatase 8	18218	https://www.ncbi.nlm.nih.gov/gene/?term=18218
Armcx5	Armcx5	armadillo repeat containing, X-linked 5	494468	https://www.ncbi.nlm.nih.gov/gene/?term=494468
Nectin3	Nectin3	nectin cell adhesion molecule 3	58998	https://www.ncbi.nlm.nih.gov/gene/?term=58998
Fa2h	Fa2h	fatty acid 2-hydroxylase	338521	https://www.ncbi.nlm.nih.gov/gene/?term=338521
Pan3	Pan3	PAN3 poly(A) specific ribonuclease subunit	72587	https://www.ncbi.nlm.nih.gov/gene/?term=72587
Peli1	Peli1	pellino 1	67245	https://www.ncbi.nlm.nih.gov/gene/?term=67245
Pbrm1	Pbrm1	polybromo 1	66923	https://www.ncbi.nlm.nih.gov/gene/?term=66923
Dmrtc2	Dmrtc2	doublesex and mab-3 related transcription factor like family C2	71241	https://www.ncbi.nlm.nih.gov/gene/?term=71241
Armcx3	Armcx3	armadillo repeat containing, X-linked	71703	https://www.ncbi.nlm.nih.gov/gene/?term=71703

Biology Open • Supplementary information

		3		
Ptbp3	Ptbp3	polypyrimidine tract binding protein 3	230257	https://www.ncbi.nlm.nih.gov/gene/?term=230257
Grik2	Grik2	glutamate receptor, ionotropic, kainate 2 (beta 2)	14806	https://www.ncbi.nlm.nih.gov/gene/?term=14806
Nek7	Nek7	NIMA (never in mitosis gene a)-related expressed kinase 7	59125	https://www.ncbi.nlm.nih.gov/gene/?term=59125
Cdc40	Cdc40	cell division cycle 40	71713	https://www.ncbi.nlm.nih.gov/gene/?term=71713
Gigyf1	Gigyf1	GRB10 interacting GYF protein 1	57330	https://www.ncbi.nlm.nih.gov/gene/?term=57330
Slc4a10	Slc4a10	solute carrier family 4, sodium bicarbonate cotransporter-like, member 10	94229	https://www.ncbi.nlm.nih.gov/gene/?term=94229
Pi4k2b	Pi4k2b	phosphatidylinositol 4-kinase type 2 beta	67073	https://www.ncbi.nlm.nih.gov/gene/?term=67073
Zfp97	Zfp97	zinc finger protein 97	22759	https://www.ncbi.nlm.nih.gov/gene/?term=22759
Tnfaip1	Tnfaip1	tumor necrosis factor, alpha-induced protein 1 (endothelial)	21927	https://www.ncbi.nlm.nih.gov/gene/?term=21927
Spire1	Spire1	spire type actin nucleation factor 1	68166	https://www.ncbi.nlm.nih.gov/gene/?term=68166
Cdyl	Cdyl	chromodomain protein, Y chromosome-like	12593	https://www.ncbi.nlm.nih.gov/gene/?term=12593
Carf	Carf	calcium response factor	241066	https://www.ncbi.nlm.nih.gov/gene/?term=241066
Sfmbt1	Sfmbt1	Scm-like with four mbt domains 1	54650	https://www.ncbi.nlm.nih.gov/gene/?term=54650
Rbm46	Rbm46	RNA binding motif protein 46	633285	https://www.ncbi.nlm.nih.gov/gene/?term=633285
Ddx55	Ddx55	DEAD (Asp-Glu-Ala-Asp) box polypeptide 55	67848	https://www.ncbi.nlm.nih.gov/gene/?term=67848
Gse1	Gse1	genetic suppressor element 1, coiled-coil protein	382034	https://www.ncbi.nlm.nih.gov/gene/?term=382034
Gpd1l	Gpd1l	glycerol-3-phosphate dehydrogenase 1-like	333433	https://www.ncbi.nlm.nih.gov/gene/?term=333433

Biology Open • Supplementary information

Ercc5	Ercc5	excision repair cross-complementing rodent repair deficiency, complementation group 5	22592	https://www.ncbi.nlm.nih.gov/gene/?term=22592
Usp33	Usp33	ubiquitin specific peptidase 33	170822	https://www.ncbi.nlm.nih.gov/gene/?term=170822
Gm14322	Gm14322	predicted gene 14322	626802	https://www.ncbi.nlm.nih.gov/gene/?term=626802
Nfat5	Nfat5	nuclear factor of activated T cells 5	54446	https://www.ncbi.nlm.nih.gov/gene/?term=54446
Ap1g1	Ap1g1	adaptor protein complex AP-1, gamma 1 subunit	11765	https://www.ncbi.nlm.nih.gov/gene/?term=11765
Zfp120	Zfp120	zinc finger protein 120	104348	https://www.ncbi.nlm.nih.gov/gene/?term=104348
Trim2	Trim2	tripartite motif-containing 2	80890	https://www.ncbi.nlm.nih.gov/gene/?term=80890
Mtx3	Mtx3	metaxin 3	382793	https://www.ncbi.nlm.nih.gov/gene/?term=382793
Wdr82	Wdr82	WD repeat domain containing 82	77305	https://www.ncbi.nlm.nih.gov/gene/?term=77305
Kcnq5	Kcnq5	potassium voltage-gated channel, subfamily Q, member 5	226922	https://www.ncbi.nlm.nih.gov/gene/?term=226922
Ccdc122	Ccdc122	coiled-coil domain containing 122	108811	https://www.ncbi.nlm.nih.gov/gene/?term=108811
Anapc16	Anapc16	anaphase promoting complex subunit 16	52717	https://www.ncbi.nlm.nih.gov/gene/?term=52717
Bend3	Bend3	BEN domain containing 3	331623	https://www.ncbi.nlm.nih.gov/gene/?term=331623
Brd1	Brd1	bromodomain containing 1	223770	https://www.ncbi.nlm.nih.gov/gene/?term=223770
Adam11	Adam11	a disintegrin and metallopeptidase domain 11	11488	https://www.ncbi.nlm.nih.gov/gene/?term=11488
Lmo1	Lmo1	LIM domain only 1	109594	https://www.ncbi.nlm.nih.gov/gene/?term=109594
Zfp976	Zfp976	zinc finger protein 976	208111	https://www.ncbi.nlm.nih.gov/gene/?term=208111
Man2a1	Man2a1	mannosidase 2, alpha 1	17158	https://www.ncbi.nlm.nih.gov/gene/?term=17158
Tada2b	Tada2b	transcriptional adaptor 2B	231151	https://www.ncbi.nlm.nih.gov/gene/?term=231151
Rorb	Rorb	RAR-related orphan receptor beta	225998	https://www.ncbi.nlm.nih.gov/gene/?term=225998
Acvr2b	Acvr2b	activin receptor IIB	11481	https://www.ncbi.nlm.nih.gov/gene/?term=11481
Patl1	Patl1	protein associated with topoisomerase II homolog 1 (yeast)	225929	https://www.ncbi.nlm.nih.gov/gene/?term=225929

Biology Open • Supplementary information

Txndc12	Txndc12	thioredoxin domain containing 12 (endoplasmic reticulum)	66073	https://www.ncbi.nlm.nih.gov/gene/?term=66073
Togaram1	Togaram1	TOG array regulator of axonemal microtubules 1	328108	https://www.ncbi.nlm.nih.gov/gene/?term=328108
Palb2	Palb2	partner and localizer of BRCA2	233826	https://www.ncbi.nlm.nih.gov/gene/?term=233826
Rnf34	Rnf34	ring finger protein 34	80751	https://www.ncbi.nlm.nih.gov/gene/?term=80751
6720489N17Rik	6720489N17Rik	RIKEN cDNA 6720489N17 gene	211378	https://www.ncbi.nlm.nih.gov/gene/?term=211378
Ubp1	Ubp1	upstream binding protein 1	22221	https://www.ncbi.nlm.nih.gov/gene/?term=22221
Gfpt1	Gfpt1	glutamine fructose-6-phosphate transaminase 1	14583	https://www.ncbi.nlm.nih.gov/gene/?term=14583
Lox	Lox	lysyl oxidase	16948	https://www.ncbi.nlm.nih.gov/gene/?term=16948
Tmem94	Tmem94	transmembrane protein 94	71947	https://www.ncbi.nlm.nih.gov/gene/?term=71947
Slc25a37	Slc25a37	solute carrier family 25, member 37	67712	https://www.ncbi.nlm.nih.gov/gene/?term=67712
Zdhhc7	Zdhhc7	zinc finger, DHHC domain containing 7	102193	https://www.ncbi.nlm.nih.gov/gene/?term=102193
Clip1	Clip1	CAP-GLY domain containing linker protein 1	56430	https://www.ncbi.nlm.nih.gov/gene/?term=56430
Gabra1	Gabra1	gamma-aminobutyric acid (GABA) A receptor, subunit alpha 1	14394	https://www.ncbi.nlm.nih.gov/gene/?term=14394
Sema4g	Sema4g	sema domain, immunoglobulin domain (Ig), transmembrane domain (TM) and short cytoplasmic domain, (semaphorin) 4G	26456	https://www.ncbi.nlm.nih.gov/gene/?term=26456
Gm6712	Gm6712	predicted gene 6712	100862203	https://www.ncbi.nlm.nih.gov/gene/?term=100862
Klhl29	Klhl29	kelch-like 29	208439	https://www.ncbi.nlm.nih.gov/gene/?term=208439
Rassf1	Rassf1	Ras association (RalGDS/AF-6) domain family member 1	56289	https://www.ncbi.nlm.nih.gov/gene/?term=56289
Mb21d2	Mb21d2	Mab-21 domain containing 2	239796	https://www.ncbi.nlm.nih.gov/gene/?term=239796

Biology Open • Supplementary information

Acsl4	Acsl4	acyl-CoA synthetase long-chain family member 4	50790	https://www.ncbi.nlm.nih.gov/gene/?term=50790
Gm6710	Gm6710	predicted gene 6710	626832	https://www.ncbi.nlm.nih.gov/gene/?term=626832
Rps6ka3	Rps6ka3	ribosomal protein S6 kinase polypeptide 3	110651	https://www.ncbi.nlm.nih.gov/gene/?term=110651
Ss18l1	Ss18l1	SS18, nBAF chromatin remodeling complex subunit like 1	269397	https://www.ncbi.nlm.nih.gov/gene/?term=269397
Zfp280d	Zfp280d	zinc finger protein 280D	235469	https://www.ncbi.nlm.nih.gov/gene/?term=235469
Fam122b	Fam122b	family with sequence similarity 122, member B	78755	https://www.ncbi.nlm.nih.gov/gene/?term=78755
Heca	Heca	hdc homolog, cell cycle regulator	380629	https://www.ncbi.nlm.nih.gov/gene/?term=380629
2210418O10Rik	2210418O10Rik	RIKEN cDNA 2210418O10 gene	100504263	https://www.ncbi.nlm.nih.gov/gene/?term=100504
Ccp110	Ccp110	centriolar coiled coil protein 110	101565	https://www.ncbi.nlm.nih.gov/gene/?term=101565
Zfp619	Zfp619	zinc finger protein 619	70227	https://www.ncbi.nlm.nih.gov/gene/?term=70227
Zfp951	Zfp951	zinc finger protein 951	626391	https://www.ncbi.nlm.nih.gov/gene/?term=626391
Klhl5	Klhl5	kelch-like 5	71778	https://www.ncbi.nlm.nih.gov/gene/?term=71778
Zic3	Zic3	zinc finger protein of the cerebellum 3	22773	https://www.ncbi.nlm.nih.gov/gene/?term=22773
Clec10a	Clec10a	C-type lectin domain family 10, member A	17312	https://www.ncbi.nlm.nih.gov/gene/?term=17312
Cyp2c39	Cyp2c39	cytochrome P450, family 2, subfamily c, polypeptide 39	13098	https://www.ncbi.nlm.nih.gov/gene/?term=13098
Entpd6	Entpd6	ectonucleoside triphosphate diphosphohydrolase 6	12497	https://www.ncbi.nlm.nih.gov/gene/?term=12497
Sec24c	Sec24c	Sec24 related gene family, member C (S. cerevisiae)	218811	https://www.ncbi.nlm.nih.gov/gene/?term=218811
Ppip5k2	Ppip5k2	diphosphoinositol pentakisphosphate kinase 2	227399	https://www.ncbi.nlm.nih.gov/gene/?term=227399
Gpr22	Gpr22	G protein-coupled receptor 22	73010	https://www.ncbi.nlm.nih.gov/gene/?term=73010

Gata6	Gata6	GATA binding protein 6	14465	https://www.ncbi.nlm.nih.gov/gene/?term=14465
Syne1	Syne1	spectrin repeat containing, nuclear envelope 1	64009	https://www.ncbi.nlm.nih.gov/gene/?term=64009
Zfp973	Zfp973	zinc finger protein 973	545490	https://www.ncbi.nlm.nih.gov/gene/?term=545490
Cdon	Cdon	cell adhesion molecule-related/down-regulated by oncogenes	57810	https://www.ncbi.nlm.nih.gov/gene/?term=57810
P2ry10	P2ry10	purinergic receptor P2Y, G-protein coupled 10	78826	https://www.ncbi.nlm.nih.gov/gene/?term=78826
Specc1l	Specc1l	sperm antigen with calponin homology and coiled-coil domains 1-like	74392	https://www.ncbi.nlm.nih.gov/gene/?term=74392
Adcy9	Adcy9	adenylate cyclase 9	11515	https://www.ncbi.nlm.nih.gov/gene/?term=11515
Spry4	Spry4	sprouty RTK signaling antagonist 4	24066	https://www.ncbi.nlm.nih.gov/gene/?term=24066
Fam3c	Fam3c	family with sequence similarity 3, member C	27999	https://www.ncbi.nlm.nih.gov/gene/?term=27999
Zfp780b	Zfp780b	zinc finger protein 780B	338354	https://www.ncbi.nlm.nih.gov/gene/?term=338354
Nr1d2	Nr1d2	nuclear receptor subfamily 1, group D, member 2	353187	https://www.ncbi.nlm.nih.gov/gene/?term=353187
Zfp317	Zfp317	zinc finger protein 317	244713	https://www.ncbi.nlm.nih.gov/gene/?term=244713
Mpp5	Mpp5	membrane protein, palmitoylated 5 (MAGUK p55 subfamily member 5)	56217	https://www.ncbi.nlm.nih.gov/gene/?term=56217
Il1a	Il1a	interleukin 1 alpha	16175	https://www.ncbi.nlm.nih.gov/gene/?term=16175
Gls	Gls	glutaminase	14660	https://www.ncbi.nlm.nih.gov/gene/?term=14660
Hoxa11	Hoxa11	homeobox A11	15396	https://www.ncbi.nlm.nih.gov/gene/?term=15396
Klf15	Klf15	Kruppel-like factor 15	66277	https://www.ncbi.nlm.nih.gov/gene/?term=66277
Zfp14	Zfp14	zinc finger protein 14	243906	https://www.ncbi.nlm.nih.gov/gene/?term=243906
Tspan13	Tspan13	tetraspanin 13	66109	https://www.ncbi.nlm.nih.gov/gene/?term=66109
Ssx2ip	Ssx2ip	synovial sarcoma, X 2 interacting	99167	https://www.ncbi.nlm.nih.gov/gene/?term=99167

Biology Open • Supplementary information

		protein		
Zfp781	Zfp781	zinc finger protein 781	331188	https://www.ncbi.nlm.nih.gov/gene/?term=331188
Cpne2	Cpne2	copine II	234577	https://www.ncbi.nlm.nih.gov/gene/?term=234577
Ubl3	Ubl3	ubiquitin-like 3	24109	https://www.ncbi.nlm.nih.gov/gene/?term=24109
Rbbp7	Rbbp7	retinoblastoma binding protein 7, chromatin remodeling factor	245688	https://www.ncbi.nlm.nih.gov/gene/?term=245688
Pou2f1	Pou2f1	POU domain, class 2, transcription factor 1	18986	https://www.ncbi.nlm.nih.gov/gene/?term=18986
Dclk1	Dclk1	doublecortin-like kinase 1	13175	https://www.ncbi.nlm.nih.gov/gene/?term=13175
Tmem165	Tmem165	transmembrane protein 165	21982	https://www.ncbi.nlm.nih.gov/gene/?term=21982
Baz2b	Baz2b	bromodomain adjacent to zinc finger domain, 2B	407823	https://www.ncbi.nlm.nih.gov/gene/?term=407823
Lrba	Lrba	LPS-responsive beige-like anchor	80877	https://www.ncbi.nlm.nih.gov/gene/?term=80877
Zic2	Zic2	zinc finger protein of the cerebellum 2	22772	https://www.ncbi.nlm.nih.gov/gene/?term=22772
Tbc1d4	Tbc1d4	TBC1 domain family, member 4	210789	https://www.ncbi.nlm.nih.gov/gene/?term=210789
Mapk1	Mapk1	mitogen-activated protein kinase 1	26413	https://www.ncbi.nlm.nih.gov/gene/?term=26413
Slc25a36	Slc25a36	solute carrier family 25, member 36	192287	https://www.ncbi.nlm.nih.gov/gene/?term=192287
Zfp970	Zfp970	zinc finger protein 970	628308	https://www.ncbi.nlm.nih.gov/gene/?term=628308
Prox1	Prox1	prospero homeobox 1	19130	https://www.ncbi.nlm.nih.gov/gene/?term=19130
Zfp966	Zfp966	zinc finger protein 966	667962	https://www.ncbi.nlm.nih.gov/gene/?term=667962
Ttc39b	Ttc39b	tetratricopeptide repeat domain 39B	69863	https://www.ncbi.nlm.nih.gov/gene/?term=69863
B4galt1	B4galt1	UDP-Gal:betaGlcNAc beta 1,4-galactosyltransferase, polypeptide 1	14595	https://www.ncbi.nlm.nih.gov/gene/?term=14595
Zfp936	Zfp936	zinc finger protein 936	668620	https://www.ncbi.nlm.nih.gov/gene/?term=668620
Ap1s3	Ap1s3	adaptor-related protein complex AP-1, sigma 3	252903	https://www.ncbi.nlm.nih.gov/gene/?term=252903
Lhx9	Lhx9	LIM homeobox protein 9	16876	https://www.ncbi.nlm.nih.gov/gene/?term=16876
Zfp960	Zfp960	zinc finger protein 960	449000	https://www.ncbi.nlm.nih.gov/gene/?term=449000

Biology Open • Supplementary information

Dmxl2	Dmxl2	Dmx-like 2	235380	https://www.ncbi.nlm.nih.gov/gene/?term=235380
Osbpl3	Osbpl3	oxysterol binding protein-like 3	71720	https://www.ncbi.nlm.nih.gov/gene/?term=71720
Zfp971	Zfp971	zinc finger protein 971	626848	https://www.ncbi.nlm.nih.gov/gene/?term=626848
Ythdc2	Ythdc2	YTH domain containing 2	240255	https://www.ncbi.nlm.nih.gov/gene/?term=240255
Sim1	Sim1	single-minded family bHLH transcription factor 1	20464	https://www.ncbi.nlm.nih.gov/gene/?term=20464
Carm1	Carm1	coactivator-associated arginine methyltransferase 1	59035	https://www.ncbi.nlm.nih.gov/gene/?term=59035
Abtb2	Abtb2	ankyrin repeat and BTB (POZ) domain containing 2	99382	https://www.ncbi.nlm.nih.gov/gene/?term=99382
Gskip	Gskip	GSK3B interacting protein	66787	https://www.ncbi.nlm.nih.gov/gene/?term=66787
2010315B03Rik	2010315B03Rik	RIKEN cDNA 2010315B03 gene	630836	https://www.ncbi.nlm.nih.gov/gene/?term=630836
Spice1	Spice1	spindle and centriole associated protein 1	212514	https://www.ncbi.nlm.nih.gov/gene/?term=212514
Sec24a	Sec24a	Sec24 related gene family, member A (S. cerevisiae)	77371	https://www.ncbi.nlm.nih.gov/gene/?term=77371
Hey2	Hey2	hairy/enhancer-of-split related with YRPW motif 2	15214	https://www.ncbi.nlm.nih.gov/gene/?term=15214
Lclat1	Lclat1	lysocardiolipin acyltransferase 1	225010	https://www.ncbi.nlm.nih.gov/gene/?term=225010
Zbtb4	Zbtb4	zinc finger and BTB domain containing 4	75580	https://www.ncbi.nlm.nih.gov/gene/?term=75580
Ube2b	Ube2b	ubiquitin-conjugating enzyme E2B	22210	https://www.ncbi.nlm.nih.gov/gene/?term=22210
Crim1	Crim1	cysteine rich transmembrane BMP regulator 1 (chordin like)	50766	https://www.ncbi.nlm.nih.gov/gene/?term=50766
Pi15	Pi15	peptidase inhibitor 15	94227	https://www.ncbi.nlm.nih.gov/gene/?term=94227
Trak1	Trak1	trafficking protein, kinesin binding 1	67095	https://www.ncbi.nlm.nih.gov/gene/?term=67095
Lrrc32	Lrrc32	leucine rich repeat containing 32	434215	https://www.ncbi.nlm.nih.gov/gene/?term=434215
Esm1	Esm1	endothelial cell-specific molecule 1	71690	https://www.ncbi.nlm.nih.gov/gene/?term=71690
Slc35f3	Slc35f3	solute carrier family 35, member F3	210027	https://www.ncbi.nlm.nih.gov/gene/?term=210027

Biology Open • Supplementary information

1700066M21Rik	1700066M21Rik	RIKEN cDNA 1700066M21 gene	73467	https://www.ncbi.nlm.nih.gov/gene/?term=73467
Phtf2	Phtf2	putative homeodomain transcription factor 2	68770	https://www.ncbi.nlm.nih.gov/gene/?term=68770
Zfp967	Zfp967	zinc finger protein 967	100303732	https://www.ncbi.nlm.nih.gov/gene/?term=100303
Sik3	Sik3	SIK family kinase 3	70661	https://www.ncbi.nlm.nih.gov/gene/?term=70661
Zfp975	Zfp975	zinc finger protein 975	434179	https://www.ncbi.nlm.nih.gov/gene/?term=434179
Zfp965	Zfp965	zinc finger protein 965	100503949	https://www.ncbi.nlm.nih.gov/gene/?term=100503
Cbx7	Cbx7	chromobox 7	52609	https://www.ncbi.nlm.nih.gov/gene/?term=52609
Ncoa2	Ncoa2	nuclear receptor coactivator 2	17978	https://www.ncbi.nlm.nih.gov/gene/?term=17978
Cpd	Cpd	carboxypeptidase D	12874	https://www.ncbi.nlm.nih.gov/gene/?term=12874
Nwd2	Nwd2	NACHT and WD repeat domain containing 2	319807	https://www.ncbi.nlm.nih.gov/gene/?term=319807
D430041D05Rik	D430041D05Rik	RIKEN cDNA D430041D05 gene	241589	https://www.ncbi.nlm.nih.gov/gene/?term=241589
Ap4e1	Ap4e1	adaptor-related protein complex AP-4, epsilon 1	108011	https://www.ncbi.nlm.nih.gov/gene/?term=108011
Gm14296	Gm14296	predicted gene 14296	102639598	https://www.ncbi.nlm.nih.gov/gene/?term=102639
Gm14440	Gm14440	predicted gene 14440	100503353	https://www.ncbi.nlm.nih.gov/gene/?term=100503
Oxr1	Oxr1	oxidation resistance 1	170719	https://www.ncbi.nlm.nih.gov/gene/?term=170719
Jade2	Jade2	jade family PHD finger 2	76901	https://www.ncbi.nlm.nih.gov/gene/?term=76901
Prpf40a	Prpf40a	pre-mRNA processing factor 40A	56194	https://www.ncbi.nlm.nih.gov/gene/?term=56194
Tiam2	Tiam2	T cell lymphoma invasion and metastasis 2	24001	https://www.ncbi.nlm.nih.gov/gene/?term=24001
Socs5	Socs5	suppressor of cytokine signaling 5	56468	https://www.ncbi.nlm.nih.gov/gene/?term=56468
Ankrd17	Ankrd17	ankyrin repeat domain 17	81702	https://www.ncbi.nlm.nih.gov/gene/?term=81702
Mex3a	Mex3a	mex3 RNA binding family member A	72640	https://www.ncbi.nlm.nih.gov/gene/?term=72640
Pgm2	Pgm2	phosphoglucomutase 2	72157	https://www.ncbi.nlm.nih.gov/gene/?term=72157
Mylip	Mylip	myosin regulatory light chain interacting protein	218203	https://www.ncbi.nlm.nih.gov/gene/?term=218203

Rasd1	Rasd1	RAS, dexamethasone-induced 1	19416	https://www.ncbi.nlm.nih.gov/gene/?term=19416
Adm	Adm	adrenomedullin	11535	https://www.ncbi.nlm.nih.gov/gene/?term=11535
Usp48	Usp48	ubiquitin specific peptidase 48	170707	https://www.ncbi.nlm.nih.gov/gene/?term=170707
E2f2	E2f2	E2F transcription factor 2	242705	https://www.ncbi.nlm.nih.gov/gene/?term=242705
Kcnj2	Kcnj2	potassium inwardly-rectifying channel, subfamily J, member 2	16518	https://www.ncbi.nlm.nih.gov/gene/?term=16518
Mylk	Mylk	myosin, light polypeptide kinase	107589	https://www.ncbi.nlm.nih.gov/gene/?term=107589
Esrra	Esrra	estrogen related receptor, alpha	26379	https://www.ncbi.nlm.nih.gov/gene/?term=26379
Acvr1	Acvr1	activin A receptor, type 1	11477	https://www.ncbi.nlm.nih.gov/gene/?term=11477
Arrb2	Arrb2	arrestin, beta 2	216869	https://www.ncbi.nlm.nih.gov/gene/?term=216869
Hhip	Hhip	Hedgehog-interacting protein	15245	https://www.ncbi.nlm.nih.gov/gene/?term=15245
Arl15	Arl15	ADP-ribosylation factor-like 15	218639	https://www.ncbi.nlm.nih.gov/gene/?term=218639
Eif5a	Eif5a	eukaryotic translation initiation factor 5A	276770	https://www.ncbi.nlm.nih.gov/gene/?term=276770
Fstl5	Fstl5	follistatin-like 5	213262	https://www.ncbi.nlm.nih.gov/gene/?term=213262
Hs3st3b1	Hs3st3b1	heparan sulfate (glucosamine) 3-O-sulfotransferase 3B1	54710	https://www.ncbi.nlm.nih.gov/gene/?term=54710
Asb7	Asb7	ankyrin repeat and SOCS box-containing 7	117589	https://www.ncbi.nlm.nih.gov/gene/?term=117589
Gpr141b	Gpr141b	G protein-coupled receptor 141B	319293	https://www.ncbi.nlm.nih.gov/gene/?term=319293
Ctnnb1	Ctnnb1	catenin (cadherin associated protein), beta 1	12387	https://www.ncbi.nlm.nih.gov/gene/?term=12387
Azin1	Azin1	antizyme inhibitor 1	54375	https://www.ncbi.nlm.nih.gov/gene/?term=54375
Gtf2a1	Gtf2a1	general transcription factor II A, 1	83602	https://www.ncbi.nlm.nih.gov/gene/?term=83602
Tmed7	Tmed7	transmembrane p24 trafficking protein 7	66676	https://www.ncbi.nlm.nih.gov/gene/?term=66676
Prkar2b	Prkar2b	protein kinase, cAMP dependent regulatory, type II beta	19088	https://www.ncbi.nlm.nih.gov/gene/?term=19088

Spock1	Spock1	sparc/osteonectin, cwcv and kazal-like domains proteoglycan 1	20745	https://www.ncbi.nlm.nih.gov/gene/?term=20745
Trappc2	Trappc2	trafficking protein particle complex 2	66226	https://www.ncbi.nlm.nih.gov/gene/?term=66226
Mdga2	Mdga2	MAM domain containing glycosylphosphatidylinositol anchor 2	320772	https://www.ncbi.nlm.nih.gov/gene/?term=320772
Tmem255a	Tmem255a	transmembrane protein 255A	245386	https://www.ncbi.nlm.nih.gov/gene/?term=245386
Nfs1	Nfs1	nitrogen fixation gene 1 (S. cerevisiae)	18041	https://www.ncbi.nlm.nih.gov/gene/?term=18041
Kif20b	Kif20b	kinesin family member 20B	240641	https://www.ncbi.nlm.nih.gov/gene/?term=240641
Cep350	Cep350	centrosomal protein 350	74081	https://www.ncbi.nlm.nih.gov/gene/?term=74081
Cdk13	Cdk13	cyclin-dependent kinase 13	69562	https://www.ncbi.nlm.nih.gov/gene/?term=69562
Zfpm2	Zfpm2	zinc finger protein, multitype 2	22762	https://www.ncbi.nlm.nih.gov/gene/?term=22762
Sult1d1	Sult1d1	sulfotransferase family 1D, member 1	53315	https://www.ncbi.nlm.nih.gov/gene/?term=53315
Utp11	Utp11	UTP11 small subunit processome component	67205	https://www.ncbi.nlm.nih.gov/gene/?term=67205
Mrc1	Mrc1	mannose receptor, C type 1	17533	https://www.ncbi.nlm.nih.gov/gene/?term=17533
Siah1a	Siah1a	siah E3 ubiquitin protein ligase 1A	20437	https://www.ncbi.nlm.nih.gov/gene/?term=20437
Sfrp2	Sfrp2	secreted frizzled-related protein 2	20319	https://www.ncbi.nlm.nih.gov/gene/?term=20319
Prpf4b	Prpf4b	pre-mRNA processing factor 4B	19134	https://www.ncbi.nlm.nih.gov/gene/?term=19134
Bnc2	Bnc2	basonuclin 2	242509	https://www.ncbi.nlm.nih.gov/gene/?term=242509
Klhl24	Klhl24	kelch-like 24	75785	https://www.ncbi.nlm.nih.gov/gene/?term=75785
Zbtb44	Zbtb44	zinc finger and BTB domain containing 44	235132	https://www.ncbi.nlm.nih.gov/gene/?term=235132
Snca	Snca	synuclein, alpha	20617	https://www.ncbi.nlm.nih.gov/gene/?term=20617
Zmynd8	Zmynd8	zinc finger, MYND-type containing 8	228880	https://www.ncbi.nlm.nih.gov/gene/?term=228880
Schip1	Schip1	schwannomin interacting protein 1	30953	https://www.ncbi.nlm.nih.gov/gene/?term=30953
Nfib	Nfib	nuclear factor I/B	18028	https://www.ncbi.nlm.nih.gov/gene/?term=18028

Biology Open • Supplementary information

Vcan	Vcan	versican	13003	https://www.ncbi.nlm.nih.gov/gene/?term=13003
Rngtt	Rngtt	RNA guanylyltransferase and 5'-phosphatase	24018	https://www.ncbi.nlm.nih.gov/gene/?term=24018
Bcar3	Bcar3	breast cancer anti-estrogen resistance 3	29815	https://www.ncbi.nlm.nih.gov/gene/?term=29815
Rtf1	Rtf1	RTF1, Paf1/RNA polymerase II complex component	76246	https://www.ncbi.nlm.nih.gov/gene/?term=76246
Chn2	Chn2	chimerin 2	69993	https://www.ncbi.nlm.nih.gov/gene/?term=69993
Lyplal1	Lyplal1	lysophospholipase-like 1	226791	https://www.ncbi.nlm.nih.gov/gene/?term=226791
Pphln1	Pphln1	periphilin 1	223828	https://www.ncbi.nlm.nih.gov/gene/?term=223828
Gng7	Gng7	guanine nucleotide binding protein (G protein), gamma 7	14708	https://www.ncbi.nlm.nih.gov/gene/?term=14708
Cyp3a25	Cyp3a25	cytochrome P450, family 3, subfamily a, polypeptide 25	56388	https://www.ncbi.nlm.nih.gov/gene/?term=56388
Snx2	Snx2	sorting nexin 2	67804	https://www.ncbi.nlm.nih.gov/gene/?term=67804
Cpne8	Cpne8	copine VIII	66871	https://www.ncbi.nlm.nih.gov/gene/?term=66871
Hdac1	Hdac1	histone deacetylase 1	433759	https://www.ncbi.nlm.nih.gov/gene/?term=433759
Zfp948	Zfp948	zinc finger protein 948	381066	https://www.ncbi.nlm.nih.gov/gene/?term=381066
Gbp7	Gbp7	guanylate binding protein 7	229900	https://www.ncbi.nlm.nih.gov/gene/?term=229900
Strbp	Strbp	spermatid perinuclear RNA binding protein	20744	https://www.ncbi.nlm.nih.gov/gene/?term=20744
Ei24	Ei24	etoposide induced 2.4 mRNA	13663	https://www.ncbi.nlm.nih.gov/gene/?term=13663
Csnk1g3	Csnk1g3	casein kinase 1, gamma 3	70425	https://www.ncbi.nlm.nih.gov/gene/?term=70425
Mab21l1	Mab21l1	mab-21-like 1 (C. elegans)	17116	https://www.ncbi.nlm.nih.gov/gene/?term=17116
Drd1	Drd1	dopamine receptor D1	13488	https://www.ncbi.nlm.nih.gov/gene/?term=13488
Cd163	Cd163	CD163 antigen	93671	https://www.ncbi.nlm.nih.gov/gene/?term=93671
Snap91	Snap91	synaptosomal-associated protein 91	20616	https://www.ncbi.nlm.nih.gov/gene/?term=20616
Ppp1r14c	Ppp1r14c	protein phosphatase 1, regulatory inhibitor subunit 14C	76142	https://www.ncbi.nlm.nih.gov/gene/?term=76142

Biology Open • Supplementary information

Sgip1	Sgip1	SH3-domain GRB2-like (endophilin) interacting protein 1	73094	https://www.ncbi.nlm.nih.gov/gene/?term=73094
Sox21	Sox21	SRY (sex determining region Y)-box 21	223227	https://www.ncbi.nlm.nih.gov/gene/?term=223227
Nhsl2	Nhsl2	NHS-like 2	100042480	https://www.ncbi.nlm.nih.gov/gene/?term=100042[illegible]
Kbtbd7	Kbtbd7	kelch repeat and BTB (POZ) domain containing 7	211255	https://www.ncbi.nlm.nih.gov/gene/?term=211255
Hmgn5	Hmgn5	high-mobility group nucleosome binding domain 5	50887	https://www.ncbi.nlm.nih.gov/gene/?term=50887
Dnm1l	Dnm1l	dynamin 1-like	74006	https://www.ncbi.nlm.nih.gov/gene/?term=74006
Tial1	Tial1	Tia1 cytotoxic granule-associated RNA binding protein-like 1	21843	https://www.ncbi.nlm.nih.gov/gene/?term=21843
Uap1	Uap1	UDP-N-acetylglucosamine pyrophosphorylase 1	107652	https://www.ncbi.nlm.nih.gov/gene/?term=107652
Calcr	Calcr	calcitonin receptor	12311	https://www.ncbi.nlm.nih.gov/gene/?term=12311
Morc3	Morc3	microrchidia 3	338467	https://www.ncbi.nlm.nih.gov/gene/?term=338467
Dennd6a	Dennd6a	DENN/MADD domain containing 6A	211922	https://www.ncbi.nlm.nih.gov/gene/?term=211922
Dixdc1	Dixdc1	DIX domain containing 1	330938	https://www.ncbi.nlm.nih.gov/gene/?term=330938
Epcam	Epcam	epithelial cell adhesion molecule	17075	https://www.ncbi.nlm.nih.gov/gene/?term=17075
Zbtb43	Zbtb43	zinc finger and BTB domain containing 43	71834	https://www.ncbi.nlm.nih.gov/gene/?term=71834
Ppfibp1	Ppfibp1	PTPRF interacting protein, binding protein 1 (liprin beta 1)	67533	https://www.ncbi.nlm.nih.gov/gene/?term=67533
Clasp2	Clasp2	CLIP associating protein 2	76499	https://www.ncbi.nlm.nih.gov/gene/?term=76499
Scoc	Scoc	short coiled-coil protein	56367	https://www.ncbi.nlm.nih.gov/gene/?term=56367
Ctbp2	Ctbp2	C-terminal binding protein 2	13017	https://www.ncbi.nlm.nih.gov/gene/?term=13017
Kif2a	Kif2a	kinesin family member 2A	16563	https://www.ncbi.nlm.nih.gov/gene/?term=16563
Hnrnpa3	Hnrnpa3	heterogeneous nuclear ribonucleoprotein A3	229279	https://www.ncbi.nlm.nih.gov/gene/?term=229279

Sar1a	Sar1a	secretion associated Ras related GTPase 1A	20224	https://www.ncbi.nlm.nih.gov/gene/?term=20224
Atp13a3	Atp13a3	ATPase type 13A3	224088	https://www.ncbi.nlm.nih.gov/gene/?term=224088
Htr2c	Htr2c	5-hydroxytryptamine (serotonin) receptor 2C	15560	https://www.ncbi.nlm.nih.gov/gene/?term=15560
Cwc22	Cwc22	CWC22 spliceosome-associated protein	80744	https://www.ncbi.nlm.nih.gov/gene/?term=80744
Metap1	Metap1	methionyl aminopeptidase 1	75624	https://www.ncbi.nlm.nih.gov/gene/?term=75624
Mob3c	Mob3c	MOB kinase activator 3C	100465	https://www.ncbi.nlm.nih.gov/gene/?term=100465
Nuak2	Nuak2	NUAK family, SNF1-like kinase, 2	74137	https://www.ncbi.nlm.nih.gov/gene/?term=74137
Bclaf3	Bclaf3	Bclaf1 and Thrap3 family member 3	382252	https://www.ncbi.nlm.nih.gov/gene/?term=382252
Stk26	Stk26	serine/threonine kinase 26	70415	https://www.ncbi.nlm.nih.gov/gene/?term=70415
2210010C04Rik	2210010C04Rik	RIKEN cDNA 2210010C04 gene	67373	https://www.ncbi.nlm.nih.gov/gene/?term=67373
Cacna1g	Cacna1g	calcium channel, voltage-dependent, T type, alpha 1G subunit	12291	https://www.ncbi.nlm.nih.gov/gene/?term=12291
Ptprm	Ptprm	protein tyrosine phosphatase, receptor type, M	19274	https://www.ncbi.nlm.nih.gov/gene/?term=19274
Pcdh19	Pcdh19	protocadherin 19	279653	https://www.ncbi.nlm.nih.gov/gene/?term=279653
Tspan7	Tspan7	tetraspanin 7	21912	https://www.ncbi.nlm.nih.gov/gene/?term=21912
Rock1	Rock1	Rho-associated coiled-coil containing protein kinase 1	19877	https://www.ncbi.nlm.nih.gov/gene/?term=19877
Bmt2	Bmt2	base methyltransferase of 25S rRNA 2	101148	https://www.ncbi.nlm.nih.gov/gene/?term=101148
Ptgs2	Ptgs2	prostaglandin-endoperoxide synthase 2	19225	https://www.ncbi.nlm.nih.gov/gene/?term=19225
Arid4b	Arid4b	AT rich interactive domain 4B (RBP1-like)	94246	https://www.ncbi.nlm.nih.gov/gene/?term=94246
Slc30a8	Slc30a8	solute carrier family 30 (zinc transporter), member 8	239436	https://www.ncbi.nlm.nih.gov/gene/?term=239436

Biology Open • Supplementary information

Ppp3r1	Ppp3r1	protein phosphatase 3, regulatory subunit B, alpha isoform (calcineurin B, type I)	19058	https://www.ncbi.nlm.nih.gov/gene/?term=19058
Psd3	Psd3	pleckstrin and Sec7 domain containing 3	234353	https://www.ncbi.nlm.nih.gov/gene/?term=234353
Six4	Six4	sine oculis-related homeobox 4	20474	https://www.ncbi.nlm.nih.gov/gene/?term=20474
Ltn1	Ltn1	listerin E3 ubiquitin protein ligase 1	78913	https://www.ncbi.nlm.nih.gov/gene/?term=78913
Hnrnpk	Hnrnpk	heterogeneous nuclear ribonucleoprotein K	15387	https://www.ncbi.nlm.nih.gov/gene/?term=15387
Taf3	Taf3	TATA-box binding protein associated factor 3	209361	https://www.ncbi.nlm.nih.gov/gene/?term=209361
Dnaja2	Dnaja2	DnaJ heat shock protein family (Hsp40) member A2	56445	https://www.ncbi.nlm.nih.gov/gene/?term=56445
Cebpg	Cebpg	CCAAT/enhancer binding protein (C/EBP), gamma	12611	https://www.ncbi.nlm.nih.gov/gene/?term=12611
Gimap4	Gimap4	GTPase, IMAP family member 4	107526	https://www.ncbi.nlm.nih.gov/gene/?term=107526
Eif4h	Eif4h	eukaryotic translation initiation factor 4H	22384	https://www.ncbi.nlm.nih.gov/gene/?term=22384
Galntl6	Galntl6	UDP-N-acetyl-alpha-D-galactosamine:polypeptide N-acetylgalactosaminyltransferase-like 6	270049	https://www.ncbi.nlm.nih.gov/gene/?term=270049
Tmem196	Tmem196	transmembrane protein 196	217951	https://www.ncbi.nlm.nih.gov/gene/?term=217951
Sema5a	Sema5a	sema domain, seven thrombospondin repeats (type 1 and type 1-like), transmembrane domain (TM) and short cytoplasmic domain, (semaphorin) 5A	20356	https://www.ncbi.nlm.nih.gov/gene/?term=20356
Btrc	Btrc	beta-transducin repeat containing protein	12234	https://www.ncbi.nlm.nih.gov/gene/?term=12234

Biology Open • Supplementary information

Mesp1	Mesp1	mesoderm posterior 1	17292	https://www.ncbi.nlm.nih.gov/gene/?term=17292
1700018B08Rik	1700018B08Rik	RIKEN cDNA 1700018B08 gene	76405	https://www.ncbi.nlm.nih.gov/gene/?term=76405
Slc22a3	Slc22a3	solute carrier family 22 (organic cation transporter), member 3	20519	https://www.ncbi.nlm.nih.gov/gene/?term=20519
Arid2	Arid2	AT rich interactive domain 2 (ARID, RFX-like)	77044	https://www.ncbi.nlm.nih.gov/gene/?term=77044
Hnrnpc	Hnrnpc	heterogeneous nuclear ribonucleoprotein C	15381	https://www.ncbi.nlm.nih.gov/gene/?term=15381
Nr2e1	Nr2e1	nuclear receptor subfamily 2, group E, member 1	21907	https://www.ncbi.nlm.nih.gov/gene/?term=21907
Mtdh	Mtdh	metadherin	67154	https://www.ncbi.nlm.nih.gov/gene/?term=67154
U2surp	U2surp	U2 snRNP-associated SURP domain containing	67958	https://www.ncbi.nlm.nih.gov/gene/?term=67958
Ttc39c	Ttc39c	tetratricopeptide repeat domain 39C	72747	https://www.ncbi.nlm.nih.gov/gene/?term=72747
Zbtb6	Zbtb6	zinc finger and BTB domain containing 6	241322	https://www.ncbi.nlm.nih.gov/gene/?term=241322
Abhd17b	Abhd17b	abhydrolase domain containing 17B	226016	https://www.ncbi.nlm.nih.gov/gene/?term=226016
Klhl2	Klhl2	kelch-like 2, Mayven	77113	https://www.ncbi.nlm.nih.gov/gene/?term=77113
Pdzrn4	Pdzrn4	PDZ domain containing RING finger 4	239618	https://www.ncbi.nlm.nih.gov/gene/?term=239618
Adamtsl1	Adamtsl1	ADAMTS-like 1	77739	https://www.ncbi.nlm.nih.gov/gene/?term=77739
Papss2	Papss2	3'-phosphoadenosine 5'-phosphosulfate synthase 2	23972	https://www.ncbi.nlm.nih.gov/gene/?term=23972
Agtr1b	Agtr1b	angiotensin II receptor, type 1b	11608	https://www.ncbi.nlm.nih.gov/gene/?term=11608
Apol7a	Apol7a	apolipoprotein L 7a	75761	https://www.ncbi.nlm.nih.gov/gene/?term=75761
Gusb	Gusb	glucuronidase, beta	110006	https://www.ncbi.nlm.nih.gov/gene/?term=110006
Ror2	Ror2	receptor tyrosine kinase-like orphan receptor 2	26564	https://www.ncbi.nlm.nih.gov/gene/?term=26564
Reep3	Reep3	receptor accessory protein 3	28193	https://www.ncbi.nlm.nih.gov/gene/?term=28193
Dner	Dner	delta/notch-like EGF repeat	227325	https://www.ncbi.nlm.nih.gov/gene/?term=227325

		containing		
Cacna2d1	Cacna2d1	calcium channel, voltage-dependent, alpha2/delta subunit 1	12293	https://www.ncbi.nlm.nih.gov/gene/?term=12293
Bicd2	Bicd2	BICD cargo adaptor 2	76895	https://www.ncbi.nlm.nih.gov/gene/?term=76895
Atp6v0b	Atp6v0b	ATPase, H+ transporting, lysosomal V0 subunit B	114143	https://www.ncbi.nlm.nih.gov/gene/?term=114143
Yy2	Yy2	Yy2 transcription factor	100073351	https://www.ncbi.nlm.nih.gov/gene/?term=100073
Arhgap15	Arhgap15	Rho GTPase activating protein 15	76117	https://www.ncbi.nlm.nih.gov/gene/?term=76117
Eea1	Eea1	early endosome antigen 1	216238	https://www.ncbi.nlm.nih.gov/gene/?term=216238
Lemd3	Lemd3	LEM domain containing 3	380664	https://www.ncbi.nlm.nih.gov/gene/?term=380664
Usp10	Usp10	ubiquitin specific peptidase 10	22224	https://www.ncbi.nlm.nih.gov/gene/?term=22224
Mdh1	Mdh1	malate dehydrogenase 1, NAD (soluble)	17449	https://www.ncbi.nlm.nih.gov/gene/?term=17449
Scai	Scai	suppressor of cancer cell invasion	320271	https://www.ncbi.nlm.nih.gov/gene/?term=320271
Arhgap32	Arhgap32	Rho GTPase activating protein 32	330914	https://www.ncbi.nlm.nih.gov/gene/?term=330914
Rlim	Rlim	ring finger protein, LIM domain interacting	19820	https://www.ncbi.nlm.nih.gov/gene/?term=19820
Zfhx4	Zfhx4	zinc finger homeodomain 4	80892	https://www.ncbi.nlm.nih.gov/gene/?term=80892
Gbx2	Gbx2	gastrulation brain homeobox 2	14472	https://www.ncbi.nlm.nih.gov/gene/?term=14472
Spry2	Spry2	sprouty RTK signaling antagonist 2	24064	https://www.ncbi.nlm.nih.gov/gene/?term=24064
Sh3bgrl2	Sh3bgrl2	SH3 domain binding glutamic acid-rich protein like 2	212531	https://www.ncbi.nlm.nih.gov/gene/?term=212531
Dmd	Dmd	dystrophin, muscular dystrophy	13405	https://www.ncbi.nlm.nih.gov/gene/?term=13405
Fam217a	Fam217a	family with sequence similarity 217, member A	71864	https://www.ncbi.nlm.nih.gov/gene/?term=71864
Brinp2	Brinp2	bone morphogenic protein/retinoic acid inducible neural-specific 2	240843	https://www.ncbi.nlm.nih.gov/gene/?term=240843
Zfp950	Zfp950	zinc finger protein 950	414758	https://www.ncbi.nlm.nih.gov/gene/?term=414758

Biology Open • Supplementary information

Nr0b1	Nr0b1	nuclear receptor subfamily 0, group B, member 1	11614	https://www.ncbi.nlm.nih.gov/gene/?term=11614
Btg1	Btg1	B cell translocation gene 1, anti-proliferative	12226	https://www.ncbi.nlm.nih.gov/gene/?term=12226
Crot	Crot	carnitine O-octanoyltransferase	74114	https://www.ncbi.nlm.nih.gov/gene/?term=74114
Egr1	Egr1	early growth response 1	13653	https://www.ncbi.nlm.nih.gov/gene/?term=13653
Mtcp1	Mtcp1	mature T cell proliferation 1	17763	https://www.ncbi.nlm.nih.gov/gene/?term=17763
Tbc1d32	Tbc1d32	TBC1 domain family, member 32	544696	https://www.ncbi.nlm.nih.gov/gene/?term=544696
Suz12	Suz12	SUZ12 polycomb repressive complex 2 subunit	52615	https://www.ncbi.nlm.nih.gov/gene/?term=52615
Ttc21b	Ttc21b	tetratricopeptide repeat domain 21B	73668	https://www.ncbi.nlm.nih.gov/gene/?term=73668
H2-M10.6	H2-M10.6	histocompatibility 2, M region locus 10.6	399549	https://www.ncbi.nlm.nih.gov/gene/?term=399549
Abce1	Abce1	ATP-binding cassette, sub-family E (OABP), member 1	24015	https://www.ncbi.nlm.nih.gov/gene/?term=24015
Gm14295	Gm14295	predicted gene 14295	100039123	https://www.ncbi.nlm.nih.gov/gene/?term=100039
Gclc	Gclc	glutamate-cysteine ligase, catalytic subunit	14629	https://www.ncbi.nlm.nih.gov/gene/?term=14629
Chrna5	Chrna5	cholinergic receptor, nicotinic, alpha polypeptide 5	110835	https://www.ncbi.nlm.nih.gov/gene/?term=110835
Crebzf	Crebzf	CREB/ATF bZIP transcription factor	233490	https://www.ncbi.nlm.nih.gov/gene/?term=233490
Msl2	Msl2	MSL complex subunit 2	77853	https://www.ncbi.nlm.nih.gov/gene/?term=77853
Ddx42	Ddx42	DEAD (Asp-Glu-Ala-Asp) box polypeptide 42	72047	https://www.ncbi.nlm.nih.gov/gene/?term=72047
Ppp4r4	Ppp4r4	protein phosphatase 4, regulatory subunit 4	74521	https://www.ncbi.nlm.nih.gov/gene/?term=74521
Cyb5r4	Cyb5r4	cytochrome b5 reductase 4	266690	https://www.ncbi.nlm.nih.gov/gene/?term=266690
Clec3a	Clec3a	C-type lectin domain family 3, member a	403395	https://www.ncbi.nlm.nih.gov/gene/?term=403395

Biology Open • Supplementary information

Zmat3	Zmat3	zinc finger matrin type 3	22401	https://www.ncbi.nlm.nih.gov/gene/?term=22401
Cxadr	Cxadr	coxsackie virus and adenovirus receptor	13052	https://www.ncbi.nlm.nih.gov/gene/?term=13052
Tmem200a	Tmem200a	transmembrane protein 200A	77220	https://www.ncbi.nlm.nih.gov/gene/?term=77220
Msi2	Msi2	musashi RNA-binding protein 2	76626	https://www.ncbi.nlm.nih.gov/gene/?term=76626
Cdyl2	Cdyl2	chromodomain protein, Y chromosome-like 2	75796	https://www.ncbi.nlm.nih.gov/gene/?term=75796
Klrb1b	Klrb1b	killer cell lectin-like receptor subfamily B member 1B	80782	https://www.ncbi.nlm.nih.gov/gene/?term=80782
Usp21	Usp21	ubiquitin specific peptidase 21	30941	https://www.ncbi.nlm.nih.gov/gene/?term=30941
Xiap	Xiap	X-linked inhibitor of apoptosis	11798	https://www.ncbi.nlm.nih.gov/gene/?term=11798
Zfp119b	Zfp119b	zinc finger protein 119b	240120	https://www.ncbi.nlm.nih.gov/gene/?term=240120
Sertm1	Sertm1	serine rich and transmembrane domain containing 1	329641	https://www.ncbi.nlm.nih.gov/gene/?term=329641
Ids	Ids	iduronate 2-sulfatase	15931	https://www.ncbi.nlm.nih.gov/gene/?term=15931
Pcdh11x	Pcdh11x	protocadherin 11 X-linked	245578	https://www.ncbi.nlm.nih.gov/gene/?term=245578
Herc4	Herc4	hect domain and RLD 4	67345	https://www.ncbi.nlm.nih.gov/gene/?term=67345
Bend4	Bend4	BEN domain containing 4	666938	https://www.ncbi.nlm.nih.gov/gene/?term=666938
Dlx1	Dlx1	distal-less homeobox 1	13390	https://www.ncbi.nlm.nih.gov/gene/?term=13390
Rif1	Rif1	replication timing regulatory factor 1	51869	https://www.ncbi.nlm.nih.gov/gene/?term=51869
Gria4	Gria4	glutamate receptor, ionotropic, AMPA4 (alpha 4)	14802	https://www.ncbi.nlm.nih.gov/gene/?term=14802
Nupl1	Nupl1	nucleoporin like 1	71844	https://www.ncbi.nlm.nih.gov/gene/?term=71844
Vxn	Vxn	vexin	76982	https://www.ncbi.nlm.nih.gov/gene/?term=76982
Adcyap1	Adcyap1	adenylate cyclase activating polypeptide 1	11516	https://www.ncbi.nlm.nih.gov/gene/?term=11516
Frmd3	Frmd3	FERM domain containing 3	242506	https://www.ncbi.nlm.nih.gov/gene/?term=242506
Neurl1a	Neurl1a	neuralized E3 ubiquitin protein ligase 1A	18011	https://www.ncbi.nlm.nih.gov/gene/?term=18011

Setx	Setx	senataxin	269254	https://www.ncbi.nlm.nih.gov/gene/?term=269254
Lhx8	Lhx8	LIM homeobox protein 8	16875	https://www.ncbi.nlm.nih.gov/gene/?term=16875
Bicd1	Bicd1	BICD cargo adaptor 1	12121	https://www.ncbi.nlm.nih.gov/gene/?term=12121
Atl2	Atl2	atlastin GTPase 2	56298	https://www.ncbi.nlm.nih.gov/gene/?term=56298
Brwd1	Brwd1	bromodomain and WD repeat domain containing 1	93871	https://www.ncbi.nlm.nih.gov/gene/?term=93871
Prr3	Prr3	proline-rich polypeptide 3	75210	https://www.ncbi.nlm.nih.gov/gene/?term=75210
Dscam	Dscam	DS cell adhesion molecule	13508	https://www.ncbi.nlm.nih.gov/gene/?term=13508
Cxxc5	Cxxc5	CXXC finger 5	67393	https://www.ncbi.nlm.nih.gov/gene/?term=67393
Kcnh8	Kcnh8	potassium voltage-gated channel, subfamily H (eag-related), member 8	211468	https://www.ncbi.nlm.nih.gov/gene/?term=211468
Dclre1a	Dclre1a	DNA cross-link repair 1A	55947	https://www.ncbi.nlm.nih.gov/gene/?term=55947
Zfp383	Zfp383	zinc finger protein 383	73729	https://www.ncbi.nlm.nih.gov/gene/?term=73729
Sash3	Sash3	SAM and SH3 domain containing 3	74131	https://www.ncbi.nlm.nih.gov/gene/?term=74131
Stard6	Stard6	StAR-related lipid transfer (START) domain containing 6	170461	https://www.ncbi.nlm.nih.gov/gene/?term=170461
Nup54	Nup54	nucleoporin 54	269113	https://www.ncbi.nlm.nih.gov/gene/?term=269113
Smad4	Smad4	SMAD family member 4	17128	https://www.ncbi.nlm.nih.gov/gene/?term=17128
Ppp4r3b	Ppp4r3b	protein phosphatase 4 regulatory subunit 3B	104570	https://www.ncbi.nlm.nih.gov/gene/?term=104570
Tmem158	Tmem158	transmembrane protein 158	72309	https://www.ncbi.nlm.nih.gov/gene/?term=72309
Slit3	Slit3	slit guidance ligand 3	20564	https://www.ncbi.nlm.nih.gov/gene/?term=20564
Slc30a10	Slc30a10	solute carrier family 30, member 10	226781	https://www.ncbi.nlm.nih.gov/gene/?term=226781
Cdc73	Cdc73	cell division cycle 73, Paf1/RNA polymerase II complex component	214498	https://www.ncbi.nlm.nih.gov/gene/?term=214498
Rrp36	Rrp36	ribosomal RNA processing 36 homolog (S. cerevisiae)	224823	https://www.ncbi.nlm.nih.gov/gene/?term=224823
Tmem68	Tmem68	transmembrane protein 68	72098	https://www.ncbi.nlm.nih.gov/gene/?term=72098

Biology Open • Supplementary information

Map3k2	Map3k2	mitogen-activated protein kinase kinase kinase 2	26405	https://www.ncbi.nlm.nih.gov/gene/?term=26405
Serpinb10	Serpinb10	serine (or cysteine) peptidase inhibitor, clade B (ovalbumin), member 10	241197	https://www.ncbi.nlm.nih.gov/gene/?term=241197
Rpe65	Rpe65	retinal pigment epithelium 65	19892	https://www.ncbi.nlm.nih.gov/gene/?term=19892
Lig3	Lig3	ligase III, DNA, ATP-dependent	16882	https://www.ncbi.nlm.nih.gov/gene/?term=16882
Daam1	Daam1	dishevelled associated activator of morphogenesis 1	208846	https://www.ncbi.nlm.nih.gov/gene/?term=208846
Calu	Calu	calumenin	12321	https://www.ncbi.nlm.nih.gov/gene/?term=12321
Kdm4c	Kdm4c	lysine (K)-specific demethylase 4C	76804	https://www.ncbi.nlm.nih.gov/gene/?term=76804
Cdh19	Cdh19	cadherin 19, type 2	227485	https://www.ncbi.nlm.nih.gov/gene/?term=227485
2510009E07Rik	2510009E07Rik	RIKEN cDNA 2510009E07 gene	72190	https://www.ncbi.nlm.nih.gov/gene/?term=72190
Fbxw27	Fbxw27	F-box and WD-40 domain protein 27	76998	https://www.ncbi.nlm.nih.gov/gene/?term=76998
Poglut1	Poglut1	protein O-glucosyltransferase 1	224143	https://www.ncbi.nlm.nih.gov/gene/?term=224143
Slc15a5	Slc15a5	solute carrier family 15, member 5	277898	https://www.ncbi.nlm.nih.gov/gene/?term=277898
Chfr	Chfr	checkpoint with forkhead and ring finger domains	231600	https://www.ncbi.nlm.nih.gov/gene/?term=231600
Sorcs1	Sorcs1	sortilin-related VPS10 domain containing receptor 1	58178	https://www.ncbi.nlm.nih.gov/gene/?term=58178
Pdhx	Pdhx	pyruvate dehydrogenase complex, component X	27402	https://www.ncbi.nlm.nih.gov/gene/?term=27402
Hoxd8	Hoxd8	homeobox D8	15437	https://www.ncbi.nlm.nih.gov/gene/?term=15437
BC003965	BC003965	cDNA sequence BC003965	214489	https://www.ncbi.nlm.nih.gov/gene/?term=214489
Sp1	Sp1	trans-acting transcription factor 1	20683	https://www.ncbi.nlm.nih.gov/gene/?term=20683
Thap2	Thap2	THAP domain containing, apoptosis associated protein 2	66816	https://www.ncbi.nlm.nih.gov/gene/?term=66816
Nt5dc3	Nt5dc3	5'-nucleotidase domain containing 3	103466	https://www.ncbi.nlm.nih.gov/gene/?term=103466
Cyb5d2	Cyb5d2	cytochrome b5 domain containing 2	192986	https://www.ncbi.nlm.nih.gov/gene/?term=192986

Biology Open • Supplementary information

Fam120c	Fam120c	family with sequence similarity 120, member C	207375	https://www.ncbi.nlm.nih.gov/gene/?term=207375
Hs3st2	Hs3st2	heparan sulfate (glucosamine) 3-O-sulfotransferase 2	195646	https://www.ncbi.nlm.nih.gov/gene/?term=195646
Ttc39a	Ttc39a	tetratricopeptide repeat domain 39A	230603	https://www.ncbi.nlm.nih.gov/gene/?term=230603
Fgf21	Fgf21	NA	56636	https://www.ncbi.nlm.nih.gov/gene/?term=56636
Ap1ar	Ap1ar	adaptor-related protein complex 1 associated regulatory protein	211556	https://www.ncbi.nlm.nih.gov/gene/?term=211556
Baz2a	Baz2a	bromodomain adjacent to zinc finger domain, 2A	116848	https://www.ncbi.nlm.nih.gov/gene/?term=116848
Vstm4	Vstm4	V-set and transmembrane domain containing 4	320736	https://www.ncbi.nlm.nih.gov/gene/?term=320736
Mro	Mro	maestro	71263	https://www.ncbi.nlm.nih.gov/gene/?term=71263
Togaram2	Togaram2	TOG array regulator of axonemal microtubules 2	320159	https://www.ncbi.nlm.nih.gov/gene/?term=320159
Nckap1	Nckap1	NCK-associated protein 1	50884	https://www.ncbi.nlm.nih.gov/gene/?term=50884
Ppp1r9a	Ppp1r9a	protein phosphatase 1, regulatory subunit 9A	243725	https://www.ncbi.nlm.nih.gov/gene/?term=243725
Dpp8	Dpp8	dipeptidylpeptidase 8	74388	https://www.ncbi.nlm.nih.gov/gene/?term=74388
Ehbp1	Ehbp1	EH domain binding protein 1	216565	https://www.ncbi.nlm.nih.gov/gene/?term=216565
Pls1	Pls1	plastin 1 (I-isoform)	102502	https://www.ncbi.nlm.nih.gov/gene/?term=102502
Ypel1	Ypel1	yippee like 1	106369	https://www.ncbi.nlm.nih.gov/gene/?term=106369
Ephb1	Ephb1	Eph receptor B1	270190	https://www.ncbi.nlm.nih.gov/gene/?term=270190
Muc15	Muc15	mucin 15	269328	https://www.ncbi.nlm.nih.gov/gene/?term=269328
Naa16	Naa16	N(alpha)-acetyltransferase 16, NatA auxiliary subunit	66897	https://www.ncbi.nlm.nih.gov/gene/?term=66897
Gpr158	Gpr158	G protein-coupled receptor 158	241263	https://www.ncbi.nlm.nih.gov/gene/?term=241263
Nckap5	Nckap5	NCK-associated protein 5	210356	https://www.ncbi.nlm.nih.gov/gene/?term=210356
Mettl21e	Mettl21e	methyltransferase like 21E	403183	https://www.ncbi.nlm.nih.gov/gene/?term=403183

Biology Open • Supplementary information

Ggnbp2	Ggnbp2	gametogenetin binding protein 2	217039	https://www.ncbi.nlm.nih.gov/gene/?term=217039
Tceal8	Tceal8	transcription elongation factor A (SII)-like 8	66684	https://www.ncbi.nlm.nih.gov/gene/?term=66684
Notum	Notum	notum palmitoleoyl-protein carboxylesterase	77583	https://www.ncbi.nlm.nih.gov/gene/?term=77583
Me3	Me3	malic enzyme 3, NADP(+)-dependent, mitochondrial	109264	https://www.ncbi.nlm.nih.gov/gene/?term=109264
Edil3	Edil3	EGF-like repeats and discoidin I-like domains 3	13612	https://www.ncbi.nlm.nih.gov/gene/?term=13612
Mycbp	Mycbp	MYC binding protein	56309	https://www.ncbi.nlm.nih.gov/gene/?term=56309
Hspa4l	Hspa4l	heat shock protein 4 like	18415	https://www.ncbi.nlm.nih.gov/gene/?term=18415
Kcnip2	Kcnip2	Kv channel-interacting protein 2	80906	https://www.ncbi.nlm.nih.gov/gene/?term=80906
Klk4	Klk4	kallikrein related-peptidase 4 (prostase, enamel matrix, prostate)	56640	https://www.ncbi.nlm.nih.gov/gene/?term=56640
Zmpste24	Zmpste24	zinc metallopeptidase, STE24	230709	https://www.ncbi.nlm.nih.gov/gene/?term=230709
Elk4	Elk4	ELK4, member of ETS oncogene family	13714	https://www.ncbi.nlm.nih.gov/gene/?term=13714
Rab10	Rab10	RAB10, member RAS oncogene family	19325	https://www.ncbi.nlm.nih.gov/gene/?term=19325
Rbm12b2	Rbm12b2	RNA binding motif protein 12 B2	77604	https://www.ncbi.nlm.nih.gov/gene/?term=77604
Eri1	Eri1	exoribonuclease 1	67276	https://www.ncbi.nlm.nih.gov/gene/?term=67276
Crebl2	Crebl2	cAMP responsive element binding protein-like 2	232430	https://www.ncbi.nlm.nih.gov/gene/?term=232430
Sim2	Sim2	single-minded family bHLH transcription factor 2	20465	https://www.ncbi.nlm.nih.gov/gene/?term=20465
Glrb	Glrb	glycine receptor, beta subunit	14658	https://www.ncbi.nlm.nih.gov/gene/?term=14658
Fchsd2	Fchsd2	FCH and double SH3 domains 2	207278	https://www.ncbi.nlm.nih.gov/gene/?term=207278
Rora	Rora	RAR-related orphan receptor alpha	19883	https://www.ncbi.nlm.nih.gov/gene/?term=19883
Zfp62	Zfp62	zinc finger protein 62	22720	https://www.ncbi.nlm.nih.gov/gene/?term=22720

Trappc10	Trappc10	trafficking protein particle complex 10	216131	https://www.ncbi.nlm.nih.gov/gene/?term=216131
Pnisr	Pnisr	PNN interacting serine/arginine-rich	66625	https://www.ncbi.nlm.nih.gov/gene/?term=66625
Pias3	Pias3	protein inhibitor of activated STAT 3	229615	https://www.ncbi.nlm.nih.gov/gene/?term=229615
Irf2	Irf2	interferon regulatory factor 2	16363	https://www.ncbi.nlm.nih.gov/gene/?term=16363
Rsu1	Rsu1	Ras suppressor protein 1	20163	https://www.ncbi.nlm.nih.gov/gene/?term=20163
Klhl20	Klhl20	kelch-like 20	226541	https://www.ncbi.nlm.nih.gov/gene/?term=226541
Fgf1	Fgf1	fibroblast growth factor 1	14164	https://www.ncbi.nlm.nih.gov/gene/?term=14164
Tcaf2	Tcaf2	TRPM8 channel-associated factor 2	232748	https://www.ncbi.nlm.nih.gov/gene/?term=232748
Spata1	Spata1	spermatogenesis associated 1	70951	https://www.ncbi.nlm.nih.gov/gene/?term=70951
Patj	Patj	PATJ, crumbs cell polarity complex component	12695	https://www.ncbi.nlm.nih.gov/gene/?term=12695
Tmem100	Tmem100	transmembrane protein 100	67888	https://www.ncbi.nlm.nih.gov/gene/?term=67888
Gpr37	Gpr37	G protein-coupled receptor 37	14763	https://www.ncbi.nlm.nih.gov/gene/?term=14763
Serpinc1	Serpinc1	serine (or cysteine) peptidase inhibitor, clade C (antithrombin), member 1	11905	https://www.ncbi.nlm.nih.gov/gene/?term=11905
Nacc1	Nacc1	nucleus accumbens associated 1, BEN and BTB (POZ) domain containing	66830	https://www.ncbi.nlm.nih.gov/gene/?term=66830
Ptgfrn	Ptgfrn	prostaglandin F2 receptor negative regulator	19221	https://www.ncbi.nlm.nih.gov/gene/?term=19221
Kpna6	Kpna6	karyopherin (importin) alpha 6	16650	https://www.ncbi.nlm.nih.gov/gene/?term=16650
Nr1h2	Nr1h2	nuclear receptor subfamily 1, group H, member 2	22260	https://www.ncbi.nlm.nih.gov/gene/?term=22260
Hif1a	Hif1a	hypoxia inducible factor 1, alpha subunit	15251	https://www.ncbi.nlm.nih.gov/gene/?term=15251
Rhag	Rhag	Rhesus blood group-associated A glycoprotein	19743	https://www.ncbi.nlm.nih.gov/gene/?term=19743

Biology Open • Supplementary information

Syt14	Syt14	synaptotagmin XIV	329324	https://www.ncbi.nlm.nih.gov/gene/?term=329324
Epb41l3	Epb41l3	erythrocyte membrane protein band 4.1 like 3	13823	https://www.ncbi.nlm.nih.gov/gene/?term=13823
Chuk	Chuk	conserved helix-loop-helix ubiquitous kinase	12675	https://www.ncbi.nlm.nih.gov/gene/?term=12675
Spx	Spx	spexin hormone	319552	https://www.ncbi.nlm.nih.gov/gene/?term=319552
Zfp830	Zfp830	zinc finger protein 830	66983	https://www.ncbi.nlm.nih.gov/gene/?term=66983
Slc26a3	Slc26a3	solute carrier family 26, member 3	13487	https://www.ncbi.nlm.nih.gov/gene/?term=13487
Psip1	Psip1	PC4 and SFRS1 interacting protein 1	101739	https://www.ncbi.nlm.nih.gov/gene/?term=101739
Ciao2a	Ciao2a	cytosolic iron-sulfur assembly component 2A	68250	https://www.ncbi.nlm.nih.gov/gene/?term=68250
Gpr149	Gpr149	G protein-coupled receptor 149	229357	https://www.ncbi.nlm.nih.gov/gene/?term=229357
Bhlhb9	Bhlhb9	basic helix-loop-helix domain containing, class B9	70237	https://www.ncbi.nlm.nih.gov/gene/?term=70237
Strn3	Strn3	striatin, calmodulin binding protein 3	94186	https://www.ncbi.nlm.nih.gov/gene/?term=94186
Nmt2	Nmt2	N-myristoyltransferase 2	18108	https://www.ncbi.nlm.nih.gov/gene/?term=18108
Glb1l3	Glb1l3	galactosidase, beta 1 like 3	70893	https://www.ncbi.nlm.nih.gov/gene/?term=70893
Vim	Vim	vimentin	22352	https://www.ncbi.nlm.nih.gov/gene/?term=22352
Pex5l	Pex5l	peroxisomal biogenesis factor 5-like	58869	https://www.ncbi.nlm.nih.gov/gene/?term=58869
Srcin1	Srcin1	SRC kinase signaling inhibitor 1	56013	https://www.ncbi.nlm.nih.gov/gene/?term=56013
Gon4l	Gon4l	gon-4-like (C.elegans)	76022	https://www.ncbi.nlm.nih.gov/gene/?term=76022
St6galnac6	St6galnac6	ST6 (alpha-N-acetyl-neuraminyl-2,3-beta-galactosyl-1,3)-N-acetylgalactosaminide alpha-2,6-sialyltransferase 6	50935	https://www.ncbi.nlm.nih.gov/gene/?term=50935
Sqstm1	Sqstm1	sequestosome 1	18412	https://www.ncbi.nlm.nih.gov/gene/?term=18412
Stx6	Stx6	syntaxin 6	58244	https://www.ncbi.nlm.nih.gov/gene/?term=58244
Gpr63	Gpr63	G protein-coupled receptor 63	81006	https://www.ncbi.nlm.nih.gov/gene/?term=81006
Atxn1l	Atxn1l	ataxin 1-like	52335	https://www.ncbi.nlm.nih.gov/gene/?term=52335

Zfp827	Zfp827	zinc finger protein 827	622675	https://www.ncbi.nlm.nih.gov/gene/?term=622675
Tmem127	Tmem127	transmembrane protein 127	69470	https://www.ncbi.nlm.nih.gov/gene/?term=69470
Lpgat1	Lpgat1	lysophosphatidylglycerol acyltransferase 1	226856	https://www.ncbi.nlm.nih.gov/gene/?term=226856
Smoc1	Smoc1	SPARC related modular calcium binding 1	64075	https://www.ncbi.nlm.nih.gov/gene/?term=64075
Egln3	Egln3	egl-9 family hypoxia-inducible factor 3	112407	https://www.ncbi.nlm.nih.gov/gene/?term=112407
Scn1a	Scn1a	sodium channel, voltage-gated, type I, alpha	20265	https://www.ncbi.nlm.nih.gov/gene/?term=20265
Rsrp1	Rsrp1	arginine/serine rich protein 1	27981	https://www.ncbi.nlm.nih.gov/gene/?term=27981
Adam9	Adam9	a disintegrin and metallopeptidase domain 9 (meltrin gamma)	11502	https://www.ncbi.nlm.nih.gov/gene/?term=11502
Ldlrap1	Ldlrap1	low density lipoprotein receptor adaptor protein 1	100017	https://www.ncbi.nlm.nih.gov/gene/?term=100017
Pkd2	Pkd2	polycystic kidney disease 2	18764	https://www.ncbi.nlm.nih.gov/gene/?term=18764
Mkrn1	Mkrn1	makorin, ring finger protein, 1	54484	https://www.ncbi.nlm.nih.gov/gene/?term=54484
Pitpna	Pitpna	phosphatidylinositol transfer protein, alpha	18738	https://www.ncbi.nlm.nih.gov/gene/?term=18738
Trip11	Trip11	thyroid hormone receptor interactor 11	109181	https://www.ncbi.nlm.nih.gov/gene/?term=109181
1600012H06Rik	1600012H06Rik	RIKEN cDNA 1600012H06 gene	67912	https://www.ncbi.nlm.nih.gov/gene/?term=67912
Heg1	Heg1	heart development protein with EGF-like domains 1	77446	https://www.ncbi.nlm.nih.gov/gene/?term=77446
Jpt1	Jpt1	Jupiter microtubule associated homolog 1	15374	https://www.ncbi.nlm.nih.gov/gene/?term=15374
Panx2	Panx2	pannexin 2	406218	https://www.ncbi.nlm.nih.gov/gene/?term=406218
Aktip	Aktip	thymoma viral proto-oncogene 1 interacting protein	14339	https://www.ncbi.nlm.nih.gov/gene/?term=14339

Biology Open • Supplementary information

Mtmr3	Mtmr3	myotubularin related protein 3	74302	https://www.ncbi.nlm.nih.gov/gene/?term=74302
Nrip3	Nrip3	nuclear receptor interacting protein 3	78593	https://www.ncbi.nlm.nih.gov/gene/?term=78593
Rgma	Rgma	repulsive guidance molecule family member A	244058	https://www.ncbi.nlm.nih.gov/gene/?term=244058
Zc3h12c	Zc3h12c	zinc finger CCCH type containing 12C	244871	https://www.ncbi.nlm.nih.gov/gene/?term=244871
Lypd6	Lypd6	LY6/PLAUR domain containing 6	320343	https://www.ncbi.nlm.nih.gov/gene/?term=320343
Retreg2	Retreg2	reticulophagy regulator family member 2	227298	https://www.ncbi.nlm.nih.gov/gene/?term=227298
Irf9	Irf9	interferon regulatory factor 9	16391	https://www.ncbi.nlm.nih.gov/gene/?term=16391
Spopl	Spopl	speckle-type POZ protein-like	76857	https://www.ncbi.nlm.nih.gov/gene/?term=76857
Btg3	Btg3	B cell translocation gene 3	12228	https://www.ncbi.nlm.nih.gov/gene/?term=12228
Znfx1	Znfx1	zinc finger, NFX1-type containing 1	98999	https://www.ncbi.nlm.nih.gov/gene/?term=98999
Ptchd4	Ptchd4	patched domain containing 4	627626	https://www.ncbi.nlm.nih.gov/gene/?term=627626
Fyco1	Fyco1	FYVE and coiled-coil domain containing 1	17281	https://www.ncbi.nlm.nih.gov/gene/?term=17281
Afg1l	Afg1l	AFG1 like ATPase	215951	https://www.ncbi.nlm.nih.gov/gene/?term=215951
Iqsec2	Iqsec2	IQ motif and Sec7 domain 2	245666	https://www.ncbi.nlm.nih.gov/gene/?term=245666
Gid4	Gid4	GID complex subunit 4, VID24 homolog	66771	https://www.ncbi.nlm.nih.gov/gene/?term=66771
Pthlh	Pthlh	parathyroid hormone-like peptide	19227	https://www.ncbi.nlm.nih.gov/gene/?term=19227
Mink1	Mink1	misshapen-like kinase 1 (zebrafish)	50932	https://www.ncbi.nlm.nih.gov/gene/?term=50932
Idua	Idua	iduronidase, alpha-L	15932	https://www.ncbi.nlm.nih.gov/gene/?term=15932
Pde3b	Pde3b	phosphodiesterase 3B, cGMP-inhibited	18576	https://www.ncbi.nlm.nih.gov/gene/?term=18576
Enpp5	Enpp5	ectonucleotide pyrophosphatase/phosphodiesterase 5	83965	https://www.ncbi.nlm.nih.gov/gene/?term=83965
Fzd3	Fzd3	frizzled class receptor 3	14365	https://www.ncbi.nlm.nih.gov/gene/?term=14365

Biology Open • Supplementary information

Topors	Topors	topoisomerase I binding, arginine/serine-rich	106021	https://www.ncbi.nlm.nih.gov/gene/?term=106021
Rufy2	Rufy2	RUN and FYVE domain-containing 2	70432	https://www.ncbi.nlm.nih.gov/gene/?term=70432
Reps2	Reps2	RALBP1 associated Eps domain containing protein 2	194590	https://www.ncbi.nlm.nih.gov/gene/?term=194590
Camk2n2	Camk2n2	calcium/calmodulin-dependent protein kinase II inhibitor 2	73047	https://www.ncbi.nlm.nih.gov/gene/?term=73047
Rp2	Rp2	retinitis pigmentosa 2 homolog	19889	https://www.ncbi.nlm.nih.gov/gene/?term=19889
Agfg2	Agfg2	ArfGAP with FG repeats 2	231801	https://www.ncbi.nlm.nih.gov/gene/?term=231801
Irf2bp2	Irf2bp2	interferon regulatory factor 2 binding protein 2	270110	https://www.ncbi.nlm.nih.gov/gene/?term=270110
Kcnk10	Kcnk10	potassium channel, subfamily K, member 10	72258	https://www.ncbi.nlm.nih.gov/gene/?term=72258
Map3k14	Map3k14	mitogen-activated protein kinase kinase kinase 14	53859	https://www.ncbi.nlm.nih.gov/gene/?term=53859
Smoc2	Smoc2	SPARC related modular calcium binding 2	64074	https://www.ncbi.nlm.nih.gov/gene/?term=64074
Kif3b	Kif3b	kinesin family member 3B	16569	https://www.ncbi.nlm.nih.gov/gene/?term=16569
Ulk1	Ulk1	unc-51 like kinase 1	22241	https://www.ncbi.nlm.nih.gov/gene/?term=22241
Kcnb1	Kcnb1	potassium voltage gated channel, Shab-related subfamily, member 1	16500	https://www.ncbi.nlm.nih.gov/gene/?term=16500
F3	F3	coagulation factor III	14066	https://www.ncbi.nlm.nih.gov/gene/?term=14066
Slc31a2	Slc31a2	solute carrier family 31, member 2	20530	https://www.ncbi.nlm.nih.gov/gene/?term=20530
Nagk	Nagk	N-acetylglucosamine kinase	56174	https://www.ncbi.nlm.nih.gov/gene/?term=56174
Usp3	Usp3	ubiquitin specific peptidase 3	235441	https://www.ncbi.nlm.nih.gov/gene/?term=235441
Sertad2	Sertad2	SERTA domain containing 2	58172	https://www.ncbi.nlm.nih.gov/gene/?term=58172
App	App	amyloid beta (A4) precursor protein	11820	https://www.ncbi.nlm.nih.gov/gene/?term=11820
Tbc1d8b	Tbc1d8b	TBC1 domain family, member 8B	245638	https://www.ncbi.nlm.nih.gov/gene/?term=245638
Lrrc55	Lrrc55	leucine rich repeat containing 55	241528	https://www.ncbi.nlm.nih.gov/gene/?term=241528

Biology Open • Supplementary information

Cast	Cast	calpastatin	12380	https://www.ncbi.nlm.nih.gov/gene/?term=12380
Kmt2b	Kmt2b	lysine (K)-specific methyltransferase 2B	75410	https://www.ncbi.nlm.nih.gov/gene/?term=75410
Wdr37	Wdr37	WD repeat domain 37	207615	https://www.ncbi.nlm.nih.gov/gene/?term=207615
Ism2	Ism2	isthmin 2	217738	https://www.ncbi.nlm.nih.gov/gene/?term=217738
Fcho2	Fcho2	FCH domain only 2	218503	https://www.ncbi.nlm.nih.gov/gene/?term=218503
Purb	Purb	purine rich element binding protein B	19291	https://www.ncbi.nlm.nih.gov/gene/?term=19291
Ssh2	Ssh2	slingshot protein phosphatase 2	237860	https://www.ncbi.nlm.nih.gov/gene/?term=237860
Kif5a	Kif5a	kinesin family member 5A	16572	https://www.ncbi.nlm.nih.gov/gene/?term=16572
Chd9	Chd9	chromodomain helicase DNA binding protein 9	109151	https://www.ncbi.nlm.nih.gov/gene/?term=109151
Dsg4	Dsg4	desmoglein 4	16769	https://www.ncbi.nlm.nih.gov/gene/?term=16769
Pxk	Pxk	PX domain containing serine/threonine kinase	218699	https://www.ncbi.nlm.nih.gov/gene/?term=218699
Cbln4	Cbln4	cerebellin 4 precursor protein	228942	https://www.ncbi.nlm.nih.gov/gene/?term=228942
Ankrd9	Ankrd9	ankyrin repeat domain 9	74251	https://www.ncbi.nlm.nih.gov/gene/?term=74251
Lrch1	Lrch1	leucine-rich repeats and calponin homology (CH) domain containing 1	380916	https://www.ncbi.nlm.nih.gov/gene/?term=380916
Ints14	Ints14	integrator complex subunit 14	69882	https://www.ncbi.nlm.nih.gov/gene/?term=69882
Col4a4	Col4a4	collagen, type IV, alpha 4	12829	https://www.ncbi.nlm.nih.gov/gene/?term=12829
Slc18a2	Slc18a2	solute carrier family 18 (vesicular monoamine), member 2	214084	https://www.ncbi.nlm.nih.gov/gene/?term=214084
Tnfrsf21	Tnfrsf21	tumor necrosis factor receptor superfamily, member 21	94185	https://www.ncbi.nlm.nih.gov/gene/?term=94185
Chrm2	Chrm2	cholinergic receptor, muscarinic 2, cardiac	243764	https://www.ncbi.nlm.nih.gov/gene/?term=243764
Midn	Midn	midnolin	59090	https://www.ncbi.nlm.nih.gov/gene/?term=59090
Chmp4c	Chmp4c	charged multivesicular body protein	66371	https://www.ncbi.nlm.nih.gov/gene/?term=66371

Biology Open • Supplementary information

		4C		
Tmem267	Tmem267	transmembrane protein 267	633640	https://www.ncbi.nlm.nih.gov/gene/?term=633640
Bbx	Bbx	bobby sox HMG box containing	70508	https://www.ncbi.nlm.nih.gov/gene/?term=70508
Trip10	Trip10	thyroid hormone receptor interactor 10	106628	https://www.ncbi.nlm.nih.gov/gene/?term=106628
Ankrd13c	Ankrd13c	ankyrin repeat domain 13c	433667	https://www.ncbi.nlm.nih.gov/gene/?term=433667
Slc40a1	Slc40a1	solute carrier family 40 (iron-regulated transporter), member 1	53945	https://www.ncbi.nlm.nih.gov/gene/?term=53945
Tnks1bp1	Tnks1bp1	tankyrase 1 binding protein 1	228140	https://www.ncbi.nlm.nih.gov/gene/?term=228140
Polr3g	Polr3g	polymerase (RNA) III (DNA directed) polypeptide G	67486	https://www.ncbi.nlm.nih.gov/gene/?term=67486
Atl3	Atl3	atlastin GTPase 3	109168	https://www.ncbi.nlm.nih.gov/gene/?term=109168
Rab11fip5	Rab11fip5	RAB11 family interacting protein 5 (class I)	52055	https://www.ncbi.nlm.nih.gov/gene/?term=52055
Tmcc3	Tmcc3	transmembrane and coiled coil domains 3	319880	https://www.ncbi.nlm.nih.gov/gene/?term=319880
Armc8	Armc8	armadillo repeat containing 8	74125	https://www.ncbi.nlm.nih.gov/gene/?term=74125
Atg16l1	Atg16l1	autophagy related 16-like 1 (S. cerevisiae)	77040	https://www.ncbi.nlm.nih.gov/gene/?term=77040
Rasl11b	Rasl11b	RAS-like, family 11, member B	68939	https://www.ncbi.nlm.nih.gov/gene/?term=68939
Prrg1	Prrg1	proline rich Gla (G-carboxyglutamic acid) 1	546336	https://www.ncbi.nlm.nih.gov/gene/?term=546336
Ptpn4	Ptpn4	protein tyrosine phosphatase, non-receptor type 4	19258	https://www.ncbi.nlm.nih.gov/gene/?term=19258
Unc80	Unc80	unc-80, NALCN activator	329178	https://www.ncbi.nlm.nih.gov/gene/?term=329178
Fbxl5	Fbxl5	F-box and leucine-rich repeat protein 5	242960	https://www.ncbi.nlm.nih.gov/gene/?term=242960
Vangl1	Vangl1	VANGL planar cell polarity 1	229658	https://www.ncbi.nlm.nih.gov/gene/?term=229658
Rnf150	Rnf150	ring finger protein 150	330812	https://www.ncbi.nlm.nih.gov/gene/?term=330812

Biology Open • Supplementary information

Biology Open (2024): doi:10.1242/bio.060308: Supplementary information

Sar1b	Sar1b	secretion associated Ras related GTPase 1B	66397	https://www.ncbi.nlm.nih.gov/gene/?term=66397
St3gal1	St3gal1	ST3 beta-galactoside alpha-2,3-sialyltransferase 1	20442	https://www.ncbi.nlm.nih.gov/gene/?term=20442
Ppp1r3e	Ppp1r3e	protein phosphatase 1, regulatory subunit 3E	105651	https://www.ncbi.nlm.nih.gov/gene/?term=105651
Frmd6	Frmd6	FERM domain containing 6	319710	https://www.ncbi.nlm.nih.gov/gene/?term=319710
Ddhd1	Ddhd1	DDHD domain containing 1	114874	https://www.ncbi.nlm.nih.gov/gene/?term=114874
Unk	Unk	unkempt family zinc finger	217331	https://www.ncbi.nlm.nih.gov/gene/?term=217331
Crybg3	Crybg3	beta-gamma crystallin domain containing 3	224273	https://www.ncbi.nlm.nih.gov/gene/?term=224273
Tnks2	Tnks2	tankyrase, TRF1-interacting ankyrin-related ADP-ribose polymerase 2	74493	https://www.ncbi.nlm.nih.gov/gene/?term=74493
Laptm4a	Laptm4a	lysosomal-associated protein transmembrane 4A	17775	https://www.ncbi.nlm.nih.gov/gene/?term=17775
Snx8	Snx8	sorting nexin 8	231834	https://www.ncbi.nlm.nih.gov/gene/?term=231834
Pdcd1lg2	Pdcd1lg2	programmed cell death 1 ligand 2	58205	https://www.ncbi.nlm.nih.gov/gene/?term=58205
Slc25a40	Slc25a40	solute carrier family 25, member 40	319653	https://www.ncbi.nlm.nih.gov/gene/?term=319653
Rab8b	Rab8b	RAB8B, member RAS oncogene family	235442	https://www.ncbi.nlm.nih.gov/gene/?term=235442
Clip4	Clip4	CAP-GLY domain containing linker protein family, member 4	78785	https://www.ncbi.nlm.nih.gov/gene/?term=78785
Rhoc	Rhoc	ras homolog family member C	11853	https://www.ncbi.nlm.nih.gov/gene/?term=11853
Mastl	Mastl	microtubule associated serine/threonine kinase-like	67121	https://www.ncbi.nlm.nih.gov/gene/?term=67121
Txnip	Txnip	thioredoxin interacting protein	56338	https://www.ncbi.nlm.nih.gov/gene/?term=56338
Kif23	Kif23	kinesin family member 23	71819	https://www.ncbi.nlm.nih.gov/gene/?term=71819
Fat2	Fat2	FAT atypical cadherin 2	245827	https://www.ncbi.nlm.nih.gov/gene/?term=245827
Fjx1	Fjx1	four jointed box 1	14221	https://www.ncbi.nlm.nih.gov/gene/?term=14221

Biology Open • Supplementary information

Coro2b	Coro2b	coronin, actin binding protein, 2B	235431	https://www.ncbi.nlm.nih.gov/gene/?term=235431
Gpr137b	Gpr137b	G protein-coupled receptor 137B	83924	https://www.ncbi.nlm.nih.gov/gene/?term=83924
Rbl2	Rbl2	RB transcriptional corepressor like 2	19651	https://www.ncbi.nlm.nih.gov/gene/?term=19651
Xrn1	Xrn1	5'-3' exoribonuclease 1	24127	https://www.ncbi.nlm.nih.gov/gene/?term=24127
Sash1	Sash1	SAM and SH3 domain containing 1	70097	https://www.ncbi.nlm.nih.gov/gene/?term=70097
Arhgef10	Arhgef10	Rho guanine nucleotide exchange factor (GEF) 10	234094	https://www.ncbi.nlm.nih.gov/gene/?term=234094
Slc17a8	Slc17a8	solute carrier family 17 (sodium-dependent inorganic phosphate cotransporter), member 8	216227	https://www.ncbi.nlm.nih.gov/gene/?term=216227
Slc24a2	Slc24a2	solute carrier family 24 (sodium/potassium/calcium exchanger), member 2	76376	https://www.ncbi.nlm.nih.gov/gene/?term=76376
Gosr1	Gosr1	golgi SNAP receptor complex member 1	53334	https://www.ncbi.nlm.nih.gov/gene/?term=53334
Bnip2	Bnip2	BCL2/adenovirus E1B interacting protein 2	12175	https://www.ncbi.nlm.nih.gov/gene/?term=12175
E2f1	E2f1	E2F transcription factor 1	13555	https://www.ncbi.nlm.nih.gov/gene/?term=13555
Cep57	Cep57	centrosomal protein 57	74360	https://www.ncbi.nlm.nih.gov/gene/?term=74360
Ccng2	Ccng2	cyclin G2	12452	https://www.ncbi.nlm.nih.gov/gene/?term=12452
B3galt2	B3galt2	UDP-Gal:betaGlcNAc beta 1,3-galactosyltransferase, polypeptide 2	26878	https://www.ncbi.nlm.nih.gov/gene/?term=26878
Elk3	Elk3	ELK3, member of ETS oncogene family	13713	https://www.ncbi.nlm.nih.gov/gene/?term=13713
Ezh1	Ezh1	enhancer of zeste 1 polycomb repressive complex 2 subunit	14055	https://www.ncbi.nlm.nih.gov/gene/?term=14055
Map3k13	Map3k13	mitogen-activated protein kinase kinase kinase 13	71751	https://www.ncbi.nlm.nih.gov/gene/?term=71751
Sall1	Sall1	spalt like transcription factor 1	58198	https://www.ncbi.nlm.nih.gov/gene/?term=58198

Lama3	Lama3	laminin, alpha 3	16774	https://www.ncbi.nlm.nih.gov/gene/?term=16774
Cmpk1	Cmpk1	cytidine monophosphate (UMP-CMP) kinase 1	66588	https://www.ncbi.nlm.nih.gov/gene/?term=66588
Map3k12	Map3k12	mitogen-activated protein kinase kinase kinase 12	26404	https://www.ncbi.nlm.nih.gov/gene/?term=26404
Ankrd52	Ankrd52	ankyrin repeat domain 52	237615	https://www.ncbi.nlm.nih.gov/gene/?term=237615
Mapre3	Mapre3	microtubule-associated protein, RP/EB family, member 3	100732	https://www.ncbi.nlm.nih.gov/gene/?term=100732
Mex3d	Mex3d	mex3 RNA binding family member D	237400	https://www.ncbi.nlm.nih.gov/gene/?term=237400
Mfap3l	Mfap3l	microfibrillar-associated protein 3-like	71306	https://www.ncbi.nlm.nih.gov/gene/?term=71306
Rab33b	Rab33b	RAB33B, member RAS oncogene family	19338	https://www.ncbi.nlm.nih.gov/gene/?term=19338
Eif4a2	Eif4a2	eukaryotic translation initiation factor 4A2	13682	https://www.ncbi.nlm.nih.gov/gene/?term=13682
Susd6	Susd6	sushi domain containing 6	217684	https://www.ncbi.nlm.nih.gov/gene/?term=217684
Uri1	Uri1	URI1, prefoldin-like chaperone	19777	https://www.ncbi.nlm.nih.gov/gene/?term=19777
Lrp8	Lrp8	low density lipoprotein receptor-related protein 8, apolipoprotein e receptor	16975	https://www.ncbi.nlm.nih.gov/gene/?term=16975
Nedd4l	Nedd4l	neural precursor cell expressed, developmentally down-regulated gene 4-like	83814	https://www.ncbi.nlm.nih.gov/gene/?term=83814
6430548M08Rik	6430548M08Rik	RIKEN cDNA 6430548M08 gene	234797	https://www.ncbi.nlm.nih.gov/gene/?term=234797
Mapk4	Mapk4	mitogen-activated protein kinase 4	225724	https://www.ncbi.nlm.nih.gov/gene/?term=225724
Pfkp	Pfkp	phosphofructokinase, platelet	56421	https://www.ncbi.nlm.nih.gov/gene/?term=56421
Npat	Npat	nuclear protein in the AT region	244879	https://www.ncbi.nlm.nih.gov/gene/?term=244879
Tars2	Tars2	threonyl-tRNA synthetase 2, mitochondrial (putative)	71807	https://www.ncbi.nlm.nih.gov/gene/?term=71807

Biology Open • Supplementary information

Fgd5	Fgd5	FYVE, RhoGEF and PH domain containing 5	232237	https://www.ncbi.nlm.nih.gov/gene/?term=232237
Itgb8	Itgb8	integrin beta 8	320910	https://www.ncbi.nlm.nih.gov/gene/?term=320910
Ptpn3	Ptpn3	protein tyrosine phosphatase, non-receptor type 3	545622	https://www.ncbi.nlm.nih.gov/gene/?term=545622
Tbcel	Tbcel	tubulin folding cofactor E-like	272589	https://www.ncbi.nlm.nih.gov/gene/?term=272589
Fastk	Fastk	Fas-activated serine/threonine kinase	66587	https://www.ncbi.nlm.nih.gov/gene/?term=66587
Cep97	Cep97	centrosomal protein 97	74201	https://www.ncbi.nlm.nih.gov/gene/?term=74201
Itpripl2	Itpripl2	inositol 1,4,5-triphosphate receptor interacting protein-like 2	319622	https://www.ncbi.nlm.nih.gov/gene/?term=319622
Fam219b	Fam219b	family with sequence similarity 219, member B	78323	https://www.ncbi.nlm.nih.gov/gene/?term=78323
Zfp661	Zfp661	zinc finger protein 661	72180	https://www.ncbi.nlm.nih.gov/gene/?term=72180
Il25	Il25	interleukin 25	140806	https://www.ncbi.nlm.nih.gov/gene/?term=140806
Hlf	Hlf	hepatic leukemia factor	217082	https://www.ncbi.nlm.nih.gov/gene/?term=217082
Ccdc71l	Ccdc71l	coiled-coil domain containing 71 like	72123	https://www.ncbi.nlm.nih.gov/gene/?term=72123
Rab5b	Rab5b	RAB5B, member RAS oncogene family	19344	https://www.ncbi.nlm.nih.gov/gene/?term=19344
Rb1cc1	Rb1cc1	RB1-inducible coiled-coil 1	12421	https://www.ncbi.nlm.nih.gov/gene/?term=12421
Gpr137c	Gpr137c	G protein-coupled receptor 137C	70713	https://www.ncbi.nlm.nih.gov/gene/?term=70713
Usp24	Usp24	ubiquitin specific peptidase 24	329908	https://www.ncbi.nlm.nih.gov/gene/?term=329908
Skor1	Skor1	SKI family transcriptional corepressor 1	207667	https://www.ncbi.nlm.nih.gov/gene/?term=207667
Arhgef11	Arhgef11	Rho guanine nucleotide exchange factor (GEF) 11	213498	https://www.ncbi.nlm.nih.gov/gene/?term=213498
Zfp91	Zfp91	zinc finger protein 91	109910	https://www.ncbi.nlm.nih.gov/gene/?term=109910
Hs3st5	Hs3st5	heparan sulfate (glucosamine) 3-O-sulfotransferase 5	319415	https://www.ncbi.nlm.nih.gov/gene/?term=319415

Biology Open • Supplementary information

Zdhhc1	Zdhhc1	zinc finger, DHHC domain containing 1	70796	https://www.ncbi.nlm.nih.gov/gene/?term=70796
Npas2	Npas2	neuronal PAS domain protein 2	18143	https://www.ncbi.nlm.nih.gov/gene/?term=18143
Rgmb	Rgmb	repulsive guidance molecule family member B	68799	https://www.ncbi.nlm.nih.gov/gene/?term=68799
Trim3	Trim3	tripartite motif-containing 3	55992	https://www.ncbi.nlm.nih.gov/gene/?term=55992
Plekha3	Plekha3	pleckstrin homology domain-containing, family A (phosphoinositide binding specific) member 3	83435	https://www.ncbi.nlm.nih.gov/gene/?term=83435
Pgbd5	Pgbd5	piggyBac transposable element derived 5	209966	https://www.ncbi.nlm.nih.gov/gene/?term=209966
Cc2d1a	Cc2d1a	coiled-coil and C2 domain containing 1A	212139	https://www.ncbi.nlm.nih.gov/gene/?term=212139
Dnal1	Dnal1	dynein, axonemal, light chain 1	105000	https://www.ncbi.nlm.nih.gov/gene/?term=105000
Btbd10	Btbd10	BTB (POZ) domain containing 10	68815	https://www.ncbi.nlm.nih.gov/gene/?term=68815
Ddhd2	Ddhd2	DDHD domain containing 2	72108	https://www.ncbi.nlm.nih.gov/gene/?term=72108
Stk4	Stk4	serine/threonine kinase 4	58231	https://www.ncbi.nlm.nih.gov/gene/?term=58231
Rad23b	Rad23b	RAD23 homolog B, nucleotide excision repair protein	19359	https://www.ncbi.nlm.nih.gov/gene/?term=19359
Rfx3	Rfx3	regulatory factor X, 3 (influences HLA class II expression)	19726	https://www.ncbi.nlm.nih.gov/gene/?term=19726
Ddr1	Ddr1	discoidin domain receptor family, member 1	12305	https://www.ncbi.nlm.nih.gov/gene/?term=12305
Bcam	Bcam	basal cell adhesion molecule	57278	https://www.ncbi.nlm.nih.gov/gene/?term=57278
Large1	Large1	LARGE xylosyl- and glucuronyltransferase 1	16795	https://www.ncbi.nlm.nih.gov/gene/?term=16795
Gpd2	Gpd2	glycerol phosphate dehydrogenase 2, mitochondrial	14571	https://www.ncbi.nlm.nih.gov/gene/?term=14571

Biology Open • Supplementary information

Tspan6	Tspan6	tetraspanin 6	56496	https://www.ncbi.nlm.nih.gov/gene/?term=56496
Tef	Tef	thyrotroph embryonic factor	21685	https://www.ncbi.nlm.nih.gov/gene/?term=21685
Mical3	Mical3	microtubule associated monooxygenase, calponin and LIM domain containing 3	194401	https://www.ncbi.nlm.nih.gov/gene/?term=194401
Myrf	Myrf	myelin regulatory factor	225908	https://www.ncbi.nlm.nih.gov/gene/?term=225908
Hspa5	Hspa5	heat shock protein 5	14828	https://www.ncbi.nlm.nih.gov/gene/?term=14828
Rassf3	Rassf3	Ras association (RalGDS/AF-6) domain family member 3	192678	https://www.ncbi.nlm.nih.gov/gene/?term=192678
Nfil3	Nfil3	nuclear factor, interleukin 3, regulated	18030	https://www.ncbi.nlm.nih.gov/gene/?term=18030
Fzd6	Fzd6	frizzled class receptor 6	14368	https://www.ncbi.nlm.nih.gov/gene/?term=14368
Atp1b3	Atp1b3	ATPase, Na+/K+ transporting, beta 3 polypeptide	11933	https://www.ncbi.nlm.nih.gov/gene/?term=11933
Hspa12a	Hspa12a	heat shock protein 12A	73442	https://www.ncbi.nlm.nih.gov/gene/?term=73442
Sun1	Sun1	Sad1 and UNC84 domain containing 1	77053	https://www.ncbi.nlm.nih.gov/gene/?term=77053
Hmcn1	Hmcn1	hemicentin 1	545370	https://www.ncbi.nlm.nih.gov/gene/?term=545370
Kpna4	Kpna4	karyopherin (importin) alpha 4	16649	https://www.ncbi.nlm.nih.gov/gene/?term=16649
Map3k11	Map3k11	mitogen-activated protein kinase kinase kinase 11	26403	https://www.ncbi.nlm.nih.gov/gene/?term=26403
Aftph	Aftph	aftiphilin	216549	https://www.ncbi.nlm.nih.gov/gene/?term=216549
Nudt10	Nudt10	nudix (nucleoside diphosphate linked moiety X)-type motif 10	102954	https://www.ncbi.nlm.nih.gov/gene/?term=102954
Ece1	Ece1	endothelin converting enzyme 1	230857	https://www.ncbi.nlm.nih.gov/gene/?term=230857
Plxna2	Plxna2	plexin A2	18845	https://www.ncbi.nlm.nih.gov/gene/?term=18845
Cacna1b	Cacna1b	calcium channel, voltage-dependent, N type, alpha 1B subunit	12287	https://www.ncbi.nlm.nih.gov/gene/?term=12287
Zfp579	Zfp579	zinc finger protein 579	68490	https://www.ncbi.nlm.nih.gov/gene/?term=68490

Biology Open • Supplementary information

Dnaaf5	Dnaaf5	dynein, axonemal assembly factor 5	433956	https://www.ncbi.nlm.nih.gov/gene/?term=433956
Taf9b	Taf9b	TATA-box binding protein associated factor 9B	407786	https://www.ncbi.nlm.nih.gov/gene/?term=407786
M6pr	M6pr	mannose-6-phosphate receptor, cation dependent	17113	https://www.ncbi.nlm.nih.gov/gene/?term=17113
Myef2	Myef2	myelin basic protein expression factor 2, repressor	17876	https://www.ncbi.nlm.nih.gov/gene/?term=17876
Ralgapa1	Ralgapa1	Ral GTPase activating protein, alpha subunit 1	56784	https://www.ncbi.nlm.nih.gov/gene/?term=56784
Naa40	Naa40	N(alpha)-acetyltransferase 40, NatD catalytic subunit	70999	https://www.ncbi.nlm.nih.gov/gene/?term=70999
Ttc7b	Ttc7b	tetratricopeptide repeat domain 7B	104718	https://www.ncbi.nlm.nih.gov/gene/?term=104718
Map7d2	Map7d2	MAP7 domain containing 2	78283	https://www.ncbi.nlm.nih.gov/gene/?term=78283
Srsf2	Srsf2	serine/arginine-rich splicing factor 2	20382	https://www.ncbi.nlm.nih.gov/gene/?term=20382
Qrfpr	Qrfpr	pyroglutamylated RFamide peptide receptor	229214	https://www.ncbi.nlm.nih.gov/gene/?term=229214
Haus2	Haus2	HAUS augmin-like complex, subunit 2	66296	https://www.ncbi.nlm.nih.gov/gene/?term=66296
Smc3	Smc3	structural maintenance of chromosomes 3	13006	https://www.ncbi.nlm.nih.gov/gene/?term=13006
Psen2	Psen2	presenilin 2	19165	https://www.ncbi.nlm.nih.gov/gene/?term=19165
Vdac1	Vdac1	voltage-dependent anion channel 1	22333	https://www.ncbi.nlm.nih.gov/gene/?term=22333
Tmpo	Tmpo	thymopoietin	21917	https://www.ncbi.nlm.nih.gov/gene/?term=21917
Mrps27	Mrps27	mitochondrial ribosomal protein S27	218506	https://www.ncbi.nlm.nih.gov/gene/?term=218506
Dgcr2	Dgcr2	DiGeorge syndrome critical region gene 2	13356	https://www.ncbi.nlm.nih.gov/gene/?term=13356
Pdcd6	Pdcd6	programmed cell death 6	18570	https://www.ncbi.nlm.nih.gov/gene/?term=18570
Spink13	Spink13	serine peptidase inhibitor, Kazal type 13	100038417	https://www.ncbi.nlm.nih.gov/gene/?term=100038[illegible]

Osbpl8	Osbpl8	oxysterol binding protein-like 8	237542	https://www.ncbi.nlm.nih.gov/gene/?term=237542
Ctdspl	Ctdspl	CTD (carboxy-terminal domain, RNA polymerase II, polypeptide A) small phosphatase-like	69274	https://www.ncbi.nlm.nih.gov/gene/?term=69274
Idh2	Idh2	isocitrate dehydrogenase 2 (NADP+), mitochondrial	269951	https://www.ncbi.nlm.nih.gov/gene/?term=269951
Pfn2	Pfn2	profilin 2	18645	https://www.ncbi.nlm.nih.gov/gene/?term=18645
Lck	Lck	lymphocyte protein tyrosine kinase	16818	https://www.ncbi.nlm.nih.gov/gene/?term=16818
Celf2	Celf2	CUGBP, Elav-like family member 2	14007	https://www.ncbi.nlm.nih.gov/gene/?term=14007
Ppp2r5c	Ppp2r5c	protein phosphatase 2, regulatory subunit B', gamma	26931	https://www.ncbi.nlm.nih.gov/gene/?term=26931
Ajap1	Ajap1	adherens junction associated protein 1	230959	https://www.ncbi.nlm.nih.gov/gene/?term=230959
Pex19	Pex19	peroxisomal biogenesis factor 19	19298	https://www.ncbi.nlm.nih.gov/gene/?term=19298
Tpm1	Tpm1	tropomyosin 1, alpha	22003	https://www.ncbi.nlm.nih.gov/gene/?term=22003
Slain1	Slain1	SLAIN motif family, member 1	105439	https://www.ncbi.nlm.nih.gov/gene/?term=105439
Csmd1	Csmd1	CUB and Sushi multiple domains 1	94109	https://www.ncbi.nlm.nih.gov/gene/?term=94109
Enah	Enah	ENAH actin regulator	13800	https://www.ncbi.nlm.nih.gov/gene/?term=13800
Ppp2ca	Ppp2ca	protein phosphatase 2 (formerly 2A), catalytic subunit, alpha isoform	19052	https://www.ncbi.nlm.nih.gov/gene/?term=19052
Mfsd6	Mfsd6	major facilitator superfamily domain containing 6	98682	https://www.ncbi.nlm.nih.gov/gene/?term=98682
Gm15319	Gm15319	predicted gene 15319	100040599	https://www.ncbi.nlm.nih.gov/gene/?term=100040
Kcng4	Kcng4	potassium voltage-gated channel, subfamily G, member 4	66733	https://www.ncbi.nlm.nih.gov/gene/?term=66733
Gjd2	Gjd2	gap junction protein, delta 2	14617	https://www.ncbi.nlm.nih.gov/gene/?term=14617
Srek1ip1	Srek1ip1	splicing regulatory glutamine/lysine-rich protein 1interacting protein 1	67288	https://www.ncbi.nlm.nih.gov/gene/?term=67288
Pdcd4	Pdcd4	programmed cell death 4	18569	https://www.ncbi.nlm.nih.gov/gene/?term=18569

Biology Open • Supplementary information

Gstt3	Gstt3	glutathione S-transferase, theta 3	103140	https://www.ncbi.nlm.nih.gov/gene/?term=103140
Rcn2	Rcn2	reticulocalbin 2	26611	https://www.ncbi.nlm.nih.gov/gene/?term=26611
Mphosph8	Mphosph8	M-phase phosphoprotein 8	75339	https://www.ncbi.nlm.nih.gov/gene/?term=75339
4930467E23Rik	4930467E23Rik	RIKEN cDNA 4930467E23 gene	626415	https://www.ncbi.nlm.nih.gov/gene/?term=626415
Ikzf5	Ikzf5	IKAROS family zinc finger 5	67143	https://www.ncbi.nlm.nih.gov/gene/?term=67143
Gripap1	Gripap1	GRIP1 associated protein 1	54645	https://www.ncbi.nlm.nih.gov/gene/?term=54645
Rreb1	Rreb1	ras responsive element binding protein 1	68750	https://www.ncbi.nlm.nih.gov/gene/?term=68750
Eml2	Eml2	echinoderm microtubule associated protein like 2	72205	https://www.ncbi.nlm.nih.gov/gene/?term=72205
Prkar1a	Prkar1a	protein kinase, cAMP dependent regulatory, type I, alpha	19084	https://www.ncbi.nlm.nih.gov/gene/?term=19084
Hif3a	Hif3a	hypoxia inducible factor 3, alpha subunit	53417	https://www.ncbi.nlm.nih.gov/gene/?term=53417
Zfp235	Zfp235	zinc finger protein 235	56525	https://www.ncbi.nlm.nih.gov/gene/?term=56525
Cbll1	Cbll1	Casitas B-lineage lymphoma-like 1	104836	https://www.ncbi.nlm.nih.gov/gene/?term=104836
Zfp788	Zfp788	zinc finger protein 788	67607	https://www.ncbi.nlm.nih.gov/gene/?term=67607
Zadh2	Zadh2	zinc binding alcohol dehydrogenase, domain containing 2	225791	https://www.ncbi.nlm.nih.gov/gene/?term=225791
Myb	Myb	myeloblastosis oncogene	17863	https://www.ncbi.nlm.nih.gov/gene/?term=17863
Tardbp	Tardbp	TAR DNA binding protein	230908	https://www.ncbi.nlm.nih.gov/gene/?term=230908
Frrs1l	Frrs1l	ferric-chelate reductase 1 like	230235	https://www.ncbi.nlm.nih.gov/gene/?term=230235
Fbxo21	Fbxo21	F-box protein 21	231670	https://www.ncbi.nlm.nih.gov/gene/?term=231670
Pom121	Pom121	nuclear pore membrane protein 121	107939	https://www.ncbi.nlm.nih.gov/gene/?term=107939
Myh1	Myh1	myosin, heavy polypeptide 1, skeletal muscle, adult	17879	https://www.ncbi.nlm.nih.gov/gene/?term=17879
Tfip11	Tfip11	tuftelin interacting protein 11	54723	https://www.ncbi.nlm.nih.gov/gene/?term=54723
Pik3r1	Pik3r1	phosphoinositide-3-kinase regulatory subunit 1	18708	https://www.ncbi.nlm.nih.gov/gene/?term=18708

Biology Open • Supplementary information

Ephb2	Ephb2	Eph receptor B2	13844	https://www.ncbi.nlm.nih.gov/gene/?term=13844
Fbxw11	Fbxw11	F-box and WD-40 domain protein 11	103583	https://www.ncbi.nlm.nih.gov/gene/?term=103583
Pign	Pign	phosphatidylinositol glycan anchor biosynthesis, class N	27392	https://www.ncbi.nlm.nih.gov/gene/?term=27392
Cip2a	Cip2a	cell proliferation regulating inhibitor of protein phosphatase 2A	224171	https://www.ncbi.nlm.nih.gov/gene/?term=224171
Them4	Them4	thioesterase superfamily member 4	75778	https://www.ncbi.nlm.nih.gov/gene/?term=75778
Adipor2	Adipor2	adiponectin receptor 2	68465	https://www.ncbi.nlm.nih.gov/gene/?term=68465
Ptx3	Ptx3	pentraxin related gene	19288	https://www.ncbi.nlm.nih.gov/gene/?term=19288
Map3k3	Map3k3	mitogen-activated protein kinase kinase kinase 3	26406	https://www.ncbi.nlm.nih.gov/gene/?term=26406
Dyrk1b	Dyrk1b	dual-specificity tyrosine-(Y)-phosphorylation regulated kinase 1b	13549	https://www.ncbi.nlm.nih.gov/gene/?term=13549
Pafah1b1	Pafah1b1	platelet-activating factor acetylhydrolase, isoform 1b, subunit 1	18472	https://www.ncbi.nlm.nih.gov/gene/?term=18472
Zfp42	Zfp42	zinc finger protein 42	22702	https://www.ncbi.nlm.nih.gov/gene/?term=22702
Pdgfrb	Pdgfrb	platelet derived growth factor receptor, beta polypeptide	18596	https://www.ncbi.nlm.nih.gov/gene/?term=18596
Prkaa2	Prkaa2	protein kinase, AMP-activated, alpha 2 catalytic subunit	108079	https://www.ncbi.nlm.nih.gov/gene/?term=108079
Pik3r3	Pik3r3	phosphoinositide-3-kinase regulatory subunit 3	18710	https://www.ncbi.nlm.nih.gov/gene/?term=18710
Tmem179b	Tmem179b	transmembrane protein 179B	67706	https://www.ncbi.nlm.nih.gov/gene/?term=67706
Car12	Car12	carbonic anhydrase 12	76459	https://www.ncbi.nlm.nih.gov/gene/?term=76459
Adam12	Adam12	a disintegrin and metallopeptidase domain 12 (meltrin alpha)	11489	https://www.ncbi.nlm.nih.gov/gene/?term=11489
Zfp930	Zfp930	zinc finger protein 930	234358	https://www.ncbi.nlm.nih.gov/gene/?term=234358

Biology Open • Supplementary information

Scn3a	Scn3a	sodium channel, voltage-gated, type III, alpha	20269	https://www.ncbi.nlm.nih.gov/gene/?term=20269
Gpr155	Gpr155	G protein-coupled receptor 155	68526	https://www.ncbi.nlm.nih.gov/gene/?term=68526
Zfp217	Zfp217	zinc finger protein 217	228913	https://www.ncbi.nlm.nih.gov/gene/?term=228913
Magohb	Magohb	mago homolog B, exon junction complex core component	66441	https://www.ncbi.nlm.nih.gov/gene/?term=66441
Copb1	Copb1	coatomer protein complex, subunit beta 1	70349	https://www.ncbi.nlm.nih.gov/gene/?term=70349
Pnn	Pnn	pinin	18949	https://www.ncbi.nlm.nih.gov/gene/?term=18949
Fth1	Fth1	ferritin heavy polypeptide 1	14319	https://www.ncbi.nlm.nih.gov/gene/?term=14319
Arhgef2	Arhgef2	rho/rac guanine nucleotide exchange factor (GEF) 2	16800	https://www.ncbi.nlm.nih.gov/gene/?term=16800
Tmem150a	Tmem150a	transmembrane protein 150A	232086	https://www.ncbi.nlm.nih.gov/gene/?term=232086
Zmym2	Zmym2	zinc finger, MYM-type 2	76007	https://www.ncbi.nlm.nih.gov/gene/?term=76007
Mboat1	Mboat1	membrane bound O-acyltransferase domain containing 1	218121	https://www.ncbi.nlm.nih.gov/gene/?term=218121
Cdh2	Cdh2	cadherin 2	12558	https://www.ncbi.nlm.nih.gov/gene/?term=12558
Phospho2	Phospho2	phosphatase, orphan 2	73373	https://www.ncbi.nlm.nih.gov/gene/?term=73373
Ptp4a1	Ptp4a1	protein tyrosine phosphatase 4a1	19243	https://www.ncbi.nlm.nih.gov/gene/?term=19243
Cramp1l	Cramp1l	cramped chromatin regulator homolog 1	57354	https://www.ncbi.nlm.nih.gov/gene/?term=57354
Shroom4	Shroom4	shroom family member 4	208431	https://www.ncbi.nlm.nih.gov/gene/?term=208431
Csgalnact1	Csgalnact1	chondroitin sulfate N-acetylgalactosaminyltransferase 1	234356	https://www.ncbi.nlm.nih.gov/gene/?term=234356
Rbms3	Rbms3	RNA binding motif, single stranded interacting protein	207181	https://www.ncbi.nlm.nih.gov/gene/?term=207181
Cpeb4	Cpeb4	cytoplasmic polyadenylation element binding protein 4	67579	https://www.ncbi.nlm.nih.gov/gene/?term=67579
Map2k7	Map2k7	mitogen-activated protein kinase	26400	https://www.ncbi.nlm.nih.gov/gene/?term=26400

Biology Open • Supplementary information

		kinase 7		
Cnot1	Cnot1	CCR4-NOT transcription complex, subunit 1	234594	https://www.ncbi.nlm.nih.gov/gene/?term=234594
Foxp4	Foxp4	forkhead box P4	74123	https://www.ncbi.nlm.nih.gov/gene/?term=74123
Ccng1	Ccng1	cyclin G1	12450	https://www.ncbi.nlm.nih.gov/gene/?term=12450
Thoc5	Thoc5	THO complex 5	107829	https://www.ncbi.nlm.nih.gov/gene/?term=107829
Snx7	Snx7	sorting nexin 7	76561	https://www.ncbi.nlm.nih.gov/gene/?term=76561
Trpm7	Trpm7	transient receptor potential cation channel, subfamily M, member 7	58800	https://www.ncbi.nlm.nih.gov/gene/?term=58800
Calb2	Calb2	calbindin 2	12308	https://www.ncbi.nlm.nih.gov/gene/?term=12308
Olfr77	Olfr77	olfactory receptor 77	258336	https://www.ncbi.nlm.nih.gov/gene/?term=258336
Vav3	Vav3	vav 3 oncogene	57257	https://www.ncbi.nlm.nih.gov/gene/?term=57257
Syt4	Syt4	synaptotagmin IV	20983	https://www.ncbi.nlm.nih.gov/gene/?term=20983
Prkg2	Prkg2	protein kinase, cGMP-dependent, type II	19092	https://www.ncbi.nlm.nih.gov/gene/?term=19092
Tnfaip3	Tnfaip3	tumor necrosis factor, alpha-induced protein 3	21929	https://www.ncbi.nlm.nih.gov/gene/?term=21929
Enpep	Enpep	glutamyl aminopeptidase	13809	https://www.ncbi.nlm.nih.gov/gene/?term=13809
Tesk2	Tesk2	testis-specific kinase 2	230661	https://www.ncbi.nlm.nih.gov/gene/?term=230661
Cyhr1	Cyhr1	cysteine and histidine rich 1	54151	https://www.ncbi.nlm.nih.gov/gene/?term=54151
Nox4	Nox4	NADPH oxidase 4	50490	https://www.ncbi.nlm.nih.gov/gene/?term=50490
Smarcd2	Smarcd2	SWI/SNF related, matrix associated, actin dependent regulator of chromatin, subfamily d, member 2	83796	https://www.ncbi.nlm.nih.gov/gene/?term=83796
Peak1	Peak1	pseudopodium-enriched atypical kinase 1	244895	https://www.ncbi.nlm.nih.gov/gene/?term=244895
Sema3a	Sema3a	sema domain, immunoglobulin domain (Ig), short basic domain, secreted, (semaphorin) 3A	20346	https://www.ncbi.nlm.nih.gov/gene/?term=20346

Biology Open • Supplementary information

Tnc	Tnc	tenascin C	21923	https://www.ncbi.nlm.nih.gov/gene/?term=21923
Slc8a1	Slc8a1	solute carrier family 8 (sodium/calcium exchanger), member 1	20541	https://www.ncbi.nlm.nih.gov/gene/?term=20541
Onecut3	Onecut3	one cut domain, family member 3	246086	https://www.ncbi.nlm.nih.gov/gene/?term=246086
Ptchd1	Ptchd1	patched domain containing 1	211612	https://www.ncbi.nlm.nih.gov/gene/?term=211612
Greb1	Greb1	gene regulated by estrogen in breast cancer protein	268527	https://www.ncbi.nlm.nih.gov/gene/?term=268527
Slc2a2	Slc2a2	solute carrier family 2 (facilitated glucose transporter), member 2	20526	https://www.ncbi.nlm.nih.gov/gene/?term=20526
Hunk	Hunk	hormonally upregulated Neu-associated kinase	26559	https://www.ncbi.nlm.nih.gov/gene/?term=26559
Ppip5k1	Ppip5k1	diphosphoinositol pentakisphosphate kinase 1	327655	https://www.ncbi.nlm.nih.gov/gene/?term=327655
Sgsm1	Sgsm1	small G protein signaling modulator 1	52850	https://www.ncbi.nlm.nih.gov/gene/?term=52850
Ipo4	Ipo4	importin 4	75751	https://www.ncbi.nlm.nih.gov/gene/?term=75751
Srsf6	Srsf6	serine/arginine-rich splicing factor 6	67996	https://www.ncbi.nlm.nih.gov/gene/?term=67996
Arfgef2	Arfgef2	ADP-ribosylation factor guanine nucleotide-exchange factor 2 (brefeldin A-inhibited)	99371	https://www.ncbi.nlm.nih.gov/gene/?term=99371
Slc39a14	Slc39a14	solute carrier family 39 (zinc transporter), member 14	213053	https://www.ncbi.nlm.nih.gov/gene/?term=213053
Reep4	Reep4	receptor accessory protein 4	72549	https://www.ncbi.nlm.nih.gov/gene/?term=72549
Col27a1	Col27a1	collagen, type XXVII, alpha 1	373864	https://www.ncbi.nlm.nih.gov/gene/?term=373864
Tns1	Tns1	tensin 1	21961	https://www.ncbi.nlm.nih.gov/gene/?term=21961
Ift52	Ift52	intraflagellar transport 52	245866	https://www.ncbi.nlm.nih.gov/gene/?term=245866
Mtmr2	Mtmr2	myotubularin related protein 2	77116	https://www.ncbi.nlm.nih.gov/gene/?term=77116
Vdac3	Vdac3	voltage-dependent anion channel 3	22335	https://www.ncbi.nlm.nih.gov/gene/?term=22335

Asxl3	Asxl3	additional sex combs like 3, transcriptional regulator	211961	https://www.ncbi.nlm.nih.gov/gene/?term=211961
Itm2b	Itm2b	integral membrane protein 2B	16432	https://www.ncbi.nlm.nih.gov/gene/?term=16432
Prdm6	Prdm6	PR domain containing 6	225518	https://www.ncbi.nlm.nih.gov/gene/?term=225518
Rfesd	Rfesd	Rieske (Fe-S) domain containing	218341	https://www.ncbi.nlm.nih.gov/gene/?term=218341
Gm21319	Gm21319	predicted gene, 21319	100861908	https://www.ncbi.nlm.nih.gov/gene/?term=100861[illegible]
Fam199x	Fam199x	family with sequence similarity 199, X-linked	245622	https://www.ncbi.nlm.nih.gov/gene/?term=245622
Ybx3	Ybx3	Y box protein 3	56449	https://www.ncbi.nlm.nih.gov/gene/?term=56449
Man1a2	Man1a2	mannosidase, alpha, class 1A, member 2	17156	https://www.ncbi.nlm.nih.gov/gene/?term=17156
Atf2	Atf2	activating transcription factor 2	11909	https://www.ncbi.nlm.nih.gov/gene/?term=11909
Ubfd1	Ubfd1	ubiquitin family domain containing 1	28018	https://www.ncbi.nlm.nih.gov/gene/?term=28018
Nfatc3	Nfatc3	nuclear factor of activated T cells, cytoplasmic, calcineurin dependent 3	18021	https://www.ncbi.nlm.nih.gov/gene/?term=18021
Nell1	Nell1	NEL-like 1	338352	https://www.ncbi.nlm.nih.gov/gene/?term=338352
Mical2	Mical2	microtubule associated monooxygenase, calponin and LIM domain containing 2	320878	https://www.ncbi.nlm.nih.gov/gene/?term=320878
Snx25	Snx25	sorting nexin 25	102141	https://www.ncbi.nlm.nih.gov/gene/?term=102141
Spam1	Spam1	sperm adhesion molecule 1	20690	https://www.ncbi.nlm.nih.gov/gene/?term=20690
Mthfd2	Mthfd2	methylenetetrahydrofolate dehydrogenase (NAD+ dependent), methenyltetrahydrofolate cyclohydrolase	17768	https://www.ncbi.nlm.nih.gov/gene/?term=17768
Lmna	Lmna	lamin A	16905	https://www.ncbi.nlm.nih.gov/gene/?term=16905
Phldb2	Phldb2	pleckstrin homology like domain, family B, member 2	208177	https://www.ncbi.nlm.nih.gov/gene/?term=208177

Biology Open • Supplementary information

B3galnt2	B3galnt2	UDP-GalNAc:betaGlcNAc beta 1,3-galactosaminyltransferase, polypeptide 2	97884	https://www.ncbi.nlm.nih.gov/gene/?term=97884
Pygo2	Pygo2	pygopus 2	68911	https://www.ncbi.nlm.nih.gov/gene/?term=68911
Myh9	Myh9	myosin, heavy polypeptide 9, non-muscle	17886	https://www.ncbi.nlm.nih.gov/gene/?term=17886
Jpt2	Jpt2	Jupiter microtubule associated homolog 2	52009	https://www.ncbi.nlm.nih.gov/gene/?term=52009
Cntn3	Cntn3	contactin 3	18488	https://www.ncbi.nlm.nih.gov/gene/?term=18488
Zswim9	Zswim9	zinc finger SWIM-type containing 9	321008	https://www.ncbi.nlm.nih.gov/gene/?term=321008
Sfxn2	Sfxn2	sideroflexin 2	94279	https://www.ncbi.nlm.nih.gov/gene/?term=94279
Ano1	Ano1	anoctamin 1, calcium activated chloride channel	101772	https://www.ncbi.nlm.nih.gov/gene/?term=101772
D16Ertd472e	D16Ertd472e	DNA segment, Chr 16, ERATO Doi 472, expressed	67102	https://www.ncbi.nlm.nih.gov/gene/?term=67102
Dse	Dse	dermatan sulfate epimerase	212898	https://www.ncbi.nlm.nih.gov/gene/?term=212898
Zfp354a	Zfp354a	zinc finger protein 354A	21408	https://www.ncbi.nlm.nih.gov/gene/?term=21408
Pi4k2a	Pi4k2a	phosphatidylinositol 4-kinase type 2 alpha	84095	https://www.ncbi.nlm.nih.gov/gene/?term=84095
Ak4	Ak4	adenylate kinase 4	11639	https://www.ncbi.nlm.nih.gov/gene/?term=11639
Elavl1	Elavl1	ELAV (embryonic lethal, abnormal vision)-like 1 (Hu antigen R)	15568	https://www.ncbi.nlm.nih.gov/gene/?term=15568
Traf3	Traf3	TNF receptor-associated factor 3	22031	https://www.ncbi.nlm.nih.gov/gene/?term=22031
Sptlc2	Sptlc2	serine palmitoyltransferase, long chain base subunit 2	20773	https://www.ncbi.nlm.nih.gov/gene/?term=20773
Colec12	Colec12	collectin sub-family member 12	140792	https://www.ncbi.nlm.nih.gov/gene/?term=140792
Pter	Pter	phosphotriesterase related	19212	https://www.ncbi.nlm.nih.gov/gene/?term=19212
Tbx18	Tbx18	T-box18	76365	https://www.ncbi.nlm.nih.gov/gene/?term=76365
Slc44a5	Slc44a5	solute carrier family 44, member 5	242259	https://www.ncbi.nlm.nih.gov/gene/?term=242259

Ube3c	Ube3c	ubiquitin protein ligase E3C	100763	https://www.ncbi.nlm.nih.gov/gene/?term=100763
Mga	Mga	MAX gene associated	29808	https://www.ncbi.nlm.nih.gov/gene/?term=29808
Dgkk	Dgkk	diacylglycerol kinase kappa	331374	https://www.ncbi.nlm.nih.gov/gene/?term=331374
Ccdc43	Ccdc43	coiled-coil domain containing 43	52715	https://www.ncbi.nlm.nih.gov/gene/?term=52715
Phf13	Phf13	PHD finger protein 13	230936	https://www.ncbi.nlm.nih.gov/gene/?term=230936
Myocd	Myocd	myocardin	214384	https://www.ncbi.nlm.nih.gov/gene/?term=214384
Pdcd6ip	Pdcd6ip	programmed cell death 6 interacting protein	18571	https://www.ncbi.nlm.nih.gov/gene/?term=18571
Slc50a1	Slc50a1	solute carrier family 50 (sugar transporter), member 1	19729	https://www.ncbi.nlm.nih.gov/gene/?term=19729
Stt3b	Stt3b	STT3, subunit of the oligosaccharyltransferase complex, homolog B (S. cerevisiae)	68292	https://www.ncbi.nlm.nih.gov/gene/?term=68292
Fyttd1	Fyttd1	forty-two-three domain containing 1	69823	https://www.ncbi.nlm.nih.gov/gene/?term=69823
Slc19a2	Slc19a2	solute carrier family 19 (thiamine transporter), member 2	116914	https://www.ncbi.nlm.nih.gov/gene/?term=116914
Wee2	Wee2	WEE1 homolog 2 (S. pombe)	381759	https://www.ncbi.nlm.nih.gov/gene/?term=381759
P4ha2	P4ha2	procollagen-proline, 2-oxoglutarate 4-dioxygenase (proline 4-hydroxylase), alpha II polypeptide	18452	https://www.ncbi.nlm.nih.gov/gene/?term=18452
Ranbp17	Ranbp17	RAN binding protein 17	66011	https://www.ncbi.nlm.nih.gov/gene/?term=66011
Kcnj14	Kcnj14	potassium inwardly-rectifying channel, subfamily J, member 14	211480	https://www.ncbi.nlm.nih.gov/gene/?term=211480
Emb	Emb	embigin	13723	https://www.ncbi.nlm.nih.gov/gene/?term=13723
Asxl1	Asxl1	additional sex combs like 1	228790	https://www.ncbi.nlm.nih.gov/gene/?term=228790
Ap3b1	Ap3b1	adaptor-related protein complex 3, beta 1 subunit	11774	https://www.ncbi.nlm.nih.gov/gene/?term=11774
Zc3h10	Zc3h10	zinc finger CCCH type containing 10	103284	https://www.ncbi.nlm.nih.gov/gene/?term=103284

Mdga1	Mdga1	MAM domain containing glycosylphosphatidylinositol anchor 1	74762	https://www.ncbi.nlm.nih.gov/gene/?term=74762
Tlnrd1	Tlnrd1	talin rod domain containing 1	80889	https://www.ncbi.nlm.nih.gov/gene/?term=80889
2510039O18Rik	2510039O18Rik	RIKEN cDNA 2510039O18 gene	77034	https://www.ncbi.nlm.nih.gov/gene/?term=77034
Insig1	Insig1	insulin induced gene 1	231070	https://www.ncbi.nlm.nih.gov/gene/?term=231070
Rhoq	Rhoq	ras homolog family member Q	104215	https://www.ncbi.nlm.nih.gov/gene/?term=104215
Nxpe3	Nxpe3	neurexophilin and PC-esterase domain family, member 3	385658	https://www.ncbi.nlm.nih.gov/gene/?term=385658
Rnf111	Rnf111	ring finger 111	93836	https://www.ncbi.nlm.nih.gov/gene/?term=93836
Arf6	Arf6	ADP-ribosylation factor 6	11845	https://www.ncbi.nlm.nih.gov/gene/?term=11845
Plpp6	Plpp6	phospholipid phosphatase 6	74411	https://www.ncbi.nlm.nih.gov/gene/?term=74411
Hlcs	Hlcs	holocarboxylase synthetase (biotin- [propriony-Coenzyme A-carboxylase (ATP-hydrolysing)] ligase)	110948	https://www.ncbi.nlm.nih.gov/gene/?term=110948
Syap1	Syap1	synapse associated protein 1	67043	https://www.ncbi.nlm.nih.gov/gene/?term=67043
Zkscan1	Zkscan1	zinc finger with KRAB and SCAN domains 1	74570	https://www.ncbi.nlm.nih.gov/gene/?term=74570
Dnajc8	Dnajc8	DnaJ heat shock protein family (Hsp40) member C8	68598	https://www.ncbi.nlm.nih.gov/gene/?term=68598
Slc9a1	Slc9a1	solute carrier family 9 (sodium/hydrogen exchanger), member 1	20544	https://www.ncbi.nlm.nih.gov/gene/?term=20544
Frem2	Frem2	Fras1 related extracellular matrix protein 2	242022	https://www.ncbi.nlm.nih.gov/gene/?term=242022
Magt1	Magt1	magnesium transporter 1	67075	https://www.ncbi.nlm.nih.gov/gene/?term=67075
Eif4e3	Eif4e3	eukaryotic translation initiation factor 4E member 3	66892	https://www.ncbi.nlm.nih.gov/gene/?term=66892
Ehd4	Ehd4	EH-domain containing 4	98878	https://www.ncbi.nlm.nih.gov/gene/?term=98878

Biology Open • Supplementary information

Arid1b	Arid1b	AT rich interactive domain 1B (SWI-like)	239985	https://www.ncbi.nlm.nih.gov/gene/?term=239985
Atp8b2	Atp8b2	ATPase, class I, type 8B, member 2	54667	https://www.ncbi.nlm.nih.gov/gene/?term=54667
Kif13a	Kif13a	kinesin family member 13A	16553	https://www.ncbi.nlm.nih.gov/gene/?term=16553
Vcl	Vcl	vinculin	22330	https://www.ncbi.nlm.nih.gov/gene/?term=22330
Mypn	Mypn	myopalladin	68802	https://www.ncbi.nlm.nih.gov/gene/?term=68802
Prdm1	Prdm1	PR domain containing 1, with ZNF domain	12142	https://www.ncbi.nlm.nih.gov/gene/?term=12142
Bahd1	Bahd1	bromo adjacent homology domain containing 1	228536	https://www.ncbi.nlm.nih.gov/gene/?term=228536
Zc3h12a	Zc3h12a	zinc finger CCCH type containing 12A	230738	https://www.ncbi.nlm.nih.gov/gene/?term=230738
Lmbrd2	Lmbrd2	LMBR1 domain containing 2	320506	https://www.ncbi.nlm.nih.gov/gene/?term=320506
Sgsm2	Sgsm2	small G protein signaling modulator 2	97761	https://www.ncbi.nlm.nih.gov/gene/?term=97761
Rnf44	Rnf44	ring finger protein 44	105239	https://www.ncbi.nlm.nih.gov/gene/?term=105239
Rc3h2	Rc3h2	ring finger and CCCH-type zinc finger domains 2	319817	https://www.ncbi.nlm.nih.gov/gene/?term=319817
Sh3bp5	Sh3bp5	SH3-domain binding protein 5 (BTK-associated)	24056	https://www.ncbi.nlm.nih.gov/gene/?term=24056
Btf3l4	Btf3l4	basic transcription factor 3-like 4	70533	https://www.ncbi.nlm.nih.gov/gene/?term=70533
Ppp6r3	Ppp6r3	protein phosphatase 6, regulatory subunit 3	52036	https://www.ncbi.nlm.nih.gov/gene/?term=52036
Tead1	Tead1	TEA domain family member 1	21676	https://www.ncbi.nlm.nih.gov/gene/?term=21676
Sptbn1	Sptbn1	spectrin beta, non-erythrocytic 1	20742	https://www.ncbi.nlm.nih.gov/gene/?term=20742
Isl1	Isl1	ISL1 transcription factor, LIM/homeodomain	16392	https://www.ncbi.nlm.nih.gov/gene/?term=16392
Negr1	Negr1	neuronal growth regulator 1	320840	https://www.ncbi.nlm.nih.gov/gene/?term=320840
Rc3h1	Rc3h1	RING CCCH (C3H) domains 1	381305	https://www.ncbi.nlm.nih.gov/gene/?term=381305
Rbm26	Rbm26	RNA binding motif protein 26	74213	https://www.ncbi.nlm.nih.gov/gene/?term=74213

Biology Open • Supplementary information

Npy1r	Npy1r	neuropeptide Y receptor Y1	18166	https://www.ncbi.nlm.nih.gov/gene/?term=18166
Glce	Glce	glucuronyl C5-epimerase	93683	https://www.ncbi.nlm.nih.gov/gene/?term=93683
Rhbdl3	Rhbdl3	rhomboid like 3	246104	https://www.ncbi.nlm.nih.gov/gene/?term=246104
Fech	Fech	ferrochelatase	14151	https://www.ncbi.nlm.nih.gov/gene/?term=14151
Il34	Il34	interleukin 34	76527	https://www.ncbi.nlm.nih.gov/gene/?term=76527
Tacc1	Tacc1	transforming, acidic coiled-coil containing protein 1	320165	https://www.ncbi.nlm.nih.gov/gene/?term=320165
Pik3c2a	Pik3c2a	phosphatidylinositol-4-phosphate 3-kinase catalytic subunit type 2 alpha	18704	https://www.ncbi.nlm.nih.gov/gene/?term=18704
B3gnt2	B3gnt2	UDP-GlcNAc:betaGal beta-1,3-N-acetylglucosaminyltransferase 2	53625	https://www.ncbi.nlm.nih.gov/gene/?term=53625
Khdrbs3	Khdrbs3	KH domain containing, RNA binding, signal transduction associated 3	13992	https://www.ncbi.nlm.nih.gov/gene/?term=13992
Ovca2	Ovca2	candidate tumor suppressor in ovarian cancer 2	246257	https://www.ncbi.nlm.nih.gov/gene/?term=246257
Osbp2	Osbp2	oxysterol binding protein 2	74309	https://www.ncbi.nlm.nih.gov/gene/?term=74309
Zbtb21	Zbtb21	zinc finger and BTB domain containing 21	114565	https://www.ncbi.nlm.nih.gov/gene/?term=114565
Gpc6	Gpc6	glypican 6	23888	https://www.ncbi.nlm.nih.gov/gene/?term=23888
Pdzd4	Pdzd4	PDZ domain containing 4	245469	https://www.ncbi.nlm.nih.gov/gene/?term=245469
Dusp7	Dusp7	dual specificity phosphatase 7	235584	https://www.ncbi.nlm.nih.gov/gene/?term=235584
Snx4	Snx4	sorting nexin 4	69150	https://www.ncbi.nlm.nih.gov/gene/?term=69150
Ces1g	Ces1g	carboxylesterase 1G	12623	https://www.ncbi.nlm.nih.gov/gene/?term=12623
Plekhm3	Plekhm3	pleckstrin homology domain containing, family M, member 3	241075	https://www.ncbi.nlm.nih.gov/gene/?term=241075
Plcxd2	Plcxd2	phosphatidylinositol-specific phospholipase C, X domain containing 2	433022	https://www.ncbi.nlm.nih.gov/gene/?term=433022
Chodl	Chodl	chondrolectin	246048	https://www.ncbi.nlm.nih.gov/gene/?term=246048

Biology Open • Supplementary information

Uggt1	Uggt1	UDP-glucose glycoprotein glucosyltransferase 1	320011	https://www.ncbi.nlm.nih.gov/gene/?term=320011
Slc16a1	Slc16a1	solute carrier family 16 (monocarboxylic acid transporters), member 1	20501	https://www.ncbi.nlm.nih.gov/gene/?term=20501
Nifk	Nifk	nucleolar protein interacting with the FHA domain of MKI67	67949	https://www.ncbi.nlm.nih.gov/gene/?term=67949
Camsap1	Camsap1	calmodulin regulated spectrin-associated protein 1	227634	https://www.ncbi.nlm.nih.gov/gene/?term=227634
Rftn1	Rftn1	raftlin lipid raft linker 1	76438	https://www.ncbi.nlm.nih.gov/gene/?term=76438
Neurod4	Neurod4	neurogenic differentiation 4	11923	https://www.ncbi.nlm.nih.gov/gene/?term=11923
Kctd12	Kctd12	potassium channel tetramerisation domain containing 12	239217	https://www.ncbi.nlm.nih.gov/gene/?term=239217
Mapkap1	Mapkap1	mitogen-activated protein kinase associated protein 1	227743	https://www.ncbi.nlm.nih.gov/gene/?term=227743
Zfp612	Zfp612	zinc finger protein 612	234725	https://www.ncbi.nlm.nih.gov/gene/?term=234725
Magee2	Magee2	melanoma antigen, family E, 2	272790	https://www.ncbi.nlm.nih.gov/gene/?term=272790
Caps2	Caps2	calcyphosphine 2	353025	https://www.ncbi.nlm.nih.gov/gene/?term=353025
Henmt1	Henmt1	HEN1 methyltransferase homolog 1 (Arabidopsis)	66715	https://www.ncbi.nlm.nih.gov/gene/?term=66715
Gabpa	Gabpa	GA repeat binding protein, alpha	14390	https://www.ncbi.nlm.nih.gov/gene/?term=14390
Oprk1	Oprk1	opioid receptor, kappa 1	18387	https://www.ncbi.nlm.nih.gov/gene/?term=18387
Rptor	Rptor	regulatory associated protein of MTOR, complex 1	74370	https://www.ncbi.nlm.nih.gov/gene/?term=74370
Havcr1	Havcr1	hepatitis A virus cellular receptor 1	171283	https://www.ncbi.nlm.nih.gov/gene/?term=171283
Vps4b	Vps4b	vacuolar protein sorting 4B	20479	https://www.ncbi.nlm.nih.gov/gene/?term=20479
Plekhg1	Plekhg1	pleckstrin homology domain containing, family G (with RhoGef domain) member 1	213783	https://www.ncbi.nlm.nih.gov/gene/?term=213783

Biology Open • Supplementary information

Ermap	Ermap	erythroblast membrane-associated protein	27028	https://www.ncbi.nlm.nih.gov/gene/?term=27028
Atp2b2	Atp2b2	ATPase, Ca++ transporting, plasma membrane 2	11941	https://www.ncbi.nlm.nih.gov/gene/?term=11941
Tram1	Tram1	translocating chain-associating membrane protein 1	72265	https://www.ncbi.nlm.nih.gov/gene/?term=72265
Pdcl	Pdcl	phosducin-like	67466	https://www.ncbi.nlm.nih.gov/gene/?term=67466
Rab5if	Rab5if	RAB5 interacting factor	67388	https://www.ncbi.nlm.nih.gov/gene/?term=67388
Fgf23	Fgf23	fibroblast growth factor 23	64654	https://www.ncbi.nlm.nih.gov/gene/?term=64654
Sort1	Sort1	sortilin 1	20661	https://www.ncbi.nlm.nih.gov/gene/?term=20661
Plxnb1	Plxnb1	plexin B1	235611	https://www.ncbi.nlm.nih.gov/gene/?term=235611
Gdap2	Gdap2	ganglioside-induced differentiation-associated-protein 2	14547	https://www.ncbi.nlm.nih.gov/gene/?term=14547
Cldnd1	Cldnd1	claudin domain containing 1	224250	https://www.ncbi.nlm.nih.gov/gene/?term=224250
Rbpjl	Rbpjl	recombination signal binding protein for immunoglobulin kappa J region-like	19668	https://www.ncbi.nlm.nih.gov/gene/?term=19668
Tab2	Tab2	TGF-beta activated kinase 1/MAP3K7 binding protein 2	68652	https://www.ncbi.nlm.nih.gov/gene/?term=68652
Dtl	Dtl	denticleless E3 ubiquitin protein ligase	76843	https://www.ncbi.nlm.nih.gov/gene/?term=76843
Snd1	Snd1	staphylococcal nuclease and tudor domain containing 1	56463	https://www.ncbi.nlm.nih.gov/gene/?term=56463
Ssb	Ssb	Sjogren syndrome antigen B	20823	https://www.ncbi.nlm.nih.gov/gene/?term=20823
Sorbs1	Sorbs1	sorbin and SH3 domain containing 1	20411	https://www.ncbi.nlm.nih.gov/gene/?term=20411
Car8	Car8	carbonic anhydrase 8	12319	https://www.ncbi.nlm.nih.gov/gene/?term=12319
Ripk4	Ripk4	receptor-interacting serine-threonine kinase 4	72388	https://www.ncbi.nlm.nih.gov/gene/?term=72388
Dusp6	Dusp6	dual specificity phosphatase 6	67603	https://www.ncbi.nlm.nih.gov/gene/?term=67603

Biology Open • Supplementary information

Rcvrn	Rcvrn	recoverin	19674	https://www.ncbi.nlm.nih.gov/gene/?term=19674
Tm9sf2	Tm9sf2	transmembrane 9 superfamily member 2	68059	https://www.ncbi.nlm.nih.gov/gene/?term=68059
Trim44	Trim44	tripartite motif-containing 44	80985	https://www.ncbi.nlm.nih.gov/gene/?term=80985
Gal3st3	Gal3st3	galactose-3-O-sulfotransferase 3	545276	https://www.ncbi.nlm.nih.gov/gene/?term=545276
Zdhhc3	Zdhhc3	zinc finger, DHHC domain containing 3	69035	https://www.ncbi.nlm.nih.gov/gene/?term=69035
Slc30a7	Slc30a7	solute carrier family 30 (zinc transporter), member 7	66500	https://www.ncbi.nlm.nih.gov/gene/?term=66500
Fam168b	Fam168b	family with sequence similarity 168, member B	214469	https://www.ncbi.nlm.nih.gov/gene/?term=214469
Tmed8	Tmed8	transmembrane p24 trafficking protein 8	382620	https://www.ncbi.nlm.nih.gov/gene/?term=382620
Mgat4a	Mgat4a	mannoside acetylglucosaminyltransferase 4, isoenzyme A	269181	https://www.ncbi.nlm.nih.gov/gene/?term=269181
Chmp7	Chmp7	charged multivesicular body protein 7	105513	https://www.ncbi.nlm.nih.gov/gene/?term=105513
Klf4	Klf4	Kruppel-like factor 4 (gut)	16600	https://www.ncbi.nlm.nih.gov/gene/?term=16600
Trim16	Trim16	tripartite motif-containing 16	94092	https://www.ncbi.nlm.nih.gov/gene/?term=94092
Rbm25	Rbm25	RNA binding motif protein 25	67039	https://www.ncbi.nlm.nih.gov/gene/?term=67039
Retnlb	Retnlb	resistin like beta	57263	https://www.ncbi.nlm.nih.gov/gene/?term=57263
Pcdh7	Pcdh7	protocadherin 7	54216	https://www.ncbi.nlm.nih.gov/gene/?term=54216
Nrxn1	Nrxn1	neurexin I	18189	https://www.ncbi.nlm.nih.gov/gene/?term=18189
Galnt13	Galnt13	polypeptide N-acetylgalactosaminyltransferase 13	271786	https://www.ncbi.nlm.nih.gov/gene/?term=271786
Lef1	Lef1	lymphoid enhancer binding factor 1	16842	https://www.ncbi.nlm.nih.gov/gene/?term=16842
Rsbn1	Rsbn1	rosbin, round spermatid basic protein 1	229675	https://www.ncbi.nlm.nih.gov/gene/?term=229675
Usp34	Usp34	ubiquitin specific peptidase 34	17847	https://www.ncbi.nlm.nih.gov/gene/?term=17847

Biology Open • Supplementary information

Vapa	Vapa	vesicle-associated membrane protein, associated protein A	30960	https://www.ncbi.nlm.nih.gov/gene/?term=30960
Rdx	Rdx	radixin	19684	https://www.ncbi.nlm.nih.gov/gene/?term=19684
Dtna	Dtna	dystrobrevin alpha	13527	https://www.ncbi.nlm.nih.gov/gene/?term=13527
Proser2	Proser2	proline and serine rich 2	227545	https://www.ncbi.nlm.nih.gov/gene/?term=227545
Zfp207	Zfp207	zinc finger protein 207	22680	https://www.ncbi.nlm.nih.gov/gene/?term=22680
Sema3e	Sema3e	sema domain, immunoglobulin domain (Ig), short basic domain, secreted, (semaphorin) 3E	20349	https://www.ncbi.nlm.nih.gov/gene/?term=20349
Krit1	Krit1	KRIT1, ankyrin repeat containing	79264	https://www.ncbi.nlm.nih.gov/gene/?term=79264
Cul2	Cul2	cullin 2	71745	https://www.ncbi.nlm.nih.gov/gene/?term=71745
Dhx40	Dhx40	DEAH (Asp-Glu-Ala-His) box polypeptide 40	67487	https://www.ncbi.nlm.nih.gov/gene/?term=67487
Thap12	Thap12	THAP domain containing 12	72981	https://www.ncbi.nlm.nih.gov/gene/?term=72981
Mynn	Mynn	myoneurin	80732	https://www.ncbi.nlm.nih.gov/gene/?term=80732
Dock5	Dock5	dedicator of cytokinesis 5	68813	https://www.ncbi.nlm.nih.gov/gene/?term=68813
Epm2aip1	Epm2aip1	EPM2A (laforin) interacting protein 1	77781	https://www.ncbi.nlm.nih.gov/gene/?term=77781
Tfg	Tfg	Trk-fused gene	21787	https://www.ncbi.nlm.nih.gov/gene/?term=21787
Tyro3	Tyro3	TYRO3 protein tyrosine kinase 3	22174	https://www.ncbi.nlm.nih.gov/gene/?term=22174
Top2a	Top2a	topoisomerase (DNA) II alpha	21973	https://www.ncbi.nlm.nih.gov/gene/?term=21973
Map2k3	Map2k3	mitogen-activated protein kinase kinase 3	26397	https://www.ncbi.nlm.nih.gov/gene/?term=26397
Tbx20	Tbx20	T-box 20	57246	https://www.ncbi.nlm.nih.gov/gene/?term=57246
Sypl	Sypl	synaptophysin-like protein	19027	https://www.ncbi.nlm.nih.gov/gene/?term=19027
Oaz1	Oaz1	ornithine decarboxylase antizyme 1	18245	https://www.ncbi.nlm.nih.gov/gene/?term=18245
Ube2g2	Ube2g2	ubiquitin-conjugating enzyme E2G 2	22213	https://www.ncbi.nlm.nih.gov/gene/?term=22213
Rimklb	Rimklb	ribosomal modification protein rimK-like family member B	108653	https://www.ncbi.nlm.nih.gov/gene/?term=108653

Biology Open • Supplementary information

Nck1	Nck1	non-catalytic region of tyrosine kinase adaptor protein 1	17973	https://www.ncbi.nlm.nih.gov/gene/?term=17973
Tra2a	Tra2a	transformer 2 alpha	101214	https://www.ncbi.nlm.nih.gov/gene/?term=101214
Ubqln1	Ubqln1	ubiquilin 1	56085	https://www.ncbi.nlm.nih.gov/gene/?term=56085
Inpp1	Inpp1	inositol polyphosphate-1-phosphatase	16329	https://www.ncbi.nlm.nih.gov/gene/?term=16329
Pik3ca	Pik3ca	phosphatidylinositol-4,5-bisphosphate 3-kinase catalytic subunit alpha	18706	https://www.ncbi.nlm.nih.gov/gene/?term=18706
Fam120a	Fam120a	family with sequence similarity 120, member A	218236	https://www.ncbi.nlm.nih.gov/gene/?term=218236
Epha5	Epha5	Eph receptor A5	13839	https://www.ncbi.nlm.nih.gov/gene/?term=13839
Dact1	Dact1	dishevelled-binding antagonist of beta-catenin 1	59036	https://www.ncbi.nlm.nih.gov/gene/?term=59036
Cherp	Cherp	calcium homeostasis endoplasmic reticulum protein	27967	https://www.ncbi.nlm.nih.gov/gene/?term=27967
Rrp1b	Rrp1b	ribosomal RNA processing 1 homolog B (S. cerevisiae)	72462	https://www.ncbi.nlm.nih.gov/gene/?term=72462
Traf5	Traf5	TNF receptor-associated factor 5	22033	https://www.ncbi.nlm.nih.gov/gene/?term=22033
Paf1	Paf1	Paf1, RNA polymerase II complex component	54624	https://www.ncbi.nlm.nih.gov/gene/?term=54624
Pced1b	Pced1b	PC-esterase domain containing 1B	239647	https://www.ncbi.nlm.nih.gov/gene/?term=239647
Zc3h7a	Zc3h7a	zinc finger CCCH type containing 7 A	106205	https://www.ncbi.nlm.nih.gov/gene/?term=106205
Gcfc2	Gcfc2	GC-rich sequence DNA binding factor 2	330361	https://www.ncbi.nlm.nih.gov/gene/?term=330361
Rtl6	Rtl6	retrotransposon Gag like 6	223732	https://www.ncbi.nlm.nih.gov/gene/?term=223732
Dmtn	Dmtn	dematin actin binding protein	13829	https://www.ncbi.nlm.nih.gov/gene/?term=13829
Kdm2a	Kdm2a	lysine (K)-specific demethylase 2A	225876	https://www.ncbi.nlm.nih.gov/gene/?term=225876
Adss	Adss	adenylosuccinate synthetase, non	11566	https://www.ncbi.nlm.nih.gov/gene/?term=11566

Biology Open • Supplementary information

		muscle		
Ar	Ar	androgen receptor	11835	https://www.ncbi.nlm.nih.gov/gene/?term=11835
Cd209b	Cd209b	CD209b antigen	69165	https://www.ncbi.nlm.nih.gov/gene/?term=69165
B3galt1	B3galt1	UDP-Gal:betaGlcNAc beta 1,3-galactosyltransferase, polypeptide 1	26877	https://www.ncbi.nlm.nih.gov/gene/?term=26877
Fam155a	Fam155a	family with sequence similarity 155, member A	270028	https://www.ncbi.nlm.nih.gov/gene/?term=270028
Rspo4	Rspo4	R-spondin 4	228770	https://www.ncbi.nlm.nih.gov/gene/?term=228770
Ndst3	Ndst3	N-deacetylase/N-sulfotransferase (heparan glucosaminyl) 3	83398	https://www.ncbi.nlm.nih.gov/gene/?term=83398
Abhd2	Abhd2	abhydrolase domain containing 2	54608	https://www.ncbi.nlm.nih.gov/gene/?term=54608
Mmp16	Mmp16	matrix metallopeptidase 16	17389	https://www.ncbi.nlm.nih.gov/gene/?term=17389
A430033K04Rik	A430033K04Rik	RIKEN cDNA A430033K04 gene	243308	https://www.ncbi.nlm.nih.gov/gene/?term=243308
Abcg4	Abcg4	ATP binding cassette subfamily G member 4	192663	https://www.ncbi.nlm.nih.gov/gene/?term=192663
Trim41	Trim41	tripartite motif-containing 41	211007	https://www.ncbi.nlm.nih.gov/gene/?term=211007
Pcp4	Pcp4	Purkinje cell protein 4	18546	https://www.ncbi.nlm.nih.gov/gene/?term=18546
Ehd1	Ehd1	EH-domain containing 1	13660	https://www.ncbi.nlm.nih.gov/gene/?term=13660
Ces2g	Ces2g	carboxylesterase 2G	72361	https://www.ncbi.nlm.nih.gov/gene/?term=72361
Tmem25	Tmem25	transmembrane protein 25	71687	https://www.ncbi.nlm.nih.gov/gene/?term=71687
Tomm22	Tomm22	translocase of outer mitochondrial membrane 22	223696	https://www.ncbi.nlm.nih.gov/gene/?term=223696
Mmp17	Mmp17	matrix metallopeptidase 17	23948	https://www.ncbi.nlm.nih.gov/gene/?term=23948
Zfp937	Zfp937	zinc finger protein 937	245174	https://www.ncbi.nlm.nih.gov/gene/?term=245174
Ptgfr	Ptgfr	prostaglandin F receptor	19220	https://www.ncbi.nlm.nih.gov/gene/?term=19220
Synpr	Synpr	synaptoporin	72003	https://www.ncbi.nlm.nih.gov/gene/?term=72003
Ebf1	Ebf1	early B cell factor 1	13591	https://www.ncbi.nlm.nih.gov/gene/?term=13591
AI597479	AI597479	expressed sequence AI597479	98404	https://www.ncbi.nlm.nih.gov/gene/?term=98404

Biology Open • Supplementary information

Mcc	Mcc	mutated in colorectal cancers	328949	https://www.ncbi.nlm.nih.gov/gene/?term=328949
Zfp345	Zfp345	zinc finger protein 345	545471	https://www.ncbi.nlm.nih.gov/gene/?term=545471
Aasdhppt	Aasdhppt	aminoadipate-semialdehyde dehydrogenase-phosphopantetheinyl transferase	67618	https://www.ncbi.nlm.nih.gov/gene/?term=67618
Tle1	Tle1	transducin-like enhancer of split 1	21885	https://www.ncbi.nlm.nih.gov/gene/?term=21885
Lpp	Lpp	LIM domain containing preferred translocation partner in lipoma	210126	https://www.ncbi.nlm.nih.gov/gene/?term=210126
Hes1	Hes1	hes family bHLH transcription factor 1	15205	https://www.ncbi.nlm.nih.gov/gene/?term=15205
Hcfc1	Hcfc1	host cell factor C1	15161	https://www.ncbi.nlm.nih.gov/gene/?term=15161
Rab11b	Rab11b	RAB11B, member RAS oncogene family	19326	https://www.ncbi.nlm.nih.gov/gene/?term=19326
Txlng	Txlng	taxilin gamma	353170	https://www.ncbi.nlm.nih.gov/gene/?term=353170
Shank2	Shank2	SH3 and multiple ankyrin repeat domains 2	210274	https://www.ncbi.nlm.nih.gov/gene/?term=210274
Akap9	Akap9	A kinase (PRKA) anchor protein (yotiao) 9	100986	https://www.ncbi.nlm.nih.gov/gene/?term=100986
Dusp10	Dusp10	dual specificity phosphatase 10	63953	https://www.ncbi.nlm.nih.gov/gene/?term=63953
Ctdspl2	Ctdspl2	CTD (carboxy-terminal domain, RNA polymerase II, polypeptide A) small phosphatase like 2	329506	https://www.ncbi.nlm.nih.gov/gene/?term=329506
Maea	Maea	macrophage erythroblast attacher	59003	https://www.ncbi.nlm.nih.gov/gene/?term=59003
Rmdn2	Rmdn2	regulator of microtubule dynamics 2	381110	https://www.ncbi.nlm.nih.gov/gene/?term=381110
Ash1l	Ash1l	ASH1 like histone lysine methyltransferase	192195	https://www.ncbi.nlm.nih.gov/gene/?term=192195
Shisa2	Shisa2	shisa family member 2	219134	https://www.ncbi.nlm.nih.gov/gene/?term=219134
E2f8	E2f8	E2F transcription factor 8	108961	https://www.ncbi.nlm.nih.gov/gene/?term=108961
Hmgb2	Hmgb2	high mobility group box 2	97165	https://www.ncbi.nlm.nih.gov/gene/?term=97165

Biology Open • Supplementary information

Sost	Sost	sclerostin	74499	https://www.ncbi.nlm.nih.gov/gene/?term=74499
Minpp1	Minpp1	multiple inositol polyphosphate histidine phosphatase 1	17330	https://www.ncbi.nlm.nih.gov/gene/?term=17330
Myf5	Myf5	myogenic factor 5	17877	https://www.ncbi.nlm.nih.gov/gene/?term=17877
Cep41	Cep41	centrosomal protein 41	83922	https://www.ncbi.nlm.nih.gov/gene/?term=83922
Ccnjl	Ccnjl	cyclin J-like	380694	https://www.ncbi.nlm.nih.gov/gene/?term=380694
Grhl1	Grhl1	grainyhead like transcription factor 1	195733	https://www.ncbi.nlm.nih.gov/gene/?term=195733
Isca1	Isca1	iron-sulfur cluster assembly 1	69046	https://www.ncbi.nlm.nih.gov/gene/?term=69046
Zfp521	Zfp521	zinc finger protein 521	225207	https://www.ncbi.nlm.nih.gov/gene/?term=225207
Fnip1	Fnip1	folliculin interacting protein 1	216742	https://www.ncbi.nlm.nih.gov/gene/?term=216742
Dpp10	Dpp10	dipeptidylpeptidase 10	269109	https://www.ncbi.nlm.nih.gov/gene/?term=269109
Rnf38	Rnf38	ring finger protein 38	73469	https://www.ncbi.nlm.nih.gov/gene/?term=73469
Atxn3	Atxn3	ataxin 3	110616	https://www.ncbi.nlm.nih.gov/gene/?term=110616
Dcaf6	Dcaf6	DDB1 and CUL4 associated factor 6	74106	https://www.ncbi.nlm.nih.gov/gene/?term=74106
Ptpro	Ptpro	protein tyrosine phosphatase, receptor type, O	19277	https://www.ncbi.nlm.nih.gov/gene/?term=19277
Syn2	Syn2	synapsin II	20965	https://www.ncbi.nlm.nih.gov/gene/?term=20965
Med19	Med19	mediator complex subunit 19	381379	https://www.ncbi.nlm.nih.gov/gene/?term=381379
Hecw1	Hecw1	HECT, C2 and WW domain containing E3 ubiquitin protein ligase 1	94253	https://www.ncbi.nlm.nih.gov/gene/?term=94253
Edem1	Edem1	ER degradation enhancer, mannosidase alpha-like 1	192193	https://www.ncbi.nlm.nih.gov/gene/?term=192193
Hand2	Hand2	heart and neural crest derivatives expressed 2	15111	https://www.ncbi.nlm.nih.gov/gene/?term=15111
G3bp2	G3bp2	GTPase activating protein (SH3 domain) binding protein 2	23881	https://www.ncbi.nlm.nih.gov/gene/?term=23881
Dennd1b	Dennd1b	DENN/MADD domain containing 1B	329260	https://www.ncbi.nlm.nih.gov/gene/?term=329260

Fam160b1	Fam160b1	family with sequence similarity 160, member B1	226252	https://www.ncbi.nlm.nih.gov/gene/?term=226252
Atp7a	Atp7a	ATPase, Cu++ transporting, alpha polypeptide	11977	https://www.ncbi.nlm.nih.gov/gene/?term=11977
Dsc2	Dsc2	desmocollin 2	13506	https://www.ncbi.nlm.nih.gov/gene/?term=13506
Dennd4b	Dennd4b	DENN/MADD domain containing 4B	229541	https://www.ncbi.nlm.nih.gov/gene/?term=229541
Iqgap2	Iqgap2	IQ motif containing GTPase activating protein 2	544963	https://www.ncbi.nlm.nih.gov/gene/?term=544963
Ppp1r37	Ppp1r37	protein phosphatase 1, regulatory subunit 37	232947	https://www.ncbi.nlm.nih.gov/gene/?term=232947
Cdca7l	Cdca7l	cell division cycle associated 7 like	217946	https://www.ncbi.nlm.nih.gov/gene/?term=217946
Trim65	Trim65	tripartite motif-containing 65	338364	https://www.ncbi.nlm.nih.gov/gene/?term=338364
Ddc	Ddc	dopa decarboxylase	13195	https://www.ncbi.nlm.nih.gov/gene/?term=13195
Phlpp2	Phlpp2	PH domain and leucine rich repeat protein phosphatase 2	244650	https://www.ncbi.nlm.nih.gov/gene/?term=244650
Zfyve21	Zfyve21	zinc finger, FYVE domain containing 21	68520	https://www.ncbi.nlm.nih.gov/gene/?term=68520
Cic	Cic	capicua transcriptional repressor	71722	https://www.ncbi.nlm.nih.gov/gene/?term=71722
Ssbp2	Ssbp2	single-stranded DNA binding protein 2	66970	https://www.ncbi.nlm.nih.gov/gene/?term=66970
Snx13	Snx13	sorting nexin 13	217463	https://www.ncbi.nlm.nih.gov/gene/?term=217463
Itga6	Itga6	integrin alpha 6	16403	https://www.ncbi.nlm.nih.gov/gene/?term=16403
Itgav	Itgav	integrin alpha V	16410	https://www.ncbi.nlm.nih.gov/gene/?term=16410
Spryd4	Spryd4	SPRY domain containing 4	66701	https://www.ncbi.nlm.nih.gov/gene/?term=66701
Cdk16	Cdk16	cyclin-dependent kinase 16	18555	https://www.ncbi.nlm.nih.gov/gene/?term=18555
Myo1b	Myo1b	myosin IB	17912	https://www.ncbi.nlm.nih.gov/gene/?term=17912
Klhl14	Klhl14	kelch-like 14	225266	https://www.ncbi.nlm.nih.gov/gene/?term=225266
Gpr180	Gpr180	G protein-coupled receptor 180	58245	https://www.ncbi.nlm.nih.gov/gene/?term=58245
Mia3	Mia3	melanoma inhibitory activity 3	338366	https://www.ncbi.nlm.nih.gov/gene/?term=338366

Biology Open • Supplementary information

Tob2	Tob2	transducer of ERBB2, 2	57259	https://www.ncbi.nlm.nih.gov/gene/?term=57259
P3h3	P3h3	prolyl 3-hydroxylase 3	14789	https://www.ncbi.nlm.nih.gov/gene/?term=14789
Lhfpl2	Lhfpl2	lipoma HMGIC fusion partner-like 2	218454	https://www.ncbi.nlm.nih.gov/gene/?term=218454
Ergic2	Ergic2	ERGIC and golgi 2	67456	https://www.ncbi.nlm.nih.gov/gene/?term=67456
Pp2d1	Pp2d1	protein phosphatase 2C-like domain containing 1	110332	https://www.ncbi.nlm.nih.gov/gene/?term=110332
Grp	Grp	gastrin releasing peptide	225642	https://www.ncbi.nlm.nih.gov/gene/?term=225642
Pcolce2	Pcolce2	procollagen C-endopeptidase enhancer 2	76477	https://www.ncbi.nlm.nih.gov/gene/?term=76477
Glra1	Glra1	glycine receptor, alpha 1 subunit	14654	https://www.ncbi.nlm.nih.gov/gene/?term=14654
Hivep1	Hivep1	human immunodeficiency virus type I enhancer binding protein 1	110521	https://www.ncbi.nlm.nih.gov/gene/?term=110521
Rhpn2	Rhpn2	rhophilin, Rho GTPase binding protein 2	52428	https://www.ncbi.nlm.nih.gov/gene/?term=52428
Fmn2	Fmn2	formin 2	54418	https://www.ncbi.nlm.nih.gov/gene/?term=54418
Golga4	Golga4	golgi autoantigen, golgin subfamily a, 4	54214	https://www.ncbi.nlm.nih.gov/gene/?term=54214
Fancm	Fancm	Fanconi anemia, complementation group M	104806	https://www.ncbi.nlm.nih.gov/gene/?term=104806
Nefm	Nefm	neurofilament, medium polypeptide	18040	https://www.ncbi.nlm.nih.gov/gene/?term=18040
Tulp4	Tulp4	tubby like protein 4	68842	https://www.ncbi.nlm.nih.gov/gene/?term=68842
Evx2	Evx2	even-skipped homeobox 2	14029	https://www.ncbi.nlm.nih.gov/gene/?term=14029
Gnpda2	Gnpda2	glucosamine-6-phosphate deaminase 2	67980	https://www.ncbi.nlm.nih.gov/gene/?term=67980
Wwp2	Wwp2	WW domain containing E3 ubiquitin protein ligase 2	66894	https://www.ncbi.nlm.nih.gov/gene/?term=66894
Cd69	Cd69	CD69 antigen	12515	https://www.ncbi.nlm.nih.gov/gene/?term=12515
Adam10	Adam10	a disintegrin and metallopeptidase domain 10	11487	https://www.ncbi.nlm.nih.gov/gene/?term=11487

Dmxl1	Dmxl1	Dmx-like 1	240283	https://www.ncbi.nlm.nih.gov/gene/?term=240283
Paxbp1	Paxbp1	PAX3 and PAX7 binding protein 1	67367	https://www.ncbi.nlm.nih.gov/gene/?term=67367
Tent4a	Tent4a	terminal nucleotidyltransferase 4A	210106	https://www.ncbi.nlm.nih.gov/gene/?term=210106
Slc12a5	Slc12a5	solute carrier family 12, member 5	57138	https://www.ncbi.nlm.nih.gov/gene/?term=57138
Ugp2	Ugp2	UDP-glucose pyrophosphorylase 2	216558	https://www.ncbi.nlm.nih.gov/gene/?term=216558
Dnajb9	Dnajb9	DnaJ heat shock protein family (Hsp40) member B9	27362	https://www.ncbi.nlm.nih.gov/gene/?term=27362
Gfpt2	Gfpt2	glutamine fructose-6-phosphate transaminase 2	14584	https://www.ncbi.nlm.nih.gov/gene/?term=14584
Cux1	Cux1	cut-like homeobox 1	13047	https://www.ncbi.nlm.nih.gov/gene/?term=13047
Adam23	Adam23	a disintegrin and metallopeptidase domain 23	23792	https://www.ncbi.nlm.nih.gov/gene/?term=23792
A830018L16Rik	A830018L16Rik	RIKEN cDNA A830018L16 gene	320492	https://www.ncbi.nlm.nih.gov/gene/?term=320492
Cldn11	Cldn11	claudin 11	18417	https://www.ncbi.nlm.nih.gov/gene/?term=18417
Ppcs	Ppcs	phosphopantothenoylcysteine synthetase	106564	https://www.ncbi.nlm.nih.gov/gene/?term=106564
Ptar1	Ptar1	protein prenyltransferase alpha subunit repeat containing 1	72351	https://www.ncbi.nlm.nih.gov/gene/?term=72351
Pip5k1c	Pip5k1c	phosphatidylinositol-4-phosphate 5-kinase, type 1 gamma	18717	https://www.ncbi.nlm.nih.gov/gene/?term=18717
Usp28	Usp28	ubiquitin specific peptidase 28	235323	https://www.ncbi.nlm.nih.gov/gene/?term=235323
Kcns3	Kcns3	potassium voltage-gated channel, delayed-rectifier, subfamily S, member 3	238076	https://www.ncbi.nlm.nih.gov/gene/?term=238076
Ccser1	Ccser1	coiled-coil serine rich 1	232035	https://www.ncbi.nlm.nih.gov/gene/?term=232035
Trappc6b	Trappc6b	trafficking protein particle complex 6B	78232	https://www.ncbi.nlm.nih.gov/gene/?term=78232
Zfp420	Zfp420	zinc finger protein 420	233058	https://www.ncbi.nlm.nih.gov/gene/?term=233058

Biology Open • Supplementary information

Asxl2	Asxl2	additional sex combs like 2, transcriptional regulator	75302	https://www.ncbi.nlm.nih.gov/gene/?term=75302
Mttp	Mttp	microsomal triglyceride transfer protein	17777	https://www.ncbi.nlm.nih.gov/gene/?term=17777
Csde1	Csde1	cold shock domain containing E1, RNA binding	229663	https://www.ncbi.nlm.nih.gov/gene/?term=229663
Rarres1	Rarres1	retinoic acid receptor responder (tazarotene induced) 1	109222	https://www.ncbi.nlm.nih.gov/gene/?term=109222
Tsc1	Tsc1	tuberous sclerosis 1	64930	https://www.ncbi.nlm.nih.gov/gene/?term=64930
Kcnab1	Kcnab1	potassium voltage-gated channel, shaker-related subfamily, beta member 1	16497	https://www.ncbi.nlm.nih.gov/gene/?term=16497
Slc44a4	Slc44a4	solute carrier family 44, member 4	70129	https://www.ncbi.nlm.nih.gov/gene/?term=70129
Ptgs1	Ptgs1	prostaglandin-endoperoxide synthase 1	19224	https://www.ncbi.nlm.nih.gov/gene/?term=19224
Mex3b	Mex3b	mex3 RNA binding family member B	108797	https://www.ncbi.nlm.nih.gov/gene/?term=108797
Slco1c1	Slco1c1	solute carrier organic anion transporter family, member 1c1	58807	https://www.ncbi.nlm.nih.gov/gene/?term=58807
Prkci	Prkci	protein kinase C, iota	18759	https://www.ncbi.nlm.nih.gov/gene/?term=18759
Fam20c	Fam20c	family with sequence similarity 20, member C	80752	https://www.ncbi.nlm.nih.gov/gene/?term=80752
Foxj3	Foxj3	forkhead box J3	230700	https://www.ncbi.nlm.nih.gov/gene/?term=230700
Stx7	Stx7	syntaxin 7	53331	https://www.ncbi.nlm.nih.gov/gene/?term=53331
Matr3	Matr3	matrin 3	17184	https://www.ncbi.nlm.nih.gov/gene/?term=17184
Cks2	Cks2	CDC28 protein kinase regulatory subunit 2	66197	https://www.ncbi.nlm.nih.gov/gene/?term=66197
Mamdc2	Mamdc2	MAM domain containing 2	71738	https://www.ncbi.nlm.nih.gov/gene/?term=71738
Zfp804a	Zfp804a	zinc finger protein 804A	241514	https://www.ncbi.nlm.nih.gov/gene/?term=241514
Atrx	Atrx	ATRX, chromatin remodeler	22589	https://www.ncbi.nlm.nih.gov/gene/?term=22589

Biology Open • Supplementary information

Ccdc146	Ccdc146	coiled-coil domain containing 146	75172	https://www.ncbi.nlm.nih.gov/gene/?term=75172
Trpa1	Trpa1	transient receptor potential cation channel, subfamily A, member 1	277328	https://www.ncbi.nlm.nih.gov/gene/?term=277328
Rer1	Rer1	retention in endoplasmic reticulum sorting receptor 1	67830	https://www.ncbi.nlm.nih.gov/gene/?term=67830
Fmr1	Fmr1	fragile X mental retardation 1	14265	https://www.ncbi.nlm.nih.gov/gene/?term=14265
Abcc5	Abcc5	ATP-binding cassette, sub-family C (CFTR/MRP), member 5	27416	https://www.ncbi.nlm.nih.gov/gene/?term=27416
D230025D16Rik	D230025D16Rik	RIKEN cDNA D230025D16 gene	234678	https://www.ncbi.nlm.nih.gov/gene/?term=234678
Eda	Eda	ectodysplasin-A	13607	https://www.ncbi.nlm.nih.gov/gene/?term=13607
Ddx6	Ddx6	DEAD (Asp-Glu-Ala-Asp) box polypeptide 6	13209	https://www.ncbi.nlm.nih.gov/gene/?term=13209
Ago1	Ago1	argonaute RISC catalytic subunit 1	236511	https://www.ncbi.nlm.nih.gov/gene/?term=236511
Miga2	Miga2	mitoguardin 2	108958	https://www.ncbi.nlm.nih.gov/gene/?term=108958
Pou4f1	Pou4f1	POU domain, class 4, transcription factor 1	18996	https://www.ncbi.nlm.nih.gov/gene/?term=18996
Skida1	Skida1	SKI/DACH domain containing 1	72668	https://www.ncbi.nlm.nih.gov/gene/?term=72668
Map3k8	Map3k8	mitogen-activated protein kinase kinase kinase 8	26410	https://www.ncbi.nlm.nih.gov/gene/?term=26410
Impdh1	Impdh1	inosine monophosphate dehydrogenase 1	23917	https://www.ncbi.nlm.nih.gov/gene/?term=23917
Ccdc126	Ccdc126	coiled-coil domain containing 126	57895	https://www.ncbi.nlm.nih.gov/gene/?term=57895
Vps37a	Vps37a	vacuolar protein sorting 37A	52348	https://www.ncbi.nlm.nih.gov/gene/?term=52348
Emx2	Emx2	empty spiracles homeobox 2	13797	https://www.ncbi.nlm.nih.gov/gene/?term=13797
Cep170	Cep170	centrosomal protein 170	545389	https://www.ncbi.nlm.nih.gov/gene/?term=545389
Rfx7	Rfx7	regulatory factor X, 7	319758	https://www.ncbi.nlm.nih.gov/gene/?term=319758
Mphosph9	Mphosph9	M-phase phosphoprotein 9	269702	https://www.ncbi.nlm.nih.gov/gene/?term=269702
Caprin2	Caprin2	caprin family member 2	232560	https://www.ncbi.nlm.nih.gov/gene/?term=232560

Biology Open • Supplementary information

Kbtbd8	Kbtbd8	kelch repeat and BTB (POZ) domain containing 8	243574	https://www.ncbi.nlm.nih.gov/gene/?term=243574
Lcorl	Lcorl	ligand dependent nuclear receptor corepressor-like	209707	https://www.ncbi.nlm.nih.gov/gene/?term=209707
Sybu	Sybu	syntabulin (syntaxin-interacting)	319613	https://www.ncbi.nlm.nih.gov/gene/?term=319613
St18	St18	suppression of tumorigenicity 18	240690	https://www.ncbi.nlm.nih.gov/gene/?term=240690
Slmap	Slmap	sarcolemma associated protein	83997	https://www.ncbi.nlm.nih.gov/gene/?term=83997
Garem1	Garem1	GRB2 associated regulator of MAPK1 subtype 1	381126	https://www.ncbi.nlm.nih.gov/gene/?term=381126
Hprt	Hprt	hypoxanthine guanine phosphoribosyl transferase	15452	https://www.ncbi.nlm.nih.gov/gene/?term=15452
Kcna4	Kcna4	potassium voltage-gated channel, shaker-related subfamily, member 4	16492	https://www.ncbi.nlm.nih.gov/gene/?term=16492
Arap2	Arap2	ArfGAP with RhoGAP domain, ankyrin repeat and PH domain 2	212285	https://www.ncbi.nlm.nih.gov/gene/?term=212285
Mllt6	Mllt6	myeloid/lymphoid or mixed-lineage leukemia; translocated to, 6	246198	https://www.ncbi.nlm.nih.gov/gene/?term=246198
Cbfb	Cbfb	core binding factor beta	12400	https://www.ncbi.nlm.nih.gov/gene/?term=12400
Spg20	Spg20	spastic paraplegia 20, spartin (Troyer syndrome) homolog (human)	229285	https://www.ncbi.nlm.nih.gov/gene/?term=229285
Wee1	Wee1	WEE 1 homolog 1 (S. pombe)	22390	https://www.ncbi.nlm.nih.gov/gene/?term=22390
Rnf216	Rnf216	ring finger protein 216	108086	https://www.ncbi.nlm.nih.gov/gene/?term=108086
Usp8	Usp8	ubiquitin specific peptidase 8	84092	https://www.ncbi.nlm.nih.gov/gene/?term=84092
Adcy1	Adcy1	adenylate cyclase 1	432530	https://www.ncbi.nlm.nih.gov/gene/?term=432530
Prkd3	Prkd3	protein kinase D3	75292	https://www.ncbi.nlm.nih.gov/gene/?term=75292
Sh3d19	Sh3d19	SH3 domain protein D19	27059	https://www.ncbi.nlm.nih.gov/gene/?term=27059
Ago4	Ago4	argonaute RISC catalytic subunit 4	76850	https://www.ncbi.nlm.nih.gov/gene/?term=76850
Casd1	Casd1	CAS1 domain containing 1	213819	https://www.ncbi.nlm.nih.gov/gene/?term=213819

Cnot7	Cnot7	CCR4-NOT transcription complex, subunit 7	18983	https://www.ncbi.nlm.nih.gov/gene/?term=18983
Lonrf3	Lonrf3	LON peptidase N-terminal domain and ring finger 3	74365	https://www.ncbi.nlm.nih.gov/gene/?term=74365
Btbd7	Btbd7	BTB (POZ) domain containing 7	238386	https://www.ncbi.nlm.nih.gov/gene/?term=238386
Tmem159	Tmem159	transmembrane protein 159	233806	https://www.ncbi.nlm.nih.gov/gene/?term=233806
Lrrtm2	Lrrtm2	leucine rich repeat transmembrane neuronal 2	107065	https://www.ncbi.nlm.nih.gov/gene/?term=107065
Akap11	Akap11	A kinase (PRKA) anchor protein 11	219181	https://www.ncbi.nlm.nih.gov/gene/?term=219181
Mid1ip1	Mid1ip1	Mid1 interacting protein 1 (gastrulation specific G12-like (zebrafish))	68041	https://www.ncbi.nlm.nih.gov/gene/?term=68041
Lrp4	Lrp4	low density lipoprotein receptor-related protein 4	228357	https://www.ncbi.nlm.nih.gov/gene/?term=228357
Erbin	Erbin	Erbb2 interacting protein	59079	https://www.ncbi.nlm.nih.gov/gene/?term=59079
Tbc1d8	Tbc1d8	TBC1 domain family, member 8	54610	https://www.ncbi.nlm.nih.gov/gene/?term=54610
Sowahb	Sowahb	sosondowah ankyrin repeat domain family member B	78088	https://www.ncbi.nlm.nih.gov/gene/?term=78088
Sel1l3	Sel1l3	sel-1 suppressor of lin-12-like 3 (C. elegans)	231238	https://www.ncbi.nlm.nih.gov/gene/?term=231238
Fibin	Fibin	fin bud initiation factor homolog (zebrafish)	67606	https://www.ncbi.nlm.nih.gov/gene/?term=67606
Dcbld2	Dcbld2	discoidin, CUB and LCCL domain containing 2	73379	https://www.ncbi.nlm.nih.gov/gene/?term=73379
Memo1	Memo1	mediator of cell motility 1	76890	https://www.ncbi.nlm.nih.gov/gene/?term=76890
Ston2	Ston2	stonin 2	108800	https://www.ncbi.nlm.nih.gov/gene/?term=108800
Nefl	Nefl	neurofilament, light polypeptide	18039	https://www.ncbi.nlm.nih.gov/gene/?term=18039
Mitd1	Mitd1	MIT, microtubule interacting and transport, domain containing 1	69028	https://www.ncbi.nlm.nih.gov/gene/?term=69028

Biology Open • Supplementary information

Lrrc17	Lrrc17	leucine rich repeat containing 17	74511	https://www.ncbi.nlm.nih.gov/gene/?term=74511
Ubn2	Ubn2	ubinuclein 2	320538	https://www.ncbi.nlm.nih.gov/gene/?term=320538
Ptpn2	Ptpn2	protein tyrosine phosphatase, non-receptor type 2	19255	https://www.ncbi.nlm.nih.gov/gene/?term=19255
4933409G03Rik	4933409G03Rik	RIKEN cDNA 4933409G03 gene	227998	https://www.ncbi.nlm.nih.gov/gene/?term=227998
Fam83f	Fam83f	family with sequence similarity 83, member F	213956	https://www.ncbi.nlm.nih.gov/gene/?term=213956
Fst	Fst	follistatin	14313	https://www.ncbi.nlm.nih.gov/gene/?term=14313
Lipi	Lipi	lipase, member I	320355	https://www.ncbi.nlm.nih.gov/gene/?term=320355
Actr1a	Actr1a	ARP1 actin-related protein 1A, centractin alpha	54130	https://www.ncbi.nlm.nih.gov/gene/?term=54130
Abcd2	Abcd2	ATP-binding cassette, sub-family D (ALD), member 2	26874	https://www.ncbi.nlm.nih.gov/gene/?term=26874
Terf1	Terf1	telomeric repeat binding factor 1	21749	https://www.ncbi.nlm.nih.gov/gene/?term=21749
Znrf1	Znrf1	zinc and ring finger 1	170737	https://www.ncbi.nlm.nih.gov/gene/?term=170737
Atp6v0d1	Atp6v0d1	ATPase, H+ transporting, lysosomal V0 subunit D1	11972	https://www.ncbi.nlm.nih.gov/gene/?term=11972
Exoc6	Exoc6	exocyst complex component 6	107371	https://www.ncbi.nlm.nih.gov/gene/?term=107371
Tcfl5	Tcfl5	transcription factor-like 5 (basic helix-loop-helix)	277353	https://www.ncbi.nlm.nih.gov/gene/?term=277353
Taok1	Taok1	TAO kinase 1	216965	https://www.ncbi.nlm.nih.gov/gene/?term=216965
Stim2	Stim2	stromal interaction molecule 2	116873	https://www.ncbi.nlm.nih.gov/gene/?term=116873
Glcci1	Glcci1	glucocorticoid induced transcript 1	170772	https://www.ncbi.nlm.nih.gov/gene/?term=170772
Limch1	Limch1	LIM and calponin homology domains 1	77569	https://www.ncbi.nlm.nih.gov/gene/?term=77569
Pcdh17	Pcdh17	protocadherin 17	219228	https://www.ncbi.nlm.nih.gov/gene/?term=219228
Stk35	Stk35	serine/threonine kinase 35	67333	https://www.ncbi.nlm.nih.gov/gene/?term=67333
Cfap61	Cfap61	cilia and flagella associated protein 61	78774	https://www.ncbi.nlm.nih.gov/gene/?term=78774

Dlg5	Dlg5	discs large MAGUK scaffold protein 5	71228	https://www.ncbi.nlm.nih.gov/gene/?term=71228
Khnyn	Khnyn	KH and NYN domain containing	219094	https://www.ncbi.nlm.nih.gov/gene/?term=219094
Pdss1	Pdss1	prenyl (solanesyl) diphosphate synthase, subunit 1	56075	https://www.ncbi.nlm.nih.gov/gene/?term=56075
Rnf157	Rnf157	ring finger protein 157	217340	https://www.ncbi.nlm.nih.gov/gene/?term=217340
Gldc	Gldc	glycine decarboxylase	104174	https://www.ncbi.nlm.nih.gov/gene/?term=104174
Tfdp1	Tfdp1	transcription factor Dp 1	21781	https://www.ncbi.nlm.nih.gov/gene/?term=21781
Grm3	Grm3	glutamate receptor, metabotropic 3	108069	https://www.ncbi.nlm.nih.gov/gene/?term=108069
Tcp11l2	Tcp11l2	t-complex 11 (mouse) like 2	216198	https://www.ncbi.nlm.nih.gov/gene/?term=216198
Ppp1r18	Ppp1r18	protein phosphatase 1, regulatory subunit 18	76448	https://www.ncbi.nlm.nih.gov/gene/?term=76448
Galnt7	Galnt7	polypeptide N-acetylgalactosaminyltransferase 7	108150	https://www.ncbi.nlm.nih.gov/gene/?term=108150
Htr4	Htr4	5 hydroxytryptamine (serotonin) receptor 4	15562	https://www.ncbi.nlm.nih.gov/gene/?term=15562
Ppp2r1b	Ppp2r1b	protein phosphatase 2, regulatory subunit A, beta	73699	https://www.ncbi.nlm.nih.gov/gene/?term=73699
Mkrn3	Mkrn3	makorin, ring finger protein, 3	22652	https://www.ncbi.nlm.nih.gov/gene/?term=22652
Picalm	Picalm	phosphatidylinositol binding clathrin assembly protein	233489	https://www.ncbi.nlm.nih.gov/gene/?term=233489
Nt5e	Nt5e	5' nucleotidase, ecto	23959	https://www.ncbi.nlm.nih.gov/gene/?term=23959
Sema6b	Sema6b	sema domain, transmembrane domain (TM), and cytoplasmic domain, (semaphorin) 6B	20359	https://www.ncbi.nlm.nih.gov/gene/?term=20359
Adamts3	Adamts3	a disintegrin-like and metallopeptidase (reprolysin type) with thrombospondin type 1 motif, 3	330119	https://www.ncbi.nlm.nih.gov/gene/?term=330119
Shoc2	Shoc2	Shoc2, leucine rich repeat scaffold protein	56392	https://www.ncbi.nlm.nih.gov/gene/?term=56392

Biology Open • Supplementary information

Adra2a	Adra2a	adrenergic receptor, alpha 2a	11551	https://www.ncbi.nlm.nih.gov/gene/?term=11551
Asah1	Asah1	N-acylsphingosine amidohydrolase 1	11886	https://www.ncbi.nlm.nih.gov/gene/?term=11886
Snapin	Snapin	SNAP-associated protein	20615	https://www.ncbi.nlm.nih.gov/gene/?term=20615
Capza1	Capza1	capping protein (actin filament) muscle Z-line, alpha 1	12340	https://www.ncbi.nlm.nih.gov/gene/?term=12340
Abcc9	Abcc9	ATP-binding cassette, sub-family C (CFTR/MRP), member 9	20928	https://www.ncbi.nlm.nih.gov/gene/?term=20928
Siah2	Siah2	siah E3 ubiquitin protein ligase 2	20439	https://www.ncbi.nlm.nih.gov/gene/?term=20439
Sox9	Sox9	SRY (sex determining region Y)-box 9	20682	https://www.ncbi.nlm.nih.gov/gene/?term=20682
Chl1	Chl1	cell adhesion molecule L1-like	12661	https://www.ncbi.nlm.nih.gov/gene/?term=12661
R3hdm1	R3hdm1	R3H domain containing 1	226412	https://www.ncbi.nlm.nih.gov/gene/?term=226412
Nap1l5	Nap1l5	nucleosome assembly protein 1-like 5	58243	https://www.ncbi.nlm.nih.gov/gene/?term=58243
Csnk1a1	Csnk1a1	casein kinase 1, alpha 1	93687	https://www.ncbi.nlm.nih.gov/gene/?term=93687
Runx2	Runx2	runt related transcription factor 2	12393	https://www.ncbi.nlm.nih.gov/gene/?term=12393
Fbxo45	Fbxo45	F-box protein 45	268882	https://www.ncbi.nlm.nih.gov/gene/?term=268882
Slc36a1	Slc36a1	solute carrier family 36 (proton/amino acid symporter), member 1	215335	https://www.ncbi.nlm.nih.gov/gene/?term=215335
Impact	Impact	impact, RWD domain protein	16210	https://www.ncbi.nlm.nih.gov/gene/?term=16210
Cysltr1	Cysltr1	cysteinyl leukotriene receptor 1	58861	https://www.ncbi.nlm.nih.gov/gene/?term=58861
Srsf7	Srsf7	serine/arginine-rich splicing factor 7	225027	https://www.ncbi.nlm.nih.gov/gene/?term=225027
Polr3e	Polr3e	polymerase (RNA) III (DNA directed) polypeptide E	26939	https://www.ncbi.nlm.nih.gov/gene/?term=26939
Ube2v2	Ube2v2	ubiquitin-conjugating enzyme E2 variant 2	70620	https://www.ncbi.nlm.nih.gov/gene/?term=70620
Ip6k3	Ip6k3	inositol hexaphosphate kinase 3	271424	https://www.ncbi.nlm.nih.gov/gene/?term=271424
Gjc2	Gjc2	gap junction protein, gamma 2	118454	https://www.ncbi.nlm.nih.gov/gene/?term=118454
Afap1l2	Afap1l2	actin filament associated protein 1-	226250	https://www.ncbi.nlm.nih.gov/gene/?term=226250

Biology Open • Supplementary information

		like 2		
Scn9a	Scn9a	sodium channel, voltage-gated, type IX, alpha	20274	https://www.ncbi.nlm.nih.gov/gene/?term=20274
Mcf2l	Mcf2l	mcf.2 transforming sequence-like	17207	https://www.ncbi.nlm.nih.gov/gene/?term=17207
Map3k5	Map3k5	mitogen-activated protein kinase kinase kinase 5	26408	https://www.ncbi.nlm.nih.gov/gene/?term=26408
Ano4	Ano4	anoctamin 4	320091	https://www.ncbi.nlm.nih.gov/gene/?term=320091
Samd8	Samd8	sterile alpha motif domain containing 8	67630	https://www.ncbi.nlm.nih.gov/gene/?term=67630
Slc7a10	Slc7a10	solute carrier family 7 (cationic amino acid transporter, y+ system), member 10	53896	https://www.ncbi.nlm.nih.gov/gene/?term=53896
Ankhd1	Ankhd1	ankyrin repeat and KH domain containing 1	108857	https://www.ncbi.nlm.nih.gov/gene/?term=108857
Cadps	Cadps	Ca2+-dependent secretion activator	27062	https://www.ncbi.nlm.nih.gov/gene/?term=27062
Scara5	Scara5	scavenger receptor class A, member 5	71145	https://www.ncbi.nlm.nih.gov/gene/?term=71145
Rfx6	Rfx6	regulatory factor X, 6	320995	https://www.ncbi.nlm.nih.gov/gene/?term=320995
Foxg1	Foxg1	forkhead box G1	15228	https://www.ncbi.nlm.nih.gov/gene/?term=15228
Setd5	Setd5	SET domain containing 5	72895	https://www.ncbi.nlm.nih.gov/gene/?term=72895
Socs3	Socs3	suppressor of cytokine signaling 3	12702	https://www.ncbi.nlm.nih.gov/gene/?term=12702
Ago3	Ago3	argonaute RISC catalytic subunit 3	214150	https://www.ncbi.nlm.nih.gov/gene/?term=214150
Actc1	Actc1	actin, alpha, cardiac muscle 1	11464	https://www.ncbi.nlm.nih.gov/gene/?term=11464
Rgs8	Rgs8	regulator of G-protein signaling 8	67792	https://www.ncbi.nlm.nih.gov/gene/?term=67792
Tbc1d15	Tbc1d15	TBC1 domain family, member 15	66687	https://www.ncbi.nlm.nih.gov/gene/?term=66687
Capn7	Capn7	calpain 7	12339	https://www.ncbi.nlm.nih.gov/gene/?term=12339
Fbxo32	Fbxo32	F-box protein 32	67731	https://www.ncbi.nlm.nih.gov/gene/?term=67731
Cnot9	Cnot9	CCR4-NOT transcription complex, subunit 9	58184	https://www.ncbi.nlm.nih.gov/gene/?term=58184

Biology Open • Supplementary information

Ednra	Ednra	endothelin receptor type A	13617	https://www.ncbi.nlm.nih.gov/gene/?term=13617
Atg3	Atg3	autophagy related 3	67841	https://www.ncbi.nlm.nih.gov/gene/?term=67841
Zcchc2	Zcchc2	zinc finger, CCHC domain containing 2	227449	https://www.ncbi.nlm.nih.gov/gene/?term=227449
Ifi213	Ifi213	interferon activated gene 213	623121	https://www.ncbi.nlm.nih.gov/gene/?term=623121
Ankrd55	Ankrd55	ankyrin repeat domain 55	77318	https://www.ncbi.nlm.nih.gov/gene/?term=77318
Ttbk1	Ttbk1	tau tubulin kinase 1	106763	https://www.ncbi.nlm.nih.gov/gene/?term=106763
Csnk1g1	Csnk1g1	casein kinase 1, gamma 1	214897	https://www.ncbi.nlm.nih.gov/gene/?term=214897
Ppid	Ppid	peptidylprolyl isomerase D (cyclophilin D)	67738	https://www.ncbi.nlm.nih.gov/gene/?term=67738
Ror1	Ror1	receptor tyrosine kinase-like orphan receptor 1	26563	https://www.ncbi.nlm.nih.gov/gene/?term=26563
Chd1	Chd1	chromodomain helicase DNA binding protein 1	12648	https://www.ncbi.nlm.nih.gov/gene/?term=12648
Frmpd1	Frmpd1	FERM and PDZ domain containing 1	666060	https://www.ncbi.nlm.nih.gov/gene/?term=666060
Gzf1	Gzf1	GDNF-inducible zinc finger protein 1	74533	https://www.ncbi.nlm.nih.gov/gene/?term=74533
Ppargc1b	Ppargc1b	peroxisome proliferative activated receptor, gamma, coactivator 1 beta	170826	https://www.ncbi.nlm.nih.gov/gene/?term=170826
Ythdf3	Ythdf3	YTH N6-methyladenosine RNA binding protein 3	229096	https://www.ncbi.nlm.nih.gov/gene/?term=229096
Map3k21	Map3k21	mitogen-activated protein kinase kinase kinase 21	234878	https://www.ncbi.nlm.nih.gov/gene/?term=234878
Rai14	Rai14	retinoic acid induced 14	75646	https://www.ncbi.nlm.nih.gov/gene/?term=75646
Slc38a7	Slc38a7	solute carrier family 38, member 7	234595	https://www.ncbi.nlm.nih.gov/gene/?term=234595
Pde7a	Pde7a	phosphodiesterase 7A	18583	https://www.ncbi.nlm.nih.gov/gene/?term=18583
Ppp3ca	Ppp3ca	protein phosphatase 3, catalytic subunit, alpha isoform	19055	https://www.ncbi.nlm.nih.gov/gene/?term=19055
Extl2	Extl2	exostoses (multiple)-like 2	58193	https://www.ncbi.nlm.nih.gov/gene/?term=58193
Sh3rf1	Sh3rf1	SH3 domain containing ring finger 1	59009	https://www.ncbi.nlm.nih.gov/gene/?term=59009

Biology Open • Supplementary information

Erich5	Erich5	glutamate rich 5	239368	https://www.ncbi.nlm.nih.gov/gene/?term=239368
Jph4	Jph4	junctophilin 4	319984	https://www.ncbi.nlm.nih.gov/gene/?term=319984
Tbl1xr1	Tbl1xr1	transducin (beta)-like 1X-linked receptor 1	81004	https://www.ncbi.nlm.nih.gov/gene/?term=81004
Katnbl1	Katnbl1	katanin p80 subunit B like 1	72425	https://www.ncbi.nlm.nih.gov/gene/?term=72425
Xpo1	Xpo1	exportin 1	103573	https://www.ncbi.nlm.nih.gov/gene/?term=103573
C9orf72	C9orf72	C9orf72, member of C9orf72-SMCR8 complex	73205	https://www.ncbi.nlm.nih.gov/gene/?term=73205
Pip4k2a	Pip4k2a	phosphatidylinositol-5-phosphate 4-kinase, type II, alpha	18718	https://www.ncbi.nlm.nih.gov/gene/?term=18718
Tbc1d10b	Tbc1d10b	TBC1 domain family, member 10b	68449	https://www.ncbi.nlm.nih.gov/gene/?term=68449
Rasgef1a	Rasgef1a	RasGEF domain family, member 1A	70727	https://www.ncbi.nlm.nih.gov/gene/?term=70727
Klf9	Klf9	Kruppel-like factor 9	16601	https://www.ncbi.nlm.nih.gov/gene/?term=16601
Rimbp2	Rimbp2	RIMS binding protein 2	231760	https://www.ncbi.nlm.nih.gov/gene/?term=231760
Necap1	Necap1	NECAP endocytosis associated 1	67602	https://www.ncbi.nlm.nih.gov/gene/?term=67602
Elavl2	Elavl2	ELAV like RNA binding protein 1	15569	https://www.ncbi.nlm.nih.gov/gene/?term=15569
Slc35d3	Slc35d3	solute carrier family 35, member D3	76157	https://www.ncbi.nlm.nih.gov/gene/?term=76157
Slc4a7	Slc4a7	solute carrier family 4, sodium bicarbonate cotransporter, member 7	218756	https://www.ncbi.nlm.nih.gov/gene/?term=218756
Lrrk2	Lrrk2	leucine-rich repeat kinase 2	66725	https://www.ncbi.nlm.nih.gov/gene/?term=66725
Tmod2	Tmod2	tropomodulin 2	50876	https://www.ncbi.nlm.nih.gov/gene/?term=50876
Wdr7	Wdr7	WD repeat domain 7	104082	https://www.ncbi.nlm.nih.gov/gene/?term=104082
Sptssb	Sptssb	serine palmitoyltransferase, small subunit B	66183	https://www.ncbi.nlm.nih.gov/gene/?term=66183
Ccnt2	Ccnt2	cyclin T2	72949	https://www.ncbi.nlm.nih.gov/gene/?term=72949
Dll4	Dll4	delta like canonical Notch ligand 4	54485	https://www.ncbi.nlm.nih.gov/gene/?term=54485
Cdc37l1	Cdc37l1	cell division cycle 37-like 1	67072	https://www.ncbi.nlm.nih.gov/gene/?term=67072
Tecrl	Tecrl	trans-2,3-enoyl-CoA reductase-like	243078	https://www.ncbi.nlm.nih.gov/gene/?term=243078

Biology Open • Supplementary information

Rapgef2	Rapgef2	Rap guanine nucleotide exchange factor (GEF) 2	76089	https://www.ncbi.nlm.nih.gov/gene/?term=76089
Gigyf2	Gigyf2	GRB10 interacting GYF protein 2	227331	https://www.ncbi.nlm.nih.gov/gene/?term=227331
Avl9	Avl9	AVL9 cell migration associated	78937	https://www.ncbi.nlm.nih.gov/gene/?term=78937
Rnf122	Rnf122	ring finger protein 122	68867	https://www.ncbi.nlm.nih.gov/gene/?term=68867
Eed	Eed	embryonic ectoderm development	13626	https://www.ncbi.nlm.nih.gov/gene/?term=13626
Gria2	Gria2	glutamate receptor, ionotropic, AMPA2 (alpha 2)	14800	https://www.ncbi.nlm.nih.gov/gene/?term=14800
Myh11	Myh11	myosin, heavy polypeptide 11, smooth muscle	17880	https://www.ncbi.nlm.nih.gov/gene/?term=17880
Rab32	Rab32	RAB32, member RAS oncogene family	67844	https://www.ncbi.nlm.nih.gov/gene/?term=67844
Gabrb1	Gabrb1	gamma-aminobutyric acid (GABA) A receptor, subunit beta 1	14400	https://www.ncbi.nlm.nih.gov/gene/?term=14400
Ascc3	Ascc3	activating signal cointegrator 1 complex subunit 3	77987	https://www.ncbi.nlm.nih.gov/gene/?term=77987
Klf8	Klf8	Kruppel-like factor 8	245671	https://www.ncbi.nlm.nih.gov/gene/?term=245671
Ankra2	Ankra2	ankyrin repeat, family A (RFXANK-like), 2	68558	https://www.ncbi.nlm.nih.gov/gene/?term=68558
Erlin1	Erlin1	ER lipid raft associated 1	226144	https://www.ncbi.nlm.nih.gov/gene/?term=226144
Zfp507	Zfp507	zinc finger protein 507	668501	https://www.ncbi.nlm.nih.gov/gene/?term=668501
Hnrnpul2	Hnrnpul2	heterogeneous nuclear ribonucleoprotein U-like 2	68693	https://www.ncbi.nlm.nih.gov/gene/?term=68693
Skp2	Skp2	S-phase kinase-associated protein 2 (p45)	27401	https://www.ncbi.nlm.nih.gov/gene/?term=27401
Klf10	Klf10	Kruppel-like factor 10	21847	https://www.ncbi.nlm.nih.gov/gene/?term=21847
Bnc1	Bnc1	basonuclin 1	12173	https://www.ncbi.nlm.nih.gov/gene/?term=12173
Zfp608	Zfp608	zinc finger protein 608	269023	https://www.ncbi.nlm.nih.gov/gene/?term=269023
Vat1	Vat1	vesicle amine transport 1	26949	https://www.ncbi.nlm.nih.gov/gene/?term=26949

Biology Open • Supplementary information

Snx33	Snx33	sorting nexin 33	235406	https://www.ncbi.nlm.nih.gov/gene/?term=235406
Prlr	Prlr	prolactin receptor	19116	https://www.ncbi.nlm.nih.gov/gene/?term=19116
Rundc3b	Rundc3b	RUN domain containing 3B	242819	https://www.ncbi.nlm.nih.gov/gene/?term=242819
Gnai2	Gnai2	guanine nucleotide binding protein (G protein), alpha inhibiting 2	14678	https://www.ncbi.nlm.nih.gov/gene/?term=14678
Socs1	Socs1	suppressor of cytokine signaling 1	12703	https://www.ncbi.nlm.nih.gov/gene/?term=12703
Plch1	Plch1	phospholipase C, eta 1	269437	https://www.ncbi.nlm.nih.gov/gene/?term=269437
Snx18	Snx18	sorting nexin 18	170625	https://www.ncbi.nlm.nih.gov/gene/?term=170625
Zfp518a	Zfp518a	zinc finger protein 518A	72672	https://www.ncbi.nlm.nih.gov/gene/?term=72672
Slc15a2	Slc15a2	solute carrier family 15 (H+/peptide transporter), member 2	57738	https://www.ncbi.nlm.nih.gov/gene/?term=57738
Gabra5	Gabra5	gamma-aminobutyric acid (GABA) A receptor, subunit alpha 5	110886	https://www.ncbi.nlm.nih.gov/gene/?term=110886
Rrad	Rrad	Ras-related associated with diabetes	56437	https://www.ncbi.nlm.nih.gov/gene/?term=56437
Hdac9	Hdac9	histone deacetylase 9	79221	https://www.ncbi.nlm.nih.gov/gene/?term=79221
Prps1l3	Prps1l3	phosphoribosyl pyrophosphate synthetase 1-like 3	328099	https://www.ncbi.nlm.nih.gov/gene/?term=328099
Twf1	Twf1	twinfilin actin binding protein 1	19230	https://www.ncbi.nlm.nih.gov/gene/?term=19230
Zdhhc20	Zdhhc20	zinc finger, DHHC domain containing 20	75965	https://www.ncbi.nlm.nih.gov/gene/?term=75965
Arhgef6	Arhgef6	Rac/Cdc42 guanine nucleotide exchange factor (GEF) 6	73341	https://www.ncbi.nlm.nih.gov/gene/?term=73341
Slc35c1	Slc35c1	solute carrier family 35, member C1	228368	https://www.ncbi.nlm.nih.gov/gene/?term=228368
Chst2	Chst2	carbohydrate sulfotransferase 2	54371	https://www.ncbi.nlm.nih.gov/gene/?term=54371
Sdad1	Sdad1	SDA1 domain containing 1	231452	https://www.ncbi.nlm.nih.gov/gene/?term=231452
Pik3cd	Pik3cd	phosphatidylinositol-4,5-bisphosphate 3-kinase catalytic subunit delta	18707	https://www.ncbi.nlm.nih.gov/gene/?term=18707

Biology Open • Supplementary information

Ttll7	Ttll7	tubulin tyrosine ligase-like family, member 7	70892	https://www.ncbi.nlm.nih.gov/gene/?term=70892
Col13a1	Col13a1	collagen, type XIII, alpha 1	12817	https://www.ncbi.nlm.nih.gov/gene/?term=12817
Zfp560	Zfp560	zinc finger protein 560	434377	https://www.ncbi.nlm.nih.gov/gene/?term=434377
Vopp1	Vopp1	vesicular, overexpressed in cancer, prosurvival protein 1	232023	https://www.ncbi.nlm.nih.gov/gene/?term=232023
Lrfn2	Lrfn2	leucine rich repeat and fibronectin type III domain containing 2	70530	https://www.ncbi.nlm.nih.gov/gene/?term=70530
Plagl2	Plagl2	pleiomorphic adenoma gene-like 2	54711	https://www.ncbi.nlm.nih.gov/gene/?term=54711
Myo9b	Myo9b	myosin IXb	17925	https://www.ncbi.nlm.nih.gov/gene/?term=17925
Gtf2h4	Gtf2h4	general transcription factor II H, polypeptide 4	14885	https://www.ncbi.nlm.nih.gov/gene/?term=14885
Asb3	Asb3	ankyrin repeat and SOCS box-containing 3	65257	https://www.ncbi.nlm.nih.gov/gene/?term=65257
Trip12	Trip12	thyroid hormone receptor interactor 12	14897	https://www.ncbi.nlm.nih.gov/gene/?term=14897
Tmem181a	Tmem181a	transmembrane protein 181A	77106	https://www.ncbi.nlm.nih.gov/gene/?term=77106
Ptpn13	Ptpn13	protein tyrosine phosphatase, non-receptor type 13	19249	https://www.ncbi.nlm.nih.gov/gene/?term=19249
Celsr3	Celsr3	cadherin, EGF LAG seven-pass G-type receptor 3	107934	https://www.ncbi.nlm.nih.gov/gene/?term=107934
Cyp24a1	Cyp24a1	cytochrome P450, family 24, subfamily a, polypeptide 1	13081	https://www.ncbi.nlm.nih.gov/gene/?term=13081
Scn2a	Scn2a	sodium channel, voltage-gated, type II, alpha	110876	https://www.ncbi.nlm.nih.gov/gene/?term=110876
Rnf220	Rnf220	ring finger protein 220	66743	https://www.ncbi.nlm.nih.gov/gene/?term=66743
Mbtps2	Mbtps2	membrane-bound transcription factor peptidase, site 2	270669	https://www.ncbi.nlm.nih.gov/gene/?term=270669
Rab38	Rab38	RAB38, member RAS oncogene	72433	https://www.ncbi.nlm.nih.gov/gene/?term=72433

Biology Open • Supplementary information

		family		
Ddah1	Ddah1	dimethylarginine dimethylaminohydrolase 1	69219	https://www.ncbi.nlm.nih.gov/gene/?term=69219
Pawr	Pawr	PRKC, apoptosis, WT1, regulator	114774	https://www.ncbi.nlm.nih.gov/gene/?term=114774
Six1	Six1	sine oculis-related homeobox 1	20471	https://www.ncbi.nlm.nih.gov/gene/?term=20471
Arid5b	Arid5b	AT rich interactive domain 5B (MRF1-like)	71371	https://www.ncbi.nlm.nih.gov/gene/?term=71371
Frzb	Frzb	frizzled-related protein	20378	https://www.ncbi.nlm.nih.gov/gene/?term=20378
9930021J03Rik	9930021J03Rik	RIKEN cDNA 9930021J03 gene	240613	https://www.ncbi.nlm.nih.gov/gene/?term=240613
Dolpp1	Dolpp1	dolichyl pyrophosphate phosphatase 1	57170	https://www.ncbi.nlm.nih.gov/gene/?term=57170
Eml1	Eml1	echinoderm microtubule associated protein like 1	68519	https://www.ncbi.nlm.nih.gov/gene/?term=68519
Adgra3	Adgra3	adhesion G protein-coupled receptor A3	70693	https://www.ncbi.nlm.nih.gov/gene/?term=70693
Ppp1r12a	Ppp1r12a	protein phosphatase 1, regulatory subunit 12A	17931	https://www.ncbi.nlm.nih.gov/gene/?term=17931
Ypel5	Ypel5	yippee like 5	383295	https://www.ncbi.nlm.nih.gov/gene/?term=383295
Esyt3	Esyt3	extended synaptotagmin-like protein 3	272636	https://www.ncbi.nlm.nih.gov/gene/?term=272636
Reep1	Reep1	receptor accessory protein 1	52250	https://www.ncbi.nlm.nih.gov/gene/?term=52250
Terb2	Terb2	telomere repeat binding bouquet formation protein 2	74401	https://www.ncbi.nlm.nih.gov/gene/?term=74401
B3gnt5	B3gnt5	UDP-GlcNAc:betaGal beta-1,3-N-acetylglucosaminyltransferase 5	108105	https://www.ncbi.nlm.nih.gov/gene/?term=108105
Brcc3	Brcc3	BRCA1/BRCA2-containing complex, subunit 3	210766	https://www.ncbi.nlm.nih.gov/gene/?term=210766
Steap2	Steap2	six transmembrane epithelial antigen of prostate 2	74051	https://www.ncbi.nlm.nih.gov/gene/?term=74051

Sp4	Sp4	trans-acting transcription factor 4	20688	https://www.ncbi.nlm.nih.gov/gene/?term=20688
Smarce1	Smarce1	SWI/SNF related, matrix associated, actin dependent regulator of chromatin, subfamily e, member 1	57376	https://www.ncbi.nlm.nih.gov/gene/?term=57376
Plp1	Plp1	proteolipid protein (myelin) 1	18823	https://www.ncbi.nlm.nih.gov/gene/?term=18823
Rasgef1b	Rasgef1b	RasGEF domain family, member 1B	320292	https://www.ncbi.nlm.nih.gov/gene/?term=320292
Rab27b	Rab27b	RAB27B, member RAS oncogene family	80718	https://www.ncbi.nlm.nih.gov/gene/?term=80718
Klhl34	Klhl34	kelch-like 34	245683	https://www.ncbi.nlm.nih.gov/gene/?term=245683
Pkn2	Pkn2	protein kinase N2	109333	https://www.ncbi.nlm.nih.gov/gene/?term=109333
Ccdc88a	Ccdc88a	coiled coil domain containing 88A	108686	https://www.ncbi.nlm.nih.gov/gene/?term=108686
Far2	Far2	fatty acyl CoA reductase 2	330450	https://www.ncbi.nlm.nih.gov/gene/?term=330450
Olfr1033	Olfr1033	olfactory receptor 1033	258571	https://www.ncbi.nlm.nih.gov/gene/?term=258571
Sult3a1	Sult3a1	sulfotransferase family 3A, member 1	57430	https://www.ncbi.nlm.nih.gov/gene/?term=57430
Acbd5	Acbd5	acyl-Coenzyme A binding domain containing 5	74159	https://www.ncbi.nlm.nih.gov/gene/?term=74159
Pitx2	Pitx2	paired-like homeodomain transcription factor 2	18741	https://www.ncbi.nlm.nih.gov/gene/?term=18741
Stxbp5l	Stxbp5l	syntaxin binding protein 5-like	207227	https://www.ncbi.nlm.nih.gov/gene/?term=207227
Nxpe4	Nxpe4	neurexophilin and PC-esterase domain family, member 4	244853	https://www.ncbi.nlm.nih.gov/gene/?term=244853
Neurog1	Neurog1	neurogenin 1	18014	https://www.ncbi.nlm.nih.gov/gene/?term=18014
Ryr2	Ryr2	ryanodine receptor 2, cardiac	20191	https://www.ncbi.nlm.nih.gov/gene/?term=20191
Angptl1	Angptl1	angiopoietin-like 1	72713	https://www.ncbi.nlm.nih.gov/gene/?term=72713
Etv1	Etv1	ets variant 1	14009	https://www.ncbi.nlm.nih.gov/gene/?term=14009
Hccs	Hccs	holocytochrome c synthetase	15159	https://www.ncbi.nlm.nih.gov/gene/?term=15159
Pclo	Pclo	piccolo (presynaptic cytomatrix protein)	26875	https://www.ncbi.nlm.nih.gov/gene/?term=26875

Biology Open • Supplementary information

Pgap1	Pgap1	post-GPI attachment to proteins 1	241062	https://www.ncbi.nlm.nih.gov/gene/?term=241062
Lrrtm3	Lrrtm3	leucine rich repeat transmembrane neuronal 3	216028	https://www.ncbi.nlm.nih.gov/gene/?term=216028
Bcor	Bcor	BCL6 interacting corepressor	71458	https://www.ncbi.nlm.nih.gov/gene/?term=71458
Tmed5	Tmed5	transmembrane p24 trafficking protein 5	73130	https://www.ncbi.nlm.nih.gov/gene/?term=73130
Ank3	Ank3	ankyrin 3, epithelial	11735	https://www.ncbi.nlm.nih.gov/gene/?term=11735
Runx1t1	Runx1t1	RUNX1 translocation partner 1	12395	https://www.ncbi.nlm.nih.gov/gene/?term=12395
Mageb16	Mageb16	melanoma antigen family B, 16	71967	https://www.ncbi.nlm.nih.gov/gene/?term=71967
Abi1	Abi1	abl-interactor 1	11308	https://www.ncbi.nlm.nih.gov/gene/?term=11308
Ppat	Ppat	phosphoribosyl pyrophosphate amidotransferase	231327	https://www.ncbi.nlm.nih.gov/gene/?term=231327
Jade1	Jade1	jade family PHD finger 1	269424	https://www.ncbi.nlm.nih.gov/gene/?term=269424
Zfp616	Zfp616	zinc finger protein 616	327963	https://www.ncbi.nlm.nih.gov/gene/?term=327963
Enpp2	Enpp2	ectonucleotide pyrophosphatase/phosphodiesterase 2	18606	https://www.ncbi.nlm.nih.gov/gene/?term=18606
Tshz1	Tshz1	teashirt zinc finger family member 1	110796	https://www.ncbi.nlm.nih.gov/gene/?term=110796
Lrp11	Lrp11	low density lipoprotein receptor-related protein 11	237253	https://www.ncbi.nlm.nih.gov/gene/?term=237253
Nt5m	Nt5m	5',3'-nucleotidase, mitochondrial	103850	https://www.ncbi.nlm.nih.gov/gene/?term=103850
Homer1	Homer1	homer scaffolding protein 1	26556	https://www.ncbi.nlm.nih.gov/gene/?term=26556
Ippk	Ippk	inositol 1,3,4,5,6-pentakisphosphate 2-kinase	75678	https://www.ncbi.nlm.nih.gov/gene/?term=75678
Acvr2a	Acvr2a	activin receptor IIA	11480	https://www.ncbi.nlm.nih.gov/gene/?term=11480
Nars2	Nars2	asparaginyl-tRNA synthetase 2 (mitochondrial)(putative)	244141	https://www.ncbi.nlm.nih.gov/gene/?term=244141
Elac1	Elac1	elaC ribonuclease Z 1	114615	https://www.ncbi.nlm.nih.gov/gene/?term=114615
Lmo7	Lmo7	LIM domain only 7	380928	https://www.ncbi.nlm.nih.gov/gene/?term=380928

Diaph1	Diaph1	diaphanous related formin 1	13367	https://www.ncbi.nlm.nih.gov/gene/?term=13367
Papolg	Papolg	poly(A) polymerase gamma	216578	https://www.ncbi.nlm.nih.gov/gene/?term=216578
Flrt3	Flrt3	fibronectin leucine rich transmembrane protein 3	71436	https://www.ncbi.nlm.nih.gov/gene/?term=71436
Etf1	Etf1	eukaryotic translation termination factor 1	225363	https://www.ncbi.nlm.nih.gov/gene/?term=225363
Herc6	Herc6	hect domain and RLD 6	67138	https://www.ncbi.nlm.nih.gov/gene/?term=67138
Xndc1	Xndc1	Xrcc1 N-terminal domain containing 1	102443350	https://www.ncbi.nlm.nih.gov/gene/?term=102443
Pou3f2	Pou3f2	POU domain, class 3, transcription factor 2	18992	https://www.ncbi.nlm.nih.gov/gene/?term=18992
Phf24	Phf24	PHD finger protein 24	230085	https://www.ncbi.nlm.nih.gov/gene/?term=230085
Ifi202b	Ifi202b	interferon activated gene 202B	26388	https://www.ncbi.nlm.nih.gov/gene/?term=26388
Tmem30a	Tmem30a	transmembrane protein 30A	69981	https://www.ncbi.nlm.nih.gov/gene/?term=69981
Abca9	Abca9	ATP-binding cassette, sub-family A (ABC1), member 9	217262	https://www.ncbi.nlm.nih.gov/gene/?term=217262
Trp63	Trp63	transformation related protein 63	22061	https://www.ncbi.nlm.nih.gov/gene/?term=22061
Dstn	Dstn	destrin	56431	https://www.ncbi.nlm.nih.gov/gene/?term=56431
Crispld2	Crispld2	cysteine-rich secretory protein LCCL domain containing 2	78892	https://www.ncbi.nlm.nih.gov/gene/?term=78892
Zfp867	Zfp867	zinc finger protein 867	237775	https://www.ncbi.nlm.nih.gov/gene/?term=237775
Rnf14	Rnf14	ring finger protein 14	56736	https://www.ncbi.nlm.nih.gov/gene/?term=56736
Toe1	Toe1	target of EGR1, member 1 (nuclear)	68276	https://www.ncbi.nlm.nih.gov/gene/?term=68276
Rap2a	Rap2a	RAS related protein 2a	76108	https://www.ncbi.nlm.nih.gov/gene/?term=76108
Gucy1a2	Gucy1a2	guanylate cyclase 1, soluble, alpha 2	234889	https://www.ncbi.nlm.nih.gov/gene/?term=234889
Nipa1	Nipa1	non imprinted in Prader-Willi/Angelman syndrome 1 homolog (human)	233280	https://www.ncbi.nlm.nih.gov/gene/?term=233280
Ufl1	Ufl1	UFM1 specific ligase 1	67490	https://www.ncbi.nlm.nih.gov/gene/?term=67490

Mmp12	Mmp12	matrix metallopeptidase 12	17381	https://www.ncbi.nlm.nih.gov/gene/?term=17381
Pabpc1	Pabpc1	poly(A) binding protein, cytoplasmic 1	18458	https://www.ncbi.nlm.nih.gov/gene/?term=18458
Megf10	Megf10	multiple EGF-like-domains 10	70417	https://www.ncbi.nlm.nih.gov/gene/?term=70417
Agmo	Agmo	alkylglycerol monooxygenase	319660	https://www.ncbi.nlm.nih.gov/gene/?term=319660
Jcad	Jcad	junctional cadherin 5 associated	240185	https://www.ncbi.nlm.nih.gov/gene/?term=240185
Rgs7bp	Rgs7bp	regulator of G-protein signalling 7 binding protein	52882	https://www.ncbi.nlm.nih.gov/gene/?term=52882
Rap2b	Rap2b	RAP2B, member of RAS oncogene family	74012	https://www.ncbi.nlm.nih.gov/gene/?term=74012
Dscaml1	Dscaml1	DS cell adhesion molecule like 1	114873	https://www.ncbi.nlm.nih.gov/gene/?term=114873
Irf6	Irf6	interferon regulatory factor 6	54139	https://www.ncbi.nlm.nih.gov/gene/?term=54139
Ppp2r2b	Ppp2r2b	protein phosphatase 2, regulatory subunit B, beta	72930	https://www.ncbi.nlm.nih.gov/gene/?term=72930
Tmem106b	Tmem106b	transmembrane protein 106B	71900	https://www.ncbi.nlm.nih.gov/gene/?term=71900
Stc1	Stc1	stanniocalcin 1	20855	https://www.ncbi.nlm.nih.gov/gene/?term=20855
Fbxl4	Fbxl4	F-box and leucine-rich repeat protein 4	269514	https://www.ncbi.nlm.nih.gov/gene/?term=269514
Agps	Agps	alkylglycerone phosphate synthase	228061	https://www.ncbi.nlm.nih.gov/gene/?term=228061
Akap7	Akap7	A kinase (PRKA) anchor protein 7	432442	https://www.ncbi.nlm.nih.gov/gene/?term=432442
Nkain3	Nkain3	Na+/K+ transporting ATPase interacting 3	269513	https://www.ncbi.nlm.nih.gov/gene/?term=269513
Thsd7a	Thsd7a	thrombospondin, type I, domain containing 7A	330267	https://www.ncbi.nlm.nih.gov/gene/?term=330267
Ube2d2b	Ube2d2b	ubiquitin-conjugating enzyme E2D 2B	73318	https://www.ncbi.nlm.nih.gov/gene/?term=73318
Tmem47	Tmem47	transmembrane protein 47	192216	https://www.ncbi.nlm.nih.gov/gene/?term=192216
Nek1	Nek1	NIMA (never in mitosis gene a)-related expressed kinase 1	18004	https://www.ncbi.nlm.nih.gov/gene/?term=18004

Biology Open • Supplementary information

Ncoa5	Ncoa5	nuclear receptor coactivator 5	228869	https://www.ncbi.nlm.nih.gov/gene/?term=228869
Angpt1	Angpt1	angiopoietin 1	11600	https://www.ncbi.nlm.nih.gov/gene/?term=11600
Gpr85	Gpr85	G protein-coupled receptor 85	64450	https://www.ncbi.nlm.nih.gov/gene/?term=64450
Tmeff2	Tmeff2	transmembrane protein with EGF-like and two follistatin-like domains 2	56363	https://www.ncbi.nlm.nih.gov/gene/?term=56363
Atp6v1a	Atp6v1a	ATPase, H+ transporting, lysosomal V1 subunit A	11964	https://www.ncbi.nlm.nih.gov/gene/?term=11964
Msr1	Msr1	macrophage scavenger receptor 1	20288	https://www.ncbi.nlm.nih.gov/gene/?term=20288
Ces2c	Ces2c	carboxylesterase 2C	234671	https://www.ncbi.nlm.nih.gov/gene/?term=234671
Samhd1	Samhd1	SAM domain and HD domain, 1	56045	https://www.ncbi.nlm.nih.gov/gene/?term=56045
Grpr	Grpr	gastrin releasing peptide receptor	14829	https://www.ncbi.nlm.nih.gov/gene/?term=14829
Zc3h14	Zc3h14	zinc finger CCCH type containing 14	75553	https://www.ncbi.nlm.nih.gov/gene/?term=75553
Flywch1	Flywch1	FLYWCH-type zinc finger 1	224613	https://www.ncbi.nlm.nih.gov/gene/?term=224613
Myo6	Myo6	myosin VI	17920	https://www.ncbi.nlm.nih.gov/gene/?term=17920
Nup37	Nup37	nucleoporin 37	69736	https://www.ncbi.nlm.nih.gov/gene/?term=69736
Zfp605	Zfp605	zinc finger protein 605	675812	https://www.ncbi.nlm.nih.gov/gene/?term=675812
Pax8	Pax8	paired box 8	18510	https://www.ncbi.nlm.nih.gov/gene/?term=18510
Gm597	Gm597	predicted gene 597	210962	https://www.ncbi.nlm.nih.gov/gene/?term=210962
Abhd5	Abhd5	abhydrolase domain containing 5	67469	https://www.ncbi.nlm.nih.gov/gene/?term=67469
Ifi44	Ifi44	interferon-induced protein 44	99899	https://www.ncbi.nlm.nih.gov/gene/?term=99899
Slc9a2	Slc9a2	solute carrier family 9 (sodium/hydrogen exchanger), member 2	226999	https://www.ncbi.nlm.nih.gov/gene/?term=226999
Adtrp	Adtrp	androgen dependent TFPI regulating protein	109254	https://www.ncbi.nlm.nih.gov/gene/?term=109254
Mapt	Mapt	microtubule-associated protein tau	17762	https://www.ncbi.nlm.nih.gov/gene/?term=17762
Evi2	Evi2	ecotropic viral integration site 2	101488212	https://www.ncbi.nlm.nih.gov/gene/?term=101488
Cat	Cat	catalase	12359	https://www.ncbi.nlm.nih.gov/gene/?term=12359
Cd226	Cd226	CD226 antigen	225825	https://www.ncbi.nlm.nih.gov/gene/?term=225825

Biology Open • Supplementary information

Dpp7	Dpp7	dipeptidylpeptidase 7	83768	https://www.ncbi.nlm.nih.gov/gene/?term=83768
Ambra1	Ambra1	autophagy/beclin 1 regulator 1	228361	https://www.ncbi.nlm.nih.gov/gene/?term=228361
Gm7073	Gm7073	predicted gene 7073	631784	https://www.ncbi.nlm.nih.gov/gene/?term=631784
Lrfn5	Lrfn5	leucine rich repeat and fibronectin type III domain containing 5	238205	https://www.ncbi.nlm.nih.gov/gene/?term=238205
Evi2b	Evi2b	ecotropic viral integration site 2b	216984	https://www.ncbi.nlm.nih.gov/gene/?term=216984
Btbd16	Btbd16	BTB (POZ) domain containing 16	330660	https://www.ncbi.nlm.nih.gov/gene/?term=330660
Ubtf	Ubtf	upstream binding transcription factor, RNA polymerase I	21429	https://www.ncbi.nlm.nih.gov/gene/?term=21429
Golga5	Golga5	golgi autoantigen, golgin subfamily a, 5	27277	https://www.ncbi.nlm.nih.gov/gene/?term=27277
Gmcl1	Gmcl1	germ cell-less, spermatogenesis associated 1	23885	https://www.ncbi.nlm.nih.gov/gene/?term=23885
Krtap3-1	Krtap3-1	keratin associated protein 3-1	69473	https://www.ncbi.nlm.nih.gov/gene/?term=69473
Pwwp2a	Pwwp2a	PWWP domain containing 2A	70802	https://www.ncbi.nlm.nih.gov/gene/?term=70802
Tln2	Tln2	talin 2	70549	https://www.ncbi.nlm.nih.gov/gene/?term=70549
Smg1	Smg1	SMG1 homolog, phosphatidylinositol 3-kinase-related kinase (C. elegans)	233789	https://www.ncbi.nlm.nih.gov/gene/?term=233789
Camta1	Camta1	calmodulin binding transcription activator 1	100072	https://www.ncbi.nlm.nih.gov/gene/?term=100072
Ppm1l	Ppm1l	protein phosphatase 1 (formerly 2C)-like	242083	https://www.ncbi.nlm.nih.gov/gene/?term=242083
Ezh2	Ezh2	enhancer of zeste 2 polycomb repressive complex 2 subunit	14056	https://www.ncbi.nlm.nih.gov/gene/?term=14056
Slc9a9	Slc9a9	solute carrier family 9 (sodium/hydrogen exchanger), member 9	331004	https://www.ncbi.nlm.nih.gov/gene/?term=331004
Trim46	Trim46	tripartite motif-containing 46	360213	https://www.ncbi.nlm.nih.gov/gene/?term=360213

Psma1	Psma1	proteasome (prosome, macropain) subunit, alpha type 1	26440	https://www.ncbi.nlm.nih.gov/gene/?term=26440
Zfp790	Zfp790	zinc finger protein 790	233056	https://www.ncbi.nlm.nih.gov/gene/?term=233056
Srsf11	Srsf11	serine/arginine-rich splicing factor 11	69207	https://www.ncbi.nlm.nih.gov/gene/?term=69207
Ptpre	Ptpre	protein tyrosine phosphatase, receptor type, E	19267	https://www.ncbi.nlm.nih.gov/gene/?term=19267
Gabrg2	Gabrg2	gamma-aminobutyric acid (GABA) A receptor, subunit gamma 2	14406	https://www.ncbi.nlm.nih.gov/gene/?term=14406
Smg7	Smg7	Smg-7 homolog, nonsense mediated mRNA decay factor (C. elegans)	226517	https://www.ncbi.nlm.nih.gov/gene/?term=226517
Nbl1	Nbl1	neuroblastoma, suppression of tumorigenicity 1	17965	https://www.ncbi.nlm.nih.gov/gene/?term=17965
Frat1	Frat1	frequently rearranged in advanced T cell lymphomas	14296	https://www.ncbi.nlm.nih.gov/gene/?term=14296
Olfm1	Olfm1	olfactomedin 1	56177	https://www.ncbi.nlm.nih.gov/gene/?term=56177
Tmem178b	Tmem178b	transmembrane protein 178B	434008	https://www.ncbi.nlm.nih.gov/gene/?term=434008
Tpd52l2	Tpd52l2	tumor protein D52-like 2	66314	https://www.ncbi.nlm.nih.gov/gene/?term=66314
Nr2c2	Nr2c2	nuclear receptor subfamily 2, group C, member 2	22026	https://www.ncbi.nlm.nih.gov/gene/?term=22026
Gtpbp2	Gtpbp2	GTP binding protein 2	56055	https://www.ncbi.nlm.nih.gov/gene/?term=56055
Arhgap18	Arhgap18	Rho GTPase activating protein 18	73910	https://www.ncbi.nlm.nih.gov/gene/?term=73910
Cavin1	Cavin1	caveolae associated 1	19285	https://www.ncbi.nlm.nih.gov/gene/?term=19285
Spsb1	Spsb1	splA/ryanodine receptor domain and SOCS box containing 1	74646	https://www.ncbi.nlm.nih.gov/gene/?term=74646
Leprotl1	Leprotl1	leptin receptor overlapping transcript-like 1	68192	https://www.ncbi.nlm.nih.gov/gene/?term=68192
Zc3h6	Zc3h6	zinc finger CCCH type containing 6	78751	https://www.ncbi.nlm.nih.gov/gene/?term=78751
Amph	Amph	amphiphysin	218038	https://www.ncbi.nlm.nih.gov/gene/?term=218038

Biology Open • Supplementary information

Gab2	Gab2	growth factor receptor bound protein 2-associated protein 2	14389	https://www.ncbi.nlm.nih.gov/gene/?term=14389
Ndor1	Ndor1	NADPH dependent diflavin oxidoreductase 1	78797	https://www.ncbi.nlm.nih.gov/gene/?term=78797
Pacrg	Pacrg	PARK2 co-regulated	69310	https://www.ncbi.nlm.nih.gov/gene/?term=69310
Rb1	Rb1	RB transcriptional corepressor 1	19645	https://www.ncbi.nlm.nih.gov/gene/?term=19645
Natd1	Natd1	N-acetyltransferase domain containing 1	24083	https://www.ncbi.nlm.nih.gov/gene/?term=24083
Slc25a12	Slc25a12	solute carrier family 25 (mitochondrial carrier, Aralar), member 12	78830	https://www.ncbi.nlm.nih.gov/gene/?term=78830
Tmod1	Tmod1	tropomodulin 1	21916	https://www.ncbi.nlm.nih.gov/gene/?term=21916
Arhgef12	Arhgef12	Rho guanine nucleotide exchange factor (GEF) 12	69632	https://www.ncbi.nlm.nih.gov/gene/?term=69632
Macrod2	Macrod2	MACRO domain containing 2	72899	https://www.ncbi.nlm.nih.gov/gene/?term=72899
Dbf4	Dbf4	DBF4 zinc finger	27214	https://www.ncbi.nlm.nih.gov/gene/?term=27214
Rbm24	Rbm24	RNA binding motif protein 24	666794	https://www.ncbi.nlm.nih.gov/gene/?term=666794
Gabrg1	Gabrg1	gamma-aminobutyric acid (GABA) A receptor, subunit gamma 1	14405	https://www.ncbi.nlm.nih.gov/gene/?term=14405
Gphn	Gphn	gephyrin	268566	https://www.ncbi.nlm.nih.gov/gene/?term=268566
Rhot1	Rhot1	ras homolog family member T1	59040	https://www.ncbi.nlm.nih.gov/gene/?term=59040
Casp12	Casp12	caspase 12	12364	https://www.ncbi.nlm.nih.gov/gene/?term=12364
Slc41a2	Slc41a2	solute carrier family 41, member 2	338365	https://www.ncbi.nlm.nih.gov/gene/?term=338365
Kcnk1	Kcnk1	potassium channel, subfamily K, member 1	16525	https://www.ncbi.nlm.nih.gov/gene/?term=16525
Tbx15	Tbx15	T-box 15	21384	https://www.ncbi.nlm.nih.gov/gene/?term=21384
Prl5a1	Prl5a1	prolactin family 5, subfamily a, member 1	28078	https://www.ncbi.nlm.nih.gov/gene/?term=28078

Gprasp2	Gprasp2	G protein-coupled receptor associated sorting protein 2	245607	https://www.ncbi.nlm.nih.gov/gene/?term=245607
St8sia2	St8sia2	ST8 alpha-N-acetyl-neuraminide alpha-2,8-sialyltransferase 2	20450	https://www.ncbi.nlm.nih.gov/gene/?term=20450
Plekhb1	Plekhb1	pleckstrin homology domain containing, family B (evectins) member 1	27276	https://www.ncbi.nlm.nih.gov/gene/?term=27276
Cttnbp2	Cttnbp2	cortactin binding protein 2	30785	https://www.ncbi.nlm.nih.gov/gene/?term=30785
Myog	Myog	myogenin	17928	https://www.ncbi.nlm.nih.gov/gene/?term=17928
Ccdc81	Ccdc81	coiled-coil domain containing 81	70884	https://www.ncbi.nlm.nih.gov/gene/?term=70884
Lamp5	Lamp5	lysosomal-associated membrane protein family, member 5	76161	https://www.ncbi.nlm.nih.gov/gene/?term=76161
Pdlim5	Pdlim5	PDZ and LIM domain 5	56376	https://www.ncbi.nlm.nih.gov/gene/?term=56376
Zfp994	Zfp994	zinc finger protein 994	240038	https://www.ncbi.nlm.nih.gov/gene/?term=240038
Stt3a	Stt3a	STT3, subunit of the oligosaccharyltransferase complex, homolog A (S. cerevisiae)	16430	https://www.ncbi.nlm.nih.gov/gene/?term=16430
Pax3	Pax3	paired box 3	18505	https://www.ncbi.nlm.nih.gov/gene/?term=18505
Necab1	Necab1	N-terminal EF-hand calcium binding protein 1	69352	https://www.ncbi.nlm.nih.gov/gene/?term=69352
Far1	Far1	fatty acyl CoA reductase 1	67420	https://www.ncbi.nlm.nih.gov/gene/?term=67420
Abhd11	Abhd11	abhydrolase domain containing 11	68758	https://www.ncbi.nlm.nih.gov/gene/?term=68758
Chmp5	Chmp5	charged multivesicular body protein 5	76959	https://www.ncbi.nlm.nih.gov/gene/?term=76959
Slco5a1	Slco5a1	solute carrier organic anion transporter family, member 5A1	240726	https://www.ncbi.nlm.nih.gov/gene/?term=240726
Slc10a2	Slc10a2	solute carrier family 10, member 2	20494	https://www.ncbi.nlm.nih.gov/gene/?term=20494
Tceal7	Tceal7	transcription elongation factor A (SII)-like 7	100040972	https://www.ncbi.nlm.nih.gov/gene/?term=100040[illegible]

Biology Open • Supplementary information

Slc5a7	Slc5a7	solute carrier family 5 (choline transporter), member 7	63993	https://www.ncbi.nlm.nih.gov/gene/?term=63993
Cyp26a1	Cyp26a1	cytochrome P450, family 26, subfamily a, polypeptide 1	13082	https://www.ncbi.nlm.nih.gov/gene/?term=13082
Cnot2	Cnot2	CCR4-NOT transcription complex, subunit 2	72068	https://www.ncbi.nlm.nih.gov/gene/?term=72068
Jak2	Jak2	Janus kinase 2	16452	https://www.ncbi.nlm.nih.gov/gene/?term=16452
Sdf2	Sdf2	stromal cell derived factor 2	20316	https://www.ncbi.nlm.nih.gov/gene/?term=20316
Ebf2	Ebf2	early B cell factor 2	13592	https://www.ncbi.nlm.nih.gov/gene/?term=13592
Gatb	Gatb	glutamyl-tRNA(Gln) amidotransferase, subunit B	229487	https://www.ncbi.nlm.nih.gov/gene/?term=229487
Spsb4	Spsb4	splA/ryanodine receptor domain and SOCS box containing 4	211949	https://www.ncbi.nlm.nih.gov/gene/?term=211949
Mrpl50	Mrpl50	mitochondrial ribosomal protein L50	28028	https://www.ncbi.nlm.nih.gov/gene/?term=28028
Nars	Nars	asparaginyl-tRNA synthetase	70223	https://www.ncbi.nlm.nih.gov/gene/?term=70223
Tmc5	Tmc5	transmembrane channel-like gene family 5	74424	https://www.ncbi.nlm.nih.gov/gene/?term=74424
Wasf1	Wasf1	WAS protein family, member 1	83767	https://www.ncbi.nlm.nih.gov/gene/?term=83767
Kcmf1	Kcmf1	potassium channel modulatory factor 1	74287	https://www.ncbi.nlm.nih.gov/gene/?term=74287
Utp6	Utp6	UTP6 small subunit processome component	216987	https://www.ncbi.nlm.nih.gov/gene/?term=216987
Lcmt2	Lcmt2	leucine carboxyl methyltransferase 2	329504	https://www.ncbi.nlm.nih.gov/gene/?term=329504
Enox2	Enox2	ecto-NOX disulfide-thiol exchanger 2	209224	https://www.ncbi.nlm.nih.gov/gene/?term=209224
Ppp1r26	Ppp1r26	protein phosphatase 1, regulatory subunit 26	241289	https://www.ncbi.nlm.nih.gov/gene/?term=241289
Rpn1	Rpn1	ribophorin I	103963	https://www.ncbi.nlm.nih.gov/gene/?term=103963
Vdac2	Vdac2	voltage-dependent anion channel 2	22334	https://www.ncbi.nlm.nih.gov/gene/?term=22334

Shb	Shb	src homology 2 domain-containing transforming protein B	230126	https://www.ncbi.nlm.nih.gov/gene/?term=230126
Hpcal4	Hpcal4	hippocalcin-like 4	170638	https://www.ncbi.nlm.nih.gov/gene/?term=170638
Selenof	Selenof	selenoprotein F	93684	https://www.ncbi.nlm.nih.gov/gene/?term=93684
Pcdhb22	Pcdhb22	protocadherin beta 22	93893	https://www.ncbi.nlm.nih.gov/gene/?term=93893
Poc1b	Poc1b	POC1 centriolar protein B	382406	https://www.ncbi.nlm.nih.gov/gene/?term=382406
Mmp9	Mmp9	matrix metallopeptidase 9	17395	https://www.ncbi.nlm.nih.gov/gene/?term=17395
Nol3	Nol3	nucleolar protein 3 (apoptosis repressor with CARD domain)	78688	https://www.ncbi.nlm.nih.gov/gene/?term=78688
Bcl2a1d	Bcl2a1d	B cell leukemia/lymphoma 2 related protein A1d	12047	https://www.ncbi.nlm.nih.gov/gene/?term=12047
Ano3	Ano3	anoctamin 3	228432	https://www.ncbi.nlm.nih.gov/gene/?term=228432
Tslp	Tslp	thymic stromal lymphopoietin	53603	https://www.ncbi.nlm.nih.gov/gene/?term=53603
Lpar4	Lpar4	lysophosphatidic acid receptor 4	78134	https://www.ncbi.nlm.nih.gov/gene/?term=78134
Fgf7	Fgf7	fibroblast growth factor 7	14178	https://www.ncbi.nlm.nih.gov/gene/?term=14178
Bcl2a1b	Bcl2a1b	B cell leukemia/lymphoma 2 related protein A1b	12045	https://www.ncbi.nlm.nih.gov/gene/?term=12045
Tmem123	Tmem123	transmembrane protein 123	71929	https://www.ncbi.nlm.nih.gov/gene/?term=71929
Bcl2a1a	Bcl2a1a	B cell leukemia/lymphoma 2 related protein A1a	12044	https://www.ncbi.nlm.nih.gov/gene/?term=12044
Erp44	Erp44	endoplasmic reticulum protein 44	76299	https://www.ncbi.nlm.nih.gov/gene/?term=76299
Zxdb	Zxdb	zinc finger, X-linked, duplicated B	668166	https://www.ncbi.nlm.nih.gov/gene/?term=668166
Lrrn3	Lrrn3	leucine rich repeat protein 3, neuronal	16981	https://www.ncbi.nlm.nih.gov/gene/?term=16981
Tmem229a	Tmem229a	transmembrane protein 229A	319832	https://www.ncbi.nlm.nih.gov/gene/?term=319832
Tnfrsf9	Tnfrsf9	tumor necrosis factor receptor superfamily, member 9	21942	https://www.ncbi.nlm.nih.gov/gene/?term=21942
Pum1	Pum1	pumilio RNA-binding family member 1	80912	https://www.ncbi.nlm.nih.gov/gene/?term=80912

Zfp641	Zfp641	zinc finger protein 641	239652	https://www.ncbi.nlm.nih.gov/gene/?term=239652
Cdc14a	Cdc14a	CDC14 cell division cycle 14A	229776	https://www.ncbi.nlm.nih.gov/gene/?term=229776
Pank4	Pank4	pantothenate kinase 4	269614	https://www.ncbi.nlm.nih.gov/gene/?term=269614
Ccpg1	Ccpg1	cell cycle progression 1	72278	https://www.ncbi.nlm.nih.gov/gene/?term=72278
Pla2g4a	Pla2g4a	phospholipase A2, group IVA (cytosolic, calcium-dependent)	18783	https://www.ncbi.nlm.nih.gov/gene/?term=18783
Gabrb3	Gabrb3	gamma-aminobutyric acid (GABA) A receptor, subunit beta 3	14402	https://www.ncbi.nlm.nih.gov/gene/?term=14402
Enpp6	Enpp6	ectonucleotide pyrophosphatase/phosphodiesterase 6	320981	https://www.ncbi.nlm.nih.gov/gene/?term=320981
Cps1	Cps1	carbamoyl-phosphate synthetase 1	227231	https://www.ncbi.nlm.nih.gov/gene/?term=227231
Hnrnpa2b1	Hnrnpa2b1	heterogeneous nuclear ribonucleoprotein A2/B1	53379	https://www.ncbi.nlm.nih.gov/gene/?term=53379
D630023F18Rik	D630023F18Rik	RIKEN cDNA D630023F18 gene	98303	https://www.ncbi.nlm.nih.gov/gene/?term=98303
Gnb4	Gnb4	guanine nucleotide binding protein (G protein), beta 4	14696	https://www.ncbi.nlm.nih.gov/gene/?term=14696
Ccdc125	Ccdc125	coiled-coil domain containing 125	76041	https://www.ncbi.nlm.nih.gov/gene/?term=76041
Ngfr	Ngfr	nerve growth factor receptor (TNFR superfamily, member 16)	18053	https://www.ncbi.nlm.nih.gov/gene/?term=18053
Golt1a	Golt1a	golgi transport 1A	68338	https://www.ncbi.nlm.nih.gov/gene/?term=68338
Vps33b	Vps33b	vacuolar protein sorting 33B	233405	https://www.ncbi.nlm.nih.gov/gene/?term=233405
Plag1	Plag1	pleiomorphic adenoma gene 1	56711	https://www.ncbi.nlm.nih.gov/gene/?term=56711
Jakmip3	Jakmip3	janus kinase and microtubule interacting protein 3	74004	https://www.ncbi.nlm.nih.gov/gene/?term=74004
Atp2a2	Atp2a2	ATPase, Ca++ transporting, cardiac muscle, slow twitch 2	11938	https://www.ncbi.nlm.nih.gov/gene/?term=11938
Cartpt	Cartpt	CART prepropeptide	27220	https://www.ncbi.nlm.nih.gov/gene/?term=27220

Kcnq2	Kcnq2	potassium voltage-gated channel, subfamily Q, member 2	16536	https://www.ncbi.nlm.nih.gov/gene/?term=16536
Pfkfb2	Pfkfb2	6-phosphofructo-2-kinase/fructose-2,6-biphosphatase 2	18640	https://www.ncbi.nlm.nih.gov/gene/?term=18640
Treml2	Treml2	triggering receptor expressed on myeloid cells-like 2	328833	https://www.ncbi.nlm.nih.gov/gene/?term=328833
Cplx2	Cplx2	complexin 2	12890	https://www.ncbi.nlm.nih.gov/gene/?term=12890
Xkr6	Xkr6	X-linked Kx blood group related 6	219149	https://www.ncbi.nlm.nih.gov/gene/?term=219149
Cacfd1	Cacfd1	calcium channel flower domain containing 1	381356	https://www.ncbi.nlm.nih.gov/gene/?term=381356
Ncam2	Ncam2	neural cell adhesion molecule 2	17968	https://www.ncbi.nlm.nih.gov/gene/?term=17968
Rock2	Rock2	Rho-associated coiled-coil containing protein kinase 2	19878	https://www.ncbi.nlm.nih.gov/gene/?term=19878
Wdr64	Wdr64	WD repeat domain 64	75820	https://www.ncbi.nlm.nih.gov/gene/?term=75820
Fuca2	Fuca2	fucosidase, alpha-L- 2, plasma	66848	https://www.ncbi.nlm.nih.gov/gene/?term=66848
Top2b	Top2b	topoisomerase (DNA) II beta	21974	https://www.ncbi.nlm.nih.gov/gene/?term=21974
Senp8	Senp8	SUMO/sentrin specific peptidase 8	71599	https://www.ncbi.nlm.nih.gov/gene/?term=71599
Ccrl2	Ccrl2	chemokine (C-C motif) receptor-like 2	54199	https://www.ncbi.nlm.nih.gov/gene/?term=54199
Lrrcc1	Lrrcc1	leucine rich repeat and coiled-coil domain containing 1	71710	https://www.ncbi.nlm.nih.gov/gene/?term=71710
Dpp6	Dpp6	dipeptidylpeptidase 6	13483	https://www.ncbi.nlm.nih.gov/gene/?term=13483
Lrrn1	Lrrn1	leucine rich repeat protein 1, neuronal	16979	https://www.ncbi.nlm.nih.gov/gene/?term=16979
Ttc33	Ttc33	tetratricopeptide repeat domain 33	67515	https://www.ncbi.nlm.nih.gov/gene/?term=67515
Arpp21	Arpp21	cyclic AMP-regulated phosphoprotein, 21	74100	https://www.ncbi.nlm.nih.gov/gene/?term=74100
Mecom	Mecom	MDS1 and EVI1 complex locus	14013	https://www.ncbi.nlm.nih.gov/gene/?term=14013
Srp9	Srp9	signal recognition particle 9	27058	https://www.ncbi.nlm.nih.gov/gene/?term=27058

Biology Open • Supplementary information

Grk2	Grk2	G protein-coupled receptor kinase 2	110355	https://www.ncbi.nlm.nih.gov/gene/?term=110355
Pum2	Pum2	pumilio RNA-binding family member 2	80913	https://www.ncbi.nlm.nih.gov/gene/?term=80913
Chst13	Chst13	carbohydrate sulfotransferase 13	71797	https://www.ncbi.nlm.nih.gov/gene/?term=71797
Hecw2	Hecw2	HECT, C2 and WW domain containing E3 ubiquitin protein ligase 2	329152	https://www.ncbi.nlm.nih.gov/gene/?term=329152
Sema3d	Sema3d	sema domain, immunoglobulin domain (Ig), short basic domain, secreted, (semaphorin) 3D	108151	https://www.ncbi.nlm.nih.gov/gene/?term=108151
Zfp518b	Zfp518b	zinc finger protein 518B	100515	https://www.ncbi.nlm.nih.gov/gene/?term=100515
Usp7	Usp7	ubiquitin specific peptidase 7	252870	https://www.ncbi.nlm.nih.gov/gene/?term=252870
U2af2	U2af2	U2 small nuclear ribonucleoprotein auxiliary factor (U2AF) 2	22185	https://www.ncbi.nlm.nih.gov/gene/?term=22185
Scaf11	Scaf11	SR-related CTD-associated factor 11	72193	https://www.ncbi.nlm.nih.gov/gene/?term=72193
Nr4a3	Nr4a3	nuclear receptor subfamily 4, group A, member 3	18124	https://www.ncbi.nlm.nih.gov/gene/?term=18124
Chrnb1	Chrnb1	cholinergic receptor, nicotinic, beta polypeptide 1 (muscle)	11443	https://www.ncbi.nlm.nih.gov/gene/?term=11443
Chsy3	Chsy3	chondroitin sulfate synthase 3	78923	https://www.ncbi.nlm.nih.gov/gene/?term=78923
Rbm41	Rbm41	RNA binding motif protein 41	237073	https://www.ncbi.nlm.nih.gov/gene/?term=237073
Cul4b	Cul4b	cullin 4B	72584	https://www.ncbi.nlm.nih.gov/gene/?term=72584
Sec61a2	Sec61a2	Sec61, alpha subunit 2 (S. cerevisiae)	57743	https://www.ncbi.nlm.nih.gov/gene/?term=57743
Tsen34	Tsen34	tRNA splicing endonuclease subunit 34	66078	https://www.ncbi.nlm.nih.gov/gene/?term=66078
Prr5l	Prr5l	proline rich 5 like	72446	https://www.ncbi.nlm.nih.gov/gene/?term=72446
Mecp2	Mecp2	methyl CpG binding protein 2	17257	https://www.ncbi.nlm.nih.gov/gene/?term=17257
Rgs13	Rgs13	regulator of G-protein signaling 13	246709	https://www.ncbi.nlm.nih.gov/gene/?term=246709
Cxcr4	Cxcr4	chemokine (C-X-C motif) receptor 4	12767	https://www.ncbi.nlm.nih.gov/gene/?term=12767

Biology Open • Supplementary information

Spink5	Spink5	serine peptidase inhibitor, Kazal type 5	72432	https://www.ncbi.nlm.nih.gov/gene/?term=72432
Pnma5	Pnma5	paraneoplastic antigen family 5	385377	https://www.ncbi.nlm.nih.gov/gene/?term=385377
Faxc	Faxc	failed axon connections homolog	76132	https://www.ncbi.nlm.nih.gov/gene/?term=76132
Pqlc3	Pqlc3	PQ loop repeat containing	217430	https://www.ncbi.nlm.nih.gov/gene/?term=217430
Tomm70a	Tomm70a	translocase of outer mitochondrial membrane 70A	28185	https://www.ncbi.nlm.nih.gov/gene/?term=28185
Fgf13	Fgf13	fibroblast growth factor 13	14168	https://www.ncbi.nlm.nih.gov/gene/?term=14168
Pank3	Pank3	pantothenate kinase 3	211347	https://www.ncbi.nlm.nih.gov/gene/?term=211347
Tm2d1	Tm2d1	TM2 domain containing 1	94043	https://www.ncbi.nlm.nih.gov/gene/?term=94043
Dync1li2	Dync1li2	dynein, cytoplasmic 1 light intermediate chain 2	234663	https://www.ncbi.nlm.nih.gov/gene/?term=234663
Plekhf2	Plekhf2	pleckstrin homology domain containing, family F (with FYVE domain) member 2	71801	https://www.ncbi.nlm.nih.gov/gene/?term=71801
Mmgt1	Mmgt1	membrane magnesium transporter 1	236792	https://www.ncbi.nlm.nih.gov/gene/?term=236792
Dck	Dck	deoxycytidine kinase	13178	https://www.ncbi.nlm.nih.gov/gene/?term=13178
Unc93a	Unc93a	unc-93 homolog A	381058	https://www.ncbi.nlm.nih.gov/gene/?term=381058
Crp	Crp	C-reactive protein, pentraxin-related	12944	https://www.ncbi.nlm.nih.gov/gene/?term=12944
Slc39a10	Slc39a10	solute carrier family 39 (zinc transporter), member 10	227059	https://www.ncbi.nlm.nih.gov/gene/?term=227059
Dach1	Dach1	dachshund family transcription factor 1	13134	https://www.ncbi.nlm.nih.gov/gene/?term=13134
Irf2bpl	Irf2bpl	interferon regulatory factor 2 binding protein-like	238330	https://www.ncbi.nlm.nih.gov/gene/?term=238330
Rnf8	Rnf8	ring finger protein 8	58230	https://www.ncbi.nlm.nih.gov/gene/?term=58230
Tcea1	Tcea1	transcription elongation factor A (SII) 1	21399	https://www.ncbi.nlm.nih.gov/gene/?term=21399
Zdhhc17	Zdhhc17	zinc finger, DHHC domain containing	320150	https://www.ncbi.nlm.nih.gov/gene/?term=320150

		17		
Aco1	Aco1	aconitase 1	11428	https://www.ncbi.nlm.nih.gov/gene/?term=11428
Brd4	Brd4	bromodomain containing 4	57261	https://www.ncbi.nlm.nih.gov/gene/?term=57261
Zbtb11	Zbtb11	zinc finger and BTB domain containing 11	271377	https://www.ncbi.nlm.nih.gov/gene/?term=271377
AF366264	AF366264	cDNA sequence AF366264	231201	https://www.ncbi.nlm.nih.gov/gene/?term=231201
Nmu	Nmu	neuromedin U	56183	https://www.ncbi.nlm.nih.gov/gene/?term=56183
Slit1	Slit1	slit guidance ligand 1	20562	https://www.ncbi.nlm.nih.gov/gene/?term=20562
Camkk2	Camkk2	calcium/calmodulin-dependent protein kinase kinase 2, beta	207565	https://www.ncbi.nlm.nih.gov/gene/?term=207565
Zbtb1	Zbtb1	zinc finger and BTB domain containing 1	268564	https://www.ncbi.nlm.nih.gov/gene/?term=268564
Eid2b	Eid2b	EP300 interacting inhibitor of differentiation 2B	434156	https://www.ncbi.nlm.nih.gov/gene/?term=434156
Tnks	Tnks	tankyrase, TRF1-interacting ankyrin-related ADP-ribose polymerase	21951	https://www.ncbi.nlm.nih.gov/gene/?term=21951
Wdr44	Wdr44	WD repeat domain 44	72404	https://www.ncbi.nlm.nih.gov/gene/?term=72404
Eif1b	Eif1b	eukaryotic translation initiation factor 1B	68969	https://www.ncbi.nlm.nih.gov/gene/?term=68969
Nbdy	Nbdy	negative regulator of P-body association	70123	https://www.ncbi.nlm.nih.gov/gene/?term=70123
Nrxn3	Nrxn3	neurexin III	18191	https://www.ncbi.nlm.nih.gov/gene/?term=18191
Synj2bp	Synj2bp	synaptojanin 2 binding protein	24071	https://www.ncbi.nlm.nih.gov/gene/?term=24071
Pdcl3	Pdcl3	phosducin-like 3	68833	https://www.ncbi.nlm.nih.gov/gene/?term=68833
Pias2	Pias2	protein inhibitor of activated STAT 2	17344	https://www.ncbi.nlm.nih.gov/gene/?term=17344
Trim52	Trim52	tripartite motif-containing 52	212085	https://www.ncbi.nlm.nih.gov/gene/?term=212085
Hmgb1	Hmgb1	high mobility group box 1	15289	https://www.ncbi.nlm.nih.gov/gene/?term=15289
Pdp2	Pdp2	pyruvate dehyrogenase phosphatase catalytic subunit 2	382051	https://www.ncbi.nlm.nih.gov/gene/?term=382051

Biology Open • Supplementary information

Etnk1	Etnk1	ethanolamine kinase 1	75320	https://www.ncbi.nlm.nih.gov/gene/?term=75320
Ttc23l	Ttc23l	tetratricopeptide repeat domain 23-like	75777	https://www.ncbi.nlm.nih.gov/gene/?term=75777
Met	Met	met proto-oncogene	17295	https://www.ncbi.nlm.nih.gov/gene/?term=17295
Dpp4	Dpp4	dipeptidylpeptidase 4	13482	https://www.ncbi.nlm.nih.gov/gene/?term=13482
Nxf3	Nxf3	nuclear RNA export factor 3	245610	https://www.ncbi.nlm.nih.gov/gene/?term=245610
Eif1ax	Eif1ax	eukaryotic translation initiation factor 1A, X-linked	66235	https://www.ncbi.nlm.nih.gov/gene/?term=66235
Gnpnat1	Gnpnat1	glucosamine-phosphate N-acetyltransferase 1	54342	https://www.ncbi.nlm.nih.gov/gene/?term=54342
Ints2	Ints2	integrator complex subunit 2	70422	https://www.ncbi.nlm.nih.gov/gene/?term=70422
Spin4	Spin4	spindlin family, member 4	270624	https://www.ncbi.nlm.nih.gov/gene/?term=270624
Sdr16c5	Sdr16c5	short chain dehydrogenase/reductase family 16C, member 5	242285	https://www.ncbi.nlm.nih.gov/gene/?term=242285
Cxcl5	Cxcl5	chemokine (C-X-C motif) ligand 5	20311	https://www.ncbi.nlm.nih.gov/gene/?term=20311
Fam160a2	Fam160a2	family with sequence similarity 160, member A2	74349	https://www.ncbi.nlm.nih.gov/gene/?term=74349
Trpc1	Trpc1	transient receptor potential cation channel, subfamily C, member 1	22063	https://www.ncbi.nlm.nih.gov/gene/?term=22063
Tbc1d22b	Tbc1d22b	TBC1 domain family, member 22B	381085	https://www.ncbi.nlm.nih.gov/gene/?term=381085
Epb41l1	Epb41l1	erythrocyte membrane protein band 4.1 like 1	13821	https://www.ncbi.nlm.nih.gov/gene/?term=13821
Neurod6	Neurod6	neurogenic differentiation 6	11922	https://www.ncbi.nlm.nih.gov/gene/?term=11922
Emc7	Emc7	ER membrane protein complex subunit 7	73024	https://www.ncbi.nlm.nih.gov/gene/?term=73024
Dnttip2	Dnttip2	deoxynucleotidyltransferase, terminal, interacting protein 2	99480	https://www.ncbi.nlm.nih.gov/gene/?term=99480
Luc7l2	Luc7l2	LUC7-like 2 (S. cerevisiae)	192196	https://www.ncbi.nlm.nih.gov/gene/?term=192196

Tjp2	Tjp2	tight junction protein 2	21873	https://www.ncbi.nlm.nih.gov/gene/?term=21873
Hoxd1	Hoxd1	homeobox D1	15429	https://www.ncbi.nlm.nih.gov/gene/?term=15429
Rgs4	Rgs4	regulator of G-protein signaling 4	19736	https://www.ncbi.nlm.nih.gov/gene/?term=19736
Phf6	Phf6	PHD finger protein 6	70998	https://www.ncbi.nlm.nih.gov/gene/?term=70998
A630023A22Rik	A630023A22Rik	RIKEN cDNA A630023A22 gene	105518	https://www.ncbi.nlm.nih.gov/gene/?term=105518
Prkcd	Prkcd	protein kinase C, delta	18753	https://www.ncbi.nlm.nih.gov/gene/?term=18753
Nfyb	Nfyb	nuclear transcription factor-Y beta	18045	https://www.ncbi.nlm.nih.gov/gene/?term=18045
Tshz3	Tshz3	teashirt zinc finger family member 3	243931	https://www.ncbi.nlm.nih.gov/gene/?term=243931
Dst	Dst	dystonin	13518	https://www.ncbi.nlm.nih.gov/gene/?term=13518
Ddx10	Ddx10	DEAD (Asp-Glu-Ala-Asp) box polypeptide 10	77591	https://www.ncbi.nlm.nih.gov/gene/?term=77591
Apool	Apool	apolipoprotein O-like	68117	https://www.ncbi.nlm.nih.gov/gene/?term=68117
Gopc	Gopc	golgi associated PDZ and coiled-coil motif containing	94221	https://www.ncbi.nlm.nih.gov/gene/?term=94221
Znrf3	Znrf3	zinc and ring finger 3	407821	https://www.ncbi.nlm.nih.gov/gene/?term=407821
Golph3	Golph3	golgi phosphoprotein 3	66629	https://www.ncbi.nlm.nih.gov/gene/?term=66629
Dleu7	Dleu7	deleted in lymphocytic leukemia, 7	239133	https://www.ncbi.nlm.nih.gov/gene/?term=239133
Gabra2	Gabra2	gamma-aminobutyric acid (GABA) A receptor, subunit alpha 2	14395	https://www.ncbi.nlm.nih.gov/gene/?term=14395
Oas3	Oas3	2'-5' oligoadenylate synthetase 3	246727	https://www.ncbi.nlm.nih.gov/gene/?term=246727
Arhgef10l	Arhgef10l	Rho guanine nucleotide exchange factor (GEF) 10-like	72754	https://www.ncbi.nlm.nih.gov/gene/?term=72754
Casz1	Casz1	castor zinc finger 1	69743	https://www.ncbi.nlm.nih.gov/gene/?term=69743
Bmper	Bmper	BMP-binding endothelial regulator	73230	https://www.ncbi.nlm.nih.gov/gene/?term=73230
AI182371	AI182371	expressed sequence AI182371	98870	https://www.ncbi.nlm.nih.gov/gene/?term=98870
Tle4	Tle4	transducin-like enhancer of split 4	21888	https://www.ncbi.nlm.nih.gov/gene/?term=21888
Car5b	Car5b	carbonic anhydrase 5b, mitochondrial	56078	https://www.ncbi.nlm.nih.gov/gene/?term=56078
Golim4	Golim4	golgi integral membrane protein 4	73124	https://www.ncbi.nlm.nih.gov/gene/?term=73124

Rerg	Rerg	RAS-like, estrogen-regulated, growth-inhibitor	232441	https://www.ncbi.nlm.nih.gov/gene/?term=232441
Gucy1b1	Gucy1b1	guanylate cyclase 1, soluble, beta 1	54195	https://www.ncbi.nlm.nih.gov/gene/?term=54195
Usp1	Usp1	ubiquitin specific peptidase 1	230484	https://www.ncbi.nlm.nih.gov/gene/?term=230484
Uty	Uty	ubiquitously transcribed tetratricopeptide repeat gene, Y chromosome	22290	https://www.ncbi.nlm.nih.gov/gene/?term=22290
Usp6nl	Usp6nl	USP6 N-terminal like	98910	https://www.ncbi.nlm.nih.gov/gene/?term=98910
Ptprr	Ptprr	protein tyrosine phosphatase, receptor type, R	19279	https://www.ncbi.nlm.nih.gov/gene/?term=19279
Dip2b	Dip2b	disco interacting protein 2 homolog B	239667	https://www.ncbi.nlm.nih.gov/gene/?term=239667
Cxxc4	Cxxc4	CXXC finger 4	319478	https://www.ncbi.nlm.nih.gov/gene/?term=319478
Grk6	Grk6	G protein-coupled receptor kinase 6	26385	https://www.ncbi.nlm.nih.gov/gene/?term=26385
Mis18a	Mis18a	MIS18 kinetochore protein A	66578	https://www.ncbi.nlm.nih.gov/gene/?term=66578
Fgf4	Fgf4	fibroblast growth factor 4	14175	https://www.ncbi.nlm.nih.gov/gene/?term=14175
Lin52	Lin52	lin-52 homolog (C. elegans)	217708	https://www.ncbi.nlm.nih.gov/gene/?term=217708
Adamts1	Adamts1	a disintegrin-like and metallopeptidase (reprolysin type) with thrombospondin type 1 motif, 1	11504	https://www.ncbi.nlm.nih.gov/gene/?term=11504
Tbc1d24	Tbc1d24	TBC1 domain family, member 24	224617	https://www.ncbi.nlm.nih.gov/gene/?term=224617
Riok3	Riok3	RIO kinase 3	66878	https://www.ncbi.nlm.nih.gov/gene/?term=66878
Nceh1	Nceh1	neutral cholesterol ester hydrolase 1	320024	https://www.ncbi.nlm.nih.gov/gene/?term=320024
Wdr43	Wdr43	WD repeat domain 43	72515	https://www.ncbi.nlm.nih.gov/gene/?term=72515
Etaa1	Etaa1	Ewing tumor-associated antigen 1	68145	https://www.ncbi.nlm.nih.gov/gene/?term=68145
Bmp5	Bmp5	bone morphogenetic protein 5	12160	https://www.ncbi.nlm.nih.gov/gene/?term=12160
Skint9	Skint9	selection and upkeep of intraepithelial T cells 9	329918	https://www.ncbi.nlm.nih.gov/gene/?term=329918

Biology Open • Supplementary information

Pcsk5	Pcsk5	proprotein convertase subtilisin/kexin type 5	18552	https://www.ncbi.nlm.nih.gov/gene/?term=18552
Cenpi	Cenpi	centromere protein I	102920	https://www.ncbi.nlm.nih.gov/gene/?term=102920
Spock3	Spock3	sparc/osteonectin, cwcv and kazal-like domains proteoglycan 3	72902	https://www.ncbi.nlm.nih.gov/gene/?term=72902
Gpm6b	Gpm6b	glycoprotein m6b	14758	https://www.ncbi.nlm.nih.gov/gene/?term=14758
Apobec3	Apobec3	apolipoprotein B mRNA editing enzyme, catalytic polypeptide 3	80287	https://www.ncbi.nlm.nih.gov/gene/?term=80287
Gm5592	Gm5592	predicted gene 5592	434172	https://www.ncbi.nlm.nih.gov/gene/?term=434172
Fgd4	Fgd4	FYVE, RhoGEF and PH domain containing 4	224014	https://www.ncbi.nlm.nih.gov/gene/?term=224014
Gtf2b	Gtf2b	general transcription factor IIB	229906	https://www.ncbi.nlm.nih.gov/gene/?term=229906
Rbbp5	Rbbp5	retinoblastoma binding protein 5, histone lysine methyltransferase complex subunit	213464	https://www.ncbi.nlm.nih.gov/gene/?term=213464
Mei4	Mei4	meiotic double-stranded break formation protein 4	75033	https://www.ncbi.nlm.nih.gov/gene/?term=75033
Arhgef7	Arhgef7	Rho guanine nucleotide exchange factor (GEF7)	54126	https://www.ncbi.nlm.nih.gov/gene/?term=54126
Il1b	Il1b	interleukin 1 beta	16176	https://www.ncbi.nlm.nih.gov/gene/?term=16176
Dph6	Dph6	diphthamine biosynthesis 6	66632	https://www.ncbi.nlm.nih.gov/gene/?term=66632
Svil	Svil	supervillin	225115	https://www.ncbi.nlm.nih.gov/gene/?term=225115
Diaph2	Diaph2	diaphanous related formin 2	54004	https://www.ncbi.nlm.nih.gov/gene/?term=54004
Fgl2	Fgl2	fibrinogen-like protein 2	14190	https://www.ncbi.nlm.nih.gov/gene/?term=14190
Fgfr2	Fgfr2	fibroblast growth factor receptor 2	14183	https://www.ncbi.nlm.nih.gov/gene/?term=14183
Ccdc166	Ccdc166	coiled-coil domain containing 166	223648	https://www.ncbi.nlm.nih.gov/gene/?term=223648
St8sia4	St8sia4	ST8 alpha-N-acetyl-neuraminide alpha-2,8-sialyltransferase 4	20452	https://www.ncbi.nlm.nih.gov/gene/?term=20452

Biology Open • Supplementary information

Tceanc	Tceanc	transcription elongation factor A (SII) N-terminal and central domain containing	245695	https://www.ncbi.nlm.nih.gov/gene/?term=245695
Ptprb	Ptprb	protein tyrosine phosphatase, receptor type, B	19263	https://www.ncbi.nlm.nih.gov/gene/?term=19263
Mzt1	Mzt1	mitotic spindle organizing protein 1	76789	https://www.ncbi.nlm.nih.gov/gene/?term=76789
Fbh1	Fbh1	F-box DNA helicase 1	50755	https://www.ncbi.nlm.nih.gov/gene/?term=50755
Nup35	Nup35	nucleoporin 35	69482	https://www.ncbi.nlm.nih.gov/gene/?term=69482
Gm4884	Gm4884	predicted gene 4884	233164	https://www.ncbi.nlm.nih.gov/gene/?term=233164
Cenpc1	Cenpc1	centromere protein C1	12617	https://www.ncbi.nlm.nih.gov/gene/?term=12617
Cdc42se2	Cdc42se2	CDC42 small effector 2	72729	https://www.ncbi.nlm.nih.gov/gene/?term=72729
Sf3a1	Sf3a1	splicing factor 3a, subunit 1	67465	https://www.ncbi.nlm.nih.gov/gene/?term=67465
Gm5114	Gm5114	predicted gene 5114	330513	https://www.ncbi.nlm.nih.gov/gene/?term=330513
Mkln1	Mkln1	muskelin 1, intracellular mediator containing kelch motifs	27418	https://www.ncbi.nlm.nih.gov/gene/?term=27418
Loxl3	Loxl3	lysyl oxidase-like 3	16950	https://www.ncbi.nlm.nih.gov/gene/?term=16950
Mdm1	Mdm1	transformed mouse 3T3 cell double minute 1	17245	https://www.ncbi.nlm.nih.gov/gene/?term=17245
Cxcl16	Cxcl16	chemokine (C-X-C motif) ligand 16	66102	https://www.ncbi.nlm.nih.gov/gene/?term=66102
Mfsd2a	Mfsd2a	major facilitator superfamily domain containing 2A	76574	https://www.ncbi.nlm.nih.gov/gene/?term=76574
Rai1	Rai1	retinoic acid induced 1	19377	https://www.ncbi.nlm.nih.gov/gene/?term=19377
Ccdc169	Ccdc169	coiled-coil domain containing 169	320604	https://www.ncbi.nlm.nih.gov/gene/?term=320604
Zmym5	Zmym5	zinc finger, MYM-type 5	219105	https://www.ncbi.nlm.nih.gov/gene/?term=219105
Tbx22	Tbx22	T-box 22	245572	https://www.ncbi.nlm.nih.gov/gene/?term=245572
Mex3c	Mex3c	mex3 RNA binding family member C	240396	https://www.ncbi.nlm.nih.gov/gene/?term=240396
Six6	Six6	sine oculis-related homeobox 6	20476	https://www.ncbi.nlm.nih.gov/gene/?term=20476
Cbln1	Cbln1	cerebellin 1 precursor protein	12404	https://www.ncbi.nlm.nih.gov/gene/?term=12404

Biology Open • Supplementary information

Alg6	Alg6	asparagine-linked glycosylation 6 (alpha-1,3,-glucosyltransferase)	320438	https://www.ncbi.nlm.nih.gov/gene/?term=320438
Gm45915	Gm45915	predicted gene, 45915	100188919	https://www.ncbi.nlm.nih.gov/gene/?term=100188[illegible]
Grip1	Grip1	glutamate receptor interacting protein 1	74053	https://www.ncbi.nlm.nih.gov/gene/?term=74053
Vps35	Vps35	VPS35 retromer complex component	65114	https://www.ncbi.nlm.nih.gov/gene/?term=65114
Cul3	Cul3	cullin 3	26554	https://www.ncbi.nlm.nih.gov/gene/?term=26554
Prr12	Prr12	proline rich 12	233210	https://www.ncbi.nlm.nih.gov/gene/?term=233210
Eml6	Eml6	echinoderm microtubule associated protein like 6	237711	https://www.ncbi.nlm.nih.gov/gene/?term=237711
Ppm1h	Ppm1h	protein phosphatase 1H (PP2C domain containing)	319468	https://www.ncbi.nlm.nih.gov/gene/?term=319468
Arl6ip6	Arl6ip6	ADP-ribosylation factor-like 6 interacting protein 6	65103	https://www.ncbi.nlm.nih.gov/gene/?term=65103
Hoxb3	Hoxb3	homeobox B3	15410	https://www.ncbi.nlm.nih.gov/gene/?term=15410
Lin54	Lin54	lin-54 homolog (C. elegans)	231506	https://www.ncbi.nlm.nih.gov/gene/?term=231506
Itgb1	Itgb1	integrin beta 1 (fibronectin receptor beta)	16412	https://www.ncbi.nlm.nih.gov/gene/?term=16412
Ankrd1	Ankrd1	ankyrin repeat domain 1 (cardiac muscle)	107765	https://www.ncbi.nlm.nih.gov/gene/?term=107765
Smad7	Smad7	SMAD family member 7	17131	https://www.ncbi.nlm.nih.gov/gene/?term=17131
Pcf11	Pcf11	PCF11 cleavage and polyadenylation factor subunit	74737	https://www.ncbi.nlm.nih.gov/gene/?term=74737
Itch	Itch	itchy, E3 ubiquitin protein ligase	16396	https://www.ncbi.nlm.nih.gov/gene/?term=16396
Ttc7	Ttc7	tetratricopeptide repeat domain 7	225049	https://www.ncbi.nlm.nih.gov/gene/?term=225049
Orc1	Orc1	origin recognition complex, subunit 1	18392	https://www.ncbi.nlm.nih.gov/gene/?term=18392
Glipr1l2	Glipr1l2	GLI pathogenesis-related 1 like 2	67537	https://www.ncbi.nlm.nih.gov/gene/?term=67537
Zfp592	Zfp592	zinc finger protein 592	233410	https://www.ncbi.nlm.nih.gov/gene/?term=233410
Mrpl39	Mrpl39	mitochondrial ribosomal protein L39	27393	https://www.ncbi.nlm.nih.gov/gene/?term=27393

Fbxl17	Fbxl17	F-box and leucine-rich repeat protein 17	50758	https://www.ncbi.nlm.nih.gov/gene/?term=50758
Mpc1	Mpc1	mitochondrial pyruvate carrier 1	55951	https://www.ncbi.nlm.nih.gov/gene/?term=55951
Rad21	Rad21	RAD21 cohesin complex component	19357	https://www.ncbi.nlm.nih.gov/gene/?term=19357
Lrig3	Lrig3	leucine-rich repeats and immunoglobulin-like domains 3	320398	https://www.ncbi.nlm.nih.gov/gene/?term=320398
Hnrnpll	Hnrnpll	heterogeneous nuclear ribonucleoprotein L-like	72692	https://www.ncbi.nlm.nih.gov/gene/?term=72692
Mid2	Mid2	midline 2	23947	https://www.ncbi.nlm.nih.gov/gene/?term=23947
Itga2	Itga2	integrin alpha 2	16398	https://www.ncbi.nlm.nih.gov/gene/?term=16398
Zfp874b	Zfp874b	zinc finger protein 874b	408067	https://www.ncbi.nlm.nih.gov/gene/?term=408067
Otud6b	Otud6b	OTU domain containing 6B	72201	https://www.ncbi.nlm.nih.gov/gene/?term=72201
Fosb	Fosb	FBJ osteosarcoma oncogene B	14282	https://www.ncbi.nlm.nih.gov/gene/?term=14282
Skint7	Skint7	selection and upkeep of intraepithelial T cells 7	328505	https://www.ncbi.nlm.nih.gov/gene/?term=328505
Eif4ebp1	Eif4ebp1	eukaryotic translation initiation factor 4E binding protein 1	13685	https://www.ncbi.nlm.nih.gov/gene/?term=13685
Aspm	Aspm	abnormal spindle microtubule assembly	12316	https://www.ncbi.nlm.nih.gov/gene/?term=12316
Mpzl1	Mpzl1	myelin protein zero-like 1	68481	https://www.ncbi.nlm.nih.gov/gene/?term=68481
Erich1	Erich1	glutamate rich 1	234086	https://www.ncbi.nlm.nih.gov/gene/?term=234086
Cep126	Cep126	centrosomal protein 126	234915	https://www.ncbi.nlm.nih.gov/gene/?term=234915
Zfand5	Zfand5	zinc finger, AN1-type domain 5	22682	https://www.ncbi.nlm.nih.gov/gene/?term=22682
Adgre4	Adgre4	adhesion G protein-coupled receptor E4	52614	https://www.ncbi.nlm.nih.gov/gene/?term=52614
Rasef	Rasef	RAS and EF hand domain containing	242505	https://www.ncbi.nlm.nih.gov/gene/?term=242505
Trhr	Trhr	thyrotropin releasing hormone receptor	22045	https://www.ncbi.nlm.nih.gov/gene/?term=22045
Lats1	Lats1	large tumor suppressor	16798	https://www.ncbi.nlm.nih.gov/gene/?term=16798

Biology Open • Supplementary information

Zfp689	Zfp689	zinc finger protein 689	71131	https://www.ncbi.nlm.nih.gov/gene/?term=71131
Phrf1	Phrf1	PHD and ring finger domains 1	101471	https://www.ncbi.nlm.nih.gov/gene/?term=101471
Nck2	Nck2	non-catalytic region of tyrosine kinase adaptor protein 2	17974	https://www.ncbi.nlm.nih.gov/gene/?term=17974
Tubgcp5	Tubgcp5	tubulin, gamma complex associated protein 5	233276	https://www.ncbi.nlm.nih.gov/gene/?term=233276
Msx2	Msx2	msh homeobox 2	17702	https://www.ncbi.nlm.nih.gov/gene/?term=17702
Ier3	Ier3	immediate early response 3	15937	https://www.ncbi.nlm.nih.gov/gene/?term=15937
Olfr658	Olfr658	olfactory receptor 658	259051	https://www.ncbi.nlm.nih.gov/gene/?term=259051
Cd53	Cd53	CD53 antigen	12508	https://www.ncbi.nlm.nih.gov/gene/?term=12508
Akap13	Akap13	A kinase (PRKA) anchor protein 13	75547	https://www.ncbi.nlm.nih.gov/gene/?term=75547
Slc5a8	Slc5a8	solute carrier family 5 (iodide transporter), member 8	216225	https://www.ncbi.nlm.nih.gov/gene/?term=216225
Nup93	Nup93	nucleoporin 93	71805	https://www.ncbi.nlm.nih.gov/gene/?term=71805
Dennd4a	Dennd4a	DENN/MADD domain containing 4A	102442	https://www.ncbi.nlm.nih.gov/gene/?term=102442
Il3	Il3	interleukin 3	16187	https://www.ncbi.nlm.nih.gov/gene/?term=16187
Hopx	Hopx	HOP homeobox	74318	https://www.ncbi.nlm.nih.gov/gene/?term=74318
Stag2	Stag2	stromal antigen 2	20843	https://www.ncbi.nlm.nih.gov/gene/?term=20843
Npr3	Npr3	natriuretic peptide receptor 3	18162	https://www.ncbi.nlm.nih.gov/gene/?term=18162
Golph3l	Golph3l	golgi phosphoprotein 3-like	229593	https://www.ncbi.nlm.nih.gov/gene/?term=229593
Rai2	Rai2	retinoic acid induced 2	24004	https://www.ncbi.nlm.nih.gov/gene/?term=24004
Nat8f2	Nat8f2	N-acetyltransferase 8 (GCN5-related) family member 2	93673	https://www.ncbi.nlm.nih.gov/gene/?term=93673
Ywhah	Ywhah	tyrosine 3-monooxygenase/tryptophan 5-monooxygenase activation protein, eta polypeptide	22629	https://www.ncbi.nlm.nih.gov/gene/?term=22629
Slc4a4	Slc4a4	solute carrier family 4 (anion exchanger), member 4	54403	https://www.ncbi.nlm.nih.gov/gene/?term=54403

Cdkn2c	Cdkn2c	cyclin dependent kinase inhibitor 2C	12580	https://www.ncbi.nlm.nih.gov/gene/?term=12580
Arhgap31	Arhgap31	Rho GTPase activating protein 31	12549	https://www.ncbi.nlm.nih.gov/gene/?term=12549
Sprr3	Sprr3	small proline-rich protein 3	20766	https://www.ncbi.nlm.nih.gov/gene/?term=20766
Ngly1	Ngly1	N-glycanase 1	59007	https://www.ncbi.nlm.nih.gov/gene/?term=59007
Sh3bgrl	Sh3bgrl	SH3-binding domain glutamic acid-rich protein like	56726	https://www.ncbi.nlm.nih.gov/gene/?term=56726
Nsmaf	Nsmaf	neutral sphingomyelinase (N-SMase) activation associated factor	18201	https://www.ncbi.nlm.nih.gov/gene/?term=18201
Chchd6	Chchd6	coiled-coil-helix-coiled-coil-helix domain containing 6	66098	https://www.ncbi.nlm.nih.gov/gene/?term=66098
Bmpr1b	Bmpr1b	bone morphogenetic protein receptor, type 1B	12167	https://www.ncbi.nlm.nih.gov/gene/?term=12167
Ift122	Ift122	intraflagellar transport 122	81896	https://www.ncbi.nlm.nih.gov/gene/?term=81896
Slc5a3	Slc5a3	solute carrier family 5 (inositol transporters), member 3	53881	https://www.ncbi.nlm.nih.gov/gene/?term=53881
Fbrsl1	Fbrsl1	fibrosin-like 1	381668	https://www.ncbi.nlm.nih.gov/gene/?term=381668
Trpm3	Trpm3	transient receptor potential cation channel, subfamily M, member 3	226025	https://www.ncbi.nlm.nih.gov/gene/?term=226025
Bmp2	Bmp2	bone morphogenetic protein 2	12156	https://www.ncbi.nlm.nih.gov/gene/?term=12156
Ocrl	Ocrl	OCRL, inositol polyphosphate-5-phosphatase	320634	https://www.ncbi.nlm.nih.gov/gene/?term=320634
Kansl1l	Kansl1l	KAT8 regulatory NSL complex subunit 1-like	68691	https://www.ncbi.nlm.nih.gov/gene/?term=68691
Myoz1	Myoz1	myozenin 1	59011	https://www.ncbi.nlm.nih.gov/gene/?term=59011
Phlda1	Phlda1	pleckstrin homology like domain, family A, member 1	21664	https://www.ncbi.nlm.nih.gov/gene/?term=21664
Shisa6	Shisa6	shisa family member 6	380702	https://www.ncbi.nlm.nih.gov/gene/?term=380702
Pom121l2	Pom121l2	POM121 membrane glycoprotein-like 2 (rat)	195236	https://www.ncbi.nlm.nih.gov/gene/?term=195236

Flt3	Flt3	FMS-like tyrosine kinase 3	14255	https://www.ncbi.nlm.nih.gov/gene/?term=14255
Foxa2	Foxa2	forkhead box A2	15376	https://www.ncbi.nlm.nih.gov/gene/?term=15376
Kazn	Kazn	kazrin, periplakin interacting protein	71529	https://www.ncbi.nlm.nih.gov/gene/?term=71529
Ubr5	Ubr5	ubiquitin protein ligase E3 component n-recognin 5	70790	https://www.ncbi.nlm.nih.gov/gene/?term=70790
Tspan14	Tspan14	tetraspanin 14	52588	https://www.ncbi.nlm.nih.gov/gene/?term=52588
Ppp4r2	Ppp4r2	protein phosphatase 4, regulatory subunit 2	232314	https://www.ncbi.nlm.nih.gov/gene/?term=232314
Capn10	Capn10	calpain 10	23830	https://www.ncbi.nlm.nih.gov/gene/?term=23830
Eya2	Eya2	EYA transcriptional coactivator and phosphatase 2	14049	https://www.ncbi.nlm.nih.gov/gene/?term=14049
Flnc	Flnc	filamin C, gamma	68794	https://www.ncbi.nlm.nih.gov/gene/?term=68794
Arhgef9	Arhgef9	CDC42 guanine nucleotide exchange factor (GEF) 9	236915	https://www.ncbi.nlm.nih.gov/gene/?term=236915
Sppl2a	Sppl2a	signal peptide peptidase like 2A	66552	https://www.ncbi.nlm.nih.gov/gene/?term=66552
Slc25a31	Slc25a31	solute carrier family 25 (mitochondrial carrier; adenine nucleotide translocator), member 31	73333	https://www.ncbi.nlm.nih.gov/gene/?term=73333
Atp8b1	Atp8b1	ATPase, class I, type 8B, member 1	54670	https://www.ncbi.nlm.nih.gov/gene/?term=54670
Mthfd2l	Mthfd2l	methylenetetrahydrofolate dehydrogenase (NADP+ dependent) 2-like	665563	https://www.ncbi.nlm.nih.gov/gene/?term=665563
Sh3tc2	Sh3tc2	SH3 domain and tetratricopeptide repeats 2	225608	https://www.ncbi.nlm.nih.gov/gene/?term=225608
Pip5k1a	Pip5k1a	phosphatidylinositol-4-phosphate 5-kinase, type 1 alpha	18720	https://www.ncbi.nlm.nih.gov/gene/?term=18720
Mpp6	Mpp6	membrane protein, palmitoylated 6 (MAGUK p55 subfamily member 6)	56524	https://www.ncbi.nlm.nih.gov/gene/?term=56524
Syndig1	Syndig1	synapse differentiation inducing 1	433485	https://www.ncbi.nlm.nih.gov/gene/?term=433485

Biology Open • Supplementary information

Ccl17	Ccl17	chemokine (C-C motif) ligand 17	20295	https://www.ncbi.nlm.nih.gov/gene/?term=20295
Atp8b4	Atp8b4	ATPase, class I, type 8B, member 4	241633	https://www.ncbi.nlm.nih.gov/gene/?term=241633
Chst5	Chst5	carbohydrate (N-acetylglucosamine 6-O) sulfotransferase 5	56773	https://www.ncbi.nlm.nih.gov/gene/?term=56773
Tox	Tox	thymocyte selection-associated high mobility group box	252838	https://www.ncbi.nlm.nih.gov/gene/?term=252838
Cyfip2	Cyfip2	cytoplasmic FMR1 interacting protein 2	76884	https://www.ncbi.nlm.nih.gov/gene/?term=76884
Nsd2	Nsd2	nuclear receptor binding SET domain protein 2	107823	https://www.ncbi.nlm.nih.gov/gene/?term=107823
Rbfox1	Rbfox1	RNA binding protein, fox-1 homolog (C. elegans) 1	268859	https://www.ncbi.nlm.nih.gov/gene/?term=268859
Sfpq	Sfpq	splicing factor proline/glutamine rich (polypyrimidine tract binding protein associated)	71514	https://www.ncbi.nlm.nih.gov/gene/?term=71514
Fgf10	Fgf10	fibroblast growth factor 10	14165	https://www.ncbi.nlm.nih.gov/gene/?term=14165
Smap1	Smap1	small ArfGAP 1	98366	https://www.ncbi.nlm.nih.gov/gene/?term=98366
Snx15	Snx15	sorting nexin 15	69024	https://www.ncbi.nlm.nih.gov/gene/?term=69024
Senp5	Senp5	SUMO/sentrin specific peptidase 5	320213	https://www.ncbi.nlm.nih.gov/gene/?term=320213
Map1b	Map1b	microtubule-associated protein 1B	17755	https://www.ncbi.nlm.nih.gov/gene/?term=17755
Sstr3	Sstr3	somatostatin receptor 3	20607	https://www.ncbi.nlm.nih.gov/gene/?term=20607
Rbm27	Rbm27	RNA binding motif protein 27	225432	https://www.ncbi.nlm.nih.gov/gene/?term=225432
Cks1brt	Cks1brt	CDC28 protein kinase 1b, retrogene	624855	https://www.ncbi.nlm.nih.gov/gene/?term=624855
Enox1	Enox1	ecto-NOX disulfide-thiol exchanger 1	239188	https://www.ncbi.nlm.nih.gov/gene/?term=239188
Srsf3	Srsf3	serine/arginine-rich splicing factor 3	20383	https://www.ncbi.nlm.nih.gov/gene/?term=20383
Cadm1	Cadm1	cell adhesion molecule 1	54725	https://www.ncbi.nlm.nih.gov/gene/?term=54725
Snrpd1	Snrpd1	small nuclear ribonucleoprotein D1	20641	https://www.ncbi.nlm.nih.gov/gene/?term=20641
Baiap2l1	Baiap2l1	BAI1-associated protein 2-like 1	66898	https://www.ncbi.nlm.nih.gov/gene/?term=66898
Glis1	Glis1	GLIS family zinc finger 1	230587	https://www.ncbi.nlm.nih.gov/gene/?term=230587

Biology Open • Supplementary information

Adgrg3	Adgrg3	adhesion G protein-coupled receptor G3	54672	https://www.ncbi.nlm.nih.gov/gene/?term=54672
Dlx3	Dlx3	distal-less homeobox 3	13393	https://www.ncbi.nlm.nih.gov/gene/?term=13393
Cops7b	Cops7b	COP9 signalosome subunit 7B	26895	https://www.ncbi.nlm.nih.gov/gene/?term=26895
Cdh7	Cdh7	cadherin 7, type 2	241201	https://www.ncbi.nlm.nih.gov/gene/?term=241201
Dcc	Dcc	deleted in colorectal carcinoma	13176	https://www.ncbi.nlm.nih.gov/gene/?term=13176
Arhgap44	Arhgap44	Rho GTPase activating protein 44	216831	https://www.ncbi.nlm.nih.gov/gene/?term=216831
Draxin	Draxin	dorsal inhibitory axon guidance protein	70433	https://www.ncbi.nlm.nih.gov/gene/?term=70433
Sp5	Sp5	trans-acting transcription factor 5	64406	https://www.ncbi.nlm.nih.gov/gene/?term=64406
Mark1	Mark1	MAP/microtubule affinity regulating kinase 1	226778	https://www.ncbi.nlm.nih.gov/gene/?term=226778
Tor1aip2	Tor1aip2	torsin A interacting protein 2	240832	https://www.ncbi.nlm.nih.gov/gene/?term=240832
Fmn1	Fmn1	formin 1	14260	https://www.ncbi.nlm.nih.gov/gene/?term=14260
Rab12	Rab12	RAB12, member RAS oncogene family	19328	https://www.ncbi.nlm.nih.gov/gene/?term=19328
Gna11	Gna11	guanine nucleotide binding protein, alpha 11	14672	https://www.ncbi.nlm.nih.gov/gene/?term=14672
Egfl6	Egfl6	EGF-like-domain, multiple 6	54156	https://www.ncbi.nlm.nih.gov/gene/?term=54156
Slc37a3	Slc37a3	solute carrier family 37 (glycerol-3-phosphate transporter), member 3	72144	https://www.ncbi.nlm.nih.gov/gene/?term=72144
Ptpn23	Ptpn23	protein tyrosine phosphatase, non-receptor type 23	104831	https://www.ncbi.nlm.nih.gov/gene/?term=104831
Il6st	Il6st	interleukin 6 signal transducer	16195	https://www.ncbi.nlm.nih.gov/gene/?term=16195
Fmnl2	Fmnl2	formin-like 2	71409	https://www.ncbi.nlm.nih.gov/gene/?term=71409
Bod1	Bod1	biorientation of chromosomes in cell division 1	69556	https://www.ncbi.nlm.nih.gov/gene/?term=69556
Hgs	Hgs	HGF-regulated tyrosine kinase substrate	15239	https://www.ncbi.nlm.nih.gov/gene/?term=15239

Akt1s1	Akt1s1	AKT1 substrate 1 (proline-rich)	67605	https://www.ncbi.nlm.nih.gov/gene/?term=67605
Arntl	Arntl	aryl hydrocarbon receptor nuclear translocator-like	11865	https://www.ncbi.nlm.nih.gov/gene/?term=11865
Gorab	Gorab	golgin, RAB6-interacting	98376	https://www.ncbi.nlm.nih.gov/gene/?term=98376
Smug1	Smug1	single-strand selective monofunctional uracil DNA glycosylase	71726	https://www.ncbi.nlm.nih.gov/gene/?term=71726
Hectd1	Hectd1	HECT domain E3 ubiquitin protein ligase 1	207304	https://www.ncbi.nlm.nih.gov/gene/?term=207304
Tmem59	Tmem59	transmembrane protein 59	56374	https://www.ncbi.nlm.nih.gov/gene/?term=56374
Osbp	Osbp	oxysterol binding protein	76303	https://www.ncbi.nlm.nih.gov/gene/?term=76303
Aqp4	Aqp4	aquaporin 4	11829	https://www.ncbi.nlm.nih.gov/gene/?term=11829
Golga7b	Golga7b	golgi autoantigen, golgin subfamily a, 7B	71146	https://www.ncbi.nlm.nih.gov/gene/?term=71146
Dpf2	Dpf2	D4, zinc and double PHD fingers family 2	19708	https://www.ncbi.nlm.nih.gov/gene/?term=19708
Prl7b1	Prl7b1	prolactin family 7, subfamily b, member 1	75596	https://www.ncbi.nlm.nih.gov/gene/?term=75596
Acbd6	Acbd6	acyl-Coenzyme A binding domain containing 6	72482	https://www.ncbi.nlm.nih.gov/gene/?term=72482
Tspan18	Tspan18	tetraspanin 18	241556	https://www.ncbi.nlm.nih.gov/gene/?term=241556
Dnajb4	Dnajb4	DnaJ heat shock protein family (Hsp40) member B4	67035	https://www.ncbi.nlm.nih.gov/gene/?term=67035
Krtap13	Krtap13	keratin associated protein 13	16699	https://www.ncbi.nlm.nih.gov/gene/?term=16699
Htra3	Htra3	HtrA serine peptidase 3	78558	https://www.ncbi.nlm.nih.gov/gene/?term=78558
Sesn2	Sesn2	sestrin 2	230784	https://www.ncbi.nlm.nih.gov/gene/?term=230784
Gatad2b	Gatad2b	GATA zinc finger domain containing 2B	229542	https://www.ncbi.nlm.nih.gov/gene/?term=229542
Slc17a2	Slc17a2	solute carrier family 17 (sodium phosphate), member 2	218103	https://www.ncbi.nlm.nih.gov/gene/?term=218103

Cyb561a3	Cyb561a3	cytochrome b561 family, member A3	225912	https://www.ncbi.nlm.nih.gov/gene/?term=225912
Fam117a	Fam117a	family with sequence similarity 117, member A	215512	https://www.ncbi.nlm.nih.gov/gene/?term=215512
Map4k1	Map4k1	mitogen-activated protein kinase kinase kinase kinase 1	26411	https://www.ncbi.nlm.nih.gov/gene/?term=26411
Nampt	Nampt	nicotinamide phosphoribosyltransferase	59027	https://www.ncbi.nlm.nih.gov/gene/?term=59027
Tnfrsf23	Tnfrsf23	tumor necrosis factor receptor superfamily, member 23	79201	https://www.ncbi.nlm.nih.gov/gene/?term=79201
Vma21	Vma21	VMA21 vacuolar H+-ATPase homolog (S. cerevisiae)	67048	https://www.ncbi.nlm.nih.gov/gene/?term=67048
Tnfrsf22	Tnfrsf22	tumor necrosis factor receptor superfamily, member 22	79202	https://www.ncbi.nlm.nih.gov/gene/?term=79202
Clta	Clta	clathrin, light polypeptide (Lca)	12757	https://www.ncbi.nlm.nih.gov/gene/?term=12757
Fads1	Fads1	fatty acid desaturase 1	76267	https://www.ncbi.nlm.nih.gov/gene/?term=76267
Plpp2	Plpp2	phospholipid phosphatase 2	50784	https://www.ncbi.nlm.nih.gov/gene/?term=50784
Arrb1	Arrb1	arrestin, beta 1	109689	https://www.ncbi.nlm.nih.gov/gene/?term=109689
Timm17a	Timm17a	translocase of inner mitochondrial membrane 17a	21854	https://www.ncbi.nlm.nih.gov/gene/?term=21854
Virma	Virma	vir like m6A methyltransferase associated	66185	https://www.ncbi.nlm.nih.gov/gene/?term=66185
Rbmx	Rbmx	RNA binding motif protein, X chromosome	19655	https://www.ncbi.nlm.nih.gov/gene/?term=19655
Map4	Map4	microtubule-associated protein 4	17758	https://www.ncbi.nlm.nih.gov/gene/?term=17758
Sertad4	Sertad4	SERTA domain containing 4	214791	https://www.ncbi.nlm.nih.gov/gene/?term=214791
Ptprz1	Ptprz1	protein tyrosine phosphatase, receptor type Z, polypeptide 1	19283	https://www.ncbi.nlm.nih.gov/gene/?term=19283
Sgpp1	Sgpp1	sphingosine-1-phosphate phosphatase 1	81535	https://www.ncbi.nlm.nih.gov/gene/?term=81535

Biology Open • Supplementary information

Gabpb2	Gabpb2	GA repeat binding protein, beta 2	213054	https://www.ncbi.nlm.nih.gov/gene/?term=213054
Rffl	Rffl	ring finger and FYVE like domain containing protein	67338	https://www.ncbi.nlm.nih.gov/gene/?term=67338
Sec61a1	Sec61a1	Sec61 alpha 1 subunit (S. cerevisiae)	53421	https://www.ncbi.nlm.nih.gov/gene/?term=53421
Tagln2	Tagln2	transgelin 2	21346	https://www.ncbi.nlm.nih.gov/gene/?term=21346
Shisa5	Shisa5	shisa family member 5	66940	https://www.ncbi.nlm.nih.gov/gene/?term=66940
Otc	Otc	ornithine transcarbamylase	18416	https://www.ncbi.nlm.nih.gov/gene/?term=18416
Actn4	Actn4	actinin alpha 4	60595	https://www.ncbi.nlm.nih.gov/gene/?term=60595
Tfap2d	Tfap2d	transcription factor AP-2, delta	226896	https://www.ncbi.nlm.nih.gov/gene/?term=226896
Praf2	Praf2	PRA1 domain family 2	54637	https://www.ncbi.nlm.nih.gov/gene/?term=54637
Enc1	Enc1	ectodermal-neural cortex 1	13803	https://www.ncbi.nlm.nih.gov/gene/?term=13803
Lasp1	Lasp1	LIM and SH3 protein 1	16796	https://www.ncbi.nlm.nih.gov/gene/?term=16796
Vps54	Vps54	VPS54 GARP complex subunit	245944	https://www.ncbi.nlm.nih.gov/gene/?term=245944
Tax1bp1	Tax1bp1	Tax1 (human T cell leukemia virus type I) binding protein 1	52440	https://www.ncbi.nlm.nih.gov/gene/?term=52440
Maml1	Maml1	mastermind like transcriptional coactivator 1	103806	https://www.ncbi.nlm.nih.gov/gene/?term=103806
Ptbp1	Ptbp1	polypyrimidine tract binding protein 1	19205	https://www.ncbi.nlm.nih.gov/gene/?term=19205
Fam117b	Fam117b	family with sequence similarity 117, member B	72750	https://www.ncbi.nlm.nih.gov/gene/?term=72750
Ankrd28	Ankrd28	ankyrin repeat domain 28	105522	https://www.ncbi.nlm.nih.gov/gene/?term=105522
Elfn1	Elfn1	leucine rich repeat and fibronectin type III, extracellular 1	243312	https://www.ncbi.nlm.nih.gov/gene/?term=243312
P2rx4	P2rx4	purinergic receptor P2X, ligand-gated ion channel 4	18438	https://www.ncbi.nlm.nih.gov/gene/?term=18438
Garnl3	Garnl3	GTPase activating RANGAP domain-like 3	99326	https://www.ncbi.nlm.nih.gov/gene/?term=99326

Biology Open • Supplementary information

Hsd17b11	Hsd17b11	hydroxysteroid (17-beta) dehydrogenase 11	114664	https://www.ncbi.nlm.nih.gov/gene/?term=114664
Zc3h11a	Zc3h11a	zinc finger CCCH type containing 11A	70579	https://www.ncbi.nlm.nih.gov/gene/?term=70579
Smarcd1	Smarcd1	SWI/SNF related, matrix associated, actin dependent regulator of chromatin, subfamily d, member 1	83797	https://www.ncbi.nlm.nih.gov/gene/?term=83797
Sec61b	Sec61b	Sec61 beta subunit	66212	https://www.ncbi.nlm.nih.gov/gene/?term=66212
Ppp2cb	Ppp2cb	protein phosphatase 2 (formerly 2A), catalytic subunit, beta isoform	19053	https://www.ncbi.nlm.nih.gov/gene/?term=19053
Acat3	Acat3	acetyl-Coenzyme A acetyltransferase 3	224530	https://www.ncbi.nlm.nih.gov/gene/?term=224530
Uba2	Uba2	ubiquitin-like modifier activating enzyme 2	50995	https://www.ncbi.nlm.nih.gov/gene/?term=50995
Dpysl4	Dpysl4	dihydropyrimidinase-like 4	26757	https://www.ncbi.nlm.nih.gov/gene/?term=26757
Yes1	Yes1	YES proto-oncogene 1, Src family tyrosine kinase	22612	https://www.ncbi.nlm.nih.gov/gene/?term=22612
Gabarapl1	Gabarapl1	gamma-aminobutyric acid (GABA) A receptor-associated protein-like 1	57436	https://www.ncbi.nlm.nih.gov/gene/?term=57436
Glra2	Glra2	glycine receptor, alpha 2 subunit	237213	https://www.ncbi.nlm.nih.gov/gene/?term=237213
Eif4a1	Eif4a1	eukaryotic translation initiation factor 4A1	13681	https://www.ncbi.nlm.nih.gov/gene/?term=13681
Emp2	Emp2	epithelial membrane protein 2	13731	https://www.ncbi.nlm.nih.gov/gene/?term=13731
Tspan15	Tspan15	tetraspanin 15	70423	https://www.ncbi.nlm.nih.gov/gene/?term=70423
Itm2a	Itm2a	integral membrane protein 2A	16431	https://www.ncbi.nlm.nih.gov/gene/?term=16431
Ap5s1	Ap5s1	adaptor-related protein 5 complex, sigma 1 subunit	69596	https://www.ncbi.nlm.nih.gov/gene/?term=69596
Xkrx	Xkrx	X-linked Kx blood group related, X-linked	331524	https://www.ncbi.nlm.nih.gov/gene/?term=331524
Phactr4	Phactr4	phosphatase and actin regulator 4	100169	https://www.ncbi.nlm.nih.gov/gene/?term=100169

Biology Open • Supplementary information

5730455P16Rik	5730455P16Rik	RIKEN cDNA 5730455P16 gene	70591	https://www.ncbi.nlm.nih.gov/gene/?term=70591
Inpp4a	Inpp4a	inositol polyphosphate-4-phosphatase, type I	269180	https://www.ncbi.nlm.nih.gov/gene/?term=269180
Syvn1	Syvn1	synovial apoptosis inhibitor 1, synoviolin	74126	https://www.ncbi.nlm.nih.gov/gene/?term=74126
Vps26a	Vps26a	VPS26 retromer complex component A	30930	https://www.ncbi.nlm.nih.gov/gene/?term=30930
Efna5	Efna5	ephrin A5	13640	https://www.ncbi.nlm.nih.gov/gene/?term=13640
Hs6st2	Hs6st2	heparan sulfate 6-O-sulfotransferase 2	50786	https://www.ncbi.nlm.nih.gov/gene/?term=50786
Zfx	Zfx	zinc finger protein X-linked	22764	https://www.ncbi.nlm.nih.gov/gene/?term=22764
Jph1	Jph1	junctophilin 1	57339	https://www.ncbi.nlm.nih.gov/gene/?term=57339
Camk1d	Camk1d	calcium/calmodulin-dependent protein kinase ID	227541	https://www.ncbi.nlm.nih.gov/gene/?term=227541
Stx12	Stx12	syntaxin 12	100226	https://www.ncbi.nlm.nih.gov/gene/?term=100226
Tec	Tec	tec protein tyrosine kinase	21682	https://www.ncbi.nlm.nih.gov/gene/?term=21682
Alg1	Alg1	asparagine-linked glycosylation 1 (beta-1,4-mannosyltransferase)	208211	https://www.ncbi.nlm.nih.gov/gene/?term=208211
Zbtb49	Zbtb49	zinc finger and BTB domain containing 49	75079	https://www.ncbi.nlm.nih.gov/gene/?term=75079
Grhl3	Grhl3	grainyhead like transcription factor 3	230824	https://www.ncbi.nlm.nih.gov/gene/?term=230824
Bdnf	Bdnf	brain derived neurotrophic factor	12064	https://www.ncbi.nlm.nih.gov/gene/?term=12064
Zmym3	Zmym3	zinc finger, MYM-type 3	56364	https://www.ncbi.nlm.nih.gov/gene/?term=56364
Utp18	Utp18	UTP18 small subunit processome component	217109	https://www.ncbi.nlm.nih.gov/gene/?term=217109
Twist2	Twist2	twist basic helix-loop-helix transcription factor 2	13345	https://www.ncbi.nlm.nih.gov/gene/?term=13345
Itga4	Itga4	integrin alpha 4	16401	https://www.ncbi.nlm.nih.gov/gene/?term=16401
BC048403	BC048403	cDNA sequence BC048403	270802	https://www.ncbi.nlm.nih.gov/gene/?term=270802

Biology Open • Supplementary information

Fzd8	Fzd8	frizzled class receptor 8	14370	https://www.ncbi.nlm.nih.gov/gene/?term=14370
Adamtsl3	Adamtsl3	ADAMTS-like 3	269959	https://www.ncbi.nlm.nih.gov/gene/?term=269959
Zfp236	Zfp236	zinc finger protein 236	329002	https://www.ncbi.nlm.nih.gov/gene/?term=329002
Zfp182	Zfp182	zinc finger protein 182	319535	https://www.ncbi.nlm.nih.gov/gene/?term=319535
Rpain	Rpain	RPA interacting protein	69723	https://www.ncbi.nlm.nih.gov/gene/?term=69723
Hnrnpr	Hnrnpr	heterogeneous nuclear ribonucleoprotein R	74326	https://www.ncbi.nlm.nih.gov/gene/?term=74326
Slc28a3	Slc28a3	solute carrier family 28 (sodium-coupled nucleoside transporter), member 3	114304	https://www.ncbi.nlm.nih.gov/gene/?term=114304
Bche	Bche	butyrylcholinesterase	12038	https://www.ncbi.nlm.nih.gov/gene/?term=12038
Fbxo30	Fbxo30	F-box protein 30	71865	https://www.ncbi.nlm.nih.gov/gene/?term=71865
Dll1	Dll1	delta like canonical Notch ligand 1	13388	https://www.ncbi.nlm.nih.gov/gene/?term=13388
Tmem33	Tmem33	transmembrane protein 33	67878	https://www.ncbi.nlm.nih.gov/gene/?term=67878
Cnnm4	Cnnm4	cyclin M4	94220	https://www.ncbi.nlm.nih.gov/gene/?term=94220
Cflar	Cflar	CASP8 and FADD-like apoptosis regulator	12633	https://www.ncbi.nlm.nih.gov/gene/?term=12633
Rbbp9	Rbbp9	retinoblastoma binding protein 9, serine hydrolase	26450	https://www.ncbi.nlm.nih.gov/gene/?term=26450
Id1	Id1	inhibitor of DNA binding 1	15901	https://www.ncbi.nlm.nih.gov/gene/?term=15901
Ralyl	Ralyl	RALY RNA binding protein-like	76897	https://www.ncbi.nlm.nih.gov/gene/?term=76897
Fgf12	Fgf12	fibroblast growth factor 12	14167	https://www.ncbi.nlm.nih.gov/gene/?term=14167
Dlx4	Dlx4	distal-less homeobox 4	13394	https://www.ncbi.nlm.nih.gov/gene/?term=13394
Ccdc185	Ccdc185	coiled-coil domain containing 185	433386	https://www.ncbi.nlm.nih.gov/gene/?term=433386
Ddx60	Ddx60	DEAD (Asp-Glu-Ala-Asp) box polypeptide 60	234311	https://www.ncbi.nlm.nih.gov/gene/?term=234311
Ppp1r21	Ppp1r21	protein phosphatase 1, regulatory subunit 21	73825	https://www.ncbi.nlm.nih.gov/gene/?term=73825
Dclk2	Dclk2	doublecortin-like kinase 2	70762	https://www.ncbi.nlm.nih.gov/gene/?term=70762

Biology Open • Supplementary information

Vmp1	Vmp1	vacuole membrane protein 1	75909	https://www.ncbi.nlm.nih.gov/gene/?term=75909
Timm10	Timm10	translocase of inner mitochondrial membrane 10	30059	https://www.ncbi.nlm.nih.gov/gene/?term=30059
Atad5	Atad5	ATPase family, AAA domain containing 5	237877	https://www.ncbi.nlm.nih.gov/gene/?term=237877
Rbl1	Rbl1	RB transcriptional corepressor like 1	19650	https://www.ncbi.nlm.nih.gov/gene/?term=19650
Zic1	Zic1	zinc finger protein of the cerebellum 1	22771	https://www.ncbi.nlm.nih.gov/gene/?term=22771
Slc7a1	Slc7a1	solute carrier family 7 (cationic amino acid transporter, y+ system), member 1	11987	https://www.ncbi.nlm.nih.gov/gene/?term=11987
Anks1b	Anks1b	ankyrin repeat and sterile alpha motif domain containing 1B	77531	https://www.ncbi.nlm.nih.gov/gene/?term=77531
Gpcpd1	Gpcpd1	glycerophosphocholine phosphodiesterase 1	74182	https://www.ncbi.nlm.nih.gov/gene/?term=74182
Snrk	Snrk	SNF related kinase	20623	https://www.ncbi.nlm.nih.gov/gene/?term=20623
Kit	Kit	KIT proto-oncogene receptor tyrosine kinase	16590	https://www.ncbi.nlm.nih.gov/gene/?term=16590
Hmgxb4	Hmgxb4	HMG box domain containing 4	70823	https://www.ncbi.nlm.nih.gov/gene/?term=70823
Sez6l	Sez6l	seizure related 6 homolog like	56747	https://www.ncbi.nlm.nih.gov/gene/?term=56747
Myf6	Myf6	myogenic factor 6	17878	https://www.ncbi.nlm.nih.gov/gene/?term=17878
Mbd5	Mbd5	methyl-CpG binding domain protein 5	109241	https://www.ncbi.nlm.nih.gov/gene/?term=109241
Ptpra	Ptpra	protein tyrosine phosphatase, receptor type, A	19262	https://www.ncbi.nlm.nih.gov/gene/?term=19262
Ifngr1	Ifngr1	interferon gamma receptor 1	15979	https://www.ncbi.nlm.nih.gov/gene/?term=15979
Ice1	Ice1	interactor of little elongation complex ELL subunit 1	218333	https://www.ncbi.nlm.nih.gov/gene/?term=218333
Irx3	Irx3	Iroquois related homeobox 3	16373	https://www.ncbi.nlm.nih.gov/gene/?term=16373

Biology Open • Supplementary information

Msantd2	Msantd2	Myb/SANT-like DNA-binding domain containing 2	235184	https://www.ncbi.nlm.nih.gov/gene/?term=235184
Atad1	Atad1	ATPase family, AAA domain containing 1	67979	https://www.ncbi.nlm.nih.gov/gene/?term=67979
Tek	Tek	TEK receptor tyrosine kinase	21687	https://www.ncbi.nlm.nih.gov/gene/?term=21687
Yy1	Yy1	YY1 transcription factor	22632	https://www.ncbi.nlm.nih.gov/gene/?term=22632
Pdzrn3	Pdzrn3	PDZ domain containing RING finger 3	55983	https://www.ncbi.nlm.nih.gov/gene/?term=55983
Akap12	Akap12	A kinase (PRKA) anchor protein (gravin) 12	83397	https://www.ncbi.nlm.nih.gov/gene/?term=83397
Acr	Acr	acrosin prepropeptide	11434	https://www.ncbi.nlm.nih.gov/gene/?term=11434
Asf1a	Asf1a	anti-silencing function 1A histone chaperone	66403	https://www.ncbi.nlm.nih.gov/gene/?term=66403
Kdm6a	Kdm6a	lysine (K)-specific demethylase 6A	22289	https://www.ncbi.nlm.nih.gov/gene/?term=22289
Sorcs3	Sorcs3	sortilin-related VPS10 domain containing receptor 3	66673	https://www.ncbi.nlm.nih.gov/gene/?term=66673
Ccna2	Ccna2	cyclin A2	12428	https://www.ncbi.nlm.nih.gov/gene/?term=12428
Usp31	Usp31	ubiquitin specific peptidase 31	76179	https://www.ncbi.nlm.nih.gov/gene/?term=76179
Klf2	Klf2	Kruppel-like factor 2 (lung)	16598	https://www.ncbi.nlm.nih.gov/gene/?term=16598
Ccn3	Ccn3	cellular communication network factor 3	18133	https://www.ncbi.nlm.nih.gov/gene/?term=18133
Pcsk2	Pcsk2	proprotein convertase subtilisin/kexin type 2	18549	https://www.ncbi.nlm.nih.gov/gene/?term=18549
1110059E24Rik	1110059E24Rik	RIKEN cDNA 1110059E24 gene	66206	https://www.ncbi.nlm.nih.gov/gene/?term=66206
Nfkbia	Nfkbia	nuclear factor of kappa light polypeptide gene enhancer in B cells inhibitor, alpha	18035	https://www.ncbi.nlm.nih.gov/gene/?term=18035
Ensa	Ensa	endosulfine alpha	56205	https://www.ncbi.nlm.nih.gov/gene/?term=56205
Golga2	Golga2	golgi autoantigen, golgin subfamily a, 2	99412	https://www.ncbi.nlm.nih.gov/gene/?term=99412

Biology Open • Supplementary information

Malt1	Malt1	MALT1 paracaspase	240354	https://www.ncbi.nlm.nih.gov/gene/?term=240354
Snx14	Snx14	sorting nexin 14	244962	https://www.ncbi.nlm.nih.gov/gene/?term=244962
Tm4sf4	Tm4sf4	transmembrane 4 superfamily member 4	229302	https://www.ncbi.nlm.nih.gov/gene/?term=229302
Zfp248	Zfp248	zinc finger protein 248	72720	https://www.ncbi.nlm.nih.gov/gene/?term=72720
Ube2w	Ube2w	ubiquitin-conjugating enzyme E2W (putative)	66799	https://www.ncbi.nlm.nih.gov/gene/?term=66799
Tm9sf3	Tm9sf3	transmembrane 9 superfamily member 3	107358	https://www.ncbi.nlm.nih.gov/gene/?term=107358
Fbxo43	Fbxo43	F-box protein 43	78803	https://www.ncbi.nlm.nih.gov/gene/?term=78803
Rbm5	Rbm5	RNA binding motif protein 5	83486	https://www.ncbi.nlm.nih.gov/gene/?term=83486
Pitpnb	Pitpnb	phosphatidylinositol transfer protein, beta	56305	https://www.ncbi.nlm.nih.gov/gene/?term=56305
Cab39	Cab39	calcium binding protein 39	12283	https://www.ncbi.nlm.nih.gov/gene/?term=12283
Wipf3	Wipf3	WAS/WASL interacting protein family, member 3	330319	https://www.ncbi.nlm.nih.gov/gene/?term=330319
Slc38a1	Slc38a1	solute carrier family 38, member 1	105727	https://www.ncbi.nlm.nih.gov/gene/?term=105727
Trh	Trh	thyrotropin releasing hormone	22044	https://www.ncbi.nlm.nih.gov/gene/?term=22044
Psma3	Psma3	proteasome (prosome, macropain) subunit, alpha type 3	19167	https://www.ncbi.nlm.nih.gov/gene/?term=19167
Eid2	Eid2	EP300 interacting inhibitor of differentiation 2	386655	https://www.ncbi.nlm.nih.gov/gene/?term=386655
Pias1	Pias1	protein inhibitor of activated STAT 1	56469	https://www.ncbi.nlm.nih.gov/gene/?term=56469
Lgr4	Lgr4	leucine-rich repeat-containing G protein-coupled receptor 4	107515	https://www.ncbi.nlm.nih.gov/gene/?term=107515
Pcdh8	Pcdh8	protocadherin 8	18530	https://www.ncbi.nlm.nih.gov/gene/?term=18530
Zswim6	Zswim6	zinc finger SWIM-type containing 6	67263	https://www.ncbi.nlm.nih.gov/gene/?term=67263
Slitrk6	Slitrk6	SLIT and NTRK-like family, member 6	239250	https://www.ncbi.nlm.nih.gov/gene/?term=239250
Prdm16	Prdm16	PR domain containing 16	70673	https://www.ncbi.nlm.nih.gov/gene/?term=70673

Foxd4	Foxd4	forkhead box D4	14237	https://www.ncbi.nlm.nih.gov/gene/?term=14237
Igfbp1	Igfbp1	insulin-like growth factor binding protein 1	16006	https://www.ncbi.nlm.nih.gov/gene/?term=16006
Fam76b	Fam76b	family with sequence similarity 76, member B	72826	https://www.ncbi.nlm.nih.gov/gene/?term=72826
Slc5a1	Slc5a1	solute carrier family 5 (sodium/glucose cotransporter), member 1	20537	https://www.ncbi.nlm.nih.gov/gene/?term=20537
Spindoc	Spindoc	spindlin interactor and repressor of chromatin binding	68229	https://www.ncbi.nlm.nih.gov/gene/?term=68229
Srgap3	Srgap3	SLIT-ROBO Rho GTPase activating protein 3	259302	https://www.ncbi.nlm.nih.gov/gene/?term=259302
Stk24	Stk24	serine/threonine kinase 24	223255	https://www.ncbi.nlm.nih.gov/gene/?term=223255
Rfx8	Rfx8	regulatory factor X 8	619289	https://www.ncbi.nlm.nih.gov/gene/?term=619289
Adam22	Adam22	a disintegrin and metallopeptidase domain 22	11496	https://www.ncbi.nlm.nih.gov/gene/?term=11496
Abca14	Abca14	ATP-binding cassette, sub-family A (ABC1), member 14	67928	https://www.ncbi.nlm.nih.gov/gene/?term=67928
Rnf4	Rnf4	ring finger protein 4	19822	https://www.ncbi.nlm.nih.gov/gene/?term=19822
Grk5	Grk5	G protein-coupled receptor kinase 5	14773	https://www.ncbi.nlm.nih.gov/gene/?term=14773
Lrrc42	Lrrc42	leucine rich repeat containing 42	77809	https://www.ncbi.nlm.nih.gov/gene/?term=77809
Ahdc1	Ahdc1	AT hook, DNA binding motif, containing 1	230793	https://www.ncbi.nlm.nih.gov/gene/?term=230793
Tspoap1	Tspoap1	TSPO associated protein 1	207777	https://www.ncbi.nlm.nih.gov/gene/?term=207777
Zc2hc1a	Zc2hc1a	zinc finger, C2HC-type containing 1A	67306	https://www.ncbi.nlm.nih.gov/gene/?term=67306
Aqr	Aqr	aquarius	11834	https://www.ncbi.nlm.nih.gov/gene/?term=11834
Slc6a19	Slc6a19	solute carrier family 6 (neurotransmitter transporter), member 19	74338	https://www.ncbi.nlm.nih.gov/gene/?term=74338

Biology Open • Supplementary information

Kcna2	Kcna2	potassium voltage-gated channel, shaker-related subfamily, member 2	16490	https://www.ncbi.nlm.nih.gov/gene/?term=16490
Tspan2	Tspan2	tetraspanin 2	70747	https://www.ncbi.nlm.nih.gov/gene/?term=70747
Kcnh5	Kcnh5	potassium voltage-gated channel, subfamily H (eag-related), member 5	238271	https://www.ncbi.nlm.nih.gov/gene/?term=238271
Dnm3	Dnm3	dynamin 3	103967	https://www.ncbi.nlm.nih.gov/gene/?term=103967
Kbtbd2	Kbtbd2	kelch repeat and BTB (POZ) domain containing 2	210973	https://www.ncbi.nlm.nih.gov/gene/?term=210973
Gabrb2	Gabrb2	gamma-aminobutyric acid (GABA) A receptor, subunit beta 2	14401	https://www.ncbi.nlm.nih.gov/gene/?term=14401
Denr	Denr	density-regulated protein	68184	https://www.ncbi.nlm.nih.gov/gene/?term=68184
Kif11	Kif11	kinesin family member 11	16551	https://www.ncbi.nlm.nih.gov/gene/?term=16551
Slc25a16	Slc25a16	solute carrier family 25 (mitochondrial carrier, Graves disease autoantigen), member 16	73132	https://www.ncbi.nlm.nih.gov/gene/?term=73132
Hivep2	Hivep2	human immunodeficiency virus type I enhancer binding protein 2	15273	https://www.ncbi.nlm.nih.gov/gene/?term=15273
Mysm1	Mysm1	myb-like, SWIRM and MPN domains 1	320713	https://www.ncbi.nlm.nih.gov/gene/?term=320713
Ppp1r15b	Ppp1r15b	protein phosphatase 1, regulatory subunit 15B	108954	https://www.ncbi.nlm.nih.gov/gene/?term=108954
Htr5a	Htr5a	5-hydroxytryptamine (serotonin) receptor 5A	15563	https://www.ncbi.nlm.nih.gov/gene/?term=15563
Arl8a	Arl8a	ADP-ribosylation factor-like 8A	68724	https://www.ncbi.nlm.nih.gov/gene/?term=68724
Prickle1	Prickle1	prickle planar cell polarity protein 1	106042	https://www.ncbi.nlm.nih.gov/gene/?term=106042
Foxa1	Foxa1	forkhead box A1	15375	https://www.ncbi.nlm.nih.gov/gene/?term=15375
Mbd2	Mbd2	methyl-CpG binding domain protein 2	17191	https://www.ncbi.nlm.nih.gov/gene/?term=17191
Traf3ip1	Traf3ip1	TRAF3 interacting protein 1	74019	https://www.ncbi.nlm.nih.gov/gene/?term=74019

Biology Open • Supplementary information

Arl14epl	Arl14epl	ADP-ribosylation factor-like 14 effector protein-like	381142	https://www.ncbi.nlm.nih.gov/gene/?term=381142
Lmo3	Lmo3	LIM domain only 3	109593	https://www.ncbi.nlm.nih.gov/gene/?term=109593
Dock3	Dock3	dedicator of cyto-kinesis 3	208869	https://www.ncbi.nlm.nih.gov/gene/?term=208869
Syngr3	Syngr3	synaptogyrin 3	20974	https://www.ncbi.nlm.nih.gov/gene/?term=20974
Ripply2	Ripply2	ripply transcriptional repressor 2	382089	https://www.ncbi.nlm.nih.gov/gene/?term=382089
Rab18	Rab18	RAB18, member RAS oncogene family	19330	https://www.ncbi.nlm.nih.gov/gene/?term=19330
She	She	src homology 2 domain-containing transforming protein E	214547	https://www.ncbi.nlm.nih.gov/gene/?term=214547
Morn4	Morn4	MORN repeat containing 4	226123	https://www.ncbi.nlm.nih.gov/gene/?term=226123
6030498E09Rik	6030498E09Rik	RIKEN cDNA 6030498E09 gene	77883	https://www.ncbi.nlm.nih.gov/gene/?term=77883
Ints10	Ints10	integrator complex subunit 10	70885	https://www.ncbi.nlm.nih.gov/gene/?term=70885
Cwc25	Cwc25	CWC25 spliceosome-associated protein	67480	https://www.ncbi.nlm.nih.gov/gene/?term=67480
Pigm	Pigm	phosphatidylinositol glycan anchor biosynthesis, class M	67556	https://www.ncbi.nlm.nih.gov/gene/?term=67556
Slc23a2	Slc23a2	solute carrier family 23 (nucleobase transporters), member 2	54338	https://www.ncbi.nlm.nih.gov/gene/?term=54338
Zfyve27	Zfyve27	zinc finger, FYVE domain containing 27	319740	https://www.ncbi.nlm.nih.gov/gene/?term=319740
Iigp1	Iigp1	interferon inducible GTPase 1	60440	https://www.ncbi.nlm.nih.gov/gene/?term=60440
Kat6a	Kat6a	K(lysine) acetyltransferase 6A	244349	https://www.ncbi.nlm.nih.gov/gene/?term=244349
Dzip3	Dzip3	DAZ interacting protein 3, zinc finger	224170	https://www.ncbi.nlm.nih.gov/gene/?term=224170
Ndrg4	Ndrg4	N-myc downstream regulated gene 4	234593	https://www.ncbi.nlm.nih.gov/gene/?term=234593
Gdf10	Gdf10	growth differentiation factor 10	14560	https://www.ncbi.nlm.nih.gov/gene/?term=14560
Pde4d	Pde4d	phosphodiesterase 4D, cAMP specific	238871	https://www.ncbi.nlm.nih.gov/gene/?term=238871
Pcdh10	Pcdh10	protocadherin 10	18526	https://www.ncbi.nlm.nih.gov/gene/?term=18526

Biology Open • Supplementary information

Dip2c	Dip2c	disco interacting protein 2 homolog C	208440	https://www.ncbi.nlm.nih.gov/gene/?term=208440
Meis2	Meis2	Meis homeobox 2	17536	https://www.ncbi.nlm.nih.gov/gene/?term=17536
Psph	Psph	phosphoserine phosphatase	100678	https://www.ncbi.nlm.nih.gov/gene/?term=100678
Birc6	Birc6	baculoviral IAP repeat-containing 6	12211	https://www.ncbi.nlm.nih.gov/gene/?term=12211
Arx	Arx	aristaless related homeobox	11878	https://www.ncbi.nlm.nih.gov/gene/?term=11878
Fos	Fos	FBJ osteosarcoma oncogene	14281	https://www.ncbi.nlm.nih.gov/gene/?term=14281
Ofd1	Ofd1	OFD1, centriole and centriolar satellite protein	237222	https://www.ncbi.nlm.nih.gov/gene/?term=237222
Akirin2	Akirin2	akirin 2	433693	https://www.ncbi.nlm.nih.gov/gene/?term=433693
Pde3a	Pde3a	phosphodiesterase 3A, cGMP inhibited	54611	https://www.ncbi.nlm.nih.gov/gene/?term=54611
Tgif1	Tgif1	TGFB-induced factor homeobox 1	21815	https://www.ncbi.nlm.nih.gov/gene/?term=21815
Slk	Slk	STE20-like kinase	20874	https://www.ncbi.nlm.nih.gov/gene/?term=20874
Gpr171	Gpr171	G protein-coupled receptor 171	229323	https://www.ncbi.nlm.nih.gov/gene/?term=229323
Rab6a	Rab6a	RAB6A, member RAS oncogene family	19346	https://www.ncbi.nlm.nih.gov/gene/?term=19346
Scg2	Scg2	secretogranin II	20254	https://www.ncbi.nlm.nih.gov/gene/?term=20254
Gm17359	Gm17359	predicted gene, 17359	100233207	https://www.ncbi.nlm.nih.gov/gene/?term=100233
Tm4sf20	Tm4sf20	transmembrane 4 L six family member 20	66261	https://www.ncbi.nlm.nih.gov/gene/?term=66261
Cox16	Cox16	cytochrome c oxidase assembly protein 16	66272	https://www.ncbi.nlm.nih.gov/gene/?term=66272
Bicral	Bicral	BRD4 interacting chromatin remodeling complex associated protein like	210982	https://www.ncbi.nlm.nih.gov/gene/?term=210982
Samd4b	Samd4b	sterile alpha motif domain containing 4B	233033	https://www.ncbi.nlm.nih.gov/gene/?term=233033
Wdr26	Wdr26	WD repeat domain 26	226757	https://www.ncbi.nlm.nih.gov/gene/?term=226757

Biology Open • Supplementary information

Tpt1	Tpt1	tumor protein, translationally-controlled 1	22070	https://www.ncbi.nlm.nih.gov/gene/?term=22070
Hk3	Hk3	hexokinase 3	212032	https://www.ncbi.nlm.nih.gov/gene/?term=212032
Gtf3c2	Gtf3c2	general transcription factor IIIC, polypeptide 2, beta	71752	https://www.ncbi.nlm.nih.gov/gene/?term=71752
Gabarapl2	Gabarapl2	gamma-aminobutyric acid (GABA) A receptor-associated protein-like 2	93739	https://www.ncbi.nlm.nih.gov/gene/?term=93739
Otub2	Otub2	OTU domain, ubiquitin aldehyde binding 2	68149	https://www.ncbi.nlm.nih.gov/gene/?term=68149
Ppih	Ppih	peptidyl prolyl isomerase H	66101	https://www.ncbi.nlm.nih.gov/gene/?term=66101
Ptpn9	Ptpn9	protein tyrosine phosphatase, non-receptor type 9	56294	https://www.ncbi.nlm.nih.gov/gene/?term=56294
Tenm3	Tenm3	teneurin transmembrane protein 3	23965	https://www.ncbi.nlm.nih.gov/gene/?term=23965
Camk2n1	Camk2n1	calcium/calmodulin-dependent protein kinase II inhibitor 1	66259	https://www.ncbi.nlm.nih.gov/gene/?term=66259
Tti1	Tti1	TELO2 interacting protein 1	75425	https://www.ncbi.nlm.nih.gov/gene/?term=75425
Nol4	Nol4	nucleolar protein 4	319211	https://www.ncbi.nlm.nih.gov/gene/?term=319211
Fbxo42	Fbxo42	F-box protein 42	213499	https://www.ncbi.nlm.nih.gov/gene/?term=213499
Sccpdh	Sccpdh	saccharopine dehydrogenase (putative)	109232	https://www.ncbi.nlm.nih.gov/gene/?term=109232
Slc12a2	Slc12a2	solute carrier family 12, member 2	20496	https://www.ncbi.nlm.nih.gov/gene/?term=20496
Hfm1	Hfm1	HFM1, ATP-dependent DNA helicase homolog	330149	https://www.ncbi.nlm.nih.gov/gene/?term=330149
Mob2	Mob2	MOB kinase activator 2	101513	https://www.ncbi.nlm.nih.gov/gene/?term=101513
Hrh1	Hrh1	histamine receptor H1	15465	https://www.ncbi.nlm.nih.gov/gene/?term=15465
Amot	Amot	angiomotin	27494	https://www.ncbi.nlm.nih.gov/gene/?term=27494
Ddit4	Ddit4	DNA-damage-inducible transcript 4	74747	https://www.ncbi.nlm.nih.gov/gene/?term=74747
Net1	Net1	neuroepithelial cell transforming gene 1	56349	https://www.ncbi.nlm.nih.gov/gene/?term=56349

Biology Open • Supplementary information

Prpf38a	Prpf38a	PRP38 pre-mRNA processing factor 38 (yeast) domain containing A	230596	https://www.ncbi.nlm.nih.gov/gene/?term=230596
Styx	Styx	serine/threonine/tyrosine interaction protein	56291	https://www.ncbi.nlm.nih.gov/gene/?term=56291
Batf3	Batf3	basic leucine zipper transcription factor, ATF-like 3	381319	https://www.ncbi.nlm.nih.gov/gene/?term=381319
Elovl6	Elovl6	ELOVL family member 6, elongation of long chain fatty acids (yeast)	170439	https://www.ncbi.nlm.nih.gov/gene/?term=170439
Phf8	Phf8	PHD finger protein 8	320595	https://www.ncbi.nlm.nih.gov/gene/?term=320595
Cebpd	Cebpd	CCAAT/enhancer binding protein (C/EBP), delta	12609	https://www.ncbi.nlm.nih.gov/gene/?term=12609
Clic4	Clic4	chloride intracellular channel 4 (mitochondrial)	29876	https://www.ncbi.nlm.nih.gov/gene/?term=29876
Hoxa4	Hoxa4	homeobox A4	15401	https://www.ncbi.nlm.nih.gov/gene/?term=15401
4930523C07Rik	4930523C07Rik	RIKEN cDNA 4930523C07 gene	67647	https://www.ncbi.nlm.nih.gov/gene/?term=67647
Cenpv	Cenpv	centromere protein V	73139	https://www.ncbi.nlm.nih.gov/gene/?term=73139
Ubn1	Ubn1	ubinuclein 1	170644	https://www.ncbi.nlm.nih.gov/gene/?term=170644
Cacul1	Cacul1	CDK2 associated, cullin domain 1	78832	https://www.ncbi.nlm.nih.gov/gene/?term=78832
Lrrc1	Lrrc1	leucine rich repeat containing 1	214345	https://www.ncbi.nlm.nih.gov/gene/?term=214345
Rbm15	Rbm15	RNA binding motif protein 15	229700	https://www.ncbi.nlm.nih.gov/gene/?term=229700
Akt3	Akt3	thymoma viral proto-oncogene 3	23797	https://www.ncbi.nlm.nih.gov/gene/?term=23797
Gm1110	Gm1110	predicted gene 1110	382064	https://www.ncbi.nlm.nih.gov/gene/?term=382064
Ttpa	Ttpa	tocopherol (alpha) transfer protein	50500	https://www.ncbi.nlm.nih.gov/gene/?term=50500
Pigr	Pigr	polymeric immunoglobulin receptor	18703	https://www.ncbi.nlm.nih.gov/gene/?term=18703
Hacd3	Hacd3	3-hydroxyacyl-CoA dehydratase 3	57874	https://www.ncbi.nlm.nih.gov/gene/?term=57874
Ahcyl2	Ahcyl2	S-adenosylhomocysteine hydrolase-like 2	74340	https://www.ncbi.nlm.nih.gov/gene/?term=74340
Ttc8	Ttc8	tetratricopeptide repeat domain 8	76260	https://www.ncbi.nlm.nih.gov/gene/?term=76260
Acta2	Acta2	actin, alpha 2, smooth muscle, aorta	11475	https://www.ncbi.nlm.nih.gov/gene/?term=11475

Biology Open • Supplementary information

G2e3	G2e3	G2/M-phase specific E3 ubiquitin ligase	217558	https://www.ncbi.nlm.nih.gov/gene/?term=217558
Azi2	Azi2	5-azacytidine induced gene 2	27215	https://www.ncbi.nlm.nih.gov/gene/?term=27215
Cetn3	Cetn3	centrin 3	12626	https://www.ncbi.nlm.nih.gov/gene/?term=12626
Krt222	Krt222	keratin 222	268481	https://www.ncbi.nlm.nih.gov/gene/?term=268481
Eng	Eng	endoglin	13805	https://www.ncbi.nlm.nih.gov/gene/?term=13805
Fam110c	Fam110c	family with sequence similarity 110, member C	104943	https://www.ncbi.nlm.nih.gov/gene/?term=104943
A1cf	A1cf	APOBEC1 complementation factor	69865	https://www.ncbi.nlm.nih.gov/gene/?term=69865
Rfc1	Rfc1	replication factor C (activator 1) 1	19687	https://www.ncbi.nlm.nih.gov/gene/?term=19687
Galnt17	Galnt17	polypeptide N-acetylgalactosaminyltransferase 17	212996	https://www.ncbi.nlm.nih.gov/gene/?term=212996
Trak2	Trak2	trafficking protein, kinesin binding 2	70827	https://www.ncbi.nlm.nih.gov/gene/?term=70827
Sh3bp2	Sh3bp2	SH3-domain binding protein 2	24055	https://www.ncbi.nlm.nih.gov/gene/?term=24055
Afap1l1	Afap1l1	actin filament associated protein 1-like 1	106877	https://www.ncbi.nlm.nih.gov/gene/?term=106877
Lama4	Lama4	laminin, alpha 4	16775	https://www.ncbi.nlm.nih.gov/gene/?term=16775
Mfsd9	Mfsd9	major facilitator superfamily domain containing 9	211798	https://www.ncbi.nlm.nih.gov/gene/?term=211798
Sap25	Sap25	NA	751865	https://www.ncbi.nlm.nih.gov/gene/?term=751865
Igf2	Igf2	insulin-like growth factor 2	16002	https://www.ncbi.nlm.nih.gov/gene/?term=16002
Cacna1c	Cacna1c	calcium channel, voltage-dependent, L type, alpha 1C subunit	12288	https://www.ncbi.nlm.nih.gov/gene/?term=12288
Asah2	Asah2	N-acylsphingosine amidohydrolase 2	54447	https://www.ncbi.nlm.nih.gov/gene/?term=54447
Dnlz	Dnlz	DNL-type zinc finger	52838	https://www.ncbi.nlm.nih.gov/gene/?term=52838
Podn	Podn	podocan	242608	https://www.ncbi.nlm.nih.gov/gene/?term=242608
Orai2	Orai2	ORAI calcium release-activated calcium modulator 2	269717	https://www.ncbi.nlm.nih.gov/gene/?term=269717
D430019H16Rik	D430019H16Rik	RIKEN cDNA D430019H16 gene	268595	https://www.ncbi.nlm.nih.gov/gene/?term=268595

Biology Open • Supplementary information

Snrpn	Snrpn	small nuclear ribonucleoprotein N	20646	https://www.ncbi.nlm.nih.gov/gene/?term=20646
Tmem132b	Tmem132b	transmembrane protein 132B	208151	https://www.ncbi.nlm.nih.gov/gene/?term=208151
Prr18	Prr18	proline rich 18	320111	https://www.ncbi.nlm.nih.gov/gene/?term=320111
D430042O09Rik	D430042O09Rik	RIKEN cDNA D430042O09 gene	233865	https://www.ncbi.nlm.nih.gov/gene/?term=233865
Kcnj1	Kcnj1	potassium inwardly-rectifying channel, subfamily J, member 1	56379	https://www.ncbi.nlm.nih.gov/gene/?term=56379
Adra1b	Adra1b	adrenergic receptor, alpha 1b	11548	https://www.ncbi.nlm.nih.gov/gene/?term=11548
Lbp	Lbp	lipopolysaccharide binding protein	16803	https://www.ncbi.nlm.nih.gov/gene/?term=16803
Pou4f2	Pou4f2	POU domain, class 4, transcription factor 2	18997	https://www.ncbi.nlm.nih.gov/gene/?term=18997
Scn3b	Scn3b	sodium channel, voltage-gated, type III, beta	235281	https://www.ncbi.nlm.nih.gov/gene/?term=235281
Sytl5	Sytl5	synaptotagmin-like 5	236643	https://www.ncbi.nlm.nih.gov/gene/?term=236643
Ostm1	Ostm1	osteopetrosis associated transmembrane protein 1	14628	https://www.ncbi.nlm.nih.gov/gene/?term=14628
Adhfe1	Adhfe1	alcohol dehydrogenase, iron containing, 1	76187	https://www.ncbi.nlm.nih.gov/gene/?term=76187
Bean1	Bean1	brain expressed, associated with Nedd4, 1	65115	https://www.ncbi.nlm.nih.gov/gene/?term=65115
Cask	Cask	calcium/calmodulin-dependent serine protein kinase (MAGUK family)	12361	https://www.ncbi.nlm.nih.gov/gene/?term=12361
AW554918	AW554918	expressed sequence AW554918	225289	https://www.ncbi.nlm.nih.gov/gene/?term=225289
Cdc27	Cdc27	cell division cycle 27	217232	https://www.ncbi.nlm.nih.gov/gene/?term=217232
Sox1	Sox1	SRY (sex determining region Y)-box 1	20664	https://www.ncbi.nlm.nih.gov/gene/?term=20664
Bcl2	Bcl2	B cell leukemia/lymphoma 2	12043	https://www.ncbi.nlm.nih.gov/gene/?term=12043
Cstf3	Cstf3	cleavage stimulation factor, 3' pre-RNA, subunit 3	228410	https://www.ncbi.nlm.nih.gov/gene/?term=228410

Dkk1	Dkk1	dickkopf WNT signaling pathway inhibitor 1	13380	https://www.ncbi.nlm.nih.gov/gene/?term=13380
Zfand2a	Zfand2a	zinc finger, AN1-type domain 2A	100494	https://www.ncbi.nlm.nih.gov/gene/?term=100494
Kif1a	Kif1a	kinesin family member 1A	16560	https://www.ncbi.nlm.nih.gov/gene/?term=16560
Smarca2	Smarca2	SWI/SNF related, matrix associated, actin dependent regulator of chromatin, subfamily a, member 2	67155	https://www.ncbi.nlm.nih.gov/gene/?term=67155
Elp4	Elp4	elongator acetyltransferase complex subunit 4	77766	https://www.ncbi.nlm.nih.gov/gene/?term=77766
Pcdhgb6	Pcdhgb6	protocadherin gamma subfamily B, 6	93703	https://www.ncbi.nlm.nih.gov/gene/?term=93703
Zfp239	Zfp239	zinc finger protein 239	22685	https://www.ncbi.nlm.nih.gov/gene/?term=22685
Zrsr2	Zrsr2	zinc finger (CCCH type), RNA binding motif and serine/arginine rich 2	22184	https://www.ncbi.nlm.nih.gov/gene/?term=22184
Cd99l2	Cd99l2	CD99 antigen-like 2	171486	https://www.ncbi.nlm.nih.gov/gene/?term=171486
Xrcc3	Xrcc3	X-ray repair complementing defective repair in Chinese hamster cells 3	74335	https://www.ncbi.nlm.nih.gov/gene/?term=74335
Amotl1	Amotl1	angiomotin-like 1	75723	https://www.ncbi.nlm.nih.gov/gene/?term=75723
Slc38a6	Slc38a6	solute carrier family 38, member 6	625098	https://www.ncbi.nlm.nih.gov/gene/?term=625098
Ogdh	Ogdh	oxoglutarate (alpha-ketoglutarate) dehydrogenase (lipoamide)	18293	https://www.ncbi.nlm.nih.gov/gene/?term=18293
Vsig10l	Vsig10l	V-set and immunoglobulin domain containing 10 like	75690	https://www.ncbi.nlm.nih.gov/gene/?term=75690
Zfp37	Zfp37	zinc finger protein 37	22696	https://www.ncbi.nlm.nih.gov/gene/?term=22696
Nqo2	Nqo2	N-ribosyldihydronicotinamide quinone reductase 2	18105	https://www.ncbi.nlm.nih.gov/gene/?term=18105
Nab2	Nab2	Ngfi-A binding protein 2	17937	https://www.ncbi.nlm.nih.gov/gene/?term=17937
Cyfip1	Cyfip1	cytoplasmic FMR1 interacting protein 1	20430	https://www.ncbi.nlm.nih.gov/gene/?term=20430

Biology Open • Supplementary information

Zfp764	Zfp764	zinc finger protein 764	233893	https://www.ncbi.nlm.nih.gov/gene/?term=233893
Rbm34	Rbm34	RNA binding motif protein 34	52202	https://www.ncbi.nlm.nih.gov/gene/?term=52202
Olig2	Olig2	oligodendrocyte transcription factor 2	50913	https://www.ncbi.nlm.nih.gov/gene/?term=50913
Tigd5	Tigd5	tigger transposable element derived 5	105734	https://www.ncbi.nlm.nih.gov/gene/?term=105734
Zfp365	Zfp365	zinc finger protein 365	216049	https://www.ncbi.nlm.nih.gov/gene/?term=216049
Bdh1	Bdh1	3-hydroxybutyrate dehydrogenase, type 1	71911	https://www.ncbi.nlm.nih.gov/gene/?term=71911
Lcp2	Lcp2	lymphocyte cytosolic protein 2	16822	https://www.ncbi.nlm.nih.gov/gene/?term=16822
Pcdhga12	Pcdhga12	protocadherin gamma subfamily A, 12	93724	https://www.ncbi.nlm.nih.gov/gene/?term=93724
Iars	Iars	isoleucine-tRNA synthetase	105148	https://www.ncbi.nlm.nih.gov/gene/?term=105148
Adarb2	Adarb2	adenosine deaminase, RNA-specific, B2	94191	https://www.ncbi.nlm.nih.gov/gene/?term=94191
Wars2	Wars2	tryptophanyl tRNA synthetase 2 (mitochondrial)	70560	https://www.ncbi.nlm.nih.gov/gene/?term=70560
Ggact	Ggact	gamma-glutamylamine cyclotransferase	223267	https://www.ncbi.nlm.nih.gov/gene/?term=223267
Ralgapa2	Ralgapa2	Ral GTPase activating protein, alpha subunit 2 (catalytic)	241694	https://www.ncbi.nlm.nih.gov/gene/?term=241694
Opcml	Opcml	opioid binding protein/cell adhesion molecule-like	330908	https://www.ncbi.nlm.nih.gov/gene/?term=330908
Ppfia2	Ppfia2	protein tyrosine phosphatase, receptor type, f polypeptide (PTPRF), interacting protein (liprin), alpha 2	327814	https://www.ncbi.nlm.nih.gov/gene/?term=327814
Slc17a5	Slc17a5	solute carrier family 17 (anion/sugar transporter), member 5	235504	https://www.ncbi.nlm.nih.gov/gene/?term=235504
Gfra2	Gfra2	glial cell line derived neurotrophic factor family receptor alpha 2	14586	https://www.ncbi.nlm.nih.gov/gene/?term=14586

Kank2	Kank2	KN motif and ankyrin repeat domains 2	235041	https://www.ncbi.nlm.nih.gov/gene/?term=235041
Fgf11	Fgf11	fibroblast growth factor 11	14166	https://www.ncbi.nlm.nih.gov/gene/?term=14166
Foxk1	Foxk1	forkhead box K1	17425	https://www.ncbi.nlm.nih.gov/gene/?term=17425
Cd33	Cd33	CD33 antigen	12489	https://www.ncbi.nlm.nih.gov/gene/?term=12489
Slc4a8	Slc4a8	solute carrier family 4 (anion exchanger), member 8	59033	https://www.ncbi.nlm.nih.gov/gene/?term=59033
Vegfb	Vegfb	vascular endothelial growth factor B	22340	https://www.ncbi.nlm.nih.gov/gene/?term=22340
Zfp46	Zfp46	zinc finger protein 46	22704	https://www.ncbi.nlm.nih.gov/gene/?term=22704
Ssbp4	Ssbp4	single stranded DNA binding protein 4	76900	https://www.ncbi.nlm.nih.gov/gene/?term=76900
Rhou	Rhou	ras homolog family member U	69581	https://www.ncbi.nlm.nih.gov/gene/?term=69581
Iffo2	Iffo2	intermediate filament family orphan 2	212632	https://www.ncbi.nlm.nih.gov/gene/?term=212632
Wrn	Wrn	Werner syndrome RecQ like helicase	22427	https://www.ncbi.nlm.nih.gov/gene/?term=22427
Dcx	Dcx	doublecortin	13193	https://www.ncbi.nlm.nih.gov/gene/?term=13193
Stambp	Stambp	STAM binding protein	70527	https://www.ncbi.nlm.nih.gov/gene/?term=70527
Phactr3	Phactr3	phosphatase and actin regulator 3	74189	https://www.ncbi.nlm.nih.gov/gene/?term=74189
Cnih3	Cnih3	cornichon family AMPA receptor auxiliary protein 3	72978	https://www.ncbi.nlm.nih.gov/gene/?term=72978
Kcnj15	Kcnj15	potassium inwardly-rectifying channel, subfamily J, member 15	16516	https://www.ncbi.nlm.nih.gov/gene/?term=16516
Zfp516	Zfp516	zinc finger protein 516	329003	https://www.ncbi.nlm.nih.gov/gene/?term=329003
Maf	Maf	avian musculoaponeurotic fibrosarcoma oncogene homolog	17132	https://www.ncbi.nlm.nih.gov/gene/?term=17132
Wiz	Wiz	widely-interspaced zinc finger motifs	22404	https://www.ncbi.nlm.nih.gov/gene/?term=22404
Fbxo31	Fbxo31	F-box protein 31	76454	https://www.ncbi.nlm.nih.gov/gene/?term=76454
Bmp4	Bmp4	bone morphogenetic protein 4	12159	https://www.ncbi.nlm.nih.gov/gene/?term=12159
Thada	Thada	thyroid adenoma associated	240174	https://www.ncbi.nlm.nih.gov/gene/?term=240174

Lhx6	Lhx6	LIM homeobox protein 6	16874	https://www.ncbi.nlm.nih.gov/gene/?term=16874
Asap3	Asap3	ArfGAP with SH3 domain, ankyrin repeat and PH domain 3	230837	https://www.ncbi.nlm.nih.gov/gene/?term=230837
Zfp575	Zfp575	zinc finger protein 575	101544	https://www.ncbi.nlm.nih.gov/gene/?term=101544
Cyp2j12	Cyp2j12	cytochrome P450, family 2, subfamily j, polypeptide 12	242546	https://www.ncbi.nlm.nih.gov/gene/?term=242546
Acpp	Acpp	acid phosphatase, prostate	56318	https://www.ncbi.nlm.nih.gov/gene/?term=56318
Zfp266	Zfp266	zinc finger protein 266	77519	https://www.ncbi.nlm.nih.gov/gene/?term=77519
Sdf4	Sdf4	stromal cell derived factor 4	20318	https://www.ncbi.nlm.nih.gov/gene/?term=20318
Tcte1	Tcte1	t-complex-associated testis expressed 1	21645	https://www.ncbi.nlm.nih.gov/gene/?term=21645
Gm14434	Gm14434	predicted gene 14434	668039	https://www.ncbi.nlm.nih.gov/gene/?term=668039
Rsph4a	Rsph4a	radial spoke head 4 homolog A (Chlamydomonas)	212892	https://www.ncbi.nlm.nih.gov/gene/?term=212892
Gata4	Gata4	GATA binding protein 4	14463	https://www.ncbi.nlm.nih.gov/gene/?term=14463
Cd84	Cd84	CD84 antigen	12523	https://www.ncbi.nlm.nih.gov/gene/?term=12523
Prkca	Prkca	protein kinase C, alpha	18750	https://www.ncbi.nlm.nih.gov/gene/?term=18750
Napb	Napb	N-ethylmaleimide sensitive fusion protein attachment protein beta	17957	https://www.ncbi.nlm.nih.gov/gene/?term=17957
Vapb	Vapb	vesicle-associated membrane protein, associated protein B and C	56491	https://www.ncbi.nlm.nih.gov/gene/?term=56491
Cacna2d2	Cacna2d2	calcium channel, voltage-dependent, alpha 2/delta subunit 2	56808	https://www.ncbi.nlm.nih.gov/gene/?term=56808
Cbln3	Cbln3	cerebellin 3 precursor protein	56410	https://www.ncbi.nlm.nih.gov/gene/?term=56410
Sarm1	Sarm1	sterile alpha and HEAT/Armadillo motif containing 1	237868	https://www.ncbi.nlm.nih.gov/gene/?term=237868
Rab7	Rab7	RAB7, member RAS oncogene family	19349	https://www.ncbi.nlm.nih.gov/gene/?term=19349
Vti1a	Vti1a	vesicle transport through interaction with t-SNAREs 1A	53611	https://www.ncbi.nlm.nih.gov/gene/?term=53611

Biology Open • Supplementary information

Dapp1	Dapp1	dual adaptor for phosphotyrosine and 3-phosphoinositides 1	26377	https://www.ncbi.nlm.nih.gov/gene/?term=26377
Rnft2	Rnft2	ring finger protein, transmembrane 2	269695	https://www.ncbi.nlm.nih.gov/gene/?term=269695
Zscan29	Zscan29	zinc finger SCAN domains 29	99334	https://www.ncbi.nlm.nih.gov/gene/?term=99334
Rit2	Rit2	Ras-like without CAAX 2	19762	https://www.ncbi.nlm.nih.gov/gene/?term=19762
Rassf2	Rassf2	Ras association (RalGDS/AF-6) domain family member 2	215653	https://www.ncbi.nlm.nih.gov/gene/?term=215653
Deptor	Deptor	DEP domain containing MTOR-interacting protein	97998	https://www.ncbi.nlm.nih.gov/gene/?term=97998
1810065E05Rik	1810065E05Rik	RIKEN cDNA 1810065E05 gene	69864	https://www.ncbi.nlm.nih.gov/gene/?term=69864
Cfap74	Cfap74	cilia and flagella associated protein 74	544678	https://www.ncbi.nlm.nih.gov/gene/?term=544678
Bmpr2	Bmpr2	bone morphogenetic protein receptor, type II (serine/threonine kinase)	12168	https://www.ncbi.nlm.nih.gov/gene/?term=12168
Pacsin2	Pacsin2	protein kinase C and casein kinase substrate in neurons 2	23970	https://www.ncbi.nlm.nih.gov/gene/?term=23970
Ceacam2	Ceacam2	carcinoembryonic antigen-related cell adhesion molecule 2	26367	https://www.ncbi.nlm.nih.gov/gene/?term=26367
Cd300ld	Cd300ld	CD300 molecule like family member d	217305	https://www.ncbi.nlm.nih.gov/gene/?term=217305
Pura	Pura	purine rich element binding protein A	19290	https://www.ncbi.nlm.nih.gov/gene/?term=19290
Pstpip2	Pstpip2	proline-serine-threonine phosphatase-interacting protein 2	19201	https://www.ncbi.nlm.nih.gov/gene/?term=19201
Gpr45	Gpr45	G protein-coupled receptor 45	93690	https://www.ncbi.nlm.nih.gov/gene/?term=93690
Muc13	Muc13	mucin 13, epithelial transmembrane	17063	https://www.ncbi.nlm.nih.gov/gene/?term=17063
Snap25	Snap25	synaptosomal-associated protein 25	20614	https://www.ncbi.nlm.nih.gov/gene/?term=20614

Biology Open • Supplementary information

Fsd1l	Fsd1l	fibronectin type III and SPRY domain containing 1-like	319636	https://www.ncbi.nlm.nih.gov/gene/?term=319636
Kcnj16	Kcnj16	potassium inwardly-rectifying channel, subfamily J, member 16	16517	https://www.ncbi.nlm.nih.gov/gene/?term=16517
Flnb	Flnb	filamin, beta	286940	https://www.ncbi.nlm.nih.gov/gene/?term=286940
Pcdhga10	Pcdhga10	protocadherin gamma subfamily A, 10	93722	https://www.ncbi.nlm.nih.gov/gene/?term=93722
Mllt3	Mllt3	myeloid/lymphoid or mixed-lineage leukemia; translocated to, 3	70122	https://www.ncbi.nlm.nih.gov/gene/?term=70122
Dcdc2a	Dcdc2a	doublecortin domain containing 2a	195208	https://www.ncbi.nlm.nih.gov/gene/?term=195208
Cyp4a31	Cyp4a31	cytochrome P450, family 4, subfamily a, polypeptide 31	666168	https://www.ncbi.nlm.nih.gov/gene/?term=666168
Hspb7	Hspb7	heat shock protein family, member 7 (cardiovascular)	29818	https://www.ncbi.nlm.nih.gov/gene/?term=29818
Lsm1	Lsm1	LSM1 homolog, mRNA degradation associated	67207	https://www.ncbi.nlm.nih.gov/gene/?term=67207
Coq8a	Coq8a	coenzyme Q8A	67426	https://www.ncbi.nlm.nih.gov/gene/?term=67426
Actr1b	Actr1b	ARP1 actin-related protein 1B, centractin beta	226977	https://www.ncbi.nlm.nih.gov/gene/?term=226977
Cd47	Cd47	CD47 antigen (Rh-related antigen, integrin-associated signal transducer)	16423	https://www.ncbi.nlm.nih.gov/gene/?term=16423
Ackr4	Ackr4	atypical chemokine receptor 4	252837	https://www.ncbi.nlm.nih.gov/gene/?term=252837
Mdm2	Mdm2	transformed mouse 3T3 cell double minute 2	17246	https://www.ncbi.nlm.nih.gov/gene/?term=17246
Arhgap26	Arhgap26	Rho GTPase activating protein 26	71302	https://www.ncbi.nlm.nih.gov/gene/?term=71302
Hpca	Hpca	hippocalcin	15444	https://www.ncbi.nlm.nih.gov/gene/?term=15444
Tmem52b	Tmem52b	transmembrane protein 52B	330428	https://www.ncbi.nlm.nih.gov/gene/?term=330428
Ccdc115	Ccdc115	coiled-coil domain containing 115	69668	https://www.ncbi.nlm.nih.gov/gene/?term=69668

Dhfr	Dhfr	dihydrofolate reductase	13361	https://www.ncbi.nlm.nih.gov/gene/?term=13361
Fam174b	Fam174b	family with sequence similarity 174, member B	100038347	https://www.ncbi.nlm.nih.gov/gene/?term=100038
Lnx2	Lnx2	ligand of numb-protein X 2	140887	https://www.ncbi.nlm.nih.gov/gene/?term=140887
Xk	Xk	X-linked Kx blood group	22439	https://www.ncbi.nlm.nih.gov/gene/?term=22439
Xylb	Xylb	xylulokinase homolog (H. influenzae)	102448	https://www.ncbi.nlm.nih.gov/gene/?term=102448
Pogk	Pogk	pogo transposable element with KRAB domain	71592	https://www.ncbi.nlm.nih.gov/gene/?term=71592
Kcnc1	Kcnc1	potassium voltage gated channel, Shaw-related subfamily, member 1	16502	https://www.ncbi.nlm.nih.gov/gene/?term=16502
Tnr	Tnr	tenascin R	21960	https://www.ncbi.nlm.nih.gov/gene/?term=21960
Homer2	Homer2	homer scaffolding protein 2	26557	https://www.ncbi.nlm.nih.gov/gene/?term=26557
Nfya	Nfya	nuclear transcription factor-Y alpha	18044	https://www.ncbi.nlm.nih.gov/gene/?term=18044
2810021J22Rik	2810021J22Rik	RIKEN cDNA 2810021J22 gene	69944	https://www.ncbi.nlm.nih.gov/gene/?term=69944
Cnksr2	Cnksr2	connector enhancer of kinase suppressor of Ras 2	245684	https://www.ncbi.nlm.nih.gov/gene/?term=245684
Cxcl15	Cxcl15	chemokine (C-X-C motif) ligand 15	20309	https://www.ncbi.nlm.nih.gov/gene/?term=20309
Tnfrsf13c	Tnfrsf13c	tumor necrosis factor receptor superfamily, member 13c	72049	https://www.ncbi.nlm.nih.gov/gene/?term=72049
Zfp655	Zfp655	zinc finger protein 655	72611	https://www.ncbi.nlm.nih.gov/gene/?term=72611
Nmrk2	Nmrk2	nicotinamide riboside kinase 2	69564	https://www.ncbi.nlm.nih.gov/gene/?term=69564
Pank1	Pank1	pantothenate kinase 1	75735	https://www.ncbi.nlm.nih.gov/gene/?term=75735
Pard3b	Pard3b	par-3 family cell polarity regulator beta	72823	https://www.ncbi.nlm.nih.gov/gene/?term=72823
Usp45	Usp45	ubiquitin specific petidase 45	77593	https://www.ncbi.nlm.nih.gov/gene/?term=77593
Frk	Frk	fyn-related kinase	14302	https://www.ncbi.nlm.nih.gov/gene/?term=14302
Pcdhgb7	Pcdhgb7	protocadherin gamma subfamily B, 7	93704	https://www.ncbi.nlm.nih.gov/gene/?term=93704
Nmrk1	Nmrk1	nicotinamide riboside kinase 1	225994	https://www.ncbi.nlm.nih.gov/gene/?term=225994

Lrtm2	Lrtm2	leucine-rich repeats and transmembrane domains 2	211187	https://www.ncbi.nlm.nih.gov/gene/?term=211187
Alox8	Alox8	arachidonate 8-lipoxygenase	11688	https://www.ncbi.nlm.nih.gov/gene/?term=11688
Triobp	Triobp	TRIO and F-actin binding protein	110253	https://www.ncbi.nlm.nih.gov/gene/?term=110253
Grk3	Grk3	G protein-coupled receptor kinase 3	320129	https://www.ncbi.nlm.nih.gov/gene/?term=320129
Cer1	Cer1	cerberus 1, DAN family BMP antagonist	12622	https://www.ncbi.nlm.nih.gov/gene/?term=12622
Prelid3a	Prelid3a	PRELI domain containing 3A	225655	https://www.ncbi.nlm.nih.gov/gene/?term=225655
Ift57	Ift57	intraflagellar transport 57	73916	https://www.ncbi.nlm.nih.gov/gene/?term=73916
Pgm5	Pgm5	phosphoglucomutase 5	226041	https://www.ncbi.nlm.nih.gov/gene/?term=226041
Arhgdib	Arhgdib	Rho, GDP dissociation inhibitor (GDI) beta	11857	https://www.ncbi.nlm.nih.gov/gene/?term=11857
Atp11a	Atp11a	ATPase, class VI, type 11A	50770	https://www.ncbi.nlm.nih.gov/gene/?term=50770
Rhobtb1	Rhobtb1	Rho-related BTB domain containing 1	69288	https://www.ncbi.nlm.nih.gov/gene/?term=69288
Smo	Smo	smoothened, frizzled class receptor	319757	https://www.ncbi.nlm.nih.gov/gene/?term=319757
Snap23	Snap23	synaptosomal-associated protein 23	20619	https://www.ncbi.nlm.nih.gov/gene/?term=20619
Prkcg	Prkcg	protein kinase C, gamma	18752	https://www.ncbi.nlm.nih.gov/gene/?term=18752
Prdm12	Prdm12	PR domain containing 12	381359	https://www.ncbi.nlm.nih.gov/gene/?term=381359
Cd160	Cd160	CD160 antigen	54215	https://www.ncbi.nlm.nih.gov/gene/?term=54215
Ms4a5	Ms4a5	membrane-spanning 4-domains, subfamily A, member 5	269063	https://www.ncbi.nlm.nih.gov/gene/?term=269063
Srxn1	Srxn1	sulfiredoxin 1 homolog (S. cerevisiae)	76650	https://www.ncbi.nlm.nih.gov/gene/?term=76650
Adgra1	Adgra1	adhesion G protein-coupled receptor A1	52389	https://www.ncbi.nlm.nih.gov/gene/?term=52389
Csf2ra	Csf2ra	colony stimulating factor 2 receptor, alpha, low-affinity (granulocyte-macrophage)	12982	https://www.ncbi.nlm.nih.gov/gene/?term=12982
Zfp329	Zfp329	zinc finger protein 329	67230	https://www.ncbi.nlm.nih.gov/gene/?term=67230
Kcnip3	Kcnip3	Kv channel interacting protein 3,	56461	https://www.ncbi.nlm.nih.gov/gene/?term=56461

Biology Open • Supplementary information

		calsenilin		
Pigh	Pigh	phosphatidylinositol glycan anchor biosynthesis, class H	110417	https://www.ncbi.nlm.nih.gov/gene/?term=110417
Uncx	Uncx	UNC homeobox	22255	https://www.ncbi.nlm.nih.gov/gene/?term=22255
Alg12	Alg12	asparagine-linked glycosylation 12 (alpha-1,6-mannosyltransferase)	223774	https://www.ncbi.nlm.nih.gov/gene/?term=223774
Zfp810	Zfp810	zinc finger protein 810	235050	https://www.ncbi.nlm.nih.gov/gene/?term=235050
Ms4a4c	Ms4a4c	membrane-spanning 4-domains, subfamily A, member 4C	64380	https://www.ncbi.nlm.nih.gov/gene/?term=64380
Oprm1	Oprm1	opioid receptor, mu 1	18390	https://www.ncbi.nlm.nih.gov/gene/?term=18390
Sipa1l1	Sipa1l1	signal-induced proliferation-associated 1 like 1	217692	https://www.ncbi.nlm.nih.gov/gene/?term=217692
Klhl13	Klhl13	kelch-like 13	67455	https://www.ncbi.nlm.nih.gov/gene/?term=67455
Tirap	Tirap	toll-interleukin 1 receptor (TIR) domain-containing adaptor protein	117149	https://www.ncbi.nlm.nih.gov/gene/?term=117149
Acot2	Acot2	acyl-CoA thioesterase 2	171210	https://www.ncbi.nlm.nih.gov/gene/?term=171210
Asb13	Asb13	ankyrin repeat and SOCS box-containing 13	142688	https://www.ncbi.nlm.nih.gov/gene/?term=142688
Tmem141	Tmem141	transmembrane protein 141	51875	https://www.ncbi.nlm.nih.gov/gene/?term=51875
Dhh	Dhh	desert hedgehog	13363	https://www.ncbi.nlm.nih.gov/gene/?term=13363
Tmem150c	Tmem150c	transmembrane protein 150C	231503	https://www.ncbi.nlm.nih.gov/gene/?term=231503
Ly6g6c	Ly6g6c	lymphocyte antigen 6 complex, locus G6C	68468	https://www.ncbi.nlm.nih.gov/gene/?term=68468
Pgrmc1	Pgrmc1	progesterone receptor membrane component 1	53328	https://www.ncbi.nlm.nih.gov/gene/?term=53328
Chrnb4	Chrnb4	cholinergic receptor, nicotinic, beta polypeptide 4	108015	https://www.ncbi.nlm.nih.gov/gene/?term=108015
Arhgef17	Arhgef17	Rho guanine nucleotide exchange factor (GEF) 17	207212	https://www.ncbi.nlm.nih.gov/gene/?term=207212

Biology Open • Supplementary information

Mtus2	Mtus2	microtubule associated tumor suppressor candidate 2	77521	https://www.ncbi.nlm.nih.gov/gene/?term=77521
Mapk8	Mapk8	mitogen-activated protein kinase 8	26419	https://www.ncbi.nlm.nih.gov/gene/?term=26419
Apba1	Apba1	amyloid beta (A4) precursor protein binding, family A, member 1	319924	https://www.ncbi.nlm.nih.gov/gene/?term=319924
Maml3	Maml3	mastermind like transcriptional coactivator 3	433586	https://www.ncbi.nlm.nih.gov/gene/?term=433586
Rfk	Rfk	riboflavin kinase	54391	https://www.ncbi.nlm.nih.gov/gene/?term=54391
Gm20498	Gm20498	predicted gene 20498	105940408	https://www.ncbi.nlm.nih.gov/gene/?term=105940[illegible]
Fblim1	Fblim1	filamin binding LIM protein 1	74202	https://www.ncbi.nlm.nih.gov/gene/?term=74202
St8sia1	St8sia1	ST8 alpha-N-acetyl-neuraminide alpha-2,8-sialyltransferase 1	20449	https://www.ncbi.nlm.nih.gov/gene/?term=20449
Cdc6	Cdc6	cell division cycle 6	23834	https://www.ncbi.nlm.nih.gov/gene/?term=23834
Pcdhgc5	Pcdhgc5	protocadherin gamma subfamily C, 5	93708	https://www.ncbi.nlm.nih.gov/gene/?term=93708
Cpm	Cpm	carboxypeptidase M	70574	https://www.ncbi.nlm.nih.gov/gene/?term=70574
Urb2	Urb2	URB2 ribosome biogenesis 2 homolog (S. cerevisiae)	382038	https://www.ncbi.nlm.nih.gov/gene/?term=382038
4921509C19Rik	4921509C19Rik	RIKEN cDNA 4921509C19 gene	381393	https://www.ncbi.nlm.nih.gov/gene/?term=381393
Ifnlr1	Ifnlr1	interferon lambda receptor 1	242700	https://www.ncbi.nlm.nih.gov/gene/?term=242700
Rgr	Rgr	retinal G protein coupled receptor	57811	https://www.ncbi.nlm.nih.gov/gene/?term=57811
Cox15	Cox15	cytochrome c oxidase assembly protein 15	226139	https://www.ncbi.nlm.nih.gov/gene/?term=226139
Mobp	Mobp	myelin-associated oligodendrocytic basic protein	17433	https://www.ncbi.nlm.nih.gov/gene/?term=17433
Lrrc61	Lrrc61	leucine rich repeat containing 61	243371	https://www.ncbi.nlm.nih.gov/gene/?term=243371
Chst3	Chst3	carbohydrate sulfotransferase 3	53374	https://www.ncbi.nlm.nih.gov/gene/?term=53374
Rp1	Rp1	retinitis pigmentosa 1 (human)	19888	https://www.ncbi.nlm.nih.gov/gene/?term=19888
Accs	Accs	1-aminocyclopropane-1-carboxylate synthase (non-functional)	329470	https://www.ncbi.nlm.nih.gov/gene/?term=329470

Slc22a8	Slc22a8	solute carrier family 22 (organic anion transporter), member 8	19879	https://www.ncbi.nlm.nih.gov/gene/?term=19879
Prex2	Prex2	phosphatidylinositol-3,4,5-trisphosphate-dependent Rac exchange factor 2	109294	https://www.ncbi.nlm.nih.gov/gene/?term=109294
Tacr2	Tacr2	tachykinin receptor 2	21337	https://www.ncbi.nlm.nih.gov/gene/?term=21337
Rrm2	Rrm2	ribonucleotide reductase M2	20135	https://www.ncbi.nlm.nih.gov/gene/?term=20135
Serinc3	Serinc3	serine incorporator 3	26943	https://www.ncbi.nlm.nih.gov/gene/?term=26943
Hdac8	Hdac8	histone deacetylase 8	70315	https://www.ncbi.nlm.nih.gov/gene/?term=70315
Nkx2-9	Nkx2-9	NK2 homeobox 9	18094	https://www.ncbi.nlm.nih.gov/gene/?term=18094
Tph1	Tph1	tryptophan hydroxylase 1	21990	https://www.ncbi.nlm.nih.gov/gene/?term=21990
Oxsm	Oxsm	3-oxoacyl-ACP synthase, mitochondrial	71147	https://www.ncbi.nlm.nih.gov/gene/?term=71147
Zfp606	Zfp606	zinc finger protein 606	67370	https://www.ncbi.nlm.nih.gov/gene/?term=67370
Nab1	Nab1	Ngfi-A binding protein 1	17936	https://www.ncbi.nlm.nih.gov/gene/?term=17936
Ddx51	Ddx51	DEAD (Asp-Glu-Ala-Asp) box polypeptide 51	69663	https://www.ncbi.nlm.nih.gov/gene/?term=69663
Otx1	Otx1	orthodenticle homeobox 1	18423	https://www.ncbi.nlm.nih.gov/gene/?term=18423
Sod2	Sod2	superoxide dismutase 2, mitochondrial	20656	https://www.ncbi.nlm.nih.gov/gene/?term=20656
Ank2	Ank2	ankyrin 2, brain	109676	https://www.ncbi.nlm.nih.gov/gene/?term=109676
Thsd4	Thsd4	thrombospondin, type I, domain containing 4	207596	https://www.ncbi.nlm.nih.gov/gene/?term=207596
Trpc7	Trpc7	transient receptor potential cation channel, subfamily C, member 7	26946	https://www.ncbi.nlm.nih.gov/gene/?term=26946
Bmp7	Bmp7	bone morphogenetic protein 7	12162	https://www.ncbi.nlm.nih.gov/gene/?term=12162
Elfn2	Elfn2	leucine rich repeat and fibronectin type III, extracellular 2	207393	https://www.ncbi.nlm.nih.gov/gene/?term=207393
Pcdhga2	Pcdhga2	protocadherin gamma subfamily A, 2	93710	https://www.ncbi.nlm.nih.gov/gene/?term=93710

Biology Open • Supplementary information

Pilra	Pilra	paired immunoglobin-like type 2 receptor alpha	231805	https://www.ncbi.nlm.nih.gov/gene/?term=231805
Dgkg	Dgkg	diacylglycerol kinase, gamma	110197	https://www.ncbi.nlm.nih.gov/gene/?term=110197
Gm4841	Gm4841	predicted gene 4841	225594	https://www.ncbi.nlm.nih.gov/gene/?term=225594
Acss1	Acss1	acyl-CoA synthetase short-chain family member 1	68738	https://www.ncbi.nlm.nih.gov/gene/?term=68738
Pou2f2	Pou2f2	POU domain, class 2, transcription factor 2	18987	https://www.ncbi.nlm.nih.gov/gene/?term=18987
Foxn1	Foxn1	forkhead box N1	15218	https://www.ncbi.nlm.nih.gov/gene/?term=15218
Pirt	Pirt	phosphoinositide-interacting regulator of transient receptor potential channels	193003	https://www.ncbi.nlm.nih.gov/gene/?term=193003
Srf	Srf	serum response factor	20807	https://www.ncbi.nlm.nih.gov/gene/?term=20807
Casp6	Casp6	caspase 6	12368	https://www.ncbi.nlm.nih.gov/gene/?term=12368
Ascl4	Ascl4	achaete-scute family bHLH transcription factor 4	67341	https://www.ncbi.nlm.nih.gov/gene/?term=67341
2610002M06Rik	2610002M06Rik	RIKEN cDNA 2610002M06 gene	67028	https://www.ncbi.nlm.nih.gov/gene/?term=67028
Gna13	Gna13	guanine nucleotide binding protein, alpha 13	14674	https://www.ncbi.nlm.nih.gov/gene/?term=14674
Wipf2	Wipf2	WAS/WASL interacting protein family, member 2	68524	https://www.ncbi.nlm.nih.gov/gene/?term=68524
Cdh12	Cdh12	cadherin 12	215654	https://www.ncbi.nlm.nih.gov/gene/?term=215654
Cds2	Cds2	CDP-diacylglycerol synthase (phosphatidate cytidylyltransferase) 2	110911	https://www.ncbi.nlm.nih.gov/gene/?term=110911
Tyw3	Tyw3	tRNA-yW synthesizing protein 3 homolog (S. cerevisiae)	209584	https://www.ncbi.nlm.nih.gov/gene/?term=209584
Flrt1	Flrt1	fibronectin leucine rich transmembrane protein 1	396184	https://www.ncbi.nlm.nih.gov/gene/?term=396184

Biology Open • Supplementary information

Fbrs	Fbrs	fibrosin	14123	https://www.ncbi.nlm.nih.gov/gene/?term=14123
Rab9b	Rab9b	RAB9B, member RAS oncogene family	319642	https://www.ncbi.nlm.nih.gov/gene/?term=319642
Cgnl1	Cgnl1	cingulin-like 1	68178	https://www.ncbi.nlm.nih.gov/gene/?term=68178
Ugt2b34	Ugt2b34	UDP glucuronosyltransferase 2 family, polypeptide B34	100727	https://www.ncbi.nlm.nih.gov/gene/?term=100727
Cacna1e	Cacna1e	calcium channel, voltage-dependent, R type, alpha 1E subunit	12290	https://www.ncbi.nlm.nih.gov/gene/?term=12290
Bdp1	Bdp1	B double prime 1, subunit of RNA polymerase III transcription initiation factor IIIB	544971	https://www.ncbi.nlm.nih.gov/gene/?term=544971
Rslcan18	Rslcan18	regulator of sex-limitation candidate 18	432770	https://www.ncbi.nlm.nih.gov/gene/?term=432770
Ceacam1	Ceacam1	carcinoembryonic antigen-related cell adhesion molecule 1	26365	https://www.ncbi.nlm.nih.gov/gene/?term=26365
Zfp691	Zfp691	zinc finger protein 691	195522	https://www.ncbi.nlm.nih.gov/gene/?term=195522
Adamts14	Adamts14	a disintegrin-like and metallopeptidase (reprolysin type) with thrombospondin type 1 motif, 14	237360	https://www.ncbi.nlm.nih.gov/gene/?term=237360
Slc22a15	Slc22a15	solute carrier family 22 (organic anion/cation transporter), member 15	242126	https://www.ncbi.nlm.nih.gov/gene/?term=242126
Sv2b	Sv2b	synaptic vesicle glycoprotein 2 b	64176	https://www.ncbi.nlm.nih.gov/gene/?term=64176
Casp8	Casp8	caspase 8	12370	https://www.ncbi.nlm.nih.gov/gene/?term=12370
Senp1	Senp1	SUMO1/sentrin specific peptidase 1	223870	https://www.ncbi.nlm.nih.gov/gene/?term=223870
Fam169b	Fam169b	family with sequence similarity 169, member B	434197	https://www.ncbi.nlm.nih.gov/gene/?term=434197
Rnasel	Rnasel	ribonuclease L (2', 5'-oligoisoadenylate synthetase-	24014	https://www.ncbi.nlm.nih.gov/gene/?term=24014

		dependent)		
Ccdc85a	Ccdc85a	coiled-coil domain containing 85A	216613	https://www.ncbi.nlm.nih.gov/gene/?term=216613
Slc6a17	Slc6a17	solute carrier family 6 (neurotransmitter transporter), member 17	229706	https://www.ncbi.nlm.nih.gov/gene/?term=229706
Chn1	Chn1	chimerin 1	108699	https://www.ncbi.nlm.nih.gov/gene/?term=108699
Ctse	Ctse	cathepsin E	13034	https://www.ncbi.nlm.nih.gov/gene/?term=13034
Itga11	Itga11	integrin alpha 11	319480	https://www.ncbi.nlm.nih.gov/gene/?term=319480
Dlg4	Dlg4	discs large MAGUK scaffold protein 4	13385	https://www.ncbi.nlm.nih.gov/gene/?term=13385
Igsf6	Igsf6	immunoglobulin superfamily, member 6	80719	https://www.ncbi.nlm.nih.gov/gene/?term=80719
Rabl2	Rabl2	RAB, member RAS oncogene family-like 2	68708	https://www.ncbi.nlm.nih.gov/gene/?term=68708
Ttc4	Ttc4	tetratricopeptide repeat domain 4	72354	https://www.ncbi.nlm.nih.gov/gene/?term=72354
Cd4	Cd4	CD4 antigen	12504	https://www.ncbi.nlm.nih.gov/gene/?term=12504
Tc2n	Tc2n	tandem C2 domains, nuclear	74413	https://www.ncbi.nlm.nih.gov/gene/?term=74413
Pde10a	Pde10a	phosphodiesterase 10A	23984	https://www.ncbi.nlm.nih.gov/gene/?term=23984
Tpgs2	Tpgs2	tubulin polyglutamylase complex subunit 2	66648	https://www.ncbi.nlm.nih.gov/gene/?term=66648
Prss42	Prss42	protease, serine 42	235628	https://www.ncbi.nlm.nih.gov/gene/?term=235628
Msrb3	Msrb3	methionine sulfoxide reductase B3	320183	https://www.ncbi.nlm.nih.gov/gene/?term=320183
Rspo1	Rspo1	R-spondin 1	192199	https://www.ncbi.nlm.nih.gov/gene/?term=192199
Pou3f4	Pou3f4	POU domain, class 3, transcription factor 4	18994	https://www.ncbi.nlm.nih.gov/gene/?term=18994
Tsn	Tsn	translin	22099	https://www.ncbi.nlm.nih.gov/gene/?term=22099
Gdpgp1	Gdpgp1	GDP-D-glucose phosphorylase 1	269952	https://www.ncbi.nlm.nih.gov/gene/?term=269952
Nr4a2	Nr4a2	nuclear receptor subfamily 4, group A, member 2	18227	https://www.ncbi.nlm.nih.gov/gene/?term=18227

Biology Open • Supplementary information

Saa4	Saa4	serum amyloid A 4	20211	https://www.ncbi.nlm.nih.gov/gene/?term=20211
Cntn2	Cntn2	contactin 2	21367	https://www.ncbi.nlm.nih.gov/gene/?term=21367
Irgm2	Irgm2	immunity-related GTPase family M member 2	54396	https://www.ncbi.nlm.nih.gov/gene/?term=54396
Grik3	Grik3	glutamate receptor, ionotropic, kainate 3	14807	https://www.ncbi.nlm.nih.gov/gene/?term=14807
Smpd4	Smpd4	sphingomyelin phosphodiesterase 4	77626	https://www.ncbi.nlm.nih.gov/gene/?term=77626
Zfp446	Zfp446	zinc finger protein 446	269870	https://www.ncbi.nlm.nih.gov/gene/?term=269870
Ppp1r16b	Ppp1r16b	protein phosphatase 1, regulatory subunit 16B	228852	https://www.ncbi.nlm.nih.gov/gene/?term=228852
Cox10	Cox10	heme A:farnesyltransferase cytochrome c oxidase assembly factor 10	70383	https://www.ncbi.nlm.nih.gov/gene/?term=70383
Sp100	Sp100	nuclear antigen Sp100	20684	https://www.ncbi.nlm.nih.gov/gene/?term=20684
Aptx	Aptx	aprataxin	66408	https://www.ncbi.nlm.nih.gov/gene/?term=66408
Runx3	Runx3	runt related transcription factor 3	12399	https://www.ncbi.nlm.nih.gov/gene/?term=12399
Zbtb7b	Zbtb7b	zinc finger and BTB domain containing 7B	22724	https://www.ncbi.nlm.nih.gov/gene/?term=22724
Fbxw5	Fbxw5	F-box and WD-40 domain protein 5	30839	https://www.ncbi.nlm.nih.gov/gene/?term=30839
Acp1	Acp1	acid phosphatase 1, soluble	11431	https://www.ncbi.nlm.nih.gov/gene/?term=11431
Gucy2e	Gucy2e	guanylate cyclase 2e	14919	https://www.ncbi.nlm.nih.gov/gene/?term=14919
Nmnat2	Nmnat2	nicotinamide nucleotide adenylyltransferase 2	226518	https://www.ncbi.nlm.nih.gov/gene/?term=226518
Stk32a	Stk32a	serine/threonine kinase 32A	269019	https://www.ncbi.nlm.nih.gov/gene/?term=269019
Ctdsp2	Ctdsp2	CTD (carboxy-terminal domain, RNA polymerase II, polypeptide A) small phosphatase 2	52468	https://www.ncbi.nlm.nih.gov/gene/?term=52468
Yipf6	Yipf6	Yip1 domain family, member 6	77929	https://www.ncbi.nlm.nih.gov/gene/?term=77929

Nufip1	Nufip1	nuclear fragile X mental retardation protein interacting protein 1	27275	https://www.ncbi.nlm.nih.gov/gene/?term=27275
Pecam1	Pecam1	platelet/endothelial cell adhesion molecule 1	18613	https://www.ncbi.nlm.nih.gov/gene/?term=18613
Rgs5	Rgs5	regulator of G-protein signaling 5	19737	https://www.ncbi.nlm.nih.gov/gene/?term=19737
Mapk10	Mapk10	mitogen-activated protein kinase 10	26414	https://www.ncbi.nlm.nih.gov/gene/?term=26414
Cmah	Cmah	cytidine monophospho-N-acetylneuraminic acid hydroxylase	12763	https://www.ncbi.nlm.nih.gov/gene/?term=12763
Pcdhga1	Pcdhga1	protocadherin gamma subfamily A, 1	93709	https://www.ncbi.nlm.nih.gov/gene/?term=93709
B3gnt9	B3gnt9	UDP-GlcNAc:betaGal beta-1,3-N-acetylglucosaminyltransferase 9	97440	https://www.ncbi.nlm.nih.gov/gene/?term=97440
Sh2d2a	Sh2d2a	SH2 domain containing 2A	27371	https://www.ncbi.nlm.nih.gov/gene/?term=27371
Ap3s2	Ap3s2	adaptor-related protein complex 3, sigma 2 subunit	11778	https://www.ncbi.nlm.nih.gov/gene/?term=11778
Camk2a	Camk2a	calcium/calmodulin-dependent protein kinase II alpha	12322	https://www.ncbi.nlm.nih.gov/gene/?term=12322
Pfn1	Pfn1	profilin 1	18643	https://www.ncbi.nlm.nih.gov/gene/?term=18643
Bgn	Bgn	biglycan	12111	https://www.ncbi.nlm.nih.gov/gene/?term=12111
Psd2	Psd2	pleckstrin and Sec7 domain containing 2	74002	https://www.ncbi.nlm.nih.gov/gene/?term=74002
Cyb5r3	Cyb5r3	cytochrome b5 reductase 3	109754	https://www.ncbi.nlm.nih.gov/gene/?term=109754
Rmi1	Rmi1	RecQ mediated genome instability 1	74386	https://www.ncbi.nlm.nih.gov/gene/?term=74386
Shroom3	Shroom3	shroom family member 3	27428	https://www.ncbi.nlm.nih.gov/gene/?term=27428
Gnal	Gnal	guanine nucleotide binding protein, alpha stimulating, olfactory type	14680	https://www.ncbi.nlm.nih.gov/gene/?term=14680
Havcr2	Havcr2	hepatitis A virus cellular receptor 2	171285	https://www.ncbi.nlm.nih.gov/gene/?term=171285
Ddi2	Ddi2	DNA-damage inducible protein 2	68817	https://www.ncbi.nlm.nih.gov/gene/?term=68817
Chrnd	Chrnd	cholinergic receptor, nicotinic, delta polypeptide	11447	https://www.ncbi.nlm.nih.gov/gene/?term=11447

Biology Open • Supplementary information

Mcidas	Mcidas	multiciliate differentiation and DNA synthesis associated cell cycle protein	622408	https://www.ncbi.nlm.nih.gov/gene/?term=622408
Pcdhga3	Pcdhga3	protocadherin gamma subfamily A, 3	93711	https://www.ncbi.nlm.nih.gov/gene/?term=93711
Map3k7	Map3k7	mitogen-activated protein kinase kinase kinase 7	26409	https://www.ncbi.nlm.nih.gov/gene/?term=26409
Pitpnc1	Pitpnc1	phosphatidylinositol transfer protein, cytoplasmic 1	71795	https://www.ncbi.nlm.nih.gov/gene/?term=71795
Pcdhga8	Pcdhga8	protocadherin gamma subfamily A, 8	93716	https://www.ncbi.nlm.nih.gov/gene/?term=93716
Mtmr12	Mtmr12	myotubularin related protein 12	268783	https://www.ncbi.nlm.nih.gov/gene/?term=268783
Kirrel3	Kirrel3	kirre like nephrin family adhesion molecule 3	67703	https://www.ncbi.nlm.nih.gov/gene/?term=67703
Sike1	Sike1	suppressor of IKBKE 1	66641	https://www.ncbi.nlm.nih.gov/gene/?term=66641
Cgas	Cgas	cyclic GMP-AMP synthase	214763	https://www.ncbi.nlm.nih.gov/gene/?term=214763
Cradd	Cradd	CASP2 and RIPK1 domain containing adaptor with death domain	12905	https://www.ncbi.nlm.nih.gov/gene/?term=12905
Rad18	Rad18	RAD18 E3 ubiquitin protein ligase	58186	https://www.ncbi.nlm.nih.gov/gene/?term=58186
Prkn	Prkn	parkin RBR E3 ubiquitin protein ligase	50873	https://www.ncbi.nlm.nih.gov/gene/?term=50873
P4ha3	P4ha3	procollagen-proline, 2-oxoglutarate 4-dioxygenase (proline 4-hydroxylase), alpha polypeptide III	320452	https://www.ncbi.nlm.nih.gov/gene/?term=320452
Meltf	Meltf	melanotransferrin	30060	https://www.ncbi.nlm.nih.gov/gene/?term=30060
1700025G04Rik	1700025G04Rik	RIKEN cDNA 1700025G04 gene	69399	https://www.ncbi.nlm.nih.gov/gene/?term=69399
Pik3r5	Pik3r5	NA	320207	https://www.ncbi.nlm.nih.gov/gene/?term=320207
Igf2bp2	Igf2bp2	insulin-like growth factor 2 mRNA binding protein 2	319765	https://www.ncbi.nlm.nih.gov/gene/?term=319765
Rab6b	Rab6b	RAB6B, member RAS oncogene family	270192	https://www.ncbi.nlm.nih.gov/gene/?term=270192

Biology Open • Supplementary information

Gas2l1	Gas2l1	growth arrest-specific 2 like 1	78926	https://www.ncbi.nlm.nih.gov/gene/?term=78926
Prrc2b	Prrc2b	proline-rich coiled-coil 2B	227723	https://www.ncbi.nlm.nih.gov/gene/?term=227723
Fndc7	Fndc7	fibronectin type III domain containing 7	320181	https://www.ncbi.nlm.nih.gov/gene/?term=320181
Med14	Med14	mediator complex subunit 14	26896	https://www.ncbi.nlm.nih.gov/gene/?term=26896
Fcrl6	Fcrl6	Fc receptor-like 6	677296	https://www.ncbi.nlm.nih.gov/gene/?term=677296
Igfbp3	Igfbp3	insulin-like growth factor binding protein 3	16009	https://www.ncbi.nlm.nih.gov/gene/?term=16009
Neurod2	Neurod2	neurogenic differentiation 2	18013	https://www.ncbi.nlm.nih.gov/gene/?term=18013
Sri	Sri	sorcin	109552	https://www.ncbi.nlm.nih.gov/gene/?term=109552
Enpp1	Enpp1	ectonucleotide pyrophosphatase/phosphodiesterase 1	18605	https://www.ncbi.nlm.nih.gov/gene/?term=18605
Cdh6	Cdh6	cadherin 6	12563	https://www.ncbi.nlm.nih.gov/gene/?term=12563
Setd3	Setd3	SET domain containing 3	52690	https://www.ncbi.nlm.nih.gov/gene/?term=52690
BC030500	BC030500	cDNA sequence BC030500	234290	https://www.ncbi.nlm.nih.gov/gene/?term=234290
Glyr1	Glyr1	glyoxylate reductase 1 homolog (Arabidopsis)	74022	https://www.ncbi.nlm.nih.gov/gene/?term=74022
Ric8b	Ric8b	RIC8 guanine nucleotide exchange factor B	237422	https://www.ncbi.nlm.nih.gov/gene/?term=237422
Aldh8a1	Aldh8a1	aldehyde dehydrogenase 8 family, member A1	237320	https://www.ncbi.nlm.nih.gov/gene/?term=237320
Flt4	Flt4	FMS-like tyrosine kinase 4	14257	https://www.ncbi.nlm.nih.gov/gene/?term=14257
Zfp39	Zfp39	zinc finger protein 39	22698	https://www.ncbi.nlm.nih.gov/gene/?term=22698
Ado	Ado	2-aminoethanethiol (cysteamine) dioxygenase	211488	https://www.ncbi.nlm.nih.gov/gene/?term=211488
Snurf	Snurf	SNRPN upstream reading frame	84704	https://www.ncbi.nlm.nih.gov/gene/?term=84704
Pcdhga11	Pcdhga11	protocadherin gamma subfamily A, 11	93723	https://www.ncbi.nlm.nih.gov/gene/?term=93723

Biology Open • Supplementary information

Heatr6	Heatr6	HEAT repeat containing 6	217026	https://www.ncbi.nlm.nih.gov/gene/?term=217026
Acsm2	Acsm2	acyl-CoA synthetase medium-chain family member 2	233799	https://www.ncbi.nlm.nih.gov/gene/?term=233799
Gatc	Gatc	glutamyl-tRNA(Gln) amidotransferase, subunit C	384281	https://www.ncbi.nlm.nih.gov/gene/?term=384281
Il22ra1	Il22ra1	interleukin 22 receptor, alpha 1	230828	https://www.ncbi.nlm.nih.gov/gene/?term=230828
Acot4	Acot4	acyl-CoA thioesterase 4	171282	https://www.ncbi.nlm.nih.gov/gene/?term=171282
Setd7	Setd7	SET domain containing (lysine methyltransferase) 7	73251	https://www.ncbi.nlm.nih.gov/gene/?term=73251
Tacr1	Tacr1	tachykinin receptor 1	21336	https://www.ncbi.nlm.nih.gov/gene/?term=21336
Cnga4	Cnga4	cyclic nucleotide gated channel alpha 4	233649	https://www.ncbi.nlm.nih.gov/gene/?term=233649
Kmt5a	Kmt5a	lysine methyltransferase 5A	67956	https://www.ncbi.nlm.nih.gov/gene/?term=67956
Trp53i11	Trp53i11	transformation related protein 53 inducible protein 11	277414	https://www.ncbi.nlm.nih.gov/gene/?term=277414
9530003J23Rik	9530003J23Rik	RIKEN cDNA 9530003J23 gene	77397	https://www.ncbi.nlm.nih.gov/gene/?term=77397
Pcdhga6	Pcdhga6	protocadherin gamma subfamily A, 6	93714	https://www.ncbi.nlm.nih.gov/gene/?term=93714
Stat6	Stat6	signal transducer and activator of transcription 6	20852	https://www.ncbi.nlm.nih.gov/gene/?term=20852
Prim2	Prim2	DNA primase, p58 subunit	19076	https://www.ncbi.nlm.nih.gov/gene/?term=19076
Fat3	Fat3	FAT atypical cadherin 3	270120	https://www.ncbi.nlm.nih.gov/gene/?term=270120
Hexim2	Hexim2	hexamethylene bis-acetamide inducible 2	71059	https://www.ncbi.nlm.nih.gov/gene/?term=71059
Fam107a	Fam107a	family with sequence similarity 107, member A	268709	https://www.ncbi.nlm.nih.gov/gene/?term=268709
Pcdhga9	Pcdhga9	protocadherin gamma subfamily A, 9	93717	https://www.ncbi.nlm.nih.gov/gene/?term=93717
Gabrq	Gabrq	gamma-aminobutyric acid (GABA) A receptor, subunit theta	57249	https://www.ncbi.nlm.nih.gov/gene/?term=57249
Tcp11l1	Tcp11l1	t-complex 11 like 1	320554	https://www.ncbi.nlm.nih.gov/gene/?term=320554

Biology Open • Supplementary information

Prkaa1	Prkaa1	protein kinase, AMP-activated, alpha 1 catalytic subunit	105787	https://www.ncbi.nlm.nih.gov/gene/?term=105787
Slc16a5	Slc16a5	solute carrier family 16 (monocarboxylic acid transporters), member 5	217316	https://www.ncbi.nlm.nih.gov/gene/?term=217316
Ubap2l	Ubap2l	ubiquitin-associated protein 2-like	74383	https://www.ncbi.nlm.nih.gov/gene/?term=74383
Oxtr	Oxtr	oxytocin receptor	18430	https://www.ncbi.nlm.nih.gov/gene/?term=18430
Prps1l1	Prps1l1	phosphoribosyl pyrophosphate synthetase 1-like 1	75456	https://www.ncbi.nlm.nih.gov/gene/?term=75456
Mapk11	Mapk11	mitogen-activated protein kinase 11	19094	https://www.ncbi.nlm.nih.gov/gene/?term=19094
Ildr2	Ildr2	immunoglobulin-like domain containing receptor 2	100039795	https://www.ncbi.nlm.nih.gov/gene/?term=100039
Uhrf1bp1l	Uhrf1bp1l	UHRF1 (ICBP90) binding protein 1-like	75089	https://www.ncbi.nlm.nih.gov/gene/?term=75089
Nkap	Nkap	NFKB activating protein	67050	https://www.ncbi.nlm.nih.gov/gene/?term=67050
Arhgap25	Arhgap25	Rho GTPase activating protein 25	232201	https://www.ncbi.nlm.nih.gov/gene/?term=232201
Kcnv2	Kcnv2	potassium channel, subfamily V, member 2	240595	https://www.ncbi.nlm.nih.gov/gene/?term=240595
Pck2	Pck2	phosphoenolpyruvate carboxykinase 2 (mitochondrial)	74551	https://www.ncbi.nlm.nih.gov/gene/?term=74551
Htt	Htt	huntingtin	15194	https://www.ncbi.nlm.nih.gov/gene/?term=15194
Coq5	Coq5	coenzyme Q5 methyltransferase	52064	https://www.ncbi.nlm.nih.gov/gene/?term=52064
Itgam	Itgam	integrin alpha M	16409	https://www.ncbi.nlm.nih.gov/gene/?term=16409
Grasp	Grasp	GRP1 (general receptor for phosphoinositides 1)-associated scaffold protein	56149	https://www.ncbi.nlm.nih.gov/gene/?term=56149
Gnaz	Gnaz	guanine nucleotide binding protein, alpha z subunit	14687	https://www.ncbi.nlm.nih.gov/gene/?term=14687
Ralb	Ralb	v-ral simian leukemia viral oncogene	64143	https://www.ncbi.nlm.nih.gov/gene/?term=64143

Biology Open • Supplementary information

		B		
Gm12886	Gm12886	predicted gene 12886	666921	https://www.ncbi.nlm.nih.gov/gene/?term=666921
Wscd1	Wscd1	WSC domain containing 1	216881	https://www.ncbi.nlm.nih.gov/gene/?term=216881
Hnrnpa0	Hnrnpa0	heterogeneous nuclear ribonucleoprotein A0	77134	https://www.ncbi.nlm.nih.gov/gene/?term=77134
Vtcn1	Vtcn1	V-set domain containing T cell activation inhibitor 1	242122	https://www.ncbi.nlm.nih.gov/gene/?term=242122
Mta3	Mta3	metastasis associated 3	116871	https://www.ncbi.nlm.nih.gov/gene/?term=116871
Grid1	Grid1	glutamate receptor, ionotropic, delta 1	14803	https://www.ncbi.nlm.nih.gov/gene/?term=14803
Gadl1	Gadl1	glutamate decarboxylase-like 1	73748	https://www.ncbi.nlm.nih.gov/gene/?term=73748
Bst1	Bst1	bone marrow stromal cell antigen 1	12182	https://www.ncbi.nlm.nih.gov/gene/?term=12182
Sfrp1	Sfrp1	secreted frizzled-related protein 1	20377	https://www.ncbi.nlm.nih.gov/gene/?term=20377
Efcab14	Efcab14	EF-hand calcium binding domain 14	230648	https://www.ncbi.nlm.nih.gov/gene/?term=230648
Zmat4	Zmat4	zinc finger, matrin type 4	320158	https://www.ncbi.nlm.nih.gov/gene/?term=320158
Dctd	Dctd	dCMP deaminase	320685	https://www.ncbi.nlm.nih.gov/gene/?term=320685
Nhsl1	Nhsl1	NHS-like 1	215819	https://www.ncbi.nlm.nih.gov/gene/?term=215819
Mrvi1	Mrvi1	MRV integration site 1	17540	https://www.ncbi.nlm.nih.gov/gene/?term=17540
Mon1b	Mon1b	MON1 homolog B, secretory traffciking associated	270096	https://www.ncbi.nlm.nih.gov/gene/?term=270096
Deup1	Deup1	deuterosome assembly protein 1	234964	https://www.ncbi.nlm.nih.gov/gene/?term=234964
Cd27	Cd27	CD27 antigen	21940	https://www.ncbi.nlm.nih.gov/gene/?term=21940
Atrn	Atrn	attractin	11990	https://www.ncbi.nlm.nih.gov/gene/?term=11990
Pcdhgb1	Pcdhgb1	protocadherin gamma subfamily B, 1	93699	https://www.ncbi.nlm.nih.gov/gene/?term=93699
Insr	Insr	insulin receptor	16337	https://www.ncbi.nlm.nih.gov/gene/?term=16337
Acot3	Acot3	acyl-CoA thioesterase 3	171281	https://www.ncbi.nlm.nih.gov/gene/?term=171281
D630003M21Rik	D630003M21Rik	RIKEN cDNA D630003M21 gene	228846	https://www.ncbi.nlm.nih.gov/gene/?term=228846
Nos1	Nos1	nitric oxide synthase 1, neuronal	18125	https://www.ncbi.nlm.nih.gov/gene/?term=18125
Gm14137	Gm14137	predicted gene 14137	623781	https://www.ncbi.nlm.nih.gov/gene/?term=623781

Biology Open • Supplementary information

3425401B19Rik	3425401B19Rik	RIKEN cDNA 3425401B19 gene	100504518	https://www.ncbi.nlm.nih.gov/gene/?term=100504
Depdc5	Depdc5	DEP domain containing 5	277854	https://www.ncbi.nlm.nih.gov/gene/?term=277854
Trmt2b	Trmt2b	TRM2 tRNA methyltransferase 2B	215201	https://www.ncbi.nlm.nih.gov/gene/?term=215201
Mymk	Mymk	myomaker, myoblast fusion factor	66139	https://www.ncbi.nlm.nih.gov/gene/?term=66139
Iars2	Iars2	isoleucine-tRNA synthetase 2, mitochondrial	381314	https://www.ncbi.nlm.nih.gov/gene/?term=381314
Gm38666	Gm38666	predicted gene, 38666	103611158	https://www.ncbi.nlm.nih.gov/gene/?term=103611
Urod	Urod	uroporphyrinogen decarboxylase	22275	https://www.ncbi.nlm.nih.gov/gene/?term=22275
Micall1	Micall1	microtubule associated monooxygenase, calponin and LIM domain containing -like 1	27008	https://www.ncbi.nlm.nih.gov/gene/?term=27008
Cdc42ep4	Cdc42ep4	CDC42 effector protein (Rho GTPase binding) 4	56699	https://www.ncbi.nlm.nih.gov/gene/?term=56699
Gpr68	Gpr68	G protein-coupled receptor 68	238377	https://www.ncbi.nlm.nih.gov/gene/?term=238377
Napepld	Napepld	N-acyl phosphatidylethanolamine phospholipase D	242864	https://www.ncbi.nlm.nih.gov/gene/?term=242864
Pcdhgc3	Pcdhgc3	protocadherin gamma subfamily C, 3	93706	https://www.ncbi.nlm.nih.gov/gene/?term=93706
Wdr78	Wdr78	WD repeat domain 78	242584	https://www.ncbi.nlm.nih.gov/gene/?term=242584
Plpbp	Plpbp	pyridoxal phosphate binding protein	114863	https://www.ncbi.nlm.nih.gov/gene/?term=114863
Elovl5	Elovl5	ELOVL family member 5, elongation of long chain fatty acids (yeast)	68801	https://www.ncbi.nlm.nih.gov/gene/?term=68801
Proser3	Proser3	proline and serine rich 3	333193	https://www.ncbi.nlm.nih.gov/gene/?term=333193
Mafb	Mafb	v-maf musculoaponeurotic fibrosarcoma oncogene family, protein B (avian)	16658	https://www.ncbi.nlm.nih.gov/gene/?term=16658
Prokr2	Prokr2	prokineticin receptor 2	246313	https://www.ncbi.nlm.nih.gov/gene/?term=246313
Mnt	Mnt	max binding protein	17428	https://www.ncbi.nlm.nih.gov/gene/?term=17428
Hoxb4	Hoxb4	homeobox B4	15412	https://www.ncbi.nlm.nih.gov/gene/?term=15412
Clec2l	Clec2l	C-type lectin domain family 2,	665180	https://www.ncbi.nlm.nih.gov/gene/?term=665180

Biology Open • Supplementary information

		member L		
Dppa1	Dppa1	developmental pluripotency associated 1	347708	https://www.ncbi.nlm.nih.gov/gene/?term=347708
Tfap2b	Tfap2b	transcription factor AP-2 beta	21419	https://www.ncbi.nlm.nih.gov/gene/?term=21419
Fermt1	Fermt1	fermitin family member 1	241639	https://www.ncbi.nlm.nih.gov/gene/?term=241639
Garem2	Garem2	GRB2 associated regulator of MAPK1 subtype 2	242915	https://www.ncbi.nlm.nih.gov/gene/?term=242915
Slc8a3	Slc8a3	solute carrier family 8 (sodium/calcium exchanger), member 3	110893	https://www.ncbi.nlm.nih.gov/gene/?term=110893
Vipr2	Vipr2	vasoactive intestinal peptide receptor 2	22355	https://www.ncbi.nlm.nih.gov/gene/?term=22355
Klhl9	Klhl9	kelch-like 9	242521	https://www.ncbi.nlm.nih.gov/gene/?term=242521
Creg2	Creg2	cellular repressor of E1A-stimulated genes 2	263764	https://www.ncbi.nlm.nih.gov/gene/?term=263764
Lgals12	Lgals12	lectin, galactose binding, soluble 12	56072	https://www.ncbi.nlm.nih.gov/gene/?term=56072
Gpr173	Gpr173	G-protein coupled receptor 173	70771	https://www.ncbi.nlm.nih.gov/gene/?term=70771
Rab11fip1	Rab11fip1	RAB11 family interacting protein 1 (class I)	75767	https://www.ncbi.nlm.nih.gov/gene/?term=75767
Pcdhgb2	Pcdhgb2	protocadherin gamma subfamily B, 2	93700	https://www.ncbi.nlm.nih.gov/gene/?term=93700
Ceacam18	Ceacam18	carcinoembryonic antigen-related cell adhesion molecule 18	72431	https://www.ncbi.nlm.nih.gov/gene/?term=72431
Srpx2	Srpx2	sushi-repeat-containing protein, X-linked 2	68792	https://www.ncbi.nlm.nih.gov/gene/?term=68792
Il18r1	Il18r1	interleukin 18 receptor 1	16182	https://www.ncbi.nlm.nih.gov/gene/?term=16182
Cyb5b	Cyb5b	cytochrome b5 type B	66427	https://www.ncbi.nlm.nih.gov/gene/?term=66427
Cend1	Cend1	cell cycle exit and neuronal differentiation 1	57754	https://www.ncbi.nlm.nih.gov/gene/?term=57754
Rpp25	Rpp25	ribonuclease P/MRP 25 subunit	102614	https://www.ncbi.nlm.nih.gov/gene/?term=102614

Sh3rf3	Sh3rf3	SH3 domain containing ring finger 3	237353	https://www.ncbi.nlm.nih.gov/gene/?term=237353
Tbc1d30	Tbc1d30	TBC1 domain family, member 30	74694	https://www.ncbi.nlm.nih.gov/gene/?term=74694
Esr2	Esr2	estrogen receptor 2 (beta)	13983	https://www.ncbi.nlm.nih.gov/gene/?term=13983
Lnpk	Lnpk	lunapark, ER junction formation factor	69605	https://www.ncbi.nlm.nih.gov/gene/?term=69605
Gas2l3	Gas2l3	growth arrest-specific 2 like 3	237436	https://www.ncbi.nlm.nih.gov/gene/?term=237436
Vamp5	Vamp5	vesicle-associated membrane protein 5	53620	https://www.ncbi.nlm.nih.gov/gene/?term=53620
P2ry13	P2ry13	purinergic receptor P2Y, G-protein coupled 13	74191	https://www.ncbi.nlm.nih.gov/gene/?term=74191
Ddo	Ddo	D-aspartate oxidase	70503	https://www.ncbi.nlm.nih.gov/gene/?term=70503
Gtf2h2	Gtf2h2	general transcription factor II H, polypeptide 2	23894	https://www.ncbi.nlm.nih.gov/gene/?term=23894
Chic2	Chic2	cysteine-rich hydrophobic domain 2	74277	https://www.ncbi.nlm.nih.gov/gene/?term=74277
Rasal2	Rasal2	RAS protein activator like 2	226525	https://www.ncbi.nlm.nih.gov/gene/?term=226525
Gm37013	Gm37013	predicted gene, 37013	100384868	https://www.ncbi.nlm.nih.gov/gene/?term=100384
Edaradd	Edaradd	EDAR (ectodysplasin-A receptor)-associated death domain	171211	https://www.ncbi.nlm.nih.gov/gene/?term=171211
F10	F10	coagulation factor X	14058	https://www.ncbi.nlm.nih.gov/gene/?term=14058
Nlgn3	Nlgn3	neuroligin 3	245537	https://www.ncbi.nlm.nih.gov/gene/?term=245537
Syt15	Syt15	synaptotagmin XV	319508	https://www.ncbi.nlm.nih.gov/gene/?term=319508
Creb3l1	Creb3l1	cAMP responsive element binding protein 3-like 1	26427	https://www.ncbi.nlm.nih.gov/gene/?term=26427
Fam149b	Fam149b	family with sequence similarity 149, member B	105428	https://www.ncbi.nlm.nih.gov/gene/?term=105428
Srsf12	Srsf12	serine/arginine-rich splicing factor 12	272009	https://www.ncbi.nlm.nih.gov/gene/?term=272009
Nphp3	Nphp3	nephronophthisis 3 (adolescent)	74025	https://www.ncbi.nlm.nih.gov/gene/?term=74025
Ttc12	Ttc12	tetratricopeptide repeat domain 12	235330	https://www.ncbi.nlm.nih.gov/gene/?term=235330
AI429214	AI429214	expressed sequence AI429214	621080	https://www.ncbi.nlm.nih.gov/gene/?term=621080

Biology Open • Supplementary information

Vangl2	Vangl2	VANGL planar cell polarity 2	93840	https://www.ncbi.nlm.nih.gov/gene/?term=93840
Slc35e2	Slc35e2	solute carrier family 35, member E2	320541	https://www.ncbi.nlm.nih.gov/gene/?term=320541
Sema5b	Sema5b	sema domain, seven thrombospondin repeats (type 1 and type 1-like), transmembrane domain (TM) and short cytoplasmic domain, (semaphorin) 5B	20357	https://www.ncbi.nlm.nih.gov/gene/?term=20357
B4galnt2	B4galnt2	beta-1,4-N-acetyl-galactosaminyl transferase 2	14422	https://www.ncbi.nlm.nih.gov/gene/?term=14422
Zfp799	Zfp799	zinc finger protein 799	240064	https://www.ncbi.nlm.nih.gov/gene/?term=240064
Adgrf5	Adgrf5	adhesion G protein-coupled receptor F5	224792	https://www.ncbi.nlm.nih.gov/gene/?term=224792
Mettl27	Mettl27	methyltransferase like 27	79565	https://www.ncbi.nlm.nih.gov/gene/?term=79565
Man1c1	Man1c1	mannosidase, alpha, class 1C, member 1	230815	https://www.ncbi.nlm.nih.gov/gene/?term=230815
Ttll1	Ttll1	tubulin tyrosine ligase-like 1	319953	https://www.ncbi.nlm.nih.gov/gene/?term=319953
Atp2b3	Atp2b3	ATPase, Ca++ transporting, plasma membrane 3	320707	https://www.ncbi.nlm.nih.gov/gene/?term=320707
Pgbd1	Pgbd1	piggyBac transposable element derived 1	319207	https://www.ncbi.nlm.nih.gov/gene/?term=319207
Capn6	Capn6	calpain 6	12338	https://www.ncbi.nlm.nih.gov/gene/?term=12338
Pld5	Pld5	phospholipase D family, member 5	319455	https://www.ncbi.nlm.nih.gov/gene/?term=319455
Dnase1l3	Dnase1l3	deoxyribonuclease 1-like 3	13421	https://www.ncbi.nlm.nih.gov/gene/?term=13421
Hspg2	Hspg2	perlecan (heparan sulfate proteoglycan 2)	15530	https://www.ncbi.nlm.nih.gov/gene/?term=15530
C5ar2	C5ar2	complement component 5a receptor 2	319430	https://www.ncbi.nlm.nih.gov/gene/?term=319430
B4galt6	B4galt6	UDP-Gal:betaGlcNAc beta 1,4-galactosyltransferase, polypeptide 6	56386	https://www.ncbi.nlm.nih.gov/gene/?term=56386
Vsnl1	Vsnl1	visinin-like 1	26950	https://www.ncbi.nlm.nih.gov/gene/?term=26950

Biology Open • Supplementary information

Gabbr1	Gabbr1	gamma-aminobutyric acid (GABA) B receptor, 1	54393	https://www.ncbi.nlm.nih.gov/gene/?term=54393
Prdm8	Prdm8	PR domain containing 8	77630	https://www.ncbi.nlm.nih.gov/gene/?term=77630
Pou6f1	Pou6f1	POU domain, class 6, transcription factor 1	19009	https://www.ncbi.nlm.nih.gov/gene/?term=19009
Neu1	Neu1	neuraminidase 1	18010	https://www.ncbi.nlm.nih.gov/gene/?term=18010
Naip6	Naip6	NLR family, apoptosis inhibitory protein 6	17952	https://www.ncbi.nlm.nih.gov/gene/?term=17952
Stxbp4	Stxbp4	syntaxin binding protein 4	20913	https://www.ncbi.nlm.nih.gov/gene/?term=20913
Trub2	Trub2	TruB pseudouridine (psi) synthase family member 2	227682	https://www.ncbi.nlm.nih.gov/gene/?term=227682
D630039A03Rik	D630039A03Rik	RIKEN cDNA D630039A03 gene	242484	https://www.ncbi.nlm.nih.gov/gene/?term=242484
Rsc1a1	Rsc1a1	regulatory solute carrier protein, family 1, member 1	69994	https://www.ncbi.nlm.nih.gov/gene/?term=69994
Tmco1	Tmco1	transmembrane and coiled-coil domains 1	68944	https://www.ncbi.nlm.nih.gov/gene/?term=68944
Actr10	Actr10	ARP10 actin-related protein 10	56444	https://www.ncbi.nlm.nih.gov/gene/?term=56444
Pcdhgc4	Pcdhgc4	protocadherin gamma subfamily C, 4	93707	https://www.ncbi.nlm.nih.gov/gene/?term=93707
Snai2	Snai2	snail family zinc finger 2	20583	https://www.ncbi.nlm.nih.gov/gene/?term=20583
Zfp169	Zfp169	zinc finger protein 169	67911	https://www.ncbi.nlm.nih.gov/gene/?term=67911
Epas1	Epas1	endothelial PAS domain protein 1	13819	https://www.ncbi.nlm.nih.gov/gene/?term=13819
Map3k20	Map3k20	mitogen-activated protein kinase kinase kinase 20	65964	https://www.ncbi.nlm.nih.gov/gene/?term=65964
Prkcb	Prkcb	protein kinase C, beta	18751	https://www.ncbi.nlm.nih.gov/gene/?term=18751
Pappa2	Pappa2	pappalysin 2	23850	https://www.ncbi.nlm.nih.gov/gene/?term=23850
Trpc5	Trpc5	transient receptor potential cation channel, subfamily C, member 5	22067	https://www.ncbi.nlm.nih.gov/gene/?term=22067
Supt7l	Supt7l	suppressor of Ty 7-like	72195	https://www.ncbi.nlm.nih.gov/gene/?term=72195
Zfp318	Zfp318	zinc finger protein 318	57908	https://www.ncbi.nlm.nih.gov/gene/?term=57908

Htra4	Htra4	HtrA serine peptidase 4	330723	https://www.ncbi.nlm.nih.gov/gene/?term=330723
Lyrm9	Lyrm9	LYR motif containing 9	66274	https://www.ncbi.nlm.nih.gov/gene/?term=66274
Iglon5	Iglon5	IgLON family member 5	210094	https://www.ncbi.nlm.nih.gov/gene/?term=210094
Tvp23a	Tvp23a	trans-golgi network vesicle protein 23A	383103	https://www.ncbi.nlm.nih.gov/gene/?term=383103
Osr1	Osr1	odd-skipped related transcription factor 1	23967	https://www.ncbi.nlm.nih.gov/gene/?term=23967
Pcdhga7	Pcdhga7	protocadherin gamma subfamily A, 7	93715	https://www.ncbi.nlm.nih.gov/gene/?term=93715
Brix1	Brix1	BRX1, biogenesis of ribosomes	67832	https://www.ncbi.nlm.nih.gov/gene/?term=67832
Zc3h12d	Zc3h12d	zinc finger CCCH type containing 12D	237256	https://www.ncbi.nlm.nih.gov/gene/?term=237256
Angpt2	Angpt2	angiopoietin 2	11601	https://www.ncbi.nlm.nih.gov/gene/?term=11601
Ascl1	Ascl1	achaete-scute family bHLH transcription factor 1	17172	https://www.ncbi.nlm.nih.gov/gene/?term=17172
Cap2	Cap2	CAP, adenylate cyclase-associated protein, 2 (yeast)	67252	https://www.ncbi.nlm.nih.gov/gene/?term=67252
Uvssa	Uvssa	UV stimulated scaffold protein A	71101	https://www.ncbi.nlm.nih.gov/gene/?term=71101
Katnal1	Katnal1	katanin p60 subunit A-like 1	231912	https://www.ncbi.nlm.nih.gov/gene/?term=231912
Plppr3	Plppr3	phospholipid phosphatase related 3	216152	https://www.ncbi.nlm.nih.gov/gene/?term=216152
Mrgpre	Mrgpre	MAS-related GPR, member E	244238	https://www.ncbi.nlm.nih.gov/gene/?term=244238
Gm14308	Gm14308	predicted gene 14308	100043381	https://www.ncbi.nlm.nih.gov/gene/?term=100043
Pcdhga4	Pcdhga4	protocadherin gamma subfamily A, 4	93712	https://www.ncbi.nlm.nih.gov/gene/?term=93712
Peg3	Peg3	paternally expressed 3	18616	https://www.ncbi.nlm.nih.gov/gene/?term=18616
Calcoco1	Calcoco1	calcium binding and coiled coil domain 1	67488	https://www.ncbi.nlm.nih.gov/gene/?term=67488
9130023H24Rik	9130023H24Rik	RIKEN cDNA 9130023H24 gene	100043133	https://www.ncbi.nlm.nih.gov/gene/?term=100043
Ggt5	Ggt5	gamma-glutamyltransferase 5	23887	https://www.ncbi.nlm.nih.gov/gene/?term=23887
Dtx3l	Dtx3l	deltex 3-like, E3 ubiquitin ligase	209200	https://www.ncbi.nlm.nih.gov/gene/?term=209200
Metrnl	Metrnl	meteorin, glial cell differentiation regulator-like	210029	https://www.ncbi.nlm.nih.gov/gene/?term=210029

Rab2b	Rab2b	RAB2B, member RAS oncogene family	76338	https://www.ncbi.nlm.nih.gov/gene/?term=76338
Mrpl19	Mrpl19	mitochondrial ribosomal protein L19	56284	https://www.ncbi.nlm.nih.gov/gene/?term=56284
Kcnmb1	Kcnmb1	potassium large conductance calcium-activated channel, subfamily M, beta member 1	16533	https://www.ncbi.nlm.nih.gov/gene/?term=16533
Mocs1	Mocs1	molybdenum cofactor synthesis 1	56738	https://www.ncbi.nlm.nih.gov/gene/?term=56738
Nkain2	Nkain2	Na+/K+ transporting ATPase interacting 2	432450	https://www.ncbi.nlm.nih.gov/gene/?term=432450
Extl3	Extl3	exostoses (multiple)-like 3	54616	https://www.ncbi.nlm.nih.gov/gene/?term=54616
Zfp488	Zfp488	zinc finger protein 488	382867	https://www.ncbi.nlm.nih.gov/gene/?term=382867
Tnfrsf11a	Tnfrsf11a	tumor necrosis factor receptor superfamily, member 11a, NFKB activator	21934	https://www.ncbi.nlm.nih.gov/gene/?term=21934
Prss23	Prss23	protease, serine 23	76453	https://www.ncbi.nlm.nih.gov/gene/?term=76453
Ehd3	Ehd3	EH-domain containing 3	57440	https://www.ncbi.nlm.nih.gov/gene/?term=57440
Shisal1	Shisal1	shisa like 1	72301	https://www.ncbi.nlm.nih.gov/gene/?term=72301
Man2a2	Man2a2	mannosidase 2, alpha 2	140481	https://www.ncbi.nlm.nih.gov/gene/?term=140481
Klhl23	Klhl23	kelch-like 23	277396	https://www.ncbi.nlm.nih.gov/gene/?term=277396
Knstrn	Knstrn	kinetochore-localized astrin/SPAG5 binding	51944	https://www.ncbi.nlm.nih.gov/gene/?term=51944
Rab9	Rab9	RAB9, member RAS oncogene family	56382	https://www.ncbi.nlm.nih.gov/gene/?term=56382
Gm14151	Gm14151	predicted gene 14151	433486	https://www.ncbi.nlm.nih.gov/gene/?term=433486
Doc2b	Doc2b	double C2, beta	13447	https://www.ncbi.nlm.nih.gov/gene/?term=13447
B3galt6	B3galt6	UDP-Gal:betaGal beta 1,3-galactosyltransferase, polypeptide 6	117592	https://www.ncbi.nlm.nih.gov/gene/?term=117592
Zfp663	Zfp663	zinc finger protein 663	381405	https://www.ncbi.nlm.nih.gov/gene/?term=381405
Atg10	Atg10	autophagy related 10	66795	https://www.ncbi.nlm.nih.gov/gene/?term=66795

Terf2ip	Terf2ip	telomeric repeat binding factor 2, interacting protein	57321	https://www.ncbi.nlm.nih.gov/gene/?term=57321
Sp9	Sp9	trans-acting transcription factor 9	381373	https://www.ncbi.nlm.nih.gov/gene/?term=381373
Lrat	Lrat	lecithin-retinol acyltransferase (phosphatidylcholine-retinol-O-acyltransferase)	79235	https://www.ncbi.nlm.nih.gov/gene/?term=79235
Rgs9bp	Rgs9bp	regulator of G-protein signalling 9 binding protein	243923	https://www.ncbi.nlm.nih.gov/gene/?term=243923
Jmjd8	Jmjd8	jumonji domain containing 8	72106	https://www.ncbi.nlm.nih.gov/gene/?term=72106
Samd7	Samd7	sterile alpha motif domain containing 7	75953	https://www.ncbi.nlm.nih.gov/gene/?term=75953
Ankrd34a	Ankrd34a	ankyrin repeat domain 34A	545554	https://www.ncbi.nlm.nih.gov/gene/?term=545554
Wnt4	Wnt4	wingless-type MMTV integration site family, member 4	22417	https://www.ncbi.nlm.nih.gov/gene/?term=22417
Gm14391	Gm14391	predicted gene 14391	665001	https://www.ncbi.nlm.nih.gov/gene/?term=665001
Zfp712	Zfp712	zinc finger protein 712	78251	https://www.ncbi.nlm.nih.gov/gene/?term=78251
Gm38667	Gm38667	predicted gene, 38667	103611159	https://www.ncbi.nlm.nih.gov/gene/?term=103611
Scd3	Scd3	stearoyl-coenzyme A desaturase 3	30049	https://www.ncbi.nlm.nih.gov/gene/?term=30049
Tub	Tub	tubby bipartite transcription factor	22141	https://www.ncbi.nlm.nih.gov/gene/?term=22141
Plekhg5	Plekhg5	pleckstrin homology domain containing, family G (with RhoGef domain) member 5	269608	https://www.ncbi.nlm.nih.gov/gene/?term=269608
Pcdhgb4	Pcdhgb4	protocadherin gamma subfamily B, 4	93701	https://www.ncbi.nlm.nih.gov/gene/?term=93701
Zfp74	Zfp74	zinc finger protein 74	72723	https://www.ncbi.nlm.nih.gov/gene/?term=72723
Slco2a1	Slco2a1	solute carrier organic anion transporter family, member 2a1	24059	https://www.ncbi.nlm.nih.gov/gene/?term=24059
Vwa5b2	Vwa5b2	von Willebrand factor A domain containing 5B2	328643	https://www.ncbi.nlm.nih.gov/gene/?term=328643
Bmp3	Bmp3	bone morphogenetic protein 3	110075	https://www.ncbi.nlm.nih.gov/gene/?term=110075

Cmc2	Cmc2	COX assembly mitochondrial protein 2	66531	https://www.ncbi.nlm.nih.gov/gene/?term=66531
Dcaf1	Dcaf1	DDB1 and CUL4 associated factor 1	321006	https://www.ncbi.nlm.nih.gov/gene/?term=321006
Gcm2	Gcm2	glial cells missing homolog 2	107889	https://www.ncbi.nlm.nih.gov/gene/?term=107889
Ncan	Ncan	neurocan	13004	https://www.ncbi.nlm.nih.gov/gene/?term=13004
Csrnp2	Csrnp2	cysteine-serine-rich nuclear protein 2	207785	https://www.ncbi.nlm.nih.gov/gene/?term=207785
Slc25a21	Slc25a21	solute carrier family 25 (mitochondrial oxodicarboxylate carrier), member 21	217593	https://www.ncbi.nlm.nih.gov/gene/?term=217593
Plac9b	Plac9b	placenta specific 9b	100039246	https://www.ncbi.nlm.nih.gov/gene/?term=100039
Gm4925	Gm4925	predicted gene 4925	237433	https://www.ncbi.nlm.nih.gov/gene/?term=237433
Smyd3	Smyd3	SET and MYND domain containing 3	69726	https://www.ncbi.nlm.nih.gov/gene/?term=69726
Btbd8	Btbd8	BTB (POZ) domain containing 8	100503185	https://www.ncbi.nlm.nih.gov/gene/?term=100503
Dcaf17	Dcaf17	DDB1 and CUL4 associated factor 17	75763	https://www.ncbi.nlm.nih.gov/gene/?term=75763
Pcdhga5	Pcdhga5	protocadherin gamma subfamily A, 5	93713	https://www.ncbi.nlm.nih.gov/gene/?term=93713
Bin2	Bin2	bridging integrator 2	668218	https://www.ncbi.nlm.nih.gov/gene/?term=668218
Igsf11	Igsf11	immunoglobulin superfamily, member 11	207683	https://www.ncbi.nlm.nih.gov/gene/?term=207683
Nsun3	Nsun3	NOL1/NOP2/Sun domain family member 3	106338	https://www.ncbi.nlm.nih.gov/gene/?term=106338
Slc6a6	Slc6a6	solute carrier family 6 (neurotransmitter transporter, taurine), member 6	21366	https://www.ncbi.nlm.nih.gov/gene/?term=21366
Zfp92	Zfp92	zinc finger protein 92	22754	https://www.ncbi.nlm.nih.gov/gene/?term=22754
Megf8	Megf8	multiple EGF-like-domains 8	269878	https://www.ncbi.nlm.nih.gov/gene/?term=269878
Stk10	Stk10	serine/threonine kinase 10	20868	https://www.ncbi.nlm.nih.gov/gene/?term=20868
Sox7	Sox7	SRY (sex determining region Y)-box 7	20680	https://www.ncbi.nlm.nih.gov/gene/?term=20680
Usp25	Usp25	ubiquitin specific peptidase 25	30940	https://www.ncbi.nlm.nih.gov/gene/?term=30940

Biology Open • Supplementary information

Tmem151b	Tmem151b	transmembrane protein 151B	210573	https://www.ncbi.nlm.nih.gov/gene/?term=210573
Sox12	Sox12	SRY (sex determining region Y)-box 12	20667	https://www.ncbi.nlm.nih.gov/gene/?term=20667
Zfp747	Zfp747	zinc finger protein 747	269997	https://www.ncbi.nlm.nih.gov/gene/?term=269997
Pcdhgb5	Pcdhgb5	protocadherin gamma subfamily B, 5	93702	https://www.ncbi.nlm.nih.gov/gene/?term=93702
Prpf4	Prpf4	pre-mRNA processing factor 4	70052	https://www.ncbi.nlm.nih.gov/gene/?term=70052
Gcnt4	Gcnt4	glucosaminyl (N-acetyl) transferase 4, core 2 (beta-1,6-N-acetylglucosaminyltransferase)	218476	https://www.ncbi.nlm.nih.gov/gene/?term=218476
Tlx1	Tlx1	T cell leukemia, homeobox 1	21908	https://www.ncbi.nlm.nih.gov/gene/?term=21908
Hivep3	Hivep3	human immunodeficiency virus type I enhancer binding protein 3	16656	https://www.ncbi.nlm.nih.gov/gene/?term=16656
Tubb4a	Tubb4a	tubulin, beta 4A class IVA	22153	https://www.ncbi.nlm.nih.gov/gene/?term=22153
Micu2	Micu2	mitochondrial calcium uptake 2	68514	https://www.ncbi.nlm.nih.gov/gene/?term=68514
Cd274	Cd274	CD274 antigen	60533	https://www.ncbi.nlm.nih.gov/gene/?term=60533
Kcna7	Kcna7	potassium voltage-gated channel, shaker-related subfamily, member 7	16495	https://www.ncbi.nlm.nih.gov/gene/?term=16495
Gjc3	Gjc3	gap junction protein, gamma 3	118446	https://www.ncbi.nlm.nih.gov/gene/?term=118446
Trpc6	Trpc6	transient receptor potential cation channel, subfamily C, member 6	22068	https://www.ncbi.nlm.nih.gov/gene/?term=22068
Gm4724	Gm4724	predicted gene 4724	100043915	https://www.ncbi.nlm.nih.gov/gene/?term=100043[illegible]
Trem1	Trem1	triggering receptor expressed on myeloid cells 1	58217	https://www.ncbi.nlm.nih.gov/gene/?term=58217
Tgfbrap1	Tgfbrap1	transforming growth factor, beta receptor associated protein 1	73122	https://www.ncbi.nlm.nih.gov/gene/?term=73122
Tmem104	Tmem104	transmembrane protein 104	320534	https://www.ncbi.nlm.nih.gov/gene/?term=320534
Kdm6b	Kdm6b	KDM1 lysine (K)-specific demethylase 6B	216850	https://www.ncbi.nlm.nih.gov/gene/?term=216850
Ctsc	Ctsc	cathepsin C	13032	https://www.ncbi.nlm.nih.gov/gene/?term=13032

Muc4	Muc4	mucin 4	140474	https://www.ncbi.nlm.nih.gov/gene/?term=140474
Spock2	Spock2	sparc/osteonectin, cwcv and kazal-like domains proteoglycan 2	94214	https://www.ncbi.nlm.nih.gov/gene/?term=94214
Cstad	Cstad	CSA-conditional, T cell activation-dependent protein	78617	https://www.ncbi.nlm.nih.gov/gene/?term=78617
1700028K03Rik	1700028K03Rik	RIKEN cDNA 1700028K03 gene	76421	https://www.ncbi.nlm.nih.gov/gene/?term=76421
Dhdh	Dhdh	dihydrodiol dehydrogenase (dimeric)	71755	https://www.ncbi.nlm.nih.gov/gene/?term=71755
Nagpa	Nagpa	N-acetylglucosamine-1-phosphodiester alpha-N-acetylglucosaminidase	27426	https://www.ncbi.nlm.nih.gov/gene/?term=27426
Fbxw28	Fbxw28	F-box and WD-40 domain protein 28	668758	https://www.ncbi.nlm.nih.gov/gene/?term=668758
Rpusd2	Rpusd2	RNA pseudouridylate synthase domain containing 2	271842	https://www.ncbi.nlm.nih.gov/gene/?term=271842
Mlec	Mlec	malectin	109154	https://www.ncbi.nlm.nih.gov/gene/?term=109154
Creg1	Creg1	cellular repressor of E1A-stimulated genes 1	433375	https://www.ncbi.nlm.nih.gov/gene/?term=433375
Nat8l	Nat8l	N-acetyltransferase 8-like	269642	https://www.ncbi.nlm.nih.gov/gene/?term=269642
Snx12	Snx12	sorting nexin 12	55988	https://www.ncbi.nlm.nih.gov/gene/?term=55988
Trim56	Trim56	tripartite motif-containing 56	384309	https://www.ncbi.nlm.nih.gov/gene/?term=384309
St8sia3	St8sia3	ST8 alpha-N-acetyl-neuraminide alpha-2,8-sialyltransferase 3	20451	https://www.ncbi.nlm.nih.gov/gene/?term=20451
Ccdc3	Ccdc3	coiled-coil domain containing 3	74186	https://www.ncbi.nlm.nih.gov/gene/?term=74186
Slc7a8	Slc7a8	solute carrier family 7 (cationic amino acid transporter, y+ system), member 8	50934	https://www.ncbi.nlm.nih.gov/gene/?term=50934
Chst11	Chst11	carbohydrate sulfotransferase 11	58250	https://www.ncbi.nlm.nih.gov/gene/?term=58250
Dmrta1	Dmrta1	doublesex and mab-3 related transcription factor like family A1	242523	https://www.ncbi.nlm.nih.gov/gene/?term=242523
Tns4	Tns4	tensin 4	217169	https://www.ncbi.nlm.nih.gov/gene/?term=217169

Biology Open • Supplementary information

Atm	Atm	ataxia telangiectasia mutated	11920	https://www.ncbi.nlm.nih.gov/gene/?term=11920
Tmtc3	Tmtc3	transmembrane and tetratricopeptide repeat containing 3	237500	https://www.ncbi.nlm.nih.gov/gene/?term=237500
Nwd1	Nwd1	NACHT and WD repeat domain containing 1	319555	https://www.ncbi.nlm.nih.gov/gene/?term=319555
Oacyl	Oacyl	O-acyltransferase like	319888	https://www.ncbi.nlm.nih.gov/gene/?term=319888
Tbx10	Tbx10	T-box 10	109575	https://www.ncbi.nlm.nih.gov/gene/?term=109575
Ms4a4d	Ms4a4d	membrane-spanning 4-domains, subfamily A, member 4D	66607	https://www.ncbi.nlm.nih.gov/gene/?term=66607
Trnt1	Trnt1	tRNA nucleotidyl transferase, CCA-adding, 1	70047	https://www.ncbi.nlm.nih.gov/gene/?term=70047
Cnih1	Cnih1	cornichon family AMPA receptor auxiliary protein 1	12793	https://www.ncbi.nlm.nih.gov/gene/?term=12793
Slc4a5	Slc4a5	solute carrier family 4, sodium bicarbonate cotransporter, member 5	232156	https://www.ncbi.nlm.nih.gov/gene/?term=232156
Tmem243	Tmem243	transmembrane protein 243, mitochondrial	652925	https://www.ncbi.nlm.nih.gov/gene/?term=652925
CK137956	CK137956	cDNA sequence CK137956	635169	https://www.ncbi.nlm.nih.gov/gene/?term=635169
Ralgapb	Ralgapb	Ral GTPase activating protein, beta subunit (non-catalytic)	228850	https://www.ncbi.nlm.nih.gov/gene/?term=228850
Adgrb1	Adgrb1	adhesion G protein-coupled receptor B1	107831	https://www.ncbi.nlm.nih.gov/gene/?term=107831
Dynll2	Dynll2	dynein light chain LC8-type 2	68097	https://www.ncbi.nlm.nih.gov/gene/?term=68097
Dars	Dars	aspartyl-tRNA synthetase	226414	https://www.ncbi.nlm.nih.gov/gene/?term=226414
Cdc42	Cdc42	cell division cycle 42	12540	https://www.ncbi.nlm.nih.gov/gene/?term=12540
Creb3l2	Creb3l2	cAMP responsive element binding protein 3-like 2	208647	https://www.ncbi.nlm.nih.gov/gene/?term=208647
Grem2	Grem2	gremlin 2, DAN family BMP	23893	https://www.ncbi.nlm.nih.gov/gene/?term=23893

Biology Open • Supplementary information

		antagonist		
Synm	Synm	synemin, intermediate filament protein	233335	https://www.ncbi.nlm.nih.gov/gene/?term=233335
Csmd2	Csmd2	CUB and Sushi multiple domains 2	329942	https://www.ncbi.nlm.nih.gov/gene/?term=329942
Slc16a2	Slc16a2	solute carrier family 16 (monocarboxylic acid transporters), member 2	20502	https://www.ncbi.nlm.nih.gov/gene/?term=20502
Krtap5-5	Krtap5-5	keratin associated protein 5-5	114666	https://www.ncbi.nlm.nih.gov/gene/?term=114666
Zfp142	Zfp142	zinc finger protein 142	77264	https://www.ncbi.nlm.nih.gov/gene/?term=77264
Sez6l2	Sez6l2	seizure related 6 homolog like 2	233878	https://www.ncbi.nlm.nih.gov/gene/?term=233878
Syn1	Syn1	synapsin I	20964	https://www.ncbi.nlm.nih.gov/gene/?term=20964
Flot2	Flot2	flotillin 2	14252	https://www.ncbi.nlm.nih.gov/gene/?term=14252
Magi2	Magi2	membrane associated guanylate kinase, WW and PDZ domain containing 2	50791	https://www.ncbi.nlm.nih.gov/gene/?term=50791
Rab3d	Rab3d	RAB3D, member RAS oncogene family	19340	https://www.ncbi.nlm.nih.gov/gene/?term=19340
Gdf5	Gdf5	growth differentiation factor 5	14563	https://www.ncbi.nlm.nih.gov/gene/?term=14563
Pcna	Pcna	proliferating cell nuclear antigen	18538	https://www.ncbi.nlm.nih.gov/gene/?term=18538
Cgn	Cgn	cingulin	70737	https://www.ncbi.nlm.nih.gov/gene/?term=70737
Ntrk2	Ntrk2	neurotrophic tyrosine kinase, receptor, type 2	18212	https://www.ncbi.nlm.nih.gov/gene/?term=18212
Zfp157	Zfp157	zinc finger protein 157	72154	https://www.ncbi.nlm.nih.gov/gene/?term=72154
Rnf165	Rnf165	ring finger protein 165	225743	https://www.ncbi.nlm.nih.gov/gene/?term=225743
Slc41a1	Slc41a1	solute carrier family 41, member 1	98396	https://www.ncbi.nlm.nih.gov/gene/?term=98396
Zfp938	Zfp938	zinc finger protein 938	237411	https://www.ncbi.nlm.nih.gov/gene/?term=237411
Pak2	Pak2	p21 (RAC1) activated kinase 2	224105	https://www.ncbi.nlm.nih.gov/gene/?term=224105
Cldn23	Cldn23	claudin 23	71908	https://www.ncbi.nlm.nih.gov/gene/?term=71908
Glod5	Glod5	glyoxalase domain containing 5	69824	https://www.ncbi.nlm.nih.gov/gene/?term=69824

Biology Open • Supplementary information

Nucb2	Nucb2	nucleobindin 2	53322	https://www.ncbi.nlm.nih.gov/gene/?term=53322
Notch2	Notch2	notch 2	18129	https://www.ncbi.nlm.nih.gov/gene/?term=18129
Klk5	Klk5	kallikrein related-peptidase 5	68668	https://www.ncbi.nlm.nih.gov/gene/?term=68668
Ccdc174	Ccdc174	coiled-coil domain containing 174	232236	https://www.ncbi.nlm.nih.gov/gene/?term=232236
Kcne1	Kcne1	potassium voltage-gated channel, Isk-related subfamily, member 1	16509	https://www.ncbi.nlm.nih.gov/gene/?term=16509
Hps5	Hps5	HPS5, biogenesis of lysosomal organelles complex 2 subunit 2	246694	https://www.ncbi.nlm.nih.gov/gene/?term=246694
Prune2	Prune2	prune homolog 2	353211	https://www.ncbi.nlm.nih.gov/gene/?term=353211
Vps37c	Vps37c	vacuolar protein sorting 37C	107305	https://www.ncbi.nlm.nih.gov/gene/?term=107305
Ubox5	Ubox5	U box domain containing 5	140629	https://www.ncbi.nlm.nih.gov/gene/?term=140629
Rbak	Rbak	RB-associated KRAB zinc finger	57782	https://www.ncbi.nlm.nih.gov/gene/?term=57782
Pik3r2	Pik3r2	phosphoinositide-3-kinase regulatory subunit 2	18709	https://www.ncbi.nlm.nih.gov/gene/?term=18709
Mob1b	Mob1b	MOB kinase activator 1B	68473	https://www.ncbi.nlm.nih.gov/gene/?term=68473
Kalrn	Kalrn	kalirin, RhoGEF kinase	545156	https://www.ncbi.nlm.nih.gov/gene/?term=545156
Kcnj6	Kcnj6	potassium inwardly-rectifying channel, subfamily J, member 6	16522	https://www.ncbi.nlm.nih.gov/gene/?term=16522
Dag1	Dag1	dystroglycan 1	13138	https://www.ncbi.nlm.nih.gov/gene/?term=13138
Clvs2	Clvs2	clavesin 2	215890	https://www.ncbi.nlm.nih.gov/gene/?term=215890
Kdm1b	Kdm1b	lysine (K)-specific demethylase 1B	218214	https://www.ncbi.nlm.nih.gov/gene/?term=218214
Orc5	Orc5	origin recognition complex, subunit 5	26429	https://www.ncbi.nlm.nih.gov/gene/?term=26429
Wscd2	Wscd2	WSC domain containing 2	320916	https://www.ncbi.nlm.nih.gov/gene/?term=320916
Slc9a3r2	Slc9a3r2	solute carrier family 9 (sodium/hydrogen exchanger), member 3 regulator 2	65962	https://www.ncbi.nlm.nih.gov/gene/?term=65962
Shisa7	Shisa7	shisa family member 7	232813	https://www.ncbi.nlm.nih.gov/gene/?term=232813
Rsbn1l	Rsbn1l	round spermatid basic protein 1-like	242860	https://www.ncbi.nlm.nih.gov/gene/?term=242860
4930562C15Rik	4930562C15Rik	RIKEN cDNA 4930562C15 gene	78809	https://www.ncbi.nlm.nih.gov/gene/?term=78809

Entpd7	Entpd7	ectonucleoside triphosphate diphosphohydrolase 7	93685	https://www.ncbi.nlm.nih.gov/gene/?term=93685
Zranb2	Zranb2	zinc finger, RAN-binding domain containing 2	53861	https://www.ncbi.nlm.nih.gov/gene/?term=53861
Vamp2	Vamp2	vesicle-associated membrane protein 2	22318	https://www.ncbi.nlm.nih.gov/gene/?term=22318
Slc25a5	Slc25a5	NA	11740	https://www.ncbi.nlm.nih.gov/gene/?term=11740
Sdcbp	Sdcbp	syndecan binding protein	53378	https://www.ncbi.nlm.nih.gov/gene/?term=53378
Cplx1	Cplx1	complexin 1	12889	https://www.ncbi.nlm.nih.gov/gene/?term=12889
Kcnd1	Kcnd1	potassium voltage-gated channel, Shal-related family, member 1	16506	https://www.ncbi.nlm.nih.gov/gene/?term=16506
Ssr2	Ssr2	signal sequence receptor, beta	66256	https://www.ncbi.nlm.nih.gov/gene/?term=66256
Atp11b	Atp11b	ATPase, class VI, type 11B	76295	https://www.ncbi.nlm.nih.gov/gene/?term=76295
Syt2	Syt2	synaptotagmin II	20980	https://www.ncbi.nlm.nih.gov/gene/?term=20980
Slc16a6	Slc16a6	solute carrier family 16 (monocarboxylic acid transporters), member 6	104681	https://www.ncbi.nlm.nih.gov/gene/?term=104681
Bsn	Bsn	bassoon	12217	https://www.ncbi.nlm.nih.gov/gene/?term=12217
Irf4	Irf4	interferon regulatory factor 4	16364	https://www.ncbi.nlm.nih.gov/gene/?term=16364
Vdr	Vdr	vitamin D (1,25-dihydroxyvitamin D3) receptor	22337	https://www.ncbi.nlm.nih.gov/gene/?term=22337
Fam83h	Fam83h	family with sequence similarity 83, member H	105732	https://www.ncbi.nlm.nih.gov/gene/?term=105732
Crb2	Crb2	crumbs family member 2	241324	https://www.ncbi.nlm.nih.gov/gene/?term=241324
Nup210	Nup210	nucleoporin 210	54563	https://www.ncbi.nlm.nih.gov/gene/?term=54563
Sema4b	Sema4b	sema domain, immunoglobulin domain (Ig), transmembrane domain (TM) and short cytoplasmic domain, (semaphorin) 4B	20352	https://www.ncbi.nlm.nih.gov/gene/?term=20352

Was	Was	Wiskott-Aldrich syndrome	22376	https://www.ncbi.nlm.nih.gov/gene/?term=22376
Abhd6	Abhd6	abhydrolase domain containing 6	66082	https://www.ncbi.nlm.nih.gov/gene/?term=66082
AU040320	AU040320	expressed sequence AU040320	100317	https://www.ncbi.nlm.nih.gov/gene/?term=100317
Scn2b	Scn2b	sodium channel, voltage-gated, type II, beta	72821	https://www.ncbi.nlm.nih.gov/gene/?term=72821
Ptpn1	Ptpn1	protein tyrosine phosphatase, non-receptor type 1	19246	https://www.ncbi.nlm.nih.gov/gene/?term=19246
Dhx33	Dhx33	DEAH (Asp-Glu-Ala-His) box polypeptide 33	216877	https://www.ncbi.nlm.nih.gov/gene/?term=216877
Nbeal2	Nbeal2	neurobeachin-like 2	235627	https://www.ncbi.nlm.nih.gov/gene/?term=235627
Zfp523	Zfp523	zinc finger protein 523	224656	https://www.ncbi.nlm.nih.gov/gene/?term=224656
Pcgf6	Pcgf6	polycomb group ring finger 6	71041	https://www.ncbi.nlm.nih.gov/gene/?term=71041
Ier3ip1	Ier3ip1	immediate early response 3 interacting protein 1	66191	https://www.ncbi.nlm.nih.gov/gene/?term=66191
Lfng	Lfng	LFNG O-fucosylpeptide 3-beta-N-acetylglucosaminyltransferase	16848	https://www.ncbi.nlm.nih.gov/gene/?term=16848
Crem	Crem	cAMP responsive element modulator	12916	https://www.ncbi.nlm.nih.gov/gene/?term=12916
Klf13	Klf13	Kruppel-like factor 13	50794	https://www.ncbi.nlm.nih.gov/gene/?term=50794
Nrm	Nrm	nurim (nuclear envelope membrane protein)	106582	https://www.ncbi.nlm.nih.gov/gene/?term=106582
Ovol1	Ovol1	ovo like zinc finger 1	18426	https://www.ncbi.nlm.nih.gov/gene/?term=18426
Osbpl9	Osbpl9	oxysterol binding protein-like 9	100273	https://www.ncbi.nlm.nih.gov/gene/?term=100273
Bet1	Bet1	Bet1 golgi vesicular membrane trafficking protein	12068	https://www.ncbi.nlm.nih.gov/gene/?term=12068
Taz	Taz	tafazzin	66826	https://www.ncbi.nlm.nih.gov/gene/?term=66826
Sgpl1	Sgpl1	sphingosine phosphate lyase 1	20397	https://www.ncbi.nlm.nih.gov/gene/?term=20397
2610528J11Rik	2610528J11Rik	RIKEN cDNA 2610528J11 gene	66451	https://www.ncbi.nlm.nih.gov/gene/?term=66451
Samd10	Samd10	sterile alpha motif domain containing 10	229011	https://www.ncbi.nlm.nih.gov/gene/?term=229011

Biology Open • Supplementary information

Npl	Npl	N-acetylneuraminate pyruvate lyase	74091	https://www.ncbi.nlm.nih.gov/gene/?term=74091
Sema4d	Sema4d	sema domain, immunoglobulin domain (Ig), transmembrane domain (TM) and short cytoplasmic domain, (semaphorin) 4D	20354	https://www.ncbi.nlm.nih.gov/gene/?term=20354
Sbno1	Sbno1	strawberry notch 1	243272	https://www.ncbi.nlm.nih.gov/gene/?term=243272
Slc35a4	Slc35a4	solute carrier family 35, member A4	67843	https://www.ncbi.nlm.nih.gov/gene/?term=67843
Gga2	Gga2	golgi associated, gamma adaptin ear containing, ARF binding protein 2	74105	https://www.ncbi.nlm.nih.gov/gene/?term=74105
Triap1	Triap1	TP53 regulated inhibitor of apoptosis 1	69076	https://www.ncbi.nlm.nih.gov/gene/?term=69076
Tmem161b	Tmem161b	transmembrane protein 161B	72745	https://www.ncbi.nlm.nih.gov/gene/?term=72745
Blzf1	Blzf1	basic leucine zipper nuclear factor 1	66352	https://www.ncbi.nlm.nih.gov/gene/?term=66352
Tmem120b	Tmem120b	transmembrane protein 120B	330189	https://www.ncbi.nlm.nih.gov/gene/?term=330189
Slc25a35	Slc25a35	solute carrier family 25, member 35	71998	https://www.ncbi.nlm.nih.gov/gene/?term=71998
Rap1a	Rap1a	RAS-related protein 1a	109905	https://www.ncbi.nlm.nih.gov/gene/?term=109905
Grsf1	Grsf1	G-rich RNA sequence binding factor 1	231413	https://www.ncbi.nlm.nih.gov/gene/?term=231413
Tspan12	Tspan12	tetraspanin 12	269831	https://www.ncbi.nlm.nih.gov/gene/?term=269831
Ubr7	Ubr7	ubiquitin protein ligase E3 component n-recognin 7 (putative)	66622	https://www.ncbi.nlm.nih.gov/gene/?term=66622
Rbm20	Rbm20	RNA binding motif protein 20	73713	https://www.ncbi.nlm.nih.gov/gene/?term=73713
Xirp1	Xirp1	xin actin-binding repeat containing 1	22437	https://www.ncbi.nlm.nih.gov/gene/?term=22437
Necab3	Necab3	N-terminal EF-hand calcium binding protein 3	56846	https://www.ncbi.nlm.nih.gov/gene/?term=56846
Diras1	Diras1	DIRAS family, GTP-binding RAS-like 1	208666	https://www.ncbi.nlm.nih.gov/gene/?term=208666
Ier2	Ier2	immediate early response 2	15936	https://www.ncbi.nlm.nih.gov/gene/?term=15936
Sh3bp5l	Sh3bp5l	SH3 binding domain protein 5 like	79566	https://www.ncbi.nlm.nih.gov/gene/?term=79566

Biology Open • Supplementary information

Dus1l	Dus1l	dihydrouridine synthase 1-like (S. cerevisiae)	68730	https://www.ncbi.nlm.nih.gov/gene/?term=68730
Eif1ad	Eif1ad	eukaryotic translation initiation factor 1A domain containing	69860	https://www.ncbi.nlm.nih.gov/gene/?term=69860
Cdc42se1	Cdc42se1	CDC42 small effector 1	57912	https://www.ncbi.nlm.nih.gov/gene/?term=57912
Dynlt3	Dynlt3	dynein light chain Tctex-type 3	67117	https://www.ncbi.nlm.nih.gov/gene/?term=67117
Sec14l2	Sec14l2	SEC14-like lipid binding 2	67815	https://www.ncbi.nlm.nih.gov/gene/?term=67815
Tmprss13	Tmprss13	transmembrane protease, serine 13	214531	https://www.ncbi.nlm.nih.gov/gene/?term=214531
Cdc42bpg	Cdc42bpg	CDC42 binding protein kinase gamma (DMPK-like)	240505	https://www.ncbi.nlm.nih.gov/gene/?term=240505
Tnfsf4	Tnfsf4	tumor necrosis factor (ligand) superfamily, member 4	22164	https://www.ncbi.nlm.nih.gov/gene/?term=22164
Ccnj	Ccnj	cyclin J	240665	https://www.ncbi.nlm.nih.gov/gene/?term=240665
Ptpn7	Ptpn7	protein tyrosine phosphatase, non-receptor type 7	320139	https://www.ncbi.nlm.nih.gov/gene/?term=320139
Lactb	Lactb	lactamase, beta	80907	https://www.ncbi.nlm.nih.gov/gene/?term=80907
Gcnt1	Gcnt1	glucosaminyl (N-acetyl) transferase 1, core 2	14537	https://www.ncbi.nlm.nih.gov/gene/?term=14537
Caprin1	Caprin1	cell cycle associated protein 1	53872	https://www.ncbi.nlm.nih.gov/gene/?term=53872
Wnk1	Wnk1	WNK lysine deficient protein kinase 1	232341	https://www.ncbi.nlm.nih.gov/gene/?term=232341
Ncbp2	Ncbp2	nuclear cap binding protein subunit 2	68092	https://www.ncbi.nlm.nih.gov/gene/?term=68092
Nkapl	Nkapl	NFKB activating protein-like	66707	https://www.ncbi.nlm.nih.gov/gene/?term=66707
Ap1s1	Ap1s1	adaptor protein complex AP-1, sigma 1	11769	https://www.ncbi.nlm.nih.gov/gene/?term=11769
Xpo6	Xpo6	exportin 6	74204	https://www.ncbi.nlm.nih.gov/gene/?term=74204
Cebpz	Cebpz	CCAAT/enhancer binding protein zeta	12607	https://www.ncbi.nlm.nih.gov/gene/?term=12607
Ust	Ust	uronyl-2-sulfotransferase	338362	https://www.ncbi.nlm.nih.gov/gene/?term=338362
Sprn	Sprn	shadow of prion protein	212518	https://www.ncbi.nlm.nih.gov/gene/?term=212518

Cap1	Cap1	CAP, adenylate cyclase-associated protein 1 (yeast)	12331	https://www.ncbi.nlm.nih.gov/gene/?term=12331
Pax7	Pax7	paired box 7	18509	https://www.ncbi.nlm.nih.gov/gene/?term=18509
Cttnbp2nl	Cttnbp2nl	CTTNBP2 N-terminal like	80281	https://www.ncbi.nlm.nih.gov/gene/?term=80281
Caap1	Caap1	caspase activity and apoptosis inhibitor 1	67770	https://www.ncbi.nlm.nih.gov/gene/?term=67770
Mtx1	Mtx1	metaxin 1	17827	https://www.ncbi.nlm.nih.gov/gene/?term=17827
Plxna4	Plxna4	plexin A4	243743	https://www.ncbi.nlm.nih.gov/gene/?term=243743
Tpm3	Tpm3	tropomyosin 3, gamma	59069	https://www.ncbi.nlm.nih.gov/gene/?term=59069
Stc2	Stc2	stanniocalcin 2	20856	https://www.ncbi.nlm.nih.gov/gene/?term=20856
Phax	Phax	phosphorylated adaptor for RNA export	56698	https://www.ncbi.nlm.nih.gov/gene/?term=56698
Arcn1	Arcn1	archain 1	213827	https://www.ncbi.nlm.nih.gov/gene/?term=213827
Cmpk2	Cmpk2	cytidine monophosphate (UMP-CMP) kinase 2, mitochondrial	22169	https://www.ncbi.nlm.nih.gov/gene/?term=22169
G6pdx	G6pdx	glucose-6-phosphate dehydrogenase X-linked	14381	https://www.ncbi.nlm.nih.gov/gene/?term=14381
Map1a	Map1a	microtubule-associated protein 1 A	17754	https://www.ncbi.nlm.nih.gov/gene/?term=17754
4930563E22Rik	4930563E22Rik	RIKEN cDNA 4930563E22 gene	75304	https://www.ncbi.nlm.nih.gov/gene/?term=75304
Mon2	Mon2	MON2 homolog, regulator of endosome to Golgi trafficking	67074	https://www.ncbi.nlm.nih.gov/gene/?term=67074
Hnrnpu	Hnrnpu	heterogeneous nuclear ribonucleoprotein U	51810	https://www.ncbi.nlm.nih.gov/gene/?term=51810
Tra2b	Tra2b	transformer 2 beta	20462	https://www.ncbi.nlm.nih.gov/gene/?term=20462
Cited2	Cited2	Cbp/p300-interacting transactivator, with Glu/Asp-rich carboxy-terminal domain, 2	17684	https://www.ncbi.nlm.nih.gov/gene/?term=17684
Hsp90b1	Hsp90b1	heat shock protein 90, beta (Grp94), member 1	22027	https://www.ncbi.nlm.nih.gov/gene/?term=22027

Biology Open • Supplementary information

Ankrd29	Ankrd29	ankyrin repeat domain 29	225187	https://www.ncbi.nlm.nih.gov/gene/?term=225187
Gja1	Gja1	gap junction protein, alpha 1	14609	https://www.ncbi.nlm.nih.gov/gene/?term=14609
Ss18	Ss18	SS18, nBAF chromatin remodeling complex subunit	268996	https://www.ncbi.nlm.nih.gov/gene/?term=268996
Mfsd14a	Mfsd14a	major facilitator superfamily domain containing 14A	15247	https://www.ncbi.nlm.nih.gov/gene/?term=15247
Esp34	Esp34	exocrine gland secreted peptide 34	100126773	https://www.ncbi.nlm.nih.gov/gene/?term=100126
Adprm	Adprm	ADP-ribose/CDP-alcohol diphosphatase, manganese dependent	66358	https://www.ncbi.nlm.nih.gov/gene/?term=66358
Ip6k2	Ip6k2	inositol hexaphosphate kinase 2	76500	https://www.ncbi.nlm.nih.gov/gene/?term=76500
Tex11	Tex11	testis expressed gene 11	83558	https://www.ncbi.nlm.nih.gov/gene/?term=83558
Hinfp	Hinfp	histone H4 transcription factor	102423	https://www.ncbi.nlm.nih.gov/gene/?term=102423
Osbpl7	Osbpl7	oxysterol binding protein-like 7	71240	https://www.ncbi.nlm.nih.gov/gene/?term=71240
Ccsap	Ccsap	centriole, cilia and spindle associated protein	73420	https://www.ncbi.nlm.nih.gov/gene/?term=73420
Ddx5	Ddx5	DEAD (Asp-Glu-Ala-Asp) box polypeptide 5	13207	https://www.ncbi.nlm.nih.gov/gene/?term=13207
Tppp	Tppp	tubulin polymerization promoting protein	72948	https://www.ncbi.nlm.nih.gov/gene/?term=72948
Serp1	Serp1	stress-associated endoplasmic reticulum protein 1	28146	https://www.ncbi.nlm.nih.gov/gene/?term=28146
Mmd2	Mmd2	monocyte to macrophage differentiation-associated 2	75104	https://www.ncbi.nlm.nih.gov/gene/?term=75104
Kmt2e	Kmt2e	lysine (K)-specific methyltransferase 2E	69188	https://www.ncbi.nlm.nih.gov/gene/?term=69188
Tmem178	Tmem178	transmembrane protein 178	68027	https://www.ncbi.nlm.nih.gov/gene/?term=68027
Dach2	Dach2	dachshund family transcription factor 2	93837	https://www.ncbi.nlm.nih.gov/gene/?term=93837

Biology Open • Supplementary information

Tmem18	Tmem18	transmembrane protein 18	211986	https://www.ncbi.nlm.nih.gov/gene/?term=211986
Unc119b	Unc119b	unc-119 lipid binding chaperone B	106840	https://www.ncbi.nlm.nih.gov/gene/?term=106840
Hspd1	Hspd1	heat shock protein 1 (chaperonin)	15510	https://www.ncbi.nlm.nih.gov/gene/?term=15510
Cdk14	Cdk14	cyclin-dependent kinase 14	18647	https://www.ncbi.nlm.nih.gov/gene/?term=18647
Adar	Adar	adenosine deaminase, RNA-specific	56417	https://www.ncbi.nlm.nih.gov/gene/?term=56417
Sec22b	Sec22b	SEC22 homolog B, vesicle trafficking protein	20333	https://www.ncbi.nlm.nih.gov/gene/?term=20333
Neto1	Neto1	neuropilin (NRP) and tolloid (TLL)-like 1	246317	https://www.ncbi.nlm.nih.gov/gene/?term=246317
Smim14	Smim14	small integral membrane protein 14	68552	https://www.ncbi.nlm.nih.gov/gene/?term=68552
Ube4a	Ube4a	ubiquitination factor E4A	140630	https://www.ncbi.nlm.nih.gov/gene/?term=140630
Ppib	Ppib	peptidylprolyl isomerase B	19035	https://www.ncbi.nlm.nih.gov/gene/?term=19035
Wdr48	Wdr48	WD repeat domain 48	67561	https://www.ncbi.nlm.nih.gov/gene/?term=67561
Syt1	Syt1	synaptotagmin I	20979	https://www.ncbi.nlm.nih.gov/gene/?term=20979
Ywhaz	Ywhaz	tyrosine 3-monooxygenase/tryptophan 5-monooxygenase activation protein, zeta polypeptide	22631	https://www.ncbi.nlm.nih.gov/gene/?term=22631
Gnl3l	Gnl3l	guanine nucleotide binding protein-like 3 (nucleolar)-like	237107	https://www.ncbi.nlm.nih.gov/gene/?term=237107
Zcchc18	Zcchc18	zinc finger, CCHC domain containing 18	66995	https://www.ncbi.nlm.nih.gov/gene/?term=66995
Snx27	Snx27	sorting nexin family member 27	76742	https://www.ncbi.nlm.nih.gov/gene/?term=76742
Kat7	Kat7	K(lysine) acetyltransferase 7	217127	https://www.ncbi.nlm.nih.gov/gene/?term=217127
Upp2	Upp2	uridine phosphorylase 2	76654	https://www.ncbi.nlm.nih.gov/gene/?term=76654
Neurog2	Neurog2	neurogenin 2	11924	https://www.ncbi.nlm.nih.gov/gene/?term=11924
Zfp729b	Zfp729b	zinc finger protein 729b	100416706	https://www.ncbi.nlm.nih.gov/gene/?term=100416
Tspan3	Tspan3	tetraspanin 3	56434	https://www.ncbi.nlm.nih.gov/gene/?term=56434
Pes1	Pes1	pescadillo ribosomal biogenesis	64934	https://www.ncbi.nlm.nih.gov/gene/?term=64934

		factor 1		
Tpbpa	Tpbpa	trophoblast specific protein alpha	21984	https://www.ncbi.nlm.nih.gov/gene/?term=21984
Zfp503	Zfp503	zinc finger protein 503	218820	https://www.ncbi.nlm.nih.gov/gene/?term=218820
Eme1	Eme1	essential meiotic structure-specific endonuclease 1	268465	https://www.ncbi.nlm.nih.gov/gene/?term=268465
Ubtd2	Ubtd2	ubiquitin domain containing 2	327900	https://www.ncbi.nlm.nih.gov/gene/?term=327900
Pdgfrl	Pdgfrl	platelet-derived growth factor receptor-like	68797	https://www.ncbi.nlm.nih.gov/gene/?term=68797
Map10	Map10	microtubule-associated protein 10	74393	https://www.ncbi.nlm.nih.gov/gene/?term=74393
Srgap1	Srgap1	SLIT-ROBO Rho GTPase activating protein 1	117600	https://www.ncbi.nlm.nih.gov/gene/?term=117600
Npm1	Npm1	nucleophosmin 1	18148	https://www.ncbi.nlm.nih.gov/gene/?term=18148
Zmynd11	Zmynd11	zinc finger, MYND domain containing 11	66505	https://www.ncbi.nlm.nih.gov/gene/?term=66505
Lin9	Lin9	lin-9 homolog (C. elegans)	72568	https://www.ncbi.nlm.nih.gov/gene/?term=72568
Psmc6	Psmc6	proteasome (prosome, macropain) 26S subunit, ATPase, 6	67089	https://www.ncbi.nlm.nih.gov/gene/?term=67089
Ms4a4b	Ms4a4b	membrane-spanning 4-domains, subfamily A, member 4B	60361	https://www.ncbi.nlm.nih.gov/gene/?term=60361
2310030G06Rik	2310030G06Rik	RIKEN cDNA 2310030G06 gene	66952	https://www.ncbi.nlm.nih.gov/gene/?term=66952
Rab11a	Rab11a	RAB11A, member RAS oncogene family	53869	https://www.ncbi.nlm.nih.gov/gene/?term=53869
Trpc3	Trpc3	transient receptor potential cation channel, subfamily C, member 3	22065	https://www.ncbi.nlm.nih.gov/gene/?term=22065
Pla2g4e	Pla2g4e	phospholipase A2, group IVE	329502	https://www.ncbi.nlm.nih.gov/gene/?term=329502
Bmp2k	Bmp2k	BMP2 inducible kinase	140780	https://www.ncbi.nlm.nih.gov/gene/?term=140780
Dab2ip	Dab2ip	disabled 2 interacting protein	69601	https://www.ncbi.nlm.nih.gov/gene/?term=69601
Ubxn7	Ubxn7	UBX domain protein 7	224111	https://www.ncbi.nlm.nih.gov/gene/?term=224111
Snx6	Snx6	sorting nexin 6	72183	https://www.ncbi.nlm.nih.gov/gene/?term=72183

Biology Open • Supplementary information

Cdkl1	Cdkl1	cyclin-dependent kinase-like 1 (CDC2-related kinase)	71091	https://www.ncbi.nlm.nih.gov/gene/?term=71091
Klb	Klb	klotho beta	83379	https://www.ncbi.nlm.nih.gov/gene/?term=83379
Dnajc6	Dnajc6	DnaJ heat shock protein family (Hsp40) member C6	72685	https://www.ncbi.nlm.nih.gov/gene/?term=72685
Elovl7	Elovl7	ELOVL family member 7, elongation of long chain fatty acids (yeast)	74559	https://www.ncbi.nlm.nih.gov/gene/?term=74559
Inpp5j	Inpp5j	inositol polyphosphate 5-phosphatase J	170835	https://www.ncbi.nlm.nih.gov/gene/?term=170835
Sdk1	Sdk1	sidekick cell adhesion molecule 1	330222	https://www.ncbi.nlm.nih.gov/gene/?term=330222
Gprc5b	Gprc5b	G protein-coupled receptor, family C, group 5, member B	64297	https://www.ncbi.nlm.nih.gov/gene/?term=64297
Ubr1	Ubr1	ubiquitin protein ligase E3 component n-recognin 1	22222	https://www.ncbi.nlm.nih.gov/gene/?term=22222
Mef2d	Mef2d	myocyte enhancer factor 2D	17261	https://www.ncbi.nlm.nih.gov/gene/?term=17261
Pigg	Pigg	phosphatidylinositol glycan anchor biosynthesis, class G	433931	https://www.ncbi.nlm.nih.gov/gene/?term=433931
Olfr976	Olfr976	olfactory receptor 976	258364	https://www.ncbi.nlm.nih.gov/gene/?term=258364
Atg14	Atg14	autophagy related 14	100504663	https://www.ncbi.nlm.nih.gov/gene/?term=100504
Nubpl	Nubpl	nucleotide binding protein-like	76826	https://www.ncbi.nlm.nih.gov/gene/?term=76826
Nipa2	Nipa2	non imprinted in Prader-Willi/Angelman syndrome 2 homolog (human)	93790	https://www.ncbi.nlm.nih.gov/gene/?term=93790
Stard7	Stard7	START domain containing 7	99138	https://www.ncbi.nlm.nih.gov/gene/?term=99138
Tpm4	Tpm4	tropomyosin 4	326618	https://www.ncbi.nlm.nih.gov/gene/?term=326618
Tnfsf8	Tnfsf8	tumor necrosis factor (ligand) superfamily, member 8	21949	https://www.ncbi.nlm.nih.gov/gene/?term=21949
Mbtps1	Mbtps1	membrane-bound transcription factor peptidase, site 1	56453	https://www.ncbi.nlm.nih.gov/gene/?term=56453

Cyb561d1	Cyb561d1	cytochrome b-561 domain containing 1	72023	https://www.ncbi.nlm.nih.gov/gene/?term=72023
Abcf3	Abcf3	ATP-binding cassette, sub-family F (GCN20), member 3	27406	https://www.ncbi.nlm.nih.gov/gene/?term=27406
Nek9	Nek9	NIMA (never in mitosis gene a)-related expressed kinase 9	217718	https://www.ncbi.nlm.nih.gov/gene/?term=217718
Npdc1	Npdc1	neural proliferation, differentiation and control 1	18146	https://www.ncbi.nlm.nih.gov/gene/?term=18146
Alcam	Alcam	activated leukocyte cell adhesion molecule	11658	https://www.ncbi.nlm.nih.gov/gene/?term=11658
Cyth1	Cyth1	cytohesin 1	19157	https://www.ncbi.nlm.nih.gov/gene/?term=19157
Ccdc181	Ccdc181	coiled-coil domain containing 181	74895	https://www.ncbi.nlm.nih.gov/gene/?term=74895
Ccin	Ccin	calicin	442829	https://www.ncbi.nlm.nih.gov/gene/?term=442829
Tmem252	Tmem252	transmembrane protein 252	226040	https://www.ncbi.nlm.nih.gov/gene/?term=226040
Apoh	Apoh	apolipoprotein H	11818	https://www.ncbi.nlm.nih.gov/gene/?term=11818
Pitpnm3	Pitpnm3	PITPNM family member 3	327958	https://www.ncbi.nlm.nih.gov/gene/?term=327958
Srp19	Srp19	signal recognition particle 19	66384	https://www.ncbi.nlm.nih.gov/gene/?term=66384
Cwc27	Cwc27	CWC27 spliceosome-associated protein	67285	https://www.ncbi.nlm.nih.gov/gene/?term=67285
Olfm3	Olfm3	olfactomedin 3	229759	https://www.ncbi.nlm.nih.gov/gene/?term=229759
Rmdn3	Rmdn3	regulator of microtubule dynamics 3	67809	https://www.ncbi.nlm.nih.gov/gene/?term=67809
Crym	Crym	crystallin, mu	12971	https://www.ncbi.nlm.nih.gov/gene/?term=12971
Tfcp2l1	Tfcp2l1	transcription factor CP2-like 1	81879	https://www.ncbi.nlm.nih.gov/gene/?term=81879
Actr6	Actr6	ARP6 actin-related protein 6	67019	https://www.ncbi.nlm.nih.gov/gene/?term=67019
Apbb2	Apbb2	amyloid beta (A4) precursor protein-binding, family B, member 2	11787	https://www.ncbi.nlm.nih.gov/gene/?term=11787
Etfrf1	Etfrf1	electron transfer flavoprotein regulatory factor 1	67636	https://www.ncbi.nlm.nih.gov/gene/?term=67636

Hsd3b6	Hsd3b6	hydroxy-delta-5-steroid dehydrogenase, 3 beta- and steroid delta-isomerase 6	15497	https://www.ncbi.nlm.nih.gov/gene/?term=15497
Tuba4a	Tuba4a	tubulin, alpha 4A	22145	https://www.ncbi.nlm.nih.gov/gene/?term=22145
Sult1c2	Sult1c2	sulfotransferase family, cytosolic, 1C, member 2	69083	https://www.ncbi.nlm.nih.gov/gene/?term=69083
Ghitm	Ghitm	growth hormone inducible transmembrane protein	66092	https://www.ncbi.nlm.nih.gov/gene/?term=66092
9530068E07Rik	9530068E07Rik	RIKEN cDNA 9530068E07 gene	213673	https://www.ncbi.nlm.nih.gov/gene/?term=213673
B230219D22Rik	B230219D22Rik	RIKEN cDNA B230219D22 gene	78521	https://www.ncbi.nlm.nih.gov/gene/?term=78521
Cntrob	Cntrob	centrobin, centrosomal BRCA2 interacting protein	216846	https://www.ncbi.nlm.nih.gov/gene/?term=216846
Wbp2	Wbp2	WW domain binding protein 2	22378	https://www.ncbi.nlm.nih.gov/gene/?term=22378
Spats2l	Spats2l	spermatogenesis associated, serine-rich 2-like	67198	https://www.ncbi.nlm.nih.gov/gene/?term=67198
Gpc4	Gpc4	glypican 4	14735	https://www.ncbi.nlm.nih.gov/gene/?term=14735
Tut7	Tut7	terminal uridylyl transferase 7	214290	https://www.ncbi.nlm.nih.gov/gene/?term=214290
Slc12a1	Slc12a1	solute carrier family 12, member 1	20495	https://www.ncbi.nlm.nih.gov/gene/?term=20495
Gm11992	Gm11992	predicted gene 11992	626870	https://www.ncbi.nlm.nih.gov/gene/?term=626870
Rbbp4	Rbbp4	retinoblastoma binding protein 4, chromatin remodeling factor	19646	https://www.ncbi.nlm.nih.gov/gene/?term=19646
Megf11	Megf11	multiple EGF-like-domains 11	214058	https://www.ncbi.nlm.nih.gov/gene/?term=214058
Tex9	Tex9	testis expressed gene 9	21778	https://www.ncbi.nlm.nih.gov/gene/?term=21778
Drg2	Drg2	developmentally regulated GTP binding protein 2	13495	https://www.ncbi.nlm.nih.gov/gene/?term=13495
Ryr3	Ryr3	ryanodine receptor 3	20192	https://www.ncbi.nlm.nih.gov/gene/?term=20192
Ang4	Ang4	angiogenin, ribonuclease A family, member 4	219033	https://www.ncbi.nlm.nih.gov/gene/?term=219033

Biology Open • Supplementary information

Ugt2b1	Ugt2b1	UDP glucuronosyltransferase 2 family, polypeptide B1	71773	https://www.ncbi.nlm.nih.gov/gene/?term=71773
Cnr1	Cnr1	cannabinoid receptor 1 (brain)	12801	https://www.ncbi.nlm.nih.gov/gene/?term=12801
Cmtr1	Cmtr1	cap methyltransferase 1	74157	https://www.ncbi.nlm.nih.gov/gene/?term=74157
Cdkn2aip	Cdkn2aip	CDKN2A interacting protein	70925	https://www.ncbi.nlm.nih.gov/gene/?term=70925
Etfa	Etfa	electron transferring flavoprotein, alpha polypeptide	110842	https://www.ncbi.nlm.nih.gov/gene/?term=110842
Zfp407	Zfp407	zinc finger protein 407	240476	https://www.ncbi.nlm.nih.gov/gene/?term=240476
Tmem43	Tmem43	transmembrane protein 43	74122	https://www.ncbi.nlm.nih.gov/gene/?term=74122
Peg12	Peg12	paternally expressed 12	27412	https://www.ncbi.nlm.nih.gov/gene/?term=27412
Mapk9	Mapk9	mitogen-activated protein kinase 9	26420	https://www.ncbi.nlm.nih.gov/gene/?term=26420
Carnmt1	Carnmt1	carnosine N-methyltransferase 1	67383	https://www.ncbi.nlm.nih.gov/gene/?term=67383
Cox7b	Cox7b	cytochrome c oxidase subunit 7B	66142	https://www.ncbi.nlm.nih.gov/gene/?term=66142
Myo5c	Myo5c	myosin VC	208943	https://www.ncbi.nlm.nih.gov/gene/?term=208943
Pmel	Pmel	premelanosome protein	20431	https://www.ncbi.nlm.nih.gov/gene/?term=20431
Dlgap4	Dlgap4	DLG associated protein 4	228836	https://www.ncbi.nlm.nih.gov/gene/?term=228836
Ewsr1	Ewsr1	Ewing sarcoma breakpoint region 1	14030	https://www.ncbi.nlm.nih.gov/gene/?term=14030
Zfp26	Zfp26	zinc finger protein 26	22688	https://www.ncbi.nlm.nih.gov/gene/?term=22688
Lrp2bp	Lrp2bp	Lrp2 binding protein	67620	https://www.ncbi.nlm.nih.gov/gene/?term=67620
Nemp1	Nemp1	nuclear envelope integral membrane protein 1	210035	https://www.ncbi.nlm.nih.gov/gene/?term=210035
Ppp1r3b	Ppp1r3b	protein phosphatase 1, regulatory subunit 3B	244416	https://www.ncbi.nlm.nih.gov/gene/?term=244416
Npy5r	Npy5r	neuropeptide Y receptor Y5	18168	https://www.ncbi.nlm.nih.gov/gene/?term=18168
Clec2h	Clec2h	C-type lectin domain family 2, member h	94071	https://www.ncbi.nlm.nih.gov/gene/?term=94071
4930596D02Rik	4930596D02Rik	RIKEN cDNA 4930596D02 gene	239036	https://www.ncbi.nlm.nih.gov/gene/?term=239036
Chmp3	Chmp3	charged multivesicular body protein 3	66700	https://www.ncbi.nlm.nih.gov/gene/?term=66700

Biology Open • Supplementary information

Hey1	Hey1	hairy/enhancer-of-split related with YRPW motif 1	15213	https://www.ncbi.nlm.nih.gov/gene/?term=15213
Foxo1	Foxo1	forkhead box O1	56458	https://www.ncbi.nlm.nih.gov/gene/?term=56458
Srsf4	Srsf4	serine/arginine-rich splicing factor 4	57317	https://www.ncbi.nlm.nih.gov/gene/?term=57317
Slc16a10	Slc16a10	solute carrier family 16 (monocarboxylic acid transporters), member 10	72472	https://www.ncbi.nlm.nih.gov/gene/?term=72472
Rtl5	Rtl5	retrotransposon Gag like 5	331474	https://www.ncbi.nlm.nih.gov/gene/?term=331474
Piwil1	Piwil1	piwi-like RNA-mediated gene silencing 1	57749	https://www.ncbi.nlm.nih.gov/gene/?term=57749
Akain1	Akain1	A kinase (PRKA) anchor inhibitor 1	320722	https://www.ncbi.nlm.nih.gov/gene/?term=320722
Agk	Agk	acylglycerol kinase	69923	https://www.ncbi.nlm.nih.gov/gene/?term=69923
1110004F10Rik	1110004F10Rik	RIKEN cDNA 1110004F10 gene	56372	https://www.ncbi.nlm.nih.gov/gene/?term=56372
Ugt8a	Ugt8a	UDP galactosyltransferase 8A	22239	https://www.ncbi.nlm.nih.gov/gene/?term=22239
Kif20a	Kif20a	kinesin family member 20A	19348	https://www.ncbi.nlm.nih.gov/gene/?term=19348
Tbx4	Tbx4	T-box 4	21387	https://www.ncbi.nlm.nih.gov/gene/?term=21387
Fzd5	Fzd5	frizzled class receptor 5	14367	https://www.ncbi.nlm.nih.gov/gene/?term=14367
Csn2	Csn2	casein beta	12991	https://www.ncbi.nlm.nih.gov/gene/?term=12991
Cemip	Cemip	cell migration inducing protein, hyaluronan binding	80982	https://www.ncbi.nlm.nih.gov/gene/?term=80982
Nipbl	Nipbl	NIPBL cohesin loading factor	71175	https://www.ncbi.nlm.nih.gov/gene/?term=71175
Myo9a	Myo9a	myosin IXa	270163	https://www.ncbi.nlm.nih.gov/gene/?term=270163
Ltbp1	Ltbp1	latent transforming growth factor beta binding protein 1	268977	https://www.ncbi.nlm.nih.gov/gene/?term=268977
Srpk2	Srpk2	serine/arginine-rich protein specific kinase 2	20817	https://www.ncbi.nlm.nih.gov/gene/?term=20817
N6amt1	N6amt1	N-6 adenine-specific DNA methyltransferase 1 (putative)	67768	https://www.ncbi.nlm.nih.gov/gene/?term=67768

Ythdf1	Ythdf1	YTH N6-methyladenosine RNA binding protein 1	228994	https://www.ncbi.nlm.nih.gov/gene/?term=228994
Cebpb	Cebpb	CCAAT/enhancer binding protein (C/EBP), beta	12608	https://www.ncbi.nlm.nih.gov/gene/?term=12608
Numbl	Numbl	numb-like	18223	https://www.ncbi.nlm.nih.gov/gene/?term=18223
Parp8	Parp8	poly (ADP-ribose) polymerase family, member 8	52552	https://www.ncbi.nlm.nih.gov/gene/?term=52552
Fancf	Fancf	Fanconi anemia, complementation group F	100040608	https://www.ncbi.nlm.nih.gov/gene/?term=100040
Tead3	Tead3	TEA domain family member 3	21678	https://www.ncbi.nlm.nih.gov/gene/?term=21678
Rfx4	Rfx4	regulatory factor X, 4 (influences HLA class II expression)	71137	https://www.ncbi.nlm.nih.gov/gene/?term=71137
Pnrc1	Pnrc1	proline-rich nuclear receptor coactivator 1	108767	https://www.ncbi.nlm.nih.gov/gene/?term=108767
Nop58	Nop58	NOP58 ribonucleoprotein	55989	https://www.ncbi.nlm.nih.gov/gene/?term=55989
Plk4	Plk4	polo like kinase 4	20873	https://www.ncbi.nlm.nih.gov/gene/?term=20873
Pde4b	Pde4b	phosphodiesterase 4B, cAMP specific	18578	https://www.ncbi.nlm.nih.gov/gene/?term=18578
Fezf1	Fezf1	Fez family zinc finger 1	73191	https://www.ncbi.nlm.nih.gov/gene/?term=73191
Csf3r	Csf3r	colony stimulating factor 3 receptor (granulocyte)	12986	https://www.ncbi.nlm.nih.gov/gene/?term=12986
Prim1	Prim1	DNA primase, p49 subunit	19075	https://www.ncbi.nlm.nih.gov/gene/?term=19075
Suv39h2	Suv39h2	suppressor of variegation 3-9 2	64707	https://www.ncbi.nlm.nih.gov/gene/?term=64707
Zfp281	Zfp281	zinc finger protein 281	226442	https://www.ncbi.nlm.nih.gov/gene/?term=226442
Dlg3	Dlg3	discs large MAGUK scaffold protein 3	53310	https://www.ncbi.nlm.nih.gov/gene/?term=53310
Plat	Plat	plasminogen activator, tissue	18791	https://www.ncbi.nlm.nih.gov/gene/?term=18791
Spag7	Spag7	sperm associated antigen 7	216873	https://www.ncbi.nlm.nih.gov/gene/?term=216873
Setd2	Setd2	SET domain containing 2	235626	https://www.ncbi.nlm.nih.gov/gene/?term=235626
Brpf1	Brpf1	bromodomain and PHD finger containing, 1	78783	https://www.ncbi.nlm.nih.gov/gene/?term=78783

Spata7	Spata7	spermatogenesis associated 7	104871	https://www.ncbi.nlm.nih.gov/gene/?term=104871
Snx17	Snx17	sorting nexin 17	266781	https://www.ncbi.nlm.nih.gov/gene/?term=266781
P4ha1	P4ha1	procollagen-proline, 2-oxoglutarate 4-dioxygenase (proline 4-hydroxylase), alpha 1 polypeptide	18451	https://www.ncbi.nlm.nih.gov/gene/?term=18451
Prdm15	Prdm15	PR domain containing 15	114604	https://www.ncbi.nlm.nih.gov/gene/?term=114604
Pkm	Pkm	pyruvate kinase, muscle	18746	https://www.ncbi.nlm.nih.gov/gene/?term=18746
Micu3	Micu3	mitochondrial calcium uptake family, member 3	78506	https://www.ncbi.nlm.nih.gov/gene/?term=78506
Gys1	Gys1	glycogen synthase 1, muscle	14936	https://www.ncbi.nlm.nih.gov/gene/?term=14936
Aldoa	Aldoa	aldolase A, fructose-bisphosphate	11674	https://www.ncbi.nlm.nih.gov/gene/?term=11674
Arfip2	Arfip2	ADP-ribosylation factor interacting protein 2	76932	https://www.ncbi.nlm.nih.gov/gene/?term=76932
Rfpl4	Rfpl4	ret finger protein-like 4	192658	https://www.ncbi.nlm.nih.gov/gene/?term=192658
Slc25a34	Slc25a34	solute carrier family 25, member 34	384071	https://www.ncbi.nlm.nih.gov/gene/?term=384071
Txnrd3	Txnrd3	thioredoxin reductase 3	232223	https://www.ncbi.nlm.nih.gov/gene/?term=232223
Pnrc2	Pnrc2	proline-rich nuclear receptor coactivator 2	52830	https://www.ncbi.nlm.nih.gov/gene/?term=52830
Clec4b2	Clec4b2	C-type lectin domain family 4, member b2	381809	https://www.ncbi.nlm.nih.gov/gene/?term=381809
Rnf13	Rnf13	ring finger protein 13	24017	https://www.ncbi.nlm.nih.gov/gene/?term=24017
Spire2	Spire2	spire type actin nucleation factor 2	234857	https://www.ncbi.nlm.nih.gov/gene/?term=234857
Aadat	Aadat	aminoadipate aminotransferase	23923	https://www.ncbi.nlm.nih.gov/gene/?term=23923
Akr1c14	Akr1c14	aldo-keto reductase family 1, member C14	105387	https://www.ncbi.nlm.nih.gov/gene/?term=105387
Sspn	Sspn	sarcospan	16651	https://www.ncbi.nlm.nih.gov/gene/?term=16651
Akr1c6	Akr1c6	aldo-keto reductase family 1, member C6	83702	https://www.ncbi.nlm.nih.gov/gene/?term=83702
Tmem81	Tmem81	transmembrane protein 81	74626	https://www.ncbi.nlm.nih.gov/gene/?term=74626

Tnfsf9	Tnfsf9	tumor necrosis factor (ligand) superfamily, member 9	21950	https://www.ncbi.nlm.nih.gov/gene/?term=21950
8030462N17Rik	8030462N17Rik	RIKEN cDNA 8030462N17 gene	212163	https://www.ncbi.nlm.nih.gov/gene/?term=212163
Ccdc153	Ccdc153	coiled-coil domain containing 153	270150	https://www.ncbi.nlm.nih.gov/gene/?term=270150
Per3	Per3	period circadian clock 3	18628	https://www.ncbi.nlm.nih.gov/gene/?term=18628
Gm13212	Gm13212	predicted gene 13212	433801	https://www.ncbi.nlm.nih.gov/gene/?term=433801
Tom1l1	Tom1l1	target of myb1-like 1 (chicken)	71943	https://www.ncbi.nlm.nih.gov/gene/?term=71943
Kctd5	Kctd5	potassium channel tetramerisation domain containing 5	69259	https://www.ncbi.nlm.nih.gov/gene/?term=69259
1810037I17Rik	1810037I17Rik	RIKEN cDNA 1810037I17 gene	67704	https://www.ncbi.nlm.nih.gov/gene/?term=67704
Stradb	Stradb	STE20-related kinase adaptor beta	227154	https://www.ncbi.nlm.nih.gov/gene/?term=227154
Gba2	Gba2	glucosidase beta 2	230101	https://www.ncbi.nlm.nih.gov/gene/?term=230101
Fat4	Fat4	FAT atypical cadherin 4	329628	https://www.ncbi.nlm.nih.gov/gene/?term=329628
Dcaf7	Dcaf7	DDB1 and CUL4 associated factor 7	71833	https://www.ncbi.nlm.nih.gov/gene/?term=71833
Egfr	Egfr	epidermal growth factor receptor	13649	https://www.ncbi.nlm.nih.gov/gene/?term=13649
Shprh	Shprh	SNF2 histone linker PHD RING helicase	268281	https://www.ncbi.nlm.nih.gov/gene/?term=268281
Snn	Snn	stannin	20621	https://www.ncbi.nlm.nih.gov/gene/?term=20621
Robo4	Robo4	roundabout guidance receptor 4	74144	https://www.ncbi.nlm.nih.gov/gene/?term=74144
Cnst	Cnst	consortin, connexin sorting protein	226744	https://www.ncbi.nlm.nih.gov/gene/?term=226744
Zfp81	Zfp81	zinc finger protein 81	224694	https://www.ncbi.nlm.nih.gov/gene/?term=224694
Rbm15b	Rbm15b	RNA binding motif protein 15B	109095	https://www.ncbi.nlm.nih.gov/gene/?term=109095
Pln	Pln	phospholamban	18821	https://www.ncbi.nlm.nih.gov/gene/?term=18821
Mesp2	Mesp2	mesoderm posterior 2	17293	https://www.ncbi.nlm.nih.gov/gene/?term=17293
Cpn2	Cpn2	carboxypeptidase N, polypeptide 2	71756	https://www.ncbi.nlm.nih.gov/gene/?term=71756
Wdsub1	Wdsub1	WD repeat, SAM and U-box domain containing 1	72137	https://www.ncbi.nlm.nih.gov/gene/?term=72137
Bclaf1	Bclaf1	BCL2-associated transcription factor 1	72567	https://www.ncbi.nlm.nih.gov/gene/?term=72567

Biology Open • Supplementary information

Shq1	Shq1	SHQ1 homolog (S. cerevisiae)	72171	https://www.ncbi.nlm.nih.gov/gene/?term=72171
Fgf6	Fgf6	fibroblast growth factor 6	14177	https://www.ncbi.nlm.nih.gov/gene/?term=14177
Atp10b	Atp10b	ATPase, class V, type 10B	319767	https://www.ncbi.nlm.nih.gov/gene/?term=319767
Ggct	Ggct	gamma-glutamyl cyclotransferase	110175	https://www.ncbi.nlm.nih.gov/gene/?term=110175
Neurl3	Neurl3	neuralized E3 ubiquitin protein ligase 3	214854	https://www.ncbi.nlm.nih.gov/gene/?term=214854
Ankrd12	Ankrd12	ankyrin repeat domain 12	106585	https://www.ncbi.nlm.nih.gov/gene/?term=106585
Ehmt1	Ehmt1	euchromatic histone methyltransferase 1	77683	https://www.ncbi.nlm.nih.gov/gene/?term=77683
Kpna3	Kpna3	karyopherin (importin) alpha 3	16648	https://www.ncbi.nlm.nih.gov/gene/?term=16648
Cplx4	Cplx4	complexin 4	225644	https://www.ncbi.nlm.nih.gov/gene/?term=225644
Gnao1	Gnao1	guanine nucleotide binding protein, alpha O	14681	https://www.ncbi.nlm.nih.gov/gene/?term=14681
Tada2a	Tada2a	transcriptional adaptor 2A	217031	https://www.ncbi.nlm.nih.gov/gene/?term=217031
Enoph1	Enoph1	enolase-phosphatase 1	67870	https://www.ncbi.nlm.nih.gov/gene/?term=67870
Nacc2	Nacc2	nucleus accumbens associated 2, BEN and BTB (POZ) domain containing	67991	https://www.ncbi.nlm.nih.gov/gene/?term=67991
Defb1	Defb1	defensin beta 1	13214	https://www.ncbi.nlm.nih.gov/gene/?term=13214
Atp1b1	Atp1b1	ATPase, Na+/K+ transporting, beta 1 polypeptide	11931	https://www.ncbi.nlm.nih.gov/gene/?term=11931
Wdr72	Wdr72	WD repeat domain 72	546144	https://www.ncbi.nlm.nih.gov/gene/?term=546144
Rab39b	Rab39b	RAB39B, member RAS oncogene family	67790	https://www.ncbi.nlm.nih.gov/gene/?term=67790
Smchd1	Smchd1	SMC hinge domain containing 1	74355	https://www.ncbi.nlm.nih.gov/gene/?term=74355
Vsx2	Vsx2	visual system homeobox 2	12677	https://www.ncbi.nlm.nih.gov/gene/?term=12677
Oasl2	Oasl2	2'-5' oligoadenylate synthetase-like 2	23962	https://www.ncbi.nlm.nih.gov/gene/?term=23962
Cep57l1	Cep57l1	centrosomal protein 57-like 1	103268	https://www.ncbi.nlm.nih.gov/gene/?term=103268

Cyp20a1	Cyp20a1	cytochrome P450, family 20, subfamily a, polypeptide 1	77951	https://www.ncbi.nlm.nih.gov/gene/?term=77951
Ophn1	Ophn1	oligophrenin 1	94190	https://www.ncbi.nlm.nih.gov/gene/?term=94190
Rnf182	Rnf182	ring finger protein 182	328234	https://www.ncbi.nlm.nih.gov/gene/?term=328234
Trdmt1	Trdmt1	tRNA aspartic acid methyltransferase 1	13434	https://www.ncbi.nlm.nih.gov/gene/?term=13434
Slc45a2	Slc45a2	solute carrier family 45, member 2	22293	https://www.ncbi.nlm.nih.gov/gene/?term=22293
Kif27	Kif27	kinesin family member 27	75050	https://www.ncbi.nlm.nih.gov/gene/?term=75050
Ifih1	Ifih1	interferon induced with helicase C domain 1	71586	https://www.ncbi.nlm.nih.gov/gene/?term=71586
Mcur1	Mcur1	mitochondrial calcium uniporter regulator 1	76137	https://www.ncbi.nlm.nih.gov/gene/?term=76137
Prps2	Prps2	phosphoribosyl pyrophosphate synthetase 2	110639	https://www.ncbi.nlm.nih.gov/gene/?term=110639
Pip4p2	Pip4p2	phosphatidylinositol-4,5-bisphosphate 4-phosphatase 2	72519	https://www.ncbi.nlm.nih.gov/gene/?term=72519
Acer3	Acer3	alkaline ceramidase 3	66190	https://www.ncbi.nlm.nih.gov/gene/?term=66190
Sohlh2	Sohlh2	spermatogenesis and oogenesis specific basic helix-loop-helix 2	74434	https://www.ncbi.nlm.nih.gov/gene/?term=74434
Wwp1	Wwp1	WW domain containing E3 ubiquitin protein ligase 1	107568	https://www.ncbi.nlm.nih.gov/gene/?term=107568
Slco2b1	Slco2b1	solute carrier organic anion transporter family, member 2b1	101488	https://www.ncbi.nlm.nih.gov/gene/?term=101488
Fntb	Fntb	farnesyltransferase, CAAX box, beta	110606	https://www.ncbi.nlm.nih.gov/gene/?term=110606
Kcnk6	Kcnk6	potassium inwardly-rectifying channel, subfamily K, member 6	52150	https://www.ncbi.nlm.nih.gov/gene/?term=52150
Bid	Bid	BH3 interacting domain death agonist	12122	https://www.ncbi.nlm.nih.gov/gene/?term=12122
Dagla	Dagla	diacylglycerol lipase, alpha	269060	https://www.ncbi.nlm.nih.gov/gene/?term=269060

Biology Open • Supplementary information

Usb1	Usb1	U6 snRNA biogenesis 1	101985	https://www.ncbi.nlm.nih.gov/gene/?term=101985
Nfic	Nfic	nuclear factor I/C	18029	https://www.ncbi.nlm.nih.gov/gene/?term=18029
Pcdh15	Pcdh15	protocadherin 15	11994	https://www.ncbi.nlm.nih.gov/gene/?term=11994
Rapgef1	Rapgef1	Rap guanine nucleotide exchange factor (GEF) 1	107746	https://www.ncbi.nlm.nih.gov/gene/?term=107746
Kcne4	Kcne4	potassium voltage-gated channel, Isk-related subfamily, gene 4	57814	https://www.ncbi.nlm.nih.gov/gene/?term=57814
Zfp775	Zfp775	zinc finger protein 775	243372	https://www.ncbi.nlm.nih.gov/gene/?term=243372
Fut4	Fut4	fucosyltransferase 4	14345	https://www.ncbi.nlm.nih.gov/gene/?term=14345
Dmpk	Dmpk	dystrophia myotonica-protein kinase	13400	https://www.ncbi.nlm.nih.gov/gene/?term=13400
Fezf2	Fezf2	Fez family zinc finger 2	54713	https://www.ncbi.nlm.nih.gov/gene/?term=54713
Tpcn1	Tpcn1	two pore channel 1	252972	https://www.ncbi.nlm.nih.gov/gene/?term=252972
Mpeg1	Mpeg1	macrophage expressed gene 1	17476	https://www.ncbi.nlm.nih.gov/gene/?term=17476
Llgl1	Llgl1	LLGL1 scribble cell polarity complex component	16897	https://www.ncbi.nlm.nih.gov/gene/?term=16897
Kcnk3	Kcnk3	potassium channel, subfamily K, member 3	16527	https://www.ncbi.nlm.nih.gov/gene/?term=16527
Ucp2	Ucp2	uncoupling protein 2 (mitochondrial, proton carrier)	22228	https://www.ncbi.nlm.nih.gov/gene/?term=22228
Ndst1	Ndst1	N-deacetylase/N-sulfotransferase (heparan glucosaminyl) 1	15531	https://www.ncbi.nlm.nih.gov/gene/?term=15531
Car7	Car7	carbonic anhydrase 7	12354	https://www.ncbi.nlm.nih.gov/gene/?term=12354
Flna	Flna	filamin, alpha	192176	https://www.ncbi.nlm.nih.gov/gene/?term=192176
Epsti1	Epsti1	epithelial stromal interaction 1 (breast)	108670	https://www.ncbi.nlm.nih.gov/gene/?term=108670
Cd247	Cd247	CD247 antigen	12503	https://www.ncbi.nlm.nih.gov/gene/?term=12503
Rfng	Rfng	RFNG O-fucosylpeptide 3-beta-N-acetylglucosaminyltransferase	19719	https://www.ncbi.nlm.nih.gov/gene/?term=19719

Pacs1	Pacs1	phosphofurin acidic cluster sorting protein 1	107975	https://www.ncbi.nlm.nih.gov/gene/?term=107975
Sh3gl1	Sh3gl1	SH3-domain GRB2-like 1	20405	https://www.ncbi.nlm.nih.gov/gene/?term=20405
Sh2b1	Sh2b1	SH2B adaptor protein 1	20399	https://www.ncbi.nlm.nih.gov/gene/?term=20399
Rspry1	Rspry1	ring finger and SPRY domain containing 1	67610	https://www.ncbi.nlm.nih.gov/gene/?term=67610
Prss8	Prss8	protease, serine 8 (prostasin)	76560	https://www.ncbi.nlm.nih.gov/gene/?term=76560
Zfand3	Zfand3	zinc finger, AN1-type domain 3	21769	https://www.ncbi.nlm.nih.gov/gene/?term=21769
Ctdnep1	Ctdnep1	CTD nuclear envelope phosphatase 1	67181	https://www.ncbi.nlm.nih.gov/gene/?term=67181
Tlk1	Tlk1	tousled-like kinase 1	228012	https://www.ncbi.nlm.nih.gov/gene/?term=228012
Gja3	Gja3	gap junction protein, alpha 3	14611	https://www.ncbi.nlm.nih.gov/gene/?term=14611
Faf1	Faf1	Fas-associated factor 1	14084	https://www.ncbi.nlm.nih.gov/gene/?term=14084
Zkscan8	Zkscan8	zinc finger with KRAB and SCAN domains 8	93681	https://www.ncbi.nlm.nih.gov/gene/?term=93681
BC024978	BC024978	cDNA sequence BC024978	414069	https://www.ncbi.nlm.nih.gov/gene/?term=414069
Ifrd2	Ifrd2	interferon-related developmental regulator 2	15983	https://www.ncbi.nlm.nih.gov/gene/?term=15983
Ralbp1	Ralbp1	ralA binding protein 1	19765	https://www.ncbi.nlm.nih.gov/gene/?term=19765
Psmb11	Psmb11	proteasome (prosome, macropain) subunit, beta type, 11	73902	https://www.ncbi.nlm.nih.gov/gene/?term=73902
Sorl1	Sorl1	sortilin-related receptor, LDLR class A repeats-containing	20660	https://www.ncbi.nlm.nih.gov/gene/?term=20660
Cacna2d3	Cacna2d3	calcium channel, voltage-dependent, alpha2/delta subunit 3	12294	https://www.ncbi.nlm.nih.gov/gene/?term=12294
Lurap1l	Lurap1l	leucine rich adaptor protein 1-like	52829	https://www.ncbi.nlm.nih.gov/gene/?term=52829
Ppme1	Ppme1	protein phosphatase methylesterase 1	72590	https://www.ncbi.nlm.nih.gov/gene/?term=72590
Gpr88	Gpr88	G-protein coupled receptor 88	64378	https://www.ncbi.nlm.nih.gov/gene/?term=64378
Nadk2	Nadk2	NAD kinase 2, mitochondrial	68646	https://www.ncbi.nlm.nih.gov/gene/?term=68646

Dnah5	Dnah5	dynein, axonemal, heavy chain 5	110082	https://www.ncbi.nlm.nih.gov/gene/?term=110082
Asap2	Asap2	ArfGAP with SH3 domain, ankyrin repeat and PH domain 2	211914	https://www.ncbi.nlm.nih.gov/gene/?term=211914
Sdhc	Sdhc	succinate dehydrogenase complex, subunit C, integral membrane protein	66052	https://www.ncbi.nlm.nih.gov/gene/?term=66052
S1pr5	S1pr5	sphingosine-1-phosphate receptor 5	94226	https://www.ncbi.nlm.nih.gov/gene/?term=94226
Bin3	Bin3	bridging integrator 3	57784	https://www.ncbi.nlm.nih.gov/gene/?term=57784
Ambn	Ambn	ameloblastin	11698	https://www.ncbi.nlm.nih.gov/gene/?term=11698
Csad	Csad	cysteine sulfinic acid decarboxylase	246277	https://www.ncbi.nlm.nih.gov/gene/?term=246277
Acsm4	Acsm4	acyl-CoA synthetase medium-chain family member 4	233801	https://www.ncbi.nlm.nih.gov/gene/?term=233801
Ifi204	Ifi204	interferon activated gene 204	15951	https://www.ncbi.nlm.nih.gov/gene/?term=15951
Cenpq	Cenpq	centromere protein Q	83815	https://www.ncbi.nlm.nih.gov/gene/?term=83815
Csnk1d	Csnk1d	casein kinase 1, delta	104318	https://www.ncbi.nlm.nih.gov/gene/?term=104318
Sgcd	Sgcd	sarcoglycan, delta (dystrophin-associated glycoprotein)	24052	https://www.ncbi.nlm.nih.gov/gene/?term=24052
Soat1	Soat1	sterol O-acyltransferase 1	20652	https://www.ncbi.nlm.nih.gov/gene/?term=20652
Cdk12	Cdk12	cyclin-dependent kinase 12	69131	https://www.ncbi.nlm.nih.gov/gene/?term=69131
Mapk1ip1l	Mapk1ip1l	mitogen-activated protein kinase 1 interacting protein 1-like	218975	https://www.ncbi.nlm.nih.gov/gene/?term=218975
Sytl4	Sytl4	synaptotagmin-like 4	27359	https://www.ncbi.nlm.nih.gov/gene/?term=27359
Zfp811	Zfp811	zinc finger protein 811	240063	https://www.ncbi.nlm.nih.gov/gene/?term=240063
Nfe2l3	Nfe2l3	nuclear factor, erythroid derived 2, like 3	18025	https://www.ncbi.nlm.nih.gov/gene/?term=18025
2310022A10Rik	2310022A10Rik	RIKEN cDNA 2310022A10 gene	66367	https://www.ncbi.nlm.nih.gov/gene/?term=66367
Slc10a6	Slc10a6	solute carrier family 10 (sodium/bile acid cotransporter family), member 6	75750	https://www.ncbi.nlm.nih.gov/gene/?term=75750

Biology Open • Supplementary information

Zfp185	Zfp185	zinc finger protein 185	22673	https://www.ncbi.nlm.nih.gov/gene/?term=22673
Erc1	Erc1	ELKS/RAB6-interacting/CAST family member 1	111173	https://www.ncbi.nlm.nih.gov/gene/?term=111173
Ogfrl1	Ogfrl1	opioid growth factor receptor-like 1	70155	https://www.ncbi.nlm.nih.gov/gene/?term=70155
Rab1a	Rab1a	RAB1A, member RAS oncogene family	19324	https://www.ncbi.nlm.nih.gov/gene/?term=19324
Zdbf2	Zdbf2	zinc finger, DBF-type containing 2	73884	https://www.ncbi.nlm.nih.gov/gene/?term=73884
Timm29	Timm29	translocase of inner mitochondrial membrane 29	69773	https://www.ncbi.nlm.nih.gov/gene/?term=69773
Manea	Manea	mannosidase, endo-alpha	242362	https://www.ncbi.nlm.nih.gov/gene/?term=242362
Htr2b	Htr2b	5-hydroxytryptamine (serotonin) receptor 2B	15559	https://www.ncbi.nlm.nih.gov/gene/?term=15559
Lsm8	Lsm8	LSM8 homolog, U6 small nuclear RNA associated	76522	https://www.ncbi.nlm.nih.gov/gene/?term=76522
Sdhaf4	Sdhaf4	succinate dehydrogenase complex assembly factor 4	68002	https://www.ncbi.nlm.nih.gov/gene/?term=68002
Rabgap1	Rabgap1	RAB GTPase activating protein 1	227800	https://www.ncbi.nlm.nih.gov/gene/?term=227800
Mms22l	Mms22l	MMS22-like, DNA repair protein	212377	https://www.ncbi.nlm.nih.gov/gene/?term=212377
4930568D16Rik	4930568D16Rik	RIKEN cDNA 4930568D16 gene	75859	https://www.ncbi.nlm.nih.gov/gene/?term=75859
Irs4	Irs4	insulin receptor substrate 4	16370	https://www.ncbi.nlm.nih.gov/gene/?term=16370
Efemp1	Efemp1	epidermal growth factor-containing fibulin-like extracellular matrix protein 1	216616	https://www.ncbi.nlm.nih.gov/gene/?term=216616
Xrcc4	Xrcc4	X-ray repair complementing defective repair in Chinese hamster cells 4	108138	https://www.ncbi.nlm.nih.gov/gene/?term=108138
Bcl9	Bcl9	B cell CLL/lymphoma 9	77578	https://www.ncbi.nlm.nih.gov/gene/?term=77578
Slf2	Slf2	SMC5-SMC6 complex localization factor 2	226151	https://www.ncbi.nlm.nih.gov/gene/?term=226151

Biology Open • Supplementary information

Klc1	Klc1	kinesin light chain 1	16593	https://www.ncbi.nlm.nih.gov/gene/?term=16593
Mgam	Mgam	maltase-glucoamylase	232714	https://www.ncbi.nlm.nih.gov/gene/?term=232714
Dgkq	Dgkq	diacylglycerol kinase, theta	110524	https://www.ncbi.nlm.nih.gov/gene/?term=110524
Zfp808	Zfp808	zinc finger protein 808	630579	https://www.ncbi.nlm.nih.gov/gene/?term=630579
Bcl6	Bcl6	B cell leukemia/lymphoma 6	12053	https://www.ncbi.nlm.nih.gov/gene/?term=12053
Tgs1	Tgs1	trimethylguanosine synthase 1	116940	https://www.ncbi.nlm.nih.gov/gene/?term=116940
Fryl	Fryl	FRY like transcription coactivator	72313	https://www.ncbi.nlm.nih.gov/gene/?term=72313
Gli3	Gli3	GLI-Kruppel family member GLI3	14634	https://www.ncbi.nlm.nih.gov/gene/?term=14634
Dsc3	Dsc3	desmocollin 3	13507	https://www.ncbi.nlm.nih.gov/gene/?term=13507
Plec	Plec	plectin	18810	https://www.ncbi.nlm.nih.gov/gene/?term=18810
Ccdc130	Ccdc130	coiled-coil domain containing 130	67736	https://www.ncbi.nlm.nih.gov/gene/?term=67736
Map6d1	Map6d1	MAP6 domain containing 1	208158	https://www.ncbi.nlm.nih.gov/gene/?term=208158
Abt1	Abt1	activator of basal transcription 1	30946	https://www.ncbi.nlm.nih.gov/gene/?term=30946
Tmem248	Tmem248	transmembrane protein 248	71667	https://www.ncbi.nlm.nih.gov/gene/?term=71667
Ptprf	Ptprf	protein tyrosine phosphatase, receptor type, F	19268	https://www.ncbi.nlm.nih.gov/gene/?term=19268
Hdlbp	Hdlbp	high density lipoprotein (HDL) binding protein	110611	https://www.ncbi.nlm.nih.gov/gene/?term=110611
Lingo4	Lingo4	leucine rich repeat and Ig domain containing 4	320747	https://www.ncbi.nlm.nih.gov/gene/?term=320747
Otop2	Otop2	otopetrin 2	237987	https://www.ncbi.nlm.nih.gov/gene/?term=237987
Mrps21	Mrps21	mitochondrial ribosomal protein S21	66292	https://www.ncbi.nlm.nih.gov/gene/?term=66292
Pate3	Pate3	prostate and testis expressed 3	100312956	https://www.ncbi.nlm.nih.gov/gene/?term=100312
Dmbx1	Dmbx1	diencephalon/mesencephalon homeobox 1	140477	https://www.ncbi.nlm.nih.gov/gene/?term=140477
Serpina6	Serpina6	serine (or cysteine) peptidase inhibitor, clade A, member 6	12401	https://www.ncbi.nlm.nih.gov/gene/?term=12401
Prr29	Prr29	proline rich 29	75573	https://www.ncbi.nlm.nih.gov/gene/?term=75573

Bsg	Bsg	basigin	12215	https://www.ncbi.nlm.nih.gov/gene/?term=12215
Pink1	Pink1	PTEN induced putative kinase 1	68943	https://www.ncbi.nlm.nih.gov/gene/?term=68943
Zhx3	Zhx3	zinc fingers and homeoboxes 3	320799	https://www.ncbi.nlm.nih.gov/gene/?term=320799
Mrpl41	Mrpl41	mitochondrial ribosomal protein L41	107733	https://www.ncbi.nlm.nih.gov/gene/?term=107733
Cript	Cript	cysteine-rich PDZ-binding protein	56724	https://www.ncbi.nlm.nih.gov/gene/?term=56724
Dbt	Dbt	dihydrolipoamide branched chain transacylase E2	13171	https://www.ncbi.nlm.nih.gov/gene/?term=13171
Plekhb2	Plekhb2	pleckstrin homology domain containing, family B (evectins) member 2	226971	https://www.ncbi.nlm.nih.gov/gene/?term=226971
Ercc4	Ercc4	excision repair cross-complementing rodent repair deficiency, complementation group 4	50505	https://www.ncbi.nlm.nih.gov/gene/?term=50505
Jhy	Jhy	junctional cadherin complex regulator	70989	https://www.ncbi.nlm.nih.gov/gene/?term=70989
Trmt10a	Trmt10a	tRNA methyltransferase 10A	108943	https://www.ncbi.nlm.nih.gov/gene/?term=108943
Itgal	Itgal	integrin alpha L	16408	https://www.ncbi.nlm.nih.gov/gene/?term=16408
Lysmd2	Lysmd2	LysM, putative peptidoglycan-binding, domain containing 2	70082	https://www.ncbi.nlm.nih.gov/gene/?term=70082
Snai3	Snai3	snail family zinc finger 3	30927	https://www.ncbi.nlm.nih.gov/gene/?term=30927
Zfp462	Zfp462	zinc finger protein 462	242466	https://www.ncbi.nlm.nih.gov/gene/?term=242466
Lrrc28	Lrrc28	leucine rich repeat containing 28	67867	https://www.ncbi.nlm.nih.gov/gene/?term=67867
Amy2a2	Amy2a2	amylase 2a2	100043688	https://www.ncbi.nlm.nih.gov/gene/?term=100043
Stim1	Stim1	stromal interaction molecule 1	20866	https://www.ncbi.nlm.nih.gov/gene/?term=20866
Arl6ip5	Arl6ip5	ADP-ribosylation factor-like 6 interacting protein 5	65106	https://www.ncbi.nlm.nih.gov/gene/?term=65106
Trappc9	Trappc9	trafficking protein particle complex 9	76510	https://www.ncbi.nlm.nih.gov/gene/?term=76510
Lsm14b	Lsm14b	LSM family member 14B	241846	https://www.ncbi.nlm.nih.gov/gene/?term=241846

Dars2	Dars2	aspartyl-tRNA synthetase 2 (mitochondrial)	226539	https://www.ncbi.nlm.nih.gov/gene/?term=226539
Amy2a3	Amy2a3	amylase 2a3	100043686	https://www.ncbi.nlm.nih.gov/gene/?term=100043
Sdc1	Sdc1	syndecan 1	20969	https://www.ncbi.nlm.nih.gov/gene/?term=20969
Fam107b	Fam107b	family with sequence similarity 107, member B	66540	https://www.ncbi.nlm.nih.gov/gene/?term=66540
Zfp454	Zfp454	zinc finger protein 454	237758	https://www.ncbi.nlm.nih.gov/gene/?term=237758
Clint1	Clint1	clathrin interactor 1	216705	https://www.ncbi.nlm.nih.gov/gene/?term=216705
Amy2a4	Amy2a4	amylase 2a4	100043684	https://www.ncbi.nlm.nih.gov/gene/?term=100043
Anapc10	Anapc10	anaphase promoting complex subunit 10	68999	https://www.ncbi.nlm.nih.gov/gene/?term=68999
Ereg	Ereg	epiregulin	13874	https://www.ncbi.nlm.nih.gov/gene/?term=13874
Bhlhe22	Bhlhe22	basic helix-loop-helix family, member e22	59058	https://www.ncbi.nlm.nih.gov/gene/?term=59058
Ppwd1	Ppwd1	peptidylprolyl isomerase domain and WD repeat containing 1	238831	https://www.ncbi.nlm.nih.gov/gene/?term=238831
Slc24a5	Slc24a5	solute carrier family 24, member 5	317750	https://www.ncbi.nlm.nih.gov/gene/?term=317750

Biology Open • Supplementary information

Table S11. Genes predicted to be modulated by miRNAs differentially expressed between P18 and Lac.

userId	geneSymbol	geneName	entrezgene	gLink
Pdik1l	Pdik1l	PDLIM1 interacting kinase 1 like	230809	https://www.ncbi.nlm.nih.gov/gene/?term=230809
Smim4	Smim4	small integral membrane protein 4	66487	https://www.ncbi.nlm.nih.gov/gene/?term=66487
Strap	Strap	serine/threonine kinase receptor associated protein	20901	https://www.ncbi.nlm.nih.gov/gene/?term=20901
Mospd2	Mospd2	motile sperm domain containing 2	76763	https://www.ncbi.nlm.nih.gov/gene/?term=76763
Rdh10	Rdh10	retinol dehydrogenase 10 (all-trans)	98711	https://www.ncbi.nlm.nih.gov/gene/?term=98711
Zfand6	Zfand6	zinc finger, AN1-type domain 6	65098	https://www.ncbi.nlm.nih.gov/gene/?term=65098
Rusc2	Rusc2	RUN and SH3 domain containing 2	100213	https://www.ncbi.nlm.nih.gov/gene/?term=100213
Nbr1	Nbr1	NBR1, autophagy cargo receptor	17966	https://www.ncbi.nlm.nih.gov/gene/?term=17966
Dek	Dek	DEK oncogene (DNA binding)	110052	https://www.ncbi.nlm.nih.gov/gene/?term=110052
Fli1	Fli1	Friend leukemia integration 1	14247	https://www.ncbi.nlm.nih.gov/gene/?term=14247
Cdk17	Cdk17	cyclin-dependent kinase 17	237459	https://www.ncbi.nlm.nih.gov/gene/?term=237459
Slc30a4	Slc30a4	solute carrier family 30 (zinc transporter), member 4	22785	https://www.ncbi.nlm.nih.gov/gene/?term=22785
Nr5a2	Nr5a2	nuclear receptor subfamily 5, group A, member 2	26424	https://www.ncbi.nlm.nih.gov/gene/?term=26424
Eloc	Eloc	elongin C	67923	https://www.ncbi.nlm.nih.gov/gene/?term=67923
Rims2	Rims2	regulating synaptic membrane exocytosis 2	116838	https://www.ncbi.nlm.nih.gov/gene/?term=116838
Xkr4	Xkr4	X-linked Kx blood group related 4	497097	https://www.ncbi.nlm.nih.gov/gene/?term=497097
Wapl	Wapl	WAPL cohesin release factor	218914	https://www.ncbi.nlm.nih.gov/gene/?term=218914
Mosmo	Mosmo	modulator of smoothened	233812	https://www.ncbi.nlm.nih.gov/gene/?term=233812
Cntn1	Cntn1	contactin 1	12805	https://www.ncbi.nlm.nih.gov/gene/?term=12805
Pfpl	Pfpl	pore forming protein-like	56093	https://www.ncbi.nlm.nih.gov/gene/?term=56093
Lhfp	Lhfp	lipoma HMGIC fusion partner	108927	https://www.ncbi.nlm.nih.gov/gene/?term=108927

Biology Open • Supplementary information

Slit2	Slit2	slit guidance ligand 2	20563	https://www.ncbi.nlm.nih.gov/gene/?term=20563
Rab11fip2	Rab11fip2	RAB11 family interacting protein 2 (class I)	74998	https://www.ncbi.nlm.nih.gov/gene/?term=74998
Pom121l12	Pom121l12	POM121 membrane glycoprotein-like 12	432536	https://www.ncbi.nlm.nih.gov/gene/?term=432536
Pcmtd1	Pcmtd1	protein-L-isoaspartate (D-aspartate) O-methyltransferase domain containing 1	319263	https://www.ncbi.nlm.nih.gov/gene/?term=319263
Dlc1	Dlc1	deleted in liver cancer 1	50768	https://www.ncbi.nlm.nih.gov/gene/?term=50768
Pds5b	Pds5b	PDS5 cohesin associated factor B	100710	https://www.ncbi.nlm.nih.gov/gene/?term=100710
Arhgap20	Arhgap20	Rho GTPase activating protein 20	244867	https://www.ncbi.nlm.nih.gov/gene/?term=244867
Hmbox1	Hmbox1	homeobox containing 1	219150	https://www.ncbi.nlm.nih.gov/gene/?term=219150
Thap1	Thap1	THAP domain containing, apoptosis associated protein 1	73754	https://www.ncbi.nlm.nih.gov/gene/?term=73754
Qk	Qk	quaking	19317	https://www.ncbi.nlm.nih.gov/gene/?term=19317
Atp11c	Atp11c	ATPase, class VI, type 11C	320940	https://www.ncbi.nlm.nih.gov/gene/?term=320940
Zfp131	Zfp131	zinc finger protein 131	72465	https://www.ncbi.nlm.nih.gov/gene/?term=72465
Fez2	Fez2	fasciculation and elongation protein zeta 2 (zygin II)	225020	https://www.ncbi.nlm.nih.gov/gene/?term=225020
Sulf1	Sulf1	sulfatase 1	240725	https://www.ncbi.nlm.nih.gov/gene/?term=240725
Cnot6	Cnot6	CCR4-NOT transcription complex, subunit 6	104625	https://www.ncbi.nlm.nih.gov/gene/?term=104625
Golga1	Golga1	golgi autoantigen, golgin subfamily a, 1	76899	https://www.ncbi.nlm.nih.gov/gene/?term=76899
Elmod2	Elmod2	ELMO/CED-12 domain containing 2	244548	https://www.ncbi.nlm.nih.gov/gene/?term=244548
Rap1b	Rap1b	RAS related protein 1b	215449	https://www.ncbi.nlm.nih.gov/gene/?term=215449
Rab21	Rab21	RAB21, member RAS oncogene family	216344	https://www.ncbi.nlm.nih.gov/gene/?term=216344

Biology Open • Supplementary information

Phf11d	Phf11d	PHD finger protein 11D	219132	https://www.ncbi.nlm.nih.gov/gene/?term=219132
Khdrbs1	Khdrbs1	KH domain containing, RNA binding, signal transduction associated 1	20218	https://www.ncbi.nlm.nih.gov/gene/?term=20218
Tsc22d2	Tsc22d2	TSC22 domain family, member 2	72033	https://www.ncbi.nlm.nih.gov/gene/?term=72033
Chrdl1	Chrdl1	chordin-like 1	83453	https://www.ncbi.nlm.nih.gov/gene/?term=83453
Zfp711	Zfp711	zinc finger protein 711	245595	https://www.ncbi.nlm.nih.gov/gene/?term=245595
Rnd3	Rnd3	Rho family GTPase 3	74194	https://www.ncbi.nlm.nih.gov/gene/?term=74194
Slitrk1	Slitrk1	SLIT and NTRK-like family, member 1	76965	https://www.ncbi.nlm.nih.gov/gene/?term=76965
Immp2l	Immp2l	IMP2 inner mitochondrial membrane peptidase-like (S. cerevisiae)	93757	https://www.ncbi.nlm.nih.gov/gene/?term=93757
Fbxo33	Fbxo33	F-box protein 33	70611	https://www.ncbi.nlm.nih.gov/gene/?term=70611
Tfap2a	Tfap2a	transcription factor AP-2, alpha	21418	https://www.ncbi.nlm.nih.gov/gene/?term=21418
Ap1s2	Ap1s2	adaptor-related protein complex 1, sigma 2 subunit	108012	https://www.ncbi.nlm.nih.gov/gene/?term=108012
Spryd7	Spryd7	SPRY domain containing 7	66674	https://www.ncbi.nlm.nih.gov/gene/?term=66674
Tjp1	Tjp1	tight junction protein 1	21872	https://www.ncbi.nlm.nih.gov/gene/?term=21872
Ppp1r9b	Ppp1r9b	protein phosphatase 1, regulatory subunit 9B	217124	https://www.ncbi.nlm.nih.gov/gene/?term=217124
Gpr161	Gpr161	G protein-coupled receptor 161	240888	https://www.ncbi.nlm.nih.gov/gene/?term=240888
Fndc3b	Fndc3b	fibronectin type III domain containing 3B	72007	https://www.ncbi.nlm.nih.gov/gene/?term=72007
Hdhd2	Hdhd2	haloacid dehalogenase-like hydrolase domain containing 2	76987	https://www.ncbi.nlm.nih.gov/gene/?term=76987
Cfl2	Cfl2	cofilin 2, muscle	12632	https://www.ncbi.nlm.nih.gov/gene/?term=12632

Biology Open • Supplementary information

Ywhag	Ywhag	tyrosine 3-monooxygenase/tryptophan 5-monooxygenase activation protein, gamma polypeptide	22628	https://www.ncbi.nlm.nih.gov/gene/?term=22628
Desi2	Desi2	desumoylating isopeptidase 2	78825	https://www.ncbi.nlm.nih.gov/gene/?term=78825
Dennd5a	Dennd5a	DENN/MADD domain containing 5A	19347	https://www.ncbi.nlm.nih.gov/gene/?term=19347
Gpm6a	Gpm6a	glycoprotein m6a	234267	https://www.ncbi.nlm.nih.gov/gene/?term=234267
Adamts6	Adamts6	a disintegrin-like and metallopeptidase (reprolysin type) with thrombospondin type 1 motif, 6	108154	https://www.ncbi.nlm.nih.gov/gene/?term=108154
Atxn1	Atxn1	ataxin 1	20238	https://www.ncbi.nlm.nih.gov/gene/?term=20238
Osbpl11	Osbpl11	oxysterol binding protein-like 11	106326	https://www.ncbi.nlm.nih.gov/gene/?term=106326
Sec23a	Sec23a	SEC23 homolog A, COPII coat complex component	20334	https://www.ncbi.nlm.nih.gov/gene/?term=20334
Ptpn21	Ptpn21	protein tyrosine phosphatase, non-receptor type 21	24000	https://www.ncbi.nlm.nih.gov/gene/?term=24000
Sema6d	Sema6d	sema domain, transmembrane domain (TM), and cytoplasmic domain, (semaphorin) 6D	214968	https://www.ncbi.nlm.nih.gov/gene/?term=214968
Hipk3	Hipk3	homeodomain interacting protein kinase 3	15259	https://www.ncbi.nlm.nih.gov/gene/?term=15259
Fignl2	Fignl2	fidgetin-like 2	668225	https://www.ncbi.nlm.nih.gov/gene/?term=668225
Pi4kb	Pi4kb	phosphatidylinositol 4-kinase beta	107650	https://www.ncbi.nlm.nih.gov/gene/?term=107650
Pik3cb	Pik3cb	phosphatidylinositol-4,5-bisphosphate 3-kinase catalytic subunit beta	74769	https://www.ncbi.nlm.nih.gov/gene/?term=74769

Biology Open • Supplementary information

Lrp1b	Lrp1b	low density lipoprotein-related protein 1B (deleted in tumors)	94217	https://www.ncbi.nlm.nih.gov/gene/?term=94217
Fbxw7	Fbxw7	F-box and WD-40 domain protein 7	50754	https://www.ncbi.nlm.nih.gov/gene/?term=50754
Gpatch8	Gpatch8	G patch domain containing 8	237943	https://www.ncbi.nlm.nih.gov/gene/?term=237943
Nr3c1	Nr3c1	nuclear receptor subfamily 3, group C, member 1	14815	https://www.ncbi.nlm.nih.gov/gene/?term=14815
Erg	Erg	ETS transcription factor	13876	https://www.ncbi.nlm.nih.gov/gene/?term=13876
Vash2	Vash2	vasohibin 2	226841	https://www.ncbi.nlm.nih.gov/gene/?term=226841
Synj1	Synj1	synaptojanin 1	104015	https://www.ncbi.nlm.nih.gov/gene/?term=104015
Evi5	Evi5	ecotropic viral integration site 5	14020	https://www.ncbi.nlm.nih.gov/gene/?term=14020
Elmod1	Elmod1	ELMO/CED-12 domain containing 1	270162	https://www.ncbi.nlm.nih.gov/gene/?term=270162
Syncrip	Syncrip	synaptotagmin binding, cytoplasmic RNA interacting protein	56403	https://www.ncbi.nlm.nih.gov/gene/?term=56403
Tubb5	Tubb5	tubulin, beta 5 class I	22154	https://www.ncbi.nlm.nih.gov/gene/?term=22154
Ppm1f	Ppm1f	protein phosphatase 1F (PP2C domain containing)	68606	https://www.ncbi.nlm.nih.gov/gene/?term=68606
Dpy19l1	Dpy19l1	dpy-19-like 1 (C. elegans)	244745	https://www.ncbi.nlm.nih.gov/gene/?term=244745
Mprip	Mprip	myosin phosphatase Rho interacting protein	26936	https://www.ncbi.nlm.nih.gov/gene/?term=26936
Ankrd45	Ankrd45	ankyrin repeat domain 45	73844	https://www.ncbi.nlm.nih.gov/gene/?term=73844
Plcl1	Plcl1	phospholipase C-like 1	227120	https://www.ncbi.nlm.nih.gov/gene/?term=227120
Dennd5b	Dennd5b	DENN/MADD domain containing 5B	320560	https://www.ncbi.nlm.nih.gov/gene/?term=320560
Rps6kb1	Rps6kb1	ribosomal protein S6 kinase, polypeptide 1	72508	https://www.ncbi.nlm.nih.gov/gene/?term=72508
Bag5	Bag5	BCL2-associated athanogene 5	70369	https://www.ncbi.nlm.nih.gov/gene/?term=70369
Med13	Med13	mediator complex subunit 13	327987	https://www.ncbi.nlm.nih.gov/gene/?term=327987
Rbsn	Rbsn	rabenosyn, RAB effector	78287	https://www.ncbi.nlm.nih.gov/gene/?term=78287

Biology Open • Supplementary information

Prr11	Prr11	proline rich 11	270906	https://www.ncbi.nlm.nih.gov/gene/?term=270906
Foxp1	Foxp1	forkhead box P1	108655	https://www.ncbi.nlm.nih.gov/gene/?term=108655
Sox5	Sox5	SRY (sex determining region Y)-box 5	20678	https://www.ncbi.nlm.nih.gov/gene/?term=20678
Wipf1	Wipf1	WAS/WASL interacting protein family, member 1	215280	https://www.ncbi.nlm.nih.gov/gene/?term=215280
Vash1	Vash1	vasohibin 1	238328	https://www.ncbi.nlm.nih.gov/gene/?term=238328
Nrf1	Nrf1	nuclear respiratory factor 1	18181	https://www.ncbi.nlm.nih.gov/gene/?term=18181
Cdh20	Cdh20	cadherin 20	23836	https://www.ncbi.nlm.nih.gov/gene/?term=23836
Ago2	Ago2	argonaute RISC catalytic subunit 2	239528	https://www.ncbi.nlm.nih.gov/gene/?term=239528
St6galnac5	St6galnac5	ST6 (alpha-N-acetyl-neuraminyl-2,3-beta-galactosyl-1,3)-N-acetylgalactosaminide alpha-2,6-sialyltransferase 5	26938	https://www.ncbi.nlm.nih.gov/gene/?term=26938
Jun	Jun	jun proto-oncogene	16476	https://www.ncbi.nlm.nih.gov/gene/?term=16476
Ypel2	Ypel2	yippee like 2	77864	https://www.ncbi.nlm.nih.gov/gene/?term=77864
Nfia	Nfia	nuclear factor I/A	18027	https://www.ncbi.nlm.nih.gov/gene/?term=18027
Zeb2	Zeb2	zinc finger E-box binding homeobox 2	24136	https://www.ncbi.nlm.nih.gov/gene/?term=24136
Rasa2	Rasa2	RAS p21 protein activator 2	114713	https://www.ncbi.nlm.nih.gov/gene/?term=114713
Cd302	Cd302	CD302 antigen	66205	https://www.ncbi.nlm.nih.gov/gene/?term=66205
Phf21b	Phf21b	PHD finger protein 21B	271305	https://www.ncbi.nlm.nih.gov/gene/?term=271305
Lats2	Lats2	large tumor suppressor 2	50523	https://www.ncbi.nlm.nih.gov/gene/?term=50523
Slc14a1	Slc14a1	solute carrier family 14 (urea transporter), member 1	108052	https://www.ncbi.nlm.nih.gov/gene/?term=108052
Esrrg	Esrrg	estrogen-related receptor gamma	26381	https://www.ncbi.nlm.nih.gov/gene/?term=26381
Map2	Map2	microtubule-associated protein 2	17756	https://www.ncbi.nlm.nih.gov/gene/?term=17756
Ric1	Ric1	RAB6A GEF complex partner 1	226089	https://www.ncbi.nlm.nih.gov/gene/?term=226089

Biology Open • Supplementary information

Tbc1d12	Tbc1d12	TBC1D12: TBC1 domain family, member 12	209478	https://www.ncbi.nlm.nih.gov/gene/?term=209478
Egln1	Egln1	egl-9 family hypoxia-inducible factor 1	112405	https://www.ncbi.nlm.nih.gov/gene/?term=112405
Crtap	Crtap	cartilage associated protein	56693	https://www.ncbi.nlm.nih.gov/gene/?term=56693
Ddx3y	Ddx3y	DEAD (Asp-Glu-Ala-Asp) box polypeptide 3, Y-linked	26900	https://www.ncbi.nlm.nih.gov/gene/?term=26900
Uba6	Uba6	ubiquitin-like modifier activating enzyme 6	231380	https://www.ncbi.nlm.nih.gov/gene/?term=231380
Nova1	Nova1	neuro-oncological ventral antigen 1	664883	https://www.ncbi.nlm.nih.gov/gene/?term=664883
Fhl1	Fhl1	four and a half LIM domains 1	14199	https://www.ncbi.nlm.nih.gov/gene/?term=14199
Crkl	Crkl	v-crk avian sarcoma virus CT10 oncogene homolog-like	12929	https://www.ncbi.nlm.nih.gov/gene/?term=12929
Ptpn12	Ptpn12	protein tyrosine phosphatase, non-receptor type 12	19248	https://www.ncbi.nlm.nih.gov/gene/?term=19248
Asap1	Asap1	ArfGAP with SH3 domain, ankyrin repeat and PH domain1	13196	https://www.ncbi.nlm.nih.gov/gene/?term=13196
Tbk1	Tbk1	TANK-binding kinase 1	56480	https://www.ncbi.nlm.nih.gov/gene/?term=56480
Hook3	Hook3	hook microtubule tethering protein 3	320191	https://www.ncbi.nlm.nih.gov/gene/?term=320191
Tob1	Tob1	transducer of ErbB-2.1	22057	https://www.ncbi.nlm.nih.gov/gene/?term=22057
Slc38a2	Slc38a2	solute carrier family 38, member 2	67760	https://www.ncbi.nlm.nih.gov/gene/?term=67760
Amfr	Amfr	autocrine motility factor receptor	23802	https://www.ncbi.nlm.nih.gov/gene/?term=23802
Golga7	Golga7	golgi autoantigen, golgin subfamily a, 7	57437	https://www.ncbi.nlm.nih.gov/gene/?term=57437
Aff3	Aff3	AF4/FMR2 family, member 3	16764	https://www.ncbi.nlm.nih.gov/gene/?term=16764
Slc35a2	Slc35a2	solute carrier family 35 (UDP-galactose transporter), member A2	22232	https://www.ncbi.nlm.nih.gov/gene/?term=22232

Biology Open • Supplementary information

Trim33	Trim33	tripartite motif-containing 33	94093	https://www.ncbi.nlm.nih.gov/gene/?term=94093
Errfi1	Errfi1	ERBB receptor feedback inhibitor 1	74155	https://www.ncbi.nlm.nih.gov/gene/?term=74155
Msn	Msn	moesin	17698	https://www.ncbi.nlm.nih.gov/gene/?term=17698
Zeb1	Zeb1	zinc finger E-box binding homeobox 1	21417	https://www.ncbi.nlm.nih.gov/gene/?term=21417
Jazf1	Jazf1	JAZF zinc finger 1	231986	https://www.ncbi.nlm.nih.gov/gene/?term=231986
Mmd	Mmd	monocyte to macrophage differentiation-associated	67468	https://www.ncbi.nlm.nih.gov/gene/?term=67468
Rap2c	Rap2c	RAP2C, member of RAS oncogene family	72065	https://www.ncbi.nlm.nih.gov/gene/?term=72065
Rnf169	Rnf169	ring finger protein 169	108937	https://www.ncbi.nlm.nih.gov/gene/?term=108937
Phf11b	Phf11b	PHD finger protein 11B	236451	https://www.ncbi.nlm.nih.gov/gene/?term=236451
Tent4b	Tent4b	terminal nucleotidyltransferase 4B	214627	https://www.ncbi.nlm.nih.gov/gene/?term=214627
Spag9	Spag9	sperm associated antigen 9	70834	https://www.ncbi.nlm.nih.gov/gene/?term=70834
Unc5d	Unc5d	unc-5 netrin receptor D	210801	https://www.ncbi.nlm.nih.gov/gene/?term=210801
2700081O15Rik	2700081O15Rik	RIKEN cDNA 2700081O15 gene	108899	https://www.ncbi.nlm.nih.gov/gene/?term=108899
Agfg1	Agfg1	ArfGAP with FG repeats 1	15463	https://www.ncbi.nlm.nih.gov/gene/?term=15463
Ccnyl1	Ccnyl1	cyclin Y-like 1	227210	https://www.ncbi.nlm.nih.gov/gene/?term=227210
Bap1	Bap1	Brca1 associated protein 1	104416	https://www.ncbi.nlm.nih.gov/gene/?term=104416
Eps8	Eps8	epidermal growth factor receptor pathway substrate 8	13860	https://www.ncbi.nlm.nih.gov/gene/?term=13860
Igsf10	Igsf10	immunoglobulin superfamily, member 10	242050	https://www.ncbi.nlm.nih.gov/gene/?term=242050
Rbfox2	Rbfox2	RNA binding protein, fox-1 homolog (C. elegans) 2	93686	https://www.ncbi.nlm.nih.gov/gene/?term=93686
Scd1	Scd1	stearoyl-Coenzyme A desaturase 1	20249	https://www.ncbi.nlm.nih.gov/gene/?term=20249
Pkd1	Pkd1	polycystin 1, transient receptor poteintial channel interacting	18763	https://www.ncbi.nlm.nih.gov/gene/?term=18763

Mboat2	Mboat2	membrane bound O-acyltransferase domain containing 2	67216	https://www.ncbi.nlm.nih.gov/gene/?term=67216
Ano5	Ano5	anoctamin 5	233246	https://www.ncbi.nlm.nih.gov/gene/?term=233246
Ranbp9	Ranbp9	RAN binding protein 9	56705	https://www.ncbi.nlm.nih.gov/gene/?term=56705
Arih1	Arih1	ariadne RBR E3 ubiquitin protein ligase 1	23806	https://www.ncbi.nlm.nih.gov/gene/?term=23806
Clasp1	Clasp1	CLIP associating protein 1	76707	https://www.ncbi.nlm.nih.gov/gene/?term=76707
Cecr2	Cecr2	CECR2, histone acetyl-lysine reader	330409	https://www.ncbi.nlm.nih.gov/gene/?term=330409
Mindy2	Mindy2	MINDY lysine 48 deubiquitinase 2	235461	https://www.ncbi.nlm.nih.gov/gene/?term=235461
Mgat2	Mgat2	mannoside acetylglucosaminyltransferase 2	217664	https://www.ncbi.nlm.nih.gov/gene/?term=217664
1810013L24Rik	1810013L24Rik	RIKEN cDNA 1810013L24 gene	69053	https://www.ncbi.nlm.nih.gov/gene/?term=69053
Hnrnpf	Hnrnpf	heterogeneous nuclear ribonucleoprotein F	98758	https://www.ncbi.nlm.nih.gov/gene/?term=98758
Lpar1	Lpar1	lysophosphatidic acid receptor 1	14745	https://www.ncbi.nlm.nih.gov/gene/?term=14745
Oosp3	Oosp3	oocyte secreted protein 3	225923	https://www.ncbi.nlm.nih.gov/gene/?term=225923
Gucy1a1	Gucy1a1	guanylate cyclase 1, soluble, alpha 1	60596	https://www.ncbi.nlm.nih.gov/gene/?term=60596
Mybl1	Mybl1	myeloblastosis oncogene-like 1	17864	https://www.ncbi.nlm.nih.gov/gene/?term=17864
Ntng1	Ntng1	netrin G1	80883	https://www.ncbi.nlm.nih.gov/gene/?term=80883
Dusp3	Dusp3	dual specificity phosphatase 3 (vaccinia virus phosphatase VH1-related)	72349	https://www.ncbi.nlm.nih.gov/gene/?term=72349
Mbnl1	Mbnl1	muscleblind like splicing factor 1	56758	https://www.ncbi.nlm.nih.gov/gene/?term=56758
Arfgef3	Arfgef3	ARFGEF family member 3	215821	https://www.ncbi.nlm.nih.gov/gene/?term=215821
Rnf145	Rnf145	ring finger protein 145	74315	https://www.ncbi.nlm.nih.gov/gene/?term=74315
Myt1l	Myt1l	myelin transcription factor 1-like	17933	https://www.ncbi.nlm.nih.gov/gene/?term=17933

Biology Open • Supplementary information

Kif3a	Kif3a	kinesin family member 3A	16568	https://www.ncbi.nlm.nih.gov/gene/?term=16568
Dr1	Dr1	down-regulator of transcription 1	13486	https://www.ncbi.nlm.nih.gov/gene/?term=13486
Cyth3	Cyth3	cytohesin 3	19159	https://www.ncbi.nlm.nih.gov/gene/?term=19159
Pcdh9	Pcdh9	protocadherin 9	211712	https://www.ncbi.nlm.nih.gov/gene/?term=211712
Dmwd	Dmwd	dystrophia myotonica-containing WD repeat motif	13401	https://www.ncbi.nlm.nih.gov/gene/?term=13401
Fbxl2	Fbxl2	F-box and leucine-rich repeat protein 2	72179	https://www.ncbi.nlm.nih.gov/gene/?term=72179
Ulk2	Ulk2	unc-51 like kinase 2	29869	https://www.ncbi.nlm.nih.gov/gene/?term=29869
Cyp26b1	Cyp26b1	cytochrome P450, family 26, subfamily b, polypeptide 1	232174	https://www.ncbi.nlm.nih.gov/gene/?term=232174
Ccne2	Ccne2	cyclin E2	12448	https://www.ncbi.nlm.nih.gov/gene/?term=12448
Myh10	Myh10	myosin, heavy polypeptide 10, non-muscle	77579	https://www.ncbi.nlm.nih.gov/gene/?term=77579
Gpc2	Gpc2	glypican 2 (cerebroglycan)	71951	https://www.ncbi.nlm.nih.gov/gene/?term=71951
Simc1	Simc1	SUMO-interacting motifs containing 1	319719	https://www.ncbi.nlm.nih.gov/gene/?term=319719
Sacm1l	Sacm1l	SAC1 suppressor of actin mutations 1-like (yeast)	83493	https://www.ncbi.nlm.nih.gov/gene/?term=83493
Klhl28	Klhl28	kelch-like 28	66689	https://www.ncbi.nlm.nih.gov/gene/?term=66689
Osbpl6	Osbpl6	oxysterol binding protein-like 6	99031	https://www.ncbi.nlm.nih.gov/gene/?term=99031
Yaf2	Yaf2	YY1 associated factor 2	67057	https://www.ncbi.nlm.nih.gov/gene/?term=67057
Hs2st1	Hs2st1	heparan sulfate 2-O-sulfotransferase 1	23908	https://www.ncbi.nlm.nih.gov/gene/?term=23908
Myadm	Myadm	myeloid-associated differentiation marker	50918	https://www.ncbi.nlm.nih.gov/gene/?term=50918
Zfr	Zfr	zinc finger RNA binding protein	22763	https://www.ncbi.nlm.nih.gov/gene/?term=22763
Crebrf	Crebrf	CREB3 regulatory factor	77128	https://www.ncbi.nlm.nih.gov/gene/?term=77128

Biology Open • Supplementary information

Epn1	Epn1	epsin 1	13854	https://www.ncbi.nlm.nih.gov/gene/?term=13854
Mindy3	Mindy3	MINDY lysine 48 deubiquitinase 3	66960	https://www.ncbi.nlm.nih.gov/gene/?term=66960
Rsad2	Rsad2	radical S-adenosyl methionine domain containing 2	58185	https://www.ncbi.nlm.nih.gov/gene/?term=58185
Cryz	Cryz	crystallin, zeta	12972	https://www.ncbi.nlm.nih.gov/gene/?term=12972
Ikzf2	Ikzf2	IKAROS family zinc finger 2	22779	https://www.ncbi.nlm.nih.gov/gene/?term=22779
Dcp2	Dcp2	decapping mRNA 2	70640	https://www.ncbi.nlm.nih.gov/gene/?term=70640
Crlf3	Crlf3	cytokine receptor-like factor 3	54394	https://www.ncbi.nlm.nih.gov/gene/?term=54394
Rnft1	Rnft1	ring finger protein, transmembrane 1	76892	https://www.ncbi.nlm.nih.gov/gene/?term=76892
Dstyk	Dstyk	dual serine/threonine and tyrosine protein kinase	213452	https://www.ncbi.nlm.nih.gov/gene/?term=213452
Tcf12	Tcf12	transcription factor 12	21406	https://www.ncbi.nlm.nih.gov/gene/?term=21406
Ube3a	Ube3a	ubiquitin protein ligase E3A	22215	https://www.ncbi.nlm.nih.gov/gene/?term=22215
Ythdf2	Ythdf2	YTH N6-methyladenosine RNA binding protein 2	213541	https://www.ncbi.nlm.nih.gov/gene/?term=213541
St3gal3	St3gal3	ST3 beta-galactoside alpha-2,3-sialyltransferase 3	20441	https://www.ncbi.nlm.nih.gov/gene/?term=20441
Iqschfp	Iqschfp	Iqcj and Schip1 fusion protein	100505386	https://www.ncbi.nlm.nih.gov/gene/?term=100505
4921524J17Rik	4921524J17Rik	RIKEN cDNA 4921524J17 gene	66714	https://www.ncbi.nlm.nih.gov/gene/?term=66714
Supt6	Supt6	suppressor of Ty 6	20926	https://www.ncbi.nlm.nih.gov/gene/?term=20926
Tgfb2	Tgfb2	transforming growth factor, beta 2	21808	https://www.ncbi.nlm.nih.gov/gene/?term=21808
Mrpl17	Mrpl17	mitochondrial ribosomal protein L17	27397	https://www.ncbi.nlm.nih.gov/gene/?term=27397
Pgrmc2	Pgrmc2	progesterone receptor membrane component 2	70804	https://www.ncbi.nlm.nih.gov/gene/?term=70804
Prkacb	Prkacb	protein kinase, cAMP dependent, catalytic, beta	18749	https://www.ncbi.nlm.nih.gov/gene/?term=18749

Biology Open • Supplementary information

Chd2	Chd2	chromodomain helicase DNA binding protein 2	244059	https://www.ncbi.nlm.nih.gov/gene/?term=244059
Nkd1	Nkd1	naked cuticle 1	93960	https://www.ncbi.nlm.nih.gov/gene/?term=93960
Sobp	Sobp	sine oculis binding protein	109205	https://www.ncbi.nlm.nih.gov/gene/?term=109205
Wdfy3	Wdfy3	WD repeat and FYVE domain containing 3	72145	https://www.ncbi.nlm.nih.gov/gene/?term=72145
Trhde	Trhde	TRH-degrading enzyme	237553	https://www.ncbi.nlm.nih.gov/gene/?term=237553
Ccdc80	Ccdc80	coiled-coil domain containing 80	67896	https://www.ncbi.nlm.nih.gov/gene/?term=67896
Zbtb34	Zbtb34	zinc finger and BTB domain containing 34	241311	https://www.ncbi.nlm.nih.gov/gene/?term=241311
Lysmd3	Lysmd3	LysM, putative peptidoglycan-binding, domain containing 3	80289	https://www.ncbi.nlm.nih.gov/gene/?term=80289
Atxn7	Atxn7	ataxin 7	246103	https://www.ncbi.nlm.nih.gov/gene/?term=246103
Astn1	Astn1	astrotactin 1	11899	https://www.ncbi.nlm.nih.gov/gene/?term=11899
Stx2	Stx2	syntaxin 2	13852	https://www.ncbi.nlm.nih.gov/gene/?term=13852
Brd3	Brd3	bromodomain containing 3	67382	https://www.ncbi.nlm.nih.gov/gene/?term=67382
Map7d1	Map7d1	MAP7 domain containing 1	245877	https://www.ncbi.nlm.nih.gov/gene/?term=245877
Cdk8	Cdk8	cyclin-dependent kinase 8	264064	https://www.ncbi.nlm.nih.gov/gene/?term=264064
Npas3	Npas3	neuronal PAS domain protein 3	27386	https://www.ncbi.nlm.nih.gov/gene/?term=27386
Tada1	Tada1	transcriptional adaptor 1	27878	https://www.ncbi.nlm.nih.gov/gene/?term=27878
Nfasc	Nfasc	neurofascin	269116	https://www.ncbi.nlm.nih.gov/gene/?term=269116
Lbr	Lbr	lamin B receptor	98386	https://www.ncbi.nlm.nih.gov/gene/?term=98386
Rheb	Rheb	Ras homolog enriched in brain	19744	https://www.ncbi.nlm.nih.gov/gene/?term=19744
Lrrc8a	Lrrc8a	leucine rich repeat containing 8A	241296	https://www.ncbi.nlm.nih.gov/gene/?term=241296
Zfp644	Zfp644	zinc finger protein 644	52397	https://www.ncbi.nlm.nih.gov/gene/?term=52397
Ccdc177	Ccdc177	coiled-coil domain containing 177	380768	https://www.ncbi.nlm.nih.gov/gene/?term=380768
Mbnl3	Mbnl3	muscleblind like splicing factor 3	171170	https://www.ncbi.nlm.nih.gov/gene/?term=171170
Cdc25a	Cdc25a	cell division cycle 25A	12530	https://www.ncbi.nlm.nih.gov/gene/?term=12530

Biology Open • Supplementary information

C3ar1	C3ar1	complement component 3a receptor 1	12267	https://www.ncbi.nlm.nih.gov/gene/?term=12267
E2f3	E2f3	E2F transcription factor 3	13557	https://www.ncbi.nlm.nih.gov/gene/?term=13557
9330159F19Rik	9330159F19Rik	RIKEN cDNA 9330159F19 gene	212448	https://www.ncbi.nlm.nih.gov/gene/?term=212448
Tcerg1	Tcerg1	transcription elongation regulator 1 (CA150)	56070	https://www.ncbi.nlm.nih.gov/gene/?term=56070
Zfp597	Zfp597	zinc finger protein 597	71063	https://www.ncbi.nlm.nih.gov/gene/?term=71063
Kcng3	Kcng3	potassium voltage-gated channel, subfamily G, member 3	225030	https://www.ncbi.nlm.nih.gov/gene/?term=225030
Tnrc6b	Tnrc6b	trinucleotide repeat containing 6b	213988	https://www.ncbi.nlm.nih.gov/gene/?term=213988
Stk17b	Stk17b	serine/threonine kinase 17b (apoptosis-inducing)	98267	https://www.ncbi.nlm.nih.gov/gene/?term=98267
Stxbp5	Stxbp5	syntaxin binding protein 5 (tomosyn)	78808	https://www.ncbi.nlm.nih.gov/gene/?term=78808
Cpeb3	Cpeb3	cytoplasmic polyadenylation element binding protein 3	208922	https://www.ncbi.nlm.nih.gov/gene/?term=208922
Fam227a	Fam227a	family with sequence similarity 227, member A	75729	https://www.ncbi.nlm.nih.gov/gene/?term=75729
Dcun1d3	Dcun1d3	DCN1, defective in cullin neddylation 1, domain containing 3 (S. cerevisiae)	233805	https://www.ncbi.nlm.nih.gov/gene/?term=233805
Tiam1	Tiam1	T cell lymphoma invasion and metastasis 1	21844	https://www.ncbi.nlm.nih.gov/gene/?term=21844
Tmem135	Tmem135	transmembrane protein 135	72759	https://www.ncbi.nlm.nih.gov/gene/?term=72759
Taf12	Taf12	TATA-box binding protein associated factor 12	66464	https://www.ncbi.nlm.nih.gov/gene/?term=66464
Tmem221	Tmem221	transmembrane protein 221	434325	https://www.ncbi.nlm.nih.gov/gene/?term=434325
Fnip2	Fnip2	folliculin interacting protein 2	329679	https://www.ncbi.nlm.nih.gov/gene/?term=329679

Biology Open • Supplementary information

Atrnl1	Atrnl1	attractin like 1	226255	https://www.ncbi.nlm.nih.gov/gene/?term=226255
Tmeff1	Tmeff1	transmembrane protein with EGF-like and two follistatin-like domains 1	230157	https://www.ncbi.nlm.nih.gov/gene/?term=230157
Arpc5	Arpc5	actin related protein 2/3 complex, subunit 5	67771	https://www.ncbi.nlm.nih.gov/gene/?term=67771
Fam91a1	Fam91a1	family with sequence similarity 91, member A1	210998	https://www.ncbi.nlm.nih.gov/gene/?term=210998
Ranbp6	Ranbp6	RAN binding protein 6	240614	https://www.ncbi.nlm.nih.gov/gene/?term=240614
Nrp1	Nrp1	neuropilin 1	18186	https://www.ncbi.nlm.nih.gov/gene/?term=18186
Kctd8	Kctd8	potassium channel tetramerisation domain containing 8	243043	https://www.ncbi.nlm.nih.gov/gene/?term=243043
Adck2	Adck2	aarF domain containing kinase 2	57869	https://www.ncbi.nlm.nih.gov/gene/?term=57869
Jag1	Jag1	jagged 1	16449	https://www.ncbi.nlm.nih.gov/gene/?term=16449
Tfap2c	Tfap2c	transcription factor AP-2, gamma	21420	https://www.ncbi.nlm.nih.gov/gene/?term=21420
Hcn1	Hcn1	hyperpolarization-activated, cyclic nucleotide-gated K+ 1	15165	https://www.ncbi.nlm.nih.gov/gene/?term=15165
Msantd4	Msantd4	Myb/SANT-like DNA-binding domain containing 4 with coiled-coils	78100	https://www.ncbi.nlm.nih.gov/gene/?term=78100
Tmem237	Tmem237	transmembrane protein 237	381259	https://www.ncbi.nlm.nih.gov/gene/?term=381259
Abhd18	Abhd18	abhydrolase domain containing 18	269423	https://www.ncbi.nlm.nih.gov/gene/?term=269423
Map2k4	Map2k4	mitogen-activated protein kinase kinase 4	26398	https://www.ncbi.nlm.nih.gov/gene/?term=26398
Qser1	Qser1	glutamine and serine rich 1	99003	https://www.ncbi.nlm.nih.gov/gene/?term=99003
Hmg20a	Hmg20a	high mobility group 20A	66867	https://www.ncbi.nlm.nih.gov/gene/?term=66867
Adgrb3	Adgrb3	adhesion G protein-coupled receptor B3	210933	https://www.ncbi.nlm.nih.gov/gene/?term=210933

Biology Open • Supplementary information

Stag1	Stag1	stromal antigen 1	20842	https://www.ncbi.nlm.nih.gov/gene/?term=20842
Slc6a9	Slc6a9	solute carrier family 6 (neurotransmitter transporter, glycine), member 9	14664	https://www.ncbi.nlm.nih.gov/gene/?term=14664
Hephl1	Hephl1	hephaestin-like 1	244698	https://www.ncbi.nlm.nih.gov/gene/?term=244698
Zyg11b	Zyg11b	zyg-ll family member B, cell cycle regulator	414872	https://www.ncbi.nlm.nih.gov/gene/?term=414872
Cfap97	Cfap97	cilia and flagella associated protein 97	66756	https://www.ncbi.nlm.nih.gov/gene/?term=66756
Ppm1e	Ppm1e	protein phosphatase 1E (PP2C domain containing)	320472	https://www.ncbi.nlm.nih.gov/gene/?term=320472
Il13ra2	Il13ra2	interleukin 13 receptor, alpha 2	16165	https://www.ncbi.nlm.nih.gov/gene/?term=16165
Adpgk	Adpgk	ADP-dependent glucokinase	72141	https://www.ncbi.nlm.nih.gov/gene/?term=72141
Mtmr6	Mtmr6	myotubularin related protein 6	219135	https://www.ncbi.nlm.nih.gov/gene/?term=219135
Ttpal	Ttpal	tocopherol (alpha) transfer protein-like	76080	https://www.ncbi.nlm.nih.gov/gene/?term=76080
Ttbk2	Ttbk2	tau tubulin kinase 2	140810	https://www.ncbi.nlm.nih.gov/gene/?term=140810
Arhgef18	Arhgef18	rho/rac guanine nucleotide exchange factor (GEF) 18	102098	https://www.ncbi.nlm.nih.gov/gene/?term=102098
Klf6	Klf6	Kruppel-like factor 6	23849	https://www.ncbi.nlm.nih.gov/gene/?term=23849
Sipa1l2	Sipa1l2	signal-induced proliferation-associated 1 like 2	244668	https://www.ncbi.nlm.nih.gov/gene/?term=244668
Klf12	Klf12	Kruppel-like factor 12	16597	https://www.ncbi.nlm.nih.gov/gene/?term=16597
Epha7	Epha7	Eph receptor A7	13841	https://www.ncbi.nlm.nih.gov/gene/?term=13841
Dnajc13	Dnajc13	DnaJ heat shock protein family (Hsp40) member C13	235567	https://www.ncbi.nlm.nih.gov/gene/?term=235567

Nufip2	Nufip2	nuclear fragile X mental retardation protein interacting protein 2	68564	https://www.ncbi.nlm.nih.gov/gene/?term=68564
Reck	Reck	reversion-inducing-cysteine-rich protein with kazal motifs	53614	https://www.ncbi.nlm.nih.gov/gene/?term=53614
Kif5b	Kif5b	kinesin family member 5B	16573	https://www.ncbi.nlm.nih.gov/gene/?term=16573
Ankrd44	Ankrd44	ankyrin repeat domain 44	329154	https://www.ncbi.nlm.nih.gov/gene/?term=329154
Ptprg	Ptprg	protein tyrosine phosphatase, receptor type, G	19270	https://www.ncbi.nlm.nih.gov/gene/?term=19270
Greb1l	Greb1l	growth regulation by estrogen in breast cancer-like	381157	https://www.ncbi.nlm.nih.gov/gene/?term=381157
Pdcd10	Pdcd10	programmed cell death 10	56426	https://www.ncbi.nlm.nih.gov/gene/?term=56426
Mbnl2	Mbnl2	muscleblind like splicing factor 2	105559	https://www.ncbi.nlm.nih.gov/gene/?term=105559
Foxf2	Foxf2	forkhead box F2	14238	https://www.ncbi.nlm.nih.gov/gene/?term=14238
Rac1	Rac1	Rac family small GTPase 1	19353	https://www.ncbi.nlm.nih.gov/gene/?term=19353
Tmem170b	Tmem170b	transmembrane protein 170B	621976	https://www.ncbi.nlm.nih.gov/gene/?term=621976
Tectb	Tectb	tectorin beta	21684	https://www.ncbi.nlm.nih.gov/gene/?term=21684
Gpr146	Gpr146	G protein-coupled receptor 146	80290	https://www.ncbi.nlm.nih.gov/gene/?term=80290
Clock	Clock	circadian locomotor output cycles kaput	12753	https://www.ncbi.nlm.nih.gov/gene/?term=12753
Zbtb37	Zbtb37	zinc finger and BTB domain containing 37	240869	https://www.ncbi.nlm.nih.gov/gene/?term=240869
Hoxa9	Hoxa9	homeobox A9	15405	https://www.ncbi.nlm.nih.gov/gene/?term=15405
Prelid3b	Prelid3b	PRELI domain containing 3B	66390	https://www.ncbi.nlm.nih.gov/gene/?term=66390
Slc44a2	Slc44a2	solute carrier family 44, member 2	68682	https://www.ncbi.nlm.nih.gov/gene/?term=68682
Msra	Msra	methionine sulfoxide reductase A	110265	https://www.ncbi.nlm.nih.gov/gene/?term=110265
Egr3	Egr3	early growth response 3	13655	https://www.ncbi.nlm.nih.gov/gene/?term=13655

Biology Open • Supplementary information

Sinhcaf	Sinhcaf	SIN3-HDAC complex associated factor	56306	https://www.ncbi.nlm.nih.gov/gene/?term=56306
Phyhipl	Phyhipl	phytanoyl-CoA hydroxylase interacting protein-like	70911	https://www.ncbi.nlm.nih.gov/gene/?term=70911
Vamp3	Vamp3	vesicle-associated membrane protein 3	22319	https://www.ncbi.nlm.nih.gov/gene/?term=22319
Grb2	Grb2	growth factor receptor bound protein 2	14784	https://www.ncbi.nlm.nih.gov/gene/?term=14784
Ncald	Ncald	neurocalcin delta	52589	https://www.ncbi.nlm.nih.gov/gene/?term=52589
Braf	Braf	Braf transforming gene	109880	https://www.ncbi.nlm.nih.gov/gene/?term=109880
Prrg3	Prrg3	proline rich Gla (G-carboxyglutamic acid) 3 (transmembrane)	208748	https://www.ncbi.nlm.nih.gov/gene/?term=208748
Palld	Palld	palladin, cytoskeletal associated protein	72333	https://www.ncbi.nlm.nih.gov/gene/?term=72333
Mtss1	Mtss1	metastasis suppressor 1	211401	https://www.ncbi.nlm.nih.gov/gene/?term=211401
Ankrd27	Ankrd27	ankyrin repeat domain 27 (VPS9 domain)	245886	https://www.ncbi.nlm.nih.gov/gene/?term=245886
Spin1	Spin1	spindlin 1	20729	https://www.ncbi.nlm.nih.gov/gene/?term=20729
Coro1c	Coro1c	coronin, actin binding protein 1C	23790	https://www.ncbi.nlm.nih.gov/gene/?term=23790
Tro	Tro	trophinin	56191	https://www.ncbi.nlm.nih.gov/gene/?term=56191
Fam126b	Fam126b	family with sequence similarity 126, member B	213056	https://www.ncbi.nlm.nih.gov/gene/?term=213056
Ednrb	Ednrb	endothelin receptor type B	13618	https://www.ncbi.nlm.nih.gov/gene/?term=13618
Vezt	Vezt	vezatin, adherens junctions transmembrane protein	215008	https://www.ncbi.nlm.nih.gov/gene/?term=215008
Add3	Add3	adducin 3 (gamma)	27360	https://www.ncbi.nlm.nih.gov/gene/?term=27360
Taf15	Taf15	TATA-box binding protein associated factor 15	70439	https://www.ncbi.nlm.nih.gov/gene/?term=70439

Etl4	Etl4	enhancer trap locus 4	208618	https://www.ncbi.nlm.nih.gov/gene/?term=208618
Phf20l1	Phf20l1	PHD finger protein 20-like 1	239510	https://www.ncbi.nlm.nih.gov/gene/?term=239510
Prkce	Prkce	protein kinase C, epsilon	18754	https://www.ncbi.nlm.nih.gov/gene/?term=18754
Mitf	Mitf	melanogenesis associated transcription factor	17342	https://www.ncbi.nlm.nih.gov/gene/?term=17342
Nudt13	Nudt13	nudix (nucleoside diphosphate linked moiety X)-type motif 13	67725	https://www.ncbi.nlm.nih.gov/gene/?term=67725
Gli2	Gli2	GLI-Kruppel family member GLI2	14633	https://www.ncbi.nlm.nih.gov/gene/?term=14633
Cd2ap	Cd2ap	CD2-associated protein	12488	https://www.ncbi.nlm.nih.gov/gene/?term=12488
Sp3	Sp3	trans-acting transcription factor 3	20687	https://www.ncbi.nlm.nih.gov/gene/?term=20687
Thbs2	Thbs2	thrombospondin 2	21826	https://www.ncbi.nlm.nih.gov/gene/?term=21826
Arhgef3	Arhgef3	Rho guanine nucleotide exchange factor (GEF) 3	71704	https://www.ncbi.nlm.nih.gov/gene/?term=71704
Inpp5a	Inpp5a	inositol polyphosphate-5-phosphatase A	212111	https://www.ncbi.nlm.nih.gov/gene/?term=212111
Shc4	Shc4	SHC (Src homology 2 domain containing) family, member 4	271849	https://www.ncbi.nlm.nih.gov/gene/?term=271849
Bnip3	Bnip3	NA	12176	https://www.ncbi.nlm.nih.gov/gene/?term=12176
Lmtk2	Lmtk2	lemur tyrosine kinase 2	231876	https://www.ncbi.nlm.nih.gov/gene/?term=231876
Magi1	Magi1	membrane associated guanylate kinase, WW and PDZ domain containing 1	14924	https://www.ncbi.nlm.nih.gov/gene/?term=14924
Stx5a	Stx5a	syntaxin 5A	56389	https://www.ncbi.nlm.nih.gov/gene/?term=56389
Nptx1	Nptx1	neuronal pentraxin 1	18164	https://www.ncbi.nlm.nih.gov/gene/?term=18164
Rasa1	Rasa1	RAS p21 protein activator 1	218397	https://www.ncbi.nlm.nih.gov/gene/?term=218397
Wasl	Wasl	Wiskott-Aldrich syndrome-like (human)	73178	https://www.ncbi.nlm.nih.gov/gene/?term=73178
Trabd2b	Trabd2b	TraB domain containing 2B	666048	https://www.ncbi.nlm.nih.gov/gene/?term=666048

Biology Open • Supplementary information

Elavl4	Elavl4	ELAV like RNA binding protein 4	15572	https://www.ncbi.nlm.nih.gov/gene/?term=15572
Wdr76	Wdr76	WD repeat domain 76	241627	https://www.ncbi.nlm.nih.gov/gene/?term=241627
Fam43a	Fam43a	family with sequence similarity 43, member A	224093	https://www.ncbi.nlm.nih.gov/gene/?term=224093
Topbp1	Topbp1	topoisomerase (DNA) II binding protein 1	235559	https://www.ncbi.nlm.nih.gov/gene/?term=235559
Slc39a9	Slc39a9	solute carrier family 39 (zinc transporter), member 9	328133	https://www.ncbi.nlm.nih.gov/gene/?term=328133
Rnf152	Rnf152	ring finger protein 152	320311	https://www.ncbi.nlm.nih.gov/gene/?term=320311
Rapgef5	Rapgef5	Rap guanine nucleotide exchange factor (GEF) 5	217944	https://www.ncbi.nlm.nih.gov/gene/?term=217944
Kdm2b	Kdm2b	lysine (K)-specific demethylase 2B	30841	https://www.ncbi.nlm.nih.gov/gene/?term=30841
Adcy6	Adcy6	adenylate cyclase 6	11512	https://www.ncbi.nlm.nih.gov/gene/?term=11512
Slc1a1	Slc1a1	solute carrier family 1 (neuronal/epithelial high affinity glutamate transporter, system Xag), member 1	20510	https://www.ncbi.nlm.nih.gov/gene/?term=20510
Pcnx	Pcnx	pecanex homolog	54604	https://www.ncbi.nlm.nih.gov/gene/?term=54604
Myrip	Myrip	myosin VIIA and Rab interacting protein	245049	https://www.ncbi.nlm.nih.gov/gene/?term=245049
Phip	Phip	pleckstrin homology domain interacting protein	83946	https://www.ncbi.nlm.nih.gov/gene/?term=83946
Stox2	Stox2	storkhead box 2	71069	https://www.ncbi.nlm.nih.gov/gene/?term=71069
Wdr5b	Wdr5b	WD repeat domain 5B	69544	https://www.ncbi.nlm.nih.gov/gene/?term=69544
Ell	Ell	elongation factor RNA polymerase II	13716	https://www.ncbi.nlm.nih.gov/gene/?term=13716

Biology Open • Supplementary information

Dcun1d1	Dcun1d1	DCN1, defective in cullin neddylation 1, domain containing 1 (S. cerevisiae)	114893	https://www.ncbi.nlm.nih.gov/gene/?term=114893
Tmem145	Tmem145	transmembrane protein 145	330485	https://www.ncbi.nlm.nih.gov/gene/?term=330485
Pnpla2	Pnpla2	patatin-like phospholipase domain containing 2	66853	https://www.ncbi.nlm.nih.gov/gene/?term=66853
Kif19a	Kif19a	kinesin family member 19A	286942	https://www.ncbi.nlm.nih.gov/gene/?term=286942
Aldh6a1	Aldh6a1	aldehyde dehydrogenase family 6, subfamily A1	104776	https://www.ncbi.nlm.nih.gov/gene/?term=104776
Ell2	Ell2	elongation factor RNA polymerase II 2	192657	https://www.ncbi.nlm.nih.gov/gene/?term=192657
Lims1	Lims1	LIM and senescent cell antigen-like domains 1	110829	https://www.ncbi.nlm.nih.gov/gene/?term=110829
Cacnb4	Cacnb4	calcium channel, voltage-dependent, beta 4 subunit	12298	https://www.ncbi.nlm.nih.gov/gene/?term=12298
Gfm2	Gfm2	G elongation factor, mitochondrial 2	320806	https://www.ncbi.nlm.nih.gov/gene/?term=320806
Xpr1	Xpr1	xenotropic and polytropic retrovirus receptor 1	19775	https://www.ncbi.nlm.nih.gov/gene/?term=19775
Cbarp	Cbarp	calcium channel, voltage-dependent, beta subunit associated regulatory protein	100503659	https://www.ncbi.nlm.nih.gov/gene/?term=100503
Pcx	Pcx	pyruvate carboxylase	18563	https://www.ncbi.nlm.nih.gov/gene/?term=18563
Zcchc14	Zcchc14	zinc finger, CCHC domain containing 14	142682	https://www.ncbi.nlm.nih.gov/gene/?term=142682
Yipf4	Yipf4	Yip1 domain family, member 4	67864	https://www.ncbi.nlm.nih.gov/gene/?term=67864
Ints13	Ints13	integrator complex subunit 13	71177	https://www.ncbi.nlm.nih.gov/gene/?term=71177
Magel2	Magel2	melanoma antigen, family L, 2	27385	https://www.ncbi.nlm.nih.gov/gene/?term=27385

Biology Open • Supplementary information

Dok4	Dok4	docking protein 4	114255	https://www.ncbi.nlm.nih.gov/gene/?term=114255
Chic1	Chic1	cysteine-rich hydrophobic domain 1	12212	https://www.ncbi.nlm.nih.gov/gene/?term=12212
Cdv3	Cdv3	carnitine deficiency-associated gene expressed in ventricle 3	321022	https://www.ncbi.nlm.nih.gov/gene/?term=321022
Gnaq	Gnaq	guanine nucleotide binding protein, alpha q polypeptide	14682	https://www.ncbi.nlm.nih.gov/gene/?term=14682
Bcl11a	Bcl11a	B cell CLL/lymphoma 11A (zinc finger protein)	14025	https://www.ncbi.nlm.nih.gov/gene/?term=14025
Slitrk4	Slitrk4	SLIT and NTRK-like family, member 4	245446	https://www.ncbi.nlm.nih.gov/gene/?term=245446
Dab1	Dab1	disabled 1	13131	https://www.ncbi.nlm.nih.gov/gene/?term=13131
Ralgps2	Ralgps2	Ral GEF with PH domain and SH3 binding motif 2	78255	https://www.ncbi.nlm.nih.gov/gene/?term=78255
Prtg	Prtg	protogenin	235472	https://www.ncbi.nlm.nih.gov/gene/?term=235472
Rgs17	Rgs17	regulator of G-protein signaling 17	56533	https://www.ncbi.nlm.nih.gov/gene/?term=56533
Slc1a2	Slc1a2	solute carrier family 1 (glial high affinity glutamate transporter), member 2	20511	https://www.ncbi.nlm.nih.gov/gene/?term=20511
Retreg1	Retreg1	reticulophagy regulator 1	66270	https://www.ncbi.nlm.nih.gov/gene/?term=66270
Brpf3	Brpf3	bromodomain and PHD finger containing, 3	268936	https://www.ncbi.nlm.nih.gov/gene/?term=268936
Mrtfb	Mrtfb	myocardin related transcription factor B	239719	https://www.ncbi.nlm.nih.gov/gene/?term=239719
Arf4	Arf4	ADP-ribosylation factor 4	11843	https://www.ncbi.nlm.nih.gov/gene/?term=11843
Igsf3	Igsf3	immunoglobulin superfamily, member 3	78908	https://www.ncbi.nlm.nih.gov/gene/?term=78908
Zc3h15	Zc3h15	zinc finger CCCH-type containing 15	69082	https://www.ncbi.nlm.nih.gov/gene/?term=69082

Biology Open • Supplementary information

Dock1	Dock1	dedicator of cytokinesis 1	330662	https://www.ncbi.nlm.nih.gov/gene/?term=330662
Rarg	Rarg	retinoic acid receptor, gamma	19411	https://www.ncbi.nlm.nih.gov/gene/?term=19411
Dcaf4	Dcaf4	DDB1 and CUL4 associated factor 4	73828	https://www.ncbi.nlm.nih.gov/gene/?term=73828
Mak	Mak	male germ cell-associated kinase	17152	https://www.ncbi.nlm.nih.gov/gene/?term=17152
Nrn1	Nrn1	neuritin 1	68404	https://www.ncbi.nlm.nih.gov/gene/?term=68404
Anxa11	Anxa11	annexin A11	11744	https://www.ncbi.nlm.nih.gov/gene/?term=11744
Fut9	Fut9	fucosyltransferase 9	14348	https://www.ncbi.nlm.nih.gov/gene/?term=14348
Gys2	Gys2	glycogen synthase 2	232493	https://www.ncbi.nlm.nih.gov/gene/?term=232493
Cep250	Cep250	centrosomal protein 250	16328	https://www.ncbi.nlm.nih.gov/gene/?term=16328
Foxo3	Foxo3	forkhead box O3	56484	https://www.ncbi.nlm.nih.gov/gene/?term=56484
Brms1l	Brms1l	breast cancer metastasis-suppressor 1-like	52592	https://www.ncbi.nlm.nih.gov/gene/?term=52592
Plekha7	Plekha7	pleckstrin homology domain containing, family A member 7	233765	https://www.ncbi.nlm.nih.gov/gene/?term=233765
Baalc	Baalc	brain and acute leukemia, cytoplasmic	118452	https://www.ncbi.nlm.nih.gov/gene/?term=118452
Igf1r	Igf1r	insulin-like growth factor I receptor	16001	https://www.ncbi.nlm.nih.gov/gene/?term=16001
Snx30	Snx30	sorting nexin family member 30	209131	https://www.ncbi.nlm.nih.gov/gene/?term=209131
Fam171a1	Fam171a1	family with sequence similarity 171, member A1	269233	https://www.ncbi.nlm.nih.gov/gene/?term=269233
Frs2	Frs2	fibroblast growth factor receptor substrate 2	327826	https://www.ncbi.nlm.nih.gov/gene/?term=327826
Pbx2	Pbx2	pre B cell leukemia homeobox 2	18515	https://www.ncbi.nlm.nih.gov/gene/?term=18515
Slc39a1	Slc39a1	solute carrier family 39 (zinc transporter), member 1	30791	https://www.ncbi.nlm.nih.gov/gene/?term=30791
Fam168a	Fam168a	family with sequence similarity 168, member A	319604	https://www.ncbi.nlm.nih.gov/gene/?term=319604
Cnnm3	Cnnm3	cyclin M3	94218	https://www.ncbi.nlm.nih.gov/gene/?term=94218

Biology Open • Supplementary information

Ccny	Ccny	cyclin Y	67974	https://www.ncbi.nlm.nih.gov/gene/?term=67974
Usp13	Usp13	ubiquitin specific peptidase 13 (isopeptidase T-3)	72607	https://www.ncbi.nlm.nih.gov/gene/?term=72607
L1cam	L1cam	L1 cell adhesion molecule	16728	https://www.ncbi.nlm.nih.gov/gene/?term=16728
Wwc2	Wwc2	WW, C2 and coiled-coil domain containing 2	52357	https://www.ncbi.nlm.nih.gov/gene/?term=52357
Chst1	Chst1	carbohydrate sulfotransferase 1	76969	https://www.ncbi.nlm.nih.gov/gene/?term=76969
N4bp1	N4bp1	NEDD4 binding protein 1	80750	https://www.ncbi.nlm.nih.gov/gene/?term=80750
Has2	Has2	hyaluronan synthase 2	15117	https://www.ncbi.nlm.nih.gov/gene/?term=15117
Nid1	Nid1	nidogen 1	18073	https://www.ncbi.nlm.nih.gov/gene/?term=18073
Mast4	Mast4	microtubule associated serine/threonine kinase family member 4	328329	https://www.ncbi.nlm.nih.gov/gene/?term=328329
Dock4	Dock4	dedicator of cytokinesis 4	238130	https://www.ncbi.nlm.nih.gov/gene/?term=238130
Cbfa2t3	Cbfa2t3	CBFA2/RUNX1 translocation partner 3	12398	https://www.ncbi.nlm.nih.gov/gene/?term=12398
Pdzd8	Pdzd8	PDZ domain containing 8	107368	https://www.ncbi.nlm.nih.gov/gene/?term=107368
Rnf208	Rnf208	ring finger protein 208	68846	https://www.ncbi.nlm.nih.gov/gene/?term=68846
Smad1	Smad1	SMAD family member 1	17125	https://www.ncbi.nlm.nih.gov/gene/?term=17125
Rev1	Rev1	REV1, DNA directed polymerase	56210	https://www.ncbi.nlm.nih.gov/gene/?term=56210
Bcl2l12	Bcl2l12	BCL2-like 12 (proline rich)	75736	https://www.ncbi.nlm.nih.gov/gene/?term=75736
Kdelr2	Kdelr2	KDEL (Lys-Asp-Glu-Leu) endoplasmic reticulum protein retention receptor 2	66913	https://www.ncbi.nlm.nih.gov/gene/?term=66913
Ipcef1	Ipcef1	interaction protein for cytohesin exchange factors 1	320495	https://www.ncbi.nlm.nih.gov/gene/?term=320495
Adgrl2	Adgrl2	adhesion G protein-coupled receptor L2	99633	https://www.ncbi.nlm.nih.gov/gene/?term=99633

Biology Open • Supplementary information

Cttn	Cttn	cortactin	13043	https://www.ncbi.nlm.nih.gov/gene/?term=13043
Nexmif	Nexmif	neurite extension and migration factor	245555	https://www.ncbi.nlm.nih.gov/gene/?term=245555
Zfc3h1	Zfc3h1	zinc finger, C3H1-type containing	216345	https://www.ncbi.nlm.nih.gov/gene/?term=216345
Cep83	Cep83	centrosomal protein 83	77048	https://www.ncbi.nlm.nih.gov/gene/?term=77048
Ube2q2	Ube2q2	ubiquitin-conjugating enzyme E2Q family member 2	109161	https://www.ncbi.nlm.nih.gov/gene/?term=109161
Rab2a	Rab2a	RAB2A, member RAS oncogene family	59021	https://www.ncbi.nlm.nih.gov/gene/?term=59021
Slco3a1	Slco3a1	solute carrier organic anion transporter family, member 3a1	108116	https://www.ncbi.nlm.nih.gov/gene/?term=108116
Zbtb41	Zbtb41	zinc finger and BTB domain containing 41	226470	https://www.ncbi.nlm.nih.gov/gene/?term=226470
Ahcyl1	Ahcyl1	S-adenosylhomocysteine hydrolase-like 1	229709	https://www.ncbi.nlm.nih.gov/gene/?term=229709
Satb2	Satb2	special AT-rich sequence binding protein 2	212712	https://www.ncbi.nlm.nih.gov/gene/?term=212712
Col25a1	Col25a1	collagen, type XXV, alpha 1	77018	https://www.ncbi.nlm.nih.gov/gene/?term=77018
Adra2c	Adra2c	adrenergic receptor, alpha 2c	11553	https://www.ncbi.nlm.nih.gov/gene/?term=11553
Bcl2l13	Bcl2l13	BCL2-like 13 (apoptosis facilitator)	94044	https://www.ncbi.nlm.nih.gov/gene/?term=94044
Bach2	Bach2	BTB and CNC homology, basic leucine zipper transcription factor 2	12014	https://www.ncbi.nlm.nih.gov/gene/?term=12014
Ppm1k	Ppm1k	protein phosphatase 1K (PP2C domain containing)	243382	https://www.ncbi.nlm.nih.gov/gene/?term=243382
Tapt1	Tapt1	transmembrane anterior posterior transformation 1	231225	https://www.ncbi.nlm.nih.gov/gene/?term=231225
Taf4b	Taf4b	TATA-box binding protein associated factor 4b	72504	https://www.ncbi.nlm.nih.gov/gene/?term=72504

Cadm2	Cadm2	cell adhesion molecule 2	239857	https://www.ncbi.nlm.nih.gov/gene/?term=239857
Fam118a	Fam118a	family with sequence similarity 118, member A	73225	https://www.ncbi.nlm.nih.gov/gene/?term=73225
Coa7	Coa7	cytochrome c oxidase assembly factor 7	69893	https://www.ncbi.nlm.nih.gov/gene/?term=69893
Htr1a	Htr1a	5-hydroxytryptamine (serotonin) receptor 1A	15550	https://www.ncbi.nlm.nih.gov/gene/?term=15550
Tns3	Tns3	tensin 3	319939	https://www.ncbi.nlm.nih.gov/gene/?term=319939
Atat1	Atat1	alpha tubulin acetyltransferase 1	73242	https://www.ncbi.nlm.nih.gov/gene/?term=73242
Zfp36l1	Zfp36l1	zinc finger protein 36, C3H type-like 1	12192	https://www.ncbi.nlm.nih.gov/gene/?term=12192
Tagln3	Tagln3	transgelin 3	56370	https://www.ncbi.nlm.nih.gov/gene/?term=56370
Ppil1	Ppil1	peptidylprolyl isomerase (cyclophilin)-like 1	68816	https://www.ncbi.nlm.nih.gov/gene/?term=68816
Mtm1	Mtm1	X-linked myotubular myopathy gene 1	17772	https://www.ncbi.nlm.nih.gov/gene/?term=17772
Foxn2	Foxn2	forkhead box N2	14236	https://www.ncbi.nlm.nih.gov/gene/?term=14236
0610030E20Rik	0610030E20Rik	RIKEN cDNA 0610030E20 gene	68364	https://www.ncbi.nlm.nih.gov/gene/?term=68364
Wipi2	Wipi2	WD repeat domain, phosphoinositide interacting 2	74781	https://www.ncbi.nlm.nih.gov/gene/?term=74781
E330021D16Rik	E330021D16Rik	RIKEN cDNA E330021D16 gene	100502936	https://www.ncbi.nlm.nih.gov/gene/?term=100502
Csgalnact1	Csgalnact1	chondroitin sulfate N-acetylgalactosaminyltransferase 1	234356	https://www.ncbi.nlm.nih.gov/gene/?term=234356
Ttc17	Ttc17	tetratricopeptide repeat domain 17	74569	https://www.ncbi.nlm.nih.gov/gene/?term=74569
Zfp599	Zfp599	zinc finger protein 599	235048	https://www.ncbi.nlm.nih.gov/gene/?term=235048
Rnf38	Rnf38	ring finger protein 38	73469	https://www.ncbi.nlm.nih.gov/gene/?term=73469

Biology Open • Supplementary information

Nfkbid	Nfkbid	nuclear factor of kappa light polypeptide gene enhancer in B cells inhibitor, delta	243910	https://www.ncbi.nlm.nih.gov/gene/?term=243910
Ilf3	Ilf3	interleukin enhancer binding factor 3	16201	https://www.ncbi.nlm.nih.gov/gene/?term=16201
Cpne8	Cpne8	copine VIII	66871	https://www.ncbi.nlm.nih.gov/gene/?term=66871
Gls	Gls	glutaminase	14660	https://www.ncbi.nlm.nih.gov/gene/?term=14660
Ceacam18	Ceacam18	carcinoembryonic antigen-related cell adhesion molecule 18	72431	https://www.ncbi.nlm.nih.gov/gene/?term=72431
Nasp	Nasp	nuclear autoantigenic sperm protein (histone-binding)	50927	https://www.ncbi.nlm.nih.gov/gene/?term=50927
Ms4a6c	Ms4a6c	membrane-spanning 4-domains, subfamily A, member 6C	73656	https://www.ncbi.nlm.nih.gov/gene/?term=73656
Sumo1	Sumo1	small ubiquitin-like modifier 1	22218	https://www.ncbi.nlm.nih.gov/gene/?term=22218
Tgm6	Tgm6	transglutaminase 6	241636	https://www.ncbi.nlm.nih.gov/gene/?term=241636
Ythdf1	Ythdf1	YTH N6-methyladenosine RNA binding protein 1	228994	https://www.ncbi.nlm.nih.gov/gene/?term=228994
Tbx6	Tbx6	T-box 6	21389	https://www.ncbi.nlm.nih.gov/gene/?term=21389
Stn1	Stn1	STN1, CST complex subunit	108689	https://www.ncbi.nlm.nih.gov/gene/?term=108689
Nus1	Nus1	NUS1 dehydrodolichyl diphosphate synthase subunit	52014	https://www.ncbi.nlm.nih.gov/gene/?term=52014
Ncam1	Ncam1	neural cell adhesion molecule 1	17967	https://www.ncbi.nlm.nih.gov/gene/?term=17967
Acvr1b	Acvr1b	activin A receptor, type 1B	11479	https://www.ncbi.nlm.nih.gov/gene/?term=11479
Ranbp3	Ranbp3	RAN binding protein 3	71810	https://www.ncbi.nlm.nih.gov/gene/?term=71810
Ripply3	Ripply3	ripply transcriptional repressor 3	170765	https://www.ncbi.nlm.nih.gov/gene/?term=170765
Tfpi2	Tfpi2	tissue factor pathway inhibitor 2	21789	https://www.ncbi.nlm.nih.gov/gene/?term=21789
A1bg	A1bg	alpha-1-B glycoprotein	117586	https://www.ncbi.nlm.nih.gov/gene/?term=117586
Plpp3	Plpp3	phospholipid phosphatase 3	67916	https://www.ncbi.nlm.nih.gov/gene/?term=67916

Biology Open • Supplementary information

Insig2	Insig2	insulin induced gene 2	72999	https://www.ncbi.nlm.nih.gov/gene/?term=72999
Armc1	Armc1	armadillo repeat containing 1	74252	https://www.ncbi.nlm.nih.gov/gene/?term=74252
Foxc1	Foxc1	forkhead box C1	17300	https://www.ncbi.nlm.nih.gov/gene/?term=17300
Atad2	Atad2	ATPase family, AAA domain containing 2	70472	https://www.ncbi.nlm.nih.gov/gene/?term=70472
Wnt5a	Wnt5a	wingless-type MMTV integration site family, member 5A	22418	https://www.ncbi.nlm.nih.gov/gene/?term=22418
Tmprss11e	Tmprss11e	transmembrane protease, serine 11e	243084	https://www.ncbi.nlm.nih.gov/gene/?term=243084
Ubr3	Ubr3	ubiquitin protein ligase E3 component n-recognin 3	68795	https://www.ncbi.nlm.nih.gov/gene/?term=68795
Usp53	Usp53	ubiquitin specific peptidase 53	99526	https://www.ncbi.nlm.nih.gov/gene/?term=99526
Cyp2c50	Cyp2c50	cytochrome P450, family 2, subfamily c, polypeptide 50	107141	https://www.ncbi.nlm.nih.gov/gene/?term=107141
Jchain	Jchain	immunoglobulin joining chain	16069	https://www.ncbi.nlm.nih.gov/gene/?term=16069
Kif3c	Kif3c	kinesin family member 3C	16570	https://www.ncbi.nlm.nih.gov/gene/?term=16570
4930447C04Rik	4930447C04Rik	RIKEN cDNA 4930447C04 gene	75801	https://www.ncbi.nlm.nih.gov/gene/?term=75801
Usp22	Usp22	ubiquitin specific peptidase 22	216825	https://www.ncbi.nlm.nih.gov/gene/?term=216825
Syt6	Syt6	synaptotagmin VI	54524	https://www.ncbi.nlm.nih.gov/gene/?term=54524
Ism1	Ism1	isthmin 1, angiogenesis inhibitor	319909	https://www.ncbi.nlm.nih.gov/gene/?term=319909
Sgms1	Sgms1	sphingomyelin synthase 1	208449	https://www.ncbi.nlm.nih.gov/gene/?term=208449
Nptn	Nptn	neuroplastin	20320	https://www.ncbi.nlm.nih.gov/gene/?term=20320
Arpp19	Arpp19	cAMP-regulated phosphoprotein 19	59046	https://www.ncbi.nlm.nih.gov/gene/?term=59046
Sik1	Sik1	salt inducible kinase 1	17691	https://www.ncbi.nlm.nih.gov/gene/?term=17691
Smad2	Smad2	SMAD family member 2	17126	https://www.ncbi.nlm.nih.gov/gene/?term=17126
Rab34	Rab34	RAB34, member RAS oncogene family	19376	https://www.ncbi.nlm.nih.gov/gene/?term=19376

Biology Open • Supplementary information

Barhl2	Barhl2	BarH like homeobox 2	104382	https://www.ncbi.nlm.nih.gov/gene/?term=104382
Arhgap21	Arhgap21	Rho GTPase activating protein 21	71435	https://www.ncbi.nlm.nih.gov/gene/?term=71435
Plaa	Plaa	phospholipase A2, activating protein	18786	https://www.ncbi.nlm.nih.gov/gene/?term=18786
Ucp3	Ucp3	uncoupling protein 3 (mitochondrial, proton carrier)	22229	https://www.ncbi.nlm.nih.gov/gene/?term=22229
Cand1	Cand1	cullin associated and neddylation disassociated 1	71902	https://www.ncbi.nlm.nih.gov/gene/?term=71902
Bcl2l11	Bcl2l11	BCL2-like 11 (apoptosis facilitator)	12125	https://www.ncbi.nlm.nih.gov/gene/?term=12125
Otud4	Otud4	OTU domain containing 4	73945	https://www.ncbi.nlm.nih.gov/gene/?term=73945
Oas1c	Oas1c	2'-5' oligoadenylate synthetase 1C	114643	https://www.ncbi.nlm.nih.gov/gene/?term=114643
Ptpn14	Ptpn14	protein tyrosine phosphatase, non-receptor type 14	19250	https://www.ncbi.nlm.nih.gov/gene/?term=19250
S1pr1	S1pr1	sphingosine-1-phosphate receptor 1	13609	https://www.ncbi.nlm.nih.gov/gene/?term=13609
Sos2	Sos2	SOS Ras/Rho guanine nucleotide exchange factor 2	20663	https://www.ncbi.nlm.nih.gov/gene/?term=20663
Itsn2	Itsn2	intersectin 2	20403	https://www.ncbi.nlm.nih.gov/gene/?term=20403
Npepl1	Npepl1	aminopeptidase-like 1	228961	https://www.ncbi.nlm.nih.gov/gene/?term=228961
Rtca	Rtca	RNA 3'-terminal phosphate cyclase	66368	https://www.ncbi.nlm.nih.gov/gene/?term=66368
Naaladl2	Naaladl2	N-acetylated alpha-linked acidic dipeptidase-like 2	635702	https://www.ncbi.nlm.nih.gov/gene/?term=635702
Maf1	Maf1	MAF1 homolog, negative regulator of RNA polymerase III	68877	https://www.ncbi.nlm.nih.gov/gene/?term=68877
Esr1	Esr1	estrogen receptor 1 (alpha)	13982	https://www.ncbi.nlm.nih.gov/gene/?term=13982
Atp8a1	Atp8a1	ATPase, aminophospholipid transporter (APLT), class I, type 8A, member 1	11980	https://www.ncbi.nlm.nih.gov/gene/?term=11980

Tmem9b	Tmem9b	TMEM9 domain family, member B	56786	https://www.ncbi.nlm.nih.gov/gene/?term=56786
Epn2	Epn2	epsin 2	13855	https://www.ncbi.nlm.nih.gov/gene/?term=13855
Szrd1	Szrd1	SUZ RNA binding domain containing 1	213491	https://www.ncbi.nlm.nih.gov/gene/?term=213491
Btbd3	Btbd3	BTB (POZ) domain containing 3	228662	https://www.ncbi.nlm.nih.gov/gene/?term=228662
Dicer1	Dicer1	dicer 1, ribonuclease type III	192119	https://www.ncbi.nlm.nih.gov/gene/?term=192119
Stam	Stam	signal transducing adaptor molecule (SH3 domain and ITAM motif) 1	20844	https://www.ncbi.nlm.nih.gov/gene/?term=20844
Akap1	Akap1	A kinase (PRKA) anchor protein 1	11640	https://www.ncbi.nlm.nih.gov/gene/?term=11640
Fbn1	Fbn1	fibrillin 1	14118	https://www.ncbi.nlm.nih.gov/gene/?term=14118
Ube3b	Ube3b	ubiquitin protein ligase E3B	117146	https://www.ncbi.nlm.nih.gov/gene/?term=117146
Ctxn3	Ctxn3	cortexin 3	629147	https://www.ncbi.nlm.nih.gov/gene/?term=629147
Arl6ip1	Arl6ip1	ADP-ribosylation factor-like 6 interacting protein 1	54208	https://www.ncbi.nlm.nih.gov/gene/?term=54208
Lrp2	Lrp2	low density lipoprotein receptor-related protein 2	14725	https://www.ncbi.nlm.nih.gov/gene/?term=14725
Cdk19	Cdk19	cyclin-dependent kinase 19	78334	https://www.ncbi.nlm.nih.gov/gene/?term=78334
Mospd1	Mospd1	motile sperm domain containing 1	70380	https://www.ncbi.nlm.nih.gov/gene/?term=70380
Tgfa	Tgfa	transforming growth factor alpha	21802	https://www.ncbi.nlm.nih.gov/gene/?term=21802
Spty2d1	Spty2d1	SPT2, Suppressor of Ty, domain containing 1 (S. cerevisiae)	101685	https://www.ncbi.nlm.nih.gov/gene/?term=101685
Ywhab	Ywhab	tyrosine 3-monooxygenase/tryptophan 5-monooxygenase activation protein, beta polypeptide	54401	https://www.ncbi.nlm.nih.gov/gene/?term=54401
Usp32	Usp32	ubiquitin specific peptidase 32	237898	https://www.ncbi.nlm.nih.gov/gene/?term=237898
Med12l	Med12l	mediator complex subunit 12-like	329650	https://www.ncbi.nlm.nih.gov/gene/?term=329650

Biology Open • Supplementary information

Nrarp	Nrarp	Notch-regulated ankyrin repeat protein	67122	https://www.ncbi.nlm.nih.gov/gene/?term=67122
Tmem54	Tmem54	transmembrane protein 54	66260	https://www.ncbi.nlm.nih.gov/gene/?term=66260
Fam104a	Fam104a	family with sequence similarity 104, member A	28081	https://www.ncbi.nlm.nih.gov/gene/?term=28081
Col4a1	Col4a1	collagen, type IV, alpha 1	12826	https://www.ncbi.nlm.nih.gov/gene/?term=12826
Hepacam2	Hepacam2	HEPACAM family member 2	101202	https://www.ncbi.nlm.nih.gov/gene/?term=101202
B4galt5	B4galt5	UDP-Gal:betaGlcNAc beta 1,4-galactosyltransferase, polypeptide 5	56336	https://www.ncbi.nlm.nih.gov/gene/?term=56336
Jmy	Jmy	junction-mediating and regulatory protein	57748	https://www.ncbi.nlm.nih.gov/gene/?term=57748
Tnrc6c	Tnrc6c	trinucleotide repeat containing 6C	217351	https://www.ncbi.nlm.nih.gov/gene/?term=217351
Abcb7	Abcb7	ATP-binding cassette, sub-family B (MDR/TAP), member 7	11306	https://www.ncbi.nlm.nih.gov/gene/?term=11306
Arrdc3	Arrdc3	arrestin domain containing 3	105171	https://www.ncbi.nlm.nih.gov/gene/?term=105171
Btaf1	Btaf1	B-TFIID TATA-box binding protein associated factor 1	107182	https://www.ncbi.nlm.nih.gov/gene/?term=107182
Rictor	Rictor	RPTOR independent companion of MTOR, complex 2	78757	https://www.ncbi.nlm.nih.gov/gene/?term=78757
Canx	Canx	calnexin	12330	https://www.ncbi.nlm.nih.gov/gene/?term=12330
Manba	Manba	mannosidase, beta A, lysosomal	110173	https://www.ncbi.nlm.nih.gov/gene/?term=110173
Robo1	Robo1	roundabout guidance receptor 1	19876	https://www.ncbi.nlm.nih.gov/gene/?term=19876
Phactr2	Phactr2	phosphatase and actin regulator 2	215789	https://www.ncbi.nlm.nih.gov/gene/?term=215789
Amacr	Amacr	alpha-methylacyl-CoA racemase	17117	https://www.ncbi.nlm.nih.gov/gene/?term=17117
Ncoa1	Ncoa1	nuclear receptor coactivator 1	17977	https://www.ncbi.nlm.nih.gov/gene/?term=17977
Rph3al	Rph3al	rabphilin 3A-like (without C2 domains)	380714	https://www.ncbi.nlm.nih.gov/gene/?term=380714

Biology Open • Supplementary information

Sgcb	Sgcb	sarcoglycan, beta (dystrophin-associated glycoprotein)	24051	https://www.ncbi.nlm.nih.gov/gene/?term=24051
Lgalsl	Lgalsl	lectin, galactoside binding-like	216551	https://www.ncbi.nlm.nih.gov/gene/?term=216551
Ppp6r1	Ppp6r1	protein phosphatase 6, regulatory subunit 1	243819	https://www.ncbi.nlm.nih.gov/gene/?term=243819
Slco4a1	Slco4a1	solute carrier organic anion transporter family, member 4a1	108115	https://www.ncbi.nlm.nih.gov/gene/?term=108115
Robo2	Robo2	roundabout guidance receptor 2	268902	https://www.ncbi.nlm.nih.gov/gene/?term=268902
Adamts15	Adamts15	a disintegrin-like and metallopeptidase (reprolysin type) with thrombospondin type 1 motif, 15	235130	https://www.ncbi.nlm.nih.gov/gene/?term=235130
Jph3	Jph3	junctophilin 3	57340	https://www.ncbi.nlm.nih.gov/gene/?term=57340
Fam234a	Fam234a	family with sequence similarity 234, member A	106581	https://www.ncbi.nlm.nih.gov/gene/?term=106581
Hbs1l	Hbs1l	Hbs1-like (S. cerevisiae)	56422	https://www.ncbi.nlm.nih.gov/gene/?term=56422
Hs3st1	Hs3st1	heparan sulfate (glucosamine) 3-O-sulfotransferase 1	15476	https://www.ncbi.nlm.nih.gov/gene/?term=15476
Inhbb	Inhbb	inhibin beta-B	16324	https://www.ncbi.nlm.nih.gov/gene/?term=16324
Ssr1	Ssr1	signal sequence receptor, alpha	107513	https://www.ncbi.nlm.nih.gov/gene/?term=107513
Dgcr8	Dgcr8	DGCR8, microprocessor complex subunit	94223	https://www.ncbi.nlm.nih.gov/gene/?term=94223
Ube2d3	Ube2d3	ubiquitin-conjugating enzyme E2D 3	66105	https://www.ncbi.nlm.nih.gov/gene/?term=66105
Ino80	Ino80	INO80 complex subunit	68142	https://www.ncbi.nlm.nih.gov/gene/?term=68142
C1galt1	C1galt1	core 1 synthase, glycoprotein-N-acetylgalactosamine 3-beta-galactosyltransferase, 1	94192	https://www.ncbi.nlm.nih.gov/gene/?term=94192

Biology Open • Supplementary information

Ajuba	Ajuba	ajuba LIM protein	16475	https://www.ncbi.nlm.nih.gov/gene/?term=16475
D5Ertd579e	D5Ertd579e	DNA segment, Chr 5, ERATO Doi 579, expressed	320661	https://www.ncbi.nlm.nih.gov/gene/?term=320661
Tnrc6a	Tnrc6a	trinucleotide repeat containing 6a	233833	https://www.ncbi.nlm.nih.gov/gene/?term=233833
Meox2	Meox2	mesenchyme homeobox 2	17286	https://www.ncbi.nlm.nih.gov/gene/?term=17286
Mdfic	Mdfic	MyoD family inhibitor domain containing	16543	https://www.ncbi.nlm.nih.gov/gene/?term=16543
Itga9	Itga9	integrin alpha 9	104099	https://www.ncbi.nlm.nih.gov/gene/?term=104099
Rmnd5a	Rmnd5a	required for meiotic nuclear division 5 homolog A	68477	https://www.ncbi.nlm.nih.gov/gene/?term=68477
Eogt	Eogt	EGF domain-specific O-linked N-acetylglucosamine (GlcNAc) transferase	101351	https://www.ncbi.nlm.nih.gov/gene/?term=101351
Relch	Relch	RAB11 binding and LisH domain, coiled-coil and HEAT repeat containing	227446	https://www.ncbi.nlm.nih.gov/gene/?term=227446
Itga5	Itga5	integrin alpha 5 (fibronectin receptor alpha)	16402	https://www.ncbi.nlm.nih.gov/gene/?term=16402
Tnpo1	Tnpo1	transportin 1	238799	https://www.ncbi.nlm.nih.gov/gene/?term=238799
Ppp1r10	Ppp1r10	protein phosphatase 1, regulatory subunit 10	52040	https://www.ncbi.nlm.nih.gov/gene/?term=52040
Phf3	Phf3	PHD finger protein 3	213109	https://www.ncbi.nlm.nih.gov/gene/?term=213109
Mpped2	Mpped2	metallophosphoesterase domain containing 2	77015	https://www.ncbi.nlm.nih.gov/gene/?term=77015
Psmb6	Psmb6	proteasome (prosome, macropain) subunit, beta type 6	19175	https://www.ncbi.nlm.nih.gov/gene/?term=19175
Cblb	Cblb	Casitas B-lineage lymphoma b	208650	https://www.ncbi.nlm.nih.gov/gene/?term=208650

Gadd45a	Gadd45a	growth arrest and DNA-damage-inducible 45 alpha	13197	https://www.ncbi.nlm.nih.gov/gene/?term=13197
Pde1a	Pde1a	phosphodiesterase 1A, calmodulin-dependent	18573	https://www.ncbi.nlm.nih.gov/gene/?term=18573
Cmip	Cmip	c-Maf inducing protein	74440	https://www.ncbi.nlm.nih.gov/gene/?term=74440
Serpinb2	Serpinb2	serine (or cysteine) peptidase inhibitor, clade B, member 2	18788	https://www.ncbi.nlm.nih.gov/gene/?term=18788
Sh3pxd2b	Sh3pxd2b	SH3 and PX domains 2B	268396	https://www.ncbi.nlm.nih.gov/gene/?term=268396
Bcr	Bcr	breakpoint cluster region	110279	https://www.ncbi.nlm.nih.gov/gene/?term=110279
Ttyh3	Ttyh3	tweety family member 3	78339	https://www.ncbi.nlm.nih.gov/gene/?term=78339
Plod2	Plod2	procollagen lysine, 2-oxoglutarate 5-dioxygenase 2	26432	https://www.ncbi.nlm.nih.gov/gene/?term=26432
Tsku	Tsku	tsukushi, small leucine rich proteoglycan	244152	https://www.ncbi.nlm.nih.gov/gene/?term=244152
Cep170b	Cep170b	centrosomal protein 170B	217882	https://www.ncbi.nlm.nih.gov/gene/?term=217882
Sh3kbp1	Sh3kbp1	SH3-domain kinase binding protein 1	58194	https://www.ncbi.nlm.nih.gov/gene/?term=58194
Tgfbr1	Tgfbr1	transforming growth factor, beta receptor I	21812	https://www.ncbi.nlm.nih.gov/gene/?term=21812
Med1	Med1	mediator complex subunit 1	19014	https://www.ncbi.nlm.nih.gov/gene/?term=19014
Slc16a9	Slc16a9	solute carrier family 16 (monocarboxylic acid transporters), member 9	66859	https://www.ncbi.nlm.nih.gov/gene/?term=66859
Gulp1	Gulp1	GULP, engulfment adaptor PTB domain containing 1	70676	https://www.ncbi.nlm.nih.gov/gene/?term=70676
Rab23	Rab23	RAB23, member RAS oncogene family	19335	https://www.ncbi.nlm.nih.gov/gene/?term=19335

Biology Open • Supplementary information

Eif4ebp2	Eif4ebp2	eukaryotic translation initiation factor 4E binding protein 2	13688	https://www.ncbi.nlm.nih.gov/gene/?term=13688
Gpc3	Gpc3	glypican 3	14734	https://www.ncbi.nlm.nih.gov/gene/?term=14734
Cnn3	Cnn3	calponin 3, acidic	71994	https://www.ncbi.nlm.nih.gov/gene/?term=71994
Nsg1	Nsg1	neuron specific gene family member 1	18196	https://www.ncbi.nlm.nih.gov/gene/?term=18196
Celsr1	Celsr1	cadherin, EGF LAG seven-pass G-type receptor 1	12614	https://www.ncbi.nlm.nih.gov/gene/?term=12614
Hsd3b5	Hsd3b5	hydroxy-delta-5-steroid dehydrogenase, 3 beta- and steroid delta-isomerase 5	15496	https://www.ncbi.nlm.nih.gov/gene/?term=15496
Zkscan17	Zkscan17	zinc finger with KRAB and SCAN domains 17	268417	https://www.ncbi.nlm.nih.gov/gene/?term=268417
Morf4l1	Morf4l1	mortality factor 4 like 1	21761	https://www.ncbi.nlm.nih.gov/gene/?term=21761
Ogt	Ogt	O-linked N-acetylglucosamine (GlcNAc) transferase (UDP-N-acetylglucosamine:polypeptide-N-acetylglucosaminyl transferase)	108155	https://www.ncbi.nlm.nih.gov/gene/?term=108155
Klhl7	Klhl7	kelch-like 7	52323	https://www.ncbi.nlm.nih.gov/gene/?term=52323
Ralgps1	Ralgps1	Ral GEF with PH domain and SH3 binding motif 1	241308	https://www.ncbi.nlm.nih.gov/gene/?term=241308
Foxq1	Foxq1	forkhead box Q1	15220	https://www.ncbi.nlm.nih.gov/gene/?term=15220
Aqp5	Aqp5	aquaporin 5	11830	https://www.ncbi.nlm.nih.gov/gene/?term=11830
Mtor	Mtor	mechanistic target of rapamycin kinase	56717	https://www.ncbi.nlm.nih.gov/gene/?term=56717
Foxk2	Foxk2	forkhead box K2	68837	https://www.ncbi.nlm.nih.gov/gene/?term=68837
Sox6	Sox6	SRY (sex determining region Y)-box 6	20679	https://www.ncbi.nlm.nih.gov/gene/?term=20679

Biology Open • Supplementary information

Pmepa1	Pmepa1	prostate transmembrane protein, androgen induced 1	65112	https://www.ncbi.nlm.nih.gov/gene/?term=65112
Cd164	Cd164	CD164 antigen	53599	https://www.ncbi.nlm.nih.gov/gene/?term=53599
Tcf7l2	Tcf7l2	transcription factor 7 like 2, T cell specific, HMG box	21416	https://www.ncbi.nlm.nih.gov/gene/?term=21416
Olfr613	Olfr613	olfactory receptor 613	259104	https://www.ncbi.nlm.nih.gov/gene/?term=259104
Fundc2	Fundc2	FUN14 domain containing 2	67391	https://www.ncbi.nlm.nih.gov/gene/?term=67391
Pde8b	Pde8b	phosphodiesterase 8B	218461	https://www.ncbi.nlm.nih.gov/gene/?term=218461
St6galnac3	St6galnac3	ST6 (alpha-N-acetyl-neuraminyl-2,3-beta-galactosyl-1,3)-N-acetylgalactosaminide alpha-2,6-sialyltransferase 3	20447	https://www.ncbi.nlm.nih.gov/gene/?term=20447
E2f5	E2f5	E2F transcription factor 5	13559	https://www.ncbi.nlm.nih.gov/gene/?term=13559
Ahr	Ahr	aryl-hydrocarbon receptor	11622	https://www.ncbi.nlm.nih.gov/gene/?term=11622
Trib3	Trib3	tribbles pseudokinase 3	228775	https://www.ncbi.nlm.nih.gov/gene/?term=228775
Sdc2	Sdc2	syndecan 2	15529	https://www.ncbi.nlm.nih.gov/gene/?term=15529
Slain2	Slain2	SLAIN motif family, member 2	75991	https://www.ncbi.nlm.nih.gov/gene/?term=75991
Casp2	Casp2	caspase 2	12366	https://www.ncbi.nlm.nih.gov/gene/?term=12366
Arhgap24	Arhgap24	Rho GTPase activating protein 24	231532	https://www.ncbi.nlm.nih.gov/gene/?term=231532
Ebf3	Ebf3	early B cell factor 3	13593	https://www.ncbi.nlm.nih.gov/gene/?term=13593
Dgkh	Dgkh	diacylglycerol kinase, eta	380921	https://www.ncbi.nlm.nih.gov/gene/?term=380921
Tes	Tes	testis derived transcript	21753	https://www.ncbi.nlm.nih.gov/gene/?term=21753
Ntn4	Ntn4	netrin 4	57764	https://www.ncbi.nlm.nih.gov/gene/?term=57764
Map2k1	Map2k1	mitogen-activated protein kinase kinase 1	26395	https://www.ncbi.nlm.nih.gov/gene/?term=26395
Hsf5	Hsf5	heat shock transcription factor family member 5	327992	https://www.ncbi.nlm.nih.gov/gene/?term=327992

Biology Open • Supplementary information

Rassf8	Rassf8	Ras association (RalGDS/AF-6) domain family (N-terminal) member 8	71323	https://www.ncbi.nlm.nih.gov/gene/?term=71323
Plppr4	Plppr4	phospholipid phosphatase related 4	229791	https://www.ncbi.nlm.nih.gov/gene/?term=229791
Abcd1	Abcd1	ATP-binding cassette, sub-family D (ALD), member 1	11666	https://www.ncbi.nlm.nih.gov/gene/?term=11666
Tut4	Tut4	terminal uridylyl transferase 4	230594	https://www.ncbi.nlm.nih.gov/gene/?term=230594
Rnf139	Rnf139	ring finger protein 139	75841	https://www.ncbi.nlm.nih.gov/gene/?term=75841
Zcchc3	Zcchc3	zinc finger, CCHC domain containing 3	67917	https://www.ncbi.nlm.nih.gov/gene/?term=67917
Hbegf	Hbegf	heparin-binding EGF-like growth factor	15200	https://www.ncbi.nlm.nih.gov/gene/?term=15200
B4galnt1	B4galnt1	beta-1,4-N-acetyl-galactosaminyl transferase 1	14421	https://www.ncbi.nlm.nih.gov/gene/?term=14421
Cpeb1	Cpeb1	cytoplasmic polyadenylation element binding protein 1	12877	https://www.ncbi.nlm.nih.gov/gene/?term=12877
Vat1l	Vat1l	vesicle amine transport protein 1 like	270097	https://www.ncbi.nlm.nih.gov/gene/?term=270097
Gad2	Gad2	glutamic acid decarboxylase 2	14417	https://www.ncbi.nlm.nih.gov/gene/?term=14417
Tac1	Tac1	tachykinin 1	21333	https://www.ncbi.nlm.nih.gov/gene/?term=21333
Ube2g1	Ube2g1	ubiquitin-conjugating enzyme E2G 1	67128	https://www.ncbi.nlm.nih.gov/gene/?term=67128
Galnt2	Galnt2	polypeptide N-acetylgalactosaminyltransferase 2	108148	https://www.ncbi.nlm.nih.gov/gene/?term=108148
Slc12a6	Slc12a6	solute carrier family 12, member 6	107723	https://www.ncbi.nlm.nih.gov/gene/?term=107723
Tmem198	Tmem198	transmembrane protein 198	319998	https://www.ncbi.nlm.nih.gov/gene/?term=319998

Biology Open • Supplementary information

Farp1	Farp1	FERM, RhoGEF (Arhgef) and pleckstrin domain protein 1 (chondrocyte-derived)	223254	https://www.ncbi.nlm.nih.gov/gene/?term=223254
Arf2	Arf2	ADP-ribosylation factor 2	11841	https://www.ncbi.nlm.nih.gov/gene/?term=11841
Ankib1	Ankib1	ankyrin repeat and IBR domain containing 1	70797	https://www.ncbi.nlm.nih.gov/gene/?term=70797
Ezr	Ezr	ezrin	22350	https://www.ncbi.nlm.nih.gov/gene/?term=22350
Arpc5l	Arpc5l	actin related protein 2/3 complex, subunit 5-like	74192	https://www.ncbi.nlm.nih.gov/gene/?term=74192
Dlat	Dlat	dihydrolipoamide S-acetyltransferase (E2 component of pyruvate dehydrogenase complex)	235339	https://www.ncbi.nlm.nih.gov/gene/?term=235339
Tbr1	Tbr1	T-box brain gene 1	21375	https://www.ncbi.nlm.nih.gov/gene/?term=21375
Prrt3	Prrt3	proline-rich transmembrane protein 3	210673	https://www.ncbi.nlm.nih.gov/gene/?term=210673
Zfp449	Zfp449	zinc finger protein 449	78619	https://www.ncbi.nlm.nih.gov/gene/?term=78619
Nova2	Nova2	neuro-oncological ventral antigen 2	384569	https://www.ncbi.nlm.nih.gov/gene/?term=384569
Pnpla8	Pnpla8	patatin-like phospholipase domain containing 8	67452	https://www.ncbi.nlm.nih.gov/gene/?term=67452
Gpr21	Gpr21	G protein-coupled receptor 21	338346	https://www.ncbi.nlm.nih.gov/gene/?term=338346
Eif5	Eif5	NA	217869	https://www.ncbi.nlm.nih.gov/gene/?term=217869
Rapgef4	Rapgef4	Rap guanine nucleotide exchange factor (GEF) 4	56508	https://www.ncbi.nlm.nih.gov/gene/?term=56508
Spast	Spast	spastin	50850	https://www.ncbi.nlm.nih.gov/gene/?term=50850
Grhl2	Grhl2	grainyhead like transcription factor 2	252973	https://www.ncbi.nlm.nih.gov/gene/?term=252973
Stmnd1	Stmnd1	stathmin domain containing 1	380842	https://www.ncbi.nlm.nih.gov/gene/?term=380842
Uck2	Uck2	uridine-cytidine kinase 2	80914	https://www.ncbi.nlm.nih.gov/gene/?term=80914

Biology Open • Supplementary information

Arsj	Arsj	arylsulfatase J	271970	https://www.ncbi.nlm.nih.gov/gene/?term=271970
Irs1	Irs1	insulin receptor substrate 1	16367	https://www.ncbi.nlm.nih.gov/gene/?term=16367
Jmjd1c	Jmjd1c	jumonji domain containing 1C	108829	https://www.ncbi.nlm.nih.gov/gene/?term=108829
Unc13c	Unc13c	unc-13 homolog C	208898	https://www.ncbi.nlm.nih.gov/gene/?term=208898
2610507B11Rik	2610507B11Rik	RIKEN cDNA 2610507B11 gene	72503	https://www.ncbi.nlm.nih.gov/gene/?term=72503
Adgra2	Adgra2	adhesion G protein-coupled receptor A2	78560	https://www.ncbi.nlm.nih.gov/gene/?term=78560
Bsdc1	Bsdc1	BSD domain containing 1	100383	https://www.ncbi.nlm.nih.gov/gene/?term=100383
Frmd5	Frmd5	FERM domain containing 5	228564	https://www.ncbi.nlm.nih.gov/gene/?term=228564
Epha3	Epha3	Eph receptor A3	13837	https://www.ncbi.nlm.nih.gov/gene/?term=13837
Snx16	Snx16	sorting nexin 16	74718	https://www.ncbi.nlm.nih.gov/gene/?term=74718
Ppp4r3a	Ppp4r3a	protein phosphatase 4 regulatory subunit 3A	68734	https://www.ncbi.nlm.nih.gov/gene/?term=68734
Rab3c	Rab3c	RAB3C, member RAS oncogene family	67295	https://www.ncbi.nlm.nih.gov/gene/?term=67295
Nlgn2	Nlgn2	neuroligin 2	216856	https://www.ncbi.nlm.nih.gov/gene/?term=216856
Nptx2	Nptx2	neuronal pentraxin 2	53324	https://www.ncbi.nlm.nih.gov/gene/?term=53324
Ctdsp1	Ctdsp1	CTD (carboxy-terminal domain, RNA polymerase II, polypeptide A) small phosphatase 1	227292	https://www.ncbi.nlm.nih.gov/gene/?term=227292
Spen	Spen	spen family transcription repressor	56381	https://www.ncbi.nlm.nih.gov/gene/?term=56381
Alk	Alk	anaplastic lymphoma kinase	11682	https://www.ncbi.nlm.nih.gov/gene/?term=11682
Oxsr1	Oxsr1	oxidative-stress responsive 1	108737	https://www.ncbi.nlm.nih.gov/gene/?term=108737
Galr1	Galr1	galanin receptor 1	14427	https://www.ncbi.nlm.nih.gov/gene/?term=14427
Umad1	Umad1	UMAP1-MVP12 associated (UMA) domain containing 1	100036521	https://www.ncbi.nlm.nih.gov/gene/?term=100036
Tmem230	Tmem230	transmembrane protein 230	70612	https://www.ncbi.nlm.nih.gov/gene/?term=70612

Biology Open • Supplementary information

Mapkap1	Mapkap1	mitogen-activated protein kinase associated protein 1	227743	https://www.ncbi.nlm.nih.gov/gene/?term=227743
Snca	Snca	synuclein, alpha	20617	https://www.ncbi.nlm.nih.gov/gene/?term=20617
Fam131b	Fam131b	family with sequence similarity 131, member B	76156	https://www.ncbi.nlm.nih.gov/gene/?term=76156
Socs2	Socs2	suppressor of cytokine signaling 2	216233	https://www.ncbi.nlm.nih.gov/gene/?term=216233
Fbxw2	Fbxw2	F-box and WD-40 domain protein 2	30050	https://www.ncbi.nlm.nih.gov/gene/?term=30050
Kif16b	Kif16b	kinesin family member 16B	16558	https://www.ncbi.nlm.nih.gov/gene/?term=16558
Rnf144a	Rnf144a	ring finger protein 144A	108089	https://www.ncbi.nlm.nih.gov/gene/?term=108089
Ccdc120	Ccdc120	coiled-coil domain containing 120	54648	https://www.ncbi.nlm.nih.gov/gene/?term=54648
Cnppd1	Cnppd1	cyclin Pas1/PHO80 domain containing 1	69171	https://www.ncbi.nlm.nih.gov/gene/?term=69171
Rnf41	Rnf41	ring finger protein 41	67588	https://www.ncbi.nlm.nih.gov/gene/?term=67588
Itga4	Itga4	integrin alpha 4	16401	https://www.ncbi.nlm.nih.gov/gene/?term=16401
Nr4a3	Nr4a3	nuclear receptor subfamily 4, group A, member 3	18124	https://www.ncbi.nlm.nih.gov/gene/?term=18124
Pan2	Pan2	PAN2 poly(A) specific ribonuclease subunit	103135	https://www.ncbi.nlm.nih.gov/gene/?term=103135
Chsy3	Chsy3	chondroitin sulfate synthase 3	78923	https://www.ncbi.nlm.nih.gov/gene/?term=78923
Gtf2a1	Gtf2a1	general transcription factor II A, 1	83602	https://www.ncbi.nlm.nih.gov/gene/?term=83602
Pole4	Pole4	polymerase (DNA-directed), epsilon 4 (p12 subunit)	66979	https://www.ncbi.nlm.nih.gov/gene/?term=66979
Zfp287	Zfp287	zinc finger protein 287	170740	https://www.ncbi.nlm.nih.gov/gene/?term=170740
Lzts3	Lzts3	leucine zipper, putative tumor suppressor family member 3	241638	https://www.ncbi.nlm.nih.gov/gene/?term=241638
Ints10	Ints10	integrator complex subunit 10	70885	https://www.ncbi.nlm.nih.gov/gene/?term=70885
Gkn1	Gkn1	gastrokine 1	66283	https://www.ncbi.nlm.nih.gov/gene/?term=66283
Wdr47	Wdr47	WD repeat domain 47	99512	https://www.ncbi.nlm.nih.gov/gene/?term=99512

Biology Open • Supplementary information

Clasp2	Clasp2	CLIP associating protein 2	76499	https://www.ncbi.nlm.nih.gov/gene/?term=76499
Ddit4	Ddit4	DNA-damage-inducible transcript 4	74747	https://www.ncbi.nlm.nih.gov/gene/?term=74747
Mknk1	Mknk1	MAP kinase-interacting serine/threonine kinase 1	17346	https://www.ncbi.nlm.nih.gov/gene/?term=17346
Pfn2	Pfn2	profilin 2	18645	https://www.ncbi.nlm.nih.gov/gene/?term=18645
Arid4a	Arid4a	AT rich interactive domain 4A (RBP1-like)	238247	https://www.ncbi.nlm.nih.gov/gene/?term=238247
Chd3	Chd3	chromodomain helicase DNA binding protein 3	216848	https://www.ncbi.nlm.nih.gov/gene/?term=216848
Pilra	Pilra	paired immunoglobin-like type 2 receptor alpha	231805	https://www.ncbi.nlm.nih.gov/gene/?term=231805
Vdac3	Vdac3	voltage-dependent anion channel 3	22335	https://www.ncbi.nlm.nih.gov/gene/?term=22335
Wipf2	Wipf2	WAS/WASL interacting protein family, member 2	68524	https://www.ncbi.nlm.nih.gov/gene/?term=68524
Ckap4	Ckap4	cytoskeleton-associated protein 4	216197	https://www.ncbi.nlm.nih.gov/gene/?term=216197
Oxr1	Oxr1	oxidation resistance 1	170719	https://www.ncbi.nlm.nih.gov/gene/?term=170719
Slc17a1	Slc17a1	solute carrier family 17 (sodium phosphate), member 1	20504	https://www.ncbi.nlm.nih.gov/gene/?term=20504
Atp2b2	Atp2b2	ATPase, Ca++ transporting, plasma membrane 2	11941	https://www.ncbi.nlm.nih.gov/gene/?term=11941
Cct4	Cct4	chaperonin containing Tcp1, subunit 4 (delta)	12464	https://www.ncbi.nlm.nih.gov/gene/?term=12464
Gmeb1	Gmeb1	glucocorticoid modulatory element binding protein 1	56809	https://www.ncbi.nlm.nih.gov/gene/?term=56809
Sh3glb1	Sh3glb1	SH3-domain GRB2-like B1 (endophilin)	54673	https://www.ncbi.nlm.nih.gov/gene/?term=54673

Biology Open • Supplementary information

Ptgfrn	Ptgfrn	prostaglandin F2 receptor negative regulator	19221	https://www.ncbi.nlm.nih.gov/gene/?term=19221
Parp1	Parp1	poly (ADP-ribose) polymerase family, member 1	11545	https://www.ncbi.nlm.nih.gov/gene/?term=11545
Kdm3b	Kdm3b	KDM3B lysine (K)-specific demethylase 3B	277250	https://www.ncbi.nlm.nih.gov/gene/?term=277250
Hhip	Hhip	Hedgehog-interacting protein	15245	https://www.ncbi.nlm.nih.gov/gene/?term=15245
Rab5if	Rab5if	RAB5 interacting factor	67388	https://www.ncbi.nlm.nih.gov/gene/?term=67388
Bloc1s4	Bloc1s4	biogenesis of lysosomal organelles complex-1, subunit 4, cappuccino	117197	https://www.ncbi.nlm.nih.gov/gene/?term=117197
Ide	Ide	insulin degrading enzyme	15925	https://www.ncbi.nlm.nih.gov/gene/?term=15925
Rsbn1l	Rsbn1l	round spermatid basic protein 1-like	242860	https://www.ncbi.nlm.nih.gov/gene/?term=242860
Casp9	Casp9	caspase 9	12371	https://www.ncbi.nlm.nih.gov/gene/?term=12371
Nr1h2	Nr1h2	nuclear receptor subfamily 1, group H, member 2	22260	https://www.ncbi.nlm.nih.gov/gene/?term=22260
Zfp248	Zfp248	zinc finger protein 248	72720	https://www.ncbi.nlm.nih.gov/gene/?term=72720
Nrep	Nrep	neuronal regeneration related protein	27528	https://www.ncbi.nlm.nih.gov/gene/?term=27528
Spata2	Spata2	spermatogenesis associated 2	263876	https://www.ncbi.nlm.nih.gov/gene/?term=263876
Cdon	Cdon	cell adhesion molecule-related/down-regulated by oncogenes	57810	https://www.ncbi.nlm.nih.gov/gene/?term=57810
Adcy9	Adcy9	adenylate cyclase 9	11515	https://www.ncbi.nlm.nih.gov/gene/?term=11515
Rgs7bp	Rgs7bp	regulator of G-protein signalling 7 binding protein	52882	https://www.ncbi.nlm.nih.gov/gene/?term=52882
Smyd5	Smyd5	SET and MYND domain containing 5	232187	https://www.ncbi.nlm.nih.gov/gene/?term=232187
Herpud2	Herpud2	HERPUD family member 2	80517	https://www.ncbi.nlm.nih.gov/gene/?term=80517

Plxna1	Plxna1	plexin A1	18844	https://www.ncbi.nlm.nih.gov/gene/?term=18844
Atf7	Atf7	activating transcription factor 7	223922	https://www.ncbi.nlm.nih.gov/gene/?term=223922
Pom121l2	Pom121l2	POM121 membrane glycoprotein-like 2 (rat)	195236	https://www.ncbi.nlm.nih.gov/gene/?term=195236
Smim12	Smim12	small integral membrane protein 12	80284	https://www.ncbi.nlm.nih.gov/gene/?term=80284
Ssbp2	Ssbp2	single-stranded DNA binding protein 2	66970	https://www.ncbi.nlm.nih.gov/gene/?term=66970
Raf1	Raf1	v-raf-leukemia viral oncogene 1	110157	https://www.ncbi.nlm.nih.gov/gene/?term=110157
Ctsb	Ctsb	cathepsin B	13030	https://www.ncbi.nlm.nih.gov/gene/?term=13030
Champ1	Champ1	chromosome alignment maintaining phosphoprotein 1	101994	https://www.ncbi.nlm.nih.gov/gene/?term=101994
Itch	Itch	itchy, E3 ubiquitin protein ligase	16396	https://www.ncbi.nlm.nih.gov/gene/?term=16396
Gal3st3	Gal3st3	galactose-3-O-sulfotransferase 3	545276	https://www.ncbi.nlm.nih.gov/gene/?term=545276
Asxl1	Asxl1	additional sex combs like 1	228790	https://www.ncbi.nlm.nih.gov/gene/?term=228790
Sp1	Sp1	trans-acting transcription factor 1	20683	https://www.ncbi.nlm.nih.gov/gene/?term=20683
Prkcb	Prkcb	protein kinase C, beta	18751	https://www.ncbi.nlm.nih.gov/gene/?term=18751
Ezh1	Ezh1	enhancer of zeste 1 polycomb repressive complex 2 subunit	14055	https://www.ncbi.nlm.nih.gov/gene/?term=14055
Rnf141	Rnf141	ring finger protein 141	67150	https://www.ncbi.nlm.nih.gov/gene/?term=67150
Atp10a	Atp10a	ATPase, class V, type 10A	11982	https://www.ncbi.nlm.nih.gov/gene/?term=11982
Fam168b	Fam168b	family with sequence similarity 168, member B	214469	https://www.ncbi.nlm.nih.gov/gene/?term=214469
Pcnx2	Pcnx2	pecanex homolog 2	270109	https://www.ncbi.nlm.nih.gov/gene/?term=270109
Zc3h4	Zc3h4	zinc finger CCCH-type containing 4	330474	https://www.ncbi.nlm.nih.gov/gene/?term=330474
Mapk4	Mapk4	mitogen-activated protein kinase 4	225724	https://www.ncbi.nlm.nih.gov/gene/?term=225724
Pde4a	Pde4a	phosphodiesterase 4A, cAMP specific	18577	https://www.ncbi.nlm.nih.gov/gene/?term=18577

Zbtb4	Zbtb4	zinc finger and BTB domain containing 4	75580	https://www.ncbi.nlm.nih.gov/gene/?term=75580
Fndc4	Fndc4	fibronectin type III domain containing 4	64339	https://www.ncbi.nlm.nih.gov/gene/?term=64339
Klf4	Klf4	Kruppel-like factor 4 (gut)	16600	https://www.ncbi.nlm.nih.gov/gene/?term=16600
Edar	Edar	ectodysplasin-A receptor	13608	https://www.ncbi.nlm.nih.gov/gene/?term=13608
Strn3	Strn3	striatin, calmodulin binding protein 3	94186	https://www.ncbi.nlm.nih.gov/gene/?term=94186
Xpo7	Xpo7	exportin 7	65246	https://www.ncbi.nlm.nih.gov/gene/?term=65246
B3gnt6	B3gnt6	UDP-GlcNAc:betaGal beta-1,3-N-acetylglucosaminyltransferase 6 (core 3 synthase)	272411	https://www.ncbi.nlm.nih.gov/gene/?term=272411
Bcorl1	Bcorl1	BCL6 co-repressor-like 1	320376	https://www.ncbi.nlm.nih.gov/gene/?term=320376
Ipo11	Ipo11	importin 11	76582	https://www.ncbi.nlm.nih.gov/gene/?term=76582
Dkk3	Dkk3	dickkopf WNT signaling pathway inhibitor 3	50781	https://www.ncbi.nlm.nih.gov/gene/?term=50781
Hdlbp	Hdlbp	high density lipoprotein (HDL) binding protein	110611	https://www.ncbi.nlm.nih.gov/gene/?term=110611
Rsbn1	Rsbn1	rosbin, round spermatid basic protein 1	229675	https://www.ncbi.nlm.nih.gov/gene/?term=229675
Trio	Trio	triple functional domain (PTPRF interacting)	223435	https://www.ncbi.nlm.nih.gov/gene/?term=223435
Plin5	Plin5	perilipin 5	66968	https://www.ncbi.nlm.nih.gov/gene/?term=66968
Ago1	Ago1	argonaute RISC catalytic subunit 1	236511	https://www.ncbi.nlm.nih.gov/gene/?term=236511
Krt6b	Krt6b	keratin 6B	16688	https://www.ncbi.nlm.nih.gov/gene/?term=16688
Tmem167	Tmem167	transmembrane protein 167	66074	https://www.ncbi.nlm.nih.gov/gene/?term=66074
H2-M10.5	H2-M10.5	histocompatibility 2, M region locus 10.5	224761	https://www.ncbi.nlm.nih.gov/gene/?term=224761

Biology Open • Supplementary information

Cfb	Cfb	complement factor B	14962	https://www.ncbi.nlm.nih.gov/gene/?term=14962
Syt11	Syt11	synaptotagmin XI	229521	https://www.ncbi.nlm.nih.gov/gene/?term=229521
Zfp446	Zfp446	zinc finger protein 446	269870	https://www.ncbi.nlm.nih.gov/gene/?term=269870
Nup98	Nup98	nucleoporin 98	269966	https://www.ncbi.nlm.nih.gov/gene/?term=269966
H2-M10.3	H2-M10.3	histocompatibility 2, M region locus 10.3	110696	https://www.ncbi.nlm.nih.gov/gene/?term=110696
Cd200r3	Cd200r3	CD200 receptor 3	74603	https://www.ncbi.nlm.nih.gov/gene/?term=74603
Gpr22	Gpr22	G protein-coupled receptor 22	73010	https://www.ncbi.nlm.nih.gov/gene/?term=73010
Wnt9a	Wnt9a	wingless-type MMTV integration site family, member 9A	216795	https://www.ncbi.nlm.nih.gov/gene/?term=216795
Uqcrb	Uqcrb	ubiquinol-cytochrome c reductase binding protein	67530	https://www.ncbi.nlm.nih.gov/gene/?term=67530
Lrrc75b	Lrrc75b	leucine rich repeat containing 75B	192734	https://www.ncbi.nlm.nih.gov/gene/?term=192734
Fign	Fign	fidgetin	60344	https://www.ncbi.nlm.nih.gov/gene/?term=60344
Acvr1c	Acvr1c	activin A receptor, type IC	269275	https://www.ncbi.nlm.nih.gov/gene/?term=269275
Fam78a	Fam78a	family with sequence similarity 78, member A	241303	https://www.ncbi.nlm.nih.gov/gene/?term=241303
Sh3gl2	Sh3gl2	SH3-domain GRB2-like 2	20404	https://www.ncbi.nlm.nih.gov/gene/?term=20404
Il2rg	Il2rg	interleukin 2 receptor, gamma chain	16186	https://www.ncbi.nlm.nih.gov/gene/?term=16186
Ptchd4	Ptchd4	patched domain containing 4	627626	https://www.ncbi.nlm.nih.gov/gene/?term=627626
Rufy3	Rufy3	RUN and FYVE domain containing 3	52822	https://www.ncbi.nlm.nih.gov/gene/?term=52822
Ecm2	Ecm2	extracellular matrix protein 2, female organ and adipocyte specific	407800	https://www.ncbi.nlm.nih.gov/gene/?term=407800
Slc9a6	Slc9a6	solute carrier family 9 (sodium/hydrogen exchanger), member 6	236794	https://www.ncbi.nlm.nih.gov/gene/?term=236794

Biology Open • Supplementary information

Klhl31	Klhl31	kelch-like 31	244923	https://www.ncbi.nlm.nih.gov/gene/?term=244923
Hoxb7	Hoxb7	homeobox B7	15415	https://www.ncbi.nlm.nih.gov/gene/?term=15415
Zfyve26	Zfyve26	zinc finger, FYVE domain containing 26	211978	https://www.ncbi.nlm.nih.gov/gene/?term=211978
Cyp2b23	Cyp2b23	cytochrome P450, family 2, subfamily b, polypeptide 23	243881	https://www.ncbi.nlm.nih.gov/gene/?term=243881
Hapln1	Hapln1	hyaluronan and proteoglycan link protein 1	12950	https://www.ncbi.nlm.nih.gov/gene/?term=12950
Hand1	Hand1	heart and neural crest derivatives expressed 1	15110	https://www.ncbi.nlm.nih.gov/gene/?term=15110
Zfp512b	Zfp512b	zinc finger protein 512B	269401	https://www.ncbi.nlm.nih.gov/gene/?term=269401
Stk40	Stk40	serine/threonine kinase 40	74178	https://www.ncbi.nlm.nih.gov/gene/?term=74178
Utrn	Utrn	utrophin	22288	https://www.ncbi.nlm.nih.gov/gene/?term=22288
Adamts8	Adamts8	a disintegrin-like and metallopeptidase (reprolysin type) with thrombospondin type 1 motif, 8	30806	https://www.ncbi.nlm.nih.gov/gene/?term=30806
Gata6	Gata6	GATA binding protein 6	14465	https://www.ncbi.nlm.nih.gov/gene/?term=14465
Ntrk2	Ntrk2	neurotrophic tyrosine kinase, receptor, type 2	18212	https://www.ncbi.nlm.nih.gov/gene/?term=18212
Tsc22d3	Tsc22d3	TSC22 domain family, member 3	14605	https://www.ncbi.nlm.nih.gov/gene/?term=14605
Pbx1	Pbx1	pre B cell leukemia homeobox 1	18514	https://www.ncbi.nlm.nih.gov/gene/?term=18514
Rnf138	Rnf138	ring finger protein 138	56515	https://www.ncbi.nlm.nih.gov/gene/?term=56515
Mcoln1	Mcoln1	mucolipin 1	94178	https://www.ncbi.nlm.nih.gov/gene/?term=94178
Ucp1	Ucp1	uncoupling protein 1 (mitochondrial, proton carrier)	22227	https://www.ncbi.nlm.nih.gov/gene/?term=22227
Pde1c	Pde1c	phosphodiesterase 1C	18575	https://www.ncbi.nlm.nih.gov/gene/?term=18575

Biology Open • Supplementary information

Dyrk1a	Dyrk1a	dual-specificity tyrosine-(Y)-phosphorylation regulated kinase 1a	13548	https://www.ncbi.nlm.nih.gov/gene/?term=13548
Hyal4	Hyal4	hyaluronoglucosaminidase 4	77042	https://www.ncbi.nlm.nih.gov/gene/?term=77042
Glis3	Glis3	GLIS family zinc finger 3	226075	https://www.ncbi.nlm.nih.gov/gene/?term=226075
Slc43a1	Slc43a1	solute carrier family 43, member 1	72401	https://www.ncbi.nlm.nih.gov/gene/?term=72401
Pdp1	Pdp1	pyruvate dehyrogenase phosphatase catalytic subunit 1	381511	https://www.ncbi.nlm.nih.gov/gene/?term=381511
Tnfaip8	Tnfaip8	tumor necrosis factor, alpha-induced protein 8	106869	https://www.ncbi.nlm.nih.gov/gene/?term=106869
Efemp2	Efemp2	epidermal growth factor-containing fibulin-like extracellular matrix protein 2	58859	https://www.ncbi.nlm.nih.gov/gene/?term=58859
Nrg3	Nrg3	neuregulin 3	18183	https://www.ncbi.nlm.nih.gov/gene/?term=18183
Creb5	Creb5	cAMP responsive element binding protein 5	231991	https://www.ncbi.nlm.nih.gov/gene/?term=231991
St7	St7	suppression of tumorigenicity 7	64213	https://www.ncbi.nlm.nih.gov/gene/?term=64213
Tgfbr2	Tgfbr2	transforming growth factor, beta receptor II	21813	https://www.ncbi.nlm.nih.gov/gene/?term=21813
Onecut2	Onecut2	one cut domain, family member 2	225631	https://www.ncbi.nlm.nih.gov/gene/?term=225631
Napg	Napg	N-ethylmaleimide sensitive fusion protein attachment protein gamma	108123	https://www.ncbi.nlm.nih.gov/gene/?term=108123
Trim12c	Trim12c	tripartite motif-containing 12C	319236	https://www.ncbi.nlm.nih.gov/gene/?term=319236
P2rx7	P2rx7	purinergic receptor P2X, ligand-gated ion channel, 7	18439	https://www.ncbi.nlm.nih.gov/gene/?term=18439
Ssrp1	Ssrp1	structure specific recognition protein 1	20833	https://www.ncbi.nlm.nih.gov/gene/?term=20833
Nbea	Nbea	neurobeachin	26422	https://www.ncbi.nlm.nih.gov/gene/?term=26422

Biology Open • Supplementary information

Lin7a	Lin7a	lin-7 homolog A (C. elegans)	108030	https://www.ncbi.nlm.nih.gov/gene/?term=108030
Zcchc24	Zcchc24	zinc finger, CCHC domain containing 24	71918	https://www.ncbi.nlm.nih.gov/gene/?term=71918
Gcnt2	Gcnt2	glucosaminyl (N-acetyl) transferase 2, I-branching enzyme	14538	https://www.ncbi.nlm.nih.gov/gene/?term=14538
Ap2a2	Ap2a2	adaptor-related protein complex 2, alpha 2 subunit	11772	https://www.ncbi.nlm.nih.gov/gene/?term=11772
Lgals3	Lgals3	lectin, galactose binding, soluble 3	16854	https://www.ncbi.nlm.nih.gov/gene/?term=16854
Adamts5	Adamts5	a disintegrin-like and metallopeptidase (reprolysin type) with thrombospondin type 1 motif, 5 (aggrecanase-2)	23794	https://www.ncbi.nlm.nih.gov/gene/?term=23794
Trp53inp1	Trp53inp1	transformation related protein 53 inducible nuclear protein 1	60599	https://www.ncbi.nlm.nih.gov/gene/?term=60599
Dnajc14	Dnajc14	DnaJ heat shock protein family (Hsp40) member C14	74330	https://www.ncbi.nlm.nih.gov/gene/?term=74330
Crtac1	Crtac1	cartilage acidic protein 1	72832	https://www.ncbi.nlm.nih.gov/gene/?term=72832
Gpatch11	Gpatch11	G patch domain containing 11	53951	https://www.ncbi.nlm.nih.gov/gene/?term=53951
Ccdc117	Ccdc117	coiled-coil domain containing 117	104479	https://www.ncbi.nlm.nih.gov/gene/?term=104479
Dmtf1	Dmtf1	cyclin D binding myb-like transcription factor 1	23857	https://www.ncbi.nlm.nih.gov/gene/?term=23857
Prrx1	Prrx1	paired related homeobox 1	18933	https://www.ncbi.nlm.nih.gov/gene/?term=18933
Mgat3	Mgat3	mannoside acetylglucosaminyltransferase 3	17309	https://www.ncbi.nlm.nih.gov/gene/?term=17309
Zfhx3	Zfhx3	zinc finger homeobox 3	11906	https://www.ncbi.nlm.nih.gov/gene/?term=11906
Ncoa7	Ncoa7	nuclear receptor coactivator 7	211329	https://www.ncbi.nlm.nih.gov/gene/?term=211329
Cox5a	Cox5a	cytochrome c oxidase subunit 5A	12858	https://www.ncbi.nlm.nih.gov/gene/?term=12858
Chp1	Chp1	calcineurin-like EF hand protein 1	56398	https://www.ncbi.nlm.nih.gov/gene/?term=56398

Gba	Gba	glucosidase, beta, acid	14466	https://www.ncbi.nlm.nih.gov/gene/?term=14466
Ttyh1	Ttyh1	tweety family member 1	57776	https://www.ncbi.nlm.nih.gov/gene/?term=57776
Rnf170	Rnf170	ring finger protein 170	77733	https://www.ncbi.nlm.nih.gov/gene/?term=77733
Sos1	Sos1	SOS Ras/Rac guanine nucleotide exchange factor 1	20662	https://www.ncbi.nlm.nih.gov/gene/?term=20662
Pid1	Pid1	phosphotyrosine interaction domain containing 1	98496	https://www.ncbi.nlm.nih.gov/gene/?term=98496
Ppargc1a	Ppargc1a	peroxisome proliferative activated receptor, gamma, coactivator 1 alpha	19017	https://www.ncbi.nlm.nih.gov/gene/?term=19017
Lamp1	Lamp1	lysosomal-associated membrane protein 1	16783	https://www.ncbi.nlm.nih.gov/gene/?term=16783
Spred1	Spred1	sprouty protein with EVH-1 domain 1, related sequence	114715	https://www.ncbi.nlm.nih.gov/gene/?term=114715
4930402H24Rik	4930402H24Rik	RIKEN cDNA 4930402H24 gene	228602	https://www.ncbi.nlm.nih.gov/gene/?term=228602
Dtwd1	Dtwd1	DTW domain containing 1	69185	https://www.ncbi.nlm.nih.gov/gene/?term=69185
Tox3	Tox3	TOX high mobility group box family member 3	244579	https://www.ncbi.nlm.nih.gov/gene/?term=244579
Spop	Spop	speckle-type POZ protein	20747	https://www.ncbi.nlm.nih.gov/gene/?term=20747
Fras1	Fras1	Fraser extracellular matrix complex subunit 1	231470	https://www.ncbi.nlm.nih.gov/gene/?term=231470
Slc38a9	Slc38a9	solute carrier family 38, member 9	268706	https://www.ncbi.nlm.nih.gov/gene/?term=268706
Sirt1	Sirt1	sirtuin 1	93759	https://www.ncbi.nlm.nih.gov/gene/?term=93759
Dppa2	Dppa2	developmental pluripotency associated 2	73703	https://www.ncbi.nlm.nih.gov/gene/?term=73703
Abcb10	Abcb10	ATP-binding cassette, sub-family B (MDR/TAP), member 10	56199	https://www.ncbi.nlm.nih.gov/gene/?term=56199
Abraxas2	Abraxas2	BRISC complex subunit	109359	https://www.ncbi.nlm.nih.gov/gene/?term=109359

Wdr46	Wdr46	WD repeat domain 46	57315	https://www.ncbi.nlm.nih.gov/gene/?term=57315
Rhobtb3	Rhobtb3	Rho-related BTB domain containing 3	73296	https://www.ncbi.nlm.nih.gov/gene/?term=73296
Lsm5	Lsm5	LSM5 homolog, U6 small nuclear RNA and mRNA degradation associated	66373	https://www.ncbi.nlm.nih.gov/gene/?term=66373
Nr3c2	Nr3c2	nuclear receptor subfamily 3, group C, member 2	110784	https://www.ncbi.nlm.nih.gov/gene/?term=110784
Pcgf3	Pcgf3	polycomb group ring finger 3	69587	https://www.ncbi.nlm.nih.gov/gene/?term=69587
Zfp423	Zfp423	zinc finger protein 423	94187	https://www.ncbi.nlm.nih.gov/gene/?term=94187
Gpbp1l1	Gpbp1l1	GC-rich promoter binding protein 1-like 1	77110	https://www.ncbi.nlm.nih.gov/gene/?term=77110
Arhgap11a	Arhgap11a	Rho GTPase activating protein 11A	228482	https://www.ncbi.nlm.nih.gov/gene/?term=228482
Grk4	Grk4	G protein-coupled receptor kinase 4	14772	https://www.ncbi.nlm.nih.gov/gene/?term=14772
Nrbf2	Nrbf2	nuclear receptor binding factor 2	641340	https://www.ncbi.nlm.nih.gov/gene/?term=641340
Fbn2	Fbn2	fibrillin 2	14119	https://www.ncbi.nlm.nih.gov/gene/?term=14119
Dpy19l4	Dpy19l4	dpy-19-like 4 (C. elegans)	381510	https://www.ncbi.nlm.nih.gov/gene/?term=381510
Rell1	Rell1	RELT-like 1	100532	https://www.ncbi.nlm.nih.gov/gene/?term=100532
Plcb1	Plcb1	phospholipase C, beta 1	18795	https://www.ncbi.nlm.nih.gov/gene/?term=18795
Rab22a	Rab22a	RAB22A, member RAS oncogene family	19334	https://www.ncbi.nlm.nih.gov/gene/?term=19334
Camk1	Camk1	calcium/calmodulin-dependent protein kinase I	52163	https://www.ncbi.nlm.nih.gov/gene/?term=52163
Mapre2	Mapre2	microtubule-associated protein, RP/EB family, member 2	212307	https://www.ncbi.nlm.nih.gov/gene/?term=212307
Slc39a11	Slc39a11	solute carrier family 39 (metal ion transporter), member 11	69806	https://www.ncbi.nlm.nih.gov/gene/?term=69806

Biology Open • Supplementary information

Larp4b	Larp4b	La ribonucleoprotein domain family, member 4B	217980	https://www.ncbi.nlm.nih.gov/gene/?term=217980
Dera	Dera	deoxyribose-phosphate aldolase (putative)	232449	https://www.ncbi.nlm.nih.gov/gene/?term=232449
Jarid2	Jarid2	jumonji, AT rich interactive domain 2	16468	https://www.ncbi.nlm.nih.gov/gene/?term=16468
Shc1	Shc1	src homology 2 domain-containing transforming protein C1	20416	https://www.ncbi.nlm.nih.gov/gene/?term=20416
Clmp	Clmp	CXADR-like membrane protein	71566	https://www.ncbi.nlm.nih.gov/gene/?term=71566
Fry	Fry	FRY microtubule binding protein	320365	https://www.ncbi.nlm.nih.gov/gene/?term=320365
Itpr1	Itpr1	inositol 1,4,5-trisphosphate receptor 1	16438	https://www.ncbi.nlm.nih.gov/gene/?term=16438
Ssr3	Ssr3	signal sequence receptor, gamma	67437	https://www.ncbi.nlm.nih.gov/gene/?term=67437
Dnm2	Dnm2	dynamin 2	13430	https://www.ncbi.nlm.nih.gov/gene/?term=13430
Ptprd	Ptprd	protein tyrosine phosphatase, receptor type, D	19266	https://www.ncbi.nlm.nih.gov/gene/?term=19266
Pde12	Pde12	phosphodiesterase 12	211948	https://www.ncbi.nlm.nih.gov/gene/?term=211948
Mlxip	Mlxip	MLX interacting protein	208104	https://www.ncbi.nlm.nih.gov/gene/?term=208104
Smim13	Smim13	small integral membrane protein 13	108934	https://www.ncbi.nlm.nih.gov/gene/?term=108934
Ephb6	Ephb6	Eph receptor B6	13848	https://www.ncbi.nlm.nih.gov/gene/?term=13848
Epha4	Epha4	Eph receptor A4	13838	https://www.ncbi.nlm.nih.gov/gene/?term=13838
Arl8b	Arl8b	ADP-ribosylation factor-like 8B	67166	https://www.ncbi.nlm.nih.gov/gene/?term=67166
Hoxc8	Hoxc8	homeobox C8	15426	https://www.ncbi.nlm.nih.gov/gene/?term=15426
Ankfy1	Ankfy1	ankyrin repeat and FYVE domain containing 1	11736	https://www.ncbi.nlm.nih.gov/gene/?term=11736
Lrrn4cl	Lrrn4cl	LRRN4 C-terminal like	68852	https://www.ncbi.nlm.nih.gov/gene/?term=68852

Fam126a	Fam126a	family with sequence similarity 126, member A	84652	https://www.ncbi.nlm.nih.gov/gene/?term=84652
Arid3b	Arid3b	AT rich interactive domain 3B (BRIGHT-like)	56380	https://www.ncbi.nlm.nih.gov/gene/?term=56380
Zfp629	Zfp629	zinc finger protein 629	320683	https://www.ncbi.nlm.nih.gov/gene/?term=320683
Tmem164	Tmem164	transmembrane protein 164	209497	https://www.ncbi.nlm.nih.gov/gene/?term=209497
Fam149a	Fam149a	family with sequence similarity 149, member A	212326	https://www.ncbi.nlm.nih.gov/gene/?term=212326
Rere	Rere	arginine glutamic acid dipeptide (RE) repeats	68703	https://www.ncbi.nlm.nih.gov/gene/?term=68703
Evc2	Evc2	EvC ciliary complex subunit 2	68525	https://www.ncbi.nlm.nih.gov/gene/?term=68525
Tmem263	Tmem263	transmembrane protein 263	103266	https://www.ncbi.nlm.nih.gov/gene/?term=103266
Elavl3	Elavl3	ELAV like RNA binding protein 3	15571	https://www.ncbi.nlm.nih.gov/gene/?term=15571
Pcdh12	Pcdh12	protocadherin 12	53601	https://www.ncbi.nlm.nih.gov/gene/?term=53601
Naa15	Naa15	N(alpha)-acetyltransferase 15, NatA auxiliary subunit	74838	https://www.ncbi.nlm.nih.gov/gene/?term=74838
Ankrd13a	Ankrd13a	ankyrin repeat domain 13a	68420	https://www.ncbi.nlm.nih.gov/gene/?term=68420
Mrps33	Mrps33	mitochondrial ribosomal protein S33	14548	https://www.ncbi.nlm.nih.gov/gene/?term=14548
Nanos1	Nanos1	nanos C2HC-type zinc finger 1	332397	https://www.ncbi.nlm.nih.gov/gene/?term=332397
Ccdc68	Ccdc68	coiled-coil domain containing 68	381175	https://www.ncbi.nlm.nih.gov/gene/?term=381175
Sox11	Sox11	SRY (sex determining region Y)-box 11	20666	https://www.ncbi.nlm.nih.gov/gene/?term=20666
Slc2a1	Slc2a1	solute carrier family 2 (facilitated glucose transporter), member 1	20525	https://www.ncbi.nlm.nih.gov/gene/?term=20525
Thy1	Thy1	thymus cell antigen 1, theta	21838	https://www.ncbi.nlm.nih.gov/gene/?term=21838
Sgk1	Sgk1	serum/glucocorticoid regulated kinase 1	20393	https://www.ncbi.nlm.nih.gov/gene/?term=20393

Biology Open • Supplementary information

Kdm7a	Kdm7a	lysine (K)-specific demethylase 7A	338523	https://www.ncbi.nlm.nih.gov/gene/?term=338523
Tfrc	Tfrc	transferrin receptor	22042	https://www.ncbi.nlm.nih.gov/gene/?term=22042
Fastkd2	Fastkd2	FAST kinase domains 2	75619	https://www.ncbi.nlm.nih.gov/gene/?term=75619
Arpc1a	Arpc1a	actin related protein 2/3 complex, subunit 1A	56443	https://www.ncbi.nlm.nih.gov/gene/?term=56443
Zfp106	Zfp106	zinc finger protein 106	20402	https://www.ncbi.nlm.nih.gov/gene/?term=20402
Pigk	Pigk	phosphatidylinositol glycan anchor biosynthesis, class K	329777	https://www.ncbi.nlm.nih.gov/gene/?term=329777
Nedd9	Nedd9	neural precursor cell expressed, developmentally down-regulated gene 9	18003	https://www.ncbi.nlm.nih.gov/gene/?term=18003
Zfp850	Zfp850	zinc finger protein 850	100043772	https://www.ncbi.nlm.nih.gov/gene/?term=100043
Rd3	Rd3	retinal degeneration 3	74023	https://www.ncbi.nlm.nih.gov/gene/?term=74023
Hmbs	Hmbs	hydroxymethylbilane synthase	15288	https://www.ncbi.nlm.nih.gov/gene/?term=15288
Mctp1	Mctp1	multiple C2 domains, transmembrane 1	78771	https://www.ncbi.nlm.nih.gov/gene/?term=78771
Ddx19b	Ddx19b	DEAD (Asp-Glu-Ala-Asp) box polypeptide 19b	234733	https://www.ncbi.nlm.nih.gov/gene/?term=234733
Naa50	Naa50	N(alpha)-acetyltransferase 50, NatE catalytic subunit	72117	https://www.ncbi.nlm.nih.gov/gene/?term=72117
Epdr1	Epdr1	ependymin related protein 1 (zebrafish)	105298	https://www.ncbi.nlm.nih.gov/gene/?term=105298
Slc36a4	Slc36a4	solute carrier family 36 (proton/amino acid symporter), member 4	234967	https://www.ncbi.nlm.nih.gov/gene/?term=234967
Cdr1	Cdr1	cerebellar degeneration related antigen 1	631990	https://www.ncbi.nlm.nih.gov/gene/?term=631990
Fbxo11	Fbxo11	F-box protein 11	225055	https://www.ncbi.nlm.nih.gov/gene/?term=225055

Biology Open • Supplementary information

4932429P05Rik	4932429P05Rik	RIKEN cDNA 4932429P05 gene	245509	https://www.ncbi.nlm.nih.gov/gene/?term=245509
Cdkn1b	Cdkn1b	cyclin-dependent kinase inhibitor 1B	12576	https://www.ncbi.nlm.nih.gov/gene/?term=12576
Trib2	Trib2	tribbles pseudokinase 2	217410	https://www.ncbi.nlm.nih.gov/gene/?term=217410
Itprid2	Itprid2	ITPR interacting domain containing 2	70599	https://www.ncbi.nlm.nih.gov/gene/?term=70599
Fam135a	Fam135a	family with sequence similarity 135, member A	68187	https://www.ncbi.nlm.nih.gov/gene/?term=68187
Slc17a6	Slc17a6	solute carrier family 17 (sodium-dependent inorganic phosphate cotransporter), member 6	140919	https://www.ncbi.nlm.nih.gov/gene/?term=140919
Zfp292	Zfp292	zinc finger protein 292	30046	https://www.ncbi.nlm.nih.gov/gene/?term=30046
Celf4	Celf4	CUGBP, Elav-like family member 4	108013	https://www.ncbi.nlm.nih.gov/gene/?term=108013
Dpep1	Dpep1	dipeptidase 1	13479	https://www.ncbi.nlm.nih.gov/gene/?term=13479
Trim36	Trim36	tripartite motif-containing 36	28105	https://www.ncbi.nlm.nih.gov/gene/?term=28105
Trps1	Trps1	transcriptional repressor GATA binding 1	83925	https://www.ncbi.nlm.nih.gov/gene/?term=83925
Cebpa	Cebpa	CCAAT/enhancer binding protein (C/EBP), alpha	12606	https://www.ncbi.nlm.nih.gov/gene/?term=12606
Slc20a2	Slc20a2	solute carrier family 20, member 2	20516	https://www.ncbi.nlm.nih.gov/gene/?term=20516
Nlgn1	Nlgn1	neuroligin 1	192167	https://www.ncbi.nlm.nih.gov/gene/?term=192167
Serpinb9e	Serpinb9e	serine (or cysteine) peptidase inhibitor, clade B, member 9e	20710	https://www.ncbi.nlm.nih.gov/gene/?term=20710
Alkbh8	Alkbh8	alkB homolog 8, tRNA methyltransferase	67667	https://www.ncbi.nlm.nih.gov/gene/?term=67667
Tor1aip1	Tor1aip1	torsin A interacting protein 1	208263	https://www.ncbi.nlm.nih.gov/gene/?term=208263
Wsb1	Wsb1	WD repeat and SOCS box-containing 1	78889	https://www.ncbi.nlm.nih.gov/gene/?term=78889

Eda2r	Eda2r	ectodysplasin A2 receptor	245527	https://www.ncbi.nlm.nih.gov/gene/?term=245527
Serpinb9f	Serpinb9f	serine (or cysteine) peptidase inhibitor, clade B, member 9f	20709	https://www.ncbi.nlm.nih.gov/gene/?term=20709
Chd7	Chd7	chromodomain helicase DNA binding protein 7	320790	https://www.ncbi.nlm.nih.gov/gene/?term=320790
Epc2	Epc2	enhancer of polycomb homolog 2	227867	https://www.ncbi.nlm.nih.gov/gene/?term=227867
Zfp322a	Zfp322a	zinc finger protein 322A	218100	https://www.ncbi.nlm.nih.gov/gene/?term=218100
Setdb2	Setdb2	SET domain, bifurcated 2	239122	https://www.ncbi.nlm.nih.gov/gene/?term=239122
Mr1	Mr1	major histocompatibility complex, class I-related	15064	https://www.ncbi.nlm.nih.gov/gene/?term=15064
Tdrd5	Tdrd5	tudor domain containing 5	214575	https://www.ncbi.nlm.nih.gov/gene/?term=214575
Pigw	Pigw	phosphatidylinositol glycan anchor biosynthesis, class W	70325	https://www.ncbi.nlm.nih.gov/gene/?term=70325
Neurod1	Neurod1	neurogenic differentiation 1	18012	https://www.ncbi.nlm.nih.gov/gene/?term=18012
Setbp1	Setbp1	SET binding protein 1	240427	https://www.ncbi.nlm.nih.gov/gene/?term=240427
Smpdl3b	Smpdl3b	sphingomyelin phosphodiesterase, acid-like 3B	100340	https://www.ncbi.nlm.nih.gov/gene/?term=100340
Myo5a	Myo5a	myosin VA	17918	https://www.ncbi.nlm.nih.gov/gene/?term=17918
Aak1	Aak1	AP2 associated kinase 1	269774	https://www.ncbi.nlm.nih.gov/gene/?term=269774
Tigd2	Tigd2	tigger transposable element derived 2	68140	https://www.ncbi.nlm.nih.gov/gene/?term=68140
Dcaf10	Dcaf10	DDB1 and CUL4 associated factor 10	242418	https://www.ncbi.nlm.nih.gov/gene/?term=242418
Psg29	Psg29	pregnancy-specific glycoprotein 29	114872	https://www.ncbi.nlm.nih.gov/gene/?term=114872
Ktn1	Ktn1	kinectin 1	16709	https://www.ncbi.nlm.nih.gov/gene/?term=16709
Cpa3	Cpa3	carboxypeptidase A3, mast cell	12873	https://www.ncbi.nlm.nih.gov/gene/?term=12873
Ap3s1	Ap3s1	adaptor-related protein complex 3, sigma 1 subunit	11777	https://www.ncbi.nlm.nih.gov/gene/?term=11777
Usp36	Usp36	ubiquitin specific peptidase 36	72344	https://www.ncbi.nlm.nih.gov/gene/?term=72344

Biology Open • Supplementary information

Thrb	Thrb	thyroid hormone receptor beta	21834	https://www.ncbi.nlm.nih.gov/gene/?term=21834
Nppc	Nppc	natriuretic peptide type C	18159	https://www.ncbi.nlm.nih.gov/gene/?term=18159
Mtf2	Mtf2	metal response element binding transcription factor 2	17765	https://www.ncbi.nlm.nih.gov/gene/?term=17765
Mief2	Mief2	mitochondrial elongation factor 2	237781	https://www.ncbi.nlm.nih.gov/gene/?term=237781
Ank1	Ank1	ankyrin 1, erythroid	11733	https://www.ncbi.nlm.nih.gov/gene/?term=11733
Fem1c	Fem1c	fem-1 homolog c (C.elegans)	240263	https://www.ncbi.nlm.nih.gov/gene/?term=240263
Epha8	Epha8	Eph receptor A8	13842	https://www.ncbi.nlm.nih.gov/gene/?term=13842
Upf2	Upf2	UPF2 regulator of nonsense transcripts homolog (yeast)	326622	https://www.ncbi.nlm.nih.gov/gene/?term=326622
Fabp9	Fabp9	fatty acid binding protein 9, testis	21884	https://www.ncbi.nlm.nih.gov/gene/?term=21884
Chd6	Chd6	chromodomain helicase DNA binding protein 6	71389	https://www.ncbi.nlm.nih.gov/gene/?term=71389
St6galnac4	St6galnac4	ST6 (alpha-N-acetyl-neuraminyl-2,3-beta-galactosyl-1,3)-N-acetylgalactosaminide alpha-2,6-sialyltransferase 4	20448	https://www.ncbi.nlm.nih.gov/gene/?term=20448
Afap1	Afap1	actin filament associated protein 1	70292	https://www.ncbi.nlm.nih.gov/gene/?term=70292
Caln1	Caln1	calneuron 1	140904	https://www.ncbi.nlm.nih.gov/gene/?term=140904
Rara	Rara	retinoic acid receptor, alpha	19401	https://www.ncbi.nlm.nih.gov/gene/?term=19401
Psd	Psd	pleckstrin and Sec7 domain containing	73728	https://www.ncbi.nlm.nih.gov/gene/?term=73728
Plxnb2	Plxnb2	plexin B2	140570	https://www.ncbi.nlm.nih.gov/gene/?term=140570
Nkapd1	Nkapd1	NKAP domain containing 1	270156	https://www.ncbi.nlm.nih.gov/gene/?term=270156
Kmt5b	Kmt5b	lysine methyltransferase 5B	225888	https://www.ncbi.nlm.nih.gov/gene/?term=225888
Zfp148	Zfp148	zinc finger protein 148	22661	https://www.ncbi.nlm.nih.gov/gene/?term=22661
Col3a1	Col3a1	collagen, type III, alpha 1	12825	https://www.ncbi.nlm.nih.gov/gene/?term=12825

Biology Open • Supplementary information

Lsm14a	Lsm14a	LSM14A mRNA processing body assembly factor	67070	https://www.ncbi.nlm.nih.gov/gene/?term=67070
D17H6S53E	D17H6S53E	DNA segment, Chr 17, human D6S53E	114585	https://www.ncbi.nlm.nih.gov/gene/?term=114585
Unc5a	Unc5a	unc-5 netrin receptor A	107448	https://www.ncbi.nlm.nih.gov/gene/?term=107448
Lamc1	Lamc1	laminin, gamma 1	226519	https://www.ncbi.nlm.nih.gov/gene/?term=226519
Sorbs2	Sorbs2	sorbin and SH3 domain containing 2	234214	https://www.ncbi.nlm.nih.gov/gene/?term=234214
Scn8a	Scn8a	sodium channel, voltage-gated, type VIII, alpha	20273	https://www.ncbi.nlm.nih.gov/gene/?term=20273
Cnot6l	Cnot6l	CCR4-NOT transcription complex, subunit 6-like	231464	https://www.ncbi.nlm.nih.gov/gene/?term=231464
Klf11	Klf11	Kruppel-like factor 11	194655	https://www.ncbi.nlm.nih.gov/gene/?term=194655
Cped1	Cped1	cadherin-like and PC-esterase domain containing 1	214642	https://www.ncbi.nlm.nih.gov/gene/?term=214642
Clec1a	Clec1a	C-type lectin domain family 1, member a	243653	https://www.ncbi.nlm.nih.gov/gene/?term=243653
Nsmf	Nsmf	NMDA receptor synaptonuclear signaling and neuronal migration factor	56876	https://www.ncbi.nlm.nih.gov/gene/?term=56876
Sys1	Sys1	SYS1 Golgi-localized integral membrane protein homolog (S. cerevisiae)	66460	https://www.ncbi.nlm.nih.gov/gene/?term=66460
Zfp36l2	Zfp36l2	zinc finger protein 36, C3H type-like 2	12193	https://www.ncbi.nlm.nih.gov/gene/?term=12193
Atcay	Atcay	ataxia, cerebellar, Cayman type	16467	https://www.ncbi.nlm.nih.gov/gene/?term=16467
Nfix	Nfix	nuclear factor I/X	18032	https://www.ncbi.nlm.nih.gov/gene/?term=18032
Sin3a	Sin3a	transcriptional regulator, SIN3A (yeast)	20466	https://www.ncbi.nlm.nih.gov/gene/?term=20466

Biology Open • Supplementary information

Gng2	Gng2	guanine nucleotide binding protein (G protein), gamma 2	14702	https://www.ncbi.nlm.nih.gov/gene/?term=14702
L3mbtl3	L3mbtl3	L3MBTL3 histone methyl-lysine binding protein	237339	https://www.ncbi.nlm.nih.gov/gene/?term=237339
Clvs1	Clvs1	clavesin 1	74438	https://www.ncbi.nlm.nih.gov/gene/?term=74438
Tmtc4	Tmtc4	transmembrane and tetratricopeptide repeat containing 4	70551	https://www.ncbi.nlm.nih.gov/gene/?term=70551
Celf5	Celf5	CUGBP, Elav-like family member 5	319586	https://www.ncbi.nlm.nih.gov/gene/?term=319586
Fermt2	Fermt2	fermitin family member 2	218952	https://www.ncbi.nlm.nih.gov/gene/?term=218952
Sh2b3	Sh2b3	SH2B adaptor protein 3	16923	https://www.ncbi.nlm.nih.gov/gene/?term=16923
Vezf1	Vezf1	vascular endothelial zinc finger 1	22344	https://www.ncbi.nlm.nih.gov/gene/?term=22344
Uhrf1bp1	Uhrf1bp1	UHRF1 (ICBP90) binding protein 1	224648	https://www.ncbi.nlm.nih.gov/gene/?term=224648
Has3	Has3	hyaluronan synthase 3	15118	https://www.ncbi.nlm.nih.gov/gene/?term=15118
Syn3	Syn3	synapsin III	27204	https://www.ncbi.nlm.nih.gov/gene/?term=27204
Arhgap6	Arhgap6	Rho GTPase activating protein 6	11856	https://www.ncbi.nlm.nih.gov/gene/?term=11856
Pxmp4	Pxmp4	peroxisomal membrane protein 4	59038	https://www.ncbi.nlm.nih.gov/gene/?term=59038
Slc6a8	Slc6a8	solute carrier family 6 (neurotransmitter transporter, creatine), member 8	102857	https://www.ncbi.nlm.nih.gov/gene/?term=102857
Corin	Corin	corin	53419	https://www.ncbi.nlm.nih.gov/gene/?term=53419
1700017B05Rik	1700017B05Rik	RIKEN cDNA 1700017B05 gene	74211	https://www.ncbi.nlm.nih.gov/gene/?term=74211
Dcp1a	Dcp1a	decapping mRNA 1A	75901	https://www.ncbi.nlm.nih.gov/gene/?term=75901
Sptb	Sptb	spectrin beta, erythrocytic	20741	https://www.ncbi.nlm.nih.gov/gene/?term=20741
Zfp385a	Zfp385a	zinc finger protein 385A	29813	https://www.ncbi.nlm.nih.gov/gene/?term=29813
Mcu	Mcu	mitochondrial calcium uniporter	215999	https://www.ncbi.nlm.nih.gov/gene/?term=215999

Biology Open • Supplementary information

Slx4	Slx4	SLX4 structure-specific endonuclease subunit homolog (S. cerevisiae)	52864	https://www.ncbi.nlm.nih.gov/gene/?term=52864
Rasl12	Rasl12	RAS-like, family 12	70784	https://www.ncbi.nlm.nih.gov/gene/?term=70784
Ube2d1	Ube2d1	ubiquitin-conjugating enzyme E2D 1	216080	https://www.ncbi.nlm.nih.gov/gene/?term=216080
Selenoi	Selenoi	selenoprotein I	28042	https://www.ncbi.nlm.nih.gov/gene/?term=28042
Rarb	Rarb	retinoic acid receptor, beta	218772	https://www.ncbi.nlm.nih.gov/gene/?term=218772
Mef2a	Mef2a	myocyte enhancer factor 2A	17258	https://www.ncbi.nlm.nih.gov/gene/?term=17258
Igip	Igip	IgA inducing protein	109169	https://www.ncbi.nlm.nih.gov/gene/?term=109169
Afdn	Afdn	afadin, adherens junction formation factor	17356	https://www.ncbi.nlm.nih.gov/gene/?term=17356
Ehmt1	Ehmt1	euchromatic histone methyltransferase 1	77683	https://www.ncbi.nlm.nih.gov/gene/?term=77683
Sucla2	Sucla2	succinate-Coenzyme A ligase, ADP-forming, beta subunit	20916	https://www.ncbi.nlm.nih.gov/gene/?term=20916
Fbxw11	Fbxw11	F-box and WD-40 domain protein 11	103583	https://www.ncbi.nlm.nih.gov/gene/?term=103583
Teddm1b	Teddm1b	transmembrane epididymal protein 1B	433365	https://www.ncbi.nlm.nih.gov/gene/?term=433365
Shcbp1	Shcbp1	Shc SH2-domain binding protein 1	20419	https://www.ncbi.nlm.nih.gov/gene/?term=20419
Ipo8	Ipo8	importin 8	320727	https://www.ncbi.nlm.nih.gov/gene/?term=320727
Mycl	Mycl	v-myc avian myelocytomatosis viral oncogene lung carcinoma derived	16918	https://www.ncbi.nlm.nih.gov/gene/?term=16918
Ets1	Ets1	E26 avian leukemia oncogene 1, 5' domain	23871	https://www.ncbi.nlm.nih.gov/gene/?term=23871
Tshz3	Tshz3	teashirt zinc finger family member 3	243931	https://www.ncbi.nlm.nih.gov/gene/?term=243931

Biology Open • Supplementary information

Rfx3	Rfx3	regulatory factor X, 3 (influences HLA class II expression)	19726	https://www.ncbi.nlm.nih.gov/gene/?term=19726
Smoc1	Smoc1	SPARC related modular calcium binding 1	64075	https://www.ncbi.nlm.nih.gov/gene/?term=64075
Limch1	Limch1	LIM and calponin homology domains 1	77569	https://www.ncbi.nlm.nih.gov/gene/?term=77569
Zfp207	Zfp207	zinc finger protein 207	22680	https://www.ncbi.nlm.nih.gov/gene/?term=22680
Scn1a	Scn1a	sodium channel, voltage-gated, type I, alpha	20265	https://www.ncbi.nlm.nih.gov/gene/?term=20265
Atp5g2	Atp5g2	ATP synthase, H+ transporting, mitochondrial F0 complex, subunit C2 (subunit 9)	67942	https://www.ncbi.nlm.nih.gov/gene/?term=67942
Rnaseh1	Rnaseh1	ribonuclease H1	19819	https://www.ncbi.nlm.nih.gov/gene/?term=19819
Cttnbp2	Cttnbp2	cortactin binding protein 2	30785	https://www.ncbi.nlm.nih.gov/gene/?term=30785
Hif1a	Hif1a	hypoxia inducible factor 1, alpha subunit	15251	https://www.ncbi.nlm.nih.gov/gene/?term=15251
Emp1	Emp1	epithelial membrane protein 1	13730	https://www.ncbi.nlm.nih.gov/gene/?term=13730
Meis2	Meis2	Meis homeobox 2	17536	https://www.ncbi.nlm.nih.gov/gene/?term=17536
Arhgap42	Arhgap42	Rho GTPase activating protein 42	71544	https://www.ncbi.nlm.nih.gov/gene/?term=71544
Hccs	Hccs	holocytochrome c synthetase	15159	https://www.ncbi.nlm.nih.gov/gene/?term=15159
Pank1	Pank1	pantothenate kinase 1	75735	https://www.ncbi.nlm.nih.gov/gene/?term=75735
Kctd10	Kctd10	potassium channel tetramerisation domain containing 10	330171	https://www.ncbi.nlm.nih.gov/gene/?term=330171
Tnfsf11	Tnfsf11	tumor necrosis factor (ligand) superfamily, member 11	21943	https://www.ncbi.nlm.nih.gov/gene/?term=21943
Begain	Begain	brain-enriched guanylate kinase-associated	380785	https://www.ncbi.nlm.nih.gov/gene/?term=380785
Kif2a	Kif2a	kinesin family member 2A	16563	https://www.ncbi.nlm.nih.gov/gene/?term=16563

Biology Open • Supplementary information

Oprk1	Oprk1	opioid receptor, kappa 1	18387	https://www.ncbi.nlm.nih.gov/gene/?term=18387
Slc23a2	Slc23a2	solute carrier family 23 (nucleobase transporters), member 2	54338	https://www.ncbi.nlm.nih.gov/gene/?term=54338
Impact	Impact	impact, RWD domain protein	16210	https://www.ncbi.nlm.nih.gov/gene/?term=16210
Aldh1a3	Aldh1a3	aldehyde dehydrogenase family 1, subfamily A3	56847	https://www.ncbi.nlm.nih.gov/gene/?term=56847
Tet2	Tet2	tet methylcytosine dioxygenase 2	214133	https://www.ncbi.nlm.nih.gov/gene/?term=214133
Wee1	Wee1	WEE 1 homolog 1 (S. pombe)	22390	https://www.ncbi.nlm.nih.gov/gene/?term=22390
Zbtb18	Zbtb18	zinc finger and BTB domain containing 18	30928	https://www.ncbi.nlm.nih.gov/gene/?term=30928
Usp42	Usp42	ubiquitin specific peptidase 42	76800	https://www.ncbi.nlm.nih.gov/gene/?term=76800
Nin	Nin	ninein	18080	https://www.ncbi.nlm.nih.gov/gene/?term=18080
Togaram1	Togaram1	TOG array regulator of axonemal microtubules 1	328108	https://www.ncbi.nlm.nih.gov/gene/?term=328108
Cct6a	Cct6a	chaperonin containing Tcp1, subunit 6a (zeta)	12466	https://www.ncbi.nlm.nih.gov/gene/?term=12466
Vkorc1l1	Vkorc1l1	vitamin K epoxide reductase complex, subunit 1-like 1	69568	https://www.ncbi.nlm.nih.gov/gene/?term=69568
Plekhg1	Plekhg1	pleckstrin homology domain containing, family G (with RhoGef domain) member 1	213783	https://www.ncbi.nlm.nih.gov/gene/?term=213783
Slc5a7	Slc5a7	solute carrier family 5 (choline transporter), member 7	63993	https://www.ncbi.nlm.nih.gov/gene/?term=63993
Kat6a	Kat6a	K(lysine) acetyltransferase 6A	244349	https://www.ncbi.nlm.nih.gov/gene/?term=244349
Rbm48	Rbm48	RNA binding motif protein 48	269623	https://www.ncbi.nlm.nih.gov/gene/?term=269623
Zdhhc21	Zdhhc21	zinc finger, DHHC domain containing 21	68268	https://www.ncbi.nlm.nih.gov/gene/?term=68268

Biology Open • Supplementary information

Scfd1	Scfd1	Sec1 family domain containing 1	76983	https://www.ncbi.nlm.nih.gov/gene/?term=76983
Nid2	Nid2	nidogen 2	18074	https://www.ncbi.nlm.nih.gov/gene/?term=18074
Fat4	Fat4	FAT atypical cadherin 4	329628	https://www.ncbi.nlm.nih.gov/gene/?term=329628
Tek	Tek	TEK receptor tyrosine kinase	21687	https://www.ncbi.nlm.nih.gov/gene/?term=21687
Sap30l	Sap30l	SAP30-like	50724	https://www.ncbi.nlm.nih.gov/gene/?term=50724
Mycn	Mycn	v-myc avian myelocytomatosis viral related oncogene, neuroblastoma derived	18109	https://www.ncbi.nlm.nih.gov/gene/?term=18109
Sorcs3	Sorcs3	sortilin-related VPS10 domain containing receptor 3	66673	https://www.ncbi.nlm.nih.gov/gene/?term=66673
Olfr701	Olfr701	olfactory receptor 701	66786	https://www.ncbi.nlm.nih.gov/gene/?term=66786
Gdf10	Gdf10	growth differentiation factor 10	14560	https://www.ncbi.nlm.nih.gov/gene/?term=14560
Zcchc2	Zcchc2	zinc finger, CCHC domain containing 2	227449	https://www.ncbi.nlm.nih.gov/gene/?term=227449
Tmem196	Tmem196	transmembrane protein 196	217951	https://www.ncbi.nlm.nih.gov/gene/?term=217951
Ugp2	Ugp2	UDP-glucose pyrophosphorylase 2	216558	https://www.ncbi.nlm.nih.gov/gene/?term=216558
Ptpn9	Ptpn9	protein tyrosine phosphatase, non-receptor type 9	56294	https://www.ncbi.nlm.nih.gov/gene/?term=56294
Smad4	Smad4	SMAD family member 4	17128	https://www.ncbi.nlm.nih.gov/gene/?term=17128
Med4	Med4	mediator complex subunit 4	67381	https://www.ncbi.nlm.nih.gov/gene/?term=67381
Adamts17	Adamts17	a disintegrin-like and metallopeptidase (reprolysin type) with thrombospondin type 1 motif, 17	233332	https://www.ncbi.nlm.nih.gov/gene/?term=233332
Pdcl	Pdcl	phosducin-like	67466	https://www.ncbi.nlm.nih.gov/gene/?term=67466
Tfap4	Tfap4	transcription factor AP4	83383	https://www.ncbi.nlm.nih.gov/gene/?term=83383
Arid2	Arid2	AT rich interactive domain 2 (ARID, RFX-like)	77044	https://www.ncbi.nlm.nih.gov/gene/?term=77044

Biology Open • Supplementary information

Dcbld2	Dcbld2	discoidin, CUB and LCCL domain containing 2	73379	https://www.ncbi.nlm.nih.gov/gene/?term=73379
Nfe2l2	Nfe2l2	nuclear factor, erythroid derived 2, like 2	18024	https://www.ncbi.nlm.nih.gov/gene/?term=18024
Fndc3a	Fndc3a	fibronectin type III domain containing 3A	319448	https://www.ncbi.nlm.nih.gov/gene/?term=319448
Brpf1	Brpf1	bromodomain and PHD finger containing, 1	78783	https://www.ncbi.nlm.nih.gov/gene/?term=78783
Pnrc1	Pnrc1	proline-rich nuclear receptor coactivator 1	108767	https://www.ncbi.nlm.nih.gov/gene/?term=108767
Mapk6	Mapk6	mitogen-activated protein kinase 6	50772	https://www.ncbi.nlm.nih.gov/gene/?term=50772
Mob4	Mob4	MOB family member 4, phocein	19070	https://www.ncbi.nlm.nih.gov/gene/?term=19070
Htra3	Htra3	HtrA serine peptidase 3	78558	https://www.ncbi.nlm.nih.gov/gene/?term=78558
Rin2	Rin2	Ras and Rab interactor 2	74030	https://www.ncbi.nlm.nih.gov/gene/?term=74030
Ube2d2a	Ube2d2a	ubiquitin-conjugating enzyme E2D 2A	56550	https://www.ncbi.nlm.nih.gov/gene/?term=56550
Cacna2d1	Cacna2d1	calcium channel, voltage-dependent, alpha2/delta subunit 1	12293	https://www.ncbi.nlm.nih.gov/gene/?term=12293
Bicd2	Bicd2	BICD cargo adaptor 2	76895	https://www.ncbi.nlm.nih.gov/gene/?term=76895
Nr1d2	Nr1d2	nuclear receptor subfamily 1, group D, member 2	353187	https://www.ncbi.nlm.nih.gov/gene/?term=353187
Marf1	Marf1	meiosis regulator and mRNA stability 1	223989	https://www.ncbi.nlm.nih.gov/gene/?term=223989
Shisa6	Shisa6	shisa family member 6	380702	https://www.ncbi.nlm.nih.gov/gene/?term=380702
Map3k4	Map3k4	mitogen-activated protein kinase kinase kinase 4	26407	https://www.ncbi.nlm.nih.gov/gene/?term=26407
Ssb	Ssb	Sjogren syndrome antigen B	20823	https://www.ncbi.nlm.nih.gov/gene/?term=20823
Cav3	Cav3	caveolin 3	12391	https://www.ncbi.nlm.nih.gov/gene/?term=12391

Biology Open • Supplementary information

Fam76b	Fam76b	family with sequence similarity 76, member B	72826	https://www.ncbi.nlm.nih.gov/gene/?term=72826
Stc1	Stc1	stanniocalcin 1	20855	https://www.ncbi.nlm.nih.gov/gene/?term=20855
Sp4	Sp4	trans-acting transcription factor 4	20688	https://www.ncbi.nlm.nih.gov/gene/?term=20688
Pcdh18	Pcdh18	protocadherin 18	73173	https://www.ncbi.nlm.nih.gov/gene/?term=73173
Als2	Als2	alsin Rho guanine nucleotide exchange factor	74018	https://www.ncbi.nlm.nih.gov/gene/?term=74018
Slc25a36	Slc25a36	solute carrier family 25, member 36	192287	https://www.ncbi.nlm.nih.gov/gene/?term=192287
Cdh11	Cdh11	cadherin 11	12552	https://www.ncbi.nlm.nih.gov/gene/?term=12552
Gbe1	Gbe1	glucan (1,4-alpha-), branching enzyme 1	74185	https://www.ncbi.nlm.nih.gov/gene/?term=74185
Ccng2	Ccng2	cyclin G2	12452	https://www.ncbi.nlm.nih.gov/gene/?term=12452
Nacc2	Nacc2	nucleus accumbens associated 2, BEN and BTB (POZ) domain containing	67991	https://www.ncbi.nlm.nih.gov/gene/?term=67991
Uevld	Uevld	UEV and lactate/malate dehyrogenase domains	54122	https://www.ncbi.nlm.nih.gov/gene/?term=54122
Sall1	Sall1	spalt like transcription factor 1	58198	https://www.ncbi.nlm.nih.gov/gene/?term=58198
Garnl3	Garnl3	GTPase activating RANGAP domain-like 3	99326	https://www.ncbi.nlm.nih.gov/gene/?term=99326
Golt1b	Golt1b	golgi transport 1B	66964	https://www.ncbi.nlm.nih.gov/gene/?term=66964
Uchl3	Uchl3	ubiquitin carboxyl-terminal esterase L3 (ubiquitin thiolesterase)	50933	https://www.ncbi.nlm.nih.gov/gene/?term=50933
Atp1b1	Atp1b1	ATPase, Na+/K+ transporting, beta 1 polypeptide	11931	https://www.ncbi.nlm.nih.gov/gene/?term=11931
Sec24a	Sec24a	Sec24 related gene family, member A (S. cerevisiae)	77371	https://www.ncbi.nlm.nih.gov/gene/?term=77371

Biology Open • Supplementary information

Ppp2r2a	Ppp2r2a	protein phosphatase 2, regulatory subunit B, alpha	71978	https://www.ncbi.nlm.nih.gov/gene/?term=71978
Kcnmb2	Kcnmb2	potassium large conductance calcium-activated channel, subfamily M, beta member 2	72413	https://www.ncbi.nlm.nih.gov/gene/?term=72413
Ptprj	Ptprj	protein tyrosine phosphatase, receptor type, J	19271	https://www.ncbi.nlm.nih.gov/gene/?term=19271
Arid1a	Arid1a	AT rich interactive domain 1A (SWI-like)	93760	https://www.ncbi.nlm.nih.gov/gene/?term=93760
Uba2	Uba2	ubiquitin-like modifier activating enzyme 2	50995	https://www.ncbi.nlm.nih.gov/gene/?term=50995
Atp2b1	Atp2b1	ATPase, Ca++ transporting, plasma membrane 1	67972	https://www.ncbi.nlm.nih.gov/gene/?term=67972
Bmpr1b	Bmpr1b	bone morphogenetic protein receptor, type 1B	12167	https://www.ncbi.nlm.nih.gov/gene/?term=12167
Nlk	Nlk	nemo like kinase	18099	https://www.ncbi.nlm.nih.gov/gene/?term=18099
Herpud1	Herpud1	homocysteine-inducible, endoplasmic reticulum stress-inducible, ubiquitin-like domain member 1	64209	https://www.ncbi.nlm.nih.gov/gene/?term=64209
Sco1	Sco1	SCO1 cytochrome c oxidase assembly protein	52892	https://www.ncbi.nlm.nih.gov/gene/?term=52892
Megf9	Megf9	multiple EGF-like-domains 9	230316	https://www.ncbi.nlm.nih.gov/gene/?term=230316
9930021J03Rik	9930021J03Rik	RIKEN cDNA 9930021J03 gene	240613	https://www.ncbi.nlm.nih.gov/gene/?term=240613
Ddx59	Ddx59	DEAD (Asp-Glu-Ala-Asp) box polypeptide 59	67997	https://www.ncbi.nlm.nih.gov/gene/?term=67997
Cmc1	Cmc1	COX assembly mitochondrial protein 1	67899	https://www.ncbi.nlm.nih.gov/gene/?term=67899

Biology Open • Supplementary information

Msx1	Msx1	msh homeobox 1	17701	https://www.ncbi.nlm.nih.gov/gene/?term=17701
Kcnh8	Kcnh8	potassium voltage-gated channel, subfamily H (eag-related), member 8	211468	https://www.ncbi.nlm.nih.gov/gene/?term=211468
Kmt5c	Kmt5c	lysine methyltransferase 5C	232811	https://www.ncbi.nlm.nih.gov/gene/?term=232811
Vegfa	Vegfa	vascular endothelial growth factor A	22339	https://www.ncbi.nlm.nih.gov/gene/?term=22339
Pcdha12	Pcdha12	protocadherin alpha 12	192164	https://www.ncbi.nlm.nih.gov/gene/?term=192164
Tll1	Tll1	tolloid-like	21892	https://www.ncbi.nlm.nih.gov/gene/?term=21892
Sestd1	Sestd1	SEC14 and spectrin domains 1	228071	https://www.ncbi.nlm.nih.gov/gene/?term=228071
Dgkd	Dgkd	diacylglycerol kinase, delta	227333	https://www.ncbi.nlm.nih.gov/gene/?term=227333
Timd2	Timd2	T cell immunoglobulin and mucin domain containing 2	171284	https://www.ncbi.nlm.nih.gov/gene/?term=171284
Col6a3	Col6a3	collagen, type VI, alpha 3	12835	https://www.ncbi.nlm.nih.gov/gene/?term=12835
Eomes	Eomes	eomesodermin	13813	https://www.ncbi.nlm.nih.gov/gene/?term=13813
Col4a3	Col4a3	collagen, type IV, alpha 3	12828	https://www.ncbi.nlm.nih.gov/gene/?term=12828
Foxj2	Foxj2	forkhead box J2	60611	https://www.ncbi.nlm.nih.gov/gene/?term=60611
Stmn2	Stmn2	stathmin-like 2	20257	https://www.ncbi.nlm.nih.gov/gene/?term=20257
Kif26a	Kif26a	kinesin family member 26A	668303	https://www.ncbi.nlm.nih.gov/gene/?term=668303
Igf1	Igf1	insulin-like growth factor 1	16000	https://www.ncbi.nlm.nih.gov/gene/?term=16000
Nup160	Nup160	nucleoporin 160	59015	https://www.ncbi.nlm.nih.gov/gene/?term=59015
Col1a1	Col1a1	collagen, type I, alpha 1	12842	https://www.ncbi.nlm.nih.gov/gene/?term=12842
Hormad1	Hormad1	HORMA domain containing 1	67981	https://www.ncbi.nlm.nih.gov/gene/?term=67981
Pcdha7	Pcdha7	protocadherin alpha 7	12939	https://www.ncbi.nlm.nih.gov/gene/?term=12939
Tet1	Tet1	tet methylcytosine dioxygenase 1	52463	https://www.ncbi.nlm.nih.gov/gene/?term=52463
Dio2	Dio2	deiodinase, iodothyronine, type II	13371	https://www.ncbi.nlm.nih.gov/gene/?term=13371
Rapgefl1	Rapgefl1	Rap guanine nucleotide exchange factor (GEF)-like 1	268480	https://www.ncbi.nlm.nih.gov/gene/?term=268480

Biology Open • Supplementary information

Frat2	Frat2	frequently rearranged in advanced T cell lymphomas 2	212398	https://www.ncbi.nlm.nih.gov/gene/?term=212398
Rnpepl1	Rnpepl1	arginyl aminopeptidase (aminopeptidase B)-like 1	108657	https://www.ncbi.nlm.nih.gov/gene/?term=108657
Kdm5b	Kdm5b	lysine (K)-specific demethylase 5B	75605	https://www.ncbi.nlm.nih.gov/gene/?term=75605
Kcna5	Kcna5	potassium voltage-gated channel, shaker-related subfamily, member 5	16493	https://www.ncbi.nlm.nih.gov/gene/?term=16493
Traf4	Traf4	TNF receptor associated factor 4	22032	https://www.ncbi.nlm.nih.gov/gene/?term=22032
Iffo1	Iffo1	intermediate filament family orphan 1	320678	https://www.ncbi.nlm.nih.gov/gene/?term=320678
Slc31a1	Slc31a1	solute carrier family 31, member 1	20529	https://www.ncbi.nlm.nih.gov/gene/?term=20529
Elf2	Elf2	E74-like factor 2	69257	https://www.ncbi.nlm.nih.gov/gene/?term=69257
Col19a1	Col19a1	collagen, type XIX, alpha 1	12823	https://www.ncbi.nlm.nih.gov/gene/?term=12823
Igsf9b	Igsf9b	immunoglobulin superfamily, member 9B	235086	https://www.ncbi.nlm.nih.gov/gene/?term=235086
Tfeb	Tfeb	transcription factor EB	21425	https://www.ncbi.nlm.nih.gov/gene/?term=21425
Rab30	Rab30	RAB30, member RAS oncogene family	75985	https://www.ncbi.nlm.nih.gov/gene/?term=75985
Col5a3	Col5a3	collagen, type V, alpha 3	53867	https://www.ncbi.nlm.nih.gov/gene/?term=53867
Gid8	Gid8	GID complex subunit 8	76425	https://www.ncbi.nlm.nih.gov/gene/?term=76425
Dnmt3b	Dnmt3b	DNA methyltransferase 3B	13436	https://www.ncbi.nlm.nih.gov/gene/?term=13436
Cldn1	Cldn1	claudin 1	12737	https://www.ncbi.nlm.nih.gov/gene/?term=12737
Tarbp1	Tarbp1	TAR RNA binding protein 1	212728	https://www.ncbi.nlm.nih.gov/gene/?term=212728
Rnf19a	Rnf19a	ring finger protein 19A	30945	https://www.ncbi.nlm.nih.gov/gene/?term=30945
Erlin2	Erlin2	ER lipid raft associated 2	244373	https://www.ncbi.nlm.nih.gov/gene/?term=244373
Narf	Narf	nuclear prelamin A recognition factor	67608	https://www.ncbi.nlm.nih.gov/gene/?term=67608

Eml4	Eml4	echinoderm microtubule associated protein like 4	78798	https://www.ncbi.nlm.nih.gov/gene/?term=78798
Adamts2	Adamts2	a disintegrin-like and metallopeptidase (reprolysin type) with thrombospondin type 1 motif, 2	216725	https://www.ncbi.nlm.nih.gov/gene/?term=216725
Dnmt3a	Dnmt3a	DNA methyltransferase 3A	13435	https://www.ncbi.nlm.nih.gov/gene/?term=13435
Zfp282	Zfp282	zinc finger protein 282	101095	https://www.ncbi.nlm.nih.gov/gene/?term=101095
Zbtb5	Zbtb5	zinc finger and BTB domain containing 5	230119	https://www.ncbi.nlm.nih.gov/gene/?term=230119
Sh3pxd2a	Sh3pxd2a	SH3 and PX domains 2A	14218	https://www.ncbi.nlm.nih.gov/gene/?term=14218
Ireb2	Ireb2	iron responsive element binding protein 2	64602	https://www.ncbi.nlm.nih.gov/gene/?term=64602
Ttc9	Ttc9	tetratricopeptide repeat domain 9	69480	https://www.ncbi.nlm.nih.gov/gene/?term=69480
Pcdha10	Pcdha10	protocadherin alpha 10	12943	https://www.ncbi.nlm.nih.gov/gene/?term=12943
Pcdhac1	Pcdhac1	protocadherin alpha subfamily C, 1	353236	https://www.ncbi.nlm.nih.gov/gene/?term=353236
Col4a5	Col4a5	collagen, type IV, alpha 5	12830	https://www.ncbi.nlm.nih.gov/gene/?term=12830
Pxdn	Pxdn	peroxidasin	69675	https://www.ncbi.nlm.nih.gov/gene/?term=69675
Eln	Eln	elastin	13717	https://www.ncbi.nlm.nih.gov/gene/?term=13717
Tpk1	Tpk1	thiamine pyrophosphokinase	29807	https://www.ncbi.nlm.nih.gov/gene/?term=29807
Slc25a3	Slc25a3	solute carrier family 25 (mitochondrial carrier, phosphate carrier), member 3	18674	https://www.ncbi.nlm.nih.gov/gene/?term=18674
Pcdha4	Pcdha4	protocadherin alpha 4	12936	https://www.ncbi.nlm.nih.gov/gene/?term=12936
Slc43a2	Slc43a2	solute carrier family 43, member 2	215113	https://www.ncbi.nlm.nih.gov/gene/?term=215113
Pcdha5	Pcdha5	protocadherin alpha 5	12941	https://www.ncbi.nlm.nih.gov/gene/?term=12941
Brwd3	Brwd3	bromodomain and WD repeat domain containing 3	382236	https://www.ncbi.nlm.nih.gov/gene/?term=382236

Biology Open • Supplementary information

Pcdha8	Pcdha8	protocadherin alpha 8	353235	https://www.ncbi.nlm.nih.gov/gene/?term=353235
Samd4	Samd4	sterile alpha motif domain containing 4	74480	https://www.ncbi.nlm.nih.gov/gene/?term=74480
Unc13b	Unc13b	unc-13 homolog B	22249	https://www.ncbi.nlm.nih.gov/gene/?term=22249
Nap1l3	Nap1l3	nucleosome assembly protein 1-like 3	54561	https://www.ncbi.nlm.nih.gov/gene/?term=54561
Serpinh1	Serpinh1	serine (or cysteine) peptidase inhibitor, clade H, member 1	12406	https://www.ncbi.nlm.nih.gov/gene/?term=12406
Tmem183a	Tmem183a	transmembrane protein 183A	57439	https://www.ncbi.nlm.nih.gov/gene/?term=57439
Map6	Map6	microtubule-associated protein 6	17760	https://www.ncbi.nlm.nih.gov/gene/?term=17760
Glis2	Glis2	GLIS family zinc finger 2	83396	https://www.ncbi.nlm.nih.gov/gene/?term=83396
Atp2b4	Atp2b4	ATPase, Ca++ transporting, plasma membrane 4	381290	https://www.ncbi.nlm.nih.gov/gene/?term=381290
Pmp22	Pmp22	peripheral myelin protein 22	18858	https://www.ncbi.nlm.nih.gov/gene/?term=18858
Chsy1	Chsy1	chondroitin sulfate synthase 1	269941	https://www.ncbi.nlm.nih.gov/gene/?term=269941
Cd276	Cd276	CD276 antigen	102657	https://www.ncbi.nlm.nih.gov/gene/?term=102657
Col22a1	Col22a1	collagen, type XXII, alpha 1	69700	https://www.ncbi.nlm.nih.gov/gene/?term=69700
Stard8	Stard8	START domain containing 8	236920	https://www.ncbi.nlm.nih.gov/gene/?term=236920
Tet3	Tet3	tet methylcytosine dioxygenase 3	194388	https://www.ncbi.nlm.nih.gov/gene/?term=194388
Pcdha6	Pcdha6	protocadherin alpha 6	12937	https://www.ncbi.nlm.nih.gov/gene/?term=12937
Zfp704	Zfp704	zinc finger protein 704	170753	https://www.ncbi.nlm.nih.gov/gene/?term=170753
Tcf4	Tcf4	transcription factor 4	21413	https://www.ncbi.nlm.nih.gov/gene/?term=21413
Col5a1	Col5a1	collagen, type V, alpha 1	12831	https://www.ncbi.nlm.nih.gov/gene/?term=12831
Col7a1	Col7a1	collagen, type VII, alpha 1	12836	https://www.ncbi.nlm.nih.gov/gene/?term=12836
Dcun1d4	Dcun1d4	DCN1, defective in cullin neddylation 1, domain containing 4 (S. cerevisiae)	100737	https://www.ncbi.nlm.nih.gov/gene/?term=100737
Pgap2	Pgap2	post-GPI attachment to proteins 2	233575	https://www.ncbi.nlm.nih.gov/gene/?term=233575

Biology Open • Supplementary information

Ddx3x	Ddx3x	DEAD/H (Asp-Glu-Ala-Asp/His) box polypeptide 3, X-linked	13205	https://www.ncbi.nlm.nih.gov/gene/?term=13205
Col15a1	Col15a1	collagen, type XV, alpha 1	12819	https://www.ncbi.nlm.nih.gov/gene/?term=12819
Pcdha3	Pcdha3	protocadherin alpha 3	192163	https://www.ncbi.nlm.nih.gov/gene/?term=192163
Mapkbp1	Mapkbp1	mitogen-activated protein kinase binding protein 1	26390	https://www.ncbi.nlm.nih.gov/gene/?term=26390
Fer	Fer	fer (fms/fps related) protein kinase	14158	https://www.ncbi.nlm.nih.gov/gene/?term=14158
Mcl1	Mcl1	myeloid cell leukemia sequence 1	17210	https://www.ncbi.nlm.nih.gov/gene/?term=17210
Adamts9	Adamts9	a disintegrin-like and metallopeptidase (reprolysin type) with thrombospondin type 1 motif, 9	101401	https://www.ncbi.nlm.nih.gov/gene/?term=101401
Ifi30	Ifi30	interferon gamma inducible protein 30	65972	https://www.ncbi.nlm.nih.gov/gene/?term=65972
Adamts18	Adamts18	a disintegrin-like and metallopeptidase (reprolysin type) with thrombospondin type 1 motif, 18	208936	https://www.ncbi.nlm.nih.gov/gene/?term=208936
Col4a2	Col4a2	collagen, type IV, alpha 2	12827	https://www.ncbi.nlm.nih.gov/gene/?term=12827
Nav1	Nav1	neuron navigator 1	215690	https://www.ncbi.nlm.nih.gov/gene/?term=215690
Pcdha2	Pcdha2	protocadherin alpha 2	353234	https://www.ncbi.nlm.nih.gov/gene/?term=353234
Ppic	Ppic	peptidylprolyl isomerase C	19038	https://www.ncbi.nlm.nih.gov/gene/?term=19038
Lif	Lif	leukemia inhibitory factor	16878	https://www.ncbi.nlm.nih.gov/gene/?term=16878
Amer1	Amer1	APC membrane recruitment 1	72345	https://www.ncbi.nlm.nih.gov/gene/?term=72345
Pcdhac2	Pcdhac2	protocadherin alpha subfamily C, 2	353237	https://www.ncbi.nlm.nih.gov/gene/?term=353237
Ankrd13b	Ankrd13b	ankyrin repeat domain 13b	268445	https://www.ncbi.nlm.nih.gov/gene/?term=268445
Col5a2	Col5a2	collagen, type V, alpha 2	12832	https://www.ncbi.nlm.nih.gov/gene/?term=12832

Biology Open • Supplementary information

Taf5	Taf5	TATA-box binding protein associated factor 5	226182	https://www.ncbi.nlm.nih.gov/gene/?term=226182
Dusp2	Dusp2	dual specificity phosphatase 2	13537	https://www.ncbi.nlm.nih.gov/gene/?term=13537
Eva1b	Eva1b	eva-1 homolog B (C. elegans)	230752	https://www.ncbi.nlm.nih.gov/gene/?term=230752
Nav2	Nav2	neuron navigator 2	78286	https://www.ncbi.nlm.nih.gov/gene/?term=78286
Mark3	Mark3	MAP/microtubule affinity regulating kinase 3	17169	https://www.ncbi.nlm.nih.gov/gene/?term=17169
Pcdha9	Pcdha9	protocadherin alpha 9	192161	https://www.ncbi.nlm.nih.gov/gene/?term=192161
Dpysl5	Dpysl5	dihydropyrimidinase-like 5	65254	https://www.ncbi.nlm.nih.gov/gene/?term=65254
Camk4	Camk4	calcium/calmodulin-dependent protein kinase IV	12326	https://www.ncbi.nlm.nih.gov/gene/?term=12326
Col2a1	Col2a1	collagen, type II, alpha 1	12824	https://www.ncbi.nlm.nih.gov/gene/?term=12824
Il1rap	Il1rap	interleukin 1 receptor accessory protein	16180	https://www.ncbi.nlm.nih.gov/gene/?term=16180
Lysmd1	Lysmd1	LysM, putative peptidoglycan-binding, domain containing 1	217779	https://www.ncbi.nlm.nih.gov/gene/?term=217779
Zfp346	Zfp346	zinc finger protein 346	26919	https://www.ncbi.nlm.nih.gov/gene/?term=26919
Dlg2	Dlg2	discs large MAGUK scaffold protein 2	23859	https://www.ncbi.nlm.nih.gov/gene/?term=23859
Fam167a	Fam167a	family with sequence similarity 167, member A	219148	https://www.ncbi.nlm.nih.gov/gene/?term=219148
Ppm1d	Ppm1d	protein phosphatase 1D magnesium-dependent, delta isoform	53892	https://www.ncbi.nlm.nih.gov/gene/?term=53892
N4bp2l1	N4bp2l1	NEDD4 binding protein 2-like 1	100637	https://www.ncbi.nlm.nih.gov/gene/?term=100637
Col4a6	Col4a6	collagen, type IV, alpha 6	94216	https://www.ncbi.nlm.nih.gov/gene/?term=94216
Col9a1	Col9a1	collagen, type IX, alpha 1	12839	https://www.ncbi.nlm.nih.gov/gene/?term=12839

Biology Open • Supplementary information

Fem1b	Fem1b	feminization 1 homolog b (C. elegans)	14155	https://www.ncbi.nlm.nih.gov/gene/?term=14155
Smtnl2	Smtnl2	smoothelin-like 2	276829	https://www.ncbi.nlm.nih.gov/gene/?term=276829
Hdac4	Hdac4	histone deacetylase 4	208727	https://www.ncbi.nlm.nih.gov/gene/?term=208727
Atp1b4	Atp1b4	ATPase, (Na+)/K+ transporting, beta 4 polypeptide	67821	https://www.ncbi.nlm.nih.gov/gene/?term=67821
Zmiz1	Zmiz1	zinc finger, MIZ-type containing 1	328365	https://www.ncbi.nlm.nih.gov/gene/?term=328365
Pcdha1	Pcdha1	protocadherin alpha 1	116731	https://www.ncbi.nlm.nih.gov/gene/?term=116731
D630045J12Rik	D630045J12Rik	RIKEN cDNA D630045J12 gene	330286	https://www.ncbi.nlm.nih.gov/gene/?term=330286
Proser1	Proser1	proline and serine rich 1	212127	https://www.ncbi.nlm.nih.gov/gene/?term=212127
Carmil1	Carmil1	capping protein regulator and myosin 1 linker 1	68732	https://www.ncbi.nlm.nih.gov/gene/?term=68732
Trim63	Trim63	tripartite motif-containing 63	433766	https://www.ncbi.nlm.nih.gov/gene/?term=433766
Rest	Rest	RE1-silencing transcription factor	19712	https://www.ncbi.nlm.nih.gov/gene/?term=19712
Bmf	Bmf	BCL2 modifying factor	171543	https://www.ncbi.nlm.nih.gov/gene/?term=171543
Col11a1	Col11a1	collagen, type XI, alpha 1	12814	https://www.ncbi.nlm.nih.gov/gene/?term=12814
Morf4l2	Morf4l2	mortality factor 4 like 2	56397	https://www.ncbi.nlm.nih.gov/gene/?term=56397
C1qtnf6	C1qtnf6	C1q and tumor necrosis factor related protein 6	72709	https://www.ncbi.nlm.nih.gov/gene/?term=72709
Adam19	Adam19	a disintegrin and metallopeptidase domain 19 (meltrin beta)	11492	https://www.ncbi.nlm.nih.gov/gene/?term=11492
Cdk6	Cdk6	cyclin-dependent kinase 6	12571	https://www.ncbi.nlm.nih.gov/gene/?term=12571
Crispld1	Crispld1	cysteine-rich secretory protein LCCL domain containing 1	83691	https://www.ncbi.nlm.nih.gov/gene/?term=83691
Pten	Pten	phosphatase and tensin homolog	19211	https://www.ncbi.nlm.nih.gov/gene/?term=19211
Hbp1	Hbp1	high mobility group box transcription factor 1	73389	https://www.ncbi.nlm.nih.gov/gene/?term=73389
Fstl1	Fstl1	follistatin-like 1	14314	https://www.ncbi.nlm.nih.gov/gene/?term=14314

Biology Open • Supplementary information

Pcdha11	Pcdha11	protocadherin alpha 11	12942	https://www.ncbi.nlm.nih.gov/gene/?term=12942
Zbtb10	Zbtb10	zinc finger and BTB domain containing 10	229055	https://www.ncbi.nlm.nih.gov/gene/?term=229055
Smurf2	Smurf2	SMAD specific E3 ubiquitin protein ligase 2	66313	https://www.ncbi.nlm.nih.gov/gene/?term=66313
Sparc	Sparc	secreted acidic cysteine rich glycoprotein	20692	https://www.ncbi.nlm.nih.gov/gene/?term=20692
Purg	Purg	purine-rich element binding protein G	75029	https://www.ncbi.nlm.nih.gov/gene/?term=75029
Nsd1	Nsd1	nuclear receptor-binding SET-domain protein 1	18193	https://www.ncbi.nlm.nih.gov/gene/?term=18193
Fzd8	Fzd8	frizzled class receptor 8	14370	https://www.ncbi.nlm.nih.gov/gene/?term=14370
Slc16a2	Slc16a2	solute carrier family 16 (monocarboxylic acid transporters), member 2	20502	https://www.ncbi.nlm.nih.gov/gene/?term=20502
Man1a2	Man1a2	mannosidase, alpha, class 1A, member 2	17156	https://www.ncbi.nlm.nih.gov/gene/?term=17156
Gphn	Gphn	gephyrin	268566	https://www.ncbi.nlm.nih.gov/gene/?term=268566
Wdr55	Wdr55	WD repeat domain 55	67936	https://www.ncbi.nlm.nih.gov/gene/?term=67936
Zfand2a	Zfand2a	zinc finger, AN1-type domain 2A	100494	https://www.ncbi.nlm.nih.gov/gene/?term=100494
Mcf2	Mcf2	mcf.2 transforming sequence	109904	https://www.ncbi.nlm.nih.gov/gene/?term=109904
Pitpna	Pitpna	phosphatidylinositol transfer protein, alpha	18738	https://www.ncbi.nlm.nih.gov/gene/?term=18738
Ylpm1	Ylpm1	YLP motif containing 1	56531	https://www.ncbi.nlm.nih.gov/gene/?term=56531
Rasgrp1	Rasgrp1	RAS guanyl releasing protein 1	19419	https://www.ncbi.nlm.nih.gov/gene/?term=19419
Cnot7	Cnot7	CCR4-NOT transcription complex, subunit 7	18983	https://www.ncbi.nlm.nih.gov/gene/?term=18983
Fst	Fst	follistatin	14313	https://www.ncbi.nlm.nih.gov/gene/?term=14313

Biology Open • Supplementary information

Lipo2	Lipo2	lipase, member O2	101055671	https://www.ncbi.nlm.nih.gov/gene/?term=101055
Chd9	Chd9	chromodomain helicase DNA binding protein 9	109151	https://www.ncbi.nlm.nih.gov/gene/?term=109151
Gnat1	Gnat1	guanine nucleotide binding protein, alpha transducing 1	14685	https://www.ncbi.nlm.nih.gov/gene/?term=14685
Ywhaq	Ywhaq	tyrosine 3-monooxygenase/tryptophan 5-monooxygenase activation protein theta	22630	https://www.ncbi.nlm.nih.gov/gene/?term=22630
Mob3c	Mob3c	MOB kinase activator 3C	100465	https://www.ncbi.nlm.nih.gov/gene/?term=100465
Igf2bp3	Igf2bp3	insulin-like growth factor 2 mRNA binding protein 3	140488	https://www.ncbi.nlm.nih.gov/gene/?term=140488
Prkar2a	Prkar2a	protein kinase, cAMP dependent regulatory, type II alpha	19087	https://www.ncbi.nlm.nih.gov/gene/?term=19087
Ufm1	Ufm1	ubiquitin-fold modifier 1	67890	https://www.ncbi.nlm.nih.gov/gene/?term=67890
Crhbp	Crhbp	corticotropin releasing hormone binding protein	12919	https://www.ncbi.nlm.nih.gov/gene/?term=12919
Scn5a	Scn5a	sodium channel, voltage-gated, type V, alpha	20271	https://www.ncbi.nlm.nih.gov/gene/?term=20271
Seh1l	Seh1l	SEH1-like (S. cerevisiae	72124	https://www.ncbi.nlm.nih.gov/gene/?term=72124
Six3	Six3	sine oculis-related homeobox 3	20473	https://www.ncbi.nlm.nih.gov/gene/?term=20473
Serpinb10	Serpinb10	serine (or cysteine) peptidase inhibitor, clade B (ovalbumin), member 10	241197	https://www.ncbi.nlm.nih.gov/gene/?term=241197
Dusp8	Dusp8	dual specificity phosphatase 8	18218	https://www.ncbi.nlm.nih.gov/gene/?term=18218
Rpe65	Rpe65	retinal pigment epithelium 65	19892	https://www.ncbi.nlm.nih.gov/gene/?term=19892
Nefl	Nefl	neurofilament, light polypeptide	18039	https://www.ncbi.nlm.nih.gov/gene/?term=18039

Biology Open • Supplementary information

Mitd1	Mitd1	MIT, microtubule interacting and transport, domain containing 1	69028	https://www.ncbi.nlm.nih.gov/gene/?term=69028
Bnc2	Bnc2	basonuclin 2	242509	https://www.ncbi.nlm.nih.gov/gene/?term=242509
Ccnjl	Ccnjl	cyclin J-like	380694	https://www.ncbi.nlm.nih.gov/gene/?term=380694
Lrrc17	Lrrc17	leucine rich repeat containing 17	74511	https://www.ncbi.nlm.nih.gov/gene/?term=74511
Ubn2	Ubn2	ubinuclein 2	320538	https://www.ncbi.nlm.nih.gov/gene/?term=320538
Ptpn2	Ptpn2	protein tyrosine phosphatase, non-receptor type 2	19255	https://www.ncbi.nlm.nih.gov/gene/?term=19255
4933409G03Rik	4933409G03Rik	RIKEN cDNA 4933409G03 gene	227998	https://www.ncbi.nlm.nih.gov/gene/?term=227998
Fam83f	Fam83f	family with sequence similarity 83, member F	213956	https://www.ncbi.nlm.nih.gov/gene/?term=213956
Lipi	Lipi	lipase, member I	320355	https://www.ncbi.nlm.nih.gov/gene/?term=320355
Actr1a	Actr1a	ARP1 actin-related protein 1A, centractin alpha	54130	https://www.ncbi.nlm.nih.gov/gene/?term=54130
Abcd2	Abcd2	ATP-binding cassette, sub-family D (ALD), member 2	26874	https://www.ncbi.nlm.nih.gov/gene/?term=26874
Terf1	Terf1	telomeric repeat binding factor 1	21749	https://www.ncbi.nlm.nih.gov/gene/?term=21749
Znrf1	Znrf1	zinc and ring finger 1	170737	https://www.ncbi.nlm.nih.gov/gene/?term=170737
Atp6v0d1	Atp6v0d1	ATPase, H+ transporting, lysosomal V0 subunit D1	11972	https://www.ncbi.nlm.nih.gov/gene/?term=11972
Exoc6	Exoc6	exocyst complex component 6	107371	https://www.ncbi.nlm.nih.gov/gene/?term=107371
Trpm7	Trpm7	transient receptor potential cation channel, subfamily M, member 7	58800	https://www.ncbi.nlm.nih.gov/gene/?term=58800
Tcfl5	Tcfl5	transcription factor-like 5 (basic helix-loop-helix)	277353	https://www.ncbi.nlm.nih.gov/gene/?term=277353
Taok1	Taok1	TAO kinase 1	216965	https://www.ncbi.nlm.nih.gov/gene/?term=216965
Stim2	Stim2	stromal interaction molecule 2	116873	https://www.ncbi.nlm.nih.gov/gene/?term=116873
Lin28a	Lin28a	lin-28 homolog A (C. elegans)	83557	https://www.ncbi.nlm.nih.gov/gene/?term=83557

Biology Open • Supplementary information

Glcci1	Glcci1	glucocorticoid induced transcript 1	170772	https://www.ncbi.nlm.nih.gov/gene/?term=170772
Lpgat1	Lpgat1	lysophosphatidylglycerol acyltransferase 1	226856	https://www.ncbi.nlm.nih.gov/gene/?term=226856
Rbm46	Rbm46	RNA binding motif protein 46	633285	https://www.ncbi.nlm.nih.gov/gene/?term=633285
Pcdh17	Pcdh17	protocadherin 17	219228	https://www.ncbi.nlm.nih.gov/gene/?term=219228
Stk35	Stk35	serine/threonine kinase 35	67333	https://www.ncbi.nlm.nih.gov/gene/?term=67333
Cfap61	Cfap61	cilia and flagella associated protein 61	78774	https://www.ncbi.nlm.nih.gov/gene/?term=78774
Dlg5	Dlg5	discs large MAGUK scaffold protein 5	71228	https://www.ncbi.nlm.nih.gov/gene/?term=71228
Khnyn	Khnyn	KH and NYN domain containing	219094	https://www.ncbi.nlm.nih.gov/gene/?term=219094
Pdss1	Pdss1	prenyl (solanesyl) diphosphate synthase, subunit 1	56075	https://www.ncbi.nlm.nih.gov/gene/?term=56075
Rnf157	Rnf157	ring finger protein 157	217340	https://www.ncbi.nlm.nih.gov/gene/?term=217340
Large1	Large1	LARGE xylosyl- and glucuronyltransferase 1	16795	https://www.ncbi.nlm.nih.gov/gene/?term=16795
Ankrd17	Ankrd17	ankyrin repeat domain 17	81702	https://www.ncbi.nlm.nih.gov/gene/?term=81702
Gldc	Gldc	glycine decarboxylase	104174	https://www.ncbi.nlm.nih.gov/gene/?term=104174
Fam160b1	Fam160b1	family with sequence similarity 160, member B1	226252	https://www.ncbi.nlm.nih.gov/gene/?term=226252
Calu	Calu	calumenin	12321	https://www.ncbi.nlm.nih.gov/gene/?term=12321
Tfdp1	Tfdp1	transcription factor Dp 1	21781	https://www.ncbi.nlm.nih.gov/gene/?term=21781
Grm3	Grm3	glutamate receptor, metabotropic 3	108069	https://www.ncbi.nlm.nih.gov/gene/?term=108069
Tcp11l2	Tcp11l2	t-complex 11 (mouse) like 2	216198	https://www.ncbi.nlm.nih.gov/gene/?term=216198
Dsc2	Dsc2	desmocollin 2	13506	https://www.ncbi.nlm.nih.gov/gene/?term=13506
Ppp1r18	Ppp1r18	protein phosphatase 1, regulatory subunit 18	76448	https://www.ncbi.nlm.nih.gov/gene/?term=76448

Biology Open • Supplementary information

Galnt7	Galnt7	polypeptide N-acetylgalactosaminyltransferase 7	108150	https://www.ncbi.nlm.nih.gov/gene/?term=108150
Htr4	Htr4	5 hydroxytryptamine (serotonin) receptor 4	15562	https://www.ncbi.nlm.nih.gov/gene/?term=15562
Pnkd	Pnkd	paroxysmal nonkinesiogenic dyskinesia	56695	https://www.ncbi.nlm.nih.gov/gene/?term=56695
Ppp2r1b	Ppp2r1b	protein phosphatase 2, regulatory subunit A, beta	73699	https://www.ncbi.nlm.nih.gov/gene/?term=73699
Mkrn3	Mkrn3	makorin, ring finger protein, 3	22652	https://www.ncbi.nlm.nih.gov/gene/?term=22652
Kdm3a	Kdm3a	lysine (K)-specific demethylase 3A	104263	https://www.ncbi.nlm.nih.gov/gene/?term=104263
Azin1	Azin1	antizyme inhibitor 1	54375	https://www.ncbi.nlm.nih.gov/gene/?term=54375
Picalm	Picalm	phosphatidylinositol binding clathrin assembly protein	233489	https://www.ncbi.nlm.nih.gov/gene/?term=233489
Nt5e	Nt5e	5' nucleotidase, ecto	23959	https://www.ncbi.nlm.nih.gov/gene/?term=23959
Sema6b	Sema6b	sema domain, transmembrane domain (TM), and cytoplasmic domain, (semaphorin) 6B	20359	https://www.ncbi.nlm.nih.gov/gene/?term=20359
Adamts3	Adamts3	a disintegrin-like and metallopeptidase (reprolysin type) with thrombospondin type 1 motif, 3	330119	https://www.ncbi.nlm.nih.gov/gene/?term=330119
Shoc2	Shoc2	Shoc2, leucine rich repeat scaffold protein	56392	https://www.ncbi.nlm.nih.gov/gene/?term=56392
Adra2a	Adra2a	adrenergic receptor, alpha 2a	11551	https://www.ncbi.nlm.nih.gov/gene/?term=11551
Asah1	Asah1	N-acylsphingosine amidohydrolase 1	11886	https://www.ncbi.nlm.nih.gov/gene/?term=11886
Snapin	Snapin	SNAP-associated protein	20615	https://www.ncbi.nlm.nih.gov/gene/?term=20615

Biology Open • Supplementary information

Capza1	Capza1	capping protein (actin filament) muscle Z-line, alpha 1	12340	https://www.ncbi.nlm.nih.gov/gene/?term=12340
Calcr	Calcr	calcitonin receptor	12311	https://www.ncbi.nlm.nih.gov/gene/?term=12311
Wdr82	Wdr82	WD repeat domain containing 82	77305	https://www.ncbi.nlm.nih.gov/gene/?term=77305
Abcc9	Abcc9	ATP-binding cassette, sub-family C (CFTR/MRP), member 9	20928	https://www.ncbi.nlm.nih.gov/gene/?term=20928
Siah2	Siah2	siah E3 ubiquitin protein ligase 2	20439	https://www.ncbi.nlm.nih.gov/gene/?term=20439
Sox9	Sox9	SRY (sex determining region Y)-box 9	20682	https://www.ncbi.nlm.nih.gov/gene/?term=20682
Ino80d	Ino80d	INO80 complex subunit D	227195	https://www.ncbi.nlm.nih.gov/gene/?term=227195
Chl1	Chl1	cell adhesion molecule L1-like	12661	https://www.ncbi.nlm.nih.gov/gene/?term=12661
R3hdm1	R3hdm1	R3H domain containing 1	226412	https://www.ncbi.nlm.nih.gov/gene/?term=226412
Nap1l5	Nap1l5	nucleosome assembly protein 1-like 5	58243	https://www.ncbi.nlm.nih.gov/gene/?term=58243
Mttp	Mttp	microsomal triglyceride transfer protein	17777	https://www.ncbi.nlm.nih.gov/gene/?term=17777
Csnk1a1	Csnk1a1	casein kinase 1, alpha 1	93687	https://www.ncbi.nlm.nih.gov/gene/?term=93687
Rasd1	Rasd1	RAS, dexamethasone-induced 1	19416	https://www.ncbi.nlm.nih.gov/gene/?term=19416
Runx2	Runx2	runt related transcription factor 2	12393	https://www.ncbi.nlm.nih.gov/gene/?term=12393
Fbxo45	Fbxo45	F-box protein 45	268882	https://www.ncbi.nlm.nih.gov/gene/?term=268882
Slc36a1	Slc36a1	solute carrier family 36 (proton/amino acid symporter), member 1	215335	https://www.ncbi.nlm.nih.gov/gene/?term=215335
Brd1	Brd1	bromodomain containing 1	223770	https://www.ncbi.nlm.nih.gov/gene/?term=223770
Mier3	Mier3	MIER family member 3	218613	https://www.ncbi.nlm.nih.gov/gene/?term=218613
Cysltr1	Cysltr1	cysteinyl leukotriene receptor 1	58861	https://www.ncbi.nlm.nih.gov/gene/?term=58861
Srsf7	Srsf7	serine/arginine-rich splicing factor 7	225027	https://www.ncbi.nlm.nih.gov/gene/?term=225027

Polr3e	Polr3e	polymerase (RNA) III (DNA directed) polypeptide E	26939	https://www.ncbi.nlm.nih.gov/gene/?term=26939
Ube2v2	Ube2v2	ubiquitin-conjugating enzyme E2 variant 2	70620	https://www.ncbi.nlm.nih.gov/gene/?term=70620
Ip6k3	Ip6k3	inositol hexaphosphate kinase 3	271424	https://www.ncbi.nlm.nih.gov/gene/?term=271424
Gatm	Gatm	glycine amidinotransferase (L-arginine:glycine amidinotransferase)	67092	https://www.ncbi.nlm.nih.gov/gene/?term=67092
Gjc2	Gjc2	gap junction protein, gamma 2	118454	https://www.ncbi.nlm.nih.gov/gene/?term=118454
Afap1l2	Afap1l2	actin filament associated protein 1-like 2	226250	https://www.ncbi.nlm.nih.gov/gene/?term=226250
Hic2	Hic2	hypermethylated in cancer 2	58180	https://www.ncbi.nlm.nih.gov/gene/?term=58180
Scn9a	Scn9a	sodium channel, voltage-gated, type IX, alpha	20274	https://www.ncbi.nlm.nih.gov/gene/?term=20274
Mcf2l	Mcf2l	mcf.2 transforming sequence-like	17207	https://www.ncbi.nlm.nih.gov/gene/?term=17207
Map3k5	Map3k5	mitogen-activated protein kinase kinase kinase 5	26408	https://www.ncbi.nlm.nih.gov/gene/?term=26408
Ano4	Ano4	anoctamin 4	320091	https://www.ncbi.nlm.nih.gov/gene/?term=320091
Klhl20	Klhl20	kelch-like 20	226541	https://www.ncbi.nlm.nih.gov/gene/?term=226541
Chka	Chka	choline kinase alpha	12660	https://www.ncbi.nlm.nih.gov/gene/?term=12660
Samd8	Samd8	sterile alpha motif domain containing 8	67630	https://www.ncbi.nlm.nih.gov/gene/?term=67630
Slc7a10	Slc7a10	solute carrier family 7 (cationic amino acid transporter, y+ system), member 10	53896	https://www.ncbi.nlm.nih.gov/gene/?term=53896
Ankhd1	Ankhd1	ankyrin repeat and KH domain containing 1	108857	https://www.ncbi.nlm.nih.gov/gene/?term=108857

Biology Open • Supplementary information

Cadps	Cadps	Ca2+-dependent secretion activator	27062	https://www.ncbi.nlm.nih.gov/gene/?term=27062
Scara5	Scara5	scavenger receptor class A, member 5	71145	https://www.ncbi.nlm.nih.gov/gene/?term=71145
Cpsf6	Cpsf6	cleavage and polyadenylation specific factor 6	432508	https://www.ncbi.nlm.nih.gov/gene/?term=432508
Rfx6	Rfx6	regulatory factor X, 6	320995	https://www.ncbi.nlm.nih.gov/gene/?term=320995
Foxg1	Foxg1	forkhead box G1	15228	https://www.ncbi.nlm.nih.gov/gene/?term=15228
Ppp3r1	Ppp3r1	protein phosphatase 3, regulatory subunit B, alpha isoform (calcineurin B, type I)	19058	https://www.ncbi.nlm.nih.gov/gene/?term=19058
Fam199x	Fam199x	family with sequence similarity 199, X-linked	245622	https://www.ncbi.nlm.nih.gov/gene/?term=245622
Setd5	Setd5	SET domain containing 5	72895	https://www.ncbi.nlm.nih.gov/gene/?term=72895
Stk39	Stk39	serine/threonine kinase 39	53416	https://www.ncbi.nlm.nih.gov/gene/?term=53416
Socs3	Socs3	suppressor of cytokine signaling 3	12702	https://www.ncbi.nlm.nih.gov/gene/?term=12702
Ago3	Ago3	argonaute RISC catalytic subunit 3	214150	https://www.ncbi.nlm.nih.gov/gene/?term=214150
Six4	Six4	sine oculis-related homeobox 4	20474	https://www.ncbi.nlm.nih.gov/gene/?term=20474
Slc35f4	Slc35f4	solute carrier family 35, member F4	75288	https://www.ncbi.nlm.nih.gov/gene/?term=75288
Actc1	Actc1	actin, alpha, cardiac muscle 1	11464	https://www.ncbi.nlm.nih.gov/gene/?term=11464
Rgs8	Rgs8	regulator of G-protein signaling 8	67792	https://www.ncbi.nlm.nih.gov/gene/?term=67792
Tbc1d15	Tbc1d15	TBC1 domain family, member 15	66687	https://www.ncbi.nlm.nih.gov/gene/?term=66687
Nfatc3	Nfatc3	nuclear factor of activated T cells, cytoplasmic, calcineurin dependent 3	18021	https://www.ncbi.nlm.nih.gov/gene/?term=18021
Capn7	Capn7	calpain 7	12339	https://www.ncbi.nlm.nih.gov/gene/?term=12339
Nr6a1	Nr6a1	nuclear receptor subfamily 6, group A, member 1	14536	https://www.ncbi.nlm.nih.gov/gene/?term=14536

Biology Open • Supplementary information

Fbxo32	Fbxo32	F-box protein 32	67731	https://www.ncbi.nlm.nih.gov/gene/?term=67731
Cnot9	Cnot9	CCR4-NOT transcription complex, subunit 9	58184	https://www.ncbi.nlm.nih.gov/gene/?term=58184
Ednra	Ednra	endothelin receptor type A	13617	https://www.ncbi.nlm.nih.gov/gene/?term=13617
Atg3	Atg3	autophagy related 3	67841	https://www.ncbi.nlm.nih.gov/gene/?term=67841
Map3k12	Map3k12	mitogen-activated protein kinase kinase kinase 12	26404	https://www.ncbi.nlm.nih.gov/gene/?term=26404
Ifi213	Ifi213	interferon activated gene 213	623121	https://www.ncbi.nlm.nih.gov/gene/?term=623121
Ankrd55	Ankrd55	ankyrin repeat domain 55	77318	https://www.ncbi.nlm.nih.gov/gene/?term=77318
Ttbk1	Ttbk1	tau tubulin kinase 1	106763	https://www.ncbi.nlm.nih.gov/gene/?term=106763
Csnk1g1	Csnk1g1	casein kinase 1, gamma 1	214897	https://www.ncbi.nlm.nih.gov/gene/?term=214897
Ppid	Ppid	peptidylprolyl isomerase D (cyclophilin D)	67738	https://www.ncbi.nlm.nih.gov/gene/?term=67738
Ror1	Ror1	receptor tyrosine kinase-like orphan receptor 1	26563	https://www.ncbi.nlm.nih.gov/gene/?term=26563
Chd1	Chd1	chromodomain helicase DNA binding protein 1	12648	https://www.ncbi.nlm.nih.gov/gene/?term=12648
Frmpd1	Frmpd1	FERM and PDZ domain containing 1	666060	https://www.ncbi.nlm.nih.gov/gene/?term=666060
Gzf1	Gzf1	GDNF-inducible zinc finger protein 1	74533	https://www.ncbi.nlm.nih.gov/gene/?term=74533
Ppargc1b	Ppargc1b	peroxisome proliferative activated receptor, gamma, coactivator 1 beta	170826	https://www.ncbi.nlm.nih.gov/gene/?term=170826
Ythdf3	Ythdf3	YTH N6-methyladenosine RNA binding protein 3	229096	https://www.ncbi.nlm.nih.gov/gene/?term=229096
Sypl	Sypl	synaptophysin-like protein	19027	https://www.ncbi.nlm.nih.gov/gene/?term=19027
Map3k21	Map3k21	mitogen-activated protein kinase kinase kinase 21	234878	https://www.ncbi.nlm.nih.gov/gene/?term=234878

Biology Open • Supplementary information

Rai14	Rai14	retinoic acid induced 14	75646	https://www.ncbi.nlm.nih.gov/gene/?term=75646
Ppp1r1c	Ppp1r1c	protein phosphatase 1, regulatory inhibitor subunit 1C	75276	https://www.ncbi.nlm.nih.gov/gene/?term=75276
Cth	Cth	cystathionase (cystathionine gamma-lyase)	107869	https://www.ncbi.nlm.nih.gov/gene/?term=107869
Slc38a7	Slc38a7	solute carrier family 38, member 7	234595	https://www.ncbi.nlm.nih.gov/gene/?term=234595
Mtdh	Mtdh	metadherin	67154	https://www.ncbi.nlm.nih.gov/gene/?term=67154
Pde7a	Pde7a	phosphodiesterase 7A	18583	https://www.ncbi.nlm.nih.gov/gene/?term=18583
Ppp3ca	Ppp3ca	protein phosphatase 3, catalytic subunit, alpha isoform	19055	https://www.ncbi.nlm.nih.gov/gene/?term=19055
Extl2	Extl2	exostoses (multiple)-like 2	58193	https://www.ncbi.nlm.nih.gov/gene/?term=58193
Polr3g	Polr3g	polymerase (RNA) III (DNA directed) polypeptide G	67486	https://www.ncbi.nlm.nih.gov/gene/?term=67486
Sh3rf1	Sh3rf1	SH3 domain containing ring finger 1	59009	https://www.ncbi.nlm.nih.gov/gene/?term=59009
Erich5	Erich5	glutamate rich 5	239368	https://www.ncbi.nlm.nih.gov/gene/?term=239368
Jph4	Jph4	junctophilin 4	319984	https://www.ncbi.nlm.nih.gov/gene/?term=319984
Tbl1xr1	Tbl1xr1	transducin (beta)-like 1X-linked receptor 1	81004	https://www.ncbi.nlm.nih.gov/gene/?term=81004
Katnbl1	Katnbl1	katanin p80 subunit B like 1	72425	https://www.ncbi.nlm.nih.gov/gene/?term=72425
Cep41	Cep41	centrosomal protein 41	83922	https://www.ncbi.nlm.nih.gov/gene/?term=83922
Xpo1	Xpo1	exportin 1	103573	https://www.ncbi.nlm.nih.gov/gene/?term=103573
C9orf72	C9orf72	C9orf72, member of C9orf72-SMCR8 complex	73205	https://www.ncbi.nlm.nih.gov/gene/?term=73205
Pip4k2a	Pip4k2a	phosphatidylinositol-5-phosphate 4-kinase, type II, alpha	18718	https://www.ncbi.nlm.nih.gov/gene/?term=18718
Tbc1d10b	Tbc1d10b	TBC1 domain family, member 10b	68449	https://www.ncbi.nlm.nih.gov/gene/?term=68449
Rasgef1a	Rasgef1a	RasGEF domain family, member 1A	70727	https://www.ncbi.nlm.nih.gov/gene/?term=70727
Klf9	Klf9	Kruppel-like factor 9	16601	https://www.ncbi.nlm.nih.gov/gene/?term=16601

Biology Open • Supplementary information

Rimbp2	Rimbp2	RIMS binding protein 2	231760	https://www.ncbi.nlm.nih.gov/gene/?term=231760
Necap1	Necap1	NECAP endocytosis associated 1	67602	https://www.ncbi.nlm.nih.gov/gene/?term=67602
Elavl2	Elavl2	ELAV like RNA binding protein 1	15569	https://www.ncbi.nlm.nih.gov/gene/?term=15569
Nfat5	Nfat5	nuclear factor of activated T cells 5	54446	https://www.ncbi.nlm.nih.gov/gene/?term=54446
Reep3	Reep3	receptor accessory protein 3	28193	https://www.ncbi.nlm.nih.gov/gene/?term=28193
Slc35d3	Slc35d3	solute carrier family 35, member D3	76157	https://www.ncbi.nlm.nih.gov/gene/?term=76157
Slc4a7	Slc4a7	solute carrier family 4, sodium bicarbonate cotransporter, member 7	218756	https://www.ncbi.nlm.nih.gov/gene/?term=218756
Lpp	Lpp	LIM domain containing preferred translocation partner in lipoma	210126	https://www.ncbi.nlm.nih.gov/gene/?term=210126
Lrrk2	Lrrk2	leucine-rich repeat kinase 2	66725	https://www.ncbi.nlm.nih.gov/gene/?term=66725
Tmod2	Tmod2	tropomodulin 2	50876	https://www.ncbi.nlm.nih.gov/gene/?term=50876
Unc5c	Unc5c	unc-5 netrin receptor C	22253	https://www.ncbi.nlm.nih.gov/gene/?term=22253
Cep170	Cep170	centrosomal protein 170	545389	https://www.ncbi.nlm.nih.gov/gene/?term=545389
Nabp1	Nabp1	nucleic acid binding protein 1	109019	https://www.ncbi.nlm.nih.gov/gene/?term=109019
Wdr7	Wdr7	WD repeat domain 7	104082	https://www.ncbi.nlm.nih.gov/gene/?term=104082
Rfx7	Rfx7	regulatory factor X, 7	319758	https://www.ncbi.nlm.nih.gov/gene/?term=319758
Sptssb	Sptssb	serine palmitoyltransferase, small subunit B	66183	https://www.ncbi.nlm.nih.gov/gene/?term=66183
Epb41l3	Epb41l3	erythrocyte membrane protein band 4.1 like 3	13823	https://www.ncbi.nlm.nih.gov/gene/?term=13823
Ccnt2	Ccnt2	cyclin T2	72949	https://www.ncbi.nlm.nih.gov/gene/?term=72949
Dll4	Dll4	delta like canonical Notch ligand 4	54485	https://www.ncbi.nlm.nih.gov/gene/?term=54485
Cdc37l1	Cdc37l1	cell division cycle 37-like 1	67072	https://www.ncbi.nlm.nih.gov/gene/?term=67072
Tecrl	Tecrl	trans-2,3-enoyl-CoA reductase-like	243078	https://www.ncbi.nlm.nih.gov/gene/?term=243078

Biology Open • Supplementary information

Rapgef2	Rapgef2	Rap guanine nucleotide exchange factor (GEF) 2	76089	https://www.ncbi.nlm.nih.gov/gene/?term=76089
Gigyf2	Gigyf2	GRB10 interacting GYF protein 2	227331	https://www.ncbi.nlm.nih.gov/gene/?term=227331
Avl9	Avl9	AVL9 cell migration associated	78937	https://www.ncbi.nlm.nih.gov/gene/?term=78937
Prkaa2	Prkaa2	protein kinase, AMP-activated, alpha 2 catalytic subunit	108079	https://www.ncbi.nlm.nih.gov/gene/?term=108079
Rnf122	Rnf122	ring finger protein 122	68867	https://www.ncbi.nlm.nih.gov/gene/?term=68867
Eed	Eed	embryonic ectoderm development	13626	https://www.ncbi.nlm.nih.gov/gene/?term=13626
Gria2	Gria2	glutamate receptor, ionotropic, AMPA2 (alpha 2)	14800	https://www.ncbi.nlm.nih.gov/gene/?term=14800
Myh11	Myh11	myosin, heavy polypeptide 11, smooth muscle	17880	https://www.ncbi.nlm.nih.gov/gene/?term=17880
Rab32	Rab32	RAB32, member RAS oncogene family	67844	https://www.ncbi.nlm.nih.gov/gene/?term=67844
Gabrb1	Gabrb1	gamma-aminobutyric acid (GABA) A receptor, subunit beta 1	14400	https://www.ncbi.nlm.nih.gov/gene/?term=14400
Ascc3	Ascc3	activating signal cointegrator 1 complex subunit 3	77987	https://www.ncbi.nlm.nih.gov/gene/?term=77987
Klf8	Klf8	Kruppel-like factor 8	245671	https://www.ncbi.nlm.nih.gov/gene/?term=245671
Ankra2	Ankra2	ankyrin repeat, family A (RFXANK-like), 2	68558	https://www.ncbi.nlm.nih.gov/gene/?term=68558
Rora	Rora	RAR-related orphan receptor alpha	19883	https://www.ncbi.nlm.nih.gov/gene/?term=19883
Erlin1	Erlin1	ER lipid raft associated 1	226144	https://www.ncbi.nlm.nih.gov/gene/?term=226144
Zfp507	Zfp507	zinc finger protein 507	668501	https://www.ncbi.nlm.nih.gov/gene/?term=668501
Hnrnpul2	Hnrnpul2	heterogeneous nuclear ribonucleoprotein U-like 2	68693	https://www.ncbi.nlm.nih.gov/gene/?term=68693
Skp2	Skp2	S-phase kinase-associated protein 2 (p45)	27401	https://www.ncbi.nlm.nih.gov/gene/?term=27401

Klf10	Klf10	Kruppel-like factor 10	21847	https://www.ncbi.nlm.nih.gov/gene/?term=21847
Bnc1	Bnc1	basonuclin 1	12173	https://www.ncbi.nlm.nih.gov/gene/?term=12173
Scn3a	Scn3a	sodium channel, voltage-gated, type III, alpha	20269	https://www.ncbi.nlm.nih.gov/gene/?term=20269
Zfp608	Zfp608	zinc finger protein 608	269023	https://www.ncbi.nlm.nih.gov/gene/?term=269023
Vat1	Vat1	vesicle amine transport 1	26949	https://www.ncbi.nlm.nih.gov/gene/?term=26949
Kcna4	Kcna4	potassium voltage-gated channel, shaker-related subfamily, member 4	16492	https://www.ncbi.nlm.nih.gov/gene/?term=16492
Snx33	Snx33	sorting nexin 33	235406	https://www.ncbi.nlm.nih.gov/gene/?term=235406
Prlr	Prlr	prolactin receptor	19116	https://www.ncbi.nlm.nih.gov/gene/?term=19116
Rundc3b	Rundc3b	RUN domain containing 3B	242819	https://www.ncbi.nlm.nih.gov/gene/?term=242819
Gnai2	Gnai2	guanine nucleotide binding protein (G protein), alpha inhibiting 2	14678	https://www.ncbi.nlm.nih.gov/gene/?term=14678
Socs1	Socs1	suppressor of cytokine signaling 1	12703	https://www.ncbi.nlm.nih.gov/gene/?term=12703
Plch1	Plch1	phospholipase C, eta 1	269437	https://www.ncbi.nlm.nih.gov/gene/?term=269437
Snx18	Snx18	sorting nexin 18	170625	https://www.ncbi.nlm.nih.gov/gene/?term=170625
Zfp518a	Zfp518a	zinc finger protein 518A	72672	https://www.ncbi.nlm.nih.gov/gene/?term=72672
Slc15a2	Slc15a2	solute carrier family 15 (H+/peptide transporter), member 2	57738	https://www.ncbi.nlm.nih.gov/gene/?term=57738
Gabra5	Gabra5	gamma-aminobutyric acid (GABA) A receptor, subunit alpha 5	110886	https://www.ncbi.nlm.nih.gov/gene/?term=110886
Rrad	Rrad	Ras-related associated with diabetes	56437	https://www.ncbi.nlm.nih.gov/gene/?term=56437
Hdac9	Hdac9	histone deacetylase 9	79221	https://www.ncbi.nlm.nih.gov/gene/?term=79221
Prps1l3	Prps1l3	phosphoribosyl pyrophosphate synthetase 1-like 3	328099	https://www.ncbi.nlm.nih.gov/gene/?term=328099

Twf1	Twf1	twinfilin actin binding protein 1	19230	https://www.ncbi.nlm.nih.gov/gene/?term=19230
Zdhhc20	Zdhhc20	zinc finger, DHHC domain containing 20	75965	https://www.ncbi.nlm.nih.gov/gene/?term=75965
Larp4	Larp4	La ribonucleoprotein domain family, member 4	207214	https://www.ncbi.nlm.nih.gov/gene/?term=207214
Runx1	Runx1	runt related transcription factor 1	12394	https://www.ncbi.nlm.nih.gov/gene/?term=12394
Arhgef6	Arhgef6	Rac/Cdc42 guanine nucleotide exchange factor (GEF) 6	73341	https://www.ncbi.nlm.nih.gov/gene/?term=73341
Slc35c1	Slc35c1	solute carrier family 35, member C1	228368	https://www.ncbi.nlm.nih.gov/gene/?term=228368
Chst2	Chst2	carbohydrate sulfotransferase 2	54371	https://www.ncbi.nlm.nih.gov/gene/?term=54371
Sdad1	Sdad1	SDA1 domain containing 1	231452	https://www.ncbi.nlm.nih.gov/gene/?term=231452
Ppp4r4	Ppp4r4	protein phosphatase 4, regulatory subunit 4	74521	https://www.ncbi.nlm.nih.gov/gene/?term=74521
Pik3cd	Pik3cd	phosphatidylinositol-4,5-bisphosphate 3-kinase catalytic subunit delta	18707	https://www.ncbi.nlm.nih.gov/gene/?term=18707
Ttll7	Ttll7	tubulin tyrosine ligase-like family, member 7	70892	https://www.ncbi.nlm.nih.gov/gene/?term=70892
Col13a1	Col13a1	collagen, type XIII, alpha 1	12817	https://www.ncbi.nlm.nih.gov/gene/?term=12817
Zfp560	Zfp560	zinc finger protein 560	434377	https://www.ncbi.nlm.nih.gov/gene/?term=434377
Vopp1	Vopp1	vesicular, overexpressed in cancer, prosurvival protein 1	232023	https://www.ncbi.nlm.nih.gov/gene/?term=232023
Evx2	Evx2	even-skipped homeobox 2	14029	https://www.ncbi.nlm.nih.gov/gene/?term=14029
Lrfn2	Lrfn2	leucine rich repeat and fibronectin type III domain containing 2	70530	https://www.ncbi.nlm.nih.gov/gene/?term=70530
Plagl2	Plagl2	pleiomorphic adenoma gene-like 2	54711	https://www.ncbi.nlm.nih.gov/gene/?term=54711
Myo9b	Myo9b	myosin IXb	17925	https://www.ncbi.nlm.nih.gov/gene/?term=17925

Biology Open • Supplementary information

Gtf2h4	Gtf2h4	general transcription factor II H, polypeptide 4	14885	https://www.ncbi.nlm.nih.gov/gene/?term=14885
Edem3	Edem3	ER degradation enhancer, mannosidase alpha-like 3	66967	https://www.ncbi.nlm.nih.gov/gene/?term=66967
Asb3	Asb3	ankyrin repeat and SOCS box-containing 3	65257	https://www.ncbi.nlm.nih.gov/gene/?term=65257
Trip12	Trip12	thyroid hormone receptor interactor 12	14897	https://www.ncbi.nlm.nih.gov/gene/?term=14897
Mfsd6	Mfsd6	major facilitator superfamily domain containing 6	98682	https://www.ncbi.nlm.nih.gov/gene/?term=98682
Tmem181a	Tmem181a	transmembrane protein 181A	77106	https://www.ncbi.nlm.nih.gov/gene/?term=77106
Lin28b	Lin28b	lin-28 homolog B (C. elegans)	380669	https://www.ncbi.nlm.nih.gov/gene/?term=380669
Ptpn13	Ptpn13	protein tyrosine phosphatase, non-receptor type 13	19249	https://www.ncbi.nlm.nih.gov/gene/?term=19249
Celsr3	Celsr3	cadherin, EGF LAG seven-pass G-type receptor 3	107934	https://www.ncbi.nlm.nih.gov/gene/?term=107934
Cyp24a1	Cyp24a1	cytochrome P450, family 24, subfamily a, polypeptide 1	13081	https://www.ncbi.nlm.nih.gov/gene/?term=13081
Scn2a	Scn2a	sodium channel, voltage-gated, type II, alpha	110876	https://www.ncbi.nlm.nih.gov/gene/?term=110876
Slc35f1	Slc35f1	solute carrier family 35, member F1	215085	https://www.ncbi.nlm.nih.gov/gene/?term=215085
Rnf220	Rnf220	ring finger protein 220	66743	https://www.ncbi.nlm.nih.gov/gene/?term=66743
Mbtps2	Mbtps2	membrane-bound transcription factor peptidase, site 2	270669	https://www.ncbi.nlm.nih.gov/gene/?term=270669
Rab38	Rab38	RAB38, member RAS oncogene family	72433	https://www.ncbi.nlm.nih.gov/gene/?term=72433
Ddah1	Ddah1	dimethylarginine dimethylaminohydrolase 1	69219	https://www.ncbi.nlm.nih.gov/gene/?term=69219

Biology Open • Supplementary information

Galnt1	Galnt1	polypeptide N-acetylgalactosaminyltransferase 1	14423	https://www.ncbi.nlm.nih.gov/gene/?term=14423
Pawr	Pawr	PRKC, apoptosis, WT1, regulator	114774	https://www.ncbi.nlm.nih.gov/gene/?term=114774
Six1	Six1	sine oculis-related homeobox 1	20471	https://www.ncbi.nlm.nih.gov/gene/?term=20471
Arid5b	Arid5b	AT rich interactive domain 5B (MRF1-like)	71371	https://www.ncbi.nlm.nih.gov/gene/?term=71371
Frzb	Frzb	frizzled-related protein	20378	https://www.ncbi.nlm.nih.gov/gene/?term=20378
Lhx8	Lhx8	LIM homeobox protein 8	16875	https://www.ncbi.nlm.nih.gov/gene/?term=16875
Dolpp1	Dolpp1	dolichyl pyrophosphate phosphatase 1	57170	https://www.ncbi.nlm.nih.gov/gene/?term=57170
Eml1	Eml1	echinoderm microtubule associated protein like 1	68519	https://www.ncbi.nlm.nih.gov/gene/?term=68519
Adgra3	Adgra3	adhesion G protein-coupled receptor A3	70693	https://www.ncbi.nlm.nih.gov/gene/?term=70693
Prdm1	Prdm1	PR domain containing 1, with ZNF domain	12142	https://www.ncbi.nlm.nih.gov/gene/?term=12142
Bahd1	Bahd1	bromo adjacent homology domain containing 1	228536	https://www.ncbi.nlm.nih.gov/gene/?term=228536
Ppp1r12a	Ppp1r12a	protein phosphatase 1, regulatory subunit 12A	17931	https://www.ncbi.nlm.nih.gov/gene/?term=17931
Ypel5	Ypel5	yippee like 5	383295	https://www.ncbi.nlm.nih.gov/gene/?term=383295
Esyt3	Esyt3	extended synaptotagmin-like protein 3	272636	https://www.ncbi.nlm.nih.gov/gene/?term=272636
Ap4e1	Ap4e1	adaptor-related protein complex AP-4, epsilon 1	108011	https://www.ncbi.nlm.nih.gov/gene/?term=108011
Reep1	Reep1	receptor accessory protein 1	52250	https://www.ncbi.nlm.nih.gov/gene/?term=52250
Terb2	Terb2	telomere repeat binding bouquet formation protein 2	74401	https://www.ncbi.nlm.nih.gov/gene/?term=74401

Biology Open • Supplementary information

Plekhm3	Plekhm3	pleckstrin homology domain containing, family M, member 3	241075	https://www.ncbi.nlm.nih.gov/gene/?term=241075
Srek1	Srek1	splicing regulatory glutamine/lysine-rich protein 1	218543	https://www.ncbi.nlm.nih.gov/gene/?term=218543
Dclk1	Dclk1	doublecortin-like kinase 1	13175	https://www.ncbi.nlm.nih.gov/gene/?term=13175
Aebp2	Aebp2	AE binding protein 2	11569	https://www.ncbi.nlm.nih.gov/gene/?term=11569
Zfp516	Zfp516	zinc finger protein 516	329003	https://www.ncbi.nlm.nih.gov/gene/?term=329003
Slc7a15	Slc7a15	solute carrier family 7 (cationic amino acid transporter, y+ system), member 15	328059	https://www.ncbi.nlm.nih.gov/gene/?term=328059
Sufu	Sufu	SUFU negative regulator of hedgehog signaling	24069	https://www.ncbi.nlm.nih.gov/gene/?term=24069
Zfp120	Zfp120	zinc finger protein 120	104348	https://www.ncbi.nlm.nih.gov/gene/?term=104348
Mindy4	Mindy4	MINDY lysine 48 deubiquitinase 4	330323	https://www.ncbi.nlm.nih.gov/gene/?term=330323
Cnot2	Cnot2	CCR4-NOT transcription complex, subunit 2	72068	https://www.ncbi.nlm.nih.gov/gene/?term=72068
Ctbp2	Ctbp2	C-terminal binding protein 2	13017	https://www.ncbi.nlm.nih.gov/gene/?term=13017
Laptm5	Laptm5	lysosomal-associated protein transmembrane 5	16792	https://www.ncbi.nlm.nih.gov/gene/?term=16792
Ccdc150	Ccdc150	coiled-coil domain containing 150	78016	https://www.ncbi.nlm.nih.gov/gene/?term=78016
Sorcs2	Sorcs2	sortilin-related VPS10 domain containing receptor 2	81840	https://www.ncbi.nlm.nih.gov/gene/?term=81840
Dhx58	Dhx58	DEXH (Asp-Glu-X-His) box polypeptide 58	80861	https://www.ncbi.nlm.nih.gov/gene/?term=80861
Slc40a1	Slc40a1	solute carrier family 40 (iron-regulated transporter), member 1	53945	https://www.ncbi.nlm.nih.gov/gene/?term=53945
Poldip2	Poldip2	polymerase (DNA-directed), delta interacting protein 2	67811	https://www.ncbi.nlm.nih.gov/gene/?term=67811

Biology Open • Supplementary information

Col12a1	Col12a1	collagen, type XII, alpha 1	12816	https://www.ncbi.nlm.nih.gov/gene/?term=12816
Zfp799	Zfp799	zinc finger protein 799	240064	https://www.ncbi.nlm.nih.gov/gene/?term=240064
BC052040	BC052040	cDNA sequence BC052040	399568	https://www.ncbi.nlm.nih.gov/gene/?term=399568
Grm5	Grm5	glutamate receptor, metabotropic 5	108071	https://www.ncbi.nlm.nih.gov/gene/?term=108071
Rit1	Rit1	Ras-like without CAAX 1	19769	https://www.ncbi.nlm.nih.gov/gene/?term=19769
Pan3	Pan3	PAN3 poly(A) specific ribonuclease subunit	72587	https://www.ncbi.nlm.nih.gov/gene/?term=72587
Pnrc2	Pnrc2	proline-rich nuclear receptor coactivator 2	52830	https://www.ncbi.nlm.nih.gov/gene/?term=52830
Syne2	Syne2	spectrin repeat containing, nuclear envelope 2	319565	https://www.ncbi.nlm.nih.gov/gene/?term=319565
Igfbp7	Igfbp7	insulin-like growth factor binding protein 7	29817	https://www.ncbi.nlm.nih.gov/gene/?term=29817
Srpr	Srpr	signal recognition particle receptor ('docking protein')	67398	https://www.ncbi.nlm.nih.gov/gene/?term=67398
Kpna3	Kpna3	karyopherin (importin) alpha 3	16648	https://www.ncbi.nlm.nih.gov/gene/?term=16648
Phf1	Phf1	PHD finger protein 1	21652	https://www.ncbi.nlm.nih.gov/gene/?term=21652
Kif23	Kif23	kinesin family member 23	71819	https://www.ncbi.nlm.nih.gov/gene/?term=71819
Pnisr	Pnisr	PNN interacting serine/arginine-rich	66625	https://www.ncbi.nlm.nih.gov/gene/?term=66625
Lix1l	Lix1l	Lix1-like	280411	https://www.ncbi.nlm.nih.gov/gene/?term=280411
Npy5r	Npy5r	neuropeptide Y receptor Y5	18168	https://www.ncbi.nlm.nih.gov/gene/?term=18168
Bmp7	Bmp7	bone morphogenetic protein 7	12162	https://www.ncbi.nlm.nih.gov/gene/?term=12162
Slc12a2	Slc12a2	solute carrier family 12, member 2	20496	https://www.ncbi.nlm.nih.gov/gene/?term=20496
Igf2r	Igf2r	insulin-like growth factor 2 receptor	16004	https://www.ncbi.nlm.nih.gov/gene/?term=16004

Biology Open • Supplementary information

Ing2	Ing2	inhibitor of growth family, member 2	69260	https://www.ncbi.nlm.nih.gov/gene/?term=69260
Luc7l3	Luc7l3	LUC7-like 3 (S. cerevisiae)	67684	https://www.ncbi.nlm.nih.gov/gene/?term=67684
Celsr2	Celsr2	cadherin, EGF LAG seven-pass G-type receptor 2	53883	https://www.ncbi.nlm.nih.gov/gene/?term=53883
Dctn4	Dctn4	dynactin 4	67665	https://www.ncbi.nlm.nih.gov/gene/?term=67665
Bcar3	Bcar3	breast cancer anti-estrogen resistance 3	29815	https://www.ncbi.nlm.nih.gov/gene/?term=29815
G3bp2	G3bp2	GTPase activating protein (SH3 domain) binding protein 2	23881	https://www.ncbi.nlm.nih.gov/gene/?term=23881
Ppp2r5e	Ppp2r5e	protein phosphatase 2, regulatory subunit B', epsilon	26932	https://www.ncbi.nlm.nih.gov/gene/?term=26932
Gna12	Gna12	guanine nucleotide binding protein, alpha 12	14673	https://www.ncbi.nlm.nih.gov/gene/?term=14673
Adamtsl3	Adamtsl3	ADAMTS-like 3	269959	https://www.ncbi.nlm.nih.gov/gene/?term=269959
Serpine2	Serpine2	serine (or cysteine) peptidase inhibitor, clade E, member 2	20720	https://www.ncbi.nlm.nih.gov/gene/?term=20720
Tubgcp3	Tubgcp3	tubulin, gamma complex associated protein 3	259279	https://www.ncbi.nlm.nih.gov/gene/?term=259279
Sema3a	Sema3a	sema domain, immunoglobulin domain (Ig), short basic domain, secreted, (semaphorin) 3A	20346	https://www.ncbi.nlm.nih.gov/gene/?term=20346
Erbb4	Erbb4	erb-b2 receptor tyrosine kinase 4	13869	https://www.ncbi.nlm.nih.gov/gene/?term=13869
Itga3	Itga3	integrin alpha 3	16400	https://www.ncbi.nlm.nih.gov/gene/?term=16400
Tgif2	Tgif2	TGFB-induced factor homeobox 2	228839	https://www.ncbi.nlm.nih.gov/gene/?term=228839
Rb1	Rb1	RB transcriptional corepressor 1	19645	https://www.ncbi.nlm.nih.gov/gene/?term=19645
Mucl2	Mucl2	mucin-like 2	20770	https://www.ncbi.nlm.nih.gov/gene/?term=20770
Pak4	Pak4	p21 (RAC1) activated kinase 4	70584	https://www.ncbi.nlm.nih.gov/gene/?term=70584

Biology Open • Supplementary information

Aplp2	Aplp2	amyloid beta (A4) precursor-like protein 2	11804	https://www.ncbi.nlm.nih.gov/gene/?term=11804
4933434E20Rik	4933434E20Rik	RIKEN cDNA 4933434E20 gene	99650	https://www.ncbi.nlm.nih.gov/gene/?term=99650
Raph1	Raph1	Ras association (RalGDS/AF-6) and pleckstrin homology domains 1	77300	https://www.ncbi.nlm.nih.gov/gene/?term=77300
Itgb8	Itgb8	integrin beta 8	320910	https://www.ncbi.nlm.nih.gov/gene/?term=320910
Rbbp6	Rbbp6	retinoblastoma binding protein 6, ubiquitin ligase	19647	https://www.ncbi.nlm.nih.gov/gene/?term=19647
Kdm6a	Kdm6a	lysine (K)-specific demethylase 6A	22289	https://www.ncbi.nlm.nih.gov/gene/?term=22289
Adam10	Adam10	a disintegrin and metallopeptidase domain 10	11487	https://www.ncbi.nlm.nih.gov/gene/?term=11487
Tmem62	Tmem62	transmembrane protein 62	96957	https://www.ncbi.nlm.nih.gov/gene/?term=96957
Cd151	Cd151	CD151 antigen	12476	https://www.ncbi.nlm.nih.gov/gene/?term=12476
Slc39a10	Slc39a10	solute carrier family 39 (zinc transporter), member 10	227059	https://www.ncbi.nlm.nih.gov/gene/?term=227059
Cdt1	Cdt1	chromatin licensing and DNA replication factor 1	67177	https://www.ncbi.nlm.nih.gov/gene/?term=67177
Rimklb	Rimklb	ribosomal modification protein rimK-like family member B	108653	https://www.ncbi.nlm.nih.gov/gene/?term=108653
Vstm4	Vstm4	V-set and transmembrane domain containing 4	320736	https://www.ncbi.nlm.nih.gov/gene/?term=320736
Mro	Mro	maestro	71263	https://www.ncbi.nlm.nih.gov/gene/?term=71263
Togaram2	Togaram2	TOG array regulator of axonemal microtubules 2	320159	https://www.ncbi.nlm.nih.gov/gene/?term=320159
Nckap1	Nckap1	NCK-associated protein 1	50884	https://www.ncbi.nlm.nih.gov/gene/?term=50884
Ppp1r9a	Ppp1r9a	protein phosphatase 1, regulatory subunit 9A	243725	https://www.ncbi.nlm.nih.gov/gene/?term=243725
Dpp8	Dpp8	dipeptidylpeptidase 8	74388	https://www.ncbi.nlm.nih.gov/gene/?term=74388

Biology Open • Supplementary information

Ehbp1	Ehbp1	EH domain binding protein 1	216565	https://www.ncbi.nlm.nih.gov/gene/?term=216565
Pls1	Pls1	plastin 1 (I-isoform)	102502	https://www.ncbi.nlm.nih.gov/gene/?term=102502
Ypel1	Ypel1	yippee like 1	106369	https://www.ncbi.nlm.nih.gov/gene/?term=106369
Ephb1	Ephb1	Eph receptor B1	270190	https://www.ncbi.nlm.nih.gov/gene/?term=270190
Muc15	Muc15	mucin 15	269328	https://www.ncbi.nlm.nih.gov/gene/?term=269328
Naa16	Naa16	N(alpha)-acetyltransferase 16, NatA auxiliary subunit	66897	https://www.ncbi.nlm.nih.gov/gene/?term=66897
Gpr158	Gpr158	G protein-coupled receptor 158	241263	https://www.ncbi.nlm.nih.gov/gene/?term=241263
Nckap5	Nckap5	NCK-associated protein 5	210356	https://www.ncbi.nlm.nih.gov/gene/?term=210356
Mettl21e	Mettl21e	methyltransferase like 21E	403183	https://www.ncbi.nlm.nih.gov/gene/?term=403183
Ggnbp2	Ggnbp2	gametogenetin binding protein 2	217039	https://www.ncbi.nlm.nih.gov/gene/?term=217039
Tceal8	Tceal8	transcription elongation factor A (SII)-like 8	66684	https://www.ncbi.nlm.nih.gov/gene/?term=66684
Notum	Notum	notum palmitoleoyl-protein carboxylesterase	77583	https://www.ncbi.nlm.nih.gov/gene/?term=77583
Me3	Me3	malic enzyme 3, NADP(+)-dependent, mitochondrial	109264	https://www.ncbi.nlm.nih.gov/gene/?term=109264
Edil3	Edil3	EGF-like repeats and discoidin I-like domains 3	13612	https://www.ncbi.nlm.nih.gov/gene/?term=13612
Mycbp	Mycbp	MYC binding protein	56309	https://www.ncbi.nlm.nih.gov/gene/?term=56309
Hspa4l	Hspa4l	heat shock protein 4 like	18415	https://www.ncbi.nlm.nih.gov/gene/?term=18415
Id4	Id4	inhibitor of DNA binding 4	15904	https://www.ncbi.nlm.nih.gov/gene/?term=15904
Fam160b2	Fam160b2	family with sequence similarity 160, member B2	239170	https://www.ncbi.nlm.nih.gov/gene/?term=239170
Far1	Far1	fatty acyl CoA reductase 1	67420	https://www.ncbi.nlm.nih.gov/gene/?term=67420
Tfdp2	Tfdp2	transcription factor Dp 2	211586	https://www.ncbi.nlm.nih.gov/gene/?term=211586
Etaa1	Etaa1	Ewing tumor-associated antigen 1	68145	https://www.ncbi.nlm.nih.gov/gene/?term=68145

Biology Open • Supplementary information

Nip7	Nip7	NIP7, nucleolar pre-rRNA processing protein	66164	https://www.ncbi.nlm.nih.gov/gene/?term=66164
Hspbap1	Hspbap1	Hspb associated protein 1	66667	https://www.ncbi.nlm.nih.gov/gene/?term=66667
Kdsr	Kdsr	3-ketodihydrosphingosine reductase	70750	https://www.ncbi.nlm.nih.gov/gene/?term=70750
Ccdc34	Ccdc34	coiled-coil domain containing 34	68201	https://www.ncbi.nlm.nih.gov/gene/?term=68201
Wdr37	Wdr37	WD repeat domain 37	207615	https://www.ncbi.nlm.nih.gov/gene/?term=207615
Limd1	Limd1	LIM domains containing 1	29806	https://www.ncbi.nlm.nih.gov/gene/?term=29806
En1	En1	engrailed 1	13798	https://www.ncbi.nlm.nih.gov/gene/?term=13798
Ankrd49	Ankrd49	ankyrin repeat domain 49	56503	https://www.ncbi.nlm.nih.gov/gene/?term=56503
Socs6	Socs6	suppressor of cytokine signaling 6	54607	https://www.ncbi.nlm.nih.gov/gene/?term=54607
Tanc2	Tanc2	tetratricopeptide repeat, ankyrin repeat and coiled-coil containing 2	77097	https://www.ncbi.nlm.nih.gov/gene/?term=77097
Fam53c	Fam53c	family with sequence similarity 53, member C	66306	https://www.ncbi.nlm.nih.gov/gene/?term=66306
Rpgr	Rpgr	retinitis pigmentosa GTPase regulator	19893	https://www.ncbi.nlm.nih.gov/gene/?term=19893
Zbtb26	Zbtb26	zinc finger and BTB domain containing 26	320633	https://www.ncbi.nlm.nih.gov/gene/?term=320633
Arhgef4	Arhgef4	Rho guanine nucleotide exchange factor (GEF) 4	226970	https://www.ncbi.nlm.nih.gov/gene/?term=226970
Zkscan1	Zkscan1	zinc finger with KRAB and SCAN domains 1	74570	https://www.ncbi.nlm.nih.gov/gene/?term=74570
Sh2b1	Sh2b1	SH2B adaptor protein 1	20399	https://www.ncbi.nlm.nih.gov/gene/?term=20399
Wdr77	Wdr77	WD repeat domain 77	70465	https://www.ncbi.nlm.nih.gov/gene/?term=70465
Arid1b	Arid1b	AT rich interactive domain 1B (SWI-like)	239985	https://www.ncbi.nlm.nih.gov/gene/?term=239985

Biology Open • Supplementary information

Kdm6b	Kdm6b	KDM1 lysine (K)-specific demethylase 6B	216850	https://www.ncbi.nlm.nih.gov/gene/?term=216850
Mrfap1	Mrfap1	Morf4 family associated protein 1	67568	https://www.ncbi.nlm.nih.gov/gene/?term=67568
Kcnip2	Kcnip2	Kv channel-interacting protein 2	80906	https://www.ncbi.nlm.nih.gov/gene/?term=80906
Klk4	Klk4	kallikrein related-peptidase 4 (prostase, enamel matrix, prostate)	56640	https://www.ncbi.nlm.nih.gov/gene/?term=56640
Zmpste24	Zmpste24	zinc metallopeptidase, STE24	230709	https://www.ncbi.nlm.nih.gov/gene/?term=230709
Elk4	Elk4	ELK4, member of ETS oncogene family	13714	https://www.ncbi.nlm.nih.gov/gene/?term=13714
Rab10	Rab10	RAB10, member RAS oncogene family	19325	https://www.ncbi.nlm.nih.gov/gene/?term=19325
Rbm12b2	Rbm12b2	RNA binding motif protein 12 B2	77604	https://www.ncbi.nlm.nih.gov/gene/?term=77604
Samd1	Samd1	sterile alpha motif domain containing 1	666704	https://www.ncbi.nlm.nih.gov/gene/?term=666704
Fam160a1	Fam160a1	family with sequence similarity 160, member A1	229488	https://www.ncbi.nlm.nih.gov/gene/?term=229488
Pxdc1	Pxdc1	PX domain containing 1	66895	https://www.ncbi.nlm.nih.gov/gene/?term=66895
Maml3	Maml3	mastermind like transcriptional coactivator 3	433586	https://www.ncbi.nlm.nih.gov/gene/?term=433586
Sgcz	Sgcz	sarcoglycan zeta	244431	https://www.ncbi.nlm.nih.gov/gene/?term=244431
Irf2bp2	Irf2bp2	interferon regulatory factor 2 binding protein 2	270110	https://www.ncbi.nlm.nih.gov/gene/?term=270110
Casp6	Casp6	caspase 6	12368	https://www.ncbi.nlm.nih.gov/gene/?term=12368
Srr	Srr	serine racemase	27364	https://www.ncbi.nlm.nih.gov/gene/?term=27364
Rnf149	Rnf149	ring finger protein 149	67702	https://www.ncbi.nlm.nih.gov/gene/?term=67702
9130008F23Rik	9130008F23Rik	RIKEN cDNA 9130008F23 gene	71583	https://www.ncbi.nlm.nih.gov/gene/?term=71583
Hnrnpdl	Hnrnpdl	heterogeneous nuclear ribonucleoprotein D-like	50926	https://www.ncbi.nlm.nih.gov/gene/?term=50926

Golim4	Golim4	golgi integral membrane protein 4	73124	https://www.ncbi.nlm.nih.gov/gene/?term=73124
Gpm6b	Gpm6b	glycoprotein m6b	14758	https://www.ncbi.nlm.nih.gov/gene/?term=14758
Anxa5	Anxa5	annexin A5	11747	https://www.ncbi.nlm.nih.gov/gene/?term=11747
Cabp5	Cabp5	calcium binding protein 5	29865	https://www.ncbi.nlm.nih.gov/gene/?term=29865
Derl2	Derl2	Der1-like domain family, member 2	116891	https://www.ncbi.nlm.nih.gov/gene/?term=116891
Fundc1	Fundc1	FUN14 domain containing 1	72018	https://www.ncbi.nlm.nih.gov/gene/?term=72018
Itk	Itk	IL2 inducible T cell kinase	16428	https://www.ncbi.nlm.nih.gov/gene/?term=16428
Wwtr1	Wwtr1	WW domain containing transcription regulator 1	97064	https://www.ncbi.nlm.nih.gov/gene/?term=97064
Wif1	Wif1	Wnt inhibitory factor 1	24117	https://www.ncbi.nlm.nih.gov/gene/?term=24117
Dync1li2	Dync1li2	dynein, cytoplasmic 1 light intermediate chain 2	234663	https://www.ncbi.nlm.nih.gov/gene/?term=234663
Ccnh	Ccnh	cyclin H	66671	https://www.ncbi.nlm.nih.gov/gene/?term=66671
Dtx2	Dtx2	deltex 2, E3 ubiquitin ligase	74198	https://www.ncbi.nlm.nih.gov/gene/?term=74198
Zdhhc5	Zdhhc5	zinc finger, DHHC domain containing 5	228136	https://www.ncbi.nlm.nih.gov/gene/?term=228136
Ube2w	Ube2w	ubiquitin-conjugating enzyme E2W (putative)	66799	https://www.ncbi.nlm.nih.gov/gene/?term=66799
Kat2b	Kat2b	K(lysine) acetyltransferase 2B	18519	https://www.ncbi.nlm.nih.gov/gene/?term=18519
Grhl1	Grhl1	grainyhead like transcription factor 1	195733	https://www.ncbi.nlm.nih.gov/gene/?term=195733
Isca1	Isca1	iron-sulfur cluster assembly 1	69046	https://www.ncbi.nlm.nih.gov/gene/?term=69046
Zfp521	Zfp521	zinc finger protein 521	225207	https://www.ncbi.nlm.nih.gov/gene/?term=225207
Bcl11b	Bcl11b	B cell leukemia/lymphoma 11B	58208	https://www.ncbi.nlm.nih.gov/gene/?term=58208
Map1b	Map1b	microtubule-associated protein 1B	17755	https://www.ncbi.nlm.nih.gov/gene/?term=17755
Adamtsl1	Adamtsl1	ADAMTS-like 1	77739	https://www.ncbi.nlm.nih.gov/gene/?term=77739
Fnip1	Fnip1	folliculin interacting protein 1	216742	https://www.ncbi.nlm.nih.gov/gene/?term=216742
Dpp10	Dpp10	dipeptidylpeptidase 10	269109	https://www.ncbi.nlm.nih.gov/gene/?term=269109

Gm5148	Gm5148	predicted gene 5148	381438	https://www.ncbi.nlm.nih.gov/gene/?term=381438
Cpeb4	Cpeb4	cytoplasmic polyadenylation element binding protein 4	67579	https://www.ncbi.nlm.nih.gov/gene/?term=67579
Atxn3	Atxn3	ataxin 3	110616	https://www.ncbi.nlm.nih.gov/gene/?term=110616
Gramd3	Gramd3	GRAM domain containing 3	107022	https://www.ncbi.nlm.nih.gov/gene/?term=107022
Dcaf6	Dcaf6	DDB1 and CUL4 associated factor 6	74106	https://www.ncbi.nlm.nih.gov/gene/?term=74106
Ptpro	Ptpro	protein tyrosine phosphatase, receptor type, O	19277	https://www.ncbi.nlm.nih.gov/gene/?term=19277
Syn2	Syn2	synapsin II	20965	https://www.ncbi.nlm.nih.gov/gene/?term=20965
Med19	Med19	mediator complex subunit 19	381379	https://www.ncbi.nlm.nih.gov/gene/?term=381379
Hecw1	Hecw1	HECT, C2 and WW domain containing E3 ubiquitin protein ligase 1	94253	https://www.ncbi.nlm.nih.gov/gene/?term=94253
Ccnc	Ccnc	cyclin C	51813	https://www.ncbi.nlm.nih.gov/gene/?term=51813
Fam20c	Fam20c	family with sequence similarity 20, member C	80752	https://www.ncbi.nlm.nih.gov/gene/?term=80752
Edem1	Edem1	ER degradation enhancer, mannosidase alpha-like 1	192193	https://www.ncbi.nlm.nih.gov/gene/?term=192193
Hand2	Hand2	heart and neural crest derivatives expressed 2	15111	https://www.ncbi.nlm.nih.gov/gene/?term=15111
Pip5k1c	Pip5k1c	phosphatidylinositol-4-phosphate 5-kinase, type 1 gamma	18717	https://www.ncbi.nlm.nih.gov/gene/?term=18717
Sgk3	Sgk3	serum/glucocorticoid regulated kinase 3	170755	https://www.ncbi.nlm.nih.gov/gene/?term=170755
Dennd1b	Dennd1b	DENN/MADD domain containing 1B	329260	https://www.ncbi.nlm.nih.gov/gene/?term=329260
Rbpms2	Rbpms2	RNA binding protein with multiple splicing 2	71973	https://www.ncbi.nlm.nih.gov/gene/?term=71973
Gla	Gla	galactosidase, alpha	11605	https://www.ncbi.nlm.nih.gov/gene/?term=11605

Biology Open • Supplementary information

Atp7a	Atp7a	ATPase, Cu++ transporting, alpha polypeptide	11977	https://www.ncbi.nlm.nih.gov/gene/?term=11977
Dennd4b	Dennd4b	DENN/MADD domain containing 4B	229541	https://www.ncbi.nlm.nih.gov/gene/?term=229541
Iqgap2	Iqgap2	IQ motif containing GTPase activating protein 2	544963	https://www.ncbi.nlm.nih.gov/gene/?term=544963
Nox4	Nox4	NADPH oxidase 4	50490	https://www.ncbi.nlm.nih.gov/gene/?term=50490
Ppp1r37	Ppp1r37	protein phosphatase 1, regulatory subunit 37	232947	https://www.ncbi.nlm.nih.gov/gene/?term=232947
Cdca7l	Cdca7l	cell division cycle associated 7 like	217946	https://www.ncbi.nlm.nih.gov/gene/?term=217946
Peak1	Peak1	pseudopodium-enriched atypical kinase 1	244895	https://www.ncbi.nlm.nih.gov/gene/?term=244895
Trim65	Trim65	tripartite motif-containing 65	338364	https://www.ncbi.nlm.nih.gov/gene/?term=338364
Ddc	Ddc	dopa decarboxylase	13195	https://www.ncbi.nlm.nih.gov/gene/?term=13195
4932438A13Rik	4932438A13Rik	RIKEN cDNA 4932438A13 gene	229227	https://www.ncbi.nlm.nih.gov/gene/?term=229227
Ptger4	Ptger4	prostaglandin E receptor 4 (subtype EP4)	19219	https://www.ncbi.nlm.nih.gov/gene/?term=19219
Phlpp2	Phlpp2	PH domain and leucine rich repeat protein phosphatase 2	244650	https://www.ncbi.nlm.nih.gov/gene/?term=244650
Zfyve21	Zfyve21	zinc finger, FYVE domain containing 21	68520	https://www.ncbi.nlm.nih.gov/gene/?term=68520
Tef	Tef	thyrotroph embryonic factor	21685	https://www.ncbi.nlm.nih.gov/gene/?term=21685
Cic	Cic	capicua transcriptional repressor	71722	https://www.ncbi.nlm.nih.gov/gene/?term=71722
Pcdh11x	Pcdh11x	protocadherin 11 X-linked	245578	https://www.ncbi.nlm.nih.gov/gene/?term=245578
Slc24a3	Slc24a3	solute carrier family 24 (sodium/potassium/calcium exchanger), member 3	94249	https://www.ncbi.nlm.nih.gov/gene/?term=94249
Snx13	Snx13	sorting nexin 13	217463	https://www.ncbi.nlm.nih.gov/gene/?term=217463
Itga6	Itga6	integrin alpha 6	16403	https://www.ncbi.nlm.nih.gov/gene/?term=16403

Biology Open • Supplementary information

Itgav	Itgav	integrin alpha V	16410	https://www.ncbi.nlm.nih.gov/gene/?term=16410
Spryd4	Spryd4	SPRY domain containing 4	66701	https://www.ncbi.nlm.nih.gov/gene/?term=66701
Cdk16	Cdk16	cyclin-dependent kinase 16	18555	https://www.ncbi.nlm.nih.gov/gene/?term=18555
Morc3	Morc3	microrchidia 3	338467	https://www.ncbi.nlm.nih.gov/gene/?term=338467
Myo1b	Myo1b	myosin IB	17912	https://www.ncbi.nlm.nih.gov/gene/?term=17912
Klhl14	Klhl14	kelch-like 14	225266	https://www.ncbi.nlm.nih.gov/gene/?term=225266
Gpr180	Gpr180	G protein-coupled receptor 180	58245	https://www.ncbi.nlm.nih.gov/gene/?term=58245
Xrn1	Xrn1	5'-3' exoribonuclease 1	24127	https://www.ncbi.nlm.nih.gov/gene/?term=24127
Mia3	Mia3	melanoma inhibitory activity 3	338366	https://www.ncbi.nlm.nih.gov/gene/?term=338366
Gid4	Gid4	GID complex subunit 4, VID24 homolog	66771	https://www.ncbi.nlm.nih.gov/gene/?term=66771
Tob2	Tob2	transducer of ERBB2, 2	57259	https://www.ncbi.nlm.nih.gov/gene/?term=57259
Cnep1r1	Cnep1r1	CTD nuclear envelope phosphatase 1 regulatory subunit 1	382030	https://www.ncbi.nlm.nih.gov/gene/?term=382030
P3h3	P3h3	prolyl 3-hydroxylase 3	14789	https://www.ncbi.nlm.nih.gov/gene/?term=14789
Man2a1	Man2a1	mannosidase 2, alpha 1	17158	https://www.ncbi.nlm.nih.gov/gene/?term=17158
Lhfpl2	Lhfpl2	lipoma HMGIC fusion partner-like 2	218454	https://www.ncbi.nlm.nih.gov/gene/?term=218454
Slc6a1	Slc6a1	solute carrier family 6 (neurotransmitter transporter, GABA), member 1	232333	https://www.ncbi.nlm.nih.gov/gene/?term=232333
Abhd13	Abhd13	abhydrolase domain containing 13	68904	https://www.ncbi.nlm.nih.gov/gene/?term=68904
Ergic2	Ergic2	ERGIC and golgi 2	67456	https://www.ncbi.nlm.nih.gov/gene/?term=67456
Pp2d1	Pp2d1	protein phosphatase 2C-like domain containing 1	110332	https://www.ncbi.nlm.nih.gov/gene/?term=110332
Grp	Grp	gastrin releasing peptide	225642	https://www.ncbi.nlm.nih.gov/gene/?term=225642
Col27a1	Col27a1	collagen, type XXVII, alpha 1	373864	https://www.ncbi.nlm.nih.gov/gene/?term=373864
Pcolce2	Pcolce2	procollagen C-endopeptidase enhancer 2	76477	https://www.ncbi.nlm.nih.gov/gene/?term=76477

Biology Open • Supplementary information

Glra1	Glra1	glycine receptor, alpha 1 subunit	14654	https://www.ncbi.nlm.nih.gov/gene/?term=14654
Lmbr1l	Lmbr1l	limb region 1 like	74775	https://www.ncbi.nlm.nih.gov/gene/?term=74775
Hivep1	Hivep1	human immunodeficiency virus type I enhancer binding protein 1	110521	https://www.ncbi.nlm.nih.gov/gene/?term=110521
Rhpn2	Rhpn2	rhophilin, Rho GTPase binding protein 2	52428	https://www.ncbi.nlm.nih.gov/gene/?term=52428
Exoc5	Exoc5	exocyst complex component 5	105504	https://www.ncbi.nlm.nih.gov/gene/?term=105504
Fmn2	Fmn2	formin 2	54418	https://www.ncbi.nlm.nih.gov/gene/?term=54418
Golga4	Golga4	golgi autoantigen, golgin subfamily a, 4	54214	https://www.ncbi.nlm.nih.gov/gene/?term=54214
Fancm	Fancm	Fanconi anemia, complementation group M	104806	https://www.ncbi.nlm.nih.gov/gene/?term=104806
Nefm	Nefm	neurofilament, medium polypeptide	18040	https://www.ncbi.nlm.nih.gov/gene/?term=18040
Arf1	Arf1	ADP-ribosylation factor 1	11840	https://www.ncbi.nlm.nih.gov/gene/?term=11840
Pkdcc	Pkdcc	protein kinase domain containing, cytoplasmic	106522	https://www.ncbi.nlm.nih.gov/gene/?term=106522
Tulp4	Tulp4	tubby like protein 4	68842	https://www.ncbi.nlm.nih.gov/gene/?term=68842
Gnpda2	Gnpda2	glucosamine-6-phosphate deaminase 2	67980	https://www.ncbi.nlm.nih.gov/gene/?term=67980
Ppp1r12c	Ppp1r12c	protein phosphatase 1, regulatory subunit 12C	232807	https://www.ncbi.nlm.nih.gov/gene/?term=232807
Wwp2	Wwp2	WW domain containing E3 ubiquitin protein ligase 2	66894	https://www.ncbi.nlm.nih.gov/gene/?term=66894
Cd69	Cd69	CD69 antigen	12515	https://www.ncbi.nlm.nih.gov/gene/?term=12515
Sertad3	Sertad3	SERTA domain containing 3	170742	https://www.ncbi.nlm.nih.gov/gene/?term=170742
Dmxl1	Dmxl1	Dmx-like 1	240283	https://www.ncbi.nlm.nih.gov/gene/?term=240283
Paxbp1	Paxbp1	PAX3 and PAX7 binding protein 1	67367	https://www.ncbi.nlm.nih.gov/gene/?term=67367

Btg2	Btg2	B cell translocation gene 2, anti-proliferative	12227	https://www.ncbi.nlm.nih.gov/gene/?term=12227
Tent4a	Tent4a	terminal nucleotidyltransferase 4A	210106	https://www.ncbi.nlm.nih.gov/gene/?term=210106
Slc12a5	Slc12a5	solute carrier family 12, member 5	57138	https://www.ncbi.nlm.nih.gov/gene/?term=57138
Dnajb9	Dnajb9	DnaJ heat shock protein family (Hsp40) member B9	27362	https://www.ncbi.nlm.nih.gov/gene/?term=27362
Gfpt2	Gfpt2	glutamine fructose-6-phosphate transaminase 2	14584	https://www.ncbi.nlm.nih.gov/gene/?term=14584
Cux1	Cux1	cut-like homeobox 1	13047	https://www.ncbi.nlm.nih.gov/gene/?term=13047
Adam23	Adam23	a disintegrin and metallopeptidase domain 23	23792	https://www.ncbi.nlm.nih.gov/gene/?term=23792
Tgif1	Tgif1	TGFB-induced factor homeobox 1	21815	https://www.ncbi.nlm.nih.gov/gene/?term=21815
A830018L16Rik	A830018L16Rik	RIKEN cDNA A830018L16 gene	320492	https://www.ncbi.nlm.nih.gov/gene/?term=320492
Gata2	Gata2	GATA binding protein 2	14461	https://www.ncbi.nlm.nih.gov/gene/?term=14461
Bcat2	Bcat2	branched chain aminotransferase 2, mitochondrial	12036	https://www.ncbi.nlm.nih.gov/gene/?term=12036
Cldn11	Cldn11	claudin 11	18417	https://www.ncbi.nlm.nih.gov/gene/?term=18417
Grip2	Grip2	glutamate receptor interacting protein 2	243547	https://www.ncbi.nlm.nih.gov/gene/?term=243547
Ppcs	Ppcs	phosphopantothenoylcysteine synthetase	106564	https://www.ncbi.nlm.nih.gov/gene/?term=106564
Ptar1	Ptar1	protein prenyltransferase alpha subunit repeat containing 1	72351	https://www.ncbi.nlm.nih.gov/gene/?term=72351
Glce	Glce	glucuronyl C5-epimerase	93683	https://www.ncbi.nlm.nih.gov/gene/?term=93683
Usp28	Usp28	ubiquitin specific peptidase 28	235323	https://www.ncbi.nlm.nih.gov/gene/?term=235323
Syt3	Syt3	synaptotagmin III	20981	https://www.ncbi.nlm.nih.gov/gene/?term=20981

Biology Open • Supplementary information

Smarca1	Smarca1	SWI/SNF related, matrix associated, actin dependent regulator of chromatin, subfamily a, member 1	93761	https://www.ncbi.nlm.nih.gov/gene/?term=93761
Kpna4	Kpna4	karyopherin (importin) alpha 4	16649	https://www.ncbi.nlm.nih.gov/gene/?term=16649
Thbs1	Thbs1	thrombospondin 1	21825	https://www.ncbi.nlm.nih.gov/gene/?term=21825
Lair1	Lair1	leukocyte-associated Ig-like receptor 1	52855	https://www.ncbi.nlm.nih.gov/gene/?term=52855
Vps35	Vps35	VPS35 retromer complex component	65114	https://www.ncbi.nlm.nih.gov/gene/?term=65114
Card11	Card11	caspase recruitment domain family, member 11	108723	https://www.ncbi.nlm.nih.gov/gene/?term=108723
Bmi1	Bmi1	Bmi1 polycomb ring finger oncogene	12151	https://www.ncbi.nlm.nih.gov/gene/?term=12151
Mfsd14a	Mfsd14a	major facilitator superfamily domain containing 14A	15247	https://www.ncbi.nlm.nih.gov/gene/?term=15247
Slc30a5	Slc30a5	solute carrier family 30 (zinc transporter), member 5	69048	https://www.ncbi.nlm.nih.gov/gene/?term=69048
Cpeb2	Cpeb2	cytoplasmic polyadenylation element binding protein 2	231207	https://www.ncbi.nlm.nih.gov/gene/?term=231207
Scrg1	Scrg1	scrapie responsive gene 1	20284	https://www.ncbi.nlm.nih.gov/gene/?term=20284
Slc4a4	Slc4a4	solute carrier family 4 (anion exchanger), member 4	54403	https://www.ncbi.nlm.nih.gov/gene/?term=54403
Ptgs2	Ptgs2	prostaglandin-endoperoxide synthase 2	19225	https://www.ncbi.nlm.nih.gov/gene/?term=19225
Gnpnat1	Gnpnat1	glucosamine-phosphate N-acetyltransferase 1	54342	https://www.ncbi.nlm.nih.gov/gene/?term=54342

Rab18	Rab18	RAB18, member RAS oncogene family	19330	https://www.ncbi.nlm.nih.gov/gene/?term=19330
Sbno1	Sbno1	strawberry notch 1	243272	https://www.ncbi.nlm.nih.gov/gene/?term=243272
Il13ra1	Il13ra1	interleukin 13 receptor, alpha 1	16164	https://www.ncbi.nlm.nih.gov/gene/?term=16164
Senp6	Senp6	SUMO/sentrin specific peptidase 6	215351	https://www.ncbi.nlm.nih.gov/gene/?term=215351
Nck2	Nck2	non-catalytic region of tyrosine kinase adaptor protein 2	17974	https://www.ncbi.nlm.nih.gov/gene/?term=17974
Kcnma1	Kcnma1	potassium large conductance calcium-activated channel, subfamily M, alpha member 1	16531	https://www.ncbi.nlm.nih.gov/gene/?term=16531
Igsf11	Igsf11	immunoglobulin superfamily, member 11	207683	https://www.ncbi.nlm.nih.gov/gene/?term=207683
Smap1	Smap1	small ArfGAP 1	98366	https://www.ncbi.nlm.nih.gov/gene/?term=98366
Cebpzos	Cebpzos	CCAAT/enhancer binding protein (C/EBP), zeta, opposite strand	68554	https://www.ncbi.nlm.nih.gov/gene/?term=68554
Csmd3	Csmd3	CUB and Sushi multiple domains 3	239420	https://www.ncbi.nlm.nih.gov/gene/?term=239420
Cmtr2	Cmtr2	cap methyltransferase 2	234728	https://www.ncbi.nlm.nih.gov/gene/?term=234728
Pi4k2a	Pi4k2a	phosphatidylinositol 4-kinase type 2 alpha	84095	https://www.ncbi.nlm.nih.gov/gene/?term=84095
Hipk2	Hipk2	homeodomain interacting protein kinase 2	15258	https://www.ncbi.nlm.nih.gov/gene/?term=15258
Pdgfra	Pdgfra	platelet derived growth factor receptor, alpha polypeptide	18595	https://www.ncbi.nlm.nih.gov/gene/?term=18595
Slc25a25	Slc25a25	solute carrier family 25 (mitochondrial carrier, phosphate carrier), member 25	227731	https://www.ncbi.nlm.nih.gov/gene/?term=227731
Rgs2	Rgs2	regulator of G-protein signaling 2	19735	https://www.ncbi.nlm.nih.gov/gene/?term=19735
Rgn	Rgn	regucalcin	19733	https://www.ncbi.nlm.nih.gov/gene/?term=19733

Biology Open • Supplementary information

Abca1	Abca1	ATP-binding cassette, sub-family A (ABC1), member 1	11303	https://www.ncbi.nlm.nih.gov/gene/?term=11303
Grm8	Grm8	glutamate receptor, metabotropic 8	14823	https://www.ncbi.nlm.nih.gov/gene/?term=14823
Gpcpd1	Gpcpd1	glycerophosphocholine phosphodiesterase 1	74182	https://www.ncbi.nlm.nih.gov/gene/?term=74182
Ankrd28	Ankrd28	ankyrin repeat domain 28	105522	https://www.ncbi.nlm.nih.gov/gene/?term=105522
Zfp281	Zfp281	zinc finger protein 281	226442	https://www.ncbi.nlm.nih.gov/gene/?term=226442
Pim3	Pim3	proviral integration site 3	223775	https://www.ncbi.nlm.nih.gov/gene/?term=223775
Ywhah	Ywhah	tyrosine 3-monooxygenase/tryptophan 5-monooxygenase activation protein, eta polypeptide	22629	https://www.ncbi.nlm.nih.gov/gene/?term=22629
Rb1cc1	Rb1cc1	RB1-inducible coiled-coil 1	12421	https://www.ncbi.nlm.nih.gov/gene/?term=12421
Tmem86a	Tmem86a	transmembrane protein 86A	67893	https://www.ncbi.nlm.nih.gov/gene/?term=67893
2410002F23Rik	2410002F23Rik	RIKEN cDNA 2410002F23 gene	668661	https://www.ncbi.nlm.nih.gov/gene/?term=668661
Hadhb	Hadhb	hydroxyacyl-Coenzyme A dehydrogenase/3-ketoacyl-Coenzyme A thiolase/enoyl-Coenzyme A hydratase (trifunctional protein), beta subunit	231086	https://www.ncbi.nlm.nih.gov/gene/?term=231086
Map3k3	Map3k3	mitogen-activated protein kinase kinase kinase 3	26406	https://www.ncbi.nlm.nih.gov/gene/?term=26406
Gripap1	Gripap1	GRIP1 associated protein 1	54645	https://www.ncbi.nlm.nih.gov/gene/?term=54645
Klhl24	Klhl24	kelch-like 24	75785	https://www.ncbi.nlm.nih.gov/gene/?term=75785
Rreb1	Rreb1	ras responsive element binding protein 1	68750	https://www.ncbi.nlm.nih.gov/gene/?term=68750

Biology Open • Supplementary information

Eml2	Eml2	echinoderm microtubule associated protein like 2	72205	https://www.ncbi.nlm.nih.gov/gene/?term=72205
Prkar1a	Prkar1a	protein kinase, cAMP dependent regulatory, type I, alpha	19084	https://www.ncbi.nlm.nih.gov/gene/?term=19084
Hif3a	Hif3a	hypoxia inducible factor 3, alpha subunit	53417	https://www.ncbi.nlm.nih.gov/gene/?term=53417
Zfp235	Zfp235	zinc finger protein 235	56525	https://www.ncbi.nlm.nih.gov/gene/?term=56525
Cbll1	Cbll1	Casitas B-lineage lymphoma-like 1	104836	https://www.ncbi.nlm.nih.gov/gene/?term=104836
Zfp788	Zfp788	zinc finger protein 788	67607	https://www.ncbi.nlm.nih.gov/gene/?term=67607
Zadh2	Zadh2	zinc binding alcohol dehydrogenase, domain containing 2	225791	https://www.ncbi.nlm.nih.gov/gene/?term=225791
Myb	Myb	myeloblastosis oncogene	17863	https://www.ncbi.nlm.nih.gov/gene/?term=17863
Tardbp	Tardbp	TAR DNA binding protein	230908	https://www.ncbi.nlm.nih.gov/gene/?term=230908
Frrs1l	Frrs1l	ferric-chelate reductase 1 like	230235	https://www.ncbi.nlm.nih.gov/gene/?term=230235
Fbxo21	Fbxo21	F-box protein 21	231670	https://www.ncbi.nlm.nih.gov/gene/?term=231670
Pom121	Pom121	nuclear pore membrane protein 121	107939	https://www.ncbi.nlm.nih.gov/gene/?term=107939
Myh1	Myh1	myosin, heavy polypeptide 1, skeletal muscle, adult	17879	https://www.ncbi.nlm.nih.gov/gene/?term=17879
Tfip11	Tfip11	tuftelin interacting protein 11	54723	https://www.ncbi.nlm.nih.gov/gene/?term=54723
Pik3r1	Pik3r1	phosphoinositide-3-kinase regulatory subunit 1	18708	https://www.ncbi.nlm.nih.gov/gene/?term=18708
Ephb2	Ephb2	Eph receptor B2	13844	https://www.ncbi.nlm.nih.gov/gene/?term=13844
Pappa	Pappa	pregnancy-associated plasma protein A	18491	https://www.ncbi.nlm.nih.gov/gene/?term=18491
Pign	Pign	phosphatidylinositol glycan anchor biosynthesis, class N	27392	https://www.ncbi.nlm.nih.gov/gene/?term=27392

Biology Open • Supplementary information

Cip2a	Cip2a	cell proliferation regulating inhibitor of protein phosphatase 2A	224171	https://www.ncbi.nlm.nih.gov/gene/?term=224171
Them4	Them4	thioesterase superfamily member 4	75778	https://www.ncbi.nlm.nih.gov/gene/?term=75778
Adipor2	Adipor2	adiponectin receptor 2	68465	https://www.ncbi.nlm.nih.gov/gene/?term=68465
Ptx3	Ptx3	pentraxin related gene	19288	https://www.ncbi.nlm.nih.gov/gene/?term=19288
Arid3a	Arid3a	AT rich interactive domain 3A (BRIGHT-like)	13496	https://www.ncbi.nlm.nih.gov/gene/?term=13496
Syvn1	Syvn1	synovial apoptosis inhibitor 1, synoviolin	74126	https://www.ncbi.nlm.nih.gov/gene/?term=74126
Irf4	Irf4	interferon regulatory factor 4	16364	https://www.ncbi.nlm.nih.gov/gene/?term=16364
Vdr	Vdr	vitamin D (1,25-dihydroxyvitamin D3) receptor	22337	https://www.ncbi.nlm.nih.gov/gene/?term=22337
Fam83h	Fam83h	family with sequence similarity 83, member H	105732	https://www.ncbi.nlm.nih.gov/gene/?term=105732
Nipal4	Nipal4	NIPA-like domain containing 4	214112	https://www.ncbi.nlm.nih.gov/gene/?term=214112
Sstr3	Sstr3	somatostatin receptor 3	20607	https://www.ncbi.nlm.nih.gov/gene/?term=20607
Crb2	Crb2	crumbs family member 2	241324	https://www.ncbi.nlm.nih.gov/gene/?term=241324
Nup210	Nup210	nucleoporin 210	54563	https://www.ncbi.nlm.nih.gov/gene/?term=54563
Kcns3	Kcns3	potassium voltage-gated channel, delayed-rectifier, subfamily S, member 3	238076	https://www.ncbi.nlm.nih.gov/gene/?term=238076
Sema4b	Sema4b	sema domain, immunoglobulin domain (Ig), transmembrane domain (TM) and short cytoplasmic domain, (semaphorin) 4B	20352	https://www.ncbi.nlm.nih.gov/gene/?term=20352
Was	Was	Wiskott-Aldrich syndrome	22376	https://www.ncbi.nlm.nih.gov/gene/?term=22376

Biology Open • Supplementary information

Abhd6	Abhd6	abhydrolase domain containing 6	66082	https://www.ncbi.nlm.nih.gov/gene/?term=66082
AU040320	AU040320	expressed sequence AU040320	100317	https://www.ncbi.nlm.nih.gov/gene/?term=100317
Scn2b	Scn2b	sodium channel, voltage-gated, type II, beta	72821	https://www.ncbi.nlm.nih.gov/gene/?term=72821
Enpep	Enpep	glutamyl aminopeptidase	13809	https://www.ncbi.nlm.nih.gov/gene/?term=13809
Zswim5	Zswim5	zinc finger SWIM-type containing 5	74464	https://www.ncbi.nlm.nih.gov/gene/?term=74464
Ptpn1	Ptpn1	protein tyrosine phosphatase, non-receptor type 1	19246	https://www.ncbi.nlm.nih.gov/gene/?term=19246
Dhx33	Dhx33	DEAH (Asp-Glu-Ala-His) box polypeptide 33	216877	https://www.ncbi.nlm.nih.gov/gene/?term=216877
Sarm1	Sarm1	sterile alpha and HEAT/Armadillo motif containing 1	237868	https://www.ncbi.nlm.nih.gov/gene/?term=237868
Zscan29	Zscan29	zinc finger SCAN domains 29	99334	https://www.ncbi.nlm.nih.gov/gene/?term=99334
Nbeal2	Nbeal2	neurobeachin-like 2	235627	https://www.ncbi.nlm.nih.gov/gene/?term=235627
Zfp523	Zfp523	zinc finger protein 523	224656	https://www.ncbi.nlm.nih.gov/gene/?term=224656
Stard13	Stard13	StAR-related lipid transfer (START) domain containing 13	243362	https://www.ncbi.nlm.nih.gov/gene/?term=243362
Pcgf6	Pcgf6	polycomb group ring finger 6	71041	https://www.ncbi.nlm.nih.gov/gene/?term=71041
Ier3ip1	Ier3ip1	immediate early response 3 interacting protein 1	66191	https://www.ncbi.nlm.nih.gov/gene/?term=66191
Lfng	Lfng	LFNG O-fucosylpeptide 3-beta-N-acetylglucosaminyltransferase	16848	https://www.ncbi.nlm.nih.gov/gene/?term=16848
Crem	Crem	cAMP responsive element modulator	12916	https://www.ncbi.nlm.nih.gov/gene/?term=12916
Klf13	Klf13	Kruppel-like factor 13	50794	https://www.ncbi.nlm.nih.gov/gene/?term=50794
E2f2	E2f2	E2F transcription factor 2	242705	https://www.ncbi.nlm.nih.gov/gene/?term=242705
Nrm	Nrm	nurim (nuclear envelope membrane protein)	106582	https://www.ncbi.nlm.nih.gov/gene/?term=106582

Biology Open • Supplementary information

Ovol1	Ovol1	ovo like zinc finger 1	18426	https://www.ncbi.nlm.nih.gov/gene/?term=18426
Osbpl9	Osbpl9	oxysterol binding protein-like 9	100273	https://www.ncbi.nlm.nih.gov/gene/?term=100273
Vps4b	Vps4b	vacuolar protein sorting 4B	20479	https://www.ncbi.nlm.nih.gov/gene/?term=20479
Bet1	Bet1	Bet1 golgi vesicular membrane trafficking protein	12068	https://www.ncbi.nlm.nih.gov/gene/?term=12068
Taz	Taz	tafazzin	66826	https://www.ncbi.nlm.nih.gov/gene/?term=66826
Kcnk10	Kcnk10	potassium channel, subfamily K, member 10	72258	https://www.ncbi.nlm.nih.gov/gene/?term=72258
Sgpl1	Sgpl1	sphingosine phosphate lyase 1	20397	https://www.ncbi.nlm.nih.gov/gene/?term=20397
Pi4k2b	Pi4k2b	phosphatidylinositol 4-kinase type 2 beta	67073	https://www.ncbi.nlm.nih.gov/gene/?term=67073
2610528J11Rik	2610528J11Rik	RIKEN cDNA 2610528J11 gene	66451	https://www.ncbi.nlm.nih.gov/gene/?term=66451
Samd10	Samd10	sterile alpha motif domain containing 10	229011	https://www.ncbi.nlm.nih.gov/gene/?term=229011
Npl	Npl	N-acetylneuraminate pyruvate lyase	74091	https://www.ncbi.nlm.nih.gov/gene/?term=74091
Retreg3	Retreg3	reticulophagy regulator family member 3	67998	https://www.ncbi.nlm.nih.gov/gene/?term=67998
Trim71	Trim71	tripartite motif-containing 71	636931	https://www.ncbi.nlm.nih.gov/gene/?term=636931
Slc6a17	Slc6a17	solute carrier family 6 (neurotransmitter transporter), member 17	229706	https://www.ncbi.nlm.nih.gov/gene/?term=229706
Sema4d	Sema4d	sema domain, immunoglobulin domain (Ig), transmembrane domain (TM) and short cytoplasmic domain, (semaphorin) 4D	20354	https://www.ncbi.nlm.nih.gov/gene/?term=20354
Msrb3	Msrb3	methionine sulfoxide reductase B3	320183	https://www.ncbi.nlm.nih.gov/gene/?term=320183

Biology Open • Supplementary information

Slc35a4	Slc35a4	solute carrier family 35, member A4	67843	https://www.ncbi.nlm.nih.gov/gene/?term=67843
Gga2	Gga2	golgi associated, gamma adaptin ear containing, ARF binding protein 2	74105	https://www.ncbi.nlm.nih.gov/gene/?term=74105
Cgn	Cgn	cingulin	70737	https://www.ncbi.nlm.nih.gov/gene/?term=70737
Triap1	Triap1	TP53 regulated inhibitor of apoptosis 1	69076	https://www.ncbi.nlm.nih.gov/gene/?term=69076
Tmem161b	Tmem161b	transmembrane protein 161B	72745	https://www.ncbi.nlm.nih.gov/gene/?term=72745
Blzf1	Blzf1	basic leucine zipper nuclear factor 1	66352	https://www.ncbi.nlm.nih.gov/gene/?term=66352
Acer2	Acer2	alkaline ceramidase 2	230379	https://www.ncbi.nlm.nih.gov/gene/?term=230379
Tmem120b	Tmem120b	transmembrane protein 120B	330189	https://www.ncbi.nlm.nih.gov/gene/?term=330189
Slc25a35	Slc25a35	solute carrier family 25, member 35	71998	https://www.ncbi.nlm.nih.gov/gene/?term=71998
Pafah1b1	Pafah1b1	platelet-activating factor acetylhydrolase, isoform 1b, subunit 1	18472	https://www.ncbi.nlm.nih.gov/gene/?term=18472
Rap1a	Rap1a	RAS-related protein 1a	109905	https://www.ncbi.nlm.nih.gov/gene/?term=109905
Dennd6a	Dennd6a	DENN/MADD domain containing 6A	211922	https://www.ncbi.nlm.nih.gov/gene/?term=211922
Grsf1	Grsf1	G-rich RNA sequence binding factor 1	231413	https://www.ncbi.nlm.nih.gov/gene/?term=231413
Tspan12	Tspan12	tetraspanin 12	269831	https://www.ncbi.nlm.nih.gov/gene/?term=269831
Ubr7	Ubr7	ubiquitin protein ligase E3 component n-recognin 7 (putative)	66622	https://www.ncbi.nlm.nih.gov/gene/?term=66622
Zswim6	Zswim6	zinc finger SWIM-type containing 6	67263	https://www.ncbi.nlm.nih.gov/gene/?term=67263
Rbm20	Rbm20	RNA binding motif protein 20	73713	https://www.ncbi.nlm.nih.gov/gene/?term=73713
Xirp1	Xirp1	xin actin-binding repeat containing 1	22437	https://www.ncbi.nlm.nih.gov/gene/?term=22437

Biology Open • Supplementary information

Necab3	Necab3	N-terminal EF-hand calcium binding protein 3	56846	https://www.ncbi.nlm.nih.gov/gene/?term=56846
Diras1	Diras1	DIRAS family, GTP-binding RAS-like 1	208666	https://www.ncbi.nlm.nih.gov/gene/?term=208666
Ier2	Ier2	immediate early response 2	15936	https://www.ncbi.nlm.nih.gov/gene/?term=15936
Sh3bp5l	Sh3bp5l	SH3 binding domain protein 5 like	79566	https://www.ncbi.nlm.nih.gov/gene/?term=79566
Atp11a	Atp11a	ATPase, class VI, type 11A	50770	https://www.ncbi.nlm.nih.gov/gene/?term=50770
Dus1l	Dus1l	dihydrouridine synthase 1-like (S. cerevisiae)	68730	https://www.ncbi.nlm.nih.gov/gene/?term=68730
Cacna1b	Cacna1b	calcium channel, voltage-dependent, N type, alpha 1B subunit	12287	https://www.ncbi.nlm.nih.gov/gene/?term=12287
Eif1ad	Eif1ad	eukaryotic translation initiation factor 1A domain containing	69860	https://www.ncbi.nlm.nih.gov/gene/?term=69860
Cdc42se1	Cdc42se1	CDC42 small effector 1	57912	https://www.ncbi.nlm.nih.gov/gene/?term=57912
Dynlt3	Dynlt3	dynein light chain Tctex-type 3	67117	https://www.ncbi.nlm.nih.gov/gene/?term=67117
Sec14l2	Sec14l2	SEC14-like lipid binding 2	67815	https://www.ncbi.nlm.nih.gov/gene/?term=67815
Sh3tc2	Sh3tc2	SH3 domain and tetratricopeptide repeats 2	225608	https://www.ncbi.nlm.nih.gov/gene/?term=225608
Tmprss13	Tmprss13	transmembrane protease, serine 13	214531	https://www.ncbi.nlm.nih.gov/gene/?term=214531
Cdc42bpg	Cdc42bpg	CDC42 binding protein kinase gamma (DMPK-like)	240505	https://www.ncbi.nlm.nih.gov/gene/?term=240505
Zfp488	Zfp488	zinc finger protein 488	382867	https://www.ncbi.nlm.nih.gov/gene/?term=382867
Tnfsf4	Tnfsf4	tumor necrosis factor (ligand) superfamily, member 4	22164	https://www.ncbi.nlm.nih.gov/gene/?term=22164
Mamdc2	Mamdc2	MAM domain containing 2	71738	https://www.ncbi.nlm.nih.gov/gene/?term=71738

Mfsd9	Mfsd9	major facilitator superfamily domain containing 9	211798	https://www.ncbi.nlm.nih.gov/gene/?term=211798
Ccnj	Ccnj	cyclin J	240665	https://www.ncbi.nlm.nih.gov/gene/?term=240665
Ptpn7	Ptpn7	protein tyrosine phosphatase, non-receptor type 7	320139	https://www.ncbi.nlm.nih.gov/gene/?term=320139
Lrp4	Lrp4	low density lipoprotein receptor-related protein 4	228357	https://www.ncbi.nlm.nih.gov/gene/?term=228357
Ncan	Ncan	neurocan	13004	https://www.ncbi.nlm.nih.gov/gene/?term=13004
Lactb	Lactb	lactamase, beta	80907	https://www.ncbi.nlm.nih.gov/gene/?term=80907
Cbx7	Cbx7	chromobox 7	52609	https://www.ncbi.nlm.nih.gov/gene/?term=52609
Dtx4	Dtx4	deltex 4, E3 ubiquitin ligase	207521	https://www.ncbi.nlm.nih.gov/gene/?term=207521
Dock3	Dock3	dedicator of cyto-kinesis 3	208869	https://www.ncbi.nlm.nih.gov/gene/?term=208869
Gcnt1	Gcnt1	glucosaminyl (N-acetyl) transferase 1, core 2	14537	https://www.ncbi.nlm.nih.gov/gene/?term=14537
Dyrk1b	Dyrk1b	dual-specificity tyrosine-(Y)-phosphorylation regulated kinase 1b	13549	https://www.ncbi.nlm.nih.gov/gene/?term=13549
Zfp42	Zfp42	zinc finger protein 42	22702	https://www.ncbi.nlm.nih.gov/gene/?term=22702
Pdgfrb	Pdgfrb	platelet derived growth factor receptor, beta polypeptide	18596	https://www.ncbi.nlm.nih.gov/gene/?term=18596
Pik3r3	Pik3r3	phosphoinositide-3-kinase regulatory subunit 3	18710	https://www.ncbi.nlm.nih.gov/gene/?term=18710
Tmem179b	Tmem179b	transmembrane protein 179B	67706	https://www.ncbi.nlm.nih.gov/gene/?term=67706
Car12	Car12	carbonic anhydrase 12	76459	https://www.ncbi.nlm.nih.gov/gene/?term=76459
Adam12	Adam12	a disintegrin and metallopeptidase domain 12 (meltrin alpha)	11489	https://www.ncbi.nlm.nih.gov/gene/?term=11489
Zfp930	Zfp930	zinc finger protein 930	234358	https://www.ncbi.nlm.nih.gov/gene/?term=234358
Gpr155	Gpr155	G protein-coupled receptor 155	68526	https://www.ncbi.nlm.nih.gov/gene/?term=68526

Biology Open • Supplementary information

Zfp217	Zfp217	zinc finger protein 217	228913	https://www.ncbi.nlm.nih.gov/gene/?term=228913
Magohb	Magohb	mago homolog B, exon junction complex core component	66441	https://www.ncbi.nlm.nih.gov/gene/?term=66441
Copb1	Copb1	coatomer protein complex, subunit beta 1	70349	https://www.ncbi.nlm.nih.gov/gene/?term=70349
Pnn	Pnn	pinin	18949	https://www.ncbi.nlm.nih.gov/gene/?term=18949
Fth1	Fth1	ferritin heavy polypeptide 1	14319	https://www.ncbi.nlm.nih.gov/gene/?term=14319
Arhgef2	Arhgef2	rho/rac guanine nucleotide exchange factor (GEF) 2	16800	https://www.ncbi.nlm.nih.gov/gene/?term=16800
Tmem150a	Tmem150a	transmembrane protein 150A	232086	https://www.ncbi.nlm.nih.gov/gene/?term=232086
Zmym2	Zmym2	zinc finger, MYM-type 2	76007	https://www.ncbi.nlm.nih.gov/gene/?term=76007
Mboat1	Mboat1	membrane bound O-acyltransferase domain containing 1	218121	https://www.ncbi.nlm.nih.gov/gene/?term=218121
Cdh2	Cdh2	cadherin 2	12558	https://www.ncbi.nlm.nih.gov/gene/?term=12558
Phospho2	Phospho2	phosphatase, orphan 2	73373	https://www.ncbi.nlm.nih.gov/gene/?term=73373
Ptp4a1	Ptp4a1	protein tyrosine phosphatase 4a1	19243	https://www.ncbi.nlm.nih.gov/gene/?term=19243
Cramp1l	Cramp1l	cramped chromatin regulator homolog 1	57354	https://www.ncbi.nlm.nih.gov/gene/?term=57354
Pex5l	Pex5l	peroxisomal biogenesis factor 5-like	58869	https://www.ncbi.nlm.nih.gov/gene/?term=58869
Srcin1	Srcin1	SRC kinase signaling inhibitor 1	56013	https://www.ncbi.nlm.nih.gov/gene/?term=56013
Gon4l	Gon4l	gon-4-like (C.elegans)	76022	https://www.ncbi.nlm.nih.gov/gene/?term=76022
St6galnac6	St6galnac6	ST6 (alpha-N-acetyl-neuraminyl-2,3-beta-galactosyl-1,3)-N-acetylgalactosaminide alpha-2,6-sialyltransferase 6	50935	https://www.ncbi.nlm.nih.gov/gene/?term=50935
Sqstm1	Sqstm1	sequestosome 1	18412	https://www.ncbi.nlm.nih.gov/gene/?term=18412

Biology Open • Supplementary information

Stx6	Stx6	syntaxin 6	58244	https://www.ncbi.nlm.nih.gov/gene/?term=58244
Atxn1l	Atxn1l	ataxin 1-like	52335	https://www.ncbi.nlm.nih.gov/gene/?term=52335
Zfp827	Zfp827	zinc finger protein 827	622675	https://www.ncbi.nlm.nih.gov/gene/?term=622675
Tmem127	Tmem127	transmembrane protein 127	69470	https://www.ncbi.nlm.nih.gov/gene/?term=69470
Egln3	Egln3	egl-9 family hypoxia-inducible factor 3	112407	https://www.ncbi.nlm.nih.gov/gene/?term=112407
Ldlrap1	Ldlrap1	low density lipoprotein receptor adaptor protein 1	100017	https://www.ncbi.nlm.nih.gov/gene/?term=100017
Pkd2	Pkd2	polycystic kidney disease 2	18764	https://www.ncbi.nlm.nih.gov/gene/?term=18764
Mkrn1	Mkrn1	makorin, ring finger protein, 1	54484	https://www.ncbi.nlm.nih.gov/gene/?term=54484
Trip11	Trip11	thyroid hormone receptor interactor 11	109181	https://www.ncbi.nlm.nih.gov/gene/?term=109181
1600012H06Rik	1600012H06Rik	RIKEN cDNA 1600012H06 gene	67912	https://www.ncbi.nlm.nih.gov/gene/?term=67912
Heg1	Heg1	heart development protein with EGF-like domains 1	77446	https://www.ncbi.nlm.nih.gov/gene/?term=77446
Panx2	Panx2	pannexin 2	406218	https://www.ncbi.nlm.nih.gov/gene/?term=406218
Aktip	Aktip	thymoma viral proto-oncogene 1 interacting protein	14339	https://www.ncbi.nlm.nih.gov/gene/?term=14339
Mtmr3	Mtmr3	myotubularin related protein 3	74302	https://www.ncbi.nlm.nih.gov/gene/?term=74302
Zfp367	Zfp367	zinc finger protein 367	238673	https://www.ncbi.nlm.nih.gov/gene/?term=238673
Nrip3	Nrip3	nuclear receptor interacting protein 3	78593	https://www.ncbi.nlm.nih.gov/gene/?term=78593
Zc3h12c	Zc3h12c	zinc finger CCCH type containing 12C	244871	https://www.ncbi.nlm.nih.gov/gene/?term=244871
Lypd6	Lypd6	LY6/PLAUR domain containing 6	320343	https://www.ncbi.nlm.nih.gov/gene/?term=320343
Irf9	Irf9	interferon regulatory factor 9	16391	https://www.ncbi.nlm.nih.gov/gene/?term=16391
Spopl	Spopl	speckle-type POZ protein-like	76857	https://www.ncbi.nlm.nih.gov/gene/?term=76857
Btg3	Btg3	B cell translocation gene 3	12228	https://www.ncbi.nlm.nih.gov/gene/?term=12228

Biology Open • Supplementary information

Znfx1	Znfx1	zinc finger, NFX1-type containing 1	98999	https://www.ncbi.nlm.nih.gov/gene/?term=98999
Fyco1	Fyco1	FYVE and coiled-coil domain containing 1	17281	https://www.ncbi.nlm.nih.gov/gene/?term=17281
Afg1l	Afg1l	AFG1 like ATPase	215951	https://www.ncbi.nlm.nih.gov/gene/?term=215951
Pthlh	Pthlh	parathyroid hormone-like peptide	19227	https://www.ncbi.nlm.nih.gov/gene/?term=19227
Mink1	Mink1	misshapen-like kinase 1 (zebrafish)	50932	https://www.ncbi.nlm.nih.gov/gene/?term=50932
Sema7a	Sema7a	sema domain, immunoglobulin domain (Ig), and GPI membrane anchor, (semaphorin) 7A	20361	https://www.ncbi.nlm.nih.gov/gene/?term=20361
Pde3b	Pde3b	phosphodiesterase 3B, cGMP-inhibited	18576	https://www.ncbi.nlm.nih.gov/gene/?term=18576
Fzd3	Fzd3	frizzled class receptor 3	14365	https://www.ncbi.nlm.nih.gov/gene/?term=14365
Topors	Topors	topoisomerase I binding, arginine/serine-rich	106021	https://www.ncbi.nlm.nih.gov/gene/?term=106021
Rufy2	Rufy2	RUN and FYVE domain-containing 2	70432	https://www.ncbi.nlm.nih.gov/gene/?term=70432
Reps2	Reps2	RALBP1 associated Eps domain containing protein 2	194590	https://www.ncbi.nlm.nih.gov/gene/?term=194590
Rp2	Rp2	retinitis pigmentosa 2 homolog	19889	https://www.ncbi.nlm.nih.gov/gene/?term=19889
Map3k14	Map3k14	mitogen-activated protein kinase kinase kinase 14	53859	https://www.ncbi.nlm.nih.gov/gene/?term=53859
Ulk1	Ulk1	unc-51 like kinase 1	22241	https://www.ncbi.nlm.nih.gov/gene/?term=22241
Kcnb1	Kcnb1	potassium voltage gated channel, Shab-related subfamily, member 1	16500	https://www.ncbi.nlm.nih.gov/gene/?term=16500
F3	F3	coagulation factor III	14066	https://www.ncbi.nlm.nih.gov/gene/?term=14066
Pgm2l1	Pgm2l1	phosphoglucomutase 2-like 1	70974	https://www.ncbi.nlm.nih.gov/gene/?term=70974
Slc31a2	Slc31a2	solute carrier family 31, member 2	20530	https://www.ncbi.nlm.nih.gov/gene/?term=20530
Arid4b	Arid4b	AT rich interactive domain 4B (RBP1-like)	94246	https://www.ncbi.nlm.nih.gov/gene/?term=94246

Biology Open • Supplementary information

Nagk	Nagk	N-acetylglucosamine kinase	56174	https://www.ncbi.nlm.nih.gov/gene/?term=56174
Usp3	Usp3	ubiquitin specific peptidase 3	235441	https://www.ncbi.nlm.nih.gov/gene/?term=235441
Sertad2	Sertad2	SERTA domain containing 2	58172	https://www.ncbi.nlm.nih.gov/gene/?term=58172
App	App	amyloid beta (A4) precursor protein	11820	https://www.ncbi.nlm.nih.gov/gene/?term=11820
Tbc1d8b	Tbc1d8b	TBC1 domain family, member 8B	245638	https://www.ncbi.nlm.nih.gov/gene/?term=245638
Lrrc55	Lrrc55	leucine rich repeat containing 55	241528	https://www.ncbi.nlm.nih.gov/gene/?term=241528
Cast	Cast	calpastatin	12380	https://www.ncbi.nlm.nih.gov/gene/?term=12380
Kmt2b	Kmt2b	lysine (K)-specific methyltransferase 2B	75410	https://www.ncbi.nlm.nih.gov/gene/?term=75410
Ism2	Ism2	isthmin 2	217738	https://www.ncbi.nlm.nih.gov/gene/?term=217738
Fcho2	Fcho2	FCH domain only 2	218503	https://www.ncbi.nlm.nih.gov/gene/?term=218503
Purb	Purb	purine rich element binding protein B	19291	https://www.ncbi.nlm.nih.gov/gene/?term=19291
Zfp800	Zfp800	zinc finger protein 800	627049	https://www.ncbi.nlm.nih.gov/gene/?term=627049
Kif5a	Kif5a	kinesin family member 5A	16572	https://www.ncbi.nlm.nih.gov/gene/?term=16572
Ankrd9	Ankrd9	ankyrin repeat domain 9	74251	https://www.ncbi.nlm.nih.gov/gene/?term=74251
Col4a4	Col4a4	collagen, type IV, alpha 4	12829	https://www.ncbi.nlm.nih.gov/gene/?term=12829
Tnfrsf21	Tnfrsf21	tumor necrosis factor receptor superfamily, member 21	94185	https://www.ncbi.nlm.nih.gov/gene/?term=94185
Chrm2	Chrm2	cholinergic receptor, muscarinic 2, cardiac	243764	https://www.ncbi.nlm.nih.gov/gene/?term=243764
Tmem267	Tmem267	transmembrane protein 267	633640	https://www.ncbi.nlm.nih.gov/gene/?term=633640
U2surp	U2surp	U2 snRNP-associated SURP domain containing	67958	https://www.ncbi.nlm.nih.gov/gene/?term=67958
Tmcc3	Tmcc3	transmembrane and coiled coil domains 3	319880	https://www.ncbi.nlm.nih.gov/gene/?term=319880
Armc8	Armc8	armadillo repeat containing 8	74125	https://www.ncbi.nlm.nih.gov/gene/?term=74125

Biology Open • Supplementary information

Atg16l1	Atg16l1	autophagy related 16-like 1 (S. cerevisiae)	77040	https://www.ncbi.nlm.nih.gov/gene/?term=77040
Rasl11b	Rasl11b	RAS-like, family 11, member B	68939	https://www.ncbi.nlm.nih.gov/gene/?term=68939
Prrg1	Prrg1	proline rich Gla (G-carboxyglutamic acid) 1	546336	https://www.ncbi.nlm.nih.gov/gene/?term=546336
Ptpn4	Ptpn4	protein tyrosine phosphatase, non-receptor type 4	19258	https://www.ncbi.nlm.nih.gov/gene/?term=19258
Fbxl5	Fbxl5	F-box and leucine-rich repeat protein 5	242960	https://www.ncbi.nlm.nih.gov/gene/?term=242960
Vangl1	Vangl1	VANGL planar cell polarity 1	229658	https://www.ncbi.nlm.nih.gov/gene/?term=229658
Rnf150	Rnf150	ring finger protein 150	330812	https://www.ncbi.nlm.nih.gov/gene/?term=330812
Sar1b	Sar1b	secretion associated Ras related GTPase 1B	66397	https://www.ncbi.nlm.nih.gov/gene/?term=66397
St3gal1	St3gal1	ST3 beta-galactoside alpha-2,3-sialyltransferase 1	20442	https://www.ncbi.nlm.nih.gov/gene/?term=20442
Ppp1r3e	Ppp1r3e	protein phosphatase 1, regulatory subunit 3E	105651	https://www.ncbi.nlm.nih.gov/gene/?term=105651
Ddhd1	Ddhd1	DDHD domain containing 1	114874	https://www.ncbi.nlm.nih.gov/gene/?term=114874
Unk	Unk	unkempt family zinc finger	217331	https://www.ncbi.nlm.nih.gov/gene/?term=217331
Crybg3	Crybg3	beta-gamma crystallin domain containing 3	224273	https://www.ncbi.nlm.nih.gov/gene/?term=224273
Tnks2	Tnks2	tankyrase, TRF1-interacting ankyrin-related ADP-ribose polymerase 2	74493	https://www.ncbi.nlm.nih.gov/gene/?term=74493
Laptm4a	Laptm4a	lysosomal-associated protein transmembrane 4A	17775	https://www.ncbi.nlm.nih.gov/gene/?term=17775
Pdcd1lg2	Pdcd1lg2	programmed cell death 1 ligand 2	58205	https://www.ncbi.nlm.nih.gov/gene/?term=58205
Slc25a40	Slc25a40	solute carrier family 25, member 40	319653	https://www.ncbi.nlm.nih.gov/gene/?term=319653

Biology Open • Supplementary information

Rab8b	Rab8b	RAB8B, member RAS oncogene family	235442	https://www.ncbi.nlm.nih.gov/gene/?term=235442
Clip4	Clip4	CAP-GLY domain containing linker protein family, member 4	78785	https://www.ncbi.nlm.nih.gov/gene/?term=78785
Rhoc	Rhoc	ras homolog family member C	11853	https://www.ncbi.nlm.nih.gov/gene/?term=11853
Suco	Suco	SUN domain containing ossification factor	226551	https://www.ncbi.nlm.nih.gov/gene/?term=226551
Mastl	Mastl	microtubule associated serine/threonine kinase-like	67121	https://www.ncbi.nlm.nih.gov/gene/?term=67121
Arhgap1	Arhgap1	Rho GTPase activating protein 1	228359	https://www.ncbi.nlm.nih.gov/gene/?term=228359
Iqsec2	Iqsec2	IQ motif and Sec7 domain 2	245666	https://www.ncbi.nlm.nih.gov/gene/?term=245666
Fat2	Fat2	FAT atypical cadherin 2	245827	https://www.ncbi.nlm.nih.gov/gene/?term=245827
Coro2b	Coro2b	coronin, actin binding protein, 2B	235431	https://www.ncbi.nlm.nih.gov/gene/?term=235431
Gab1	Gab1	growth factor receptor bound protein 2-associated protein 1	14388	https://www.ncbi.nlm.nih.gov/gene/?term=14388
Rbl2	Rbl2	RB transcriptional corepressor like 2	19651	https://www.ncbi.nlm.nih.gov/gene/?term=19651
Crot	Crot	carnitine O-octanoyltransferase	74114	https://www.ncbi.nlm.nih.gov/gene/?term=74114
Sash1	Sash1	SAM and SH3 domain containing 1	70097	https://www.ncbi.nlm.nih.gov/gene/?term=70097
Zfpm2	Zfpm2	zinc finger protein, multitype 2	22762	https://www.ncbi.nlm.nih.gov/gene/?term=22762
Arhgef10	Arhgef10	Rho guanine nucleotide exchange factor (GEF) 10	234094	https://www.ncbi.nlm.nih.gov/gene/?term=234094
Slc24a2	Slc24a2	solute carrier family 24 (sodium/potassium/calcium exchanger), member 2	76376	https://www.ncbi.nlm.nih.gov/gene/?term=76376
Gosr1	Gosr1	golgi SNAP receptor complex member 1	53334	https://www.ncbi.nlm.nih.gov/gene/?term=53334
Arhgap12	Arhgap12	Rho GTPase activating protein 12	75415	https://www.ncbi.nlm.nih.gov/gene/?term=75415

Biology Open • Supplementary information

C2cd2	C2cd2	C2 calcium-dependent domain containing 2	207781	https://www.ncbi.nlm.nih.gov/gene/?term=207781
E2f1	E2f1	E2F transcription factor 1	13555	https://www.ncbi.nlm.nih.gov/gene/?term=13555
B3galt2	B3galt2	UDP-Gal:betaGlcNAc beta 1,3-galactosyltransferase, polypeptide 2	26878	https://www.ncbi.nlm.nih.gov/gene/?term=26878
Elk3	Elk3	ELK3, member of ETS oncogene family	13713	https://www.ncbi.nlm.nih.gov/gene/?term=13713
Map3k13	Map3k13	mitogen-activated protein kinase kinase kinase 13	71751	https://www.ncbi.nlm.nih.gov/gene/?term=71751
Lama3	Lama3	laminin, alpha 3	16774	https://www.ncbi.nlm.nih.gov/gene/?term=16774
Ankrd52	Ankrd52	ankyrin repeat domain 52	237615	https://www.ncbi.nlm.nih.gov/gene/?term=237615
Mapre3	Mapre3	microtubule-associated protein, RP/EB family, member 3	100732	https://www.ncbi.nlm.nih.gov/gene/?term=100732
Mex3d	Mex3d	mex3 RNA binding family member D	237400	https://www.ncbi.nlm.nih.gov/gene/?term=237400
Acsl4	Acsl4	acyl-CoA synthetase long-chain family member 4	50790	https://www.ncbi.nlm.nih.gov/gene/?term=50790
Mfap3l	Mfap3l	microfibrillar-associated protein 3-like	71306	https://www.ncbi.nlm.nih.gov/gene/?term=71306
Eif4a2	Eif4a2	eukaryotic translation initiation factor 4A2	13682	https://www.ncbi.nlm.nih.gov/gene/?term=13682
Susd6	Susd6	sushi domain containing 6	217684	https://www.ncbi.nlm.nih.gov/gene/?term=217684
Uri1	Uri1	URI1, prefoldin-like chaperone	19777	https://www.ncbi.nlm.nih.gov/gene/?term=19777
Nedd4l	Nedd4l	neural precursor cell expressed, developmentally down-regulated gene 4-like	83814	https://www.ncbi.nlm.nih.gov/gene/?term=83814
6430548M08Rik	6430548M08Rik	RIKEN cDNA 6430548M08 gene	234797	https://www.ncbi.nlm.nih.gov/gene/?term=234797

Biology Open • Supplementary information

Tars2	Tars2	threonyl-tRNA synthetase 2, mitochondrial (putative)	71807	https://www.ncbi.nlm.nih.gov/gene/?term=71807
Fgd5	Fgd5	FYVE, RhoGEF and PH domain containing 5	232237	https://www.ncbi.nlm.nih.gov/gene/?term=232237
Ptpn3	Ptpn3	protein tyrosine phosphatase, non-receptor type 3	545622	https://www.ncbi.nlm.nih.gov/gene/?term=545622
Tbcel	Tbcel	tubulin folding cofactor E-like	272589	https://www.ncbi.nlm.nih.gov/gene/?term=272589
Cep97	Cep97	centrosomal protein 97	74201	https://www.ncbi.nlm.nih.gov/gene/?term=74201
Map3k2	Map3k2	mitogen-activated protein kinase kinase kinase 2	26405	https://www.ncbi.nlm.nih.gov/gene/?term=26405
Itpripl2	Itpripl2	inositol 1,4,5-triphosphate receptor interacting protein-like 2	319622	https://www.ncbi.nlm.nih.gov/gene/?term=319622
Fam219b	Fam219b	family with sequence similarity 219, member B	78323	https://www.ncbi.nlm.nih.gov/gene/?term=78323
Zfp661	Zfp661	zinc finger protein 661	72180	https://www.ncbi.nlm.nih.gov/gene/?term=72180
Il25	Il25	interleukin 25	140806	https://www.ncbi.nlm.nih.gov/gene/?term=140806
Rab5b	Rab5b	RAB5B, member RAS oncogene family	19344	https://www.ncbi.nlm.nih.gov/gene/?term=19344
Txnip	Txnip	thioredoxin interacting protein	56338	https://www.ncbi.nlm.nih.gov/gene/?term=56338
Creb1	Creb1	cAMP responsive element binding protein 1	12912	https://www.ncbi.nlm.nih.gov/gene/?term=12912
Gpr137c	Gpr137c	G protein-coupled receptor 137C	70713	https://www.ncbi.nlm.nih.gov/gene/?term=70713
Usp24	Usp24	ubiquitin specific peptidase 24	329908	https://www.ncbi.nlm.nih.gov/gene/?term=329908
Arhgef11	Arhgef11	Rho guanine nucleotide exchange factor (GEF) 11	213498	https://www.ncbi.nlm.nih.gov/gene/?term=213498
Zfp91	Zfp91	zinc finger protein 91	109910	https://www.ncbi.nlm.nih.gov/gene/?term=109910
Hs3st5	Hs3st5	heparan sulfate (glucosamine) 3-O-sulfotransferase 5	319415	https://www.ncbi.nlm.nih.gov/gene/?term=319415

Biology Open • Supplementary information

Npas2	Npas2	neuronal PAS domain protein 2	18143	https://www.ncbi.nlm.nih.gov/gene/?term=18143
Rgmb	Rgmb	repulsive guidance molecule family member B	68799	https://www.ncbi.nlm.nih.gov/gene/?term=68799
Plekha3	Plekha3	pleckstrin homology domain-containing, family A (phosphoinositide binding specific) member 3	83435	https://www.ncbi.nlm.nih.gov/gene/?term=83435
Pgbd5	Pgbd5	piggyBac transposable element derived 5	209966	https://www.ncbi.nlm.nih.gov/gene/?term=209966
Btbd10	Btbd10	BTB (POZ) domain containing 10	68815	https://www.ncbi.nlm.nih.gov/gene/?term=68815
Ddhd2	Ddhd2	DDHD domain containing 2	72108	https://www.ncbi.nlm.nih.gov/gene/?term=72108
Prpf4b	Prpf4b	pre-mRNA processing factor 4B	19134	https://www.ncbi.nlm.nih.gov/gene/?term=19134
Fhl2	Fhl2	four and a half LIM domains 2	14200	https://www.ncbi.nlm.nih.gov/gene/?term=14200
2310022A10Rik	2310022A10Rik	RIKEN cDNA 2310022A10 gene	66367	https://www.ncbi.nlm.nih.gov/gene/?term=66367
Dnm1	Dnm1	dynamin 1	13429	https://www.ncbi.nlm.nih.gov/gene/?term=13429
Cstf2	Cstf2	cleavage stimulation factor, 3' pre-RNA subunit 2	108062	https://www.ncbi.nlm.nih.gov/gene/?term=108062
Asb1	Asb1	ankyrin repeat and SOCS box-containing 1	65247	https://www.ncbi.nlm.nih.gov/gene/?term=65247
Pik3c2a	Pik3c2a	phosphatidylinositol-4-phosphate 3-kinase catalytic subunit type 2 alpha	18704	https://www.ncbi.nlm.nih.gov/gene/?term=18704
Tank	Tank	TRAF family member-associated Nf-kappa B activator	21353	https://www.ncbi.nlm.nih.gov/gene/?term=21353
Otub2	Otub2	OTU domain, ubiquitin aldehyde binding 2	68149	https://www.ncbi.nlm.nih.gov/gene/?term=68149
Gpc4	Gpc4	glypican 4	14735	https://www.ncbi.nlm.nih.gov/gene/?term=14735
Pvr	Pvr	poliovirus receptor	52118	https://www.ncbi.nlm.nih.gov/gene/?term=52118

Biology Open • Supplementary information

Cnga4	Cnga4	cyclic nucleotide gated channel alpha 4	233649	https://www.ncbi.nlm.nih.gov/gene/?term=233649
Adam22	Adam22	a disintegrin and metallopeptidase domain 22	11496	https://www.ncbi.nlm.nih.gov/gene/?term=11496
Hebp1	Hebp1	heme binding protein 1	15199	https://www.ncbi.nlm.nih.gov/gene/?term=15199
Plet1	Plet1	placenta expressed transcript 1	76509	https://www.ncbi.nlm.nih.gov/gene/?term=76509
F2r	F2r	coagulation factor II (thrombin) receptor	14062	https://www.ncbi.nlm.nih.gov/gene/?term=14062
Mkrn2	Mkrn2	makorin, ring finger protein, 2	67027	https://www.ncbi.nlm.nih.gov/gene/?term=67027
Slc8a1	Slc8a1	solute carrier family 8 (sodium/calcium exchanger), member 1	20541	https://www.ncbi.nlm.nih.gov/gene/?term=20541
Enah	Enah	ENAH actin regulator	13800	https://www.ncbi.nlm.nih.gov/gene/?term=13800
Hoxd10	Hoxd10	homeobox D10	15430	https://www.ncbi.nlm.nih.gov/gene/?term=15430
Rer1	Rer1	retention in endoplasmic reticulum sorting receptor 1	67830	https://www.ncbi.nlm.nih.gov/gene/?term=67830
Tmem117	Tmem117	transmembrane protein 117	320709	https://www.ncbi.nlm.nih.gov/gene/?term=320709
Dmd	Dmd	dystrophin, muscular dystrophy	13405	https://www.ncbi.nlm.nih.gov/gene/?term=13405
Atp2c1	Atp2c1	ATPase, Ca++-sequestering	235574	https://www.ncbi.nlm.nih.gov/gene/?term=235574
Rbm24	Rbm24	RNA binding motif protein 24	666794	https://www.ncbi.nlm.nih.gov/gene/?term=666794
Fam160a2	Fam160a2	family with sequence similarity 160, member A2	74349	https://www.ncbi.nlm.nih.gov/gene/?term=74349
Cxadr	Cxadr	coxsackie virus and adenovirus receptor	13052	https://www.ncbi.nlm.nih.gov/gene/?term=13052
Cask	Cask	calcium/calmodulin-dependent serine protein kinase (MAGUK family)	12361	https://www.ncbi.nlm.nih.gov/gene/?term=12361

Biology Open • Supplementary information

Slc9a4	Slc9a4	solute carrier family 9 (sodium/hydrogen exchanger), member 4	110895	https://www.ncbi.nlm.nih.gov/gene/?term=110895
Jph1	Jph1	junctophilin 1	57339	https://www.ncbi.nlm.nih.gov/gene/?term=57339
Pum2	Pum2	pumilio RNA-binding family member 2	80913	https://www.ncbi.nlm.nih.gov/gene/?term=80913
Cyfip1	Cyfip1	cytoplasmic FMR1 interacting protein 1	20430	https://www.ncbi.nlm.nih.gov/gene/?term=20430
Kcnk1	Kcnk1	potassium channel, subfamily K, member 1	16525	https://www.ncbi.nlm.nih.gov/gene/?term=16525
Olfm3	Olfm3	olfactomedin 3	229759	https://www.ncbi.nlm.nih.gov/gene/?term=229759
Noc3l	Noc3l	NOC3 like DNA replication regulator	57753	https://www.ncbi.nlm.nih.gov/gene/?term=57753
Gatad1	Gatad1	GATA zinc finger domain containing 1	67210	https://www.ncbi.nlm.nih.gov/gene/?term=67210
Prl5a1	Prl5a1	prolactin family 5, subfamily a, member 1	28078	https://www.ncbi.nlm.nih.gov/gene/?term=28078
Ttpa	Ttpa	tocopherol (alpha) transfer protein	50500	https://www.ncbi.nlm.nih.gov/gene/?term=50500
Hecw2	Hecw2	HECT, C2 and WW domain containing E3 ubiquitin protein ligase 2	329152	https://www.ncbi.nlm.nih.gov/gene/?term=329152
Abi3bp	Abi3bp	ABI gene family, member 3 (NESH) binding protein	320712	https://www.ncbi.nlm.nih.gov/gene/?term=320712
Wdr26	Wdr26	WD repeat domain 26	226757	https://www.ncbi.nlm.nih.gov/gene/?term=226757
Slk	Slk	STE20-like kinase	20874	https://www.ncbi.nlm.nih.gov/gene/?term=20874
Nr2c1	Nr2c1	nuclear receptor subfamily 2, group C, member 1	22025	https://www.ncbi.nlm.nih.gov/gene/?term=22025
Cdc14a	Cdc14a	CDC14 cell division cycle 14A	229776	https://www.ncbi.nlm.nih.gov/gene/?term=229776

Biology Open • Supplementary information

Asb7	Asb7	ankyrin repeat and SOCS box-containing 7	117589	https://www.ncbi.nlm.nih.gov/gene/?term=117589
Pou2f1	Pou2f1	POU domain, class 2, transcription factor 1	18986	https://www.ncbi.nlm.nih.gov/gene/?term=18986
Fbxo30	Fbxo30	F-box protein 30	71865	https://www.ncbi.nlm.nih.gov/gene/?term=71865
Nog	Nog	noggin	18121	https://www.ncbi.nlm.nih.gov/gene/?term=18121
Tmem33	Tmem33	transmembrane protein 33	67878	https://www.ncbi.nlm.nih.gov/gene/?term=67878
Gucy1b1	Gucy1b1	guanylate cyclase 1, soluble, beta 1	54195	https://www.ncbi.nlm.nih.gov/gene/?term=54195
Smarcad1	Smarcad1	SWI/SNF-related, matrix-associated actin-dependent regulator of chromatin, subfamily a, containing DEAD/H box 1	13990	https://www.ncbi.nlm.nih.gov/gene/?term=13990
Rabgap1l	Rabgap1l	RAB GTPase activating protein 1-like	29809	https://www.ncbi.nlm.nih.gov/gene/?term=29809
Hoxb2	Hoxb2	homeobox B2	103889	https://www.ncbi.nlm.nih.gov/gene/?term=103889
Fgf12	Fgf12	fibroblast growth factor 12	14167	https://www.ncbi.nlm.nih.gov/gene/?term=14167
Map3k1	Map3k1	mitogen-activated protein kinase kinase kinase 1	26401	https://www.ncbi.nlm.nih.gov/gene/?term=26401
Sgip1	Sgip1	SH3-domain GRB2-like (endophilin) interacting protein 1	73094	https://www.ncbi.nlm.nih.gov/gene/?term=73094
Cwc25	Cwc25	CWC25 spliceosome-associated protein	67480	https://www.ncbi.nlm.nih.gov/gene/?term=67480
Prkar2b	Prkar2b	protein kinase, cAMP dependent regulatory, type II beta	19088	https://www.ncbi.nlm.nih.gov/gene/?term=19088
Rab11b	Rab11b	RAB11B, member RAS oncogene family	19326	https://www.ncbi.nlm.nih.gov/gene/?term=19326
Apaf1	Apaf1	apoptotic peptidase activating factor 1	11783	https://www.ncbi.nlm.nih.gov/gene/?term=11783

Fbxl3	Fbxl3	F-box and leucine-rich repeat protein 3	50789	https://www.ncbi.nlm.nih.gov/gene/?term=50789
Vmp1	Vmp1	vacuole membrane protein 1	75909	https://www.ncbi.nlm.nih.gov/gene/?term=75909
Xrcc5	Xrcc5	X-ray repair complementing defective repair in Chinese hamster cells 5	22596	https://www.ncbi.nlm.nih.gov/gene/?term=22596
Vps54	Vps54	VPS54 GARP complex subunit	245944	https://www.ncbi.nlm.nih.gov/gene/?term=245944
Ubxn7	Ubxn7	UBX domain protein 7	224111	https://www.ncbi.nlm.nih.gov/gene/?term=224111
Gabpa	Gabpa	GA repeat binding protein, alpha	14390	https://www.ncbi.nlm.nih.gov/gene/?term=14390
Atp13a3	Atp13a3	ATPase type 13A3	224088	https://www.ncbi.nlm.nih.gov/gene/?term=224088
D16Ertd472e	D16Ertd472e	DNA segment, Chr 16, ERATO Doi 472, expressed	67102	https://www.ncbi.nlm.nih.gov/gene/?term=67102
Pum1	Pum1	pumilio RNA-binding family member 1	80912	https://www.ncbi.nlm.nih.gov/gene/?term=80912
Fgf4	Fgf4	fibroblast growth factor 4	14175	https://www.ncbi.nlm.nih.gov/gene/?term=14175
Adamts1	Adamts1	a disintegrin-like and metallopeptidase (reprolysin type) with thrombospondin type 1 motif, 1	11504	https://www.ncbi.nlm.nih.gov/gene/?term=11504
Mcmbp	Mcmbp	minichromosome maintenance complex binding protein	210711	https://www.ncbi.nlm.nih.gov/gene/?term=210711
Uba3	Uba3	ubiquitin-like modifier activating enzyme 3	22200	https://www.ncbi.nlm.nih.gov/gene/?term=22200
Kat6b	Kat6b	K(lysine) acetyltransferase 6B	54169	https://www.ncbi.nlm.nih.gov/gene/?term=54169
Zfp503	Zfp503	zinc finger protein 503	218820	https://www.ncbi.nlm.nih.gov/gene/?term=218820
Map4k3	Map4k3	mitogen-activated protein kinase kinase kinase kinase 3	225028	https://www.ncbi.nlm.nih.gov/gene/?term=225028
Sft2d2	Sft2d2	SFT2 domain containing 2	108735	https://www.ncbi.nlm.nih.gov/gene/?term=108735

Biology Open • Supplementary information

G2e3	G2e3	G2/M-phase specific E3 ubiquitin ligase	217558	https://www.ncbi.nlm.nih.gov/gene/?term=217558
Pabpc5	Pabpc5	poly(A) binding protein, cytoplasmic 5	93728	https://www.ncbi.nlm.nih.gov/gene/?term=93728
Nxt2	Nxt2	nuclear transport factor 2-like export factor 2	237082	https://www.ncbi.nlm.nih.gov/gene/?term=237082
Btf3l4	Btf3l4	basic transcription factor 3-like 4	70533	https://www.ncbi.nlm.nih.gov/gene/?term=70533
Arl5a	Arl5a	ADP-ribosylation factor-like 5A	75423	https://www.ncbi.nlm.nih.gov/gene/?term=75423
Prpmp5	Prpmp5	proline-rich protein MP5	381832	https://www.ncbi.nlm.nih.gov/gene/?term=381832
Ice1	Ice1	interactor of little elongation complex ELL subunit 1	218333	https://www.ncbi.nlm.nih.gov/gene/?term=218333
Acvr2a	Acvr2a	activin receptor IIA	11480	https://www.ncbi.nlm.nih.gov/gene/?term=11480
Atad1	Atad1	ATPase family, AAA domain containing 1	67979	https://www.ncbi.nlm.nih.gov/gene/?term=67979
Cdh12	Cdh12	cadherin 12	215654	https://www.ncbi.nlm.nih.gov/gene/?term=215654
Blcap	Blcap	bladder cancer associated protein	53619	https://www.ncbi.nlm.nih.gov/gene/?term=53619
Rbms1	Rbms1	RNA binding motif, single stranded interacting protein 1	56878	https://www.ncbi.nlm.nih.gov/gene/?term=56878
Sumo2	Sumo2	small ubiquitin-like modifier 2	170930	https://www.ncbi.nlm.nih.gov/gene/?term=170930
Pip5k1b	Pip5k1b	phosphatidylinositol-4-phosphate 5-kinase, type 1 beta	18719	https://www.ncbi.nlm.nih.gov/gene/?term=18719
Slc24a5	Slc24a5	solute carrier family 24, member 5	317750	https://www.ncbi.nlm.nih.gov/gene/?term=317750
Gm6710	Gm6710	predicted gene 6710	626832	https://www.ncbi.nlm.nih.gov/gene/?term=626832
Greb1	Greb1	gene regulated by estrogen in breast cancer protein	268527	https://www.ncbi.nlm.nih.gov/gene/?term=268527
Mical2	Mical2	microtubule associated monooxygenase, calponin and LIM domain containing 2	320878	https://www.ncbi.nlm.nih.gov/gene/?term=320878

Biology Open • Supplementary information

Shq1	Shq1	SHQ1 homolog (S. cerevisiae)	72171	https://www.ncbi.nlm.nih.gov/gene/?term=72171
Peli1	Peli1	pellino 1	67245	https://www.ncbi.nlm.nih.gov/gene/?term=67245
Trp63	Trp63	transformation related protein 63	22061	https://www.ncbi.nlm.nih.gov/gene/?term=22061
Srgap1	Srgap1	SLIT-ROBO Rho GTPase activating protein 1	117600	https://www.ncbi.nlm.nih.gov/gene/?term=117600
Tmem209	Tmem209	transmembrane protein 209	72649	https://www.ncbi.nlm.nih.gov/gene/?term=72649
Snx3	Snx3	sorting nexin 3	54198	https://www.ncbi.nlm.nih.gov/gene/?term=54198
Mtus1	Mtus1	mitochondrial tumor suppressor 1	102103	https://www.ncbi.nlm.nih.gov/gene/?term=102103
Phldb2	Phldb2	pleckstrin homology like domain, family B, member 2	208177	https://www.ncbi.nlm.nih.gov/gene/?term=208177
Fgf13	Fgf13	fibroblast growth factor 13	14168	https://www.ncbi.nlm.nih.gov/gene/?term=14168
Senp5	Senp5	SUMO/sentrin specific peptidase 5	320213	https://www.ncbi.nlm.nih.gov/gene/?term=320213
Pdcd4	Pdcd4	programmed cell death 4	18569	https://www.ncbi.nlm.nih.gov/gene/?term=18569
Igfbp3	Igfbp3	insulin-like growth factor binding protein 3	16009	https://www.ncbi.nlm.nih.gov/gene/?term=16009
Tpp2	Tpp2	tripeptidyl peptidase II	22019	https://www.ncbi.nlm.nih.gov/gene/?term=22019
Kctd14	Kctd14	potassium channel tetramerisation domain containing 14	233529	https://www.ncbi.nlm.nih.gov/gene/?term=233529
Zfp652	Zfp652	zinc finger protein 652	268469	https://www.ncbi.nlm.nih.gov/gene/?term=268469
Arhgef26	Arhgef26	Rho guanine nucleotide exchange factor (GEF) 26	622434	https://www.ncbi.nlm.nih.gov/gene/?term=622434
Krtap20-2	Krtap20-2	keratin associated protein 20-2	622935	https://www.ncbi.nlm.nih.gov/gene/?term=622935
Zdhhc17	Zdhhc17	zinc finger, DHHC domain containing 17	320150	https://www.ncbi.nlm.nih.gov/gene/?term=320150
Klhl2	Klhl2	kelch-like 2, Mayven	77113	https://www.ncbi.nlm.nih.gov/gene/?term=77113
Nck1	Nck1	non-catalytic region of tyrosine kinase adaptor protein 1	17973	https://www.ncbi.nlm.nih.gov/gene/?term=17973

Biology Open • Supplementary information

Eif3j1	Eif3j1	eukaryotic translation initiation factor 3, subunit J1	78655	https://www.ncbi.nlm.nih.gov/gene/?term=78655
Atxn7l2	Atxn7l2	ataxin 7-like 2	72522	https://www.ncbi.nlm.nih.gov/gene/?term=72522
Galnt3	Galnt3	polypeptide N-acetylgalactosaminyltransferase 3	14425	https://www.ncbi.nlm.nih.gov/gene/?term=14425
Secisbp2l	Secisbp2l	SECIS binding protein 2-like	70354	https://www.ncbi.nlm.nih.gov/gene/?term=70354
Ppm1b	Ppm1b	protein phosphatase 1B, magnesium dependent, beta isoform	19043	https://www.ncbi.nlm.nih.gov/gene/?term=19043
Rspo3	Rspo3	R-spondin 3	72780	https://www.ncbi.nlm.nih.gov/gene/?term=72780
Tmem245	Tmem245	transmembrane protein 245	242474	https://www.ncbi.nlm.nih.gov/gene/?term=242474
Ipmk	Ipmk	inositol polyphosphate multikinase	69718	https://www.ncbi.nlm.nih.gov/gene/?term=69718
Hnrnph3	Hnrnph3	heterogeneous nuclear ribonucleoprotein H3	432467	https://www.ncbi.nlm.nih.gov/gene/?term=432467
Mphosph9	Mphosph9	M-phase phosphoprotein 9	269702	https://www.ncbi.nlm.nih.gov/gene/?term=269702
Eif5a2	Eif5a2	eukaryotic translation initiation factor 5A2	208691	https://www.ncbi.nlm.nih.gov/gene/?term=208691
Caprin2	Caprin2	caprin family member 2	232560	https://www.ncbi.nlm.nih.gov/gene/?term=232560
Dnajc25	Dnajc25	DnaJ heat shock protein family (Hsp40) member C25	72429	https://www.ncbi.nlm.nih.gov/gene/?term=72429
Syde2	Syde2	synapse defective 1, Rho GTPase, homolog 2 (C. elegans)	214804	https://www.ncbi.nlm.nih.gov/gene/?term=214804
Pgm3	Pgm3	phosphoglucomutase 3	109785	https://www.ncbi.nlm.nih.gov/gene/?term=109785
Tdo2	Tdo2	tryptophan 2,3-dioxygenase	56720	https://www.ncbi.nlm.nih.gov/gene/?term=56720
Sgce	Sgce	sarcoglycan, epsilon	20392	https://www.ncbi.nlm.nih.gov/gene/?term=20392
Jam3	Jam3	junction adhesion molecule 3	83964	https://www.ncbi.nlm.nih.gov/gene/?term=83964
Mylip	Mylip	myosin regulatory light chain interacting protein	218203	https://www.ncbi.nlm.nih.gov/gene/?term=218203

Biology Open • Supplementary information

Trank1	Trank1	tetratricopeptide repeat and ankyrin repeat containing 1	320429	https://www.ncbi.nlm.nih.gov/gene/?term=320429
Zdhhc15	Zdhhc15	zinc finger, DHHC domain containing 15	108672	https://www.ncbi.nlm.nih.gov/gene/?term=108672
Stxbp4	Stxbp4	syntaxin binding protein 4	20913	https://www.ncbi.nlm.nih.gov/gene/?term=20913
Thsd7a	Thsd7a	thrombospondin, type I, domain containing 7A	330267	https://www.ncbi.nlm.nih.gov/gene/?term=330267
Lrig3	Lrig3	leucine-rich repeats and immunoglobulin-like domains 3	320398	https://www.ncbi.nlm.nih.gov/gene/?term=320398
Cep57	Cep57	centrosomal protein 57	74360	https://www.ncbi.nlm.nih.gov/gene/?term=74360
Rev3l	Rev3l	REV3 like, DNA directed polymerase zeta catalytic subunit	19714	https://www.ncbi.nlm.nih.gov/gene/?term=19714
Plat	Plat	plasminogen activator, tissue	18791	https://www.ncbi.nlm.nih.gov/gene/?term=18791
Ddx42	Ddx42	DEAD (Asp-Glu-Ala-Asp) box polypeptide 42	72047	https://www.ncbi.nlm.nih.gov/gene/?term=72047
Dlgap2	Dlgap2	DLG associated protein 2	244310	https://www.ncbi.nlm.nih.gov/gene/?term=244310
Kcnh5	Kcnh5	potassium voltage-gated channel, subfamily H (eag-related), member 5	238271	https://www.ncbi.nlm.nih.gov/gene/?term=238271
Rnf111	Rnf111	ring finger 111	93836	https://www.ncbi.nlm.nih.gov/gene/?term=93836
Fgf7	Fgf7	fibroblast growth factor 7	14178	https://www.ncbi.nlm.nih.gov/gene/?term=14178
Arf6	Arf6	ADP-ribosylation factor 6	11845	https://www.ncbi.nlm.nih.gov/gene/?term=11845
Tmem64	Tmem64	transmembrane protein 64	100201	https://www.ncbi.nlm.nih.gov/gene/?term=100201
Eif3j2	Eif3j2	eukaryotic translation initiation factor 3, subunit J2	100042807	https://www.ncbi.nlm.nih.gov/gene/?term=100042[illegible]
Esyt2	Esyt2	extended synaptotagmin-like protein 2	52635	https://www.ncbi.nlm.nih.gov/gene/?term=52635

Biology Open • Supplementary information

Xpot	Xpot	exportin, tRNA (nuclear export receptor for tRNAs)	73192	https://www.ncbi.nlm.nih.gov/gene/?term=73192
Syap1	Syap1	synapse associated protein 1	67043	https://www.ncbi.nlm.nih.gov/gene/?term=67043
Plppr5	Plppr5	phospholipid phosphatase related 5	75769	https://www.ncbi.nlm.nih.gov/gene/?term=75769
Myo6	Myo6	myosin VI	17920	https://www.ncbi.nlm.nih.gov/gene/?term=17920
Rif1	Rif1	replication timing regulatory factor 1	51869	https://www.ncbi.nlm.nih.gov/gene/?term=51869
Sbno2	Sbno2	strawberry notch 2	216161	https://www.ncbi.nlm.nih.gov/gene/?term=216161
Arl13b	Arl13b	ADP-ribosylation factor-like 13B	68146	https://www.ncbi.nlm.nih.gov/gene/?term=68146
Cyp2c70	Cyp2c70	cytochrome P450, family 2, subfamily c, polypeptide 70	226105	https://www.ncbi.nlm.nih.gov/gene/?term=226105
1810024B03Rik	1810024B03Rik	RIKEN cDNA 1810024B03 gene	329509	https://www.ncbi.nlm.nih.gov/gene/?term=329509
Usp25	Usp25	ubiquitin specific peptidase 25	30940	https://www.ncbi.nlm.nih.gov/gene/?term=30940
Arg1	Arg1	arginase, liver	11846	https://www.ncbi.nlm.nih.gov/gene/?term=11846
R3hdml	R3hdml	R3H domain containing-like	100043899	https://www.ncbi.nlm.nih.gov/gene/?term=100043
Kif13a	Kif13a	kinesin family member 13A	16553	https://www.ncbi.nlm.nih.gov/gene/?term=16553
Cdh9	Cdh9	cadherin 9	12565	https://www.ncbi.nlm.nih.gov/gene/?term=12565
Galnt13	Galnt13	polypeptide N-acetylgalactosaminyltransferase 13	271786	https://www.ncbi.nlm.nih.gov/gene/?term=271786
Rab11a	Rab11a	RAB11A, member RAS oncogene family	53869	https://www.ncbi.nlm.nih.gov/gene/?term=53869
Fam234b	Fam234b	family with sequence similarity 234, member B	74525	https://www.ncbi.nlm.nih.gov/gene/?term=74525
Mecom	Mecom	MDS1 and EVI1 complex locus	14013	https://www.ncbi.nlm.nih.gov/gene/?term=14013
Ewsr1	Ewsr1	Ewing sarcoma breakpoint region 1	14030	https://www.ncbi.nlm.nih.gov/gene/?term=14030

Biology Open • Supplementary information

Kcnn3	Kcnn3	potassium intermediate/small conductance calcium-activated channel, subfamily N, member 3	140493	https://www.ncbi.nlm.nih.gov/gene/?term=140493
Eps8l2	Eps8l2	EPS8-like 2	98845	https://www.ncbi.nlm.nih.gov/gene/?term=98845
Ccser2	Ccser2	coiled-coil serine rich 2	72972	https://www.ncbi.nlm.nih.gov/gene/?term=72972
Pak7	Pak7	p21 (RAC1) activated kinase 7	241656	https://www.ncbi.nlm.nih.gov/gene/?term=241656
Cd38	Cd38	CD38 antigen	12494	https://www.ncbi.nlm.nih.gov/gene/?term=12494
Lin9	Lin9	lin-9 homolog (C. elegans)	72568	https://www.ncbi.nlm.nih.gov/gene/?term=72568
Dtl	Dtl	denticleless E3 ubiquitin protein ligase	76843	https://www.ncbi.nlm.nih.gov/gene/?term=76843
Lhx9	Lhx9	LIM homeobox protein 9	16876	https://www.ncbi.nlm.nih.gov/gene/?term=16876
Uqcc1	Uqcc1	ubiquinol-cytochrome c reductase complex assembly factor 1	56046	https://www.ncbi.nlm.nih.gov/gene/?term=56046
2810459M11Rik	2810459M11Rik	RIKEN cDNA 2810459M11 gene	72792	https://www.ncbi.nlm.nih.gov/gene/?term=72792
Ext1	Ext1	exostoses (multiple) 1	14042	https://www.ncbi.nlm.nih.gov/gene/?term=14042
Ddo	Ddo	D-aspartate oxidase	70503	https://www.ncbi.nlm.nih.gov/gene/?term=70503
Gsk3b	Gsk3b	glycogen synthase kinase 3 beta	56637	https://www.ncbi.nlm.nih.gov/gene/?term=56637
Dact1	Dact1	dishevelled-binding antagonist of beta-catenin 1	59036	https://www.ncbi.nlm.nih.gov/gene/?term=59036
Slc30a7	Slc30a7	solute carrier family 30 (zinc transporter), member 7	66500	https://www.ncbi.nlm.nih.gov/gene/?term=66500
Ppm1j	Ppm1j	protein phosphatase 1J	71887	https://www.ncbi.nlm.nih.gov/gene/?term=71887
Plscr4	Plscr4	phospholipid scramblase 4	235527	https://www.ncbi.nlm.nih.gov/gene/?term=235527
Cdip1	Cdip1	cell death inducing Trp53 target 1	66626	https://www.ncbi.nlm.nih.gov/gene/?term=66626
Hoxc4	Hoxc4	homeobox C4	15423	https://www.ncbi.nlm.nih.gov/gene/?term=15423
Uvssa	Uvssa	UV stimulated scaffold protein A	71101	https://www.ncbi.nlm.nih.gov/gene/?term=71101
Folr1	Folr1	folate receptor 1 (adult)	14275	https://www.ncbi.nlm.nih.gov/gene/?term=14275

Zc2hc1c	Zc2hc1c	zinc finger, C2HC-type containing 1C	72350	https://www.ncbi.nlm.nih.gov/gene/?term=72350
Perp	Perp	PERP, TP53 apoptosis effector	64058	https://www.ncbi.nlm.nih.gov/gene/?term=64058
Gm4984	Gm4984	predicted pseudogene 4984	245347	https://www.ncbi.nlm.nih.gov/gene/?term=245347
Krtap12-1	Krtap12-1	keratin associated protein 12-1	16694	https://www.ncbi.nlm.nih.gov/gene/?term=16694
Mark1	Mark1	MAP/microtubule affinity regulating kinase 1	226778	https://www.ncbi.nlm.nih.gov/gene/?term=226778
Plcxd2	Plcxd2	phosphatidylinositol-specific phospholipase C, X domain containing 2	433022	https://www.ncbi.nlm.nih.gov/gene/?term=433022
Chodl	Chodl	chondrolectin	246048	https://www.ncbi.nlm.nih.gov/gene/?term=246048
Uggt1	Uggt1	UDP-glucose glycoprotein glucosyltransferase 1	320011	https://www.ncbi.nlm.nih.gov/gene/?term=320011
Slc16a1	Slc16a1	solute carrier family 16 (monocarboxylic acid transporters), member 1	20501	https://www.ncbi.nlm.nih.gov/gene/?term=20501
Nifk	Nifk	nucleolar protein interacting with the FHA domain of MKI67	67949	https://www.ncbi.nlm.nih.gov/gene/?term=67949
Camsap1	Camsap1	calmodulin regulated spectrin-associated protein 1	227634	https://www.ncbi.nlm.nih.gov/gene/?term=227634
Adam9	Adam9	a disintegrin and metallopeptidase domain 9 (meltrin gamma)	11502	https://www.ncbi.nlm.nih.gov/gene/?term=11502
Rftn1	Rftn1	raftlin lipid raft linker 1	76438	https://www.ncbi.nlm.nih.gov/gene/?term=76438
Neurod4	Neurod4	neurogenic differentiation 4	11923	https://www.ncbi.nlm.nih.gov/gene/?term=11923
Kctd12	Kctd12	potassium channel tetramerisation domain containing 12	239217	https://www.ncbi.nlm.nih.gov/gene/?term=239217
Zfp612	Zfp612	zinc finger protein 612	234725	https://www.ncbi.nlm.nih.gov/gene/?term=234725
Magee2	Magee2	melanoma antigen, family E, 2	272790	https://www.ncbi.nlm.nih.gov/gene/?term=272790

Biology Open • Supplementary information

Tacc1	Tacc1	transforming, acidic coiled-coil containing protein 1	320165	https://www.ncbi.nlm.nih.gov/gene/?term=320165
Caps2	Caps2	calcyphosphine 2	353025	https://www.ncbi.nlm.nih.gov/gene/?term=353025
Henmt1	Henmt1	HEN1 methyltransferase homolog 1 (Arabidopsis)	66715	https://www.ncbi.nlm.nih.gov/gene/?term=66715
Rptor	Rptor	regulatory associated protein of MTOR, complex 1	74370	https://www.ncbi.nlm.nih.gov/gene/?term=74370
Havcr1	Havcr1	hepatitis A virus cellular receptor 1	171283	https://www.ncbi.nlm.nih.gov/gene/?term=171283
Ermap	Ermap	erythroblast membrane-associated protein	27028	https://www.ncbi.nlm.nih.gov/gene/?term=27028
Tram1	Tram1	translocating chain-associating membrane protein 1	72265	https://www.ncbi.nlm.nih.gov/gene/?term=72265
Hspa12a	Hspa12a	heat shock protein 12A	73442	https://www.ncbi.nlm.nih.gov/gene/?term=73442
Pakap	Pakap	paralemmin A kinase anchor protein	677884	https://www.ncbi.nlm.nih.gov/gene/?term=677884
Fgf23	Fgf23	fibroblast growth factor 23	64654	https://www.ncbi.nlm.nih.gov/gene/?term=64654
Sort1	Sort1	sortilin 1	20661	https://www.ncbi.nlm.nih.gov/gene/?term=20661
Plxnb1	Plxnb1	plexin B1	235611	https://www.ncbi.nlm.nih.gov/gene/?term=235611
Gdap2	Gdap2	ganglioside-induced differentiation-associated-protein 2	14547	https://www.ncbi.nlm.nih.gov/gene/?term=14547
Cep350	Cep350	centrosomal protein 350	74081	https://www.ncbi.nlm.nih.gov/gene/?term=74081
Cldnd1	Cldnd1	claudin domain containing 1	224250	https://www.ncbi.nlm.nih.gov/gene/?term=224250
Rbpjl	Rbpjl	recombination signal binding protein for immunoglobulin kappa J region-like	19668	https://www.ncbi.nlm.nih.gov/gene/?term=19668
Tab2	Tab2	TGF-beta activated kinase 1/MAP3K7 binding protein 2	68652	https://www.ncbi.nlm.nih.gov/gene/?term=68652

Biology Open • Supplementary information

Snd1	Snd1	staphylococcal nuclease and tudor domain containing 1	56463	https://www.ncbi.nlm.nih.gov/gene/?term=56463
Sorbs1	Sorbs1	sorbin and SH3 domain containing 1	20411	https://www.ncbi.nlm.nih.gov/gene/?term=20411
Car8	Car8	carbonic anhydrase 8	12319	https://www.ncbi.nlm.nih.gov/gene/?term=12319
Ripk4	Ripk4	receptor-interacting serine-threonine kinase 4	72388	https://www.ncbi.nlm.nih.gov/gene/?term=72388
Dusp6	Dusp6	dual specificity phosphatase 6	67603	https://www.ncbi.nlm.nih.gov/gene/?term=67603
Rcvrn	Rcvrn	recoverin	19674	https://www.ncbi.nlm.nih.gov/gene/?term=19674
Tm9sf2	Tm9sf2	transmembrane 9 superfamily member 2	68059	https://www.ncbi.nlm.nih.gov/gene/?term=68059
Tbc1d4	Tbc1d4	TBC1 domain family, member 4	210789	https://www.ncbi.nlm.nih.gov/gene/?term=210789
Trim44	Trim44	tripartite motif-containing 44	80985	https://www.ncbi.nlm.nih.gov/gene/?term=80985
Zdhhc3	Zdhhc3	zinc finger, DHHC domain containing 3	69035	https://www.ncbi.nlm.nih.gov/gene/?term=69035
Adamts12	Adamts12	a disintegrin-like and metallopeptidase (reprolysin type) with thrombospondin type 1 motif, 12	239337	https://www.ncbi.nlm.nih.gov/gene/?term=239337
2010315B03Rik	2010315B03Rik	RIKEN cDNA 2010315B03 gene	630836	https://www.ncbi.nlm.nih.gov/gene/?term=630836
Tmed8	Tmed8	transmembrane p24 trafficking protein 8	382620	https://www.ncbi.nlm.nih.gov/gene/?term=382620
Mgat4a	Mgat4a	mannoside acetylglucosaminyltransferase 4, isoenzyme A	269181	https://www.ncbi.nlm.nih.gov/gene/?term=269181
Chmp7	Chmp7	charged multivesicular body protein 7	105513	https://www.ncbi.nlm.nih.gov/gene/?term=105513
Hlf	Hlf	hepatic leukemia factor	217082	https://www.ncbi.nlm.nih.gov/gene/?term=217082
Trim16	Trim16	tripartite motif-containing 16	94092	https://www.ncbi.nlm.nih.gov/gene/?term=94092

Biology Open • Supplementary information

Rbm25	Rbm25	RNA binding motif protein 25	67039	https://www.ncbi.nlm.nih.gov/gene/?term=67039
Retnlb	Retnlb	resistin like beta	57263	https://www.ncbi.nlm.nih.gov/gene/?term=57263
Pcdh7	Pcdh7	protocadherin 7	54216	https://www.ncbi.nlm.nih.gov/gene/?term=54216
Nrxn1	Nrxn1	neurexin I	18189	https://www.ncbi.nlm.nih.gov/gene/?term=18189
Lef1	Lef1	lymphoid enhancer binding factor 1	16842	https://www.ncbi.nlm.nih.gov/gene/?term=16842
Tmprss2	Tmprss2	transmembrane protease, serine 2	50528	https://www.ncbi.nlm.nih.gov/gene/?term=50528
Rpgrip1l	Rpgrip1l	Rpgrip1-like	244585	https://www.ncbi.nlm.nih.gov/gene/?term=244585
Mmp1b	Mmp1b	matrix metallopeptidase 1b (interstitial collagenase)	83996	https://www.ncbi.nlm.nih.gov/gene/?term=83996
Stk3	Stk3	serine/threonine kinase 3	56274	https://www.ncbi.nlm.nih.gov/gene/?term=56274
Gm11541	Gm11541	predicted gene 11541	432589	https://www.ncbi.nlm.nih.gov/gene/?term=432589
Cxcl16	Cxcl16	chemokine (C-X-C motif) ligand 16	66102	https://www.ncbi.nlm.nih.gov/gene/?term=66102
Hs3st2	Hs3st2	heparan sulfate (glucosamine) 3-O-sulfotransferase 2	195646	https://www.ncbi.nlm.nih.gov/gene/?term=195646
Ttc39a	Ttc39a	tetratricopeptide repeat domain 39A	230603	https://www.ncbi.nlm.nih.gov/gene/?term=230603
Hs3st3b1	Hs3st3b1	heparan sulfate (glucosamine) 3-O-sulfotransferase 3B1	54710	https://www.ncbi.nlm.nih.gov/gene/?term=54710
Fgf21	Fgf21	NA	56636	https://www.ncbi.nlm.nih.gov/gene/?term=56636
Ap1ar	Ap1ar	adaptor-related protein complex 1 associated regulatory protein	211556	https://www.ncbi.nlm.nih.gov/gene/?term=211556
Baz2a	Baz2a	bromodomain adjacent to zinc finger domain, 2A	116848	https://www.ncbi.nlm.nih.gov/gene/?term=116848
Rtl6	Rtl6	retrotransposon Gag like 6	223732	https://www.ncbi.nlm.nih.gov/gene/?term=223732
Dmtn	Dmtn	dematin actin binding protein	13829	https://www.ncbi.nlm.nih.gov/gene/?term=13829
Kdm2a	Kdm2a	lysine (K)-specific demethylase 2A	225876	https://www.ncbi.nlm.nih.gov/gene/?term=225876
Adss	Adss	adenylosuccinate synthetase, non muscle	11566	https://www.ncbi.nlm.nih.gov/gene/?term=11566

Biology Open • Supplementary information

Ar	Ar	androgen receptor	11835	https://www.ncbi.nlm.nih.gov/gene/?term=11835
Cd209b	Cd209b	CD209b antigen	69165	https://www.ncbi.nlm.nih.gov/gene/?term=69165
B3galt1	B3galt1	UDP-Gal:betaGlcNAc beta 1,3-galactosyltransferase, polypeptide 1	26877	https://www.ncbi.nlm.nih.gov/gene/?term=26877
Fam155a	Fam155a	family with sequence similarity 155, member A	270028	https://www.ncbi.nlm.nih.gov/gene/?term=270028
Rspo4	Rspo4	R-spondin 4	228770	https://www.ncbi.nlm.nih.gov/gene/?term=228770
Ndst3	Ndst3	N-deacetylase/N-sulfotransferase (heparan glucosaminyl) 3	83398	https://www.ncbi.nlm.nih.gov/gene/?term=83398
Nectin3	Nectin3	nectin cell adhesion molecule 3	58998	https://www.ncbi.nlm.nih.gov/gene/?term=58998
Abhd2	Abhd2	abhydrolase domain containing 2	54608	https://www.ncbi.nlm.nih.gov/gene/?term=54608
Mmp16	Mmp16	matrix metallopeptidase 16	17389	https://www.ncbi.nlm.nih.gov/gene/?term=17389
A430033K04Rik	A430033K04Rik	RIKEN cDNA A430033K04 gene	243308	https://www.ncbi.nlm.nih.gov/gene/?term=243308
Abcg4	Abcg4	ATP binding cassette subfamily G member 4	192663	https://www.ncbi.nlm.nih.gov/gene/?term=192663
Trim41	Trim41	tripartite motif-containing 41	211007	https://www.ncbi.nlm.nih.gov/gene/?term=211007
Pcp4	Pcp4	Purkinje cell protein 4	18546	https://www.ncbi.nlm.nih.gov/gene/?term=18546
Ehd1	Ehd1	EH-domain containing 1	13660	https://www.ncbi.nlm.nih.gov/gene/?term=13660
Ces2g	Ces2g	carboxylesterase 2G	72361	https://www.ncbi.nlm.nih.gov/gene/?term=72361
Hey2	Hey2	hairy/enhancer-of-split related with YRPW motif 2	15214	https://www.ncbi.nlm.nih.gov/gene/?term=15214
Tmem25	Tmem25	transmembrane protein 25	71687	https://www.ncbi.nlm.nih.gov/gene/?term=71687
Tomm22	Tomm22	translocase of outer mitochondrial membrane 22	223696	https://www.ncbi.nlm.nih.gov/gene/?term=223696
Mmp17	Mmp17	matrix metallopeptidase 17	23948	https://www.ncbi.nlm.nih.gov/gene/?term=23948
D430041D05Rik	D430041D05Rik	RIKEN cDNA D430041D05 gene	241589	https://www.ncbi.nlm.nih.gov/gene/?term=241589

Camk1d	Camk1d	calcium/calmodulin-dependent protein kinase ID	227541	https://www.ncbi.nlm.nih.gov/gene/?term=227541
Pou2f2	Pou2f2	POU domain, class 2, transcription factor 2	18987	https://www.ncbi.nlm.nih.gov/gene/?term=18987
Znrf3	Znrf3	zinc and ring finger 3	407821	https://www.ncbi.nlm.nih.gov/gene/?term=407821
Reep6	Reep6	receptor accessory protein 6	70335	https://www.ncbi.nlm.nih.gov/gene/?term=70335
Zc3h6	Zc3h6	zinc finger CCCH type containing 6	78751	https://www.ncbi.nlm.nih.gov/gene/?term=78751
Tead1	Tead1	TEA domain family member 1	21676	https://www.ncbi.nlm.nih.gov/gene/?term=21676
Aida	Aida	axin interactor, dorsalization associated	108909	https://www.ncbi.nlm.nih.gov/gene/?term=108909
Slc7a7	Slc7a7	solute carrier family 7 (cationic amino acid transporter, y+ system), member 7	20540	https://www.ncbi.nlm.nih.gov/gene/?term=20540
Slc10a7	Slc10a7	solute carrier family 10 (sodium/bile acid cotransporter family), member 7	76775	https://www.ncbi.nlm.nih.gov/gene/?term=76775
Ptgfr	Ptgfr	prostaglandin F receptor	19220	https://www.ncbi.nlm.nih.gov/gene/?term=19220
Slc35b3	Slc35b3	solute carrier family 35, member B3	108652	https://www.ncbi.nlm.nih.gov/gene/?term=108652
Neurod6	Neurod6	neurogenic differentiation 6	11922	https://www.ncbi.nlm.nih.gov/gene/?term=11922
Zfx	Zfx	zinc finger protein X-linked	22764	https://www.ncbi.nlm.nih.gov/gene/?term=22764
Smad5	Smad5	SMAD family member 5	17129	https://www.ncbi.nlm.nih.gov/gene/?term=17129
Camk2d	Camk2d	calcium/calmodulin-dependent protein kinase II, delta	108058	https://www.ncbi.nlm.nih.gov/gene/?term=108058
Aqp4	Aqp4	aquaporin 4	11829	https://www.ncbi.nlm.nih.gov/gene/?term=11829
Mapk1ip1l	Mapk1ip1l	mitogen-activated protein kinase 1 interacting protein 1-like	218975	https://www.ncbi.nlm.nih.gov/gene/?term=218975
Pramel3	Pramel3	preferentially expressed antigen in melanoma-like 3	83565	https://www.ncbi.nlm.nih.gov/gene/?term=83565

Biology Open • Supplementary information

Rsrp1	Rsrp1	arginine/serine rich protein 1	27981	https://www.ncbi.nlm.nih.gov/gene/?term=27981
Bcat1	Bcat1	branched chain aminotransferase 1, cytosolic	12035	https://www.ncbi.nlm.nih.gov/gene/?term=12035
Caprin1	Caprin1	cell cycle associated protein 1	53872	https://www.ncbi.nlm.nih.gov/gene/?term=53872
Osbpl8	Osbpl8	oxysterol binding protein-like 8	237542	https://www.ncbi.nlm.nih.gov/gene/?term=237542
Ptpdc1	Ptpdc1	protein tyrosine phosphatase domain containing 1	218232	https://www.ncbi.nlm.nih.gov/gene/?term=218232
Gabra4	Gabra4	gamma-aminobutyric acid (GABA) A receptor, subunit alpha 4	14397	https://www.ncbi.nlm.nih.gov/gene/?term=14397
Cers6	Cers6	ceramide synthase 6	241447	https://www.ncbi.nlm.nih.gov/gene/?term=241447
AV320801	AV320801	expressed sequence AV320801	331531	https://www.ncbi.nlm.nih.gov/gene/?term=331531
Pgap1	Pgap1	post-GPI attachment to proteins 1	241062	https://www.ncbi.nlm.nih.gov/gene/?term=241062
Hoxa5	Hoxa5	homeobox A5	15402	https://www.ncbi.nlm.nih.gov/gene/?term=15402
Rag1	Rag1	recombination activating gene 1	19373	https://www.ncbi.nlm.nih.gov/gene/?term=19373
Ccdc126	Ccdc126	coiled-coil domain containing 126	57895	https://www.ncbi.nlm.nih.gov/gene/?term=57895
Ctdspl2	Ctdspl2	CTD (carboxy-terminal domain, RNA polymerase II, polypeptide A) small phosphatase like 2	329506	https://www.ncbi.nlm.nih.gov/gene/?term=329506
Phf19	Phf19	PHD finger protein 19	74016	https://www.ncbi.nlm.nih.gov/gene/?term=74016
Hspa5	Hspa5	heat shock protein 5	14828	https://www.ncbi.nlm.nih.gov/gene/?term=14828
Ola1	Ola1	Obg-like ATPase 1	67059	https://www.ncbi.nlm.nih.gov/gene/?term=67059
D6Wsu163e	D6Wsu163e	DNA segment, Chr 6, Wayne State University 163, expressed	28040	https://www.ncbi.nlm.nih.gov/gene/?term=28040
Kdm4a	Kdm4a	lysine (K)-specific demethylase 4A	230674	https://www.ncbi.nlm.nih.gov/gene/?term=230674
Bhlhe22	Bhlhe22	basic helix-loop-helix family, member e22	59058	https://www.ncbi.nlm.nih.gov/gene/?term=59058
Lclat1	Lclat1	lysocardiolipin acyltransferase 1	225010	https://www.ncbi.nlm.nih.gov/gene/?term=225010

Biology Open • Supplementary information

Eef1e1	Eef1e1	eukaryotic translation elongation factor 1 epsilon 1	66143	https://www.ncbi.nlm.nih.gov/gene/?term=66143
Polr3a	Polr3a	polymerase (RNA) III (DNA directed) polypeptide A	218832	https://www.ncbi.nlm.nih.gov/gene/?term=218832
D630023F18Rik	D630023F18Rik	RIKEN cDNA D630023F18 gene	98303	https://www.ncbi.nlm.nih.gov/gene/?term=98303
Cbfb	Cbfb	core binding factor beta	12400	https://www.ncbi.nlm.nih.gov/gene/?term=12400
Nsrp1	Nsrp1	nuclear speckle regulatory protein 1	237859	https://www.ncbi.nlm.nih.gov/gene/?term=237859
Gfpt1	Gfpt1	glutamine fructose-6-phosphate transaminase 1	14583	https://www.ncbi.nlm.nih.gov/gene/?term=14583
Pank3	Pank3	pantothenate kinase 3	211347	https://www.ncbi.nlm.nih.gov/gene/?term=211347
Fkbp7	Fkbp7	FK506 binding protein 7	14231	https://www.ncbi.nlm.nih.gov/gene/?term=14231
Phlpp1	Phlpp1	PH domain and leucine rich repeat protein phosphatase 1	98432	https://www.ncbi.nlm.nih.gov/gene/?term=98432
Nsd3	Nsd3	nuclear receptor binding SET domain protein 3	234135	https://www.ncbi.nlm.nih.gov/gene/?term=234135
Pex13	Pex13	peroxisomal biogenesis factor 13	72129	https://www.ncbi.nlm.nih.gov/gene/?term=72129
Rbm12	Rbm12	RNA binding motif protein 12	75710	https://www.ncbi.nlm.nih.gov/gene/?term=75710
Ptprf	Ptprf	protein tyrosine phosphatase, receptor type, F	19268	https://www.ncbi.nlm.nih.gov/gene/?term=19268
Car13	Car13	carbonic anhydrase 13	71934	https://www.ncbi.nlm.nih.gov/gene/?term=71934
Tmigd3	Tmigd3	transmembrane and immunoglobulin domain containing 3	69296	https://www.ncbi.nlm.nih.gov/gene/?term=69296
Cbx5	Cbx5	chromobox 5	12419	https://www.ncbi.nlm.nih.gov/gene/?term=12419
Gm5796	Gm5796	predicted gene 5796	545007	https://www.ncbi.nlm.nih.gov/gene/?term=545007

Biology Open • Supplementary information

Plekhg4	Plekhg4	pleckstrin homology domain containing, family G (with RhoGef domain) member 4	102075	https://www.ncbi.nlm.nih.gov/gene/?term=102075
Tm9sf3	Tm9sf3	transmembrane 9 superfamily member 3	107358	https://www.ncbi.nlm.nih.gov/gene/?term=107358
Qtrt2	Qtrt2	queuine tRNA-ribosyltransferase accessory subunit 2	106248	https://www.ncbi.nlm.nih.gov/gene/?term=106248
Adora3	Adora3	adenosine A3 receptor	11542	https://www.ncbi.nlm.nih.gov/gene/?term=11542
Nhsl1	Nhsl1	NHS-like 1	215819	https://www.ncbi.nlm.nih.gov/gene/?term=215819
Kl	Kl	klotho	16591	https://www.ncbi.nlm.nih.gov/gene/?term=16591
Elavl1	Elavl1	ELAV (embryonic lethal, abnormal vision)-like 1 (Hu antigen R)	15568	https://www.ncbi.nlm.nih.gov/gene/?term=15568
Hnrnpc	Hnrnpc	heterogeneous nuclear ribonucleoprotein C	15381	https://www.ncbi.nlm.nih.gov/gene/?term=15381
Ndufaf5	Ndufaf5	NADH:ubiquinone oxidoreductase complex assembly factor 5	69487	https://www.ncbi.nlm.nih.gov/gene/?term=69487
Scai	Scai	suppressor of cancer cell invasion	320271	https://www.ncbi.nlm.nih.gov/gene/?term=320271
Fam204a	Fam204a	family with sequence similarity 204, member A	76539	https://www.ncbi.nlm.nih.gov/gene/?term=76539
Gcc2	Gcc2	GRIP and coiled-coil domain containing 2	70297	https://www.ncbi.nlm.nih.gov/gene/?term=70297
Etnk1	Etnk1	ethanolamine kinase 1	75320	https://www.ncbi.nlm.nih.gov/gene/?term=75320
Zfhx4	Zfhx4	zinc finger homeodomain 4	80892	https://www.ncbi.nlm.nih.gov/gene/?term=80892
Taf9b	Taf9b	TATA-box binding protein associated factor 9B	407786	https://www.ncbi.nlm.nih.gov/gene/?term=407786
Lrba	Lrba	LPS-responsive beige-like anchor	80877	https://www.ncbi.nlm.nih.gov/gene/?term=80877
Cops7b	Cops7b	COP9 signalosome subunit 7B	26895	https://www.ncbi.nlm.nih.gov/gene/?term=26895

Biology Open • Supplementary information

Dkk4	Dkk4	dickkopf WNT signaling pathway inhibitor 4	234130	https://www.ncbi.nlm.nih.gov/gene/?term=234130
Orc1	Orc1	origin recognition complex, subunit 1	18392	https://www.ncbi.nlm.nih.gov/gene/?term=18392
Nkain3	Nkain3	Na+/K+ transporting ATPase interacting 3	269513	https://www.ncbi.nlm.nih.gov/gene/?term=269513
Epha5	Epha5	Eph receptor A5	13839	https://www.ncbi.nlm.nih.gov/gene/?term=13839
Satb1	Satb1	special AT-rich sequence binding protein 1	20230	https://www.ncbi.nlm.nih.gov/gene/?term=20230
Nucks1	Nucks1	nuclear casein kinase and cyclin-dependent kinase substrate 1	98415	https://www.ncbi.nlm.nih.gov/gene/?term=98415
Gm5128	Gm5128	predicted gene 5128	331529	https://www.ncbi.nlm.nih.gov/gene/?term=331529
Chm	Chm	choroidermia (RAB escort protein 1)	12662	https://www.ncbi.nlm.nih.gov/gene/?term=12662
Cdk12	Cdk12	cyclin-dependent kinase 12	69131	https://www.ncbi.nlm.nih.gov/gene/?term=69131
Rhoq	Rhoq	ras homolog family member Q	104215	https://www.ncbi.nlm.nih.gov/gene/?term=104215
Prkd3	Prkd3	protein kinase D3	75292	https://www.ncbi.nlm.nih.gov/gene/?term=75292
Scel	Scel	sciellin	64929	https://www.ncbi.nlm.nih.gov/gene/?term=64929
Tent5a	Tent5a	terminal nucleotidyltransferase 5A	212943	https://www.ncbi.nlm.nih.gov/gene/?term=212943
Bclaf1	Bclaf1	BCL2-associated transcription factor 1	72567	https://www.ncbi.nlm.nih.gov/gene/?term=72567
Mvb12b	Mvb12b	multivesicular body subunit 12B	72543	https://www.ncbi.nlm.nih.gov/gene/?term=72543
Klk11	Klk11	kallikrein related-peptidase 11	56538	https://www.ncbi.nlm.nih.gov/gene/?term=56538
Gcfc2	Gcfc2	GC-rich sequence DNA binding factor 2	330361	https://www.ncbi.nlm.nih.gov/gene/?term=330361
Bicral	Bicral	BRD4 interacting chromatin remodeling complex associated protein like	210982	https://www.ncbi.nlm.nih.gov/gene/?term=210982

1700066M21Rik	1700066M21Rik	RIKEN cDNA 1700066M21 gene	73467	https://www.ncbi.nlm.nih.gov/gene/?term=73467
Rc3h1	Rc3h1	RING CCCH (C3H) domains 1	381305	https://www.ncbi.nlm.nih.gov/gene/?term=381305
Arhgef12	Arhgef12	Rho guanine nucleotide exchange factor (GEF) 12	69632	https://www.ncbi.nlm.nih.gov/gene/?term=69632
Gm7903	Gm7903	predicted gene 7903	666040	https://www.ncbi.nlm.nih.gov/gene/?term=666040
Prickle1	Prickle1	prickle planar cell polarity protein 1	106042	https://www.ncbi.nlm.nih.gov/gene/?term=106042
Atrx	Atrx	ATRX, chromatin remodeler	22589	https://www.ncbi.nlm.nih.gov/gene/?term=22589
Frmd3	Frmd3	FERM domain containing 3	242506	https://www.ncbi.nlm.nih.gov/gene/?term=242506
Ckap2l	Ckap2l	cytoskeleton associated protein 2-like	70466	https://www.ncbi.nlm.nih.gov/gene/?term=70466
Mgst1	Mgst1	microsomal glutathione S-transferase 1	56615	https://www.ncbi.nlm.nih.gov/gene/?term=56615
Zfp937	Zfp937	zinc finger protein 937	245174	https://www.ncbi.nlm.nih.gov/gene/?term=245174
Synpr	Synpr	synaptoporin	72003	https://www.ncbi.nlm.nih.gov/gene/?term=72003
Ebf1	Ebf1	early B cell factor 1	13591	https://www.ncbi.nlm.nih.gov/gene/?term=13591
AI597479	AI597479	expressed sequence AI597479	98404	https://www.ncbi.nlm.nih.gov/gene/?term=98404
Mcc	Mcc	mutated in colorectal cancers	328949	https://www.ncbi.nlm.nih.gov/gene/?term=328949
Zfp345	Zfp345	zinc finger protein 345	545471	https://www.ncbi.nlm.nih.gov/gene/?term=545471
Aasdhppt	Aasdhppt	aminoadipate-semialdehyde dehydrogenase-phosphopantetheinyl transferase	67618	https://www.ncbi.nlm.nih.gov/gene/?term=67618
Tle1	Tle1	transducin-like enhancer of split 1	21885	https://www.ncbi.nlm.nih.gov/gene/?term=21885
Hes1	Hes1	hes family bHLH transcription factor 1	15205	https://www.ncbi.nlm.nih.gov/gene/?term=15205
Hcfc1	Hcfc1	host cell factor C1	15161	https://www.ncbi.nlm.nih.gov/gene/?term=15161
Txlng	Txlng	taxilin gamma	353170	https://www.ncbi.nlm.nih.gov/gene/?term=353170
Shank2	Shank2	SH3 and multiple ankyrin repeat domains 2	210274	https://www.ncbi.nlm.nih.gov/gene/?term=210274

Biology Open • Supplementary information

Akap9	Akap9	A kinase (PRKA) anchor protein (yotiao) 9	100986	https://www.ncbi.nlm.nih.gov/gene/?term=100986
Dusp10	Dusp10	dual specificity phosphatase 10	63953	https://www.ncbi.nlm.nih.gov/gene/?term=63953
Maea	Maea	macrophage erythroblast attacher	59003	https://www.ncbi.nlm.nih.gov/gene/?term=59003
Rmdn2	Rmdn2	regulator of microtubule dynamics 2	381110	https://www.ncbi.nlm.nih.gov/gene/?term=381110
Ash1l	Ash1l	ASH1 like histone lysine methyltransferase	192195	https://www.ncbi.nlm.nih.gov/gene/?term=192195
Shisa2	Shisa2	shisa family member 2	219134	https://www.ncbi.nlm.nih.gov/gene/?term=219134
E2f8	E2f8	E2F transcription factor 8	108961	https://www.ncbi.nlm.nih.gov/gene/?term=108961
Hmgb2	Hmgb2	high mobility group box 2	97165	https://www.ncbi.nlm.nih.gov/gene/?term=97165
Sost	Sost	sclerostin	74499	https://www.ncbi.nlm.nih.gov/gene/?term=74499
Minpp1	Minpp1	multiple inositol polyphosphate histidine phosphatase 1	17330	https://www.ncbi.nlm.nih.gov/gene/?term=17330
Zbtb6	Zbtb6	zinc finger and BTB domain containing 6	241322	https://www.ncbi.nlm.nih.gov/gene/?term=241322
Rbm47	Rbm47	RNA binding motif protein 47	245945	https://www.ncbi.nlm.nih.gov/gene/?term=245945
Cadm1	Cadm1	cell adhesion molecule 1	54725	https://www.ncbi.nlm.nih.gov/gene/?term=54725
Senp7	Senp7	SUMO1/sentrin specific peptidase 7	66315	https://www.ncbi.nlm.nih.gov/gene/?term=66315
Mdh1b	Mdh1b	malate dehydrogenase 1B, NAD (soluble)	76668	https://www.ncbi.nlm.nih.gov/gene/?term=76668
Nf1	Nf1	neurofibromin 1	18015	https://www.ncbi.nlm.nih.gov/gene/?term=18015
Rcbtb1	Rcbtb1	regulator of chromosome condensation (RCC1) and BTB (POZ) domain containing protein 1	71330	https://www.ncbi.nlm.nih.gov/gene/?term=71330
Mgrn1	Mgrn1	mahogunin, ring finger 1	17237	https://www.ncbi.nlm.nih.gov/gene/?term=17237

Biology Open • Supplementary information

Strbp	Strbp	spermatid perinuclear RNA binding protein	20744	https://www.ncbi.nlm.nih.gov/gene/?term=20744
Sdha	Sdha	succinate dehydrogenase complex, subunit A, flavoprotein (Fp)	66945	https://www.ncbi.nlm.nih.gov/gene/?term=66945
Gm3286	Gm3286	predicted gene 3286	100041354	https://www.ncbi.nlm.nih.gov/gene/?term=100041
Spag6l	Spag6l	sperm associated antigen 6-like	50525	https://www.ncbi.nlm.nih.gov/gene/?term=50525
Selenot	Selenot	selenoprotein T	69227	https://www.ncbi.nlm.nih.gov/gene/?term=69227
Ptprm	Ptprm	protein tyrosine phosphatase, receptor type, M	19274	https://www.ncbi.nlm.nih.gov/gene/?term=19274
Chn1	Chn1	chimerin 1	108699	https://www.ncbi.nlm.nih.gov/gene/?term=108699
Fut8	Fut8	fucosyltransferase 8	53618	https://www.ncbi.nlm.nih.gov/gene/?term=53618
Slc35f5	Slc35f5	solute carrier family 35, member F5	74150	https://www.ncbi.nlm.nih.gov/gene/?term=74150
Lmod3	Lmod3	leiomodin 3 (fetal)	320502	https://www.ncbi.nlm.nih.gov/gene/?term=320502
Cep55	Cep55	centrosomal protein 55	74107	https://www.ncbi.nlm.nih.gov/gene/?term=74107
Invs	Invs	inversin	16348	https://www.ncbi.nlm.nih.gov/gene/?term=16348
Lrch3	Lrch3	leucine-rich repeats and calponin homology (CH) domain containing 3	70144	https://www.ncbi.nlm.nih.gov/gene/?term=70144
Abhd17b	Abhd17b	abhydrolase domain containing 17B	226016	https://www.ncbi.nlm.nih.gov/gene/?term=226016
Cltc	Cltc	clathrin, heavy polypeptide (Hc)	67300	https://www.ncbi.nlm.nih.gov/gene/?term=67300
Ctps2	Ctps2	cytidine 5'-triphosphate synthase 2	55936	https://www.ncbi.nlm.nih.gov/gene/?term=55936
Slc35a1	Slc35a1	solute carrier family 35 (CMP-sialic acid transporter), member 1	24060	https://www.ncbi.nlm.nih.gov/gene/?term=24060
Sh2d4a	Sh2d4a	SH2 domain containing 4A	72281	https://www.ncbi.nlm.nih.gov/gene/?term=72281
Rab11fip1	Rab11fip1	RAB11 family interacting protein 1 (class I)	75767	https://www.ncbi.nlm.nih.gov/gene/?term=75767
Dusp7	Dusp7	dual specificity phosphatase 7	235584	https://www.ncbi.nlm.nih.gov/gene/?term=235584
Sbf2	Sbf2	SET binding factor 2	319934	https://www.ncbi.nlm.nih.gov/gene/?term=319934

Biology Open • Supplementary information

Frk	Frk	fyn-related kinase	14302	https://www.ncbi.nlm.nih.gov/gene/?term=14302
4921517D22Rik	4921517D22Rik	RIKEN cDNA 4921517D22 gene	70900	https://www.ncbi.nlm.nih.gov/gene/?term=70900
E330014E10Rik	E330014E10Rik	RIKEN cDNA E330014E10 gene	665943	https://www.ncbi.nlm.nih.gov/gene/?term=665943
Wnk3	Wnk3	WNK lysine deficient protein kinase 3	279561	https://www.ncbi.nlm.nih.gov/gene/?term=279561
Lrp1	Lrp1	low density lipoprotein receptor-related protein 1	16971	https://www.ncbi.nlm.nih.gov/gene/?term=16971
Zbtb40	Zbtb40	zinc finger and BTB domain containing 40	230848	https://www.ncbi.nlm.nih.gov/gene/?term=230848
Rubcn	Rubcn	RUN domain and cysteine-rich domain containing, Beclin 1-interacting protein	100502698	https://www.ncbi.nlm.nih.gov/gene/?term=100502
Zfand3	Zfand3	zinc finger, AN1-type domain 3	21769	https://www.ncbi.nlm.nih.gov/gene/?term=21769
Fbln5	Fbln5	fibulin 5	23876	https://www.ncbi.nlm.nih.gov/gene/?term=23876
Tspan11	Tspan11	tetraspanin 11	68498	https://www.ncbi.nlm.nih.gov/gene/?term=68498
Pim1	Pim1	proviral integration site 1	18712	https://www.ncbi.nlm.nih.gov/gene/?term=18712
Ammecr1l	Ammecr1l	AMME chromosomal region gene 1-like	225339	https://www.ncbi.nlm.nih.gov/gene/?term=225339
Gas7	Gas7	growth arrest specific 7	14457	https://www.ncbi.nlm.nih.gov/gene/?term=14457
Pdcl2	Pdcl2	phosducin-like 2	79455	https://www.ncbi.nlm.nih.gov/gene/?term=79455
Rexo2	Rexo2	RNA exonuclease 2	104444	https://www.ncbi.nlm.nih.gov/gene/?term=104444
Slc16a3	Slc16a3	solute carrier family 16 (monocarboxylic acid transporters), member 3	80879	https://www.ncbi.nlm.nih.gov/gene/?term=80879
Zfp710	Zfp710	zinc finger protein 710	209225	https://www.ncbi.nlm.nih.gov/gene/?term=209225
Pdrg1	Pdrg1	p53 and DNA damage regulated 1	68559	https://www.ncbi.nlm.nih.gov/gene/?term=68559
Prr14l	Prr14l	proline rich 14-like	215476	https://www.ncbi.nlm.nih.gov/gene/?term=215476

Biology Open • Supplementary information

Mllt10	Mllt10	myeloid/lymphoid or mixed-lineage leukemia; translocated to, 10	17354	https://www.ncbi.nlm.nih.gov/gene/?term=17354
Pcbp2	Pcbp2	poly(rC) binding protein 2	18521	https://www.ncbi.nlm.nih.gov/gene/?term=18521
Sall4	Sall4	spalt like transcription factor 4	99377	https://www.ncbi.nlm.nih.gov/gene/?term=99377
Rcsd1	Rcsd1	RCSD domain containing 1	226594	https://www.ncbi.nlm.nih.gov/gene/?term=226594
Afmid	Afmid	arylformamidase	71562	https://www.ncbi.nlm.nih.gov/gene/?term=71562
Jakmip2	Jakmip2	janus kinase and microtubule interacting protein 2	76217	https://www.ncbi.nlm.nih.gov/gene/?term=76217
0610010K14Rik	0610010K14Rik	RIKEN cDNA 0610010K14 gene	104457	https://www.ncbi.nlm.nih.gov/gene/?term=104457
Sec24c	Sec24c	Sec24 related gene family, member C (S. cerevisiae)	218811	https://www.ncbi.nlm.nih.gov/gene/?term=218811
Rasgef1c	Rasgef1c	RasGEF domain family, member 1C	74563	https://www.ncbi.nlm.nih.gov/gene/?term=74563
Stx8	Stx8	syntaxin 8	55943	https://www.ncbi.nlm.nih.gov/gene/?term=55943
Lsm12	Lsm12	LSM12 homolog	268490	https://www.ncbi.nlm.nih.gov/gene/?term=268490
Nfatc2	Nfatc2	nuclear factor of activated T cells, cytoplasmic, calcineurin dependent 2	18019	https://www.ncbi.nlm.nih.gov/gene/?term=18019
Enox2	Enox2	ecto-NOX disulfide-thiol exchanger 2	209224	https://www.ncbi.nlm.nih.gov/gene/?term=209224
Fchsd1	Fchsd1	FCH and double SH3 domains 1	319262	https://www.ncbi.nlm.nih.gov/gene/?term=319262
Rexo1	Rexo1	REX1, RNA exonuclease 1	66932	https://www.ncbi.nlm.nih.gov/gene/?term=66932
Cbl	Cbl	Casitas B-lineage lymphoma	12402	https://www.ncbi.nlm.nih.gov/gene/?term=12402
Il17rd	Il17rd	interleukin 17 receptor D	171463	https://www.ncbi.nlm.nih.gov/gene/?term=171463
Myoz3	Myoz3	myozenin 3	170947	https://www.ncbi.nlm.nih.gov/gene/?term=170947
Myo18a	Myo18a	myosin XVIIIA	360013	https://www.ncbi.nlm.nih.gov/gene/?term=360013
Cetn2	Cetn2	centrin 2	26370	https://www.ncbi.nlm.nih.gov/gene/?term=26370

Biology Open • Supplementary information

Tab3	Tab3	TGF-beta activated kinase 1/MAP3K7 binding protein 3	66724	https://www.ncbi.nlm.nih.gov/gene/?term=66724
Kif7	Kif7	kinesin family member 7	16576	https://www.ncbi.nlm.nih.gov/gene/?term=16576
1700010I14Rik	1700010I14Rik	RIKEN cDNA 1700010I14 gene	66931	https://www.ncbi.nlm.nih.gov/gene/?term=66931
Exoc3	Exoc3	exocyst complex component 3	211446	https://www.ncbi.nlm.nih.gov/gene/?term=211446
Cpsf4	Cpsf4	cleavage and polyadenylation specific factor 4	54188	https://www.ncbi.nlm.nih.gov/gene/?term=54188
Arl2	Arl2	ADP-ribosylation factor-like 2	56327	https://www.ncbi.nlm.nih.gov/gene/?term=56327
Kbtbd2	Kbtbd2	kelch repeat and BTB (POZ) domain containing 2	210973	https://www.ncbi.nlm.nih.gov/gene/?term=210973
Peli3	Peli3	pellino 3	240518	https://www.ncbi.nlm.nih.gov/gene/?term=240518
Lrrc1	Lrrc1	leucine rich repeat containing 1	214345	https://www.ncbi.nlm.nih.gov/gene/?term=214345
Fgfr1	Fgfr1	fibroblast growth factor receptor 1	14182	https://www.ncbi.nlm.nih.gov/gene/?term=14182
Luzp1	Luzp1	leucine zipper protein 1	269593	https://www.ncbi.nlm.nih.gov/gene/?term=269593
Clint1	Clint1	clathrin interactor 1	216705	https://www.ncbi.nlm.nih.gov/gene/?term=216705
Usp20	Usp20	ubiquitin specific peptidase 20	74270	https://www.ncbi.nlm.nih.gov/gene/?term=74270
Fgf14	Fgf14	fibroblast growth factor 14	14169	https://www.ncbi.nlm.nih.gov/gene/?term=14169
Scp2d1	Scp2d1	SCP2 sterol-binding domain containing 1	66328	https://www.ncbi.nlm.nih.gov/gene/?term=66328
Snx12	Snx12	sorting nexin 12	55988	https://www.ncbi.nlm.nih.gov/gene/?term=55988
Pgf	Pgf	placental growth factor	18654	https://www.ncbi.nlm.nih.gov/gene/?term=18654
B3gnt5	B3gnt5	UDP-GlcNAc:betaGal beta-1,3-N-acetylglucosaminyltransferase 5	108105	https://www.ncbi.nlm.nih.gov/gene/?term=108105
Usp34	Usp34	ubiquitin specific peptidase 34	17847	https://www.ncbi.nlm.nih.gov/gene/?term=17847
Brcc3	Brcc3	BRCA1/BRCA2-containing complex, subunit 3	210766	https://www.ncbi.nlm.nih.gov/gene/?term=210766
Steap2	Steap2	six transmembrane epithelial antigen of prostate 2	74051	https://www.ncbi.nlm.nih.gov/gene/?term=74051

Biology Open • Supplementary information

Smarce1	Smarce1	SWI/SNF related, matrix associated, actin dependent regulator of chromatin, subfamily e, member 1	57376	https://www.ncbi.nlm.nih.gov/gene/?term=57376
Plp1	Plp1	proteolipid protein (myelin) 1	18823	https://www.ncbi.nlm.nih.gov/gene/?term=18823
Rasgef1b	Rasgef1b	RasGEF domain family, member 1B	320292	https://www.ncbi.nlm.nih.gov/gene/?term=320292
Rab27b	Rab27b	RAB27B, member RAS oncogene family	80718	https://www.ncbi.nlm.nih.gov/gene/?term=80718
Klhl34	Klhl34	kelch-like 34	245683	https://www.ncbi.nlm.nih.gov/gene/?term=245683
Snx7	Snx7	sorting nexin 7	76561	https://www.ncbi.nlm.nih.gov/gene/?term=76561
Pkn2	Pkn2	protein kinase N2	109333	https://www.ncbi.nlm.nih.gov/gene/?term=109333
Ccdc88a	Ccdc88a	coiled coil domain containing 88A	108686	https://www.ncbi.nlm.nih.gov/gene/?term=108686
Syt4	Syt4	synaptotagmin IV	20983	https://www.ncbi.nlm.nih.gov/gene/?term=20983
Far2	Far2	fatty acyl CoA reductase 2	330450	https://www.ncbi.nlm.nih.gov/gene/?term=330450
Olfr1033	Olfr1033	olfactory receptor 1033	258571	https://www.ncbi.nlm.nih.gov/gene/?term=258571
Sult3a1	Sult3a1	sulfotransferase family 3A, member 1	57430	https://www.ncbi.nlm.nih.gov/gene/?term=57430
Acbd5	Acbd5	acyl-Coenzyme A binding domain containing 5	74159	https://www.ncbi.nlm.nih.gov/gene/?term=74159
Pitx2	Pitx2	paired-like homeodomain transcription factor 2	18741	https://www.ncbi.nlm.nih.gov/gene/?term=18741
Stxbp5l	Stxbp5l	syntaxin binding protein 5-like	207227	https://www.ncbi.nlm.nih.gov/gene/?term=207227
Cep135	Cep135	centrosomal protein 135	381644	https://www.ncbi.nlm.nih.gov/gene/?term=381644
Nxpe4	Nxpe4	neurexophilin and PC-esterase domain family, member 4	244853	https://www.ncbi.nlm.nih.gov/gene/?term=244853
Neurog1	Neurog1	neurogenin 1	18014	https://www.ncbi.nlm.nih.gov/gene/?term=18014
Ryr2	Ryr2	ryanodine receptor 2, cardiac	20191	https://www.ncbi.nlm.nih.gov/gene/?term=20191
Angptl1	Angptl1	angiopoietin-like 1	72713	https://www.ncbi.nlm.nih.gov/gene/?term=72713

Biology Open • Supplementary information

Etv1	Etv1	ets variant 1	14009	https://www.ncbi.nlm.nih.gov/gene/?term=14009
Pclo	Pclo	piccolo (presynaptic cytomatrix protein)	26875	https://www.ncbi.nlm.nih.gov/gene/?term=26875
Lrrtm3	Lrrtm3	leucine rich repeat transmembrane neuronal 3	216028	https://www.ncbi.nlm.nih.gov/gene/?term=216028
Bcor	Bcor	BCL6 interacting corepressor	71458	https://www.ncbi.nlm.nih.gov/gene/?term=71458
Tmed5	Tmed5	transmembrane p24 trafficking protein 5	73130	https://www.ncbi.nlm.nih.gov/gene/?term=73130
Ank3	Ank3	ankyrin 3, epithelial	11735	https://www.ncbi.nlm.nih.gov/gene/?term=11735
Fgd6	Fgd6	FYVE, RhoGEF and PH domain containing 6	13998	https://www.ncbi.nlm.nih.gov/gene/?term=13998
Runx1t1	Runx1t1	RUNX1 translocation partner 1	12395	https://www.ncbi.nlm.nih.gov/gene/?term=12395
Mageb16	Mageb16	melanoma antigen family B, 16	71967	https://www.ncbi.nlm.nih.gov/gene/?term=71967
Abi1	Abi1	abl-interactor 1	11308	https://www.ncbi.nlm.nih.gov/gene/?term=11308
Ppat	Ppat	phosphoribosyl pyrophosphate amidotransferase	231327	https://www.ncbi.nlm.nih.gov/gene/?term=231327
Jade1	Jade1	jade family PHD finger 1	269424	https://www.ncbi.nlm.nih.gov/gene/?term=269424
Zfp616	Zfp616	zinc finger protein 616	327963	https://www.ncbi.nlm.nih.gov/gene/?term=327963
Enpp2	Enpp2	ectonucleotide pyrophosphatase/phosphodiesterase 2	18606	https://www.ncbi.nlm.nih.gov/gene/?term=18606
Tshz1	Tshz1	teashirt zinc finger family member 1	110796	https://www.ncbi.nlm.nih.gov/gene/?term=110796
Lrp11	Lrp11	low density lipoprotein receptor-related protein 11	237253	https://www.ncbi.nlm.nih.gov/gene/?term=237253
Nt5m	Nt5m	5',3'-nucleotidase, mitochondrial	103850	https://www.ncbi.nlm.nih.gov/gene/?term=103850
Homer1	Homer1	homer scaffolding protein 1	26556	https://www.ncbi.nlm.nih.gov/gene/?term=26556

Biology Open • Supplementary information

Ippk	Ippk	inositol 1,3,4,5,6-pentakisphosphate 2-kinase	75678	https://www.ncbi.nlm.nih.gov/gene/?term=75678
Nars2	Nars2	asparaginyl-tRNA synthetase 2 (mitochondrial)(putative)	244141	https://www.ncbi.nlm.nih.gov/gene/?term=244141
Cntrl	Cntrl	centriolin	26920	https://www.ncbi.nlm.nih.gov/gene/?term=26920
Elac1	Elac1	elaC ribonuclease Z 1	114615	https://www.ncbi.nlm.nih.gov/gene/?term=114615
Lmo7	Lmo7	LIM domain only 7	380928	https://www.ncbi.nlm.nih.gov/gene/?term=380928
Diaph1	Diaph1	diaphanous related formin 1	13367	https://www.ncbi.nlm.nih.gov/gene/?term=13367
Papolg	Papolg	poly(A) polymerase gamma	216578	https://www.ncbi.nlm.nih.gov/gene/?term=216578
Flrt3	Flrt3	fibronectin leucine rich transmembrane protein 3	71436	https://www.ncbi.nlm.nih.gov/gene/?term=71436
Etf1	Etf1	eukaryotic translation termination factor 1	225363	https://www.ncbi.nlm.nih.gov/gene/?term=225363
Ehf	Ehf	ets homologous factor	13661	https://www.ncbi.nlm.nih.gov/gene/?term=13661
Herc6	Herc6	hect domain and RLD 6	67138	https://www.ncbi.nlm.nih.gov/gene/?term=67138
Zfp354a	Zfp354a	zinc finger protein 354A	21408	https://www.ncbi.nlm.nih.gov/gene/?term=21408
Xndc1	Xndc1	Xrcc1 N-terminal domain containing 1	102443350	https://www.ncbi.nlm.nih.gov/gene/?term=102443[illegible]
Pou3f2	Pou3f2	POU domain, class 3, transcription factor 2	18992	https://www.ncbi.nlm.nih.gov/gene/?term=18992
Phf24	Phf24	PHD finger protein 24	230085	https://www.ncbi.nlm.nih.gov/gene/?term=230085
Ifi202b	Ifi202b	interferon activated gene 202B	26388	https://www.ncbi.nlm.nih.gov/gene/?term=26388
Tmem30a	Tmem30a	transmembrane protein 30A	69981	https://www.ncbi.nlm.nih.gov/gene/?term=69981
Abca9	Abca9	ATP-binding cassette, sub-family A (ABC1), member 9	217262	https://www.ncbi.nlm.nih.gov/gene/?term=217262
Dstn	Dstn	destrin	56431	https://www.ncbi.nlm.nih.gov/gene/?term=56431
Crispld2	Crispld2	cysteine-rich secretory protein LCCL domain containing 2	78892	https://www.ncbi.nlm.nih.gov/gene/?term=78892

Biology Open • Supplementary information

Zic3	Zic3	zinc finger protein of the cerebellum 3	22773	https://www.ncbi.nlm.nih.gov/gene/?term=22773
Zfp867	Zfp867	zinc finger protein 867	237775	https://www.ncbi.nlm.nih.gov/gene/?term=237775
Hoxa10	Hoxa10	homeobox A10	15395	https://www.ncbi.nlm.nih.gov/gene/?term=15395
Rnf14	Rnf14	ring finger protein 14	56736	https://www.ncbi.nlm.nih.gov/gene/?term=56736
Toe1	Toe1	target of EGR1, member 1 (nuclear)	68276	https://www.ncbi.nlm.nih.gov/gene/?term=68276
Rap2a	Rap2a	RAS related protein 2a	76108	https://www.ncbi.nlm.nih.gov/gene/?term=76108
Zbtb21	Zbtb21	zinc finger and BTB domain containing 21	114565	https://www.ncbi.nlm.nih.gov/gene/?term=114565
Cntn3	Cntn3	contactin 3	18488	https://www.ncbi.nlm.nih.gov/gene/?term=18488
Gucy1a2	Gucy1a2	guanylate cyclase 1, soluble, alpha 2	234889	https://www.ncbi.nlm.nih.gov/gene/?term=234889
Ube2g2	Ube2g2	ubiquitin-conjugating enzyme E2G 2	22213	https://www.ncbi.nlm.nih.gov/gene/?term=22213
Nipa1	Nipa1	non imprinted in Prader-Willi/Angelman syndrome 1 homolog (human)	233280	https://www.ncbi.nlm.nih.gov/gene/?term=233280
Ufl1	Ufl1	UFM1 specific ligase 1	67490	https://www.ncbi.nlm.nih.gov/gene/?term=67490
Mmp12	Mmp12	matrix metallopeptidase 12	17381	https://www.ncbi.nlm.nih.gov/gene/?term=17381
Pabpc1	Pabpc1	poly(A) binding protein, cytoplasmic 1	18458	https://www.ncbi.nlm.nih.gov/gene/?term=18458
Fgf18	Fgf18	fibroblast growth factor 18	14172	https://www.ncbi.nlm.nih.gov/gene/?term=14172
Megf10	Megf10	multiple EGF-like-domains 10	70417	https://www.ncbi.nlm.nih.gov/gene/?term=70417
Agmo	Agmo	alkylglycerol monooxygenase	319660	https://www.ncbi.nlm.nih.gov/gene/?term=319660
Jcad	Jcad	junctional cadherin 5 associated	240185	https://www.ncbi.nlm.nih.gov/gene/?term=240185
Rap2b	Rap2b	RAP2B, member of RAS oncogene family	74012	https://www.ncbi.nlm.nih.gov/gene/?term=74012
Atf2	Atf2	activating transcription factor 2	11909	https://www.ncbi.nlm.nih.gov/gene/?term=11909

Biology Open • Supplementary information

Cdk13	Cdk13	cyclin-dependent kinase 13	69562	https://www.ncbi.nlm.nih.gov/gene/?term=69562
Dscaml1	Dscaml1	DS cell adhesion molecule like 1	114873	https://www.ncbi.nlm.nih.gov/gene/?term=114873
Irf6	Irf6	interferon regulatory factor 6	54139	https://www.ncbi.nlm.nih.gov/gene/?term=54139
Zfp317	Zfp317	zinc finger protein 317	244713	https://www.ncbi.nlm.nih.gov/gene/?term=244713
Ppp2r2b	Ppp2r2b	protein phosphatase 2, regulatory subunit B, beta	72930	https://www.ncbi.nlm.nih.gov/gene/?term=72930
Tmem106b	Tmem106b	transmembrane protein 106B	71900	https://www.ncbi.nlm.nih.gov/gene/?term=71900
Fbxl4	Fbxl4	F-box and leucine-rich repeat protein 4	269514	https://www.ncbi.nlm.nih.gov/gene/?term=269514
Agps	Agps	alkylglycerone phosphate synthase	228061	https://www.ncbi.nlm.nih.gov/gene/?term=228061
Akap7	Akap7	A kinase (PRKA) anchor protein 7	432442	https://www.ncbi.nlm.nih.gov/gene/?term=432442
Slc17a8	Slc17a8	solute carrier family 17 (sodium-dependent inorganic phosphate cotransporter), member 8	216227	https://www.ncbi.nlm.nih.gov/gene/?term=216227
Ttc21b	Ttc21b	tetratricopeptide repeat domain 21B	73668	https://www.ncbi.nlm.nih.gov/gene/?term=73668
Ube2d2b	Ube2d2b	ubiquitin-conjugating enzyme E2D 2B	73318	https://www.ncbi.nlm.nih.gov/gene/?term=73318
Tmem47	Tmem47	transmembrane protein 47	192216	https://www.ncbi.nlm.nih.gov/gene/?term=192216
Nek1	Nek1	NIMA (never in mitosis gene a)-related expressed kinase 1	18004	https://www.ncbi.nlm.nih.gov/gene/?term=18004
Ncoa5	Ncoa5	nuclear receptor coactivator 5	228869	https://www.ncbi.nlm.nih.gov/gene/?term=228869
Angpt1	Angpt1	angiopoietin 1	11600	https://www.ncbi.nlm.nih.gov/gene/?term=11600
Gpr85	Gpr85	G protein-coupled receptor 85	64450	https://www.ncbi.nlm.nih.gov/gene/?term=64450
Tmeff2	Tmeff2	transmembrane protein with EGF-like and two follistatin-like domains 2	56363	https://www.ncbi.nlm.nih.gov/gene/?term=56363
Msi2	Msi2	musashi RNA-binding protein 2	76626	https://www.ncbi.nlm.nih.gov/gene/?term=76626

Biology Open • Supplementary information

Lifr	Lifr	LIF receptor alpha	16880	https://www.ncbi.nlm.nih.gov/gene/?term=16880
Atp6v1a	Atp6v1a	ATPase, H+ transporting, lysosomal V1 subunit A	11964	https://www.ncbi.nlm.nih.gov/gene/?term=11964
Msr1	Msr1	macrophage scavenger receptor 1	20288	https://www.ncbi.nlm.nih.gov/gene/?term=20288
Ces2c	Ces2c	carboxylesterase 2C	234671	https://www.ncbi.nlm.nih.gov/gene/?term=234671
Samhd1	Samhd1	SAM domain and HD domain, 1	56045	https://www.ncbi.nlm.nih.gov/gene/?term=56045
Grpr	Grpr	gastrin releasing peptide receptor	14829	https://www.ncbi.nlm.nih.gov/gene/?term=14829
Zc3h14	Zc3h14	zinc finger CCCH type containing 14	75553	https://www.ncbi.nlm.nih.gov/gene/?term=75553
Flywch1	Flywch1	FLYWCH-type zinc finger 1	224613	https://www.ncbi.nlm.nih.gov/gene/?term=224613
Nup37	Nup37	nucleoporin 37	69736	https://www.ncbi.nlm.nih.gov/gene/?term=69736
Zfp605	Zfp605	zinc finger protein 605	675812	https://www.ncbi.nlm.nih.gov/gene/?term=675812
Pax8	Pax8	paired box 8	18510	https://www.ncbi.nlm.nih.gov/gene/?term=18510
Gm597	Gm597	predicted gene 597	210962	https://www.ncbi.nlm.nih.gov/gene/?term=210962
Abhd5	Abhd5	abhydrolase domain containing 5	67469	https://www.ncbi.nlm.nih.gov/gene/?term=67469
Brwd1	Brwd1	bromodomain and WD repeat domain containing 1	93871	https://www.ncbi.nlm.nih.gov/gene/?term=93871
Arl15	Arl15	ADP-ribosylation factor-like 15	218639	https://www.ncbi.nlm.nih.gov/gene/?term=218639
Ifi44	Ifi44	interferon-induced protein 44	99899	https://www.ncbi.nlm.nih.gov/gene/?term=99899
Slc9a2	Slc9a2	solute carrier family 9 (sodium/hydrogen exchanger), member 2	226999	https://www.ncbi.nlm.nih.gov/gene/?term=226999
Adtrp	Adtrp	androgen dependent TFPI regulating protein	109254	https://www.ncbi.nlm.nih.gov/gene/?term=109254
Mapt	Mapt	microtubule-associated protein tau	17762	https://www.ncbi.nlm.nih.gov/gene/?term=17762
Evi2	Evi2	ecotropic viral integration site 2	101488212	https://www.ncbi.nlm.nih.gov/gene/?term=101488
Cat	Cat	catalase	12359	https://www.ncbi.nlm.nih.gov/gene/?term=12359
Cd226	Cd226	CD226 antigen	225825	https://www.ncbi.nlm.nih.gov/gene/?term=225825
Dpp7	Dpp7	dipeptidylpeptidase 7	83768	https://www.ncbi.nlm.nih.gov/gene/?term=83768

Biology Open • Supplementary information

Ambra1	Ambra1	autophagy/beclin 1 regulator 1	228361	https://www.ncbi.nlm.nih.gov/gene/?term=228361
Zmynd8	Zmynd8	zinc finger, MYND-type containing 8	228880	https://www.ncbi.nlm.nih.gov/gene/?term=228880
Gm7073	Gm7073	predicted gene 7073	631784	https://www.ncbi.nlm.nih.gov/gene/?term=631784
Lrfn5	Lrfn5	leucine rich repeat and fibronectin type III domain containing 5	238205	https://www.ncbi.nlm.nih.gov/gene/?term=238205
Evi2b	Evi2b	ecotropic viral integration site 2b	216984	https://www.ncbi.nlm.nih.gov/gene/?term=216984
Tln2	Tln2	talin 2	70549	https://www.ncbi.nlm.nih.gov/gene/?term=70549
Anks1	Anks1	ankyrin repeat and SAM domain containing 1	224650	https://www.ncbi.nlm.nih.gov/gene/?term=224650
Smg1	Smg1	SMG1 homolog, phosphatidylinositol 3-kinase-related kinase (C. elegans)	233789	https://www.ncbi.nlm.nih.gov/gene/?term=233789
Gpatch2l	Gpatch2l	G patch domain containing 2 like	70373	https://www.ncbi.nlm.nih.gov/gene/?term=70373
Impdh1	Impdh1	inosine monophosphate dehydrogenase 1	23917	https://www.ncbi.nlm.nih.gov/gene/?term=23917
Hspa9	Hspa9	heat shock protein 9	15526	https://www.ncbi.nlm.nih.gov/gene/?term=15526
Camta1	Camta1	calmodulin binding transcription activator 1	100072	https://www.ncbi.nlm.nih.gov/gene/?term=100072
Kcnq5	Kcnq5	potassium voltage-gated channel, subfamily Q, member 5	226922	https://www.ncbi.nlm.nih.gov/gene/?term=226922
Ppm1l	Ppm1l	protein phosphatase 1 (formerly 2C)-like	242083	https://www.ncbi.nlm.nih.gov/gene/?term=242083
Ezh2	Ezh2	enhancer of zeste 2 polycomb repressive complex 2 subunit	14056	https://www.ncbi.nlm.nih.gov/gene/?term=14056
Slc9a9	Slc9a9	solute carrier family 9 (sodium/hydrogen exchanger), member 9	331004	https://www.ncbi.nlm.nih.gov/gene/?term=331004

Biology Open • Supplementary information

Trim46	Trim46	tripartite motif-containing 46	360213	https://www.ncbi.nlm.nih.gov/gene/?term=360213
Psma1	Psma1	proteasome (prosome, macropain) subunit, alpha type 1	26440	https://www.ncbi.nlm.nih.gov/gene/?term=26440
Zfp790	Zfp790	zinc finger protein 790	233056	https://www.ncbi.nlm.nih.gov/gene/?term=233056
Srsf11	Srsf11	serine/arginine-rich splicing factor 11	69207	https://www.ncbi.nlm.nih.gov/gene/?term=69207
Ptpre	Ptpre	protein tyrosine phosphatase, receptor type, E	19267	https://www.ncbi.nlm.nih.gov/gene/?term=19267
Gabrg2	Gabrg2	gamma-aminobutyric acid (GABA) A receptor, subunit gamma 2	14406	https://www.ncbi.nlm.nih.gov/gene/?term=14406
Smg7	Smg7	Smg-7 homolog, nonsense mediated mRNA decay factor (C. elegans)	226517	https://www.ncbi.nlm.nih.gov/gene/?term=226517
Nbl1	Nbl1	neuroblastoma, suppression of tumorigenicity 1	17965	https://www.ncbi.nlm.nih.gov/gene/?term=17965
Frat1	Frat1	frequently rearranged in advanced T cell lymphomas	14296	https://www.ncbi.nlm.nih.gov/gene/?term=14296
Olfm1	Olfm1	olfactomedin 1	56177	https://www.ncbi.nlm.nih.gov/gene/?term=56177
Tmem178b	Tmem178b	transmembrane protein 178B	434008	https://www.ncbi.nlm.nih.gov/gene/?term=434008
Tpd52l2	Tpd52l2	tumor protein D52-like 2	66314	https://www.ncbi.nlm.nih.gov/gene/?term=66314
Nr2c2	Nr2c2	nuclear receptor subfamily 2, group C, member 2	22026	https://www.ncbi.nlm.nih.gov/gene/?term=22026
Gtpbp2	Gtpbp2	GTP binding protein 2	56055	https://www.ncbi.nlm.nih.gov/gene/?term=56055
Arhgap18	Arhgap18	Rho GTPase activating protein 18	73910	https://www.ncbi.nlm.nih.gov/gene/?term=73910
Cavin1	Cavin1	caveolae associated 1	19285	https://www.ncbi.nlm.nih.gov/gene/?term=19285
Csde1	Csde1	cold shock domain containing E1, RNA binding	229663	https://www.ncbi.nlm.nih.gov/gene/?term=229663

Biology Open • Supplementary information

Spsb1	Spsb1	splA/ryanodine receptor domain and SOCS box containing 1	74646	https://www.ncbi.nlm.nih.gov/gene/?term=74646
Leprotl1	Leprotl1	leptin receptor overlapping transcript-like 1	68192	https://www.ncbi.nlm.nih.gov/gene/?term=68192
Garem1	Garem1	GRB2 associated regulator of MAPK1 subtype 1	381126	https://www.ncbi.nlm.nih.gov/gene/?term=381126
Amph	Amph	amphiphysin	218038	https://www.ncbi.nlm.nih.gov/gene/?term=218038
Gab2	Gab2	growth factor receptor bound protein 2-associated protein 2	14389	https://www.ncbi.nlm.nih.gov/gene/?term=14389
Ndor1	Ndor1	NADPH dependent diflavin oxidoreductase 1	78797	https://www.ncbi.nlm.nih.gov/gene/?term=78797
Pacrg	Pacrg	PARK2 co-regulated	69310	https://www.ncbi.nlm.nih.gov/gene/?term=69310
Natd1	Natd1	N-acetyltransferase domain containing 1	24083	https://www.ncbi.nlm.nih.gov/gene/?term=24083
Slc25a12	Slc25a12	solute carrier family 25 (mitochondrial carrier, Aralar), member 12	78830	https://www.ncbi.nlm.nih.gov/gene/?term=78830
Tmod1	Tmod1	tropomodulin 1	21916	https://www.ncbi.nlm.nih.gov/gene/?term=21916
Macrod2	Macrod2	MACRO domain containing 2	72899	https://www.ncbi.nlm.nih.gov/gene/?term=72899
Ube4b	Ube4b	ubiquitination factor E4B	63958	https://www.ncbi.nlm.nih.gov/gene/?term=63958
Mex3c	Mex3c	mex3 RNA binding family member C	240396	https://www.ncbi.nlm.nih.gov/gene/?term=240396
Usp2	Usp2	ubiquitin specific peptidase 2	53376	https://www.ncbi.nlm.nih.gov/gene/?term=53376
Hoxd1	Hoxd1	homeobox D1	15429	https://www.ncbi.nlm.nih.gov/gene/?term=15429
Ccne1	Ccne1	cyclin E1	12447	https://www.ncbi.nlm.nih.gov/gene/?term=12447
Kif1c	Kif1c	kinesin family member 1C	16562	https://www.ncbi.nlm.nih.gov/gene/?term=16562
Pth	Pth	parathyroid hormone	19226	https://www.ncbi.nlm.nih.gov/gene/?term=19226
Ccnd2	Ccnd2	cyclin D2	12444	https://www.ncbi.nlm.nih.gov/gene/?term=12444

Biology Open • Supplementary information

Pip4p2	Pip4p2	phosphatidylinositol-4,5-bisphosphate 4-phosphatase 2	72519	https://www.ncbi.nlm.nih.gov/gene/?term=72519
Kcnj2	Kcnj2	potassium inwardly-rectifying channel, subfamily J, member 2	16518	https://www.ncbi.nlm.nih.gov/gene/?term=16518
Fam122a	Fam122a	family with sequence similarity 122, member A	68034	https://www.ncbi.nlm.nih.gov/gene/?term=68034
Ccnd1	Ccnd1	cyclin D1	12443	https://www.ncbi.nlm.nih.gov/gene/?term=12443
Akt3	Akt3	thymoma viral proto-oncogene 3	23797	https://www.ncbi.nlm.nih.gov/gene/?term=23797
Ilf2	Ilf2	interleukin enhancer binding factor 2	67781	https://www.ncbi.nlm.nih.gov/gene/?term=67781
Fmr1	Fmr1	fragile X mental retardation 1	14265	https://www.ncbi.nlm.nih.gov/gene/?term=14265
Thoc1	Thoc1	THO complex 1	225160	https://www.ncbi.nlm.nih.gov/gene/?term=225160
Rbm11	Rbm11	RNA binding motif protein 11	224344	https://www.ncbi.nlm.nih.gov/gene/?term=224344
Rcbtb2	Rcbtb2	regulator of chromosome condensation (RCC1) and BTB (POZ) domain containing protein 2	105670	https://www.ncbi.nlm.nih.gov/gene/?term=105670
Nfyb	Nfyb	nuclear transcription factor-Y beta	18045	https://www.ncbi.nlm.nih.gov/gene/?term=18045
Hipk1	Hipk1	homeodomain interacting protein kinase 1	15257	https://www.ncbi.nlm.nih.gov/gene/?term=15257
Sparcl1	Sparcl1	SPARC-like 1	13602	https://www.ncbi.nlm.nih.gov/gene/?term=13602
Vapb	Vapb	vesicle-associated membrane protein, associated protein B and C	56491	https://www.ncbi.nlm.nih.gov/gene/?term=56491
Hectd2	Hectd2	HECT domain E3 ubiquitin protein ligase 2	226098	https://www.ncbi.nlm.nih.gov/gene/?term=226098
Etv3	Etv3	ets variant 3	27049	https://www.ncbi.nlm.nih.gov/gene/?term=27049
Ptbp3	Ptbp3	polypyrimidine tract binding protein 3	230257	https://www.ncbi.nlm.nih.gov/gene/?term=230257

Biology Open • Supplementary information

Mllt6	Mllt6	myeloid/lymphoid or mixed-lineage leukemia; translocated to, 6	246198	https://www.ncbi.nlm.nih.gov/gene/?term=246198
Kit	Kit	KIT proto-oncogene receptor tyrosine kinase	16590	https://www.ncbi.nlm.nih.gov/gene/?term=16590
Dcaf12	Dcaf12	DDB1 and CUL4 associated factor 12	68970	https://www.ncbi.nlm.nih.gov/gene/?term=68970
Tmcc1	Tmcc1	transmembrane and coiled coil domains 1	330401	https://www.ncbi.nlm.nih.gov/gene/?term=330401
Adar	Adar	adenosine deaminase, RNA-specific	56417	https://www.ncbi.nlm.nih.gov/gene/?term=56417
Irx5	Irx5	Iroquois homeobox 5	54352	https://www.ncbi.nlm.nih.gov/gene/?term=54352
Gabra1	Gabra1	gamma-aminobutyric acid (GABA) A receptor, subunit alpha 1	14394	https://www.ncbi.nlm.nih.gov/gene/?term=14394
Bend4	Bend4	BEN domain containing 4	666938	https://www.ncbi.nlm.nih.gov/gene/?term=666938
2510009E07Rik	2510009E07Rik	RIKEN cDNA 2510009E07 gene	72190	https://www.ncbi.nlm.nih.gov/gene/?term=72190
Dmrt3	Dmrt3	doublesex and mab-3 related transcription factor 3	240590	https://www.ncbi.nlm.nih.gov/gene/?term=240590
Sybu	Sybu	syntabulin (syntaxin-interacting)	319613	https://www.ncbi.nlm.nih.gov/gene/?term=319613
Smarca5	Smarca5	SWI/SNF related, matrix associated, actin dependent regulator of chromatin, subfamily a, member 5	93762	https://www.ncbi.nlm.nih.gov/gene/?term=93762
Midn	Midn	midnolin	59090	https://www.ncbi.nlm.nih.gov/gene/?term=59090
Cacna1c	Cacna1c	calcium channel, voltage-dependent, L type, alpha 1C subunit	12288	https://www.ncbi.nlm.nih.gov/gene/?term=12288
Jag2	Jag2	jagged 2	16450	https://www.ncbi.nlm.nih.gov/gene/?term=16450
Gucy2f	Gucy2f	guanylate cyclase 2f	245650	https://www.ncbi.nlm.nih.gov/gene/?term=245650

Biology Open • Supplementary information

Stx12	Stx12	syntaxin 12	100226	https://www.ncbi.nlm.nih.gov/gene/?term=100226
Myct1	Myct1	myc target 1	68632	https://www.ncbi.nlm.nih.gov/gene/?term=68632
Rpl22	Rpl22	ribosomal protein L22	19934	https://www.ncbi.nlm.nih.gov/gene/?term=19934
Lrif1	Lrif1	ligand dependent nuclear receptor interacting factor 1	321000	https://www.ncbi.nlm.nih.gov/gene/?term=321000
Nmral1	Nmral1	NmrA-like family domain containing 1	67824	https://www.ncbi.nlm.nih.gov/gene/?term=67824
Grhl3	Grhl3	grainyhead like transcription factor 3	230824	https://www.ncbi.nlm.nih.gov/gene/?term=230824
Gtf2i	Gtf2i	general transcription factor II I	14886	https://www.ncbi.nlm.nih.gov/gene/?term=14886
Ppp1r14c	Ppp1r14c	protein phosphatase 1, regulatory inhibitor subunit 14C	76142	https://www.ncbi.nlm.nih.gov/gene/?term=76142
Id1	Id1	inhibitor of DNA binding 1	15901	https://www.ncbi.nlm.nih.gov/gene/?term=15901
Tmem39a	Tmem39a	transmembrane protein 39a	67846	https://www.ncbi.nlm.nih.gov/gene/?term=67846
Actr3	Actr3	ARP3 actin-related protein 3	74117	https://www.ncbi.nlm.nih.gov/gene/?term=74117
Vcpip1	Vcpip1	valosin containing protein (p97)/p47 complex interacting protein 1	70675	https://www.ncbi.nlm.nih.gov/gene/?term=70675
Atp6v1h	Atp6v1h	ATPase, H+ transporting, lysosomal V1 subunit H	108664	https://www.ncbi.nlm.nih.gov/gene/?term=108664
Kalrn	Kalrn	kalirin, RhoGEF kinase	545156	https://www.ncbi.nlm.nih.gov/gene/?term=545156
Prpf38b	Prpf38b	PRP38 pre-mRNA processing factor 38 (yeast) domain containing B	66921	https://www.ncbi.nlm.nih.gov/gene/?term=66921
Atad5	Atad5	ATPase family, AAA domain containing 5	237877	https://www.ncbi.nlm.nih.gov/gene/?term=237877
Zswim4	Zswim4	zinc finger SWIM-type containing 4	212168	https://www.ncbi.nlm.nih.gov/gene/?term=212168
Adgrg2	Adgrg2	adhesion G protein-coupled receptor G2	237175	https://www.ncbi.nlm.nih.gov/gene/?term=237175

Biology Open • Supplementary information

Spock3	Spock3	sparc/osteonectin, cwcv and kazal-like domains proteoglycan 3	72902	https://www.ncbi.nlm.nih.gov/gene/?term=72902
Trappc2	Trappc2	trafficking protein particle complex 2	66226	https://www.ncbi.nlm.nih.gov/gene/?term=66226
Dcc	Dcc	deleted in colorectal carcinoma	13176	https://www.ncbi.nlm.nih.gov/gene/?term=13176
Yy1	Yy1	YY1 transcription factor	22632	https://www.ncbi.nlm.nih.gov/gene/?term=22632
Psd3	Psd3	pleckstrin and Sec7 domain containing 3	234353	https://www.ncbi.nlm.nih.gov/gene/?term=234353
Crk	Crk	v-crk avian sarcoma virus CT10 oncogene homolog	12928	https://www.ncbi.nlm.nih.gov/gene/?term=12928
Srsf1	Srsf1	serine/arginine-rich splicing factor 1	110809	https://www.ncbi.nlm.nih.gov/gene/?term=110809
Fgfr2	Fgfr2	fibroblast growth factor receptor 2	14183	https://www.ncbi.nlm.nih.gov/gene/?term=14183
Ncoa2	Ncoa2	nuclear receptor coactivator 2	17978	https://www.ncbi.nlm.nih.gov/gene/?term=17978
Cbln4	Cbln4	cerebellin 4 precursor protein	228942	https://www.ncbi.nlm.nih.gov/gene/?term=228942
Ppp4r3b	Ppp4r3b	protein phosphatase 4 regulatory subunit 3B	104570	https://www.ncbi.nlm.nih.gov/gene/?term=104570
Nfkbia	Nfkbia	nuclear factor of kappa light polypeptide gene enhancer in B cells inhibitor, alpha	18035	https://www.ncbi.nlm.nih.gov/gene/?term=18035
Med14	Med14	mediator complex subunit 14	26896	https://www.ncbi.nlm.nih.gov/gene/?term=26896
Kif20b	Kif20b	kinesin family member 20B	240641	https://www.ncbi.nlm.nih.gov/gene/?term=240641
Casp8ap2	Casp8ap2	caspase 8 associated protein 2	26885	https://www.ncbi.nlm.nih.gov/gene/?term=26885
Lrrc4	Lrrc4	leucine rich repeat containing 4	192198	https://www.ncbi.nlm.nih.gov/gene/?term=192198
Cdk1	Cdk1	cyclin-dependent kinase 1	12534	https://www.ncbi.nlm.nih.gov/gene/?term=12534
Fbxo43	Fbxo43	F-box protein 43	78803	https://www.ncbi.nlm.nih.gov/gene/?term=78803
Mblac2	Mblac2	metallo-beta-lactamase domain containing 2	72852	https://www.ncbi.nlm.nih.gov/gene/?term=72852

Biology Open • Supplementary information

Rabggtb	Rabggtb	Rab geranylgeranyl transferase, b subunit	19352	https://www.ncbi.nlm.nih.gov/gene/?term=19352
Cwc22	Cwc22	CWC22 spliceosome-associated protein	80744	https://www.ncbi.nlm.nih.gov/gene/?term=80744
Arpp21	Arpp21	cyclic AMP-regulated phosphoprotein, 21	74100	https://www.ncbi.nlm.nih.gov/gene/?term=74100
Gpc6	Gpc6	glypican 6	23888	https://www.ncbi.nlm.nih.gov/gene/?term=23888
Prr12	Prr12	proline rich 12	233210	https://www.ncbi.nlm.nih.gov/gene/?term=233210
P2ry10b	P2ry10b	purinergic receptor P2Y, G-protein coupled 10B	213438	https://www.ncbi.nlm.nih.gov/gene/?term=213438
Wac	Wac	WW domain containing adaptor with coiled-coil	225131	https://www.ncbi.nlm.nih.gov/gene/?term=225131
Lgr4	Lgr4	leucine-rich repeat-containing G protein-coupled receptor 4	107515	https://www.ncbi.nlm.nih.gov/gene/?term=107515
Nktr	Nktr	natural killer tumor recognition sequence	18087	https://www.ncbi.nlm.nih.gov/gene/?term=18087
Tusc3	Tusc3	tumor suppressor candidate 3	80286	https://www.ncbi.nlm.nih.gov/gene/?term=80286
Gramd4	Gramd4	GRAM domain containing 4	223752	https://www.ncbi.nlm.nih.gov/gene/?term=223752
Foxd4	Foxd4	forkhead box D4	14237	https://www.ncbi.nlm.nih.gov/gene/?term=14237
Rnpc3	Rnpc3	RNA-binding region (RNP1, RRM) containing 3	67225	https://www.ncbi.nlm.nih.gov/gene/?term=67225
Ubr1	Ubr1	ubiquitin protein ligase E3 component n-recognin 1	22222	https://www.ncbi.nlm.nih.gov/gene/?term=22222
Pdap1	Pdap1	PDGFA associated protein 1	231887	https://www.ncbi.nlm.nih.gov/gene/?term=231887
Zfp715	Zfp715	zinc finger protein 715	69930	https://www.ncbi.nlm.nih.gov/gene/?term=69930
Ubn1	Ubn1	ubinuclein 1	170644	https://www.ncbi.nlm.nih.gov/gene/?term=170644
Kif11	Kif11	kinesin family member 11	16551	https://www.ncbi.nlm.nih.gov/gene/?term=16551

Biology Open • Supplementary information

Lrp6	Lrp6	low density lipoprotein receptor-related protein 6	16974	https://www.ncbi.nlm.nih.gov/gene/?term=16974
Ppp1r15b	Ppp1r15b	protein phosphatase 1, regulatory subunit 15B	108954	https://www.ncbi.nlm.nih.gov/gene/?term=108954
Insm2	Insm2	insulinoma-associated 2	56856	https://www.ncbi.nlm.nih.gov/gene/?term=56856
Abca8a	Abca8a	ATP-binding cassette, sub-family A (ABC1), member 8a	217258	https://www.ncbi.nlm.nih.gov/gene/?term=217258
Emp2	Emp2	epithelial membrane protein 2	13731	https://www.ncbi.nlm.nih.gov/gene/?term=13731
Npy1r	Npy1r	neuropeptide Y receptor Y1	18166	https://www.ncbi.nlm.nih.gov/gene/?term=18166
Eif4g2	Eif4g2	eukaryotic translation initiation factor 4, gamma 2	13690	https://www.ncbi.nlm.nih.gov/gene/?term=13690
Auh	Auh	AU RNA binding protein/enoyl-coenzyme A hydratase	11992	https://www.ncbi.nlm.nih.gov/gene/?term=11992
Pde2a	Pde2a	phosphodiesterase 2A, cGMP-stimulated	207728	https://www.ncbi.nlm.nih.gov/gene/?term=207728
B020004C17Rik	B020004C17Rik	RIKEN cDNA B020004C17 gene	432860	https://www.ncbi.nlm.nih.gov/gene/?term=432860
Spata4	Spata4	spermatogenesis associated 4	69281	https://www.ncbi.nlm.nih.gov/gene/?term=69281
Yrdc	Yrdc	yrdC domain containing (E.coli)	230734	https://www.ncbi.nlm.nih.gov/gene/?term=230734
Rfng	Rfng	RFNG O-fucosylpeptide 3-beta-N-acetylglucosaminyltransferase	19719	https://www.ncbi.nlm.nih.gov/gene/?term=19719
Gpatch2	Gpatch2	G patch domain containing 2	67769	https://www.ncbi.nlm.nih.gov/gene/?term=67769
Lingo2	Lingo2	leucine rich repeat and Ig domain containing 2	242384	https://www.ncbi.nlm.nih.gov/gene/?term=242384
Micu2	Micu2	mitochondrial calcium uptake 2	68514	https://www.ncbi.nlm.nih.gov/gene/?term=68514
Tmem252	Tmem252	transmembrane protein 252	226040	https://www.ncbi.nlm.nih.gov/gene/?term=226040
Lrrc56	Lrrc56	leucine rich repeat containing 56	70552	https://www.ncbi.nlm.nih.gov/gene/?term=70552
Trmt112	Trmt112	tRNA methyltransferase 11-2	67674	https://www.ncbi.nlm.nih.gov/gene/?term=67674

Biology Open • Supplementary information

Eya1	Eya1	EYA transcriptional coactivator and phosphatase 1	14048	https://www.ncbi.nlm.nih.gov/gene/?term=14048
Insig1	Insig1	insulin induced gene 1	231070	https://www.ncbi.nlm.nih.gov/gene/?term=231070
Eif4enif1	Eif4enif1	eukaryotic translation initiation factor 4E nuclear import factor 1	74203	https://www.ncbi.nlm.nih.gov/gene/?term=74203
Hdac8	Hdac8	histone deacetylase 8	70315	https://www.ncbi.nlm.nih.gov/gene/?term=70315
Tsg101	Tsg101	tumor susceptibility gene 101	22088	https://www.ncbi.nlm.nih.gov/gene/?term=22088
Plxdc2	Plxdc2	plexin domain containing 2	67448	https://www.ncbi.nlm.nih.gov/gene/?term=67448
Samd12	Samd12	sterile alpha motif domain containing 12	320679	https://www.ncbi.nlm.nih.gov/gene/?term=320679
Ccdc50	Ccdc50	coiled-coil domain containing 50	67501	https://www.ncbi.nlm.nih.gov/gene/?term=67501
Armcx3	Armcx3	armadillo repeat containing, X-linked 3	71703	https://www.ncbi.nlm.nih.gov/gene/?term=71703
Grik2	Grik2	glutamate receptor, ionotropic, kainate 2 (beta 2)	14806	https://www.ncbi.nlm.nih.gov/gene/?term=14806
Nek7	Nek7	NIMA (never in mitosis gene a)-related expressed kinase 7	59125	https://www.ncbi.nlm.nih.gov/gene/?term=59125
Cdc40	Cdc40	cell division cycle 40	71713	https://www.ncbi.nlm.nih.gov/gene/?term=71713
Kmt2c	Kmt2c	lysine (K)-specific methyltransferase 2C	231051	https://www.ncbi.nlm.nih.gov/gene/?term=231051
Gigyf1	Gigyf1	GRB10 interacting GYF protein 1	57330	https://www.ncbi.nlm.nih.gov/gene/?term=57330
Slc4a10	Slc4a10	solute carrier family 4, sodium bicarbonate cotransporter-like, member 10	94229	https://www.ncbi.nlm.nih.gov/gene/?term=94229
Zfp97	Zfp97	zinc finger protein 97	22759	https://www.ncbi.nlm.nih.gov/gene/?term=22759
Tnfaip1	Tnfaip1	tumor necrosis factor, alpha-induced protein 1 (endothelial)	21927	https://www.ncbi.nlm.nih.gov/gene/?term=21927
Spire1	Spire1	spire type actin nucleation factor 1	68166	https://www.ncbi.nlm.nih.gov/gene/?term=68166

Biology Open • Supplementary information

Cdyl	Cdyl	chromodomain protein, Y chromosome-like	12593	https://www.ncbi.nlm.nih.gov/gene/?term=12593
Carf	Carf	calcium response factor	241066	https://www.ncbi.nlm.nih.gov/gene/?term=241066
Sfmbt1	Sfmbt1	Scm-like with four mbt domains 1	54650	https://www.ncbi.nlm.nih.gov/gene/?term=54650
Ddx55	Ddx55	DEAD (Asp-Glu-Ala-Asp) box polypeptide 55	67848	https://www.ncbi.nlm.nih.gov/gene/?term=67848
Gse1	Gse1	genetic suppressor element 1, coiled-coil protein	382034	https://www.ncbi.nlm.nih.gov/gene/?term=382034
Gpd1l	Gpd1l	glycerol-3-phosphate dehydrogenase 1-like	333433	https://www.ncbi.nlm.nih.gov/gene/?term=333433
Ercc5	Ercc5	excision repair cross-complementing rodent repair deficiency, complementation group 5	22592	https://www.ncbi.nlm.nih.gov/gene/?term=22592
Usp33	Usp33	ubiquitin specific peptidase 33	170822	https://www.ncbi.nlm.nih.gov/gene/?term=170822
Gm14322	Gm14322	predicted gene 14322	626802	https://www.ncbi.nlm.nih.gov/gene/?term=626802
Ap1g1	Ap1g1	adaptor protein complex AP-1, gamma 1 subunit	11765	https://www.ncbi.nlm.nih.gov/gene/?term=11765
Trim2	Trim2	tripartite motif-containing 2	80890	https://www.ncbi.nlm.nih.gov/gene/?term=80890
Mtx3	Mtx3	metaxin 3	382793	https://www.ncbi.nlm.nih.gov/gene/?term=382793
Ccdc122	Ccdc122	coiled-coil domain containing 122	108811	https://www.ncbi.nlm.nih.gov/gene/?term=108811
Anapc16	Anapc16	anaphase promoting complex subunit 16	52717	https://www.ncbi.nlm.nih.gov/gene/?term=52717
Bend3	Bend3	BEN domain containing 3	331623	https://www.ncbi.nlm.nih.gov/gene/?term=331623
Adam11	Adam11	a disintegrin and metallopeptidase domain 11	11488	https://www.ncbi.nlm.nih.gov/gene/?term=11488
Lmo1	Lmo1	LIM domain only 1	109594	https://www.ncbi.nlm.nih.gov/gene/?term=109594
Zfp976	Zfp976	zinc finger protein 976	208111	https://www.ncbi.nlm.nih.gov/gene/?term=208111

Biology Open • Supplementary information

Tada2b	Tada2b	transcriptional adaptor 2B	231151	https://www.ncbi.nlm.nih.gov/gene/?term=231151
Rorb	Rorb	RAR-related orphan receptor beta	225998	https://www.ncbi.nlm.nih.gov/gene/?term=225998
Acvr2b	Acvr2b	activin receptor IIB	11481	https://www.ncbi.nlm.nih.gov/gene/?term=11481
Patl1	Patl1	protein associated with topoisomerase II homolog 1 (yeast)	225929	https://www.ncbi.nlm.nih.gov/gene/?term=225929
Txndc12	Txndc12	thioredoxin domain containing 12 (endoplasmic reticulum)	66073	https://www.ncbi.nlm.nih.gov/gene/?term=66073
Palb2	Palb2	partner and localizer of BRCA2	233826	https://www.ncbi.nlm.nih.gov/gene/?term=233826
Rnf34	Rnf34	ring finger protein 34	80751	https://www.ncbi.nlm.nih.gov/gene/?term=80751
6720489N17Rik	6720489N17Rik	RIKEN cDNA 6720489N17 gene	211378	https://www.ncbi.nlm.nih.gov/gene/?term=211378
Ubp1	Ubp1	upstream binding protein 1	22221	https://www.ncbi.nlm.nih.gov/gene/?term=22221
Lox	Lox	lysyl oxidase	16948	https://www.ncbi.nlm.nih.gov/gene/?term=16948
Tmem94	Tmem94	transmembrane protein 94	71947	https://www.ncbi.nlm.nih.gov/gene/?term=71947
Slc25a37	Slc25a37	solute carrier family 25, member 37	67712	https://www.ncbi.nlm.nih.gov/gene/?term=67712
Zdhhc7	Zdhhc7	zinc finger, DHHC domain containing 7	102193	https://www.ncbi.nlm.nih.gov/gene/?term=102193
Clip1	Clip1	CAP-GLY domain containing linker protein 1	56430	https://www.ncbi.nlm.nih.gov/gene/?term=56430
Sema4g	Sema4g	sema domain, immunoglobulin domain (Ig), transmembrane domain (TM) and short cytoplasmic domain, (semaphorin) 4G	26456	https://www.ncbi.nlm.nih.gov/gene/?term=26456
Gm6712	Gm6712	predicted gene 6712	100862203	https://www.ncbi.nlm.nih.gov/gene/?term=100862
Klhl29	Klhl29	kelch-like 29	208439	https://www.ncbi.nlm.nih.gov/gene/?term=208439
Rassf1	Rassf1	Ras association (RalGDS/AF-6) domain family member 1	56289	https://www.ncbi.nlm.nih.gov/gene/?term=56289
Mb21d2	Mb21d2	Mab-21 domain containing 2	239796	https://www.ncbi.nlm.nih.gov/gene/?term=239796

Biology Open • Supplementary information

Rps6ka3	Rps6ka3	ribosomal protein S6 kinase polypeptide 3	110651	https://www.ncbi.nlm.nih.gov/gene/?term=110651
Ss18l1	Ss18l1	SS18, nBAF chromatin remodeling complex subunit like 1	269397	https://www.ncbi.nlm.nih.gov/gene/?term=269397
Zfp280d	Zfp280d	zinc finger protein 280D	235469	https://www.ncbi.nlm.nih.gov/gene/?term=235469
Fam122b	Fam122b	family with sequence similarity 122, member B	78755	https://www.ncbi.nlm.nih.gov/gene/?term=78755
Heca	Heca	hdc homolog, cell cycle regulator	380629	https://www.ncbi.nlm.nih.gov/gene/?term=380629
2210418O10Rik	2210418O10Rik	RIKEN cDNA 2210418O10 gene	100504263	https://www.ncbi.nlm.nih.gov/gene/?term=100504[illegible]
Ccp110	Ccp110	centriolar coiled coil protein 110	101565	https://www.ncbi.nlm.nih.gov/gene/?term=101565
Zfp619	Zfp619	zinc finger protein 619	70227	https://www.ncbi.nlm.nih.gov/gene/?term=70227
Zfp951	Zfp951	zinc finger protein 951	626391	https://www.ncbi.nlm.nih.gov/gene/?term=626391
Klhl5	Klhl5	kelch-like 5	71778	https://www.ncbi.nlm.nih.gov/gene/?term=71778
Clec10a	Clec10a	C-type lectin domain family 10, member A	17312	https://www.ncbi.nlm.nih.gov/gene/?term=17312
Cyp2c39	Cyp2c39	cytochrome P450, family 2, subfamily c, polypeptide 39	13098	https://www.ncbi.nlm.nih.gov/gene/?term=13098
Entpd6	Entpd6	ectonucleoside triphosphate diphosphohydrolase 6	12497	https://www.ncbi.nlm.nih.gov/gene/?term=12497
Ppip5k2	Ppip5k2	diphosphoinositol pentakisphosphate kinase 2	227399	https://www.ncbi.nlm.nih.gov/gene/?term=227399
Syne1	Syne1	spectrin repeat containing, nuclear envelope 1	64009	https://www.ncbi.nlm.nih.gov/gene/?term=64009
Zfp973	Zfp973	zinc finger protein 973	545490	https://www.ncbi.nlm.nih.gov/gene/?term=545490
P2ry10	P2ry10	purinergic receptor P2Y, G-protein coupled 10	78826	https://www.ncbi.nlm.nih.gov/gene/?term=78826

Biology Open • Supplementary information

Specc1l	Specc1l	sperm antigen with calponin homology and coiled-coil domains 1-like	74392	https://www.ncbi.nlm.nih.gov/gene/?term=74392
Spry4	Spry4	sprouty RTK signaling antagonist 4	24066	https://www.ncbi.nlm.nih.gov/gene/?term=24066
Fam3c	Fam3c	family with sequence similarity 3, member C	27999	https://www.ncbi.nlm.nih.gov/gene/?term=27999
Zfp780b	Zfp780b	zinc finger protein 780B	338354	https://www.ncbi.nlm.nih.gov/gene/?term=338354
Mpp5	Mpp5	membrane protein, palmitoylated 5 (MAGUK p55 subfamily member 5)	56217	https://www.ncbi.nlm.nih.gov/gene/?term=56217
Il1a	Il1a	interleukin 1 alpha	16175	https://www.ncbi.nlm.nih.gov/gene/?term=16175
Hoxa11	Hoxa11	homeobox A11	15396	https://www.ncbi.nlm.nih.gov/gene/?term=15396
Klf15	Klf15	Kruppel-like factor 15	66277	https://www.ncbi.nlm.nih.gov/gene/?term=66277
Zfp14	Zfp14	zinc finger protein 14	243906	https://www.ncbi.nlm.nih.gov/gene/?term=243906
Tspan13	Tspan13	tetraspanin 13	66109	https://www.ncbi.nlm.nih.gov/gene/?term=66109
Ssx2ip	Ssx2ip	synovial sarcoma, X 2 interacting protein	99167	https://www.ncbi.nlm.nih.gov/gene/?term=99167
Zfp781	Zfp781	zinc finger protein 781	331188	https://www.ncbi.nlm.nih.gov/gene/?term=331188
Cpne2	Cpne2	copine II	234577	https://www.ncbi.nlm.nih.gov/gene/?term=234577
Ubl3	Ubl3	ubiquitin-like 3	24109	https://www.ncbi.nlm.nih.gov/gene/?term=24109
Rbbp7	Rbbp7	retinoblastoma binding protein 7, chromatin remodeling factor	245688	https://www.ncbi.nlm.nih.gov/gene/?term=245688
Tmem165	Tmem165	transmembrane protein 165	21982	https://www.ncbi.nlm.nih.gov/gene/?term=21982
Baz2b	Baz2b	bromodomain adjacent to zinc finger domain, 2B	407823	https://www.ncbi.nlm.nih.gov/gene/?term=407823
Zic2	Zic2	zinc finger protein of the cerebellum 2	22772	https://www.ncbi.nlm.nih.gov/gene/?term=22772
Mapk1	Mapk1	mitogen-activated protein kinase 1	26413	https://www.ncbi.nlm.nih.gov/gene/?term=26413

Biology Open • Supplementary information

Zfp970	Zfp970	zinc finger protein 970	628308	https://www.ncbi.nlm.nih.gov/gene/?term=628308
Prox1	Prox1	prospero homeobox 1	19130	https://www.ncbi.nlm.nih.gov/gene/?term=19130
Zfp966	Zfp966	zinc finger protein 966	667962	https://www.ncbi.nlm.nih.gov/gene/?term=667962
Ttc39b	Ttc39b	tetratricopeptide repeat domain 39B	69863	https://www.ncbi.nlm.nih.gov/gene/?term=69863
B4galt1	B4galt1	UDP-Gal:betaGlcNAc beta 1,4-galactosyltransferase, polypeptide 1	14595	https://www.ncbi.nlm.nih.gov/gene/?term=14595
Zfp936	Zfp936	zinc finger protein 936	668620	https://www.ncbi.nlm.nih.gov/gene/?term=668620
Ap1s3	Ap1s3	adaptor-related protein complex AP-1, sigma 3	252903	https://www.ncbi.nlm.nih.gov/gene/?term=252903
Plcl2	Plcl2	phospholipase C-like 2	224860	https://www.ncbi.nlm.nih.gov/gene/?term=224860
Zfp960	Zfp960	zinc finger protein 960	449000	https://www.ncbi.nlm.nih.gov/gene/?term=449000
Dmxl2	Dmxl2	Dmx-like 2	235380	https://www.ncbi.nlm.nih.gov/gene/?term=235380
Osbpl3	Osbpl3	oxysterol binding protein-like 3	71720	https://www.ncbi.nlm.nih.gov/gene/?term=71720
Zfp971	Zfp971	zinc finger protein 971	626848	https://www.ncbi.nlm.nih.gov/gene/?term=626848
Ythdc2	Ythdc2	YTH domain containing 2	240255	https://www.ncbi.nlm.nih.gov/gene/?term=240255
Sim1	Sim1	single-minded family bHLH transcription factor 1	20464	https://www.ncbi.nlm.nih.gov/gene/?term=20464
Carm1	Carm1	coactivator-associated arginine methyltransferase 1	59035	https://www.ncbi.nlm.nih.gov/gene/?term=59035
Abtb2	Abtb2	ankyrin repeat and BTB (POZ) domain containing 2	99382	https://www.ncbi.nlm.nih.gov/gene/?term=99382
Gskip	Gskip	GSK3B interacting protein	66787	https://www.ncbi.nlm.nih.gov/gene/?term=66787
Spice1	Spice1	spindle and centriole associated protein 1	212514	https://www.ncbi.nlm.nih.gov/gene/?term=212514
Ube2b	Ube2b	ubiquitin-conjugating enzyme E2B	22210	https://www.ncbi.nlm.nih.gov/gene/?term=22210

Biology Open • Supplementary information

Crim1	Crim1	cysteine rich transmembrane BMP regulator 1 (chordin like)	50766	https://www.ncbi.nlm.nih.gov/gene/?term=50766
Pi15	Pi15	peptidase inhibitor 15	94227	https://www.ncbi.nlm.nih.gov/gene/?term=94227
Trak1	Trak1	trafficking protein, kinesin binding 1	67095	https://www.ncbi.nlm.nih.gov/gene/?term=67095
Lrrc32	Lrrc32	leucine rich repeat containing 32	434215	https://www.ncbi.nlm.nih.gov/gene/?term=434215
Esm1	Esm1	endothelial cell-specific molecule 1	71690	https://www.ncbi.nlm.nih.gov/gene/?term=71690
Slc35f3	Slc35f3	solute carrier family 35, member F3	210027	https://www.ncbi.nlm.nih.gov/gene/?term=210027
Sowaha	Sowaha	sosondowah ankyrin repeat domain family member A	237761	https://www.ncbi.nlm.nih.gov/gene/?term=237761
Phtf2	Phtf2	putative homeodomain transcription factor 2	68770	https://www.ncbi.nlm.nih.gov/gene/?term=68770
Zfp967	Zfp967	zinc finger protein 967	100303732	https://www.ncbi.nlm.nih.gov/gene/?term=100303
Sik3	Sik3	SIK family kinase 3	70661	https://www.ncbi.nlm.nih.gov/gene/?term=70661
Zfp975	Zfp975	zinc finger protein 975	434179	https://www.ncbi.nlm.nih.gov/gene/?term=434179
Zfp965	Zfp965	zinc finger protein 965	100503949	https://www.ncbi.nlm.nih.gov/gene/?term=100503
Cpd	Cpd	carboxypeptidase D	12874	https://www.ncbi.nlm.nih.gov/gene/?term=12874
Nwd2	Nwd2	NACHT and WD repeat domain containing 2	319807	https://www.ncbi.nlm.nih.gov/gene/?term=319807
Gm14296	Gm14296	predicted gene 14296	102639598	https://www.ncbi.nlm.nih.gov/gene/?term=102639
Gm14440	Gm14440	predicted gene 14440	100503353	https://www.ncbi.nlm.nih.gov/gene/?term=100503
Ccser1	Ccser1	coiled-coil serine rich 1	232035	https://www.ncbi.nlm.nih.gov/gene/?term=232035
Trappc6b	Trappc6b	trafficking protein particle complex 6B	78232	https://www.ncbi.nlm.nih.gov/gene/?term=78232
Zfp420	Zfp420	zinc finger protein 420	233058	https://www.ncbi.nlm.nih.gov/gene/?term=233058
Asxl2	Asxl2	additional sex combs like 2, transcriptional regulator	75302	https://www.ncbi.nlm.nih.gov/gene/?term=75302

Biology Open • Supplementary information

Rarres1	Rarres1	retinoic acid receptor responder (tazarotene induced) 1	109222	https://www.ncbi.nlm.nih.gov/gene/?term=109222
Tsc1	Tsc1	tuberous sclerosis 1	64930	https://www.ncbi.nlm.nih.gov/gene/?term=64930
Kcnab1	Kcnab1	potassium voltage-gated channel, shaker-related subfamily, beta member 1	16497	https://www.ncbi.nlm.nih.gov/gene/?term=16497
Slc44a4	Slc44a4	solute carrier family 44, member 4	70129	https://www.ncbi.nlm.nih.gov/gene/?term=70129
Ptgs1	Ptgs1	prostaglandin-endoperoxide synthase 1	19224	https://www.ncbi.nlm.nih.gov/gene/?term=19224
Tspan5	Tspan5	tetraspanin 5	56224	https://www.ncbi.nlm.nih.gov/gene/?term=56224
Mex3b	Mex3b	mex3 RNA binding family member B	108797	https://www.ncbi.nlm.nih.gov/gene/?term=108797
Slco1c1	Slco1c1	solute carrier organic anion transporter family, member 1c1	58807	https://www.ncbi.nlm.nih.gov/gene/?term=58807
Prkci	Prkci	protein kinase C, iota	18759	https://www.ncbi.nlm.nih.gov/gene/?term=18759
Foxj3	Foxj3	forkhead box J3	230700	https://www.ncbi.nlm.nih.gov/gene/?term=230700
Stx7	Stx7	syntaxin 7	53331	https://www.ncbi.nlm.nih.gov/gene/?term=53331
Matr3	Matr3	matrin 3	17184	https://www.ncbi.nlm.nih.gov/gene/?term=17184
Cks2	Cks2	CDC28 protein kinase regulatory subunit 2	66197	https://www.ncbi.nlm.nih.gov/gene/?term=66197
Zfp804a	Zfp804a	zinc finger protein 804A	241514	https://www.ncbi.nlm.nih.gov/gene/?term=241514
Ccdc146	Ccdc146	coiled-coil domain containing 146	75172	https://www.ncbi.nlm.nih.gov/gene/?term=75172
Plekhh1	Plekhh1	pleckstrin homology domain containing, family H (with MyTH4 domain) member 1	211945	https://www.ncbi.nlm.nih.gov/gene/?term=211945
Trpa1	Trpa1	transient receptor potential cation channel, subfamily A, member 1	277328	https://www.ncbi.nlm.nih.gov/gene/?term=277328
Usp7	Usp7	ubiquitin specific peptidase 7	252870	https://www.ncbi.nlm.nih.gov/gene/?term=252870

Biology Open • Supplementary information

Ugcg	Ugcg	UDP-glucose ceramide glucosyltransferase	22234	https://www.ncbi.nlm.nih.gov/gene/?term=22234
Plec	Plec	plectin	18810	https://www.ncbi.nlm.nih.gov/gene/?term=18810
Ccdc130	Ccdc130	coiled-coil domain containing 130	67736	https://www.ncbi.nlm.nih.gov/gene/?term=67736
Map6d1	Map6d1	MAP6 domain containing 1	208158	https://www.ncbi.nlm.nih.gov/gene/?term=208158
Srf	Srf	serum response factor	20807	https://www.ncbi.nlm.nih.gov/gene/?term=20807
Abt1	Abt1	activator of basal transcription 1	30946	https://www.ncbi.nlm.nih.gov/gene/?term=30946
Tmem248	Tmem248	transmembrane protein 248	71667	https://www.ncbi.nlm.nih.gov/gene/?term=71667
Runx3	Runx3	runt related transcription factor 3	12399	https://www.ncbi.nlm.nih.gov/gene/?term=12399
Psd2	Psd2	pleckstrin and Sec7 domain containing 2	74002	https://www.ncbi.nlm.nih.gov/gene/?term=74002
Lingo4	Lingo4	leucine rich repeat and Ig domain containing 4	320747	https://www.ncbi.nlm.nih.gov/gene/?term=320747
Otop2	Otop2	otopetrin 2	237987	https://www.ncbi.nlm.nih.gov/gene/?term=237987
Mnt	Mnt	max binding protein	17428	https://www.ncbi.nlm.nih.gov/gene/?term=17428
Mrps21	Mrps21	mitochondrial ribosomal protein S21	66292	https://www.ncbi.nlm.nih.gov/gene/?term=66292
Car7	Car7	carbonic anhydrase 7	12354	https://www.ncbi.nlm.nih.gov/gene/?term=12354
Pate3	Pate3	prostate and testis expressed 3	100312956	https://www.ncbi.nlm.nih.gov/gene/?term=100312
Pappa2	Pappa2	pappalysin 2	23850	https://www.ncbi.nlm.nih.gov/gene/?term=23850
Adcy1	Adcy1	adenylate cyclase 1	432530	https://www.ncbi.nlm.nih.gov/gene/?term=432530
Gpr37	Gpr37	G protein-coupled receptor 37	14763	https://www.ncbi.nlm.nih.gov/gene/?term=14763
Rspry1	Rspry1	ring finger and SPRY domain containing 1	67610	https://www.ncbi.nlm.nih.gov/gene/?term=67610
Tnfrsf23	Tnfrsf23	tumor necrosis factor receptor superfamily, member 23	79201	https://www.ncbi.nlm.nih.gov/gene/?term=79201
Vma21	Vma21	VMA21 vacuolar H+-ATPase homolog (S. cerevisiae)	67048	https://www.ncbi.nlm.nih.gov/gene/?term=67048

Biology Open • Supplementary information

Tnfrsf22	Tnfrsf22	tumor necrosis factor receptor superfamily, member 22	79202	https://www.ncbi.nlm.nih.gov/gene/?term=79202
Clta	Clta	clathrin, light polypeptide (Lca)	12757	https://www.ncbi.nlm.nih.gov/gene/?term=12757
Fads1	Fads1	fatty acid desaturase 1	76267	https://www.ncbi.nlm.nih.gov/gene/?term=76267
Plpp2	Plpp2	phospholipid phosphatase 2	50784	https://www.ncbi.nlm.nih.gov/gene/?term=50784
Arrb1	Arrb1	arrestin, beta 1	109689	https://www.ncbi.nlm.nih.gov/gene/?term=109689
Timm17a	Timm17a	translocase of inner mitochondrial membrane 17a	21854	https://www.ncbi.nlm.nih.gov/gene/?term=21854
Virma	Virma	vir like m6A methyltransferase associated	66185	https://www.ncbi.nlm.nih.gov/gene/?term=66185
Rbmx	Rbmx	RNA binding motif protein, X chromosome	19655	https://www.ncbi.nlm.nih.gov/gene/?term=19655
Map4	Map4	microtubule-associated protein 4	17758	https://www.ncbi.nlm.nih.gov/gene/?term=17758
Foxp4	Foxp4	forkhead box P4	74123	https://www.ncbi.nlm.nih.gov/gene/?term=74123
Sertad4	Sertad4	SERTA domain containing 4	214791	https://www.ncbi.nlm.nih.gov/gene/?term=214791
Ptprz1	Ptprz1	protein tyrosine phosphatase, receptor type Z, polypeptide 1	19283	https://www.ncbi.nlm.nih.gov/gene/?term=19283
Sgpp1	Sgpp1	sphingosine-1-phosphate phosphatase 1	81535	https://www.ncbi.nlm.nih.gov/gene/?term=81535
Gabpb2	Gabpb2	GA repeat binding protein, beta 2	213054	https://www.ncbi.nlm.nih.gov/gene/?term=213054
Aftph	Aftph	aftiphilin	216549	https://www.ncbi.nlm.nih.gov/gene/?term=216549
Rffl	Rffl	ring finger and FYVE like domain containing protein	67338	https://www.ncbi.nlm.nih.gov/gene/?term=67338
Sec61a1	Sec61a1	Sec61 alpha 1 subunit (S. cerevisiae)	53421	https://www.ncbi.nlm.nih.gov/gene/?term=53421
Tagln2	Tagln2	transgelin 2	21346	https://www.ncbi.nlm.nih.gov/gene/?term=21346
Shisa5	Shisa5	shisa family member 5	66940	https://www.ncbi.nlm.nih.gov/gene/?term=66940
Csnk1g3	Csnk1g3	casein kinase 1, gamma 3	70425	https://www.ncbi.nlm.nih.gov/gene/?term=70425

Biology Open • Supplementary information

Otc	Otc	ornithine transcarbamylase	18416	https://www.ncbi.nlm.nih.gov/gene/?term=18416
Actn4	Actn4	actinin alpha 4	60595	https://www.ncbi.nlm.nih.gov/gene/?term=60595
Tfap2d	Tfap2d	transcription factor AP-2, delta	226896	https://www.ncbi.nlm.nih.gov/gene/?term=226896
Praf2	Praf2	PRA1 domain family 2	54637	https://www.ncbi.nlm.nih.gov/gene/?term=54637
Enc1	Enc1	ectodermal-neural cortex 1	13803	https://www.ncbi.nlm.nih.gov/gene/?term=13803
Lasp1	Lasp1	LIM and SH3 protein 1	16796	https://www.ncbi.nlm.nih.gov/gene/?term=16796
Tax1bp1	Tax1bp1	Tax1 (human T cell leukemia virus type I) binding protein 1	52440	https://www.ncbi.nlm.nih.gov/gene/?term=52440
Maml1	Maml1	mastermind like transcriptional coactivator 1	103806	https://www.ncbi.nlm.nih.gov/gene/?term=103806
Ptbp1	Ptbp1	polypyrimidine tract binding protein 1	19205	https://www.ncbi.nlm.nih.gov/gene/?term=19205
Fam117b	Fam117b	family with sequence similarity 117, member B	72750	https://www.ncbi.nlm.nih.gov/gene/?term=72750
Elfn1	Elfn1	leucine rich repeat and fibronectin type III, extracellular 1	243312	https://www.ncbi.nlm.nih.gov/gene/?term=243312
P2rx4	P2rx4	purinergic receptor P2X, ligand-gated ion channel 4	18438	https://www.ncbi.nlm.nih.gov/gene/?term=18438
Cmpk1	Cmpk1	cytidine monophosphate (UMP-CMP) kinase 1	66588	https://www.ncbi.nlm.nih.gov/gene/?term=66588
Hsd17b11	Hsd17b11	hydroxysteroid (17-beta) dehydrogenase 11	114664	https://www.ncbi.nlm.nih.gov/gene/?term=114664
Cdyl2	Cdyl2	chromodomain protein, Y chromosome-like 2	75796	https://www.ncbi.nlm.nih.gov/gene/?term=75796
Zc3h11a	Zc3h11a	zinc finger CCCH type containing 11A	70579	https://www.ncbi.nlm.nih.gov/gene/?term=70579

Biology Open • Supplementary information

Smarcd1	Smarcd1	SWI/SNF related, matrix associated, actin dependent regulator of chromatin, subfamily d, member 1	83797	https://www.ncbi.nlm.nih.gov/gene/?term=83797
Sec61b	Sec61b	Sec61 beta subunit	66212	https://www.ncbi.nlm.nih.gov/gene/?term=66212
Ppp2cb	Ppp2cb	protein phosphatase 2 (formerly 2A), catalytic subunit, beta isoform	19053	https://www.ncbi.nlm.nih.gov/gene/?term=19053
Acat3	Acat3	acetyl-Coenzyme A acetyltransferase 3	224530	https://www.ncbi.nlm.nih.gov/gene/?term=224530
Dpysl4	Dpysl4	dihydropyrimidinase-like 4	26757	https://www.ncbi.nlm.nih.gov/gene/?term=26757
Adcyap1	Adcyap1	adenylate cyclase activating polypeptide 1	11516	https://www.ncbi.nlm.nih.gov/gene/?term=11516
Yes1	Yes1	YES proto-oncogene 1, Src family tyrosine kinase	22612	https://www.ncbi.nlm.nih.gov/gene/?term=22612
Gabarapl1	Gabarapl1	gamma-aminobutyric acid (GABA) A receptor-associated protein-like 1	57436	https://www.ncbi.nlm.nih.gov/gene/?term=57436
Glra2	Glra2	glycine receptor, alpha 2 subunit	237213	https://www.ncbi.nlm.nih.gov/gene/?term=237213
Eif4a1	Eif4a1	eukaryotic translation initiation factor 4A1	13681	https://www.ncbi.nlm.nih.gov/gene/?term=13681
Tspan15	Tspan15	tetraspanin 15	70423	https://www.ncbi.nlm.nih.gov/gene/?term=70423
Otud1	Otud1	OTU domain containing 1	71198	https://www.ncbi.nlm.nih.gov/gene/?term=71198
Stag2	Stag2	stromal antigen 2	20843	https://www.ncbi.nlm.nih.gov/gene/?term=20843
Alpk3	Alpk3	alpha-kinase 3	116904	https://www.ncbi.nlm.nih.gov/gene/?term=116904
Hepacam	Hepacam	hepatocyte cell adhesion molecule	72927	https://www.ncbi.nlm.nih.gov/gene/?term=72927
Snx27	Snx27	sorting nexin family member 27	76742	https://www.ncbi.nlm.nih.gov/gene/?term=76742
Slc25a32	Slc25a32	solute carrier family 25, member 32	69906	https://www.ncbi.nlm.nih.gov/gene/?term=69906

Fubp3	Fubp3	far upstream element (FUSE) binding protein 3	320267	https://www.ncbi.nlm.nih.gov/gene/?term=320267
Krit1	Krit1	KRIT1, ankyrin repeat containing	79264	https://www.ncbi.nlm.nih.gov/gene/?term=79264
Rnf121	Rnf121	ring finger protein 121	75212	https://www.ncbi.nlm.nih.gov/gene/?term=75212
Frmpd4	Frmpd4	FERM and PDZ domain containing 4	333605	https://www.ncbi.nlm.nih.gov/gene/?term=333605
Tinagl1	Tinagl1	tubulointerstitial nephritis antigen-like 1	94242	https://www.ncbi.nlm.nih.gov/gene/?term=94242
Plagl1	Plagl1	pleiomorphic adenoma gene-like 1	22634	https://www.ncbi.nlm.nih.gov/gene/?term=22634
Pter	Pter	phosphotriesterase related	19212	https://www.ncbi.nlm.nih.gov/gene/?term=19212
Spata13	Spata13	spermatogenesis associated 13	219140	https://www.ncbi.nlm.nih.gov/gene/?term=219140
Dennd1a	Dennd1a	DENN/MADD domain containing 1A	227801	https://www.ncbi.nlm.nih.gov/gene/?term=227801
Uspl1	Uspl1	ubiquitin specific peptidase like 1	231915	https://www.ncbi.nlm.nih.gov/gene/?term=231915
Cckbr	Cckbr	cholecystokinin B receptor	12426	https://www.ncbi.nlm.nih.gov/gene/?term=12426
Ranbp3l	Ranbp3l	RAN binding protein 3-like	223332	https://www.ncbi.nlm.nih.gov/gene/?term=223332
Slitrk5	Slitrk5	SLIT and NTRK-like family, member 5	75409	https://www.ncbi.nlm.nih.gov/gene/?term=75409
Fubp1	Fubp1	far upstream element (FUSE) binding protein 1	51886	https://www.ncbi.nlm.nih.gov/gene/?term=51886
Eno2	Eno2	enolase 2, gamma neuronal	13807	https://www.ncbi.nlm.nih.gov/gene/?term=13807
Paxip1	Paxip1	PAX interacting (with transcription-activation domain) protein 1	55982	https://www.ncbi.nlm.nih.gov/gene/?term=55982
Txnl1	Txnl1	thioredoxin-like 1	53382	https://www.ncbi.nlm.nih.gov/gene/?term=53382
Zfp609	Zfp609	zinc finger protein 609	214812	https://www.ncbi.nlm.nih.gov/gene/?term=214812
Morc2a	Morc2a	microrchidia 2A	74522	https://www.ncbi.nlm.nih.gov/gene/?term=74522
Tbx20	Tbx20	T-box 20	57246	https://www.ncbi.nlm.nih.gov/gene/?term=57246
Hars2	Hars2	histidyl-tRNA synthetase 2	70791	https://www.ncbi.nlm.nih.gov/gene/?term=70791
Cnnm4	Cnnm4	cyclin M4	94220	https://www.ncbi.nlm.nih.gov/gene/?term=94220
Nhsl2	Nhsl2	NHS-like 2	100042480	https://www.ncbi.nlm.nih.gov/gene/?term=100042

Hoxb6	Hoxb6	homeobox B6	15414	https://www.ncbi.nlm.nih.gov/gene/?term=15414
Kdr	Kdr	kinase insert domain protein receptor	16542	https://www.ncbi.nlm.nih.gov/gene/?term=16542
Mas1	Mas1	MAS1 oncogene	17171	https://www.ncbi.nlm.nih.gov/gene/?term=17171
Mtmr4	Mtmr4	myotubularin related protein 4	170749	https://www.ncbi.nlm.nih.gov/gene/?term=170749
Kdm5c	Kdm5c	lysine (K)-specific demethylase 5C	20591	https://www.ncbi.nlm.nih.gov/gene/?term=20591
Clvs2	Clvs2	clavesin 2	215890	https://www.ncbi.nlm.nih.gov/gene/?term=215890
Prss8	Prss8	protease, serine 8 (prostasin)	76560	https://www.ncbi.nlm.nih.gov/gene/?term=76560
Acnat1	Acnat1	acyl-coenzyme A amino acid N-acyltransferase 1	230161	https://www.ncbi.nlm.nih.gov/gene/?term=230161
Tgfb3	Tgfb3	transforming growth factor, beta 3	21809	https://www.ncbi.nlm.nih.gov/gene/?term=21809
Prkcq	Prkcq	protein kinase C, theta	18761	https://www.ncbi.nlm.nih.gov/gene/?term=18761
Trim66	Trim66	tripartite motif-containing 66	330627	https://www.ncbi.nlm.nih.gov/gene/?term=330627
Ube2q1	Ube2q1	ubiquitin-conjugating enzyme E2Q family member 1	70093	https://www.ncbi.nlm.nih.gov/gene/?term=70093
Tmem260	Tmem260	transmembrane protein 260	218989	https://www.ncbi.nlm.nih.gov/gene/?term=218989
8030462N17Rik	8030462N17Rik	RIKEN cDNA 8030462N17 gene	212163	https://www.ncbi.nlm.nih.gov/gene/?term=212163
Manf	Manf	mesencephalic astrocyte-derived neurotrophic factor	74840	https://www.ncbi.nlm.nih.gov/gene/?term=74840
Slc6a7	Slc6a7	solute carrier family 6 (neurotransmitter transporter, L-proline), member 7	240332	https://www.ncbi.nlm.nih.gov/gene/?term=240332
Pou2f3	Pou2f3	POU domain, class 2, transcription factor 3	18988	https://www.ncbi.nlm.nih.gov/gene/?term=18988
Syndig1	Syndig1	synapse differentiation inducing 1	433485	https://www.ncbi.nlm.nih.gov/gene/?term=433485
Sertm1	Sertm1	serine rich and transmembrane domain containing 1	329641	https://www.ncbi.nlm.nih.gov/gene/?term=329641

Biology Open • Supplementary information

Smc1a	Smc1a	structural maintenance of chromosomes 1A	24061	https://www.ncbi.nlm.nih.gov/gene/?term=24061
Trim8	Trim8	tripartite motif-containing 8	93679	https://www.ncbi.nlm.nih.gov/gene/?term=93679
Tmco5b	Tmco5b	transmembrane and coiled-coil domains 5B	75275	https://www.ncbi.nlm.nih.gov/gene/?term=75275
Ccdc191	Ccdc191	coiled-coil domain containing 191	212153	https://www.ncbi.nlm.nih.gov/gene/?term=212153
Pcca	Pcca	propionyl-Coenzyme A carboxylase, alpha polypeptide	110821	https://www.ncbi.nlm.nih.gov/gene/?term=110821
Dlx5	Dlx5	distal-less homeobox 5	13395	https://www.ncbi.nlm.nih.gov/gene/?term=13395
Clcn3	Clcn3	chloride channel, voltage-sensitive 3	12725	https://www.ncbi.nlm.nih.gov/gene/?term=12725
Fam118b	Fam118b	family with sequence similarity 118, member B	109229	https://www.ncbi.nlm.nih.gov/gene/?term=109229
Cxxc4	Cxxc4	CXXC finger 4	319478	https://www.ncbi.nlm.nih.gov/gene/?term=319478
Dnajc19	Dnajc19	DnaJ heat shock protein family (Hsp40) member C19	67713	https://www.ncbi.nlm.nih.gov/gene/?term=67713
St8sia4	St8sia4	ST8 alpha-N-acetyl-neuraminide alpha-2,8-sialyltransferase 4	20452	https://www.ncbi.nlm.nih.gov/gene/?term=20452
Pck1	Pck1	phosphoenolpyruvate carboxykinase 1, cytosolic	18534	https://www.ncbi.nlm.nih.gov/gene/?term=18534
H2bfm	H2bfm	H2B histone family, member M	69389	https://www.ncbi.nlm.nih.gov/gene/?term=69389
Vdac2	Vdac2	voltage-dependent anion channel 2	22334	https://www.ncbi.nlm.nih.gov/gene/?term=22334
Wwp1	Wwp1	WW domain containing E3 ubiquitin protein ligase 1	107568	https://www.ncbi.nlm.nih.gov/gene/?term=107568
Vav3	Vav3	vav 3 oncogene	57257	https://www.ncbi.nlm.nih.gov/gene/?term=57257
Cox16	Cox16	cytochrome c oxidase assembly protein 16	66272	https://www.ncbi.nlm.nih.gov/gene/?term=66272

Biology Open • Supplementary information

Gpr119	Gpr119	G-protein coupled receptor 119	236781	https://www.ncbi.nlm.nih.gov/gene/?term=236781
Ttc41	Ttc41	tetratricopeptide repeat domain 41	103220	https://www.ncbi.nlm.nih.gov/gene/?term=103220
Gorasp1	Gorasp1	golgi reassembly stacking protein 1	74498	https://www.ncbi.nlm.nih.gov/gene/?term=74498
Rnf146	Rnf146	ring finger protein 146	68031	https://www.ncbi.nlm.nih.gov/gene/?term=68031
Prkg1	Prkg1	protein kinase, cGMP-dependent, type I	19091	https://www.ncbi.nlm.nih.gov/gene/?term=19091
Rsf1	Rsf1	remodeling and spacing factor 1	233532	https://www.ncbi.nlm.nih.gov/gene/?term=233532
Arap2	Arap2	ArfGAP with RhoGAP domain, ankyrin repeat and PH domain 2	212285	https://www.ncbi.nlm.nih.gov/gene/?term=212285
9230112D13Rik	9230112D13Rik	RIKEN cDNA 9230112D13 gene	78243	https://www.ncbi.nlm.nih.gov/gene/?term=78243
Zfp275	Zfp275	zinc finger protein 275	27081	https://www.ncbi.nlm.nih.gov/gene/?term=27081
Acadsb	Acadsb	acyl-Coenzyme A dehydrogenase, short/branched chain	66885	https://www.ncbi.nlm.nih.gov/gene/?term=66885
Fam102b	Fam102b	family with sequence similarity 102, member B	329739	https://www.ncbi.nlm.nih.gov/gene/?term=329739
Gngt1	Gngt1	guanine nucleotide binding protein (G protein), gamma transducing activity polypeptide 1	14699	https://www.ncbi.nlm.nih.gov/gene/?term=14699
Gm20498	Gm20498	predicted gene 20498	105940408	https://www.ncbi.nlm.nih.gov/gene/?term=105940
Lmx1a	Lmx1a	LIM homeobox transcription factor 1 alpha	110648	https://www.ncbi.nlm.nih.gov/gene/?term=110648
Slain1	Slain1	SLAIN motif family, member 1	105439	https://www.ncbi.nlm.nih.gov/gene/?term=105439
Taf3	Taf3	TATA-box binding protein associated factor 3	209361	https://www.ncbi.nlm.nih.gov/gene/?term=209361
Etfdh	Etfdh	electron transferring flavoprotein, dehydrogenase	66841	https://www.ncbi.nlm.nih.gov/gene/?term=66841
Unc80	Unc80	unc-80, NALCN activator	329178	https://www.ncbi.nlm.nih.gov/gene/?term=329178
Efcab14	Efcab14	EF-hand calcium binding domain 14	230648	https://www.ncbi.nlm.nih.gov/gene/?term=230648

Biology Open • Supplementary information

Trip13	Trip13	thyroid hormone receptor interactor 13	69716	https://www.ncbi.nlm.nih.gov/gene/?term=69716
Mcmdc2	Mcmdc2	minichromosome maintenance domain containing 2	240697	https://www.ncbi.nlm.nih.gov/gene/?term=240697
Ppp1r42	Ppp1r42	protein phosphatase 1, regulatory subunit 42	69312	https://www.ncbi.nlm.nih.gov/gene/?term=69312
Lrrc58	Lrrc58	leucine rich repeat containing 58	320184	https://www.ncbi.nlm.nih.gov/gene/?term=320184
Kif5c	Kif5c	kinesin family member 5C	16574	https://www.ncbi.nlm.nih.gov/gene/?term=16574
Iqsec1	Iqsec1	IQ motif and Sec7 domain 1	232227	https://www.ncbi.nlm.nih.gov/gene/?term=232227
Iltifb	Iltifb	interleukin 10-related T cell-derived inducible factor beta	116849	https://www.ncbi.nlm.nih.gov/gene/?term=116849
Npat	Npat	nuclear protein in the AT region	244879	https://www.ncbi.nlm.nih.gov/gene/?term=244879
Ccdc71l	Ccdc71l	coiled-coil domain containing 71 like	72123	https://www.ncbi.nlm.nih.gov/gene/?term=72123
Olfr630	Olfr630	olfactory receptor 630	259102	https://www.ncbi.nlm.nih.gov/gene/?term=259102
Orc3	Orc3	origin recognition complex, subunit 3	50793	https://www.ncbi.nlm.nih.gov/gene/?term=50793
Cyp11b1	Cyp11b1	cytochrome P450, family 11, subfamily b, polypeptide 1	110115	https://www.ncbi.nlm.nih.gov/gene/?term=110115
Oprl1	Oprl1	opioid receptor-like 1	18389	https://www.ncbi.nlm.nih.gov/gene/?term=18389
Clic5	Clic5	chloride intracellular channel 5	224796	https://www.ncbi.nlm.nih.gov/gene/?term=224796
Klf3	Klf3	Kruppel-like factor 3 (basic)	16599	https://www.ncbi.nlm.nih.gov/gene/?term=16599
Akr1b3	Akr1b3	aldo-keto reductase family 1, member B3 (aldose reductase)	11677	https://www.ncbi.nlm.nih.gov/gene/?term=11677
Ehd4	Ehd4	EH-domain containing 4	98878	https://www.ncbi.nlm.nih.gov/gene/?term=98878
Gm3985	Gm3985	predicted gene 3985	100042715	https://www.ncbi.nlm.nih.gov/gene/?term=100042
Zfp128	Zfp128	zinc finger protein 128	243833	https://www.ncbi.nlm.nih.gov/gene/?term=243833
Ttc9c	Ttc9c	tetratricopeptide repeat domain 9C	70387	https://www.ncbi.nlm.nih.gov/gene/?term=70387

Biology Open • Supplementary information

2310009B15Rik	2310009B15Rik	RIKEN cDNA 2310009B15 gene	69549	https://www.ncbi.nlm.nih.gov/gene/?term=69549
Elp3	Elp3	elongator acetyltransferase complex subunit 3	74195	https://www.ncbi.nlm.nih.gov/gene/?term=74195
Cd200r2	Cd200r2	Cd200 receptor 2	271375	https://www.ncbi.nlm.nih.gov/gene/?term=271375
Serpinb3c	Serpinb3c	serine (or cysteine) peptidase inhibitor, clade B, member 3C	381286	https://www.ncbi.nlm.nih.gov/gene/?term=381286
Nhlh2	Nhlh2	nescient helix loop helix 2	18072	https://www.ncbi.nlm.nih.gov/gene/?term=18072
Psg16	Psg16	pregnancy specific glycoprotein 16	26436	https://www.ncbi.nlm.nih.gov/gene/?term=26436
Gm609	Gm609	predicted gene 609	208166	https://www.ncbi.nlm.nih.gov/gene/?term=208166
Stmn1	Stmn1	stathmin 1	16765	https://www.ncbi.nlm.nih.gov/gene/?term=16765
Magi2	Magi2	membrane associated guanylate kinase, WW and PDZ domain containing 2	50791	https://www.ncbi.nlm.nih.gov/gene/?term=50791
Rnf2	Rnf2	ring finger protein 2	19821	https://www.ncbi.nlm.nih.gov/gene/?term=19821
Serpinb3b	Serpinb3b	serine (or cysteine) peptidase inhibitor, clade B (ovalbumin), member 3B	383548	https://www.ncbi.nlm.nih.gov/gene/?term=383548
Serpinb3a	Serpinb3a	serine (or cysteine) peptidase inhibitor, clade B (ovalbumin), member 3A	20248	https://www.ncbi.nlm.nih.gov/gene/?term=20248
Itm2a	Itm2a	integral membrane protein 2A	16431	https://www.ncbi.nlm.nih.gov/gene/?term=16431
Gpr141b	Gpr141b	G protein-coupled receptor 141B	319293	https://www.ncbi.nlm.nih.gov/gene/?term=319293
Ap5s1	Ap5s1	adaptor-related protein 5 complex, sigma 1 subunit	69596	https://www.ncbi.nlm.nih.gov/gene/?term=69596
Xkrx	Xkrx	X-linked Kx blood group related, X-linked	331524	https://www.ncbi.nlm.nih.gov/gene/?term=331524
Tesk2	Tesk2	testis-specific kinase 2	230661	https://www.ncbi.nlm.nih.gov/gene/?term=230661

Biology Open • Supplementary information

Tpt1	Tpt1	tumor protein, translationally-controlled 1	22070	https://www.ncbi.nlm.nih.gov/gene/?term=22070
Hk3	Hk3	hexokinase 3	212032	https://www.ncbi.nlm.nih.gov/gene/?term=212032
Gtf3c2	Gtf3c2	general transcription factor IIIC, polypeptide 2, beta	71752	https://www.ncbi.nlm.nih.gov/gene/?term=71752
Gabarapl2	Gabarapl2	gamma-aminobutyric acid (GABA) A receptor-associated protein-like 2	93739	https://www.ncbi.nlm.nih.gov/gene/?term=93739
Sar1a	Sar1a	secretion associated Ras related GTPase 1A	20224	https://www.ncbi.nlm.nih.gov/gene/?term=20224
Ppih	Ppih	peptidyl prolyl isomerase H	66101	https://www.ncbi.nlm.nih.gov/gene/?term=66101
Tenm3	Tenm3	teneurin transmembrane protein 3	23965	https://www.ncbi.nlm.nih.gov/gene/?term=23965
Camk2n1	Camk2n1	calcium/calmodulin-dependent protein kinase II inhibitor 1	66259	https://www.ncbi.nlm.nih.gov/gene/?term=66259
Tti1	Tti1	TELO2 interacting protein 1	75425	https://www.ncbi.nlm.nih.gov/gene/?term=75425
Cul3	Cul3	cullin 3	26554	https://www.ncbi.nlm.nih.gov/gene/?term=26554
Nol4	Nol4	nucleolar protein 4	319211	https://www.ncbi.nlm.nih.gov/gene/?term=319211
Colec12	Colec12	collectin sub-family member 12	140792	https://www.ncbi.nlm.nih.gov/gene/?term=140792
Fbxo42	Fbxo42	F-box protein 42	213499	https://www.ncbi.nlm.nih.gov/gene/?term=213499
Sccpdh	Sccpdh	saccharopine dehydrogenase (putative)	109232	https://www.ncbi.nlm.nih.gov/gene/?term=109232
Tor1b	Tor1b	torsin family 1, member B	30934	https://www.ncbi.nlm.nih.gov/gene/?term=30934
Hfm1	Hfm1	HFM1, ATP-dependent DNA helicase homolog	330149	https://www.ncbi.nlm.nih.gov/gene/?term=330149
Mob2	Mob2	MOB kinase activator 2	101513	https://www.ncbi.nlm.nih.gov/gene/?term=101513
Myf5	Myf5	myogenic factor 5	17877	https://www.ncbi.nlm.nih.gov/gene/?term=17877
Chmp4c	Chmp4c	charged multivesicular body protein 4C	66371	https://www.ncbi.nlm.nih.gov/gene/?term=66371

Biology Open • Supplementary information

Bnip2	Bnip2	BCL2/adenovirus E1B interacting protein 2	12175	https://www.ncbi.nlm.nih.gov/gene/?term=12175
Zhx2	Zhx2	zinc fingers and homeoboxes 2	387609	https://www.ncbi.nlm.nih.gov/gene/?term=387609
Miga2	Miga2	mitoguardin 2	108958	https://www.ncbi.nlm.nih.gov/gene/?term=108958
Fstl5	Fstl5	follistatin-like 5	213262	https://www.ncbi.nlm.nih.gov/gene/?term=213262
Polq	Polq	polymerase (DNA directed), theta	77782	https://www.ncbi.nlm.nih.gov/gene/?term=77782
Zfp11	Zfp11	zinc finger protein 11	22648	https://www.ncbi.nlm.nih.gov/gene/?term=22648
Skida1	Skida1	SKI/DACH domain containing 1	72668	https://www.ncbi.nlm.nih.gov/gene/?term=72668
Map3k8	Map3k8	mitogen-activated protein kinase kinase kinase 8	26410	https://www.ncbi.nlm.nih.gov/gene/?term=26410
Tspyl2	Tspyl2	TSPY-like 2	52808	https://www.ncbi.nlm.nih.gov/gene/?term=52808
Emx2	Emx2	empty spiracles homeobox 2	13797	https://www.ncbi.nlm.nih.gov/gene/?term=13797
Suv39h1	Suv39h1	suppressor of variegation 3-9 1	20937	https://www.ncbi.nlm.nih.gov/gene/?term=20937
Mtbp	Mtbp	Mdm2, transformed 3T3 cell double minute p53 binding protein	105837	https://www.ncbi.nlm.nih.gov/gene/?term=105837
Snx5	Snx5	sorting nexin 5	69178	https://www.ncbi.nlm.nih.gov/gene/?term=69178
Pdzd7	Pdzd7	PDZ domain containing 7	100503041	https://www.ncbi.nlm.nih.gov/gene/?term=100503
Cldn8	Cldn8	claudin 8	54420	https://www.ncbi.nlm.nih.gov/gene/?term=54420
Ubxn8	Ubxn8	UBX domain protein 8	108159	https://www.ncbi.nlm.nih.gov/gene/?term=108159
Naa30	Naa30	N(alpha)-acetyltransferase 30, NatC catalytic subunit	70646	https://www.ncbi.nlm.nih.gov/gene/?term=70646
Myrf	Myrf	myelin regulatory factor	225908	https://www.ncbi.nlm.nih.gov/gene/?term=225908
Kcnd2	Kcnd2	potassium voltage-gated channel, Shal-related family, member 2	16508	https://www.ncbi.nlm.nih.gov/gene/?term=16508
Rnf216	Rnf216	ring finger protein 216	108086	https://www.ncbi.nlm.nih.gov/gene/?term=108086
Fgf9	Fgf9	fibroblast growth factor 9	14180	https://www.ncbi.nlm.nih.gov/gene/?term=14180
Casd1	Casd1	CAS1 domain containing 1	213819	https://www.ncbi.nlm.nih.gov/gene/?term=213819
Btbd7	Btbd7	BTB (POZ) domain containing 7	238386	https://www.ncbi.nlm.nih.gov/gene/?term=238386

Biology Open • Supplementary information

Fastk	Fastk	Fas-activated serine/threonine kinase	66587	https://www.ncbi.nlm.nih.gov/gene/?term=66587
E2f7	E2f7	E2F transcription factor 7	52679	https://www.ncbi.nlm.nih.gov/gene/?term=52679
Rtn1	Rtn1	reticulon 1	104001	https://www.ncbi.nlm.nih.gov/gene/?term=104001
Mylk	Mylk	myosin, light polypeptide kinase	107589	https://www.ncbi.nlm.nih.gov/gene/?term=107589
Abhd3	Abhd3	abhydrolase domain containing 3	106861	https://www.ncbi.nlm.nih.gov/gene/?term=106861
Erbin	Erbin	Erbb2 interacting protein	59079	https://www.ncbi.nlm.nih.gov/gene/?term=59079
Wasf1	Wasf1	WAS protein family, member 1	83767	https://www.ncbi.nlm.nih.gov/gene/?term=83767
Jakmip1	Jakmip1	janus kinase and microtubule interacting protein 1	76071	https://www.ncbi.nlm.nih.gov/gene/?term=76071
Fibin	Fibin	fin bud initiation factor homolog (zebrafish)	67606	https://www.ncbi.nlm.nih.gov/gene/?term=67606
Apcdd1	Apcdd1	adenomatosis polyposis coli down-regulated 1	494504	https://www.ncbi.nlm.nih.gov/gene/?term=494504
Cdk2	Cdk2	cyclin-dependent kinase 2	12566	https://www.ncbi.nlm.nih.gov/gene/?term=12566
Map7	Map7	microtubule-associated protein 7	17761	https://www.ncbi.nlm.nih.gov/gene/?term=17761
Tnks1bp1	Tnks1bp1	tankyrase 1 binding protein 1	228140	https://www.ncbi.nlm.nih.gov/gene/?term=228140
Vwde	Vwde	von Willebrand factor D and EGF domains	232585	https://www.ncbi.nlm.nih.gov/gene/?term=232585
Rab5c	Rab5c	RAB5C, member RAS oncogene family	19345	https://www.ncbi.nlm.nih.gov/gene/?term=19345
Dpysl2	Dpysl2	dihydropyrimidinase-like 2	12934	https://www.ncbi.nlm.nih.gov/gene/?term=12934
Yme1l1	Yme1l1	YME1-like 1 (S. cerevisiae)	27377	https://www.ncbi.nlm.nih.gov/gene/?term=27377
Itfg1	Itfg1	integrin alpha FG-GAP repeat containing 1	71927	https://www.ncbi.nlm.nih.gov/gene/?term=71927
Man1a	Man1a	mannosidase 1, alpha	17155	https://www.ncbi.nlm.nih.gov/gene/?term=17155
Myof	Myof	myoferlin	226101	https://www.ncbi.nlm.nih.gov/gene/?term=226101
Wdr36	Wdr36	WD repeat domain 36	225348	https://www.ncbi.nlm.nih.gov/gene/?term=225348

Biology Open • Supplementary information

Cep95	Cep95	centrosomal protein 95	320162	https://www.ncbi.nlm.nih.gov/gene/?term=320162
0610009B22Rik	0610009B22Rik	RIKEN cDNA 0610009B22 gene	66050	https://www.ncbi.nlm.nih.gov/gene/?term=66050
Cul4b	Cul4b	cullin 4B	72584	https://www.ncbi.nlm.nih.gov/gene/?term=72584
Zfp383	Zfp383	zinc finger protein 383	73729	https://www.ncbi.nlm.nih.gov/gene/?term=73729
Zic1	Zic1	zinc finger protein of the cerebellum 1	22771	https://www.ncbi.nlm.nih.gov/gene/?term=22771
Mettl9	Mettl9	methyltransferase like 9	59052	https://www.ncbi.nlm.nih.gov/gene/?term=59052
Tmem29	Tmem29	transmembrane protein 29	382245	https://www.ncbi.nlm.nih.gov/gene/?term=382245
Rad23a	Rad23a	RAD23 homolog A, nucleotide excision repair protein	19358	https://www.ncbi.nlm.nih.gov/gene/?term=19358
Vbp1	Vbp1	von Hippel-Lindau binding protein 1	22327	https://www.ncbi.nlm.nih.gov/gene/?term=22327
Iqub	Iqub	IQ motif and ubiquitin domain containing	214704	https://www.ncbi.nlm.nih.gov/gene/?term=214704
Ankrd12	Ankrd12	ankyrin repeat domain 12	106585	https://www.ncbi.nlm.nih.gov/gene/?term=106585
Zfand4	Zfand4	zinc finger, AN1-type domain 4	67492	https://www.ncbi.nlm.nih.gov/gene/?term=67492
Jrkl	Jrkl	Jrk-like	77532	https://www.ncbi.nlm.nih.gov/gene/?term=77532
Kcnj12	Kcnj12	potassium inwardly-rectifying channel, subfamily J, member 12	16515	https://www.ncbi.nlm.nih.gov/gene/?term=16515
Kmt2e	Kmt2e	lysine (K)-specific methyltransferase 2E	69188	https://www.ncbi.nlm.nih.gov/gene/?term=69188
Lpar4	Lpar4	lysophosphatidic acid receptor 4	78134	https://www.ncbi.nlm.nih.gov/gene/?term=78134
Tent2	Tent2	terminal nucleotidyltransferase 2	100715	https://www.ncbi.nlm.nih.gov/gene/?term=100715
Tmtc3	Tmtc3	transmembrane and tetratricopeptide repeat containing 3	237500	https://www.ncbi.nlm.nih.gov/gene/?term=237500
Dclre1a	Dclre1a	DNA cross-link repair 1A	55947	https://www.ncbi.nlm.nih.gov/gene/?term=55947

Biology Open • Supplementary information

Tmed7	Tmed7	transmembrane p24 trafficking protein 7	66676	https://www.ncbi.nlm.nih.gov/gene/?term=66676
Sash3	Sash3	SAM and SH3 domain containing 3	74131	https://www.ncbi.nlm.nih.gov/gene/?term=74131
Stard6	Stard6	StAR-related lipid transfer (START) domain containing 6	170461	https://www.ncbi.nlm.nih.gov/gene/?term=170461
Nup54	Nup54	nucleoporin 54	269113	https://www.ncbi.nlm.nih.gov/gene/?term=269113
Galntl6	Galntl6	UDP-N-acetyl-alpha-D-galactosamine:polypeptide N-acetylgalactosaminyltransferase-like 6	270049	https://www.ncbi.nlm.nih.gov/gene/?term=270049
Tmem158	Tmem158	transmembrane protein 158	72309	https://www.ncbi.nlm.nih.gov/gene/?term=72309
Slit3	Slit3	slit guidance ligand 3	20564	https://www.ncbi.nlm.nih.gov/gene/?term=20564
Slc30a10	Slc30a10	solute carrier family 30, member 10	226781	https://www.ncbi.nlm.nih.gov/gene/?term=226781
Cdc73	Cdc73	cell division cycle 73, Paf1/RNA polymerase II complex component	214498	https://www.ncbi.nlm.nih.gov/gene/?term=214498
Rrp36	Rrp36	ribosomal RNA processing 36 homolog (S. cerevisiae)	224823	https://www.ncbi.nlm.nih.gov/gene/?term=224823
Tmem68	Tmem68	transmembrane protein 68	72098	https://www.ncbi.nlm.nih.gov/gene/?term=72098
Gm3696	Gm3696	predicted gene 3696	100042149	https://www.ncbi.nlm.nih.gov/gene/?term=100042
Anks1b	Anks1b	ankyrin repeat and sterile alpha motif domain containing 1B	77531	https://www.ncbi.nlm.nih.gov/gene/?term=77531
Erlec1	Erlec1	endoplasmic reticulum lectin 1	66753	https://www.ncbi.nlm.nih.gov/gene/?term=66753
Sftpa1	Sftpa1	surfactant associated protein A1	20387	https://www.ncbi.nlm.nih.gov/gene/?term=20387
Gm3411	Gm3411	predicted gene 3411	100041569	https://www.ncbi.nlm.nih.gov/gene/?term=100041
Npnt	Npnt	nephronectin	114249	https://www.ncbi.nlm.nih.gov/gene/?term=114249
Rad21	Rad21	RAD21 cohesin complex component	19357	https://www.ncbi.nlm.nih.gov/gene/?term=19357

Biology Open • Supplementary information

Ralgapa1	Ralgapa1	Ral GTPase activating protein, alpha subunit 1	56784	https://www.ncbi.nlm.nih.gov/gene/?term=56784
Lpin3	Lpin3	lipin 3	64899	https://www.ncbi.nlm.nih.gov/gene/?term=64899
Cngb1	Cngb1	cyclic nucleotide gated channel beta 1	333329	https://www.ncbi.nlm.nih.gov/gene/?term=333329
Tmem41b	Tmem41b	transmembrane protein 41B	233724	https://www.ncbi.nlm.nih.gov/gene/?term=233724
4930555G01Rik	4930555G01Rik	RIKEN cDNA 4930555G01 gene	108978	https://www.ncbi.nlm.nih.gov/gene/?term=108978
Bicd1	Bicd1	BICD cargo adaptor 1	12121	https://www.ncbi.nlm.nih.gov/gene/?term=12121
Fat3	Fat3	FAT atypical cadherin 3	270120	https://www.ncbi.nlm.nih.gov/gene/?term=270120
Ercc2	Ercc2	excision repair cross-complementing rodent repair deficiency, complementation group 2	13871	https://www.ncbi.nlm.nih.gov/gene/?term=13871
Slc39a8	Slc39a8	solute carrier family 39 (metal ion transporter), member 8	67547	https://www.ncbi.nlm.nih.gov/gene/?term=67547
Foxa2	Foxa2	forkhead box A2	15376	https://www.ncbi.nlm.nih.gov/gene/?term=15376
Cdh5	Cdh5	cadherin 5	12562	https://www.ncbi.nlm.nih.gov/gene/?term=12562
Mmgt1	Mmgt1	membrane magnesium transporter 1	236792	https://www.ncbi.nlm.nih.gov/gene/?term=236792
Slc16a6	Slc16a6	solute carrier family 16 (monocarboxylic acid transporters), member 6	104681	https://www.ncbi.nlm.nih.gov/gene/?term=104681
Zfp160	Zfp160	zinc finger protein 160	224585	https://www.ncbi.nlm.nih.gov/gene/?term=224585
Eri1	Eri1	exoribonuclease 1	67276	https://www.ncbi.nlm.nih.gov/gene/?term=67276
Pnoc	Pnoc	prepronociceptin	18155	https://www.ncbi.nlm.nih.gov/gene/?term=18155
Aqp9	Aqp9	aquaporin 9	64008	https://www.ncbi.nlm.nih.gov/gene/?term=64008
Vmn1r32	Vmn1r32	vomeronasal 1 receptor 32	171188	https://www.ncbi.nlm.nih.gov/gene/?term=171188

Biology Open • Supplementary information

Asap2	Asap2	ArfGAP with SH3 domain, ankyrin repeat and PH domain 2	211914	https://www.ncbi.nlm.nih.gov/gene/?term=211914
Cdca4	Cdca4	cell division cycle associated 4	71963	https://www.ncbi.nlm.nih.gov/gene/?term=71963
Acot2	Acot2	acyl-CoA thioesterase 2	171210	https://www.ncbi.nlm.nih.gov/gene/?term=171210
Dll3	Dll3	delta like canonical Notch ligand 3	13389	https://www.ncbi.nlm.nih.gov/gene/?term=13389
Ttr	Ttr	transthyretin	22139	https://www.ncbi.nlm.nih.gov/gene/?term=22139
Tmem108	Tmem108	transmembrane protein 108	81907	https://www.ncbi.nlm.nih.gov/gene/?term=81907
Pcna	Pcna	proliferating cell nuclear antigen	18538	https://www.ncbi.nlm.nih.gov/gene/?term=18538
Hivep2	Hivep2	human immunodeficiency virus type I enhancer binding protein 2	15273	https://www.ncbi.nlm.nih.gov/gene/?term=15273
Nkain2	Nkain2	Na+/K+ transporting ATPase interacting 2	432450	https://www.ncbi.nlm.nih.gov/gene/?term=432450
Arhgap26	Arhgap26	Rho GTPase activating protein 26	71302	https://www.ncbi.nlm.nih.gov/gene/?term=71302
Brk1	Brk1	BRICK1, SCAR/WAVE actin-nucleating complex subunit	101314	https://www.ncbi.nlm.nih.gov/gene/?term=101314
Sike1	Sike1	suppressor of IKBKE 1	66641	https://www.ncbi.nlm.nih.gov/gene/?term=66641
Fam210b	Fam210b	family with sequence similarity 210, member B	67017	https://www.ncbi.nlm.nih.gov/gene/?term=67017
Prpf8	Prpf8	pre-mRNA processing factor 8	192159	https://www.ncbi.nlm.nih.gov/gene/?term=192159
Ablim1	Ablim1	actin-binding LIM protein 1	226251	https://www.ncbi.nlm.nih.gov/gene/?term=226251
Rtl5	Rtl5	retrotransposon Gag like 5	331474	https://www.ncbi.nlm.nih.gov/gene/?term=331474
Primpol	Primpol	primase and polymerase (DNA-directed)	408022	https://www.ncbi.nlm.nih.gov/gene/?term=408022
Fancl	Fancl	Fanconi anemia, complementation group L	67030	https://www.ncbi.nlm.nih.gov/gene/?term=67030
Chst11	Chst11	carbohydrate sulfotransferase 11	58250	https://www.ncbi.nlm.nih.gov/gene/?term=58250
Zfp101	Zfp101	zinc finger protein 101	22643	https://www.ncbi.nlm.nih.gov/gene/?term=22643

Biology Open • Supplementary information

Wnk1	Wnk1	WNK lysine deficient protein kinase 1	232341	https://www.ncbi.nlm.nih.gov/gene/?term=232341
Ptprr	Ptprr	protein tyrosine phosphatase, receptor type, R	19279	https://www.ncbi.nlm.nih.gov/gene/?term=19279
Ncbp2	Ncbp2	nuclear cap binding protein subunit 2	68092	https://www.ncbi.nlm.nih.gov/gene/?term=68092
Tfg	Tfg	Trk-fused gene	21787	https://www.ncbi.nlm.nih.gov/gene/?term=21787
Nkapl	Nkapl	NFKB activating protein-like	66707	https://www.ncbi.nlm.nih.gov/gene/?term=66707
Ap1s1	Ap1s1	adaptor protein complex AP-1, sigma 1	11769	https://www.ncbi.nlm.nih.gov/gene/?term=11769
Hacd3	Hacd3	3-hydroxyacyl-CoA dehydratase 3	57874	https://www.ncbi.nlm.nih.gov/gene/?term=57874
Bdnf	Bdnf	brain derived neurotrophic factor	12064	https://www.ncbi.nlm.nih.gov/gene/?term=12064
Snx2	Snx2	sorting nexin 2	67804	https://www.ncbi.nlm.nih.gov/gene/?term=67804
Xpo6	Xpo6	exportin 6	74204	https://www.ncbi.nlm.nih.gov/gene/?term=74204
Cebpz	Cebpz	CCAAT/enhancer binding protein zeta	12607	https://www.ncbi.nlm.nih.gov/gene/?term=12607
Ust	Ust	uronyl-2-sulfotransferase	338362	https://www.ncbi.nlm.nih.gov/gene/?term=338362
Sprn	Sprn	shadow of prion protein	212518	https://www.ncbi.nlm.nih.gov/gene/?term=212518
Cap1	Cap1	CAP, adenylate cyclase-associated protein 1 (yeast)	12331	https://www.ncbi.nlm.nih.gov/gene/?term=12331
Pax7	Pax7	paired box 7	18509	https://www.ncbi.nlm.nih.gov/gene/?term=18509
Cttnbp2nl	Cttnbp2nl	CTTNBP2 N-terminal like	80281	https://www.ncbi.nlm.nih.gov/gene/?term=80281
Caap1	Caap1	caspase activity and apoptosis inhibitor 1	67770	https://www.ncbi.nlm.nih.gov/gene/?term=67770
Msantd2	Msantd2	Myb/SANT-like DNA-binding domain containing 2	235184	https://www.ncbi.nlm.nih.gov/gene/?term=235184
Asxl3	Asxl3	additional sex combs like 3, transcriptional regulator	211961	https://www.ncbi.nlm.nih.gov/gene/?term=211961

Biology Open • Supplementary information

Mtx1	Mtx1	metaxin 1	17827	https://www.ncbi.nlm.nih.gov/gene/?term=17827
Plxna4	Plxna4	plexin A4	243743	https://www.ncbi.nlm.nih.gov/gene/?term=243743
Tpm3	Tpm3	tropomyosin 3, gamma	59069	https://www.ncbi.nlm.nih.gov/gene/?term=59069
Ccdc166	Ccdc166	coiled-coil domain containing 166	223648	https://www.ncbi.nlm.nih.gov/gene/?term=223648
Stc2	Stc2	stanniocalcin 2	20856	https://www.ncbi.nlm.nih.gov/gene/?term=20856
Kcnip3	Kcnip3	Kv channel interacting protein 3, calsenilin	56461	https://www.ncbi.nlm.nih.gov/gene/?term=56461
Phax	Phax	phosphorylated adaptor for RNA export	56698	https://www.ncbi.nlm.nih.gov/gene/?term=56698
Arcn1	Arcn1	archain 1	213827	https://www.ncbi.nlm.nih.gov/gene/?term=213827
Cmpk2	Cmpk2	cytidine monophosphate (UMP-CMP) kinase 2, mitochondrial	22169	https://www.ncbi.nlm.nih.gov/gene/?term=22169
G6pdx	G6pdx	glucose-6-phosphate dehydrogenase X-linked	14381	https://www.ncbi.nlm.nih.gov/gene/?term=14381
Lrch1	Lrch1	leucine-rich repeats and calponin homology (CH) domain containing 1	380916	https://www.ncbi.nlm.nih.gov/gene/?term=380916
Map1a	Map1a	microtubule-associated protein 1 A	17754	https://www.ncbi.nlm.nih.gov/gene/?term=17754
Sri	Sri	sorcin	109552	https://www.ncbi.nlm.nih.gov/gene/?term=109552
4930563E22Rik	4930563E22Rik	RIKEN cDNA 4930563E22 gene	75304	https://www.ncbi.nlm.nih.gov/gene/?term=75304
Mon2	Mon2	MON2 homolog, regulator of endosome to Golgi trafficking	67074	https://www.ncbi.nlm.nih.gov/gene/?term=67074
Hnrnpu	Hnrnpu	heterogeneous nuclear ribonucleoprotein U	51810	https://www.ncbi.nlm.nih.gov/gene/?term=51810
Hmcn1	Hmcn1	hemicentin 1	545370	https://www.ncbi.nlm.nih.gov/gene/?term=545370
Tra2b	Tra2b	transformer 2 beta	20462	https://www.ncbi.nlm.nih.gov/gene/?term=20462

Biology Open • Supplementary information

Cited2	Cited2	Cbp/p300-interacting transactivator, with Glu/Asp-rich carboxy-terminal domain, 2	17684	https://www.ncbi.nlm.nih.gov/gene/?term=17684
Uhrf1bp1l	Uhrf1bp1l	UHRF1 (ICBP90) binding protein 1-like	75089	https://www.ncbi.nlm.nih.gov/gene/?term=75089
Hsp90b1	Hsp90b1	heat shock protein 90, beta (Grp94), member 1	22027	https://www.ncbi.nlm.nih.gov/gene/?term=22027
Ankrd29	Ankrd29	ankyrin repeat domain 29	225187	https://www.ncbi.nlm.nih.gov/gene/?term=225187
Gja1	Gja1	gap junction protein, alpha 1	14609	https://www.ncbi.nlm.nih.gov/gene/?term=14609
Edn1	Edn1	endothelin 1	13614	https://www.ncbi.nlm.nih.gov/gene/?term=13614
Ss18	Ss18	SS18, nBAF chromatin remodeling complex subunit	268996	https://www.ncbi.nlm.nih.gov/gene/?term=268996
Esp34	Esp34	exocrine gland secreted peptide 34	100126773	https://www.ncbi.nlm.nih.gov/gene/?term=100126
Adprm	Adprm	ADP-ribose/CDP-alcohol diphosphatase, manganese dependent	66358	https://www.ncbi.nlm.nih.gov/gene/?term=66358
Ip6k2	Ip6k2	inositol hexaphosphate kinase 2	76500	https://www.ncbi.nlm.nih.gov/gene/?term=76500
Tex11	Tex11	testis expressed gene 11	83558	https://www.ncbi.nlm.nih.gov/gene/?term=83558
Hinfp	Hinfp	histone H4 transcription factor	102423	https://www.ncbi.nlm.nih.gov/gene/?term=102423
Osbpl7	Osbpl7	oxysterol binding protein-like 7	71240	https://www.ncbi.nlm.nih.gov/gene/?term=71240
Ccsap	Ccsap	centriole, cilia and spindle associated protein	73420	https://www.ncbi.nlm.nih.gov/gene/?term=73420
Ddx5	Ddx5	DEAD (Asp-Glu-Ala-Asp) box polypeptide 5	13207	https://www.ncbi.nlm.nih.gov/gene/?term=13207
Snai2	Snai2	snail family zinc finger 2	20583	https://www.ncbi.nlm.nih.gov/gene/?term=20583
Tppp	Tppp	tubulin polymerization promoting protein	72948	https://www.ncbi.nlm.nih.gov/gene/?term=72948

Biology Open • Supplementary information

Serp1	Serp1	stress-associated endoplasmic reticulum protein 1	28146	https://www.ncbi.nlm.nih.gov/gene/?term=28146
Mmd2	Mmd2	monocyte to macrophage differentiation-associated 2	75104	https://www.ncbi.nlm.nih.gov/gene/?term=75104
Tmem178	Tmem178	transmembrane protein 178	68027	https://www.ncbi.nlm.nih.gov/gene/?term=68027
Eif1ax	Eif1ax	eukaryotic translation initiation factor 1A, X-linked	66235	https://www.ncbi.nlm.nih.gov/gene/?term=66235
Dach2	Dach2	dachshund family transcription factor 2	93837	https://www.ncbi.nlm.nih.gov/gene/?term=93837
Tmem18	Tmem18	transmembrane protein 18	211986	https://www.ncbi.nlm.nih.gov/gene/?term=211986
Unc119b	Unc119b	unc-119 lipid binding chaperone B	106840	https://www.ncbi.nlm.nih.gov/gene/?term=106840
Hspd1	Hspd1	heat shock protein 1 (chaperonin)	15510	https://www.ncbi.nlm.nih.gov/gene/?term=15510
Cdk14	Cdk14	cyclin-dependent kinase 14	18647	https://www.ncbi.nlm.nih.gov/gene/?term=18647
Sec22b	Sec22b	SEC22 homolog B, vesicle trafficking protein	20333	https://www.ncbi.nlm.nih.gov/gene/?term=20333
Neto1	Neto1	neuropilin (NRP) and tolloid (TLL)-like 1	246317	https://www.ncbi.nlm.nih.gov/gene/?term=246317
Smim14	Smim14	small integral membrane protein 14	68552	https://www.ncbi.nlm.nih.gov/gene/?term=68552
Ube4a	Ube4a	ubiquitination factor E4A	140630	https://www.ncbi.nlm.nih.gov/gene/?term=140630
Ppib	Ppib	peptidylprolyl isomerase B	19035	https://www.ncbi.nlm.nih.gov/gene/?term=19035
Wdr48	Wdr48	WD repeat domain 48	67561	https://www.ncbi.nlm.nih.gov/gene/?term=67561
Syt1	Syt1	synaptotagmin I	20979	https://www.ncbi.nlm.nih.gov/gene/?term=20979
Ywhaz	Ywhaz	tyrosine 3-monooxygenase/tryptophan 5-monooxygenase activation protein, zeta polypeptide	22631	https://www.ncbi.nlm.nih.gov/gene/?term=22631

Biology Open • Supplementary information

Mrps31	Mrps31	mitochondrial ribosomal protein S31	57312	https://www.ncbi.nlm.nih.gov/gene/?term=57312
Efr3a	Efr3a	EFR3 homolog A	76740	https://www.ncbi.nlm.nih.gov/gene/?term=76740
Csrnp3	Csrnp3	cysteine-serine-rich nuclear protein 3	77771	https://www.ncbi.nlm.nih.gov/gene/?term=77771
Anxa10	Anxa10	annexin A10	26359	https://www.ncbi.nlm.nih.gov/gene/?term=26359
Oas3	Oas3	2'-5' oligoadenylate synthetase 3	246727	https://www.ncbi.nlm.nih.gov/gene/?term=246727
Zfp462	Zfp462	zinc finger protein 462	242466	https://www.ncbi.nlm.nih.gov/gene/?term=242466
Prkx	Prkx	protein kinase, X-linked	19108	https://www.ncbi.nlm.nih.gov/gene/?term=19108
Tmx1	Tmx1	thioredoxin-related transmembrane protein 1	72736	https://www.ncbi.nlm.nih.gov/gene/?term=72736
Lrpprc	Lrpprc	leucine-rich PPR-motif containing	72416	https://www.ncbi.nlm.nih.gov/gene/?term=72416
Itgb3	Itgb3	integrin beta 3	16416	https://www.ncbi.nlm.nih.gov/gene/?term=16416
Gga3	Gga3	golgi associated, gamma adaptin ear containing, ARF binding protein 3	260302	https://www.ncbi.nlm.nih.gov/gene/?term=260302
Lck	Lck	lymphocyte protein tyrosine kinase	16818	https://www.ncbi.nlm.nih.gov/gene/?term=16818
Hemgn	Hemgn	hemogen	93966	https://www.ncbi.nlm.nih.gov/gene/?term=93966
Ptprb	Ptprb	protein tyrosine phosphatase, receptor type, B	19263	https://www.ncbi.nlm.nih.gov/gene/?term=19263
B230219D22Rik	B230219D22Rik	RIKEN cDNA B230219D22 gene	78521	https://www.ncbi.nlm.nih.gov/gene/?term=78521
Parp14	Parp14	poly (ADP-ribose) polymerase family, member 14	547253	https://www.ncbi.nlm.nih.gov/gene/?term=547253
Eif4e	Eif4e	eukaryotic translation initiation factor 4E	13684	https://www.ncbi.nlm.nih.gov/gene/?term=13684
Marveld2	Marveld2	MARVEL (membrane-associating) domain containing 2	218518	https://www.ncbi.nlm.nih.gov/gene/?term=218518
Scrn1	Scrn1	secernin 1	69938	https://www.ncbi.nlm.nih.gov/gene/?term=69938

Biology Open • Supplementary information

Prdm16	Prdm16	PR domain containing 16	70673	https://www.ncbi.nlm.nih.gov/gene/?term=70673
Kcna2	Kcna2	potassium voltage-gated channel, shaker-related subfamily, member 2	16490	https://www.ncbi.nlm.nih.gov/gene/?term=16490
Cckar	Cckar	cholecystokinin A receptor	12425	https://www.ncbi.nlm.nih.gov/gene/?term=12425
Strn4	Strn4	striatin, calmodulin binding protein 4	97387	https://www.ncbi.nlm.nih.gov/gene/?term=97387
Rock2	Rock2	Rho-associated coiled-coil containing protein kinase 2	19878	https://www.ncbi.nlm.nih.gov/gene/?term=19878
Xlr	Xlr	X-linked lymphocyte-regulated	22441	https://www.ncbi.nlm.nih.gov/gene/?term=22441
Rad51c	Rad51c	RAD51 paralog C	114714	https://www.ncbi.nlm.nih.gov/gene/?term=114714
Smad3	Smad3	SMAD family member 3	17127	https://www.ncbi.nlm.nih.gov/gene/?term=17127
Cd274	Cd274	CD274 antigen	60533	https://www.ncbi.nlm.nih.gov/gene/?term=60533
1700028K03Rik	1700028K03Rik	RIKEN cDNA 1700028K03 gene	76421	https://www.ncbi.nlm.nih.gov/gene/?term=76421
Arhgap19	Arhgap19	Rho GTPase activating protein 19	71085	https://www.ncbi.nlm.nih.gov/gene/?term=71085
Foxk1	Foxk1	forkhead box K1	17425	https://www.ncbi.nlm.nih.gov/gene/?term=17425
Prkab2	Prkab2	protein kinase, AMP-activated, beta 2 non-catalytic subunit	108097	https://www.ncbi.nlm.nih.gov/gene/?term=108097
Pira2	Pira2	paired-Ig-like receptor A2	18725	https://www.ncbi.nlm.nih.gov/gene/?term=18725
Mip	Mip	major intrinsic protein of lens fiber	17339	https://www.ncbi.nlm.nih.gov/gene/?term=17339
Snrk	Snrk	SNF related kinase	20623	https://www.ncbi.nlm.nih.gov/gene/?term=20623
Stk38	Stk38	serine/threonine kinase 38	106504	https://www.ncbi.nlm.nih.gov/gene/?term=106504
Bpifc	Bpifc	BPI fold containing family C	270757	https://www.ncbi.nlm.nih.gov/gene/?term=270757
Cds2	Cds2	CDP-diacylglycerol synthase (phosphatidate cytidylyltransferase) 2	110911	https://www.ncbi.nlm.nih.gov/gene/?term=110911
Scube2	Scube2	signal peptide, CUB domain, EGF-like 2	56788	https://www.ncbi.nlm.nih.gov/gene/?term=56788

Biology Open • Supplementary information

Amer2	Amer2	APC membrane recruitment 2	72125	https://www.ncbi.nlm.nih.gov/gene/?term=72125
Il10ra	Il10ra	interleukin 10 receptor, alpha	16154	https://www.ncbi.nlm.nih.gov/gene/?term=16154
Sv2a	Sv2a	synaptic vesicle glycoprotein 2 a	64051	https://www.ncbi.nlm.nih.gov/gene/?term=64051
Smpdl3a	Smpdl3a	sphingomyelin phosphodiesterase, acid-like 3A	57319	https://www.ncbi.nlm.nih.gov/gene/?term=57319
Dhcr24	Dhcr24	24-dehydrocholesterol reductase	74754	https://www.ncbi.nlm.nih.gov/gene/?term=74754
2610301B20Rik	2610301B20Rik	RIKEN cDNA 2610301B20 gene	67157	https://www.ncbi.nlm.nih.gov/gene/?term=67157
Syk	Syk	spleen tyrosine kinase	20963	https://www.ncbi.nlm.nih.gov/gene/?term=20963
Ndrg3	Ndrg3	N-myc downstream regulated gene 3	29812	https://www.ncbi.nlm.nih.gov/gene/?term=29812
Xrn2	Xrn2	5'-3' exoribonuclease 2	24128	https://www.ncbi.nlm.nih.gov/gene/?term=24128
Lrp8	Lrp8	low density lipoprotein receptor-related protein 8, apolipoprotein e receptor	16975	https://www.ncbi.nlm.nih.gov/gene/?term=16975
Tnfrsf11a	Tnfrsf11a	tumor necrosis factor receptor superfamily, member 11a, NFKB activator	21934	https://www.ncbi.nlm.nih.gov/gene/?term=21934
Phc2	Phc2	polyhomeotic 2	54383	https://www.ncbi.nlm.nih.gov/gene/?term=54383
Cst6	Cst6	cystatin E/M	73720	https://www.ncbi.nlm.nih.gov/gene/?term=73720
Phf21a	Phf21a	PHD finger protein 21A	192285	https://www.ncbi.nlm.nih.gov/gene/?term=192285
R3hdm4	R3hdm4	R3H domain containing 4	109284	https://www.ncbi.nlm.nih.gov/gene/?term=109284
Gnl3l	Gnl3l	guanine nucleotide binding protein-like 3 (nucleolar)-like	237107	https://www.ncbi.nlm.nih.gov/gene/?term=237107
Zcchc18	Zcchc18	zinc finger, CCHC domain containing 18	66995	https://www.ncbi.nlm.nih.gov/gene/?term=66995
Kat7	Kat7	K(lysine) acetyltransferase 7	217127	https://www.ncbi.nlm.nih.gov/gene/?term=217127
Apool	Apool	apolipoprotein O-like	68117	https://www.ncbi.nlm.nih.gov/gene/?term=68117
Upp2	Upp2	uridine phosphorylase 2	76654	https://www.ncbi.nlm.nih.gov/gene/?term=76654

Biology Open • Supplementary information

Rnf44	Rnf44	ring finger protein 44	105239	https://www.ncbi.nlm.nih.gov/gene/?term=105239
Gabra2	Gabra2	gamma-aminobutyric acid (GABA) A receptor, subunit alpha 2	14395	https://www.ncbi.nlm.nih.gov/gene/?term=14395
Neurog2	Neurog2	neurogenin 2	11924	https://www.ncbi.nlm.nih.gov/gene/?term=11924
Zfp729b	Zfp729b	zinc finger protein 729b	100416706	https://www.ncbi.nlm.nih.gov/gene/?term=100416
Fsd1l	Fsd1l	fibronectin type III and SPRY domain containing 1-like	319636	https://www.ncbi.nlm.nih.gov/gene/?term=319636
Ppp1r21	Ppp1r21	protein phosphatase 1, regulatory subunit 21	73825	https://www.ncbi.nlm.nih.gov/gene/?term=73825
Tspan3	Tspan3	tetraspanin 3	56434	https://www.ncbi.nlm.nih.gov/gene/?term=56434
Pes1	Pes1	pescadillo ribosomal biogenesis factor 1	64934	https://www.ncbi.nlm.nih.gov/gene/?term=64934
Mis18a	Mis18a	MIS18 kinetochore protein A	66578	https://www.ncbi.nlm.nih.gov/gene/?term=66578
Tpbpa	Tpbpa	trophoblast specific protein alpha	21984	https://www.ncbi.nlm.nih.gov/gene/?term=21984
Lin52	Lin52	lin-52 homolog (C. elegans)	217708	https://www.ncbi.nlm.nih.gov/gene/?term=217708
Riok3	Riok3	RIO kinase 3	66878	https://www.ncbi.nlm.nih.gov/gene/?term=66878
Eme1	Eme1	essential meiotic structure-specific endonuclease 1	268465	https://www.ncbi.nlm.nih.gov/gene/?term=268465
Mei4	Mei4	meiotic double-stranded break formation protein 4	75033	https://www.ncbi.nlm.nih.gov/gene/?term=75033
Ubtd2	Ubtd2	ubiquitin domain containing 2	327900	https://www.ncbi.nlm.nih.gov/gene/?term=327900
Pdgfrl	Pdgfrl	platelet-derived growth factor receptor-like	68797	https://www.ncbi.nlm.nih.gov/gene/?term=68797
Nup35	Nup35	nucleoporin 35	69482	https://www.ncbi.nlm.nih.gov/gene/?term=69482
Map10	Map10	microtubule-associated protein 10	74393	https://www.ncbi.nlm.nih.gov/gene/?term=74393
Npm1	Npm1	nucleophosmin 1	18148	https://www.ncbi.nlm.nih.gov/gene/?term=18148
Zmynd11	Zmynd11	zinc finger, MYND domain containing 11	66505	https://www.ncbi.nlm.nih.gov/gene/?term=66505

Biology Open • Supplementary information

Ccdc169	Ccdc169	coiled-coil domain containing 169	320604	https://www.ncbi.nlm.nih.gov/gene/?term=320604
Six6	Six6	sine oculis-related homeobox 6	20476	https://www.ncbi.nlm.nih.gov/gene/?term=20476
Pfkfb2	Pfkfb2	6-phosphofructo-2-kinase/fructose-2,6-biphosphatase 2	18640	https://www.ncbi.nlm.nih.gov/gene/?term=18640
Psmc6	Psmc6	proteasome (prosome, macropain) 26S subunit, ATPase, 6	67089	https://www.ncbi.nlm.nih.gov/gene/?term=67089
Birc6	Birc6	baculoviral IAP repeat-containing 6	12211	https://www.ncbi.nlm.nih.gov/gene/?term=12211
Ms4a4b	Ms4a4b	membrane-spanning 4-domains, subfamily A, member 4B	60361	https://www.ncbi.nlm.nih.gov/gene/?term=60361
2310030G06Rik	2310030G06Rik	RIKEN cDNA 2310030G06 gene	66952	https://www.ncbi.nlm.nih.gov/gene/?term=66952
Tubgcp5	Tubgcp5	tubulin, gamma complex associated protein 5	233276	https://www.ncbi.nlm.nih.gov/gene/?term=233276
Olfr658	Olfr658	olfactory receptor 658	259051	https://www.ncbi.nlm.nih.gov/gene/?term=259051
Ikzf5	Ikzf5	IKAROS family zinc finger 5	67143	https://www.ncbi.nlm.nih.gov/gene/?term=67143
Zfp12	Zfp12	zinc finger protein 12	231866	https://www.ncbi.nlm.nih.gov/gene/?term=231866
Usf3	Usf3	upstream transcription factor family member 3	207806	https://www.ncbi.nlm.nih.gov/gene/?term=207806
Lad1	Lad1	ladinin	16763	https://www.ncbi.nlm.nih.gov/gene/?term=16763
Rgl1	Rgl1	ral guanine nucleotide dissociation stimulator,-like 1	19731	https://www.ncbi.nlm.nih.gov/gene/?term=19731
Wrn	Wrn	Werner syndrome RecQ like helicase	22427	https://www.ncbi.nlm.nih.gov/gene/?term=22427
Sh3bgrl3	Sh3bgrl3	SH3 domain binding glutamic acid-rich protein-like 3	73723	https://www.ncbi.nlm.nih.gov/gene/?term=73723
Spata1	Spata1	spermatogenesis associated 1	70951	https://www.ncbi.nlm.nih.gov/gene/?term=70951
Btbd6	Btbd6	BTB (POZ) domain containing 6	399566	https://www.ncbi.nlm.nih.gov/gene/?term=399566
Sap18b	Sap18b	Sin3-associated polypeptide 18B	100041953	https://www.ncbi.nlm.nih.gov/gene/?term=100041
Hcar2	Hcar2	hydroxycarboxylic acid receptor 2	80885	https://www.ncbi.nlm.nih.gov/gene/?term=80885

Biology Open • Supplementary information

Hspb6	Hspb6	heat shock protein, alpha-crystallin-related, B6	243912	https://www.ncbi.nlm.nih.gov/gene/?term=243912
Prkab1	Prkab1	protein kinase, AMP-activated, beta 1 non-catalytic subunit	19079	https://www.ncbi.nlm.nih.gov/gene/?term=19079
Ndst1	Ndst1	N-deacetylase/N-sulfotransferase (heparan glucosaminyl) 1	15531	https://www.ncbi.nlm.nih.gov/gene/?term=15531
Polr3f	Polr3f	polymerase (RNA) III (DNA directed) polypeptide F	70408	https://www.ncbi.nlm.nih.gov/gene/?term=70408
Gpr137b	Gpr137b	G protein-coupled receptor 137B	83924	https://www.ncbi.nlm.nih.gov/gene/?term=83924
Lbh	Lbh	limb-bud and heart	77889	https://www.ncbi.nlm.nih.gov/gene/?term=77889
Nrp2	Nrp2	neuropilin 2	18187	https://www.ncbi.nlm.nih.gov/gene/?term=18187
Pax1	Pax1	paired box 1	18503	https://www.ncbi.nlm.nih.gov/gene/?term=18503
Lgi2	Lgi2	leucine-rich repeat LGI family, member 2	246316	https://www.ncbi.nlm.nih.gov/gene/?term=246316
Ppp3cc	Ppp3cc	protein phosphatase 3, catalytic subunit, gamma isoform	19057	https://www.ncbi.nlm.nih.gov/gene/?term=19057
Ccz1	Ccz1	CCZ1 vacuolar protein trafficking and biogenesis associated	231874	https://www.ncbi.nlm.nih.gov/gene/?term=231874
Atp5c1	Atp5c1	ATP synthase, H+ transporting, mitochondrial F1 complex, gamma polypeptide 1	11949	https://www.ncbi.nlm.nih.gov/gene/?term=11949
Vsnl1	Vsnl1	visinin-like 1	26950	https://www.ncbi.nlm.nih.gov/gene/?term=26950
Rpf1	Rpf1	ribosome production factor 1 homolog	70285	https://www.ncbi.nlm.nih.gov/gene/?term=70285
Tmem131	Tmem131	transmembrane protein 131	56030	https://www.ncbi.nlm.nih.gov/gene/?term=56030
Kbtbd6	Kbtbd6	kelch repeat and BTB (POZ) domain containing 6	432879	https://www.ncbi.nlm.nih.gov/gene/?term=432879
Top1	Top1	topoisomerase (DNA) I	21969	https://www.ncbi.nlm.nih.gov/gene/?term=21969

Biology Open • Supplementary information

Trim59	Trim59	tripartite motif-containing 59	66949	https://www.ncbi.nlm.nih.gov/gene/?term=66949
Trpc3	Trpc3	transient receptor potential cation channel, subfamily C, member 3	22065	https://www.ncbi.nlm.nih.gov/gene/?term=22065
Pla2g4e	Pla2g4e	phospholipase A2, group IVE	329502	https://www.ncbi.nlm.nih.gov/gene/?term=329502
Bmp2k	Bmp2k	BMP2 inducible kinase	140780	https://www.ncbi.nlm.nih.gov/gene/?term=140780
Dab2ip	Dab2ip	disabled 2 interacting protein	69601	https://www.ncbi.nlm.nih.gov/gene/?term=69601
Snx6	Snx6	sorting nexin 6	72183	https://www.ncbi.nlm.nih.gov/gene/?term=72183
Cdkl1	Cdkl1	cyclin-dependent kinase-like 1 (CDC2-related kinase)	71091	https://www.ncbi.nlm.nih.gov/gene/?term=71091
Fgd4	Fgd4	FYVE, RhoGEF and PH domain containing 4	224014	https://www.ncbi.nlm.nih.gov/gene/?term=224014
St8sia5	St8sia5	ST8 alpha-N-acetyl-neuraminide alpha-2,8-sialyltransferase 5	225742	https://www.ncbi.nlm.nih.gov/gene/?term=225742
Usp5	Usp5	ubiquitin specific peptidase 5 (isopeptidase T)	22225	https://www.ncbi.nlm.nih.gov/gene/?term=22225
Baiap2	Baiap2	brain-specific angiogenesis inhibitor 1-associated protein 2	108100	https://www.ncbi.nlm.nih.gov/gene/?term=108100
Zbtb2	Zbtb2	zinc finger and BTB domain containing 2	381990	https://www.ncbi.nlm.nih.gov/gene/?term=381990
AW146154	AW146154	expressed sequence AW146154	101835	https://www.ncbi.nlm.nih.gov/gene/?term=101835
Tspoap1	Tspoap1	TSPO associated protein 1	207777	https://www.ncbi.nlm.nih.gov/gene/?term=207777
Atp6v0d2	Atp6v0d2	ATPase, H+ transporting, lysosomal V0 subunit D2	242341	https://www.ncbi.nlm.nih.gov/gene/?term=242341
Ccar2	Ccar2	cell cycle activator and apoptosis regulator 2	219158	https://www.ncbi.nlm.nih.gov/gene/?term=219158
Alpi	Alpi	alkaline phosphatase, intestinal	76768	https://www.ncbi.nlm.nih.gov/gene/?term=76768
Mab21l2	Mab21l2	mab-21-like 2 (C. elegans)	23937	https://www.ncbi.nlm.nih.gov/gene/?term=23937
Scfd2	Scfd2	Sec1 family domain containing 2	212986	https://www.ncbi.nlm.nih.gov/gene/?term=212986

Biology Open • Supplementary information

Epb41l1	Epb41l1	erythrocyte membrane protein band 4.1 like 1	13821	https://www.ncbi.nlm.nih.gov/gene/?term=13821
Rnf185	Rnf185	ring finger protein 185	193670	https://www.ncbi.nlm.nih.gov/gene/?term=193670
Ralgapa2	Ralgapa2	Ral GTPase activating protein, alpha subunit 2 (catalytic)	241694	https://www.ncbi.nlm.nih.gov/gene/?term=241694
Fgf11	Fgf11	fibroblast growth factor 11	14166	https://www.ncbi.nlm.nih.gov/gene/?term=14166
Ubxn10	Ubxn10	UBX domain protein 10	212190	https://www.ncbi.nlm.nih.gov/gene/?term=212190
Clcn4	Clcn4	chloride channel, voltage-sensitive 4	12727	https://www.ncbi.nlm.nih.gov/gene/?term=12727
Ggt7	Ggt7	gamma-glutamyltransferase 7	207182	https://www.ncbi.nlm.nih.gov/gene/?term=207182
Metap1	Metap1	methionyl aminopeptidase 1	75624	https://www.ncbi.nlm.nih.gov/gene/?term=75624
Dag1	Dag1	dystroglycan 1	13138	https://www.ncbi.nlm.nih.gov/gene/?term=13138
Sh2d1b1	Sh2d1b1	SH2 domain containing 1B1	26904	https://www.ncbi.nlm.nih.gov/gene/?term=26904
Gpx7	Gpx7	glutathione peroxidase 7	67305	https://www.ncbi.nlm.nih.gov/gene/?term=67305
Kif9	Kif9	kinesin family member 9	16578	https://www.ncbi.nlm.nih.gov/gene/?term=16578
Emilin3	Emilin3	elastin microfibril interfacer 3	280635	https://www.ncbi.nlm.nih.gov/gene/?term=280635
Parva	Parva	parvin, alpha	57342	https://www.ncbi.nlm.nih.gov/gene/?term=57342
Tspan18	Tspan18	tetraspanin 18	241556	https://www.ncbi.nlm.nih.gov/gene/?term=241556
Ppp1r3f	Ppp1r3f	protein phosphatase 1, regulatory subunit 3F	54646	https://www.ncbi.nlm.nih.gov/gene/?term=54646
Alad	Alad	aminolevulinate, delta-, dehydratase	17025	https://www.ncbi.nlm.nih.gov/gene/?term=17025
Ubiad1	Ubiad1	UbiA prenyltransferase domain containing 1	71707	https://www.ncbi.nlm.nih.gov/gene/?term=71707
Ccdc127	Ccdc127	coiled-coil domain containing 127	67433	https://www.ncbi.nlm.nih.gov/gene/?term=67433
Grpel2	Grpel2	GrpE-like 2, mitochondrial	17714	https://www.ncbi.nlm.nih.gov/gene/?term=17714
Tor4a	Tor4a	torsin family 4, member A	227612	https://www.ncbi.nlm.nih.gov/gene/?term=227612
Hip1	Hip1	huntingtin interacting protein 1	215114	https://www.ncbi.nlm.nih.gov/gene/?term=215114

Chrm1	Chrm1	cholinergic receptor, muscarinic 1, CNS	12669	https://www.ncbi.nlm.nih.gov/gene/?term=12669
Soga1	Soga1	suppressor of glucose, autophagy associated 1	320706	https://www.ncbi.nlm.nih.gov/gene/?term=320706
Palm	Palm	paralemmin	18483	https://www.ncbi.nlm.nih.gov/gene/?term=18483
Vldlr	Vldlr	very low density lipoprotein receptor	22359	https://www.ncbi.nlm.nih.gov/gene/?term=22359
Glod5	Glod5	glyoxalase domain containing 5	69824	https://www.ncbi.nlm.nih.gov/gene/?term=69824
Lrrc3	Lrrc3	leucine rich repeat containing 3	237387	https://www.ncbi.nlm.nih.gov/gene/?term=237387
Srsf3	Srsf3	serine/arginine-rich splicing factor 3	20383	https://www.ncbi.nlm.nih.gov/gene/?term=20383
Amotl1	Amotl1	angiomotin-like 1	75723	https://www.ncbi.nlm.nih.gov/gene/?term=75723
Exd2	Exd2	exonuclease 3'-5' domain containing 2	97827	https://www.ncbi.nlm.nih.gov/gene/?term=97827
Nnat	Nnat	neuronatin	18111	https://www.ncbi.nlm.nih.gov/gene/?term=18111
Tbc1d22a	Tbc1d22a	TBC1 domain family, member 22a	223754	https://www.ncbi.nlm.nih.gov/gene/?term=223754
Bmper	Bmper	BMP-binding endothelial regulator	73230	https://www.ncbi.nlm.nih.gov/gene/?term=73230
Dlgap1	Dlgap1	DLG associated protein 1	224997	https://www.ncbi.nlm.nih.gov/gene/?term=224997
Baz1a	Baz1a	bromodomain adjacent to zinc finger domain 1A	217578	https://www.ncbi.nlm.nih.gov/gene/?term=217578
Tarsl2	Tarsl2	threonyl-tRNA synthetase-like 2	272396	https://www.ncbi.nlm.nih.gov/gene/?term=272396
Ptprk	Ptprk	protein tyrosine phosphatase, receptor type, K	19272	https://www.ncbi.nlm.nih.gov/gene/?term=19272
Hps6	Hps6	HPS6, biogenesis of lysosomal organelles complex 2 subunit 3	20170	https://www.ncbi.nlm.nih.gov/gene/?term=20170
Nphp1	Nphp1	nephronophthisis 1 (juvenile) homolog (human)	53885	https://www.ncbi.nlm.nih.gov/gene/?term=53885
Fzd10	Fzd10	frizzled class receptor 10	93897	https://www.ncbi.nlm.nih.gov/gene/?term=93897

Biology Open • Supplementary information

Psd4	Psd4	pleckstrin and Sec7 domain containing 4	215632	https://www.ncbi.nlm.nih.gov/gene/?term=215632
Chic2	Chic2	cysteine-rich hydrophobic domain 2	74277	https://www.ncbi.nlm.nih.gov/gene/?term=74277
Pnma2	Pnma2	paraneoplastic antigen MA2	239157	https://www.ncbi.nlm.nih.gov/gene/?term=239157
Gm5544	Gm5544	predicted gene 5544	433632	https://www.ncbi.nlm.nih.gov/gene/?term=433632
Pde9a	Pde9a	phosphodiesterase 9A	18585	https://www.ncbi.nlm.nih.gov/gene/?term=18585
Ccnl2	Ccnl2	cyclin L2	56036	https://www.ncbi.nlm.nih.gov/gene/?term=56036
Med13l	Med13l	mediator complex subunit 13-like	76199	https://www.ncbi.nlm.nih.gov/gene/?term=76199
Zmat4	Zmat4	zinc finger, matrin type 4	320158	https://www.ncbi.nlm.nih.gov/gene/?term=320158
Xk	Xk	X-linked Kx blood group	22439	https://www.ncbi.nlm.nih.gov/gene/?term=22439
Cfap70	Cfap70	cilia and flagella associated protein 70	76670	https://www.ncbi.nlm.nih.gov/gene/?term=76670
Patz1	Patz1	POZ (BTB) and AT hook containing zinc finger 1	56218	https://www.ncbi.nlm.nih.gov/gene/?term=56218
Scn3b	Scn3b	sodium channel, voltage-gated, type III, beta	235281	https://www.ncbi.nlm.nih.gov/gene/?term=235281
Zfp712	Zfp712	zinc finger protein 712	78251	https://www.ncbi.nlm.nih.gov/gene/?term=78251
Dazap1	Dazap1	DAZ associated protein 1	70248	https://www.ncbi.nlm.nih.gov/gene/?term=70248
Rbms3	Rbms3	RNA binding motif, single stranded interacting protein	207181	https://www.ncbi.nlm.nih.gov/gene/?term=207181
Csk	Csk	c-src tyrosine kinase	12988	https://www.ncbi.nlm.nih.gov/gene/?term=12988
Fasl	Fasl	Fas ligand (TNF superfamily, member 6)	14103	https://www.ncbi.nlm.nih.gov/gene/?term=14103
Rab40b	Rab40b	Rab40B, member RAS oncogene family	217371	https://www.ncbi.nlm.nih.gov/gene/?term=217371
Rgs13	Rgs13	regulator of G-protein signaling 13	246709	https://www.ncbi.nlm.nih.gov/gene/?term=246709

Biology Open • Supplementary information

Spink5	Spink5	serine peptidase inhibitor, Kazal type 5	72432	https://www.ncbi.nlm.nih.gov/gene/?term=72432
Pcdhb4	Pcdhb4	protocadherin beta 4	93875	https://www.ncbi.nlm.nih.gov/gene/?term=93875
Jak2	Jak2	Janus kinase 2	16452	https://www.ncbi.nlm.nih.gov/gene/?term=16452
Tmem170	Tmem170	transmembrane protein 170	66817	https://www.ncbi.nlm.nih.gov/gene/?term=66817
Chmp1b	Chmp1b	charged multivesicular body protein 1B	67064	https://www.ncbi.nlm.nih.gov/gene/?term=67064
Mysm1	Mysm1	myb-like, SWIRM and MPN domains 1	320713	https://www.ncbi.nlm.nih.gov/gene/?term=320713
Tmem255a	Tmem255a	transmembrane protein 255A	245386	https://www.ncbi.nlm.nih.gov/gene/?term=245386
Sde2	Sde2	SDE2 telomere maintenance homolog (S. pombe)	208768	https://www.ncbi.nlm.nih.gov/gene/?term=208768
Lmo4	Lmo4	LIM domain only 4	16911	https://www.ncbi.nlm.nih.gov/gene/?term=16911
Ostn	Ostn	osteocrin	239790	https://www.ncbi.nlm.nih.gov/gene/?term=239790
Zfp189	Zfp189	zinc finger protein 189	230162	https://www.ncbi.nlm.nih.gov/gene/?term=230162
Fam120a	Fam120a	family with sequence similarity 120, member A	218236	https://www.ncbi.nlm.nih.gov/gene/?term=218236
Kansl1	Kansl1	KAT8 regulatory NSL complex subunit 1	76719	https://www.ncbi.nlm.nih.gov/gene/?term=76719
Nfkbiz	Nfkbiz	nuclear factor of kappa light polypeptide gene enhancer in B cells inhibitor, zeta	80859	https://www.ncbi.nlm.nih.gov/gene/?term=80859
Tmf1	Tmf1	TATA element modulatory factor 1	232286	https://www.ncbi.nlm.nih.gov/gene/?term=232286
Whrn	Whrn	whirlin	73750	https://www.ncbi.nlm.nih.gov/gene/?term=73750
Thoc2	Thoc2	THO complex 2	331401	https://www.ncbi.nlm.nih.gov/gene/?term=331401
BC024978	BC024978	cDNA sequence BC024978	414069	https://www.ncbi.nlm.nih.gov/gene/?term=414069
Fchsd2	Fchsd2	FCH and double SH3 domains 2	207278	https://www.ncbi.nlm.nih.gov/gene/?term=207278
Trmt2a	Trmt2a	TRM2 tRNA methyltransferase 2A	15547	https://www.ncbi.nlm.nih.gov/gene/?term=15547

Biology Open • Supplementary information

Prelid3a	Prelid3a	PRELI domain containing 3A	225655	https://www.ncbi.nlm.nih.gov/gene/?term=225655
Utp23	Utp23	UTP23 small subunit processome component	78581	https://www.ncbi.nlm.nih.gov/gene/?term=78581
Soga3	Soga3	SOGA family member 3	67412	https://www.ncbi.nlm.nih.gov/gene/?term=67412
Kpnb1	Kpnb1	karyopherin (importin) beta 1	16211	https://www.ncbi.nlm.nih.gov/gene/?term=16211
Nras	Nras	neuroblastoma ras oncogene	18176	https://www.ncbi.nlm.nih.gov/gene/?term=18176
Fam214a	Fam214a	family with sequence similarity 214, member A	235493	https://www.ncbi.nlm.nih.gov/gene/?term=235493
Zbtb11	Zbtb11	zinc finger and BTB domain containing 11	271377	https://www.ncbi.nlm.nih.gov/gene/?term=271377
Ppp2r2c	Ppp2r2c	protein phosphatase 2, regulatory subunit B, gamma	269643	https://www.ncbi.nlm.nih.gov/gene/?term=269643
Ric3	Ric3	RIC3 acetylcholine receptor chaperone	320360	https://www.ncbi.nlm.nih.gov/gene/?term=320360
Msl2	Msl2	MSL complex subunit 2	77853	https://www.ncbi.nlm.nih.gov/gene/?term=77853
Snrnp70	Snrnp70	small nuclear ribonucleoprotein 70 (U1)	20637	https://www.ncbi.nlm.nih.gov/gene/?term=20637
Pipox	Pipox	pipecolic acid oxidase	19193	https://www.ncbi.nlm.nih.gov/gene/?term=19193
Gprc5a	Gprc5a	G protein-coupled receptor, family C, group 5, member A	232431	https://www.ncbi.nlm.nih.gov/gene/?term=232431
Elmo2	Elmo2	engulfment and cell motility 2	140579	https://www.ncbi.nlm.nih.gov/gene/?term=140579
Ubfd1	Ubfd1	ubiquitin family domain containing 1	28018	https://www.ncbi.nlm.nih.gov/gene/?term=28018
Ttyh2	Ttyh2	tweety family member 2	117160	https://www.ncbi.nlm.nih.gov/gene/?term=117160
Cops7a	Cops7a	COP9 signalosome subunit 7A	26894	https://www.ncbi.nlm.nih.gov/gene/?term=26894
Ralbp1	Ralbp1	ralA binding protein 1	19765	https://www.ncbi.nlm.nih.gov/gene/?term=19765
Tfap2b	Tfap2b	transcription factor AP-2 beta	21419	https://www.ncbi.nlm.nih.gov/gene/?term=21419
Abraxas1	Abraxas1	BRCA1 A complex subunit	70681	https://www.ncbi.nlm.nih.gov/gene/?term=70681

Nudt4	Nudt4	nudix (nucleoside diphosphate linked moiety X)-type motif 4	71207	https://www.ncbi.nlm.nih.gov/gene/?term=71207
Pttg1ip	Pttg1ip	pituitary tumor-transforming 1 interacting protein	108705	https://www.ncbi.nlm.nih.gov/gene/?term=108705
Cers5	Cers5	ceramide synthase 5	71949	https://www.ncbi.nlm.nih.gov/gene/?term=71949
Col23a1	Col23a1	collagen, type XXIII, alpha 1	237759	https://www.ncbi.nlm.nih.gov/gene/?term=237759
Ntan1	Ntan1	N-terminal Asn amidase	18203	https://www.ncbi.nlm.nih.gov/gene/?term=18203
Ly6g5b	Ly6g5b	lymphocyte antigen 6 complex, locus G5B	266614	https://www.ncbi.nlm.nih.gov/gene/?term=266614
Trpm3	Trpm3	transient receptor potential cation channel, subfamily M, member 3	226025	https://www.ncbi.nlm.nih.gov/gene/?term=226025
Msrb2	Msrb2	methionine sulfoxide reductase B2	76467	https://www.ncbi.nlm.nih.gov/gene/?term=76467
Psmd12	Psmd12	proteasome (prosome, macropain) 26S subunit, non-ATPase, 12	66997	https://www.ncbi.nlm.nih.gov/gene/?term=66997
Plpp7	Plpp7	phospholipid phosphatase 7 (inactive)	227721	https://www.ncbi.nlm.nih.gov/gene/?term=227721
Slc16a5	Slc16a5	solute carrier family 16 (monocarboxylic acid transporters), member 5	217316	https://www.ncbi.nlm.nih.gov/gene/?term=217316
Serpini1	Serpini1	serine (or cysteine) peptidase inhibitor, clade I, member 1	20713	https://www.ncbi.nlm.nih.gov/gene/?term=20713
Fbxo28	Fbxo28	F-box protein 28	67948	https://www.ncbi.nlm.nih.gov/gene/?term=67948
Bub1	Bub1	BUB1, mitotic checkpoint serine/threonine kinase	12235	https://www.ncbi.nlm.nih.gov/gene/?term=12235
Scoc	Scoc	short coiled-coil protein	56367	https://www.ncbi.nlm.nih.gov/gene/?term=56367
Atp6v1d	Atp6v1d	ATPase, H+ transporting, lysosomal V1 subunit D	73834	https://www.ncbi.nlm.nih.gov/gene/?term=73834

Biology Open • Supplementary information

Nln	Nln	neurolysin (metallopeptidase M3 family)	75805	https://www.ncbi.nlm.nih.gov/gene/?term=75805
Klhl4	Klhl4	kelch-like 4	237010	https://www.ncbi.nlm.nih.gov/gene/?term=237010
Stk32b	Stk32b	serine/threonine kinase 32B	64293	https://www.ncbi.nlm.nih.gov/gene/?term=64293
Ctsc	Ctsc	cathepsin C	13032	https://www.ncbi.nlm.nih.gov/gene/?term=13032
Faap20	Faap20	Fanconi anemia core complex associated protein 20	67513	https://www.ncbi.nlm.nih.gov/gene/?term=67513
Cbx3	Cbx3	chromobox 3	12417	https://www.ncbi.nlm.nih.gov/gene/?term=12417
Fkbp1b	Fkbp1b	FK506 binding protein 1b	14226	https://www.ncbi.nlm.nih.gov/gene/?term=14226
Cnih4	Cnih4	cornichon family AMPA receptor auxiliary protein 4	98417	https://www.ncbi.nlm.nih.gov/gene/?term=98417
Iffo2	Iffo2	intermediate filament family orphan 2	212632	https://www.ncbi.nlm.nih.gov/gene/?term=212632
Dll1	Dll1	delta like canonical Notch ligand 1	13388	https://www.ncbi.nlm.nih.gov/gene/?term=13388
Arel1	Arel1	apoptosis resistant E3 ubiquitin protein ligase 1	68497	https://www.ncbi.nlm.nih.gov/gene/?term=68497
Eloa	Eloa	elongin A	27224	https://www.ncbi.nlm.nih.gov/gene/?term=27224
Igf2bp1	Igf2bp1	insulin-like growth factor 2 mRNA binding protein 1	140486	https://www.ncbi.nlm.nih.gov/gene/?term=140486
Fbxo41	Fbxo41	F-box protein 41	330369	https://www.ncbi.nlm.nih.gov/gene/?term=330369
Lsm14b	Lsm14b	LSM family member 14B	241846	https://www.ncbi.nlm.nih.gov/gene/?term=241846
Pkp2	Pkp2	plakophilin 2	67451	https://www.ncbi.nlm.nih.gov/gene/?term=67451
Tnks	Tnks	tankyrase, TRF1-interacting ankyrin-related ADP-ribose polymerase	21951	https://www.ncbi.nlm.nih.gov/gene/?term=21951
Olfr121	Olfr121	olfactory receptor 121	258622	https://www.ncbi.nlm.nih.gov/gene/?term=258622
Phldb1	Phldb1	pleckstrin homology like domain, family B, member 1	102693	https://www.ncbi.nlm.nih.gov/gene/?term=102693
Amotl2	Amotl2	angiomotin-like 2	56332	https://www.ncbi.nlm.nih.gov/gene/?term=56332

Dnm3	Dnm3	dynamin 3	103967	https://www.ncbi.nlm.nih.gov/gene/?term=103967
Zfp958	Zfp958	zinc finger protein 958	233987	https://www.ncbi.nlm.nih.gov/gene/?term=233987
Fbxl16	Fbxl16	F-box and leucine-rich repeat protein 16	214931	https://www.ncbi.nlm.nih.gov/gene/?term=214931
Map4k5	Map4k5	mitogen-activated protein kinase kinase kinase kinase 5	399510	https://www.ncbi.nlm.nih.gov/gene/?term=399510
Appl2	Appl2	adaptor protein, phosphotyrosine interaction, PH domain and leucine zipper containing 2	216190	https://www.ncbi.nlm.nih.gov/gene/?term=216190
Grk2	Grk2	G protein-coupled receptor kinase 2	110355	https://www.ncbi.nlm.nih.gov/gene/?term=110355
Fkbp1a	Fkbp1a	FK506 binding protein 1a	14225	https://www.ncbi.nlm.nih.gov/gene/?term=14225
Lonrf1	Lonrf1	LON peptidase N-terminal domain and ring finger 1	244421	https://www.ncbi.nlm.nih.gov/gene/?term=244421
Rbm27	Rbm27	RNA binding motif protein 27	225432	https://www.ncbi.nlm.nih.gov/gene/?term=225432
Arhgef10l	Arhgef10l	Rho guanine nucleotide exchange factor (GEF) 10-like	72754	https://www.ncbi.nlm.nih.gov/gene/?term=72754
Mapk8	Mapk8	mitogen-activated protein kinase 8	26419	https://www.ncbi.nlm.nih.gov/gene/?term=26419
Fam172a	Fam172a	family with sequence similarity 172, member A	68675	https://www.ncbi.nlm.nih.gov/gene/?term=68675
Ptpn11	Ptpn11	protein tyrosine phosphatase, non-receptor type 11	19247	https://www.ncbi.nlm.nih.gov/gene/?term=19247
Nfkb2	Nfkb2	nuclear factor of kappa light polypeptide gene enhancer in B cells 2, p49/p100	18034	https://www.ncbi.nlm.nih.gov/gene/?term=18034
5730507C01Rik	5730507C01Rik	RIKEN cDNA 5730507C01 gene	236366	https://www.ncbi.nlm.nih.gov/gene/?term=236366
Trib1	Trib1	tribbles pseudokinase 1	211770	https://www.ncbi.nlm.nih.gov/gene/?term=211770

Biology Open • Supplementary information

Fam71f2	Fam71f2	family with sequence similarity 71, member F2	245884	https://www.ncbi.nlm.nih.gov/gene/?term=245884
Sema6a	Sema6a	sema domain, transmembrane domain (TM), and cytoplasmic domain, (semaphorin) 6A	20358	https://www.ncbi.nlm.nih.gov/gene/?term=20358
Vmn1r56	Vmn1r56	vomeronasal 1 receptor 56	81015	https://www.ncbi.nlm.nih.gov/gene/?term=81015
Bdh1	Bdh1	3-hydroxybutyrate dehydrogenase, type 1	71911	https://www.ncbi.nlm.nih.gov/gene/?term=71911
Zdhhc14	Zdhhc14	zinc finger, DHHC domain containing 14	224454	https://www.ncbi.nlm.nih.gov/gene/?term=224454
Efnb3	Efnb3	ephrin B3	13643	https://www.ncbi.nlm.nih.gov/gene/?term=13643
Tmem129	Tmem129	transmembrane protein 129	68366	https://www.ncbi.nlm.nih.gov/gene/?term=68366
Myef2	Myef2	myelin basic protein expression factor 2, repressor	17876	https://www.ncbi.nlm.nih.gov/gene/?term=17876
Csgalnact2	Csgalnact2	chondroitin sulfate N-acetylgalactosaminyltransferase 2	78752	https://www.ncbi.nlm.nih.gov/gene/?term=78752
Osbpl5	Osbpl5	oxysterol binding protein-like 5	79196	https://www.ncbi.nlm.nih.gov/gene/?term=79196
Rras2	Rras2	related RAS viral (r-ras) oncogene 2	66922	https://www.ncbi.nlm.nih.gov/gene/?term=66922
Acaca	Acaca	acetyl-Coenzyme A carboxylase alpha	107476	https://www.ncbi.nlm.nih.gov/gene/?term=107476
Drd1	Drd1	dopamine receptor D1	13488	https://www.ncbi.nlm.nih.gov/gene/?term=13488
Per2	Per2	period circadian clock 2	18627	https://www.ncbi.nlm.nih.gov/gene/?term=18627
Spred2	Spred2	sprouty-related, EVH1 domain containing 2	114716	https://www.ncbi.nlm.nih.gov/gene/?term=114716
Vgll3	Vgll3	vestigial like family member 3	73569	https://www.ncbi.nlm.nih.gov/gene/?term=73569
Kdm1b	Kdm1b	lysine (K)-specific demethylase 1B	218214	https://www.ncbi.nlm.nih.gov/gene/?term=218214
Dyrk2	Dyrk2	dual-specificity tyrosine-(Y)-phosphorylation regulated kinase 2	69181	https://www.ncbi.nlm.nih.gov/gene/?term=69181

Biology Open • Supplementary information

Setd7	Setd7	SET domain containing (lysine methyltransferase) 7	73251	https://www.ncbi.nlm.nih.gov/gene/?term=73251
Lancl3	Lancl3	LanC lantibiotic synthetase component C-like 3 (bacterial)	236285	https://www.ncbi.nlm.nih.gov/gene/?term=236285
Entpd7	Entpd7	ectonucleoside triphosphate diphosphohydrolase 7	93685	https://www.ncbi.nlm.nih.gov/gene/?term=93685
Gabbr2	Gabbr2	gamma-aminobutyric acid (GABA) B receptor, 2	242425	https://www.ncbi.nlm.nih.gov/gene/?term=242425
Arhgap32	Arhgap32	Rho GTPase activating protein 32	330914	https://www.ncbi.nlm.nih.gov/gene/?term=330914
Ntsr1	Ntsr1	neurotensin receptor 1	18216	https://www.ncbi.nlm.nih.gov/gene/?term=18216
Med26	Med26	mediator complex subunit 26	70625	https://www.ncbi.nlm.nih.gov/gene/?term=70625
Zc3h10	Zc3h10	zinc finger CCCH type containing 10	103284	https://www.ncbi.nlm.nih.gov/gene/?term=103284
Shh	Shh	sonic hedgehog	20423	https://www.ncbi.nlm.nih.gov/gene/?term=20423
Wtap	Wtap	Wilms tumour 1-associating protein	60532	https://www.ncbi.nlm.nih.gov/gene/?term=60532
Ccn4	Ccn4	cellular communication network factor 4	22402	https://www.ncbi.nlm.nih.gov/gene/?term=22402
Fscn1	Fscn1	fascin actin-bundling protein 1	14086	https://www.ncbi.nlm.nih.gov/gene/?term=14086
Yod1	Yod1	YOD1 deubiquitinase	226418	https://www.ncbi.nlm.nih.gov/gene/?term=226418
Fanci	Fanci	Fanconi anemia, complementation group I	208836	https://www.ncbi.nlm.nih.gov/gene/?term=208836
Agk	Agk	acylglycerol kinase	69923	https://www.ncbi.nlm.nih.gov/gene/?term=69923
1110004F10Rik	1110004F10Rik	RIKEN cDNA 1110004F10 gene	56372	https://www.ncbi.nlm.nih.gov/gene/?term=56372
Pou4f1	Pou4f1	POU domain, class 4, transcription factor 1	18996	https://www.ncbi.nlm.nih.gov/gene/?term=18996
Zfp37	Zfp37	zinc finger protein 37	22696	https://www.ncbi.nlm.nih.gov/gene/?term=22696
Ugt8a	Ugt8a	UDP galactosyltransferase 8A	22239	https://www.ncbi.nlm.nih.gov/gene/?term=22239

Biology Open • Supplementary information

Sema3d	Sema3d	sema domain, immunoglobulin domain (Ig), short basic domain, secreted, (semaphorin) 3D	108151	https://www.ncbi.nlm.nih.gov/gene/?term=108151
Kif20a	Kif20a	kinesin family member 20A	19348	https://www.ncbi.nlm.nih.gov/gene/?term=19348
Tle4	Tle4	transducin-like enhancer of split 4	21888	https://www.ncbi.nlm.nih.gov/gene/?term=21888
Tbx4	Tbx4	T-box 4	21387	https://www.ncbi.nlm.nih.gov/gene/?term=21387
Fzd5	Fzd5	frizzled class receptor 5	14367	https://www.ncbi.nlm.nih.gov/gene/?term=14367
Csn2	Csn2	casein beta	12991	https://www.ncbi.nlm.nih.gov/gene/?term=12991
Cemip	Cemip	cell migration inducing protein, hyaluronan binding	80982	https://www.ncbi.nlm.nih.gov/gene/?term=80982
Nipbl	Nipbl	NIPBL cohesin loading factor	71175	https://www.ncbi.nlm.nih.gov/gene/?term=71175
Myo9a	Myo9a	myosin IXa	270163	https://www.ncbi.nlm.nih.gov/gene/?term=270163
Ltbp1	Ltbp1	latent transforming growth factor beta binding protein 1	268977	https://www.ncbi.nlm.nih.gov/gene/?term=268977
Srpk2	Srpk2	serine/arginine-rich protein specific kinase 2	20817	https://www.ncbi.nlm.nih.gov/gene/?term=20817
Pikfyve	Pikfyve	phosphoinositide kinase, FYVE type zinc finger containing	18711	https://www.ncbi.nlm.nih.gov/gene/?term=18711
N6amt1	N6amt1	N-6 adenine-specific DNA methyltransferase 1 (putative)	67768	https://www.ncbi.nlm.nih.gov/gene/?term=67768
Cebpb	Cebpb	CCAAT/enhancer binding protein (C/EBP), beta	12608	https://www.ncbi.nlm.nih.gov/gene/?term=12608
Numbl	Numbl	numb-like	18223	https://www.ncbi.nlm.nih.gov/gene/?term=18223
Parp8	Parp8	poly (ADP-ribose) polymerase family, member 8	52552	https://www.ncbi.nlm.nih.gov/gene/?term=52552
Fancf	Fancf	Fanconi anemia, complementation group F	100040608	https://www.ncbi.nlm.nih.gov/gene/?term=100040[illegible]
Tead3	Tead3	TEA domain family member 3	21678	https://www.ncbi.nlm.nih.gov/gene/?term=21678

Biology Open • Supplementary information

Rfx4	Rfx4	regulatory factor X, 4 (influences HLA class II expression)	71137	https://www.ncbi.nlm.nih.gov/gene/?term=71137
Agtr1b	Agtr1b	angiotensin II receptor, type 1b	11608	https://www.ncbi.nlm.nih.gov/gene/?term=11608
Nop58	Nop58	NOP58 ribonucleoprotein	55989	https://www.ncbi.nlm.nih.gov/gene/?term=55989
Plk4	Plk4	polo like kinase 4	20873	https://www.ncbi.nlm.nih.gov/gene/?term=20873
Pde4b	Pde4b	phosphodiesterase 4B, cAMP specific	18578	https://www.ncbi.nlm.nih.gov/gene/?term=18578
Yy2	Yy2	Yy2 transcription factor	100073351	https://www.ncbi.nlm.nih.gov/gene/?term=100073
Fezf1	Fezf1	Fez family zinc finger 1	73191	https://www.ncbi.nlm.nih.gov/gene/?term=73191
Elovl5	Elovl5	ELOVL family member 5, elongation of long chain fatty acids (yeast)	68801	https://www.ncbi.nlm.nih.gov/gene/?term=68801
Lin54	Lin54	lin-54 homolog (C. elegans)	231506	https://www.ncbi.nlm.nih.gov/gene/?term=231506
Hoxb3	Hoxb3	homeobox B3	15410	https://www.ncbi.nlm.nih.gov/gene/?term=15410
Gbx2	Gbx2	gastrulation brain homeobox 2	14472	https://www.ncbi.nlm.nih.gov/gene/?term=14472
Csf3r	Csf3r	colony stimulating factor 3 receptor (granulocyte)	12986	https://www.ncbi.nlm.nih.gov/gene/?term=12986
Prim1	Prim1	DNA primase, p49 subunit	19075	https://www.ncbi.nlm.nih.gov/gene/?term=19075
Suv39h2	Suv39h2	suppressor of variegation 3-9 2	64707	https://www.ncbi.nlm.nih.gov/gene/?term=64707
Dlg3	Dlg3	discs large MAGUK scaffold protein 3	53310	https://www.ncbi.nlm.nih.gov/gene/?term=53310
Spag7	Spag7	sperm associated antigen 7	216873	https://www.ncbi.nlm.nih.gov/gene/?term=216873
Ntf3	Ntf3	neurotrophin 3	18205	https://www.ncbi.nlm.nih.gov/gene/?term=18205
Setd2	Setd2	SET domain containing 2	235626	https://www.ncbi.nlm.nih.gov/gene/?term=235626
Trappc8	Trappc8	trafficking protein particle complex 8	75964	https://www.ncbi.nlm.nih.gov/gene/?term=75964
Spata7	Spata7	spermatogenesis associated 7	104871	https://www.ncbi.nlm.nih.gov/gene/?term=104871
Snx17	Snx17	sorting nexin 17	266781	https://www.ncbi.nlm.nih.gov/gene/?term=266781

P4ha1	P4ha1	procollagen-proline, 2-oxoglutarate 4-dioxygenase (proline 4-hydroxylase), alpha 1 polypeptide	18451	https://www.ncbi.nlm.nih.gov/gene/?term=18451
Slc41a1	Slc41a1	solute carrier family 41, member 1	98396	https://www.ncbi.nlm.nih.gov/gene/?term=98396
Prdm15	Prdm15	PR domain containing 15	114604	https://www.ncbi.nlm.nih.gov/gene/?term=114604
Golph3l	Golph3l	golgi phosphoprotein 3-like	229593	https://www.ncbi.nlm.nih.gov/gene/?term=229593
Pkm	Pkm	pyruvate kinase, muscle	18746	https://www.ncbi.nlm.nih.gov/gene/?term=18746
Car10	Car10	carbonic anhydrase 10	72605	https://www.ncbi.nlm.nih.gov/gene/?term=72605
Micu3	Micu3	mitochondrial calcium uptake family, member 3	78506	https://www.ncbi.nlm.nih.gov/gene/?term=78506
Gys1	Gys1	glycogen synthase 1, muscle	14936	https://www.ncbi.nlm.nih.gov/gene/?term=14936
Aldoa	Aldoa	aldolase A, fructose-bisphosphate	11674	https://www.ncbi.nlm.nih.gov/gene/?term=11674
Arfip2	Arfip2	ADP-ribosylation factor interacting protein 2	76932	https://www.ncbi.nlm.nih.gov/gene/?term=76932
Rfpl4	Rfpl4	ret finger protein-like 4	192658	https://www.ncbi.nlm.nih.gov/gene/?term=192658
Slc7a1	Slc7a1	solute carrier family 7 (cationic amino acid transporter, y+ system), member 1	11987	https://www.ncbi.nlm.nih.gov/gene/?term=11987
Slc25a34	Slc25a34	solute carrier family 25, member 34	384071	https://www.ncbi.nlm.nih.gov/gene/?term=384071
Txnrd3	Txnrd3	thioredoxin reductase 3	232223	https://www.ncbi.nlm.nih.gov/gene/?term=232223
Hps5	Hps5	HPS5, biogenesis of lysosomal organelles complex 2 subunit 2	246694	https://www.ncbi.nlm.nih.gov/gene/?term=246694
Prune2	Prune2	prune homolog 2	353211	https://www.ncbi.nlm.nih.gov/gene/?term=353211
Zbtb44	Zbtb44	zinc finger and BTB domain containing 44	235132	https://www.ncbi.nlm.nih.gov/gene/?term=235132
Vps37c	Vps37c	vacuolar protein sorting 37C	107305	https://www.ncbi.nlm.nih.gov/gene/?term=107305
Ubox5	Ubox5	U box domain containing 5	140629	https://www.ncbi.nlm.nih.gov/gene/?term=140629

Biology Open • Supplementary information

Bach1	Bach1	BTB and CNC homology 1, basic leucine zipper transcription factor 1	12013	https://www.ncbi.nlm.nih.gov/gene/?term=12013
Hif1an	Hif1an	hypoxia-inducible factor 1, alpha subunit inhibitor	319594	https://www.ncbi.nlm.nih.gov/gene/?term=319594
Rbak	Rbak	RB-associated KRAB zinc finger	57782	https://www.ncbi.nlm.nih.gov/gene/?term=57782
Pik3r2	Pik3r2	phosphoinositide-3-kinase regulatory subunit 2	18709	https://www.ncbi.nlm.nih.gov/gene/?term=18709
Mob1b	Mob1b	MOB kinase activator 1B	68473	https://www.ncbi.nlm.nih.gov/gene/?term=68473
Slco5a1	Slco5a1	solute carrier organic anion transporter family, member 5A1	240726	https://www.ncbi.nlm.nih.gov/gene/?term=240726
Kcnj6	Kcnj6	potassium inwardly-rectifying channel, subfamily J, member 6	16522	https://www.ncbi.nlm.nih.gov/gene/?term=16522
Mtmr2	Mtmr2	myotubularin related protein 2	77116	https://www.ncbi.nlm.nih.gov/gene/?term=77116
Ppp6r3	Ppp6r3	protein phosphatase 6, regulatory subunit 3	52036	https://www.ncbi.nlm.nih.gov/gene/?term=52036
Cacna1e	Cacna1e	calcium channel, voltage-dependent, R type, alpha 1E subunit	12290	https://www.ncbi.nlm.nih.gov/gene/?term=12290
Orc5	Orc5	origin recognition complex, subunit 5	26429	https://www.ncbi.nlm.nih.gov/gene/?term=26429
Esrra	Esrra	estrogen related receptor, alpha	26379	https://www.ncbi.nlm.nih.gov/gene/?term=26379
Gria3	Gria3	glutamate receptor, ionotropic, AMPA3 (alpha 3)	53623	https://www.ncbi.nlm.nih.gov/gene/?term=53623
Dip2c	Dip2c	disco interacting protein 2 homolog C	208440	https://www.ncbi.nlm.nih.gov/gene/?term=208440
Pitpnc1	Pitpnc1	phosphatidylinositol transfer protein, cytoplasmic 1	71795	https://www.ncbi.nlm.nih.gov/gene/?term=71795
Wscd2	Wscd2	WSC domain containing 2	320916	https://www.ncbi.nlm.nih.gov/gene/?term=320916

Slc9a3r2	Slc9a3r2	solute carrier family 9 (sodium/hydrogen exchanger), member 3 regulator 2	65962	https://www.ncbi.nlm.nih.gov/gene/?term=65962
Shisa7	Shisa7	shisa family member 7	232813	https://www.ncbi.nlm.nih.gov/gene/?term=232813
4930562C15Rik	4930562C15Rik	RIKEN cDNA 4930562C15 gene	78809	https://www.ncbi.nlm.nih.gov/gene/?term=78809
Ado	Ado	2-aminoethanethiol (cysteamine) dioxygenase	211488	https://www.ncbi.nlm.nih.gov/gene/?term=211488
Ildr2	Ildr2	immunoglobulin-like domain containing receptor 2	100039795	https://www.ncbi.nlm.nih.gov/gene/?term=100039
Trpc6	Trpc6	transient receptor potential cation channel, subfamily C, member 6	22068	https://www.ncbi.nlm.nih.gov/gene/?term=22068
Zranb2	Zranb2	zinc finger, RAN-binding domain containing 2	53861	https://www.ncbi.nlm.nih.gov/gene/?term=53861
Rasal2	Rasal2	RAS protein activator like 2	226525	https://www.ncbi.nlm.nih.gov/gene/?term=226525
Vamp2	Vamp2	vesicle-associated membrane protein 2	22318	https://www.ncbi.nlm.nih.gov/gene/?term=22318
Cplx2	Cplx2	complexin 2	12890	https://www.ncbi.nlm.nih.gov/gene/?term=12890
Slc25a5	Slc25a5	NA	11740	https://www.ncbi.nlm.nih.gov/gene/?term=11740
Sdcbp	Sdcbp	syndecan binding protein	53378	https://www.ncbi.nlm.nih.gov/gene/?term=53378
Cplx1	Cplx1	complexin 1	12889	https://www.ncbi.nlm.nih.gov/gene/?term=12889
Kcnd1	Kcnd1	potassium voltage-gated channel, Shal-related family, member 1	16506	https://www.ncbi.nlm.nih.gov/gene/?term=16506
Ssr2	Ssr2	signal sequence receptor, beta	66256	https://www.ncbi.nlm.nih.gov/gene/?term=66256
Ints2	Ints2	integrator complex subunit 2	70422	https://www.ncbi.nlm.nih.gov/gene/?term=70422
Atp11b	Atp11b	ATPase, class VI, type 11B	76295	https://www.ncbi.nlm.nih.gov/gene/?term=76295
Syt2	Syt2	synaptotagmin II	20980	https://www.ncbi.nlm.nih.gov/gene/?term=20980
Bsn	Bsn	bassoon	12217	https://www.ncbi.nlm.nih.gov/gene/?term=12217
Gdf6	Gdf6	growth differentiation factor 6	242316	https://www.ncbi.nlm.nih.gov/gene/?term=242316

Cntn5	Cntn5	contactin 5	244682	https://www.ncbi.nlm.nih.gov/gene/?term=244682
Mab21l1	Mab21l1	mab-21-like 1 (C. elegans)	17116	https://www.ncbi.nlm.nih.gov/gene/?term=17116
Radil	Radil	Ras association and DIL domains	231858	https://www.ncbi.nlm.nih.gov/gene/?term=231858
Mtmr7	Mtmr7	myotubularin related protein 7	54384	https://www.ncbi.nlm.nih.gov/gene/?term=54384
Rpusd4	Rpusd4	RNA pseudouridylate synthase domain containing 4	71989	https://www.ncbi.nlm.nih.gov/gene/?term=71989
Slc7a3	Slc7a3	solute carrier family 7 (cationic amino acid transporter, y+ system), member 3	11989	https://www.ncbi.nlm.nih.gov/gene/?term=11989
Nr1h5	Nr1h5	nuclear receptor subfamily 1, group H, member 5	381463	https://www.ncbi.nlm.nih.gov/gene/?term=381463
Hoxc10	Hoxc10	homeobox C10	209448	https://www.ncbi.nlm.nih.gov/gene/?term=209448
Irak2	Irak2	interleukin-1 receptor-associated kinase 2	108960	https://www.ncbi.nlm.nih.gov/gene/?term=108960
Ccdc7a	Ccdc7a	coiled-coil domain containing 7A	74703	https://www.ncbi.nlm.nih.gov/gene/?term=74703
Cry2	Cry2	cryptochrome 2 (photolyase-like)	12953	https://www.ncbi.nlm.nih.gov/gene/?term=12953
Vps26a	Vps26a	VPS26 retromer complex component A	30930	https://www.ncbi.nlm.nih.gov/gene/?term=30930
St8sia2	St8sia2	ST8 alpha-N-acetyl-neuraminide alpha-2,8-sialyltransferase 2	20450	https://www.ncbi.nlm.nih.gov/gene/?term=20450
Ugt2b37	Ugt2b37	UDP glucuronosyltransferase 2 family, polypeptide B37	112417	https://www.ncbi.nlm.nih.gov/gene/?term=112417
Tcta	Tcta	T cell leukemia translocation altered gene	102791	https://www.ncbi.nlm.nih.gov/gene/?term=102791
Zfp202	Zfp202	zinc finger protein 202	80902	https://www.ncbi.nlm.nih.gov/gene/?term=80902
Nr2e1	Nr2e1	nuclear receptor subfamily 2, group E, member 1	21907	https://www.ncbi.nlm.nih.gov/gene/?term=21907

Sox10	Sox10	SRY (sex determining region Y)-box 10	20665	https://www.ncbi.nlm.nih.gov/gene/?term=20665
Col1a2	Col1a2	collagen, type I, alpha 2	12843	https://www.ncbi.nlm.nih.gov/gene/?term=12843
Pak1	Pak1	p21 (RAC1) activated kinase 1	18479	https://www.ncbi.nlm.nih.gov/gene/?term=18479
Ube2i	Ube2i	ubiquitin-conjugating enzyme E2I	22196	https://www.ncbi.nlm.nih.gov/gene/?term=22196
Rnf222	Rnf222	ring finger protein 222	320040	https://www.ncbi.nlm.nih.gov/gene/?term=320040
Ubxn4	Ubxn4	UBX domain protein 4	67812	https://www.ncbi.nlm.nih.gov/gene/?term=67812
Rps6ka2	Rps6ka2	NA	20112	https://www.ncbi.nlm.nih.gov/gene/?term=20112
Pacs1	Pacs1	phosphofurin acidic cluster sorting protein 1	107975	https://www.ncbi.nlm.nih.gov/gene/?term=107975
Pif1	Pif1	PIF1 5'-to-3' DNA helicase	208084	https://www.ncbi.nlm.nih.gov/gene/?term=208084
Clec4b2	Clec4b2	C-type lectin domain family 4, member b2	381809	https://www.ncbi.nlm.nih.gov/gene/?term=381809
Rnf13	Rnf13	ring finger protein 13	24017	https://www.ncbi.nlm.nih.gov/gene/?term=24017
Spire2	Spire2	spire type actin nucleation factor 2	234857	https://www.ncbi.nlm.nih.gov/gene/?term=234857
Aadat	Aadat	aminoadipate aminotransferase	23923	https://www.ncbi.nlm.nih.gov/gene/?term=23923
Akr1c14	Akr1c14	aldo-keto reductase family 1, member C14	105387	https://www.ncbi.nlm.nih.gov/gene/?term=105387
Sspn	Sspn	sarcospan	16651	https://www.ncbi.nlm.nih.gov/gene/?term=16651
Akr1c6	Akr1c6	aldo-keto reductase family 1, member C6	83702	https://www.ncbi.nlm.nih.gov/gene/?term=83702
Tmem81	Tmem81	transmembrane protein 81	74626	https://www.ncbi.nlm.nih.gov/gene/?term=74626
Rerg	Rerg	RAS-like, estrogen-regulated, growth-inhibitor	232441	https://www.ncbi.nlm.nih.gov/gene/?term=232441
Tnfsf9	Tnfsf9	tumor necrosis factor (ligand) superfamily, member 9	21950	https://www.ncbi.nlm.nih.gov/gene/?term=21950
Ccdc153	Ccdc153	coiled-coil domain containing 153	270150	https://www.ncbi.nlm.nih.gov/gene/?term=270150
Per3	Per3	period circadian clock 3	18628	https://www.ncbi.nlm.nih.gov/gene/?term=18628

Gm13212	Gm13212	predicted gene 13212	433801	https://www.ncbi.nlm.nih.gov/gene/?term=433801
Tom1l1	Tom1l1	target of myb1-like 1 (chicken)	71943	https://www.ncbi.nlm.nih.gov/gene/?term=71943
Kctd5	Kctd5	potassium channel tetramerisation domain containing 5	69259	https://www.ncbi.nlm.nih.gov/gene/?term=69259
1810037I17Rik	1810037I17Rik	RIKEN cDNA 1810037I17 gene	67704	https://www.ncbi.nlm.nih.gov/gene/?term=67704
Stradb	Stradb	STE20-related kinase adaptor beta	227154	https://www.ncbi.nlm.nih.gov/gene/?term=227154
Ing5	Ing5	inhibitor of growth family, member 5	66262	https://www.ncbi.nlm.nih.gov/gene/?term=66262
Mapk10	Mapk10	mitogen-activated protein kinase 10	26414	https://www.ncbi.nlm.nih.gov/gene/?term=26414
Gba2	Gba2	glucosidase beta 2	230101	https://www.ncbi.nlm.nih.gov/gene/?term=230101
Otud6b	Otud6b	OTU domain containing 6B	72201	https://www.ncbi.nlm.nih.gov/gene/?term=72201
Dcaf7	Dcaf7	DDB1 and CUL4 associated factor 7	71833	https://www.ncbi.nlm.nih.gov/gene/?term=71833
Plaur	Plaur	plasminogen activator, urokinase receptor	18793	https://www.ncbi.nlm.nih.gov/gene/?term=18793
Mybpc1	Mybpc1	myosin binding protein C, slow-type	109272	https://www.ncbi.nlm.nih.gov/gene/?term=109272
Tex2	Tex2	testis expressed gene 2	21763	https://www.ncbi.nlm.nih.gov/gene/?term=21763
Fam83d	Fam83d	family with sequence similarity 83, member D	71878	https://www.ncbi.nlm.nih.gov/gene/?term=71878
Ankrd40	Ankrd40	ankyrin repeat domain 40	71452	https://www.ncbi.nlm.nih.gov/gene/?term=71452
Zrsr2	Zrsr2	zinc finger (CCCH type), RNA binding motif and serine/arginine rich 2	22184	https://www.ncbi.nlm.nih.gov/gene/?term=22184
Tgm4	Tgm4	transglutaminase 4 (prostate)	331046	https://www.ncbi.nlm.nih.gov/gene/?term=331046
Pja1	Pja1	praja ring finger ubiquitin ligase 1	18744	https://www.ncbi.nlm.nih.gov/gene/?term=18744
Ccnd3	Ccnd3	cyclin D3	12445	https://www.ncbi.nlm.nih.gov/gene/?term=12445

Biology Open • Supplementary information

Bmpr2	Bmpr2	bone morphogenetic protein receptor, type II (serine/threonine kinase)	12168	https://www.ncbi.nlm.nih.gov/gene/?term=12168
Isg20	Isg20	interferon-stimulated protein	57444	https://www.ncbi.nlm.nih.gov/gene/?term=57444
Tmem215	Tmem215	transmembrane protein 215	320500	https://www.ncbi.nlm.nih.gov/gene/?term=320500
Etv5	Etv5	ets variant 5	104156	https://www.ncbi.nlm.nih.gov/gene/?term=104156
Irx2	Irx2	Iroquois homeobox 2	16372	https://www.ncbi.nlm.nih.gov/gene/?term=16372
Krt42	Krt42	keratin 42	68239	https://www.ncbi.nlm.nih.gov/gene/?term=68239
Kcnb2	Kcnb2	potassium voltage gated channel, Shab-related subfamily, member 2	98741	https://www.ncbi.nlm.nih.gov/gene/?term=98741
Bbof1	Bbof1	basal body orientation factor 1	72873	https://www.ncbi.nlm.nih.gov/gene/?term=72873
Tmem231	Tmem231	transmembrane protein 231	234740	https://www.ncbi.nlm.nih.gov/gene/?term=234740
Il22ra2	Il22ra2	interleukin 22 receptor, alpha 2	237310	https://www.ncbi.nlm.nih.gov/gene/?term=237310
Saal1	Saal1	serum amyloid A-like 1	78935	https://www.ncbi.nlm.nih.gov/gene/?term=78935
Mpeg1	Mpeg1	macrophage expressed gene 1	17476	https://www.ncbi.nlm.nih.gov/gene/?term=17476
Bbx	Bbx	bobby sox HMG box containing	70508	https://www.ncbi.nlm.nih.gov/gene/?term=70508
Adat1	Adat1	adenosine deaminase, tRNA-specific 1	30947	https://www.ncbi.nlm.nih.gov/gene/?term=30947
Sub1	Sub1	SUB1 homolog (S. cerevisiae)	20024	https://www.ncbi.nlm.nih.gov/gene/?term=20024
Nsmce3	Nsmce3	NSE3 homolog, SMC5-SMC6 complex component	66647	https://www.ncbi.nlm.nih.gov/gene/?term=66647
Miga1	Miga1	mitoguardin 1	215708	https://www.ncbi.nlm.nih.gov/gene/?term=215708
Ndnf	Ndnf	neuron-derived neurotrophic factor	68169	https://www.ncbi.nlm.nih.gov/gene/?term=68169
Myo5b	Myo5b	myosin VB	17919	https://www.ncbi.nlm.nih.gov/gene/?term=17919
Rnf126	Rnf126	ring finger protein 126	70294	https://www.ncbi.nlm.nih.gov/gene/?term=70294
Abca5	Abca5	ATP-binding cassette, sub-family A (ABC1), member 5	217265	https://www.ncbi.nlm.nih.gov/gene/?term=217265

Itm2c	Itm2c	integral membrane protein 2C	64294	https://www.ncbi.nlm.nih.gov/gene/?term=64294
Abhd17c	Abhd17c	abhydrolase domain containing 17C	70178	https://www.ncbi.nlm.nih.gov/gene/?term=70178
Trpc5	Trpc5	transient receptor potential cation channel, subfamily C, member 5	22067	https://www.ncbi.nlm.nih.gov/gene/?term=22067
Zmat3	Zmat3	zinc finger matrin type 3	22401	https://www.ncbi.nlm.nih.gov/gene/?term=22401
Dlx1	Dlx1	distal-less homeobox 1	13390	https://www.ncbi.nlm.nih.gov/gene/?term=13390
Vxn	Vxn	vexin	76982	https://www.ncbi.nlm.nih.gov/gene/?term=76982
Ptk2b	Ptk2b	PTK2 protein tyrosine kinase 2 beta	19229	https://www.ncbi.nlm.nih.gov/gene/?term=19229
Nkrf	Nkrf	NF-kappaB repressing factor	77286	https://www.ncbi.nlm.nih.gov/gene/?term=77286
BC053393	BC053393	cDNA sequence BC053393	407814	https://www.ncbi.nlm.nih.gov/gene/?term=407814
Ebf2	Ebf2	early B cell factor 2	13592	https://www.ncbi.nlm.nih.gov/gene/?term=13592
Atoh1	Atoh1	atonal bHLH transcription factor 1	11921	https://www.ncbi.nlm.nih.gov/gene/?term=11921
Gipc1	Gipc1	GIPC PDZ domain containing family, member 1	67903	https://www.ncbi.nlm.nih.gov/gene/?term=67903
Fam169a	Fam169a	family with sequence similarity 169, member A	320557	https://www.ncbi.nlm.nih.gov/gene/?term=320557
Pfkfb4	Pfkfb4	6-phosphofructo-2-kinase/fructose-2,6-biphosphatase 4	270198	https://www.ncbi.nlm.nih.gov/gene/?term=270198
Fcrl5	Fcrl5	Fc receptor-like 5	329693	https://www.ncbi.nlm.nih.gov/gene/?term=329693
Phf6	Phf6	PHD finger protein 6	70998	https://www.ncbi.nlm.nih.gov/gene/?term=70998
Rad23b	Rad23b	RAD23 homolog B, nucleotide excision repair protein	19359	https://www.ncbi.nlm.nih.gov/gene/?term=19359
Prickle2	Prickle2	prickle planar cell polarity protein 2	243548	https://www.ncbi.nlm.nih.gov/gene/?term=243548
Adamts7	Adamts7	a disintegrin-like and metallopeptidase (reprolysin type) with thrombospondin type 1 motif, 7	108153	https://www.ncbi.nlm.nih.gov/gene/?term=108153
Chdh	Chdh	choline dehydrogenase	218865	https://www.ncbi.nlm.nih.gov/gene/?term=218865

Zfp81	Zfp81	zinc finger protein 81	224694	https://www.ncbi.nlm.nih.gov/gene/?term=224694
Foxo4	Foxo4	forkhead box O4	54601	https://www.ncbi.nlm.nih.gov/gene/?term=54601
Mmrn1	Mmrn1	multimerin 1	70945	https://www.ncbi.nlm.nih.gov/gene/?term=70945
F7	F7	coagulation factor VII	14068	https://www.ncbi.nlm.nih.gov/gene/?term=14068
Sipa1l1	Sipa1l1	signal-induced proliferation-associated 1 like 1	217692	https://www.ncbi.nlm.nih.gov/gene/?term=217692
Rbm7	Rbm7	RNA binding motif protein 7	67010	https://www.ncbi.nlm.nih.gov/gene/?term=67010
Rock1	Rock1	Rho-associated coiled-coil containing protein kinase 1	19877	https://www.ncbi.nlm.nih.gov/gene/?term=19877
Npy2r	Npy2r	neuropeptide Y receptor Y2	18167	https://www.ncbi.nlm.nih.gov/gene/?term=18167
Gmnc	Gmnc	geminin coiled-coil domain containing	239789	https://www.ncbi.nlm.nih.gov/gene/?term=239789
Bri3	Bri3	brain protein I3	55950	https://www.ncbi.nlm.nih.gov/gene/?term=55950
Igf2bp2	Igf2bp2	insulin-like growth factor 2 mRNA binding protein 2	319765	https://www.ncbi.nlm.nih.gov/gene/?term=319765
Txn1	Txn1	thioredoxin 1	22166	https://www.ncbi.nlm.nih.gov/gene/?term=22166
Tnik	Tnik	TRAF2 and NCK interacting kinase	665113	https://www.ncbi.nlm.nih.gov/gene/?term=665113
Tbca	Tbca	tubulin cofactor A	21371	https://www.ncbi.nlm.nih.gov/gene/?term=21371
Negr1	Negr1	neuronal growth regulator 1	320840	https://www.ncbi.nlm.nih.gov/gene/?term=320840
Pcnp	Pcnp	PEST proteolytic signal containing nuclear protein	76302	https://www.ncbi.nlm.nih.gov/gene/?term=76302
Ercc8	Ercc8	excision repaiross-complementing rodent repair deficiency, complementation group 8	71991	https://www.ncbi.nlm.nih.gov/gene/?term=71991
Rnf32	Rnf32	ring finger protein 32	56874	https://www.ncbi.nlm.nih.gov/gene/?term=56874
Itgb6	Itgb6	integrin beta 6	16420	https://www.ncbi.nlm.nih.gov/gene/?term=16420
Pof1b	Pof1b	premature ovarian failure 1B	69693	https://www.ncbi.nlm.nih.gov/gene/?term=69693

Biology Open • Supplementary information

Arfip1	Arfip1	ADP-ribosylation factor interacting protein 1	99889	https://www.ncbi.nlm.nih.gov/gene/?term=99889
Car2	Car2	carbonic anhydrase 2	12349	https://www.ncbi.nlm.nih.gov/gene/?term=12349
Ehd3	Ehd3	EH-domain containing 3	57440	https://www.ncbi.nlm.nih.gov/gene/?term=57440
Doc2b	Doc2b	double C2, beta	13447	https://www.ncbi.nlm.nih.gov/gene/?term=13447
Slc33a1	Slc33a1	solute carrier family 33 (acetyl-CoA transporter), member 1	11416	https://www.ncbi.nlm.nih.gov/gene/?term=11416
Rnaseh2c	Rnaseh2c	ribonuclease H2, subunit C	68209	https://www.ncbi.nlm.nih.gov/gene/?term=68209
Ift27	Ift27	intraflagellar transport 27	67042	https://www.ncbi.nlm.nih.gov/gene/?term=67042
Cnot4	Cnot4	CCR4-NOT transcription complex, subunit 4	53621	https://www.ncbi.nlm.nih.gov/gene/?term=53621
Rab7	Rab7	RAB7, member RAS oncogene family	19349	https://www.ncbi.nlm.nih.gov/gene/?term=19349
Phf20	Phf20	PHD finger protein 20	228829	https://www.ncbi.nlm.nih.gov/gene/?term=228829
Slc25a15	Slc25a15	solute carrier family 25 (mitochondrial carrier ornithine transporter), member 15	18408	https://www.ncbi.nlm.nih.gov/gene/?term=18408
Sycp1	Sycp1	synaptonemal complex protein 1	20957	https://www.ncbi.nlm.nih.gov/gene/?term=20957
0610040J01Rik	0610040J01Rik	RIKEN cDNA 0610040J01 gene	76261	https://www.ncbi.nlm.nih.gov/gene/?term=76261
Dnajc16	Dnajc16	DnaJ heat shock protein family (Hsp40) member C16	214063	https://www.ncbi.nlm.nih.gov/gene/?term=214063
Lrrtm2	Lrrtm2	leucine rich repeat transmembrane neuronal 2	107065	https://www.ncbi.nlm.nih.gov/gene/?term=107065
E2f4	E2f4	E2F transcription factor 4	104394	https://www.ncbi.nlm.nih.gov/gene/?term=104394
Gm11487	Gm11487	predicted gene 11487	433719	https://www.ncbi.nlm.nih.gov/gene/?term=433719
Pcm1	Pcm1	pericentriolar material 1	18536	https://www.ncbi.nlm.nih.gov/gene/?term=18536
Caml	Caml	calcium modulating ligand	12328	https://www.ncbi.nlm.nih.gov/gene/?term=12328
Noxo1	Noxo1	NADPH oxidase organizer 1	71893	https://www.ncbi.nlm.nih.gov/gene/?term=71893

Biology Open • Supplementary information

Utp18	Utp18	UTP18 small subunit processome component	217109	https://www.ncbi.nlm.nih.gov/gene/?term=217109
Pag1	Pag1	phosphoprotein associated with glycosphingolipid microdomains 1	94212	https://www.ncbi.nlm.nih.gov/gene/?term=94212
Asb10	Asb10	ankyrin repeat and SOCS box-containing 10	117590	https://www.ncbi.nlm.nih.gov/gene/?term=117590
Hsdl1	Hsdl1	hydroxysteroid dehydrogenase like 1	72552	https://www.ncbi.nlm.nih.gov/gene/?term=72552
Arfgef2	Arfgef2	ADP-ribosylation factor guanine nucleotide-exchange factor 2 (brefeldin A-inhibited)	99371	https://www.ncbi.nlm.nih.gov/gene/?term=99371
Nuf2	Nuf2	NUF2, NDC80 kinetochore complex component	66977	https://www.ncbi.nlm.nih.gov/gene/?term=66977
Syf2	Syf2	SYF2 homolog, RNA splicing factor (S. cerevisiae)	68592	https://www.ncbi.nlm.nih.gov/gene/?term=68592
Ccdc82	Ccdc82	coiled-coil domain containing 82	66396	https://www.ncbi.nlm.nih.gov/gene/?term=66396
Piga	Piga	phosphatidylinositol glycan anchor biosynthesis, class A	18700	https://www.ncbi.nlm.nih.gov/gene/?term=18700
Cnih2	Cnih2	cornichon family AMPA receptor auxiliary protein 2	12794	https://www.ncbi.nlm.nih.gov/gene/?term=12794
Ncapg	Ncapg	non-SMC condensin I complex, subunit G	54392	https://www.ncbi.nlm.nih.gov/gene/?term=54392
Uba5	Uba5	ubiquitin-like modifier activating enzyme 5	66663	https://www.ncbi.nlm.nih.gov/gene/?term=66663
Cacnb2	Cacnb2	calcium channel, voltage-dependent, beta 2 subunit	12296	https://www.ncbi.nlm.nih.gov/gene/?term=12296
Tspan2	Tspan2	tetraspanin 2	70747	https://www.ncbi.nlm.nih.gov/gene/?term=70747
Prok2	Prok2	prokineticin 2	50501	https://www.ncbi.nlm.nih.gov/gene/?term=50501

Biology Open • Supplementary information

Vkorc1	Vkorc1	vitamin K epoxide reductase complex, subunit 1	27973	https://www.ncbi.nlm.nih.gov/gene/?term=27973
En2	En2	engrailed 2	13799	https://www.ncbi.nlm.nih.gov/gene/?term=13799
Adssl1	Adssl1	adenylosuccinate synthetase like 1	11565	https://www.ncbi.nlm.nih.gov/gene/?term=11565
Arfgef1	Arfgef1	ADP-ribosylation factor guanine nucleotide-exchange factor 1(brefeldin A-inhibited)	211673	https://www.ncbi.nlm.nih.gov/gene/?term=211673
Oasl2	Oasl2	2'-5' oligoadenylate synthetase-like 2	23962	https://www.ncbi.nlm.nih.gov/gene/?term=23962
Cep57l1	Cep57l1	centrosomal protein 57-like 1	103268	https://www.ncbi.nlm.nih.gov/gene/?term=103268
Cyp20a1	Cyp20a1	cytochrome P450, family 20, subfamily a, polypeptide 1	77951	https://www.ncbi.nlm.nih.gov/gene/?term=77951
Ophn1	Ophn1	oligophrenin 1	94190	https://www.ncbi.nlm.nih.gov/gene/?term=94190
Rnf182	Rnf182	ring finger protein 182	328234	https://www.ncbi.nlm.nih.gov/gene/?term=328234
Pde10a	Pde10a	phosphodiesterase 10A	23984	https://www.ncbi.nlm.nih.gov/gene/?term=23984
Zbtb43	Zbtb43	zinc finger and BTB domain containing 43	71834	https://www.ncbi.nlm.nih.gov/gene/?term=71834
Trdmt1	Trdmt1	tRNA aspartic acid methyltransferase 1	13434	https://www.ncbi.nlm.nih.gov/gene/?term=13434
Slc45a2	Slc45a2	solute carrier family 45, member 2	22293	https://www.ncbi.nlm.nih.gov/gene/?term=22293
Kif27	Kif27	kinesin family member 27	75050	https://www.ncbi.nlm.nih.gov/gene/?term=75050
Ifih1	Ifih1	interferon induced with helicase C domain 1	71586	https://www.ncbi.nlm.nih.gov/gene/?term=71586
Scml2	Scml2	Scm polycomb group protein like 2	107815	https://www.ncbi.nlm.nih.gov/gene/?term=107815
Mcur1	Mcur1	mitochondrial calcium uniporter regulator 1	76137	https://www.ncbi.nlm.nih.gov/gene/?term=76137
Prps2	Prps2	phosphoribosyl pyrophosphate synthetase 2	110639	https://www.ncbi.nlm.nih.gov/gene/?term=110639

Biology Open • Supplementary information

Ercc6l	Ercc6l	excision repair cross-complementing rodent repair deficiency complementation group 6 like	236930	https://www.ncbi.nlm.nih.gov/gene/?term=236930
Acer3	Acer3	alkaline ceramidase 3	66190	https://www.ncbi.nlm.nih.gov/gene/?term=66190
Sohlh2	Sohlh2	spermatogenesis and oogenesis specific basic helix-loop-helix 2	74434	https://www.ncbi.nlm.nih.gov/gene/?term=74434
D230025D16Rik	D230025D16Rik	RIKEN cDNA D230025D16 gene	234678	https://www.ncbi.nlm.nih.gov/gene/?term=234678
Sox8	Sox8	SRY (sex determining region Y)-box 8	20681	https://www.ncbi.nlm.nih.gov/gene/?term=20681
Ptprc	Ptprc	protein tyrosine phosphatase, receptor type, C	19264	https://www.ncbi.nlm.nih.gov/gene/?term=19264
Tlk1	Tlk1	tousled-like kinase 1	228012	https://www.ncbi.nlm.nih.gov/gene/?term=228012
Gpatch1	Gpatch1	G patch domain containing 1	67471	https://www.ncbi.nlm.nih.gov/gene/?term=67471
Ptk2	Ptk2	PTK2 protein tyrosine kinase 2	14083	https://www.ncbi.nlm.nih.gov/gene/?term=14083
Rapgef6	Rapgef6	Rap guanine nucleotide exchange factor (GEF) 6	192786	https://www.ncbi.nlm.nih.gov/gene/?term=192786
Dcx	Dcx	doublecortin	13193	https://www.ncbi.nlm.nih.gov/gene/?term=13193
Nap1l4	Nap1l4	nucleosome assembly protein 1-like 4	17955	https://www.ncbi.nlm.nih.gov/gene/?term=17955
Tubb4b	Tubb4b	tubulin, beta 4B class IVB	227613	https://www.ncbi.nlm.nih.gov/gene/?term=227613
Cacna2d2	Cacna2d2	calcium channel, voltage-dependent, alpha 2/delta subunit 2	56808	https://www.ncbi.nlm.nih.gov/gene/?term=56808
Csmd2	Csmd2	CUB and Sushi multiple domains 2	329942	https://www.ncbi.nlm.nih.gov/gene/?term=329942
Sdc3	Sdc3	syndecan 3	20970	https://www.ncbi.nlm.nih.gov/gene/?term=20970
Nol4l	Nol4l	nucleolar protein 4-like	329540	https://www.ncbi.nlm.nih.gov/gene/?term=329540

Biology Open • Supplementary information

Mllt3	Mllt3	myeloid/lymphoid or mixed-lineage leukemia; translocated to, 3	70122	https://www.ncbi.nlm.nih.gov/gene/?term=70122
Leo1	Leo1	Leo1, Paf1/RNA polymerase II complex component	235497	https://www.ncbi.nlm.nih.gov/gene/?term=235497
Tpm1	Tpm1	tropomyosin 1, alpha	22003	https://www.ncbi.nlm.nih.gov/gene/?term=22003
Zfp385b	Zfp385b	zinc finger protein 385B	241494	https://www.ncbi.nlm.nih.gov/gene/?term=241494
Taf6	Taf6	TATA-box binding protein associated factor 6	21343	https://www.ncbi.nlm.nih.gov/gene/?term=21343
Clk4	Clk4	CDC like kinase 4	12750	https://www.ncbi.nlm.nih.gov/gene/?term=12750
Skil	Skil	SKI-like	20482	https://www.ncbi.nlm.nih.gov/gene/?term=20482
Ppip5k1	Ppip5k1	diphosphoinositol pentakisphosphate kinase 1	327655	https://www.ncbi.nlm.nih.gov/gene/?term=327655
Apc	Apc	APC, WNT signaling pathway regulator	11789	https://www.ncbi.nlm.nih.gov/gene/?term=11789
Cxcr4	Cxcr4	chemokine (C-X-C motif) receptor 4	12767	https://www.ncbi.nlm.nih.gov/gene/?term=12767
Cemip2	Cemip2	cell migration inducing hyaluronidase 2	83921	https://www.ncbi.nlm.nih.gov/gene/?term=83921
Arhgef7	Arhgef7	Rho guanine nucleotide exchange factor (GEF7)	54126	https://www.ncbi.nlm.nih.gov/gene/?term=54126
Trrap	Trrap	transformation/transcription domain-associated protein	100683	https://www.ncbi.nlm.nih.gov/gene/?term=100683
Brd8	Brd8	bromodomain containing 8	78656	https://www.ncbi.nlm.nih.gov/gene/?term=78656
Ubqln1	Ubqln1	ubiquilin 1	56085	https://www.ncbi.nlm.nih.gov/gene/?term=56085
Ormdl3	Ormdl3	ORM1-like 3 (S. cerevisiae)	66612	https://www.ncbi.nlm.nih.gov/gene/?term=66612
Ppp2ca	Ppp2ca	protein phosphatase 2 (formerly 2A), catalytic subunit, alpha isoform	19052	https://www.ncbi.nlm.nih.gov/gene/?term=19052

Ccna2	Ccna2	cyclin A2	12428	https://www.ncbi.nlm.nih.gov/gene/?term=12428
Fat1	Fat1	FAT atypical cadherin 1	14107	https://www.ncbi.nlm.nih.gov/gene/?term=14107
Mill1	Mill1	MHC I like leukocyte 1	266815	https://www.ncbi.nlm.nih.gov/gene/?term=266815
Wdr90	Wdr90	WD repeat domain 90	106618	https://www.ncbi.nlm.nih.gov/gene/?term=106618
Snx25	Snx25	sorting nexin 25	102141	https://www.ncbi.nlm.nih.gov/gene/?term=102141
Fzd7	Fzd7	frizzled class receptor 7	14369	https://www.ncbi.nlm.nih.gov/gene/?term=14369
Tm7sf3	Tm7sf3	transmembrane 7 superfamily member 3	67623	https://www.ncbi.nlm.nih.gov/gene/?term=67623
Ubqln2	Ubqln2	ubiquilin 2	54609	https://www.ncbi.nlm.nih.gov/gene/?term=54609
Hmgcs1	Hmgcs1	3-hydroxy-3-methylglutaryl-Coenzyme A synthase 1	208715	https://www.ncbi.nlm.nih.gov/gene/?term=208715
Rbm5	Rbm5	RNA binding motif protein 5	83486	https://www.ncbi.nlm.nih.gov/gene/?term=83486
Cacul1	Cacul1	CDK2 associated, cullin domain 1	78832	https://www.ncbi.nlm.nih.gov/gene/?term=78832
Jakmip3	Jakmip3	janus kinase and microtubule interacting protein 3	74004	https://www.ncbi.nlm.nih.gov/gene/?term=74004
Atp2a2	Atp2a2	ATPase, Ca++ transporting, cardiac muscle, slow twitch 2	11938	https://www.ncbi.nlm.nih.gov/gene/?term=11938
Neo1	Neo1	neogenin	18007	https://www.ncbi.nlm.nih.gov/gene/?term=18007
Derl1	Derl1	Der1-like domain family, member 1	67819	https://www.ncbi.nlm.nih.gov/gene/?term=67819
Ksr2	Ksr2	kinase suppressor of ras 2	333050	https://www.ncbi.nlm.nih.gov/gene/?term=333050
Snrpd1	Snrpd1	small nuclear ribonucleoprotein D1	20641	https://www.ncbi.nlm.nih.gov/gene/?term=20641
Ccdc6	Ccdc6	coiled-coil domain containing 6	76551	https://www.ncbi.nlm.nih.gov/gene/?term=76551
Rnps1	Rnps1	ribonucleic acid binding protein S1	19826	https://www.ncbi.nlm.nih.gov/gene/?term=19826
Prrc2c	Prrc2c	proline-rich coiled-coil 2C	226562	https://www.ncbi.nlm.nih.gov/gene/?term=226562
Nrxn3	Nrxn3	neurexin III	18191	https://www.ncbi.nlm.nih.gov/gene/?term=18191
Prex1	Prex1	phosphatidylinositol-3,4,5-trisphosphate-dependent Rac exchange factor 1	277360	https://www.ncbi.nlm.nih.gov/gene/?term=277360

Kdelr1	Kdelr1	KDEL (Lys-Asp-Glu-Leu) endoplasmic reticulum protein retention receptor 1	68137	https://www.ncbi.nlm.nih.gov/gene/?term=68137
Rab40c	Rab40c	Rab40C, member RAS oncogene family	224624	https://www.ncbi.nlm.nih.gov/gene/?term=224624
Fmnl2	Fmnl2	formin-like 2	71409	https://www.ncbi.nlm.nih.gov/gene/?term=71409
Atp5a1	Atp5a1	ATP synthase, H+ transporting, mitochondrial F1 complex, alpha subunit 1	11946	https://www.ncbi.nlm.nih.gov/gene/?term=11946
Rab14	Rab14	RAB14, member RAS oncogene family	68365	https://www.ncbi.nlm.nih.gov/gene/?term=68365
Il1r1	Il1r1	interleukin 1 receptor, type I	16177	https://www.ncbi.nlm.nih.gov/gene/?term=16177
Spry2	Spry2	sprouty RTK signaling antagonist 2	24064	https://www.ncbi.nlm.nih.gov/gene/?term=24064
Sox2	Sox2	SRY (sex determining region Y)-box 2	20674	https://www.ncbi.nlm.nih.gov/gene/?term=20674
Btg1	Btg1	B cell translocation gene 1, anti-proliferative	12226	https://www.ncbi.nlm.nih.gov/gene/?term=12226
Rab19	Rab19	RAB19, member RAS oncogene family	19331	https://www.ncbi.nlm.nih.gov/gene/?term=19331
Brix1	Brix1	BRX1, biogenesis of ribosomes	67832	https://www.ncbi.nlm.nih.gov/gene/?term=67832
Pdgfc	Pdgfc	platelet-derived growth factor, C polypeptide	54635	https://www.ncbi.nlm.nih.gov/gene/?term=54635
Zfand5	Zfand5	zinc finger, AN1-type domain 5	22682	https://www.ncbi.nlm.nih.gov/gene/?term=22682
Usp21	Usp21	ubiquitin specific peptidase 21	30941	https://www.ncbi.nlm.nih.gov/gene/?term=30941
Gmcl1	Gmcl1	germ cell-less, spermatogenesis associated 1	23885	https://www.ncbi.nlm.nih.gov/gene/?term=23885
Gabrb2	Gabrb2	gamma-aminobutyric acid (GABA) A receptor, subunit beta 2	14401	https://www.ncbi.nlm.nih.gov/gene/?term=14401

Biology Open • Supplementary information

Csmd1	Csmd1	CUB and Sushi multiple domains 1	94109	https://www.ncbi.nlm.nih.gov/gene/?term=94109
Lrp5	Lrp5	low density lipoprotein receptor-related protein 5	16973	https://www.ncbi.nlm.nih.gov/gene/?term=16973
Nampt	Nampt	nicotinamide phosphoribosyltransferase	59027	https://www.ncbi.nlm.nih.gov/gene/?term=59027
Atp6v1g1	Atp6v1g1	ATPase, H+ transporting, lysosomal V1 subunit G1	66290	https://www.ncbi.nlm.nih.gov/gene/?term=66290
Srebf1	Srebf1	sterol regulatory element binding transcription factor 1	20787	https://www.ncbi.nlm.nih.gov/gene/?term=20787
Lrig1	Lrig1	leucine-rich repeats and immunoglobulin-like domains 1	16206	https://www.ncbi.nlm.nih.gov/gene/?term=16206
Dennd4a	Dennd4a	DENN/MADD domain containing 4A	102442	https://www.ncbi.nlm.nih.gov/gene/?term=102442
Sh2d3c	Sh2d3c	SH2 domain containing 3C	27387	https://www.ncbi.nlm.nih.gov/gene/?term=27387
Daam2	Daam2	dishevelled associated activator of morphogenesis 2	76441	https://www.ncbi.nlm.nih.gov/gene/?term=76441
Pdc	Pdc	phosducin	20028	https://www.ncbi.nlm.nih.gov/gene/?term=20028
Arhgap44	Arhgap44	Rho GTPase activating protein 44	216831	https://www.ncbi.nlm.nih.gov/gene/?term=216831
Svip	Svip	small VCP/p97-interacting protein	75744	https://www.ncbi.nlm.nih.gov/gene/?term=75744
Rhot1	Rhot1	ras homolog family member T1	59040	https://www.ncbi.nlm.nih.gov/gene/?term=59040
Ankrd50	Ankrd50	ankyrin repeat domain 50	99696	https://www.ncbi.nlm.nih.gov/gene/?term=99696
Gpr63	Gpr63	G protein-coupled receptor 63	81006	https://www.ncbi.nlm.nih.gov/gene/?term=81006
Mef2c	Mef2c	myocyte enhancer factor 2C	17260	https://www.ncbi.nlm.nih.gov/gene/?term=17260
Cdh13	Cdh13	cadherin 13	12554	https://www.ncbi.nlm.nih.gov/gene/?term=12554
Basp1	Basp1	brain abundant, membrane attached signal protein 1	70350	https://www.ncbi.nlm.nih.gov/gene/?term=70350
Scaf11	Scaf11	SR-related CTD-associated factor 11	72193	https://www.ncbi.nlm.nih.gov/gene/?term=72193
Zfp384	Zfp384	zinc finger protein 384	269800	https://www.ncbi.nlm.nih.gov/gene/?term=269800

Biology Open • Supplementary information

Gtf2ird1	Gtf2ird1	general transcription factor II I repeat domain-containing 1	57080	https://www.ncbi.nlm.nih.gov/gene/?term=57080
Pard6b	Pard6b	par-6 family cell polarity regulator beta	58220	https://www.ncbi.nlm.nih.gov/gene/?term=58220
Dennd4c	Dennd4c	DENN/MADD domain containing 4C	329877	https://www.ncbi.nlm.nih.gov/gene/?term=329877
Snrpb2	Snrpb2	U2 small nuclear ribonucleoprotein B	20639	https://www.ncbi.nlm.nih.gov/gene/?term=20639
Ms4a4c	Ms4a4c	membrane-spanning 4-domains, subfamily A, member 4C	64380	https://www.ncbi.nlm.nih.gov/gene/?term=64380
Apoo	Apoo	apolipoprotein O	68316	https://www.ncbi.nlm.nih.gov/gene/?term=68316
Fam24b	Fam24b	family with sequence similarity 24 member B	69318	https://www.ncbi.nlm.nih.gov/gene/?term=69318
Ube2cbp	Ube2cbp	ubiquitin-conjugating enzyme E2C binding protein	70348	https://www.ncbi.nlm.nih.gov/gene/?term=70348
Gdnf	Gdnf	glial cell line derived neurotrophic factor	14573	https://www.ncbi.nlm.nih.gov/gene/?term=14573
Mbd5	Mbd5	methyl-CpG binding domain protein 5	109241	https://www.ncbi.nlm.nih.gov/gene/?term=109241
Fbxl22	Fbxl22	F-box and leucine-rich repeat protein 22	74165	https://www.ncbi.nlm.nih.gov/gene/?term=74165
Faxc	Faxc	failed axon connections homolog	76132	https://www.ncbi.nlm.nih.gov/gene/?term=76132
Rab5a	Rab5a	RAB5A, member RAS oncogene family	271457	https://www.ncbi.nlm.nih.gov/gene/?term=271457
Sat1	Sat1	spermidine/spermine N1-acetyl transferase 1	20229	https://www.ncbi.nlm.nih.gov/gene/?term=20229
Bcl7a	Bcl7a	B cell CLL/lymphoma 7A	77045	https://www.ncbi.nlm.nih.gov/gene/?term=77045
9530068E07Rik	9530068E07Rik	RIKEN cDNA 9530068E07 gene	213673	https://www.ncbi.nlm.nih.gov/gene/?term=213673
Pcgf2	Pcgf2	polycomb group ring finger 2	22658	https://www.ncbi.nlm.nih.gov/gene/?term=22658

Biology Open • Supplementary information

Antxr1	Antxr1	anthrax toxin receptor 1	69538	https://www.ncbi.nlm.nih.gov/gene/?term=69538
Dach1	Dach1	dachshund family transcription factor 1	13134	https://www.ncbi.nlm.nih.gov/gene/?term=13134
Cts8	Cts8	cathepsin 8	56094	https://www.ncbi.nlm.nih.gov/gene/?term=56094
Gm8369	Gm8369	predicted gene 8369	666926	https://www.ncbi.nlm.nih.gov/gene/?term=666926
Pitpnb	Pitpnb	phosphatidylinositol transfer protein, beta	56305	https://www.ncbi.nlm.nih.gov/gene/?term=56305
Brd7	Brd7	bromodomain containing 7	26992	https://www.ncbi.nlm.nih.gov/gene/?term=26992
Itgam	Itgam	integrin alpha M	16409	https://www.ncbi.nlm.nih.gov/gene/?term=16409
Foxo1	Foxo1	forkhead box O1	56458	https://www.ncbi.nlm.nih.gov/gene/?term=56458
Lrguk	Lrguk	leucine-rich repeats and guanylate kinase domain containing	74354	https://www.ncbi.nlm.nih.gov/gene/?term=74354
Itgb1	Itgb1	integrin beta 1 (fibronectin receptor beta)	16412	https://www.ncbi.nlm.nih.gov/gene/?term=16412
Cspp1	Cspp1	centrosome and spindle pole associated protein 1	211660	https://www.ncbi.nlm.nih.gov/gene/?term=211660
Nlgn3	Nlgn3	neuroligin 3	245537	https://www.ncbi.nlm.nih.gov/gene/?term=245537
Gad1	Gad1	glutamate decarboxylase 1	14415	https://www.ncbi.nlm.nih.gov/gene/?term=14415
Ahdc1	Ahdc1	AT hook, DNA binding motif, containing 1	230793	https://www.ncbi.nlm.nih.gov/gene/?term=230793
Faim	Faim	Fas apoptotic inhibitory molecule	23873	https://www.ncbi.nlm.nih.gov/gene/?term=23873
Axin1	Axin1	axin 1	12005	https://www.ncbi.nlm.nih.gov/gene/?term=12005
Lman1	Lman1	lectin, mannose-binding, 1	70361	https://www.ncbi.nlm.nih.gov/gene/?term=70361
Dld	Dld	dihydrolipoamide dehydrogenase	13382	https://www.ncbi.nlm.nih.gov/gene/?term=13382
Cog3	Cog3	component of oligomeric golgi complex 3	338337	https://www.ncbi.nlm.nih.gov/gene/?term=338337
Esx1	Esx1	extraembryonic, spermatogenesis, homeobox 1	13984	https://www.ncbi.nlm.nih.gov/gene/?term=13984

Biology Open • Supplementary information

Dhx36	Dhx36	DEAH (Asp-Glu-Ala-His) box polypeptide 36	72162	https://www.ncbi.nlm.nih.gov/gene/?term=72162
Ak5	Ak5	adenylate kinase 5	229949	https://www.ncbi.nlm.nih.gov/gene/?term=229949
M6pr	M6pr	mannose-6-phosphate receptor, cation dependent	17113	https://www.ncbi.nlm.nih.gov/gene/?term=17113
Tgfbrap1	Tgfbrap1	transforming growth factor, beta receptor associated protein 1	73122	https://www.ncbi.nlm.nih.gov/gene/?term=73122
Tubgcp4	Tubgcp4	tubulin, gamma complex associated protein 4	51885	https://www.ncbi.nlm.nih.gov/gene/?term=51885
Ints8	Ints8	integrator complex subunit 8	72656	https://www.ncbi.nlm.nih.gov/gene/?term=72656
Megf11	Megf11	multiple EGF-like-domains 11	214058	https://www.ncbi.nlm.nih.gov/gene/?term=214058
Ltbp3	Ltbp3	latent transforming growth factor beta binding protein 3	16998	https://www.ncbi.nlm.nih.gov/gene/?term=16998
Kif1a	Kif1a	kinesin family member 1A	16560	https://www.ncbi.nlm.nih.gov/gene/?term=16560
Lsm2	Lsm2	LSM2 homolog, U6 small nuclear RNA and mRNA degradation associated	27756	https://www.ncbi.nlm.nih.gov/gene/?term=27756
Rpain	Rpain	RPA interacting protein	69723	https://www.ncbi.nlm.nih.gov/gene/?term=69723
Pla2g12a	Pla2g12a	phospholipase A2, group XIIA	66350	https://www.ncbi.nlm.nih.gov/gene/?term=66350
Mdm2	Mdm2	transformed mouse 3T3 cell double minute 2	17246	https://www.ncbi.nlm.nih.gov/gene/?term=17246
Disp3	Disp3	dispatched RND transporter family member 3	242748	https://www.ncbi.nlm.nih.gov/gene/?term=242748
Plek2	Plek2	pleckstrin 2	27260	https://www.ncbi.nlm.nih.gov/gene/?term=27260
Mynn	Mynn	myoneurin	80732	https://www.ncbi.nlm.nih.gov/gene/?term=80732
Dctn5	Dctn5	dynactin 5	59288	https://www.ncbi.nlm.nih.gov/gene/?term=59288
Rflnb	Rflnb	refilin B	76566	https://www.ncbi.nlm.nih.gov/gene/?term=76566

Vamp1	Vamp1	vesicle-associated membrane protein 1	22317	https://www.ncbi.nlm.nih.gov/gene/?term=22317
Adamts14	Adamts14	a disintegrin-like and metallopeptidase (reprolysin type) with thrombospondin type 1 motif, 14	237360	https://www.ncbi.nlm.nih.gov/gene/?term=237360
Shisa9	Shisa9	shisa family member 9	72555	https://www.ncbi.nlm.nih.gov/gene/?term=72555
Zfp935	Zfp935	zinc finger protein 935	71508	https://www.ncbi.nlm.nih.gov/gene/?term=71508
Papola	Papola	poly (A) polymerase alpha	18789	https://www.ncbi.nlm.nih.gov/gene/?term=18789
Ctnnbip1	Ctnnbip1	catenin beta interacting protein 1	67087	https://www.ncbi.nlm.nih.gov/gene/?term=67087
Aatk	Aatk	apoptosis-associated tyrosine kinase	11302	https://www.ncbi.nlm.nih.gov/gene/?term=11302
Chd5	Chd5	chromodomain helicase DNA binding protein 5	269610	https://www.ncbi.nlm.nih.gov/gene/?term=269610
Ppm1h	Ppm1h	protein phosphatase 1H (PP2C domain containing)	319468	https://www.ncbi.nlm.nih.gov/gene/?term=319468
Phc1	Phc1	polyhomeotic 1	13619	https://www.ncbi.nlm.nih.gov/gene/?term=13619
Peg10	Peg10	paternally expressed 10	170676	https://www.ncbi.nlm.nih.gov/gene/?term=170676
Cdr2l	Cdr2l	cerebellar degeneration-related protein 2-like	237988	https://www.ncbi.nlm.nih.gov/gene/?term=237988
Tcp10c	Tcp10c	t-complex protein 10c	100041352	https://www.ncbi.nlm.nih.gov/gene/?term=100041
Cdx1	Cdx1	caudal type homeobox 1	12590	https://www.ncbi.nlm.nih.gov/gene/?term=12590
Tcp10b	Tcp10b	t-complex protein 10b	21462	https://www.ncbi.nlm.nih.gov/gene/?term=21462
Tcp10a	Tcp10a	t-complex protein 10a	21461	https://www.ncbi.nlm.nih.gov/gene/?term=21461
C130050O18Rik	C130050O18Rik	RIKEN cDNA C130050O18 gene	319772	https://www.ncbi.nlm.nih.gov/gene/?term=319772
Slc37a3	Slc37a3	solute carrier family 37 (glycerol-3-phosphate transporter), member 3	72144	https://www.ncbi.nlm.nih.gov/gene/?term=72144

Ppp1r7	Ppp1r7	protein phosphatase 1, regulatory subunit 7	66385	https://www.ncbi.nlm.nih.gov/gene/?term=66385
Prdx1	Prdx1	peroxiredoxin 1	18477	https://www.ncbi.nlm.nih.gov/gene/?term=18477
Pdcd7	Pdcd7	programmed cell death 7	50996	https://www.ncbi.nlm.nih.gov/gene/?term=50996
Plxnc1	Plxnc1	plexin C1	54712	https://www.ncbi.nlm.nih.gov/gene/?term=54712
Hyal1	Hyal1	hyaluronoglucosaminidase 1	15586	https://www.ncbi.nlm.nih.gov/gene/?term=15586
H6pd	H6pd	hexose-6-phosphate dehydrogenase (glucose 1-dehydrogenase)	100198	https://www.ncbi.nlm.nih.gov/gene/?term=100198
Rfc5	Rfc5	replication factor C (activator 1) 5	72151	https://www.ncbi.nlm.nih.gov/gene/?term=72151
Atp23	Atp23	ATP23 metallopeptidase and ATP synthase assembly factor homolog	68876	https://www.ncbi.nlm.nih.gov/gene/?term=68876
Zmat5	Zmat5	zinc finger, matrin type 5	67178	https://www.ncbi.nlm.nih.gov/gene/?term=67178
T	T	brachyury, T-box transcription factor T	20997	https://www.ncbi.nlm.nih.gov/gene/?term=20997
Ephb3	Ephb3	Eph receptor B3	13845	https://www.ncbi.nlm.nih.gov/gene/?term=13845
Npr3	Npr3	natriuretic peptide receptor 3	18162	https://www.ncbi.nlm.nih.gov/gene/?term=18162
Inpp4b	Inpp4b	inositol polyphosphate-4-phosphatase, type II	234515	https://www.ncbi.nlm.nih.gov/gene/?term=234515
Tmem258	Tmem258	transmembrane protein 258	69038	https://www.ncbi.nlm.nih.gov/gene/?term=69038
Gdpd4	Gdpd4	glycerophosphodiester phosphodiesterase domain containing 4	233537	https://www.ncbi.nlm.nih.gov/gene/?term=233537
Srp68	Srp68	signal recognition particle 68	217337	https://www.ncbi.nlm.nih.gov/gene/?term=217337
Triobp	Triobp	TRIO and F-actin binding protein	110253	https://www.ncbi.nlm.nih.gov/gene/?term=110253
Rai2	Rai2	retinoic acid induced 2	24004	https://www.ncbi.nlm.nih.gov/gene/?term=24004
Mecp2	Mecp2	methyl CpG binding protein 2	17257	https://www.ncbi.nlm.nih.gov/gene/?term=17257
Uncx	Uncx	UNC homeobox	22255	https://www.ncbi.nlm.nih.gov/gene/?term=22255

Biology Open • Supplementary information

Slc10a2	Slc10a2	solute carrier family 10, member 2	20494	https://www.ncbi.nlm.nih.gov/gene/?term=20494
Fam114a1	Fam114a1	family with sequence similarity 114, member A1	68303	https://www.ncbi.nlm.nih.gov/gene/?term=68303
Tceanc2	Tceanc2	transcription elongation factor A (SII) N-terminal and central domain containing 2	66526	https://www.ncbi.nlm.nih.gov/gene/?term=66526
AI593442	AI593442	expressed sequence AI593442	330941	https://www.ncbi.nlm.nih.gov/gene/?term=330941
Ap1g2	Ap1g2	adaptor protein complex AP-1, gamma 2 subunit	11766	https://www.ncbi.nlm.nih.gov/gene/?term=11766
Rasl11a	Rasl11a	RAS-like, family 11, member A	68895	https://www.ncbi.nlm.nih.gov/gene/?term=68895
Cox11	Cox11	cytochrome c oxidase assembly protein 11, copper chaperone	69802	https://www.ncbi.nlm.nih.gov/gene/?term=69802
Iqce	Iqce	IQ motif containing E	74239	https://www.ncbi.nlm.nih.gov/gene/?term=74239
Tmed6	Tmed6	transmembrane p24 trafficking protein 6	66269	https://www.ncbi.nlm.nih.gov/gene/?term=66269
Tstd2	Tstd2	thiosulfate sulfurtransferase (rhodanese)-like domain containing 2	272027	https://www.ncbi.nlm.nih.gov/gene/?term=272027
Ovca2	Ovca2	candidate tumor suppressor in ovarian cancer 2	246257	https://www.ncbi.nlm.nih.gov/gene/?term=246257
Larp1	Larp1	La ribonucleoprotein domain family, member 1	73158	https://www.ncbi.nlm.nih.gov/gene/?term=73158
Chchd3	Chchd3	coiled-coil-helix-coiled-coil-helix domain containing 3	66075	https://www.ncbi.nlm.nih.gov/gene/?term=66075
Nr2f2	Nr2f2	nuclear receptor subfamily 2, group F, member 2	11819	https://www.ncbi.nlm.nih.gov/gene/?term=11819
Grid1	Grid1	glutamate receptor, ionotropic, delta 1	14803	https://www.ncbi.nlm.nih.gov/gene/?term=14803

Biology Open • Supplementary information

Usp30	Usp30	ubiquitin specific peptidase 30	100756	https://www.ncbi.nlm.nih.gov/gene/?term=100756
Iars2	Iars2	isoleucine-tRNA synthetase 2, mitochondrial	381314	https://www.ncbi.nlm.nih.gov/gene/?term=381314
B4galnt2	B4galnt2	beta-1,4-N-acetyl-galactosaminyl transferase 2	14422	https://www.ncbi.nlm.nih.gov/gene/?term=14422
Tlnrd1	Tlnrd1	talin rod domain containing 1	80889	https://www.ncbi.nlm.nih.gov/gene/?term=80889
Fam98a	Fam98a	family with sequence similarity 98, member A	72722	https://www.ncbi.nlm.nih.gov/gene/?term=72722
Ccdc62	Ccdc62	coiled-coil domain containing 62	208908	https://www.ncbi.nlm.nih.gov/gene/?term=208908
Capn3	Capn3	calpain 3	12335	https://www.ncbi.nlm.nih.gov/gene/?term=12335
Fgf10	Fgf10	fibroblast growth factor 10	14165	https://www.ncbi.nlm.nih.gov/gene/?term=14165
Cntn4	Cntn4	contactin 4	269784	https://www.ncbi.nlm.nih.gov/gene/?term=269784
Appl1	Appl1	adaptor protein, phosphotyrosine interaction, PH domain and leucine zipper containing 1	72993	https://www.ncbi.nlm.nih.gov/gene/?term=72993
Lrrcc1	Lrrcc1	leucine rich repeat and coiled-coil domain containing 1	71710	https://www.ncbi.nlm.nih.gov/gene/?term=71710
Snrpn	Snrpn	small nuclear ribonucleoprotein N	20646	https://www.ncbi.nlm.nih.gov/gene/?term=20646
Nfib	Nfib	nuclear factor I/B	18028	https://www.ncbi.nlm.nih.gov/gene/?term=18028
Rbm39	Rbm39	RNA binding motif protein 39	170791	https://www.ncbi.nlm.nih.gov/gene/?term=170791
Nadk	Nadk	NAD kinase	192185	https://www.ncbi.nlm.nih.gov/gene/?term=192185
Gpr45	Gpr45	G protein-coupled receptor 45	93690	https://www.ncbi.nlm.nih.gov/gene/?term=93690
Rc3h2	Rc3h2	ring finger and CCCH-type zinc finger domains 2	319817	https://www.ncbi.nlm.nih.gov/gene/?term=319817
Slc26a3	Slc26a3	solute carrier family 26, member 3	13487	https://www.ncbi.nlm.nih.gov/gene/?term=13487

Biology Open • Supplementary information

Plekha8	Plekha8	pleckstrin homology domain containing, family A (phosphoinositide binding specific) member 8	231999	https://www.ncbi.nlm.nih.gov/gene/?term=231999
Asph	Asph	aspartate-beta-hydroxylase	65973	https://www.ncbi.nlm.nih.gov/gene/?term=65973
Sptbn1	Sptbn1	spectrin beta, non-erythrocytic 1	20742	https://www.ncbi.nlm.nih.gov/gene/?term=20742
Elp1	Elp1	elongator complex protein 1	230233	https://www.ncbi.nlm.nih.gov/gene/?term=230233
Zfp963	Zfp963	zinc finger protein 963	620419	https://www.ncbi.nlm.nih.gov/gene/?term=620419
Ccdc136	Ccdc136	coiled-coil domain containing 136	232664	https://www.ncbi.nlm.nih.gov/gene/?term=232664
Enthd1	Enthd1	ENTH domain containing 1	383075	https://www.ncbi.nlm.nih.gov/gene/?term=383075
Gm1604b	Gm1604b	predicted gene 1604b	381059	https://www.ncbi.nlm.nih.gov/gene/?term=381059
Arhgap5	Arhgap5	Rho GTPase activating protein 5	11855	https://www.ncbi.nlm.nih.gov/gene/?term=11855
Akap11	Akap11	A kinase (PRKA) anchor protein 11	219181	https://www.ncbi.nlm.nih.gov/gene/?term=219181
Dnaja2	Dnaja2	DnaJ heat shock protein family (Hsp40) member A2	56445	https://www.ncbi.nlm.nih.gov/gene/?term=56445
Bcap29	Bcap29	B cell receptor associated protein 29	12033	https://www.ncbi.nlm.nih.gov/gene/?term=12033
Pabpc4l	Pabpc4l	poly(A) binding protein, cytoplasmic 4-like	241989	https://www.ncbi.nlm.nih.gov/gene/?term=241989
Snurf	Snurf	SNRPN upstream reading frame	84704	https://www.ncbi.nlm.nih.gov/gene/?term=84704
Cab39	Cab39	calcium binding protein 39	12283	https://www.ncbi.nlm.nih.gov/gene/?term=12283
Socs5	Socs5	suppressor of cytokine signaling 5	56468	https://www.ncbi.nlm.nih.gov/gene/?term=56468
Mrps14	Mrps14	mitochondrial ribosomal protein S14	64659	https://www.ncbi.nlm.nih.gov/gene/?term=64659
Phox2b	Phox2b	paired-like homeobox 2b	18935	https://www.ncbi.nlm.nih.gov/gene/?term=18935
Rbm4b	Rbm4b	RNA binding motif protein 4B	66704	https://www.ncbi.nlm.nih.gov/gene/?term=66704

Biology Open • Supplementary information

Stt3b	Stt3b	STT3, subunit of the oligosaccharyltransferase complex, homolog B (S. cerevisiae)	68292	https://www.ncbi.nlm.nih.gov/gene/?term=68292
Gli3	Gli3	GLI-Kruppel family member GLI3	14634	https://www.ncbi.nlm.nih.gov/gene/?term=14634
Slc22a17	Slc22a17	solute carrier family 22 (organic cation transporter), member 17	59049	https://www.ncbi.nlm.nih.gov/gene/?term=59049
Ctdnep1	Ctdnep1	CTD nuclear envelope phosphatase 1	67181	https://www.ncbi.nlm.nih.gov/gene/?term=67181
Crebl2	Crebl2	cAMP responsive element binding protein-like 2	232430	https://www.ncbi.nlm.nih.gov/gene/?term=232430
Ccng1	Ccng1	cyclin G1	12450	https://www.ncbi.nlm.nih.gov/gene/?term=12450
Mex3a	Mex3a	mex3 RNA binding family member A	72640	https://www.ncbi.nlm.nih.gov/gene/?term=72640
Nr2f6	Nr2f6	nuclear receptor subfamily 2, group F, member 6	13864	https://www.ncbi.nlm.nih.gov/gene/?term=13864
Gja3	Gja3	gap junction protein, alpha 3	14611	https://www.ncbi.nlm.nih.gov/gene/?term=14611
Faf1	Faf1	Fas-associated factor 1	14084	https://www.ncbi.nlm.nih.gov/gene/?term=14084
Zkscan8	Zkscan8	zinc finger with KRAB and SCAN domains 8	93681	https://www.ncbi.nlm.nih.gov/gene/?term=93681
Mdga2	Mdga2	MAM domain containing glycosylphosphatidylinositol anchor 2	320772	https://www.ncbi.nlm.nih.gov/gene/?term=320772
Ifrd2	Ifrd2	interferon-related developmental regulator 2	15983	https://www.ncbi.nlm.nih.gov/gene/?term=15983
Wipf3	Wipf3	WAS/WASL interacting protein family, member 3	330319	https://www.ncbi.nlm.nih.gov/gene/?term=330319
Psmb11	Psmb11	proteasome (prosome, macropain) subunit, beta type, 11	73902	https://www.ncbi.nlm.nih.gov/gene/?term=73902

Biology Open • Supplementary information

Sorl1	Sorl1	sortilin-related receptor, LDLR class A repeats-containing	20660	https://www.ncbi.nlm.nih.gov/gene/?term=20660
Cacna2d3	Cacna2d3	calcium channel, voltage-dependent, alpha2/delta subunit 3	12294	https://www.ncbi.nlm.nih.gov/gene/?term=12294
Lurap1l	Lurap1l	leucine rich adaptor protein 1-like	52829	https://www.ncbi.nlm.nih.gov/gene/?term=52829
Ppme1	Ppme1	protein phosphatase methylesterase 1	72590	https://www.ncbi.nlm.nih.gov/gene/?term=72590
Gpr88	Gpr88	G-protein coupled receptor 88	64378	https://www.ncbi.nlm.nih.gov/gene/?term=64378
Pdia6	Pdia6	protein disulfide isomerase associated 6	71853	https://www.ncbi.nlm.nih.gov/gene/?term=71853
Nt5c2	Nt5c2	5'-nucleotidase, cytosolic II	76952	https://www.ncbi.nlm.nih.gov/gene/?term=76952
Spta1	Spta1	spectrin alpha, erythrocytic 1	20739	https://www.ncbi.nlm.nih.gov/gene/?term=20739
Eif4b	Eif4b	eukaryotic translation initiation factor 4B	75705	https://www.ncbi.nlm.nih.gov/gene/?term=75705
Zfp607b	Zfp607b	zinc finger protein 607B	112415	https://www.ncbi.nlm.nih.gov/gene/?term=112415
Serinc5	Serinc5	serine incorporator 5	218442	https://www.ncbi.nlm.nih.gov/gene/?term=218442
Dagla	Dagla	diacylglycerol lipase, alpha	269060	https://www.ncbi.nlm.nih.gov/gene/?term=269060
Itgb5	Itgb5	integrin beta 5	16419	https://www.ncbi.nlm.nih.gov/gene/?term=16419
Zfp407	Zfp407	zinc finger protein 407	240476	https://www.ncbi.nlm.nih.gov/gene/?term=240476
Slc7a12	Slc7a12	solute carrier family 7 (cationic amino acid transporter, y+ system), member 12	140918	https://www.ncbi.nlm.nih.gov/gene/?term=140918
Zfp563	Zfp563	zinc finger protein 563	240068	https://www.ncbi.nlm.nih.gov/gene/?term=240068
Ikbkg	Ikbkg	inhibitor of kappaB kinase gamma	16151	https://www.ncbi.nlm.nih.gov/gene/?term=16151
Ralgapb	Ralgapb	Ral GTPase activating protein, beta subunit (non-catalytic)	228850	https://www.ncbi.nlm.nih.gov/gene/?term=228850
Sgcd	Sgcd	sarcoglycan, delta (dystrophin-associated glycoprotein)	24052	https://www.ncbi.nlm.nih.gov/gene/?term=24052

Biology Open • Supplementary information

Gm13871	Gm13871	predicted gene 13871	667780	https://www.ncbi.nlm.nih.gov/gene/?term=667780
Rala	Rala	v-ral simian leukemia viral oncogene A (ras related)	56044	https://www.ncbi.nlm.nih.gov/gene/?term=56044
Dazap2	Dazap2	DAZ associated protein 2	23994	https://www.ncbi.nlm.nih.gov/gene/?term=23994
Hdac1	Hdac1	histone deacetylase 1	433759	https://www.ncbi.nlm.nih.gov/gene/?term=433759
Ehhadh	Ehhadh	enoyl-Coenzyme A, hydratase/3-hydroxyacyl Coenzyme A dehydrogenase	74147	https://www.ncbi.nlm.nih.gov/gene/?term=74147
Gm11757	Gm11757	predicted gene 11757	623272	https://www.ncbi.nlm.nih.gov/gene/?term=623272
Oxct1	Oxct1	3-oxoacid CoA transferase 1	67041	https://www.ncbi.nlm.nih.gov/gene/?term=67041
Lrtm2	Lrtm2	leucine-rich repeats and transmembrane domains 2	211187	https://www.ncbi.nlm.nih.gov/gene/?term=211187
Cobl	Cobl	cordon-bleu WH2 repeat	12808	https://www.ncbi.nlm.nih.gov/gene/?term=12808
Gm11758	Gm11758	predicted gene 11758	545637	https://www.ncbi.nlm.nih.gov/gene/?term=545637
Olfr976	Olfr976	olfactory receptor 976	258364	https://www.ncbi.nlm.nih.gov/gene/?term=258364
Kif3b	Kif3b	kinesin family member 3B	16569	https://www.ncbi.nlm.nih.gov/gene/?term=16569
Gm11236	Gm11236	predicted gene 11236	100862015	https://www.ncbi.nlm.nih.gov/gene/?term=100862
Nipa2	Nipa2	non imprinted in Prader-Willi/Angelman syndrome 2 homolog (human)	93790	https://www.ncbi.nlm.nih.gov/gene/?term=93790
Twist1	Twist1	twist basic helix-loop-helix transcription factor 1	22160	https://www.ncbi.nlm.nih.gov/gene/?term=22160
Sox14	Sox14	SRY (sex determining region Y)-box 14	20669	https://www.ncbi.nlm.nih.gov/gene/?term=20669
Rnf165	Rnf165	ring finger protein 165	225743	https://www.ncbi.nlm.nih.gov/gene/?term=225743
Gm428	Gm428	predicted gene 428	242502	https://www.ncbi.nlm.nih.gov/gene/?term=242502
Cbln1	Cbln1	cerebellin 1 precursor protein	12404	https://www.ncbi.nlm.nih.gov/gene/?term=12404

Biology Open • Supplementary information

Amz1	Amz1	archaelysin family metallopeptidase 1	231842	https://www.ncbi.nlm.nih.gov/gene/?term=231842
Mid1	Mid1	midline 1	17318	https://www.ncbi.nlm.nih.gov/gene/?term=17318
Gm38423	Gm38423	predicted gene, 38423	100502924	https://www.ncbi.nlm.nih.gov/gene/?term=100502
Gm11238	Gm11238	predicted gene 11238	100502896	https://www.ncbi.nlm.nih.gov/gene/?term=100502
Gm11237	Gm11237	predicted gene 11237	623197	https://www.ncbi.nlm.nih.gov/gene/?term=623197
Tmod3	Tmod3	tropomodulin 3	50875	https://www.ncbi.nlm.nih.gov/gene/?term=50875
Gm11239	Gm11239	predicted gene 11239	101055863	https://www.ncbi.nlm.nih.gov/gene/?term=101055
Ergic1	Ergic1	endoplasmic reticulum-golgi intermediate compartment (ERGIC) 1	67458	https://www.ncbi.nlm.nih.gov/gene/?term=67458
Man1c1	Man1c1	mannosidase, alpha, class 1C, member 1	230815	https://www.ncbi.nlm.nih.gov/gene/?term=230815
Cops8	Cops8	COP9 signalosome subunit 8	108679	https://www.ncbi.nlm.nih.gov/gene/?term=108679
Aff4	Aff4	AF4/FMR2 family, member 4	93736	https://www.ncbi.nlm.nih.gov/gene/?term=93736
Lrit1	Lrit1	leucine-rich repeat, immunoglobulin-like and transmembrane domains 1	239037	https://www.ncbi.nlm.nih.gov/gene/?term=239037
Csf2rb	Csf2rb	colony stimulating factor 2 receptor, beta, low-affinity (granulocyte-macrophage)	12983	https://www.ncbi.nlm.nih.gov/gene/?term=12983
Mical3	Mical3	microtubule associated monooxygenase, calponin and LIM domain containing 3	194401	https://www.ncbi.nlm.nih.gov/gene/?term=194401
Fads3	Fads3	fatty acid desaturase 3	60527	https://www.ncbi.nlm.nih.gov/gene/?term=60527
Tmcc2	Tmcc2	NA	68875	https://www.ncbi.nlm.nih.gov/gene/?term=68875
Sgo1	Sgo1	shugoshin 1	72415	https://www.ncbi.nlm.nih.gov/gene/?term=72415
Pisd	Pisd	phosphatidylserine decarboxylase	320951	https://www.ncbi.nlm.nih.gov/gene/?term=320951

Biology Open • Supplementary information

Pptc7	Pptc7	PTC7 protein phosphatase homolog	320717	https://www.ncbi.nlm.nih.gov/gene/?term=320717
Gpr62	Gpr62	G protein-coupled receptor 62	436090	https://www.ncbi.nlm.nih.gov/gene/?term=436090
Capn5	Capn5	calpain 5	12337	https://www.ncbi.nlm.nih.gov/gene/?term=12337
Mrgprg	Mrgprg	MAS-related GPR, member G	381974	https://www.ncbi.nlm.nih.gov/gene/?term=381974
Cd40	Cd40	CD40 antigen	21939	https://www.ncbi.nlm.nih.gov/gene/?term=21939
Cmc2	Cmc2	COX assembly mitochondrial protein 2	66531	https://www.ncbi.nlm.nih.gov/gene/?term=66531
Prpf39	Prpf39	pre-mRNA processing factor 39	328110	https://www.ncbi.nlm.nih.gov/gene/?term=328110
Anapc10	Anapc10	anaphase promoting complex subunit 10	68999	https://www.ncbi.nlm.nih.gov/gene/?term=68999
Emc7	Emc7	ER membrane protein complex subunit 7	73024	https://www.ncbi.nlm.nih.gov/gene/?term=73024
Rfesd	Rfesd	Rieske (Fe-S) domain containing	218341	https://www.ncbi.nlm.nih.gov/gene/?term=218341
Wdr44	Wdr44	WD repeat domain 44	72404	https://www.ncbi.nlm.nih.gov/gene/?term=72404
Ereg	Ereg	epiregulin	13874	https://www.ncbi.nlm.nih.gov/gene/?term=13874
Gm14295	Gm14295	predicted gene 14295	100039123	https://www.ncbi.nlm.nih.gov/gene/?term=100039[illegible]
Lcorl	Lcorl	ligand dependent nuclear receptor corepressor-like	209707	https://www.ncbi.nlm.nih.gov/gene/?term=209707
Ppwd1	Ppwd1	peptidylprolyl isomerase domain and WD repeat containing 1	238831	https://www.ncbi.nlm.nih.gov/gene/?term=238831

Biology Open • Supplementary information

Table S12. Genes predicted to be modulated by miRNAs differentially expressed between Lac and Inv.

userId	geneSymbol	geneName	entrezgene	gLink
Pdik1l	Pdik1l	PDLIM1 interacting kinase 1 like	230809	https://www.ncbi.nlm.nih.gov/gene/?term=230809
Smim4	Smim4	small integral membrane protein 4	66487	https://www.ncbi.nlm.nih.gov/gene/?term=66487
Strap	Strap	serine/threonine kinase receptor associated protein	20901	https://www.ncbi.nlm.nih.gov/gene/?term=20901
Mospd2	Mospd2	motile sperm domain containing 2	76763	https://www.ncbi.nlm.nih.gov/gene/?term=76763
Rdh10	Rdh10	retinol dehydrogenase 10 (all-trans)	98711	https://www.ncbi.nlm.nih.gov/gene/?term=98711
Zfand6	Zfand6	zinc finger, AN1-type domain 6	65098	https://www.ncbi.nlm.nih.gov/gene/?term=65098
Rusc2	Rusc2	RUN and SH3 domain containing 2	100213	https://www.ncbi.nlm.nih.gov/gene/?term=100213
Nbr1	Nbr1	NBR1, autophagy cargo receptor	17966	https://www.ncbi.nlm.nih.gov/gene/?term=17966

Biology Open • Supplementary information

Dek	Dek	DEK oncogene (DNA binding)	110052	https://www.ncbi.nlm.nih.gov/gene/?term=110052
Fli1	Fli1	Friend leukemia integration 1	14247	https://www.ncbi.nlm.nih.gov/gene/?term=14247
Cdk17	Cdk17	cyclin-dependent kinase 17	237459	https://www.ncbi.nlm.nih.gov/gene/?term=237459
Slc30a4	Slc30a4	solute carrier family 30 (zinc transporter), member 4	22785	https://www.ncbi.nlm.nih.gov/gene/?term=22785
Nr5a2	Nr5a2	nuclear receptor subfamily 5, group A, member 2	26424	https://www.ncbi.nlm.nih.gov/gene/?term=26424
Eloc	Eloc	elongin C	67923	https://www.ncbi.nlm.nih.gov/gene/?term=67923
Rims2	Rims2	regulating synaptic membrane exocytosis 2	116838	https://www.ncbi.nlm.nih.gov/gene/?term=116838
Xkr4	Xkr4	X-linked Kx blood group related 4	497097	https://www.ncbi.nlm.nih.gov/gene/?term=497097
Wapl	Wapl	WAPL cohesin release factor	218914	https://www.ncbi.nlm.nih.gov/gene/?term=218914

Biology Open • Supplementary information

Mosmo	Mosmo	modulator of smoothened	233812	https://www.ncbi.nlm.nih.gov/gene/?term=233812
Cntn1	Cntn1	contactin 1	12805	https://www.ncbi.nlm.nih.gov/gene/?term=12805
Pfpl	Pfpl	pore forming protein-like	56093	https://www.ncbi.nlm.nih.gov/gene/?term=56093
Lhfp	Lhfp	lipoma HMGIC fusion partner	108927	https://www.ncbi.nlm.nih.gov/gene/?term=108927
Slit2	Slit2	slit guidance ligand 2	20563	https://www.ncbi.nlm.nih.gov/gene/?term=20563
Rab11fip2	Rab11fip2	RAB11 family interacting protein 2 (class I)	74998	https://www.ncbi.nlm.nih.gov/gene/?term=74998
Pom121l12	Pom121l12	POM121 membrane glycoprotein-like 12	432536	https://www.ncbi.nlm.nih.gov/gene/?term=432536
Pcmtd1	Pcmtd1	protein-L-isoaspartate (D-aspartate) O-methyltransferase domain containing 1	319263	https://www.ncbi.nlm.nih.gov/gene/?term=319263
Dlc1	Dlc1	deleted in liver cancer 1	50768	https://www.ncbi.nlm.nih.gov/gene/?term=50768

Biology Open • Supplementary information

Pds5b	Pds5b	PDS5 cohesin associated factor B	100710	https://www.ncbi.nlm.nih.gov/gene/?term=100710
Arhgap20	Arhgap20	Rho GTPase activating protein 20	244867	https://www.ncbi.nlm.nih.gov/gene/?term=244867
Hmbox1	Hmbox1	homeobox containing 1	219150	https://www.ncbi.nlm.nih.gov/gene/?term=219150
Thap1	Thap1	THAP domain containing, apoptosis associated protein 1	73754	https://www.ncbi.nlm.nih.gov/gene/?term=73754
Qk	Qk	quaking	19317	https://www.ncbi.nlm.nih.gov/gene/?term=19317
Atp11c	Atp11c	ATPase, class VI, type 11C	320940	https://www.ncbi.nlm.nih.gov/gene/?term=320940
Zfp131	Zfp131	zinc finger protein 131	72465	https://www.ncbi.nlm.nih.gov/gene/?term=72465
Fez2	Fez2	fasciculation and elongation protein zeta 2 (zygin II)	225020	https://www.ncbi.nlm.nih.gov/gene/?term=225020
Sulf1	Sulf1	sulfatase 1	240725	https://www.ncbi.nlm.nih.gov/gene/?term=240725

Cnot6	Cnot6	CCR4-NOT transcription complex, subunit 6	104625	https://www.ncbi.nlm.nih.gov/gene/?term=104625
Golga1	Golga1	golgi autoantigen, golgin subfamily a, 1	76899	https://www.ncbi.nlm.nih.gov/gene/?term=76899
Elmod2	Elmod2	ELMO/CED-12 domain containing 2	244548	https://www.ncbi.nlm.nih.gov/gene/?term=244548
Rap1b	Rap1b	RAS related protein 1b	215449	https://www.ncbi.nlm.nih.gov/gene/?term=215449
Rab21	Rab21	RAB21, member RAS oncogene family	216344	https://www.ncbi.nlm.nih.gov/gene/?term=216344
Phf11d	Phf11d	PHD finger protein 11D	219132	https://www.ncbi.nlm.nih.gov/gene/?term=219132
Khdrbs1	Khdrbs1	KH domain containing, RNA binding, signal transduction associated 1	20218	https://www.ncbi.nlm.nih.gov/gene/?term=20218
Tsc22d2	Tsc22d2	TSC22 domain family, member 2	72033	https://www.ncbi.nlm.nih.gov/gene/?term=72033
Chrdl1	Chrdl1	chordin-like 1	83453	https://www.ncbi.nlm.nih.gov/gene/?term=83453

Biology Open • Supplementary information

Zfp711	Zfp711	zinc finger protein 711	245595	https://www.ncbi.nlm.nih.gov/gene/?term=245595
Rnd3	Rnd3	Rho family GTPase 3	74194	https://www.ncbi.nlm.nih.gov/gene/?term=74194
Slitrk1	Slitrk1	SLIT and NTRK-like family, member 1	76965	https://www.ncbi.nlm.nih.gov/gene/?term=76965
Immp2l	Immp2l	IMP2 inner mitochondrial membrane peptidase-like (S. cerevisiae)	93757	https://www.ncbi.nlm.nih.gov/gene/?term=93757
Fbxo33	Fbxo33	F-box protein 33	70611	https://www.ncbi.nlm.nih.gov/gene/?term=70611
Tfap2a	Tfap2a	transcription factor AP-2, alpha	21418	https://www.ncbi.nlm.nih.gov/gene/?term=21418
Ap1s2	Ap1s2	adaptor-related protein complex 1, sigma 2 subunit	108012	https://www.ncbi.nlm.nih.gov/gene/?term=108012
Spryd7	Spryd7	SPRY domain containing 7	66674	https://www.ncbi.nlm.nih.gov/gene/?term=66674
Tjp1	Tjp1	tight junction protein 1	21872	https://www.ncbi.nlm.nih.gov/gene/?term=21872

Biology Open • Supplementary information

Ppp1r9b	Ppp1r9b	protein phosphatase 1, regulatory subunit 9B	217124	https://www.ncbi.nlm.nih.gov/gene/?term=217124
Gpr161	Gpr161	G protein-coupled receptor 161	240888	https://www.ncbi.nlm.nih.gov/gene/?term=240888
Fndc3b	Fndc3b	fibronectin type III domain containing 3B	72007	https://www.ncbi.nlm.nih.gov/gene/?term=72007
Hdhd2	Hdhd2	haloacid dehalogenase-like hydrolase domain containing 2	76987	https://www.ncbi.nlm.nih.gov/gene/?term=76987
Cfl2	Cfl2	cofilin 2, muscle	12632	https://www.ncbi.nlm.nih.gov/gene/?term=12632
Ywhag	Ywhag	tyrosine 3-monooxygenase/tryptophan 5-monooxygenase activation protein, gamma polypeptide	22628	https://www.ncbi.nlm.nih.gov/gene/?term=22628
Desi2	Desi2	desumoylating isopeptidase 2	78825	https://www.ncbi.nlm.nih.gov/gene/?term=78825
Dennd5a	Dennd5a	DENN/MADD domain containing 5A	19347	https://www.ncbi.nlm.nih.gov/gene/?term=19347
Gpm6a	Gpm6a	glycoprotein m6a	234267	https://www.ncbi.nlm.nih.gov/gene/?term=234267

Biology Open • Supplementary information

Adamts6	Adamts6	a disintegrin-like and metallopeptidase (reprolysin type) with thrombospondin type 1 motif, 6	108154	https://www.ncbi.nlm.nih.gov/gene/?term=108154
Atxn1	Atxn1	ataxin 1	20238	https://www.ncbi.nlm.nih.gov/gene/?term=20238
Osbpl11	Osbpl11	oxysterol binding protein-like 11	106326	https://www.ncbi.nlm.nih.gov/gene/?term=106326
Sec23a	Sec23a	SEC23 homolog A, COPII coat complex component	20334	https://www.ncbi.nlm.nih.gov/gene/?term=20334
Ptpn21	Ptpn21	protein tyrosine phosphatase, non-receptor type 21	24000	https://www.ncbi.nlm.nih.gov/gene/?term=24000
Sema6d	Sema6d	sema domain, transmembrane domain (TM), and cytoplasmic domain, (semaphorin) 6D	214968	https://www.ncbi.nlm.nih.gov/gene/?term=214968
Hipk3	Hipk3	homeodomain interacting protein kinase 3	15259	https://www.ncbi.nlm.nih.gov/gene/?term=15259
Fignl2	Fignl2	fidgetin-like 2	668225	https://www.ncbi.nlm.nih.gov/gene/?term=668225
Pi4kb	Pi4kb	phosphatidylinositol 4-kinase beta	107650	https://www.ncbi.nlm.nih.gov/gene/?term=107650

Biology Open • Supplementary information

Pik3cb	Pik3cb	phosphatidylinositol-4,5-bisphosphate 3-kinase catalytic subunit beta	74769	https://www.ncbi.nlm.nih.gov/gene/?term=74769
Lrp1b	Lrp1b	low density lipoprotein-related protein 1B (deleted in tumors)	94217	https://www.ncbi.nlm.nih.gov/gene/?term=94217
Fbxw7	Fbxw7	F-box and WD-40 domain protein 7	50754	https://www.ncbi.nlm.nih.gov/gene/?term=50754
Gpatch8	Gpatch8	G patch domain containing 8	237943	https://www.ncbi.nlm.nih.gov/gene/?term=237943
Nr3c1	Nr3c1	nuclear receptor subfamily 3, group C, member 1	14815	https://www.ncbi.nlm.nih.gov/gene/?term=14815
Erg	Erg	ETS transcription factor	13876	https://www.ncbi.nlm.nih.gov/gene/?term=13876
Vash2	Vash2	vasohibin 2	226841	https://www.ncbi.nlm.nih.gov/gene/?term=226841
Synj1	Synj1	synaptojanin 1	104015	https://www.ncbi.nlm.nih.gov/gene/?term=104015
Evi5	Evi5	ecotropic viral integration site 5	14020	https://www.ncbi.nlm.nih.gov/gene/?term=14020

Biology Open • Supplementary information

Elmod1	Elmod1	ELMO/CED-12 domain containing 1	270162	https://www.ncbi.nlm.nih.gov/gene/?term=270162
Syncrip	Syncrip	synaptotagmin binding, cytoplasmic RNA interacting protein	56403	https://www.ncbi.nlm.nih.gov/gene/?term=56403
Tubb5	Tubb5	tubulin, beta 5 class I	22154	https://www.ncbi.nlm.nih.gov/gene/?term=22154
Ppm1f	Ppm1f	protein phosphatase 1F (PP2C domain containing)	68606	https://www.ncbi.nlm.nih.gov/gene/?term=68606
Dpy19l1	Dpy19l1	dpy-19-like 1 (C. elegans)	244745	https://www.ncbi.nlm.nih.gov/gene/?term=244745
Mprip	Mprip	myosin phosphatase Rho interacting protein	26936	https://www.ncbi.nlm.nih.gov/gene/?term=26936
Ankrd45	Ankrd45	ankyrin repeat domain 45	73844	https://www.ncbi.nlm.nih.gov/gene/?term=73844
Plcl1	Plcl1	phospholipase C-like 1	227120	https://www.ncbi.nlm.nih.gov/gene/?term=227120
Dennd5b	Dennd5b	DENN/MADD domain containing 5B	320560	https://www.ncbi.nlm.nih.gov/gene/?term=320560

Biology Open • Supplementary information

Rps6kb1	Rps6kb1	ribosomal protein S6 kinase, polypeptide 1	72508	https://www.ncbi.nlm.nih.gov/gene/?term=72508
Bag5	Bag5	BCL2-associated athanogene 5	70369	https://www.ncbi.nlm.nih.gov/gene/?term=70369
Med13	Med13	mediator complex subunit 13	327987	https://www.ncbi.nlm.nih.gov/gene/?term=327987
Rbsn	Rbsn	rabenosyn, RAB effector	78287	https://www.ncbi.nlm.nih.gov/gene/?term=78287
Prr11	Prr11	proline rich 11	270906	https://www.ncbi.nlm.nih.gov/gene/?term=270906
Foxp1	Foxp1	forkhead box P1	108655	https://www.ncbi.nlm.nih.gov/gene/?term=108655
Sox5	Sox5	SRY (sex determining region Y)-box 5	20678	https://www.ncbi.nlm.nih.gov/gene/?term=20678
Wipf1	Wipf1	WAS/WASL interacting protein family, member 1	215280	https://www.ncbi.nlm.nih.gov/gene/?term=215280
Vash1	Vash1	vasohibin 1	238328	https://www.ncbi.nlm.nih.gov/gene/?term=238328

Biology Open • Supplementary information

Nrf1	Nrf1	nuclear respiratory factor 1	18181	https://www.ncbi.nlm.nih.gov/gene/?term=18181
Cdh20	Cdh20	cadherin 20	23836	https://www.ncbi.nlm.nih.gov/gene/?term=23836
Ago2	Ago2	argonaute RISC catalytic subunit 2	239528	https://www.ncbi.nlm.nih.gov/gene/?term=239528
St6galnac5	St6galnac5	ST6 (alpha-N-acetyl-neuraminyl-2,3-beta-galactosyl-1,3)-N-acetylgalactosaminide alpha-2,6-sialyltransferase 5	26938	https://www.ncbi.nlm.nih.gov/gene/?term=26938
Jun	Jun	jun proto-oncogene	16476	https://www.ncbi.nlm.nih.gov/gene/?term=16476
Ypel2	Ypel2	yippee like 2	77864	https://www.ncbi.nlm.nih.gov/gene/?term=77864
Nfia	Nfia	nuclear factor I/A	18027	https://www.ncbi.nlm.nih.gov/gene/?term=18027
Zeb2	Zeb2	zinc finger E-box binding homeobox 2	24136	https://www.ncbi.nlm.nih.gov/gene/?term=24136
Rasa2	Rasa2	RAS p21 protein activator 2	114713	https://www.ncbi.nlm.nih.gov/gene/?term=114713

Cd302	Cd302	CD302 antigen	66205	https://www.ncbi.nlm.nih.gov/gene/?term=66205
Phf21b	Phf21b	PHD finger protein 21B	271305	https://www.ncbi.nlm.nih.gov/gene/?term=271305
Lats2	Lats2	large tumor suppressor 2	50523	https://www.ncbi.nlm.nih.gov/gene/?term=50523
Slc14a1	Slc14a1	solute carrier family 14 (urea transporter), member 1	108052	https://www.ncbi.nlm.nih.gov/gene/?term=108052
Esrrg	Esrrg	estrogen-related receptor gamma	26381	https://www.ncbi.nlm.nih.gov/gene/?term=26381
Map2	Map2	microtubule-associated protein 2	17756	https://www.ncbi.nlm.nih.gov/gene/?term=17756
Ric1	Ric1	RAB6A GEF complex partner 1	226089	https://www.ncbi.nlm.nih.gov/gene/?term=226089
Tbc1d12	Tbc1d12	TBC1D12: TBC1 domain family, member 12	209478	https://www.ncbi.nlm.nih.gov/gene/?term=209478
Egln1	Egln1	egl-9 family hypoxia-inducible factor 1	112405	https://www.ncbi.nlm.nih.gov/gene/?term=112405

Biology Open • Supplementary information

Crtap	Crtap	cartilage associated protein	56693	https://www.ncbi.nlm.nih.gov/gene/?term=56693
Ddx3y	Ddx3y	DEAD (Asp-Glu-Ala-Asp) box polypeptide 3, Y-linked	26900	https://www.ncbi.nlm.nih.gov/gene/?term=26900
Uba6	Uba6	ubiquitin-like modifier activating enzyme 6	231380	https://www.ncbi.nlm.nih.gov/gene/?term=231380
Nova1	Nova1	neuro-oncological ventral antigen 1	664883	https://www.ncbi.nlm.nih.gov/gene/?term=664883
Fhl1	Fhl1	four and a half LIM domains 1	14199	https://www.ncbi.nlm.nih.gov/gene/?term=14199
Crkl	Crkl	v-crk avian sarcoma virus CT10 oncogene homolog-like	12929	https://www.ncbi.nlm.nih.gov/gene/?term=12929
Ptpn12	Ptpn12	protein tyrosine phosphatase, non-receptor type 12	19248	https://www.ncbi.nlm.nih.gov/gene/?term=19248
Asap1	Asap1	ArfGAP with SH3 domain, ankyrin repeat and PH domain1	13196	https://www.ncbi.nlm.nih.gov/gene/?term=13196
Tbk1	Tbk1	TANK-binding kinase 1	56480	https://www.ncbi.nlm.nih.gov/gene/?term=56480

Hook3	Hook3	hook microtubule tethering protein 3	320191	https://www.ncbi.nlm.nih.gov/gene/?term=320191
Tob1	Tob1	transducer of ErbB-2.1	22057	https://www.ncbi.nlm.nih.gov/gene/?term=22057
Slc38a2	Slc38a2	solute carrier family 38, member 2	67760	https://www.ncbi.nlm.nih.gov/gene/?term=67760
Amfr	Amfr	autocrine motility factor receptor	23802	https://www.ncbi.nlm.nih.gov/gene/?term=23802
Golga7	Golga7	golgi autoantigen, golgin subfamily a, 7	57437	https://www.ncbi.nlm.nih.gov/gene/?term=57437
Aff3	Aff3	AF4/FMR2 family, member 3	16764	https://www.ncbi.nlm.nih.gov/gene/?term=16764
Slc35a2	Slc35a2	solute carrier family 35 (UDP-galactose transporter), member A2	22232	https://www.ncbi.nlm.nih.gov/gene/?term=22232
Trim33	Trim33	tripartite motif-containing 33	94093	https://www.ncbi.nlm.nih.gov/gene/?term=94093
Errfi1	Errfi1	ERBB receptor feedback inhibitor 1	74155	https://www.ncbi.nlm.nih.gov/gene/?term=74155

Biology Open • Supplementary information

Msn	Msn	moesin	17698	https://www.ncbi.nlm.nih.gov/gene/?term=17698
Zeb1	Zeb1	zinc finger E-box binding homeobox 1	21417	https://www.ncbi.nlm.nih.gov/gene/?term=21417
Jazf1	Jazf1	JAZF zinc finger 1	231986	https://www.ncbi.nlm.nih.gov/gene/?term=231986
Mmd	Mmd	monocyte to macrophage differentiation-associated	67468	https://www.ncbi.nlm.nih.gov/gene/?term=67468
Rap2c	Rap2c	RAP2C, member of RAS oncogene family	72065	https://www.ncbi.nlm.nih.gov/gene/?term=72065
Rnf169	Rnf169	ring finger protein 169	108937	https://www.ncbi.nlm.nih.gov/gene/?term=108937
Phf11b	Phf11b	PHD finger protein 11B	236451	https://www.ncbi.nlm.nih.gov/gene/?term=236451
Tent4b	Tent4b	terminal nucleotidyltransferase 4B	214627	https://www.ncbi.nlm.nih.gov/gene/?term=214627
Spag9	Spag9	sperm associated antigen 9	70834	https://www.ncbi.nlm.nih.gov/gene/?term=70834

Biology Open • Supplementary information

Unc5d	Unc5d	unc-5 netrin receptor D	210801	https://www.ncbi.nlm.nih.gov/gene/?term=210801
2700081O15Rik	2700081O15Rik	RIKEN cDNA 2700081O15 gene	108899	https://www.ncbi.nlm.nih.gov/gene/?term=108899
Agfg1	Agfg1	ArfGAP with FG repeats 1	15463	https://www.ncbi.nlm.nih.gov/gene/?term=15463
Ccnyl1	Ccnyl1	cyclin Y-like 1	227210	https://www.ncbi.nlm.nih.gov/gene/?term=227210
Bap1	Bap1	Brca1 associated protein 1	104416	https://www.ncbi.nlm.nih.gov/gene/?term=104416
Eps8	Eps8	epidermal growth factor receptor pathway substrate 8	13860	https://www.ncbi.nlm.nih.gov/gene/?term=13860
Igsf10	Igsf10	immunoglobulin superfamily, member 10	242050	https://www.ncbi.nlm.nih.gov/gene/?term=242050
Rbfox2	Rbfox2	RNA binding protein, fox-1 homolog (C. elegans) 2	93686	https://www.ncbi.nlm.nih.gov/gene/?term=93686
Scd1	Scd1	stearoyl-Coenzyme A desaturase 1	20249	https://www.ncbi.nlm.nih.gov/gene/?term=20249

Biology Open • Supplementary information

Pkd1	Pkd1	polycystin 1, transient receptor poteintial channel interacting	18763	https://www.ncbi.nlm.nih.gov/gene/?term=18763
Mboat2	Mboat2	membrane bound O-acyltransferase domain containing 2	67216	https://www.ncbi.nlm.nih.gov/gene/?term=67216
Ano5	Ano5	anoctamin 5	233246	https://www.ncbi.nlm.nih.gov/gene/?term=233246
Ranbp9	Ranbp9	RAN binding protein 9	56705	https://www.ncbi.nlm.nih.gov/gene/?term=56705
Arih1	Arih1	ariadne RBR E3 ubiquitin protein ligase 1	23806	https://www.ncbi.nlm.nih.gov/gene/?term=23806
Clasp1	Clasp1	CLIP associating protein 1	76707	https://www.ncbi.nlm.nih.gov/gene/?term=76707
Cecr2	Cecr2	CECR2, histone acetyl-lysine reader	330409	https://www.ncbi.nlm.nih.gov/gene/?term=330409
Mindy2	Mindy2	MINDY lysine 48 deubiquitinase 2	235461	https://www.ncbi.nlm.nih.gov/gene/?term=235461
Mgat2	Mgat2	mannoside acetylglucosaminyltransferase 2	217664	https://www.ncbi.nlm.nih.gov/gene/?term=217664

1810013L24Rik	1810013L24Rik	RIKEN cDNA 1810013L24 gene	69053	https://www.ncbi.nlm.nih.gov/gene/?term=69053
Hnrnpf	Hnrnpf	heterogeneous nuclear ribonucleoprotein F	98758	https://www.ncbi.nlm.nih.gov/gene/?term=98758
Lpar1	Lpar1	lysophosphatidic acid receptor 1	14745	https://www.ncbi.nlm.nih.gov/gene/?term=14745
Oosp3	Oosp3	oocyte secreted protein 3	225923	https://www.ncbi.nlm.nih.gov/gene/?term=225923
Gucy1a1	Gucy1a1	guanylate cyclase 1, soluble, alpha 1	60596	https://www.ncbi.nlm.nih.gov/gene/?term=60596
Mybl1	Mybl1	myeloblastosis oncogene-like 1	17864	https://www.ncbi.nlm.nih.gov/gene/?term=17864
Ntng1	Ntng1	netrin G1	80883	https://www.ncbi.nlm.nih.gov/gene/?term=80883
Dusp3	Dusp3	dual specificity phosphatase 3 (vaccinia virus phosphatase VH1-related)	72349	https://www.ncbi.nlm.nih.gov/gene/?term=72349
Mbnl1	Mbnl1	muscleblind like splicing factor 1	56758	https://www.ncbi.nlm.nih.gov/gene/?term=56758

Biology Open • Supplementary information

Arfgef3	Arfgef3	ARFGEF family member 3	215821	https://www.ncbi.nlm.nih.gov/gene/?term=215821
Rnf145	Rnf145	ring finger protein 145	74315	https://www.ncbi.nlm.nih.gov/gene/?term=74315
Myt1l	Myt1l	myelin transcription factor 1-like	17933	https://www.ncbi.nlm.nih.gov/gene/?term=17933
Kif3a	Kif3a	kinesin family member 3A	16568	https://www.ncbi.nlm.nih.gov/gene/?term=16568
Dr1	Dr1	down-regulator of transcription 1	13486	https://www.ncbi.nlm.nih.gov/gene/?term=13486
Cyth3	Cyth3	cytohesin 3	19159	https://www.ncbi.nlm.nih.gov/gene/?term=19159
Pcdh9	Pcdh9	protocadherin 9	211712	https://www.ncbi.nlm.nih.gov/gene/?term=211712
Dmwd	Dmwd	dystrophia myotonica-containing WD repeat motif	13401	https://www.ncbi.nlm.nih.gov/gene/?term=13401
Fbxl2	Fbxl2	F-box and leucine-rich repeat protein 2	72179	https://www.ncbi.nlm.nih.gov/gene/?term=72179

Biology Open • Supplementary information

Ulk2	Ulk2	unc-51 like kinase 2	29869	https://www.ncbi.nlm.nih.gov/gene/?term=29869
Cyp26b1	Cyp26b1	cytochrome P450, family 26, subfamily b, polypeptide 1	232174	https://www.ncbi.nlm.nih.gov/gene/?term=232174
Ccne2	Ccne2	cyclin E2	12448	https://www.ncbi.nlm.nih.gov/gene/?term=12448
Myh10	Myh10	myosin, heavy polypeptide 10, non-muscle	77579	https://www.ncbi.nlm.nih.gov/gene/?term=77579
Gpc2	Gpc2	glypican 2 (cerebroglycan)	71951	https://www.ncbi.nlm.nih.gov/gene/?term=71951
Simc1	Simc1	SUMO-interacting motifs containing 1	319719	https://www.ncbi.nlm.nih.gov/gene/?term=319719
Sacm1l	Sacm1l	SAC1 suppressor of actin mutations 1-like (yeast)	83493	https://www.ncbi.nlm.nih.gov/gene/?term=83493
Klhl28	Klhl28	kelch-like 28	66689	https://www.ncbi.nlm.nih.gov/gene/?term=66689
Osbpl6	Osbpl6	oxysterol binding protein-like 6	99031	https://www.ncbi.nlm.nih.gov/gene/?term=99031

Biology Open • Supplementary information

Yaf2	Yaf2	YY1 associated factor 2	67057	https://www.ncbi.nlm.nih.gov/gene/?term=67057
Hs2st1	Hs2st1	heparan sulfate 2-O-sulfotransferase 1	23908	https://www.ncbi.nlm.nih.gov/gene/?term=23908
Myadm	Myadm	myeloid-associated differentiation marker	50918	https://www.ncbi.nlm.nih.gov/gene/?term=50918
Zfr	Zfr	zinc finger RNA binding protein	22763	https://www.ncbi.nlm.nih.gov/gene/?term=22763
Crebrf	Crebrf	CREB3 regulatory factor	77128	https://www.ncbi.nlm.nih.gov/gene/?term=77128
Epn1	Epn1	epsin 1	13854	https://www.ncbi.nlm.nih.gov/gene/?term=13854
Mindy3	Mindy3	MINDY lysine 48 deubiquitinase 3	66960	https://www.ncbi.nlm.nih.gov/gene/?term=66960
Rsad2	Rsad2	radical S-adenosyl methionine domain containing 2	58185	https://www.ncbi.nlm.nih.gov/gene/?term=58185
Cryz	Cryz	crystallin, zeta	12972	https://www.ncbi.nlm.nih.gov/gene/?term=12972

Biology Open • Supplementary information

Ikzf2	Ikzf2	IKAROS family zinc finger 2	22779	https://www.ncbi.nlm.nih.gov/gene/?term=22779
Dcp2	Dcp2	decapping mRNA 2	70640	https://www.ncbi.nlm.nih.gov/gene/?term=70640
Crlf3	Crlf3	cytokine receptor-like factor 3	54394	https://www.ncbi.nlm.nih.gov/gene/?term=54394
Rnft1	Rnft1	ring finger protein, transmembrane 1	76892	https://www.ncbi.nlm.nih.gov/gene/?term=76892
Dstyk	Dstyk	dual serine/threonine and tyrosine protein kinase	213452	https://www.ncbi.nlm.nih.gov/gene/?term=213452
Tcf12	Tcf12	transcription factor 12	21406	https://www.ncbi.nlm.nih.gov/gene/?term=21406
Ube3a	Ube3a	ubiquitin protein ligase E3A	22215	https://www.ncbi.nlm.nih.gov/gene/?term=22215
Ythdf2	Ythdf2	YTH N6-methyladenosine RNA binding protein 2	213541	https://www.ncbi.nlm.nih.gov/gene/?term=213541
St3gal3	St3gal3	ST3 beta-galactoside alpha-2,3-sialyltransferase 3	20441	https://www.ncbi.nlm.nih.gov/gene/?term=20441

Iqschfp	Iqschfp	Iqcj and Schip1 fusion protein	100505386	https://www.ncbi.nlm.nih.gov/gene/?term=100505
4921524J17Rik	4921524J17Rik	RIKEN cDNA 4921524J17 gene	66714	https://www.ncbi.nlm.nih.gov/gene/?term=66714
Supt6	Supt6	suppressor of Ty 6	20926	https://www.ncbi.nlm.nih.gov/gene/?term=20926
Tgfb2	Tgfb2	transforming growth factor, beta 2	21808	https://www.ncbi.nlm.nih.gov/gene/?term=21808
Mrpl17	Mrpl17	mitochondrial ribosomal protein L17	27397	https://www.ncbi.nlm.nih.gov/gene/?term=27397
Pgrmc2	Pgrmc2	progesterone receptor membrane component 2	70804	https://www.ncbi.nlm.nih.gov/gene/?term=70804
Prkacb	Prkacb	protein kinase, cAMP dependent, catalytic, beta	18749	https://www.ncbi.nlm.nih.gov/gene/?term=18749
Chd2	Chd2	chromodomain helicase DNA binding protein 2	244059	https://www.ncbi.nlm.nih.gov/gene/?term=244059
Nkd1	Nkd1	naked cuticle 1	93960	https://www.ncbi.nlm.nih.gov/gene/?term=93960

Biology Open • Supplementary information

Sobp	Sobp	sine oculis binding protein	109205	https://www.ncbi.nlm.nih.gov/gene/?term=109205
Wdfy3	Wdfy3	WD repeat and FYVE domain containing 3	72145	https://www.ncbi.nlm.nih.gov/gene/?term=72145
Trhde	Trhde	TRH-degrading enzyme	237553	https://www.ncbi.nlm.nih.gov/gene/?term=237553
Ccdc80	Ccdc80	coiled-coil domain containing 80	67896	https://www.ncbi.nlm.nih.gov/gene/?term=67896
Zbtb34	Zbtb34	zinc finger and BTB domain containing 34	241311	https://www.ncbi.nlm.nih.gov/gene/?term=241311
Lysmd3	Lysmd3	LysM, putative peptidoglycan-binding, domain containing 3	80289	https://www.ncbi.nlm.nih.gov/gene/?term=80289
Atxn7	Atxn7	ataxin 7	246103	https://www.ncbi.nlm.nih.gov/gene/?term=246103
Astn1	Astn1	astrotactin 1	11899	https://www.ncbi.nlm.nih.gov/gene/?term=11899
Stx2	Stx2	syntaxin 2	13852	https://www.ncbi.nlm.nih.gov/gene/?term=13852

Biology Open • Supplementary information

Brd3	Brd3	bromodomain containing 3	67382	https://www.ncbi.nlm.nih.gov/gene/?term=67382
Map7d1	Map7d1	MAP7 domain containing 1	245877	https://www.ncbi.nlm.nih.gov/gene/?term=245877
Cdk8	Cdk8	cyclin-dependent kinase 8	264064	https://www.ncbi.nlm.nih.gov/gene/?term=264064
Npas3	Npas3	neuronal PAS domain protein 3	27386	https://www.ncbi.nlm.nih.gov/gene/?term=27386
Tada1	Tada1	transcriptional adaptor 1	27878	https://www.ncbi.nlm.nih.gov/gene/?term=27878
Nfasc	Nfasc	neurofascin	269116	https://www.ncbi.nlm.nih.gov/gene/?term=269116
Lbr	Lbr	lamin B receptor	98386	https://www.ncbi.nlm.nih.gov/gene/?term=98386
Rheb	Rheb	Ras homolog enriched in brain	19744	https://www.ncbi.nlm.nih.gov/gene/?term=19744
Lrrc8a	Lrrc8a	leucine rich repeat containing 8A	241296	https://www.ncbi.nlm.nih.gov/gene/?term=241296

Biology Open • Supplementary information

Zfp644	Zfp644	zinc finger protein 644	52397	https://www.ncbi.nlm.nih.gov/gene/?term=52397
Ccdc177	Ccdc177	coiled-coil domain containing 177	380768	https://www.ncbi.nlm.nih.gov/gene/?term=380768
Mbnl3	Mbnl3	muscleblind like splicing factor 3	171170	https://www.ncbi.nlm.nih.gov/gene/?term=171170
Cdc25a	Cdc25a	cell division cycle 25A	12530	https://www.ncbi.nlm.nih.gov/gene/?term=12530
C3ar1	C3ar1	complement component 3a receptor 1	12267	https://www.ncbi.nlm.nih.gov/gene/?term=12267
E2f3	E2f3	E2F transcription factor 3	13557	https://www.ncbi.nlm.nih.gov/gene/?term=13557
9330159F19Rik	9330159F19Rik	RIKEN cDNA 9330159F19 gene	212448	https://www.ncbi.nlm.nih.gov/gene/?term=212448
Tcerg1	Tcerg1	transcription elongation regulator 1 (CA150)	56070	https://www.ncbi.nlm.nih.gov/gene/?term=56070
Zfp597	Zfp597	zinc finger protein 597	71063	https://www.ncbi.nlm.nih.gov/gene/?term=71063

Biology Open • Supplementary information

Kcng3	Kcng3	potassium voltage-gated channel, subfamily G, member 3	225030	https://www.ncbi.nlm.nih.gov/gene/?term=225030
Tnrc6b	Tnrc6b	trinucleotide repeat containing 6b	213988	https://www.ncbi.nlm.nih.gov/gene/?term=213988
Stk17b	Stk17b	serine/threonine kinase 17b (apoptosis-inducing)	98267	https://www.ncbi.nlm.nih.gov/gene/?term=98267
Stxbp5	Stxbp5	syntaxin binding protein 5 (tomosyn)	78808	https://www.ncbi.nlm.nih.gov/gene/?term=78808
Cpeb3	Cpeb3	cytoplasmic polyadenylation element binding protein 3	208922	https://www.ncbi.nlm.nih.gov/gene/?term=208922
Fam227a	Fam227a	family with sequence similarity 227, member A	75729	https://www.ncbi.nlm.nih.gov/gene/?term=75729
Dcun1d3	Dcun1d3	DCN1, defective in cullin neddylation 1, domain containing 3 (S. cerevisiae)	233805	https://www.ncbi.nlm.nih.gov/gene/?term=233805
Tiam1	Tiam1	T cell lymphoma invasion and metastasis 1	21844	https://www.ncbi.nlm.nih.gov/gene/?term=21844
Tmem135	Tmem135	transmembrane protein 135	72759	https://www.ncbi.nlm.nih.gov/gene/?term=72759

Biology Open • Supplementary information

Taf12	Taf12	TATA-box binding protein associated factor 12	66464	https://www.ncbi.nlm.nih.gov/gene/?term=66464
Tmem221	Tmem221	transmembrane protein 221	434325	https://www.ncbi.nlm.nih.gov/gene/?term=434325
Fnip2	Fnip2	folliculin interacting protein 2	329679	https://www.ncbi.nlm.nih.gov/gene/?term=329679
Atrnl1	Atrnl1	attractin like 1	226255	https://www.ncbi.nlm.nih.gov/gene/?term=226255
Tmeff1	Tmeff1	transmembrane protein with EGF-like and two follistatin-like domains 1	230157	https://www.ncbi.nlm.nih.gov/gene/?term=230157
Arpc5	Arpc5	actin related protein 2/3 complex, subunit 5	67771	https://www.ncbi.nlm.nih.gov/gene/?term=67771
Fam91a1	Fam91a1	family with sequence similarity 91, member A1	210998	https://www.ncbi.nlm.nih.gov/gene/?term=210998
Ranbp6	Ranbp6	RAN binding protein 6	240614	https://www.ncbi.nlm.nih.gov/gene/?term=240614
Nrp1	Nrp1	neuropilin 1	18186	https://www.ncbi.nlm.nih.gov/gene/?term=18186

Kctd8	Kctd8	potassium channel tetramerisation domain containing 8	243043	https://www.ncbi.nlm.nih.gov/gene/?term=243043
Adck2	Adck2	aarF domain containing kinase 2	57869	https://www.ncbi.nlm.nih.gov/gene/?term=57869
Jag1	Jag1	jagged 1	16449	https://www.ncbi.nlm.nih.gov/gene/?term=16449
Tfap2c	Tfap2c	transcription factor AP-2, gamma	21420	https://www.ncbi.nlm.nih.gov/gene/?term=21420
Hcn1	Hcn1	hyperpolarization-activated, cyclic nucleotide-gated K+ 1	15165	https://www.ncbi.nlm.nih.gov/gene/?term=15165
Msantd4	Msantd4	Myb/SANT-like DNA-binding domain containing 4 with coiled-coils	78100	https://www.ncbi.nlm.nih.gov/gene/?term=78100
Tmem237	Tmem237	transmembrane protein 237	381259	https://www.ncbi.nlm.nih.gov/gene/?term=381259
Abhd18	Abhd18	abhydrolase domain containing 18	269423	https://www.ncbi.nlm.nih.gov/gene/?term=269423
Map2k4	Map2k4	mitogen-activated protein kinase kinase 4	26398	https://www.ncbi.nlm.nih.gov/gene/?term=26398

Biology Open • Supplementary information

Qser1	Qser1	glutamine and serine rich 1	99003	https://www.ncbi.nlm.nih.gov/gene/?term=99003
Hmg20a	Hmg20a	high mobility group 20A	66867	https://www.ncbi.nlm.nih.gov/gene/?term=66867
Adgrb3	Adgrb3	adhesion G protein-coupled receptor B3	210933	https://www.ncbi.nlm.nih.gov/gene/?term=210933
Stag1	Stag1	stromal antigen 1	20842	https://www.ncbi.nlm.nih.gov/gene/?term=20842
Slc6a9	Slc6a9	solute carrier family 6 (neurotransmitter transporter, glycine), member 9	14664	https://www.ncbi.nlm.nih.gov/gene/?term=14664
Hephl1	Hephl1	hephaestin-like 1	244698	https://www.ncbi.nlm.nih.gov/gene/?term=244698
Zyg11b	Zyg11b	zyg-ll family member B, cell cycle regulator	414872	https://www.ncbi.nlm.nih.gov/gene/?term=414872
Cfap97	Cfap97	cilia and flagella associated protein 97	66756	https://www.ncbi.nlm.nih.gov/gene/?term=66756
Ppm1e	Ppm1e	protein phosphatase 1E (PP2C domain containing)	320472	https://www.ncbi.nlm.nih.gov/gene/?term=320472

Biology Open • Supplementary information

Il13ra2	Il13ra2	interleukin 13 receptor, alpha 2	16165	https://www.ncbi.nlm.nih.gov/gene/?term=16165
Adpgk	Adpgk	ADP-dependent glucokinase	72141	https://www.ncbi.nlm.nih.gov/gene/?term=72141
Mtmr6	Mtmr6	myotubularin related protein 6	219135	https://www.ncbi.nlm.nih.gov/gene/?term=219135
Ttpal	Ttpal	tocopherol (alpha) transfer protein-like	76080	https://www.ncbi.nlm.nih.gov/gene/?term=76080
Ttbk2	Ttbk2	tau tubulin kinase 2	140810	https://www.ncbi.nlm.nih.gov/gene/?term=140810
Arhgef18	Arhgef18	rho/rac guanine nucleotide exchange factor (GEF) 18	102098	https://www.ncbi.nlm.nih.gov/gene/?term=102098
Klf6	Klf6	Kruppel-like factor 6	23849	https://www.ncbi.nlm.nih.gov/gene/?term=23849
Sipa1l2	Sipa1l2	signal-induced proliferation-associated 1 like 2	244668	https://www.ncbi.nlm.nih.gov/gene/?term=244668
Klf12	Klf12	Kruppel-like factor 12	16597	https://www.ncbi.nlm.nih.gov/gene/?term=16597

Biology Open • Supplementary information

Epha7	Epha7	Eph receptor A7	13841	https://www.ncbi.nlm.nih.gov/gene/?term=13841
Dnajc13	Dnajc13	DnaJ heat shock protein family (Hsp40) member C13	235567	https://www.ncbi.nlm.nih.gov/gene/?term=235567
Nufip2	Nufip2	nuclear fragile X mental retardation protein interacting protein 2	68564	https://www.ncbi.nlm.nih.gov/gene/?term=68564
Reck	Reck	reversion-inducing-cysteine-rich protein with kazal motifs	53614	https://www.ncbi.nlm.nih.gov/gene/?term=53614
Kif5b	Kif5b	kinesin family member 5B	16573	https://www.ncbi.nlm.nih.gov/gene/?term=16573
Ankrd44	Ankrd44	ankyrin repeat domain 44	329154	https://www.ncbi.nlm.nih.gov/gene/?term=329154
Ptprg	Ptprg	protein tyrosine phosphatase, receptor type, G	19270	https://www.ncbi.nlm.nih.gov/gene/?term=19270
Greb1l	Greb1l	growth regulation by estrogen in breast cancer-like	381157	https://www.ncbi.nlm.nih.gov/gene/?term=381157
Pdcd10	Pdcd10	programmed cell death 10	56426	https://www.ncbi.nlm.nih.gov/gene/?term=56426

Biology Open • Supplementary information

Mbnl2	Mbnl2	muscleblind like splicing factor 2	105559	https://www.ncbi.nlm.nih.gov/gene/?term=105559
Foxf2	Foxf2	forkhead box F2	14238	https://www.ncbi.nlm.nih.gov/gene/?term=14238
Rac1	Rac1	Rac family small GTPase 1	19353	https://www.ncbi.nlm.nih.gov/gene/?term=19353
Tmem170b	Tmem170b	transmembrane protein 170B	621976	https://www.ncbi.nlm.nih.gov/gene/?term=621976
Tectb	Tectb	tectorin beta	21684	https://www.ncbi.nlm.nih.gov/gene/?term=21684
Gpr146	Gpr146	G protein-coupled receptor 146	80290	https://www.ncbi.nlm.nih.gov/gene/?term=80290
Clock	Clock	circadian locomotor output cycles kaput	12753	https://www.ncbi.nlm.nih.gov/gene/?term=12753
Zbtb37	Zbtb37	zinc finger and BTB domain containing 37	240869	https://www.ncbi.nlm.nih.gov/gene/?term=240869
Hoxa9	Hoxa9	homeobox A9	15405	https://www.ncbi.nlm.nih.gov/gene/?term=15405

Biology Open • Supplementary information

Prelid3b	Prelid3b	PRELI domain containing 3B	66390	https://www.ncbi.nlm.nih.gov/gene/?term=66390
Slc44a2	Slc44a2	solute carrier family 44, member 2	68682	https://www.ncbi.nlm.nih.gov/gene/?term=68682
Msra	Msra	methionine sulfoxide reductase A	110265	https://www.ncbi.nlm.nih.gov/gene/?term=110265
Egr3	Egr3	early growth response 3	13655	https://www.ncbi.nlm.nih.gov/gene/?term=13655
Sinhcaf	Sinhcaf	SIN3-HDAC complex associated factor	56306	https://www.ncbi.nlm.nih.gov/gene/?term=56306
Phyhipl	Phyhipl	phytanoyl-CoA hydroxylase interacting protein-like	70911	https://www.ncbi.nlm.nih.gov/gene/?term=70911
Vamp3	Vamp3	vesicle-associated membrane protein 3	22319	https://www.ncbi.nlm.nih.gov/gene/?term=22319
Grb2	Grb2	growth factor receptor bound protein 2	14784	https://www.ncbi.nlm.nih.gov/gene/?term=14784
Ncald	Ncald	neurocalcin delta	52589	https://www.ncbi.nlm.nih.gov/gene/?term=52589

Biology Open • Supplementary information

Braf	Braf	Braf transforming gene	109880	https://www.ncbi.nlm.nih.gov/gene/?term=109880
Prrg3	Prrg3	proline rich Gla (G-carboxyglutamic acid) 3 (transmembrane)	208748	https://www.ncbi.nlm.nih.gov/gene/?term=208748
Palld	Palld	palladin, cytoskeletal associated protein	72333	https://www.ncbi.nlm.nih.gov/gene/?term=72333
Mtss1	Mtss1	metastasis suppressor 1	211401	https://www.ncbi.nlm.nih.gov/gene/?term=211401
Ankrd27	Ankrd27	ankyrin repeat domain 27 (VPS9 domain)	245886	https://www.ncbi.nlm.nih.gov/gene/?term=245886
Spin1	Spin1	spindlin 1	20729	https://www.ncbi.nlm.nih.gov/gene/?term=20729
Coro1c	Coro1c	coronin, actin binding protein 1C	23790	https://www.ncbi.nlm.nih.gov/gene/?term=23790
Tro	Tro	trophinin	56191	https://www.ncbi.nlm.nih.gov/gene/?term=56191
Fam126b	Fam126b	family with sequence similarity 126, member B	213056	https://www.ncbi.nlm.nih.gov/gene/?term=213056

Biology Open • Supplementary information

Ednrb	Ednrb	endothelin receptor type B	13618	https://www.ncbi.nlm.nih.gov/gene/?term=13618
Vezt	Vezt	vezatin, adherens junctions transmembrane protein	215008	https://www.ncbi.nlm.nih.gov/gene/?term=215008
Add3	Add3	adducin 3 (gamma)	27360	https://www.ncbi.nlm.nih.gov/gene/?term=27360
Taf15	Taf15	TATA-box binding protein associated factor 15	70439	https://www.ncbi.nlm.nih.gov/gene/?term=70439
Etl4	Etl4	enhancer trap locus 4	208618	https://www.ncbi.nlm.nih.gov/gene/?term=208618
Phf20l1	Phf20l1	PHD finger protein 20-like 1	239510	https://www.ncbi.nlm.nih.gov/gene/?term=239510
Prkce	Prkce	protein kinase C, epsilon	18754	https://www.ncbi.nlm.nih.gov/gene/?term=18754
Mitf	Mitf	melanogenesis associated transcription factor	17342	https://www.ncbi.nlm.nih.gov/gene/?term=17342
Nudt13	Nudt13	nudix (nucleoside diphosphate linked moiety X)-type motif 13	67725	https://www.ncbi.nlm.nih.gov/gene/?term=67725

Biology Open • Supplementary information

Gli2	Gli2	GLI-Kruppel family member GLI2	14633	https://www.ncbi.nlm.nih.gov/gene/?term=14633
Cd2ap	Cd2ap	CD2-associated protein	12488	https://www.ncbi.nlm.nih.gov/gene/?term=12488
Sp3	Sp3	trans-acting transcription factor 3	20687	https://www.ncbi.nlm.nih.gov/gene/?term=20687
Thbs2	Thbs2	thrombospondin 2	21826	https://www.ncbi.nlm.nih.gov/gene/?term=21826
Arhgef3	Arhgef3	Rho guanine nucleotide exchange factor (GEF) 3	71704	https://www.ncbi.nlm.nih.gov/gene/?term=71704
Inpp5a	Inpp5a	inositol polyphosphate-5-phosphatase A	212111	https://www.ncbi.nlm.nih.gov/gene/?term=212111
Shc4	Shc4	SHC (Src homology 2 domain containing) family, member 4	271849	https://www.ncbi.nlm.nih.gov/gene/?term=271849
Bnip3	Bnip3	NA	12176	https://www.ncbi.nlm.nih.gov/gene/?term=12176
Lmtk2	Lmtk2	lemur tyrosine kinase 2	231876	https://www.ncbi.nlm.nih.gov/gene/?term=231876

Magi1	Magi1	membrane associated guanylate kinase, WW and PDZ domain containing 1	14924	https://www.ncbi.nlm.nih.gov/gene/?term=14924
Stx5a	Stx5a	syntaxin 5A	56389	https://www.ncbi.nlm.nih.gov/gene/?term=56389
Nptx1	Nptx1	neuronal pentraxin 1	18164	https://www.ncbi.nlm.nih.gov/gene/?term=18164
Rasa1	Rasa1	RAS p21 protein activator 1	218397	https://www.ncbi.nlm.nih.gov/gene/?term=218397
Wasl	Wasl	Wiskott-Aldrich syndrome-like (human)	73178	https://www.ncbi.nlm.nih.gov/gene/?term=73178
Trabd2b	Trabd2b	TraB domain containing 2B	666048	https://www.ncbi.nlm.nih.gov/gene/?term=666048
Elavl4	Elavl4	ELAV like RNA binding protein 4	15572	https://www.ncbi.nlm.nih.gov/gene/?term=15572
Wdr76	Wdr76	WD repeat domain 76	241627	https://www.ncbi.nlm.nih.gov/gene/?term=241627
Fam43a	Fam43a	family with sequence similarity 43, member A	224093	https://www.ncbi.nlm.nih.gov/gene/?term=224093

Biology Open • Supplementary information

Topbp1	Topbp1	topoisomerase (DNA) II binding protein 1	235559	https://www.ncbi.nlm.nih.gov/gene/?term=235559
Slc39a9	Slc39a9	solute carrier family 39 (zinc transporter), member 9	328133	https://www.ncbi.nlm.nih.gov/gene/?term=328133
Rnf152	Rnf152	ring finger protein 152	320311	https://www.ncbi.nlm.nih.gov/gene/?term=320311
Rapgef5	Rapgef5	Rap guanine nucleotide exchange factor (GEF) 5	217944	https://www.ncbi.nlm.nih.gov/gene/?term=217944
Kdm2b	Kdm2b	lysine (K)-specific demethylase 2B	30841	https://www.ncbi.nlm.nih.gov/gene/?term=30841
Adcy6	Adcy6	adenylate cyclase 6	11512	https://www.ncbi.nlm.nih.gov/gene/?term=11512
Slc1a1	Slc1a1	solute carrier family 1 (neuronal/epithelial high affinity glutamate transporter, system Xag), member 1	20510	https://www.ncbi.nlm.nih.gov/gene/?term=20510
Pcnx	Pcnx	pecanex homolog	54604	https://www.ncbi.nlm.nih.gov/gene/?term=54604
Myrip	Myrip	myosin VIIA and Rab interacting protein	245049	https://www.ncbi.nlm.nih.gov/gene/?term=245049

Biology Open • Supplementary information

Phip	Phip	pleckstrin homology domain interacting protein	83946	https://www.ncbi.nlm.nih.gov/gene/?term=83946
Stox2	Stox2	storkhead box 2	71069	https://www.ncbi.nlm.nih.gov/gene/?term=71069
Wdr5b	Wdr5b	WD repeat domain 5B	69544	https://www.ncbi.nlm.nih.gov/gene/?term=69544
Ell	Ell	elongation factor RNA polymerase II	13716	https://www.ncbi.nlm.nih.gov/gene/?term=13716
Dcun1d1	Dcun1d1	DCN1, defective in cullin neddylation 1, domain containing 1 (S. cerevisiae)	114893	https://www.ncbi.nlm.nih.gov/gene/?term=114893
Tmem145	Tmem145	transmembrane protein 145	330485	https://www.ncbi.nlm.nih.gov/gene/?term=330485
Pnpla2	Pnpla2	patatin-like phospholipase domain containing 2	66853	https://www.ncbi.nlm.nih.gov/gene/?term=66853
Kif19a	Kif19a	kinesin family member 19A	286942	https://www.ncbi.nlm.nih.gov/gene/?term=286942
Aldh6a1	Aldh6a1	aldehyde dehydrogenase family 6, subfamily A1	104776	https://www.ncbi.nlm.nih.gov/gene/?term=104776

Ell2	Ell2	elongation factor RNA polymerase II 2	192657	https://www.ncbi.nlm.nih.gov/gene/?term=192657
Lims1	Lims1	LIM and senescent cell antigen-like domains 1	110829	https://www.ncbi.nlm.nih.gov/gene/?term=110829
Cacnb4	Cacnb4	calcium channel, voltage-dependent, beta 4 subunit	12298	https://www.ncbi.nlm.nih.gov/gene/?term=12298
Gfm2	Gfm2	G elongation factor, mitochondrial 2	320806	https://www.ncbi.nlm.nih.gov/gene/?term=320806
Xpr1	Xpr1	xenotropic and polytropic retrovirus receptor 1	19775	https://www.ncbi.nlm.nih.gov/gene/?term=19775
Cbarp	Cbarp	calcium channel, voltage-dependent, beta subunit associated regulatory protein	100503659	https://www.ncbi.nlm.nih.gov/gene/?term=100503
Pcx	Pcx	pyruvate carboxylase	18563	https://www.ncbi.nlm.nih.gov/gene/?term=18563
Zcchc14	Zcchc14	zinc finger, CCHC domain containing 14	142682	https://www.ncbi.nlm.nih.gov/gene/?term=142682
Yipf4	Yipf4	Yip1 domain family, member 4	67864	https://www.ncbi.nlm.nih.gov/gene/?term=67864

Biology Open • Supplementary information

Ints13	Ints13	integrator complex subunit 13	71177	https://www.ncbi.nlm.nih.gov/gene/?term=71177
Magel2	Magel2	melanoma antigen, family L, 2	27385	https://www.ncbi.nlm.nih.gov/gene/?term=27385
Dok4	Dok4	docking protein 4	114255	https://www.ncbi.nlm.nih.gov/gene/?term=114255
Chic1	Chic1	cysteine-rich hydrophobic domain 1	12212	https://www.ncbi.nlm.nih.gov/gene/?term=12212
Cdv3	Cdv3	carnitine deficiency-associated gene expressed in ventricle 3	321022	https://www.ncbi.nlm.nih.gov/gene/?term=321022
Gnaq	Gnaq	guanine nucleotide binding protein, alpha q polypeptide	14682	https://www.ncbi.nlm.nih.gov/gene/?term=14682
Bcl11a	Bcl11a	B cell CLL/lymphoma 11A (zinc finger protein)	14025	https://www.ncbi.nlm.nih.gov/gene/?term=14025
Slitrk4	Slitrk4	SLIT and NTRK-like family, member 4	245446	https://www.ncbi.nlm.nih.gov/gene/?term=245446
Dab1	Dab1	disabled 1	13131	https://www.ncbi.nlm.nih.gov/gene/?term=13131

Biology Open • Supplementary information

Ralgps2	Ralgps2	Ral GEF with PH domain and SH3 binding motif 2	78255	https://www.ncbi.nlm.nih.gov/gene/?term=78255
Prtg	Prtg	protogenin	235472	https://www.ncbi.nlm.nih.gov/gene/?term=235472
Rgs17	Rgs17	regulator of G-protein signaling 17	56533	https://www.ncbi.nlm.nih.gov/gene/?term=56533
Slc1a2	Slc1a2	solute carrier family 1 (glial high affinity glutamate transporter), member 2	20511	https://www.ncbi.nlm.nih.gov/gene/?term=20511
Retreg1	Retreg1	reticulophagy regulator 1	66270	https://www.ncbi.nlm.nih.gov/gene/?term=66270
Brpf3	Brpf3	bromodomain and PHD finger containing, 3	268936	https://www.ncbi.nlm.nih.gov/gene/?term=268936
Mrtfb	Mrtfb	myocardin related transcription factor B	239719	https://www.ncbi.nlm.nih.gov/gene/?term=239719
Arf4	Arf4	ADP-ribosylation factor 4	11843	https://www.ncbi.nlm.nih.gov/gene/?term=11843
Igsf3	Igsf3	immunoglobulin superfamily, member 3	78908	https://www.ncbi.nlm.nih.gov/gene/?term=78908

Biology Open • Supplementary information

Zc3h15	Zc3h15	zinc finger CCCH-type containing 15	69082	https://www.ncbi.nlm.nih.gov/gene/?term=69082
Dock1	Dock1	dedicator of cytokinesis 1	330662	https://www.ncbi.nlm.nih.gov/gene/?term=330662
Rarg	Rarg	retinoic acid receptor, gamma	19411	https://www.ncbi.nlm.nih.gov/gene/?term=19411
Dcaf4	Dcaf4	DDB1 and CUL4 associated factor 4	73828	https://www.ncbi.nlm.nih.gov/gene/?term=73828
Mak	Mak	male germ cell-associated kinase	17152	https://www.ncbi.nlm.nih.gov/gene/?term=17152
Nrn1	Nrn1	neuritin 1	68404	https://www.ncbi.nlm.nih.gov/gene/?term=68404
Anxa11	Anxa11	annexin A11	11744	https://www.ncbi.nlm.nih.gov/gene/?term=11744
Fut9	Fut9	fucosyltransferase 9	14348	https://www.ncbi.nlm.nih.gov/gene/?term=14348
Gys2	Gys2	glycogen synthase 2	232493	https://www.ncbi.nlm.nih.gov/gene/?term=232493

Cep250	Cep250	centrosomal protein 250	16328	https://www.ncbi.nlm.nih.gov/gene/?term=16328
Foxo3	Foxo3	forkhead box O3	56484	https://www.ncbi.nlm.nih.gov/gene/?term=56484
Brms1l	Brms1l	breast cancer metastasis-suppressor 1-like	52592	https://www.ncbi.nlm.nih.gov/gene/?term=52592
Plekha7	Plekha7	pleckstrin homology domain containing, family A member 7	233765	https://www.ncbi.nlm.nih.gov/gene/?term=233765
Baalc	Baalc	brain and acute leukemia, cytoplasmic	118452	https://www.ncbi.nlm.nih.gov/gene/?term=118452
Igf1r	Igf1r	insulin-like growth factor I receptor	16001	https://www.ncbi.nlm.nih.gov/gene/?term=16001
Snx30	Snx30	sorting nexin family member 30	209131	https://www.ncbi.nlm.nih.gov/gene/?term=209131
Fam171a1	Fam171a1	family with sequence similarity 171, member A1	269233	https://www.ncbi.nlm.nih.gov/gene/?term=269233
Frs2	Frs2	fibroblast growth factor receptor substrate 2	327826	https://www.ncbi.nlm.nih.gov/gene/?term=327826

Biology Open • Supplementary information

Pbx2	Pbx2	pre B cell leukemia homeobox 2	18515	https://www.ncbi.nlm.nih.gov/gene/?term=18515
Slc39a1	Slc39a1	solute carrier family 39 (zinc transporter), member 1	30791	https://www.ncbi.nlm.nih.gov/gene/?term=30791
Fam168a	Fam168a	family with sequence similarity 168, member A	319604	https://www.ncbi.nlm.nih.gov/gene/?term=319604
Cnnm3	Cnnm3	cyclin M3	94218	https://www.ncbi.nlm.nih.gov/gene/?term=94218
Ccny	Ccny	cyclin Y	67974	https://www.ncbi.nlm.nih.gov/gene/?term=67974
Usp13	Usp13	ubiquitin specific peptidase 13 (isopeptidase T-3)	72607	https://www.ncbi.nlm.nih.gov/gene/?term=72607
L1cam	L1cam	L1 cell adhesion molecule	16728	https://www.ncbi.nlm.nih.gov/gene/?term=16728
Wwc2	Wwc2	WW, C2 and coiled-coil domain containing 2	52357	https://www.ncbi.nlm.nih.gov/gene/?term=52357
Chst1	Chst1	carbohydrate sulfotransferase 1	76969	https://www.ncbi.nlm.nih.gov/gene/?term=76969

N4bp1	N4bp1	NEDD4 binding protein 1	80750	https://www.ncbi.nlm.nih.gov/gene/?term=80750
Has2	Has2	hyaluronan synthase 2	15117	https://www.ncbi.nlm.nih.gov/gene/?term=15117
Nid1	Nid1	nidogen 1	18073	https://www.ncbi.nlm.nih.gov/gene/?term=18073
Mast4	Mast4	microtubule associated serine/threonine kinase family member 4	328329	https://www.ncbi.nlm.nih.gov/gene/?term=328329
Dock4	Dock4	dedicator of cytokinesis 4	238130	https://www.ncbi.nlm.nih.gov/gene/?term=238130
Cbfa2t3	Cbfa2t3	CBFA2/RUNX1 translocation partner 3	12398	https://www.ncbi.nlm.nih.gov/gene/?term=12398
Pdzd8	Pdzd8	PDZ domain containing 8	107368	https://www.ncbi.nlm.nih.gov/gene/?term=107368
Rnf208	Rnf208	ring finger protein 208	68846	https://www.ncbi.nlm.nih.gov/gene/?term=68846
Smad1	Smad1	SMAD family member 1	17125	https://www.ncbi.nlm.nih.gov/gene/?term=17125

Rev1	Rev1	REV1, DNA directed polymerase	56210	https://www.ncbi.nlm.nih.gov/gene/?term=56210
Bcl2l12	Bcl2l12	BCL2-like 12 (proline rich)	75736	https://www.ncbi.nlm.nih.gov/gene/?term=75736
Kdelr2	Kdelr2	KDEL (Lys-Asp-Glu-Leu) endoplasmic reticulum protein retention receptor 2	66913	https://www.ncbi.nlm.nih.gov/gene/?term=66913
Ipcef1	Ipcef1	interaction protein for cytohesin exchange factors 1	320495	https://www.ncbi.nlm.nih.gov/gene/?term=320495
Adgrl2	Adgrl2	adhesion G protein-coupled receptor L2	99633	https://www.ncbi.nlm.nih.gov/gene/?term=99633
Cttn	Cttn	cortactin	13043	https://www.ncbi.nlm.nih.gov/gene/?term=13043
Nexmif	Nexmif	neurite extension and migration factor	245555	https://www.ncbi.nlm.nih.gov/gene/?term=245555
Zfc3h1	Zfc3h1	zinc finger, C3H1-type containing	216345	https://www.ncbi.nlm.nih.gov/gene/?term=216345
Cep83	Cep83	centrosomal protein 83	77048	https://www.ncbi.nlm.nih.gov/gene/?term=77048

Ube2q2	Ube2q2	ubiquitin-conjugating enzyme E2Q family member 2	109161	https://www.ncbi.nlm.nih.gov/gene/?term=109161
Rab2a	Rab2a	RAB2A, member RAS oncogene family	59021	https://www.ncbi.nlm.nih.gov/gene/?term=59021
Slco3a1	Slco3a1	solute carrier organic anion transporter family, member 3a1	108116	https://www.ncbi.nlm.nih.gov/gene/?term=108116
Zbtb41	Zbtb41	zinc finger and BTB domain containing 41	226470	https://www.ncbi.nlm.nih.gov/gene/?term=226470
Ahcyl1	Ahcyl1	S-adenosylhomocysteine hydrolase-like 1	229709	https://www.ncbi.nlm.nih.gov/gene/?term=229709
Satb2	Satb2	special AT-rich sequence binding protein 2	212712	https://www.ncbi.nlm.nih.gov/gene/?term=212712
Col25a1	Col25a1	collagen, type XXV, alpha 1	77018	https://www.ncbi.nlm.nih.gov/gene/?term=77018
Adra2c	Adra2c	adrenergic receptor, alpha 2c	11553	https://www.ncbi.nlm.nih.gov/gene/?term=11553
Bcl2l13	Bcl2l13	BCL2-like 13 (apoptosis facilitator)	94044	https://www.ncbi.nlm.nih.gov/gene/?term=94044

Bach2	Bach2	BTB and CNC homology, basic leucine zipper transcription factor 2	12014	https://www.ncbi.nlm.nih.gov/gene/?term=12014
Ppm1k	Ppm1k	protein phosphatase 1K (PP2C domain containing)	243382	https://www.ncbi.nlm.nih.gov/gene/?term=243382
Tapt1	Tapt1	transmembrane anterior posterior transformation 1	231225	https://www.ncbi.nlm.nih.gov/gene/?term=231225
Taf4b	Taf4b	TATA-box binding protein associated factor 4b	72504	https://www.ncbi.nlm.nih.gov/gene/?term=72504
Cadm2	Cadm2	cell adhesion molecule 2	239857	https://www.ncbi.nlm.nih.gov/gene/?term=239857
Fam118a	Fam118a	family with sequence similarity 118, member A	73225	https://www.ncbi.nlm.nih.gov/gene/?term=73225
Coa7	Coa7	cytochrome c oxidase assembly factor 7	69893	https://www.ncbi.nlm.nih.gov/gene/?term=69893
Htr1a	Htr1a	5-hydroxytryptamine (serotonin) receptor 1A	15550	https://www.ncbi.nlm.nih.gov/gene/?term=15550
Tns3	Tns3	tensin 3	319939	https://www.ncbi.nlm.nih.gov/gene/?term=319939

Atat1	Atat1	alpha tubulin acetyltransferase 1	73242	https://www.ncbi.nlm.nih.gov/gene/?term=73242
Zfp36l1	Zfp36l1	zinc finger protein 36, C3H type-like 1	12192	https://www.ncbi.nlm.nih.gov/gene/?term=12192
Tagln3	Tagln3	transgelin 3	56370	https://www.ncbi.nlm.nih.gov/gene/?term=56370
Ppil1	Ppil1	peptidylprolyl isomerase (cyclophilin)-like 1	68816	https://www.ncbi.nlm.nih.gov/gene/?term=68816
Mtm1	Mtm1	X-linked myotubular myopathy gene 1	17772	https://www.ncbi.nlm.nih.gov/gene/?term=17772
Foxn2	Foxn2	forkhead box N2	14236	https://www.ncbi.nlm.nih.gov/gene/?term=14236
0610030E20Rik	0610030E20Rik	RIKEN cDNA 0610030E20 gene	68364	https://www.ncbi.nlm.nih.gov/gene/?term=68364
Wipi2	Wipi2	WD repeat domain, phosphoinositide interacting 2	74781	https://www.ncbi.nlm.nih.gov/gene/?term=74781
Irak1	Irak1	interleukin-1 receptor-associated kinase 1	16179	https://www.ncbi.nlm.nih.gov/gene/?term=16179

Fbxw2	Fbxw2	F-box and WD-40 domain protein 2	30050	https://www.ncbi.nlm.nih.gov/gene/?term=30050
Slc10a3	Slc10a3	solute carrier family 10 (sodium/bile acid cotransporter family), member 3	214601	https://www.ncbi.nlm.nih.gov/gene/?term=214601
Eif4g2	Eif4g2	eukaryotic translation initiation factor 4, gamma 2	13690	https://www.ncbi.nlm.nih.gov/gene/?term=13690
Numb	Numb	NUMB endocytic adaptor protein	18222	https://www.ncbi.nlm.nih.gov/gene/?term=18222
Kras	Kras	Kirsten rat sarcoma viral oncogene homolog	16653	https://www.ncbi.nlm.nih.gov/gene/?term=16653
Rbm18	Rbm18	RNA binding motif protein 18	67889	https://www.ncbi.nlm.nih.gov/gene/?term=67889
Tdrkh	Tdrkh	tudor and KH domain containing protein	72634	https://www.ncbi.nlm.nih.gov/gene/?term=72634
Phc3	Phc3	polyhomeotic 3	241915	https://www.ncbi.nlm.nih.gov/gene/?term=241915
Sec23ip	Sec23ip	Sec23 interacting protein	207352	https://www.ncbi.nlm.nih.gov/gene/?term=207352

Biology Open • Supplementary information

Traf6	Traf6	TNF receptor-associated factor 6	22034	https://www.ncbi.nlm.nih.gov/gene/?term=22034
Med1	Med1	mediator complex subunit 1	19014	https://www.ncbi.nlm.nih.gov/gene/?term=19014
Paqr5	Paqr5	progestin and adipoQ receptor family member V	74090	https://www.ncbi.nlm.nih.gov/gene/?term=74090
Eif5a2	Eif5a2	eukaryotic translation initiation factor 5A2	208691	https://www.ncbi.nlm.nih.gov/gene/?term=208691
D030056L22Rik	D030056L22Rik	RIKEN cDNA D030056L22 gene	225995	https://www.ncbi.nlm.nih.gov/gene/?term=225995
Esyt2	Esyt2	extended synaptotagmin-like protein 2	52635	https://www.ncbi.nlm.nih.gov/gene/?term=52635
Sbspon	Sbspon	somatomedin B and thrombospondin, type 1 domain containing	226866	https://www.ncbi.nlm.nih.gov/gene/?term=226866
Appl1	Appl1	adaptor protein, phosphotyrosine interaction, PH domain and leucine zipper containing 1	72993	https://www.ncbi.nlm.nih.gov/gene/?term=72993
Mri1	Mri1	methylthioribose-1-phosphate isomerase 1	67873	https://www.ncbi.nlm.nih.gov/gene/?term=67873

Biology Open • Supplementary information

Nus1	Nus1	NUS1 dehydrodolichyl diphosphate synthase subunit	52014	https://www.ncbi.nlm.nih.gov/gene/?term=52014
Ncam1	Ncam1	neural cell adhesion molecule 1	17967	https://www.ncbi.nlm.nih.gov/gene/?term=17967
Acvr1b	Acvr1b	activin A receptor, type 1B	11479	https://www.ncbi.nlm.nih.gov/gene/?term=11479
Ranbp3	Ranbp3	RAN binding protein 3	71810	https://www.ncbi.nlm.nih.gov/gene/?term=71810
Ripply3	Ripply3	ripply transcriptional repressor 3	170765	https://www.ncbi.nlm.nih.gov/gene/?term=170765
Tfpi2	Tfpi2	tissue factor pathway inhibitor 2	21789	https://www.ncbi.nlm.nih.gov/gene/?term=21789
A1bg	A1bg	alpha-1-B glycoprotein	117586	https://www.ncbi.nlm.nih.gov/gene/?term=117586
Plpp3	Plpp3	phospholipid phosphatase 3	67916	https://www.ncbi.nlm.nih.gov/gene/?term=67916
Insig2	Insig2	insulin induced gene 2	72999	https://www.ncbi.nlm.nih.gov/gene/?term=72999

Armc1	Armc1	armadillo repeat containing 1	74252	https://www.ncbi.nlm.nih.gov/gene/?term=74252
Foxc1	Foxc1	forkhead box C1	17300	https://www.ncbi.nlm.nih.gov/gene/?term=17300
Atad2	Atad2	ATPase family, AAA domain containing 2	70472	https://www.ncbi.nlm.nih.gov/gene/?term=70472
Wnt5a	Wnt5a	wingless-type MMTV integration site family, member 5A	22418	https://www.ncbi.nlm.nih.gov/gene/?term=22418
Tmprss11e	Tmprss11e	transmembrane protease, serine 11e	243084	https://www.ncbi.nlm.nih.gov/gene/?term=243084
Ubr3	Ubr3	ubiquitin protein ligase E3 component n-recognin 3	68795	https://www.ncbi.nlm.nih.gov/gene/?term=68795
Usp53	Usp53	ubiquitin specific peptidase 53	99526	https://www.ncbi.nlm.nih.gov/gene/?term=99526
Cyp2c50	Cyp2c50	cytochrome P450, family 2, subfamily c, polypeptide 50	107141	https://www.ncbi.nlm.nih.gov/gene/?term=107141
Jchain	Jchain	immunoglobulin joining chain	16069	https://www.ncbi.nlm.nih.gov/gene/?term=16069

Biology Open • Supplementary information

Kif3c	Kif3c	kinesin family member 3C	16570	https://www.ncbi.nlm.nih.gov/gene/?term=16570
4930447C04Rik	4930447C04Rik	RIKEN cDNA 4930447C04 gene	75801	https://www.ncbi.nlm.nih.gov/gene/?term=75801
Usp22	Usp22	ubiquitin specific peptidase 22	216825	https://www.ncbi.nlm.nih.gov/gene/?term=216825
Syt6	Syt6	synaptotagmin VI	54524	https://www.ncbi.nlm.nih.gov/gene/?term=54524
Tgfbr3	Tgfbr3	transforming growth factor, beta receptor III	21814	https://www.ncbi.nlm.nih.gov/gene/?term=21814
Sun2	Sun2	Sad1 and UNC84 domain containing 2	223697	https://www.ncbi.nlm.nih.gov/gene/?term=223697
Plcxd2	Plcxd2	phosphatidylinositol-specific phospholipase C, X domain containing 2	433022	https://www.ncbi.nlm.nih.gov/gene/?term=433022
Rnf38	Rnf38	ring finger protein 38	73469	https://www.ncbi.nlm.nih.gov/gene/?term=73469
Nrp2	Nrp2	neuropilin 2	18187	https://www.ncbi.nlm.nih.gov/gene/?term=18187

Biology Open • Supplementary information

Rab1b	Rab1b	RAB1B, member RAS oncogene family	76308	https://www.ncbi.nlm.nih.gov/gene/?term=76308
Nf1	Nf1	neurofibromin 1	18015	https://www.ncbi.nlm.nih.gov/gene/?term=18015
Iars	Iars	isoleucine-tRNA synthetase	105148	https://www.ncbi.nlm.nih.gov/gene/?term=105148
Gpr63	Gpr63	G protein-coupled receptor 63	81006	https://www.ncbi.nlm.nih.gov/gene/?term=81006
Cyp3a41a	Cyp3a41a	cytochrome P450, family 3, subfamily a, polypeptide 41A	53973	https://www.ncbi.nlm.nih.gov/gene/?term=53973
Cant1	Cant1	calcium activated nucleotidase 1	76025	https://www.ncbi.nlm.nih.gov/gene/?term=76025
Ppp6r2	Ppp6r2	protein phosphatase 6, regulatory subunit 2	71474	https://www.ncbi.nlm.nih.gov/gene/?term=71474
Cacna2d1	Cacna2d1	calcium channel, voltage-dependent, alpha2/delta subunit 1	12293	https://www.ncbi.nlm.nih.gov/gene/?term=12293
Hdgfl3	Hdgfl3	HDGF like 3	29877	https://www.ncbi.nlm.nih.gov/gene/?term=29877

Biology Open • Supplementary information

Abl2	Abl2	v-abl Abelson murine leukemia viral oncogene 2 (arg, Abelson-related gene)	11352	https://www.ncbi.nlm.nih.gov/gene/?term=11352
Mrpl53	Mrpl53	mitochondrial ribosomal protein L53	68499	https://www.ncbi.nlm.nih.gov/gene/?term=68499
Pth	Pth	parathyroid hormone	19226	https://www.ncbi.nlm.nih.gov/gene/?term=19226
Mef2d	Mef2d	myocyte enhancer factor 2D	17261	https://www.ncbi.nlm.nih.gov/gene/?term=17261
Hacd2	Hacd2	3-hydroxyacyl-CoA dehydratase 2	70757	https://www.ncbi.nlm.nih.gov/gene/?term=70757
Ncor1	Ncor1	nuclear receptor co-repressor 1	20185	https://www.ncbi.nlm.nih.gov/gene/?term=20185
Mapk8	Mapk8	mitogen-activated protein kinase 8	26419	https://www.ncbi.nlm.nih.gov/gene/?term=26419
Snrk	Snrk	SNF related kinase	20623	https://www.ncbi.nlm.nih.gov/gene/?term=20623
Acvr2b	Acvr2b	activin receptor IIB	11481	https://www.ncbi.nlm.nih.gov/gene/?term=11481

Biology Open • Supplementary information

Usp42	Usp42	ubiquitin specific peptidase 42	76800	https://www.ncbi.nlm.nih.gov/gene/?term=76800
Gga3	Gga3	golgi associated, gamma adaptin ear containing, ARF binding protein 3	260302	https://www.ncbi.nlm.nih.gov/gene/?term=260302
Aste1	Aste1	asteroid homolog 1	66595	https://www.ncbi.nlm.nih.gov/gene/?term=66595
Clip1	Clip1	CAP-GLY domain containing linker protein 1	56430	https://www.ncbi.nlm.nih.gov/gene/?term=56430
Dync1li2	Dync1li2	dynein, cytoplasmic 1 light intermediate chain 2	234663	https://www.ncbi.nlm.nih.gov/gene/?term=234663
Six4	Six4	sine oculis-related homeobox 4	20474	https://www.ncbi.nlm.nih.gov/gene/?term=20474
Zhx1	Zhx1	zinc fingers and homeoboxes 1	22770	https://www.ncbi.nlm.nih.gov/gene/?term=22770
Sptan1	Sptan1	spectrin alpha, non-erythrocytic 1	20740	https://www.ncbi.nlm.nih.gov/gene/?term=20740
Jakmip2	Jakmip2	janus kinase and microtubule interacting protein 2	76217	https://www.ncbi.nlm.nih.gov/gene/?term=76217

Biology Open • Supplementary information

Zfp280d	Zfp280d	zinc finger protein 280D	235469	https://www.ncbi.nlm.nih.gov/gene/?term=235469
Ror1	Ror1	receptor tyrosine kinase-like orphan receptor 1	26563	https://www.ncbi.nlm.nih.gov/gene/?term=26563
Tbkbp1	Tbkbp1	TBK1 binding protein 1	73174	https://www.ncbi.nlm.nih.gov/gene/?term=73174
Chd1	Chd1	chromodomain helicase DNA binding protein 1	12648	https://www.ncbi.nlm.nih.gov/gene/?term=12648
Spats2l	Spats2l	spermatogenesis associated, serine-rich 2-like	67198	https://www.ncbi.nlm.nih.gov/gene/?term=67198
Znrf2	Znrf2	zinc and ring finger 2	387524	https://www.ncbi.nlm.nih.gov/gene/?term=387524
Gpc6	Gpc6	glypican 6	23888	https://www.ncbi.nlm.nih.gov/gene/?term=23888
Fermt2	Fermt2	fermitin family member 2	218952	https://www.ncbi.nlm.nih.gov/gene/?term=218952
Ahsa2	Ahsa2	AHA1, activator of heat shock protein ATPase 2	268390	https://www.ncbi.nlm.nih.gov/gene/?term=268390

Biology Open • Supplementary information

Kif21a	Kif21a	kinesin family member 21A	16564	https://www.ncbi.nlm.nih.gov/gene/?term=16564
Atf7	Atf7	activating transcription factor 7	223922	https://www.ncbi.nlm.nih.gov/gene/?term=223922
Eif4b	Eif4b	eukaryotic translation initiation factor 4B	75705	https://www.ncbi.nlm.nih.gov/gene/?term=75705
Kif23	Kif23	kinesin family member 23	71819	https://www.ncbi.nlm.nih.gov/gene/?term=71819
Med26	Med26	mediator complex subunit 26	70625	https://www.ncbi.nlm.nih.gov/gene/?term=70625
Zfpm2	Zfpm2	zinc finger protein, multitype 2	22762	https://www.ncbi.nlm.nih.gov/gene/?term=22762
Ano3	Ano3	anoctamin 3	228432	https://www.ncbi.nlm.nih.gov/gene/?term=228432
Axin2	Axin2	axin 2	12006	https://www.ncbi.nlm.nih.gov/gene/?term=12006
Fam117b	Fam117b	family with sequence similarity 117, member B	72750	https://www.ncbi.nlm.nih.gov/gene/?term=72750

Biology Open • Supplementary information

Pappa2	Pappa2	pappalysin 2	23850	https://www.ncbi.nlm.nih.gov/gene/?term=23850
Twf1	Twf1	twinfilin actin binding protein 1	19230	https://www.ncbi.nlm.nih.gov/gene/?term=19230
Nectin1	Nectin1	nectin cell adhesion molecule 1	58235	https://www.ncbi.nlm.nih.gov/gene/?term=58235
Dynlt3	Dynlt3	dynein light chain Tctex-type 3	67117	https://www.ncbi.nlm.nih.gov/gene/?term=67117
Cyb5r4	Cyb5r4	cytochrome b5 reductase 4	266690	https://www.ncbi.nlm.nih.gov/gene/?term=266690
Ago4	Ago4	argonaute RISC catalytic subunit 4	76850	https://www.ncbi.nlm.nih.gov/gene/?term=76850
Grpel1	Grpel1	GrpE-like 1, mitochondrial	17713	https://www.ncbi.nlm.nih.gov/gene/?term=17713
Tmem45b	Tmem45b	transmembrane protein 45b	235135	https://www.ncbi.nlm.nih.gov/gene/?term=235135
Cyp3a41b	Cyp3a41b	cytochrome P450, family 3, subfamily a, polypeptide 41B	100041375	https://www.ncbi.nlm.nih.gov/gene/?term=100041

Biology Open • Supplementary information

Hykk	Hykk	hydroxylysine kinase 1	235386	https://www.ncbi.nlm.nih.gov/gene/?term=235386
Ism1	Ism1	isthmin 1, angiogenesis inhibitor	319909	https://www.ncbi.nlm.nih.gov/gene/?term=319909
Sgms1	Sgms1	sphingomyelin synthase 1	208449	https://www.ncbi.nlm.nih.gov/gene/?term=208449
Nptn	Nptn	neuroplastin	20320	https://www.ncbi.nlm.nih.gov/gene/?term=20320
Arpp19	Arpp19	cAMP-regulated phosphoprotein 19	59046	https://www.ncbi.nlm.nih.gov/gene/?term=59046
Sik1	Sik1	salt inducible kinase 1	17691	https://www.ncbi.nlm.nih.gov/gene/?term=17691
Smad2	Smad2	SMAD family member 2	17126	https://www.ncbi.nlm.nih.gov/gene/?term=17126
Rab34	Rab34	RAB34, member RAS oncogene family	19376	https://www.ncbi.nlm.nih.gov/gene/?term=19376
Barhl2	Barhl2	BarH like homeobox 2	104382	https://www.ncbi.nlm.nih.gov/gene/?term=104382

Biology Open • Supplementary information

Arhgap21	Arhgap21	Rho GTPase activating protein 21	71435	https://www.ncbi.nlm.nih.gov/gene/?term=71435
Plaa	Plaa	phospholipase A2, activating protein	18786	https://www.ncbi.nlm.nih.gov/gene/?term=18786
Ucp3	Ucp3	uncoupling protein 3 (mitochondrial, proton carrier)	22229	https://www.ncbi.nlm.nih.gov/gene/?term=22229
Cand1	Cand1	cullin associated and neddylation disassociated 1	71902	https://www.ncbi.nlm.nih.gov/gene/?term=71902
Bcl2l11	Bcl2l11	BCL2-like 11 (apoptosis facilitator)	12125	https://www.ncbi.nlm.nih.gov/gene/?term=12125
Otud4	Otud4	OTU domain containing 4	73945	https://www.ncbi.nlm.nih.gov/gene/?term=73945
Oas1c	Oas1c	2'-5' oligoadenylate synthetase 1C	114643	https://www.ncbi.nlm.nih.gov/gene/?term=114643
Ptpn14	Ptpn14	protein tyrosine phosphatase, non-receptor type 14	19250	https://www.ncbi.nlm.nih.gov/gene/?term=19250
S1pr1	S1pr1	sphingosine-1-phosphate receptor 1	13609	https://www.ncbi.nlm.nih.gov/gene/?term=13609

Biology Open • Supplementary information

Sos2	Sos2	SOS Ras/Rho guanine nucleotide exchange factor 2	20663	https://www.ncbi.nlm.nih.gov/gene/?term=20663
Itsn2	Itsn2	intersectin 2	20403	https://www.ncbi.nlm.nih.gov/gene/?term=20403
Npepl1	Npepl1	aminopeptidase-like 1	228961	https://www.ncbi.nlm.nih.gov/gene/?term=228961
Rtca	Rtca	RNA 3'-terminal phosphate cyclase	66368	https://www.ncbi.nlm.nih.gov/gene/?term=66368
Naaladl2	Naaladl2	N-acetylated alpha-linked acidic dipeptidase-like 2	635702	https://www.ncbi.nlm.nih.gov/gene/?term=635702
Maf1	Maf1	MAF1 homolog, negative regulator of RNA polymerase III	68877	https://www.ncbi.nlm.nih.gov/gene/?term=68877
Esr1	Esr1	estrogen receptor 1 (alpha)	13982	https://www.ncbi.nlm.nih.gov/gene/?term=13982
Atp8a1	Atp8a1	ATPase, aminophospholipid transporter (APLT), class I, type 8A, member 1	11980	https://www.ncbi.nlm.nih.gov/gene/?term=11980
Tmem9b	Tmem9b	TMEM9 domain family, member B	56786	https://www.ncbi.nlm.nih.gov/gene/?term=56786

Biology Open • Supplementary information

Epn2	Epn2	epsin 2	13855	https://www.ncbi.nlm.nih.gov/gene/?term=13855
Szrd1	Szrd1	SUZ RNA binding domain containing 1	213491	https://www.ncbi.nlm.nih.gov/gene/?term=213491
Btbd3	Btbd3	BTB (POZ) domain containing 3	228662	https://www.ncbi.nlm.nih.gov/gene/?term=228662
Dicer1	Dicer1	dicer 1, ribonuclease type III	192119	https://www.ncbi.nlm.nih.gov/gene/?term=192119
Stam	Stam	signal transducing adaptor molecule (SH3 domain and ITAM motif) 1	20844	https://www.ncbi.nlm.nih.gov/gene/?term=20844
Akap1	Akap1	A kinase (PRKA) anchor protein 1	11640	https://www.ncbi.nlm.nih.gov/gene/?term=11640
Fbn1	Fbn1	fibrillin 1	14118	https://www.ncbi.nlm.nih.gov/gene/?term=14118
Ube3b	Ube3b	ubiquitin protein ligase E3B	117146	https://www.ncbi.nlm.nih.gov/gene/?term=117146
Ctxn3	Ctxn3	cortexin 3	629147	https://www.ncbi.nlm.nih.gov/gene/?term=629147

Biology Open • Supplementary information

Arl6ip1	Arl6ip1	ADP-ribosylation factor-like 6 interacting protein 1	54208	https://www.ncbi.nlm.nih.gov/gene/?term=54208
Lrp2	Lrp2	low density lipoprotein receptor-related protein 2	14725	https://www.ncbi.nlm.nih.gov/gene/?term=14725
Cdk19	Cdk19	cyclin-dependent kinase 19	78334	https://www.ncbi.nlm.nih.gov/gene/?term=78334
Mospd1	Mospd1	motile sperm domain containing 1	70380	https://www.ncbi.nlm.nih.gov/gene/?term=70380
Tgfa	Tgfa	transforming growth factor alpha	21802	https://www.ncbi.nlm.nih.gov/gene/?term=21802
Spty2d1	Spty2d1	SPT2, Suppressor of Ty, domain containing 1 (S. cerevisiae)	101685	https://www.ncbi.nlm.nih.gov/gene/?term=101685
Ywhab	Ywhab	tyrosine 3-monooxygenase/tryptophan 5-monooxygenase activation protein, beta polypeptide	54401	https://www.ncbi.nlm.nih.gov/gene/?term=54401
Usp32	Usp32	ubiquitin specific peptidase 32	237898	https://www.ncbi.nlm.nih.gov/gene/?term=237898
Med12l	Med12l	mediator complex subunit 12-like	329650	https://www.ncbi.nlm.nih.gov/gene/?term=329650

Biology Open • Supplementary information

Nrarp	Nrarp	Notch-regulated ankyrin repeat protein	67122	https://www.ncbi.nlm.nih.gov/gene/?term=67122
Tmem54	Tmem54	transmembrane protein 54	66260	https://www.ncbi.nlm.nih.gov/gene/?term=66260
Fam104a	Fam104a	family with sequence similarity 104, member A	28081	https://www.ncbi.nlm.nih.gov/gene/?term=28081
Col4a1	Col4a1	collagen, type IV, alpha 1	12826	https://www.ncbi.nlm.nih.gov/gene/?term=12826
Hepacam2	Hepacam2	HEPACAM family member 2	101202	https://www.ncbi.nlm.nih.gov/gene/?term=101202
B4galt5	B4galt5	UDP-Gal:betaGlcNAc beta 1,4-galactosyltransferase, polypeptide 5	56336	https://www.ncbi.nlm.nih.gov/gene/?term=56336
Jmy	Jmy	junction-mediating and regulatory protein	57748	https://www.ncbi.nlm.nih.gov/gene/?term=57748
Tnrc6c	Tnrc6c	trinucleotide repeat containing 6C	217351	https://www.ncbi.nlm.nih.gov/gene/?term=217351
Abcb7	Abcb7	ATP-binding cassette, sub-family B (MDR/TAP), member 7	11306	https://www.ncbi.nlm.nih.gov/gene/?term=11306

Arrdc3	Arrdc3	arrestin domain containing 3	105171	https://www.ncbi.nlm.nih.gov/gene/?term=105171
Btaf1	Btaf1	B-TFIID TATA-box binding protein associated factor 1	107182	https://www.ncbi.nlm.nih.gov/gene/?term=107182
Rictor	Rictor	RPTOR independent companion of MTOR, complex 2	78757	https://www.ncbi.nlm.nih.gov/gene/?term=78757
Canx	Canx	calnexin	12330	https://www.ncbi.nlm.nih.gov/gene/?term=12330
Manba	Manba	mannosidase, beta A, lysosomal	110173	https://www.ncbi.nlm.nih.gov/gene/?term=110173
Robo1	Robo1	roundabout guidance receptor 1	19876	https://www.ncbi.nlm.nih.gov/gene/?term=19876
Phactr2	Phactr2	phosphatase and actin regulator 2	215789	https://www.ncbi.nlm.nih.gov/gene/?term=215789
Amacr	Amacr	alpha-methylacyl-CoA racemase	17117	https://www.ncbi.nlm.nih.gov/gene/?term=17117
Ncoa1	Ncoa1	nuclear receptor coactivator 1	17977	https://www.ncbi.nlm.nih.gov/gene/?term=17977

Biology Open • Supplementary information

Rph3al	Rph3al	rabphilin 3A-like (without C2 domains)	380714	https://www.ncbi.nlm.nih.gov/gene/?term=380714
Sgcb	Sgcb	sarcoglycan, beta (dystrophin-associated glycoprotein)	24051	https://www.ncbi.nlm.nih.gov/gene/?term=24051
Lgalsl	Lgalsl	lectin, galactoside binding-like	216551	https://www.ncbi.nlm.nih.gov/gene/?term=216551
Ppp6r1	Ppp6r1	protein phosphatase 6, regulatory subunit 1	243819	https://www.ncbi.nlm.nih.gov/gene/?term=243819
Slco4a1	Slco4a1	solute carrier organic anion transporter family, member 4a1	108115	https://www.ncbi.nlm.nih.gov/gene/?term=108115
Robo2	Robo2	roundabout guidance receptor 2	268902	https://www.ncbi.nlm.nih.gov/gene/?term=268902
Adamts15	Adamts15	a disintegrin-like and metallopeptidase (reprolysin type) with thrombospondin type 1 motif, 15	235130	https://www.ncbi.nlm.nih.gov/gene/?term=235130
Jph3	Jph3	junctophilin 3	57340	https://www.ncbi.nlm.nih.gov/gene/?term=57340
Fam234a	Fam234a	family with sequence similarity 234, member A	106581	https://www.ncbi.nlm.nih.gov/gene/?term=106581

Biology Open • Supplementary information

Hbs1l	Hbs1l	Hbs1-like (S. cerevisiae)	56422	https://www.ncbi.nlm.nih.gov/gene/?term=56422
Hs3st1	Hs3st1	heparan sulfate (glucosamine) 3-O-sulfotransferase 1	15476	https://www.ncbi.nlm.nih.gov/gene/?term=15476
Inhbb	Inhbb	inhibin beta-B	16324	https://www.ncbi.nlm.nih.gov/gene/?term=16324
Ssr1	Ssr1	signal sequence receptor, alpha	107513	https://www.ncbi.nlm.nih.gov/gene/?term=107513
Dgcr8	Dgcr8	DGCR8, microprocessor complex subunit	94223	https://www.ncbi.nlm.nih.gov/gene/?term=94223
Ube2d3	Ube2d3	ubiquitin-conjugating enzyme E2D 3	66105	https://www.ncbi.nlm.nih.gov/gene/?term=66105
Ino80	Ino80	INO80 complex subunit	68142	https://www.ncbi.nlm.nih.gov/gene/?term=68142
C1galt1	C1galt1	core 1 synthase, glycoprotein-N-acetylgalactosamine 3-beta-galactosyltransferase, 1	94192	https://www.ncbi.nlm.nih.gov/gene/?term=94192
Ajuba	Ajuba	ajuba LIM protein	16475	https://www.ncbi.nlm.nih.gov/gene/?term=16475

Biology Open • Supplementary information

D5Ertd579e	D5Ertd579e	DNA segment, Chr 5, ERATO Doi 579, expressed	320661	https://www.ncbi.nlm.nih.gov/gene/?term=320661
Tnrc6a	Tnrc6a	trinucleotide repeat containing 6a	233833	https://www.ncbi.nlm.nih.gov/gene/?term=233833
Meox2	Meox2	mesenchyme homeobox 2	17286	https://www.ncbi.nlm.nih.gov/gene/?term=17286
Mdfic	Mdfic	MyoD family inhibitor domain containing	16543	https://www.ncbi.nlm.nih.gov/gene/?term=16543
Itga9	Itga9	integrin alpha 9	104099	https://www.ncbi.nlm.nih.gov/gene/?term=104099
Rmnd5a	Rmnd5a	required for meiotic nuclear division 5 homolog A	68477	https://www.ncbi.nlm.nih.gov/gene/?term=68477
Eogt	Eogt	EGF domain-specific O-linked N-acetylglucosamine (GlcNAc) transferase	101351	https://www.ncbi.nlm.nih.gov/gene/?term=101351
Relch	Relch	RAB11 binding and LisH domain, coiled-coil and HEAT repeat containing	227446	https://www.ncbi.nlm.nih.gov/gene/?term=227446
Itga5	Itga5	integrin alpha 5 (fibronectin receptor alpha)	16402	https://www.ncbi.nlm.nih.gov/gene/?term=16402

Biology Open • Supplementary information

Tnpo1	Tnpo1	transportin 1	238799	https://www.ncbi.nlm.nih.gov/gene/?term=238799
Ppp1r10	Ppp1r10	protein phosphatase 1, regulatory subunit 10	52040	https://www.ncbi.nlm.nih.gov/gene/?term=52040
Phf3	Phf3	PHD finger protein 3	213109	https://www.ncbi.nlm.nih.gov/gene/?term=213109
Mpped2	Mpped2	metallophosphoesterase domain containing 2	77015	https://www.ncbi.nlm.nih.gov/gene/?term=77015
Psmb6	Psmb6	proteasome (prosome, macropain) subunit, beta type 6	19175	https://www.ncbi.nlm.nih.gov/gene/?term=19175
Cblb	Cblb	Casitas B-lineage lymphoma b	208650	https://www.ncbi.nlm.nih.gov/gene/?term=208650
Gadd45a	Gadd45a	growth arrest and DNA-damage-inducible 45 alpha	13197	https://www.ncbi.nlm.nih.gov/gene/?term=13197
Pde1a	Pde1a	phosphodiesterase 1A, calmodulin-dependent	18573	https://www.ncbi.nlm.nih.gov/gene/?term=18573
Cmip	Cmip	c-Maf inducing protein	74440	https://www.ncbi.nlm.nih.gov/gene/?term=74440

Biology Open • Supplementary information

Serpinb2	Serpinb2	serine (or cysteine) peptidase inhibitor, clade B, member 2	18788	https://www.ncbi.nlm.nih.gov/gene/?term=18788
Sh3pxd2b	Sh3pxd2b	SH3 and PX domains 2B	268396	https://www.ncbi.nlm.nih.gov/gene/?term=268396
Bcr	Bcr	breakpoint cluster region	110279	https://www.ncbi.nlm.nih.gov/gene/?term=110279
Ttyh3	Ttyh3	tweety family member 3	78339	https://www.ncbi.nlm.nih.gov/gene/?term=78339
Plod2	Plod2	procollagen lysine, 2-oxoglutarate 5-dioxygenase 2	26432	https://www.ncbi.nlm.nih.gov/gene/?term=26432
Tsku	Tsku	tsukushi, small leucine rich proteoglycan	244152	https://www.ncbi.nlm.nih.gov/gene/?term=244152
Cep170b	Cep170b	centrosomal protein 170B	217882	https://www.ncbi.nlm.nih.gov/gene/?term=217882
Sh3kbp1	Sh3kbp1	SH3-domain kinase binding protein 1	58194	https://www.ncbi.nlm.nih.gov/gene/?term=58194
Tgfbr1	Tgfbr1	transforming growth factor, beta receptor I	21812	https://www.ncbi.nlm.nih.gov/gene/?term=21812

Biology Open • Supplementary information

Slc16a9	Slc16a9	solute carrier family 16 (monocarboxylic acid transporters), member 9	66859	https://www.ncbi.nlm.nih.gov/gene/?term=66859
Gulp1	Gulp1	GULP, engulfment adaptor PTB domain containing 1	70676	https://www.ncbi.nlm.nih.gov/gene/?term=70676
Rab23	Rab23	RAB23, member RAS oncogene family	19335	https://www.ncbi.nlm.nih.gov/gene/?term=19335
Eif4ebp2	Eif4ebp2	eukaryotic translation initiation factor 4E binding protein 2	13688	https://www.ncbi.nlm.nih.gov/gene/?term=13688
Gpc3	Gpc3	glypican 3	14734	https://www.ncbi.nlm.nih.gov/gene/?term=14734
Cnn3	Cnn3	calponin 3, acidic	71994	https://www.ncbi.nlm.nih.gov/gene/?term=71994
Nsg1	Nsg1	neuron specific gene family member 1	18196	https://www.ncbi.nlm.nih.gov/gene/?term=18196
Celsr1	Celsr1	cadherin, EGF LAG seven-pass G-type receptor 1	12614	https://www.ncbi.nlm.nih.gov/gene/?term=12614
Hsd3b5	Hsd3b5	hydroxy-delta-5-steroid dehydrogenase, 3 beta- and steroid delta-isomerase 5	15496	https://www.ncbi.nlm.nih.gov/gene/?term=15496

Biology Open • Supplementary information

Zkscan17	Zkscan17	zinc finger with KRAB and SCAN domains 17	268417	https://www.ncbi.nlm.nih.gov/gene/?term=268417
Morf4l1	Morf4l1	mortality factor 4 like 1	21761	https://www.ncbi.nlm.nih.gov/gene/?term=21761
Ogt	Ogt	O-linked N-acetylglucosamine (GlcNAc) transferase (UDP-N-acetylglucosamine:polypeptide-N-acetylglucosaminyl transferase)	108155	https://www.ncbi.nlm.nih.gov/gene/?term=108155
Klhl7	Klhl7	kelch-like 7	52323	https://www.ncbi.nlm.nih.gov/gene/?term=52323
Ralgps1	Ralgps1	Ral GEF with PH domain and SH3 binding motif 1	241308	https://www.ncbi.nlm.nih.gov/gene/?term=241308
Foxq1	Foxq1	forkhead box Q1	15220	https://www.ncbi.nlm.nih.gov/gene/?term=15220
Aqp5	Aqp5	aquaporin 5	11830	https://www.ncbi.nlm.nih.gov/gene/?term=11830
Mtor	Mtor	mechanistic target of rapamycin kinase	56717	https://www.ncbi.nlm.nih.gov/gene/?term=56717
Foxk2	Foxk2	forkhead box K2	68837	https://www.ncbi.nlm.nih.gov/gene/?term=68837

Biology Open • Supplementary information

Sox6	Sox6	SRY (sex determining region Y)-box 6	20679	https://www.ncbi.nlm.nih.gov/gene/?term=20679
Pmepa1	Pmepa1	prostate transmembrane protein, androgen induced 1	65112	https://www.ncbi.nlm.nih.gov/gene/?term=65112
Cd164	Cd164	CD164 antigen	53599	https://www.ncbi.nlm.nih.gov/gene/?term=53599
Tcf7l2	Tcf7l2	transcription factor 7 like 2, T cell specific, HMG box	21416	https://www.ncbi.nlm.nih.gov/gene/?term=21416
Olfr613	Olfr613	olfactory receptor 613	259104	https://www.ncbi.nlm.nih.gov/gene/?term=259104
Fundc2	Fundc2	FUN14 domain containing 2	67391	https://www.ncbi.nlm.nih.gov/gene/?term=67391
Pde8b	Pde8b	phosphodiesterase 8B	218461	https://www.ncbi.nlm.nih.gov/gene/?term=218461
St6galnac3	St6galnac3	ST6 (alpha-N-acetyl-neuraminyl-2,3-beta-galactosyl-1,3)-N-acetylgalactosaminide alpha-2,6-sialyltransferase 3	20447	https://www.ncbi.nlm.nih.gov/gene/?term=20447
E2f5	E2f5	E2F transcription factor 5	13559	https://www.ncbi.nlm.nih.gov/gene/?term=13559

Biology Open • Supplementary information

Ahr	Ahr	aryl-hydrocarbon receptor	11622	https://www.ncbi.nlm.nih.gov/gene/?term=11622
Trib3	Trib3	tribbles pseudokinase 3	228775	https://www.ncbi.nlm.nih.gov/gene/?term=228775
Sdc2	Sdc2	syndecan 2	15529	https://www.ncbi.nlm.nih.gov/gene/?term=15529
Slain2	Slain2	SLAIN motif family, member 2	75991	https://www.ncbi.nlm.nih.gov/gene/?term=75991
Casp2	Casp2	caspase 2	12366	https://www.ncbi.nlm.nih.gov/gene/?term=12366
Arhgap24	Arhgap24	Rho GTPase activating protein 24	231532	https://www.ncbi.nlm.nih.gov/gene/?term=231532
Ebf3	Ebf3	early B cell factor 3	13593	https://www.ncbi.nlm.nih.gov/gene/?term=13593
Dgkh	Dgkh	diacylglycerol kinase, eta	380921	https://www.ncbi.nlm.nih.gov/gene/?term=380921
Tes	Tes	testis derived transcript	21753	https://www.ncbi.nlm.nih.gov/gene/?term=21753

Ntn4	Ntn4	netrin 4	57764	https://www.ncbi.nlm.nih.gov/gene/?term=57764
Map2k1	Map2k1	mitogen-activated protein kinase kinase 1	26395	https://www.ncbi.nlm.nih.gov/gene/?term=26395
Hsf5	Hsf5	heat shock transcription factor family member 5	327992	https://www.ncbi.nlm.nih.gov/gene/?term=327992
Rassf8	Rassf8	Ras association (RalGDS/AF-6) domain family (N-terminal) member 8	71323	https://www.ncbi.nlm.nih.gov/gene/?term=71323
Plppr4	Plppr4	phospholipid phosphatase related 4	229791	https://www.ncbi.nlm.nih.gov/gene/?term=229791
Abcd1	Abcd1	ATP-binding cassette, sub-family D (ALD), member 1	11666	https://www.ncbi.nlm.nih.gov/gene/?term=11666
Tut4	Tut4	terminal uridylyl transferase 4	230594	https://www.ncbi.nlm.nih.gov/gene/?term=230594
Rnf139	Rnf139	ring finger protein 139	75841	https://www.ncbi.nlm.nih.gov/gene/?term=75841
Zcchc3	Zcchc3	zinc finger, CCHC domain containing 3	67917	https://www.ncbi.nlm.nih.gov/gene/?term=67917

Biology Open • Supplementary information

Hbegf	Hbegf	heparin-binding EGF-like growth factor	15200	https://www.ncbi.nlm.nih.gov/gene/?term=15200
B4galnt1	B4galnt1	beta-1,4-N-acetyl-galactosaminyl transferase 1	14421	https://www.ncbi.nlm.nih.gov/gene/?term=14421
Cpeb1	Cpeb1	cytoplasmic polyadenylation element binding protein 1	12877	https://www.ncbi.nlm.nih.gov/gene/?term=12877
Vat1l	Vat1l	vesicle amine transport protein 1 like	270097	https://www.ncbi.nlm.nih.gov/gene/?term=270097
Gad2	Gad2	glutamic acid decarboxylase 2	14417	https://www.ncbi.nlm.nih.gov/gene/?term=14417
Tac1	Tac1	tachykinin 1	21333	https://www.ncbi.nlm.nih.gov/gene/?term=21333
Ube2g1	Ube2g1	ubiquitin-conjugating enzyme E2G 1	67128	https://www.ncbi.nlm.nih.gov/gene/?term=67128
Galnt2	Galnt2	polypeptide N-acetylgalactosaminyltransferase 2	108148	https://www.ncbi.nlm.nih.gov/gene/?term=108148
Slc12a6	Slc12a6	solute carrier family 12, member 6	107723	https://www.ncbi.nlm.nih.gov/gene/?term=107723

Biology Open • Supplementary information

Tmem198	Tmem198	transmembrane protein 198	319998	https://www.ncbi.nlm.nih.gov/gene/?term=319998
Farp1	Farp1	FERM, RhoGEF (Arhgef) and pleckstrin domain protein 1 (chondrocyte-derived)	223254	https://www.ncbi.nlm.nih.gov/gene/?term=223254
Arf2	Arf2	ADP-ribosylation factor 2	11841	https://www.ncbi.nlm.nih.gov/gene/?term=11841
Ankib1	Ankib1	ankyrin repeat and IBR domain containing 1	70797	https://www.ncbi.nlm.nih.gov/gene/?term=70797
Ezr	Ezr	ezrin	22350	https://www.ncbi.nlm.nih.gov/gene/?term=22350
Arpc5l	Arpc5l	actin related protein 2/3 complex, subunit 5-like	74192	https://www.ncbi.nlm.nih.gov/gene/?term=74192
Dlat	Dlat	dihydrolipoamide S-acetyltransferase (E2 component of pyruvate dehydrogenase complex)	235339	https://www.ncbi.nlm.nih.gov/gene/?term=235339
Tbr1	Tbr1	T-box brain gene 1	21375	https://www.ncbi.nlm.nih.gov/gene/?term=21375
Prrt3	Prrt3	proline-rich transmembrane protein 3	210673	https://www.ncbi.nlm.nih.gov/gene/?term=210673

Biology Open • Supplementary information

Zfp449	Zfp449	zinc finger protein 449	78619	https://www.ncbi.nlm.nih.gov/gene/?term=78619
Nova2	Nova2	neuro-oncological ventral antigen 2	384569	https://www.ncbi.nlm.nih.gov/gene/?term=384569
Pnpla8	Pnpla8	patatin-like phospholipase domain containing 8	67452	https://www.ncbi.nlm.nih.gov/gene/?term=67452
Gpr21	Gpr21	G protein-coupled receptor 21	338346	https://www.ncbi.nlm.nih.gov/gene/?term=338346
Eif5	Eif5	NA	217869	https://www.ncbi.nlm.nih.gov/gene/?term=217869
Rapgef4	Rapgef4	Rap guanine nucleotide exchange factor (GEF) 4	56508	https://www.ncbi.nlm.nih.gov/gene/?term=56508
Spast	Spast	spastin	50850	https://www.ncbi.nlm.nih.gov/gene/?term=50850
Grhl2	Grhl2	grainyhead like transcription factor 2	252973	https://www.ncbi.nlm.nih.gov/gene/?term=252973
Stmnd1	Stmnd1	stathmin domain containing 1	380842	https://www.ncbi.nlm.nih.gov/gene/?term=380842

Biology Open • Supplementary information

Uck2	Uck2	uridine-cytidine kinase 2	80914	https://www.ncbi.nlm.nih.gov/gene/?term=80914
Arsj	Arsj	arylsulfatase J	271970	https://www.ncbi.nlm.nih.gov/gene/?term=271970
Irs1	Irs1	insulin receptor substrate 1	16367	https://www.ncbi.nlm.nih.gov/gene/?term=16367
Jmjd1c	Jmjd1c	jumonji domain containing 1C	108829	https://www.ncbi.nlm.nih.gov/gene/?term=108829
Unc13c	Unc13c	unc-13 homolog C	208898	https://www.ncbi.nlm.nih.gov/gene/?term=208898
2610507B11Rik	2610507B11Rik	RIKEN cDNA 2610507B11 gene	72503	https://www.ncbi.nlm.nih.gov/gene/?term=72503
Adgra2	Adgra2	adhesion G protein-coupled receptor A2	78560	https://www.ncbi.nlm.nih.gov/gene/?term=78560
Bsdc1	Bsdc1	BSD domain containing 1	100383	https://www.ncbi.nlm.nih.gov/gene/?term=100383
Frmd5	Frmd5	FERM domain containing 5	228564	https://www.ncbi.nlm.nih.gov/gene/?term=228564

Biology Open • Supplementary information

Epha3	Epha3	Eph receptor A3	13837	https://www.ncbi.nlm.nih.gov/gene/?term=13837
Snx16	Snx16	sorting nexin 16	74718	https://www.ncbi.nlm.nih.gov/gene/?term=74718
Ppp4r3a	Ppp4r3a	protein phosphatase 4 regulatory subunit 3A	68734	https://www.ncbi.nlm.nih.gov/gene/?term=68734
Rab3c	Rab3c	RAB3C, member RAS oncogene family	67295	https://www.ncbi.nlm.nih.gov/gene/?term=67295
Nlgn2	Nlgn2	neuroligin 2	216856	https://www.ncbi.nlm.nih.gov/gene/?term=216856
Nptx2	Nptx2	neuronal pentraxin 2	53324	https://www.ncbi.nlm.nih.gov/gene/?term=53324
Ctdsp1	Ctdsp1	CTD (carboxy-terminal domain, RNA polymerase II, polypeptide A) small phosphatase 1	227292	https://www.ncbi.nlm.nih.gov/gene/?term=227292
Spen	Spen	spen family transcription repressor	56381	https://www.ncbi.nlm.nih.gov/gene/?term=56381
Alk	Alk	anaplastic lymphoma kinase	11682	https://www.ncbi.nlm.nih.gov/gene/?term=11682

Biology Open • Supplementary information

Oxsr1	Oxsr1	oxidative-stress responsive 1	108737	https://www.ncbi.nlm.nih.gov/gene/?term=108737
Galr1	Galr1	galanin receptor 1	14427	https://www.ncbi.nlm.nih.gov/gene/?term=14427
Umad1	Umad1	UMAP1-MVP12 associated (UMA) domain containing 1	100036521	https://www.ncbi.nlm.nih.gov/gene/?term=100036
Tmem230	Tmem230	transmembrane protein 230	70612	https://www.ncbi.nlm.nih.gov/gene/?term=70612
Mapkap1	Mapkap1	mitogen-activated protein kinase associated protein 1	227743	https://www.ncbi.nlm.nih.gov/gene/?term=227743
Snca	Snca	synuclein, alpha	20617	https://www.ncbi.nlm.nih.gov/gene/?term=20617
Fam131b	Fam131b	family with sequence similarity 131, member B	76156	https://www.ncbi.nlm.nih.gov/gene/?term=76156
Socs2	Socs2	suppressor of cytokine signaling 2	216233	https://www.ncbi.nlm.nih.gov/gene/?term=216233
Kif16b	Kif16b	kinesin family member 16B	16558	https://www.ncbi.nlm.nih.gov/gene/?term=16558

Biology Open • Supplementary information

Rnf144a	Rnf144a	ring finger protein 144A	108089	https://www.ncbi.nlm.nih.gov/gene/?term=108089
Ccdc120	Ccdc120	coiled-coil domain containing 120	54648	https://www.ncbi.nlm.nih.gov/gene/?term=54648
Cnppd1	Cnppd1	cyclin Pas1/PHO80 domain containing 1	69171	https://www.ncbi.nlm.nih.gov/gene/?term=69171
Rnf41	Rnf41	ring finger protein 41	67588	https://www.ncbi.nlm.nih.gov/gene/?term=67588
Itga4	Itga4	integrin alpha 4	16401	https://www.ncbi.nlm.nih.gov/gene/?term=16401
Nr4a3	Nr4a3	nuclear receptor subfamily 4, group A, member 3	18124	https://www.ncbi.nlm.nih.gov/gene/?term=18124
Pan2	Pan2	PAN2 poly(A) specific ribonuclease subunit	103135	https://www.ncbi.nlm.nih.gov/gene/?term=103135
Chsy3	Chsy3	chondroitin sulfate synthase 3	78923	https://www.ncbi.nlm.nih.gov/gene/?term=78923
Gtf2a1	Gtf2a1	general transcription factor II A, 1	83602	https://www.ncbi.nlm.nih.gov/gene/?term=83602

Pole4	Pole4	polymerase (DNA-directed), epsilon 4 (p12 subunit)	66979	https://www.ncbi.nlm.nih.gov/gene/?term=66979
Zfp287	Zfp287	zinc finger protein 287	170740	https://www.ncbi.nlm.nih.gov/gene/?term=170740
Lzts3	Lzts3	leucine zipper, putative tumor suppressor family member 3	241638	https://www.ncbi.nlm.nih.gov/gene/?term=241638
Ints10	Ints10	integrator complex subunit 10	70885	https://www.ncbi.nlm.nih.gov/gene/?term=70885
Gkn1	Gkn1	gastrokine 1	66283	https://www.ncbi.nlm.nih.gov/gene/?term=66283
Wdr47	Wdr47	WD repeat domain 47	99512	https://www.ncbi.nlm.nih.gov/gene/?term=99512
Clasp2	Clasp2	CLIP associating protein 2	76499	https://www.ncbi.nlm.nih.gov/gene/?term=76499
Ddit4	Ddit4	DNA-damage-inducible transcript 4	74747	https://www.ncbi.nlm.nih.gov/gene/?term=74747
Mknk1	Mknk1	MAP kinase-interacting serine/threonine kinase 1	17346	https://www.ncbi.nlm.nih.gov/gene/?term=17346

Biology Open • Supplementary information

Pfn2	Pfn2	profilin 2	18645	https://www.ncbi.nlm.nih.gov/gene/?term=18645
Arid4a	Arid4a	AT rich interactive domain 4A (RBP1-like)	238247	https://www.ncbi.nlm.nih.gov/gene/?term=238247
Chd3	Chd3	chromodomain helicase DNA binding protein 3	216848	https://www.ncbi.nlm.nih.gov/gene/?term=216848
Pilra	Pilra	paired immunoglobin-like type 2 receptor alpha	231805	https://www.ncbi.nlm.nih.gov/gene/?term=231805
Vdac3	Vdac3	voltage-dependent anion channel 3	22335	https://www.ncbi.nlm.nih.gov/gene/?term=22335
Wipf2	Wipf2	WAS/WASL interacting protein family, member 2	68524	https://www.ncbi.nlm.nih.gov/gene/?term=68524
Ckap4	Ckap4	cytoskeleton-associated protein 4	216197	https://www.ncbi.nlm.nih.gov/gene/?term=216197
Oxr1	Oxr1	oxidation resistance 1	170719	https://www.ncbi.nlm.nih.gov/gene/?term=170719
Slc17a1	Slc17a1	solute carrier family 17 (sodium phosphate), member 1	20504	https://www.ncbi.nlm.nih.gov/gene/?term=20504

Atp2b2	Atp2b2	ATPase, Ca++ transporting, plasma membrane 2	11941	https://www.ncbi.nlm.nih.gov/gene/?term=11941
Cct4	Cct4	chaperonin containing Tcp1, subunit 4 (delta)	12464	https://www.ncbi.nlm.nih.gov/gene/?term=12464
Gmeb1	Gmeb1	glucocorticoid modulatory element binding protein 1	56809	https://www.ncbi.nlm.nih.gov/gene/?term=56809
Sh3glb1	Sh3glb1	SH3-domain GRB2-like B1 (endophilin)	54673	https://www.ncbi.nlm.nih.gov/gene/?term=54673
Ptgfrn	Ptgfrn	prostaglandin F2 receptor negative regulator	19221	https://www.ncbi.nlm.nih.gov/gene/?term=19221
Parp1	Parp1	poly (ADP-ribose) polymerase family, member 1	11545	https://www.ncbi.nlm.nih.gov/gene/?term=11545
Kdm3b	Kdm3b	KDM3B lysine (K)-specific demethylase 3B	277250	https://www.ncbi.nlm.nih.gov/gene/?term=277250
Hhip	Hhip	Hedgehog-interacting protein	15245	https://www.ncbi.nlm.nih.gov/gene/?term=15245
Rab5if	Rab5if	RAB5 interacting factor	67388	https://www.ncbi.nlm.nih.gov/gene/?term=67388

Biology Open • Supplementary information

Bloc1s4	Bloc1s4	biogenesis of lysosomal organelles complex-1, subunit 4, cappuccino	117197	https://www.ncbi.nlm.nih.gov/gene/?term=117197
Ide	Ide	insulin degrading enzyme	15925	https://www.ncbi.nlm.nih.gov/gene/?term=15925
Rsbn1l	Rsbn1l	round spermatid basic protein 1-like	242860	https://www.ncbi.nlm.nih.gov/gene/?term=242860
Casp9	Casp9	caspase 9	12371	https://www.ncbi.nlm.nih.gov/gene/?term=12371
Nr1h2	Nr1h2	nuclear receptor subfamily 1, group H, member 2	22260	https://www.ncbi.nlm.nih.gov/gene/?term=22260
Zfp248	Zfp248	zinc finger protein 248	72720	https://www.ncbi.nlm.nih.gov/gene/?term=72720
Nrep	Nrep	neuronal regeneration related protein	27528	https://www.ncbi.nlm.nih.gov/gene/?term=27528
Spata2	Spata2	spermatogenesis associated 2	263876	https://www.ncbi.nlm.nih.gov/gene/?term=263876
Cdon	Cdon	cell adhesion molecule-related/down-regulated by oncogenes	57810	https://www.ncbi.nlm.nih.gov/gene/?term=57810

Biology Open • Supplementary information

Adcy9	Adcy9	adenylate cyclase 9	11515	https://www.ncbi.nlm.nih.gov/gene/?term=11515
Rgs7bp	Rgs7bp	regulator of G-protein signalling 7 binding protein	52882	https://www.ncbi.nlm.nih.gov/gene/?term=52882
Smyd5	Smyd5	SET and MYND domain containing 5	232187	https://www.ncbi.nlm.nih.gov/gene/?term=232187
Herpud2	Herpud2	HERPUD family member 2	80517	https://www.ncbi.nlm.nih.gov/gene/?term=80517
Plxna1	Plxna1	plexin A1	18844	https://www.ncbi.nlm.nih.gov/gene/?term=18844
Pom121l2	Pom121l2	POM121 membrane glycoprotein-like 2 (rat)	195236	https://www.ncbi.nlm.nih.gov/gene/?term=195236
Smim12	Smim12	small integral membrane protein 12	80284	https://www.ncbi.nlm.nih.gov/gene/?term=80284
Ssbp2	Ssbp2	single-stranded DNA binding protein 2	66970	https://www.ncbi.nlm.nih.gov/gene/?term=66970
Raf1	Raf1	v-raf-leukemia viral oncogene 1	110157	https://www.ncbi.nlm.nih.gov/gene/?term=110157

Biology Open • Supplementary information

Ctsb	Ctsb	cathepsin B	13030	https://www.ncbi.nlm.nih.gov/gene/?term=13030
Champ1	Champ1	chromosome alignment maintaining phosphoprotein 1	101994	https://www.ncbi.nlm.nih.gov/gene/?term=101994
Itch	Itch	itchy, E3 ubiquitin protein ligase	16396	https://www.ncbi.nlm.nih.gov/gene/?term=16396
Gal3st3	Gal3st3	galactose-3-O-sulfotransferase 3	545276	https://www.ncbi.nlm.nih.gov/gene/?term=545276
Asxl1	Asxl1	additional sex combs like 1	228790	https://www.ncbi.nlm.nih.gov/gene/?term=228790
Sp1	Sp1	trans-acting transcription factor 1	20683	https://www.ncbi.nlm.nih.gov/gene/?term=20683
Prkcb	Prkcb	protein kinase C, beta	18751	https://www.ncbi.nlm.nih.gov/gene/?term=18751
Ezh1	Ezh1	enhancer of zeste 1 polycomb repressive complex 2 subunit	14055	https://www.ncbi.nlm.nih.gov/gene/?term=14055
Rnf141	Rnf141	ring finger protein 141	67150	https://www.ncbi.nlm.nih.gov/gene/?term=67150

Biology Open • Supplementary information

Atp10a	Atp10a	ATPase, class V, type 10A	11982	https://www.ncbi.nlm.nih.gov/gene/?term=11982
Fam168b	Fam168b	family with sequence similarity 168, member B	214469	https://www.ncbi.nlm.nih.gov/gene/?term=214469
Pcnx2	Pcnx2	pecanex homolog 2	270109	https://www.ncbi.nlm.nih.gov/gene/?term=270109
Zc3h4	Zc3h4	zinc finger CCCH-type containing 4	330474	https://www.ncbi.nlm.nih.gov/gene/?term=330474
Mapk4	Mapk4	mitogen-activated protein kinase 4	225724	https://www.ncbi.nlm.nih.gov/gene/?term=225724
Pde4a	Pde4a	phosphodiesterase 4A, cAMP specific	18577	https://www.ncbi.nlm.nih.gov/gene/?term=18577
Zbtb4	Zbtb4	zinc finger and BTB domain containing 4	75580	https://www.ncbi.nlm.nih.gov/gene/?term=75580
Fndc4	Fndc4	fibronectin type III domain containing 4	64339	https://www.ncbi.nlm.nih.gov/gene/?term=64339
Klf4	Klf4	Kruppel-like factor 4 (gut)	16600	https://www.ncbi.nlm.nih.gov/gene/?term=16600

Biology Open • Supplementary information

Edar	Edar	ectodysplasin-A receptor	13608	https://www.ncbi.nlm.nih.gov/gene/?term=13608
Strn3	Strn3	striatin, calmodulin binding protein 3	94186	https://www.ncbi.nlm.nih.gov/gene/?term=94186
Xpo7	Xpo7	exportin 7	65246	https://www.ncbi.nlm.nih.gov/gene/?term=65246
B3gnt6	B3gnt6	UDP-GlcNAc:betaGal beta-1,3-N-acetylglucosaminyltransferase 6 (core 3 synthase)	272411	https://www.ncbi.nlm.nih.gov/gene/?term=272411
Bcorl1	Bcorl1	BCL6 co-repressor-like 1	320376	https://www.ncbi.nlm.nih.gov/gene/?term=320376
Ipo11	Ipo11	importin 11	76582	https://www.ncbi.nlm.nih.gov/gene/?term=76582
Dkk3	Dkk3	dickkopf WNT signaling pathway inhibitor 3	50781	https://www.ncbi.nlm.nih.gov/gene/?term=50781
Hdlbp	Hdlbp	high density lipoprotein (HDL) binding protein	110611	https://www.ncbi.nlm.nih.gov/gene/?term=110611
Rsbn1	Rsbn1	rosbin, round spermatid basic protein 1	229675	https://www.ncbi.nlm.nih.gov/gene/?term=229675

Biology Open • Supplementary information

Trio	Trio	triple functional domain (PTPRF interacting)	223435	https://www.ncbi.nlm.nih.gov/gene/?term=223435
Plin5	Plin5	perilipin 5	66968	https://www.ncbi.nlm.nih.gov/gene/?term=66968
Ago1	Ago1	argonaute RISC catalytic subunit 1	236511	https://www.ncbi.nlm.nih.gov/gene/?term=236511
Krt6b	Krt6b	keratin 6B	16688	https://www.ncbi.nlm.nih.gov/gene/?term=16688
Tmem167	Tmem167	transmembrane protein 167	66074	https://www.ncbi.nlm.nih.gov/gene/?term=66074
H2-M10.5	H2-M10.5	histocompatibility 2, M region locus 10.5	224761	https://www.ncbi.nlm.nih.gov/gene/?term=224761
Cfb	Cfb	complement factor B	14962	https://www.ncbi.nlm.nih.gov/gene/?term=14962
Syt11	Syt11	synaptotagmin XI	229521	https://www.ncbi.nlm.nih.gov/gene/?term=229521
Zfp446	Zfp446	zinc finger protein 446	269870	https://www.ncbi.nlm.nih.gov/gene/?term=269870

Biology Open • Supplementary information

Nup98	Nup98	nucleoporin 98	269966	https://www.ncbi.nlm.nih.gov/gene/?term=269966
H2-M10.3	H2-M10.3	histocompatibility 2, M region locus 10.3	110696	https://www.ncbi.nlm.nih.gov/gene/?term=110696
Cd200r3	Cd200r3	CD200 receptor 3	74603	https://www.ncbi.nlm.nih.gov/gene/?term=74603
Gpr22	Gpr22	G protein-coupled receptor 22	73010	https://www.ncbi.nlm.nih.gov/gene/?term=73010
Slc2a1	Slc2a1	solute carrier family 2 (facilitated glucose transporter), member 1	20525	https://www.ncbi.nlm.nih.gov/gene/?term=20525
Thy1	Thy1	thymus cell antigen 1, theta	21838	https://www.ncbi.nlm.nih.gov/gene/?term=21838
Sgk1	Sgk1	serum/glucocorticoid regulated kinase 1	20393	https://www.ncbi.nlm.nih.gov/gene/?term=20393
Kdm7a	Kdm7a	lysine (K)-specific demethylase 7A	338523	https://www.ncbi.nlm.nih.gov/gene/?term=338523
Tfrc	Tfrc	transferrin receptor	22042	https://www.ncbi.nlm.nih.gov/gene/?term=22042

Fastkd2	Fastkd2	FAST kinase domains 2	75619	https://www.ncbi.nlm.nih.gov/gene/?term=75619
Arpc1a	Arpc1a	actin related protein 2/3 complex, subunit 1A	56443	https://www.ncbi.nlm.nih.gov/gene/?term=56443
Zfp106	Zfp106	zinc finger protein 106	20402	https://www.ncbi.nlm.nih.gov/gene/?term=20402
Pigk	Pigk	phosphatidylinositol glycan anchor biosynthesis, class K	329777	https://www.ncbi.nlm.nih.gov/gene/?term=329777
Nedd9	Nedd9	neural precursor cell expressed, developmentally down-regulated gene 9	18003	https://www.ncbi.nlm.nih.gov/gene/?term=18003
Zfp850	Zfp850	zinc finger protein 850	100043772	https://www.ncbi.nlm.nih.gov/gene/?term=100043
Rd3	Rd3	retinal degeneration 3	74023	https://www.ncbi.nlm.nih.gov/gene/?term=74023
Hmbs	Hmbs	hydroxymethylbilane synthase	15288	https://www.ncbi.nlm.nih.gov/gene/?term=15288
Mctp1	Mctp1	multiple C2 domains, transmembrane 1	78771	https://www.ncbi.nlm.nih.gov/gene/?term=78771

Biology Open • Supplementary information

Ddx19b	Ddx19b	DEAD (Asp-Glu-Ala-Asp) box polypeptide 19b	234733	https://www.ncbi.nlm.nih.gov/gene/?term=234733
Naa50	Naa50	N(alpha)-acetyltransferase 50, NatE catalytic subunit	72117	https://www.ncbi.nlm.nih.gov/gene/?term=72117
Camk1	Camk1	calcium/calmodulin-dependent protein kinase I	52163	https://www.ncbi.nlm.nih.gov/gene/?term=52163
Epdr1	Epdr1	ependymin related protein 1 (zebrafish)	105298	https://www.ncbi.nlm.nih.gov/gene/?term=105298
Slc36a4	Slc36a4	solute carrier family 36 (proton/amino acid symporter), member 4	234967	https://www.ncbi.nlm.nih.gov/gene/?term=234967
Cdr1	Cdr1	cerebellar degeneration related antigen 1	631990	https://www.ncbi.nlm.nih.gov/gene/?term=631990
Fbxo11	Fbxo11	F-box protein 11	225055	https://www.ncbi.nlm.nih.gov/gene/?term=225055
4932429P05Rik	4932429P05Rik	RIKEN cDNA 4932429P05 gene	245509	https://www.ncbi.nlm.nih.gov/gene/?term=245509
Cdkn1b	Cdkn1b	cyclin-dependent kinase inhibitor 1B	12576	https://www.ncbi.nlm.nih.gov/gene/?term=12576

Biology Open • Supplementary information

Trib2	Trib2	tribbles pseudokinase 2	217410	https://www.ncbi.nlm.nih.gov/gene/?term=217410
Itprid2	Itprid2	ITPR interacting domain containing 2	70599	https://www.ncbi.nlm.nih.gov/gene/?term=70599
Fam135a	Fam135a	family with sequence similarity 135, member A	68187	https://www.ncbi.nlm.nih.gov/gene/?term=68187
Daam2	Daam2	dishevelled associated activator of morphogenesis 2	76441	https://www.ncbi.nlm.nih.gov/gene/?term=76441
Gpatch2l	Gpatch2l	G patch domain containing 2 like	70373	https://www.ncbi.nlm.nih.gov/gene/?term=70373
Scarb2	Scarb2	scavenger receptor class B, member 2	12492	https://www.ncbi.nlm.nih.gov/gene/?term=12492
Myo1c	Myo1c	myosin IC	17913	https://www.ncbi.nlm.nih.gov/gene/?term=17913
Brdt	Brdt	bromodomain, testis-specific	114642	https://www.ncbi.nlm.nih.gov/gene/?term=114642
Pcyt1a	Pcyt1a	phosphate cytidylyltransferase 1, choline, alpha isoform	13026	https://www.ncbi.nlm.nih.gov/gene/?term=13026

Biology Open • Supplementary information

Mfsd12	Mfsd12	major facilitator superfamily domain containing 12	73822	https://www.ncbi.nlm.nih.gov/gene/?term=73822
Unc5c	Unc5c	unc-5 netrin receptor C	22253	https://www.ncbi.nlm.nih.gov/gene/?term=22253
Sos1	Sos1	SOS Ras/Rac guanine nucleotide exchange factor 1	20662	https://www.ncbi.nlm.nih.gov/gene/?term=20662
Ptpmt1	Ptpmt1	protein tyrosine phosphatase, mitochondrial 1	66461	https://www.ncbi.nlm.nih.gov/gene/?term=66461
6430571L13Rik	6430571L13Rik	RIKEN cDNA 6430571L13 gene	235599	https://www.ncbi.nlm.nih.gov/gene/?term=235599
Pak6	Pak6	p21 (RAC1) activated kinase 6	214230	https://www.ncbi.nlm.nih.gov/gene/?term=214230
Slc24a3	Slc24a3	solute carrier family 24 (sodium/potassium/calcium exchanger), member 3	94249	https://www.ncbi.nlm.nih.gov/gene/?term=94249
Erbb4	Erbb4	erb-b2 receptor tyrosine kinase 4	13869	https://www.ncbi.nlm.nih.gov/gene/?term=13869
Tbck	Tbck	TBC1 domain containing kinase	271981	https://www.ncbi.nlm.nih.gov/gene/?term=271981

Slc15a1	Slc15a1	solute carrier family 15 (oligopeptide transporter), member 1	56643	https://www.ncbi.nlm.nih.gov/gene/?term=56643
Prpf4b	Prpf4b	pre-mRNA processing factor 4B	19134	https://www.ncbi.nlm.nih.gov/gene/?term=19134
Asah2	Asah2	N-acylsphingosine amidohydrolase 2	54447	https://www.ncbi.nlm.nih.gov/gene/?term=54447
Nek7	Nek7	NIMA (never in mitosis gene a)-related expressed kinase 7	59125	https://www.ncbi.nlm.nih.gov/gene/?term=59125
Cdc40	Cdc40	cell division cycle 40	71713	https://www.ncbi.nlm.nih.gov/gene/?term=71713
Map4k4	Map4k4	mitogen-activated protein kinase kinase kinase kinase 4	26921	https://www.ncbi.nlm.nih.gov/gene/?term=26921
Stx12	Stx12	syntaxin 12	100226	https://www.ncbi.nlm.nih.gov/gene/?term=100226
Myct1	Myct1	myc target 1	68632	https://www.ncbi.nlm.nih.gov/gene/?term=68632
Txnrd1	Txnrd1	thioredoxin reductase 1	50493	https://www.ncbi.nlm.nih.gov/gene/?term=50493

Biology Open • Supplementary information

Camk2d	Camk2d	calcium/calmodulin-dependent protein kinase II, delta	108058	https://www.ncbi.nlm.nih.gov/gene/?term=108058
Elf5	Elf5	E74-like factor 5	13711	https://www.ncbi.nlm.nih.gov/gene/?term=13711
Satb1	Satb1	special AT-rich sequence binding protein 1	20230	https://www.ncbi.nlm.nih.gov/gene/?term=20230
Gxylt1	Gxylt1	glucoside xylosyltransferase 1	223827	https://www.ncbi.nlm.nih.gov/gene/?term=223827
Igsf8	Igsf8	immunoglobulin superfamily, member 8	140559	https://www.ncbi.nlm.nih.gov/gene/?term=140559
C330018D20Rik	C330018D20Rik	RIKEN cDNA C330018D20 gene	77422	https://www.ncbi.nlm.nih.gov/gene/?term=77422
Cdh17	Cdh17	cadherin 17	12557	https://www.ncbi.nlm.nih.gov/gene/?term=12557
Rras2	Rras2	related RAS viral (r-ras) oncogene 2	66922	https://www.ncbi.nlm.nih.gov/gene/?term=66922
Cnot6l	Cnot6l	CCR4-NOT transcription complex, subunit 6-like	231464	https://www.ncbi.nlm.nih.gov/gene/?term=231464

Biology Open • Supplementary information

Sowahc	Sowahc	sosondowah ankyrin repeat domain family member C	268301	https://www.ncbi.nlm.nih.gov/gene/?term=268301
Kbtbd8	Kbtbd8	kelch repeat and BTB (POZ) domain containing 8	243574	https://www.ncbi.nlm.nih.gov/gene/?term=243574
Ep400	Ep400	E1A binding protein p400	75560	https://www.ncbi.nlm.nih.gov/gene/?term=75560
Zfp839	Zfp839	zinc finger protein 839	72805	https://www.ncbi.nlm.nih.gov/gene/?term=72805
Pknox1	Pknox1	Pbx/knotted 1 homeobox	18771	https://www.ncbi.nlm.nih.gov/gene/?term=18771
Wnk1	Wnk1	WNK lysine deficient protein kinase 1	232341	https://www.ncbi.nlm.nih.gov/gene/?term=232341
Agap1	Agap1	ArfGAP with GTPase domain, ankyrin repeat and PH domain 1	347722	https://www.ncbi.nlm.nih.gov/gene/?term=347722
Nr6a1	Nr6a1	nuclear receptor subfamily 6, group A, member 1	14536	https://www.ncbi.nlm.nih.gov/gene/?term=14536
Pkp4	Pkp4	plakophilin 4	227937	https://www.ncbi.nlm.nih.gov/gene/?term=227937

Biology Open • Supplementary information

Nek6	Nek6	NIMA (never in mitosis gene a)-related expressed kinase 6	59126	https://www.ncbi.nlm.nih.gov/gene/?term=59126
Csnk1g3	Csnk1g3	casein kinase 1, gamma 3	70425	https://www.ncbi.nlm.nih.gov/gene/?term=70425
Adgrg2	Adgrg2	adhesion G protein-coupled receptor G2	237175	https://www.ncbi.nlm.nih.gov/gene/?term=237175
Fbxo9	Fbxo9	f-box protein 9	71538	https://www.ncbi.nlm.nih.gov/gene/?term=71538
Auh	Auh	AU RNA binding protein/enoyl-coenzyme A hydratase	11992	https://www.ncbi.nlm.nih.gov/gene/?term=11992
Ptger4	Ptger4	prostaglandin E receptor 4 (subtype EP4)	19219	https://www.ncbi.nlm.nih.gov/gene/?term=19219
Camta1	Camta1	calmodulin binding transcription activator 1	100072	https://www.ncbi.nlm.nih.gov/gene/?term=100072
Map3k1	Map3k1	mitogen-activated protein kinase kinase kinase 1	26401	https://www.ncbi.nlm.nih.gov/gene/?term=26401
Nsd2	Nsd2	nuclear receptor binding SET domain protein 2	107823	https://www.ncbi.nlm.nih.gov/gene/?term=107823

Zbtb16	Zbtb16	zinc finger and BTB domain containing 16	235320	https://www.ncbi.nlm.nih.gov/gene/?term=235320
Tmpo	Tmpo	thymopoietin	21917	https://www.ncbi.nlm.nih.gov/gene/?term=21917
Btla	Btla	B and T lymphocyte associated	208154	https://www.ncbi.nlm.nih.gov/gene/?term=208154
Nap1l5	Nap1l5	nucleosome assembly protein 1-like 5	58243	https://www.ncbi.nlm.nih.gov/gene/?term=58243
Entpd5	Entpd5	ectonucleoside triphosphate diphosphohydrolase 5	12499	https://www.ncbi.nlm.nih.gov/gene/?term=12499
Elf2	Elf2	E74-like factor 2	69257	https://www.ncbi.nlm.nih.gov/gene/?term=69257
Cpeb2	Cpeb2	cytoplasmic polyadenylation element binding protein 2	231207	https://www.ncbi.nlm.nih.gov/gene/?term=231207
Thap12	Thap12	THAP domain containing 12	72981	https://www.ncbi.nlm.nih.gov/gene/?term=72981
Nuak2	Nuak2	NUAK family, SNF1-like kinase, 2	74137	https://www.ncbi.nlm.nih.gov/gene/?term=74137

Biology Open • Supplementary information

Tmed5	Tmed5	transmembrane p24 trafficking protein 5	73130	https://www.ncbi.nlm.nih.gov/gene/?term=73130
Zic1	Zic1	zinc finger protein of the cerebellum 1	22771	https://www.ncbi.nlm.nih.gov/gene/?term=22771
Srpk2	Srpk2	serine/arginine-rich protein specific kinase 2	20817	https://www.ncbi.nlm.nih.gov/gene/?term=20817
Wee1	Wee1	WEE 1 homolog 1 (S. pombe)	22390	https://www.ncbi.nlm.nih.gov/gene/?term=22390
Cldn12	Cldn12	claudin 12	64945	https://www.ncbi.nlm.nih.gov/gene/?term=64945
Sorcs1	Sorcs1	sortilin-related VPS10 domain containing receptor 1	58178	https://www.ncbi.nlm.nih.gov/gene/?term=58178
Celf2	Celf2	CUGBP, Elav-like family member 2	14007	https://www.ncbi.nlm.nih.gov/gene/?term=14007
Pcdh19	Pcdh19	protocadherin 19	279653	https://www.ncbi.nlm.nih.gov/gene/?term=279653
6720489N17Rik	6720489N17Rik	RIKEN cDNA 6720489N17 gene	211378	https://www.ncbi.nlm.nih.gov/gene/?term=211378

Biology Open • Supplementary information

Map3k5	Map3k5	mitogen-activated protein kinase kinase kinase 5	26408	https://www.ncbi.nlm.nih.gov/gene/?term=26408
Klhl15	Klhl15	kelch-like 15	236904	https://www.ncbi.nlm.nih.gov/gene/?term=236904
Ankhd1	Ankhd1	ankyrin repeat and KH domain containing 1	108857	https://www.ncbi.nlm.nih.gov/gene/?term=108857
Asxl3	Asxl3	additional sex combs like 3, transcriptional regulator	211961	https://www.ncbi.nlm.nih.gov/gene/?term=211961
Zfp85	Zfp85	zinc finger protein 85	22746	https://www.ncbi.nlm.nih.gov/gene/?term=22746
Tril	Tril	TLR4 interactor with leucine-rich repeats	66873	https://www.ncbi.nlm.nih.gov/gene/?term=66873
Tent5a	Tent5a	terminal nucleotidyltransferase 5A	212943	https://www.ncbi.nlm.nih.gov/gene/?term=212943
Trrap	Trrap	transformation/transcription domain-associated protein	100683	https://www.ncbi.nlm.nih.gov/gene/?term=100683
Col4a5	Col4a5	collagen, type IV, alpha 5	12830	https://www.ncbi.nlm.nih.gov/gene/?term=12830

Retreg3	Retreg3	reticulophagy regulator family member 3	67998	https://www.ncbi.nlm.nih.gov/gene/?term=67998
Zfp395	Zfp395	zinc finger protein 395	380912	https://www.ncbi.nlm.nih.gov/gene/?term=380912
Fut4	Fut4	fucosyltransferase 4	14345	https://www.ncbi.nlm.nih.gov/gene/?term=14345
Dnajc6	Dnajc6	DnaJ heat shock protein family (Hsp40) member C6	72685	https://www.ncbi.nlm.nih.gov/gene/?term=72685
Kdm6a	Kdm6a	lysine (K)-specific demethylase 6A	22289	https://www.ncbi.nlm.nih.gov/gene/?term=22289
Fbxo32	Fbxo32	F-box protein 32	67731	https://www.ncbi.nlm.nih.gov/gene/?term=67731
Pde10a	Pde10a	phosphodiesterase 10A	23984	https://www.ncbi.nlm.nih.gov/gene/?term=23984
Kdm1b	Kdm1b	lysine (K)-specific demethylase 1B	218214	https://www.ncbi.nlm.nih.gov/gene/?term=218214
Spsb4	Spsb4	splA/ryanodine receptor domain and SOCS box containing 4	211949	https://www.ncbi.nlm.nih.gov/gene/?term=211949

Biology Open • Supplementary information

Top1	Top1	topoisomerase (DNA) I	21969	https://www.ncbi.nlm.nih.gov/gene/?term=21969
Zcchc2	Zcchc2	zinc finger, CCHC domain containing 2	227449	https://www.ncbi.nlm.nih.gov/gene/?term=227449
Slc4a4	Slc4a4	solute carrier family 4 (anion exchanger), member 4	54403	https://www.ncbi.nlm.nih.gov/gene/?term=54403
Ppargc1a	Ppargc1a	peroxisome proliferative activated receptor, gamma, coactivator 1 alpha	19017	https://www.ncbi.nlm.nih.gov/gene/?term=19017
Lamp1	Lamp1	lysosomal-associated membrane protein 1	16783	https://www.ncbi.nlm.nih.gov/gene/?term=16783
Ambra1	Ambra1	autophagy/beclin 1 regulator 1	228361	https://www.ncbi.nlm.nih.gov/gene/?term=228361
Clec1a	Clec1a	C-type lectin domain family 1, member a	243653	https://www.ncbi.nlm.nih.gov/gene/?term=243653
Stat5b	Stat5b	signal transducer and activator of transcription 5B	20851	https://www.ncbi.nlm.nih.gov/gene/?term=20851
Tox3	Tox3	TOX high mobility group box family member 3	244579	https://www.ncbi.nlm.nih.gov/gene/?term=244579

Biology Open • Supplementary information

Wbp2	Wbp2	WW domain binding protein 2	22378	https://www.ncbi.nlm.nih.gov/gene/?term=22378
Slc6a14	Slc6a14	solute carrier family 6 (neurotransmitter transporter), member 14	56774	https://www.ncbi.nlm.nih.gov/gene/?term=56774
Ntrk2	Ntrk2	neurotrophic tyrosine kinase, receptor, type 2	18212	https://www.ncbi.nlm.nih.gov/gene/?term=18212
Eif4enif1	Eif4enif1	eukaryotic translation initiation factor 4E nuclear import factor 1	74203	https://www.ncbi.nlm.nih.gov/gene/?term=74203
Pde7a	Pde7a	phosphodiesterase 7A	18583	https://www.ncbi.nlm.nih.gov/gene/?term=18583
Ttc7b	Ttc7b	tetratricopeptide repeat domain 7B	104718	https://www.ncbi.nlm.nih.gov/gene/?term=104718
Wbp4	Wbp4	WW domain binding protein 4	22380	https://www.ncbi.nlm.nih.gov/gene/?term=22380
Zfp292	Zfp292	zinc finger protein 292	30046	https://www.ncbi.nlm.nih.gov/gene/?term=30046
Pdia6	Pdia6	protein disulfide isomerase associated 6	71853	https://www.ncbi.nlm.nih.gov/gene/?term=71853

Biology Open • Supplementary information

Fyb2	Fyb2	FYN binding protein 2	242594	https://www.ncbi.nlm.nih.gov/gene/?term=242594
Rap1a	Rap1a	RAS-related protein 1a	109905	https://www.ncbi.nlm.nih.gov/gene/?term=109905
Cab39	Cab39	calcium binding protein 39	12283	https://www.ncbi.nlm.nih.gov/gene/?term=12283
Casp7	Casp7	caspase 7	12369	https://www.ncbi.nlm.nih.gov/gene/?term=12369
Dars2	Dars2	aspartyl-tRNA synthetase 2 (mitochondrial)	226539	https://www.ncbi.nlm.nih.gov/gene/?term=226539
Slc38a1	Slc38a1	solute carrier family 38, member 1	105727	https://www.ncbi.nlm.nih.gov/gene/?term=105727
Swt1	Swt1	SWT1 RNA endoribonuclease homolog (S. cerevisiae)	66875	https://www.ncbi.nlm.nih.gov/gene/?term=66875
Cul3	Cul3	cullin 3	26554	https://www.ncbi.nlm.nih.gov/gene/?term=26554
Txndc16	Txndc16	thioredoxin domain containing 16	70561	https://www.ncbi.nlm.nih.gov/gene/?term=70561

Biology Open • Supplementary information

Nol4	Nol4	nucleolar protein 4	319211	https://www.ncbi.nlm.nih.gov/gene/?term=319211
Slc4a7	Slc4a7	solute carrier family 4, sodium bicarbonate cotransporter, member 7	218756	https://www.ncbi.nlm.nih.gov/gene/?term=218756
Lpp	Lpp	LIM domain containing preferred translocation partner in lipoma	210126	https://www.ncbi.nlm.nih.gov/gene/?term=210126
Tnks2	Tnks2	tankyrase, TRF1-interacting ankyrin-related ADP-ribose polymerase 2	74493	https://www.ncbi.nlm.nih.gov/gene/?term=74493
Pde4b	Pde4b	phosphodiesterase 4B, cAMP specific	18578	https://www.ncbi.nlm.nih.gov/gene/?term=18578
Sms	Sms	spermine synthase	20603	https://www.ncbi.nlm.nih.gov/gene/?term=20603
Tspyl5	Tspyl5	testis-specific protein, Y-encoded-like 5	239364	https://www.ncbi.nlm.nih.gov/gene/?term=239364
Snx5	Snx5	sorting nexin 5	69178	https://www.ncbi.nlm.nih.gov/gene/?term=69178
Cbll1	Cbll1	Casitas B-lineage lymphoma-like 1	104836	https://www.ncbi.nlm.nih.gov/gene/?term=104836

Biology Open • Supplementary information

Pax9	Pax9	paired box 9	18511	https://www.ncbi.nlm.nih.gov/gene/?term=18511
Socs6	Socs6	suppressor of cytokine signaling 6	54607	https://www.ncbi.nlm.nih.gov/gene/?term=54607
Vkorc1l1	Vkorc1l1	vitamin K epoxide reductase complex, subunit 1-like 1	69568	https://www.ncbi.nlm.nih.gov/gene/?term=69568
Etnk1	Etnk1	ethanolamine kinase 1	75320	https://www.ncbi.nlm.nih.gov/gene/?term=75320
Ascc3	Ascc3	activating signal cointegrator 1 complex subunit 3	77987	https://www.ncbi.nlm.nih.gov/gene/?term=77987
Aldh1a2	Aldh1a2	aldehyde dehydrogenase family 1, subfamily A2	19378	https://www.ncbi.nlm.nih.gov/gene/?term=19378
Nup50	Nup50	nucleoporin 50	18141	https://www.ncbi.nlm.nih.gov/gene/?term=18141
Uqcrfs1	Uqcrfs1	ubiquinol-cytochrome c reductase, Rieske iron-sulfur polypeptide 1	66694	https://www.ncbi.nlm.nih.gov/gene/?term=66694
Foxp2	Foxp2	forkhead box P2	114142	https://www.ncbi.nlm.nih.gov/gene/?term=114142

Biology Open • Supplementary information

Tab3	Tab3	TGF-beta activated kinase 1/MAP3K7 binding protein 3	66724	https://www.ncbi.nlm.nih.gov/gene/?term=66724
Cav2	Cav2	caveolin 2	12390	https://www.ncbi.nlm.nih.gov/gene/?term=12390
Sft2d1	Sft2d1	SFT2 domain containing 1	106489	https://www.ncbi.nlm.nih.gov/gene/?term=106489
Grm5	Grm5	glutamate receptor, metabotropic 5	108071	https://www.ncbi.nlm.nih.gov/gene/?term=108071
Aldh1l2	Aldh1l2	aldehyde dehydrogenase 1 family, member L2	216188	https://www.ncbi.nlm.nih.gov/gene/?term=216188
Ppif	Ppif	peptidylprolyl isomerase F (cyclophilin F)	105675	https://www.ncbi.nlm.nih.gov/gene/?term=105675
Ubap2	Ubap2	ubiquitin-associated protein 2	68926	https://www.ncbi.nlm.nih.gov/gene/?term=68926
Car2	Car2	carbonic anhydrase 2	12349	https://www.ncbi.nlm.nih.gov/gene/?term=12349
Mab21l2	Mab21l2	mab-21-like 2 (C. elegans)	23937	https://www.ncbi.nlm.nih.gov/gene/?term=23937

Biology Open • Supplementary information

Ctcf	Ctcf	CCCTC-binding factor	13018	https://www.ncbi.nlm.nih.gov/gene/?term=13018
Nacc2	Nacc2	nucleus accumbens associated 2, BEN and BTB (POZ) domain containing	67991	https://www.ncbi.nlm.nih.gov/gene/?term=67991
Ube2o	Ube2o	ubiquitin-conjugating enzyme E2O	217342	https://www.ncbi.nlm.nih.gov/gene/?term=217342
Rad17	Rad17	RAD17 checkpoint clamp loader component	19356	https://www.ncbi.nlm.nih.gov/gene/?term=19356
Slc25a31	Slc25a31	solute carrier family 25 (mitochondrial carrier; adenine nucleotide translocator), member 31	73333	https://www.ncbi.nlm.nih.gov/gene/?term=73333
Ppm1d	Ppm1d	protein phosphatase 1D magnesium-dependent, delta isoform	53892	https://www.ncbi.nlm.nih.gov/gene/?term=53892
Elovl3	Elovl3	elongation of very long chain fatty acids (FEN1/Elo2, SUR4/Elo3, yeast)-like 3	12686	https://www.ncbi.nlm.nih.gov/gene/?term=12686
Il6ra	Il6ra	interleukin 6 receptor, alpha	16194	https://www.ncbi.nlm.nih.gov/gene/?term=16194
Ppp4r4	Ppp4r4	protein phosphatase 4, regulatory subunit 4	74521	https://www.ncbi.nlm.nih.gov/gene/?term=74521

Cpsf4	Cpsf4	cleavage and polyadenylation specific factor 4	54188	https://www.ncbi.nlm.nih.gov/gene/?term=54188
Mapre1	Mapre1	microtubule-associated protein, RP/EB family, member 1	13589	https://www.ncbi.nlm.nih.gov/gene/?term=13589
Mbtd1	Mbtd1	mbt domain containing 1	103537	https://www.ncbi.nlm.nih.gov/gene/?term=103537
Intu	Intu	inturned planar cell polarity protein	380614	https://www.ncbi.nlm.nih.gov/gene/?term=380614
Mrc1	Mrc1	mannose receptor, C type 1	17533	https://www.ncbi.nlm.nih.gov/gene/?term=17533
Rab39b	Rab39b	RAB39B, member RAS oncogene family	67790	https://www.ncbi.nlm.nih.gov/gene/?term=67790
Rbm47	Rbm47	RNA binding motif protein 47	245945	https://www.ncbi.nlm.nih.gov/gene/?term=245945
Erbin	Erbin	Erbb2 interacting protein	59079	https://www.ncbi.nlm.nih.gov/gene/?term=59079
Usp24	Usp24	ubiquitin specific peptidase 24	329908	https://www.ncbi.nlm.nih.gov/gene/?term=329908

Biology Open • Supplementary information

Hmgb2	Hmgb2	high mobility group box 2	97165	https://www.ncbi.nlm.nih.gov/gene/?term=97165
Ccm2	Ccm2	cerebral cavernous malformation 2	216527	https://www.ncbi.nlm.nih.gov/gene/?term=216527
Calcrl	Calcrl	calcitonin receptor-like	54598	https://www.ncbi.nlm.nih.gov/gene/?term=54598
Myh2	Myh2	myosin, heavy polypeptide 2, skeletal muscle, adult	17882	https://www.ncbi.nlm.nih.gov/gene/?term=17882
Nrxn1	Nrxn1	neurexin I	18189	https://www.ncbi.nlm.nih.gov/gene/?term=18189
Ccdc47	Ccdc47	coiled-coil domain containing 47	67163	https://www.ncbi.nlm.nih.gov/gene/?term=67163
Jarid2	Jarid2	jumonji, AT rich interactive domain 2	16468	https://www.ncbi.nlm.nih.gov/gene/?term=16468
Atp11b	Atp11b	ATPase, class VI, type 11B	76295	https://www.ncbi.nlm.nih.gov/gene/?term=76295
Vwa3a	Vwa3a	von Willebrand factor A domain containing 3A	233813	https://www.ncbi.nlm.nih.gov/gene/?term=233813

Biology Open • Supplementary information

Atxn7l3b	Atxn7l3b	ataxin 7-like 3B	382423	https://www.ncbi.nlm.nih.gov/gene/?term=382423
Rer1	Rer1	retention in endoplasmic reticulum sorting receptor 1	67830	https://www.ncbi.nlm.nih.gov/gene/?term=67830
Pten	Pten	phosphatase and tensin homolog	19211	https://www.ncbi.nlm.nih.gov/gene/?term=19211
Bora	Bora	bora, aurora kinase A activator	77744	https://www.ncbi.nlm.nih.gov/gene/?term=77744
Dock3	Dock3	dedicator of cyto-kinesis 3	208869	https://www.ncbi.nlm.nih.gov/gene/?term=208869
Top2b	Top2b	topoisomerase (DNA) II beta	21974	https://www.ncbi.nlm.nih.gov/gene/?term=21974
Smurf2	Smurf2	SMAD specific E3 ubiquitin protein ligase 2	66313	https://www.ncbi.nlm.nih.gov/gene/?term=66313
Trmt10a	Trmt10a	tRNA methyltransferase 10A	108943	https://www.ncbi.nlm.nih.gov/gene/?term=108943
Reep1	Reep1	receptor accessory protein 1	52250	https://www.ncbi.nlm.nih.gov/gene/?term=52250

Fam234b	Fam234b	family with sequence similarity 234, member B	74525	https://www.ncbi.nlm.nih.gov/gene/?term=74525
Pkia	Pkia	protein kinase inhibitor, alpha	18767	https://www.ncbi.nlm.nih.gov/gene/?term=18767
Tmem168	Tmem168	transmembrane protein 168	101118	https://www.ncbi.nlm.nih.gov/gene/?term=101118
Epg5	Epg5	ectopic P-granules autophagy protein 5 homolog (C. elegans)	100502841	https://www.ncbi.nlm.nih.gov/gene/?term=100502
Ippk	Ippk	inositol 1,3,4,5,6-pentakisphosphate 2-kinase	75678	https://www.ncbi.nlm.nih.gov/gene/?term=75678
Slc17a6	Slc17a6	solute carrier family 17 (sodium-dependent inorganic phosphate cotransporter), member 6	140919	https://www.ncbi.nlm.nih.gov/gene/?term=140919
Celf4	Celf4	CUGBP, Elav-like family member 4	108013	https://www.ncbi.nlm.nih.gov/gene/?term=108013
Dpep1	Dpep1	dipeptidase 1	13479	https://www.ncbi.nlm.nih.gov/gene/?term=13479
Trim36	Trim36	tripartite motif-containing 36	28105	https://www.ncbi.nlm.nih.gov/gene/?term=28105

Biology Open • Supplementary information

Trps1	Trps1	transcriptional repressor GATA binding 1	83925	https://www.ncbi.nlm.nih.gov/gene/?term=83925
Cebpa	Cebpa	CCAAT/enhancer binding protein (C/EBP), alpha	12606	https://www.ncbi.nlm.nih.gov/gene/?term=12606
Slc20a2	Slc20a2	solute carrier family 20, member 2	20516	https://www.ncbi.nlm.nih.gov/gene/?term=20516
Nlgn1	Nlgn1	neuroligin 1	192167	https://www.ncbi.nlm.nih.gov/gene/?term=192167
Serpinb9e	Serpinb9e	serine (or cysteine) peptidase inhibitor, clade B, member 9e	20710	https://www.ncbi.nlm.nih.gov/gene/?term=20710
Alkbh8	Alkbh8	alkB homolog 8, tRNA methyltransferase	67667	https://www.ncbi.nlm.nih.gov/gene/?term=67667
Tor1aip1	Tor1aip1	torsin A interacting protein 1	208263	https://www.ncbi.nlm.nih.gov/gene/?term=208263
Wsb1	Wsb1	WD repeat and SOCS box-containing 1	78889	https://www.ncbi.nlm.nih.gov/gene/?term=78889
Eda2r	Eda2r	ectodysplasin A2 receptor	245527	https://www.ncbi.nlm.nih.gov/gene/?term=245527

Biology Open • Supplementary information

Serpinb9f	Serpinb9f	serine (or cysteine) peptidase inhibitor, clade B, member 9f	20709	https://www.ncbi.nlm.nih.gov/gene/?term=20709
Chd7	Chd7	chromodomain helicase DNA binding protein 7	320790	https://www.ncbi.nlm.nih.gov/gene/?term=320790
Epc2	Epc2	enhancer of polycomb homolog 2	227867	https://www.ncbi.nlm.nih.gov/gene/?term=227867
Zfp322a	Zfp322a	zinc finger protein 322A	218100	https://www.ncbi.nlm.nih.gov/gene/?term=218100
Setdb2	Setdb2	SET domain, bifurcated 2	239122	https://www.ncbi.nlm.nih.gov/gene/?term=239122
Mr1	Mr1	major histocompatibility complex, class I-related	15064	https://www.ncbi.nlm.nih.gov/gene/?term=15064
Tdrd5	Tdrd5	tudor domain containing 5	214575	https://www.ncbi.nlm.nih.gov/gene/?term=214575
Pigw	Pigw	phosphatidylinositol glycan anchor biosynthesis, class W	70325	https://www.ncbi.nlm.nih.gov/gene/?term=70325
Neurod1	Neurod1	neurogenic differentiation 1	18012	https://www.ncbi.nlm.nih.gov/gene/?term=18012

Biology Open • Supplementary information

Setbp1	Setbp1	SET binding protein 1	240427	https://www.ncbi.nlm.nih.gov/gene/?term=240427
Smpdl3b	Smpdl3b	sphingomyelin phosphodiesterase, acid-like 3B	100340	https://www.ncbi.nlm.nih.gov/gene/?term=100340
Myo5a	Myo5a	myosin VA	17918	https://www.ncbi.nlm.nih.gov/gene/?term=17918
Aak1	Aak1	AP2 associated kinase 1	269774	https://www.ncbi.nlm.nih.gov/gene/?term=269774
Tigd2	Tigd2	tigger transposable element derived 2	68140	https://www.ncbi.nlm.nih.gov/gene/?term=68140
Dcaf10	Dcaf10	DDB1 and CUL4 associated factor 10	242418	https://www.ncbi.nlm.nih.gov/gene/?term=242418
Psg29	Psg29	pregnancy-specific glycoprotein 29	114872	https://www.ncbi.nlm.nih.gov/gene/?term=114872
Ktn1	Ktn1	kinectin 1	16709	https://www.ncbi.nlm.nih.gov/gene/?term=16709
Cpa3	Cpa3	carboxypeptidase A3, mast cell	12873	https://www.ncbi.nlm.nih.gov/gene/?term=12873

Ap3s1	Ap3s1	adaptor-related protein complex 3, sigma 1 subunit	11777	https://www.ncbi.nlm.nih.gov/gene/?term=11777
Usp36	Usp36	ubiquitin specific peptidase 36	72344	https://www.ncbi.nlm.nih.gov/gene/?term=72344
Stag2	Stag2	stromal antigen 2	20843	https://www.ncbi.nlm.nih.gov/gene/?term=20843
Sucla2	Sucla2	succinate-Coenzyme A ligase, ADP-forming, beta subunit	20916	https://www.ncbi.nlm.nih.gov/gene/?term=20916
Atl2	Atl2	atlastin GTPase 2	56298	https://www.ncbi.nlm.nih.gov/gene/?term=56298
Pcdh17	Pcdh17	protocadherin 17	219228	https://www.ncbi.nlm.nih.gov/gene/?term=219228
Sva	Sva	seminal vesicle antigen	20939	https://www.ncbi.nlm.nih.gov/gene/?term=20939
Ccdc160	Ccdc160	coiled-coil domain containing 160	434778	https://www.ncbi.nlm.nih.gov/gene/?term=434778
Txlng	Txlng	taxilin gamma	353170	https://www.ncbi.nlm.nih.gov/gene/?term=353170

Biology Open • Supplementary information

Kctd11	Kctd11	potassium channel tetramerisation domain containing 11	216858	https://www.ncbi.nlm.nih.gov/gene/?term=216858
Rbm7	Rbm7	RNA binding motif protein 7	67010	https://www.ncbi.nlm.nih.gov/gene/?term=67010
Fndc5	Fndc5	fibronectin type III domain containing 5	384061	https://www.ncbi.nlm.nih.gov/gene/?term=384061
Mecp2	Mecp2	methyl CpG binding protein 2	17257	https://www.ncbi.nlm.nih.gov/gene/?term=17257
Gpr149	Gpr149	G protein-coupled receptor 149	229357	https://www.ncbi.nlm.nih.gov/gene/?term=229357
Ythdf1	Ythdf1	YTH N6-methyladenosine RNA binding protein 1	228994	https://www.ncbi.nlm.nih.gov/gene/?term=228994
Cntn6	Cntn6	contactin 6	53870	https://www.ncbi.nlm.nih.gov/gene/?term=53870
P2ry1	P2ry1	purinergic receptor P2Y, G-protein coupled 1	18441	https://www.ncbi.nlm.nih.gov/gene/?term=18441
Zc3h7b	Zc3h7b	zinc finger CCCH type containing 7B	20286	https://www.ncbi.nlm.nih.gov/gene/?term=20286

Biology Open • Supplementary information

Gm6710	Gm6710	predicted gene 6710	626832	https://www.ncbi.nlm.nih.gov/gene/?term=626832
Dip2c	Dip2c	disco interacting protein 2 homolog C	208440	https://www.ncbi.nlm.nih.gov/gene/?term=208440
2210418O10Rik	2210418O10Rik	RIKEN cDNA 2210418O10 gene	100504263	https://www.ncbi.nlm.nih.gov/gene/?term=100504
Tgfb3	Tgfb3	transforming growth factor, beta 3	21809	https://www.ncbi.nlm.nih.gov/gene/?term=21809
Ubqln2	Ubqln2	ubiquilin 2	54609	https://www.ncbi.nlm.nih.gov/gene/?term=54609
Sub1	Sub1	SUB1 homolog (S. cerevisiae)	20024	https://www.ncbi.nlm.nih.gov/gene/?term=20024
Olig3	Olig3	oligodendrocyte transcription factor 3	94222	https://www.ncbi.nlm.nih.gov/gene/?term=94222
Git1	Git1	GIT ArfGAP 1	216963	https://www.ncbi.nlm.nih.gov/gene/?term=216963
Tra2a	Tra2a	transformer 2 alpha	101214	https://www.ncbi.nlm.nih.gov/gene/?term=101214

Biology Open • Supplementary information

Ugt2b35	Ugt2b35	UDP glucuronosyltransferase 2 family, polypeptide B35	243085	https://www.ncbi.nlm.nih.gov/gene/?term=243085
Pcdh8	Pcdh8	protocadherin 8	18530	https://www.ncbi.nlm.nih.gov/gene/?term=18530
Ndst1	Ndst1	N-deacetylase/N-sulfotransferase (heparan glucosaminyl) 1	15531	https://www.ncbi.nlm.nih.gov/gene/?term=15531
Zfp781	Zfp781	zinc finger protein 781	331188	https://www.ncbi.nlm.nih.gov/gene/?term=331188
Zc3h6	Zc3h6	zinc finger CCCH type containing 6	78751	https://www.ncbi.nlm.nih.gov/gene/?term=78751
Btg1	Btg1	B cell translocation gene 1, anti-proliferative	12226	https://www.ncbi.nlm.nih.gov/gene/?term=12226
Zfp970	Zfp970	zinc finger protein 970	628308	https://www.ncbi.nlm.nih.gov/gene/?term=628308
Tmem159	Tmem159	transmembrane protein 159	233806	https://www.ncbi.nlm.nih.gov/gene/?term=233806
Adam10	Adam10	a disintegrin and metallopeptidase domain 10	11487	https://www.ncbi.nlm.nih.gov/gene/?term=11487

Biology Open • Supplementary information

Zfp967	Zfp967	zinc finger protein 967	100303732	https://www.ncbi.nlm.nih.gov/gene/?term=100303
Rasip1	Rasip1	Ras interacting protein 1	69903	https://www.ncbi.nlm.nih.gov/gene/?term=69903
Gprin3	Gprin3	GPRIN family member 3	243385	https://www.ncbi.nlm.nih.gov/gene/?term=243385
Gm14440	Gm14440	predicted gene 14440	100503353	https://www.ncbi.nlm.nih.gov/gene/?term=100503
Bsn	Bsn	bassoon	12217	https://www.ncbi.nlm.nih.gov/gene/?term=12217
Ube2d1	Ube2d1	ubiquitin-conjugating enzyme E2D 1	216080	https://www.ncbi.nlm.nih.gov/gene/?term=216080
Selenoi	Selenoi	selenoprotein I	28042	https://www.ncbi.nlm.nih.gov/gene/?term=28042
Rarb	Rarb	retinoic acid receptor, beta	218772	https://www.ncbi.nlm.nih.gov/gene/?term=218772
Mef2a	Mef2a	myocyte enhancer factor 2A	17258	https://www.ncbi.nlm.nih.gov/gene/?term=17258

Biology Open • Supplementary information

Igip	Igip	IgA inducing protein	109169	https://www.ncbi.nlm.nih.gov/gene/?term=109169
Afdn	Afdn	afadin, adherens junction formation factor	17356	https://www.ncbi.nlm.nih.gov/gene/?term=17356
Ehmt1	Ehmt1	euchromatic histone methyltransferase 1	77683	https://www.ncbi.nlm.nih.gov/gene/?term=77683
Fbxw11	Fbxw11	F-box and WD-40 domain protein 11	103583	https://www.ncbi.nlm.nih.gov/gene/?term=103583
Teddm1b	Teddm1b	transmembrane epididymal protein 1B	433365	https://www.ncbi.nlm.nih.gov/gene/?term=433365
Shcbp1	Shcbp1	Shc SH2-domain binding protein 1	20419	https://www.ncbi.nlm.nih.gov/gene/?term=20419
Ipo8	Ipo8	importin 8	320727	https://www.ncbi.nlm.nih.gov/gene/?term=320727
Mycl	Mycl	v-myc avian myelocytomatosis viral oncogene lung carcinoma derived	16918	https://www.ncbi.nlm.nih.gov/gene/?term=16918
Ets1	Ets1	E26 avian leukemia oncogene 1, 5' domain	23871	https://www.ncbi.nlm.nih.gov/gene/?term=23871

Biology Open • Supplementary information

Tshz3	Tshz3	teashirt zinc finger family member 3	243931	https://www.ncbi.nlm.nih.gov/gene/?term=243931
Rfx3	Rfx3	regulatory factor X, 3 (influences HLA class II expression)	19726	https://www.ncbi.nlm.nih.gov/gene/?term=19726
Smoc1	Smoc1	SPARC related modular calcium binding 1	64075	https://www.ncbi.nlm.nih.gov/gene/?term=64075
Limch1	Limch1	LIM and calponin homology domains 1	77569	https://www.ncbi.nlm.nih.gov/gene/?term=77569
Zfp207	Zfp207	zinc finger protein 207	22680	https://www.ncbi.nlm.nih.gov/gene/?term=22680
Scn1a	Scn1a	sodium channel, voltage-gated, type I, alpha	20265	https://www.ncbi.nlm.nih.gov/gene/?term=20265
Atp5g2	Atp5g2	ATP synthase, H+ transporting, mitochondrial F0 complex, subunit C2 (subunit 9)	67942	https://www.ncbi.nlm.nih.gov/gene/?term=67942
Rnaseh1	Rnaseh1	ribonuclease H1	19819	https://www.ncbi.nlm.nih.gov/gene/?term=19819
Cttnbp2	Cttnbp2	cortactin binding protein 2	30785	https://www.ncbi.nlm.nih.gov/gene/?term=30785

Biology Open • Supplementary information

Hif1a	Hif1a	hypoxia inducible factor 1, alpha subunit	15251	https://www.ncbi.nlm.nih.gov/gene/?term=15251
Emp1	Emp1	epithelial membrane protein 1	13730	https://www.ncbi.nlm.nih.gov/gene/?term=13730
Meis2	Meis2	Meis homeobox 2	17536	https://www.ncbi.nlm.nih.gov/gene/?term=17536
Arhgap42	Arhgap42	Rho GTPase activating protein 42	71544	https://www.ncbi.nlm.nih.gov/gene/?term=71544
Hccs	Hccs	holocytochrome c synthetase	15159	https://www.ncbi.nlm.nih.gov/gene/?term=15159
Pank1	Pank1	pantothenate kinase 1	75735	https://www.ncbi.nlm.nih.gov/gene/?term=75735
Kctd10	Kctd10	potassium channel tetramerisation domain containing 10	330171	https://www.ncbi.nlm.nih.gov/gene/?term=330171
Tnfsf11	Tnfsf11	tumor necrosis factor (ligand) superfamily, member 11	21943	https://www.ncbi.nlm.nih.gov/gene/?term=21943
Begain	Begain	brain-enriched guanylate kinase-associated	380785	https://www.ncbi.nlm.nih.gov/gene/?term=380785

Biology Open • Supplementary information

Kif2a	Kif2a	kinesin family member 2A	16563	https://www.ncbi.nlm.nih.gov/gene/?term=16563
Oprk1	Oprk1	opioid receptor, kappa 1	18387	https://www.ncbi.nlm.nih.gov/gene/?term=18387
Slc23a2	Slc23a2	solute carrier family 23 (nucleobase transporters), member 2	54338	https://www.ncbi.nlm.nih.gov/gene/?term=54338
Impact	Impact	impact, RWD domain protein	16210	https://www.ncbi.nlm.nih.gov/gene/?term=16210
Aldh1a3	Aldh1a3	aldehyde dehydrogenase family 1, subfamily A3	56847	https://www.ncbi.nlm.nih.gov/gene/?term=56847
Tet2	Tet2	tet methylcytosine dioxygenase 2	214133	https://www.ncbi.nlm.nih.gov/gene/?term=214133
Zbtb18	Zbtb18	zinc finger and BTB domain containing 18	30928	https://www.ncbi.nlm.nih.gov/gene/?term=30928
Nin	Nin	ninein	18080	https://www.ncbi.nlm.nih.gov/gene/?term=18080
Togaram1	Togaram1	TOG array regulator of axonemal microtubules 1	328108	https://www.ncbi.nlm.nih.gov/gene/?term=328108

Biology Open • Supplementary information

Cct6a	Cct6a	chaperonin containing Tcp1, subunit 6a (zeta)	12466	https://www.ncbi.nlm.nih.gov/gene/?term=12466
Plekhg1	Plekhg1	pleckstrin homology domain containing, family G (with RhoGef domain) member 1	213783	https://www.ncbi.nlm.nih.gov/gene/?term=213783
Slc5a7	Slc5a7	solute carrier family 5 (choline transporter), member 7	63993	https://www.ncbi.nlm.nih.gov/gene/?term=63993
Zfp148	Zfp148	zinc finger protein 148	22661	https://www.ncbi.nlm.nih.gov/gene/?term=22661
Kat6a	Kat6a	K(lysine) acetyltransferase 6A	244349	https://www.ncbi.nlm.nih.gov/gene/?term=244349
Rbm48	Rbm48	RNA binding motif protein 48	269623	https://www.ncbi.nlm.nih.gov/gene/?term=269623
Zdhhc21	Zdhhc21	zinc finger, DHHC domain containing 21	68268	https://www.ncbi.nlm.nih.gov/gene/?term=68268
Scfd1	Scfd1	Sec1 family domain containing 1	76983	https://www.ncbi.nlm.nih.gov/gene/?term=76983
Nid2	Nid2	nidogen 2	18074	https://www.ncbi.nlm.nih.gov/gene/?term=18074

Fat4	Fat4	FAT atypical cadherin 4	329628	https://www.ncbi.nlm.nih.gov/gene/?term=329628
Tek	Tek	TEK receptor tyrosine kinase	21687	https://www.ncbi.nlm.nih.gov/gene/?term=21687
Sap30l	Sap30l	SAP30-like	50724	https://www.ncbi.nlm.nih.gov/gene/?term=50724
Mycn	Mycn	v-myc avian myelocytomatosis viral related oncogene, neuroblastoma derived	18109	https://www.ncbi.nlm.nih.gov/gene/?term=18109
Sorcs3	Sorcs3	sortilin-related VPS10 domain containing receptor 3	66673	https://www.ncbi.nlm.nih.gov/gene/?term=66673
Olfr701	Olfr701	olfactory receptor 701	66786	https://www.ncbi.nlm.nih.gov/gene/?term=66786
Scn8a	Scn8a	sodium channel, voltage-gated, type VIII, alpha	20273	https://www.ncbi.nlm.nih.gov/gene/?term=20273
Gdf10	Gdf10	growth differentiation factor 10	14560	https://www.ncbi.nlm.nih.gov/gene/?term=14560
Tmem196	Tmem196	transmembrane protein 196	217951	https://www.ncbi.nlm.nih.gov/gene/?term=217951

Biology Open • Supplementary information

Ugp2	Ugp2	UDP-glucose pyrophosphorylase 2	216558	https://www.ncbi.nlm.nih.gov/gene/?term=216558
Ptpn9	Ptpn9	protein tyrosine phosphatase, non-receptor type 9	56294	https://www.ncbi.nlm.nih.gov/gene/?term=56294
Smad4	Smad4	SMAD family member 4	17128	https://www.ncbi.nlm.nih.gov/gene/?term=17128
Med4	Med4	mediator complex subunit 4	67381	https://www.ncbi.nlm.nih.gov/gene/?term=67381
Adamts17	Adamts17	a disintegrin-like and metallopeptidase (reprolysin type) with thrombospondin type 1 motif, 17	233332	https://www.ncbi.nlm.nih.gov/gene/?term=233332
Pdcl	Pdcl	phosducin-like	67466	https://www.ncbi.nlm.nih.gov/gene/?term=67466
Tfap4	Tfap4	transcription factor AP4	83383	https://www.ncbi.nlm.nih.gov/gene/?term=83383
Arid2	Arid2	AT rich interactive domain 2 (ARID, RFX-like)	77044	https://www.ncbi.nlm.nih.gov/gene/?term=77044
Dcbld2	Dcbld2	discoidin, CUB and LCCL domain containing 2	73379	https://www.ncbi.nlm.nih.gov/gene/?term=73379

Biology Open • Supplementary information

Zfp36l2	Zfp36l2	zinc finger protein 36, C3H type-like 2	12193	https://www.ncbi.nlm.nih.gov/gene/?term=12193
Nfe2l2	Nfe2l2	nuclear factor, erythroid derived 2, like 2	18024	https://www.ncbi.nlm.nih.gov/gene/?term=18024
Fndc3a	Fndc3a	fibronectin type III domain containing 3A	319448	https://www.ncbi.nlm.nih.gov/gene/?term=319448
Brpf1	Brpf1	bromodomain and PHD finger containing, 1	78783	https://www.ncbi.nlm.nih.gov/gene/?term=78783
Pnrc1	Pnrc1	proline-rich nuclear receptor coactivator 1	108767	https://www.ncbi.nlm.nih.gov/gene/?term=108767
Mapk6	Mapk6	mitogen-activated protein kinase 6	50772	https://www.ncbi.nlm.nih.gov/gene/?term=50772
Fbn2	Fbn2	fibrillin 2	14119	https://www.ncbi.nlm.nih.gov/gene/?term=14119
Mob4	Mob4	MOB family member 4, phocein	19070	https://www.ncbi.nlm.nih.gov/gene/?term=19070
Htra3	Htra3	HtrA serine peptidase 3	78558	https://www.ncbi.nlm.nih.gov/gene/?term=78558

Biology Open • Supplementary information

Rin2	Rin2	Ras and Rab interactor 2	74030	https://www.ncbi.nlm.nih.gov/gene/?term=74030
Ube2d2a	Ube2d2a	ubiquitin-conjugating enzyme E2D 2A	56550	https://www.ncbi.nlm.nih.gov/gene/?term=56550
Bicd2	Bicd2	BICD cargo adaptor 2	76895	https://www.ncbi.nlm.nih.gov/gene/?term=76895
Nr1d2	Nr1d2	nuclear receptor subfamily 1, group D, member 2	353187	https://www.ncbi.nlm.nih.gov/gene/?term=353187
Marf1	Marf1	meiosis regulator and mRNA stability 1	223989	https://www.ncbi.nlm.nih.gov/gene/?term=223989
Shisa6	Shisa6	shisa family member 6	380702	https://www.ncbi.nlm.nih.gov/gene/?term=380702
Map3k4	Map3k4	mitogen-activated protein kinase kinase kinase 4	26407	https://www.ncbi.nlm.nih.gov/gene/?term=26407
Ssb	Ssb	Sjogren syndrome antigen B	20823	https://www.ncbi.nlm.nih.gov/gene/?term=20823
Cav3	Cav3	caveolin 3	12391	https://www.ncbi.nlm.nih.gov/gene/?term=12391

Fam76b	Fam76b	family with sequence similarity 76, member B	72826	https://www.ncbi.nlm.nih.gov/gene/?term=72826
Stc1	Stc1	stanniocalcin 1	20855	https://www.ncbi.nlm.nih.gov/gene/?term=20855
Sp4	Sp4	trans-acting transcription factor 4	20688	https://www.ncbi.nlm.nih.gov/gene/?term=20688
Pcdh18	Pcdh18	protocadherin 18	73173	https://www.ncbi.nlm.nih.gov/gene/?term=73173
Als2	Als2	alsin Rho guanine nucleotide exchange factor	74018	https://www.ncbi.nlm.nih.gov/gene/?term=74018
Slc25a36	Slc25a36	solute carrier family 25, member 36	192287	https://www.ncbi.nlm.nih.gov/gene/?term=192287
Cdh11	Cdh11	cadherin 11	12552	https://www.ncbi.nlm.nih.gov/gene/?term=12552
Gbe1	Gbe1	glucan (1,4-alpha-), branching enzyme 1	74185	https://www.ncbi.nlm.nih.gov/gene/?term=74185
Ccng2	Ccng2	cyclin G2	12452	https://www.ncbi.nlm.nih.gov/gene/?term=12452

Biology Open • Supplementary information

Uevld	Uevld	UEV and lactate/malate dehyrogenase domains	54122	https://www.ncbi.nlm.nih.gov/gene/?term=54122
Sall1	Sall1	spalt like transcription factor 1	58198	https://www.ncbi.nlm.nih.gov/gene/?term=58198
Garnl3	Garnl3	GTPase activating RANGAP domain-like 3	99326	https://www.ncbi.nlm.nih.gov/gene/?term=99326
Golt1b	Golt1b	golgi transport 1B	66964	https://www.ncbi.nlm.nih.gov/gene/?term=66964
Uchl3	Uchl3	ubiquitin carboxyl-terminal esterase L3 (ubiquitin thiolesterase)	50933	https://www.ncbi.nlm.nih.gov/gene/?term=50933
Atp1b1	Atp1b1	ATPase, Na+/K+ transporting, beta 1 polypeptide	11931	https://www.ncbi.nlm.nih.gov/gene/?term=11931
Sec24a	Sec24a	Sec24 related gene family, member A (S. cerevisiae)	77371	https://www.ncbi.nlm.nih.gov/gene/?term=77371
Sh2b3	Sh2b3	SH2B adaptor protein 3	16923	https://www.ncbi.nlm.nih.gov/gene/?term=16923
Ppp2r2a	Ppp2r2a	protein phosphatase 2, regulatory subunit B, alpha	71978	https://www.ncbi.nlm.nih.gov/gene/?term=71978

Kcnmb2	Kcnmb2	potassium large conductance calcium-activated channel, subfamily M, beta member 2	72413	https://www.ncbi.nlm.nih.gov/gene/?term=72413
Ptprj	Ptprj	protein tyrosine phosphatase, receptor type, J	19271	https://www.ncbi.nlm.nih.gov/gene/?term=19271
Arid1a	Arid1a	AT rich interactive domain 1A (SWI-like)	93760	https://www.ncbi.nlm.nih.gov/gene/?term=93760
Uba2	Uba2	ubiquitin-like modifier activating enzyme 2	50995	https://www.ncbi.nlm.nih.gov/gene/?term=50995
Atp2b1	Atp2b1	ATPase, Ca++ transporting, plasma membrane 1	67972	https://www.ncbi.nlm.nih.gov/gene/?term=67972
Bmpr1b	Bmpr1b	bone morphogenetic protein receptor, type 1B	12167	https://www.ncbi.nlm.nih.gov/gene/?term=12167
Nlk	Nlk	nemo like kinase	18099	https://www.ncbi.nlm.nih.gov/gene/?term=18099
Herpud1	Herpud1	homocysteine-inducible, endoplasmic reticulum stress-inducible, ubiquitin-like domain member 1	64209	https://www.ncbi.nlm.nih.gov/gene/?term=64209
Sco1	Sco1	SCO1 cytochrome c oxidase assembly protein	52892	https://www.ncbi.nlm.nih.gov/gene/?term=52892

Biology Open • Supplementary information

Megf9	Megf9	multiple EGF-like-domains 9	230316	https://www.ncbi.nlm.nih.gov/gene/?term=230316
9930021J03Rik	9930021J03Rik	RIKEN cDNA 9930021J03 gene	240613	https://www.ncbi.nlm.nih.gov/gene/?term=240613
Ddx59	Ddx59	DEAD (Asp-Glu-Ala-Asp) box polypeptide 59	67997	https://www.ncbi.nlm.nih.gov/gene/?term=67997
Cmc1	Cmc1	COX assembly mitochondrial protein 1	67899	https://www.ncbi.nlm.nih.gov/gene/?term=67899
Msx1	Msx1	msh homeobox 1	17701	https://www.ncbi.nlm.nih.gov/gene/?term=17701
Kcnh8	Kcnh8	potassium voltage-gated channel, subfamily H (eag-related), member 8	211468	https://www.ncbi.nlm.nih.gov/gene/?term=211468
Spc25	Spc25	SPC25, NDC80 kinetochore complex component, homolog (S. cerevisiae)	66442	https://www.ncbi.nlm.nih.gov/gene/?term=66442
Rgs9	Rgs9	regulator of G-protein signaling 9	19739	https://www.ncbi.nlm.nih.gov/gene/?term=19739
Trex2	Trex2	three prime repair exonuclease 2	24102	https://www.ncbi.nlm.nih.gov/gene/?term=24102

Biology Open • Supplementary information

Cpsf6	Cpsf6	cleavage and polyadenylation specific factor 6	432508	https://www.ncbi.nlm.nih.gov/gene/?term=432508
Srsf1	Srsf1	serine/arginine-rich splicing factor 1	110809	https://www.ncbi.nlm.nih.gov/gene/?term=110809
Saa3	Saa3	serum amyloid A 3	20210	https://www.ncbi.nlm.nih.gov/gene/?term=20210
Mogs	Mogs	mannosyl-oligosaccharide glucosidase	57377	https://www.ncbi.nlm.nih.gov/gene/?term=57377
Aplp1	Aplp1	amyloid beta (A4) precursor-like protein 1	11803	https://www.ncbi.nlm.nih.gov/gene/?term=11803
Fzd8	Fzd8	frizzled class receptor 8	14370	https://www.ncbi.nlm.nih.gov/gene/?term=14370
Slc16a2	Slc16a2	solute carrier family 16 (monocarboxylic acid transporters), member 2	20502	https://www.ncbi.nlm.nih.gov/gene/?term=20502
Man1a2	Man1a2	mannosidase, alpha, class 1A, member 2	17156	https://www.ncbi.nlm.nih.gov/gene/?term=17156
Gphn	Gphn	gephyrin	268566	https://www.ncbi.nlm.nih.gov/gene/?term=268566

Biology Open • Supplementary information

Chsy1	Chsy1	chondroitin sulfate synthase 1	269941	https://www.ncbi.nlm.nih.gov/gene/?term=269941
Armcx1	Armcx1	armadillo repeat containing, X-linked 1	78248	https://www.ncbi.nlm.nih.gov/gene/?term=78248
Fgf18	Fgf18	fibroblast growth factor 18	14172	https://www.ncbi.nlm.nih.gov/gene/?term=14172
Fasl	Fasl	Fas ligand (TNF superfamily, member 6)	14103	https://www.ncbi.nlm.nih.gov/gene/?term=14103
Gramd3	Gramd3	GRAM domain containing 3	107022	https://www.ncbi.nlm.nih.gov/gene/?term=107022
Spry1	Spry1	sprouty RTK signaling antagonist 1	24063	https://www.ncbi.nlm.nih.gov/gene/?term=24063
Tipin	Tipin	timeless interacting protein	66131	https://www.ncbi.nlm.nih.gov/gene/?term=66131
Ccl20	Ccl20	chemokine (C-C motif) ligand 20	20297	https://www.ncbi.nlm.nih.gov/gene/?term=20297
Ifrd1	Ifrd1	interferon-related developmental regulator 1	15982	https://www.ncbi.nlm.nih.gov/gene/?term=15982

Biology Open • Supplementary information

Nkiras1	Nkiras1	NFKB inhibitor interacting Ras-like protein 1	69721	https://www.ncbi.nlm.nih.gov/gene/?term=69721
Tgfbi	Tgfbi	transforming growth factor, beta induced	21810	https://www.ncbi.nlm.nih.gov/gene/?term=21810
Ski	Ski	ski sarcoma viral oncogene homolog (avian)	20481	https://www.ncbi.nlm.nih.gov/gene/?term=20481
Ankrd49	Ankrd49	ankyrin repeat domain 49	56503	https://www.ncbi.nlm.nih.gov/gene/?term=56503
Caskin1	Caskin1	CASK interacting protein 1	268932	https://www.ncbi.nlm.nih.gov/gene/?term=268932
Il21	Il21	interleukin 21	60505	https://www.ncbi.nlm.nih.gov/gene/?term=60505
Zfp367	Zfp367	zinc finger protein 367	238673	https://www.ncbi.nlm.nih.gov/gene/?term=238673
Scml2	Scml2	Scm polycomb group protein like 2	107815	https://www.ncbi.nlm.nih.gov/gene/?term=107815
Ntf3	Ntf3	neurotrophin 3	18205	https://www.ncbi.nlm.nih.gov/gene/?term=18205

Biology Open • Supplementary information

Pnpt1	Pnpt1	polyribonucleotide nucleotidyltransferase 1	71701	https://www.ncbi.nlm.nih.gov/gene/?term=71701
Ccl1	Ccl1	chemokine (C-C motif) ligand 1	20290	https://www.ncbi.nlm.nih.gov/gene/?term=20290
Pcmtd2	Pcmtd2	protein-L-isoaspartate (D-aspartate) O-methyltransferase domain containing 2	245867	https://www.ncbi.nlm.nih.gov/gene/?term=245867
Spink14	Spink14	serine peptidase inhibitor, Kazal type 14	433178	https://www.ncbi.nlm.nih.gov/gene/?term=433178
Bcl11b	Bcl11b	B cell leukemia/lymphoma 11B	58208	https://www.ncbi.nlm.nih.gov/gene/?term=58208
Tmem236	Tmem236	transmembrane protein 236	625286	https://www.ncbi.nlm.nih.gov/gene/?term=625286
Bcl7a	Bcl7a	B cell CLL/lymphoma 7A	77045	https://www.ncbi.nlm.nih.gov/gene/?term=77045
Tsga10	Tsga10	testis specific 10	211484	https://www.ncbi.nlm.nih.gov/gene/?term=211484
Dusp8	Dusp8	dual specificity phosphatase 8	18218	https://www.ncbi.nlm.nih.gov/gene/?term=18218

Biology Open • Supplementary information

Armcx5	Armcx5	armadillo repeat containing, X-linked 5	494468	https://www.ncbi.nlm.nih.gov/gene/?term=494468
Nectin3	Nectin3	nectin cell adhesion molecule 3	58998	https://www.ncbi.nlm.nih.gov/gene/?term=58998
Fa2h	Fa2h	fatty acid 2-hydroxylase	338521	https://www.ncbi.nlm.nih.gov/gene/?term=338521
Pan3	Pan3	PAN3 poly(A) specific ribonuclease subunit	72587	https://www.ncbi.nlm.nih.gov/gene/?term=72587
Peli1	Peli1	pellino 1	67245	https://www.ncbi.nlm.nih.gov/gene/?term=67245
Pbrm1	Pbrm1	polybromo 1	66923	https://www.ncbi.nlm.nih.gov/gene/?term=66923
Dmrtc2	Dmrtc2	doublesex and mab-3 related transcription factor like family C2	71241	https://www.ncbi.nlm.nih.gov/gene/?term=71241
Armcx3	Armcx3	armadillo repeat containing, X-linked 3	71703	https://www.ncbi.nlm.nih.gov/gene/?term=71703
Ptbp3	Ptbp3	polypyrimidine tract binding protein 3	230257	https://www.ncbi.nlm.nih.gov/gene/?term=230257

Biology Open • Supplementary information

Grik2	Grik2	glutamate receptor, ionotropic, kainate 2 (beta 2)	14806	https://www.ncbi.nlm.nih.gov/gene/?term=14806
Kmt2c	Kmt2c	lysine (K)-specific methyltransferase 2C	231051	https://www.ncbi.nlm.nih.gov/gene/?term=231051
Pcdha12	Pcdha12	protocadherin alpha 12	192164	https://www.ncbi.nlm.nih.gov/gene/?term=192164
Gigyf1	Gigyf1	GRB10 interacting GYF protein 1	57330	https://www.ncbi.nlm.nih.gov/gene/?term=57330
Thrb	Thrb	thyroid hormone receptor beta	21834	https://www.ncbi.nlm.nih.gov/gene/?term=21834
Slc4a10	Slc4a10	solute carrier family 4, sodium bicarbonate cotransporter-like, member 10	94229	https://www.ncbi.nlm.nih.gov/gene/?term=94229
Pi4k2b	Pi4k2b	phosphatidylinositol 4-kinase type 2 beta	67073	https://www.ncbi.nlm.nih.gov/gene/?term=67073
Zfp97	Zfp97	zinc finger protein 97	22759	https://www.ncbi.nlm.nih.gov/gene/?term=22759
Tnfaip1	Tnfaip1	tumor necrosis factor, alpha-induced protein 1 (endothelial)	21927	https://www.ncbi.nlm.nih.gov/gene/?term=21927

Spire1	Spire1	spire type actin nucleation factor 1	68166	https://www.ncbi.nlm.nih.gov/gene/?term=68166
Cdyl	Cdyl	chromodomain protein, Y chromosome-like	12593	https://www.ncbi.nlm.nih.gov/gene/?term=12593
Carf	Carf	calcium response factor	241066	https://www.ncbi.nlm.nih.gov/gene/?term=241066
Sfmbt1	Sfmbt1	Scm-like with four mbt domains 1	54650	https://www.ncbi.nlm.nih.gov/gene/?term=54650
Dlg2	Dlg2	discs large MAGUK scaffold protein 2	23859	https://www.ncbi.nlm.nih.gov/gene/?term=23859
Rbm46	Rbm46	RNA binding motif protein 46	633285	https://www.ncbi.nlm.nih.gov/gene/?term=633285
Mtf2	Mtf2	metal response element binding transcription factor 2	17765	https://www.ncbi.nlm.nih.gov/gene/?term=17765
Ddx55	Ddx55	DEAD (Asp-Glu-Ala-Asp) box polypeptide 55	67848	https://www.ncbi.nlm.nih.gov/gene/?term=67848
Pcdha7	Pcdha7	protocadherin alpha 7	12939	https://www.ncbi.nlm.nih.gov/gene/?term=12939

Biology Open • Supplementary information

Gse1	Gse1	genetic suppressor element 1, coiled-coil protein	382034	https://www.ncbi.nlm.nih.gov/gene/?term=382034
Gpd1l	Gpd1l	glycerol-3-phosphate dehydrogenase 1-like	333433	https://www.ncbi.nlm.nih.gov/gene/?term=333433
Fign	Fign	fidgetin	60344	https://www.ncbi.nlm.nih.gov/gene/?term=60344
Ercc5	Ercc5	excision repair cross-complementing rodent repair deficiency, complementation group 5	22592	https://www.ncbi.nlm.nih.gov/gene/?term=22592
Usp33	Usp33	ubiquitin specific peptidase 33	170822	https://www.ncbi.nlm.nih.gov/gene/?term=170822
Gm14322	Gm14322	predicted gene 14322	626802	https://www.ncbi.nlm.nih.gov/gene/?term=626802
Nfat5	Nfat5	nuclear factor of activated T cells 5	54446	https://www.ncbi.nlm.nih.gov/gene/?term=54446
Ap1g1	Ap1g1	adaptor protein complex AP-1, gamma 1 subunit	11765	https://www.ncbi.nlm.nih.gov/gene/?term=11765
Zfp120	Zfp120	zinc finger protein 120	104348	https://www.ncbi.nlm.nih.gov/gene/?term=104348

Biology Open • Supplementary information

Trim2	Trim2	tripartite motif-containing 2	80890	https://www.ncbi.nlm.nih.gov/gene/?term=80890
Mtx3	Mtx3	metaxin 3	382793	https://www.ncbi.nlm.nih.gov/gene/?term=382793
Wdr82	Wdr82	WD repeat domain containing 82	77305	https://www.ncbi.nlm.nih.gov/gene/?term=77305
Kcnq5	Kcnq5	potassium voltage-gated channel, subfamily Q, member 5	226922	https://www.ncbi.nlm.nih.gov/gene/?term=226922
Ccdc122	Ccdc122	coiled-coil domain containing 122	108811	https://www.ncbi.nlm.nih.gov/gene/?term=108811
Ino80d	Ino80d	INO80 complex subunit D	227195	https://www.ncbi.nlm.nih.gov/gene/?term=227195
Anapc16	Anapc16	anaphase promoting complex subunit 16	52717	https://www.ncbi.nlm.nih.gov/gene/?term=52717
Bend3	Bend3	BEN domain containing 3	331623	https://www.ncbi.nlm.nih.gov/gene/?term=331623
Brd1	Brd1	bromodomain containing 1	223770	https://www.ncbi.nlm.nih.gov/gene/?term=223770

Biology Open • Supplementary information

Adam11	Adam11	a disintegrin and metallopeptidase domain 11	11488	https://www.ncbi.nlm.nih.gov/gene/?term=11488
Lmo1	Lmo1	LIM domain only 1	109594	https://www.ncbi.nlm.nih.gov/gene/?term=109594
Zfp976	Zfp976	zinc finger protein 976	208111	https://www.ncbi.nlm.nih.gov/gene/?term=208111
Man2a1	Man2a1	mannosidase 2, alpha 1	17158	https://www.ncbi.nlm.nih.gov/gene/?term=17158
Tada2b	Tada2b	transcriptional adaptor 2B	231151	https://www.ncbi.nlm.nih.gov/gene/?term=231151
Rorb	Rorb	RAR-related orphan receptor beta	225998	https://www.ncbi.nlm.nih.gov/gene/?term=225998
Patl1	Patl1	protein associated with topoisomerase II homolog 1 (yeast)	225929	https://www.ncbi.nlm.nih.gov/gene/?term=225929
Txndc12	Txndc12	thioredoxin domain containing 12 (endoplasmic reticulum)	66073	https://www.ncbi.nlm.nih.gov/gene/?term=66073
Palb2	Palb2	partner and localizer of BRCA2	233826	https://www.ncbi.nlm.nih.gov/gene/?term=233826

Rnf34	Rnf34	ring finger protein 34	80751	https://www.ncbi.nlm.nih.gov/gene/?term=80751
Larp4	Larp4	La ribonucleoprotein domain family, member 4	207214	https://www.ncbi.nlm.nih.gov/gene/?term=207214
Ubp1	Ubp1	upstream binding protein 1	22221	https://www.ncbi.nlm.nih.gov/gene/?term=22221
Gfpt1	Gfpt1	glutamine fructose-6-phosphate transaminase 1	14583	https://www.ncbi.nlm.nih.gov/gene/?term=14583
Lox	Lox	lysyl oxidase	16948	https://www.ncbi.nlm.nih.gov/gene/?term=16948
Tmem94	Tmem94	transmembrane protein 94	71947	https://www.ncbi.nlm.nih.gov/gene/?term=71947
Slc25a37	Slc25a37	solute carrier family 25, member 37	67712	https://www.ncbi.nlm.nih.gov/gene/?term=67712
Zdhhc7	Zdhhc7	zinc finger, DHHC domain containing 7	102193	https://www.ncbi.nlm.nih.gov/gene/?term=102193
Pcdha10	Pcdha10	protocadherin alpha 10	12943	https://www.ncbi.nlm.nih.gov/gene/?term=12943

Biology Open • Supplementary information

Pcdhac1	Pcdhac1	protocadherin alpha subfamily C, 1	353236	https://www.ncbi.nlm.nih.gov/gene/?term=353236
Trim71	Trim71	tripartite motif-containing 71	636931	https://www.ncbi.nlm.nih.gov/gene/?term=636931
Gabra1	Gabra1	gamma-aminobutyric acid (GABA) A receptor, subunit alpha 1	14394	https://www.ncbi.nlm.nih.gov/gene/?term=14394
Sema4g	Sema4g	sema domain, immunoglobulin domain (Ig), transmembrane domain (TM) and short cytoplasmic domain, (semaphorin) 4G	26456	https://www.ncbi.nlm.nih.gov/gene/?term=26456
Gm6712	Gm6712	predicted gene 6712	100862203	https://www.ncbi.nlm.nih.gov/gene/?term=100862
Klhl29	Klhl29	kelch-like 29	208439	https://www.ncbi.nlm.nih.gov/gene/?term=208439
Rassf1	Rassf1	Ras association (RalGDS/AF-6) domain family member 1	56289	https://www.ncbi.nlm.nih.gov/gene/?term=56289
Pcdha4	Pcdha4	protocadherin alpha 4	12936	https://www.ncbi.nlm.nih.gov/gene/?term=12936
Mb21d2	Mb21d2	Mab-21 domain containing 2	239796	https://www.ncbi.nlm.nih.gov/gene/?term=239796

Biology Open • Supplementary information

Acsl4	Acsl4	acyl-CoA synthetase long-chain family member 4	50790	https://www.ncbi.nlm.nih.gov/gene/?term=50790
Rps6ka3	Rps6ka3	ribosomal protein S6 kinase polypeptide 3	110651	https://www.ncbi.nlm.nih.gov/gene/?term=110651
Ss18l1	Ss18l1	SS18, nBAF chromatin remodeling complex subunit like 1	269397	https://www.ncbi.nlm.nih.gov/gene/?term=269397
Fam122b	Fam122b	family with sequence similarity 122, member B	78755	https://www.ncbi.nlm.nih.gov/gene/?term=78755
Zfp800	Zfp800	zinc finger protein 800	627049	https://www.ncbi.nlm.nih.gov/gene/?term=627049
Pcdha5	Pcdha5	protocadherin alpha 5	12941	https://www.ncbi.nlm.nih.gov/gene/?term=12941
Pcdha8	Pcdha8	protocadherin alpha 8	353235	https://www.ncbi.nlm.nih.gov/gene/?term=353235
Heca	Heca	hdc homolog, cell cycle regulator	380629	https://www.ncbi.nlm.nih.gov/gene/?term=380629
Ccp110	Ccp110	centriolar coiled coil protein 110	101565	https://www.ncbi.nlm.nih.gov/gene/?term=101565

Biology Open • Supplementary information

Zfp619	Zfp619	zinc finger protein 619	70227	https://www.ncbi.nlm.nih.gov/gene/?term=70227
Zfp951	Zfp951	zinc finger protein 951	626391	https://www.ncbi.nlm.nih.gov/gene/?term=626391
Klhl5	Klhl5	kelch-like 5	71778	https://www.ncbi.nlm.nih.gov/gene/?term=71778
Zic3	Zic3	zinc finger protein of the cerebellum 3	22773	https://www.ncbi.nlm.nih.gov/gene/?term=22773
Clec10a	Clec10a	C-type lectin domain family 10, member A	17312	https://www.ncbi.nlm.nih.gov/gene/?term=17312
Cyp2c39	Cyp2c39	cytochrome P450, family 2, subfamily c, polypeptide 39	13098	https://www.ncbi.nlm.nih.gov/gene/?term=13098
Entpd6	Entpd6	ectonucleoside triphosphate diphosphohydrolase 6	12497	https://www.ncbi.nlm.nih.gov/gene/?term=12497
Sec24c	Sec24c	Sec24 related gene family, member C (S. cerevisiae)	218811	https://www.ncbi.nlm.nih.gov/gene/?term=218811
Ppip5k2	Ppip5k2	diphosphoinositol pentakisphosphate kinase 2	227399	https://www.ncbi.nlm.nih.gov/gene/?term=227399

Biology Open • Supplementary information

Gata6	Gata6	GATA binding protein 6	14465	https://www.ncbi.nlm.nih.gov/gene/?term=14465
Syne1	Syne1	spectrin repeat containing, nuclear envelope 1	64009	https://www.ncbi.nlm.nih.gov/gene/?term=64009
Zfp973	Zfp973	zinc finger protein 973	545490	https://www.ncbi.nlm.nih.gov/gene/?term=545490
Pcdha6	Pcdha6	protocadherin alpha 6	12937	https://www.ncbi.nlm.nih.gov/gene/?term=12937
Zfp704	Zfp704	zinc finger protein 704	170753	https://www.ncbi.nlm.nih.gov/gene/?term=170753
P2ry10	P2ry10	purinergic receptor P2Y, G-protein coupled 10	78826	https://www.ncbi.nlm.nih.gov/gene/?term=78826
Specc1l	Specc1l	sperm antigen with calponin homology and coiled-coil domains 1-like	74392	https://www.ncbi.nlm.nih.gov/gene/?term=74392
Spry4	Spry4	sprouty RTK signaling antagonist 4	24066	https://www.ncbi.nlm.nih.gov/gene/?term=24066
Fam3c	Fam3c	family with sequence similarity 3, member C	27999	https://www.ncbi.nlm.nih.gov/gene/?term=27999

Biology Open • Supplementary information

Zfp780b	Zfp780b	zinc finger protein 780B	338354	https://www.ncbi.nlm.nih.gov/gene/?term=338354
Ddx3x	Ddx3x	DEAD/H (Asp-Glu-Ala-Asp/His) box polypeptide 3, X-linked	13205	https://www.ncbi.nlm.nih.gov/gene/?term=13205
Pcdha3	Pcdha3	protocadherin alpha 3	192163	https://www.ncbi.nlm.nih.gov/gene/?term=192163
Zfp317	Zfp317	zinc finger protein 317	244713	https://www.ncbi.nlm.nih.gov/gene/?term=244713
Mpp5	Mpp5	membrane protein, palmitoylated 5 (MAGUK p55 subfamily member 5)	56217	https://www.ncbi.nlm.nih.gov/gene/?term=56217
Il1a	Il1a	interleukin 1 alpha	16175	https://www.ncbi.nlm.nih.gov/gene/?term=16175
Gls	Gls	glutaminase	14660	https://www.ncbi.nlm.nih.gov/gene/?term=14660
Hoxa11	Hoxa11	homeobox A11	15396	https://www.ncbi.nlm.nih.gov/gene/?term=15396
Klf15	Klf15	Kruppel-like factor 15	66277	https://www.ncbi.nlm.nih.gov/gene/?term=66277

Biology Open • Supplementary information

Pcdha2	Pcdha2	protocadherin alpha 2	353234	https://www.ncbi.nlm.nih.gov/gene/?term=353234
Zfp14	Zfp14	zinc finger protein 14	243906	https://www.ncbi.nlm.nih.gov/gene/?term=243906
Tspan13	Tspan13	tetraspanin 13	66109	https://www.ncbi.nlm.nih.gov/gene/?term=66109
Ssx2ip	Ssx2ip	synovial sarcoma, X 2 interacting protein	99167	https://www.ncbi.nlm.nih.gov/gene/?term=99167
Cpne2	Cpne2	copine II	234577	https://www.ncbi.nlm.nih.gov/gene/?term=234577
Pcdhac2	Pcdhac2	protocadherin alpha subfamily C, 2	353237	https://www.ncbi.nlm.nih.gov/gene/?term=353237
Ubl3	Ubl3	ubiquitin-like 3	24109	https://www.ncbi.nlm.nih.gov/gene/?term=24109
Rbbp7	Rbbp7	retinoblastoma binding protein 7, chromatin remodeling factor	245688	https://www.ncbi.nlm.nih.gov/gene/?term=245688
Pou2f1	Pou2f1	POU domain, class 2, transcription factor 1	18986	https://www.ncbi.nlm.nih.gov/gene/?term=18986

Biology Open • Supplementary information

Dclk1	Dclk1	doublecortin-like kinase 1	13175	https://www.ncbi.nlm.nih.gov/gene/?term=13175
Tmem165	Tmem165	transmembrane protein 165	21982	https://www.ncbi.nlm.nih.gov/gene/?term=21982
Baz2b	Baz2b	bromodomain adjacent to zinc finger domain, 2B	407823	https://www.ncbi.nlm.nih.gov/gene/?term=407823
Lrba	Lrba	LPS-responsive beige-like anchor	80877	https://www.ncbi.nlm.nih.gov/gene/?term=80877
Zic2	Zic2	zinc finger protein of the cerebellum 2	22772	https://www.ncbi.nlm.nih.gov/gene/?term=22772
Pcdha9	Pcdha9	protocadherin alpha 9	192161	https://www.ncbi.nlm.nih.gov/gene/?term=192161
Tbc1d4	Tbc1d4	TBC1 domain family, member 4	210789	https://www.ncbi.nlm.nih.gov/gene/?term=210789
Mapk1	Mapk1	mitogen-activated protein kinase 1	26413	https://www.ncbi.nlm.nih.gov/gene/?term=26413
Prox1	Prox1	prospero homeobox 1	19130	https://www.ncbi.nlm.nih.gov/gene/?term=19130

Biology Open • Supplementary information

Zfp966	Zfp966	zinc finger protein 966	667962	https://www.ncbi.nlm.nih.gov/gene/?term=667962
Ttc39b	Ttc39b	tetratricopeptide repeat domain 39B	69863	https://www.ncbi.nlm.nih.gov/gene/?term=69863
B4galt1	B4galt1	UDP-Gal:betaGlcNAc beta 1,4-galactosyltransferase, polypeptide 1	14595	https://www.ncbi.nlm.nih.gov/gene/?term=14595
Zfp936	Zfp936	zinc finger protein 936	668620	https://www.ncbi.nlm.nih.gov/gene/?term=668620
Ap1s3	Ap1s3	adaptor-related protein complex AP-1, sigma 3	252903	https://www.ncbi.nlm.nih.gov/gene/?term=252903
Lhx9	Lhx9	LIM homeobox protein 9	16876	https://www.ncbi.nlm.nih.gov/gene/?term=16876
Plcl2	Plcl2	phospholipase C-like 2	224860	https://www.ncbi.nlm.nih.gov/gene/?term=224860
Zfp960	Zfp960	zinc finger protein 960	449000	https://www.ncbi.nlm.nih.gov/gene/?term=449000
Dmxl2	Dmxl2	Dmx-like 2	235380	https://www.ncbi.nlm.nih.gov/gene/?term=235380

Biology Open • Supplementary information

Epha4	Epha4	Eph receptor A4	13838	https://www.ncbi.nlm.nih.gov/gene/?term=13838
Osbpl3	Osbpl3	oxysterol binding protein-like 3	71720	https://www.ncbi.nlm.nih.gov/gene/?term=71720
Zfp971	Zfp971	zinc finger protein 971	626848	https://www.ncbi.nlm.nih.gov/gene/?term=626848
Ythdc2	Ythdc2	YTH domain containing 2	240255	https://www.ncbi.nlm.nih.gov/gene/?term=240255
Sim1	Sim1	single-minded family bHLH transcription factor 1	20464	https://www.ncbi.nlm.nih.gov/gene/?term=20464
Carm1	Carm1	coactivator-associated arginine methyltransferase 1	59035	https://www.ncbi.nlm.nih.gov/gene/?term=59035
Abtb2	Abtb2	ankyrin repeat and BTB (POZ) domain containing 2	99382	https://www.ncbi.nlm.nih.gov/gene/?term=99382
Gskip	Gskip	GSK3B interacting protein	66787	https://www.ncbi.nlm.nih.gov/gene/?term=66787
2010315B03Rik	2010315B03Rik	RIKEN cDNA 2010315B03 gene	630836	https://www.ncbi.nlm.nih.gov/gene/?term=630836

Biology Open • Supplementary information

Spice1	Spice1	spindle and centriole associated protein 1	212514	https://www.ncbi.nlm.nih.gov/gene/?term=212514
Hey2	Hey2	hairy/enhancer-of-split related with YRPW motif 2	15214	https://www.ncbi.nlm.nih.gov/gene/?term=15214
Lclat1	Lclat1	lysocardiolipin acyltransferase 1	225010	https://www.ncbi.nlm.nih.gov/gene/?term=225010
Ube2b	Ube2b	ubiquitin-conjugating enzyme E2B	22210	https://www.ncbi.nlm.nih.gov/gene/?term=22210
Hoxc8	Hoxc8	homeobox C8	15426	https://www.ncbi.nlm.nih.gov/gene/?term=15426
Crim1	Crim1	cysteine rich transmembrane BMP regulator 1 (chordin like)	50766	https://www.ncbi.nlm.nih.gov/gene/?term=50766
Pi15	Pi15	peptidase inhibitor 15	94227	https://www.ncbi.nlm.nih.gov/gene/?term=94227
Trak1	Trak1	trafficking protein, kinesin binding 1	67095	https://www.ncbi.nlm.nih.gov/gene/?term=67095
Pcdha1	Pcdha1	protocadherin alpha 1	116731	https://www.ncbi.nlm.nih.gov/gene/?term=116731

Biology Open • Supplementary information

Lrrc32	Lrrc32	leucine rich repeat containing 32	434215	https://www.ncbi.nlm.nih.gov/gene/?term=434215
Esm1	Esm1	endothelial cell-specific molecule 1	71690	https://www.ncbi.nlm.nih.gov/gene/?term=71690
Slc35f3	Slc35f3	solute carrier family 35, member F3	210027	https://www.ncbi.nlm.nih.gov/gene/?term=210027
1700066M21Rik	1700066M21Rik	RIKEN cDNA 1700066M21 gene	73467	https://www.ncbi.nlm.nih.gov/gene/?term=73467
Sowaha	Sowaha	sosondowah ankyrin repeat domain family member A	237761	https://www.ncbi.nlm.nih.gov/gene/?term=237761
Phtf2	Phtf2	putative homeodomain transcription factor 2	68770	https://www.ncbi.nlm.nih.gov/gene/?term=68770
Sik3	Sik3	SIK family kinase 3	70661	https://www.ncbi.nlm.nih.gov/gene/?term=70661
Zfp975	Zfp975	zinc finger protein 975	434179	https://www.ncbi.nlm.nih.gov/gene/?term=434179
Zfp965	Zfp965	zinc finger protein 965	100503949	https://www.ncbi.nlm.nih.gov/gene/?term=100503[illegible]

Cbx7	Cbx7	chromobox 7	52609	https://www.ncbi.nlm.nih.gov/gene/?term=52609
Ncoa2	Ncoa2	nuclear receptor coactivator 2	17978	https://www.ncbi.nlm.nih.gov/gene/?term=17978
Cpd	Cpd	carboxypeptidase D	12874	https://www.ncbi.nlm.nih.gov/gene/?term=12874
Nwd2	Nwd2	NACHT and WD repeat domain containing 2	319807	https://www.ncbi.nlm.nih.gov/gene/?term=319807
D430041D05Rik	D430041D05Rik	RIKEN cDNA D430041D05 gene	241589	https://www.ncbi.nlm.nih.gov/gene/?term=241589
Pcdha11	Pcdha11	protocadherin alpha 11	12942	https://www.ncbi.nlm.nih.gov/gene/?term=12942
Ap4e1	Ap4e1	adaptor-related protein complex AP-4, epsilon 1	108011	https://www.ncbi.nlm.nih.gov/gene/?term=108011
Gm14296	Gm14296	predicted gene 14296	102639598	https://www.ncbi.nlm.nih.gov/gene/?term=102639[illegible]
Fmr1	Fmr1	fragile X mental retardation 1	14265	https://www.ncbi.nlm.nih.gov/gene/?term=14265

Biology Open • Supplementary information

App	App	amyloid beta (A4) precursor protein	11820	https://www.ncbi.nlm.nih.gov/gene/?term=11820
Mdfi	Mdfi	MyoD family inhibitor	17240	https://www.ncbi.nlm.nih.gov/gene/?term=17240
Aspn	Aspn	asporin	66695	https://www.ncbi.nlm.nih.gov/gene/?term=66695
Elk3	Elk3	ELK3, member of ETS oncogene family	13713	https://www.ncbi.nlm.nih.gov/gene/?term=13713
Glrb	Glrb	glycine receptor, beta subunit	14658	https://www.ncbi.nlm.nih.gov/gene/?term=14658
Trub1	Trub1	TruB pseudouridine (psi) synthase family member 1	72133	https://www.ncbi.nlm.nih.gov/gene/?term=72133
Gm15093	Gm15093	predicted gene 15093	100039890	https://www.ncbi.nlm.nih.gov/gene/?term=100039
Panx2	Panx2	pannexin 2	406218	https://www.ncbi.nlm.nih.gov/gene/?term=406218
Aktip	Aktip	thymoma viral proto-oncogene 1 interacting protein	14339	https://www.ncbi.nlm.nih.gov/gene/?term=14339

Ppp1r14c	Ppp1r14c	protein phosphatase 1, regulatory inhibitor subunit 14C	76142	https://www.ncbi.nlm.nih.gov/gene/?term=76142
Morc3	Morc3	microrchidia 3	338467	https://www.ncbi.nlm.nih.gov/gene/?term=338467
Trim68	Trim68	tripartite motif-containing 68	101700	https://www.ncbi.nlm.nih.gov/gene/?term=101700
Cftr	Cftr	cystic fibrosis transmembrane conductance regulator	12638	https://www.ncbi.nlm.nih.gov/gene/?term=12638
Cxxc4	Cxxc4	CXXC finger 4	319478	https://www.ncbi.nlm.nih.gov/gene/?term=319478
Zc3h11a	Zc3h11a	zinc finger CCCH type containing 11A	70579	https://www.ncbi.nlm.nih.gov/gene/?term=70579
Manea	Manea	mannosidase, endo-alpha	242362	https://www.ncbi.nlm.nih.gov/gene/?term=242362
Gm15114	Gm15114	predicted gene 15114	100034729	https://www.ncbi.nlm.nih.gov/gene/?term=100034
Zfhx3	Zfhx3	zinc finger homeobox 3	11906	https://www.ncbi.nlm.nih.gov/gene/?term=11906

Ppfia1	Ppfia1	protein tyrosine phosphatase, receptor type, f polypeptide (PTPRF), interacting protein (liprin), alpha 1	233977	https://www.ncbi.nlm.nih.gov/gene/?term=233977
AU015228	AU015228	expressed sequence AU015228	99169	https://www.ncbi.nlm.nih.gov/gene/?term=99169
Gm15127	Gm15127	predicted gene 15127	434866	https://www.ncbi.nlm.nih.gov/gene/?term=434866
Slc25a3	Slc25a3	solute carrier family 25 (mitochondrial carrier, phosphate carrier), member 3	18674	https://www.ncbi.nlm.nih.gov/gene/?term=18674
Paxbp1	Paxbp1	PAX3 and PAX7 binding protein 1	67367	https://www.ncbi.nlm.nih.gov/gene/?term=67367
Purb	Purb	purine rich element binding protein B	19291	https://www.ncbi.nlm.nih.gov/gene/?term=19291
Gm15085	Gm15085	predicted gene 15085	100039934	https://www.ncbi.nlm.nih.gov/gene/?term=100039
Bzw1	Bzw1	basic leucine zipper and W2 domains 1	66882	https://www.ncbi.nlm.nih.gov/gene/?term=66882
Il13	Il13	interleukin 13	16163	https://www.ncbi.nlm.nih.gov/gene/?term=16163

Biology Open • Supplementary information

Get4	Get4	golgi to ER traffic protein 4	67604	https://www.ncbi.nlm.nih.gov/gene/?term=67604
Tbl1xr1	Tbl1xr1	transducin (beta)-like 1X-linked receptor 1	81004	https://www.ncbi.nlm.nih.gov/gene/?term=81004
Rai1	Rai1	retinoic acid induced 1	19377	https://www.ncbi.nlm.nih.gov/gene/?term=19377
Gja1	Gja1	gap junction protein, alpha 1	14609	https://www.ncbi.nlm.nih.gov/gene/?term=14609
Ppp1r13b	Ppp1r13b	protein phosphatase 1, regulatory subunit 13B	21981	https://www.ncbi.nlm.nih.gov/gene/?term=21981
Phlda1	Phlda1	pleckstrin homology like domain, family A, member 1	21664	https://www.ncbi.nlm.nih.gov/gene/?term=21664
Pcdh7	Pcdh7	protocadherin 7	54216	https://www.ncbi.nlm.nih.gov/gene/?term=54216
Lrrc39	Lrrc39	leucine rich repeat containing 39	109245	https://www.ncbi.nlm.nih.gov/gene/?term=109245
Tmtc3	Tmtc3	transmembrane and tetratricopeptide repeat containing 3	237500	https://www.ncbi.nlm.nih.gov/gene/?term=237500

Biology Open • Supplementary information

Lin54	Lin54	lin-54 homolog (C. elegans)	231506	https://www.ncbi.nlm.nih.gov/gene/?term=231506
Fam204a	Fam204a	family with sequence similarity 204, member A	76539	https://www.ncbi.nlm.nih.gov/gene/?term=76539
Kpna3	Kpna3	karyopherin (importin) alpha 3	16648	https://www.ncbi.nlm.nih.gov/gene/?term=16648
Tle1	Tle1	transducin-like enhancer of split 1	21885	https://www.ncbi.nlm.nih.gov/gene/?term=21885
Zfhx4	Zfhx4	zinc finger homeodomain 4	80892	https://www.ncbi.nlm.nih.gov/gene/?term=80892
Arg2	Arg2	arginase type II	11847	https://www.ncbi.nlm.nih.gov/gene/?term=11847
Gm15091	Gm15091	predicted gene 15091	382244	https://www.ncbi.nlm.nih.gov/gene/?term=382244
Gclc	Gclc	glutamate-cysteine ligase, catalytic subunit	14629	https://www.ncbi.nlm.nih.gov/gene/?term=14629
Thsd7a	Thsd7a	thrombospondin, type I, domain containing 7A	330267	https://www.ncbi.nlm.nih.gov/gene/?term=330267

Biology Open • Supplementary information

Crebzf	Crebzf	CREB/ATF bZIP transcription factor	233490	https://www.ncbi.nlm.nih.gov/gene/?term=233490
Gm15080	Gm15080	predicted gene 15080	666184	https://www.ncbi.nlm.nih.gov/gene/?term=666184
Ott	Ott	ovary testis transcribed	18422	https://www.ncbi.nlm.nih.gov/gene/?term=18422
Rnf111	Rnf111	ring finger 111	93836	https://www.ncbi.nlm.nih.gov/gene/?term=93836
Hapln1	Hapln1	hyaluronan and proteoglycan link protein 1	12950	https://www.ncbi.nlm.nih.gov/gene/?term=12950
Atp6v1a	Atp6v1a	ATPase, H+ transporting, lysosomal V1 subunit A	11964	https://www.ncbi.nlm.nih.gov/gene/?term=11964
Lrat	Lrat	lecithin-retinol acyltransferase (phosphatidylcholine-retinol-O-acyltransferase)	79235	https://www.ncbi.nlm.nih.gov/gene/?term=79235
Gnpda2	Gnpda2	glucosamine-6-phosphate deaminase 2	67980	https://www.ncbi.nlm.nih.gov/gene/?term=67980
Zfp804a	Zfp804a	zinc finger protein 804A	241514	https://www.ncbi.nlm.nih.gov/gene/?term=241514

Biology Open • Supplementary information

Scamp1	Scamp1	secretory carrier membrane protein 1	107767	https://www.ncbi.nlm.nih.gov/gene/?term=107767
E2f8	E2f8	E2F transcription factor 8	108961	https://www.ncbi.nlm.nih.gov/gene/?term=108961
Gm15097	Gm15097	predicted gene 15097	434869	https://www.ncbi.nlm.nih.gov/gene/?term=434869
Gm15107	Gm15107	predicted gene 15107	434864	https://www.ncbi.nlm.nih.gov/gene/?term=434864
Trappc8	Trappc8	trafficking protein particle complex 8	75964	https://www.ncbi.nlm.nih.gov/gene/?term=75964
Naa15	Naa15	N(alpha)-acetyltransferase 15, NatA auxiliary subunit	74838	https://www.ncbi.nlm.nih.gov/gene/?term=74838
Emp2	Emp2	epithelial membrane protein 2	13731	https://www.ncbi.nlm.nih.gov/gene/?term=13731
Amn1	Amn1	antagonist of mitotic exit network 1	232566	https://www.ncbi.nlm.nih.gov/gene/?term=232566
Nfxl1	Nfxl1	nuclear transcription factor, X-box binding-like 1	100978	https://www.ncbi.nlm.nih.gov/gene/?term=100978

Biology Open • Supplementary information

Sh2d3c	Sh2d3c	SH2 domain containing 3C	27387	https://www.ncbi.nlm.nih.gov/gene/?term=27387
Nsd1	Nsd1	nuclear receptor-binding SET-domain protein 1	18193	https://www.ncbi.nlm.nih.gov/gene/?term=18193
Eif5a	Eif5a	eukaryotic translation initiation factor 5A	276770	https://www.ncbi.nlm.nih.gov/gene/?term=276770
Fstl5	Fstl5	follistatin-like 5	213262	https://www.ncbi.nlm.nih.gov/gene/?term=213262
Hs3st3b1	Hs3st3b1	heparan sulfate (glucosamine) 3-O-sulfotransferase 3B1	54710	https://www.ncbi.nlm.nih.gov/gene/?term=54710
Asb7	Asb7	ankyrin repeat and SOCS box-containing 7	117589	https://www.ncbi.nlm.nih.gov/gene/?term=117589
Gpr141b	Gpr141b	G protein-coupled receptor 141B	319293	https://www.ncbi.nlm.nih.gov/gene/?term=319293
Ctnnb1	Ctnnb1	catenin (cadherin associated protein), beta 1	12387	https://www.ncbi.nlm.nih.gov/gene/?term=12387
Azin1	Azin1	antizyme inhibitor 1	54375	https://www.ncbi.nlm.nih.gov/gene/?term=54375

Biology Open • Supplementary information

Tmed7	Tmed7	transmembrane p24 trafficking protein 7	66676	https://www.ncbi.nlm.nih.gov/gene/?term=66676
Prkar2b	Prkar2b	protein kinase, cAMP dependent regulatory, type II beta	19088	https://www.ncbi.nlm.nih.gov/gene/?term=19088
Spock1	Spock1	sparc/osteonectin, cwcv and kazal-like domains proteoglycan 1	20745	https://www.ncbi.nlm.nih.gov/gene/?term=20745
Gid8	Gid8	GID complex subunit 8	76425	https://www.ncbi.nlm.nih.gov/gene/?term=76425
Trappc2	Trappc2	trafficking protein particle complex 2	66226	https://www.ncbi.nlm.nih.gov/gene/?term=66226
Mdga2	Mdga2	MAM domain containing glycosylphosphatidylinositol anchor 2	320772	https://www.ncbi.nlm.nih.gov/gene/?term=320772
Tmem255a	Tmem255a	transmembrane protein 255A	245386	https://www.ncbi.nlm.nih.gov/gene/?term=245386
Nfs1	Nfs1	nitrogen fixation gene 1 (S. cerevisiae)	18041	https://www.ncbi.nlm.nih.gov/gene/?term=18041
Kif20b	Kif20b	kinesin family member 20B	240641	https://www.ncbi.nlm.nih.gov/gene/?term=240641

Biology Open • Supplementary information

Cep350	Cep350	centrosomal protein 350	74081	https://www.ncbi.nlm.nih.gov/gene/?term=74081
Cdk13	Cdk13	cyclin-dependent kinase 13	69562	https://www.ncbi.nlm.nih.gov/gene/?term=69562
Sult1d1	Sult1d1	sulfotransferase family 1D, member 1	53315	https://www.ncbi.nlm.nih.gov/gene/?term=53315
Utp11	Utp11	UTP11 small subunit processome component	67205	https://www.ncbi.nlm.nih.gov/gene/?term=67205
Siah1a	Siah1a	siah E3 ubiquitin protein ligase 1A	20437	https://www.ncbi.nlm.nih.gov/gene/?term=20437
Usp38	Usp38	ubiquitin specific peptidase 38	74841	https://www.ncbi.nlm.nih.gov/gene/?term=74841
Sfrp2	Sfrp2	secreted frizzled-related protein 2	20319	https://www.ncbi.nlm.nih.gov/gene/?term=20319
Bnc2	Bnc2	basonuclin 2	242509	https://www.ncbi.nlm.nih.gov/gene/?term=242509
Klhl24	Klhl24	kelch-like 24	75785	https://www.ncbi.nlm.nih.gov/gene/?term=75785

Zbtb44	Zbtb44	zinc finger and BTB domain containing 44	235132	https://www.ncbi.nlm.nih.gov/gene/?term=235132
Zmynd8	Zmynd8	zinc finger, MYND-type containing 8	228880	https://www.ncbi.nlm.nih.gov/gene/?term=228880
Schip1	Schip1	schwannomin interacting protein 1	30953	https://www.ncbi.nlm.nih.gov/gene/?term=30953
Nfib	Nfib	nuclear factor I/B	18028	https://www.ncbi.nlm.nih.gov/gene/?term=18028
Vcan	Vcan	versican	13003	https://www.ncbi.nlm.nih.gov/gene/?term=13003
Rngtt	Rngtt	RNA guanylyltransferase and 5'-phosphatase	24018	https://www.ncbi.nlm.nih.gov/gene/?term=24018
Bcar3	Bcar3	breast cancer anti-estrogen resistance 3	29815	https://www.ncbi.nlm.nih.gov/gene/?term=29815
Rtf1	Rtf1	RTF1, Paf1/RNA polymerase II complex component	76246	https://www.ncbi.nlm.nih.gov/gene/?term=76246
Chn2	Chn2	chimerin 2	69993	https://www.ncbi.nlm.nih.gov/gene/?term=69993

Biology Open • Supplementary information

Lyplal1	Lyplal1	lysophospholipase-like 1	226791	https://www.ncbi.nlm.nih.gov/gene/?term=226791
Pphln1	Pphln1	periphilin 1	223828	https://www.ncbi.nlm.nih.gov/gene/?term=223828
Gng7	Gng7	guanine nucleotide binding protein (G protein), gamma 7	14708	https://www.ncbi.nlm.nih.gov/gene/?term=14708
Cyp3a25	Cyp3a25	cytochrome P450, family 3, subfamily a, polypeptide 25	56388	https://www.ncbi.nlm.nih.gov/gene/?term=56388
Snx2	Snx2	sorting nexin 2	67804	https://www.ncbi.nlm.nih.gov/gene/?term=67804
Kif26a	Kif26a	kinesin family member 26A	668303	https://www.ncbi.nlm.nih.gov/gene/?term=668303
Cpne8	Cpne8	copine VIII	66871	https://www.ncbi.nlm.nih.gov/gene/?term=66871
Hdac1	Hdac1	histone deacetylase 1	433759	https://www.ncbi.nlm.nih.gov/gene/?term=433759
Zfp948	Zfp948	zinc finger protein 948	381066	https://www.ncbi.nlm.nih.gov/gene/?term=381066

Biology Open • Supplementary information

Gbp7	Gbp7	guanylate binding protein 7	229900	https://www.ncbi.nlm.nih.gov/gene/?term=229900
Strbp	Strbp	spermatid perinuclear RNA binding protein	20744	https://www.ncbi.nlm.nih.gov/gene/?term=20744
Ei24	Ei24	etoposide induced 2.4 mRNA	13663	https://www.ncbi.nlm.nih.gov/gene/?term=13663
Mab21l1	Mab21l1	mab-21-like 1 (C. elegans)	17116	https://www.ncbi.nlm.nih.gov/gene/?term=17116
Drd1	Drd1	dopamine receptor D1	13488	https://www.ncbi.nlm.nih.gov/gene/?term=13488
Cd163	Cd163	CD163 antigen	93671	https://www.ncbi.nlm.nih.gov/gene/?term=93671
Snap91	Snap91	synaptosomal-associated protein 91	20616	https://www.ncbi.nlm.nih.gov/gene/?term=20616
Sgip1	Sgip1	SH3-domain GRB2-like (endophilin) interacting protein 1	73094	https://www.ncbi.nlm.nih.gov/gene/?term=73094
Sox21	Sox21	SRY (sex determining region Y)-box 21	223227	https://www.ncbi.nlm.nih.gov/gene/?term=223227

Biology Open • Supplementary information

Nhsl2	Nhsl2	NHS-like 2	100042480	https://www.ncbi.nlm.nih.gov/gene/?term=100042[illegible]
Kbtbd7	Kbtbd7	kelch repeat and BTB (POZ) domain containing 7	211255	https://www.ncbi.nlm.nih.gov/gene/?term=211255
Hmgn5	Hmgn5	high-mobility group nucleosome binding domain 5	50887	https://www.ncbi.nlm.nih.gov/gene/?term=50887
Dnm1l	Dnm1l	dynamin 1-like	74006	https://www.ncbi.nlm.nih.gov/gene/?term=74006
Tial1	Tial1	Tia1 cytotoxic granule-associated RNA binding protein-like 1	21843	https://www.ncbi.nlm.nih.gov/gene/?term=21843
Uap1	Uap1	UDP-N-acetylglucosamine pyrophosphorylase 1	107652	https://www.ncbi.nlm.nih.gov/gene/?term=107652
Calcr	Calcr	calcitonin receptor	12311	https://www.ncbi.nlm.nih.gov/gene/?term=12311
Dennd6a	Dennd6a	DENN/MADD domain containing 6A	211922	https://www.ncbi.nlm.nih.gov/gene/?term=211922
Dixdc1	Dixdc1	DIX domain containing 1	330938	https://www.ncbi.nlm.nih.gov/gene/?term=330938

Biology Open • Supplementary information

Epcam	Epcam	epithelial cell adhesion molecule	17075	https://www.ncbi.nlm.nih.gov/gene/?term=17075
Zbtb43	Zbtb43	zinc finger and BTB domain containing 43	71834	https://www.ncbi.nlm.nih.gov/gene/?term=71834
Ppfibp1	Ppfibp1	PTPRF interacting protein, binding protein 1 (liprin beta 1)	67533	https://www.ncbi.nlm.nih.gov/gene/?term=67533
Scoc	Scoc	short coiled-coil protein	56367	https://www.ncbi.nlm.nih.gov/gene/?term=56367
Ctbp2	Ctbp2	C-terminal binding protein 2	13017	https://www.ncbi.nlm.nih.gov/gene/?term=13017
Hnrnpa3	Hnrnpa3	heterogeneous nuclear ribonucleoprotein A3	229279	https://www.ncbi.nlm.nih.gov/gene/?term=229279
Sar1a	Sar1a	secretion associated Ras related GTPase 1A	20224	https://www.ncbi.nlm.nih.gov/gene/?term=20224
Atp13a3	Atp13a3	ATPase type 13A3	224088	https://www.ncbi.nlm.nih.gov/gene/?term=224088
Htr2c	Htr2c	5-hydroxytryptamine (serotonin) receptor 2C	15560	https://www.ncbi.nlm.nih.gov/gene/?term=15560

Cwc22	Cwc22	CWC22 spliceosome-associated protein	80744	https://www.ncbi.nlm.nih.gov/gene/?term=80744
Metap1	Metap1	methionyl aminopeptidase 1	75624	https://www.ncbi.nlm.nih.gov/gene/?term=75624
Mob3c	Mob3c	MOB kinase activator 3C	100465	https://www.ncbi.nlm.nih.gov/gene/?term=100465
Bclaf3	Bclaf3	Bclaf1 and Thrap3 family member 3	382252	https://www.ncbi.nlm.nih.gov/gene/?term=382252
Stk26	Stk26	serine/threonine kinase 26	70415	https://www.ncbi.nlm.nih.gov/gene/?term=70415
2210010C04Rik	2210010C04Rik	RIKEN cDNA 2210010C04 gene	67373	https://www.ncbi.nlm.nih.gov/gene/?term=67373
Cacna1g	Cacna1g	calcium channel, voltage-dependent, T type, alpha 1G subunit	12291	https://www.ncbi.nlm.nih.gov/gene/?term=12291
Ptprm	Ptprm	protein tyrosine phosphatase, receptor type, M	19274	https://www.ncbi.nlm.nih.gov/gene/?term=19274
Tspan7	Tspan7	tetraspanin 7	21912	https://www.ncbi.nlm.nih.gov/gene/?term=21912

Biology Open • Supplementary information

Eml4	Eml4	echinoderm microtubule associated protein like 4	78798	https://www.ncbi.nlm.nih.gov/gene/?term=78798
Rock1	Rock1	Rho-associated coiled-coil containing protein kinase 1	19877	https://www.ncbi.nlm.nih.gov/gene/?term=19877
Bmt2	Bmt2	base methyltransferase of 25S rRNA 2	101148	https://www.ncbi.nlm.nih.gov/gene/?term=101148
Ptgs2	Ptgs2	prostaglandin-endoperoxide synthase 2	19225	https://www.ncbi.nlm.nih.gov/gene/?term=19225
Arid4b	Arid4b	AT rich interactive domain 4B (RBP1-like)	94246	https://www.ncbi.nlm.nih.gov/gene/?term=94246
Slc30a8	Slc30a8	solute carrier family 30 (zinc transporter), member 8	239436	https://www.ncbi.nlm.nih.gov/gene/?term=239436
Ppp3r1	Ppp3r1	protein phosphatase 3, regulatory subunit B, alpha isoform (calcineurin B, type I)	19058	https://www.ncbi.nlm.nih.gov/gene/?term=19058
Psd3	Psd3	pleckstrin and Sec7 domain containing 3	234353	https://www.ncbi.nlm.nih.gov/gene/?term=234353
Ltn1	Ltn1	listerin E3 ubiquitin protein ligase 1	78913	https://www.ncbi.nlm.nih.gov/gene/?term=78913

Biology Open • Supplementary information

Hnrnpk	Hnrnpk	heterogeneous nuclear ribonucleoprotein K	15387	https://www.ncbi.nlm.nih.gov/gene/?term=15387
Taf3	Taf3	TATA-box binding protein associated factor 3	209361	https://www.ncbi.nlm.nih.gov/gene/?term=209361
Dnaja2	Dnaja2	DnaJ heat shock protein family (Hsp40) member A2	56445	https://www.ncbi.nlm.nih.gov/gene/?term=56445
Cebpg	Cebpg	CCAAT/enhancer binding protein (C/EBP), gamma	12611	https://www.ncbi.nlm.nih.gov/gene/?term=12611
Gimap4	Gimap4	GTPase, IMAP family member 4	107526	https://www.ncbi.nlm.nih.gov/gene/?term=107526
Eif4h	Eif4h	eukaryotic translation initiation factor 4H	22384	https://www.ncbi.nlm.nih.gov/gene/?term=22384
Galntl6	Galntl6	UDP-N-acetyl-alpha-D-galactosamine:polypeptide N-acetylgalactosaminyltransferase-like 6	270049	https://www.ncbi.nlm.nih.gov/gene/?term=270049
Sema5a	Sema5a	sema domain, seven thrombospondin repeats (type 1 and type 1-like), transmembrane domain (TM) and short cytoplasmic domain, (semaphorin) 5A	20356	https://www.ncbi.nlm.nih.gov/gene/?term=20356

Biology Open • Supplementary information

Btrc	Btrc	beta-transducin repeat containing protein	12234	https://www.ncbi.nlm.nih.gov/gene/?term=12234
Mesp1	Mesp1	mesoderm posterior 1	17292	https://www.ncbi.nlm.nih.gov/gene/?term=17292
Samd4	Samd4	sterile alpha motif domain containing 4	74480	https://www.ncbi.nlm.nih.gov/gene/?term=74480
Spred1	Spred1	sprouty protein with EVH-1 domain 1, related sequence	114715	https://www.ncbi.nlm.nih.gov/gene/?term=114715
1700018B08Rik	1700018B08Rik	RIKEN cDNA 1700018B08 gene	76405	https://www.ncbi.nlm.nih.gov/gene/?term=76405
Slc22a3	Slc22a3	solute carrier family 22 (organic cation transporter), member 3	20519	https://www.ncbi.nlm.nih.gov/gene/?term=20519
Hnrnpc	Hnrnpc	heterogeneous nuclear ribonucleoprotein C	15381	https://www.ncbi.nlm.nih.gov/gene/?term=15381
Nr2e1	Nr2e1	nuclear receptor subfamily 2, group E, member 1	21907	https://www.ncbi.nlm.nih.gov/gene/?term=21907
Mtdh	Mtdh	metadherin	67154	https://www.ncbi.nlm.nih.gov/gene/?term=67154

Biology Open • Supplementary information

U2surp	U2surp	U2 snRNP-associated SURP domain containing	67958	https://www.ncbi.nlm.nih.gov/gene/?term=67958
Ttc39c	Ttc39c	tetratricopeptide repeat domain 39C	72747	https://www.ncbi.nlm.nih.gov/gene/?term=72747
Pdgfra	Pdgfra	platelet derived growth factor receptor, alpha polypeptide	18595	https://www.ncbi.nlm.nih.gov/gene/?term=18595
Zbtb6	Zbtb6	zinc finger and BTB domain containing 6	241322	https://www.ncbi.nlm.nih.gov/gene/?term=241322
Abhd17b	Abhd17b	abhydrolase domain containing 17B	226016	https://www.ncbi.nlm.nih.gov/gene/?term=226016
Klhl2	Klhl2	kelch-like 2, Mayven	77113	https://www.ncbi.nlm.nih.gov/gene/?term=77113
Pdzrn4	Pdzrn4	PDZ domain containing RING finger 4	239618	https://www.ncbi.nlm.nih.gov/gene/?term=239618
Adamtsl1	Adamtsl1	ADAMTS-like 1	77739	https://www.ncbi.nlm.nih.gov/gene/?term=77739
Papss2	Papss2	3'-phosphoadenosine 5'-phosphosulfate synthase 2	23972	https://www.ncbi.nlm.nih.gov/gene/?term=23972

Biology Open • Supplementary information

Agtr1b	Agtr1b	angiotensin II receptor, type 1b	11608	https://www.ncbi.nlm.nih.gov/gene/?term=11608
Apol7a	Apol7a	apolipoprotein L 7a	75761	https://www.ncbi.nlm.nih.gov/gene/?term=75761
Gusb	Gusb	glucuronidase, beta	110006	https://www.ncbi.nlm.nih.gov/gene/?term=110006
Ror2	Ror2	receptor tyrosine kinase-like orphan receptor 2	26564	https://www.ncbi.nlm.nih.gov/gene/?term=26564
Reep3	Reep3	receptor accessory protein 3	28193	https://www.ncbi.nlm.nih.gov/gene/?term=28193
Creb1	Creb1	cAMP responsive element binding protein 1	12912	https://www.ncbi.nlm.nih.gov/gene/?term=12912
Dner	Dner	delta/notch-like EGF repeat containing	227325	https://www.ncbi.nlm.nih.gov/gene/?term=227325
Socs5	Socs5	suppressor of cytokine signaling 5	56468	https://www.ncbi.nlm.nih.gov/gene/?term=56468
Atp6v0b	Atp6v0b	ATPase, H+ transporting, lysosomal V0 subunit B	114143	https://www.ncbi.nlm.nih.gov/gene/?term=114143

Biology Open • Supplementary information

Yy2	Yy2	Yy2 transcription factor	100073351	https://www.ncbi.nlm.nih.gov/gene/?term=100073[illegible]
Arhgap15	Arhgap15	Rho GTPase activating protein 15	76117	https://www.ncbi.nlm.nih.gov/gene/?term=76117
Eea1	Eea1	early endosome antigen 1	216238	https://www.ncbi.nlm.nih.gov/gene/?term=216238
Lemd3	Lemd3	LEM domain containing 3	380664	https://www.ncbi.nlm.nih.gov/gene/?term=380664
Dera	Dera	deoxyribose-phosphate aldolase (putative)	232449	https://www.ncbi.nlm.nih.gov/gene/?term=232449
Usp10	Usp10	ubiquitin specific peptidase 10	22224	https://www.ncbi.nlm.nih.gov/gene/?term=22224
Mdh1	Mdh1	malate dehydrogenase 1, NAD (soluble)	17449	https://www.ncbi.nlm.nih.gov/gene/?term=17449
Scai	Scai	suppressor of cancer cell invasion	320271	https://www.ncbi.nlm.nih.gov/gene/?term=320271
Arhgap32	Arhgap32	Rho GTPase activating protein 32	330914	https://www.ncbi.nlm.nih.gov/gene/?term=330914

Rlim	Rlim	ring finger protein, LIM domain interacting	19820	https://www.ncbi.nlm.nih.gov/gene/?term=19820
Gbx2	Gbx2	gastrulation brain homeobox 2	14472	https://www.ncbi.nlm.nih.gov/gene/?term=14472
Fry	Fry	FRY microtubule binding protein	320365	https://www.ncbi.nlm.nih.gov/gene/?term=320365
Spry2	Spry2	sprouty RTK signaling antagonist 2	24064	https://www.ncbi.nlm.nih.gov/gene/?term=24064
Sh3bgrl2	Sh3bgrl2	SH3 domain binding glutamic acid-rich protein like 2	212531	https://www.ncbi.nlm.nih.gov/gene/?term=212531
Dmd	Dmd	dystrophin, muscular dystrophy	13405	https://www.ncbi.nlm.nih.gov/gene/?term=13405
Fam217a	Fam217a	family with sequence similarity 217, member A	71864	https://www.ncbi.nlm.nih.gov/gene/?term=71864
Brinp2	Brinp2	bone morphogenic protein/retinoic acid inducible neural-specific 2	240843	https://www.ncbi.nlm.nih.gov/gene/?term=240843
Zfp950	Zfp950	zinc finger protein 950	414758	https://www.ncbi.nlm.nih.gov/gene/?term=414758

Nr0b1	Nr0b1	nuclear receptor subfamily 0, group B, member 1	11614	https://www.ncbi.nlm.nih.gov/gene/?term=11614
Crot	Crot	carnitine O-octanoyltransferase	74114	https://www.ncbi.nlm.nih.gov/gene/?term=74114
Egr1	Egr1	early growth response 1	13653	https://www.ncbi.nlm.nih.gov/gene/?term=13653
Mtcp1	Mtcp1	mature T cell proliferation 1	17763	https://www.ncbi.nlm.nih.gov/gene/?term=17763
Tbc1d32	Tbc1d32	TBC1 domain family, member 32	544696	https://www.ncbi.nlm.nih.gov/gene/?term=544696
Suz12	Suz12	SUZ12 polycomb repressive complex 2 subunit	52615	https://www.ncbi.nlm.nih.gov/gene/?term=52615
Ttc21b	Ttc21b	tetratricopeptide repeat domain 21B	73668	https://www.ncbi.nlm.nih.gov/gene/?term=73668
H2-M10.6	H2-M10.6	histocompatibility 2, M region locus 10.6	399549	https://www.ncbi.nlm.nih.gov/gene/?term=399549
Abce1	Abce1	ATP-binding cassette, sub-family E (OABP), member 1	24015	https://www.ncbi.nlm.nih.gov/gene/?term=24015

Biology Open • Supplementary information

Gm14295	Gm14295	predicted gene 14295	100039123	https://www.ncbi.nlm.nih.gov/gene/?term=100039
Chrna5	Chrna5	cholinergic receptor, nicotinic, alpha polypeptide 5	110835	https://www.ncbi.nlm.nih.gov/gene/?term=110835
Msl2	Msl2	MSL complex subunit 2	77853	https://www.ncbi.nlm.nih.gov/gene/?term=77853
Ddx42	Ddx42	DEAD (Asp-Glu-Ala-Asp) box polypeptide 42	72047	https://www.ncbi.nlm.nih.gov/gene/?term=72047
Clec3a	Clec3a	C-type lectin domain family 3, member a	403395	https://www.ncbi.nlm.nih.gov/gene/?term=403395
Zmat3	Zmat3	zinc finger matrin type 3	22401	https://www.ncbi.nlm.nih.gov/gene/?term=22401
Cxadr	Cxadr	coxsackie virus and adenovirus receptor	13052	https://www.ncbi.nlm.nih.gov/gene/?term=13052
Tmem200a	Tmem200a	transmembrane protein 200A	77220	https://www.ncbi.nlm.nih.gov/gene/?term=77220
Msi2	Msi2	musashi RNA-binding protein 2	76626	https://www.ncbi.nlm.nih.gov/gene/?term=76626

Biology Open • Supplementary information

Cdyl2	Cdyl2	chromodomain protein, Y chromosome-like 2	75796	https://www.ncbi.nlm.nih.gov/gene/?term=75796
Klrb1b	Klrb1b	killer cell lectin-like receptor subfamily B member 1B	80782	https://www.ncbi.nlm.nih.gov/gene/?term=80782
Usp21	Usp21	ubiquitin specific peptidase 21	30941	https://www.ncbi.nlm.nih.gov/gene/?term=30941
Xiap	Xiap	X-linked inhibitor of apoptosis	11798	https://www.ncbi.nlm.nih.gov/gene/?term=11798
Zfp119b	Zfp119b	zinc finger protein 119b	240120	https://www.ncbi.nlm.nih.gov/gene/?term=240120
Sertm1	Sertm1	serine rich and transmembrane domain containing 1	329641	https://www.ncbi.nlm.nih.gov/gene/?term=329641
Ids	Ids	iduronate 2-sulfatase	15931	https://www.ncbi.nlm.nih.gov/gene/?term=15931
Pcdh11x	Pcdh11x	protocadherin 11 X-linked	245578	https://www.ncbi.nlm.nih.gov/gene/?term=245578
Herc4	Herc4	hect domain and RLD 4	67345	https://www.ncbi.nlm.nih.gov/gene/?term=67345

Biology Open • Supplementary information

Bend4	Bend4	BEN domain containing 4	666938	https://www.ncbi.nlm.nih.gov/gene/?term=666938
Dlx1	Dlx1	distal-less homeobox 1	13390	https://www.ncbi.nlm.nih.gov/gene/?term=13390
Rif1	Rif1	replication timing regulatory factor 1	51869	https://www.ncbi.nlm.nih.gov/gene/?term=51869
Gria4	Gria4	glutamate receptor, ionotropic, AMPA4 (alpha 4)	14802	https://www.ncbi.nlm.nih.gov/gene/?term=14802
Nupl1	Nupl1	nucleoporin like 1	71844	https://www.ncbi.nlm.nih.gov/gene/?term=71844
Vxn	Vxn	vexin	76982	https://www.ncbi.nlm.nih.gov/gene/?term=76982
Adcyap1	Adcyap1	adenylate cyclase activating polypeptide 1	11516	https://www.ncbi.nlm.nih.gov/gene/?term=11516
Frmd3	Frmd3	FERM domain containing 3	242506	https://www.ncbi.nlm.nih.gov/gene/?term=242506
Neurl1a	Neurl1a	neuralized E3 ubiquitin protein ligase 1A	18011	https://www.ncbi.nlm.nih.gov/gene/?term=18011

Setx	Setx	senataxin	269254	https://www.ncbi.nlm.nih.gov/gene/?term=269254
Lhx8	Lhx8	LIM homeobox protein 8	16875	https://www.ncbi.nlm.nih.gov/gene/?term=16875
Bicd1	Bicd1	BICD cargo adaptor 1	12121	https://www.ncbi.nlm.nih.gov/gene/?term=12121
Brwd1	Brwd1	bromodomain and WD repeat domain containing 1	93871	https://www.ncbi.nlm.nih.gov/gene/?term=93871
Prr3	Prr3	proline-rich polypeptide 3	75210	https://www.ncbi.nlm.nih.gov/gene/?term=75210
Dscam	Dscam	DS cell adhesion molecule	13508	https://www.ncbi.nlm.nih.gov/gene/?term=13508
Cxxc5	Cxxc5	CXXC finger 5	67393	https://www.ncbi.nlm.nih.gov/gene/?term=67393
Dclre1a	Dclre1a	DNA cross-link repair 1A	55947	https://www.ncbi.nlm.nih.gov/gene/?term=55947
Zfp383	Zfp383	zinc finger protein 383	73729	https://www.ncbi.nlm.nih.gov/gene/?term=73729

Sash3	Sash3	SAM and SH3 domain containing 3	74131	https://www.ncbi.nlm.nih.gov/gene/?term=74131
Stard6	Stard6	StAR-related lipid transfer (START) domain containing 6	170461	https://www.ncbi.nlm.nih.gov/gene/?term=170461
Nup54	Nup54	nucleoporin 54	269113	https://www.ncbi.nlm.nih.gov/gene/?term=269113
Ppp4r3b	Ppp4r3b	protein phosphatase 4 regulatory subunit 3B	104570	https://www.ncbi.nlm.nih.gov/gene/?term=104570
Tmem158	Tmem158	transmembrane protein 158	72309	https://www.ncbi.nlm.nih.gov/gene/?term=72309
Slit3	Slit3	slit guidance ligand 3	20564	https://www.ncbi.nlm.nih.gov/gene/?term=20564
Slc30a10	Slc30a10	solute carrier family 30, member 10	226781	https://www.ncbi.nlm.nih.gov/gene/?term=226781
Cdc73	Cdc73	cell division cycle 73, Paf1/RNA polymerase II complex component	214498	https://www.ncbi.nlm.nih.gov/gene/?term=214498
Rrp36	Rrp36	ribosomal RNA processing 36 homolog (S. cerevisiae)	224823	https://www.ncbi.nlm.nih.gov/gene/?term=224823

Biology Open • Supplementary information

Tmem68	Tmem68	transmembrane protein 68	72098	https://www.ncbi.nlm.nih.gov/gene/?term=72098
Map3k2	Map3k2	mitogen-activated protein kinase kinase kinase 2	26405	https://www.ncbi.nlm.nih.gov/gene/?term=26405
Cnot7	Cnot7	CCR4-NOT transcription complex, subunit 7	18983	https://www.ncbi.nlm.nih.gov/gene/?term=18983
Fst	Fst	follistatin	14313	https://www.ncbi.nlm.nih.gov/gene/?term=14313
Lipo2	Lipo2	lipase, member O2	101055671	https://www.ncbi.nlm.nih.gov/gene/?term=101055
Chd9	Chd9	chromodomain helicase DNA binding protein 9	109151	https://www.ncbi.nlm.nih.gov/gene/?term=109151
Gnat1	Gnat1	guanine nucleotide binding protein, alpha transducing 1	14685	https://www.ncbi.nlm.nih.gov/gene/?term=14685
Ywhaq	Ywhaq	tyrosine 3-monooxygenase/tryptophan 5-monooxygenase activation protein theta	22630	https://www.ncbi.nlm.nih.gov/gene/?term=22630
Igf2bp3	Igf2bp3	insulin-like growth factor 2 mRNA binding protein 3	140488	https://www.ncbi.nlm.nih.gov/gene/?term=140488

Biology Open • Supplementary information

Prkar2a	Prkar2a	protein kinase, cAMP dependent regulatory, type II alpha	19087	https://www.ncbi.nlm.nih.gov/gene/?term=19087
Ufm1	Ufm1	ubiquitin-fold modifier 1	67890	https://www.ncbi.nlm.nih.gov/gene/?term=67890
Crhbp	Crhbp	corticotropin releasing hormone binding protein	12919	https://www.ncbi.nlm.nih.gov/gene/?term=12919
Igf1	Igf1	insulin-like growth factor 1	16000	https://www.ncbi.nlm.nih.gov/gene/?term=16000
Scn5a	Scn5a	sodium channel, voltage-gated, type V, alpha	20271	https://www.ncbi.nlm.nih.gov/gene/?term=20271
Seh1l	Seh1l	SEH1-like (S. cerevisiae	72124	https://www.ncbi.nlm.nih.gov/gene/?term=72124
Six3	Six3	sine oculis-related homeobox 3	20473	https://www.ncbi.nlm.nih.gov/gene/?term=20473
Serpinb10	Serpinb10	serine (or cysteine) peptidase inhibitor, clade B (ovalbumin), member 10	241197	https://www.ncbi.nlm.nih.gov/gene/?term=241197
Rpe65	Rpe65	retinal pigment epithelium 65	19892	https://www.ncbi.nlm.nih.gov/gene/?term=19892

Biology Open • Supplementary information

Srek1	Srek1	splicing regulatory glutamine/lysine-rich protein 1	218543	https://www.ncbi.nlm.nih.gov/gene/?term=218543
Aebp2	Aebp2	AE binding protein 2	11569	https://www.ncbi.nlm.nih.gov/gene/?term=11569
Zfp516	Zfp516	zinc finger protein 516	329003	https://www.ncbi.nlm.nih.gov/gene/?term=329003
Slc7a15	Slc7a15	solute carrier family 7 (cationic amino acid transporter, y+ system), member 15	328059	https://www.ncbi.nlm.nih.gov/gene/?term=328059
Sufu	Sufu	SUFU negative regulator of hedgehog signaling	24069	https://www.ncbi.nlm.nih.gov/gene/?term=24069
Mindy4	Mindy4	MINDY lysine 48 deubiquitinase 4	330323	https://www.ncbi.nlm.nih.gov/gene/?term=330323
Cnot2	Cnot2	CCR4-NOT transcription complex, subunit 2	72068	https://www.ncbi.nlm.nih.gov/gene/?term=72068
Ap2a2	Ap2a2	adaptor-related protein complex 2, alpha 2 subunit	11772	https://www.ncbi.nlm.nih.gov/gene/?term=11772
Laptm5	Laptm5	lysosomal-associated protein transmembrane 5	16792	https://www.ncbi.nlm.nih.gov/gene/?term=16792

Biology Open • Supplementary information

Ccdc150	Ccdc150	coiled-coil domain containing 150	78016	https://www.ncbi.nlm.nih.gov/gene/?term=78016
Sorcs2	Sorcs2	sortilin-related VPS10 domain containing receptor 2	81840	https://www.ncbi.nlm.nih.gov/gene/?term=81840
Brwd3	Brwd3	bromodomain and WD repeat domain containing 3	382236	https://www.ncbi.nlm.nih.gov/gene/?term=382236
Dhx58	Dhx58	DEXH (Asp-Glu-X-His) box polypeptide 58	80861	https://www.ncbi.nlm.nih.gov/gene/?term=80861
Sirt1	Sirt1	sirtuin 1	93759	https://www.ncbi.nlm.nih.gov/gene/?term=93759
Slc40a1	Slc40a1	solute carrier family 40 (iron-regulated transporter), member 1	53945	https://www.ncbi.nlm.nih.gov/gene/?term=53945
Poldip2	Poldip2	polymerase (DNA-directed), delta interacting protein 2	67811	https://www.ncbi.nlm.nih.gov/gene/?term=67811
Col12a1	Col12a1	collagen, type XII, alpha 1	12816	https://www.ncbi.nlm.nih.gov/gene/?term=12816
Zfp799	Zfp799	zinc finger protein 799	240064	https://www.ncbi.nlm.nih.gov/gene/?term=240064

Biology Open • Supplementary information

BC052040	BC052040	cDNA sequence BC052040	399568	https://www.ncbi.nlm.nih.gov/gene/?term=399568
Vezf1	Vezf1	vascular endothelial zinc finger 1	22344	https://www.ncbi.nlm.nih.gov/gene/?term=22344
Rit1	Rit1	Ras-like without CAAX 1	19769	https://www.ncbi.nlm.nih.gov/gene/?term=19769
Pnrc2	Pnrc2	proline-rich nuclear receptor coactivator 2	52830	https://www.ncbi.nlm.nih.gov/gene/?term=52830
Syne2	Syne2	spectrin repeat containing, nuclear envelope 2	319565	https://www.ncbi.nlm.nih.gov/gene/?term=319565
Igfbp7	Igfbp7	insulin-like growth factor binding protein 7	29817	https://www.ncbi.nlm.nih.gov/gene/?term=29817
Srpr	Srpr	signal recognition particle receptor ('docking protein')	67398	https://www.ncbi.nlm.nih.gov/gene/?term=67398
Phf1	Phf1	PHD finger protein 1	21652	https://www.ncbi.nlm.nih.gov/gene/?term=21652
Pnisr	Pnisr	PNN interacting serine/arginine-rich	66625	https://www.ncbi.nlm.nih.gov/gene/?term=66625

Biology Open • Supplementary information

Lix1l	Lix1l	Lix1-like	280411	https://www.ncbi.nlm.nih.gov/gene/?term=280411
Npy5r	Npy5r	neuropeptide Y receptor Y5	18168	https://www.ncbi.nlm.nih.gov/gene/?term=18168
Bmp7	Bmp7	bone morphogenetic protein 7	12162	https://www.ncbi.nlm.nih.gov/gene/?term=12162
Slc12a2	Slc12a2	solute carrier family 12, member 2	20496	https://www.ncbi.nlm.nih.gov/gene/?term=20496
Evx2	Evx2	even-skipped homeobox 2	14029	https://www.ncbi.nlm.nih.gov/gene/?term=14029
Igf2r	Igf2r	insulin-like growth factor 2 receptor	16004	https://www.ncbi.nlm.nih.gov/gene/?term=16004
Ing2	Ing2	inhibitor of growth family, member 2	69260	https://www.ncbi.nlm.nih.gov/gene/?term=69260
Luc7l3	Luc7l3	LUC7-like 3 (S. cerevisiae)	67684	https://www.ncbi.nlm.nih.gov/gene/?term=67684
Lig3	Lig3	ligase III, DNA, ATP-dependent	16882	https://www.ncbi.nlm.nih.gov/gene/?term=16882

Biology Open • Supplementary information

Daam1	Daam1	dishevelled associated activator of morphogenesis 1	208846	https://www.ncbi.nlm.nih.gov/gene/?term=208846
Usf3	Usf3	upstream transcription factor family member 3	207806	https://www.ncbi.nlm.nih.gov/gene/?term=207806
Calu	Calu	calumenin	12321	https://www.ncbi.nlm.nih.gov/gene/?term=12321
Hand1	Hand1	heart and neural crest derivatives expressed 1	15110	https://www.ncbi.nlm.nih.gov/gene/?term=15110
Kdm4c	Kdm4c	lysine (K)-specific demethylase 4C	76804	https://www.ncbi.nlm.nih.gov/gene/?term=76804
Cdh19	Cdh19	cadherin 19, type 2	227485	https://www.ncbi.nlm.nih.gov/gene/?term=227485
2510009E07Rik	2510009E07Rik	RIKEN cDNA 2510009E07 gene	72190	https://www.ncbi.nlm.nih.gov/gene/?term=72190
Fbxw27	Fbxw27	F-box and WD-40 domain protein 27	76998	https://www.ncbi.nlm.nih.gov/gene/?term=76998
Poglut1	Poglut1	protein O-glucosyltransferase 1	224143	https://www.ncbi.nlm.nih.gov/gene/?term=224143

Biology Open • Supplementary information

Slc15a5	Slc15a5	solute carrier family 15, member 5	277898	https://www.ncbi.nlm.nih.gov/gene/?term=277898
Chfr	Chfr	checkpoint with forkhead and ring finger domains	231600	https://www.ncbi.nlm.nih.gov/gene/?term=231600
Pdhx	Pdhx	pyruvate dehydrogenase complex, component X	27402	https://www.ncbi.nlm.nih.gov/gene/?term=27402
Hoxd8	Hoxd8	homeobox D8	15437	https://www.ncbi.nlm.nih.gov/gene/?term=15437
BC003965	BC003965	cDNA sequence BC003965	214489	https://www.ncbi.nlm.nih.gov/gene/?term=214489
Thap2	Thap2	THAP domain containing, apoptosis associated protein 2	66816	https://www.ncbi.nlm.nih.gov/gene/?term=66816
Nt5dc3	Nt5dc3	5'-nucleotidase domain containing 3	103466	https://www.ncbi.nlm.nih.gov/gene/?term=103466
Cyb5d2	Cyb5d2	cytochrome b5 domain containing 2	192986	https://www.ncbi.nlm.nih.gov/gene/?term=192986
Yod1	Yod1	YOD1 deubiquitinase	226418	https://www.ncbi.nlm.nih.gov/gene/?term=226418

Biology Open • Supplementary information

Fam120c	Fam120c	family with sequence similarity 120, member C	207375	https://www.ncbi.nlm.nih.gov/gene/?term=207375
Hs3st2	Hs3st2	heparan sulfate (glucosamine) 3-O-sulfotransferase 2	195646	https://www.ncbi.nlm.nih.gov/gene/?term=195646
Ttc39a	Ttc39a	tetratricopeptide repeat domain 39A	230603	https://www.ncbi.nlm.nih.gov/gene/?term=230603
Fgf21	Fgf21	NA	56636	https://www.ncbi.nlm.nih.gov/gene/?term=56636
Ap1ar	Ap1ar	adaptor-related protein complex 1 associated regulatory protein	211556	https://www.ncbi.nlm.nih.gov/gene/?term=211556
Baz2a	Baz2a	bromodomain adjacent to zinc finger domain, 2A	116848	https://www.ncbi.nlm.nih.gov/gene/?term=116848
Celsr2	Celsr2	cadherin, EGF LAG seven-pass G-type receptor 2	53883	https://www.ncbi.nlm.nih.gov/gene/?term=53883
Dctn4	Dctn4	dynactin 4	67665	https://www.ncbi.nlm.nih.gov/gene/?term=67665
Taok1	Taok1	TAO kinase 1	216965	https://www.ncbi.nlm.nih.gov/gene/?term=216965

Biology Open • Supplementary information

G3bp2	G3bp2	GTPase activating protein (SH3 domain) binding protein 2	23881	https://www.ncbi.nlm.nih.gov/gene/?term=23881
Ppp2r5e	Ppp2r5e	protein phosphatase 2, regulatory subunit B', epsilon	26932	https://www.ncbi.nlm.nih.gov/gene/?term=26932
Gna12	Gna12	guanine nucleotide binding protein, alpha 12	14673	https://www.ncbi.nlm.nih.gov/gene/?term=14673
Adamtsl3	Adamtsl3	ADAMTS-like 3	269959	https://www.ncbi.nlm.nih.gov/gene/?term=269959
Serpine2	Serpine2	serine (or cysteine) peptidase inhibitor, clade E, member 2	20720	https://www.ncbi.nlm.nih.gov/gene/?term=20720
Galnt7	Galnt7	polypeptide N-acetylgalactosaminyltransferase 7	108150	https://www.ncbi.nlm.nih.gov/gene/?term=108150
Tubgcp3	Tubgcp3	tubulin, gamma complex associated protein 3	259279	https://www.ncbi.nlm.nih.gov/gene/?term=259279
Sema3a	Sema3a	sema domain, immunoglobulin domain (Ig), short basic domain, secreted, (semaphorin) 3A	20346	https://www.ncbi.nlm.nih.gov/gene/?term=20346
Itga3	Itga3	integrin alpha 3	16400	https://www.ncbi.nlm.nih.gov/gene/?term=16400

Biology Open • Supplementary information

Tgif2	Tgif2	TGFB-induced factor homeobox 2	228839	https://www.ncbi.nlm.nih.gov/gene/?term=228839
Rb1	Rb1	RB transcriptional corepressor 1	19645	https://www.ncbi.nlm.nih.gov/gene/?term=19645
Mucl2	Mucl2	mucin-like 2	20770	https://www.ncbi.nlm.nih.gov/gene/?term=20770
Runx1	Runx1	runt related transcription factor 1	12394	https://www.ncbi.nlm.nih.gov/gene/?term=12394
Pak4	Pak4	p21 (RAC1) activated kinase 4	70584	https://www.ncbi.nlm.nih.gov/gene/?term=70584
Aplp2	Aplp2	amyloid beta (A4) precursor-like protein 2	11804	https://www.ncbi.nlm.nih.gov/gene/?term=11804
4933434E20Rik	4933434E20Rik	RIKEN cDNA 4933434E20 gene	99650	https://www.ncbi.nlm.nih.gov/gene/?term=99650
Raph1	Raph1	Ras association (RalGDS/AF-6) and pleckstrin homology domains 1	77300	https://www.ncbi.nlm.nih.gov/gene/?term=77300
Fam199x	Fam199x	family with sequence similarity 199, X-linked	245622	https://www.ncbi.nlm.nih.gov/gene/?term=245622

Biology Open • Supplementary information

Itgb8	Itgb8	integrin beta 8	320910	https://www.ncbi.nlm.nih.gov/gene/?term=320910
Rbbp6	Rbbp6	retinoblastoma binding protein 6, ubiquitin ligase	19647	https://www.ncbi.nlm.nih.gov/gene/?term=19647
Tmem62	Tmem62	transmembrane protein 62	96957	https://www.ncbi.nlm.nih.gov/gene/?term=96957
Lin28b	Lin28b	lin-28 homolog B (C. elegans)	380669	https://www.ncbi.nlm.nih.gov/gene/?term=380669
Cd151	Cd151	CD151 antigen	12476	https://www.ncbi.nlm.nih.gov/gene/?term=12476
Slc39a10	Slc39a10	solute carrier family 39 (zinc transporter), member 10	227059	https://www.ncbi.nlm.nih.gov/gene/?term=227059
Pawr	Pawr	PRKC, apoptosis, WT1, regulator	114774	https://www.ncbi.nlm.nih.gov/gene/?term=114774
Cdt1	Cdt1	chromatin licensing and DNA replication factor 1	67177	https://www.ncbi.nlm.nih.gov/gene/?term=67177
Rimklb	Rimklb	ribosomal modification protein rimK-like family member B	108653	https://www.ncbi.nlm.nih.gov/gene/?term=108653

Biology Open • Supplementary information

Katnbl1	Katnbl1	katanin p80 subunit B like 1	72425	https://www.ncbi.nlm.nih.gov/gene/?term=72425
Vstm4	Vstm4	V-set and transmembrane domain containing 4	320736	https://www.ncbi.nlm.nih.gov/gene/?term=320736
Mro	Mro	maestro	71263	https://www.ncbi.nlm.nih.gov/gene/?term=71263
Togaram2	Togaram2	TOG array regulator of axonemal microtubules 2	320159	https://www.ncbi.nlm.nih.gov/gene/?term=320159
Nckap1	Nckap1	NCK-associated protein 1	50884	https://www.ncbi.nlm.nih.gov/gene/?term=50884
Ppp1r9a	Ppp1r9a	protein phosphatase 1, regulatory subunit 9A	243725	https://www.ncbi.nlm.nih.gov/gene/?term=243725
Dpp8	Dpp8	dipeptidylpeptidase 8	74388	https://www.ncbi.nlm.nih.gov/gene/?term=74388
Ehbp1	Ehbp1	EH domain binding protein 1	216565	https://www.ncbi.nlm.nih.gov/gene/?term=216565
Pls1	Pls1	plastin 1 (I-isoform)	102502	https://www.ncbi.nlm.nih.gov/gene/?term=102502

Biology Open • Supplementary information

Ypel1	Ypel1	yippee like 1	106369	https://www.ncbi.nlm.nih.gov/gene/?term=106369
Ephb1	Ephb1	Eph receptor B1	270190	https://www.ncbi.nlm.nih.gov/gene/?term=270190
Muc15	Muc15	mucin 15	269328	https://www.ncbi.nlm.nih.gov/gene/?term=269328
Naa16	Naa16	N(alpha)-acetyltransferase 16, NatA auxiliary subunit	66897	https://www.ncbi.nlm.nih.gov/gene/?term=66897
Gpr158	Gpr158	G protein-coupled receptor 158	241263	https://www.ncbi.nlm.nih.gov/gene/?term=241263
Nckap5	Nckap5	NCK-associated protein 5	210356	https://www.ncbi.nlm.nih.gov/gene/?term=210356
Mettl21e	Mettl21e	methyltransferase like 21E	403183	https://www.ncbi.nlm.nih.gov/gene/?term=403183
Ggnbp2	Ggnbp2	gametogenetin binding protein 2	217039	https://www.ncbi.nlm.nih.gov/gene/?term=217039
Tceal8	Tceal8	transcription elongation factor A (SII)-like 8	66684	https://www.ncbi.nlm.nih.gov/gene/?term=66684

Biology Open • Supplementary information

Notum	Notum	notum palmitoleoyl-protein carboxylesterase	77583	https://www.ncbi.nlm.nih.gov/gene/?term=77583
Me3	Me3	malic enzyme 3, NADP(+)-dependent, mitochondrial	109264	https://www.ncbi.nlm.nih.gov/gene/?term=109264
Edil3	Edil3	EGF-like repeats and discoidin I-like domains 3	13612	https://www.ncbi.nlm.nih.gov/gene/?term=13612
Mycbp	Mycbp	MYC binding protein	56309	https://www.ncbi.nlm.nih.gov/gene/?term=56309
Hspa4l	Hspa4l	heat shock protein 4 like	18415	https://www.ncbi.nlm.nih.gov/gene/?term=18415
Id4	Id4	inhibitor of DNA binding 4	15904	https://www.ncbi.nlm.nih.gov/gene/?term=15904
Fam160b2	Fam160b2	family with sequence similarity 160, member B2	239170	https://www.ncbi.nlm.nih.gov/gene/?term=239170
Far1	Far1	fatty acyl CoA reductase 1	67420	https://www.ncbi.nlm.nih.gov/gene/?term=67420
Tfdp2	Tfdp2	transcription factor Dp 2	211586	https://www.ncbi.nlm.nih.gov/gene/?term=211586

Etaa1	Etaa1	Ewing tumor-associated antigen 1	68145	https://www.ncbi.nlm.nih.gov/gene/?term=68145
Nip7	Nip7	NIP7, nucleolar pre-rRNA processing protein	66164	https://www.ncbi.nlm.nih.gov/gene/?term=66164
Hspbap1	Hspbap1	Hspb associated protein 1	66667	https://www.ncbi.nlm.nih.gov/gene/?term=66667
Kdsr	Kdsr	3-ketodihydrosphingosine reductase	70750	https://www.ncbi.nlm.nih.gov/gene/?term=70750
Ccdc34	Ccdc34	coiled-coil domain containing 34	68201	https://www.ncbi.nlm.nih.gov/gene/?term=68201
Wdr37	Wdr37	WD repeat domain 37	207615	https://www.ncbi.nlm.nih.gov/gene/?term=207615
Limd1	Limd1	LIM domains containing 1	29806	https://www.ncbi.nlm.nih.gov/gene/?term=29806
En1	En1	engrailed 1	13798	https://www.ncbi.nlm.nih.gov/gene/?term=13798
Tanc2	Tanc2	tetratricopeptide repeat, ankyrin repeat and coiled-coil containing 2	77097	https://www.ncbi.nlm.nih.gov/gene/?term=77097

Fam53c	Fam53c	family with sequence similarity 53, member C	66306	https://www.ncbi.nlm.nih.gov/gene/?term=66306
Slc6a8	Slc6a8	solute carrier family 6 (neurotransmitter transporter, creatine), member 8	102857	https://www.ncbi.nlm.nih.gov/gene/?term=102857
Rpgr	Rpgr	retinitis pigmentosa GTPase regulator	19893	https://www.ncbi.nlm.nih.gov/gene/?term=19893
Zbtb26	Zbtb26	zinc finger and BTB domain containing 26	320633	https://www.ncbi.nlm.nih.gov/gene/?term=320633
Arhgef4	Arhgef4	Rho guanine nucleotide exchange factor (GEF) 4	226970	https://www.ncbi.nlm.nih.gov/gene/?term=226970
Zkscan1	Zkscan1	zinc finger with KRAB and SCAN domains 1	74570	https://www.ncbi.nlm.nih.gov/gene/?term=74570
Sh2b1	Sh2b1	SH2B adaptor protein 1	20399	https://www.ncbi.nlm.nih.gov/gene/?term=20399
Wdr77	Wdr77	WD repeat domain 77	70465	https://www.ncbi.nlm.nih.gov/gene/?term=70465
Arid1b	Arid1b	AT rich interactive domain 1B (SWI-like)	239985	https://www.ncbi.nlm.nih.gov/gene/?term=239985

Biology Open • Supplementary information

Kdm6b	Kdm6b	KDM1 lysine (K)-specific demethylase 6B	216850	https://www.ncbi.nlm.nih.gov/gene/?term=216850
Mrfap1	Mrfap1	Morf4 family associated protein 1	67568	https://www.ncbi.nlm.nih.gov/gene/?term=67568
Samd1	Samd1	sterile alpha motif domain containing 1	666704	https://www.ncbi.nlm.nih.gov/gene/?term=666704
Grm3	Grm3	glutamate receptor, metabotropic 3	108069	https://www.ncbi.nlm.nih.gov/gene/?term=108069
Fam160a1	Fam160a1	family with sequence similarity 160, member A1	229488	https://www.ncbi.nlm.nih.gov/gene/?term=229488
Pxdc1	Pxdc1	PX domain containing 1	66895	https://www.ncbi.nlm.nih.gov/gene/?term=66895
Maml3	Maml3	mastermind like transcriptional coactivator 3	433586	https://www.ncbi.nlm.nih.gov/gene/?term=433586
Sgcz	Sgcz	sarcoglycan zeta	244431	https://www.ncbi.nlm.nih.gov/gene/?term=244431
Irf2bp2	Irf2bp2	interferon regulatory factor 2 binding protein 2	270110	https://www.ncbi.nlm.nih.gov/gene/?term=270110

Biology Open • Supplementary information

Casp6	Casp6	caspase 6	12368	https://www.ncbi.nlm.nih.gov/gene/?term=12368
Srr	Srr	serine racemase	27364	https://www.ncbi.nlm.nih.gov/gene/?term=27364
Rnf149	Rnf149	ring finger protein 149	67702	https://www.ncbi.nlm.nih.gov/gene/?term=67702
Abraxas2	Abraxas2	BRISC complex subunit	109359	https://www.ncbi.nlm.nih.gov/gene/?term=109359
Stk4	Stk4	serine/threonine kinase 4	58231	https://www.ncbi.nlm.nih.gov/gene/?term=58231
Rad23b	Rad23b	RAD23 homolog B, nucleotide excision repair protein	19359	https://www.ncbi.nlm.nih.gov/gene/?term=19359
Ddr1	Ddr1	discoidin domain receptor family, member 1	12305	https://www.ncbi.nlm.nih.gov/gene/?term=12305
Bcam	Bcam	basal cell adhesion molecule	57278	https://www.ncbi.nlm.nih.gov/gene/?term=57278
Clcn3	Clcn3	chloride channel, voltage-sensitive 3	12725	https://www.ncbi.nlm.nih.gov/gene/?term=12725

Biology Open • Supplementary information

Large1	Large1	LARGE xylosyl- and glucuronyltransferase 1	16795	https://www.ncbi.nlm.nih.gov/gene/?term=16795
Gpd2	Gpd2	glycerol phosphate dehydrogenase 2, mitochondrial	14571	https://www.ncbi.nlm.nih.gov/gene/?term=14571
Zmpste24	Zmpste24	zinc metallopeptidase, STE24	230709	https://www.ncbi.nlm.nih.gov/gene/?term=230709
Tspan6	Tspan6	tetraspanin 6	56496	https://www.ncbi.nlm.nih.gov/gene/?term=56496
Tef	Tef	thyrotroph embryonic factor	21685	https://www.ncbi.nlm.nih.gov/gene/?term=21685
Mical3	Mical3	microtubule associated monooxygenase, calponin and LIM domain containing 3	194401	https://www.ncbi.nlm.nih.gov/gene/?term=194401
Myrf	Myrf	myelin regulatory factor	225908	https://www.ncbi.nlm.nih.gov/gene/?term=225908
Hspa5	Hspa5	heat shock protein 5	14828	https://www.ncbi.nlm.nih.gov/gene/?term=14828
Rassf3	Rassf3	Ras association (RalGDS/AF-6) domain family member 3	192678	https://www.ncbi.nlm.nih.gov/gene/?term=192678

Nfil3	Nfil3	nuclear factor, interleukin 3, regulated	18030	https://www.ncbi.nlm.nih.gov/gene/?term=18030
Fzd6	Fzd6	frizzled class receptor 6	14368	https://www.ncbi.nlm.nih.gov/gene/?term=14368
Klhl20	Klhl20	kelch-like 20	226541	https://www.ncbi.nlm.nih.gov/gene/?term=226541
Atp1b3	Atp1b3	ATPase, Na+/K+ transporting, beta 3 polypeptide	11933	https://www.ncbi.nlm.nih.gov/gene/?term=11933
Hspa12a	Hspa12a	heat shock protein 12A	73442	https://www.ncbi.nlm.nih.gov/gene/?term=73442
Fzd4	Fzd4	frizzled class receptor 4	14366	https://www.ncbi.nlm.nih.gov/gene/?term=14366
Sun1	Sun1	Sad1 and UNC84 domain containing 1	77053	https://www.ncbi.nlm.nih.gov/gene/?term=77053
Hmcn1	Hmcn1	hemicentin 1	545370	https://www.ncbi.nlm.nih.gov/gene/?term=545370
Kpna4	Kpna4	karyopherin (importin) alpha 4	16649	https://www.ncbi.nlm.nih.gov/gene/?term=16649

Biology Open • Supplementary information

Gcnt2	Gcnt2	glucosaminyl (N-acetyl) transferase 2, I-branching enzyme	14538	https://www.ncbi.nlm.nih.gov/gene/?term=14538
Map3k11	Map3k11	mitogen-activated protein kinase kinase kinase 11	26403	https://www.ncbi.nlm.nih.gov/gene/?term=26403
Aftph	Aftph	aftiphilin	216549	https://www.ncbi.nlm.nih.gov/gene/?term=216549
Nudt10	Nudt10	nudix (nucleoside diphosphate linked moiety X)-type motif 10	102954	https://www.ncbi.nlm.nih.gov/gene/?term=102954
Ece1	Ece1	endothelin converting enzyme 1	230857	https://www.ncbi.nlm.nih.gov/gene/?term=230857
Plxna2	Plxna2	plexin A2	18845	https://www.ncbi.nlm.nih.gov/gene/?term=18845
Arhgap12	Arhgap12	Rho GTPase activating protein 12	75415	https://www.ncbi.nlm.nih.gov/gene/?term=75415
Cacna1b	Cacna1b	calcium channel, voltage-dependent, N type, alpha 1B subunit	12287	https://www.ncbi.nlm.nih.gov/gene/?term=12287
Zfp579	Zfp579	zinc finger protein 579	68490	https://www.ncbi.nlm.nih.gov/gene/?term=68490

Biology Open • Supplementary information

Dnaaf5	Dnaaf5	dynein, axonemal assembly factor 5	433956	https://www.ncbi.nlm.nih.gov/gene/?term=433956
Taf9b	Taf9b	TATA-box binding protein associated factor 9B	407786	https://www.ncbi.nlm.nih.gov/gene/?term=407786
M6pr	M6pr	mannose-6-phosphate receptor, cation dependent	17113	https://www.ncbi.nlm.nih.gov/gene/?term=17113
Myef2	Myef2	myelin basic protein expression factor 2, repressor	17876	https://www.ncbi.nlm.nih.gov/gene/?term=17876
Npas2	Npas2	neuronal PAS domain protein 2	18143	https://www.ncbi.nlm.nih.gov/gene/?term=18143
Ralgapa1	Ralgapa1	Ral GTPase activating protein, alpha subunit 1	56784	https://www.ncbi.nlm.nih.gov/gene/?term=56784
Naa40	Naa40	N(alpha)-acetyltransferase 40, NatD catalytic subunit	70999	https://www.ncbi.nlm.nih.gov/gene/?term=70999
Golim4	Golim4	golgi integral membrane protein 4	73124	https://www.ncbi.nlm.nih.gov/gene/?term=73124
Gpm6b	Gpm6b	glycoprotein m6b	14758	https://www.ncbi.nlm.nih.gov/gene/?term=14758

Biology Open • Supplementary information

Anxa5	Anxa5	annexin A5	11747	https://www.ncbi.nlm.nih.gov/gene/?term=11747
Glcci1	Glcci1	glucocorticoid induced transcript 1	170772	https://www.ncbi.nlm.nih.gov/gene/?term=170772
Cabp5	Cabp5	calcium binding protein 5	29865	https://www.ncbi.nlm.nih.gov/gene/?term=29865
Derl2	Derl2	Der1-like domain family, member 2	116891	https://www.ncbi.nlm.nih.gov/gene/?term=116891
Fundc1	Fundc1	FUN14 domain containing 1	72018	https://www.ncbi.nlm.nih.gov/gene/?term=72018
Itk	Itk	IL2 inducible T cell kinase	16428	https://www.ncbi.nlm.nih.gov/gene/?term=16428
Wwtr1	Wwtr1	WW domain containing transcription regulator 1	97064	https://www.ncbi.nlm.nih.gov/gene/?term=97064
Wif1	Wif1	Wnt inhibitory factor 1	24117	https://www.ncbi.nlm.nih.gov/gene/?term=24117
Ccnh	Ccnh	cyclin H	66671	https://www.ncbi.nlm.nih.gov/gene/?term=66671

Biology Open • Supplementary information

Cep41	Cep41	centrosomal protein 41	83922	https://www.ncbi.nlm.nih.gov/gene/?term=83922
Dtx2	Dtx2	deltex 2, E3 ubiquitin ligase	74198	https://www.ncbi.nlm.nih.gov/gene/?term=74198
Zdhhc5	Zdhhc5	zinc finger, DHHC domain containing 5	228136	https://www.ncbi.nlm.nih.gov/gene/?term=228136
Ccnjl	Ccnjl	cyclin J-like	380694	https://www.ncbi.nlm.nih.gov/gene/?term=380694
Ube2w	Ube2w	ubiquitin-conjugating enzyme E2W (putative)	66799	https://www.ncbi.nlm.nih.gov/gene/?term=66799
Kat2b	Kat2b	K(lysine) acetyltransferase 2B	18519	https://www.ncbi.nlm.nih.gov/gene/?term=18519
Grhl1	Grhl1	grainyhead like transcription factor 1	195733	https://www.ncbi.nlm.nih.gov/gene/?term=195733
Isca1	Isca1	iron-sulfur cluster assembly 1	69046	https://www.ncbi.nlm.nih.gov/gene/?term=69046
Zfp521	Zfp521	zinc finger protein 521	225207	https://www.ncbi.nlm.nih.gov/gene/?term=225207

Biology Open • Supplementary information

Map1b	Map1b	microtubule-associated protein 1B	17755	https://www.ncbi.nlm.nih.gov/gene/?term=17755
Fnip1	Fnip1	folliculin interacting protein 1	216742	https://www.ncbi.nlm.nih.gov/gene/?term=216742
Dpp10	Dpp10	dipeptidylpeptidase 10	269109	https://www.ncbi.nlm.nih.gov/gene/?term=269109
Gm5148	Gm5148	predicted gene 5148	381438	https://www.ncbi.nlm.nih.gov/gene/?term=381438
Cpeb4	Cpeb4	cytoplasmic polyadenylation element binding protein 4	67579	https://www.ncbi.nlm.nih.gov/gene/?term=67579
Atxn3	Atxn3	ataxin 3	110616	https://www.ncbi.nlm.nih.gov/gene/?term=110616
Dcaf6	Dcaf6	DDB1 and CUL4 associated factor 6	74106	https://www.ncbi.nlm.nih.gov/gene/?term=74106
Ptpro	Ptpro	protein tyrosine phosphatase, receptor type, O	19277	https://www.ncbi.nlm.nih.gov/gene/?term=19277
Syn2	Syn2	synapsin II	20965	https://www.ncbi.nlm.nih.gov/gene/?term=20965

Biology Open • Supplementary information

Med19	Med19	mediator complex subunit 19	381379	https://www.ncbi.nlm.nih.gov/gene/?term=381379
Hecw1	Hecw1	HECT, C2 and WW domain containing E3 ubiquitin protein ligase 1	94253	https://www.ncbi.nlm.nih.gov/gene/?term=94253
Ccnc	Ccnc	cyclin C	51813	https://www.ncbi.nlm.nih.gov/gene/?term=51813
Fam20c	Fam20c	family with sequence similarity 20, member C	80752	https://www.ncbi.nlm.nih.gov/gene/?term=80752
Edem1	Edem1	ER degradation enhancer, mannosidase alpha-like 1	192193	https://www.ncbi.nlm.nih.gov/gene/?term=192193
Hand2	Hand2	heart and neural crest derivatives expressed 2	15111	https://www.ncbi.nlm.nih.gov/gene/?term=15111
Eomes	Eomes	eomesodermin	13813	https://www.ncbi.nlm.nih.gov/gene/?term=13813
Pip5k1c	Pip5k1c	phosphatidylinositol-4-phosphate 5-kinase, type 1 gamma	18717	https://www.ncbi.nlm.nih.gov/gene/?term=18717
Sgk3	Sgk3	serum/glucocorticoid regulated kinase 3	170755	https://www.ncbi.nlm.nih.gov/gene/?term=170755

Dennd1b	Dennd1b	DENN/MADD domain containing 1B	329260	https://www.ncbi.nlm.nih.gov/gene/?term=329260
Rbpms2	Rbpms2	RNA binding protein with multiple splicing 2	71973	https://www.ncbi.nlm.nih.gov/gene/?term=71973
Kmt5b	Kmt5b	lysine methyltransferase 5B	225888	https://www.ncbi.nlm.nih.gov/gene/?term=225888
Gla	Gla	galactosidase, alpha	11605	https://www.ncbi.nlm.nih.gov/gene/?term=11605
Fam160b1	Fam160b1	family with sequence similarity 160, member B1	226252	https://www.ncbi.nlm.nih.gov/gene/?term=226252
Pitpna	Pitpna	phosphatidylinositol transfer protein, alpha	18738	https://www.ncbi.nlm.nih.gov/gene/?term=18738
Atp7a	Atp7a	ATPase, Cu++ transporting, alpha polypeptide	11977	https://www.ncbi.nlm.nih.gov/gene/?term=11977
Dsc2	Dsc2	desmocollin 2	13506	https://www.ncbi.nlm.nih.gov/gene/?term=13506
Dennd4b	Dennd4b	DENN/MADD domain containing 4B	229541	https://www.ncbi.nlm.nih.gov/gene/?term=229541

Iqgap2	Iqgap2	IQ motif containing GTPase activating protein 2	544963	https://www.ncbi.nlm.nih.gov/gene/?term=544963
Nox4	Nox4	NADPH oxidase 4	50490	https://www.ncbi.nlm.nih.gov/gene/?term=50490
Ppp1r37	Ppp1r37	protein phosphatase 1, regulatory subunit 37	232947	https://www.ncbi.nlm.nih.gov/gene/?term=232947
Cdca7l	Cdca7l	cell division cycle associated 7 like	217946	https://www.ncbi.nlm.nih.gov/gene/?term=217946
Peak1	Peak1	pseudopodium-enriched atypical kinase 1	244895	https://www.ncbi.nlm.nih.gov/gene/?term=244895
Trim65	Trim65	tripartite motif-containing 65	338364	https://www.ncbi.nlm.nih.gov/gene/?term=338364
Ddc	Ddc	dopa decarboxylase	13195	https://www.ncbi.nlm.nih.gov/gene/?term=13195
4932438A13Rik	4932438A13Rik	RIKEN cDNA 4932438A13 gene	229227	https://www.ncbi.nlm.nih.gov/gene/?term=229227
Adam19	Adam19	a disintegrin and metallopeptidase domain 19 (meltrin beta)	11492	https://www.ncbi.nlm.nih.gov/gene/?term=11492

Biology Open • Supplementary information

Phlpp2	Phlpp2	PH domain and leucine rich repeat protein phosphatase 2	244650	https://www.ncbi.nlm.nih.gov/gene/?term=244650
Zfyve21	Zfyve21	zinc finger, FYVE domain containing 21	68520	https://www.ncbi.nlm.nih.gov/gene/?term=68520
Itpr1	Itpr1	inositol 1,4,5-trisphosphate receptor 1	16438	https://www.ncbi.nlm.nih.gov/gene/?term=16438
Cic	Cic	capicua transcriptional repressor	71722	https://www.ncbi.nlm.nih.gov/gene/?term=71722
Snx13	Snx13	sorting nexin 13	217463	https://www.ncbi.nlm.nih.gov/gene/?term=217463
Itga6	Itga6	integrin alpha 6	16403	https://www.ncbi.nlm.nih.gov/gene/?term=16403
Itgav	Itgav	integrin alpha V	16410	https://www.ncbi.nlm.nih.gov/gene/?term=16410
Spryd4	Spryd4	SPRY domain containing 4	66701	https://www.ncbi.nlm.nih.gov/gene/?term=66701
Cdk16	Cdk16	cyclin-dependent kinase 16	18555	https://www.ncbi.nlm.nih.gov/gene/?term=18555

Biology Open • Supplementary information

Myo1b	Myo1b	myosin IB	17912	https://www.ncbi.nlm.nih.gov/gene/?term=17912
Klhl14	Klhl14	kelch-like 14	225266	https://www.ncbi.nlm.nih.gov/gene/?term=225266
Gpr180	Gpr180	G protein-coupled receptor 180	58245	https://www.ncbi.nlm.nih.gov/gene/?term=58245
Xrn1	Xrn1	5'-3' exoribonuclease 1	24127	https://www.ncbi.nlm.nih.gov/gene/?term=24127
Mia3	Mia3	melanoma inhibitory activity 3	338366	https://www.ncbi.nlm.nih.gov/gene/?term=338366
Gid4	Gid4	GID complex subunit 4, VID24 homolog	66771	https://www.ncbi.nlm.nih.gov/gene/?term=66771
Tob2	Tob2	transducer of ERBB2, 2	57259	https://www.ncbi.nlm.nih.gov/gene/?term=57259
Cnep1r1	Cnep1r1	CTD nuclear envelope phosphatase 1 regulatory subunit 1	382030	https://www.ncbi.nlm.nih.gov/gene/?term=382030
P3h3	P3h3	prolyl 3-hydroxylase 3	14789	https://www.ncbi.nlm.nih.gov/gene/?term=14789

Biology Open • Supplementary information

Lhfpl2	Lhfpl2	lipoma HMGIC fusion partner-like 2	218454	https://www.ncbi.nlm.nih.gov/gene/?term=218454
Slc6a1	Slc6a1	solute carrier family 6 (neurotransmitter transporter, GABA), member 1	232333	https://www.ncbi.nlm.nih.gov/gene/?term=232333
Abhd13	Abhd13	abhydrolase domain containing 13	68904	https://www.ncbi.nlm.nih.gov/gene/?term=68904
Ergic2	Ergic2	ERGIC and golgi 2	67456	https://www.ncbi.nlm.nih.gov/gene/?term=67456
Pp2d1	Pp2d1	protein phosphatase 2C-like domain containing 1	110332	https://www.ncbi.nlm.nih.gov/gene/?term=110332
Grp	Grp	gastrin releasing peptide	225642	https://www.ncbi.nlm.nih.gov/gene/?term=225642
Col27a1	Col27a1	collagen, type XXVII, alpha 1	373864	https://www.ncbi.nlm.nih.gov/gene/?term=373864
Pcolce2	Pcolce2	procollagen C-endopeptidase enhancer 2	76477	https://www.ncbi.nlm.nih.gov/gene/?term=76477
Glra1	Glra1	glycine receptor, alpha 1 subunit	14654	https://www.ncbi.nlm.nih.gov/gene/?term=14654

Lmbr1l	Lmbr1l	limb region 1 like	74775	https://www.ncbi.nlm.nih.gov/gene/?term=74775
Hivep1	Hivep1	human immunodeficiency virus type I enhancer binding protein 1	110521	https://www.ncbi.nlm.nih.gov/gene/?term=110521
Rhpn2	Rhpn2	rhophilin, Rho GTPase binding protein 2	52428	https://www.ncbi.nlm.nih.gov/gene/?term=52428
Exoc5	Exoc5	exocyst complex component 5	105504	https://www.ncbi.nlm.nih.gov/gene/?term=105504
Fmn2	Fmn2	formin 2	54418	https://www.ncbi.nlm.nih.gov/gene/?term=54418
Sh3pxd2a	Sh3pxd2a	SH3 and PX domains 2A	14218	https://www.ncbi.nlm.nih.gov/gene/?term=14218
Golga4	Golga4	golgi autoantigen, golgin subfamily a, 4	54214	https://www.ncbi.nlm.nih.gov/gene/?term=54214
Fancm	Fancm	Fanconi anemia, complementation group M	104806	https://www.ncbi.nlm.nih.gov/gene/?term=104806
Ttc9	Ttc9	tetratricopeptide repeat domain 9	69480	https://www.ncbi.nlm.nih.gov/gene/?term=69480

Biology Open • Supplementary information

Nefm	Nefm	neurofilament, medium polypeptide	18040	https://www.ncbi.nlm.nih.gov/gene/?term=18040
Arf1	Arf1	ADP-ribosylation factor 1	11840	https://www.ncbi.nlm.nih.gov/gene/?term=11840
Pkdcc	Pkdcc	protein kinase domain containing, cytoplasmic	106522	https://www.ncbi.nlm.nih.gov/gene/?term=106522
Tulp4	Tulp4	tubby like protein 4	68842	https://www.ncbi.nlm.nih.gov/gene/?term=68842
Ppp1r12c	Ppp1r12c	protein phosphatase 1, regulatory subunit 12C	232807	https://www.ncbi.nlm.nih.gov/gene/?term=232807
Wwp2	Wwp2	WW domain containing E3 ubiquitin protein ligase 2	66894	https://www.ncbi.nlm.nih.gov/gene/?term=66894
Cd69	Cd69	CD69 antigen	12515	https://www.ncbi.nlm.nih.gov/gene/?term=12515
Sertad3	Sertad3	SERTA domain containing 3	170742	https://www.ncbi.nlm.nih.gov/gene/?term=170742
Dmxl1	Dmxl1	Dmx-like 1	240283	https://www.ncbi.nlm.nih.gov/gene/?term=240283

Btg2	Btg2	B cell translocation gene 2, anti-proliferative	12227	https://www.ncbi.nlm.nih.gov/gene/?term=12227
Tent4a	Tent4a	terminal nucleotidyltransferase 4A	210106	https://www.ncbi.nlm.nih.gov/gene/?term=210106
Slc12a5	Slc12a5	solute carrier family 12, member 5	57138	https://www.ncbi.nlm.nih.gov/gene/?term=57138
Dnajb9	Dnajb9	DnaJ heat shock protein family (Hsp40) member B9	27362	https://www.ncbi.nlm.nih.gov/gene/?term=27362
Gfpt2	Gfpt2	glutamine fructose-6-phosphate transaminase 2	14584	https://www.ncbi.nlm.nih.gov/gene/?term=14584
Cux1	Cux1	cut-like homeobox 1	13047	https://www.ncbi.nlm.nih.gov/gene/?term=13047
Slx4	Slx4	SLX4 structure-specific endonuclease subunit homolog (S. cerevisiae)	52864	https://www.ncbi.nlm.nih.gov/gene/?term=52864
Adam23	Adam23	a disintegrin and metallopeptidase domain 23	23792	https://www.ncbi.nlm.nih.gov/gene/?term=23792
Tgif1	Tgif1	TGFB-induced factor homeobox 1	21815	https://www.ncbi.nlm.nih.gov/gene/?term=21815

A830018L16Rik	A830018L16Rik	RIKEN cDNA A830018L16 gene	320492	https://www.ncbi.nlm.nih.gov/gene/?term=320492
Gata2	Gata2	GATA binding protein 2	14461	https://www.ncbi.nlm.nih.gov/gene/?term=14461
Bcat2	Bcat2	branched chain aminotransferase 2, mitochondrial	12036	https://www.ncbi.nlm.nih.gov/gene/?term=12036
Cldn11	Cldn11	claudin 11	18417	https://www.ncbi.nlm.nih.gov/gene/?term=18417
Grip2	Grip2	glutamate receptor interacting protein 2	243547	https://www.ncbi.nlm.nih.gov/gene/?term=243547
Ppcs	Ppcs	phosphopantothenoylcysteine synthetase	106564	https://www.ncbi.nlm.nih.gov/gene/?term=106564
Ptar1	Ptar1	protein prenyltransferase alpha subunit repeat containing 1	72351	https://www.ncbi.nlm.nih.gov/gene/?term=72351
Pcgf3	Pcgf3	polycomb group ring finger 3	69587	https://www.ncbi.nlm.nih.gov/gene/?term=69587
Glce	Glce	glucuronyl C5-epimerase	93683	https://www.ncbi.nlm.nih.gov/gene/?term=93683

Biology Open • Supplementary information

Usp28	Usp28	ubiquitin specific peptidase 28	235323	https://www.ncbi.nlm.nih.gov/gene/?term=235323
Sox11	Sox11	SRY (sex determining region Y)-box 11	20666	https://www.ncbi.nlm.nih.gov/gene/?term=20666
Syt3	Syt3	synaptotagmin III	20981	https://www.ncbi.nlm.nih.gov/gene/?term=20981
Cadm1	Cadm1	cell adhesion molecule 1	54725	https://www.ncbi.nlm.nih.gov/gene/?term=54725
Lpgat1	Lpgat1	lysophosphatidylglycerol acyltransferase 1	226856	https://www.ncbi.nlm.nih.gov/gene/?term=226856
Cnksr2	Cnksr2	connector enhancer of kinase suppressor of Ras 2	245684	https://www.ncbi.nlm.nih.gov/gene/?term=245684
Vgll1	Vgll1	vestigial like family member 1	170828	https://www.ncbi.nlm.nih.gov/gene/?term=170828
4930519G04Rik	4930519G04Rik	RIKEN cDNA 4930519G04 gene	67593	https://www.ncbi.nlm.nih.gov/gene/?term=67593
Naip1	Naip1	NLR family, apoptosis inhibitory protein 1	17940	https://www.ncbi.nlm.nih.gov/gene/?term=17940

Biology Open • Supplementary information

Rfx4	Rfx4	regulatory factor X, 4 (influences HLA class II expression)	71137	https://www.ncbi.nlm.nih.gov/gene/?term=71137
Epb41l3	Epb41l3	erythrocyte membrane protein band 4.1 like 3	13823	https://www.ncbi.nlm.nih.gov/gene/?term=13823
Chic2	Chic2	cysteine-rich hydrophobic domain 2	74277	https://www.ncbi.nlm.nih.gov/gene/?term=74277
Mcm8	Mcm8	minichromosome maintenance 8 homologous recombination repair factor	66634	https://www.ncbi.nlm.nih.gov/gene/?term=66634
Celsr3	Celsr3	cadherin, EGF LAG seven-pass G-type receptor 3	107934	https://www.ncbi.nlm.nih.gov/gene/?term=107934
Creb5	Creb5	cAMP responsive element binding protein 5	231991	https://www.ncbi.nlm.nih.gov/gene/?term=231991
Adrb2	Adrb2	adrenergic receptor, beta 2	11555	https://www.ncbi.nlm.nih.gov/gene/?term=11555
Plekhm3	Plekhm3	pleckstrin homology domain containing, family M, member 3	241075	https://www.ncbi.nlm.nih.gov/gene/?term=241075
Smarca1	Smarca1	SWI/SNF related, matrix associated, actin dependent regulator of chromatin, subfamily a, member 1	93761	https://www.ncbi.nlm.nih.gov/gene/?term=93761

Biology Open • Supplementary information

Thbs1	Thbs1	thrombospondin 1	21825	https://www.ncbi.nlm.nih.gov/gene/?term=21825
Lair1	Lair1	leukocyte-associated Ig-like receptor 1	52855	https://www.ncbi.nlm.nih.gov/gene/?term=52855
Vps35	Vps35	VPS35 retromer complex component	65114	https://www.ncbi.nlm.nih.gov/gene/?term=65114
Card11	Card11	caspase recruitment domain family, member 11	108723	https://www.ncbi.nlm.nih.gov/gene/?term=108723
Bmi1	Bmi1	Bmi1 polycomb ring finger oncogene	12151	https://www.ncbi.nlm.nih.gov/gene/?term=12151
Mfsd14a	Mfsd14a	major facilitator superfamily domain containing 14A	15247	https://www.ncbi.nlm.nih.gov/gene/?term=15247
Slc30a5	Slc30a5	solute carrier family 30 (zinc transporter), member 5	69048	https://www.ncbi.nlm.nih.gov/gene/?term=69048
Scrg1	Scrg1	scrapie responsive gene 1	20284	https://www.ncbi.nlm.nih.gov/gene/?term=20284
Arl8b	Arl8b	ADP-ribosylation factor-like 8B	67166	https://www.ncbi.nlm.nih.gov/gene/?term=67166

Biology Open • Supplementary information

Gnpnat1	Gnpnat1	glucosamine-phosphate N-acetyltransferase 1	54342	https://www.ncbi.nlm.nih.gov/gene/?term=54342
Rab18	Rab18	RAB18, member RAS oncogene family	19330	https://www.ncbi.nlm.nih.gov/gene/?term=19330
Sbno1	Sbno1	strawberry notch 1	243272	https://www.ncbi.nlm.nih.gov/gene/?term=243272
Il13ra1	Il13ra1	interleukin 13 receptor, alpha 1	16164	https://www.ncbi.nlm.nih.gov/gene/?term=16164
Senp6	Senp6	SUMO/sentrin specific peptidase 6	215351	https://www.ncbi.nlm.nih.gov/gene/?term=215351
Nck2	Nck2	non-catalytic region of tyrosine kinase adaptor protein 2	17974	https://www.ncbi.nlm.nih.gov/gene/?term=17974
Kcnma1	Kcnma1	potassium large conductance calcium-activated channel, subfamily M, alpha member 1	16531	https://www.ncbi.nlm.nih.gov/gene/?term=16531
Igsf11	Igsf11	immunoglobulin superfamily, member 11	207683	https://www.ncbi.nlm.nih.gov/gene/?term=207683
Smap1	Smap1	small ArfGAP 1	98366	https://www.ncbi.nlm.nih.gov/gene/?term=98366

Biology Open • Supplementary information

Cebpzos	Cebpzos	CCAAT/enhancer binding protein (C/EBP), zeta, opposite strand	68554	https://www.ncbi.nlm.nih.gov/gene/?term=68554
Csmd3	Csmd3	CUB and Sushi multiple domains 3	239420	https://www.ncbi.nlm.nih.gov/gene/?term=239420
Cmtr2	Cmtr2	cap methyltransferase 2	234728	https://www.ncbi.nlm.nih.gov/gene/?term=234728
Pi4k2a	Pi4k2a	phosphatidylinositol 4-kinase type 2 alpha	84095	https://www.ncbi.nlm.nih.gov/gene/?term=84095
Hipk2	Hipk2	homeodomain interacting protein kinase 2	15258	https://www.ncbi.nlm.nih.gov/gene/?term=15258
Slc25a25	Slc25a25	solute carrier family 25 (mitochondrial carrier, phosphate carrier), member 25	227731	https://www.ncbi.nlm.nih.gov/gene/?term=227731
Rora	Rora	RAR-related orphan receptor alpha	19883	https://www.ncbi.nlm.nih.gov/gene/?term=19883
Rgs2	Rgs2	regulator of G-protein signaling 2	19735	https://www.ncbi.nlm.nih.gov/gene/?term=19735
Rgn	Rgn	regucalcin	19733	https://www.ncbi.nlm.nih.gov/gene/?term=19733

Abca1	Abca1	ATP-binding cassette, sub-family A (ABC1), member 1	11303	https://www.ncbi.nlm.nih.gov/gene/?term=11303
Grm8	Grm8	glutamate receptor, metabotropic 8	14823	https://www.ncbi.nlm.nih.gov/gene/?term=14823
Gpcpd1	Gpcpd1	glycerophosphocholine phosphodiesterase 1	74182	https://www.ncbi.nlm.nih.gov/gene/?term=74182
Ankrd28	Ankrd28	ankyrin repeat domain 28	105522	https://www.ncbi.nlm.nih.gov/gene/?term=105522
Zfp281	Zfp281	zinc finger protein 281	226442	https://www.ncbi.nlm.nih.gov/gene/?term=226442
Pim3	Pim3	proviral integration site 3	223775	https://www.ncbi.nlm.nih.gov/gene/?term=223775
Ttll7	Ttll7	tubulin tyrosine ligase-like family, member 7	70892	https://www.ncbi.nlm.nih.gov/gene/?term=70892
Ywhah	Ywhah	tyrosine 3-monooxygenase/tryptophan 5-monooxygenase activation protein, eta polypeptide	22629	https://www.ncbi.nlm.nih.gov/gene/?term=22629
Rb1cc1	Rb1cc1	RB1-inducible coiled-coil 1	12421	https://www.ncbi.nlm.nih.gov/gene/?term=12421

Biology Open • Supplementary information

Tmem86a	Tmem86a	transmembrane protein 86A	67893	https://www.ncbi.nlm.nih.gov/gene/?term=67893
2410002F23Rik	2410002F23Rik	RIKEN cDNA 2410002F23 gene	668661	https://www.ncbi.nlm.nih.gov/gene/?term=668661
Arid5b	Arid5b	AT rich interactive domain 5B (MRF1-like)	71371	https://www.ncbi.nlm.nih.gov/gene/?term=71371
Cdk6	Cdk6	cyclin-dependent kinase 6	12571	https://www.ncbi.nlm.nih.gov/gene/?term=12571
Hadhb	Hadhb	hydroxyacyl-Coenzyme A dehydrogenase/3-ketoacyl-Coenzyme A thiolase/enoyl-Coenzyme A hydratase (trifunctional protein), beta subunit	231086	https://www.ncbi.nlm.nih.gov/gene/?term=231086
Map3k3	Map3k3	mitogen-activated protein kinase kinase kinase 3	26406	https://www.ncbi.nlm.nih.gov/gene/?term=26406
Gripap1	Gripap1	GRIP1 associated protein 1	54645	https://www.ncbi.nlm.nih.gov/gene/?term=54645
Rreb1	Rreb1	ras responsive element binding protein 1	68750	https://www.ncbi.nlm.nih.gov/gene/?term=68750

Biology Open • Supplementary information

Eml2	Eml2	echinoderm microtubule associated protein like 2	72205	https://www.ncbi.nlm.nih.gov/gene/?term=72205
Prkar1a	Prkar1a	protein kinase, cAMP dependent regulatory, type I, alpha	19084	https://www.ncbi.nlm.nih.gov/gene/?term=19084
Hif3a	Hif3a	hypoxia inducible factor 3, alpha subunit	53417	https://www.ncbi.nlm.nih.gov/gene/?term=53417
Zfp235	Zfp235	zinc finger protein 235	56525	https://www.ncbi.nlm.nih.gov/gene/?term=56525
Zfp788	Zfp788	zinc finger protein 788	67607	https://www.ncbi.nlm.nih.gov/gene/?term=67607
Zadh2	Zadh2	zinc binding alcohol dehydrogenase, domain containing 2	225791	https://www.ncbi.nlm.nih.gov/gene/?term=225791
Myb	Myb	myeloblastosis oncogene	17863	https://www.ncbi.nlm.nih.gov/gene/?term=17863
Tardbp	Tardbp	TAR DNA binding protein	230908	https://www.ncbi.nlm.nih.gov/gene/?term=230908
Frrs1l	Frrs1l	ferric-chelate reductase 1 like	230235	https://www.ncbi.nlm.nih.gov/gene/?term=230235

Biology Open • Supplementary information

Fbxo21	Fbxo21	F-box protein 21	231670	https://www.ncbi.nlm.nih.gov/gene/?term=231670
Pom121	Pom121	nuclear pore membrane protein 121	107939	https://www.ncbi.nlm.nih.gov/gene/?term=107939
Myh1	Myh1	myosin, heavy polypeptide 1, skeletal muscle, adult	17879	https://www.ncbi.nlm.nih.gov/gene/?term=17879
Tfip11	Tfip11	tuftelin interacting protein 11	54723	https://www.ncbi.nlm.nih.gov/gene/?term=54723
Pik3r1	Pik3r1	phosphoinositide-3-kinase regulatory subunit 1	18708	https://www.ncbi.nlm.nih.gov/gene/?term=18708
Ephb2	Ephb2	Eph receptor B2	13844	https://www.ncbi.nlm.nih.gov/gene/?term=13844
Pappa	Pappa	pregnancy-associated plasma protein A	18491	https://www.ncbi.nlm.nih.gov/gene/?term=18491
Amer1	Amer1	APC membrane recruitment 1	72345	https://www.ncbi.nlm.nih.gov/gene/?term=72345
Ssr3	Ssr3	signal sequence receptor, gamma	67437	https://www.ncbi.nlm.nih.gov/gene/?term=67437

Biology Open • Supplementary information

Pign	Pign	phosphatidylinositol glycan anchor biosynthesis, class N	27392	https://www.ncbi.nlm.nih.gov/gene/?term=27392
Cip2a	Cip2a	cell proliferation regulating inhibitor of protein phosphatase 2A	224171	https://www.ncbi.nlm.nih.gov/gene/?term=224171
Them4	Them4	thioesterase superfamily member 4	75778	https://www.ncbi.nlm.nih.gov/gene/?term=75778
Adipor2	Adipor2	adiponectin receptor 2	68465	https://www.ncbi.nlm.nih.gov/gene/?term=68465
Ptx3	Ptx3	pentraxin related gene	19288	https://www.ncbi.nlm.nih.gov/gene/?term=19288
Arid3a	Arid3a	AT rich interactive domain 3A (BRIGHT-like)	13496	https://www.ncbi.nlm.nih.gov/gene/?term=13496
Syvn1	Syvn1	synovial apoptosis inhibitor 1, synoviolin	74126	https://www.ncbi.nlm.nih.gov/gene/?term=74126
Irf4	Irf4	interferon regulatory factor 4	16364	https://www.ncbi.nlm.nih.gov/gene/?term=16364
Vdr	Vdr	vitamin D (1,25-dihydroxyvitamin D3) receptor	22337	https://www.ncbi.nlm.nih.gov/gene/?term=22337

Fam83h	Fam83h	family with sequence similarity 83, member H	105732	https://www.ncbi.nlm.nih.gov/gene/?term=105732
Nipal4	Nipal4	NIPA-like domain containing 4	214112	https://www.ncbi.nlm.nih.gov/gene/?term=214112
Sstr3	Sstr3	somatostatin receptor 3	20607	https://www.ncbi.nlm.nih.gov/gene/?term=20607
Crb2	Crb2	crumbs family member 2	241324	https://www.ncbi.nlm.nih.gov/gene/?term=241324
Nup210	Nup210	nucleoporin 210	54563	https://www.ncbi.nlm.nih.gov/gene/?term=54563
Kcns3	Kcns3	potassium voltage-gated channel, delayed-rectifier, subfamily S, member 3	238076	https://www.ncbi.nlm.nih.gov/gene/?term=238076
Sema4b	Sema4b	sema domain, immunoglobulin domain (Ig), transmembrane domain (TM) and short cytoplasmic domain, (semaphorin) 4B	20352	https://www.ncbi.nlm.nih.gov/gene/?term=20352
Was	Was	Wiskott-Aldrich syndrome	22376	https://www.ncbi.nlm.nih.gov/gene/?term=22376
Lin28a	Lin28a	lin-28 homolog A (C. elegans)	83557	https://www.ncbi.nlm.nih.gov/gene/?term=83557

Biology Open • Supplementary information

Abhd6	Abhd6	abhydrolase domain containing 6	66082	https://www.ncbi.nlm.nih.gov/gene/?term=66082
AU040320	AU040320	expressed sequence AU040320	100317	https://www.ncbi.nlm.nih.gov/gene/?term=100317
Scn2b	Scn2b	sodium channel, voltage-gated, type II, beta	72821	https://www.ncbi.nlm.nih.gov/gene/?term=72821
Enpep	Enpep	glutamyl aminopeptidase	13809	https://www.ncbi.nlm.nih.gov/gene/?term=13809
Zswim5	Zswim5	zinc finger SWIM-type containing 5	74464	https://www.ncbi.nlm.nih.gov/gene/?term=74464
Ptpn1	Ptpn1	protein tyrosine phosphatase, non-receptor type 1	19246	https://www.ncbi.nlm.nih.gov/gene/?term=19246
Dhx33	Dhx33	DEAH (Asp-Glu-Ala-His) box polypeptide 33	216877	https://www.ncbi.nlm.nih.gov/gene/?term=216877
Sarm1	Sarm1	sterile alpha and HEAT/Armadillo motif containing 1	237868	https://www.ncbi.nlm.nih.gov/gene/?term=237868
Zscan29	Zscan29	zinc finger SCAN domains 29	99334	https://www.ncbi.nlm.nih.gov/gene/?term=99334

Biology Open • Supplementary information

Nbeal2	Nbeal2	neurobeachin-like 2	235627	https://www.ncbi.nlm.nih.gov/gene/?term=235627
Zfp523	Zfp523	zinc finger protein 523	224656	https://www.ncbi.nlm.nih.gov/gene/?term=224656
Stard13	Stard13	StAR-related lipid transfer (START) domain containing 13	243362	https://www.ncbi.nlm.nih.gov/gene/?term=243362
Pcgf6	Pcgf6	polycomb group ring finger 6	71041	https://www.ncbi.nlm.nih.gov/gene/?term=71041
Ier3ip1	Ier3ip1	immediate early response 3 interacting protein 1	66191	https://www.ncbi.nlm.nih.gov/gene/?term=66191
Lfng	Lfng	LFNG O-fucosylpeptide 3-beta-N-acetylglucosaminyltransferase	16848	https://www.ncbi.nlm.nih.gov/gene/?term=16848
Crem	Crem	cAMP responsive element modulator	12916	https://www.ncbi.nlm.nih.gov/gene/?term=12916
Klf13	Klf13	Kruppel-like factor 13	50794	https://www.ncbi.nlm.nih.gov/gene/?term=50794
E2f2	E2f2	E2F transcription factor 2	242705	https://www.ncbi.nlm.nih.gov/gene/?term=242705

Biology Open • Supplementary information

Nrm	Nrm	nurim (nuclear envelope membrane protein)	106582	https://www.ncbi.nlm.nih.gov/gene/?term=106582
Ovol1	Ovol1	ovo like zinc finger 1	18426	https://www.ncbi.nlm.nih.gov/gene/?term=18426
Osbpl9	Osbpl9	oxysterol binding protein-like 9	100273	https://www.ncbi.nlm.nih.gov/gene/?term=100273
Vps4b	Vps4b	vacuolar protein sorting 4B	20479	https://www.ncbi.nlm.nih.gov/gene/?term=20479
Bet1	Bet1	Bet1 golgi vesicular membrane trafficking protein	12068	https://www.ncbi.nlm.nih.gov/gene/?term=12068
Taz	Taz	tafazzin	66826	https://www.ncbi.nlm.nih.gov/gene/?term=66826
Kcnk10	Kcnk10	potassium channel, subfamily K, member 10	72258	https://www.ncbi.nlm.nih.gov/gene/?term=72258
Sgpl1	Sgpl1	sphingosine phosphate lyase 1	20397	https://www.ncbi.nlm.nih.gov/gene/?term=20397
Nkapd1	Nkapd1	NKAP domain containing 1	270156	https://www.ncbi.nlm.nih.gov/gene/?term=270156

Biology Open • Supplementary information

2610528J11Rik	2610528J11Rik	RIKEN cDNA 2610528J11 gene	66451	https://www.ncbi.nlm.nih.gov/gene/?term=66451
Samd10	Samd10	sterile alpha motif domain containing 10	229011	https://www.ncbi.nlm.nih.gov/gene/?term=229011
Npl	Npl	N-acetylneuraminate pyruvate lyase	74091	https://www.ncbi.nlm.nih.gov/gene/?term=74091
Slc6a17	Slc6a17	solute carrier family 6 (neurotransmitter transporter), member 17	229706	https://www.ncbi.nlm.nih.gov/gene/?term=229706
Sema4d	Sema4d	sema domain, immunoglobulin domain (Ig), transmembrane domain (TM) and short cytoplasmic domain, (semaphorin) 4D	20354	https://www.ncbi.nlm.nih.gov/gene/?term=20354
Msrb3	Msrb3	methionine sulfoxide reductase B3	320183	https://www.ncbi.nlm.nih.gov/gene/?term=320183
Slc35a4	Slc35a4	solute carrier family 35, member A4	67843	https://www.ncbi.nlm.nih.gov/gene/?term=67843
Kmt5c	Kmt5c	lysine methyltransferase 5C	232811	https://www.ncbi.nlm.nih.gov/gene/?term=232811
Gga2	Gga2	golgi associated, gamma adaptin ear containing, ARF binding protein 2	74105	https://www.ncbi.nlm.nih.gov/gene/?term=74105

Biology Open • Supplementary information

Cgn	Cgn	cingulin	70737	https://www.ncbi.nlm.nih.gov/gene/?term=70737
Triap1	Triap1	TP53 regulated inhibitor of apoptosis 1	69076	https://www.ncbi.nlm.nih.gov/gene/?term=69076
Tmem161b	Tmem161b	transmembrane protein 161B	72745	https://www.ncbi.nlm.nih.gov/gene/?term=72745
Blzf1	Blzf1	basic leucine zipper nuclear factor 1	66352	https://www.ncbi.nlm.nih.gov/gene/?term=66352
Acer2	Acer2	alkaline ceramidase 2	230379	https://www.ncbi.nlm.nih.gov/gene/?term=230379
Tmem120b	Tmem120b	transmembrane protein 120B	330189	https://www.ncbi.nlm.nih.gov/gene/?term=330189
Slc25a35	Slc25a35	solute carrier family 25, member 35	71998	https://www.ncbi.nlm.nih.gov/gene/?term=71998
Pafah1b1	Pafah1b1	platelet-activating factor acetylhydrolase, isoform 1b, subunit 1	18472	https://www.ncbi.nlm.nih.gov/gene/?term=18472
Rasgef1a	Rasgef1a	RasGEF domain family, member 1A	70727	https://www.ncbi.nlm.nih.gov/gene/?term=70727

Biology Open • Supplementary information

Grsf1	Grsf1	G-rich RNA sequence binding factor 1	231413	https://www.ncbi.nlm.nih.gov/gene/?term=231413
Tspan12	Tspan12	tetraspanin 12	269831	https://www.ncbi.nlm.nih.gov/gene/?term=269831
Mcl1	Mcl1	myeloid cell leukemia sequence 1	17210	https://www.ncbi.nlm.nih.gov/gene/?term=17210
Ubr7	Ubr7	ubiquitin protein ligase E3 component n-recognin 7 (putative)	66622	https://www.ncbi.nlm.nih.gov/gene/?term=66622
Mapre2	Mapre2	microtubule-associated protein, RP/EB family, member 2	212307	https://www.ncbi.nlm.nih.gov/gene/?term=212307
Zswim6	Zswim6	zinc finger SWIM-type containing 6	67263	https://www.ncbi.nlm.nih.gov/gene/?term=67263
Rbm20	Rbm20	RNA binding motif protein 20	73713	https://www.ncbi.nlm.nih.gov/gene/?term=73713
Bmf	Bmf	BCL2 modifying factor	171543	https://www.ncbi.nlm.nih.gov/gene/?term=171543
Xirp1	Xirp1	xin actin-binding repeat containing 1	22437	https://www.ncbi.nlm.nih.gov/gene/?term=22437

Biology Open • Supplementary information

Necab3	Necab3	N-terminal EF-hand calcium binding protein 3	56846	https://www.ncbi.nlm.nih.gov/gene/?term=56846
Diras1	Diras1	DIRAS family, GTP-binding RAS-like 1	208666	https://www.ncbi.nlm.nih.gov/gene/?term=208666
Ier2	Ier2	immediate early response 2	15936	https://www.ncbi.nlm.nih.gov/gene/?term=15936
Zfp518a	Zfp518a	zinc finger protein 518A	72672	https://www.ncbi.nlm.nih.gov/gene/?term=72672
Sh3bp5l	Sh3bp5l	SH3 binding domain protein 5 like	79566	https://www.ncbi.nlm.nih.gov/gene/?term=79566
Atp11a	Atp11a	ATPase, class VI, type 11A	50770	https://www.ncbi.nlm.nih.gov/gene/?term=50770
Dus1l	Dus1l	dihydrouridine synthase 1-like (S. cerevisiae)	68730	https://www.ncbi.nlm.nih.gov/gene/?term=68730
Eif1ad	Eif1ad	eukaryotic translation initiation factor 1A domain containing	69860	https://www.ncbi.nlm.nih.gov/gene/?term=69860
Cdc42se1	Cdc42se1	CDC42 small effector 1	57912	https://www.ncbi.nlm.nih.gov/gene/?term=57912

Biology Open • Supplementary information

Sec14l2	Sec14l2	SEC14-like lipid binding 2	67815	https://www.ncbi.nlm.nih.gov/gene/?term=67815
Sh3tc2	Sh3tc2	SH3 domain and tetratricopeptide repeats 2	225608	https://www.ncbi.nlm.nih.gov/gene/?term=225608
Tmprss13	Tmprss13	transmembrane protease, serine 13	214531	https://www.ncbi.nlm.nih.gov/gene/?term=214531
Cdc42bpg	Cdc42bpg	CDC42 binding protein kinase gamma (DMPK-like)	240505	https://www.ncbi.nlm.nih.gov/gene/?term=240505
Zfp488	Zfp488	zinc finger protein 488	382867	https://www.ncbi.nlm.nih.gov/gene/?term=382867
Tnfsf4	Tnfsf4	tumor necrosis factor (ligand) superfamily, member 4	22164	https://www.ncbi.nlm.nih.gov/gene/?term=22164
Mamdc2	Mamdc2	MAM domain containing 2	71738	https://www.ncbi.nlm.nih.gov/gene/?term=71738
Mfsd9	Mfsd9	major facilitator superfamily domain containing 9	211798	https://www.ncbi.nlm.nih.gov/gene/?term=211798
Sptb	Sptb	spectrin beta, erythrocytic	20741	https://www.ncbi.nlm.nih.gov/gene/?term=20741

Biology Open • Supplementary information

Ccnj	Ccnj	cyclin J	240665	https://www.ncbi.nlm.nih.gov/gene/?term=240665
Ptpn7	Ptpn7	protein tyrosine phosphatase, non-receptor type 7	320139	https://www.ncbi.nlm.nih.gov/gene/?term=320139
Arid3b	Arid3b	AT rich interactive domain 3B (BRIGHT-like)	56380	https://www.ncbi.nlm.nih.gov/gene/?term=56380
Lrp4	Lrp4	low density lipoprotein receptor-related protein 4	228357	https://www.ncbi.nlm.nih.gov/gene/?term=228357
Ncan	Ncan	neurocan	13004	https://www.ncbi.nlm.nih.gov/gene/?term=13004
Cyp24a1	Cyp24a1	cytochrome P450, family 24, subfamily a, polypeptide 1	13081	https://www.ncbi.nlm.nih.gov/gene/?term=13081
Lactb	Lactb	lactamase, beta	80907	https://www.ncbi.nlm.nih.gov/gene/?term=80907
Dtx4	Dtx4	deltex 4, E3 ubiquitin ligase	207521	https://www.ncbi.nlm.nih.gov/gene/?term=207521
Prdm1	Prdm1	PR domain containing 1, with ZNF domain	12142	https://www.ncbi.nlm.nih.gov/gene/?term=12142

Biology Open • Supplementary information

Gcnt1	Gcnt1	glucosaminyl (N-acetyl) transferase 1, core 2	14537	https://www.ncbi.nlm.nih.gov/gene/?term=14537
Dyrk1b	Dyrk1b	dual-specificity tyrosine-(Y)-phosphorylation regulated kinase 1b	13549	https://www.ncbi.nlm.nih.gov/gene/?term=13549
Dyrk1a	Dyrk1a	dual-specificity tyrosine-(Y)-phosphorylation regulated kinase 1a	13548	https://www.ncbi.nlm.nih.gov/gene/?term=13548
Zfp42	Zfp42	zinc finger protein 42	22702	https://www.ncbi.nlm.nih.gov/gene/?term=22702
Pdgfrb	Pdgfrb	platelet derived growth factor receptor, beta polypeptide	18596	https://www.ncbi.nlm.nih.gov/gene/?term=18596
Prkaa2	Prkaa2	protein kinase, AMP-activated, alpha 2 catalytic subunit	108079	https://www.ncbi.nlm.nih.gov/gene/?term=108079
Pik3r3	Pik3r3	phosphoinositide-3-kinase regulatory subunit 3	18710	https://www.ncbi.nlm.nih.gov/gene/?term=18710
Tmem179b	Tmem179b	transmembrane protein 179B	67706	https://www.ncbi.nlm.nih.gov/gene/?term=67706
Car12	Car12	carbonic anhydrase 12	76459	https://www.ncbi.nlm.nih.gov/gene/?term=76459

Adam12	Adam12	a disintegrin and metallopeptidase domain 12 (meltrin alpha)	11489	https://www.ncbi.nlm.nih.gov/gene/?term=11489
Zfp930	Zfp930	zinc finger protein 930	234358	https://www.ncbi.nlm.nih.gov/gene/?term=234358
Scn3a	Scn3a	sodium channel, voltage-gated, type III, alpha	20269	https://www.ncbi.nlm.nih.gov/gene/?term=20269
Gpr155	Gpr155	G protein-coupled receptor 155	68526	https://www.ncbi.nlm.nih.gov/gene/?term=68526
Zfp217	Zfp217	zinc finger protein 217	228913	https://www.ncbi.nlm.nih.gov/gene/?term=228913
Arhgap6	Arhgap6	Rho GTPase activating protein 6	11856	https://www.ncbi.nlm.nih.gov/gene/?term=11856
Magohb	Magohb	mago homolog B, exon junction complex core component	66441	https://www.ncbi.nlm.nih.gov/gene/?term=66441
Copb1	Copb1	coatomer protein complex, subunit beta 1	70349	https://www.ncbi.nlm.nih.gov/gene/?term=70349
Pnn	Pnn	pinin	18949	https://www.ncbi.nlm.nih.gov/gene/?term=18949

Biology Open • Supplementary information

Fth1	Fth1	ferritin heavy polypeptide 1	14319	https://www.ncbi.nlm.nih.gov/gene/?term=14319
Arhgef2	Arhgef2	rho/rac guanine nucleotide exchange factor (GEF) 2	16800	https://www.ncbi.nlm.nih.gov/gene/?term=16800
Tmem150a	Tmem150a	transmembrane protein 150A	232086	https://www.ncbi.nlm.nih.gov/gene/?term=232086
Zmym2	Zmym2	zinc finger, MYM-type 2	76007	https://www.ncbi.nlm.nih.gov/gene/?term=76007
Mboat1	Mboat1	membrane bound O-acyltransferase domain containing 1	218121	https://www.ncbi.nlm.nih.gov/gene/?term=218121
Cdh2	Cdh2	cadherin 2	12558	https://www.ncbi.nlm.nih.gov/gene/?term=12558
Phospho2	Phospho2	phosphatase, orphan 2	73373	https://www.ncbi.nlm.nih.gov/gene/?term=73373
Ptp4a1	Ptp4a1	protein tyrosine phosphatase 4a1	19243	https://www.ncbi.nlm.nih.gov/gene/?term=19243
Cramp1l	Cramp1l	cramped chromatin regulator homolog 1	57354	https://www.ncbi.nlm.nih.gov/gene/?term=57354

Biology Open • Supplementary information

Pex5l	Pex5l	peroxisomal biogenesis factor 5-like	58869	https://www.ncbi.nlm.nih.gov/gene/?term=58869
Srcin1	Srcin1	SRC kinase signaling inhibitor 1	56013	https://www.ncbi.nlm.nih.gov/gene/?term=56013
Gon4l	Gon4l	gon-4-like (C.elegans)	76022	https://www.ncbi.nlm.nih.gov/gene/?term=76022
St6galnac6	St6galnac6	ST6 (alpha-N-acetyl-neuraminyl-2,3-beta-galactosyl-1,3)-N-acetylgalactosaminide alpha-2,6-sialyltransferase 6	50935	https://www.ncbi.nlm.nih.gov/gene/?term=50935
Sqstm1	Sqstm1	sequestosome 1	18412	https://www.ncbi.nlm.nih.gov/gene/?term=18412
Stx6	Stx6	syntaxin 6	58244	https://www.ncbi.nlm.nih.gov/gene/?term=58244
Atxn1l	Atxn1l	ataxin 1-like	52335	https://www.ncbi.nlm.nih.gov/gene/?term=52335
Zfp827	Zfp827	zinc finger protein 827	622675	https://www.ncbi.nlm.nih.gov/gene/?term=622675
Tmem127	Tmem127	transmembrane protein 127	69470	https://www.ncbi.nlm.nih.gov/gene/?term=69470

Egln3	Egln3	egl-9 family hypoxia-inducible factor 3	112407	https://www.ncbi.nlm.nih.gov/gene/?term=112407
Ldlrap1	Ldlrap1	low density lipoprotein receptor adaptor protein 1	100017	https://www.ncbi.nlm.nih.gov/gene/?term=100017
Ankrd17	Ankrd17	ankyrin repeat domain 17	81702	https://www.ncbi.nlm.nih.gov/gene/?term=81702
Pkd2	Pkd2	polycystic kidney disease 2	18764	https://www.ncbi.nlm.nih.gov/gene/?term=18764
Mkrn1	Mkrn1	makorin, ring finger protein, 1	54484	https://www.ncbi.nlm.nih.gov/gene/?term=54484
Trip11	Trip11	thyroid hormone receptor interactor 11	109181	https://www.ncbi.nlm.nih.gov/gene/?term=109181
1600012H06Rik	1600012H06Rik	RIKEN cDNA 1600012H06 gene	67912	https://www.ncbi.nlm.nih.gov/gene/?term=67912
Tgfbr2	Tgfbr2	transforming growth factor, beta receptor II	21813	https://www.ncbi.nlm.nih.gov/gene/?term=21813
Heg1	Heg1	heart development protein with EGF-like domains 1	77446	https://www.ncbi.nlm.nih.gov/gene/?term=77446

Mtmr3	Mtmr3	myotubularin related protein 3	74302	https://www.ncbi.nlm.nih.gov/gene/?term=74302
Nrip3	Nrip3	nuclear receptor interacting protein 3	78593	https://www.ncbi.nlm.nih.gov/gene/?term=78593
Zc3h12c	Zc3h12c	zinc finger CCCH type containing 12C	244871	https://www.ncbi.nlm.nih.gov/gene/?term=244871
Lypd6	Lypd6	LY6/PLAUR domain containing 6	320343	https://www.ncbi.nlm.nih.gov/gene/?term=320343
Irf9	Irf9	interferon regulatory factor 9	16391	https://www.ncbi.nlm.nih.gov/gene/?term=16391
Spopl	Spopl	speckle-type POZ protein-like	76857	https://www.ncbi.nlm.nih.gov/gene/?term=76857
Btg3	Btg3	B cell translocation gene 3	12228	https://www.ncbi.nlm.nih.gov/gene/?term=12228
Rasd1	Rasd1	RAS, dexamethasone-induced 1	19416	https://www.ncbi.nlm.nih.gov/gene/?term=19416
Znfx1	Znfx1	zinc finger, NFX1-type containing 1	98999	https://www.ncbi.nlm.nih.gov/gene/?term=98999

Biology Open • Supplementary information

Ptchd4	Ptchd4	patched domain containing 4	627626	https://www.ncbi.nlm.nih.gov/gene/?term=627626
Fyco1	Fyco1	FYVE and coiled-coil domain containing 1	17281	https://www.ncbi.nlm.nih.gov/gene/?term=17281
Afg1l	Afg1l	AFG1 like ATPase	215951	https://www.ncbi.nlm.nih.gov/gene/?term=215951
Pthlh	Pthlh	parathyroid hormone-like peptide	19227	https://www.ncbi.nlm.nih.gov/gene/?term=19227
Mink1	Mink1	misshapen-like kinase 1 (zebrafish)	50932	https://www.ncbi.nlm.nih.gov/gene/?term=50932
Sema7a	Sema7a	sema domain, immunoglobulin domain (Ig), and GPI membrane anchor, (semaphorin) 7A	20361	https://www.ncbi.nlm.nih.gov/gene/?term=20361
Pde3b	Pde3b	phosphodiesterase 3B, cGMP-inhibited	18576	https://www.ncbi.nlm.nih.gov/gene/?term=18576
Fzd3	Fzd3	frizzled class receptor 3	14365	https://www.ncbi.nlm.nih.gov/gene/?term=14365
Topors	Topors	topoisomerase I binding, arginine/serine-rich	106021	https://www.ncbi.nlm.nih.gov/gene/?term=106021

Biology Open • Supplementary information

Rufy2	Rufy2	RUN and FYVE domain-containing 2	70432	https://www.ncbi.nlm.nih.gov/gene/?term=70432
Reps2	Reps2	RALBP1 associated Eps domain containing protein 2	194590	https://www.ncbi.nlm.nih.gov/gene/?term=194590
Dmtf1	Dmtf1	cyclin D binding myb-like transcription factor 1	23857	https://www.ncbi.nlm.nih.gov/gene/?term=23857
Rp2	Rp2	retinitis pigmentosa 2 homolog	19889	https://www.ncbi.nlm.nih.gov/gene/?term=19889
Zfyve26	Zfyve26	zinc finger, FYVE domain containing 26	211978	https://www.ncbi.nlm.nih.gov/gene/?term=211978
Map3k14	Map3k14	mitogen-activated protein kinase kinase kinase 14	53859	https://www.ncbi.nlm.nih.gov/gene/?term=53859
Ulk1	Ulk1	unc-51 like kinase 1	22241	https://www.ncbi.nlm.nih.gov/gene/?term=22241
Kcnb1	Kcnb1	potassium voltage gated channel, Shab-related subfamily, member 1	16500	https://www.ncbi.nlm.nih.gov/gene/?term=16500
F3	F3	coagulation factor III	14066	https://www.ncbi.nlm.nih.gov/gene/?term=14066

Biology Open • Supplementary information

Pgm2l1	Pgm2l1	phosphoglucomutase 2-like 1	70974	https://www.ncbi.nlm.nih.gov/gene/?term=70974
Slc31a2	Slc31a2	solute carrier family 31, member 2	20530	https://www.ncbi.nlm.nih.gov/gene/?term=20530
Nagk	Nagk	N-acetylglucosamine kinase	56174	https://www.ncbi.nlm.nih.gov/gene/?term=56174
Usp3	Usp3	ubiquitin specific peptidase 3	235441	https://www.ncbi.nlm.nih.gov/gene/?term=235441
Sertad2	Sertad2	SERTA domain containing 2	58172	https://www.ncbi.nlm.nih.gov/gene/?term=58172
Zfp512b	Zfp512b	zinc finger protein 512B	269401	https://www.ncbi.nlm.nih.gov/gene/?term=269401
Tbc1d8b	Tbc1d8b	TBC1 domain family, member 8B	245638	https://www.ncbi.nlm.nih.gov/gene/?term=245638
Lrrc55	Lrrc55	leucine rich repeat containing 55	241528	https://www.ncbi.nlm.nih.gov/gene/?term=241528
Cast	Cast	calpastatin	12380	https://www.ncbi.nlm.nih.gov/gene/?term=12380

Biology Open • Supplementary information

Kmt2b	Kmt2b	lysine (K)-specific methyltransferase 2B	75410	https://www.ncbi.nlm.nih.gov/gene/?term=75410
Ism2	Ism2	isthmin 2	217738	https://www.ncbi.nlm.nih.gov/gene/?term=217738
Fcho2	Fcho2	FCH domain only 2	218503	https://www.ncbi.nlm.nih.gov/gene/?term=218503
Kif5a	Kif5a	kinesin family member 5A	16572	https://www.ncbi.nlm.nih.gov/gene/?term=16572
Ankrd9	Ankrd9	ankyrin repeat domain 9	74251	https://www.ncbi.nlm.nih.gov/gene/?term=74251
Col4a4	Col4a4	collagen, type IV, alpha 4	12829	https://www.ncbi.nlm.nih.gov/gene/?term=12829
Tnfrsf21	Tnfrsf21	tumor necrosis factor receptor superfamily, member 21	94185	https://www.ncbi.nlm.nih.gov/gene/?term=94185
Chrm2	Chrm2	cholinergic receptor, muscarinic 2, cardiac	243764	https://www.ncbi.nlm.nih.gov/gene/?term=243764
Tmem267	Tmem267	transmembrane protein 267	633640	https://www.ncbi.nlm.nih.gov/gene/?term=633640

Biology Open • Supplementary information

Polr3g	Polr3g	polymerase (RNA) III (DNA directed) polypeptide G	67486	https://www.ncbi.nlm.nih.gov/gene/?term=67486
Tmcc3	Tmcc3	transmembrane and coiled coil domains 3	319880	https://www.ncbi.nlm.nih.gov/gene/?term=319880
Armc8	Armc8	armadillo repeat containing 8	74125	https://www.ncbi.nlm.nih.gov/gene/?term=74125
Atg16l1	Atg16l1	autophagy related 16-like 1 (S. cerevisiae)	77040	https://www.ncbi.nlm.nih.gov/gene/?term=77040
Rasl11b	Rasl11b	RAS-like, family 11, member B	68939	https://www.ncbi.nlm.nih.gov/gene/?term=68939
Prrg1	Prrg1	proline rich Gla (G-carboxyglutamic acid) 1	546336	https://www.ncbi.nlm.nih.gov/gene/?term=546336
Ptpn4	Ptpn4	protein tyrosine phosphatase, non-receptor type 4	19258	https://www.ncbi.nlm.nih.gov/gene/?term=19258
Fbxl5	Fbxl5	F-box and leucine-rich repeat protein 5	242960	https://www.ncbi.nlm.nih.gov/gene/?term=242960
Vangl1	Vangl1	VANGL planar cell polarity 1	229658	https://www.ncbi.nlm.nih.gov/gene/?term=229658

Biology Open • Supplementary information

Rnf150	Rnf150	ring finger protein 150	330812	https://www.ncbi.nlm.nih.gov/gene/?term=330812
Sar1b	Sar1b	secretion associated Ras related GTPase 1B	66397	https://www.ncbi.nlm.nih.gov/gene/?term=66397
St3gal1	St3gal1	ST3 beta-galactoside alpha-2,3-sialyltransferase 1	20442	https://www.ncbi.nlm.nih.gov/gene/?term=20442
Ppp1r3e	Ppp1r3e	protein phosphatase 1, regulatory subunit 3E	105651	https://www.ncbi.nlm.nih.gov/gene/?term=105651
Rab22a	Rab22a	RAB22A, member RAS oncogene family	19334	https://www.ncbi.nlm.nih.gov/gene/?term=19334
Ddhd1	Ddhd1	DDHD domain containing 1	114874	https://www.ncbi.nlm.nih.gov/gene/?term=114874
Unk	Unk	unkempt family zinc finger	217331	https://www.ncbi.nlm.nih.gov/gene/?term=217331
Crybg3	Crybg3	beta-gamma crystallin domain containing 3	224273	https://www.ncbi.nlm.nih.gov/gene/?term=224273
Laptm4a	Laptm4a	lysosomal-associated protein transmembrane 4A	17775	https://www.ncbi.nlm.nih.gov/gene/?term=17775

Biology Open • Supplementary information

Pdcd1lg2	Pdcd1lg2	programmed cell death 1 ligand 2	58205	https://www.ncbi.nlm.nih.gov/gene/?term=58205
Slc25a40	Slc25a40	solute carrier family 25, member 40	319653	https://www.ncbi.nlm.nih.gov/gene/?term=319653
Rab8b	Rab8b	RAB8B, member RAS oncogene family	235442	https://www.ncbi.nlm.nih.gov/gene/?term=235442
Clip4	Clip4	CAP-GLY domain containing linker protein family, member 4	78785	https://www.ncbi.nlm.nih.gov/gene/?term=78785
Rhoc	Rhoc	ras homolog family member C	11853	https://www.ncbi.nlm.nih.gov/gene/?term=11853
Suco	Suco	SUN domain containing ossification factor	226551	https://www.ncbi.nlm.nih.gov/gene/?term=226551
Mastl	Mastl	microtubule associated serine/threonine kinase-like	67121	https://www.ncbi.nlm.nih.gov/gene/?term=67121
Arhgap1	Arhgap1	Rho GTPase activating protein 1	228359	https://www.ncbi.nlm.nih.gov/gene/?term=228359
Iqsec2	Iqsec2	IQ motif and Sec7 domain 2	245666	https://www.ncbi.nlm.nih.gov/gene/?term=245666

Biology Open • Supplementary information

Fat2	Fat2	FAT atypical cadherin 2	245827	https://www.ncbi.nlm.nih.gov/gene/?term=245827
Coro2b	Coro2b	coronin, actin binding protein, 2B	235431	https://www.ncbi.nlm.nih.gov/gene/?term=235431
Gab1	Gab1	growth factor receptor bound protein 2-associated protein 1	14388	https://www.ncbi.nlm.nih.gov/gene/?term=14388
Rbl2	Rbl2	RB transcriptional corepressor like 2	19651	https://www.ncbi.nlm.nih.gov/gene/?term=19651
Sash1	Sash1	SAM and SH3 domain containing 1	70097	https://www.ncbi.nlm.nih.gov/gene/?term=70097
Arhgef10	Arhgef10	Rho guanine nucleotide exchange factor (GEF) 10	234094	https://www.ncbi.nlm.nih.gov/gene/?term=234094
Slc24a2	Slc24a2	solute carrier family 24 (sodium/potassium/calcium exchanger), member 2	76376	https://www.ncbi.nlm.nih.gov/gene/?term=76376
Gosr1	Gosr1	golgi SNAP receptor complex member 1	53334	https://www.ncbi.nlm.nih.gov/gene/?term=53334
C2cd2	C2cd2	C2 calcium-dependent domain containing 2	207781	https://www.ncbi.nlm.nih.gov/gene/?term=207781

Biology Open • Supplementary information

E2f1	E2f1	E2F transcription factor 1	13555	https://www.ncbi.nlm.nih.gov/gene/?term=13555
B3galt2	B3galt2	UDP-Gal:betaGlcNAc beta 1,3-galactosyltransferase, polypeptide 2	26878	https://www.ncbi.nlm.nih.gov/gene/?term=26878
Map3k13	Map3k13	mitogen-activated protein kinase kinase kinase 13	71751	https://www.ncbi.nlm.nih.gov/gene/?term=71751
Lama3	Lama3	laminin, alpha 3	16774	https://www.ncbi.nlm.nih.gov/gene/?term=16774
Map3k12	Map3k12	mitogen-activated protein kinase kinase kinase 12	26404	https://www.ncbi.nlm.nih.gov/gene/?term=26404
Ankrd52	Ankrd52	ankyrin repeat domain 52	237615	https://www.ncbi.nlm.nih.gov/gene/?term=237615
Mapre3	Mapre3	microtubule-associated protein, RP/EB family, member 3	100732	https://www.ncbi.nlm.nih.gov/gene/?term=100732
Mex3d	Mex3d	mex3 RNA binding family member D	237400	https://www.ncbi.nlm.nih.gov/gene/?term=237400
Mfap3l	Mfap3l	microfibrillar-associated protein 3-like	71306	https://www.ncbi.nlm.nih.gov/gene/?term=71306

Biology Open • Supplementary information

Eif4a2	Eif4a2	eukaryotic translation initiation factor 4A2	13682	https://www.ncbi.nlm.nih.gov/gene/?term=13682
Susd6	Susd6	sushi domain containing 6	217684	https://www.ncbi.nlm.nih.gov/gene/?term=217684
Uri1	Uri1	URI1, prefoldin-like chaperone	19777	https://www.ncbi.nlm.nih.gov/gene/?term=19777
Nedd4l	Nedd4l	neural precursor cell expressed, developmentally down-regulated gene 4-like	83814	https://www.ncbi.nlm.nih.gov/gene/?term=83814
6430548M08Rik	6430548M08Rik	RIKEN cDNA 6430548M08 gene	234797	https://www.ncbi.nlm.nih.gov/gene/?term=234797
Tars2	Tars2	threonyl-tRNA synthetase 2, mitochondrial (putative)	71807	https://www.ncbi.nlm.nih.gov/gene/?term=71807
Fgd5	Fgd5	FYVE, RhoGEF and PH domain containing 5	232237	https://www.ncbi.nlm.nih.gov/gene/?term=232237
Ptpn3	Ptpn3	protein tyrosine phosphatase, non-receptor type 3	545622	https://www.ncbi.nlm.nih.gov/gene/?term=545622
Tbcel	Tbcel	tubulin folding cofactor E-like	272589	https://www.ncbi.nlm.nih.gov/gene/?term=272589

Biology Open • Supplementary information

Cep97	Cep97	centrosomal protein 97	74201	https://www.ncbi.nlm.nih.gov/gene/?term=74201
Itpripl2	Itpripl2	inositol 1,4,5-triphosphate receptor interacting protein-like 2	319622	https://www.ncbi.nlm.nih.gov/gene/?term=319622
Fam219b	Fam219b	family with sequence similarity 219, member B	78323	https://www.ncbi.nlm.nih.gov/gene/?term=78323
Zfp661	Zfp661	zinc finger protein 661	72180	https://www.ncbi.nlm.nih.gov/gene/?term=72180
Il25	Il25	interleukin 25	140806	https://www.ncbi.nlm.nih.gov/gene/?term=140806
Rest	Rest	RE1-silencing transcription factor	19712	https://www.ncbi.nlm.nih.gov/gene/?term=19712
Rab5b	Rab5b	RAB5B, member RAS oncogene family	19344	https://www.ncbi.nlm.nih.gov/gene/?term=19344
Txnip	Txnip	thioredoxin interacting protein	56338	https://www.ncbi.nlm.nih.gov/gene/?term=56338
Gpr137c	Gpr137c	G protein-coupled receptor 137C	70713	https://www.ncbi.nlm.nih.gov/gene/?term=70713

Biology Open • Supplementary information

Arhgef11	Arhgef11	Rho guanine nucleotide exchange factor (GEF) 11	213498	https://www.ncbi.nlm.nih.gov/gene/?term=213498
Zfp91	Zfp91	zinc finger protein 91	109910	https://www.ncbi.nlm.nih.gov/gene/?term=109910
Hs3st5	Hs3st5	heparan sulfate (glucosamine) 3-O-sulfotransferase 5	319415	https://www.ncbi.nlm.nih.gov/gene/?term=319415
Hbp1	Hbp1	high mobility group box transcription factor 1	73389	https://www.ncbi.nlm.nih.gov/gene/?term=73389
Rgmb	Rgmb	repulsive guidance molecule family member B	68799	https://www.ncbi.nlm.nih.gov/gene/?term=68799
Plekha3	Plekha3	pleckstrin homology domain-containing, family A (phosphoinositide binding specific) member 3	83435	https://www.ncbi.nlm.nih.gov/gene/?term=83435
Pgbd5	Pgbd5	piggyBac transposable element derived 5	209966	https://www.ncbi.nlm.nih.gov/gene/?term=209966
Btbd10	Btbd10	BTB (POZ) domain containing 10	68815	https://www.ncbi.nlm.nih.gov/gene/?term=68815
Ddhd2	Ddhd2	DDHD domain containing 2	72108	https://www.ncbi.nlm.nih.gov/gene/?term=72108

Biology Open • Supplementary information

Rnf138	Rnf138	ring finger protein 138	56515	https://www.ncbi.nlm.nih.gov/gene/?term=56515
Mcoln1	Mcoln1	mucolipin 1	94178	https://www.ncbi.nlm.nih.gov/gene/?term=94178
Ucp1	Ucp1	uncoupling protein 1 (mitochondrial, proton carrier)	22227	https://www.ncbi.nlm.nih.gov/gene/?term=22227
Pde1c	Pde1c	phosphodiesterase 1C	18575	https://www.ncbi.nlm.nih.gov/gene/?term=18575
Hyal4	Hyal4	hyaluronoglucosaminidase 4	77042	https://www.ncbi.nlm.nih.gov/gene/?term=77042
Glis3	Glis3	GLIS family zinc finger 3	226075	https://www.ncbi.nlm.nih.gov/gene/?term=226075
Slc43a1	Slc43a1	solute carrier family 43, member 1	72401	https://www.ncbi.nlm.nih.gov/gene/?term=72401
Pdp1	Pdp1	pyruvate dehyrogenase phosphatase catalytic subunit 1	381511	https://www.ncbi.nlm.nih.gov/gene/?term=381511
Tnfaip8	Tnfaip8	tumor necrosis factor, alpha-induced protein 8	106869	https://www.ncbi.nlm.nih.gov/gene/?term=106869

Biology Open • Supplementary information

Efemp2	Efemp2	epidermal growth factor-containing fibulin-like extracellular matrix protein 2	58859	https://www.ncbi.nlm.nih.gov/gene/?term=58859
Nrg3	Nrg3	neuregulin 3	18183	https://www.ncbi.nlm.nih.gov/gene/?term=18183
St7	St7	suppression of tumorigenicity 7	64213	https://www.ncbi.nlm.nih.gov/gene/?term=64213
Onecut2	Onecut2	one cut domain, family member 2	225631	https://www.ncbi.nlm.nih.gov/gene/?term=225631
Napg	Napg	N-ethylmaleimide sensitive fusion protein attachment protein gamma	108123	https://www.ncbi.nlm.nih.gov/gene/?term=108123
Trim12c	Trim12c	tripartite motif-containing 12C	319236	https://www.ncbi.nlm.nih.gov/gene/?term=319236
P2rx7	P2rx7	purinergic receptor P2X, ligand-gated ion channel, 7	18439	https://www.ncbi.nlm.nih.gov/gene/?term=18439
Ssrp1	Ssrp1	structure specific recognition protein 1	20833	https://www.ncbi.nlm.nih.gov/gene/?term=20833
Nbea	Nbea	neurobeachin	26422	https://www.ncbi.nlm.nih.gov/gene/?term=26422

Biology Open • Supplementary information

Lin7a	Lin7a	lin-7 homolog A (C. elegans)	108030	https://www.ncbi.nlm.nih.gov/gene/?term=108030
Zcchc24	Zcchc24	zinc finger, CCHC domain containing 24	71918	https://www.ncbi.nlm.nih.gov/gene/?term=71918
Lgals3	Lgals3	lectin, galactose binding, soluble 3	16854	https://www.ncbi.nlm.nih.gov/gene/?term=16854
Adamts5	Adamts5	a disintegrin-like and metallopeptidase (reprolysin type) with thrombospondin type 1 motif, 5 (aggrecanase-2)	23794	https://www.ncbi.nlm.nih.gov/gene/?term=23794
Trp53inp1	Trp53inp1	transformation related protein 53 inducible nuclear protein 1	60599	https://www.ncbi.nlm.nih.gov/gene/?term=60599
Dnajc14	Dnajc14	DnaJ heat shock protein family (Hsp40) member C14	74330	https://www.ncbi.nlm.nih.gov/gene/?term=74330
Crtac1	Crtac1	cartilage acidic protein 1	72832	https://www.ncbi.nlm.nih.gov/gene/?term=72832
Gpatch11	Gpatch11	G patch domain containing 11	53951	https://www.ncbi.nlm.nih.gov/gene/?term=53951
Ccdc117	Ccdc117	coiled-coil domain containing 117	104479	https://www.ncbi.nlm.nih.gov/gene/?term=104479

Biology Open • Supplementary information

Prrx1	Prrx1	paired related homeobox 1	18933	https://www.ncbi.nlm.nih.gov/gene/?term=18933
Mgat3	Mgat3	mannoside acetylglucosaminyltransferase 3	17309	https://www.ncbi.nlm.nih.gov/gene/?term=17309
Ncoa7	Ncoa7	nuclear receptor coactivator 7	211329	https://www.ncbi.nlm.nih.gov/gene/?term=211329
Cox5a	Cox5a	cytochrome c oxidase subunit 5A	12858	https://www.ncbi.nlm.nih.gov/gene/?term=12858
Chp1	Chp1	calcineurin-like EF hand protein 1	56398	https://www.ncbi.nlm.nih.gov/gene/?term=56398
Gba	Gba	glucosidase, beta, acid	14466	https://www.ncbi.nlm.nih.gov/gene/?term=14466
Ttyh1	Ttyh1	tweety family member 1	57776	https://www.ncbi.nlm.nih.gov/gene/?term=57776
Rnf170	Rnf170	ring finger protein 170	77733	https://www.ncbi.nlm.nih.gov/gene/?term=77733
Pid1	Pid1	phosphotyrosine interaction domain containing 1	98496	https://www.ncbi.nlm.nih.gov/gene/?term=98496

Biology Open • Supplementary information

4930402H24Rik	4930402H24Rik	RIKEN cDNA 4930402H24 gene	228602	https://www.ncbi.nlm.nih.gov/gene/?term=228602
Dtwd1	Dtwd1	DTW domain containing 1	69185	https://www.ncbi.nlm.nih.gov/gene/?term=69185
Spop	Spop	speckle-type POZ protein	20747	https://www.ncbi.nlm.nih.gov/gene/?term=20747
Fras1	Fras1	Fraser extracellular matrix complex subunit 1	231470	https://www.ncbi.nlm.nih.gov/gene/?term=231470
Slc38a9	Slc38a9	solute carrier family 38, member 9	268706	https://www.ncbi.nlm.nih.gov/gene/?term=268706
Dppa2	Dppa2	developmental pluripotency associated 2	73703	https://www.ncbi.nlm.nih.gov/gene/?term=73703
Abcb10	Abcb10	ATP-binding cassette, sub-family B (MDR/TAP), member 10	56199	https://www.ncbi.nlm.nih.gov/gene/?term=56199
Wdr46	Wdr46	WD repeat domain 46	57315	https://www.ncbi.nlm.nih.gov/gene/?term=57315
Rhobtb3	Rhobtb3	Rho-related BTB domain containing 3	73296	https://www.ncbi.nlm.nih.gov/gene/?term=73296

Lsm5	Lsm5	LSM5 homolog, U6 small nuclear RNA and mRNA degradation associated	66373	https://www.ncbi.nlm.nih.gov/gene/?term=66373
Nr3c2	Nr3c2	nuclear receptor subfamily 3, group C, member 2	110784	https://www.ncbi.nlm.nih.gov/gene/?term=110784
Zfp423	Zfp423	zinc finger protein 423	94187	https://www.ncbi.nlm.nih.gov/gene/?term=94187
Gpbp1l1	Gpbp1l1	GC-rich promoter binding protein 1-like 1	77110	https://www.ncbi.nlm.nih.gov/gene/?term=77110
Arhgap11a	Arhgap11a	Rho GTPase activating protein 11A	228482	https://www.ncbi.nlm.nih.gov/gene/?term=228482
Grk4	Grk4	G protein-coupled receptor kinase 4	14772	https://www.ncbi.nlm.nih.gov/gene/?term=14772
Nrbf2	Nrbf2	nuclear receptor binding factor 2	641340	https://www.ncbi.nlm.nih.gov/gene/?term=641340
Dpy19l4	Dpy19l4	dpy-19-like 4 (C. elegans)	381510	https://www.ncbi.nlm.nih.gov/gene/?term=381510
Rell1	Rell1	RELT-like 1	100532	https://www.ncbi.nlm.nih.gov/gene/?term=100532

Biology Open • Supplementary information

Plcb1	Plcb1	phospholipase C, beta 1	18795	https://www.ncbi.nlm.nih.gov/gene/?term=18795
Slc39a11	Slc39a11	solute carrier family 39 (metal ion transporter), member 11	69806	https://www.ncbi.nlm.nih.gov/gene/?term=69806
Larp4b	Larp4b	La ribonucleoprotein domain family, member 4B	217980	https://www.ncbi.nlm.nih.gov/gene/?term=217980
Shc1	Shc1	src homology 2 domain-containing transforming protein C1	20416	https://www.ncbi.nlm.nih.gov/gene/?term=20416
Clmp	Clmp	CXADR-like membrane protein	71566	https://www.ncbi.nlm.nih.gov/gene/?term=71566
Dnm2	Dnm2	dynamin 2	13430	https://www.ncbi.nlm.nih.gov/gene/?term=13430
Ptprd	Ptprd	protein tyrosine phosphatase, receptor type, D	19266	https://www.ncbi.nlm.nih.gov/gene/?term=19266
Pde12	Pde12	phosphodiesterase 12	211948	https://www.ncbi.nlm.nih.gov/gene/?term=211948
Mlxip	Mlxip	MLX interacting protein	208104	https://www.ncbi.nlm.nih.gov/gene/?term=208104

Smim13	Smim13	small integral membrane protein 13	108934	https://www.ncbi.nlm.nih.gov/gene/?term=108934
Ephb6	Ephb6	Eph receptor B6	13848	https://www.ncbi.nlm.nih.gov/gene/?term=13848
Ankfy1	Ankfy1	ankyrin repeat and FYVE domain containing 1	11736	https://www.ncbi.nlm.nih.gov/gene/?term=11736
Lrrn4cl	Lrrn4cl	LRRN4 C-terminal like	68852	https://www.ncbi.nlm.nih.gov/gene/?term=68852
Fam126a	Fam126a	family with sequence similarity 126, member A	84652	https://www.ncbi.nlm.nih.gov/gene/?term=84652
Zfp629	Zfp629	zinc finger protein 629	320683	https://www.ncbi.nlm.nih.gov/gene/?term=320683
Tmem164	Tmem164	transmembrane protein 164	209497	https://www.ncbi.nlm.nih.gov/gene/?term=209497
Fam149a	Fam149a	family with sequence similarity 149, member A	212326	https://www.ncbi.nlm.nih.gov/gene/?term=212326
Rere	Rere	arginine glutamic acid dipeptide (RE) repeats	68703	https://www.ncbi.nlm.nih.gov/gene/?term=68703

Biology Open • Supplementary information

Evc2	Evc2	EvC ciliary complex subunit 2	68525	https://www.ncbi.nlm.nih.gov/gene/?term=68525
Tmem263	Tmem263	transmembrane protein 263	103266	https://www.ncbi.nlm.nih.gov/gene/?term=103266
Elavl3	Elavl3	ELAV like RNA binding protein 3	15571	https://www.ncbi.nlm.nih.gov/gene/?term=15571
Pcdh12	Pcdh12	protocadherin 12	53601	https://www.ncbi.nlm.nih.gov/gene/?term=53601
Ankrd13a	Ankrd13a	ankyrin repeat domain 13a	68420	https://www.ncbi.nlm.nih.gov/gene/?term=68420
Mrps33	Mrps33	mitochondrial ribosomal protein S33	14548	https://www.ncbi.nlm.nih.gov/gene/?term=14548
Nanos1	Nanos1	nanos C2HC-type zinc finger 1	332397	https://www.ncbi.nlm.nih.gov/gene/?term=332397
Ccdc68	Ccdc68	coiled-coil domain containing 68	381175	https://www.ncbi.nlm.nih.gov/gene/?term=381175
Fhl2	Fhl2	four and a half LIM domains 2	14200	https://www.ncbi.nlm.nih.gov/gene/?term=14200

Biology Open • Supplementary information

2310022A10Rik	2310022A10Rik	RIKEN cDNA 2310022A10 gene	66367	https://www.ncbi.nlm.nih.gov/gene/?term=66367
Dnm1	Dnm1	dynamin 1	13429	https://www.ncbi.nlm.nih.gov/gene/?term=13429
Cstf2	Cstf2	cleavage stimulation factor, 3' pre-RNA subunit 2	108062	https://www.ncbi.nlm.nih.gov/gene/?term=108062
Asb1	Asb1	ankyrin repeat and SOCS box-containing 1	65247	https://www.ncbi.nlm.nih.gov/gene/?term=65247
Pik3c2a	Pik3c2a	phosphatidylinositol-4-phosphate 3-kinase catalytic subunit type 2 alpha	18704	https://www.ncbi.nlm.nih.gov/gene/?term=18704
Tank	Tank	TRAF family member-associated Nf-kappa B activator	21353	https://www.ncbi.nlm.nih.gov/gene/?term=21353
Samd8	Samd8	sterile alpha motif domain containing 8	67630	https://www.ncbi.nlm.nih.gov/gene/?term=67630
Otub2	Otub2	OTU domain, ubiquitin aldehyde binding 2	68149	https://www.ncbi.nlm.nih.gov/gene/?term=68149
Gpc4	Gpc4	glypican 4	14735	https://www.ncbi.nlm.nih.gov/gene/?term=14735

Biology Open • Supplementary information

Kdm3a	Kdm3a	lysine (K)-specific demethylase 3A	104263	https://www.ncbi.nlm.nih.gov/gene/?term=104263
Pvr	Pvr	poliovirus receptor	52118	https://www.ncbi.nlm.nih.gov/gene/?term=52118
Cnga4	Cnga4	cyclic nucleotide gated channel alpha 4	233649	https://www.ncbi.nlm.nih.gov/gene/?term=233649
Adam22	Adam22	a disintegrin and metallopeptidase domain 22	11496	https://www.ncbi.nlm.nih.gov/gene/?term=11496
Hebp1	Hebp1	heme binding protein 1	15199	https://www.ncbi.nlm.nih.gov/gene/?term=15199
Plet1	Plet1	placenta expressed transcript 1	76509	https://www.ncbi.nlm.nih.gov/gene/?term=76509
Zdhhc20	Zdhhc20	zinc finger, DHHC domain containing 20	75965	https://www.ncbi.nlm.nih.gov/gene/?term=75965
F2r	F2r	coagulation factor II (thrombin) receptor	14062	https://www.ncbi.nlm.nih.gov/gene/?term=14062
Mkrn2	Mkrn2	makorin, ring finger protein, 2	67027	https://www.ncbi.nlm.nih.gov/gene/?term=67027

Biology Open • Supplementary information

Slc8a1	Slc8a1	solute carrier family 8 (sodium/calcium exchanger), member 1	20541	https://www.ncbi.nlm.nih.gov/gene/?term=20541
Fem1b	Fem1b	feminization 1 homolog b (C. elegans)	14155	https://www.ncbi.nlm.nih.gov/gene/?term=14155
Enah	Enah	ENAH actin regulator	13800	https://www.ncbi.nlm.nih.gov/gene/?term=13800
Hoxd10	Hoxd10	homeobox D10	15430	https://www.ncbi.nlm.nih.gov/gene/?term=15430
Tmem117	Tmem117	transmembrane protein 117	320709	https://www.ncbi.nlm.nih.gov/gene/?term=320709
Mecom	Mecom	MDS1 and EVI1 complex locus	14013	https://www.ncbi.nlm.nih.gov/gene/?term=14013
Wdr26	Wdr26	WD repeat domain 26	226757	https://www.ncbi.nlm.nih.gov/gene/?term=226757
Ewsr1	Ewsr1	Ewing sarcoma breakpoint region 1	14030	https://www.ncbi.nlm.nih.gov/gene/?term=14030
Kcnn3	Kcnn3	potassium intermediate/small conductance calcium-activated channel, subfamily N, member 3	140493	https://www.ncbi.nlm.nih.gov/gene/?term=140493

Biology Open • Supplementary information

Eps8l2	Eps8l2	EPS8-like 2	98845	https://www.ncbi.nlm.nih.gov/gene/?term=98845
Actc1	Actc1	actin, alpha, cardiac muscle 1	11464	https://www.ncbi.nlm.nih.gov/gene/?term=11464
Ccser2	Ccser2	coiled-coil serine rich 2	72972	https://www.ncbi.nlm.nih.gov/gene/?term=72972
Pak7	Pak7	p21 (RAC1) activated kinase 7	241656	https://www.ncbi.nlm.nih.gov/gene/?term=241656
Cd38	Cd38	CD38 antigen	12494	https://www.ncbi.nlm.nih.gov/gene/?term=12494
Lin9	Lin9	lin-9 homolog (C. elegans)	72568	https://www.ncbi.nlm.nih.gov/gene/?term=72568
Dtl	Dtl	denticleless E3 ubiquitin protein ligase	76843	https://www.ncbi.nlm.nih.gov/gene/?term=76843
Uqcc1	Uqcc1	ubiquinol-cytochrome c reductase complex assembly factor 1	56046	https://www.ncbi.nlm.nih.gov/gene/?term=56046
2810459M11Rik	2810459M11Rik	RIKEN cDNA 2810459M11 gene	72792	https://www.ncbi.nlm.nih.gov/gene/?term=72792

Biology Open • Supplementary information

Ext1	Ext1	exostoses (multiple) 1	14042	https://www.ncbi.nlm.nih.gov/gene/?term=14042
Ddo	Ddo	D-aspartate oxidase	70503	https://www.ncbi.nlm.nih.gov/gene/?term=70503
Gsk3b	Gsk3b	glycogen synthase kinase 3 beta	56637	https://www.ncbi.nlm.nih.gov/gene/?term=56637
Dact1	Dact1	dishevelled-binding antagonist of beta-catenin 1	59036	https://www.ncbi.nlm.nih.gov/gene/?term=59036
Slc30a7	Slc30a7	solute carrier family 30 (zinc transporter), member 7	66500	https://www.ncbi.nlm.nih.gov/gene/?term=66500
Ppm1j	Ppm1j	protein phosphatase 1J	71887	https://www.ncbi.nlm.nih.gov/gene/?term=71887
Plscr4	Plscr4	phospholipid scramblase 4	235527	https://www.ncbi.nlm.nih.gov/gene/?term=235527
Cdip1	Cdip1	cell death inducing Trp53 target 1	66626	https://www.ncbi.nlm.nih.gov/gene/?term=66626
Hoxc4	Hoxc4	homeobox C4	15423	https://www.ncbi.nlm.nih.gov/gene/?term=15423

Biology Open • Supplementary information

Uvssa	Uvssa	UV stimulated scaffold protein A	71101	https://www.ncbi.nlm.nih.gov/gene/?term=71101
Folr1	Folr1	folate receptor 1 (adult)	14275	https://www.ncbi.nlm.nih.gov/gene/?term=14275
Zc2hc1c	Zc2hc1c	zinc finger, C2HC-type containing 1C	72350	https://www.ncbi.nlm.nih.gov/gene/?term=72350
Perp	Perp	PERP, TP53 apoptosis effector	64058	https://www.ncbi.nlm.nih.gov/gene/?term=64058
Gm4984	Gm4984	predicted pseudogene 4984	245347	https://www.ncbi.nlm.nih.gov/gene/?term=245347
Krtap12-1	Krtap12-1	keratin associated protein 12-1	16694	https://www.ncbi.nlm.nih.gov/gene/?term=16694
Cyp2c70	Cyp2c70	cytochrome P450, family 2, subfamily c, polypeptide 70	226105	https://www.ncbi.nlm.nih.gov/gene/?term=226105
Mark1	Mark1	MAP/microtubule affinity regulating kinase 1	226778	https://www.ncbi.nlm.nih.gov/gene/?term=226778
Chodl	Chodl	chondrolectin	246048	https://www.ncbi.nlm.nih.gov/gene/?term=246048

Biology Open • Supplementary information

Uggt1	Uggt1	UDP-glucose glycoprotein glucosyltransferase 1	320011	https://www.ncbi.nlm.nih.gov/gene/?term=320011
Slc16a1	Slc16a1	solute carrier family 16 (monocarboxylic acid transporters), member 1	20501	https://www.ncbi.nlm.nih.gov/gene/?term=20501
Nifk	Nifk	nucleolar protein interacting with the FHA domain of MKI67	67949	https://www.ncbi.nlm.nih.gov/gene/?term=67949
Camsap1	Camsap1	calmodulin regulated spectrin-associated protein 1	227634	https://www.ncbi.nlm.nih.gov/gene/?term=227634
Adam9	Adam9	a disintegrin and metallopeptidase domain 9 (meltrin gamma)	11502	https://www.ncbi.nlm.nih.gov/gene/?term=11502
Rftn1	Rftn1	raftlin lipid raft linker 1	76438	https://www.ncbi.nlm.nih.gov/gene/?term=76438
Neurod4	Neurod4	neurogenic differentiation 4	11923	https://www.ncbi.nlm.nih.gov/gene/?term=11923
Kctd12	Kctd12	potassium channel tetramerisation domain containing 12	239217	https://www.ncbi.nlm.nih.gov/gene/?term=239217
Zfp612	Zfp612	zinc finger protein 612	234725	https://www.ncbi.nlm.nih.gov/gene/?term=234725

Biology Open • Supplementary information

Magee2	Magee2	melanoma antigen, family E, 2	272790	https://www.ncbi.nlm.nih.gov/gene/?term=272790
Tacc1	Tacc1	transforming, acidic coiled-coil containing protein 1	320165	https://www.ncbi.nlm.nih.gov/gene/?term=320165
Caps2	Caps2	calcyphosphine 2	353025	https://www.ncbi.nlm.nih.gov/gene/?term=353025
Henmt1	Henmt1	HEN1 methyltransferase homolog 1 (Arabidopsis)	66715	https://www.ncbi.nlm.nih.gov/gene/?term=66715
Gabpa	Gabpa	GA repeat binding protein, alpha	14390	https://www.ncbi.nlm.nih.gov/gene/?term=14390
Rptor	Rptor	regulatory associated protein of MTOR, complex 1	74370	https://www.ncbi.nlm.nih.gov/gene/?term=74370
Havcr1	Havcr1	hepatitis A virus cellular receptor 1	171283	https://www.ncbi.nlm.nih.gov/gene/?term=171283
Ermap	Ermap	erythroblast membrane-associated protein	27028	https://www.ncbi.nlm.nih.gov/gene/?term=27028
Col3a1	Col3a1	collagen, type III, alpha 1	12825	https://www.ncbi.nlm.nih.gov/gene/?term=12825

Tram1	Tram1	translocating chain-associating membrane protein 1	72265	https://www.ncbi.nlm.nih.gov/gene/?term=72265
Pakap	Pakap	paralemmin A kinase anchor protein	677884	https://www.ncbi.nlm.nih.gov/gene/?term=677884
Fgf23	Fgf23	fibroblast growth factor 23	64654	https://www.ncbi.nlm.nih.gov/gene/?term=64654
Sort1	Sort1	sortilin 1	20661	https://www.ncbi.nlm.nih.gov/gene/?term=20661
Plxnb1	Plxnb1	plexin B1	235611	https://www.ncbi.nlm.nih.gov/gene/?term=235611
Gdap2	Gdap2	ganglioside-induced differentiation-associated-protein 2	14547	https://www.ncbi.nlm.nih.gov/gene/?term=14547
Tcf4	Tcf4	transcription factor 4	21413	https://www.ncbi.nlm.nih.gov/gene/?term=21413
Cldnd1	Cldnd1	claudin domain containing 1	224250	https://www.ncbi.nlm.nih.gov/gene/?term=224250
Rbpjl	Rbpjl	recombination signal binding protein for immunoglobulin kappa J region-like	19668	https://www.ncbi.nlm.nih.gov/gene/?term=19668

Biology Open • Supplementary information

Tab2	Tab2	TGF-beta activated kinase 1/MAP3K7 binding protein 2	68652	https://www.ncbi.nlm.nih.gov/gene/?term=68652
Snd1	Snd1	staphylococcal nuclease and tudor domain containing 1	56463	https://www.ncbi.nlm.nih.gov/gene/?term=56463
Sorbs1	Sorbs1	sorbin and SH3 domain containing 1	20411	https://www.ncbi.nlm.nih.gov/gene/?term=20411
Car8	Car8	carbonic anhydrase 8	12319	https://www.ncbi.nlm.nih.gov/gene/?term=12319
Ripk4	Ripk4	receptor-interacting serine-threonine kinase 4	72388	https://www.ncbi.nlm.nih.gov/gene/?term=72388
Dusp6	Dusp6	dual specificity phosphatase 6	67603	https://www.ncbi.nlm.nih.gov/gene/?term=67603
Rcvrn	Rcvrn	recoverin	19674	https://www.ncbi.nlm.nih.gov/gene/?term=19674
Tm9sf2	Tm9sf2	transmembrane 9 superfamily member 2	68059	https://www.ncbi.nlm.nih.gov/gene/?term=68059
Trim44	Trim44	tripartite motif-containing 44	80985	https://www.ncbi.nlm.nih.gov/gene/?term=80985

Biology Open • Supplementary information

Zdhhc3	Zdhhc3	zinc finger, DHHC domain containing 3	69035	https://www.ncbi.nlm.nih.gov/gene/?term=69035
Adamts12	Adamts12	a disintegrin-like and metallopeptidase (reprolysin type) with thrombospondin type 1 motif, 12	239337	https://www.ncbi.nlm.nih.gov/gene/?term=239337
Tmed8	Tmed8	transmembrane p24 trafficking protein 8	382620	https://www.ncbi.nlm.nih.gov/gene/?term=382620
Mgat4a	Mgat4a	mannoside acetylglucosaminyltransferase 4, isoenzyme A	269181	https://www.ncbi.nlm.nih.gov/gene/?term=269181
Chmp7	Chmp7	charged multivesicular body protein 7	105513	https://www.ncbi.nlm.nih.gov/gene/?term=105513
Hlf	Hlf	hepatic leukemia factor	217082	https://www.ncbi.nlm.nih.gov/gene/?term=217082
Trim16	Trim16	tripartite motif-containing 16	94092	https://www.ncbi.nlm.nih.gov/gene/?term=94092
Rbm25	Rbm25	RNA binding motif protein 25	67039	https://www.ncbi.nlm.nih.gov/gene/?term=67039
Retnlb	Retnlb	resistin like beta	57263	https://www.ncbi.nlm.nih.gov/gene/?term=57263

Biology Open • Supplementary information

Galnt13	Galnt13	polypeptide N-acetylgalactosaminyltransferase 13	271786	https://www.ncbi.nlm.nih.gov/gene/?term=271786
Lef1	Lef1	lymphoid enhancer binding factor 1	16842	https://www.ncbi.nlm.nih.gov/gene/?term=16842
Tmprss2	Tmprss2	transmembrane protease, serine 2	50528	https://www.ncbi.nlm.nih.gov/gene/?term=50528
Usp34	Usp34	ubiquitin specific peptidase 34	17847	https://www.ncbi.nlm.nih.gov/gene/?term=17847
Vapa	Vapa	vesicle-associated membrane protein, associated protein A	30960	https://www.ncbi.nlm.nih.gov/gene/?term=30960
Rdx	Rdx	radixin	19684	https://www.ncbi.nlm.nih.gov/gene/?term=19684
Dtna	Dtna	dystrobrevin alpha	13527	https://www.ncbi.nlm.nih.gov/gene/?term=13527
Proser2	Proser2	proline and serine rich 2	227545	https://www.ncbi.nlm.nih.gov/gene/?term=227545
Sema3e	Sema3e	sema domain, immunoglobulin domain (Ig), short basic domain, secreted, (semaphorin) 3E	20349	https://www.ncbi.nlm.nih.gov/gene/?term=20349

Biology Open • Supplementary information

Atl3	Atl3	atlastin GTPase 3	109168	https://www.ncbi.nlm.nih.gov/gene/?term=109168
Tet1	Tet1	tet methylcytosine dioxygenase 1	52463	https://www.ncbi.nlm.nih.gov/gene/?term=52463
Krit1	Krit1	KRIT1, ankyrin repeat containing	79264	https://www.ncbi.nlm.nih.gov/gene/?term=79264
Cul2	Cul2	cullin 2	71745	https://www.ncbi.nlm.nih.gov/gene/?term=71745
Dhx40	Dhx40	DEAH (Asp-Glu-Ala-His) box polypeptide 40	67487	https://www.ncbi.nlm.nih.gov/gene/?term=67487
Mynn	Mynn	myoneurin	80732	https://www.ncbi.nlm.nih.gov/gene/?term=80732
Hic2	Hic2	hypermethylated in cancer 2	58180	https://www.ncbi.nlm.nih.gov/gene/?term=58180
Dock5	Dock5	dedicator of cytokinesis 5	68813	https://www.ncbi.nlm.nih.gov/gene/?term=68813
Epm2aip1	Epm2aip1	EPM2A (laforin) interacting protein 1	77781	https://www.ncbi.nlm.nih.gov/gene/?term=77781

Tfg	Tfg	Trk-fused gene	21787	https://www.ncbi.nlm.nih.gov/gene/?term=21787
Tyro3	Tyro3	TYRO3 protein tyrosine kinase 3	22174	https://www.ncbi.nlm.nih.gov/gene/?term=22174
Top2a	Top2a	topoisomerase (DNA) II alpha	21973	https://www.ncbi.nlm.nih.gov/gene/?term=21973
Map2k3	Map2k3	mitogen-activated protein kinase kinase 3	26397	https://www.ncbi.nlm.nih.gov/gene/?term=26397
Tbx20	Tbx20	T-box 20	57246	https://www.ncbi.nlm.nih.gov/gene/?term=57246
Ssh2	Ssh2	slingshot protein phosphatase 2	237860	https://www.ncbi.nlm.nih.gov/gene/?term=237860
Sypl	Sypl	synaptophysin-like protein	19027	https://www.ncbi.nlm.nih.gov/gene/?term=19027
Oaz1	Oaz1	ornithine decarboxylase antizyme 1	18245	https://www.ncbi.nlm.nih.gov/gene/?term=18245
Ube2g2	Ube2g2	ubiquitin-conjugating enzyme E2G 2	22213	https://www.ncbi.nlm.nih.gov/gene/?term=22213

Srsf2	Srsf2	serine/arginine-rich splicing factor 2	20382	https://www.ncbi.nlm.nih.gov/gene/?term=20382
Nck1	Nck1	non-catalytic region of tyrosine kinase adaptor protein 1	17973	https://www.ncbi.nlm.nih.gov/gene/?term=17973
Ubqln1	Ubqln1	ubiquilin 1	56085	https://www.ncbi.nlm.nih.gov/gene/?term=56085
Inpp1	Inpp1	inositol polyphosphate-1-phosphatase	16329	https://www.ncbi.nlm.nih.gov/gene/?term=16329
Pik3ca	Pik3ca	phosphatidylinositol-4,5-bisphosphate 3-kinase catalytic subunit alpha	18706	https://www.ncbi.nlm.nih.gov/gene/?term=18706
Fam120a	Fam120a	family with sequence similarity 120, member A	218236	https://www.ncbi.nlm.nih.gov/gene/?term=218236
Epha5	Epha5	Eph receptor A5	13839	https://www.ncbi.nlm.nih.gov/gene/?term=13839
Cherp	Cherp	calcium homeostasis endoplasmic reticulum protein	27967	https://www.ncbi.nlm.nih.gov/gene/?term=27967
Rrp1b	Rrp1b	ribosomal RNA processing 1 homolog B (S. cerevisiae)	72462	https://www.ncbi.nlm.nih.gov/gene/?term=72462

Traf5	Traf5	TNF receptor-associated factor 5	22033	https://www.ncbi.nlm.nih.gov/gene/?term=22033
Paf1	Paf1	Paf1, RNA polymerase II complex component	54624	https://www.ncbi.nlm.nih.gov/gene/?term=54624
Pced1b	Pced1b	PC-esterase domain containing 1B	239647	https://www.ncbi.nlm.nih.gov/gene/?term=239647
Zc3h7a	Zc3h7a	zinc finger CCCH type containing 7 A	106205	https://www.ncbi.nlm.nih.gov/gene/?term=106205
Gcfc2	Gcfc2	GC-rich sequence DNA binding factor 2	330361	https://www.ncbi.nlm.nih.gov/gene/?term=330361
Zfp84	Zfp84	zinc finger protein 84	74352	https://www.ncbi.nlm.nih.gov/gene/?term=74352
Hace1	Hace1	HECT domain and ankyrin repeat containing, E3 ubiquitin protein ligase 1	209462	https://www.ncbi.nlm.nih.gov/gene/?term=209462
Stau1	Stau1	staufen double-stranded RNA binding protein 1	20853	https://www.ncbi.nlm.nih.gov/gene/?term=20853
Aqp4	Aqp4	aquaporin 4	11829	https://www.ncbi.nlm.nih.gov/gene/?term=11829

Biology Open • Supplementary information

Shprh	Shprh	SNF2 histone linker PHD RING helicase	268281	https://www.ncbi.nlm.nih.gov/gene/?term=268281
Rala	Rala	v-ral simian leukemia viral oncogene A (ras related)	56044	https://www.ncbi.nlm.nih.gov/gene/?term=56044
Wrn	Wrn	Werner syndrome RecQ like helicase	22427	https://www.ncbi.nlm.nih.gov/gene/?term=22427
Cep162	Cep162	centrosomal protein 162	382090	https://www.ncbi.nlm.nih.gov/gene/?term=382090
Donson	Donson	downstream neighbor of SON	60364	https://www.ncbi.nlm.nih.gov/gene/?term=60364
Rtl3	Rtl3	retrotransposon Gag like 3	213436	https://www.ncbi.nlm.nih.gov/gene/?term=213436
Pank2	Pank2	pantothenate kinase 2	74450	https://www.ncbi.nlm.nih.gov/gene/?term=74450
Usp1	Usp1	ubiquitin specific peptidase 1	230484	https://www.ncbi.nlm.nih.gov/gene/?term=230484
Emc4	Emc4	ER membrane protein complex subunit 4	68032	https://www.ncbi.nlm.nih.gov/gene/?term=68032

Csnk1e	Csnk1e	casein kinase 1, epsilon	27373	https://www.ncbi.nlm.nih.gov/gene/?term=27373
Pclo	Pclo	piccolo (presynaptic cytomatrix protein)	26875	https://www.ncbi.nlm.nih.gov/gene/?term=26875
Cpsf2	Cpsf2	cleavage and polyadenylation specific factor 2	51786	https://www.ncbi.nlm.nih.gov/gene/?term=51786
Pde5a	Pde5a	phosphodiesterase 5A, cGMP-specific	242202	https://www.ncbi.nlm.nih.gov/gene/?term=242202
Traf3ip2	Traf3ip2	TRAF3 interacting protein 2	103213	https://www.ncbi.nlm.nih.gov/gene/?term=103213
Ttr	Ttr	transthyretin	22139	https://www.ncbi.nlm.nih.gov/gene/?term=22139
Lcorl	Lcorl	ligand dependent nuclear receptor corepressor-like	209707	https://www.ncbi.nlm.nih.gov/gene/?term=209707
Ube2j1	Ube2j1	ubiquitin-conjugating enzyme E2J 1	56228	https://www.ncbi.nlm.nih.gov/gene/?term=56228
Il1rn	Il1rn	interleukin 1 receptor antagonist	16181	https://www.ncbi.nlm.nih.gov/gene/?term=16181

Biology Open • Supplementary information

Fam13c	Fam13c	family with sequence similarity 13, member C	71721	https://www.ncbi.nlm.nih.gov/gene/?term=71721
Tead1	Tead1	TEA domain family member 1	21676	https://www.ncbi.nlm.nih.gov/gene/?term=21676
Hmgcll1	Hmgcll1	3-hydroxymethyl-3-methylglutaryl-Coenzyme A lyase-like 1	208982	https://www.ncbi.nlm.nih.gov/gene/?term=208982
Npy2r	Npy2r	neuropeptide Y receptor Y2	18167	https://www.ncbi.nlm.nih.gov/gene/?term=18167
Slc25a33	Slc25a33	solute carrier family 25, member 33	70556	https://www.ncbi.nlm.nih.gov/gene/?term=70556
Htr2a	Htr2a	5-hydroxytryptamine (serotonin) receptor 2A	15558	https://www.ncbi.nlm.nih.gov/gene/?term=15558
Ipo5	Ipo5	importin 5	70572	https://www.ncbi.nlm.nih.gov/gene/?term=70572
Nr2c2	Nr2c2	nuclear receptor subfamily 2, group C, member 2	22026	https://www.ncbi.nlm.nih.gov/gene/?term=22026
Rex2	Rex2	reduced expression 2	100043034	https://www.ncbi.nlm.nih.gov/gene/?term=100043

Biology Open • Supplementary information

Tshr	Tshr	thyroid stimulating hormone receptor	22095	https://www.ncbi.nlm.nih.gov/gene/?term=22095
Npffr2	Npffr2	neuropeptide FF receptor 2	104443	https://www.ncbi.nlm.nih.gov/gene/?term=104443
Zfp600	Zfp600	zinc finger protein 600	667666	https://www.ncbi.nlm.nih.gov/gene/?term=667666
Sgms2	Sgms2	sphingomyelin synthase 2	74442	https://www.ncbi.nlm.nih.gov/gene/?term=74442
Acyp2	Acyp2	acylphosphatase 2, muscle type	75572	https://www.ncbi.nlm.nih.gov/gene/?term=75572
Arx	Arx	aristaless related homeobox	11878	https://www.ncbi.nlm.nih.gov/gene/?term=11878
Ubr5	Ubr5	ubiquitin protein ligase E3 component n-recognin 5	70790	https://www.ncbi.nlm.nih.gov/gene/?term=70790
4930579G24Rik	4930579G24Rik	RIKEN cDNA 4930579G24 gene	75939	https://www.ncbi.nlm.nih.gov/gene/?term=75939
Capn6	Capn6	calpain 6	12338	https://www.ncbi.nlm.nih.gov/gene/?term=12338

Biology Open • Supplementary information

Il17re	Il17re	interleukin 17 receptor E	57890	https://www.ncbi.nlm.nih.gov/gene/?term=57890
Slitrk3	Slitrk3	SLIT and NTRK-like family, member 3	386750	https://www.ncbi.nlm.nih.gov/gene/?term=386750
Tmem47	Tmem47	transmembrane protein 47	192216	https://www.ncbi.nlm.nih.gov/gene/?term=192216
Arf6	Arf6	ADP-ribosylation factor 6	11845	https://www.ncbi.nlm.nih.gov/gene/?term=11845
Cdk14	Cdk14	cyclin-dependent kinase 14	18647	https://www.ncbi.nlm.nih.gov/gene/?term=18647
Mug2	Mug2	murinoglobulin 2	17837	https://www.ncbi.nlm.nih.gov/gene/?term=17837
Zc3h14	Zc3h14	zinc finger CCCH type containing 14	75553	https://www.ncbi.nlm.nih.gov/gene/?term=75553
Lrrtm2	Lrrtm2	leucine rich repeat transmembrane neuronal 2	107065	https://www.ncbi.nlm.nih.gov/gene/?term=107065
Pdss2	Pdss2	prenyl (solanesyl) diphosphate synthase, subunit 2	71365	https://www.ncbi.nlm.nih.gov/gene/?term=71365

Biology Open • Supplementary information

Hira	Hira	histone cell cycle regulator	15260	https://www.ncbi.nlm.nih.gov/gene/?term=15260
Magt1	Magt1	magnesium transporter 1	67075	https://www.ncbi.nlm.nih.gov/gene/?term=67075
Themis	Themis	thymocyte selection associated	210757	https://www.ncbi.nlm.nih.gov/gene/?term=210757
Pno1	Pno1	partner of NOB1 homolog	66249	https://www.ncbi.nlm.nih.gov/gene/?term=66249
Ypel5	Ypel5	yippee like 5	383295	https://www.ncbi.nlm.nih.gov/gene/?term=383295
Rtl6	Rtl6	retrotransposon Gag like 6	223732	https://www.ncbi.nlm.nih.gov/gene/?term=223732
Dmtn	Dmtn	dematin actin binding protein	13829	https://www.ncbi.nlm.nih.gov/gene/?term=13829
Kdm2a	Kdm2a	lysine (K)-specific demethylase 2A	225876	https://www.ncbi.nlm.nih.gov/gene/?term=225876
Adss	Adss	adenylosuccinate synthetase, non muscle	11566	https://www.ncbi.nlm.nih.gov/gene/?term=11566

Biology Open • Supplementary information

Morf4l2	Morf4l2	mortality factor 4 like 2	56397	https://www.ncbi.nlm.nih.gov/gene/?term=56397
Ar	Ar	androgen receptor	11835	https://www.ncbi.nlm.nih.gov/gene/?term=11835
Cd209b	Cd209b	CD209b antigen	69165	https://www.ncbi.nlm.nih.gov/gene/?term=69165
B3galt1	B3galt1	UDP-Gal:betaGlcNAc beta 1,3-galactosyltransferase, polypeptide 1	26877	https://www.ncbi.nlm.nih.gov/gene/?term=26877
Fam155a	Fam155a	family with sequence similarity 155, member A	270028	https://www.ncbi.nlm.nih.gov/gene/?term=270028
Rspo4	Rspo4	R-spondin 4	228770	https://www.ncbi.nlm.nih.gov/gene/?term=228770
Ndst3	Ndst3	N-deacetylase/N-sulfotransferase (heparan glucosaminyl) 3	83398	https://www.ncbi.nlm.nih.gov/gene/?term=83398
Abhd2	Abhd2	abhydrolase domain containing 2	54608	https://www.ncbi.nlm.nih.gov/gene/?term=54608
Mmp16	Mmp16	matrix metallopeptidase 16	17389	https://www.ncbi.nlm.nih.gov/gene/?term=17389

A430033K04Rik	A430033K04Rik	RIKEN cDNA A430033K04 gene	243308	https://www.ncbi.nlm.nih.gov/gene/?term=243308
Abcg4	Abcg4	ATP binding cassette subfamily G member 4	192663	https://www.ncbi.nlm.nih.gov/gene/?term=192663
Trim41	Trim41	tripartite motif-containing 41	211007	https://www.ncbi.nlm.nih.gov/gene/?term=211007
Pcp4	Pcp4	Purkinje cell protein 4	18546	https://www.ncbi.nlm.nih.gov/gene/?term=18546
Ehd1	Ehd1	EH-domain containing 1	13660	https://www.ncbi.nlm.nih.gov/gene/?term=13660
Ces2g	Ces2g	carboxylesterase 2G	72361	https://www.ncbi.nlm.nih.gov/gene/?term=72361
Tmem25	Tmem25	transmembrane protein 25	71687	https://www.ncbi.nlm.nih.gov/gene/?term=71687
Tomm22	Tomm22	translocase of outer mitochondrial membrane 22	223696	https://www.ncbi.nlm.nih.gov/gene/?term=223696
Mmp17	Mmp17	matrix metallopeptidase 17	23948	https://www.ncbi.nlm.nih.gov/gene/?term=23948

Sgce	Sgce	sarcoglycan, epsilon	20392	https://www.ncbi.nlm.nih.gov/gene/?term=20392
Slc7a7	Slc7a7	solute carrier family 7 (cationic amino acid transporter, y+ system), member 7	20540	https://www.ncbi.nlm.nih.gov/gene/?term=20540
Slc10a7	Slc10a7	solute carrier family 10 (sodium/bile acid cotransporter family), member 7	76775	https://www.ncbi.nlm.nih.gov/gene/?term=76775
Ptgfr	Ptgfr	prostaglandin F receptor	19220	https://www.ncbi.nlm.nih.gov/gene/?term=19220
Slc35b3	Slc35b3	solute carrier family 35, member B3	108652	https://www.ncbi.nlm.nih.gov/gene/?term=108652
Vegfa	Vegfa	vascular endothelial growth factor A	22339	https://www.ncbi.nlm.nih.gov/gene/?term=22339
Neurod6	Neurod6	neurogenic differentiation 6	11922	https://www.ncbi.nlm.nih.gov/gene/?term=11922
Zfx	Zfx	zinc finger protein X-linked	22764	https://www.ncbi.nlm.nih.gov/gene/?term=22764
Smad5	Smad5	SMAD family member 5	17129	https://www.ncbi.nlm.nih.gov/gene/?term=17129

Mapk1ip1l	Mapk1ip1l	mitogen-activated protein kinase 1 interacting protein 1-like	218975	https://www.ncbi.nlm.nih.gov/gene/?term=218975
Pramel3	Pramel3	preferentially expressed antigen in melanoma-like 3	83565	https://www.ncbi.nlm.nih.gov/gene/?term=83565
Hecw2	Hecw2	HECT, C2 and WW domain containing E3 ubiquitin protein ligase 2	329152	https://www.ncbi.nlm.nih.gov/gene/?term=329152
Rsrp1	Rsrp1	arginine/serine rich protein 1	27981	https://www.ncbi.nlm.nih.gov/gene/?term=27981
Bcat1	Bcat1	branched chain aminotransferase 1, cytosolic	12035	https://www.ncbi.nlm.nih.gov/gene/?term=12035
Caprin1	Caprin1	cell cycle associated protein 1	53872	https://www.ncbi.nlm.nih.gov/gene/?term=53872
Osbpl8	Osbpl8	oxysterol binding protein-like 8	237542	https://www.ncbi.nlm.nih.gov/gene/?term=237542
Cdc14a	Cdc14a	CDC14 cell division cycle 14A	229776	https://www.ncbi.nlm.nih.gov/gene/?term=229776
Ptpdc1	Ptpdc1	protein tyrosine phosphatase domain containing 1	218232	https://www.ncbi.nlm.nih.gov/gene/?term=218232

Biology Open • Supplementary information

Gabra4	Gabra4	gamma-aminobutyric acid (GABA) A receptor, subunit alpha 4	14397	https://www.ncbi.nlm.nih.gov/gene/?term=14397
Cers6	Cers6	ceramide synthase 6	241447	https://www.ncbi.nlm.nih.gov/gene/?term=241447
AV320801	AV320801	expressed sequence AV320801	331531	https://www.ncbi.nlm.nih.gov/gene/?term=331531
Pgap1	Pgap1	post-GPI attachment to proteins 1	241062	https://www.ncbi.nlm.nih.gov/gene/?term=241062
Hoxa5	Hoxa5	homeobox A5	15402	https://www.ncbi.nlm.nih.gov/gene/?term=15402
Rag1	Rag1	recombination activating gene 1	19373	https://www.ncbi.nlm.nih.gov/gene/?term=19373
Ccdc126	Ccdc126	coiled-coil domain containing 126	57895	https://www.ncbi.nlm.nih.gov/gene/?term=57895
Csnk1a1	Csnk1a1	casein kinase 1, alpha 1	93687	https://www.ncbi.nlm.nih.gov/gene/?term=93687
Ctdspl2	Ctdspl2	CTD (carboxy-terminal domain, RNA polymerase II, polypeptide A) small phosphatase like 2	329506	https://www.ncbi.nlm.nih.gov/gene/?term=329506

Biology Open • Supplementary information

Mier3	Mier3	MIER family member 3	218613	https://www.ncbi.nlm.nih.gov/gene/?term=218613
Phf19	Phf19	PHD finger protein 19	74016	https://www.ncbi.nlm.nih.gov/gene/?term=74016
Ola1	Ola1	Obg-like ATPase 1	67059	https://www.ncbi.nlm.nih.gov/gene/?term=67059
D6Wsu163e	D6Wsu163e	DNA segment, Chr 6, Wayne State University 163, expressed	28040	https://www.ncbi.nlm.nih.gov/gene/?term=28040
Kdm4a	Kdm4a	lysine (K)-specific demethylase 4A	230674	https://www.ncbi.nlm.nih.gov/gene/?term=230674
Bhlhe22	Bhlhe22	basic helix-loop-helix family, member e22	59058	https://www.ncbi.nlm.nih.gov/gene/?term=59058
Ube2v2	Ube2v2	ubiquitin-conjugating enzyme E2 variant 2	70620	https://www.ncbi.nlm.nih.gov/gene/?term=70620
Eef1e1	Eef1e1	eukaryotic translation elongation factor 1 epsilon 1	66143	https://www.ncbi.nlm.nih.gov/gene/?term=66143
Polr3a	Polr3a	polymerase (RNA) III (DNA directed) polypeptide A	218832	https://www.ncbi.nlm.nih.gov/gene/?term=218832

Biology Open • Supplementary information

D630023F18Rik	D630023F18Rik	RIKEN cDNA D630023F18 gene	98303	https://www.ncbi.nlm.nih.gov/gene/?term=98303
Cbfb	Cbfb	core binding factor beta	12400	https://www.ncbi.nlm.nih.gov/gene/?term=12400
Nsrp1	Nsrp1	nuclear speckle regulatory protein 1	237859	https://www.ncbi.nlm.nih.gov/gene/?term=237859
Pank3	Pank3	pantothenate kinase 3	211347	https://www.ncbi.nlm.nih.gov/gene/?term=211347
Fkbp7	Fkbp7	FK506 binding protein 7	14231	https://www.ncbi.nlm.nih.gov/gene/?term=14231
Phlpp1	Phlpp1	PH domain and leucine rich repeat protein phosphatase 1	98432	https://www.ncbi.nlm.nih.gov/gene/?term=98432
Nsd3	Nsd3	nuclear receptor binding SET domain protein 3	234135	https://www.ncbi.nlm.nih.gov/gene/?term=234135
Pex13	Pex13	peroxisomal biogenesis factor 13	72129	https://www.ncbi.nlm.nih.gov/gene/?term=72129
Rbm12	Rbm12	RNA binding motif protein 12	75710	https://www.ncbi.nlm.nih.gov/gene/?term=75710

Biology Open • Supplementary information

Ptprf	Ptprf	protein tyrosine phosphatase, receptor type, F	19268	https://www.ncbi.nlm.nih.gov/gene/?term=19268
Car13	Car13	carbonic anhydrase 13	71934	https://www.ncbi.nlm.nih.gov/gene/?term=71934
Tmigd3	Tmigd3	transmembrane and immunoglobulin domain containing 3	69296	https://www.ncbi.nlm.nih.gov/gene/?term=69296
Cbx5	Cbx5	chromobox 5	12419	https://www.ncbi.nlm.nih.gov/gene/?term=12419
Gm5796	Gm5796	predicted gene 5796	545007	https://www.ncbi.nlm.nih.gov/gene/?term=545007
Plekhg4	Plekhg4	pleckstrin homology domain containing, family G (with RhoGef domain) member 4	102075	https://www.ncbi.nlm.nih.gov/gene/?term=102075
Tmem33	Tmem33	transmembrane protein 33	67878	https://www.ncbi.nlm.nih.gov/gene/?term=67878
Tm9sf3	Tm9sf3	transmembrane 9 superfamily member 3	107358	https://www.ncbi.nlm.nih.gov/gene/?term=107358
Qtrt2	Qtrt2	queuine tRNA-ribosyltransferase accessory subunit 2	106248	https://www.ncbi.nlm.nih.gov/gene/?term=106248

Biology Open • Supplementary information

Cask	Cask	calcium/calmodulin-dependent serine protein kinase (MAGUK family)	12361	https://www.ncbi.nlm.nih.gov/gene/?term=12361
Adora3	Adora3	adenosine A3 receptor	11542	https://www.ncbi.nlm.nih.gov/gene/?term=11542
Nhsl1	Nhsl1	NHS-like 1	215819	https://www.ncbi.nlm.nih.gov/gene/?term=215819
Rspo3	Rspo3	R-spondin 3	72780	https://www.ncbi.nlm.nih.gov/gene/?term=72780
Kl	Kl	klotho	16591	https://www.ncbi.nlm.nih.gov/gene/?term=16591
Cep170	Cep170	centrosomal protein 170	545389	https://www.ncbi.nlm.nih.gov/gene/?term=545389
Elavl1	Elavl1	ELAV (embryonic lethal, abnormal vision)-like 1 (Hu antigen R)	15568	https://www.ncbi.nlm.nih.gov/gene/?term=15568
Ndufaf5	Ndufaf5	NADH:ubiquinone oxidoreductase complex assembly factor 5	69487	https://www.ncbi.nlm.nih.gov/gene/?term=69487
Gcc2	Gcc2	GRIP and coiled-coil domain containing 2	70297	https://www.ncbi.nlm.nih.gov/gene/?term=70297

Rundc3b	Rundc3b	RUN domain containing 3B	242819	https://www.ncbi.nlm.nih.gov/gene/?term=242819
Cops7b	Cops7b	COP9 signalosome subunit 7B	26895	https://www.ncbi.nlm.nih.gov/gene/?term=26895
Dkk4	Dkk4	dickkopf WNT signaling pathway inhibitor 4	234130	https://www.ncbi.nlm.nih.gov/gene/?term=234130
Orc1	Orc1	origin recognition complex, subunit 1	18392	https://www.ncbi.nlm.nih.gov/gene/?term=18392
Nkain3	Nkain3	Na+/K+ transporting ATPase interacting 3	269513	https://www.ncbi.nlm.nih.gov/gene/?term=269513
Nucks1	Nucks1	nuclear casein kinase and cyclin-dependent kinase substrate 1	98415	https://www.ncbi.nlm.nih.gov/gene/?term=98415
Gm5128	Gm5128	predicted gene 5128	331529	https://www.ncbi.nlm.nih.gov/gene/?term=331529
Chm	Chm	choroidermia (RAB escort protein 1)	12662	https://www.ncbi.nlm.nih.gov/gene/?term=12662
Cdk12	Cdk12	cyclin-dependent kinase 12	69131	https://www.ncbi.nlm.nih.gov/gene/?term=69131

Biology Open • Supplementary information

Rhoq	Rhoq	ras homolog family member Q	104215	https://www.ncbi.nlm.nih.gov/gene/?term=104215
Prkd3	Prkd3	protein kinase D3	75292	https://www.ncbi.nlm.nih.gov/gene/?term=75292
Kcnh5	Kcnh5	potassium voltage-gated channel, subfamily H (eag-related), member 5	238271	https://www.ncbi.nlm.nih.gov/gene/?term=238271
Scel	Scel	sciellin	64929	https://www.ncbi.nlm.nih.gov/gene/?term=64929
Bclaf1	Bclaf1	BCL2-associated transcription factor 1	72567	https://www.ncbi.nlm.nih.gov/gene/?term=72567
Mvb12b	Mvb12b	multivesicular body subunit 12B	72543	https://www.ncbi.nlm.nih.gov/gene/?term=72543
Klk11	Klk11	kallikrein related-peptidase 11	56538	https://www.ncbi.nlm.nih.gov/gene/?term=56538
Bicral	Bicral	BRD4 interacting chromatin remodeling complex associated protein like	210982	https://www.ncbi.nlm.nih.gov/gene/?term=210982
Rc3h1	Rc3h1	RING CCCH (C3H) domains 1	381305	https://www.ncbi.nlm.nih.gov/gene/?term=381305

Arhgef12	Arhgef12	Rho guanine nucleotide exchange factor (GEF) 12	69632	https://www.ncbi.nlm.nih.gov/gene/?term=69632
Gm7903	Gm7903	predicted gene 7903	666040	https://www.ncbi.nlm.nih.gov/gene/?term=666040
Prickle1	Prickle1	prickle planar cell polarity protein 1	106042	https://www.ncbi.nlm.nih.gov/gene/?term=106042
Atrx	Atrx	ATRX, chromatin remodeler	22589	https://www.ncbi.nlm.nih.gov/gene/?term=22589
Ckap2l	Ckap2l	cytoskeleton associated protein 2-like	70466	https://www.ncbi.nlm.nih.gov/gene/?term=70466
Mgst1	Mgst1	microsomal glutathione S-transferase 1	56615	https://www.ncbi.nlm.nih.gov/gene/?term=56615
Ube2a	Ube2a	ubiquitin-conjugating enzyme E2A	22209	https://www.ncbi.nlm.nih.gov/gene/?term=22209
Usp40	Usp40	ubiquitin specific peptidase 40	227334	https://www.ncbi.nlm.nih.gov/gene/?term=227334
Hsp90b1	Hsp90b1	heat shock protein 90, beta (Grp94), member 1	22027	https://www.ncbi.nlm.nih.gov/gene/?term=22027

Biology Open • Supplementary information

Gtpbp8	Gtpbp8	GTP-binding protein 8 (putative)	66067	https://www.ncbi.nlm.nih.gov/gene/?term=66067
Acsl3	Acsl3	acyl-CoA synthetase long-chain family member 3	74205	https://www.ncbi.nlm.nih.gov/gene/?term=74205
Inpp4a	Inpp4a	inositol polyphosphate-4-phosphatase, type I	269180	https://www.ncbi.nlm.nih.gov/gene/?term=269180
Stim2	Stim2	stromal interaction molecule 2	116873	https://www.ncbi.nlm.nih.gov/gene/?term=116873
Fam89a	Fam89a	family with sequence similarity 89, member A	69627	https://www.ncbi.nlm.nih.gov/gene/?term=69627
Lelp1	Lelp1	late cornified envelope-like proline-rich 1	69332	https://www.ncbi.nlm.nih.gov/gene/?term=69332
Ptbp2	Ptbp2	polypyrimidine tract binding protein 2	56195	https://www.ncbi.nlm.nih.gov/gene/?term=56195
Rubcnl	Rubcnl	RUN and cysteine rich domain containing beclin 1 interacting protein like	271221	https://www.ncbi.nlm.nih.gov/gene/?term=271221
Gk	Gk	glycerol kinase	14933	https://www.ncbi.nlm.nih.gov/gene/?term=14933

Biology Open • Supplementary information

Kctd4	Kctd4	potassium channel tetramerisation domain containing 4	67516	https://www.ncbi.nlm.nih.gov/gene/?term=67516
Mbip	Mbip	MAP3K12 binding inhibitory protein 1	217588	https://www.ncbi.nlm.nih.gov/gene/?term=217588
Cxcl10	Cxcl10	chemokine (C-X-C motif) ligand 10	15945	https://www.ncbi.nlm.nih.gov/gene/?term=15945
Fbxo8	Fbxo8	F-box protein 8	50753	https://www.ncbi.nlm.nih.gov/gene/?term=50753
Acvr2a	Acvr2a	activin receptor IIA	11480	https://www.ncbi.nlm.nih.gov/gene/?term=11480
Papola	Papola	poly (A) polymerase alpha	18789	https://www.ncbi.nlm.nih.gov/gene/?term=18789
Zxdc	Zxdc	ZXD family zinc finger C	80292	https://www.ncbi.nlm.nih.gov/gene/?term=80292
Pitpnm2	Pitpnm2	phosphatidylinositol transfer protein, membrane-associated 2	19679	https://www.ncbi.nlm.nih.gov/gene/?term=19679
Lpin1	Lpin1	lipin 1	14245	https://www.ncbi.nlm.nih.gov/gene/?term=14245

Biology Open • Supplementary information

Lrrcc1	Lrrcc1	leucine rich repeat and coiled-coil domain containing 1	71710	https://www.ncbi.nlm.nih.gov/gene/?term=71710
Dync1i1	Dync1i1	dynein cytoplasmic 1 intermediate chain 1	13426	https://www.ncbi.nlm.nih.gov/gene/?term=13426
Ptpn2	Ptpn2	protein tyrosine phosphatase, non-receptor type 2	19255	https://www.ncbi.nlm.nih.gov/gene/?term=19255
Smim26	Smim26	small integral membrane protein 26	228715	https://www.ncbi.nlm.nih.gov/gene/?term=228715
Mrgpra2a	Mrgpra2a	MAS-related GPR, member A2A	668727	https://www.ncbi.nlm.nih.gov/gene/?term=668727
Trim32	Trim32	tripartite motif-containing 32	69807	https://www.ncbi.nlm.nih.gov/gene/?term=69807
Washc4	Washc4	WASH complex subunit 4	319277	https://www.ncbi.nlm.nih.gov/gene/?term=319277
Bach1	Bach1	BTB and CNC homology 1, basic leucine zipper transcription factor 1	12013	https://www.ncbi.nlm.nih.gov/gene/?term=12013
Traf3	Traf3	TNF receptor-associated factor 3	22031	https://www.ncbi.nlm.nih.gov/gene/?term=22031

Carhsp1	Carhsp1	calcium regulated heat stable protein 1	52502	https://www.ncbi.nlm.nih.gov/gene/?term=52502
Tle4	Tle4	transducin-like enhancer of split 4	21888	https://www.ncbi.nlm.nih.gov/gene/?term=21888
Ikbke	Ikbke	inhibitor of kappaB kinase epsilon	56489	https://www.ncbi.nlm.nih.gov/gene/?term=56489
Tspan14	Tspan14	tetraspanin 14	52588	https://www.ncbi.nlm.nih.gov/gene/?term=52588
Boc	Boc	biregional cell adhesion molecule-related/down-regulated by oncogenes (Cdon) binding protein	117606	https://www.ncbi.nlm.nih.gov/gene/?term=117606
Arntl	Arntl	aryl hydrocarbon receptor nuclear translocator-like	11865	https://www.ncbi.nlm.nih.gov/gene/?term=11865
1810046K07Rik	1810046K07Rik	RIKEN cDNA 1810046K07 gene	69809	https://www.ncbi.nlm.nih.gov/gene/?term=69809
Trim75	Trim75	tripartite motif-containing 75	333307	https://www.ncbi.nlm.nih.gov/gene/?term=333307
Pigm	Pigm	phosphatidylinositol glycan anchor biosynthesis, class M	67556	https://www.ncbi.nlm.nih.gov/gene/?term=67556

Biology Open • Supplementary information

Inpp5d	Inpp5d	inositol polyphosphate-5-phosphatase D	16331	https://www.ncbi.nlm.nih.gov/gene/?term=16331
Ndn	Ndn	necdin	17984	https://www.ncbi.nlm.nih.gov/gene/?term=17984
Csnk1g2	Csnk1g2	casein kinase 1, gamma 2	103236	https://www.ncbi.nlm.nih.gov/gene/?term=103236
Zfp407	Zfp407	zinc finger protein 407	240476	https://www.ncbi.nlm.nih.gov/gene/?term=240476
Fgf7	Fgf7	fibroblast growth factor 7	14178	https://www.ncbi.nlm.nih.gov/gene/?term=14178
Cacul1	Cacul1	CDK2 associated, cullin domain 1	78832	https://www.ncbi.nlm.nih.gov/gene/?term=78832
Hivep2	Hivep2	human immunodeficiency virus type I enhancer binding protein 2	15273	https://www.ncbi.nlm.nih.gov/gene/?term=15273
Sdcbp	Sdcbp	syndecan binding protein	53378	https://www.ncbi.nlm.nih.gov/gene/?term=53378
Actl7a	Actl7a	actin-like 7a	11470	https://www.ncbi.nlm.nih.gov/gene/?term=11470

Det1	Det1	de-etiolated homolog 1 (Arabidopsis)	76375	https://www.ncbi.nlm.nih.gov/gene/?term=76375
Mrgpra2b	Mrgpra2b	MAS-related GPR, member A2B	235712	https://www.ncbi.nlm.nih.gov/gene/?term=235712
Tenm3	Tenm3	teneurin transmembrane protein 3	23965	https://www.ncbi.nlm.nih.gov/gene/?term=23965
Dph7	Dph7	diphthamine biosynethesis 7	67228	https://www.ncbi.nlm.nih.gov/gene/?term=67228
Zfp652	Zfp652	zinc finger protein 652	268469	https://www.ncbi.nlm.nih.gov/gene/?term=268469
Jpt2	Jpt2	Jupiter microtubule associated homolog 2	52009	https://www.ncbi.nlm.nih.gov/gene/?term=52009
Aicda	Aicda	activation-induced cytidine deaminase	11628	https://www.ncbi.nlm.nih.gov/gene/?term=11628
Rcn2	Rcn2	reticulocalbin 2	26611	https://www.ncbi.nlm.nih.gov/gene/?term=26611
Senp7	Senp7	SUMO1/sentrin specific peptidase 7	66315	https://www.ncbi.nlm.nih.gov/gene/?term=66315

Biology Open • Supplementary information

Mdh1b	Mdh1b	malate dehydrogenase 1B, NAD (soluble)	76668	https://www.ncbi.nlm.nih.gov/gene/?term=76668
Rcbtb1	Rcbtb1	regulator of chromosome condensation (RCC1) and BTB (POZ) domain containing protein 1	71330	https://www.ncbi.nlm.nih.gov/gene/?term=71330
Mgrn1	Mgrn1	mahogunin, ring finger 1	17237	https://www.ncbi.nlm.nih.gov/gene/?term=17237
Sdha	Sdha	succinate dehydrogenase complex, subunit A, flavoprotein (Fp)	66945	https://www.ncbi.nlm.nih.gov/gene/?term=66945
Gm3286	Gm3286	predicted gene 3286	100041354	https://www.ncbi.nlm.nih.gov/gene/?term=100041
Spag6l	Spag6l	sperm associated antigen 6-like	50525	https://www.ncbi.nlm.nih.gov/gene/?term=50525
Selenot	Selenot	selenoprotein T	69227	https://www.ncbi.nlm.nih.gov/gene/?term=69227
Chn1	Chn1	chimerin 1	108699	https://www.ncbi.nlm.nih.gov/gene/?term=108699
Fut8	Fut8	fucosyltransferase 8	53618	https://www.ncbi.nlm.nih.gov/gene/?term=53618

Biology Open • Supplementary information

Slc35f5	Slc35f5	solute carrier family 35, member F5	74150	https://www.ncbi.nlm.nih.gov/gene/?term=74150
Lmod3	Lmod3	leiomodin 3 (fetal)	320502	https://www.ncbi.nlm.nih.gov/gene/?term=320502
Lamc1	Lamc1	laminin, gamma 1	226519	https://www.ncbi.nlm.nih.gov/gene/?term=226519
Cep55	Cep55	centrosomal protein 55	74107	https://www.ncbi.nlm.nih.gov/gene/?term=74107
Invs	Invs	inversin	16348	https://www.ncbi.nlm.nih.gov/gene/?term=16348
Lrch3	Lrch3	leucine-rich repeats and calponin homology (CH) domain containing 3	70144	https://www.ncbi.nlm.nih.gov/gene/?term=70144
Cltc	Cltc	clathrin, heavy polypeptide (Hc)	67300	https://www.ncbi.nlm.nih.gov/gene/?term=67300
Ctps2	Ctps2	cytidine 5'-triphosphate synthase 2	55936	https://www.ncbi.nlm.nih.gov/gene/?term=55936
Slc35a1	Slc35a1	solute carrier family 35 (CMP-sialic acid transporter), member 1	24060	https://www.ncbi.nlm.nih.gov/gene/?term=24060

Biology Open • Supplementary information

Lrrk2	Lrrk2	leucine-rich repeat kinase 2	66725	https://www.ncbi.nlm.nih.gov/gene/?term=66725
Sh2d4a	Sh2d4a	SH2 domain containing 4A	72281	https://www.ncbi.nlm.nih.gov/gene/?term=72281
Rab11fip1	Rab11fip1	RAB11 family interacting protein 1 (class I)	75767	https://www.ncbi.nlm.nih.gov/gene/?term=75767
Dusp7	Dusp7	dual specificity phosphatase 7	235584	https://www.ncbi.nlm.nih.gov/gene/?term=235584
Sbf2	Sbf2	SET binding factor 2	319934	https://www.ncbi.nlm.nih.gov/gene/?term=319934
Frk	Frk	fyn-related kinase	14302	https://www.ncbi.nlm.nih.gov/gene/?term=14302
4921517D22Rik	4921517D22Rik	RIKEN cDNA 4921517D22 gene	70900	https://www.ncbi.nlm.nih.gov/gene/?term=70900
E330014E10Rik	E330014E10Rik	RIKEN cDNA E330014E10 gene	665943	https://www.ncbi.nlm.nih.gov/gene/?term=665943
Wnk3	Wnk3	WNK lysine deficient protein kinase 3	279561	https://www.ncbi.nlm.nih.gov/gene/?term=279561

Biology Open • Supplementary information

Lrp1	Lrp1	low density lipoprotein receptor-related protein 1	16971	https://www.ncbi.nlm.nih.gov/gene/?term=16971
Ccser1	Ccser1	coiled-coil serine rich 1	232035	https://www.ncbi.nlm.nih.gov/gene/?term=232035
Trappc6b	Trappc6b	trafficking protein particle complex 6B	78232	https://www.ncbi.nlm.nih.gov/gene/?term=78232
Zfp420	Zfp420	zinc finger protein 420	233058	https://www.ncbi.nlm.nih.gov/gene/?term=233058
Asxl2	Asxl2	additional sex combs like 2, transcriptional regulator	75302	https://www.ncbi.nlm.nih.gov/gene/?term=75302
Mttp	Mttp	microsomal triglyceride transfer protein	17777	https://www.ncbi.nlm.nih.gov/gene/?term=17777
Csde1	Csde1	cold shock domain containing E1, RNA binding	229663	https://www.ncbi.nlm.nih.gov/gene/?term=229663
Rarres1	Rarres1	retinoic acid receptor responder (tazarotene induced) 1	109222	https://www.ncbi.nlm.nih.gov/gene/?term=109222
Tsc1	Tsc1	tuberous sclerosis 1	64930	https://www.ncbi.nlm.nih.gov/gene/?term=64930

Kcnab1	Kcnab1	potassium voltage-gated channel, shaker-related subfamily, beta member 1	16497	https://www.ncbi.nlm.nih.gov/gene/?term=16497
Pxdn	Pxdn	peroxidasin	69675	https://www.ncbi.nlm.nih.gov/gene/?term=69675
Slc44a4	Slc44a4	solute carrier family 44, member 4	70129	https://www.ncbi.nlm.nih.gov/gene/?term=70129
Ptgs1	Ptgs1	prostaglandin-endoperoxide synthase 1	19224	https://www.ncbi.nlm.nih.gov/gene/?term=19224
Tspan5	Tspan5	tetraspanin 5	56224	https://www.ncbi.nlm.nih.gov/gene/?term=56224
Mex3b	Mex3b	mex3 RNA binding family member B	108797	https://www.ncbi.nlm.nih.gov/gene/?term=108797
Slco1c1	Slco1c1	solute carrier organic anion transporter family, member 1c1	58807	https://www.ncbi.nlm.nih.gov/gene/?term=58807
Prkci	Prkci	protein kinase C, iota	18759	https://www.ncbi.nlm.nih.gov/gene/?term=18759
Foxj3	Foxj3	forkhead box J3	230700	https://www.ncbi.nlm.nih.gov/gene/?term=230700

Biology Open • Supplementary information

Stx7	Stx7	syntaxin 7	53331	https://www.ncbi.nlm.nih.gov/gene/?term=53331
Matr3	Matr3	matrin 3	17184	https://www.ncbi.nlm.nih.gov/gene/?term=17184
Cks2	Cks2	CDC28 protein kinase regulatory subunit 2	66197	https://www.ncbi.nlm.nih.gov/gene/?term=66197
Ccdc146	Ccdc146	coiled-coil domain containing 146	75172	https://www.ncbi.nlm.nih.gov/gene/?term=75172
Plekhh1	Plekhh1	pleckstrin homology domain containing, family H (with MyTH4 domain) member 1	211945	https://www.ncbi.nlm.nih.gov/gene/?term=211945
Trpa1	Trpa1	transient receptor potential cation channel, subfamily A, member 1	277328	https://www.ncbi.nlm.nih.gov/gene/?term=277328
Kif1b	Kif1b	kinesin family member 1B	16561	https://www.ncbi.nlm.nih.gov/gene/?term=16561
Tbpl1	Tbpl1	TATA box binding protein-like 1	237336	https://www.ncbi.nlm.nih.gov/gene/?term=237336
Rasgef1b	Rasgef1b	RasGEF domain family, member 1B	320292	https://www.ncbi.nlm.nih.gov/gene/?term=320292

Biology Open • Supplementary information

Son	Son	Son DNA binding protein	20658	https://www.ncbi.nlm.nih.gov/gene/?term=20658
Crebl2	Crebl2	cAMP responsive element binding protein-like 2	232430	https://www.ncbi.nlm.nih.gov/gene/?term=232430
Tll1	Tll1	tolloid-like	21892	https://www.ncbi.nlm.nih.gov/gene/?term=21892
Amotl1	Amotl1	angiomotin-like 1	75723	https://www.ncbi.nlm.nih.gov/gene/?term=75723
Tlk1	Tlk1	tousled-like kinase 1	228012	https://www.ncbi.nlm.nih.gov/gene/?term=228012
Zfp622	Zfp622	zinc finger protein 622	52521	https://www.ncbi.nlm.nih.gov/gene/?term=52521
Ccne1	Ccne1	cyclin E1	12447	https://www.ncbi.nlm.nih.gov/gene/?term=12447
Ahcyl2	Ahcyl2	S-adenosylhomocysteine hydrolase-like 2	74340	https://www.ncbi.nlm.nih.gov/gene/?term=74340
Omg	Omg	oligodendrocyte myelin glycoprotein	18377	https://www.ncbi.nlm.nih.gov/gene/?term=18377

Biology Open • Supplementary information

Mfn2	Mfn2	mitofusin 2	170731	https://www.ncbi.nlm.nih.gov/gene/?term=170731
Pacsin2	Pacsin2	protein kinase C and casein kinase substrate in neurons 2	23970	https://www.ncbi.nlm.nih.gov/gene/?term=23970
Nol4l	Nol4l	nucleolar protein 4-like	329540	https://www.ncbi.nlm.nih.gov/gene/?term=329540
Atxn2	Atxn2	ataxin 2	20239	https://www.ncbi.nlm.nih.gov/gene/?term=20239
Higd1a	Higd1a	HIG1 domain family, member 1A	56295	https://www.ncbi.nlm.nih.gov/gene/?term=56295
Usp15	Usp15	ubiquitin specific peptidase 15	14479	https://www.ncbi.nlm.nih.gov/gene/?term=14479
Pwwp2b	Pwwp2b	PWWP domain containing 2B	101631	https://www.ncbi.nlm.nih.gov/gene/?term=101631
Sez6l	Sez6l	seizure related 6 homolog like	56747	https://www.ncbi.nlm.nih.gov/gene/?term=56747
Casr	Casr	calcium-sensing receptor	12374	https://www.ncbi.nlm.nih.gov/gene/?term=12374

Biology Open • Supplementary information

Trp53inp2	Trp53inp2	transformation related protein 53 inducible nuclear protein 2	68728	https://www.ncbi.nlm.nih.gov/gene/?term=68728
Gbp2b	Gbp2b	guanylate binding protein 2b	14468	https://www.ncbi.nlm.nih.gov/gene/?term=14468
Erlin2	Erlin2	ER lipid raft associated 2	244373	https://www.ncbi.nlm.nih.gov/gene/?term=244373
Ccnd2	Ccnd2	cyclin D2	12444	https://www.ncbi.nlm.nih.gov/gene/?term=12444
Armcx6	Armcx6	armadillo repeat containing, X-linked 6	278097	https://www.ncbi.nlm.nih.gov/gene/?term=278097
Adgrl1	Adgrl1	adhesion G protein-coupled receptor L1	330814	https://www.ncbi.nlm.nih.gov/gene/?term=330814
Sall4	Sall4	spalt like transcription factor 4	99377	https://www.ncbi.nlm.nih.gov/gene/?term=99377
Apln	Apln	apelin	30878	https://www.ncbi.nlm.nih.gov/gene/?term=30878
Cpsf7	Cpsf7	cleavage and polyadenylation specific factor 7	269061	https://www.ncbi.nlm.nih.gov/gene/?term=269061

Biology Open • Supplementary information

Plxna4	Plxna4	plexin A4	243743	https://www.ncbi.nlm.nih.gov/gene/?term=243743
Ash1l	Ash1l	ASH1 like histone lysine methyltransferase	192195	https://www.ncbi.nlm.nih.gov/gene/?term=192195
Ubfd1	Ubfd1	ubiquitin family domain containing 1	28018	https://www.ncbi.nlm.nih.gov/gene/?term=28018
Spryd3	Spryd3	SPRY domain containing 3	223918	https://www.ncbi.nlm.nih.gov/gene/?term=223918
Nfatc3	Nfatc3	nuclear factor of activated T cells, cytoplasmic, calcineurin dependent 3	18021	https://www.ncbi.nlm.nih.gov/gene/?term=18021
Actr2	Actr2	ARP2 actin-related protein 2	66713	https://www.ncbi.nlm.nih.gov/gene/?term=66713
Tmem74b	Tmem74b	transmembrane protein 74B	108832	https://www.ncbi.nlm.nih.gov/gene/?term=108832
Usp31	Usp31	ubiquitin specific peptidase 31	76179	https://www.ncbi.nlm.nih.gov/gene/?term=76179
1700025G04Rik	1700025G04Rik	RIKEN cDNA 1700025G04 gene	69399	https://www.ncbi.nlm.nih.gov/gene/?term=69399

Biology Open • Supplementary information

Zbtb39	Zbtb39	zinc finger and BTB domain containing 39	320080	https://www.ncbi.nlm.nih.gov/gene/?term=320080
Wnt7a	Wnt7a	wingless-type MMTV integration site family, member 7A	22421	https://www.ncbi.nlm.nih.gov/gene/?term=22421
Setd3	Setd3	SET domain containing 3	52690	https://www.ncbi.nlm.nih.gov/gene/?term=52690
Eif3a	Eif3a	eukaryotic translation initiation factor 3, subunit A	13669	https://www.ncbi.nlm.nih.gov/gene/?term=13669
Ube4b	Ube4b	ubiquitination factor E4B	63958	https://www.ncbi.nlm.nih.gov/gene/?term=63958
Unc80	Unc80	unc-80, NALCN activator	329178	https://www.ncbi.nlm.nih.gov/gene/?term=329178
Mex3c	Mex3c	mex3 RNA binding family member C	240396	https://www.ncbi.nlm.nih.gov/gene/?term=240396
Entpd7	Entpd7	ectonucleoside triphosphate diphosphohydrolase 7	93685	https://www.ncbi.nlm.nih.gov/gene/?term=93685
Ghr	Ghr	growth hormone receptor	14600	https://www.ncbi.nlm.nih.gov/gene/?term=14600

Biology Open • Supplementary information

Ccdc6	Ccdc6	coiled-coil domain containing 6	76551	https://www.ncbi.nlm.nih.gov/gene/?term=76551
Usp14	Usp14	ubiquitin specific peptidase 14	59025	https://www.ncbi.nlm.nih.gov/gene/?term=59025
Cdc37l1	Cdc37l1	cell division cycle 37-like 1	67072	https://www.ncbi.nlm.nih.gov/gene/?term=67072
Krtap11-1	Krtap11-1	keratin associated protein 11-1	16693	https://www.ncbi.nlm.nih.gov/gene/?term=16693
Ncs1	Ncs1	neuronal calcium sensor 1	14299	https://www.ncbi.nlm.nih.gov/gene/?term=14299
Syde2	Syde2	synapse defective 1, Rho GTPase, homolog 2 (C. elegans)	214804	https://www.ncbi.nlm.nih.gov/gene/?term=214804
Smad7	Smad7	SMAD family member 7	17131	https://www.ncbi.nlm.nih.gov/gene/?term=17131
Polr3f	Polr3f	polymerase (RNA) III (DNA directed) polypeptide F	70408	https://www.ncbi.nlm.nih.gov/gene/?term=70408
Cdca4	Cdca4	cell division cycle associated 4	71963	https://www.ncbi.nlm.nih.gov/gene/?term=71963

Biology Open • Supplementary information

Arfgap2	Arfgap2	ADP-ribosylation factor GTPase activating protein 2	77038	https://www.ncbi.nlm.nih.gov/gene/?term=77038
Ptprr	Ptprr	protein tyrosine phosphatase, receptor type, R	19279	https://www.ncbi.nlm.nih.gov/gene/?term=19279
Akap7	Akap7	A kinase (PRKA) anchor protein 7	432442	https://www.ncbi.nlm.nih.gov/gene/?term=432442
Il7r	Il7r	interleukin 7 receptor	16197	https://www.ncbi.nlm.nih.gov/gene/?term=16197
Ret	Ret	ret proto-oncogene	19713	https://www.ncbi.nlm.nih.gov/gene/?term=19713
Rab10	Rab10	RAB10, member RAS oncogene family	19325	https://www.ncbi.nlm.nih.gov/gene/?term=19325
Eya1	Eya1	EYA transcriptional coactivator and phosphatase 1	14048	https://www.ncbi.nlm.nih.gov/gene/?term=14048
Fgf9	Fgf9	fibroblast growth factor 9	14180	https://www.ncbi.nlm.nih.gov/gene/?term=14180
Kif5c	Kif5c	kinesin family member 5C	16574	https://www.ncbi.nlm.nih.gov/gene/?term=16574

Krtap26-1	Krtap26-1	keratin associated protein 26-1	69533	https://www.ncbi.nlm.nih.gov/gene/?term=69533
Arhgdia	Arhgdia	Rho GDP dissociation inhibitor (GDI) alpha	192662	https://www.ncbi.nlm.nih.gov/gene/?term=192662
Pip4p2	Pip4p2	phosphatidylinositol-4,5-bisphosphate 4-phosphatase 2	72519	https://www.ncbi.nlm.nih.gov/gene/?term=72519
Arl2	Arl2	ADP-ribosylation factor-like 2	56327	https://www.ncbi.nlm.nih.gov/gene/?term=56327
Kbtbd2	Kbtbd2	kelch repeat and BTB (POZ) domain containing 2	210973	https://www.ncbi.nlm.nih.gov/gene/?term=210973
Dnajc16	Dnajc16	DnaJ heat shock protein family (Hsp40) member C16	214063	https://www.ncbi.nlm.nih.gov/gene/?term=214063
Dcaf7	Dcaf7	DDB1 and CUL4 associated factor 7	71833	https://www.ncbi.nlm.nih.gov/gene/?term=71833
Atp1b4	Atp1b4	ATPase, (Na+)/K+ transporting, beta 4 polypeptide	67821	https://www.ncbi.nlm.nih.gov/gene/?term=67821
Atf6	Atf6	activating transcription factor 6	226641	https://www.ncbi.nlm.nih.gov/gene/?term=226641

Kcnj2	Kcnj2	potassium inwardly-rectifying channel, subfamily J, member 2	16518	https://www.ncbi.nlm.nih.gov/gene/?term=16518
Lats1	Lats1	large tumor suppressor	16798	https://www.ncbi.nlm.nih.gov/gene/?term=16798
Pnpla6	Pnpla6	patatin-like phospholipase domain containing 6	50767	https://www.ncbi.nlm.nih.gov/gene/?term=50767
Sptbn2	Sptbn2	spectrin beta, non-erythrocytic 2	20743	https://www.ncbi.nlm.nih.gov/gene/?term=20743
Wnt3a	Wnt3a	wingless-type MMTV integration site family, member 3A	22416	https://www.ncbi.nlm.nih.gov/gene/?term=22416
Luzp1	Luzp1	leucine zipper protein 1	269593	https://www.ncbi.nlm.nih.gov/gene/?term=269593
Sesn1	Sesn1	sestrin 1	140742	https://www.ncbi.nlm.nih.gov/gene/?term=140742
Hmga1	Hmga1	high mobility group AT-hook 1	15361	https://www.ncbi.nlm.nih.gov/gene/?term=15361
Sec14l1	Sec14l1	SEC14-like lipid binding 1	74136	https://www.ncbi.nlm.nih.gov/gene/?term=74136

Biology Open • Supplementary information

Akt3	Akt3	thymoma viral proto-oncogene 3	23797	https://www.ncbi.nlm.nih.gov/gene/?term=23797
Zbtb40	Zbtb40	zinc finger and BTB domain containing 40	230848	https://www.ncbi.nlm.nih.gov/gene/?term=230848
Rubcn	Rubcn	RUN domain and cysteine-rich domain containing, Beclin 1-interacting protein	100502698	https://www.ncbi.nlm.nih.gov/gene/?term=100502
Zfand3	Zfand3	zinc finger, AN1-type domain 3	21769	https://www.ncbi.nlm.nih.gov/gene/?term=21769
Fbln5	Fbln5	fibulin 5	23876	https://www.ncbi.nlm.nih.gov/gene/?term=23876
Tspan11	Tspan11	tetraspanin 11	68498	https://www.ncbi.nlm.nih.gov/gene/?term=68498
Pim1	Pim1	proviral integration site 1	18712	https://www.ncbi.nlm.nih.gov/gene/?term=18712
Rapgefl1	Rapgefl1	Rap guanine nucleotide exchange factor (GEF)-like 1	268480	https://www.ncbi.nlm.nih.gov/gene/?term=268480
Ammecr1l	Ammecr1l	AMME chromosomal region gene 1-like	225339	https://www.ncbi.nlm.nih.gov/gene/?term=225339

Biology Open • Supplementary information

Gas7	Gas7	growth arrest specific 7	14457	https://www.ncbi.nlm.nih.gov/gene/?term=14457
Pdcl2	Pdcl2	phosducin-like 2	79455	https://www.ncbi.nlm.nih.gov/gene/?term=79455
Rexo2	Rexo2	RNA exonuclease 2	104444	https://www.ncbi.nlm.nih.gov/gene/?term=104444
Slc16a3	Slc16a3	solute carrier family 16 (monocarboxylic acid transporters), member 3	80879	https://www.ncbi.nlm.nih.gov/gene/?term=80879
Zfp710	Zfp710	zinc finger protein 710	209225	https://www.ncbi.nlm.nih.gov/gene/?term=209225
Pdrg1	Pdrg1	p53 and DNA damage regulated 1	68559	https://www.ncbi.nlm.nih.gov/gene/?term=68559
Prr14l	Prr14l	proline rich 14-like	215476	https://www.ncbi.nlm.nih.gov/gene/?term=215476
Mllt10	Mllt10	myeloid/lymphoid or mixed-lineage leukemia; translocated to, 10	17354	https://www.ncbi.nlm.nih.gov/gene/?term=17354
Pcbp2	Pcbp2	poly(rC) binding protein 2	18521	https://www.ncbi.nlm.nih.gov/gene/?term=18521

Biology Open • Supplementary information

Rcsd1	Rcsd1	RCSD domain containing 1	226594	https://www.ncbi.nlm.nih.gov/gene/?term=226594
Afmid	Afmid	arylformamidase	71562	https://www.ncbi.nlm.nih.gov/gene/?term=71562
0610010K14Rik	0610010K14Rik	RIKEN cDNA 0610010K14 gene	104457	https://www.ncbi.nlm.nih.gov/gene/?term=104457
Rasgef1c	Rasgef1c	RasGEF domain family, member 1C	74563	https://www.ncbi.nlm.nih.gov/gene/?term=74563
Stx8	Stx8	syntaxin 8	55943	https://www.ncbi.nlm.nih.gov/gene/?term=55943
Lsm12	Lsm12	LSM12 homolog	268490	https://www.ncbi.nlm.nih.gov/gene/?term=268490
Nfatc2	Nfatc2	nuclear factor of activated T cells, cytoplasmic, calcineurin dependent 2	18019	https://www.ncbi.nlm.nih.gov/gene/?term=18019
Enox2	Enox2	ecto-NOX disulfide-thiol exchanger 2	209224	https://www.ncbi.nlm.nih.gov/gene/?term=209224
Fchsd1	Fchsd1	FCH and double SH3 domains 1	319262	https://www.ncbi.nlm.nih.gov/gene/?term=319262

Biology Open • Supplementary information

Rexo1	Rexo1	REX1, RNA exonuclease 1	66932	https://www.ncbi.nlm.nih.gov/gene/?term=66932
Cbl	Cbl	Casitas B-lineage lymphoma	12402	https://www.ncbi.nlm.nih.gov/gene/?term=12402
Il17rd	Il17rd	interleukin 17 receptor D	171463	https://www.ncbi.nlm.nih.gov/gene/?term=171463
Myoz3	Myoz3	myozenin 3	170947	https://www.ncbi.nlm.nih.gov/gene/?term=170947
Myo18a	Myo18a	myosin XVIIIA	360013	https://www.ncbi.nlm.nih.gov/gene/?term=360013
Cetn2	Cetn2	centrin 2	26370	https://www.ncbi.nlm.nih.gov/gene/?term=26370
Kif7	Kif7	kinesin family member 7	16576	https://www.ncbi.nlm.nih.gov/gene/?term=16576
1700010I14Rik	1700010I14Rik	RIKEN cDNA 1700010I14 gene	66931	https://www.ncbi.nlm.nih.gov/gene/?term=66931
Exoc3	Exoc3	exocyst complex component 3	211446	https://www.ncbi.nlm.nih.gov/gene/?term=211446

Biology Open • Supplementary information

Peli3	Peli3	pellino 3	240518	https://www.ncbi.nlm.nih.gov/gene/?term=240518
Lrrc1	Lrrc1	leucine rich repeat containing 1	214345	https://www.ncbi.nlm.nih.gov/gene/?term=214345
Fgfr1	Fgfr1	fibroblast growth factor receptor 1	14182	https://www.ncbi.nlm.nih.gov/gene/?term=14182
Clint1	Clint1	clathrin interactor 1	216705	https://www.ncbi.nlm.nih.gov/gene/?term=216705
Dolpp1	Dolpp1	dolichyl pyrophosphate phosphatase 1	57170	https://www.ncbi.nlm.nih.gov/gene/?term=57170
Usp20	Usp20	ubiquitin specific peptidase 20	74270	https://www.ncbi.nlm.nih.gov/gene/?term=74270
Fgf14	Fgf14	fibroblast growth factor 14	14169	https://www.ncbi.nlm.nih.gov/gene/?term=14169
Scp2d1	Scp2d1	SCP2 sterol-binding domain containing 1	66328	https://www.ncbi.nlm.nih.gov/gene/?term=66328
Zbtb10	Zbtb10	zinc finger and BTB domain containing 10	229055	https://www.ncbi.nlm.nih.gov/gene/?term=229055

Biology Open • Supplementary information

Snx12	Snx12	sorting nexin 12	55988	https://www.ncbi.nlm.nih.gov/gene/?term=55988
Pgf	Pgf	placental growth factor	18654	https://www.ncbi.nlm.nih.gov/gene/?term=18654
Adtrp	Adtrp	androgen dependent TFPI regulating protein	109254	https://www.ncbi.nlm.nih.gov/gene/?term=109254
Mapt	Mapt	microtubule-associated protein tau	17762	https://www.ncbi.nlm.nih.gov/gene/?term=17762
Evi2	Evi2	ecotropic viral integration site 2	101488212	https://www.ncbi.nlm.nih.gov/gene/?term=101488
Cat	Cat	catalase	12359	https://www.ncbi.nlm.nih.gov/gene/?term=12359
Cd226	Cd226	CD226 antigen	225825	https://www.ncbi.nlm.nih.gov/gene/?term=225825
Dpp7	Dpp7	dipeptidylpeptidase 7	83768	https://www.ncbi.nlm.nih.gov/gene/?term=83768
Gm7073	Gm7073	predicted gene 7073	631784	https://www.ncbi.nlm.nih.gov/gene/?term=631784

Biology Open • Supplementary information

Lrfn5	Lrfn5	leucine rich repeat and fibronectin type III domain containing 5	238205	https://www.ncbi.nlm.nih.gov/gene/?term=238205
Evi2b	Evi2b	ecotropic viral integration site 2b	216984	https://www.ncbi.nlm.nih.gov/gene/?term=216984
Tln2	Tln2	talin 2	70549	https://www.ncbi.nlm.nih.gov/gene/?term=70549
Anks1	Anks1	ankyrin repeat and SAM domain containing 1	224650	https://www.ncbi.nlm.nih.gov/gene/?term=224650
Smg1	Smg1	SMG1 homolog, phosphatidylinositol 3-kinase-related kinase (C. elegans)	233789	https://www.ncbi.nlm.nih.gov/gene/?term=233789
Impdh1	Impdh1	inosine monophosphate dehydrogenase 1	23917	https://www.ncbi.nlm.nih.gov/gene/?term=23917
Hspa9	Hspa9	heat shock protein 9	15526	https://www.ncbi.nlm.nih.gov/gene/?term=15526
Ppm1l	Ppm1l	protein phosphatase 1 (formerly 2C)-like	242083	https://www.ncbi.nlm.nih.gov/gene/?term=242083
Ezh2	Ezh2	enhancer of zeste 2 polycomb repressive complex 2 subunit	14056	https://www.ncbi.nlm.nih.gov/gene/?term=14056

Slc9a9	Slc9a9	solute carrier family 9 (sodium/hydrogen exchanger), member 9	331004	https://www.ncbi.nlm.nih.gov/gene/?term=331004
Trim46	Trim46	tripartite motif-containing 46	360213	https://www.ncbi.nlm.nih.gov/gene/?term=360213
Psma1	Psma1	proteasome (prosome, macropain) subunit, alpha type 1	26440	https://www.ncbi.nlm.nih.gov/gene/?term=26440
Zfp790	Zfp790	zinc finger protein 790	233056	https://www.ncbi.nlm.nih.gov/gene/?term=233056
Srsf11	Srsf11	serine/arginine-rich splicing factor 11	69207	https://www.ncbi.nlm.nih.gov/gene/?term=69207
Ptpre	Ptpre	protein tyrosine phosphatase, receptor type, E	19267	https://www.ncbi.nlm.nih.gov/gene/?term=19267
Gabrg2	Gabrg2	gamma-aminobutyric acid (GABA) A receptor, subunit gamma 2	14406	https://www.ncbi.nlm.nih.gov/gene/?term=14406
Smg7	Smg7	Smg-7 homolog, nonsense mediated mRNA decay factor (C. elegans)	226517	https://www.ncbi.nlm.nih.gov/gene/?term=226517
Nbl1	Nbl1	neuroblastoma, suppression of tumorigenicity 1	17965	https://www.ncbi.nlm.nih.gov/gene/?term=17965

Biology Open • Supplementary information

Frat1	Frat1	frequently rearranged in advanced T cell lymphomas	14296	https://www.ncbi.nlm.nih.gov/gene/?term=14296
Olfm1	Olfm1	olfactomedin 1	56177	https://www.ncbi.nlm.nih.gov/gene/?term=56177
Tmem178b	Tmem178b	transmembrane protein 178B	434008	https://www.ncbi.nlm.nih.gov/gene/?term=434008
Tpd52l2	Tpd52l2	tumor protein D52-like 2	66314	https://www.ncbi.nlm.nih.gov/gene/?term=66314
Gtpbp2	Gtpbp2	GTP binding protein 2	56055	https://www.ncbi.nlm.nih.gov/gene/?term=56055
Arhgap18	Arhgap18	Rho GTPase activating protein 18	73910	https://www.ncbi.nlm.nih.gov/gene/?term=73910
Cavin1	Cavin1	caveolae associated 1	19285	https://www.ncbi.nlm.nih.gov/gene/?term=19285
Spsb1	Spsb1	splA/ryanodine receptor domain and SOCS box containing 1	74646	https://www.ncbi.nlm.nih.gov/gene/?term=74646
Leprotl1	Leprotl1	leptin receptor overlapping transcript-like 1	68192	https://www.ncbi.nlm.nih.gov/gene/?term=68192

Biology Open • Supplementary information

Garem1	Garem1	GRB2 associated regulator of MAPK1 subtype 1	381126	https://www.ncbi.nlm.nih.gov/gene/?term=381126
Amph	Amph	amphiphysin	218038	https://www.ncbi.nlm.nih.gov/gene/?term=218038
Gab2	Gab2	growth factor receptor bound protein 2-associated protein 2	14389	https://www.ncbi.nlm.nih.gov/gene/?term=14389
Ndor1	Ndor1	NADPH dependent diflavin oxidoreductase 1	78797	https://www.ncbi.nlm.nih.gov/gene/?term=78797
Pacrg	Pacrg	PARK2 co-regulated	69310	https://www.ncbi.nlm.nih.gov/gene/?term=69310
Natd1	Natd1	N-acetyltransferase domain containing 1	24083	https://www.ncbi.nlm.nih.gov/gene/?term=24083
Slc25a12	Slc25a12	solute carrier family 25 (mitochondrial carrier, Aralar), member 12	78830	https://www.ncbi.nlm.nih.gov/gene/?term=78830
Tmod1	Tmod1	tropomodulin 1	21916	https://www.ncbi.nlm.nih.gov/gene/?term=21916
Macrod2	Macrod2	MACRO domain containing 2	72899	https://www.ncbi.nlm.nih.gov/gene/?term=72899

Biology Open • Supplementary information

Pabpc1	Pabpc1	poly(A) binding protein, cytoplasmic 1	18458	https://www.ncbi.nlm.nih.gov/gene/?term=18458
Usp2	Usp2	ubiquitin specific peptidase 2	53376	https://www.ncbi.nlm.nih.gov/gene/?term=53376
Hoxd1	Hoxd1	homeobox D1	15429	https://www.ncbi.nlm.nih.gov/gene/?term=15429
Kif1c	Kif1c	kinesin family member 1C	16562	https://www.ncbi.nlm.nih.gov/gene/?term=16562
Fam122a	Fam122a	family with sequence similarity 122, member A	68034	https://www.ncbi.nlm.nih.gov/gene/?term=68034
Ccnd1	Ccnd1	cyclin D1	12443	https://www.ncbi.nlm.nih.gov/gene/?term=12443
Lrrn3	Lrrn3	leucine rich repeat protein 3, neuronal	16981	https://www.ncbi.nlm.nih.gov/gene/?term=16981
Fech	Fech	ferrochelatase	14151	https://www.ncbi.nlm.nih.gov/gene/?term=14151
Tmem229a	Tmem229a	transmembrane protein 229A	319832	https://www.ncbi.nlm.nih.gov/gene/?term=319832

Biology Open • Supplementary information

Tnfrsf9	Tnfrsf9	tumor necrosis factor receptor superfamily, member 9	21942	https://www.ncbi.nlm.nih.gov/gene/?term=21942
Pum1	Pum1	pumilio RNA-binding family member 1	80912	https://www.ncbi.nlm.nih.gov/gene/?term=80912
Zfp641	Zfp641	zinc finger protein 641	239652	https://www.ncbi.nlm.nih.gov/gene/?term=239652
Pank4	Pank4	pantothenate kinase 4	269614	https://www.ncbi.nlm.nih.gov/gene/?term=269614
Ccpg1	Ccpg1	cell cycle progression 1	72278	https://www.ncbi.nlm.nih.gov/gene/?term=72278
Pla2g4a	Pla2g4a	phospholipase A2, group IVA (cytosolic, calcium-dependent)	18783	https://www.ncbi.nlm.nih.gov/gene/?term=18783
Gabrb3	Gabrb3	gamma-aminobutyric acid (GABA) A receptor, subunit beta 3	14402	https://www.ncbi.nlm.nih.gov/gene/?term=14402
Car10	Car10	carbonic anhydrase 10	72605	https://www.ncbi.nlm.nih.gov/gene/?term=72605
Plekha8	Plekha8	pleckstrin homology domain containing, family A (phosphoinositide binding specific) member 8	231999	https://www.ncbi.nlm.nih.gov/gene/?term=231999

Biology Open • Supplementary information

Enpp6	Enpp6	ectonucleotide pyrophosphatase/phosphodiesterase 6	320981	https://www.ncbi.nlm.nih.gov/gene/?term=320981
Cps1	Cps1	carbamoyl-phosphate synthetase 1	227231	https://www.ncbi.nlm.nih.gov/gene/?term=227231
Hnrnpa2b1	Hnrnpa2b1	heterogeneous nuclear ribonucleoprotein A2/B1	53379	https://www.ncbi.nlm.nih.gov/gene/?term=53379
Gnb4	Gnb4	guanine nucleotide binding protein (G protein), beta 4	14696	https://www.ncbi.nlm.nih.gov/gene/?term=14696
Ccdc125	Ccdc125	coiled-coil domain containing 125	76041	https://www.ncbi.nlm.nih.gov/gene/?term=76041
Ngfr	Ngfr	nerve growth factor receptor (TNFR superfamily, member 16)	18053	https://www.ncbi.nlm.nih.gov/gene/?term=18053
Golt1a	Golt1a	golgi transport 1A	68338	https://www.ncbi.nlm.nih.gov/gene/?term=68338
Vps33b	Vps33b	vacuolar protein sorting 33B	233405	https://www.ncbi.nlm.nih.gov/gene/?term=233405
Plag1	Plag1	pleiomorphic adenoma gene 1	56711	https://www.ncbi.nlm.nih.gov/gene/?term=56711

Biology Open • Supplementary information

Jakmip3	Jakmip3	janus kinase and microtubule interacting protein 3	74004	https://www.ncbi.nlm.nih.gov/gene/?term=74004
Atp2a2	Atp2a2	ATPase, Ca++ transporting, cardiac muscle, slow twitch 2	11938	https://www.ncbi.nlm.nih.gov/gene/?term=11938
Cartpt	Cartpt	CART prepropeptide	27220	https://www.ncbi.nlm.nih.gov/gene/?term=27220
Kcnq2	Kcnq2	potassium voltage-gated channel, subfamily Q, member 2	16536	https://www.ncbi.nlm.nih.gov/gene/?term=16536
Pfkfb2	Pfkfb2	6-phosphofructo-2-kinase/fructose-2,6-biphosphatase 2	18640	https://www.ncbi.nlm.nih.gov/gene/?term=18640
Treml2	Treml2	triggering receptor expressed on myeloid cells-like 2	328833	https://www.ncbi.nlm.nih.gov/gene/?term=328833
Cplx2	Cplx2	complexin 2	12890	https://www.ncbi.nlm.nih.gov/gene/?term=12890
Xkr6	Xkr6	X-linked Kx blood group related 6	219149	https://www.ncbi.nlm.nih.gov/gene/?term=219149
Cacfd1	Cacfd1	calcium channel flower domain containing 1	381356	https://www.ncbi.nlm.nih.gov/gene/?term=381356

Biology Open • Supplementary information

Ncam2	Ncam2	neural cell adhesion molecule 2	17968	https://www.ncbi.nlm.nih.gov/gene/?term=17968
Rock2	Rock2	Rho-associated coiled-coil containing protein kinase 2	19878	https://www.ncbi.nlm.nih.gov/gene/?term=19878
Wdr64	Wdr64	WD repeat domain 64	75820	https://www.ncbi.nlm.nih.gov/gene/?term=75820
Fuca2	Fuca2	fucosidase, alpha-L- 2, plasma	66848	https://www.ncbi.nlm.nih.gov/gene/?term=66848
Cacna1c	Cacna1c	calcium channel, voltage-dependent, L type, alpha 1C subunit	12288	https://www.ncbi.nlm.nih.gov/gene/?term=12288
Jag2	Jag2	jagged 2	16450	https://www.ncbi.nlm.nih.gov/gene/?term=16450
Gucy2f	Gucy2f	guanylate cyclase 2f	245650	https://www.ncbi.nlm.nih.gov/gene/?term=245650
Jph1	Jph1	junctophilin 1	57339	https://www.ncbi.nlm.nih.gov/gene/?term=57339
Pum2	Pum2	pumilio RNA-binding family member 2	80913	https://www.ncbi.nlm.nih.gov/gene/?term=80913

Biology Open • Supplementary information

Rpl22	Rpl22	ribosomal protein L22	19934	https://www.ncbi.nlm.nih.gov/gene/?term=19934
Lrif1	Lrif1	ligand dependent nuclear receptor interacting factor 1	321000	https://www.ncbi.nlm.nih.gov/gene/?term=321000
Trpm7	Trpm7	transient receptor potential cation channel, subfamily M, member 7	58800	https://www.ncbi.nlm.nih.gov/gene/?term=58800
Nmral1	Nmral1	NmrA-like family domain containing 1	67824	https://www.ncbi.nlm.nih.gov/gene/?term=67824
Grhl3	Grhl3	grainyhead like transcription factor 3	230824	https://www.ncbi.nlm.nih.gov/gene/?term=230824
Gtf2i	Gtf2i	general transcription factor II I	14886	https://www.ncbi.nlm.nih.gov/gene/?term=14886
Id1	Id1	inhibitor of DNA binding 1	15901	https://www.ncbi.nlm.nih.gov/gene/?term=15901
Tmem39a	Tmem39a	transmembrane protein 39a	67846	https://www.ncbi.nlm.nih.gov/gene/?term=67846
Actr3	Actr3	ARP3 actin-related protein 3	74117	https://www.ncbi.nlm.nih.gov/gene/?term=74117

Biology Open • Supplementary information

Vcpip1	Vcpip1	valosin containing protein (p97)/p47 complex interacting protein 1	70675	https://www.ncbi.nlm.nih.gov/gene/?term=70675
Apaf1	Apaf1	apoptotic peptidase activating factor 1	11783	https://www.ncbi.nlm.nih.gov/gene/?term=11783
Atp6v1h	Atp6v1h	ATPase, H+ transporting, lysosomal V1 subunit H	108664	https://www.ncbi.nlm.nih.gov/gene/?term=108664
Fbxo45	Fbxo45	F-box protein 45	268882	https://www.ncbi.nlm.nih.gov/gene/?term=268882
Kalrn	Kalrn	kalirin, RhoGEF kinase	545156	https://www.ncbi.nlm.nih.gov/gene/?term=545156
Prpf38b	Prpf38b	PRP38 pre-mRNA processing factor 38 (yeast) domain containing B	66921	https://www.ncbi.nlm.nih.gov/gene/?term=66921
Atad5	Atad5	ATPase family, AAA domain containing 5	237877	https://www.ncbi.nlm.nih.gov/gene/?term=237877
Zswim4	Zswim4	zinc finger SWIM-type containing 4	212168	https://www.ncbi.nlm.nih.gov/gene/?term=212168
Ank3	Ank3	ankyrin 3, epithelial	11735	https://www.ncbi.nlm.nih.gov/gene/?term=11735

Biology Open • Supplementary information

G2e3	G2e3	G2/M-phase specific E3 ubiquitin ligase	217558	https://www.ncbi.nlm.nih.gov/gene/?term=217558
Spock3	Spock3	sparc/osteonectin, cwcv and kazal-like domains proteoglycan 3	72902	https://www.ncbi.nlm.nih.gov/gene/?term=72902
Ano4	Ano4	anoctamin 4	320091	https://www.ncbi.nlm.nih.gov/gene/?term=320091
Dcc	Dcc	deleted in colorectal carcinoma	13176	https://www.ncbi.nlm.nih.gov/gene/?term=13176
Yy1	Yy1	YY1 transcription factor	22632	https://www.ncbi.nlm.nih.gov/gene/?term=22632
Crk	Crk	v-crk avian sarcoma virus CT10 oncogene homolog	12928	https://www.ncbi.nlm.nih.gov/gene/?term=12928
Tbc1d15	Tbc1d15	TBC1 domain family, member 15	66687	https://www.ncbi.nlm.nih.gov/gene/?term=66687
Fgfr2	Fgfr2	fibroblast growth factor receptor 2	14183	https://www.ncbi.nlm.nih.gov/gene/?term=14183
Mphosph9	Mphosph9	M-phase phosphoprotein 9	269702	https://www.ncbi.nlm.nih.gov/gene/?term=269702

Biology Open • Supplementary information

Cbln4	Cbln4	cerebellin 4 precursor protein	228942	https://www.ncbi.nlm.nih.gov/gene/?term=228942
Nfkbia	Nfkbia	nuclear factor of kappa light polypeptide gene enhancer in B cells inhibitor, alpha	18035	https://www.ncbi.nlm.nih.gov/gene/?term=18035
Med14	Med14	mediator complex subunit 14	26896	https://www.ncbi.nlm.nih.gov/gene/?term=26896
Casp8ap2	Casp8ap2	caspase 8 associated protein 2	26885	https://www.ncbi.nlm.nih.gov/gene/?term=26885
Lrrc4	Lrrc4	leucine rich repeat containing 4	192198	https://www.ncbi.nlm.nih.gov/gene/?term=192198
Cdk1	Cdk1	cyclin-dependent kinase 1	12534	https://www.ncbi.nlm.nih.gov/gene/?term=12534
Fbxo43	Fbxo43	F-box protein 43	78803	https://www.ncbi.nlm.nih.gov/gene/?term=78803
Mblac2	Mblac2	metallo-beta-lactamase domain containing 2	72852	https://www.ncbi.nlm.nih.gov/gene/?term=72852
Rabggtb	Rabggtb	Rab geranylgeranyl transferase, b subunit	19352	https://www.ncbi.nlm.nih.gov/gene/?term=19352

Biology Open • Supplementary information

Arpp21	Arpp21	cyclic AMP-regulated phosphoprotein, 21	74100	https://www.ncbi.nlm.nih.gov/gene/?term=74100
Dcun1d4	Dcun1d4	DCN1, defective in cullin neddylation 1, domain containing 4 (S. cerevisiae)	100737	https://www.ncbi.nlm.nih.gov/gene/?term=100737
Prr12	Prr12	proline rich 12	233210	https://www.ncbi.nlm.nih.gov/gene/?term=233210
P2ry10b	P2ry10b	purinergic receptor P2Y, G-protein coupled 10B	213438	https://www.ncbi.nlm.nih.gov/gene/?term=213438
Wac	Wac	WW domain containing adaptor with coiled-coil	225131	https://www.ncbi.nlm.nih.gov/gene/?term=225131
Lgr4	Lgr4	leucine-rich repeat-containing G protein-coupled receptor 4	107515	https://www.ncbi.nlm.nih.gov/gene/?term=107515
Nktr	Nktr	natural killer tumor recognition sequence	18087	https://www.ncbi.nlm.nih.gov/gene/?term=18087
Tusc3	Tusc3	tumor suppressor candidate 3	80286	https://www.ncbi.nlm.nih.gov/gene/?term=80286
Dll4	Dll4	delta like canonical Notch ligand 4	54485	https://www.ncbi.nlm.nih.gov/gene/?term=54485

Biology Open • Supplementary information

Gramd4	Gramd4	GRAM domain containing 4	223752	https://www.ncbi.nlm.nih.gov/gene/?term=223752
Foxd4	Foxd4	forkhead box D4	14237	https://www.ncbi.nlm.nih.gov/gene/?term=14237
Rnpc3	Rnpc3	RNA-binding region (RNP1, RRM) containing 3	67225	https://www.ncbi.nlm.nih.gov/gene/?term=67225
Ubr1	Ubr1	ubiquitin protein ligase E3 component n-recognin 1	22222	https://www.ncbi.nlm.nih.gov/gene/?term=22222
Pdap1	Pdap1	PDGFA associated protein 1	231887	https://www.ncbi.nlm.nih.gov/gene/?term=231887
Zfp715	Zfp715	zinc finger protein 715	69930	https://www.ncbi.nlm.nih.gov/gene/?term=69930
Ubn1	Ubn1	ubinuclein 1	170644	https://www.ncbi.nlm.nih.gov/gene/?term=170644
Kif11	Kif11	kinesin family member 11	16551	https://www.ncbi.nlm.nih.gov/gene/?term=16551
Lrp6	Lrp6	low density lipoprotein receptor-related protein 6	16974	https://www.ncbi.nlm.nih.gov/gene/?term=16974

Biology Open • Supplementary information

Trim63	Trim63	tripartite motif-containing 63	433766	https://www.ncbi.nlm.nih.gov/gene/?term=433766
Ppp1r15b	Ppp1r15b	protein phosphatase 1, regulatory subunit 15B	108954	https://www.ncbi.nlm.nih.gov/gene/?term=108954
Col11a1	Col11a1	collagen, type XI, alpha 1	12814	https://www.ncbi.nlm.nih.gov/gene/?term=12814
Insm2	Insm2	insulinoma-associated 2	56856	https://www.ncbi.nlm.nih.gov/gene/?term=56856
Abca8a	Abca8a	ATP-binding cassette, sub-family A (ABC1), member 8a	217258	https://www.ncbi.nlm.nih.gov/gene/?term=217258
Npy1r	Npy1r	neuropeptide Y receptor Y1	18166	https://www.ncbi.nlm.nih.gov/gene/?term=18166
Tmem252	Tmem252	transmembrane protein 252	226040	https://www.ncbi.nlm.nih.gov/gene/?term=226040
Chd6	Chd6	chromodomain helicase DNA binding protein 6	71389	https://www.ncbi.nlm.nih.gov/gene/?term=71389
Lrrc56	Lrrc56	leucine rich repeat containing 56	70552	https://www.ncbi.nlm.nih.gov/gene/?term=70552

Biology Open • Supplementary information

Trmt112	Trmt112	tRNA methyltransferase 11-2	67674	https://www.ncbi.nlm.nih.gov/gene/?term=67674
Insig1	Insig1	insulin induced gene 1	231070	https://www.ncbi.nlm.nih.gov/gene/?term=231070
Hdac8	Hdac8	histone deacetylase 8	70315	https://www.ncbi.nlm.nih.gov/gene/?term=70315
Tsg101	Tsg101	tumor susceptibility gene 101	22088	https://www.ncbi.nlm.nih.gov/gene/?term=22088
Plxdc2	Plxdc2	plexin domain containing 2	67448	https://www.ncbi.nlm.nih.gov/gene/?term=67448
Lrrc19	Lrrc19	leucine rich repeat containing 19	100061	https://www.ncbi.nlm.nih.gov/gene/?term=100061
Ankrd26	Ankrd26	ankyrin repeat domain 26	232339	https://www.ncbi.nlm.nih.gov/gene/?term=232339
Zfp738	Zfp738	zinc finger protein 738	408068	https://www.ncbi.nlm.nih.gov/gene/?term=408068
Calr3	Calr3	calreticulin 3	73316	https://www.ncbi.nlm.nih.gov/gene/?term=73316

Biology Open • Supplementary information

Smad9	Smad9	SMAD family member 9	55994	https://www.ncbi.nlm.nih.gov/gene/?term=55994
Acss3	Acss3	acyl-CoA synthetase short-chain family member 3	380660	https://www.ncbi.nlm.nih.gov/gene/?term=380660
Ranbp10	Ranbp10	RAN binding protein 10	74334	https://www.ncbi.nlm.nih.gov/gene/?term=74334
Tm2d2	Tm2d2	TM2 domain containing 2	69742	https://www.ncbi.nlm.nih.gov/gene/?term=69742
Fbxo5	Fbxo5	NA	67141	https://www.ncbi.nlm.nih.gov/gene/?term=67141
Slc7a11	Slc7a11	solute carrier family 7 (cationic amino acid transporter, y+ system), member 11	26570	https://www.ncbi.nlm.nih.gov/gene/?term=26570
Iqck	Iqck	IQ motif containing K	434232	https://www.ncbi.nlm.nih.gov/gene/?term=434232
Spidr	Spidr	scaffolding protein involved in DNA repair	224008	https://www.ncbi.nlm.nih.gov/gene/?term=224008
Rab6a	Rab6a	RAB6A, member RAS oncogene family	19346	https://www.ncbi.nlm.nih.gov/gene/?term=19346

Biology Open • Supplementary information

Snrnp27	Snrnp27	small nuclear ribonucleoprotein 27 (U4/U6.U5)	66618	https://www.ncbi.nlm.nih.gov/gene/?term=66618
Tmem220	Tmem220	transmembrane protein 220	338369	https://www.ncbi.nlm.nih.gov/gene/?term=338369
Anapc10	Anapc10	anaphase promoting complex subunit 10	68999	https://www.ncbi.nlm.nih.gov/gene/?term=68999
Emc7	Emc7	ER membrane protein complex subunit 7	73024	https://www.ncbi.nlm.nih.gov/gene/?term=73024
Rfesd	Rfesd	Rieske (Fe-S) domain containing	218341	https://www.ncbi.nlm.nih.gov/gene/?term=218341
Wdr44	Wdr44	WD repeat domain 44	72404	https://www.ncbi.nlm.nih.gov/gene/?term=72404
Ereg	Ereg	epiregulin	13874	https://www.ncbi.nlm.nih.gov/gene/?term=13874
Ms4a4b	Ms4a4b	membrane-spanning 4-domains, subfamily A, member 4B	60361	https://www.ncbi.nlm.nih.gov/gene/?term=60361
Ncbp2	Ncbp2	nuclear cap binding protein subunit 2	68092	https://www.ncbi.nlm.nih.gov/gene/?term=68092

Biology Open • Supplementary information

Ppwd1	Ppwd1	peptidylprolyl isomerase domain and WD repeat containing 1	238831	https://www.ncbi.nlm.nih.gov/gene/?term=238831
Slc24a5	Slc24a5	solute carrier family 24, member 5	317750	https://www.ncbi.nlm.nih.gov/gene/?term=317750
Arfgef1	Arfgef1	ADP-ribosylation factor guanine nucleotide-exchange factor 1(brefeldin A-inhibited)	211673	https://www.ncbi.nlm.nih.gov/gene/?term=211673
Npr3	Npr3	natriuretic peptide receptor 3	18162	https://www.ncbi.nlm.nih.gov/gene/?term=18162
Pkn2	Pkn2	protein kinase N2	109333	https://www.ncbi.nlm.nih.gov/gene/?term=109333
Golph3l	Golph3l	golgi phosphoprotein 3-like	229593	https://www.ncbi.nlm.nih.gov/gene/?term=229593
Hunk	Hunk	hormonally upregulated Neu-associated kinase	26559	https://www.ncbi.nlm.nih.gov/gene/?term=26559
Rai2	Rai2	retinoic acid induced 2	24004	https://www.ncbi.nlm.nih.gov/gene/?term=24004
Nat8f2	Nat8f2	N-acetyltransferase 8 (GCN5-related) family member 2	93673	https://www.ncbi.nlm.nih.gov/gene/?term=93673

Biology Open • Supplementary information

Cdkn2c	Cdkn2c	cyclin dependent kinase inhibitor 2C	12580	https://www.ncbi.nlm.nih.gov/gene/?term=12580
Arhgap31	Arhgap31	Rho GTPase activating protein 31	12549	https://www.ncbi.nlm.nih.gov/gene/?term=12549
Sprr3	Sprr3	small proline-rich protein 3	20766	https://www.ncbi.nlm.nih.gov/gene/?term=20766
Ngly1	Ngly1	N-glycanase 1	59007	https://www.ncbi.nlm.nih.gov/gene/?term=59007
Sh3bgrl	Sh3bgrl	SH3-binding domain glutamic acid-rich protein like	56726	https://www.ncbi.nlm.nih.gov/gene/?term=56726
Nsmaf	Nsmaf	neutral sphingomyelinase (N-SMase) activation associated factor	18201	https://www.ncbi.nlm.nih.gov/gene/?term=18201
Chchd6	Chchd6	coiled-coil-helix-coiled-coil-helix domain containing 6	66098	https://www.ncbi.nlm.nih.gov/gene/?term=66098
Ift122	Ift122	intraflagellar transport 122	81896	https://www.ncbi.nlm.nih.gov/gene/?term=81896
Slc5a3	Slc5a3	solute carrier family 5 (inositol transporters), member 3	53881	https://www.ncbi.nlm.nih.gov/gene/?term=53881

Biology Open • Supplementary information

Fbrsl1	Fbrsl1	fibrosin-like 1	381668	https://www.ncbi.nlm.nih.gov/gene/?term=381668
Trpm3	Trpm3	transient receptor potential cation channel, subfamily M, member 3	226025	https://www.ncbi.nlm.nih.gov/gene/?term=226025
Lipi	Lipi	lipase, member I	320355	https://www.ncbi.nlm.nih.gov/gene/?term=320355
Bmp2	Bmp2	bone morphogenetic protein 2	12156	https://www.ncbi.nlm.nih.gov/gene/?term=12156
Ocrl	Ocrl	OCRL, inositol polyphosphate-5-phosphatase	320634	https://www.ncbi.nlm.nih.gov/gene/?term=320634
Skida1	Skida1	SKI/DACH domain containing 1	72668	https://www.ncbi.nlm.nih.gov/gene/?term=72668
Kansl1l	Kansl1l	KAT8 regulatory NSL complex subunit 1-like	68691	https://www.ncbi.nlm.nih.gov/gene/?term=68691
Myoz1	Myoz1	myozenin 1	59011	https://www.ncbi.nlm.nih.gov/gene/?term=59011
Unkl	Unkl	unkempt family like zinc finger	74154	https://www.ncbi.nlm.nih.gov/gene/?term=74154

Biology Open • Supplementary information

U2af2	U2af2	U2 small nuclear ribonucleoprotein auxiliary factor (U2AF) 2	22185	https://www.ncbi.nlm.nih.gov/gene/?term=22185
Tfdp1	Tfdp1	transcription factor Dp 1	21781	https://www.ncbi.nlm.nih.gov/gene/?term=21781
Flt3	Flt3	FMS-like tyrosine kinase 3	14255	https://www.ncbi.nlm.nih.gov/gene/?term=14255
Foxa2	Foxa2	forkhead box A2	15376	https://www.ncbi.nlm.nih.gov/gene/?term=15376
Kazn	Kazn	kazrin, periplakin interacting protein	71529	https://www.ncbi.nlm.nih.gov/gene/?term=71529
Gnai2	Gnai2	guanine nucleotide binding protein (G protein), alpha inhibiting 2	14678	https://www.ncbi.nlm.nih.gov/gene/?term=14678
Ppp4r2	Ppp4r2	protein phosphatase 4, regulatory subunit 2	232314	https://www.ncbi.nlm.nih.gov/gene/?term=232314
Capn10	Capn10	calpain 10	23830	https://www.ncbi.nlm.nih.gov/gene/?term=23830
Eya2	Eya2	EYA transcriptional coactivator and phosphatase 2	14049	https://www.ncbi.nlm.nih.gov/gene/?term=14049

Biology Open • Supplementary information

Flnc	Flnc	filamin C, gamma	68794	https://www.ncbi.nlm.nih.gov/gene/?term=68794
Arhgef9	Arhgef9	CDC42 guanine nucleotide exchange factor (GEF) 9	236915	https://www.ncbi.nlm.nih.gov/gene/?term=236915
Sppl2a	Sppl2a	signal peptide peptidase like 2A	66552	https://www.ncbi.nlm.nih.gov/gene/?term=66552
Atp8b1	Atp8b1	ATPase, class I, type 8B, member 1	54670	https://www.ncbi.nlm.nih.gov/gene/?term=54670
Tmem65	Tmem65	transmembrane protein 65	74868	https://www.ncbi.nlm.nih.gov/gene/?term=74868
Mthfd2l	Mthfd2l	methylenetetrahydrofolate dehydrogenase (NADP+ dependent) 2-like	665563	https://www.ncbi.nlm.nih.gov/gene/?term=665563
Pip5k1a	Pip5k1a	phosphatidylinositol-4-phosphate 5-kinase, type 1 alpha	18720	https://www.ncbi.nlm.nih.gov/gene/?term=18720
Mpp6	Mpp6	membrane protein, palmitoylated 6 (MAGUK p55 subfamily member 6)	56524	https://www.ncbi.nlm.nih.gov/gene/?term=56524
Syndig1	Syndig1	synapse differentiation inducing 1	433485	https://www.ncbi.nlm.nih.gov/gene/?term=433485

Biology Open • Supplementary information

Ccl17	Ccl17	chemokine (C-C motif) ligand 17	20295	https://www.ncbi.nlm.nih.gov/gene/?term=20295
Atp8b4	Atp8b4	ATPase, class I, type 8B, member 4	241633	https://www.ncbi.nlm.nih.gov/gene/?term=241633
Chst5	Chst5	carbohydrate (N-acetylglucosamine 6-O) sulfotransferase 5	56773	https://www.ncbi.nlm.nih.gov/gene/?term=56773
Tox	Tox	thymocyte selection-associated high mobility group box	252838	https://www.ncbi.nlm.nih.gov/gene/?term=252838
Cyfip2	Cyfip2	cytoplasmic FMR1 interacting protein 2	76884	https://www.ncbi.nlm.nih.gov/gene/?term=76884
Shisa2	Shisa2	shisa family member 2	219134	https://www.ncbi.nlm.nih.gov/gene/?term=219134
Rbfox1	Rbfox1	RNA binding protein, fox-1 homolog (C. elegans) 1	268859	https://www.ncbi.nlm.nih.gov/gene/?term=268859
Sfpq	Sfpq	splicing factor proline/glutamine rich (polypyrimidine tract binding protein associated)	71514	https://www.ncbi.nlm.nih.gov/gene/?term=71514
Fgf10	Fgf10	fibroblast growth factor 10	14165	https://www.ncbi.nlm.nih.gov/gene/?term=14165

Biology Open • Supplementary information

Gjd2	Gjd2	gap junction protein, delta 2	14617	https://www.ncbi.nlm.nih.gov/gene/?term=14617
Sowahb	Sowahb	sosondowah ankyrin repeat domain family member B	78088	https://www.ncbi.nlm.nih.gov/gene/?term=78088
Mbtps2	Mbtps2	membrane-bound transcription factor peptidase, site 2	270669	https://www.ncbi.nlm.nih.gov/gene/?term=270669
Atp8b2	Atp8b2	ATPase, class I, type 8B, member 2	54667	https://www.ncbi.nlm.nih.gov/gene/?term=54667
Snx15	Snx15	sorting nexin 15	69024	https://www.ncbi.nlm.nih.gov/gene/?term=69024
Senp5	Senp5	SUMO/sentrin specific peptidase 5	320213	https://www.ncbi.nlm.nih.gov/gene/?term=320213
Arl15	Arl15	ADP-ribosylation factor-like 15	218639	https://www.ncbi.nlm.nih.gov/gene/?term=218639
Stk35	Stk35	serine/threonine kinase 35	67333	https://www.ncbi.nlm.nih.gov/gene/?term=67333
Lypd1	Lypd1	Ly6/Plaur domain containing 1	72585	https://www.ncbi.nlm.nih.gov/gene/?term=72585

Biology Open • Supplementary information

Ube2k	Ube2k	ubiquitin-conjugating enzyme E2K	53323	https://www.ncbi.nlm.nih.gov/gene/?term=53323
Kansl2	Kansl2	KAT8 regulatory NSL complex subunit 2	69612	https://www.ncbi.nlm.nih.gov/gene/?term=69612
Trib1	Trib1	tribbles pseudokinase 1	211770	https://www.ncbi.nlm.nih.gov/gene/?term=211770
Nxf1	Nxf1	nuclear RNA export factor 1	53319	https://www.ncbi.nlm.nih.gov/gene/?term=53319
Arhgap28	Arhgap28	Rho GTPase activating protein 28	268970	https://www.ncbi.nlm.nih.gov/gene/?term=268970
Tor1aip2	Tor1aip2	torsin A interacting protein 2	240832	https://www.ncbi.nlm.nih.gov/gene/?term=240832
Fmn1	Fmn1	formin 1	14260	https://www.ncbi.nlm.nih.gov/gene/?term=14260
Rab12	Rab12	RAB12, member RAS oncogene family	19328	https://www.ncbi.nlm.nih.gov/gene/?term=19328
Gna11	Gna11	guanine nucleotide binding protein, alpha 11	14672	https://www.ncbi.nlm.nih.gov/gene/?term=14672

Biology Open • Supplementary information

Egfl6	Egfl6	EGF-like-domain, multiple 6	54156	https://www.ncbi.nlm.nih.gov/gene/?term=54156
Slc37a3	Slc37a3	solute carrier family 37 (glycerol-3-phosphate transporter), member 3	72144	https://www.ncbi.nlm.nih.gov/gene/?term=72144
Ptpn23	Ptpn23	protein tyrosine phosphatase, non-receptor type 23	104831	https://www.ncbi.nlm.nih.gov/gene/?term=104831
Tnks	Tnks	tankyrase, TRF1-interacting ankyrin-related ADP-ribose polymerase	21951	https://www.ncbi.nlm.nih.gov/gene/?term=21951
Il6st	Il6st	interleukin 6 signal transducer	16195	https://www.ncbi.nlm.nih.gov/gene/?term=16195
Fmnl2	Fmnl2	formin-like 2	71409	https://www.ncbi.nlm.nih.gov/gene/?term=71409
Bod1	Bod1	biorientation of chromosomes in cell division 1	69556	https://www.ncbi.nlm.nih.gov/gene/?term=69556
Hgs	Hgs	HGF-regulated tyrosine kinase substrate	15239	https://www.ncbi.nlm.nih.gov/gene/?term=15239
Mark3	Mark3	MAP/microtubule affinity regulating kinase 3	17169	https://www.ncbi.nlm.nih.gov/gene/?term=17169

Biology Open • Supplementary information

Akt1s1	Akt1s1	AKT1 substrate 1 (proline-rich)	67605	https://www.ncbi.nlm.nih.gov/gene/?term=67605
Gorab	Gorab	golgin, RAB6-interacting	98376	https://www.ncbi.nlm.nih.gov/gene/?term=98376
Smug1	Smug1	single-strand selective monofunctional uracil DNA glycosylase	71726	https://www.ncbi.nlm.nih.gov/gene/?term=71726
Hectd1	Hectd1	HECT domain E3 ubiquitin protein ligase 1	207304	https://www.ncbi.nlm.nih.gov/gene/?term=207304
Tmem59	Tmem59	transmembrane protein 59	56374	https://www.ncbi.nlm.nih.gov/gene/?term=56374
Utrn	Utrn	utrophin	22288	https://www.ncbi.nlm.nih.gov/gene/?term=22288
Osbp	Osbp	oxysterol binding protein	76303	https://www.ncbi.nlm.nih.gov/gene/?term=76303
Acbd5	Acbd5	acyl-Coenzyme A binding domain containing 5	74159	https://www.ncbi.nlm.nih.gov/gene/?term=74159
Fem1c	Fem1c	fem-1 homolog c (C.elegans)	240263	https://www.ncbi.nlm.nih.gov/gene/?term=240263

Biology Open • Supplementary information

Golga7b	Golga7b	golgi autoantigen, golgin subfamily a, 7B	71146	https://www.ncbi.nlm.nih.gov/gene/?term=71146
Dpf2	Dpf2	D4, zinc and double PHD fingers family 2	19708	https://www.ncbi.nlm.nih.gov/gene/?term=19708
Prl7b1	Prl7b1	prolactin family 7, subfamily b, member 1	75596	https://www.ncbi.nlm.nih.gov/gene/?term=75596
Acbd6	Acbd6	acyl-Coenzyme A binding domain containing 6	72482	https://www.ncbi.nlm.nih.gov/gene/?term=72482
Tspan18	Tspan18	tetraspanin 18	241556	https://www.ncbi.nlm.nih.gov/gene/?term=241556
Dnajb4	Dnajb4	DnaJ heat shock protein family (Hsp40) member B4	67035	https://www.ncbi.nlm.nih.gov/gene/?term=67035
Krtap13	Krtap13	keratin associated protein 13	16699	https://www.ncbi.nlm.nih.gov/gene/?term=16699
Sesn2	Sesn2	sestrin 2	230784	https://www.ncbi.nlm.nih.gov/gene/?term=230784
Gatad2b	Gatad2b	GATA zinc finger domain containing 2B	229542	https://www.ncbi.nlm.nih.gov/gene/?term=229542

Biology Open • Supplementary information

Slc17a2	Slc17a2	solute carrier family 17 (sodium phosphate), member 2	218103	https://www.ncbi.nlm.nih.gov/gene/?term=218103
Cyb561a3	Cyb561a3	cytochrome b561 family, member A3	225912	https://www.ncbi.nlm.nih.gov/gene/?term=225912
Fam117a	Fam117a	family with sequence similarity 117, member A	215512	https://www.ncbi.nlm.nih.gov/gene/?term=215512
Map4k1	Map4k1	mitogen-activated protein kinase kinase kinase kinase 1	26411	https://www.ncbi.nlm.nih.gov/gene/?term=26411
Fam189a1	Fam189a1	family with sequence similarity 189, member A1	70638	https://www.ncbi.nlm.nih.gov/gene/?term=70638
Nampt	Nampt	nicotinamide phosphoribosyltransferase	59027	https://www.ncbi.nlm.nih.gov/gene/?term=59027
Usp7	Usp7	ubiquitin specific peptidase 7	252870	https://www.ncbi.nlm.nih.gov/gene/?term=252870
Ugcg	Ugcg	UDP-glucose ceramide glucosyltransferase	22234	https://www.ncbi.nlm.nih.gov/gene/?term=22234
Plec	Plec	plectin	18810	https://www.ncbi.nlm.nih.gov/gene/?term=18810

Biology Open • Supplementary information

Ccdc130	Ccdc130	coiled-coil domain containing 130	67736	https://www.ncbi.nlm.nih.gov/gene/?term=67736
Map6d1	Map6d1	MAP6 domain containing 1	208158	https://www.ncbi.nlm.nih.gov/gene/?term=208158
Srf	Srf	serum response factor	20807	https://www.ncbi.nlm.nih.gov/gene/?term=20807
Abt1	Abt1	activator of basal transcription 1	30946	https://www.ncbi.nlm.nih.gov/gene/?term=30946
Tmem248	Tmem248	transmembrane protein 248	71667	https://www.ncbi.nlm.nih.gov/gene/?term=71667
Runx3	Runx3	runt related transcription factor 3	12399	https://www.ncbi.nlm.nih.gov/gene/?term=12399
Psd2	Psd2	pleckstrin and Sec7 domain containing 2	74002	https://www.ncbi.nlm.nih.gov/gene/?term=74002
Lingo4	Lingo4	leucine rich repeat and Ig domain containing 4	320747	https://www.ncbi.nlm.nih.gov/gene/?term=320747
Otop2	Otop2	otopetrin 2	237987	https://www.ncbi.nlm.nih.gov/gene/?term=237987

Mnt	Mnt	max binding protein	17428	https://www.ncbi.nlm.nih.gov/gene/?term=17428
Mrps21	Mrps21	mitochondrial ribosomal protein S21	66292	https://www.ncbi.nlm.nih.gov/gene/?term=66292
Car7	Car7	carbonic anhydrase 7	12354	https://www.ncbi.nlm.nih.gov/gene/?term=12354
Pate3	Pate3	prostate and testis expressed 3	100312956	https://www.ncbi.nlm.nih.gov/gene/?term=100312
Adcy1	Adcy1	adenylate cyclase 1	432530	https://www.ncbi.nlm.nih.gov/gene/?term=432530
Gpr37	Gpr37	G protein-coupled receptor 37	14763	https://www.ncbi.nlm.nih.gov/gene/?term=14763
Rspry1	Rspry1	ring finger and SPRY domain containing 1	67610	https://www.ncbi.nlm.nih.gov/gene/?term=67610
Tnfrsf23	Tnfrsf23	tumor necrosis factor receptor superfamily, member 23	79201	https://www.ncbi.nlm.nih.gov/gene/?term=79201
Vma21	Vma21	VMA21 vacuolar H+-ATPase homolog (S. cerevisiae)	67048	https://www.ncbi.nlm.nih.gov/gene/?term=67048

Biology Open • Supplementary information

Tnfrsf22	Tnfrsf22	tumor necrosis factor receptor superfamily, member 22	79202	https://www.ncbi.nlm.nih.gov/gene/?term=79202
Clta	Clta	clathrin, light polypeptide (Lca)	12757	https://www.ncbi.nlm.nih.gov/gene/?term=12757
Fads1	Fads1	fatty acid desaturase 1	76267	https://www.ncbi.nlm.nih.gov/gene/?term=76267
Plpp2	Plpp2	phospholipid phosphatase 2	50784	https://www.ncbi.nlm.nih.gov/gene/?term=50784
Arrb1	Arrb1	arrestin, beta 1	109689	https://www.ncbi.nlm.nih.gov/gene/?term=109689
Timm17a	Timm17a	translocase of inner mitochondrial membrane 17a	21854	https://www.ncbi.nlm.nih.gov/gene/?term=21854
Virma	Virma	vir like m6A methyltransferase associated	66185	https://www.ncbi.nlm.nih.gov/gene/?term=66185
Rbmx	Rbmx	RNA binding motif protein, X chromosome	19655	https://www.ncbi.nlm.nih.gov/gene/?term=19655
Actr1a	Actr1a	ARP1 actin-related protein 1A, centractin alpha	54130	https://www.ncbi.nlm.nih.gov/gene/?term=54130

Biology Open • Supplementary information

Map4	Map4	microtubule-associated protein 4	17758	https://www.ncbi.nlm.nih.gov/gene/?term=17758
Foxp4	Foxp4	forkhead box P4	74123	https://www.ncbi.nlm.nih.gov/gene/?term=74123
Sertad4	Sertad4	SERTA domain containing 4	214791	https://www.ncbi.nlm.nih.gov/gene/?term=214791
Ptprz1	Ptprz1	protein tyrosine phosphatase, receptor type Z, polypeptide 1	19283	https://www.ncbi.nlm.nih.gov/gene/?term=19283
Sgpp1	Sgpp1	sphingosine-1-phosphate phosphatase 1	81535	https://www.ncbi.nlm.nih.gov/gene/?term=81535
Gabpb2	Gabpb2	GA repeat binding protein, beta 2	213054	https://www.ncbi.nlm.nih.gov/gene/?term=213054
Nup160	Nup160	nucleoporin 160	59015	https://www.ncbi.nlm.nih.gov/gene/?term=59015
Rffl	Rffl	ring finger and FYVE like domain containing protein	67338	https://www.ncbi.nlm.nih.gov/gene/?term=67338
Sec61a1	Sec61a1	Sec61 alpha 1 subunit (S. cerevisiae)	53421	https://www.ncbi.nlm.nih.gov/gene/?term=53421

Tagln2	Tagln2	transgelin 2	21346	https://www.ncbi.nlm.nih.gov/gene/?term=21346
Shisa5	Shisa5	shisa family member 5	66940	https://www.ncbi.nlm.nih.gov/gene/?term=66940
Otc	Otc	ornithine transcarbamylase	18416	https://www.ncbi.nlm.nih.gov/gene/?term=18416
Actn4	Actn4	actinin alpha 4	60595	https://www.ncbi.nlm.nih.gov/gene/?term=60595
Tfap2d	Tfap2d	transcription factor AP-2, delta	226896	https://www.ncbi.nlm.nih.gov/gene/?term=226896
Praf2	Praf2	PRA1 domain family 2	54637	https://www.ncbi.nlm.nih.gov/gene/?term=54637
Vat1	Vat1	vesicle amine transport 1	26949	https://www.ncbi.nlm.nih.gov/gene/?term=26949
Enc1	Enc1	ectodermal-neural cortex 1	13803	https://www.ncbi.nlm.nih.gov/gene/?term=13803
Lasp1	Lasp1	LIM and SH3 protein 1	16796	https://www.ncbi.nlm.nih.gov/gene/?term=16796

Biology Open • Supplementary information

Sh3gl2	Sh3gl2	SH3-domain GRB2-like 2	20404	https://www.ncbi.nlm.nih.gov/gene/?term=20404
Vps54	Vps54	VPS54 GARP complex subunit	245944	https://www.ncbi.nlm.nih.gov/gene/?term=245944
Tax1bp1	Tax1bp1	Tax1 (human T cell leukemia virus type I) binding protein 1	52440	https://www.ncbi.nlm.nih.gov/gene/?term=52440
Maml1	Maml1	mastermind like transcriptional coactivator 1	103806	https://www.ncbi.nlm.nih.gov/gene/?term=103806
Ptbp1	Ptbp1	polypyrimidine tract binding protein 1	19205	https://www.ncbi.nlm.nih.gov/gene/?term=19205
Elfn1	Elfn1	leucine rich repeat and fibronectin type III, extracellular 1	243312	https://www.ncbi.nlm.nih.gov/gene/?term=243312
P2rx4	P2rx4	purinergic receptor P2X, ligand-gated ion channel 4	18438	https://www.ncbi.nlm.nih.gov/gene/?term=18438
Cmpk1	Cmpk1	cytidine monophosphate (UMP-CMP) kinase 1	66588	https://www.ncbi.nlm.nih.gov/gene/?term=66588
Hsd17b11	Hsd17b11	hydroxysteroid (17-beta) dehydrogenase 11	114664	https://www.ncbi.nlm.nih.gov/gene/?term=114664

Biology Open • Supplementary information

Smarcd1	Smarcd1	SWI/SNF related, matrix associated, actin dependent regulator of chromatin, subfamily d, member 1	83797	https://www.ncbi.nlm.nih.gov/gene/?term=83797
Sec61b	Sec61b	Sec61 beta subunit	66212	https://www.ncbi.nlm.nih.gov/gene/?term=66212
Ppp2cb	Ppp2cb	protein phosphatase 2 (formerly 2A), catalytic subunit, beta isoform	19053	https://www.ncbi.nlm.nih.gov/gene/?term=19053
Acat3	Acat3	acetyl-Coenzyme A acetyltransferase 3	224530	https://www.ncbi.nlm.nih.gov/gene/?term=224530
Dpysl4	Dpysl4	dihydropyrimidinase-like 4	26757	https://www.ncbi.nlm.nih.gov/gene/?term=26757
Yes1	Yes1	YES proto-oncogene 1, Src family tyrosine kinase	22612	https://www.ncbi.nlm.nih.gov/gene/?term=22612
Gabarapl1	Gabarapl1	gamma-aminobutyric acid (GABA) A receptor-associated protein-like 1	57436	https://www.ncbi.nlm.nih.gov/gene/?term=57436
Glra2	Glra2	glycine receptor, alpha 2 subunit	237213	https://www.ncbi.nlm.nih.gov/gene/?term=237213
Eif4a1	Eif4a1	eukaryotic translation initiation factor 4A1	13681	https://www.ncbi.nlm.nih.gov/gene/?term=13681

Biology Open • Supplementary information

Tspan15	Tspan15	tetraspanin 15	70423	https://www.ncbi.nlm.nih.gov/gene/?term=70423
Eno2	Eno2	enolase 2, gamma neuronal	13807	https://www.ncbi.nlm.nih.gov/gene/?term=13807
Paxip1	Paxip1	PAX interacting (with transcription-activation domain) protein 1	55982	https://www.ncbi.nlm.nih.gov/gene/?term=55982
Txnl1	Txnl1	thioredoxin-like 1	53382	https://www.ncbi.nlm.nih.gov/gene/?term=53382
Zfp609	Zfp609	zinc finger protein 609	214812	https://www.ncbi.nlm.nih.gov/gene/?term=214812
Morc2a	Morc2a	microrchidia 2A	74522	https://www.ncbi.nlm.nih.gov/gene/?term=74522
Hars2	Hars2	histidyl-tRNA synthetase 2	70791	https://www.ncbi.nlm.nih.gov/gene/?term=70791
Cnnm4	Cnnm4	cyclin M4	94220	https://www.ncbi.nlm.nih.gov/gene/?term=94220
Hoxb6	Hoxb6	homeobox B6	15414	https://www.ncbi.nlm.nih.gov/gene/?term=15414

Biology Open • Supplementary information

Kdr	Kdr	kinase insert domain protein receptor	16542	https://www.ncbi.nlm.nih.gov/gene/?term=16542
Mas1	Mas1	MAS1 oncogene	17171	https://www.ncbi.nlm.nih.gov/gene/?term=17171
Mtmr4	Mtmr4	myotubularin related protein 4	170749	https://www.ncbi.nlm.nih.gov/gene/?term=170749
Kdm5c	Kdm5c	lysine (K)-specific demethylase 5C	20591	https://www.ncbi.nlm.nih.gov/gene/?term=20591
Clvs2	Clvs2	clavesin 2	215890	https://www.ncbi.nlm.nih.gov/gene/?term=215890
Prss8	Prss8	protease, serine 8 (prostasin)	76560	https://www.ncbi.nlm.nih.gov/gene/?term=76560
Acnat1	Acnat1	acyl-coenzyme A amino acid N-acyltransferase 1	230161	https://www.ncbi.nlm.nih.gov/gene/?term=230161
Map6	Map6	microtubule-associated protein 6	17760	https://www.ncbi.nlm.nih.gov/gene/?term=17760
Prkcq	Prkcq	protein kinase C, theta	18761	https://www.ncbi.nlm.nih.gov/gene/?term=18761

Biology Open • Supplementary information

Trim66	Trim66	tripartite motif-containing 66	330627	https://www.ncbi.nlm.nih.gov/gene/?term=330627
Ube2q1	Ube2q1	ubiquitin-conjugating enzyme E2Q family member 1	70093	https://www.ncbi.nlm.nih.gov/gene/?term=70093
Tmem260	Tmem260	transmembrane protein 260	218989	https://www.ncbi.nlm.nih.gov/gene/?term=218989
8030462N17Rik	8030462N17Rik	RIKEN cDNA 8030462N17 gene	212163	https://www.ncbi.nlm.nih.gov/gene/?term=212163
Manf	Manf	mesencephalic astrocyte-derived neurotrophic factor	74840	https://www.ncbi.nlm.nih.gov/gene/?term=74840
Slc6a7	Slc6a7	solute carrier family 6 (neurotransmitter transporter, L-proline), member 7	240332	https://www.ncbi.nlm.nih.gov/gene/?term=240332
Pou2f3	Pou2f3	POU domain, class 2, transcription factor 3	18988	https://www.ncbi.nlm.nih.gov/gene/?term=18988
Smc1a	Smc1a	structural maintenance of chromosomes 1A	24061	https://www.ncbi.nlm.nih.gov/gene/?term=24061
Trim8	Trim8	tripartite motif-containing 8	93679	https://www.ncbi.nlm.nih.gov/gene/?term=93679

Biology Open • Supplementary information

Tmco5b	Tmco5b	transmembrane and coiled-coil domains 5B	75275	https://www.ncbi.nlm.nih.gov/gene/?term=75275
Ccdc191	Ccdc191	coiled-coil domain containing 191	212153	https://www.ncbi.nlm.nih.gov/gene/?term=212153
Wwp1	Wwp1	WW domain containing E3 ubiquitin protein ligase 1	107568	https://www.ncbi.nlm.nih.gov/gene/?term=107568
Vav3	Vav3	vav 3 oncogene	57257	https://www.ncbi.nlm.nih.gov/gene/?term=57257
Cox16	Cox16	cytochrome c oxidase assembly protein 16	66272	https://www.ncbi.nlm.nih.gov/gene/?term=66272
Gpr119	Gpr119	G-protein coupled receptor 119	236781	https://www.ncbi.nlm.nih.gov/gene/?term=236781
Ttc41	Ttc41	tetratricopeptide repeat domain 41	103220	https://www.ncbi.nlm.nih.gov/gene/?term=103220
Gorasp1	Gorasp1	golgi reassembly stacking protein 1	74498	https://www.ncbi.nlm.nih.gov/gene/?term=74498
Fgf12	Fgf12	fibroblast growth factor 12	14167	https://www.ncbi.nlm.nih.gov/gene/?term=14167

Biology Open • Supplementary information

Rnf146	Rnf146	ring finger protein 146	68031	https://www.ncbi.nlm.nih.gov/gene/?term=68031
Etv1	Etv1	ets variant 1	14009	https://www.ncbi.nlm.nih.gov/gene/?term=14009
Prkg1	Prkg1	protein kinase, cGMP-dependent, type I	19091	https://www.ncbi.nlm.nih.gov/gene/?term=19091
Rsf1	Rsf1	remodeling and spacing factor 1	233532	https://www.ncbi.nlm.nih.gov/gene/?term=233532
Arap2	Arap2	ArfGAP with RhoGAP domain, ankyrin repeat and PH domain 2	212285	https://www.ncbi.nlm.nih.gov/gene/?term=212285
9230112D13Rik	9230112D13Rik	RIKEN cDNA 9230112D13 gene	78243	https://www.ncbi.nlm.nih.gov/gene/?term=78243
Vmp1	Vmp1	vacuole membrane protein 1	75909	https://www.ncbi.nlm.nih.gov/gene/?term=75909
Zfp275	Zfp275	zinc finger protein 275	27081	https://www.ncbi.nlm.nih.gov/gene/?term=27081
Acadsb	Acadsb	acyl-Coenzyme A dehydrogenase, short/branched chain	66885	https://www.ncbi.nlm.nih.gov/gene/?term=66885

Biology Open • Supplementary information

Fam102b	Fam102b	family with sequence similarity 102, member B	329739	https://www.ncbi.nlm.nih.gov/gene/?term=329739
Adamts1	Adamts1	a disintegrin-like and metallopeptidase (reprolysin type) with thrombospondin type 1 motif, 1	11504	https://www.ncbi.nlm.nih.gov/gene/?term=11504
Uba3	Uba3	ubiquitin-like modifier activating enzyme 3	22200	https://www.ncbi.nlm.nih.gov/gene/?term=22200
Gngt1	Gngt1	guanine nucleotide binding protein (G protein), gamma transducing activity polypeptide 1	14699	https://www.ncbi.nlm.nih.gov/gene/?term=14699
Gm20498	Gm20498	predicted gene 20498	105940408	https://www.ncbi.nlm.nih.gov/gene/?term=105940
Btf3l4	Btf3l4	basic transcription factor 3-like 4	70533	https://www.ncbi.nlm.nih.gov/gene/?term=70533
Lmx1a	Lmx1a	LIM homeobox transcription factor 1 alpha	110648	https://www.ncbi.nlm.nih.gov/gene/?term=110648
Slain1	Slain1	SLAIN motif family, member 1	105439	https://www.ncbi.nlm.nih.gov/gene/?term=105439
Pip5k1b	Pip5k1b	phosphatidylinositol-4-phosphate 5-kinase, type 1 beta	18719	https://www.ncbi.nlm.nih.gov/gene/?term=18719

Biology Open • Supplementary information

Capn7	Capn7	calpain 7	12339	https://www.ncbi.nlm.nih.gov/gene/?term=12339
Etfdh	Etfdh	electron transferring flavoprotein, dehydrogenase	66841	https://www.ncbi.nlm.nih.gov/gene/?term=66841
Mical2	Mical2	microtubule associated monooxygenase, calponin and LIM domain containing 2	320878	https://www.ncbi.nlm.nih.gov/gene/?term=320878
Atxn7l2	Atxn7l2	ataxin 7-like 2	72522	https://www.ncbi.nlm.nih.gov/gene/?term=72522
Efcab14	Efcab14	EF-hand calcium binding domain 14	230648	https://www.ncbi.nlm.nih.gov/gene/?term=230648
Trip13	Trip13	thyroid hormone receptor interactor 13	69716	https://www.ncbi.nlm.nih.gov/gene/?term=69716
Mcmdc2	Mcmdc2	minichromosome maintenance domain containing 2	240697	https://www.ncbi.nlm.nih.gov/gene/?term=240697
Dnajc25	Dnajc25	DnaJ heat shock protein family (Hsp40) member C25	72429	https://www.ncbi.nlm.nih.gov/gene/?term=72429
Ppp1r42	Ppp1r42	protein phosphatase 1, regulatory subunit 42	69312	https://www.ncbi.nlm.nih.gov/gene/?term=69312

Biology Open • Supplementary information

Lrrc58	Lrrc58	leucine rich repeat containing 58	320184	https://www.ncbi.nlm.nih.gov/gene/?term=320184
Pgm3	Pgm3	phosphoglucomutase 3	109785	https://www.ncbi.nlm.nih.gov/gene/?term=109785
Tdo2	Tdo2	tryptophan 2,3-dioxygenase	56720	https://www.ncbi.nlm.nih.gov/gene/?term=56720
Skp2	Skp2	S-phase kinase-associated protein 2 (p45)	27401	https://www.ncbi.nlm.nih.gov/gene/?term=27401
Socs1	Socs1	suppressor of cytokine signaling 1	12703	https://www.ncbi.nlm.nih.gov/gene/?term=12703
Iqsec1	Iqsec1	IQ motif and Sec7 domain 1	232227	https://www.ncbi.nlm.nih.gov/gene/?term=232227
Iltifb	Iltifb	interleukin 10-related T cell-derived inducible factor beta	116849	https://www.ncbi.nlm.nih.gov/gene/?term=116849
Npat	Npat	nuclear protein in the AT region	244879	https://www.ncbi.nlm.nih.gov/gene/?term=244879
Ccdc71l	Ccdc71l	coiled-coil domain containing 71 like	72123	https://www.ncbi.nlm.nih.gov/gene/?term=72123

Biology Open • Supplementary information

Olfr630	Olfr630	olfactory receptor 630	259102	https://www.ncbi.nlm.nih.gov/gene/?term=259102
Orc3	Orc3	origin recognition complex, subunit 3	50793	https://www.ncbi.nlm.nih.gov/gene/?term=50793
Cyp11b1	Cyp11b1	cytochrome P450, family 11, subfamily b, polypeptide 1	110115	https://www.ncbi.nlm.nih.gov/gene/?term=110115
Oprl1	Oprl1	opioid receptor-like 1	18389	https://www.ncbi.nlm.nih.gov/gene/?term=18389
Clic5	Clic5	chloride intracellular channel 5	224796	https://www.ncbi.nlm.nih.gov/gene/?term=224796
Klf3	Klf3	Kruppel-like factor 3 (basic)	16599	https://www.ncbi.nlm.nih.gov/gene/?term=16599
Akr1b3	Akr1b3	aldo-keto reductase family 1, member B3 (aldose reductase)	11677	https://www.ncbi.nlm.nih.gov/gene/?term=11677
Ehd4	Ehd4	EH-domain containing 4	98878	https://www.ncbi.nlm.nih.gov/gene/?term=98878
Gm3985	Gm3985	predicted gene 3985	100042715	https://www.ncbi.nlm.nih.gov/gene/?term=100042

Biology Open • Supplementary information

Fat3	Fat3	FAT atypical cadherin 3	270120	https://www.ncbi.nlm.nih.gov/gene/?term=270120
Tmem144	Tmem144	transmembrane protein 144	70652	https://www.ncbi.nlm.nih.gov/gene/?term=70652
Jade2	Jade2	jade family PHD finger 2	76901	https://www.ncbi.nlm.nih.gov/gene/?term=76901
Tpx2	Tpx2	TPX2, microtubule-associated	72119	https://www.ncbi.nlm.nih.gov/gene/?term=72119
Tmem150c	Tmem150c	transmembrane protein 150C	231503	https://www.ncbi.nlm.nih.gov/gene/?term=231503
Cxcl14	Cxcl14	chemokine (C-X-C motif) ligand 14	57266	https://www.ncbi.nlm.nih.gov/gene/?term=57266
Abhd14a	Abhd14a	abhydrolase domain containing 14A	68644	https://www.ncbi.nlm.nih.gov/gene/?term=68644
Moxd1	Moxd1	monooxygenase, DBH-like 1	59012	https://www.ncbi.nlm.nih.gov/gene/?term=59012
Tesk2	Tesk2	testis-specific kinase 2	230661	https://www.ncbi.nlm.nih.gov/gene/?term=230661

Tpt1	Tpt1	tumor protein, translationally-controlled 1	22070	https://www.ncbi.nlm.nih.gov/gene/?term=22070
Hk3	Hk3	hexokinase 3	212032	https://www.ncbi.nlm.nih.gov/gene/?term=212032
Gtf3c2	Gtf3c2	general transcription factor IIIC, polypeptide 2, beta	71752	https://www.ncbi.nlm.nih.gov/gene/?term=71752
Gabarapl2	Gabarapl2	gamma-aminobutyric acid (GABA) A receptor-associated protein-like 2	93739	https://www.ncbi.nlm.nih.gov/gene/?term=93739
Ppih	Ppih	peptidyl prolyl isomerase H	66101	https://www.ncbi.nlm.nih.gov/gene/?term=66101
Camk2n1	Camk2n1	calcium/calmodulin-dependent protein kinase II inhibitor 1	66259	https://www.ncbi.nlm.nih.gov/gene/?term=66259
Tti1	Tti1	TELO2 interacting protein 1	75425	https://www.ncbi.nlm.nih.gov/gene/?term=75425
Colec12	Colec12	collectin sub-family member 12	140792	https://www.ncbi.nlm.nih.gov/gene/?term=140792
Fbxo42	Fbxo42	F-box protein 42	213499	https://www.ncbi.nlm.nih.gov/gene/?term=213499

Biology Open • Supplementary information

Sccpdh	Sccpdh	saccharopine dehydrogenase (putative)	109232	https://www.ncbi.nlm.nih.gov/gene/?term=109232
Myo6	Myo6	myosin VI	17920	https://www.ncbi.nlm.nih.gov/gene/?term=17920
Tor1b	Tor1b	torsin family 1, member B	30934	https://www.ncbi.nlm.nih.gov/gene/?term=30934
Hfm1	Hfm1	HFM1, ATP-dependent DNA helicase homolog	330149	https://www.ncbi.nlm.nih.gov/gene/?term=330149
Mob2	Mob2	MOB kinase activator 2	101513	https://www.ncbi.nlm.nih.gov/gene/?term=101513
Myf5	Myf5	myogenic factor 5	17877	https://www.ncbi.nlm.nih.gov/gene/?term=17877
Chmp4c	Chmp4c	charged multivesicular body protein 4C	66371	https://www.ncbi.nlm.nih.gov/gene/?term=66371
Bnip2	Bnip2	BCL2/adenovirus E1B interacting protein 2	12175	https://www.ncbi.nlm.nih.gov/gene/?term=12175
Igf2	Igf2	insulin-like growth factor 2	16002	https://www.ncbi.nlm.nih.gov/gene/?term=16002

Ppp1r1c	Ppp1r1c	protein phosphatase 1, regulatory inhibitor subunit 1C	75276	https://www.ncbi.nlm.nih.gov/gene/?term=75276
Dnlz	Dnlz	DNL-type zinc finger	52838	https://www.ncbi.nlm.nih.gov/gene/?term=52838
Podn	Podn	podocan	242608	https://www.ncbi.nlm.nih.gov/gene/?term=242608
Orai2	Orai2	ORAI calcium release-activated calcium modulator 2	269717	https://www.ncbi.nlm.nih.gov/gene/?term=269717
D430019H16Rik	D430019H16Rik	RIKEN cDNA D430019H16 gene	268595	https://www.ncbi.nlm.nih.gov/gene/?term=268595
Snrpn	Snrpn	small nuclear ribonucleoprotein N	20646	https://www.ncbi.nlm.nih.gov/gene/?term=20646
Tmem132b	Tmem132b	transmembrane protein 132B	208151	https://www.ncbi.nlm.nih.gov/gene/?term=208151
Prr18	Prr18	proline rich 18	320111	https://www.ncbi.nlm.nih.gov/gene/?term=320111
D430042O09Rik	D430042O09Rik	RIKEN cDNA D430042O09 gene	233865	https://www.ncbi.nlm.nih.gov/gene/?term=233865

Biology Open • Supplementary information

Gria3	Gria3	glutamate receptor, ionotropic, AMPA3 (alpha 3)	53623	https://www.ncbi.nlm.nih.gov/gene/?term=53623
Kcnj1	Kcnj1	potassium inwardly-rectifying channel, subfamily J, member 1	56379	https://www.ncbi.nlm.nih.gov/gene/?term=56379
Adra1b	Adra1b	adrenergic receptor, alpha 1b	11548	https://www.ncbi.nlm.nih.gov/gene/?term=11548
Steap2	Steap2	six transmembrane epithelial antigen of prostate 2	74051	https://www.ncbi.nlm.nih.gov/gene/?term=74051
Lbp	Lbp	lipopolysaccharide binding protein	16803	https://www.ncbi.nlm.nih.gov/gene/?term=16803
Pou4f2	Pou4f2	POU domain, class 4, transcription factor 2	18997	https://www.ncbi.nlm.nih.gov/gene/?term=18997
Scn3b	Scn3b	sodium channel, voltage-gated, type III, beta	235281	https://www.ncbi.nlm.nih.gov/gene/?term=235281
Sytl5	Sytl5	synaptotagmin-like 5	236643	https://www.ncbi.nlm.nih.gov/gene/?term=236643
Ostm1	Ostm1	osteopetrosis associated transmembrane protein 1	14628	https://www.ncbi.nlm.nih.gov/gene/?term=14628

Biology Open • Supplementary information

Ubtf	Ubtf	upstream binding transcription factor, RNA polymerase I	21429	https://www.ncbi.nlm.nih.gov/gene/?term=21429
Adhfe1	Adhfe1	alcohol dehydrogenase, iron containing, 1	76187	https://www.ncbi.nlm.nih.gov/gene/?term=76187
Bean1	Bean1	brain expressed, associated with Nedd4, 1	65115	https://www.ncbi.nlm.nih.gov/gene/?term=65115
AW554918	AW554918	expressed sequence AW554918	225289	https://www.ncbi.nlm.nih.gov/gene/?term=225289
Cdc27	Cdc27	cell division cycle 27	217232	https://www.ncbi.nlm.nih.gov/gene/?term=217232
Plp1	Plp1	proteolipid protein (myelin) 1	18823	https://www.ncbi.nlm.nih.gov/gene/?term=18823
Sox1	Sox1	SRY (sex determining region Y)-box 1	20664	https://www.ncbi.nlm.nih.gov/gene/?term=20664
Bcl2	Bcl2	B cell leukemia/lymphoma 2	12043	https://www.ncbi.nlm.nih.gov/gene/?term=12043
Cstf3	Cstf3	cleavage stimulation factor, 3' pre-RNA, subunit 3	228410	https://www.ncbi.nlm.nih.gov/gene/?term=228410

Biology Open • Supplementary information

Dkk1	Dkk1	dickkopf WNT signaling pathway inhibitor 1	13380	https://www.ncbi.nlm.nih.gov/gene/?term=13380
Zfand2a	Zfand2a	zinc finger, AN1-type domain 2A	100494	https://www.ncbi.nlm.nih.gov/gene/?term=100494
Kif1a	Kif1a	kinesin family member 1A	16560	https://www.ncbi.nlm.nih.gov/gene/?term=16560
Smarca2	Smarca2	SWI/SNF related, matrix associated, actin dependent regulator of chromatin, subfamily a, member 2	67155	https://www.ncbi.nlm.nih.gov/gene/?term=67155
Cipc	Cipc	CLOCK interacting protein, circadian	217732	https://www.ncbi.nlm.nih.gov/gene/?term=217732
Elp4	Elp4	elongator acetyltransferase complex subunit 4	77766	https://www.ncbi.nlm.nih.gov/gene/?term=77766
Pcdhgb6	Pcdhgb6	protocadherin gamma subfamily B, 6	93703	https://www.ncbi.nlm.nih.gov/gene/?term=93703
Zfp239	Zfp239	zinc finger protein 239	22685	https://www.ncbi.nlm.nih.gov/gene/?term=22685
Zrsr2	Zrsr2	zinc finger (CCCH type), RNA binding motif and serine/arginine rich 2	22184	https://www.ncbi.nlm.nih.gov/gene/?term=22184

Cd99l2	Cd99l2	CD99 antigen-like 2	171486	https://www.ncbi.nlm.nih.gov/gene/?term=171486
Camk1d	Camk1d	calcium/calmodulin-dependent protein kinase ID	227541	https://www.ncbi.nlm.nih.gov/gene/?term=227541
Xrcc3	Xrcc3	X-ray repair complementing defective repair in Chinese hamster cells 3	74335	https://www.ncbi.nlm.nih.gov/gene/?term=74335
Rgs4	Rgs4	regulator of G-protein signaling 4	19736	https://www.ncbi.nlm.nih.gov/gene/?term=19736
Slc38a6	Slc38a6	solute carrier family 38, member 6	625098	https://www.ncbi.nlm.nih.gov/gene/?term=625098
Ogdh	Ogdh	oxoglutarate (alpha-ketoglutarate) dehydrogenase (lipoamide)	18293	https://www.ncbi.nlm.nih.gov/gene/?term=18293
Vsig10l	Vsig10l	V-set and immunoglobulin domain containing 10 like	75690	https://www.ncbi.nlm.nih.gov/gene/?term=75690
Zfp37	Zfp37	zinc finger protein 37	22696	https://www.ncbi.nlm.nih.gov/gene/?term=22696
Nqo2	Nqo2	N-ribosyldihydronicotinamide quinone reductase 2	18105	https://www.ncbi.nlm.nih.gov/gene/?term=18105

Biology Open • Supplementary information

Nab2	Nab2	Ngfi-A binding protein 2	17937	https://www.ncbi.nlm.nih.gov/gene/?term=17937
Cyfip1	Cyfip1	cytoplasmic FMR1 interacting protein 1	20430	https://www.ncbi.nlm.nih.gov/gene/?term=20430
Zmiz1	Zmiz1	zinc finger, MIZ-type containing 1	328365	https://www.ncbi.nlm.nih.gov/gene/?term=328365
Zfp764	Zfp764	zinc finger protein 764	233893	https://www.ncbi.nlm.nih.gov/gene/?term=233893
Rbm34	Rbm34	RNA binding motif protein 34	52202	https://www.ncbi.nlm.nih.gov/gene/?term=52202
Olig2	Olig2	oligodendrocyte transcription factor 2	50913	https://www.ncbi.nlm.nih.gov/gene/?term=50913
Tigd5	Tigd5	tigger transposable element derived 5	105734	https://www.ncbi.nlm.nih.gov/gene/?term=105734
Zfp365	Zfp365	zinc finger protein 365	216049	https://www.ncbi.nlm.nih.gov/gene/?term=216049
Bdh1	Bdh1	3-hydroxybutyrate dehydrogenase, type 1	71911	https://www.ncbi.nlm.nih.gov/gene/?term=71911

Biology Open • Supplementary information

Lcp2	Lcp2	lymphocyte cytosolic protein 2	16822	https://www.ncbi.nlm.nih.gov/gene/?term=16822
Pcdhga12	Pcdhga12	protocadherin gamma subfamily A, 12	93724	https://www.ncbi.nlm.nih.gov/gene/?term=93724
Adarb2	Adarb2	adenosine deaminase, RNA-specific, B2	94191	https://www.ncbi.nlm.nih.gov/gene/?term=94191
Wars2	Wars2	tryptophanyl tRNA synthetase 2 (mitochondrial)	70560	https://www.ncbi.nlm.nih.gov/gene/?term=70560
Ggact	Ggact	gamma-glutamylamine cyclotransferase	223267	https://www.ncbi.nlm.nih.gov/gene/?term=223267
Ralgapa2	Ralgapa2	Ral GTPase activating protein, alpha subunit 2 (catalytic)	241694	https://www.ncbi.nlm.nih.gov/gene/?term=241694
Opcml	Opcml	opioid binding protein/cell adhesion molecule-like	330908	https://www.ncbi.nlm.nih.gov/gene/?term=330908
Peli2	Peli2	pellino 2	93834	https://www.ncbi.nlm.nih.gov/gene/?term=93834
Tbx15	Tbx15	T-box 15	21384	https://www.ncbi.nlm.nih.gov/gene/?term=21384

Ppfia2	Ppfia2	protein tyrosine phosphatase, receptor type, f polypeptide (PTPRF), interacting protein (liprin), alpha 2	327814	https://www.ncbi.nlm.nih.gov/gene/?term=327814
Slc17a5	Slc17a5	solute carrier family 17 (anion/sugar transporter), member 5	235504	https://www.ncbi.nlm.nih.gov/gene/?term=235504
Gfra2	Gfra2	glial cell line derived neurotrophic factor family receptor alpha 2	14586	https://www.ncbi.nlm.nih.gov/gene/?term=14586
Kank2	Kank2	KN motif and ankyrin repeat domains 2	235041	https://www.ncbi.nlm.nih.gov/gene/?term=235041
Fgf11	Fgf11	fibroblast growth factor 11	14166	https://www.ncbi.nlm.nih.gov/gene/?term=14166
Foxk1	Foxk1	forkhead box K1	17425	https://www.ncbi.nlm.nih.gov/gene/?term=17425
Cd33	Cd33	CD33 antigen	12489	https://www.ncbi.nlm.nih.gov/gene/?term=12489
Slc4a8	Slc4a8	solute carrier family 4 (anion exchanger), member 8	59033	https://www.ncbi.nlm.nih.gov/gene/?term=59033
Vegfb	Vegfb	vascular endothelial growth factor B	22340	https://www.ncbi.nlm.nih.gov/gene/?term=22340

Biology Open • Supplementary information

Zfp46	Zfp46	zinc finger protein 46	22704	https://www.ncbi.nlm.nih.gov/gene/?term=22704
Ssbp4	Ssbp4	single stranded DNA binding protein 4	76900	https://www.ncbi.nlm.nih.gov/gene/?term=76900
Rhou	Rhou	ras homolog family member U	69581	https://www.ncbi.nlm.nih.gov/gene/?term=69581
Iffo2	Iffo2	intermediate filament family orphan 2	212632	https://www.ncbi.nlm.nih.gov/gene/?term=212632
Dcx	Dcx	doublecortin	13193	https://www.ncbi.nlm.nih.gov/gene/?term=13193
Tmod2	Tmod2	tropomodulin 2	50876	https://www.ncbi.nlm.nih.gov/gene/?term=50876
Stambp	Stambp	STAM binding protein	70527	https://www.ncbi.nlm.nih.gov/gene/?term=70527
Phactr3	Phactr3	phosphatase and actin regulator 3	74189	https://www.ncbi.nlm.nih.gov/gene/?term=74189
Cnih3	Cnih3	cornichon family AMPA receptor auxiliary protein 3	72978	https://www.ncbi.nlm.nih.gov/gene/?term=72978

Biology Open • Supplementary information

Kcnj15	Kcnj15	potassium inwardly-rectifying channel, subfamily J, member 15	16516	https://www.ncbi.nlm.nih.gov/gene/?term=16516
Znrf3	Znrf3	zinc and ring finger 3	407821	https://www.ncbi.nlm.nih.gov/gene/?term=407821
Maf	Maf	avian musculoaponeurotic fibrosarcoma oncogene homolog	17132	https://www.ncbi.nlm.nih.gov/gene/?term=17132
Wiz	Wiz	widely-interspaced zinc finger motifs	22404	https://www.ncbi.nlm.nih.gov/gene/?term=22404
Fbxo31	Fbxo31	F-box protein 31	76454	https://www.ncbi.nlm.nih.gov/gene/?term=76454
Bmp4	Bmp4	bone morphogenetic protein 4	12159	https://www.ncbi.nlm.nih.gov/gene/?term=12159
Thada	Thada	thyroid adenoma associated	240174	https://www.ncbi.nlm.nih.gov/gene/?term=240174
Lhx6	Lhx6	LIM homeobox protein 6	16874	https://www.ncbi.nlm.nih.gov/gene/?term=16874
Gng2	Gng2	guanine nucleotide binding protein (G protein), gamma 2	14702	https://www.ncbi.nlm.nih.gov/gene/?term=14702

Biology Open • Supplementary information

Asap3	Asap3	ArfGAP with SH3 domain, ankyrin repeat and PH domain 3	230837	https://www.ncbi.nlm.nih.gov/gene/?term=230837
Rnf44	Rnf44	ring finger protein 44	105239	https://www.ncbi.nlm.nih.gov/gene/?term=105239
Zfp575	Zfp575	zinc finger protein 575	101544	https://www.ncbi.nlm.nih.gov/gene/?term=101544
Cyp2j12	Cyp2j12	cytochrome P450, family 2, subfamily j, polypeptide 12	242546	https://www.ncbi.nlm.nih.gov/gene/?term=242546
Acpp	Acpp	acid phosphatase, prostate	56318	https://www.ncbi.nlm.nih.gov/gene/?term=56318
Zfp266	Zfp266	zinc finger protein 266	77519	https://www.ncbi.nlm.nih.gov/gene/?term=77519
Stxbp5l	Stxbp5l	syntaxin binding protein 5-like	207227	https://www.ncbi.nlm.nih.gov/gene/?term=207227
Sdf4	Sdf4	stromal cell derived factor 4	20318	https://www.ncbi.nlm.nih.gov/gene/?term=20318
Tcte1	Tcte1	t-complex-associated testis expressed 1	21645	https://www.ncbi.nlm.nih.gov/gene/?term=21645

Biology Open • Supplementary information

Gm14434	Gm14434	predicted gene 14434	668039	https://www.ncbi.nlm.nih.gov/gene/?term=668039
Rsph4a	Rsph4a	radial spoke head 4 homolog A (Chlamydomonas)	212892	https://www.ncbi.nlm.nih.gov/gene/?term=212892
Gata4	Gata4	GATA binding protein 4	14463	https://www.ncbi.nlm.nih.gov/gene/?term=14463
Cd84	Cd84	CD84 antigen	12523	https://www.ncbi.nlm.nih.gov/gene/?term=12523
Prkca	Prkca	protein kinase C, alpha	18750	https://www.ncbi.nlm.nih.gov/gene/?term=18750
Napb	Napb	N-ethylmaleimide sensitive fusion protein attachment protein beta	17957	https://www.ncbi.nlm.nih.gov/gene/?term=17957
Vapb	Vapb	vesicle-associated membrane protein, associated protein B and C	56491	https://www.ncbi.nlm.nih.gov/gene/?term=56491
Cacna2d2	Cacna2d2	calcium channel, voltage-dependent, alpha 2/delta subunit 2	56808	https://www.ncbi.nlm.nih.gov/gene/?term=56808
Mex3a	Mex3a	mex3 RNA binding family member A	72640	https://www.ncbi.nlm.nih.gov/gene/?term=72640

Biology Open • Supplementary information

Cbln3	Cbln3	cerebellin 3 precursor protein	56410	https://www.ncbi.nlm.nih.gov/gene/?term=56410
Rab7	Rab7	RAB7, member RAS oncogene family	19349	https://www.ncbi.nlm.nih.gov/gene/?term=19349
Vti1a	Vti1a	vesicle transport through interaction with t-SNAREs 1A	53611	https://www.ncbi.nlm.nih.gov/gene/?term=53611
Dapp1	Dapp1	dual adaptor for phosphotyrosine and 3-phosphoinositides 1	26377	https://www.ncbi.nlm.nih.gov/gene/?term=26377
Rnft2	Rnft2	ring finger protein, transmembrane 2	269695	https://www.ncbi.nlm.nih.gov/gene/?term=269695
Rit2	Rit2	Ras-like without CAAX 2	19762	https://www.ncbi.nlm.nih.gov/gene/?term=19762
Rassf2	Rassf2	Ras association (RalGDS/AF-6) domain family member 2	215653	https://www.ncbi.nlm.nih.gov/gene/?term=215653
Deptor	Deptor	DEP domain containing MTOR-interacting protein	97998	https://www.ncbi.nlm.nih.gov/gene/?term=97998
1810065E05Rik	1810065E05Rik	RIKEN cDNA 1810065E05 gene	69864	https://www.ncbi.nlm.nih.gov/gene/?term=69864

Biology Open • Supplementary information

Cfap74	Cfap74	cilia and flagella associated protein 74	544678	https://www.ncbi.nlm.nih.gov/gene/?term=544678
Bmpr2	Bmpr2	bone morphogenetic protein receptor, type II (serine/threonine kinase)	12168	https://www.ncbi.nlm.nih.gov/gene/?term=12168
Hrh1	Hrh1	histamine receptor H1	15465	https://www.ncbi.nlm.nih.gov/gene/?term=15465
Ceacam2	Ceacam2	carcinoembryonic antigen-related cell adhesion molecule 2	26367	https://www.ncbi.nlm.nih.gov/gene/?term=26367
Cd300ld	Cd300ld	CD300 molecule like family member d	217305	https://www.ncbi.nlm.nih.gov/gene/?term=217305
Pura	Pura	purine rich element binding protein A	19290	https://www.ncbi.nlm.nih.gov/gene/?term=19290
Pstpip2	Pstpip2	proline-serine-threonine phosphatase-interacting protein 2	19201	https://www.ncbi.nlm.nih.gov/gene/?term=19201
Gpr45	Gpr45	G protein-coupled receptor 45	93690	https://www.ncbi.nlm.nih.gov/gene/?term=93690
Cdh7	Cdh7	cadherin 7, type 2	241201	https://www.ncbi.nlm.nih.gov/gene/?term=241201

Biology Open • Supplementary information

Muc13	Muc13	mucin 13, epithelial transmembrane	17063	https://www.ncbi.nlm.nih.gov/gene/?term=17063
Snap25	Snap25	synaptosomal-associated protein 25	20614	https://www.ncbi.nlm.nih.gov/gene/?term=20614
Sema6a	Sema6a	sema domain, transmembrane domain (TM), and cytoplasmic domain, (semaphorin) 6A	20358	https://www.ncbi.nlm.nih.gov/gene/?term=20358
Fsd1l	Fsd1l	fibronectin type III and SPRY domain containing 1-like	319636	https://www.ncbi.nlm.nih.gov/gene/?term=319636
Kcnj16	Kcnj16	potassium inwardly-rectifying channel, subfamily J, member 16	16517	https://www.ncbi.nlm.nih.gov/gene/?term=16517
Flnb	Flnb	filamin, beta	286940	https://www.ncbi.nlm.nih.gov/gene/?term=286940
Pcdhga10	Pcdhga10	protocadherin gamma subfamily A, 10	93722	https://www.ncbi.nlm.nih.gov/gene/?term=93722
Mllt3	Mllt3	myeloid/lymphoid or mixed-lineage leukemia; translocated to, 3	70122	https://www.ncbi.nlm.nih.gov/gene/?term=70122
Dcdc2a	Dcdc2a	doublecortin domain containing 2a	195208	https://www.ncbi.nlm.nih.gov/gene/?term=195208

Biology Open • Supplementary information

Cyp4a31	Cyp4a31	cytochrome P450, family 4, subfamily a, polypeptide 31	666168	https://www.ncbi.nlm.nih.gov/gene/?term=666168
Hspb7	Hspb7	heat shock protein family, member 7 (cardiovascular)	29818	https://www.ncbi.nlm.nih.gov/gene/?term=29818
Lsm1	Lsm1	LSM1 homolog, mRNA degradation associated	67207	https://www.ncbi.nlm.nih.gov/gene/?term=67207
Coq8a	Coq8a	coenzyme Q8A	67426	https://www.ncbi.nlm.nih.gov/gene/?term=67426
Actr1b	Actr1b	ARP1 actin-related protein 1B, centractin beta	226977	https://www.ncbi.nlm.nih.gov/gene/?term=226977
Cd47	Cd47	CD47 antigen (Rh-related antigen, integrin-associated signal transducer)	16423	https://www.ncbi.nlm.nih.gov/gene/?term=16423
Ackr4	Ackr4	atypical chemokine receptor 4	252837	https://www.ncbi.nlm.nih.gov/gene/?term=252837
Mdm2	Mdm2	transformed mouse 3T3 cell double minute 2	17246	https://www.ncbi.nlm.nih.gov/gene/?term=17246
Arhgap26	Arhgap26	Rho GTPase activating protein 26	71302	https://www.ncbi.nlm.nih.gov/gene/?term=71302

Onecut3	Onecut3	one cut domain, family member 3	246086	https://www.ncbi.nlm.nih.gov/gene/?term=246086
Hpca	Hpca	hippocalcin	15444	https://www.ncbi.nlm.nih.gov/gene/?term=15444
Tmem52b	Tmem52b	transmembrane protein 52B	330428	https://www.ncbi.nlm.nih.gov/gene/?term=330428
Spx	Spx	spexin hormone	319552	https://www.ncbi.nlm.nih.gov/gene/?term=319552
Ccdc115	Ccdc115	coiled-coil domain containing 115	69668	https://www.ncbi.nlm.nih.gov/gene/?term=69668
Dhfr	Dhfr	dihydrofolate reductase	13361	https://www.ncbi.nlm.nih.gov/gene/?term=13361
Fam174b	Fam174b	family with sequence similarity 174, member B	100038347	https://www.ncbi.nlm.nih.gov/gene/?term=100038
Lnx2	Lnx2	ligand of numb-protein X 2	140887	https://www.ncbi.nlm.nih.gov/gene/?term=140887
Xk	Xk	X-linked Kx blood group	22439	https://www.ncbi.nlm.nih.gov/gene/?term=22439

Xylb	Xylb	xylulokinase homolog (H. influenzae)	102448	https://www.ncbi.nlm.nih.gov/gene/?term=102448
Ptchd1	Ptchd1	patched domain containing 1	211612	https://www.ncbi.nlm.nih.gov/gene/?term=211612
Pogk	Pogk	pogo transposable element with KRAB domain	71592	https://www.ncbi.nlm.nih.gov/gene/?term=71592
Kcnc1	Kcnc1	potassium voltage gated channel, Shaw-related subfamily, member 1	16502	https://www.ncbi.nlm.nih.gov/gene/?term=16502
Tnr	Tnr	tenascin R	21960	https://www.ncbi.nlm.nih.gov/gene/?term=21960
Homer2	Homer2	homer scaffolding protein 2	26557	https://www.ncbi.nlm.nih.gov/gene/?term=26557
Nfya	Nfya	nuclear transcription factor-Y alpha	18044	https://www.ncbi.nlm.nih.gov/gene/?term=18044
2810021J22Rik	2810021J22Rik	RIKEN cDNA 2810021J22 gene	69944	https://www.ncbi.nlm.nih.gov/gene/?term=69944
Greb1	Greb1	gene regulated by estrogen in breast cancer protein	268527	https://www.ncbi.nlm.nih.gov/gene/?term=268527

Zfp994	Zfp994	zinc finger protein 994	240038	https://www.ncbi.nlm.nih.gov/gene/?term=240038
Slc39a14	Slc39a14	solute carrier family 39 (zinc transporter), member 14	213053	https://www.ncbi.nlm.nih.gov/gene/?term=213053
Cxcl15	Cxcl15	chemokine (C-X-C motif) ligand 15	20309	https://www.ncbi.nlm.nih.gov/gene/?term=20309
Tnfrsf13c	Tnfrsf13c	tumor necrosis factor receptor superfamily, member 13c	72049	https://www.ncbi.nlm.nih.gov/gene/?term=72049
Zfp655	Zfp655	zinc finger protein 655	72611	https://www.ncbi.nlm.nih.gov/gene/?term=72611
Nmrk2	Nmrk2	nicotinamide riboside kinase 2	69564	https://www.ncbi.nlm.nih.gov/gene/?term=69564
Pard3b	Pard3b	par-3 family cell polarity regulator beta	72823	https://www.ncbi.nlm.nih.gov/gene/?term=72823
Usp45	Usp45	ubiquitin specific petidase 45	77593	https://www.ncbi.nlm.nih.gov/gene/?term=77593
Abcc9	Abcc9	ATP-binding cassette, sub-family C (CFTR/MRP), member 9	20928	https://www.ncbi.nlm.nih.gov/gene/?term=20928

Biology Open • Supplementary information

Pcdhgb7	Pcdhgb7	protocadherin gamma subfamily B, 7	93704	https://www.ncbi.nlm.nih.gov/gene/?term=93704
Nmrk1	Nmrk1	nicotinamide riboside kinase 1	225994	https://www.ncbi.nlm.nih.gov/gene/?term=225994
Lrtm2	Lrtm2	leucine-rich repeats and transmembrane domains 2	211187	https://www.ncbi.nlm.nih.gov/gene/?term=211187
Alox8	Alox8	arachidonate 8-lipoxygenase	11688	https://www.ncbi.nlm.nih.gov/gene/?term=11688
Triobp	Triobp	TRIO and F-actin binding protein	110253	https://www.ncbi.nlm.nih.gov/gene/?term=110253
Grk3	Grk3	G protein-coupled receptor kinase 3	320129	https://www.ncbi.nlm.nih.gov/gene/?term=320129
Trim67	Trim67	tripartite motif-containing 67	330863	https://www.ncbi.nlm.nih.gov/gene/?term=330863
Siah2	Siah2	siah E3 ubiquitin protein ligase 2	20439	https://www.ncbi.nlm.nih.gov/gene/?term=20439
Cer1	Cer1	cerberus 1, DAN family BMP antagonist	12622	https://www.ncbi.nlm.nih.gov/gene/?term=12622

Biology Open • Supplementary information

Prelid3a	Prelid3a	PRELI domain containing 3A	225655	https://www.ncbi.nlm.nih.gov/gene/?term=225655
Ift57	Ift57	intraflagellar transport 57	73916	https://www.ncbi.nlm.nih.gov/gene/?term=73916
Pgm5	Pgm5	phosphoglucomutase 5	226041	https://www.ncbi.nlm.nih.gov/gene/?term=226041
Arhgdib	Arhgdib	Rho, GDP dissociation inhibitor (GDI) beta	11857	https://www.ncbi.nlm.nih.gov/gene/?term=11857
Rhobtb1	Rhobtb1	Rho-related BTB domain containing 1	69288	https://www.ncbi.nlm.nih.gov/gene/?term=69288
Smo	Smo	smoothened, frizzled class receptor	319757	https://www.ncbi.nlm.nih.gov/gene/?term=319757
Snap23	Snap23	synaptosomal-associated protein 23	20619	https://www.ncbi.nlm.nih.gov/gene/?term=20619
Prkcg	Prkcg	protein kinase C, gamma	18752	https://www.ncbi.nlm.nih.gov/gene/?term=18752
Prdm12	Prdm12	PR domain containing 12	381359	https://www.ncbi.nlm.nih.gov/gene/?term=381359

Biology Open • Supplementary information

Cd160	Cd160	CD160 antigen	54215	https://www.ncbi.nlm.nih.gov/gene/?term=54215
Ms4a5	Ms4a5	membrane-spanning 4-domains, subfamily A, member 5	269063	https://www.ncbi.nlm.nih.gov/gene/?term=269063
Srxn1	Srxn1	sulfiredoxin 1 homolog (S. cerevisiae)	76650	https://www.ncbi.nlm.nih.gov/gene/?term=76650
Adgra1	Adgra1	adhesion G protein-coupled receptor A1	52389	https://www.ncbi.nlm.nih.gov/gene/?term=52389
Prr5l	Prr5l	proline rich 5 like	72446	https://www.ncbi.nlm.nih.gov/gene/?term=72446
Csf2ra	Csf2ra	colony stimulating factor 2 receptor, alpha, low-affinity (granulocyte-macrophage)	12982	https://www.ncbi.nlm.nih.gov/gene/?term=12982
Zfp329	Zfp329	zinc finger protein 329	67230	https://www.ncbi.nlm.nih.gov/gene/?term=67230
Kcnip3	Kcnip3	Kv channel interacting protein 3, calsenilin	56461	https://www.ncbi.nlm.nih.gov/gene/?term=56461
Pigh	Pigh	phosphatidylinositol glycan anchor biosynthesis, class H	110417	https://www.ncbi.nlm.nih.gov/gene/?term=110417

Biology Open • Supplementary information

Uncx	Uncx	UNC homeobox	22255	https://www.ncbi.nlm.nih.gov/gene/?term=22255
Slc10a2	Slc10a2	solute carrier family 10, member 2	20494	https://www.ncbi.nlm.nih.gov/gene/?term=20494
Col19a1	Col19a1	collagen, type XIX, alpha 1	12823	https://www.ncbi.nlm.nih.gov/gene/?term=12823
Alg12	Alg12	asparagine-linked glycosylation 12 (alpha-1,6-mannosyltransferase)	223774	https://www.ncbi.nlm.nih.gov/gene/?term=223774
Zfp810	Zfp810	zinc finger protein 810	235050	https://www.ncbi.nlm.nih.gov/gene/?term=235050
Ms4a4c	Ms4a4c	membrane-spanning 4-domains, subfamily A, member 4C	64380	https://www.ncbi.nlm.nih.gov/gene/?term=64380
Oprm1	Oprm1	opioid receptor, mu 1	18390	https://www.ncbi.nlm.nih.gov/gene/?term=18390
Sipa1l1	Sipa1l1	signal-induced proliferation-associated 1 like 1	217692	https://www.ncbi.nlm.nih.gov/gene/?term=217692
Klhl13	Klhl13	kelch-like 13	67455	https://www.ncbi.nlm.nih.gov/gene/?term=67455

Biology Open • Supplementary information

Tirap	Tirap	toll-interleukin 1 receptor (TIR) domain-containing adaptor protein	117149	https://www.ncbi.nlm.nih.gov/gene/?term=117149
Acot2	Acot2	acyl-CoA thioesterase 2	171210	https://www.ncbi.nlm.nih.gov/gene/?term=171210
Rara	Rara	retinoic acid receptor, alpha	19401	https://www.ncbi.nlm.nih.gov/gene/?term=19401
Asb13	Asb13	ankyrin repeat and SOCS box-containing 13	142688	https://www.ncbi.nlm.nih.gov/gene/?term=142688
Tmem141	Tmem141	transmembrane protein 141	51875	https://www.ncbi.nlm.nih.gov/gene/?term=51875
Dhh	Dhh	desert hedgehog	13363	https://www.ncbi.nlm.nih.gov/gene/?term=13363
Idua	Idua	iduronidase, alpha-L	15932	https://www.ncbi.nlm.nih.gov/gene/?term=15932
Ly6g6c	Ly6g6c	lymphocyte antigen 6 complex, locus G6C	68468	https://www.ncbi.nlm.nih.gov/gene/?term=68468
Pgrmc1	Pgrmc1	progesterone receptor membrane component 1	53328	https://www.ncbi.nlm.nih.gov/gene/?term=53328

Biology Open • Supplementary information

Runx1t1	Runx1t1	RUNX1 translocation partner 1	12395	https://www.ncbi.nlm.nih.gov/gene/?term=12395
Chrnb4	Chrnb4	cholinergic receptor, nicotinic, beta polypeptide 4	108015	https://www.ncbi.nlm.nih.gov/gene/?term=108015
Sh3bp5	Sh3bp5	SH3-domain binding protein 5 (BTK-associated)	24056	https://www.ncbi.nlm.nih.gov/gene/?term=24056
Riok3	Riok3	RIO kinase 3	66878	https://www.ncbi.nlm.nih.gov/gene/?term=66878
Arhgef17	Arhgef17	Rho guanine nucleotide exchange factor (GEF) 17	207212	https://www.ncbi.nlm.nih.gov/gene/?term=207212
Slc7a1	Slc7a1	solute carrier family 7 (cationic amino acid transporter, y+ system), member 1	11987	https://www.ncbi.nlm.nih.gov/gene/?term=11987
Mtus2	Mtus2	microtubule associated tumor suppressor candidate 2	77521	https://www.ncbi.nlm.nih.gov/gene/?term=77521
Apba1	Apba1	amyloid beta (A4) precursor protein binding, family A, member 1	319924	https://www.ncbi.nlm.nih.gov/gene/?term=319924
St18	St18	suppression of tumorigenicity 18	240690	https://www.ncbi.nlm.nih.gov/gene/?term=240690

Biology Open • Supplementary information

Rfk	Rfk	riboflavin kinase	54391	https://www.ncbi.nlm.nih.gov/gene/?term=54391
Pcdh10	Pcdh10	protocadherin 10	18526	https://www.ncbi.nlm.nih.gov/gene/?term=18526
Fblim1	Fblim1	filamin binding LIM protein 1	74202	https://www.ncbi.nlm.nih.gov/gene/?term=74202
St8sia1	St8sia1	ST8 alpha-N-acetyl-neuraminide alpha-2,8-sialyltransferase 1	20449	https://www.ncbi.nlm.nih.gov/gene/?term=20449
Cdc6	Cdc6	cell division cycle 6	23834	https://www.ncbi.nlm.nih.gov/gene/?term=23834
Pcdhgc5	Pcdhgc5	protocadherin gamma subfamily C, 5	93708	https://www.ncbi.nlm.nih.gov/gene/?term=93708
Cpm	Cpm	carboxypeptidase M	70574	https://www.ncbi.nlm.nih.gov/gene/?term=70574
Urb2	Urb2	URB2 ribosome biogenesis 2 homolog (S. cerevisiae)	382038	https://www.ncbi.nlm.nih.gov/gene/?term=382038
4921509C19Rik	4921509C19Rik	RIKEN cDNA 4921509C19 gene	381393	https://www.ncbi.nlm.nih.gov/gene/?term=381393

Biology Open • Supplementary information

Ifnlr1	Ifnlr1	interferon lambda receptor 1	242700	https://www.ncbi.nlm.nih.gov/gene/?term=242700
Rgr	Rgr	retinal G protein coupled receptor	57811	https://www.ncbi.nlm.nih.gov/gene/?term=57811
Cox15	Cox15	cytochrome c oxidase assembly protein 15	226139	https://www.ncbi.nlm.nih.gov/gene/?term=226139
Mobp	Mobp	myelin-associated oligodendrocytic basic protein	17433	https://www.ncbi.nlm.nih.gov/gene/?term=17433
Lrrc61	Lrrc61	leucine rich repeat containing 61	243371	https://www.ncbi.nlm.nih.gov/gene/?term=243371
Chst3	Chst3	carbohydrate sulfotransferase 3	53374	https://www.ncbi.nlm.nih.gov/gene/?term=53374
Rp1	Rp1	retinitis pigmentosa 1 (human)	19888	https://www.ncbi.nlm.nih.gov/gene/?term=19888
Accs	Accs	1-aminocyclopropane-1-carboxylate synthase (non-functional)	329470	https://www.ncbi.nlm.nih.gov/gene/?term=329470
Slc22a8	Slc22a8	solute carrier family 22 (organic anion transporter), member 8	19879	https://www.ncbi.nlm.nih.gov/gene/?term=19879

Biology Open • Supplementary information

Prex2	Prex2	phosphatidylinositol-3,4,5-trisphosphate-dependent Rac exchange factor 2	109294	https://www.ncbi.nlm.nih.gov/gene/?term=109294
Tacr2	Tacr2	tachykinin receptor 2	21337	https://www.ncbi.nlm.nih.gov/gene/?term=21337
Rrm2	Rrm2	ribonucleotide reductase M2	20135	https://www.ncbi.nlm.nih.gov/gene/?term=20135
Serinc3	Serinc3	serine incorporator 3	26943	https://www.ncbi.nlm.nih.gov/gene/?term=26943
Nkx2-9	Nkx2-9	NK2 homeobox 9	18094	https://www.ncbi.nlm.nih.gov/gene/?term=18094
Tph1	Tph1	tryptophan hydroxylase 1	21990	https://www.ncbi.nlm.nih.gov/gene/?term=21990
Oxsm	Oxsm	3-oxoacyl-ACP synthase, mitochondrial	71147	https://www.ncbi.nlm.nih.gov/gene/?term=71147
Tns1	Tns1	tensin 1	21961	https://www.ncbi.nlm.nih.gov/gene/?term=21961
Zfp606	Zfp606	zinc finger protein 606	67370	https://www.ncbi.nlm.nih.gov/gene/?term=67370

Biology Open • Supplementary information

Nab1	Nab1	Ngfi-A binding protein 1	17936	https://www.ncbi.nlm.nih.gov/gene/?term=17936
Ddx51	Ddx51	DEAD (Asp-Glu-Ala-Asp) box polypeptide 51	69663	https://www.ncbi.nlm.nih.gov/gene/?term=69663
Otx1	Otx1	orthodenticle homeobox 1	18423	https://www.ncbi.nlm.nih.gov/gene/?term=18423
Slc2a12	Slc2a12	solute carrier family 2 (facilitated glucose transporter), member 12	353169	https://www.ncbi.nlm.nih.gov/gene/?term=353169
Sod2	Sod2	superoxide dismutase 2, mitochondrial	20656	https://www.ncbi.nlm.nih.gov/gene/?term=20656
Ank2	Ank2	ankyrin 2, brain	109676	https://www.ncbi.nlm.nih.gov/gene/?term=109676
Thsd4	Thsd4	thrombospondin, type I, domain containing 4	207596	https://www.ncbi.nlm.nih.gov/gene/?term=207596
Trpc7	Trpc7	transient receptor potential cation channel, subfamily C, member 7	26946	https://www.ncbi.nlm.nih.gov/gene/?term=26946
Unc5a	Unc5a	unc-5 netrin receptor A	107448	https://www.ncbi.nlm.nih.gov/gene/?term=107448

Biology Open • Supplementary information

Nrk	Nrk	Nik related kinase	27206	https://www.ncbi.nlm.nih.gov/gene/?term=27206
Elfn2	Elfn2	leucine rich repeat and fibronectin type III, extracellular 2	207393	https://www.ncbi.nlm.nih.gov/gene/?term=207393
Pcdhga2	Pcdhga2	protocadherin gamma subfamily A, 2	93710	https://www.ncbi.nlm.nih.gov/gene/?term=93710
Dgkg	Dgkg	diacylglycerol kinase, gamma	110197	https://www.ncbi.nlm.nih.gov/gene/?term=110197
Gm4841	Gm4841	predicted gene 4841	225594	https://www.ncbi.nlm.nih.gov/gene/?term=225594
Acss1	Acss1	acyl-CoA synthetase short-chain family member 1	68738	https://www.ncbi.nlm.nih.gov/gene/?term=68738
Pou2f2	Pou2f2	POU domain, class 2, transcription factor 2	18987	https://www.ncbi.nlm.nih.gov/gene/?term=18987
Foxn1	Foxn1	forkhead box N1	15218	https://www.ncbi.nlm.nih.gov/gene/?term=15218
Pirt	Pirt	phosphoinositide-interacting regulator of transient receptor potential channels	193003	https://www.ncbi.nlm.nih.gov/gene/?term=193003

Biology Open • Supplementary information

Eef2k	Eef2k	eukaryotic elongation factor-2 kinase	13631	https://www.ncbi.nlm.nih.gov/gene/?term=13631
Ascl4	Ascl4	achaete-scute family bHLH transcription factor 4	67341	https://www.ncbi.nlm.nih.gov/gene/?term=67341
2610002M06Rik	2610002M06Rik	RIKEN cDNA 2610002M06 gene	67028	https://www.ncbi.nlm.nih.gov/gene/?term=67028
Gna13	Gna13	guanine nucleotide binding protein, alpha 13	14674	https://www.ncbi.nlm.nih.gov/gene/?term=14674
Krt222	Krt222	keratin 222	268481	https://www.ncbi.nlm.nih.gov/gene/?term=268481
Cdh12	Cdh12	cadherin 12	215654	https://www.ncbi.nlm.nih.gov/gene/?term=215654
Cds2	Cds2	CDP-diacylglycerol synthase (phosphatidate cytidylyltransferase) 2	110911	https://www.ncbi.nlm.nih.gov/gene/?term=110911
Tyw3	Tyw3	tRNA-yW synthesizing protein 3 homolog (S. cerevisiae)	209584	https://www.ncbi.nlm.nih.gov/gene/?term=209584
Flrt1	Flrt1	fibronectin leucine rich transmembrane protein 1	396184	https://www.ncbi.nlm.nih.gov/gene/?term=396184

Biology Open • Supplementary information

Fbrs	Fbrs	fibrosin	14123	https://www.ncbi.nlm.nih.gov/gene/?term=14123
Rab9b	Rab9b	RAB9B, member RAS oncogene family	319642	https://www.ncbi.nlm.nih.gov/gene/?term=319642
Cgnl1	Cgnl1	cingulin-like 1	68178	https://www.ncbi.nlm.nih.gov/gene/?term=68178
Ugt2b34	Ugt2b34	UDP glucuronosyltransferase 2 family, polypeptide B34	100727	https://www.ncbi.nlm.nih.gov/gene/?term=100727
Cacna1e	Cacna1e	calcium channel, voltage-dependent, R type, alpha 1E subunit	12290	https://www.ncbi.nlm.nih.gov/gene/?term=12290
Pdzrn3	Pdzrn3	PDZ domain containing RING finger 3	55983	https://www.ncbi.nlm.nih.gov/gene/?term=55983
Bdp1	Bdp1	B double prime 1, subunit of RNA polymerase III transcription initiation factor IIIB	544971	https://www.ncbi.nlm.nih.gov/gene/?term=544971
Rslcan18	Rslcan18	regulator of sex-limitation candidate 18	432770	https://www.ncbi.nlm.nih.gov/gene/?term=432770
Ceacam1	Ceacam1	carcinoembryonic antigen-related cell adhesion molecule 1	26365	https://www.ncbi.nlm.nih.gov/gene/?term=26365

Biology Open • Supplementary information

Zfp691	Zfp691	zinc finger protein 691	195522	https://www.ncbi.nlm.nih.gov/gene/?term=195522
Adamts14	Adamts14	a disintegrin-like and metallopeptidase (reprolysin type) with thrombospondin type 1 motif, 14	237360	https://www.ncbi.nlm.nih.gov/gene/?term=237360
Dph6	Dph6	diphthamine biosynthesis 6	66632	https://www.ncbi.nlm.nih.gov/gene/?term=66632
Ago3	Ago3	argonaute RISC catalytic subunit 3	214150	https://www.ncbi.nlm.nih.gov/gene/?term=214150
Slc22a15	Slc22a15	solute carrier family 22 (organic anion/cation transporter), member 15	242126	https://www.ncbi.nlm.nih.gov/gene/?term=242126
Sv2b	Sv2b	synaptic vesicle glycoprotein 2 b	64176	https://www.ncbi.nlm.nih.gov/gene/?term=64176
Casp8	Casp8	caspase 8	12370	https://www.ncbi.nlm.nih.gov/gene/?term=12370
Senp1	Senp1	SUMO1/sentrin specific peptidase 1	223870	https://www.ncbi.nlm.nih.gov/gene/?term=223870
Fam169b	Fam169b	family with sequence similarity 169, member B	434197	https://www.ncbi.nlm.nih.gov/gene/?term=434197

Biology Open • Supplementary information

Rnasel	Rnasel	ribonuclease L (2', 5'-oligoisoadenylate synthetase-dependent)	24014	https://www.ncbi.nlm.nih.gov/gene/?term=24014
Ccdc85a	Ccdc85a	coiled-coil domain containing 85A	216613	https://www.ncbi.nlm.nih.gov/gene/?term=216613
Ctse	Ctse	cathepsin E	13034	https://www.ncbi.nlm.nih.gov/gene/?term=13034
Itga11	Itga11	integrin alpha 11	319480	https://www.ncbi.nlm.nih.gov/gene/?term=319480
Dlg4	Dlg4	discs large MAGUK scaffold protein 4	13385	https://www.ncbi.nlm.nih.gov/gene/?term=13385
Igsf6	Igsf6	immunoglobulin superfamily, member 6	80719	https://www.ncbi.nlm.nih.gov/gene/?term=80719
Wipf3	Wipf3	WAS/WASL interacting protein family, member 3	330319	https://www.ncbi.nlm.nih.gov/gene/?term=330319
Rabl2	Rabl2	RAB, member RAS oncogene family-like 2	68708	https://www.ncbi.nlm.nih.gov/gene/?term=68708
Ttc4	Ttc4	tetratricopeptide repeat domain 4	72354	https://www.ncbi.nlm.nih.gov/gene/?term=72354

Cd4	Cd4	CD4 antigen	12504	https://www.ncbi.nlm.nih.gov/gene/?term=12504
Tc2n	Tc2n	tandem C2 domains, nuclear	74413	https://www.ncbi.nlm.nih.gov/gene/?term=74413
Tpgs2	Tpgs2	tubulin polyglutamylase complex subunit 2	66648	https://www.ncbi.nlm.nih.gov/gene/?term=66648
Prss42	Prss42	protease, serine 42	235628	https://www.ncbi.nlm.nih.gov/gene/?term=235628
Rspo1	Rspo1	R-spondin 1	192199	https://www.ncbi.nlm.nih.gov/gene/?term=192199
Pou3f4	Pou3f4	POU domain, class 3, transcription factor 4	18994	https://www.ncbi.nlm.nih.gov/gene/?term=18994
Tsn	Tsn	translin	22099	https://www.ncbi.nlm.nih.gov/gene/?term=22099
Gdpgp1	Gdpgp1	GDP-D-glucose phosphorylase 1	269952	https://www.ncbi.nlm.nih.gov/gene/?term=269952
Nr4a2	Nr4a2	nuclear receptor subfamily 4, group A, member 2	18227	https://www.ncbi.nlm.nih.gov/gene/?term=18227

Saa4	Saa4	serum amyloid A 4	20211	https://www.ncbi.nlm.nih.gov/gene/?term=20211
Cntn2	Cntn2	contactin 2	21367	https://www.ncbi.nlm.nih.gov/gene/?term=21367
Irgm2	Irgm2	immunity-related GTPase family M member 2	54396	https://www.ncbi.nlm.nih.gov/gene/?term=54396
Grik3	Grik3	glutamate receptor, ionotropic, kainate 3	14807	https://www.ncbi.nlm.nih.gov/gene/?term=14807
Smpd4	Smpd4	sphingomyelin phosphodiesterase 4	77626	https://www.ncbi.nlm.nih.gov/gene/?term=77626
Ppp1r16b	Ppp1r16b	protein phosphatase 1, regulatory subunit 16B	228852	https://www.ncbi.nlm.nih.gov/gene/?term=228852
Cox10	Cox10	heme A:farnesyltransferase cytochrome c oxidase assembly factor 10	70383	https://www.ncbi.nlm.nih.gov/gene/?term=70383
Ptprb	Ptprb	protein tyrosine phosphatase, receptor type, B	19263	https://www.ncbi.nlm.nih.gov/gene/?term=19263
Sp100	Sp100	nuclear antigen Sp100	20684	https://www.ncbi.nlm.nih.gov/gene/?term=20684

Biology Open • Supplementary information

Aptx	Aptx	aprataxin	66408	https://www.ncbi.nlm.nih.gov/gene/?term=66408
Ccna2	Ccna2	cyclin A2	12428	https://www.ncbi.nlm.nih.gov/gene/?term=12428
Pou3f2	Pou3f2	POU domain, class 3, transcription factor 2	18992	https://www.ncbi.nlm.nih.gov/gene/?term=18992
Draxin	Draxin	dorsal inhibitory axon guidance protein	70433	https://www.ncbi.nlm.nih.gov/gene/?term=70433
Zbtb7b	Zbtb7b	zinc finger and BTB domain containing 7B	22724	https://www.ncbi.nlm.nih.gov/gene/?term=22724
Fbxw5	Fbxw5	F-box and WD-40 domain protein 5	30839	https://www.ncbi.nlm.nih.gov/gene/?term=30839
Acp1	Acp1	acid phosphatase 1, soluble	11431	https://www.ncbi.nlm.nih.gov/gene/?term=11431
Gucy2e	Gucy2e	guanylate cyclase 2e	14919	https://www.ncbi.nlm.nih.gov/gene/?term=14919
Nmnat2	Nmnat2	nicotinamide nucleotide adenylyltransferase 2	226518	https://www.ncbi.nlm.nih.gov/gene/?term=226518

Biology Open • Supplementary information

Stk32a	Stk32a	serine/threonine kinase 32A	269019	https://www.ncbi.nlm.nih.gov/gene/?term=269019
Ctdsp2	Ctdsp2	CTD (carboxy-terminal domain, RNA polymerase II, polypeptide A) small phosphatase 2	52468	https://www.ncbi.nlm.nih.gov/gene/?term=52468
Yipf6	Yipf6	Yip1 domain family, member 6	77929	https://www.ncbi.nlm.nih.gov/gene/?term=77929
Nufip1	Nufip1	nuclear fragile X mental retardation protein interacting protein 1	27275	https://www.ncbi.nlm.nih.gov/gene/?term=27275
Pecam1	Pecam1	platelet/endothelial cell adhesion molecule 1	18613	https://www.ncbi.nlm.nih.gov/gene/?term=18613
Rgs5	Rgs5	regulator of G-protein signaling 5	19737	https://www.ncbi.nlm.nih.gov/gene/?term=19737
Mapk10	Mapk10	mitogen-activated protein kinase 10	26414	https://www.ncbi.nlm.nih.gov/gene/?term=26414
Cmah	Cmah	cytidine monophospho-N-acetylneuraminic acid hydroxylase	12763	https://www.ncbi.nlm.nih.gov/gene/?term=12763
Unc93a	Unc93a	unc-93 homolog A	381058	https://www.ncbi.nlm.nih.gov/gene/?term=381058

Biology Open • Supplementary information

Pcdhga1	Pcdhga1	protocadherin gamma subfamily A, 1	93709	https://www.ncbi.nlm.nih.gov/gene/?term=93709
B3gnt9	B3gnt9	UDP-GlcNAc:betaGal beta-1,3-N-acetylglucosaminyltransferase 9	97440	https://www.ncbi.nlm.nih.gov/gene/?term=97440
Sh2d2a	Sh2d2a	SH2 domain containing 2A	27371	https://www.ncbi.nlm.nih.gov/gene/?term=27371
Ap3s2	Ap3s2	adaptor-related protein complex 3, sigma 2 subunit	11778	https://www.ncbi.nlm.nih.gov/gene/?term=11778
Camk2a	Camk2a	calcium/calmodulin-dependent protein kinase II alpha	12322	https://www.ncbi.nlm.nih.gov/gene/?term=12322
Pfn1	Pfn1	profilin 1	18643	https://www.ncbi.nlm.nih.gov/gene/?term=18643
Bgn	Bgn	biglycan	12111	https://www.ncbi.nlm.nih.gov/gene/?term=12111
Cyb5r3	Cyb5r3	cytochrome b5 reductase 3	109754	https://www.ncbi.nlm.nih.gov/gene/?term=109754
Rmi1	Rmi1	RecQ mediated genome instability 1	74386	https://www.ncbi.nlm.nih.gov/gene/?term=74386

Shroom3	Shroom3	shroom family member 3	27428	https://www.ncbi.nlm.nih.gov/gene/?term=27428
Gnal	Gnal	guanine nucleotide binding protein, alpha stimulating, olfactory type	14680	https://www.ncbi.nlm.nih.gov/gene/?term=14680
Havcr2	Havcr2	hepatitis A virus cellular receptor 2	171285	https://www.ncbi.nlm.nih.gov/gene/?term=171285
Ddi2	Ddi2	DNA-damage inducible protein 2	68817	https://www.ncbi.nlm.nih.gov/gene/?term=68817
Chrnd	Chrnd	cholinergic receptor, nicotinic, delta polypeptide	11447	https://www.ncbi.nlm.nih.gov/gene/?term=11447
Mcidas	Mcidas	multiciliate differentiation and DNA synthesis associated cell cycle protein	622408	https://www.ncbi.nlm.nih.gov/gene/?term=622408
Pcdhga3	Pcdhga3	protocadherin gamma subfamily A, 3	93711	https://www.ncbi.nlm.nih.gov/gene/?term=93711
Map3k7	Map3k7	mitogen-activated protein kinase kinase kinase 7	26409	https://www.ncbi.nlm.nih.gov/gene/?term=26409
Pitpnc1	Pitpnc1	phosphatidylinositol transfer protein, cytoplasmic 1	71795	https://www.ncbi.nlm.nih.gov/gene/?term=71795

Biology Open • Supplementary information

Pcdhga8	Pcdhga8	protocadherin gamma subfamily A, 8	93716	https://www.ncbi.nlm.nih.gov/gene/?term=93716
Mtmr12	Mtmr12	myotubularin related protein 12	268783	https://www.ncbi.nlm.nih.gov/gene/?term=268783
Unc13b	Unc13b	unc-13 homolog B	22249	https://www.ncbi.nlm.nih.gov/gene/?term=22249
Kirrel3	Kirrel3	kirre like nephrin family adhesion molecule 3	67703	https://www.ncbi.nlm.nih.gov/gene/?term=67703
Sike1	Sike1	suppressor of IKBKE 1	66641	https://www.ncbi.nlm.nih.gov/gene/?term=66641
Cgas	Cgas	cyclic GMP-AMP synthase	214763	https://www.ncbi.nlm.nih.gov/gene/?term=214763
Cradd	Cradd	CASP2 and RIPK1 domain containing adaptor with death domain	12905	https://www.ncbi.nlm.nih.gov/gene/?term=12905
Rad18	Rad18	RAD18 E3 ubiquitin protein ligase	58186	https://www.ncbi.nlm.nih.gov/gene/?term=58186
Prkn	Prkn	parkin RBR E3 ubiquitin protein ligase	50873	https://www.ncbi.nlm.nih.gov/gene/?term=50873

Biology Open • Supplementary information

P4ha3	P4ha3	procollagen-proline, 2-oxoglutarate 4-dioxygenase (proline 4-hydroxylase), alpha polypeptide III	320452	https://www.ncbi.nlm.nih.gov/gene/?term=320452
Meltf	Meltf	melanotransferrin	30060	https://www.ncbi.nlm.nih.gov/gene/?term=30060
Pik3r5	Pik3r5	NA	320207	https://www.ncbi.nlm.nih.gov/gene/?term=320207
Igf2bp2	Igf2bp2	insulin-like growth factor 2 mRNA binding protein 2	319765	https://www.ncbi.nlm.nih.gov/gene/?term=319765
Rab6b	Rab6b	RAB6B, member RAS oncogene family	270192	https://www.ncbi.nlm.nih.gov/gene/?term=270192
Gas2l1	Gas2l1	growth arrest-specific 2 like 1	78926	https://www.ncbi.nlm.nih.gov/gene/?term=78926
Prrc2b	Prrc2b	proline-rich coiled-coil 2B	227723	https://www.ncbi.nlm.nih.gov/gene/?term=227723
Fndc7	Fndc7	fibronectin type III domain containing 7	320181	https://www.ncbi.nlm.nih.gov/gene/?term=320181
Fcrl6	Fcrl6	Fc receptor-like 6	677296	https://www.ncbi.nlm.nih.gov/gene/?term=677296

Biology Open • Supplementary information

Pdcd4	Pdcd4	programmed cell death 4	18569	https://www.ncbi.nlm.nih.gov/gene/?term=18569
Igfbp3	Igfbp3	insulin-like growth factor binding protein 3	16009	https://www.ncbi.nlm.nih.gov/gene/?term=16009
Neurod2	Neurod2	neurogenic differentiation 2	18013	https://www.ncbi.nlm.nih.gov/gene/?term=18013
Sri	Sri	sorcin	109552	https://www.ncbi.nlm.nih.gov/gene/?term=109552
Enpp1	Enpp1	ectonucleotide pyrophosphatase/phosphodiesterase 1	18605	https://www.ncbi.nlm.nih.gov/gene/?term=18605
Cdh6	Cdh6	cadherin 6	12563	https://www.ncbi.nlm.nih.gov/gene/?term=12563
BC030500	BC030500	cDNA sequence BC030500	234290	https://www.ncbi.nlm.nih.gov/gene/?term=234290
Glyr1	Glyr1	glyoxylate reductase 1 homolog (Arabidopsis)	74022	https://www.ncbi.nlm.nih.gov/gene/?term=74022
Ric8b	Ric8b	RIC8 guanine nucleotide exchange factor B	237422	https://www.ncbi.nlm.nih.gov/gene/?term=237422

Biology Open • Supplementary information

Aldh8a1	Aldh8a1	aldehyde dehydrogenase 8 family, member A1	237320	https://www.ncbi.nlm.nih.gov/gene/?term=237320
Flt4	Flt4	FMS-like tyrosine kinase 4	14257	https://www.ncbi.nlm.nih.gov/gene/?term=14257
Zfp39	Zfp39	zinc finger protein 39	22698	https://www.ncbi.nlm.nih.gov/gene/?term=22698
Cntn3	Cntn3	contactin 3	18488	https://www.ncbi.nlm.nih.gov/gene/?term=18488
Ado	Ado	2-aminoethanethiol (cysteamine) dioxygenase	211488	https://www.ncbi.nlm.nih.gov/gene/?term=211488
Snurf	Snurf	SNRPN upstream reading frame	84704	https://www.ncbi.nlm.nih.gov/gene/?term=84704
Pcdhga11	Pcdhga11	protocadherin gamma subfamily A, 11	93723	https://www.ncbi.nlm.nih.gov/gene/?term=93723
Heatr6	Heatr6	HEAT repeat containing 6	217026	https://www.ncbi.nlm.nih.gov/gene/?term=217026
Acsm2	Acsm2	acyl-CoA synthetase medium-chain family member 2	233799	https://www.ncbi.nlm.nih.gov/gene/?term=233799

Biology Open • Supplementary information

Gatc	Gatc	glutamyl-tRNA(Gln) amidotransferase, subunit C	384281	https://www.ncbi.nlm.nih.gov/gene/?term=384281
Il22ra1	Il22ra1	interleukin 22 receptor, alpha 1	230828	https://www.ncbi.nlm.nih.gov/gene/?term=230828
Acot4	Acot4	acyl-CoA thioesterase 4	171282	https://www.ncbi.nlm.nih.gov/gene/?term=171282
Setd7	Setd7	SET domain containing (lysine methyltransferase) 7	73251	https://www.ncbi.nlm.nih.gov/gene/?term=73251
Tacr1	Tacr1	tachykinin receptor 1	21336	https://www.ncbi.nlm.nih.gov/gene/?term=21336
Kmt5a	Kmt5a	lysine methyltransferase 5A	67956	https://www.ncbi.nlm.nih.gov/gene/?term=67956
Lcmt2	Lcmt2	leucine carboxyl methyltransferase 2	329504	https://www.ncbi.nlm.nih.gov/gene/?term=329504
Trp53i11	Trp53i11	transformation related protein 53 inducible protein 11	277414	https://www.ncbi.nlm.nih.gov/gene/?term=277414
9530003J23Rik	9530003J23Rik	RIKEN cDNA 9530003J23 gene	77397	https://www.ncbi.nlm.nih.gov/gene/?term=77397

Biology Open • Supplementary information

Sin3a	Sin3a	transcriptional regulator, SIN3A (yeast)	20466	https://www.ncbi.nlm.nih.gov/gene/?term=20466
Pcdhga6	Pcdhga6	protocadherin gamma subfamily A, 6	93714	https://www.ncbi.nlm.nih.gov/gene/?term=93714
Stat6	Stat6	signal transducer and activator of transcription 6	20852	https://www.ncbi.nlm.nih.gov/gene/?term=20852
Prim2	Prim2	DNA primase, p58 subunit	19076	https://www.ncbi.nlm.nih.gov/gene/?term=19076
Hexim2	Hexim2	hexamethylene bis-acetamide inducible 2	71059	https://www.ncbi.nlm.nih.gov/gene/?term=71059
Stard8	Stard8	START domain containing 8	236920	https://www.ncbi.nlm.nih.gov/gene/?term=236920
Fam107a	Fam107a	family with sequence similarity 107, member A	268709	https://www.ncbi.nlm.nih.gov/gene/?term=268709
Pcdhga9	Pcdhga9	protocadherin gamma subfamily A, 9	93717	https://www.ncbi.nlm.nih.gov/gene/?term=93717
Gabrq	Gabrq	gamma-aminobutyric acid (GABA) A receptor, subunit theta	57249	https://www.ncbi.nlm.nih.gov/gene/?term=57249

Biology Open • Supplementary information

Tcp11l1	Tcp11l1	t-complex 11 like 1	320554	https://www.ncbi.nlm.nih.gov/gene/?term=320554
Prkaa1	Prkaa1	protein kinase, AMP-activated, alpha 1 catalytic subunit	105787	https://www.ncbi.nlm.nih.gov/gene/?term=105787
Pitpnb	Pitpnb	phosphatidylinositol transfer protein, beta	56305	https://www.ncbi.nlm.nih.gov/gene/?term=56305
Slc16a5	Slc16a5	solute carrier family 16 (monocarboxylic acid transporters), member 5	217316	https://www.ncbi.nlm.nih.gov/gene/?term=217316
Ubap2l	Ubap2l	ubiquitin-associated protein 2-like	74383	https://www.ncbi.nlm.nih.gov/gene/?term=74383
Oxtr	Oxtr	oxytocin receptor	18430	https://www.ncbi.nlm.nih.gov/gene/?term=18430
Prps1l1	Prps1l1	phosphoribosyl pyrophosphate synthetase 1-like 1	75456	https://www.ncbi.nlm.nih.gov/gene/?term=75456
Mapk11	Mapk11	mitogen-activated protein kinase 11	19094	https://www.ncbi.nlm.nih.gov/gene/?term=19094
Ildr2	Ildr2	immunoglobulin-like domain containing receptor 2	100039795	https://www.ncbi.nlm.nih.gov/gene/?term=100039

Biology Open • Supplementary information

Uhrf1bp1l	Uhrf1bp1l	UHRF1 (ICBP90) binding protein 1-like	75089	https://www.ncbi.nlm.nih.gov/gene/?term=75089
Nkap	Nkap	NFKB activating protein	67050	https://www.ncbi.nlm.nih.gov/gene/?term=67050
Arhgap25	Arhgap25	Rho GTPase activating protein 25	232201	https://www.ncbi.nlm.nih.gov/gene/?term=232201
Kcnv2	Kcnv2	potassium channel, subfamily V, member 2	240595	https://www.ncbi.nlm.nih.gov/gene/?term=240595
Pck2	Pck2	phosphoenolpyruvate carboxykinase 2 (mitochondrial)	74551	https://www.ncbi.nlm.nih.gov/gene/?term=74551
Htt	Htt	huntingtin	15194	https://www.ncbi.nlm.nih.gov/gene/?term=15194
Coq5	Coq5	coenzyme Q5 methyltransferase	52064	https://www.ncbi.nlm.nih.gov/gene/?term=52064
Pfkp	Pfkp	phosphofructokinase, platelet	56421	https://www.ncbi.nlm.nih.gov/gene/?term=56421
Itgam	Itgam	integrin alpha M	16409	https://www.ncbi.nlm.nih.gov/gene/?term=16409

Grasp	Grasp	GRP1 (general receptor for phosphoinositides 1)-associated scaffold protein	56149	https://www.ncbi.nlm.nih.gov/gene/?term=56149
Gnaz	Gnaz	guanine nucleotide binding protein, alpha z subunit	14687	https://www.ncbi.nlm.nih.gov/gene/?term=14687
Ralb	Ralb	v-ral simian leukemia viral oncogene B	64143	https://www.ncbi.nlm.nih.gov/gene/?term=64143
Gm12886	Gm12886	predicted gene 12886	666921	https://www.ncbi.nlm.nih.gov/gene/?term=666921
Wscd1	Wscd1	WSC domain containing 1	216881	https://www.ncbi.nlm.nih.gov/gene/?term=216881
Hnrnpa0	Hnrnpa0	heterogeneous nuclear ribonucleoprotein A0	77134	https://www.ncbi.nlm.nih.gov/gene/?term=77134
Vtcn1	Vtcn1	V-set domain containing T cell activation inhibitor 1	242122	https://www.ncbi.nlm.nih.gov/gene/?term=242122
Mta3	Mta3	metastasis associated 3	116871	https://www.ncbi.nlm.nih.gov/gene/?term=116871
Grid1	Grid1	glutamate receptor, ionotropic, delta 1	14803	https://www.ncbi.nlm.nih.gov/gene/?term=14803

Biology Open • Supplementary information

Gadl1	Gadl1	glutamate decarboxylase-like 1	73748	https://www.ncbi.nlm.nih.gov/gene/?term=73748
Bst1	Bst1	bone marrow stromal cell antigen 1	12182	https://www.ncbi.nlm.nih.gov/gene/?term=12182
Sfrp1	Sfrp1	secreted frizzled-related protein 1	20377	https://www.ncbi.nlm.nih.gov/gene/?term=20377
Zmat4	Zmat4	zinc finger, matrin type 4	320158	https://www.ncbi.nlm.nih.gov/gene/?term=320158
Vps37a	Vps37a	vacuolar protein sorting 37A	52348	https://www.ncbi.nlm.nih.gov/gene/?term=52348
Dctd	Dctd	dCMP deaminase	320685	https://www.ncbi.nlm.nih.gov/gene/?term=320685
Mrvi1	Mrvi1	MRV integration site 1	17540	https://www.ncbi.nlm.nih.gov/gene/?term=17540
Mon1b	Mon1b	MON1 homolog B, secretory traffciking associated	270096	https://www.ncbi.nlm.nih.gov/gene/?term=270096
Deup1	Deup1	deuterosome assembly protein 1	234964	https://www.ncbi.nlm.nih.gov/gene/?term=234964

Biology Open • Supplementary information

Cd27	Cd27	CD27 antigen	21940	https://www.ncbi.nlm.nih.gov/gene/?term=21940
Aldh5a1	Aldh5a1	aldhehyde dehydrogenase family 5, subfamily A1	214579	https://www.ncbi.nlm.nih.gov/gene/?term=214579
Anks1b	Anks1b	ankyrin repeat and sterile alpha motif domain containing 1B	77531	https://www.ncbi.nlm.nih.gov/gene/?term=77531
Mapkbp1	Mapkbp1	mitogen-activated protein kinase binding protein 1	26390	https://www.ncbi.nlm.nih.gov/gene/?term=26390
Atrn	Atrn	attractin	11990	https://www.ncbi.nlm.nih.gov/gene/?term=11990
Pcdhgb1	Pcdhgb1	protocadherin gamma subfamily B, 1	93699	https://www.ncbi.nlm.nih.gov/gene/?term=93699
Insr	Insr	insulin receptor	16337	https://www.ncbi.nlm.nih.gov/gene/?term=16337
Clvs1	Clvs1	clavesin 1	74438	https://www.ncbi.nlm.nih.gov/gene/?term=74438
Acot3	Acot3	acyl-CoA thioesterase 3	171281	https://www.ncbi.nlm.nih.gov/gene/?term=171281

Biology Open • Supplementary information

Rfx7	Rfx7	regulatory factor X, 7	319758	https://www.ncbi.nlm.nih.gov/gene/?term=319758
D630003M21Rik	D630003M21Rik	RIKEN cDNA D630003M21 gene	228846	https://www.ncbi.nlm.nih.gov/gene/?term=228846
Elovl6	Elovl6	ELOVL family member 6, elongation of long chain fatty acids (yeast)	170439	https://www.ncbi.nlm.nih.gov/gene/?term=170439
Sptlc2	Sptlc2	serine palmitoyltransferase, long chain base subunit 2	20773	https://www.ncbi.nlm.nih.gov/gene/?term=20773
Nos1	Nos1	nitric oxide synthase 1, neuronal	18125	https://www.ncbi.nlm.nih.gov/gene/?term=18125
Gm14137	Gm14137	predicted gene 14137	623781	https://www.ncbi.nlm.nih.gov/gene/?term=623781
3425401B19Rik	3425401B19Rik	RIKEN cDNA 3425401B19 gene	100504518	https://www.ncbi.nlm.nih.gov/gene/?term=100504
Depdc5	Depdc5	DEP domain containing 5	277854	https://www.ncbi.nlm.nih.gov/gene/?term=277854
Trmt2b	Trmt2b	TRM2 tRNA methyltransferase 2B	215201	https://www.ncbi.nlm.nih.gov/gene/?term=215201

Mymk	Mymk	myomaker, myoblast fusion factor	66139	https://www.ncbi.nlm.nih.gov/gene/?term=66139
Iars2	Iars2	isoleucine-tRNA synthetase 2, mitochondrial	381314	https://www.ncbi.nlm.nih.gov/gene/?term=381314
Gm38666	Gm38666	predicted gene, 38666	103611158	https://www.ncbi.nlm.nih.gov/gene/?term=103611
Urod	Urod	uroporphyrinogen decarboxylase	22275	https://www.ncbi.nlm.nih.gov/gene/?term=22275
Micall1	Micall1	microtubule associated monooxygenase, calponin and LIM domain containing -like 1	27008	https://www.ncbi.nlm.nih.gov/gene/?term=27008
Cdc42ep4	Cdc42ep4	CDC42 effector protein (Rho GTPase binding) 4	56699	https://www.ncbi.nlm.nih.gov/gene/?term=56699
Gpr68	Gpr68	G protein-coupled receptor 68	238377	https://www.ncbi.nlm.nih.gov/gene/?term=238377
Napepld	Napepld	N-acyl phosphatidylethanolamine phospholipase D	242864	https://www.ncbi.nlm.nih.gov/gene/?term=242864
Pcdhgc3	Pcdhgc3	protocadherin gamma subfamily C, 3	93706	https://www.ncbi.nlm.nih.gov/gene/?term=93706

Wdr78	Wdr78	WD repeat domain 78	242584	https://www.ncbi.nlm.nih.gov/gene/?term=242584
Plpbp	Plpbp	pyridoxal phosphate binding protein	114863	https://www.ncbi.nlm.nih.gov/gene/?term=114863
Elovl5	Elovl5	ELOVL family member 5, elongation of long chain fatty acids (yeast)	68801	https://www.ncbi.nlm.nih.gov/gene/?term=68801
Nav1	Nav1	neuron navigator 1	215690	https://www.ncbi.nlm.nih.gov/gene/?term=215690
Proser3	Proser3	proline and serine rich 3	333193	https://www.ncbi.nlm.nih.gov/gene/?term=333193
Mafb	Mafb	v-maf musculoaponeurotic fibrosarcoma oncogene family, protein B (avian)	16658	https://www.ncbi.nlm.nih.gov/gene/?term=16658
Prokr2	Prokr2	prokineticin receptor 2	246313	https://www.ncbi.nlm.nih.gov/gene/?term=246313
Hoxb4	Hoxb4	homeobox B4	15412	https://www.ncbi.nlm.nih.gov/gene/?term=15412
Avl9	Avl9	AVL9 cell migration associated	78937	https://www.ncbi.nlm.nih.gov/gene/?term=78937

Synj2bp	Synj2bp	synaptojanin 2 binding protein	24071	https://www.ncbi.nlm.nih.gov/gene/?term=24071
Clec2l	Clec2l	C-type lectin domain family 2, member L	665180	https://www.ncbi.nlm.nih.gov/gene/?term=665180
Dppa1	Dppa1	developmental pluripotency associated 1	347708	https://www.ncbi.nlm.nih.gov/gene/?term=347708
Tfap2b	Tfap2b	transcription factor AP-2 beta	21419	https://www.ncbi.nlm.nih.gov/gene/?term=21419
Fermt1	Fermt1	fermitin family member 1	241639	https://www.ncbi.nlm.nih.gov/gene/?term=241639
Garem2	Garem2	GRB2 associated regulator of MAPK1 subtype 2	242915	https://www.ncbi.nlm.nih.gov/gene/?term=242915
Efna5	Efna5	ephrin A5	13640	https://www.ncbi.nlm.nih.gov/gene/?term=13640
Slc8a3	Slc8a3	solute carrier family 8 (sodium/calcium exchanger), member 3	110893	https://www.ncbi.nlm.nih.gov/gene/?term=110893
Vipr2	Vipr2	vasoactive intestinal peptide receptor 2	22355	https://www.ncbi.nlm.nih.gov/gene/?term=22355

Biology Open • Supplementary information

Klhl9	Klhl9	kelch-like 9	242521	https://www.ncbi.nlm.nih.gov/gene/?term=242521
Creg2	Creg2	cellular repressor of E1A-stimulated genes 2	263764	https://www.ncbi.nlm.nih.gov/gene/?term=263764
Lgals12	Lgals12	lectin, galactose binding, soluble 12	56072	https://www.ncbi.nlm.nih.gov/gene/?term=56072
Gpr173	Gpr173	G-protein coupled receptor 173	70771	https://www.ncbi.nlm.nih.gov/gene/?term=70771
Pcdhgb2	Pcdhgb2	protocadherin gamma subfamily B, 2	93700	https://www.ncbi.nlm.nih.gov/gene/?term=93700
Ceacam18	Ceacam18	carcinoembryonic antigen-related cell adhesion molecule 18	72431	https://www.ncbi.nlm.nih.gov/gene/?term=72431
Srpx2	Srpx2	sushi-repeat-containing protein, X-linked 2	68792	https://www.ncbi.nlm.nih.gov/gene/?term=68792
Il18r1	Il18r1	interleukin 18 receptor 1	16182	https://www.ncbi.nlm.nih.gov/gene/?term=16182
Cyb5b	Cyb5b	cytochrome b5 type B	66427	https://www.ncbi.nlm.nih.gov/gene/?term=66427

Biology Open • Supplementary information

Cend1	Cend1	cell cycle exit and neuronal differentiation 1	57754	https://www.ncbi.nlm.nih.gov/gene/?term=57754
Rpp25	Rpp25	ribonuclease P/MRP 25 subunit	102614	https://www.ncbi.nlm.nih.gov/gene/?term=102614
Srgap3	Srgap3	SLIT-ROBO Rho GTPase activating protein 3	259302	https://www.ncbi.nlm.nih.gov/gene/?term=259302
Sh3rf3	Sh3rf3	SH3 domain containing ring finger 3	237353	https://www.ncbi.nlm.nih.gov/gene/?term=237353
Tbc1d30	Tbc1d30	TBC1 domain family, member 30	74694	https://www.ncbi.nlm.nih.gov/gene/?term=74694
Esr2	Esr2	estrogen receptor 2 (beta)	13983	https://www.ncbi.nlm.nih.gov/gene/?term=13983
Lnpk	Lnpk	lunapark, ER junction formation factor	69605	https://www.ncbi.nlm.nih.gov/gene/?term=69605
Gas2l3	Gas2l3	growth arrest-specific 2 like 3	237436	https://www.ncbi.nlm.nih.gov/gene/?term=237436
Vamp5	Vamp5	vesicle-associated membrane protein 5	53620	https://www.ncbi.nlm.nih.gov/gene/?term=53620

Biology Open • Supplementary information

P2ry13	P2ry13	purinergic receptor P2Y, G-protein coupled 13	74191	https://www.ncbi.nlm.nih.gov/gene/?term=74191
Gtf2h2	Gtf2h2	general transcription factor II H, polypeptide 2	23894	https://www.ncbi.nlm.nih.gov/gene/?term=23894
Rasal2	Rasal2	RAS protein activator like 2	226525	https://www.ncbi.nlm.nih.gov/gene/?term=226525
Gm37013	Gm37013	predicted gene, 37013	100384868	https://www.ncbi.nlm.nih.gov/gene/?term=100384
Edaradd	Edaradd	EDAR (ectodysplasin-A receptor)-associated death domain	171211	https://www.ncbi.nlm.nih.gov/gene/?term=171211
F10	F10	coagulation factor X	14058	https://www.ncbi.nlm.nih.gov/gene/?term=14058
Nlgn3	Nlgn3	neuroligin 3	245537	https://www.ncbi.nlm.nih.gov/gene/?term=245537
Syt15	Syt15	synaptotagmin XV	319508	https://www.ncbi.nlm.nih.gov/gene/?term=319508
Creb3l1	Creb3l1	cAMP responsive element binding protein 3-like 1	26427	https://www.ncbi.nlm.nih.gov/gene/?term=26427

Biology Open • Supplementary information

Fam149b	Fam149b	family with sequence similarity 149, member B	105428	https://www.ncbi.nlm.nih.gov/gene/?term=105428
Srsf12	Srsf12	serine/arginine-rich splicing factor 12	272009	https://www.ncbi.nlm.nih.gov/gene/?term=272009
Nphp3	Nphp3	nephronophthisis 3 (adolescent)	74025	https://www.ncbi.nlm.nih.gov/gene/?term=74025
Ttc12	Ttc12	tetratricopeptide repeat domain 12	235330	https://www.ncbi.nlm.nih.gov/gene/?term=235330
AI429214	AI429214	expressed sequence AI429214	621080	https://www.ncbi.nlm.nih.gov/gene/?term=621080
Vangl2	Vangl2	VANGL planar cell polarity 2	93840	https://www.ncbi.nlm.nih.gov/gene/?term=93840
Slc35e2	Slc35e2	solute carrier family 35, member E2	320541	https://www.ncbi.nlm.nih.gov/gene/?term=320541
Syn3	Syn3	synapsin III	27204	https://www.ncbi.nlm.nih.gov/gene/?term=27204

Biology Open • Supplementary information

Sema5b	Sema5b	sema domain, seven thrombospondin repeats (type 1 and type 1-like), transmembrane domain (TM) and short cytoplasmic domain, (semaphorin) 5B	20357	https://www.ncbi.nlm.nih.gov/gene/?term=20357
B4galnt2	B4galnt2	beta-1,4-N-acetyl-galactosaminyl transferase 2	14422	https://www.ncbi.nlm.nih.gov/gene/?term=14422
Adgrf5	Adgrf5	adhesion G protein-coupled receptor F5	224792	https://www.ncbi.nlm.nih.gov/gene/?term=224792
Mettl27	Mettl27	methyltransferase like 27	79565	https://www.ncbi.nlm.nih.gov/gene/?term=79565
Man1c1	Man1c1	mannosidase, alpha, class 1C, member 1	230815	https://www.ncbi.nlm.nih.gov/gene/?term=230815
Ttll1	Ttll1	tubulin tyrosine ligase-like 1	319953	https://www.ncbi.nlm.nih.gov/gene/?term=319953
Atp2b3	Atp2b3	ATPase, Ca++ transporting, plasma membrane 3	320707	https://www.ncbi.nlm.nih.gov/gene/?term=320707
Pgbd1	Pgbd1	piggyBac transposable element derived 1	319207	https://www.ncbi.nlm.nih.gov/gene/?term=319207

Biology Open • Supplementary information

Pld5	Pld5	phospholipase D family, member 5	319455	https://www.ncbi.nlm.nih.gov/gene/?term=319455
Dnase1l3	Dnase1l3	deoxyribonuclease 1-like 3	13421	https://www.ncbi.nlm.nih.gov/gene/?term=13421
Hspg2	Hspg2	perlecan (heparan sulfate proteoglycan 2)	15530	https://www.ncbi.nlm.nih.gov/gene/?term=15530
C5ar2	C5ar2	complement component 5a receptor 2	319430	https://www.ncbi.nlm.nih.gov/gene/?term=319430
Il1rap	Il1rap	interleukin 1 receptor accessory protein	16180	https://www.ncbi.nlm.nih.gov/gene/?term=16180
B4galt6	B4galt6	UDP-Gal:betaGlcNAc beta 1,4-galactosyltransferase, polypeptide 6	56386	https://www.ncbi.nlm.nih.gov/gene/?term=56386
Vsnl1	Vsnl1	visinin-like 1	26950	https://www.ncbi.nlm.nih.gov/gene/?term=26950
Gabbr1	Gabbr1	gamma-aminobutyric acid (GABA) B receptor, 1	54393	https://www.ncbi.nlm.nih.gov/gene/?term=54393
Prdm8	Prdm8	PR domain containing 8	77630	https://www.ncbi.nlm.nih.gov/gene/?term=77630

Biology Open • Supplementary information

Fbxl17	Fbxl17	F-box and leucine-rich repeat protein 17	50758	https://www.ncbi.nlm.nih.gov/gene/?term=50758
Ptgdr	Ptgdr	prostaglandin D receptor	19214	https://www.ncbi.nlm.nih.gov/gene/?term=19214
Pou6f1	Pou6f1	POU domain, class 6, transcription factor 1	19009	https://www.ncbi.nlm.nih.gov/gene/?term=19009
Neu1	Neu1	neuraminidase 1	18010	https://www.ncbi.nlm.nih.gov/gene/?term=18010
Naip6	Naip6	NLR family, apoptosis inhibitory protein 6	17952	https://www.ncbi.nlm.nih.gov/gene/?term=17952
Stxbp4	Stxbp4	syntaxin binding protein 4	20913	https://www.ncbi.nlm.nih.gov/gene/?term=20913
Trub2	Trub2	TruB pseudouridine (psi) synthase family member 2	227682	https://www.ncbi.nlm.nih.gov/gene/?term=227682
D630039A03Rik	D630039A03Rik	RIKEN cDNA D630039A03 gene	242484	https://www.ncbi.nlm.nih.gov/gene/?term=242484
Rsc1a1	Rsc1a1	regulatory solute carrier protein, family 1, member 1	69994	https://www.ncbi.nlm.nih.gov/gene/?term=69994

Biology Open • Supplementary information

Tmco1	Tmco1	transmembrane and coiled-coil domains 1	68944	https://www.ncbi.nlm.nih.gov/gene/?term=68944
Actr10	Actr10	ARP10 actin-related protein 10	56444	https://www.ncbi.nlm.nih.gov/gene/?term=56444
Pcdhgc4	Pcdhgc4	protocadherin gamma subfamily C, 4	93707	https://www.ncbi.nlm.nih.gov/gene/?term=93707
Snai2	Snai2	snail family zinc finger 2	20583	https://www.ncbi.nlm.nih.gov/gene/?term=20583
Zfp169	Zfp169	zinc finger protein 169	67911	https://www.ncbi.nlm.nih.gov/gene/?term=67911
Epas1	Epas1	endothelial PAS domain protein 1	13819	https://www.ncbi.nlm.nih.gov/gene/?term=13819
Map3k20	Map3k20	mitogen-activated protein kinase kinase kinase 20	65964	https://www.ncbi.nlm.nih.gov/gene/?term=65964
Trpc5	Trpc5	transient receptor potential cation channel, subfamily C, member 5	22067	https://www.ncbi.nlm.nih.gov/gene/?term=22067
Supt7l	Supt7l	suppressor of Ty 7-like	72195	https://www.ncbi.nlm.nih.gov/gene/?term=72195

Biology Open • Supplementary information

Zfp318	Zfp318	zinc finger protein 318	57908	https://www.ncbi.nlm.nih.gov/gene/?term=57908
Htra4	Htra4	HtrA serine peptidase 4	330723	https://www.ncbi.nlm.nih.gov/gene/?term=330723
Lyrm9	Lyrm9	LYR motif containing 9	66274	https://www.ncbi.nlm.nih.gov/gene/?term=66274
Iglon5	Iglon5	IgLON family member 5	210094	https://www.ncbi.nlm.nih.gov/gene/?term=210094
Kcna2	Kcna2	potassium voltage-gated channel, shaker-related subfamily, member 2	16490	https://www.ncbi.nlm.nih.gov/gene/?term=16490
Tvp23a	Tvp23a	trans-golgi network vesicle protein 23A	383103	https://www.ncbi.nlm.nih.gov/gene/?term=383103
Osr1	Osr1	odd-skipped related transcription factor 1	23967	https://www.ncbi.nlm.nih.gov/gene/?term=23967
Pcdhga7	Pcdhga7	protocadherin gamma subfamily A, 7	93715	https://www.ncbi.nlm.nih.gov/gene/?term=93715
Chst2	Chst2	carbohydrate sulfotransferase 2	54371	https://www.ncbi.nlm.nih.gov/gene/?term=54371

Biology Open • Supplementary information

Brix1	Brix1	BRX1, biogenesis of ribosomes	67832	https://www.ncbi.nlm.nih.gov/gene/?term=67832
Zc3h12d	Zc3h12d	zinc finger CCCH type containing 12D	237256	https://www.ncbi.nlm.nih.gov/gene/?term=237256
Angpt2	Angpt2	angiopoietin 2	11601	https://www.ncbi.nlm.nih.gov/gene/?term=11601
Ascl1	Ascl1	achaete-scute family bHLH transcription factor 1	17172	https://www.ncbi.nlm.nih.gov/gene/?term=17172
Cap2	Cap2	CAP, adenylate cyclase-associated protein, 2 (yeast)	67252	https://www.ncbi.nlm.nih.gov/gene/?term=67252
Katnal1	Katnal1	katanin p60 subunit A-like 1	231912	https://www.ncbi.nlm.nih.gov/gene/?term=231912
Plppr3	Plppr3	phospholipid phosphatase related 3	216152	https://www.ncbi.nlm.nih.gov/gene/?term=216152
Nxpe3	Nxpe3	neurexophilin and PC-esterase domain family, member 3	385658	https://www.ncbi.nlm.nih.gov/gene/?term=385658
Mrgpre	Mrgpre	MAS-related GPR, member E	244238	https://www.ncbi.nlm.nih.gov/gene/?term=244238

Gm14308	Gm14308	predicted gene 14308	100043381	https://www.ncbi.nlm.nih.gov/gene/?term=100043
Pcdhga4	Pcdhga4	protocadherin gamma subfamily A, 4	93712	https://www.ncbi.nlm.nih.gov/gene/?term=93712
Peg3	Peg3	paternally expressed 3	18616	https://www.ncbi.nlm.nih.gov/gene/?term=18616
Calcoco1	Calcoco1	calcium binding and coiled coil domain 1	67488	https://www.ncbi.nlm.nih.gov/gene/?term=67488
9130023H24Rik	9130023H24Rik	RIKEN cDNA 9130023H24 gene	100043133	https://www.ncbi.nlm.nih.gov/gene/?term=100043
Ggt5	Ggt5	gamma-glutamyltransferase 5	23887	https://www.ncbi.nlm.nih.gov/gene/?term=23887
Dtx3l	Dtx3l	deltex 3-like, E3 ubiquitin ligase	209200	https://www.ncbi.nlm.nih.gov/gene/?term=209200
Metrnl	Metrnl	meteorin, glial cell differentiation regulator-like	210029	https://www.ncbi.nlm.nih.gov/gene/?term=210029
Rab2b	Rab2b	RAB2B, member RAS oncogene family	76338	https://www.ncbi.nlm.nih.gov/gene/?term=76338

Biology Open • Supplementary information

Mrpl19	Mrpl19	mitochondrial ribosomal protein L19	56284	https://www.ncbi.nlm.nih.gov/gene/?term=56284
Kcnmb1	Kcnmb1	potassium large conductance calcium-activated channel, subfamily M, beta member 1	16533	https://www.ncbi.nlm.nih.gov/gene/?term=16533
Ajap1	Ajap1	adherens junction associated protein 1	230959	https://www.ncbi.nlm.nih.gov/gene/?term=230959
Mocs1	Mocs1	molybdenum cofactor synthesis 1	56738	https://www.ncbi.nlm.nih.gov/gene/?term=56738
Nkain2	Nkain2	Na+/K+ transporting ATPase interacting 2	432450	https://www.ncbi.nlm.nih.gov/gene/?term=432450
Extl3	Extl3	exostoses (multiple)-like 3	54616	https://www.ncbi.nlm.nih.gov/gene/?term=54616
Tnfrsf11a	Tnfrsf11a	tumor necrosis factor receptor superfamily, member 11a, NFKB activator	21934	https://www.ncbi.nlm.nih.gov/gene/?term=21934
Prss23	Prss23	protease, serine 23	76453	https://www.ncbi.nlm.nih.gov/gene/?term=76453
Zfand5	Zfand5	zinc finger, AN1-type domain 5	22682	https://www.ncbi.nlm.nih.gov/gene/?term=22682

Biology Open • Supplementary information

Ehd3	Ehd3	EH-domain containing 3	57440	https://www.ncbi.nlm.nih.gov/gene/?term=57440
Shisal1	Shisal1	shisa like 1	72301	https://www.ncbi.nlm.nih.gov/gene/?term=72301
Man2a2	Man2a2	mannosidase 2, alpha 2	140481	https://www.ncbi.nlm.nih.gov/gene/?term=140481
Klhl23	Klhl23	kelch-like 23	277396	https://www.ncbi.nlm.nih.gov/gene/?term=277396
Col13a1	Col13a1	collagen, type XIII, alpha 1	12817	https://www.ncbi.nlm.nih.gov/gene/?term=12817
Knstrn	Knstrn	kinetochore-localized astrin/SPAG5 binding	51944	https://www.ncbi.nlm.nih.gov/gene/?term=51944
Rab9	Rab9	RAB9, member RAS oncogene family	56382	https://www.ncbi.nlm.nih.gov/gene/?term=56382
Gm14151	Gm14151	predicted gene 14151	433486	https://www.ncbi.nlm.nih.gov/gene/?term=433486
Doc2b	Doc2b	double C2, beta	13447	https://www.ncbi.nlm.nih.gov/gene/?term=13447

Biology Open • Supplementary information

Gabrb2	Gabrb2	gamma-aminobutyric acid (GABA) A receptor, subunit beta 2	14401	https://www.ncbi.nlm.nih.gov/gene/?term=14401
B3galt6	B3galt6	UDP-Gal:betaGal beta 1,3-galactosyltransferase, polypeptide 6	117592	https://www.ncbi.nlm.nih.gov/gene/?term=117592
Zfp663	Zfp663	zinc finger protein 663	381405	https://www.ncbi.nlm.nih.gov/gene/?term=381405
Atg10	Atg10	autophagy related 10	66795	https://www.ncbi.nlm.nih.gov/gene/?term=66795
Terf2ip	Terf2ip	telomeric repeat binding factor 2, interacting protein	57321	https://www.ncbi.nlm.nih.gov/gene/?term=57321
Sp9	Sp9	trans-acting transcription factor 9	381373	https://www.ncbi.nlm.nih.gov/gene/?term=381373
Csmd1	Csmd1	CUB and Sushi multiple domains 1	94109	https://www.ncbi.nlm.nih.gov/gene/?term=94109
Rgs9bp	Rgs9bp	regulator of G-protein signalling 9 binding protein	243923	https://www.ncbi.nlm.nih.gov/gene/?term=243923
Jmjd8	Jmjd8	jumonji domain containing 8	72106	https://www.ncbi.nlm.nih.gov/gene/?term=72106

Biology Open • Supplementary information

Sparc	Sparc	secreted acidic cysteine rich glycoprotein	20692	https://www.ncbi.nlm.nih.gov/gene/?term=20692
Samd7	Samd7	sterile alpha motif domain containing 7	75953	https://www.ncbi.nlm.nih.gov/gene/?term=75953
Ankrd34a	Ankrd34a	ankyrin repeat domain 34A	545554	https://www.ncbi.nlm.nih.gov/gene/?term=545554
Wnt4	Wnt4	wingless-type MMTV integration site family, member 4	22417	https://www.ncbi.nlm.nih.gov/gene/?term=22417
D630045J12Rik	D630045J12Rik	RIKEN cDNA D630045J12 gene	330286	https://www.ncbi.nlm.nih.gov/gene/?term=330286
Gm14391	Gm14391	predicted gene 14391	665001	https://www.ncbi.nlm.nih.gov/gene/?term=665001
Zfp712	Zfp712	zinc finger protein 712	78251	https://www.ncbi.nlm.nih.gov/gene/?term=78251
Gm38667	Gm38667	predicted gene, 38667	103611159	https://www.ncbi.nlm.nih.gov/gene/?term=103611
Scd3	Scd3	stearoyl-coenzyme A desaturase 3	30049	https://www.ncbi.nlm.nih.gov/gene/?term=30049

Biology Open • Supplementary information

Tub	Tub	tubby bipartite transcription factor	22141	https://www.ncbi.nlm.nih.gov/gene/?term=22141
Plekhg5	Plekhg5	pleckstrin homology domain containing, family G (with RhoGef domain) member 5	269608	https://www.ncbi.nlm.nih.gov/gene/?term=269608
Pcdhgb4	Pcdhgb4	protocadherin gamma subfamily B, 4	93701	https://www.ncbi.nlm.nih.gov/gene/?term=93701
Zfp74	Zfp74	zinc finger protein 74	72723	https://www.ncbi.nlm.nih.gov/gene/?term=72723
Slco2a1	Slco2a1	solute carrier organic anion transporter family, member 2a1	24059	https://www.ncbi.nlm.nih.gov/gene/?term=24059
Vwa5b2	Vwa5b2	von Willebrand factor A domain containing 5B2	328643	https://www.ncbi.nlm.nih.gov/gene/?term=328643
Trip12	Trip12	thyroid hormone receptor interactor 12	14897	https://www.ncbi.nlm.nih.gov/gene/?term=14897
Bmp3	Bmp3	bone morphogenetic protein 3	110075	https://www.ncbi.nlm.nih.gov/gene/?term=110075
Cmc2	Cmc2	COX assembly mitochondrial protein 2	66531	https://www.ncbi.nlm.nih.gov/gene/?term=66531

Biology Open • Supplementary information

Dcaf1	Dcaf1	DDB1 and CUL4 associated factor 1	321006	https://www.ncbi.nlm.nih.gov/gene/?term=321006
Gcm2	Gcm2	glial cells missing homolog 2	107889	https://www.ncbi.nlm.nih.gov/gene/?term=107889
Csrnp2	Csrnp2	cysteine-serine-rich nuclear protein 2	207785	https://www.ncbi.nlm.nih.gov/gene/?term=207785
Mfsd6	Mfsd6	major facilitator superfamily domain containing 6	98682	https://www.ncbi.nlm.nih.gov/gene/?term=98682
Slc25a21	Slc25a21	solute carrier family 25 (mitochondrial oxodicarboxylate carrier), member 21	217593	https://www.ncbi.nlm.nih.gov/gene/?term=217593
Plac9b	Plac9b	placenta specific 9b	100039246	https://www.ncbi.nlm.nih.gov/gene/?term=100039
Gm4925	Gm4925	predicted gene 4925	237433	https://www.ncbi.nlm.nih.gov/gene/?term=237433
Smyd3	Smyd3	SET and MYND domain containing 3	69726	https://www.ncbi.nlm.nih.gov/gene/?term=69726
Btbd8	Btbd8	BTB (POZ) domain containing 8	100503185	https://www.ncbi.nlm.nih.gov/gene/?term=100503

Biology Open • Supplementary information

Dcaf17	Dcaf17	DDB1 and CUL4 associated factor 17	75763	https://www.ncbi.nlm.nih.gov/gene/?term=75763
Pcdhga5	Pcdhga5	protocadherin gamma subfamily A, 5	93713	https://www.ncbi.nlm.nih.gov/gene/?term=93713
Bin2	Bin2	bridging integrator 2	668218	https://www.ncbi.nlm.nih.gov/gene/?term=668218
Foxa1	Foxa1	forkhead box A1	15375	https://www.ncbi.nlm.nih.gov/gene/?term=15375
Nsun3	Nsun3	NOL1/NOP2/Sun domain family member 3	106338	https://www.ncbi.nlm.nih.gov/gene/?term=106338
Scn2a	Scn2a	sodium channel, voltage-gated, type II, alpha	110876	https://www.ncbi.nlm.nih.gov/gene/?term=110876
Slc6a6	Slc6a6	solute carrier family 6 (neurotransmitter transporter, taurine), member 6	21366	https://www.ncbi.nlm.nih.gov/gene/?term=21366
Zfp92	Zfp92	zinc finger protein 92	22754	https://www.ncbi.nlm.nih.gov/gene/?term=22754
Megf8	Megf8	multiple EGF-like-domains 8	269878	https://www.ncbi.nlm.nih.gov/gene/?term=269878

Biology Open • Supplementary information

Stk10	Stk10	serine/threonine kinase 10	20868	https://www.ncbi.nlm.nih.gov/gene/?term=20868
Sox7	Sox7	SRY (sex determining region Y)-box 7	20680	https://www.ncbi.nlm.nih.gov/gene/?term=20680
Usp25	Usp25	ubiquitin specific peptidase 25	30940	https://www.ncbi.nlm.nih.gov/gene/?term=30940
Tmem151b	Tmem151b	transmembrane protein 151B	210573	https://www.ncbi.nlm.nih.gov/gene/?term=210573
Sox12	Sox12	SRY (sex determining region Y)-box 12	20667	https://www.ncbi.nlm.nih.gov/gene/?term=20667
Zfp747	Zfp747	zinc finger protein 747	269997	https://www.ncbi.nlm.nih.gov/gene/?term=269997
Pcdhgb5	Pcdhgb5	protocadherin gamma subfamily B, 5	93702	https://www.ncbi.nlm.nih.gov/gene/?term=93702
Prpf4	Prpf4	pre-mRNA processing factor 4	70052	https://www.ncbi.nlm.nih.gov/gene/?term=70052
Gcnt4	Gcnt4	glucosaminyl (N-acetyl) transferase 4, core 2 (beta-1,6-N-acetylglucosaminyltransferase)	218476	https://www.ncbi.nlm.nih.gov/gene/?term=218476

Biology Open • Supplementary information

Tlx1	Tlx1	T cell leukemia, homeobox 1	21908	https://www.ncbi.nlm.nih.gov/gene/?term=21908
Hivep3	Hivep3	human immunodeficiency virus type I enhancer binding protein 3	16656	https://www.ncbi.nlm.nih.gov/gene/?term=16656
Tubb4a	Tubb4a	tubulin, beta 4A class IVA	22153	https://www.ncbi.nlm.nih.gov/gene/?term=22153
Micu2	Micu2	mitochondrial calcium uptake 2	68514	https://www.ncbi.nlm.nih.gov/gene/?term=68514
Cd274	Cd274	CD274 antigen	60533	https://www.ncbi.nlm.nih.gov/gene/?term=60533
Kcna7	Kcna7	potassium voltage-gated channel, shaker-related subfamily, member 7	16495	https://www.ncbi.nlm.nih.gov/gene/?term=16495
Gjc3	Gjc3	gap junction protein, gamma 3	118446	https://www.ncbi.nlm.nih.gov/gene/?term=118446
Trpc6	Trpc6	transient receptor potential cation channel, subfamily C, member 6	22068	https://www.ncbi.nlm.nih.gov/gene/?term=22068
Gm4724	Gm4724	predicted gene 4724	100043915	https://www.ncbi.nlm.nih.gov/gene/?term=100043

Biology Open • Supplementary information

Trem1	Trem1	triggering receptor expressed on myeloid cells 1	58217	https://www.ncbi.nlm.nih.gov/gene/?term=58217
Tgfbrap1	Tgfbrap1	transforming growth factor, beta receptor associated protein 1	73122	https://www.ncbi.nlm.nih.gov/gene/?term=73122
Tmem104	Tmem104	transmembrane protein 104	320534	https://www.ncbi.nlm.nih.gov/gene/?term=320534
Akap13	Akap13	A kinase (PRKA) anchor protein 13	75547	https://www.ncbi.nlm.nih.gov/gene/?term=75547
Ctsc	Ctsc	cathepsin C	13032	https://www.ncbi.nlm.nih.gov/gene/?term=13032
Prlr	Prlr	prolactin receptor	19116	https://www.ncbi.nlm.nih.gov/gene/?term=19116
Muc4	Muc4	mucin 4	140474	https://www.ncbi.nlm.nih.gov/gene/?term=140474
Spock2	Spock2	sparc/osteonectin, cwcv and kazal-like domains proteoglycan 2	94214	https://www.ncbi.nlm.nih.gov/gene/?term=94214
Cstad	Cstad	CSA-conditional, T cell activation-dependent protein	78617	https://www.ncbi.nlm.nih.gov/gene/?term=78617

Biology Open • Supplementary information

Hmga2	Hmga2	high mobility group AT-hook 2	15364	https://www.ncbi.nlm.nih.gov/gene/?term=15364
1700028K03Rik	1700028K03Rik	RIKEN cDNA 1700028K03 gene	76421	https://www.ncbi.nlm.nih.gov/gene/?term=76421
Dhdh	Dhdh	dihydrodiol dehydrogenase (dimeric)	71755	https://www.ncbi.nlm.nih.gov/gene/?term=71755
Slc5a8	Slc5a8	solute carrier family 5 (iodide transporter), member 8	216225	https://www.ncbi.nlm.nih.gov/gene/?term=216225
Nagpa	Nagpa	N-acetylglucosamine-1-phosphodiester alpha-N-acetylglucosaminidase	27426	https://www.ncbi.nlm.nih.gov/gene/?term=27426
Fbxw28	Fbxw28	F-box and WD-40 domain protein 28	668758	https://www.ncbi.nlm.nih.gov/gene/?term=668758
Rpusd2	Rpusd2	RNA pseudouridylate synthase domain containing 2	271842	https://www.ncbi.nlm.nih.gov/gene/?term=271842
Mlec	Mlec	malectin	109154	https://www.ncbi.nlm.nih.gov/gene/?term=109154
Creg1	Creg1	cellular repressor of E1A-stimulated genes 1	433375	https://www.ncbi.nlm.nih.gov/gene/?term=433375

Biology Open • Supplementary information

Nat8l	Nat8l	N-acetyltransferase 8-like	269642	https://www.ncbi.nlm.nih.gov/gene/?term=269642
Trim56	Trim56	tripartite motif-containing 56	384309	https://www.ncbi.nlm.nih.gov/gene/?term=384309
St8sia3	St8sia3	ST8 alpha-N-acetyl-neuraminide alpha-2,8-sialyltransferase 3	20451	https://www.ncbi.nlm.nih.gov/gene/?term=20451
Ccdc3	Ccdc3	coiled-coil domain containing 3	74186	https://www.ncbi.nlm.nih.gov/gene/?term=74186
Ifi44	Ifi44	interferon-induced protein 44	99899	https://www.ncbi.nlm.nih.gov/gene/?term=99899
Slc7a8	Slc7a8	solute carrier family 7 (cationic amino acid transporter, y+ system), member 8	50934	https://www.ncbi.nlm.nih.gov/gene/?term=50934
Chst11	Chst11	carbohydrate sulfotransferase 11	58250	https://www.ncbi.nlm.nih.gov/gene/?term=58250
Dmrta1	Dmrta1	doublesex and mab-3 related transcription factor like family A1	242523	https://www.ncbi.nlm.nih.gov/gene/?term=242523
Tns4	Tns4	tensin 4	217169	https://www.ncbi.nlm.nih.gov/gene/?term=217169

Biology Open • Supplementary information

Atm	Atm	ataxia telangiectasia mutated	11920	https://www.ncbi.nlm.nih.gov/gene/?term=11920
Nwd1	Nwd1	NACHT and WD repeat domain containing 1	319555	https://www.ncbi.nlm.nih.gov/gene/?term=319555
Oacyl	Oacyl	O-acyltransferase like	319888	https://www.ncbi.nlm.nih.gov/gene/?term=319888
Zhx2	Zhx2	zinc fingers and homeoboxes 2	387609	https://www.ncbi.nlm.nih.gov/gene/?term=387609
Sorbs2	Sorbs2	sorbin and SH3 domain containing 2	234214	https://www.ncbi.nlm.nih.gov/gene/?term=234214
Miga2	Miga2	mitoguardin 2	108958	https://www.ncbi.nlm.nih.gov/gene/?term=108958
Polq	Polq	polymerase (DNA directed), theta	77782	https://www.ncbi.nlm.nih.gov/gene/?term=77782
Zfp11	Zfp11	zinc finger protein 11	22648	https://www.ncbi.nlm.nih.gov/gene/?term=22648
Map3k8	Map3k8	mitogen-activated protein kinase kinase kinase 8	26410	https://www.ncbi.nlm.nih.gov/gene/?term=26410

Biology Open • Supplementary information

Ccdc88a	Ccdc88a	coiled coil domain containing 88A	108686	https://www.ncbi.nlm.nih.gov/gene/?term=108686
Tspyl2	Tspyl2	TSPY-like 2	52808	https://www.ncbi.nlm.nih.gov/gene/?term=52808
Emx2	Emx2	empty spiracles homeobox 2	13797	https://www.ncbi.nlm.nih.gov/gene/?term=13797
Suv39h1	Suv39h1	suppressor of variegation 3-9 1	20937	https://www.ncbi.nlm.nih.gov/gene/?term=20937
Mtbp	Mtbp	Mdm2, transformed 3T3 cell double minute p53 binding protein	105837	https://www.ncbi.nlm.nih.gov/gene/?term=105837
Caprin2	Caprin2	caprin family member 2	232560	https://www.ncbi.nlm.nih.gov/gene/?term=232560
Pdzd7	Pdzd7	PDZ domain containing 7	100503041	https://www.ncbi.nlm.nih.gov/gene/?term=100503
Cldn8	Cldn8	claudin 8	54420	https://www.ncbi.nlm.nih.gov/gene/?term=54420
Ubxn8	Ubxn8	UBX domain protein 8	108159	https://www.ncbi.nlm.nih.gov/gene/?term=108159

Biology Open • Supplementary information

Naa30	Naa30	N(alpha)-acetyltransferase 30, NatC catalytic subunit	70646	https://www.ncbi.nlm.nih.gov/gene/?term=70646
Has3	Has3	hyaluronan synthase 3	15118	https://www.ncbi.nlm.nih.gov/gene/?term=15118
R3hdm1	R3hdm1	R3H domain containing 1	226412	https://www.ncbi.nlm.nih.gov/gene/?term=226412
Kcnd2	Kcnd2	potassium voltage-gated channel, Shal-related family, member 2	16508	https://www.ncbi.nlm.nih.gov/gene/?term=16508
Psd	Psd	pleckstrin and Sec7 domain containing	73728	https://www.ncbi.nlm.nih.gov/gene/?term=73728
Rnf216	Rnf216	ring finger protein 216	108086	https://www.ncbi.nlm.nih.gov/gene/?term=108086
Casd1	Casd1	CAS1 domain containing 1	213819	https://www.ncbi.nlm.nih.gov/gene/?term=213819
Btbd7	Btbd7	BTB (POZ) domain containing 7	238386	https://www.ncbi.nlm.nih.gov/gene/?term=238386
Fastk	Fastk	Fas-activated serine/threonine kinase	66587	https://www.ncbi.nlm.nih.gov/gene/?term=66587

E2f7	E2f7	E2F transcription factor 7	52679	https://www.ncbi.nlm.nih.gov/gene/?term=52679
Rtn1	Rtn1	reticulon 1	104001	https://www.ncbi.nlm.nih.gov/gene/?term=104001
Mylk	Mylk	myosin, light polypeptide kinase	107589	https://www.ncbi.nlm.nih.gov/gene/?term=107589
Abhd3	Abhd3	abhydrolase domain containing 3	106861	https://www.ncbi.nlm.nih.gov/gene/?term=106861
Csnk1g1	Csnk1g1	casein kinase 1, gamma 1	214897	https://www.ncbi.nlm.nih.gov/gene/?term=214897
Wasf1	Wasf1	WAS protein family, member 1	83767	https://www.ncbi.nlm.nih.gov/gene/?term=83767
Jakmip1	Jakmip1	janus kinase and microtubule interacting protein 1	76071	https://www.ncbi.nlm.nih.gov/gene/?term=76071
Fibin	Fibin	fin bud initiation factor homolog (zebrafish)	67606	https://www.ncbi.nlm.nih.gov/gene/?term=67606
Apcdd1	Apcdd1	adenomatosis polyposis coli down-regulated 1	494504	https://www.ncbi.nlm.nih.gov/gene/?term=494504

Biology Open • Supplementary information

Cdk2	Cdk2	cyclin-dependent kinase 2	12566	https://www.ncbi.nlm.nih.gov/gene/?term=12566
Map7	Map7	microtubule-associated protein 7	17761	https://www.ncbi.nlm.nih.gov/gene/?term=17761
Tnks1bp1	Tnks1bp1	tankyrase 1 binding protein 1	228140	https://www.ncbi.nlm.nih.gov/gene/?term=228140
Vwde	Vwde	von Willebrand factor D and EGF domains	232585	https://www.ncbi.nlm.nih.gov/gene/?term=232585
Rab5c	Rab5c	RAB5C, member RAS oncogene family	19345	https://www.ncbi.nlm.nih.gov/gene/?term=19345
Dpysl2	Dpysl2	dihydropyrimidinase-like 2	12934	https://www.ncbi.nlm.nih.gov/gene/?term=12934
Timm9	Timm9	translocase of inner mitochondrial membrane 9	30056	https://www.ncbi.nlm.nih.gov/gene/?term=30056
Mycbp2	Mycbp2	MYC binding protein 2, E3 ubiquitin protein ligase	105689	https://www.ncbi.nlm.nih.gov/gene/?term=105689
Dhx37	Dhx37	DEAH (Asp-Glu-Ala-His) box polypeptide 37	208144	https://www.ncbi.nlm.nih.gov/gene/?term=208144

Biology Open • Supplementary information

Dazap2	Dazap2	DAZ associated protein 2	23994	https://www.ncbi.nlm.nih.gov/gene/?term=23994
Tjap1	Tjap1	tight junction associated protein 1	74094	https://www.ncbi.nlm.nih.gov/gene/?term=74094
Pramel5	Pramel5	preferentially expressed antigen in melanoma like 5	384077	https://www.ncbi.nlm.nih.gov/gene/?term=384077
Lipm	Lipm	lipase, family member M	78753	https://www.ncbi.nlm.nih.gov/gene/?term=78753
Pramel4	Pramel4	preferentially expressed antigen in melanoma like 4	347710	https://www.ncbi.nlm.nih.gov/gene/?term=347710
Sppl3	Sppl3	signal peptide peptidase 3	74585	https://www.ncbi.nlm.nih.gov/gene/?term=74585
Ttc14	Ttc14	tetratricopeptide repeat domain 14	67120	https://www.ncbi.nlm.nih.gov/gene/?term=67120
Fam167a	Fam167a	family with sequence similarity 167, member A	219148	https://www.ncbi.nlm.nih.gov/gene/?term=219148
Shh	Shh	sonic hedgehog	20423	https://www.ncbi.nlm.nih.gov/gene/?term=20423

Biology Open • Supplementary information

Adgre4	Adgre4	adhesion G protein-coupled receptor E4	52614	https://www.ncbi.nlm.nih.gov/gene/?term=52614
Gm13102	Gm13102	predicted gene 13102	100041077	https://www.ncbi.nlm.nih.gov/gene/?term=100041[illegible]
Cfap36	Cfap36	cilia and flagella associated protein 36	216618	https://www.ncbi.nlm.nih.gov/gene/?term=216618
Ercc2	Ercc2	excision repair cross-complementing rodent repair deficiency, complementation group 2	13871	https://www.ncbi.nlm.nih.gov/gene/?term=13871
Slc39a8	Slc39a8	solute carrier family 39 (metal ion transporter), member 8	67547	https://www.ncbi.nlm.nih.gov/gene/?term=67547
Cdh5	Cdh5	cadherin 5	12562	https://www.ncbi.nlm.nih.gov/gene/?term=12562
Mmgt1	Mmgt1	membrane magnesium transporter 1	236792	https://www.ncbi.nlm.nih.gov/gene/?term=236792
Slc16a6	Slc16a6	solute carrier family 16 (monocarboxylic acid transporters), member 6	104681	https://www.ncbi.nlm.nih.gov/gene/?term=104681
Zfp160	Zfp160	zinc finger protein 160	224585	https://www.ncbi.nlm.nih.gov/gene/?term=224585

Biology Open • Supplementary information

Eri1	Eri1	exoribonuclease 1	67276	https://www.ncbi.nlm.nih.gov/gene/?term=67276
Pnoc	Pnoc	prepronociceptin	18155	https://www.ncbi.nlm.nih.gov/gene/?term=18155
Aqp9	Aqp9	aquaporin 9	64008	https://www.ncbi.nlm.nih.gov/gene/?term=64008
Vmn1r32	Vmn1r32	vomeronasal 1 receptor 32	171188	https://www.ncbi.nlm.nih.gov/gene/?term=171188
Asap2	Asap2	ArfGAP with SH3 domain, ankyrin repeat and PH domain 2	211914	https://www.ncbi.nlm.nih.gov/gene/?term=211914
Dll3	Dll3	delta like canonical Notch ligand 3	13389	https://www.ncbi.nlm.nih.gov/gene/?term=13389
Kctd14	Kctd14	potassium channel tetramerisation domain containing 14	233529	https://www.ncbi.nlm.nih.gov/gene/?term=233529
Tmem108	Tmem108	transmembrane protein 108	81907	https://www.ncbi.nlm.nih.gov/gene/?term=81907
Pcna	Pcna	proliferating cell nuclear antigen	18538	https://www.ncbi.nlm.nih.gov/gene/?term=18538

Biology Open • Supplementary information

Plppr5	Plppr5	phospholipid phosphatase related 5	75769	https://www.ncbi.nlm.nih.gov/gene/?term=75769
Brk1	Brk1	BRICK1, SCAR/WAVE actin-nucleating complex subunit	101314	https://www.ncbi.nlm.nih.gov/gene/?term=101314
Fam210b	Fam210b	family with sequence similarity 210, member B	67017	https://www.ncbi.nlm.nih.gov/gene/?term=67017
Prpf8	Prpf8	pre-mRNA processing factor 8	192159	https://www.ncbi.nlm.nih.gov/gene/?term=192159
Ablim1	Ablim1	actin-binding LIM protein 1	226251	https://www.ncbi.nlm.nih.gov/gene/?term=226251
Rtl5	Rtl5	retrotransposon Gag like 5	331474	https://www.ncbi.nlm.nih.gov/gene/?term=331474
Primpol	Primpol	primase and polymerase (DNA-directed)	408022	https://www.ncbi.nlm.nih.gov/gene/?term=408022
Fancl	Fancl	Fanconi anemia, complementation group L	67030	https://www.ncbi.nlm.nih.gov/gene/?term=67030
Hps5	Hps5	HPS5, biogenesis of lysosomal organelles complex 2 subunit 2	246694	https://www.ncbi.nlm.nih.gov/gene/?term=246694

Biology Open • Supplementary information

Prune2	Prune2	prune homolog 2	353211	https://www.ncbi.nlm.nih.gov/gene/?term=353211
Srsf3	Srsf3	serine/arginine-rich splicing factor 3	20383	https://www.ncbi.nlm.nih.gov/gene/?term=20383
Vps37c	Vps37c	vacuolar protein sorting 37C	107305	https://www.ncbi.nlm.nih.gov/gene/?term=107305
Ubox5	Ubox5	U box domain containing 5	140629	https://www.ncbi.nlm.nih.gov/gene/?term=140629
Hif1an	Hif1an	hypoxia-inducible factor 1, alpha subunit inhibitor	319594	https://www.ncbi.nlm.nih.gov/gene/?term=319594
Bmper	Bmper	BMP-binding endothelial regulator	73230	https://www.ncbi.nlm.nih.gov/gene/?term=73230
Rbak	Rbak	RB-associated KRAB zinc finger	57782	https://www.ncbi.nlm.nih.gov/gene/?term=57782
Pik3r2	Pik3r2	phosphoinositide-3-kinase regulatory subunit 2	18709	https://www.ncbi.nlm.nih.gov/gene/?term=18709
Mob1b	Mob1b	MOB kinase activator 1B	68473	https://www.ncbi.nlm.nih.gov/gene/?term=68473

Biology Open • Supplementary information

Slco5a1	Slco5a1	solute carrier organic anion transporter family, member 5A1	240726	https://www.ncbi.nlm.nih.gov/gene/?term=240726
Kcnj6	Kcnj6	potassium inwardly-rectifying channel, subfamily J, member 6	16522	https://www.ncbi.nlm.nih.gov/gene/?term=16522
Dag1	Dag1	dystroglycan 1	13138	https://www.ncbi.nlm.nih.gov/gene/?term=13138
Mtmr2	Mtmr2	myotubularin related protein 2	77116	https://www.ncbi.nlm.nih.gov/gene/?term=77116
Ppp6r3	Ppp6r3	protein phosphatase 6, regulatory subunit 3	52036	https://www.ncbi.nlm.nih.gov/gene/?term=52036
Trp63	Trp63	transformation related protein 63	22061	https://www.ncbi.nlm.nih.gov/gene/?term=22061
Orc5	Orc5	origin recognition complex, subunit 5	26429	https://www.ncbi.nlm.nih.gov/gene/?term=26429
Esrra	Esrra	estrogen related receptor, alpha	26379	https://www.ncbi.nlm.nih.gov/gene/?term=26379
Wscd2	Wscd2	WSC domain containing 2	320916	https://www.ncbi.nlm.nih.gov/gene/?term=320916

Slc9a3r2	Slc9a3r2	solute carrier family 9 (sodium/hydrogen exchanger), member 3 regulator 2	65962	https://www.ncbi.nlm.nih.gov/gene/?term=65962
Shisa7	Shisa7	shisa family member 7	232813	https://www.ncbi.nlm.nih.gov/gene/?term=232813
Phldb2	Phldb2	pleckstrin homology like domain, family B, member 2	208177	https://www.ncbi.nlm.nih.gov/gene/?term=208177
4930562C15Rik	4930562C15Rik	RIKEN cDNA 4930562C15 gene	78809	https://www.ncbi.nlm.nih.gov/gene/?term=78809
Col5a1	Col5a1	collagen, type V, alpha 1	12831	https://www.ncbi.nlm.nih.gov/gene/?term=12831
Zranb2	Zranb2	zinc finger, RAN-binding domain containing 2	53861	https://www.ncbi.nlm.nih.gov/gene/?term=53861
Vamp2	Vamp2	vesicle-associated membrane protein 2	22318	https://www.ncbi.nlm.nih.gov/gene/?term=22318
Runx2	Runx2	runt related transcription factor 2	12393	https://www.ncbi.nlm.nih.gov/gene/?term=12393
Ebf1	Ebf1	early B cell factor 1	13591	https://www.ncbi.nlm.nih.gov/gene/?term=13591

Biology Open • Supplementary information

Slc25a5	Slc25a5	NA	11740	https://www.ncbi.nlm.nih.gov/gene/?term=11740
Cplx1	Cplx1	complexin 1	12889	https://www.ncbi.nlm.nih.gov/gene/?term=12889
Kcnd1	Kcnd1	potassium voltage-gated channel, Shal-related family, member 1	16506	https://www.ncbi.nlm.nih.gov/gene/?term=16506
Ssr2	Ssr2	signal sequence receptor, beta	66256	https://www.ncbi.nlm.nih.gov/gene/?term=66256
Ints2	Ints2	integrator complex subunit 2	70422	https://www.ncbi.nlm.nih.gov/gene/?term=70422
Syt2	Syt2	synaptotagmin II	20980	https://www.ncbi.nlm.nih.gov/gene/?term=20980
Adamts3	Adamts3	a disintegrin-like and metallopeptidase (reprolysin type) with thrombospondin type 1 motif, 3	330119	https://www.ncbi.nlm.nih.gov/gene/?term=330119
Nkapl	Nkapl	NFKB activating protein-like	66707	https://www.ncbi.nlm.nih.gov/gene/?term=66707
Ap1s1	Ap1s1	adaptor protein complex AP-1, sigma 1	11769	https://www.ncbi.nlm.nih.gov/gene/?term=11769

Biology Open • Supplementary information

Hacd3	Hacd3	3-hydroxyacyl-CoA dehydratase 3	57874	https://www.ncbi.nlm.nih.gov/gene/?term=57874
Bdnf	Bdnf	brain derived neurotrophic factor	12064	https://www.ncbi.nlm.nih.gov/gene/?term=12064
Xpo6	Xpo6	exportin 6	74204	https://www.ncbi.nlm.nih.gov/gene/?term=74204
Cebpz	Cebpz	CCAAT/enhancer binding protein zeta	12607	https://www.ncbi.nlm.nih.gov/gene/?term=12607
Ust	Ust	uronyl-2-sulfotransferase	338362	https://www.ncbi.nlm.nih.gov/gene/?term=338362
Sprn	Sprn	shadow of prion protein	212518	https://www.ncbi.nlm.nih.gov/gene/?term=212518
Cap1	Cap1	CAP, adenylate cyclase-associated protein 1 (yeast)	12331	https://www.ncbi.nlm.nih.gov/gene/?term=12331
Pax7	Pax7	paired box 7	18509	https://www.ncbi.nlm.nih.gov/gene/?term=18509
Cttnbp2nl	Cttnbp2nl	CTTNBP2 N-terminal like	80281	https://www.ncbi.nlm.nih.gov/gene/?term=80281

Biology Open • Supplementary information

Caap1	Caap1	caspase activity and apoptosis inhibitor 1	67770	https://www.ncbi.nlm.nih.gov/gene/?term=67770
Msantd2	Msantd2	Myb/SANT-like DNA-binding domain containing 2	235184	https://www.ncbi.nlm.nih.gov/gene/?term=235184
Mtx1	Mtx1	metaxin 1	17827	https://www.ncbi.nlm.nih.gov/gene/?term=17827
Atf2	Atf2	activating transcription factor 2	11909	https://www.ncbi.nlm.nih.gov/gene/?term=11909
Tpm3	Tpm3	tropomyosin 3, gamma	59069	https://www.ncbi.nlm.nih.gov/gene/?term=59069
Ccdc166	Ccdc166	coiled-coil domain containing 166	223648	https://www.ncbi.nlm.nih.gov/gene/?term=223648
Stc2	Stc2	stanniocalcin 2	20856	https://www.ncbi.nlm.nih.gov/gene/?term=20856
Phax	Phax	phosphorylated adaptor for RNA export	56698	https://www.ncbi.nlm.nih.gov/gene/?term=56698
Arcn1	Arcn1	archain 1	213827	https://www.ncbi.nlm.nih.gov/gene/?term=213827

Biology Open • Supplementary information

Cmpk2	Cmpk2	cytidine monophosphate (UMP-CMP) kinase 2, mitochondrial	22169	https://www.ncbi.nlm.nih.gov/gene/?term=22169
G6pdx	G6pdx	glucose-6-phosphate dehydrogenase X-linked	14381	https://www.ncbi.nlm.nih.gov/gene/?term=14381
Lrch1	Lrch1	leucine-rich repeats and calponin homology (CH) domain containing 1	380916	https://www.ncbi.nlm.nih.gov/gene/?term=380916
Map1a	Map1a	microtubule-associated protein 1 A	17754	https://www.ncbi.nlm.nih.gov/gene/?term=17754
4930563E22Rik	4930563E22Rik	RIKEN cDNA 4930563E22 gene	75304	https://www.ncbi.nlm.nih.gov/gene/?term=75304
Mon2	Mon2	MON2 homolog, regulator of endosome to Golgi trafficking	67074	https://www.ncbi.nlm.nih.gov/gene/?term=67074
Hnrnpu	Hnrnpu	heterogeneous nuclear ribonucleoprotein U	51810	https://www.ncbi.nlm.nih.gov/gene/?term=51810
Tra2b	Tra2b	transformer 2 beta	20462	https://www.ncbi.nlm.nih.gov/gene/?term=20462
Cited2	Cited2	Cbp/p300-interacting transactivator, with Glu/Asp-rich carboxy-terminal domain, 2	17684	https://www.ncbi.nlm.nih.gov/gene/?term=17684

Biology Open • Supplementary information

Ankrd29	Ankrd29	ankyrin repeat domain 29	225187	https://www.ncbi.nlm.nih.gov/gene/?term=225187
Edn1	Edn1	endothelin 1	13614	https://www.ncbi.nlm.nih.gov/gene/?term=13614
Ss18	Ss18	SS18, nBAF chromatin remodeling complex subunit	268996	https://www.ncbi.nlm.nih.gov/gene/?term=268996
Esp34	Esp34	exocrine gland secreted peptide 34	100126773	https://www.ncbi.nlm.nih.gov/gene/?term=100126
Adprm	Adprm	ADP-ribose/CDP-alcohol diphosphatase, manganese dependent	66358	https://www.ncbi.nlm.nih.gov/gene/?term=66358
Ip6k2	Ip6k2	inositol hexaphosphate kinase 2	76500	https://www.ncbi.nlm.nih.gov/gene/?term=76500
Tex11	Tex11	testis expressed gene 11	83558	https://www.ncbi.nlm.nih.gov/gene/?term=83558
Hinfp	Hinfp	histone H4 transcription factor	102423	https://www.ncbi.nlm.nih.gov/gene/?term=102423
Osbpl7	Osbpl7	oxysterol binding protein-like 7	71240	https://www.ncbi.nlm.nih.gov/gene/?term=71240

Ccsap	Ccsap	centriole, cilia and spindle associated protein	73420	https://www.ncbi.nlm.nih.gov/gene/?term=73420
Ddx5	Ddx5	DEAD (Asp-Glu-Ala-Asp) box polypeptide 5	13207	https://www.ncbi.nlm.nih.gov/gene/?term=13207
Tppp	Tppp	tubulin polymerization promoting protein	72948	https://www.ncbi.nlm.nih.gov/gene/?term=72948
Serp1	Serp1	stress-associated endoplasmic reticulum protein 1	28146	https://www.ncbi.nlm.nih.gov/gene/?term=28146
Mmd2	Mmd2	monocyte to macrophage differentiation-associated 2	75104	https://www.ncbi.nlm.nih.gov/gene/?term=75104
Kmt2e	Kmt2e	lysine (K)-specific methyltransferase 2E	69188	https://www.ncbi.nlm.nih.gov/gene/?term=69188
Tmem178	Tmem178	transmembrane protein 178	68027	https://www.ncbi.nlm.nih.gov/gene/?term=68027
Eif1ax	Eif1ax	eukaryotic translation initiation factor 1A, X-linked	66235	https://www.ncbi.nlm.nih.gov/gene/?term=66235
Dach2	Dach2	dachshund family transcription factor 2	93837	https://www.ncbi.nlm.nih.gov/gene/?term=93837

Tmem18	Tmem18	transmembrane protein 18	211986	https://www.ncbi.nlm.nih.gov/gene/?term=211986
Unc119b	Unc119b	unc-119 lipid binding chaperone B	106840	https://www.ncbi.nlm.nih.gov/gene/?term=106840
Hspd1	Hspd1	heat shock protein 1 (chaperonin)	15510	https://www.ncbi.nlm.nih.gov/gene/?term=15510
Adar	Adar	adenosine deaminase, RNA-specific	56417	https://www.ncbi.nlm.nih.gov/gene/?term=56417
Sec22b	Sec22b	SEC22 homolog B, vesicle trafficking protein	20333	https://www.ncbi.nlm.nih.gov/gene/?term=20333
Neto1	Neto1	neuropilin (NRP) and tolloid (TLL)-like 1	246317	https://www.ncbi.nlm.nih.gov/gene/?term=246317
Smim14	Smim14	small integral membrane protein 14	68552	https://www.ncbi.nlm.nih.gov/gene/?term=68552
Ube4a	Ube4a	ubiquitination factor E4A	140630	https://www.ncbi.nlm.nih.gov/gene/?term=140630
Ppib	Ppib	peptidylprolyl isomerase B	19035	https://www.ncbi.nlm.nih.gov/gene/?term=19035

Wdr48	Wdr48	WD repeat domain 48	67561	https://www.ncbi.nlm.nih.gov/gene/?term=67561
Syt1	Syt1	synaptotagmin I	20979	https://www.ncbi.nlm.nih.gov/gene/?term=20979
Ywhaz	Ywhaz	tyrosine 3-monooxygenase/tryptophan 5-monooxygenase activation protein, zeta polypeptide	22631	https://www.ncbi.nlm.nih.gov/gene/?term=22631
Mrps31	Mrps31	mitochondrial ribosomal protein S31	57312	https://www.ncbi.nlm.nih.gov/gene/?term=57312
Efr3a	Efr3a	EFR3 homolog A	76740	https://www.ncbi.nlm.nih.gov/gene/?term=76740
Csrnp3	Csrnp3	cysteine-serine-rich nuclear protein 3	77771	https://www.ncbi.nlm.nih.gov/gene/?term=77771
Anxa10	Anxa10	annexin A10	26359	https://www.ncbi.nlm.nih.gov/gene/?term=26359
Oas3	Oas3	2'-5' oligoadenylate synthetase 3	246727	https://www.ncbi.nlm.nih.gov/gene/?term=246727
Sftpa1	Sftpa1	surfactant associated protein A1	20387	https://www.ncbi.nlm.nih.gov/gene/?term=20387

Biology Open • Supplementary information

Zfp462	Zfp462	zinc finger protein 462	242466	https://www.ncbi.nlm.nih.gov/gene/?term=242466
Smarcad1	Smarcad1	SWI/SNF-related, matrix-associated actin-dependent regulator of chromatin, subfamily a, containing DEAD/H box 1	13990	https://www.ncbi.nlm.nih.gov/gene/?term=13990
Prkx	Prkx	protein kinase, X-linked	19108	https://www.ncbi.nlm.nih.gov/gene/?term=19108
Tmx1	Tmx1	thioredoxin-related transmembrane protein 1	72736	https://www.ncbi.nlm.nih.gov/gene/?term=72736
D16Ertd472e	D16Ertd472e	DNA segment, Chr 16, ERATO Doi 472, expressed	67102	https://www.ncbi.nlm.nih.gov/gene/?term=67102
Lrpprc	Lrpprc	leucine-rich PPR-motif containing	72416	https://www.ncbi.nlm.nih.gov/gene/?term=72416
Itgb3	Itgb3	integrin beta 3	16416	https://www.ncbi.nlm.nih.gov/gene/?term=16416
Lck	Lck	lymphocyte protein tyrosine kinase	16818	https://www.ncbi.nlm.nih.gov/gene/?term=16818
Hemgn	Hemgn	hemogen	93966	https://www.ncbi.nlm.nih.gov/gene/?term=93966

Biology Open • Supplementary information

Shq1	Shq1	SHQ1 homolog (S. cerevisiae)	72171	https://www.ncbi.nlm.nih.gov/gene/?term=72171
B230219D22Rik	B230219D22Rik	RIKEN cDNA B230219D22 gene	78521	https://www.ncbi.nlm.nih.gov/gene/?term=78521
Parp14	Parp14	poly (ADP-ribose) polymerase family, member 14	547253	https://www.ncbi.nlm.nih.gov/gene/?term=547253
Eif4e	Eif4e	eukaryotic translation initiation factor 4E	13684	https://www.ncbi.nlm.nih.gov/gene/?term=13684
Marveld2	Marveld2	MARVEL (membrane-associating) domain containing 2	218518	https://www.ncbi.nlm.nih.gov/gene/?term=218518
Scrn1	Scrn1	secernin 1	69938	https://www.ncbi.nlm.nih.gov/gene/?term=69938
Prdm16	Prdm16	PR domain containing 16	70673	https://www.ncbi.nlm.nih.gov/gene/?term=70673
Cckar	Cckar	cholecystokinin A receptor	12425	https://www.ncbi.nlm.nih.gov/gene/?term=12425
Strn4	Strn4	striatin, calmodulin binding protein 4	97387	https://www.ncbi.nlm.nih.gov/gene/?term=97387

Xlr	Xlr	X-linked lymphocyte-regulated	22441	https://www.ncbi.nlm.nih.gov/gene/?term=22441
Rad51c	Rad51c	RAD51 paralog C	114714	https://www.ncbi.nlm.nih.gov/gene/?term=114714
Smad3	Smad3	SMAD family member 3	17127	https://www.ncbi.nlm.nih.gov/gene/?term=17127
Arhgap19	Arhgap19	Rho GTPase activating protein 19	71085	https://www.ncbi.nlm.nih.gov/gene/?term=71085
Prkab2	Prkab2	protein kinase, AMP-activated, beta 2 non-catalytic subunit	108097	https://www.ncbi.nlm.nih.gov/gene/?term=108097
Pira2	Pira2	paired-Ig-like receptor A2	18725	https://www.ncbi.nlm.nih.gov/gene/?term=18725
Mip	Mip	major intrinsic protein of lens fiber	17339	https://www.ncbi.nlm.nih.gov/gene/?term=17339
Stk38	Stk38	serine/threonine kinase 38	106504	https://www.ncbi.nlm.nih.gov/gene/?term=106504
Bpifc	Bpifc	BPI fold containing family C	270757	https://www.ncbi.nlm.nih.gov/gene/?term=270757

Biology Open • Supplementary information

Scube2	Scube2	signal peptide, CUB domain, EGF-like 2	56788	https://www.ncbi.nlm.nih.gov/gene/?term=56788
Amer2	Amer2	APC membrane recruitment 2	72125	https://www.ncbi.nlm.nih.gov/gene/?term=72125
Il10ra	Il10ra	interleukin 10 receptor, alpha	16154	https://www.ncbi.nlm.nih.gov/gene/?term=16154
Sv2a	Sv2a	synaptic vesicle glycoprotein 2 a	64051	https://www.ncbi.nlm.nih.gov/gene/?term=64051
Smpdl3a	Smpdl3a	sphingomyelin phosphodiesterase, acid-like 3A	57319	https://www.ncbi.nlm.nih.gov/gene/?term=57319
Dhcr24	Dhcr24	24-dehydrocholesterol reductase	74754	https://www.ncbi.nlm.nih.gov/gene/?term=74754
2610301B20Rik	2610301B20Rik	RIKEN cDNA 2610301B20 gene	67157	https://www.ncbi.nlm.nih.gov/gene/?term=67157
Syk	Syk	spleen tyrosine kinase	20963	https://www.ncbi.nlm.nih.gov/gene/?term=20963
Ndrg3	Ndrg3	N-myc downstream regulated gene 3	29812	https://www.ncbi.nlm.nih.gov/gene/?term=29812

Biology Open • Supplementary information

Xrn2	Xrn2	5'-3' exoribonuclease 2	24128	https://www.ncbi.nlm.nih.gov/gene/?term=24128
Lrp8	Lrp8	low density lipoprotein receptor-related protein 8, apolipoprotein e receptor	16975	https://www.ncbi.nlm.nih.gov/gene/?term=16975
Phc2	Phc2	polyhomeotic 2	54383	https://www.ncbi.nlm.nih.gov/gene/?term=54383
Cst6	Cst6	cystatin E/M	73720	https://www.ncbi.nlm.nih.gov/gene/?term=73720
Phf21a	Phf21a	PHD finger protein 21A	192285	https://www.ncbi.nlm.nih.gov/gene/?term=192285
R3hdm4	R3hdm4	R3H domain containing 4	109284	https://www.ncbi.nlm.nih.gov/gene/?term=109284
Gnl3l	Gnl3l	guanine nucleotide binding protein-like 3 (nucleolar)-like	237107	https://www.ncbi.nlm.nih.gov/gene/?term=237107
Zcchc18	Zcchc18	zinc finger, CCHC domain containing 18	66995	https://www.ncbi.nlm.nih.gov/gene/?term=66995
AI597479	AI597479	expressed sequence AI597479	98404	https://www.ncbi.nlm.nih.gov/gene/?term=98404

Snx27	Snx27	sorting nexin family member 27	76742	https://www.ncbi.nlm.nih.gov/gene/?term=76742
Kat7	Kat7	K(lysine) acetyltransferase 7	217127	https://www.ncbi.nlm.nih.gov/gene/?term=217127
Apool	Apool	apolipoprotein O-like	68117	https://www.ncbi.nlm.nih.gov/gene/?term=68117
Upp2	Upp2	uridine phosphorylase 2	76654	https://www.ncbi.nlm.nih.gov/gene/?term=76654
Gabra2	Gabra2	gamma-aminobutyric acid (GABA) A receptor, subunit alpha 2	14395	https://www.ncbi.nlm.nih.gov/gene/?term=14395
Neurog2	Neurog2	neurogenin 2	11924	https://www.ncbi.nlm.nih.gov/gene/?term=11924
Zfp729b	Zfp729b	zinc finger protein 729b	100416706	https://www.ncbi.nlm.nih.gov/gene/?term=100416
Ppp1r21	Ppp1r21	protein phosphatase 1, regulatory subunit 21	73825	https://www.ncbi.nlm.nih.gov/gene/?term=73825
Tspan3	Tspan3	tetraspanin 3	56434	https://www.ncbi.nlm.nih.gov/gene/?term=56434

Sox9	Sox9	SRY (sex determining region Y)-box 9	20682	https://www.ncbi.nlm.nih.gov/gene/?term=20682
Pes1	Pes1	pescadillo ribosomal biogenesis factor 1	64934	https://www.ncbi.nlm.nih.gov/gene/?term=64934
Mis18a	Mis18a	MIS18 kinetochore protein A	66578	https://www.ncbi.nlm.nih.gov/gene/?term=66578
Tpbpa	Tpbpa	trophoblast specific protein alpha	21984	https://www.ncbi.nlm.nih.gov/gene/?term=21984
Lin52	Lin52	lin-52 homolog (C. elegans)	217708	https://www.ncbi.nlm.nih.gov/gene/?term=217708
Zfp503	Zfp503	zinc finger protein 503	218820	https://www.ncbi.nlm.nih.gov/gene/?term=218820
Eme1	Eme1	essential meiotic structure-specific endonuclease 1	268465	https://www.ncbi.nlm.nih.gov/gene/?term=268465
Mei4	Mei4	meiotic double-stranded break formation protein 4	75033	https://www.ncbi.nlm.nih.gov/gene/?term=75033
Ubtd2	Ubtd2	ubiquitin domain containing 2	327900	https://www.ncbi.nlm.nih.gov/gene/?term=327900

Biology Open • Supplementary information

Pdgfrl	Pdgfrl	platelet-derived growth factor receptor-like	68797	https://www.ncbi.nlm.nih.gov/gene/?term=68797
Nup35	Nup35	nucleoporin 35	69482	https://www.ncbi.nlm.nih.gov/gene/?term=69482
Map10	Map10	microtubule-associated protein 10	74393	https://www.ncbi.nlm.nih.gov/gene/?term=74393
Srgap1	Srgap1	SLIT-ROBO Rho GTPase activating protein 1	117600	https://www.ncbi.nlm.nih.gov/gene/?term=117600
Npm1	Npm1	nucleophosmin 1	18148	https://www.ncbi.nlm.nih.gov/gene/?term=18148
Zmynd11	Zmynd11	zinc finger, MYND domain containing 11	66505	https://www.ncbi.nlm.nih.gov/gene/?term=66505
Synpr	Synpr	synaptoporin	72003	https://www.ncbi.nlm.nih.gov/gene/?term=72003
Ccdc169	Ccdc169	coiled-coil domain containing 169	320604	https://www.ncbi.nlm.nih.gov/gene/?term=320604
Six6	Six6	sine oculis-related homeobox 6	20476	https://www.ncbi.nlm.nih.gov/gene/?term=20476

Biology Open • Supplementary information

Psmc6	Psmc6	proteasome (prosome, macropain) 26S subunit, ATPase, 6	67089	https://www.ncbi.nlm.nih.gov/gene/?term=67089
Birc6	Birc6	baculoviral IAP repeat-containing 6	12211	https://www.ncbi.nlm.nih.gov/gene/?term=12211
2310030G06Rik	2310030G06Rik	RIKEN cDNA 2310030G06 gene	66952	https://www.ncbi.nlm.nih.gov/gene/?term=66952
Tubgcp5	Tubgcp5	tubulin, gamma complex associated protein 5	233276	https://www.ncbi.nlm.nih.gov/gene/?term=233276
Olfr658	Olfr658	olfactory receptor 658	259051	https://www.ncbi.nlm.nih.gov/gene/?term=259051
Rab11a	Rab11a	RAB11A, member RAS oncogene family	53869	https://www.ncbi.nlm.nih.gov/gene/?term=53869
Ikzf5	Ikzf5	IKAROS family zinc finger 5	67143	https://www.ncbi.nlm.nih.gov/gene/?term=67143
Zfp12	Zfp12	zinc finger protein 12	231866	https://www.ncbi.nlm.nih.gov/gene/?term=231866
Lad1	Lad1	ladinin	16763	https://www.ncbi.nlm.nih.gov/gene/?term=16763

Biology Open • Supplementary information

Rgl1	Rgl1	ral guanine nucleotide dissociation stimulator,-like 1	19731	https://www.ncbi.nlm.nih.gov/gene/?term=19731
Sh3bgrl3	Sh3bgrl3	SH3 domain binding glutamic acid-rich protein-like 3	73723	https://www.ncbi.nlm.nih.gov/gene/?term=73723
Spata1	Spata1	spermatogenesis associated 1	70951	https://www.ncbi.nlm.nih.gov/gene/?term=70951
Btbd6	Btbd6	BTB (POZ) domain containing 6	399566	https://www.ncbi.nlm.nih.gov/gene/?term=399566
Sap18b	Sap18b	Sin3-associated polypeptide 18B	100041953	https://www.ncbi.nlm.nih.gov/gene/?term=100041
Hcar2	Hcar2	hydroxycarboxylic acid receptor 2	80885	https://www.ncbi.nlm.nih.gov/gene/?term=80885
Hspb6	Hspb6	heat shock protein, alpha-crystallin-related, B6	243912	https://www.ncbi.nlm.nih.gov/gene/?term=243912
Prkab1	Prkab1	protein kinase, AMP-activated, beta 1 non-catalytic subunit	19079	https://www.ncbi.nlm.nih.gov/gene/?term=19079
Gpr137b	Gpr137b	G protein-coupled receptor 137B	83924	https://www.ncbi.nlm.nih.gov/gene/?term=83924

Lbh	Lbh	limb-bud and heart	77889	https://www.ncbi.nlm.nih.gov/gene/?term=77889
Yrdc	Yrdc	yrdC domain containing (E.coli)	230734	https://www.ncbi.nlm.nih.gov/gene/?term=230734
Pax1	Pax1	paired box 1	18503	https://www.ncbi.nlm.nih.gov/gene/?term=18503
Trpc3	Trpc3	transient receptor potential cation channel, subfamily C, member 3	22065	https://www.ncbi.nlm.nih.gov/gene/?term=22065
Pla2g4e	Pla2g4e	phospholipase A2, group IVE	329502	https://www.ncbi.nlm.nih.gov/gene/?term=329502
Bmp2k	Bmp2k	BMP2 inducible kinase	140780	https://www.ncbi.nlm.nih.gov/gene/?term=140780
Dab2ip	Dab2ip	disabled 2 interacting protein	69601	https://www.ncbi.nlm.nih.gov/gene/?term=69601
Ubxn7	Ubxn7	UBX domain protein 7	224111	https://www.ncbi.nlm.nih.gov/gene/?term=224111
Slc17a8	Slc17a8	solute carrier family 17 (sodium-dependent inorganic phosphate cotransporter), member 8	216227	https://www.ncbi.nlm.nih.gov/gene/?term=216227

Biology Open • Supplementary information

Snx6	Snx6	sorting nexin 6	72183	https://www.ncbi.nlm.nih.gov/gene/?term=72183
Cdkl1	Cdkl1	cyclin-dependent kinase-like 1 (CDC2-related kinase)	71091	https://www.ncbi.nlm.nih.gov/gene/?term=71091
Fgd4	Fgd4	FYVE, RhoGEF and PH domain containing 4	224014	https://www.ncbi.nlm.nih.gov/gene/?term=224014
Klb	Klb	klotho beta	83379	https://www.ncbi.nlm.nih.gov/gene/?term=83379
Slk	Slk	STE20-like kinase	20874	https://www.ncbi.nlm.nih.gov/gene/?term=20874
Elovl7	Elovl7	ELOVL family member 7, elongation of long chain fatty acids (yeast)	74559	https://www.ncbi.nlm.nih.gov/gene/?term=74559
Inpp5j	Inpp5j	inositol polyphosphate 5-phosphatase J	170835	https://www.ncbi.nlm.nih.gov/gene/?term=170835
Sdk1	Sdk1	sidekick cell adhesion molecule 1	330222	https://www.ncbi.nlm.nih.gov/gene/?term=330222
Kcna4	Kcna4	potassium voltage-gated channel, shaker-related subfamily, member 4	16492	https://www.ncbi.nlm.nih.gov/gene/?term=16492

Biology Open • Supplementary information

Gprc5b	Gprc5b	G protein-coupled receptor, family C, group 5, member B	64297	https://www.ncbi.nlm.nih.gov/gene/?term=64297
Pigg	Pigg	phosphatidylinositol glycan anchor biosynthesis, class G	433931	https://www.ncbi.nlm.nih.gov/gene/?term=433931
Olfr976	Olfr976	olfactory receptor 976	258364	https://www.ncbi.nlm.nih.gov/gene/?term=258364
Atg14	Atg14	autophagy related 14	100504663	https://www.ncbi.nlm.nih.gov/gene/?term=100504[illegible]
Nubpl	Nubpl	nucleotide binding protein-like	76826	https://www.ncbi.nlm.nih.gov/gene/?term=76826
Ddah1	Ddah1	dimethylarginine dimethylaminohydrolase 1	69219	https://www.ncbi.nlm.nih.gov/gene/?term=69219
Rbms3	Rbms3	RNA binding motif, single stranded interacting protein	207181	https://www.ncbi.nlm.nih.gov/gene/?term=207181
Nipa2	Nipa2	non imprinted in Prader-Willi/Angelman syndrome 2 homolog (human)	93790	https://www.ncbi.nlm.nih.gov/gene/?term=93790
Stard7	Stard7	START domain containing 7	99138	https://www.ncbi.nlm.nih.gov/gene/?term=99138

Biology Open • Supplementary information

Tpm4	Tpm4	tropomyosin 4	326618	https://www.ncbi.nlm.nih.gov/gene/?term=326618
Atp6v0d2	Atp6v0d2	ATPase, H+ transporting, lysosomal V0 subunit D2	242341	https://www.ncbi.nlm.nih.gov/gene/?term=242341
Nav2	Nav2	neuron navigator 2	78286	https://www.ncbi.nlm.nih.gov/gene/?term=78286
Ccar2	Ccar2	cell cycle activator and apoptosis regulator 2	219158	https://www.ncbi.nlm.nih.gov/gene/?term=219158
Alpi	Alpi	alkaline phosphatase, intestinal	76768	https://www.ncbi.nlm.nih.gov/gene/?term=76768
Snn	Snn	stannin	20621	https://www.ncbi.nlm.nih.gov/gene/?term=20621
Dolk	Dolk	dolichol kinase	227697	https://www.ncbi.nlm.nih.gov/gene/?term=227697
9130409I23Rik	9130409I23Rik	RIKEN cDNA 9130409I23 gene	619326	https://www.ncbi.nlm.nih.gov/gene/?term=619326
Scfd2	Scfd2	Sec1 family domain containing 2	212986	https://www.ncbi.nlm.nih.gov/gene/?term=212986

Biology Open • Supplementary information

Epb41l1	Epb41l1	erythrocyte membrane protein band 4.1 like 1	13821	https://www.ncbi.nlm.nih.gov/gene/?term=13821
Rnf185	Rnf185	ring finger protein 185	193670	https://www.ncbi.nlm.nih.gov/gene/?term=193670
Ubxn10	Ubxn10	UBX domain protein 10	212190	https://www.ncbi.nlm.nih.gov/gene/?term=212190
Clcn4	Clcn4	chloride channel, voltage-sensitive 4	12727	https://www.ncbi.nlm.nih.gov/gene/?term=12727
Ggt7	Ggt7	gamma-glutamyltransferase 7	207182	https://www.ncbi.nlm.nih.gov/gene/?term=207182
Sh2d1b1	Sh2d1b1	SH2 domain containing 1B1	26904	https://www.ncbi.nlm.nih.gov/gene/?term=26904
Gpx7	Gpx7	glutathione peroxidase 7	67305	https://www.ncbi.nlm.nih.gov/gene/?term=67305
Kif9	Kif9	kinesin family member 9	16578	https://www.ncbi.nlm.nih.gov/gene/?term=16578
Emilin3	Emilin3	elastin microfibril interfacer 3	280635	https://www.ncbi.nlm.nih.gov/gene/?term=280635

Parva	Parva	parvin, alpha	57342	https://www.ncbi.nlm.nih.gov/gene/?term=57342
Ppp1r3f	Ppp1r3f	protein phosphatase 1, regulatory subunit 3F	54646	https://www.ncbi.nlm.nih.gov/gene/?term=54646
Alad	Alad	aminolevulinate, delta-, dehydratase	17025	https://www.ncbi.nlm.nih.gov/gene/?term=17025
Ubiad1	Ubiad1	UbiA prenyltransferase domain containing 1	71707	https://www.ncbi.nlm.nih.gov/gene/?term=71707
Ccdc127	Ccdc127	coiled-coil domain containing 127	67433	https://www.ncbi.nlm.nih.gov/gene/?term=67433
Grpel2	Grpel2	GrpE-like 2, mitochondrial	17714	https://www.ncbi.nlm.nih.gov/gene/?term=17714
Tor4a	Tor4a	torsin family 4, member A	227612	https://www.ncbi.nlm.nih.gov/gene/?term=227612
Hip1	Hip1	huntingtin interacting protein 1	215114	https://www.ncbi.nlm.nih.gov/gene/?term=215114
Chrm1	Chrm1	cholinergic receptor, muscarinic 1, CNS	12669	https://www.ncbi.nlm.nih.gov/gene/?term=12669

Biology Open • Supplementary information

Soga1	Soga1	suppressor of glucose, autophagy associated 1	320706	https://www.ncbi.nlm.nih.gov/gene/?term=320706
Palm	Palm	paralemmin	18483	https://www.ncbi.nlm.nih.gov/gene/?term=18483
Vldlr	Vldlr	very low density lipoprotein receptor	22359	https://www.ncbi.nlm.nih.gov/gene/?term=22359
Glod5	Glod5	glyoxalase domain containing 5	69824	https://www.ncbi.nlm.nih.gov/gene/?term=69824
Rfng	Rfng	RFNG O-fucosylpeptide 3-beta-N-acetylglucosaminyltransferase	19719	https://www.ncbi.nlm.nih.gov/gene/?term=19719
Lrrc3	Lrrc3	leucine rich repeat containing 3	237387	https://www.ncbi.nlm.nih.gov/gene/?term=237387
Cdkl3	Cdkl3	cyclin-dependent kinase-like 3	213084	https://www.ncbi.nlm.nih.gov/gene/?term=213084
Exd2	Exd2	exonuclease 3'-5' domain containing 2	97827	https://www.ncbi.nlm.nih.gov/gene/?term=97827
Nnat	Nnat	neuronatin	18111	https://www.ncbi.nlm.nih.gov/gene/?term=18111

Tbc1d22a	Tbc1d22a	TBC1 domain family, member 22a	223754	https://www.ncbi.nlm.nih.gov/gene/?term=223754
Dlgap1	Dlgap1	DLG associated protein 1	224997	https://www.ncbi.nlm.nih.gov/gene/?term=224997
Baz1a	Baz1a	bromodomain adjacent to zinc finger domain 1A	217578	https://www.ncbi.nlm.nih.gov/gene/?term=217578
Tarsl2	Tarsl2	threonyl-tRNA synthetase-like 2	272396	https://www.ncbi.nlm.nih.gov/gene/?term=272396
Ptprk	Ptprk	protein tyrosine phosphatase, receptor type, K	19272	https://www.ncbi.nlm.nih.gov/gene/?term=19272
Hps6	Hps6	HPS6, biogenesis of lysosomal organelles complex 2 subunit 3	20170	https://www.ncbi.nlm.nih.gov/gene/?term=20170
Nphp1	Nphp1	nephronophthisis 1 (juvenile) homolog (human)	53885	https://www.ncbi.nlm.nih.gov/gene/?term=53885
Fzd10	Fzd10	frizzled class receptor 10	93897	https://www.ncbi.nlm.nih.gov/gene/?term=93897
Psd4	Psd4	pleckstrin and Sec7 domain containing 4	215632	https://www.ncbi.nlm.nih.gov/gene/?term=215632

Biology Open • Supplementary information

Rad21	Rad21	RAD21 cohesin complex component	19357	https://www.ncbi.nlm.nih.gov/gene/?term=19357
Pnma2	Pnma2	paraneoplastic antigen MA2	239157	https://www.ncbi.nlm.nih.gov/gene/?term=239157
Rev3l	Rev3l	REV3 like, DNA directed polymerase zeta catalytic subunit	19714	https://www.ncbi.nlm.nih.gov/gene/?term=19714
Gm5544	Gm5544	predicted gene 5544	433632	https://www.ncbi.nlm.nih.gov/gene/?term=433632
Pde9a	Pde9a	phosphodiesterase 9A	18585	https://www.ncbi.nlm.nih.gov/gene/?term=18585
Ccnl2	Ccnl2	cyclin L2	56036	https://www.ncbi.nlm.nih.gov/gene/?term=56036
Med13l	Med13l	mediator complex subunit 13-like	76199	https://www.ncbi.nlm.nih.gov/gene/?term=76199
Cfap70	Cfap70	cilia and flagella associated protein 70	76670	https://www.ncbi.nlm.nih.gov/gene/?term=76670
Patz1	Patz1	POZ (BTB) and AT hook containing zinc finger 1	56218	https://www.ncbi.nlm.nih.gov/gene/?term=56218

Biology Open • Supplementary information

Dazap1	Dazap1	DAZ associated protein 1	70248	https://www.ncbi.nlm.nih.gov/gene/?term=70248
Klf9	Klf9	Kruppel-like factor 9	16601	https://www.ncbi.nlm.nih.gov/gene/?term=16601
Csk	Csk	c-src tyrosine kinase	12988	https://www.ncbi.nlm.nih.gov/gene/?term=12988
Zbtb2	Zbtb2	zinc finger and BTB domain containing 2	381990	https://www.ncbi.nlm.nih.gov/gene/?term=381990
Rab40b	Rab40b	Rab40B, member RAS oncogene family	217371	https://www.ncbi.nlm.nih.gov/gene/?term=217371
Rgs13	Rgs13	regulator of G-protein signaling 13	246709	https://www.ncbi.nlm.nih.gov/gene/?term=246709
Spink5	Spink5	serine peptidase inhibitor, Kazal type 5	72432	https://www.ncbi.nlm.nih.gov/gene/?term=72432
Pcdhb4	Pcdhb4	protocadherin beta 4	93875	https://www.ncbi.nlm.nih.gov/gene/?term=93875
Jak2	Jak2	Janus kinase 2	16452	https://www.ncbi.nlm.nih.gov/gene/?term=16452

Biology Open • Supplementary information

Tmem170	Tmem170	transmembrane protein 170	66817	https://www.ncbi.nlm.nih.gov/gene/?term=66817
Chmp1b	Chmp1b	charged multivesicular body protein 1B	67064	https://www.ncbi.nlm.nih.gov/gene/?term=67064
Mysm1	Mysm1	myb-like, SWIRM and MPN domains 1	320713	https://www.ncbi.nlm.nih.gov/gene/?term=320713
Sde2	Sde2	SDE2 telomere maintenance homolog (S. pombe)	208768	https://www.ncbi.nlm.nih.gov/gene/?term=208768
Fchsd2	Fchsd2	FCH and double SH3 domains 2	207278	https://www.ncbi.nlm.nih.gov/gene/?term=207278
Trmt2a	Trmt2a	TRM2 tRNA methyltransferase 2A	15547	https://www.ncbi.nlm.nih.gov/gene/?term=15547
Utp23	Utp23	UTP23 small subunit processome component	78581	https://www.ncbi.nlm.nih.gov/gene/?term=78581
Soga3	Soga3	SOGA family member 3	67412	https://www.ncbi.nlm.nih.gov/gene/?term=67412
Kpnb1	Kpnb1	karyopherin (importin) beta 1	16211	https://www.ncbi.nlm.nih.gov/gene/?term=16211

Biology Open • Supplementary information

Nras	Nras	neuroblastoma ras oncogene	18176	https://www.ncbi.nlm.nih.gov/gene/?term=18176
Fam214a	Fam214a	family with sequence similarity 214, member A	235493	https://www.ncbi.nlm.nih.gov/gene/?term=235493
Zbtb11	Zbtb11	zinc finger and BTB domain containing 11	271377	https://www.ncbi.nlm.nih.gov/gene/?term=271377
Agmo	Agmo	alkylglycerol monooxygenase	319660	https://www.ncbi.nlm.nih.gov/gene/?term=319660
Ppp2r2c	Ppp2r2c	protein phosphatase 2, regulatory subunit B, gamma	269643	https://www.ncbi.nlm.nih.gov/gene/?term=269643
Ric3	Ric3	RIC3 acetylcholine receptor chaperone	320360	https://www.ncbi.nlm.nih.gov/gene/?term=320360
Snrnp70	Snrnp70	small nuclear ribonucleoprotein 70 (U1)	20637	https://www.ncbi.nlm.nih.gov/gene/?term=20637
Abraxas1	Abraxas1	BRCA1 A complex subunit	70681	https://www.ncbi.nlm.nih.gov/gene/?term=70681
Thoc2	Thoc2	THO complex 2	331401	https://www.ncbi.nlm.nih.gov/gene/?term=331401

Biology Open • Supplementary information

Nudt4	Nudt4	nudix (nucleoside diphosphate linked moiety X)-type motif 4	71207	https://www.ncbi.nlm.nih.gov/gene/?term=71207
Pttg1ip	Pttg1ip	pituitary tumor-transforming 1 interacting protein	108705	https://www.ncbi.nlm.nih.gov/gene/?term=108705
Cers5	Cers5	ceramide synthase 5	71949	https://www.ncbi.nlm.nih.gov/gene/?term=71949
Col23a1	Col23a1	collagen, type XXIII, alpha 1	237759	https://www.ncbi.nlm.nih.gov/gene/?term=237759
Ntan1	Ntan1	N-terminal Asn amidase	18203	https://www.ncbi.nlm.nih.gov/gene/?term=18203
Ly6g5b	Ly6g5b	lymphocyte antigen 6 complex, locus G5B	266614	https://www.ncbi.nlm.nih.gov/gene/?term=266614
Msrb2	Msrb2	methionine sulfoxide reductase B2	76467	https://www.ncbi.nlm.nih.gov/gene/?term=76467
Psmd12	Psmd12	proteasome (prosome, macropain) 26S subunit, non-ATPase, 12	66997	https://www.ncbi.nlm.nih.gov/gene/?term=66997
Faap20	Faap20	Fanconi anemia core complex associated protein 20	67513	https://www.ncbi.nlm.nih.gov/gene/?term=67513

Cbx3	Cbx3	chromobox 3	12417	https://www.ncbi.nlm.nih.gov/gene/?term=12417
Fkbp1b	Fkbp1b	FK506 binding protein 1b	14226	https://www.ncbi.nlm.nih.gov/gene/?term=14226
Cnih4	Cnih4	cornichon family AMPA receptor auxiliary protein 4	98417	https://www.ncbi.nlm.nih.gov/gene/?term=98417
Dll1	Dll1	delta like canonical Notch ligand 1	13388	https://www.ncbi.nlm.nih.gov/gene/?term=13388
Arel1	Arel1	apoptosis resistant E3 ubiquitin protein ligase 1	68497	https://www.ncbi.nlm.nih.gov/gene/?term=68497
Eloa	Eloa	elongin A	27224	https://www.ncbi.nlm.nih.gov/gene/?term=27224
Igf2bp1	Igf2bp1	insulin-like growth factor 2 mRNA binding protein 1	140486	https://www.ncbi.nlm.nih.gov/gene/?term=140486
Fbxo41	Fbxo41	F-box protein 41	330369	https://www.ncbi.nlm.nih.gov/gene/?term=330369
Lsm14b	Lsm14b	LSM family member 14B	241846	https://www.ncbi.nlm.nih.gov/gene/?term=241846

Biology Open • Supplementary information

Pkp2	Pkp2	plakophilin 2	67451	https://www.ncbi.nlm.nih.gov/gene/?term=67451
Olfr121	Olfr121	olfactory receptor 121	258622	https://www.ncbi.nlm.nih.gov/gene/?term=258622
Phldb1	Phldb1	pleckstrin homology like domain, family B, member 1	102693	https://www.ncbi.nlm.nih.gov/gene/?term=102693
Amotl2	Amotl2	angiomotin-like 2	56332	https://www.ncbi.nlm.nih.gov/gene/?term=56332
Dnm3	Dnm3	dynamin 3	103967	https://www.ncbi.nlm.nih.gov/gene/?term=103967
Zfp958	Zfp958	zinc finger protein 958	233987	https://www.ncbi.nlm.nih.gov/gene/?term=233987
Fbxl16	Fbxl16	F-box and leucine-rich repeat protein 16	214931	https://www.ncbi.nlm.nih.gov/gene/?term=214931
Map4k5	Map4k5	mitogen-activated protein kinase kinase kinase kinase 5	399510	https://www.ncbi.nlm.nih.gov/gene/?term=399510
Appl2	Appl2	adaptor protein, phosphotyrosine interaction, PH domain and leucine zipper containing 2	216190	https://www.ncbi.nlm.nih.gov/gene/?term=216190

Biology Open • Supplementary information

Smarce1	Smarce1	SWI/SNF related, matrix associated, actin dependent regulator of chromatin, subfamily e, member 1	57376	https://www.ncbi.nlm.nih.gov/gene/?term=57376
Grk2	Grk2	G protein-coupled receptor kinase 2	110355	https://www.ncbi.nlm.nih.gov/gene/?term=110355
Fkbp1a	Fkbp1a	FK506 binding protein 1a	14225	https://www.ncbi.nlm.nih.gov/gene/?term=14225
Lonrf1	Lonrf1	LON peptidase N-terminal domain and ring finger 1	244421	https://www.ncbi.nlm.nih.gov/gene/?term=244421
Rbm27	Rbm27	RNA binding motif protein 27	225432	https://www.ncbi.nlm.nih.gov/gene/?term=225432
Arhgef10l	Arhgef10l	Rho guanine nucleotide exchange factor (GEF) 10-like	72754	https://www.ncbi.nlm.nih.gov/gene/?term=72754
Ccz1	Ccz1	CCZ1 vacuolar protein trafficking and biogenesis associated	231874	https://www.ncbi.nlm.nih.gov/gene/?term=231874
Fam172a	Fam172a	family with sequence similarity 172, member A	68675	https://www.ncbi.nlm.nih.gov/gene/?term=68675
Ptpn11	Ptpn11	protein tyrosine phosphatase, non-receptor type 11	19247	https://www.ncbi.nlm.nih.gov/gene/?term=19247

Biology Open • Supplementary information

Nfkb2	Nfkb2	nuclear factor of kappa light polypeptide gene enhancer in B cells 2, p49/p100	18034	https://www.ncbi.nlm.nih.gov/gene/?term=18034
5730507C01Rik	5730507C01Rik	RIKEN cDNA 5730507C01 gene	236366	https://www.ncbi.nlm.nih.gov/gene/?term=236366
Fam71f2	Fam71f2	family with sequence similarity 71, member F2	245884	https://www.ncbi.nlm.nih.gov/gene/?term=245884
Cxcl16	Cxcl16	chemokine (C-X-C motif) ligand 16	66102	https://www.ncbi.nlm.nih.gov/gene/?term=66102
Csgalnact2	Csgalnact2	chondroitin sulfate N-acetylgalactosaminyltransferase 2	78752	https://www.ncbi.nlm.nih.gov/gene/?term=78752
Osbpl5	Osbpl5	oxysterol binding protein-like 5	79196	https://www.ncbi.nlm.nih.gov/gene/?term=79196
Cep135	Cep135	centrosomal protein 135	381644	https://www.ncbi.nlm.nih.gov/gene/?term=381644
Acaca	Acaca	acetyl-Coenzyme A carboxylase alpha	107476	https://www.ncbi.nlm.nih.gov/gene/?term=107476
Per2	Per2	period circadian clock 2	18627	https://www.ncbi.nlm.nih.gov/gene/?term=18627

Spred2	Spred2	sprouty-related, EVH1 domain containing 2	114716	https://www.ncbi.nlm.nih.gov/gene/?term=114716
Vgll3	Vgll3	vestigial like family member 3	73569	https://www.ncbi.nlm.nih.gov/gene/?term=73569
Zbtb5	Zbtb5	zinc finger and BTB domain containing 5	230119	https://www.ncbi.nlm.nih.gov/gene/?term=230119
Klf11	Klf11	Kruppel-like factor 11	194655	https://www.ncbi.nlm.nih.gov/gene/?term=194655
Dyrk2	Dyrk2	dual-specificity tyrosine-(Y)-phosphorylation regulated kinase 2	69181	https://www.ncbi.nlm.nih.gov/gene/?term=69181
Lancl3	Lancl3	LanC lantibiotic synthetase component C-like 3 (bacterial)	236285	https://www.ncbi.nlm.nih.gov/gene/?term=236285
Gabbr2	Gabbr2	gamma-aminobutyric acid (GABA) B receptor, 2	242425	https://www.ncbi.nlm.nih.gov/gene/?term=242425
Ntsr1	Ntsr1	neurotensin receptor 1	18216	https://www.ncbi.nlm.nih.gov/gene/?term=18216
Zc3h10	Zc3h10	zinc finger CCCH type containing 10	103284	https://www.ncbi.nlm.nih.gov/gene/?term=103284

Biology Open • Supplementary information

Wtap	Wtap	Wilms tumour 1-associating protein	60532	https://www.ncbi.nlm.nih.gov/gene/?term=60532
Ccn4	Ccn4	cellular communication network factor 4	22402	https://www.ncbi.nlm.nih.gov/gene/?term=22402
Fscn1	Fscn1	fascin actin-bundling protein 1	14086	https://www.ncbi.nlm.nih.gov/gene/?term=14086
Fanci	Fanci	Fanconi anemia, complementation group I	208836	https://www.ncbi.nlm.nih.gov/gene/?term=208836
Agk	Agk	acylglycerol kinase	69923	https://www.ncbi.nlm.nih.gov/gene/?term=69923
1110004F10Rik	1110004F10Rik	RIKEN cDNA 1110004F10 gene	56372	https://www.ncbi.nlm.nih.gov/gene/?term=56372
Pou4f1	Pou4f1	POU domain, class 4, transcription factor 1	18996	https://www.ncbi.nlm.nih.gov/gene/?term=18996
Ugt8a	Ugt8a	UDP galactosyltransferase 8A	22239	https://www.ncbi.nlm.nih.gov/gene/?term=22239
Sema3d	Sema3d	sema domain, immunoglobulin domain (Ig), short basic domain, secreted, (semaphorin) 3D	108151	https://www.ncbi.nlm.nih.gov/gene/?term=108151

Biology Open • Supplementary information

Kif20a	Kif20a	kinesin family member 20A	19348	https://www.ncbi.nlm.nih.gov/gene/?term=19348
Tbx4	Tbx4	T-box 4	21387	https://www.ncbi.nlm.nih.gov/gene/?term=21387
Fzd5	Fzd5	frizzled class receptor 5	14367	https://www.ncbi.nlm.nih.gov/gene/?term=14367
Csn2	Csn2	casein beta	12991	https://www.ncbi.nlm.nih.gov/gene/?term=12991
Cemip	Cemip	cell migration inducing protein, hyaluronan binding	80982	https://www.ncbi.nlm.nih.gov/gene/?term=80982
Nipbl	Nipbl	NIPBL cohesin loading factor	71175	https://www.ncbi.nlm.nih.gov/gene/?term=71175
Myo9a	Myo9a	myosin IXa	270163	https://www.ncbi.nlm.nih.gov/gene/?term=270163
Ltbp1	Ltbp1	latent transforming growth factor beta binding protein 1	268977	https://www.ncbi.nlm.nih.gov/gene/?term=268977
Fgd6	Fgd6	FYVE, RhoGEF and PH domain containing 6	13998	https://www.ncbi.nlm.nih.gov/gene/?term=13998

Biology Open • Supplementary information

Pikfyve	Pikfyve	phosphoinositide kinase, FYVE type zinc finger containing	18711	https://www.ncbi.nlm.nih.gov/gene/?term=18711
Homer1	Homer1	homer scaffolding protein 1	26556	https://www.ncbi.nlm.nih.gov/gene/?term=26556
N6amt1	N6amt1	N-6 adenine-specific DNA methyltransferase 1 (putative)	67768	https://www.ncbi.nlm.nih.gov/gene/?term=67768
Cebpb	Cebpb	CCAAT/enhancer binding protein (C/EBP), beta	12608	https://www.ncbi.nlm.nih.gov/gene/?term=12608
Numbl	Numbl	numb-like	18223	https://www.ncbi.nlm.nih.gov/gene/?term=18223
Parp8	Parp8	poly (ADP-ribose) polymerase family, member 8	52552	https://www.ncbi.nlm.nih.gov/gene/?term=52552
Fancf	Fancf	Fanconi anemia, complementation group F	100040608	https://www.ncbi.nlm.nih.gov/gene/?term=100040
Gucy1a2	Gucy1a2	guanylate cyclase 1, soluble, alpha 2	234889	https://www.ncbi.nlm.nih.gov/gene/?term=234889
Tead3	Tead3	TEA domain family member 3	21678	https://www.ncbi.nlm.nih.gov/gene/?term=21678

Nop58	Nop58	NOP58 ribonucleoprotein	55989	https://www.ncbi.nlm.nih.gov/gene/?term=55989
Plk4	Plk4	polo like kinase 4	20873	https://www.ncbi.nlm.nih.gov/gene/?term=20873
Fezf1	Fezf1	Fez family zinc finger 1	73191	https://www.ncbi.nlm.nih.gov/gene/?term=73191
Hoxb3	Hoxb3	homeobox B3	15410	https://www.ncbi.nlm.nih.gov/gene/?term=15410
Csf3r	Csf3r	colony stimulating factor 3 receptor (granulocyte)	12986	https://www.ncbi.nlm.nih.gov/gene/?term=12986
Prim1	Prim1	DNA primase, p49 subunit	19075	https://www.ncbi.nlm.nih.gov/gene/?term=19075
Suv39h2	Suv39h2	suppressor of variegation 3-9 2	64707	https://www.ncbi.nlm.nih.gov/gene/?term=64707
Dlg3	Dlg3	discs large MAGUK scaffold protein 3	53310	https://www.ncbi.nlm.nih.gov/gene/?term=53310
Plat	Plat	plasminogen activator, tissue	18791	https://www.ncbi.nlm.nih.gov/gene/?term=18791

Asb3	Asb3	ankyrin repeat and SOCS box-containing 3	65257	https://www.ncbi.nlm.nih.gov/gene/?term=65257
Spag7	Spag7	sperm associated antigen 7	216873	https://www.ncbi.nlm.nih.gov/gene/?term=216873
Setd2	Setd2	SET domain containing 2	235626	https://www.ncbi.nlm.nih.gov/gene/?term=235626
Spata7	Spata7	spermatogenesis associated 7	104871	https://www.ncbi.nlm.nih.gov/gene/?term=104871
Snx17	Snx17	sorting nexin 17	266781	https://www.ncbi.nlm.nih.gov/gene/?term=266781
P4ha1	P4ha1	procollagen-proline, 2-oxoglutarate 4-dioxygenase (proline 4-hydroxylase), alpha 1 polypeptide	18451	https://www.ncbi.nlm.nih.gov/gene/?term=18451
Slc41a1	Slc41a1	solute carrier family 41, member 1	98396	https://www.ncbi.nlm.nih.gov/gene/?term=98396
Prdm15	Prdm15	PR domain containing 15	114604	https://www.ncbi.nlm.nih.gov/gene/?term=114604
Pkm	Pkm	pyruvate kinase, muscle	18746	https://www.ncbi.nlm.nih.gov/gene/?term=18746

Biology Open • Supplementary information

Micu3	Micu3	mitochondrial calcium uptake family, member 3	78506	https://www.ncbi.nlm.nih.gov/gene/?term=78506
Gys1	Gys1	glycogen synthase 1, muscle	14936	https://www.ncbi.nlm.nih.gov/gene/?term=14936
Aldoa	Aldoa	aldolase A, fructose-bisphosphate	11674	https://www.ncbi.nlm.nih.gov/gene/?term=11674
Arfip2	Arfip2	ADP-ribosylation factor interacting protein 2	76932	https://www.ncbi.nlm.nih.gov/gene/?term=76932
Rfpl4	Rfpl4	ret finger protein-like 4	192658	https://www.ncbi.nlm.nih.gov/gene/?term=192658
Slc25a34	Slc25a34	solute carrier family 25, member 34	384071	https://www.ncbi.nlm.nih.gov/gene/?term=384071
Txnrd3	Txnrd3	thioredoxin reductase 3	232223	https://www.ncbi.nlm.nih.gov/gene/?term=232223
Gdf6	Gdf6	growth differentiation factor 6	242316	https://www.ncbi.nlm.nih.gov/gene/?term=242316
Cntn5	Cntn5	contactin 5	244682	https://www.ncbi.nlm.nih.gov/gene/?term=244682

Biology Open • Supplementary information

Radil	Radil	Ras association and DIL domains	231858	https://www.ncbi.nlm.nih.gov/gene/?term=231858
Mtmr7	Mtmr7	myotubularin related protein 7	54384	https://www.ncbi.nlm.nih.gov/gene/?term=54384
Rpusd4	Rpusd4	RNA pseudouridylate synthase domain containing 4	71989	https://www.ncbi.nlm.nih.gov/gene/?term=71989
Slc7a3	Slc7a3	solute carrier family 7 (cationic amino acid transporter, y+ system), member 3	11989	https://www.ncbi.nlm.nih.gov/gene/?term=11989
Nr1h5	Nr1h5	nuclear receptor subfamily 1, group H, member 5	381463	https://www.ncbi.nlm.nih.gov/gene/?term=381463
Hoxc10	Hoxc10	homeobox C10	209448	https://www.ncbi.nlm.nih.gov/gene/?term=209448
Irak2	Irak2	interleukin-1 receptor-associated kinase 2	108960	https://www.ncbi.nlm.nih.gov/gene/?term=108960
Ccdc7a	Ccdc7a	coiled-coil domain containing 7A	74703	https://www.ncbi.nlm.nih.gov/gene/?term=74703
Ing5	Ing5	inhibitor of growth family, member 5	66262	https://www.ncbi.nlm.nih.gov/gene/?term=66262

Biology Open • Supplementary information

Gba2	Gba2	glucosidase beta 2	230101	https://www.ncbi.nlm.nih.gov/gene/?term=230101
Kit	Kit	KIT proto-oncogene receptor tyrosine kinase	16590	https://www.ncbi.nlm.nih.gov/gene/?term=16590
Otud6b	Otud6b	OTU domain containing 6B	72201	https://www.ncbi.nlm.nih.gov/gene/?term=72201
Plaur	Plaur	plasminogen activator, urokinase receptor	18793	https://www.ncbi.nlm.nih.gov/gene/?term=18793
Mybpc1	Mybpc1	myosin binding protein C, slow-type	109272	https://www.ncbi.nlm.nih.gov/gene/?term=109272
Tex2	Tex2	testis expressed gene 2	21763	https://www.ncbi.nlm.nih.gov/gene/?term=21763
Fam83d	Fam83d	family with sequence similarity 83, member D	71878	https://www.ncbi.nlm.nih.gov/gene/?term=71878
Ankrd40	Ankrd40	ankyrin repeat domain 40	71452	https://www.ncbi.nlm.nih.gov/gene/?term=71452
Col6a3	Col6a3	collagen, type VI, alpha 3	12835	https://www.ncbi.nlm.nih.gov/gene/?term=12835

Biology Open • Supplementary information

Tgm4	Tgm4	transglutaminase 4 (prostate)	331046	https://www.ncbi.nlm.nih.gov/gene/?term=331046
Pja1	Pja1	praja ring finger ubiquitin ligase 1	18744	https://www.ncbi.nlm.nih.gov/gene/?term=18744
Ccnd3	Ccnd3	cyclin D3	12445	https://www.ncbi.nlm.nih.gov/gene/?term=12445
Isg20	Isg20	interferon-stimulated protein	57444	https://www.ncbi.nlm.nih.gov/gene/?term=57444
Tmem215	Tmem215	transmembrane protein 215	320500	https://www.ncbi.nlm.nih.gov/gene/?term=320500
Etv5	Etv5	ets variant 5	104156	https://www.ncbi.nlm.nih.gov/gene/?term=104156
Irx2	Irx2	Iroquois homeobox 2	16372	https://www.ncbi.nlm.nih.gov/gene/?term=16372
Jade1	Jade1	jade family PHD finger 1	269424	https://www.ncbi.nlm.nih.gov/gene/?term=269424
Krt42	Krt42	keratin 42	68239	https://www.ncbi.nlm.nih.gov/gene/?term=68239

Biology Open • Supplementary information

Kcnb2	Kcnb2	potassium voltage gated channel, Shab-related subfamily, member 2	98741	https://www.ncbi.nlm.nih.gov/gene/?term=98741
Bbof1	Bbof1	basal body orientation factor 1	72873	https://www.ncbi.nlm.nih.gov/gene/?term=72873
Tmem231	Tmem231	transmembrane protein 231	234740	https://www.ncbi.nlm.nih.gov/gene/?term=234740
Il22ra2	Il22ra2	interleukin 22 receptor, alpha 2	237310	https://www.ncbi.nlm.nih.gov/gene/?term=237310
Saal1	Saal1	serum amyloid A-like 1	78935	https://www.ncbi.nlm.nih.gov/gene/?term=78935
Mpeg1	Mpeg1	macrophage expressed gene 1	17476	https://www.ncbi.nlm.nih.gov/gene/?term=17476
Bbx	Bbx	bobby sox HMG box containing	70508	https://www.ncbi.nlm.nih.gov/gene/?term=70508
Rap2a	Rap2a	RAS related protein 2a	76108	https://www.ncbi.nlm.nih.gov/gene/?term=76108
Adat1	Adat1	adenosine deaminase, tRNA-specific 1	30947	https://www.ncbi.nlm.nih.gov/gene/?term=30947

Biology Open • Supplementary information

Nsmce3	Nsmce3	NSE3 homolog, SMC5-SMC6 complex component	66647	https://www.ncbi.nlm.nih.gov/gene/?term=66647
Eif3j1	Eif3j1	eukaryotic translation initiation factor 3, subunit J1	78655	https://www.ncbi.nlm.nih.gov/gene/?term=78655
Miga1	Miga1	mitoguardin 1	215708	https://www.ncbi.nlm.nih.gov/gene/?term=215708
Ndnf	Ndnf	neuron-derived neurotrophic factor	68169	https://www.ncbi.nlm.nih.gov/gene/?term=68169
Myo5b	Myo5b	myosin VB	17919	https://www.ncbi.nlm.nih.gov/gene/?term=17919
Rnf126	Rnf126	ring finger protein 126	70294	https://www.ncbi.nlm.nih.gov/gene/?term=70294
Abca5	Abca5	ATP-binding cassette, sub-family A (ABC1), member 5	217265	https://www.ncbi.nlm.nih.gov/gene/?term=217265
Dpysl5	Dpysl5	dihydropyrimidinase-like 5	65254	https://www.ncbi.nlm.nih.gov/gene/?term=65254
Itm2c	Itm2c	integral membrane protein 2C	64294	https://www.ncbi.nlm.nih.gov/gene/?term=64294

Abhd17c	Abhd17c	abhydrolase domain containing 17C	70178	https://www.ncbi.nlm.nih.gov/gene/?term=70178
Lifr	Lifr	LIF receptor alpha	16880	https://www.ncbi.nlm.nih.gov/gene/?term=16880
Eif3j2	Eif3j2	eukaryotic translation initiation factor 3, subunit J2	100042807	https://www.ncbi.nlm.nih.gov/gene/?term=100042
Sybu	Sybu	syntabulin (syntaxin-interacting)	319613	https://www.ncbi.nlm.nih.gov/gene/?term=319613
Ptk2b	Ptk2b	PTK2 protein tyrosine kinase 2 beta	19229	https://www.ncbi.nlm.nih.gov/gene/?term=19229
Nkrf	Nkrf	NF-kappaB repressing factor	77286	https://www.ncbi.nlm.nih.gov/gene/?term=77286
Gigyf2	Gigyf2	GRB10 interacting GYF protein 2	227331	https://www.ncbi.nlm.nih.gov/gene/?term=227331
BC053393	BC053393	cDNA sequence BC053393	407814	https://www.ncbi.nlm.nih.gov/gene/?term=407814
Ebf2	Ebf2	early B cell factor 2	13592	https://www.ncbi.nlm.nih.gov/gene/?term=13592

Biology Open • Supplementary information

Atoh1	Atoh1	atonal bHLH transcription factor 1	11921	https://www.ncbi.nlm.nih.gov/gene/?term=11921
Gipc1	Gipc1	GIPC PDZ domain containing family, member 1	67903	https://www.ncbi.nlm.nih.gov/gene/?term=67903
Kctd6	Kctd6	potassium channel tetramerisation domain containing 6	71393	https://www.ncbi.nlm.nih.gov/gene/?term=71393
Hes1	Hes1	hes family bHLH transcription factor 1	15205	https://www.ncbi.nlm.nih.gov/gene/?term=15205
Zbtb8a	Zbtb8a	zinc finger and BTB domain containing 8a	73680	https://www.ncbi.nlm.nih.gov/gene/?term=73680
St3gal6	St3gal6	ST3 beta-galactoside alpha-2,3-sialyltransferase 6	54613	https://www.ncbi.nlm.nih.gov/gene/?term=54613
Aurkaip1	Aurkaip1	aurora kinase A interacting protein 1	66077	https://www.ncbi.nlm.nih.gov/gene/?term=66077
Stx17	Stx17	syntaxin 17	67727	https://www.ncbi.nlm.nih.gov/gene/?term=67727
Zc3h13	Zc3h13	zinc finger CCCH type containing 13	67302	https://www.ncbi.nlm.nih.gov/gene/?term=67302

Gm11487	Gm11487	predicted gene 11487	433719	https://www.ncbi.nlm.nih.gov/gene/?term=433719
Pcm1	Pcm1	pericentriolar material 1	18536	https://www.ncbi.nlm.nih.gov/gene/?term=18536
Caml	Caml	calcium modulating ligand	12328	https://www.ncbi.nlm.nih.gov/gene/?term=12328
Noxo1	Noxo1	NADPH oxidase organizer 1	71893	https://www.ncbi.nlm.nih.gov/gene/?term=71893
Abcc5	Abcc5	ATP-binding cassette, sub-family C (CFTR/MRP), member 5	27416	https://www.ncbi.nlm.nih.gov/gene/?term=27416
D230025D16Rik	D230025D16Rik	RIKEN cDNA D230025D16 gene	234678	https://www.ncbi.nlm.nih.gov/gene/?term=234678
Eda	Eda	ectodysplasin-A	13607	https://www.ncbi.nlm.nih.gov/gene/?term=13607
Ddx6	Ddx6	DEAD (Asp-Glu-Ala-Asp) box polypeptide 6	13209	https://www.ncbi.nlm.nih.gov/gene/?term=13209
Pparg	Pparg	peroxisome proliferator activated receptor gamma	19016	https://www.ncbi.nlm.nih.gov/gene/?term=19016

Biology Open • Supplementary information

Smarcd2	Smarcd2	SWI/SNF related, matrix associated, actin dependent regulator of chromatin, subfamily d, member 2	83796	https://www.ncbi.nlm.nih.gov/gene/?term=83796
Slmap	Slmap	sarcolemma associated protein	83997	https://www.ncbi.nlm.nih.gov/gene/?term=83997
Hprt	Hprt	hypoxanthine guanine phosphoribosyl transferase	15452	https://www.ncbi.nlm.nih.gov/gene/?term=15452
Mllt6	Mllt6	myeloid/lymphoid or mixed-lineage leukemia; translocated to, 6	246198	https://www.ncbi.nlm.nih.gov/gene/?term=246198
Wnt2b	Wnt2b	wingless-type MMTV integration site family, member 2B	22414	https://www.ncbi.nlm.nih.gov/gene/?term=22414
Spg20	Spg20	spastic paraplegia 20, spartin (Troyer syndrome) homolog (human)	229285	https://www.ncbi.nlm.nih.gov/gene/?term=229285
Enpp5	Enpp5	ectonucleotide pyrophosphatase/phosphodiesterase 5	83965	https://www.ncbi.nlm.nih.gov/gene/?term=83965
Usp8	Usp8	ubiquitin specific peptidase 8	84092	https://www.ncbi.nlm.nih.gov/gene/?term=84092
Sh3d19	Sh3d19	SH3 domain protein D19	27059	https://www.ncbi.nlm.nih.gov/gene/?term=27059

Biology Open • Supplementary information

Lonrf3	Lonrf3	LON peptidase N-terminal domain and ring finger 3	74365	https://www.ncbi.nlm.nih.gov/gene/?term=74365
Akap11	Akap11	A kinase (PRKA) anchor protein 11	219181	https://www.ncbi.nlm.nih.gov/gene/?term=219181
Mid1ip1	Mid1ip1	Mid1 interacting protein 1 (gastrulation specific G12-like (zebrafish))	68041	https://www.ncbi.nlm.nih.gov/gene/?term=68041
Mdm4	Mdm4	transformed mouse 3T3 cell double minute 4	17248	https://www.ncbi.nlm.nih.gov/gene/?term=17248
Tbc1d8	Tbc1d8	TBC1 domain family, member 8	54610	https://www.ncbi.nlm.nih.gov/gene/?term=54610
Acvr1	Acvr1	activin A receptor, type 1	11477	https://www.ncbi.nlm.nih.gov/gene/?term=11477
Sel1l3	Sel1l3	sel-1 suppressor of lin-12-like 3 (C. elegans)	231238	https://www.ncbi.nlm.nih.gov/gene/?term=231238
Memo1	Memo1	mediator of cell motility 1	76890	https://www.ncbi.nlm.nih.gov/gene/?term=76890
Ston2	Ston2	stonin 2	108800	https://www.ncbi.nlm.nih.gov/gene/?term=108800

Utp18	Utp18	UTP18 small subunit processome component	217109	https://www.ncbi.nlm.nih.gov/gene/?term=217109
Pag1	Pag1	phosphoprotein associated with glycosphingolipid microdomains 1	94212	https://www.ncbi.nlm.nih.gov/gene/?term=94212
Asb10	Asb10	ankyrin repeat and SOCS box-containing 10	117590	https://www.ncbi.nlm.nih.gov/gene/?term=117590
Hsdl1	Hsdl1	hydroxysteroid dehydrogenase like 1	72552	https://www.ncbi.nlm.nih.gov/gene/?term=72552
Arfgef2	Arfgef2	ADP-ribosylation factor guanine nucleotide-exchange factor 2 (brefeldin A-inhibited)	99371	https://www.ncbi.nlm.nih.gov/gene/?term=99371
Hoxb7	Hoxb7	homeobox B7	15415	https://www.ncbi.nlm.nih.gov/gene/?term=15415
Rfx6	Rfx6	regulatory factor X, 6	320995	https://www.ncbi.nlm.nih.gov/gene/?term=320995
Nuf2	Nuf2	NUF2, NDC80 kinetochore complex component	66977	https://www.ncbi.nlm.nih.gov/gene/?term=66977
Syf2	Syf2	SYF2 homolog, RNA splicing factor (S. cerevisiae)	68592	https://www.ncbi.nlm.nih.gov/gene/?term=68592

Biology Open • Supplementary information

Ccdc82	Ccdc82	coiled-coil domain containing 82	66396	https://www.ncbi.nlm.nih.gov/gene/?term=66396
Iqub	Iqub	IQ motif and ubiquitin domain containing	214704	https://www.ncbi.nlm.nih.gov/gene/?term=214704
Piga	Piga	phosphatidylinositol glycan anchor biosynthesis, class A	18700	https://www.ncbi.nlm.nih.gov/gene/?term=18700
Cnih2	Cnih2	cornichon family AMPA receptor auxiliary protein 2	12794	https://www.ncbi.nlm.nih.gov/gene/?term=12794
Ncapg	Ncapg	non-SMC condensin I complex, subunit G	54392	https://www.ncbi.nlm.nih.gov/gene/?term=54392
Uba5	Uba5	ubiquitin-like modifier activating enzyme 5	66663	https://www.ncbi.nlm.nih.gov/gene/?term=66663
Cacnb2	Cacnb2	calcium channel, voltage-dependent, beta 2 subunit	12296	https://www.ncbi.nlm.nih.gov/gene/?term=12296
Tspan2	Tspan2	tetraspanin 2	70747	https://www.ncbi.nlm.nih.gov/gene/?term=70747
Prok2	Prok2	prokineticin 2	50501	https://www.ncbi.nlm.nih.gov/gene/?term=50501

Vkorc1	Vkorc1	vitamin K epoxide reductase complex, subunit 1	27973	https://www.ncbi.nlm.nih.gov/gene/?term=27973
En2	En2	engrailed 2	13799	https://www.ncbi.nlm.nih.gov/gene/?term=13799
Adssl1	Adssl1	adenylosuccinate synthetase like 1	11565	https://www.ncbi.nlm.nih.gov/gene/?term=11565
Ifih1	Ifih1	interferon induced with helicase C domain 1	71586	https://www.ncbi.nlm.nih.gov/gene/?term=71586
Mcur1	Mcur1	mitochondrial calcium uniporter regulator 1	76137	https://www.ncbi.nlm.nih.gov/gene/?term=76137
Prps2	Prps2	phosphoribosyl pyrophosphate synthetase 2	110639	https://www.ncbi.nlm.nih.gov/gene/?term=110639
Ercc6l	Ercc6l	excision repair cross-complementing rodent repair deficiency complementation group 6 like	236930	https://www.ncbi.nlm.nih.gov/gene/?term=236930
Acer3	Acer3	alkaline ceramidase 3	66190	https://www.ncbi.nlm.nih.gov/gene/?term=66190
Sohlh2	Sohlh2	spermatogenesis and oogenesis specific basic helix-loop-helix 2	74434	https://www.ncbi.nlm.nih.gov/gene/?term=74434

Biology Open • Supplementary information

Sox8	Sox8	SRY (sex determining region Y)-box 8	20681	https://www.ncbi.nlm.nih.gov/gene/?term=20681
Ptprc	Ptprc	protein tyrosine phosphatase, receptor type, C	19264	https://www.ncbi.nlm.nih.gov/gene/?term=19264
Gpatch1	Gpatch1	G patch domain containing 1	67471	https://www.ncbi.nlm.nih.gov/gene/?term=67471
Ptk2	Ptk2	PTK2 protein tyrosine kinase 2	14083	https://www.ncbi.nlm.nih.gov/gene/?term=14083
Rapgef6	Rapgef6	Rap guanine nucleotide exchange factor (GEF) 6	192786	https://www.ncbi.nlm.nih.gov/gene/?term=192786
Nap1l4	Nap1l4	nucleosome assembly protein 1-like 4	17955	https://www.ncbi.nlm.nih.gov/gene/?term=17955
Tubb4b	Tubb4b	tubulin, beta 4B class IVB	227613	https://www.ncbi.nlm.nih.gov/gene/?term=227613
Csmd2	Csmd2	CUB and Sushi multiple domains 2	329942	https://www.ncbi.nlm.nih.gov/gene/?term=329942
Sdc3	Sdc3	syndecan 3	20970	https://www.ncbi.nlm.nih.gov/gene/?term=20970

Biology Open • Supplementary information

Leo1	Leo1	Leo1, Paf1/RNA polymerase II complex component	235497	https://www.ncbi.nlm.nih.gov/gene/?term=235497
Tpm1	Tpm1	tropomyosin 1, alpha	22003	https://www.ncbi.nlm.nih.gov/gene/?term=22003
Kdm5b	Kdm5b	lysine (K)-specific demethylase 5B	75605	https://www.ncbi.nlm.nih.gov/gene/?term=75605
Zfp385b	Zfp385b	zinc finger protein 385B	241494	https://www.ncbi.nlm.nih.gov/gene/?term=241494
Taf6	Taf6	TATA-box binding protein associated factor 6	21343	https://www.ncbi.nlm.nih.gov/gene/?term=21343
Clk4	Clk4	CDC like kinase 4	12750	https://www.ncbi.nlm.nih.gov/gene/?term=12750
Skil	Skil	SKI-like	20482	https://www.ncbi.nlm.nih.gov/gene/?term=20482
Ppip5k1	Ppip5k1	diphosphoinositol pentakisphosphate kinase 1	327655	https://www.ncbi.nlm.nih.gov/gene/?term=327655
Apc	Apc	APC, WNT signaling pathway regulator	11789	https://www.ncbi.nlm.nih.gov/gene/?term=11789

Biology Open • Supplementary information

Lrrtm3	Lrrtm3	leucine rich repeat transmembrane neuronal 3	216028	https://www.ncbi.nlm.nih.gov/gene/?term=216028
Cxcr4	Cxcr4	chemokine (C-X-C motif) receptor 4	12767	https://www.ncbi.nlm.nih.gov/gene/?term=12767
Tshz1	Tshz1	teashirt zinc finger family member 1	110796	https://www.ncbi.nlm.nih.gov/gene/?term=110796
Cemip2	Cemip2	cell migration inducing hyaluronidase 2	83921	https://www.ncbi.nlm.nih.gov/gene/?term=83921
Arhgef7	Arhgef7	Rho guanine nucleotide exchange factor (GEF7)	54126	https://www.ncbi.nlm.nih.gov/gene/?term=54126
Brd8	Brd8	bromodomain containing 8	78656	https://www.ncbi.nlm.nih.gov/gene/?term=78656
Ormdl3	Ormdl3	ORM1-like 3 (S. cerevisiae)	66612	https://www.ncbi.nlm.nih.gov/gene/?term=66612
Ppp2ca	Ppp2ca	protein phosphatase 2 (formerly 2A), catalytic subunit, alpha isoform	19052	https://www.ncbi.nlm.nih.gov/gene/?term=19052
Fat1	Fat1	FAT atypical cadherin 1	14107	https://www.ncbi.nlm.nih.gov/gene/?term=14107

Mill1	Mill1	MHC I like leukocyte 1	266815	https://www.ncbi.nlm.nih.gov/gene/?term=266815
Wdr90	Wdr90	WD repeat domain 90	106618	https://www.ncbi.nlm.nih.gov/gene/?term=106618
Snx25	Snx25	sorting nexin 25	102141	https://www.ncbi.nlm.nih.gov/gene/?term=102141
Fzd7	Fzd7	frizzled class receptor 7	14369	https://www.ncbi.nlm.nih.gov/gene/?term=14369
Tm7sf3	Tm7sf3	transmembrane 7 superfamily member 3	67623	https://www.ncbi.nlm.nih.gov/gene/?term=67623
Hmgcs1	Hmgcs1	3-hydroxy-3-methylglutaryl-Coenzyme A synthase 1	208715	https://www.ncbi.nlm.nih.gov/gene/?term=208715
Rbm5	Rbm5	RNA binding motif protein 5	83486	https://www.ncbi.nlm.nih.gov/gene/?term=83486
Neo1	Neo1	neogenin	18007	https://www.ncbi.nlm.nih.gov/gene/?term=18007
Ankrd12	Ankrd12	ankyrin repeat domain 12	106585	https://www.ncbi.nlm.nih.gov/gene/?term=106585

Biology Open • Supplementary information

Derl1	Derl1	Der1-like domain family, member 1	67819	https://www.ncbi.nlm.nih.gov/gene/?term=67819
Ksr2	Ksr2	kinase suppressor of ras 2	333050	https://www.ncbi.nlm.nih.gov/gene/?term=333050
Snrpd1	Snrpd1	small nuclear ribonucleoprotein D1	20641	https://www.ncbi.nlm.nih.gov/gene/?term=20641
Rnps1	Rnps1	ribonucleic acid binding protein S1	19826	https://www.ncbi.nlm.nih.gov/gene/?term=19826
Prrc2c	Prrc2c	proline-rich coiled-coil 2C	226562	https://www.ncbi.nlm.nih.gov/gene/?term=226562
Nrxn3	Nrxn3	neurexin III	18191	https://www.ncbi.nlm.nih.gov/gene/?term=18191
Prex1	Prex1	phosphatidylinositol-3,4,5-trisphosphate-dependent Rac exchange factor 1	277360	https://www.ncbi.nlm.nih.gov/gene/?term=277360
Kdelr1	Kdelr1	KDEL (Lys-Asp-Glu-Leu) endoplasmic reticulum protein retention receptor 1	68137	https://www.ncbi.nlm.nih.gov/gene/?term=68137
Rab40c	Rab40c	Rab40C, member RAS oncogene family	224624	https://www.ncbi.nlm.nih.gov/gene/?term=224624

Biology Open • Supplementary information

Atp5a1	Atp5a1	ATP synthase, H+ transporting, mitochondrial F1 complex, alpha subunit 1	11946	https://www.ncbi.nlm.nih.gov/gene/?term=11946
Rab14	Rab14	RAB14, member RAS oncogene family	68365	https://www.ncbi.nlm.nih.gov/gene/?term=68365
Il1r1	Il1r1	interleukin 1 receptor, type I	16177	https://www.ncbi.nlm.nih.gov/gene/?term=16177
Taf5	Taf5	TATA-box binding protein associated factor 5	226182	https://www.ncbi.nlm.nih.gov/gene/?term=226182
Sox2	Sox2	SRY (sex determining region Y)-box 2	20674	https://www.ncbi.nlm.nih.gov/gene/?term=20674
Rab19	Rab19	RAB19, member RAS oncogene family	19331	https://www.ncbi.nlm.nih.gov/gene/?term=19331
Pdgfc	Pdgfc	platelet-derived growth factor, C polypeptide	54635	https://www.ncbi.nlm.nih.gov/gene/?term=54635
Gmcl1	Gmcl1	germ cell-less, spermatogenesis associated 1	23885	https://www.ncbi.nlm.nih.gov/gene/?term=23885
Lrp5	Lrp5	low density lipoprotein receptor-related protein 5	16973	https://www.ncbi.nlm.nih.gov/gene/?term=16973

Biology Open • Supplementary information

Atp6v1g1	Atp6v1g1	ATPase, H+ transporting, lysosomal V1 subunit G1	66290	https://www.ncbi.nlm.nih.gov/gene/?term=66290
Srebf1	Srebf1	sterol regulatory element binding transcription factor 1	20787	https://www.ncbi.nlm.nih.gov/gene/?term=20787
Kif13a	Kif13a	kinesin family member 13A	16553	https://www.ncbi.nlm.nih.gov/gene/?term=16553
Lrig1	Lrig1	leucine-rich repeats and immunoglobulin-like domains 1	16206	https://www.ncbi.nlm.nih.gov/gene/?term=16206
Dennd4a	Dennd4a	DENN/MADD domain containing 4A	102442	https://www.ncbi.nlm.nih.gov/gene/?term=102442
Slco2b1	Slco2b1	solute carrier organic anion transporter family, member 2b1	101488	https://www.ncbi.nlm.nih.gov/gene/?term=101488
Fntb	Fntb	farnesyltransferase, CAAX box, beta	110606	https://www.ncbi.nlm.nih.gov/gene/?term=110606
Kcnk6	Kcnk6	potassium inwardly-rectifying channel, subfamily K, member 6	52150	https://www.ncbi.nlm.nih.gov/gene/?term=52150
Bid	Bid	BH3 interacting domain death agonist	12122	https://www.ncbi.nlm.nih.gov/gene/?term=12122

Morn4	Morn4	MORN repeat containing 4	226123	https://www.ncbi.nlm.nih.gov/gene/?term=226123
Dagla	Dagla	diacylglycerol lipase, alpha	269060	https://www.ncbi.nlm.nih.gov/gene/?term=269060
Usb1	Usb1	U6 snRNA biogenesis 1	101985	https://www.ncbi.nlm.nih.gov/gene/?term=101985
Nfic	Nfic	nuclear factor I/C	18029	https://www.ncbi.nlm.nih.gov/gene/?term=18029
Pcdh15	Pcdh15	protocadherin 15	11994	https://www.ncbi.nlm.nih.gov/gene/?term=11994
Rapgef1	Rapgef1	Rap guanine nucleotide exchange factor (GEF) 1	107746	https://www.ncbi.nlm.nih.gov/gene/?term=107746
Kcne4	Kcne4	potassium voltage-gated channel, Isk-related subfamily, gene 4	57814	https://www.ncbi.nlm.nih.gov/gene/?term=57814
Zfp775	Zfp775	zinc finger protein 775	243372	https://www.ncbi.nlm.nih.gov/gene/?term=243372
Diaph1	Diaph1	diaphanous related formin 1	13367	https://www.ncbi.nlm.nih.gov/gene/?term=13367

Biology Open • Supplementary information

Dmpk	Dmpk	dystrophia myotonica-protein kinase	13400	https://www.ncbi.nlm.nih.gov/gene/?term=13400
Fezf2	Fezf2	Fez family zinc finger 2	54713	https://www.ncbi.nlm.nih.gov/gene/?term=54713
Tpcn1	Tpcn1	two pore channel 1	252972	https://www.ncbi.nlm.nih.gov/gene/?term=252972
Llgl1	Llgl1	LLGL1 scribble cell polarity complex component	16897	https://www.ncbi.nlm.nih.gov/gene/?term=16897
Nfix	Nfix	nuclear factor I/X	18032	https://www.ncbi.nlm.nih.gov/gene/?term=18032
Kcnk3	Kcnk3	potassium channel, subfamily K, member 3	16527	https://www.ncbi.nlm.nih.gov/gene/?term=16527
Ucp2	Ucp2	uncoupling protein 2 (mitochondrial, proton carrier)	22228	https://www.ncbi.nlm.nih.gov/gene/?term=22228
Celf5	Celf5	CUGBP, Elav-like family member 5	319586	https://www.ncbi.nlm.nih.gov/gene/?term=319586
Flna	Flna	filamin, alpha	192176	https://www.ncbi.nlm.nih.gov/gene/?term=192176

Biology Open • Supplementary information

Epsti1	Epsti1	epithelial stromal interaction 1 (breast)	108670	https://www.ncbi.nlm.nih.gov/gene/?term=108670
Cd247	Cd247	CD247 antigen	12503	https://www.ncbi.nlm.nih.gov/gene/?term=12503
Pacs1	Pacs1	phosphofurin acidic cluster sorting protein 1	107975	https://www.ncbi.nlm.nih.gov/gene/?term=107975
Sh3gl1	Sh3gl1	SH3-domain GRB2-like 1	20405	https://www.ncbi.nlm.nih.gov/gene/?term=20405
Kpna6	Kpna6	karyopherin (importin) alpha 6	16650	https://www.ncbi.nlm.nih.gov/gene/?term=16650
Pmp22	Pmp22	peripheral myelin protein 22	18858	https://www.ncbi.nlm.nih.gov/gene/?term=18858
Pdc	Pdc	phosducin	20028	https://www.ncbi.nlm.nih.gov/gene/?term=20028
Arhgap44	Arhgap44	Rho GTPase activating protein 44	216831	https://www.ncbi.nlm.nih.gov/gene/?term=216831
Svip	Svip	small VCP/p97-interacting protein	75744	https://www.ncbi.nlm.nih.gov/gene/?term=75744

Rhot1	Rhot1	ras homolog family member T1	59040	https://www.ncbi.nlm.nih.gov/gene/?term=59040
Ankrd50	Ankrd50	ankyrin repeat domain 50	99696	https://www.ncbi.nlm.nih.gov/gene/?term=99696
Syt4	Syt4	synaptotagmin IV	20983	https://www.ncbi.nlm.nih.gov/gene/?term=20983
Hipk1	Hipk1	homeodomain interacting protein kinase 1	15257	https://www.ncbi.nlm.nih.gov/gene/?term=15257
Mef2c	Mef2c	myocyte enhancer factor 2C	17260	https://www.ncbi.nlm.nih.gov/gene/?term=17260
Cdh13	Cdh13	cadherin 13	12554	https://www.ncbi.nlm.nih.gov/gene/?term=12554
Basp1	Basp1	brain abundant, membrane attached signal protein 1	70350	https://www.ncbi.nlm.nih.gov/gene/?term=70350
Scaf11	Scaf11	SR-related CTD-associated factor 11	72193	https://www.ncbi.nlm.nih.gov/gene/?term=72193
Zfp384	Zfp384	zinc finger protein 384	269800	https://www.ncbi.nlm.nih.gov/gene/?term=269800

Biology Open • Supplementary information

Gtf2ird1	Gtf2ird1	general transcription factor II I repeat domain-containing 1	57080	https://www.ncbi.nlm.nih.gov/gene/?term=57080
Pard6b	Pard6b	par-6 family cell polarity regulator beta	58220	https://www.ncbi.nlm.nih.gov/gene/?term=58220
Dennd4c	Dennd4c	DENN/MADD domain containing 4C	329877	https://www.ncbi.nlm.nih.gov/gene/?term=329877
Snrpb2	Snrpb2	U2 small nuclear ribonucleoprotein B	20639	https://www.ncbi.nlm.nih.gov/gene/?term=20639
Apoo	Apoo	apolipoprotein O	68316	https://www.ncbi.nlm.nih.gov/gene/?term=68316
Fam24b	Fam24b	family with sequence similarity 24 member B	69318	https://www.ncbi.nlm.nih.gov/gene/?term=69318
Ube2cbp	Ube2cbp	ubiquitin-conjugating enzyme E2C binding protein	70348	https://www.ncbi.nlm.nih.gov/gene/?term=70348
Gdnf	Gdnf	glial cell line derived neurotrophic factor	14573	https://www.ncbi.nlm.nih.gov/gene/?term=14573
Mbd5	Mbd5	methyl-CpG binding domain protein 5	109241	https://www.ncbi.nlm.nih.gov/gene/?term=109241

Biology Open • Supplementary information

Fbxl22	Fbxl22	F-box and leucine-rich repeat protein 22	74165	https://www.ncbi.nlm.nih.gov/gene/?term=74165
Faxc	Faxc	failed axon connections homolog	76132	https://www.ncbi.nlm.nih.gov/gene/?term=76132
Rab5a	Rab5a	RAB5A, member RAS oncogene family	271457	https://www.ncbi.nlm.nih.gov/gene/?term=271457
Sat1	Sat1	spermidine/spermine N1-acetyl transferase 1	20229	https://www.ncbi.nlm.nih.gov/gene/?term=20229
9530068E07Rik	9530068E07Rik	RIKEN cDNA 9530068E07 gene	213673	https://www.ncbi.nlm.nih.gov/gene/?term=213673
Pcgf2	Pcgf2	polycomb group ring finger 2	22658	https://www.ncbi.nlm.nih.gov/gene/?term=22658
Antxr1	Antxr1	anthrax toxin receptor 1	69538	https://www.ncbi.nlm.nih.gov/gene/?term=69538
Dach1	Dach1	dachshund family transcription factor 1	13134	https://www.ncbi.nlm.nih.gov/gene/?term=13134
Cts8	Cts8	cathepsin 8	56094	https://www.ncbi.nlm.nih.gov/gene/?term=56094

Biology Open • Supplementary information

Gm8369	Gm8369	predicted gene 8369	666926	https://www.ncbi.nlm.nih.gov/gene/?term=666926
Zdhhc17	Zdhhc17	zinc finger, DHHC domain containing 17	320150	https://www.ncbi.nlm.nih.gov/gene/?term=320150
Brd7	Brd7	bromodomain containing 7	26992	https://www.ncbi.nlm.nih.gov/gene/?term=26992
Foxo1	Foxo1	forkhead box O1	56458	https://www.ncbi.nlm.nih.gov/gene/?term=56458
Lrguk	Lrguk	leucine-rich repeats and guanylate kinase domain containing	74354	https://www.ncbi.nlm.nih.gov/gene/?term=74354
Tmem106b	Tmem106b	transmembrane protein 106B	71900	https://www.ncbi.nlm.nih.gov/gene/?term=71900
Itgb1	Itgb1	integrin beta 1 (fibronectin receptor beta)	16412	https://www.ncbi.nlm.nih.gov/gene/?term=16412
Cspp1	Cspp1	centrosome and spindle pole associated protein 1	211660	https://www.ncbi.nlm.nih.gov/gene/?term=211660
Bnc1	Bnc1	basonuclin 1	12173	https://www.ncbi.nlm.nih.gov/gene/?term=12173

Biology Open • Supplementary information

Gad1	Gad1	glutamate decarboxylase 1	14415	https://www.ncbi.nlm.nih.gov/gene/?term=14415
Ahdc1	Ahdc1	AT hook, DNA binding motif, containing 1	230793	https://www.ncbi.nlm.nih.gov/gene/?term=230793
Gabra5	Gabra5	gamma-aminobutyric acid (GABA) A receptor, subunit alpha 5	110886	https://www.ncbi.nlm.nih.gov/gene/?term=110886
Faim	Faim	Fas apoptotic inhibitory molecule	23873	https://www.ncbi.nlm.nih.gov/gene/?term=23873
Axin1	Axin1	axin 1	12005	https://www.ncbi.nlm.nih.gov/gene/?term=12005
Lman1	Lman1	lectin, mannose-binding, 1	70361	https://www.ncbi.nlm.nih.gov/gene/?term=70361
Dld	Dld	dihydrolipoamide dehydrogenase	13382	https://www.ncbi.nlm.nih.gov/gene/?term=13382
Fubp1	Fubp1	far upstream element (FUSE) binding protein 1	51886	https://www.ncbi.nlm.nih.gov/gene/?term=51886
Cog3	Cog3	component of oligomeric golgi complex 3	338337	https://www.ncbi.nlm.nih.gov/gene/?term=338337

Esx1	Esx1	extraembryonic, spermatogenesis, homeobox 1	13984	https://www.ncbi.nlm.nih.gov/gene/?term=13984
Dhx36	Dhx36	DEAH (Asp-Glu-Ala-His) box polypeptide 36	72162	https://www.ncbi.nlm.nih.gov/gene/?term=72162
Ak5	Ak5	adenylate kinase 5	229949	https://www.ncbi.nlm.nih.gov/gene/?term=229949
Kif27	Kif27	kinesin family member 27	75050	https://www.ncbi.nlm.nih.gov/gene/?term=75050
Tubgcp4	Tubgcp4	tubulin, gamma complex associated protein 4	51885	https://www.ncbi.nlm.nih.gov/gene/?term=51885
Ints8	Ints8	integrator complex subunit 8	72656	https://www.ncbi.nlm.nih.gov/gene/?term=72656
Megf11	Megf11	multiple EGF-like-domains 11	214058	https://www.ncbi.nlm.nih.gov/gene/?term=214058
Ltbp3	Ltbp3	latent transforming growth factor beta binding protein 3	16998	https://www.ncbi.nlm.nih.gov/gene/?term=16998
Frg1	Frg1	FSHD region gene 1	14300	https://www.ncbi.nlm.nih.gov/gene/?term=14300

Tmx4	Tmx4	thioredoxin-related transmembrane protein 4	52837	https://www.ncbi.nlm.nih.gov/gene/?term=52837
Rpgrip1l	Rpgrip1l	Rpgrip1-like	244585	https://www.ncbi.nlm.nih.gov/gene/?term=244585
Nfkb1	Nfkb1	nuclear factor of kappa light polypeptide gene enhancer in B cells 1, p105	18033	https://www.ncbi.nlm.nih.gov/gene/?term=18033
Xrcc2	Xrcc2	X-ray repair complementing defective repair in Chinese hamster cells 2	57434	https://www.ncbi.nlm.nih.gov/gene/?term=57434
Fam124b	Fam124b	family with sequence similarity 124, member B	241128	https://www.ncbi.nlm.nih.gov/gene/?term=241128
Cycs	Cycs	cytochrome c, somatic	13063	https://www.ncbi.nlm.nih.gov/gene/?term=13063
Slc25a4	Slc25a4	solute carrier family 25 (mitochondrial carrier, adenine nucleotide translocator), member 4	11739	https://www.ncbi.nlm.nih.gov/gene/?term=11739
Slc30a9	Slc30a9	solute carrier family 30 (zinc transporter), member 9	109108	https://www.ncbi.nlm.nih.gov/gene/?term=109108
Gatad2a	Gatad2a	GATA zinc finger domain containing 2A	234366	https://www.ncbi.nlm.nih.gov/gene/?term=234366

Pibf1	Pibf1	progesterone immunomodulatory binding factor 1	52023	https://www.ncbi.nlm.nih.gov/gene/?term=52023
Myo9b	Myo9b	myosin IXb	17925	https://www.ncbi.nlm.nih.gov/gene/?term=17925
Scrib	Scrib	scribbled planar cell polarity	105782	https://www.ncbi.nlm.nih.gov/gene/?term=105782
Arhgap17	Arhgap17	Rho GTPase activating protein 17	70497	https://www.ncbi.nlm.nih.gov/gene/?term=70497
Naa35	Naa35	N(alpha)-acetyltransferase 35, NatC auxiliary subunit	78689	https://www.ncbi.nlm.nih.gov/gene/?term=78689
Lmo7	Lmo7	LIM domain only 7	380928	https://www.ncbi.nlm.nih.gov/gene/?term=380928
Ndfip2	Ndfip2	Nedd4 family interacting protein 2	76273	https://www.ncbi.nlm.nih.gov/gene/?term=76273
Slc6a15	Slc6a15	solute carrier family 6 (neurotransmitter transporter), member 15	103098	https://www.ncbi.nlm.nih.gov/gene/?term=103098
Coa3	Coa3	cytochrome C oxidase assembly factor 3	52469	https://www.ncbi.nlm.nih.gov/gene/?term=52469

Biology Open • Supplementary information

Serpind1	Serpind1	serine (or cysteine) peptidase inhibitor, clade D, member 1	15160	https://www.ncbi.nlm.nih.gov/gene/?term=15160
Pdcd6ip	Pdcd6ip	programmed cell death 6 interacting protein	18571	https://www.ncbi.nlm.nih.gov/gene/?term=18571
Agps	Agps	alkylglycerone phosphate synthase	228061	https://www.ncbi.nlm.nih.gov/gene/?term=228061
Lypla2	Lypla2	lysophospholipase 2	26394	https://www.ncbi.nlm.nih.gov/gene/?term=26394
Rbpj	Rbpj	recombination signal binding protein for immunoglobulin kappa J region	19664	https://www.ncbi.nlm.nih.gov/gene/?term=19664
T	T	brachyury, T-box transcription factor T	20997	https://www.ncbi.nlm.nih.gov/gene/?term=20997
Ephb3	Ephb3	Eph receptor B3	13845	https://www.ncbi.nlm.nih.gov/gene/?term=13845
Inpp4b	Inpp4b	inositol polyphosphate-4-phosphatase, type II	234515	https://www.ncbi.nlm.nih.gov/gene/?term=234515
Tmem258	Tmem258	transmembrane protein 258	69038	https://www.ncbi.nlm.nih.gov/gene/?term=69038

Biology Open • Supplementary information

Gdpd4	Gdpd4	glycerophosphodiester phosphodiesterase domain containing 4	233537	https://www.ncbi.nlm.nih.gov/gene/?term=233537
Srp68	Srp68	signal recognition particle 68	217337	https://www.ncbi.nlm.nih.gov/gene/?term=217337
Fam114a1	Fam114a1	family with sequence similarity 114, member A1	68303	https://www.ncbi.nlm.nih.gov/gene/?term=68303
Tceanc2	Tceanc2	transcription elongation factor A (SII) N-terminal and central domain containing 2	66526	https://www.ncbi.nlm.nih.gov/gene/?term=66526
Bcor	Bcor	BCL6 interacting corepressor	71458	https://www.ncbi.nlm.nih.gov/gene/?term=71458
AI593442	AI593442	expressed sequence AI593442	330941	https://www.ncbi.nlm.nih.gov/gene/?term=330941
Ap1g2	Ap1g2	adaptor protein complex AP-1, gamma 2 subunit	11766	https://www.ncbi.nlm.nih.gov/gene/?term=11766
Rasl11a	Rasl11a	RAS-like, family 11, member A	68895	https://www.ncbi.nlm.nih.gov/gene/?term=68895
Cox11	Cox11	cytochrome c oxidase assembly protein 11, copper chaperone	69802	https://www.ncbi.nlm.nih.gov/gene/?term=69802

Prdx1	Prdx1	peroxiredoxin 1	18477	https://www.ncbi.nlm.nih.gov/gene/?term=18477
Iqce	Iqce	IQ motif containing E	74239	https://www.ncbi.nlm.nih.gov/gene/?term=74239
Tmed6	Tmed6	transmembrane p24 trafficking protein 6	66269	https://www.ncbi.nlm.nih.gov/gene/?term=66269
Atp2c1	Atp2c1	ATPase, Ca++-sequestering	235574	https://www.ncbi.nlm.nih.gov/gene/?term=235574
Tstd2	Tstd2	thiosulfate sulfurtransferase (rhodanese)-like domain containing 2	272027	https://www.ncbi.nlm.nih.gov/gene/?term=272027
Ovca2	Ovca2	candidate tumor suppressor in ovarian cancer 2	246257	https://www.ncbi.nlm.nih.gov/gene/?term=246257
Larp1	Larp1	La ribonucleoprotein domain family, member 1	73158	https://www.ncbi.nlm.nih.gov/gene/?term=73158
Chchd3	Chchd3	coiled-coil-helix-coiled-coil-helix domain containing 3	66075	https://www.ncbi.nlm.nih.gov/gene/?term=66075
Nr2f2	Nr2f2	nuclear receptor subfamily 2, group F, member 2	11819	https://www.ncbi.nlm.nih.gov/gene/?term=11819

Biology Open • Supplementary information

Usp30	Usp30	ubiquitin specific peptidase 30	100756	https://www.ncbi.nlm.nih.gov/gene/?term=100756
Tlnrd1	Tlnrd1	talin rod domain containing 1	80889	https://www.ncbi.nlm.nih.gov/gene/?term=80889
Fam98a	Fam98a	family with sequence similarity 98, member A	72722	https://www.ncbi.nlm.nih.gov/gene/?term=72722
Ccdc62	Ccdc62	coiled-coil domain containing 62	208908	https://www.ncbi.nlm.nih.gov/gene/?term=208908
Capn3	Capn3	calpain 3	12335	https://www.ncbi.nlm.nih.gov/gene/?term=12335
Cntn4	Cntn4	contactin 4	269784	https://www.ncbi.nlm.nih.gov/gene/?term=269784
Cnr1	Cnr1	cannabinoid receptor 1 (brain)	12801	https://www.ncbi.nlm.nih.gov/gene/?term=12801
Prickle2	Prickle2	prickle planar cell polarity protein 2	243548	https://www.ncbi.nlm.nih.gov/gene/?term=243548
Rbm39	Rbm39	RNA binding motif protein 39	170791	https://www.ncbi.nlm.nih.gov/gene/?term=170791

Biology Open • Supplementary information

Nadk	Nadk	NAD kinase	192185	https://www.ncbi.nlm.nih.gov/gene/?term=192185
Shoc2	Shoc2	Shoc2, leucine rich repeat scaffold protein	56392	https://www.ncbi.nlm.nih.gov/gene/?term=56392
Rc3h2	Rc3h2	ring finger and CCCH-type zinc finger domains 2	319817	https://www.ncbi.nlm.nih.gov/gene/?term=319817
Slc26a3	Slc26a3	solute carrier family 26, member 3	13487	https://www.ncbi.nlm.nih.gov/gene/?term=13487
Asph	Asph	aspartate-beta-hydroxylase	65973	https://www.ncbi.nlm.nih.gov/gene/?term=65973
Sptbn1	Sptbn1	spectrin beta, non-erythrocytic 1	20742	https://www.ncbi.nlm.nih.gov/gene/?term=20742
Elp1	Elp1	elongator complex protein 1	230233	https://www.ncbi.nlm.nih.gov/gene/?term=230233
Zfp963	Zfp963	zinc finger protein 963	620419	https://www.ncbi.nlm.nih.gov/gene/?term=620419
Cadps	Cadps	Ca2+-dependent secretion activator	27062	https://www.ncbi.nlm.nih.gov/gene/?term=27062

Biology Open • Supplementary information

Ccdc136	Ccdc136	coiled-coil domain containing 136	232664	https://www.ncbi.nlm.nih.gov/gene/?term=232664
Enthd1	Enthd1	ENTH domain containing 1	383075	https://www.ncbi.nlm.nih.gov/gene/?term=383075
Gm1604b	Gm1604b	predicted gene 1604b	381059	https://www.ncbi.nlm.nih.gov/gene/?term=381059
Arhgap5	Arhgap5	Rho GTPase activating protein 5	11855	https://www.ncbi.nlm.nih.gov/gene/?term=11855
Bcap29	Bcap29	B cell receptor associated protein 29	12033	https://www.ncbi.nlm.nih.gov/gene/?term=12033
Pabpc4l	Pabpc4l	poly(A) binding protein, cytoplasmic 4-like	241989	https://www.ncbi.nlm.nih.gov/gene/?term=241989
Mrps14	Mrps14	mitochondrial ribosomal protein S14	64659	https://www.ncbi.nlm.nih.gov/gene/?term=64659
Phox2b	Phox2b	paired-like homeobox 2b	18935	https://www.ncbi.nlm.nih.gov/gene/?term=18935
Rbm4b	Rbm4b	RNA binding motif protein 4B	66704	https://www.ncbi.nlm.nih.gov/gene/?term=66704

Biology Open • Supplementary information

Stt3b	Stt3b	STT3, subunit of the oligosaccharyltransferase complex, homolog B (S. cerevisiae)	68292	https://www.ncbi.nlm.nih.gov/gene/?term=68292
Slc9a6	Slc9a6	solute carrier family 9 (sodium/hydrogen exchanger), member 6	236794	https://www.ncbi.nlm.nih.gov/gene/?term=236794
Dcp1a	Dcp1a	decapping mRNA 1A	75901	https://www.ncbi.nlm.nih.gov/gene/?term=75901
Gli3	Gli3	GLI-Kruppel family member GLI3	14634	https://www.ncbi.nlm.nih.gov/gene/?term=14634
Smarca5	Smarca5	SWI/SNF related, matrix associated, actin dependent regulator of chromatin, subfamily a, member 5	93762	https://www.ncbi.nlm.nih.gov/gene/?term=93762
Slc22a17	Slc22a17	solute carrier family 22 (organic cation transporter), member 17	59049	https://www.ncbi.nlm.nih.gov/gene/?term=59049
Nt5c2	Nt5c2	5'-nucleotidase, cytosolic II	76952	https://www.ncbi.nlm.nih.gov/gene/?term=76952
Spta1	Spta1	spectrin alpha, erythrocytic 1	20739	https://www.ncbi.nlm.nih.gov/gene/?term=20739
Dscaml1	Dscaml1	DS cell adhesion molecule like 1	114873	https://www.ncbi.nlm.nih.gov/gene/?term=114873

Biology Open • Supplementary information

Rbms1	Rbms1	RNA binding motif, single stranded interacting protein 1	56878	https://www.ncbi.nlm.nih.gov/gene/?term=56878
Zfp607b	Zfp607b	zinc finger protein 607B	112415	https://www.ncbi.nlm.nih.gov/gene/?term=112415
Serinc5	Serinc5	serine incorporator 5	218442	https://www.ncbi.nlm.nih.gov/gene/?term=218442
Itgb5	Itgb5	integrin beta 5	16419	https://www.ncbi.nlm.nih.gov/gene/?term=16419
Crispld2	Crispld2	cysteine-rich secretory protein LCCL domain containing 2	78892	https://www.ncbi.nlm.nih.gov/gene/?term=78892
Slc7a12	Slc7a12	solute carrier family 7 (cationic amino acid transporter, y+ system), member 12	140918	https://www.ncbi.nlm.nih.gov/gene/?term=140918
Eml1	Eml1	echinoderm microtubule associated protein like 1	68519	https://www.ncbi.nlm.nih.gov/gene/?term=68519
Zfp563	Zfp563	zinc finger protein 563	240068	https://www.ncbi.nlm.nih.gov/gene/?term=240068
Ikbkg	Ikbkg	inhibitor of kappaB kinase gamma	16151	https://www.ncbi.nlm.nih.gov/gene/?term=16151

Ralgapb	Ralgapb	Ral GTPase activating protein, beta subunit (non-catalytic)	228850	https://www.ncbi.nlm.nih.gov/gene/?term=228850
Sgcd	Sgcd	sarcoglycan, delta (dystrophin-associated glycoprotein)	24052	https://www.ncbi.nlm.nih.gov/gene/?term=24052
Gm13871	Gm13871	predicted gene 13871	667780	https://www.ncbi.nlm.nih.gov/gene/?term=667780
Ehhadh	Ehhadh	enoyl-Coenzyme A, hydratase/3-hydroxyacyl Coenzyme A dehydrogenase	74147	https://www.ncbi.nlm.nih.gov/gene/?term=74147
Gm11757	Gm11757	predicted gene 11757	623272	https://www.ncbi.nlm.nih.gov/gene/?term=623272
Oxct1	Oxct1	3-oxoacid CoA transferase 1	67041	https://www.ncbi.nlm.nih.gov/gene/?term=67041
Cobl	Cobl	cordon-bleu WH2 repeat	12808	https://www.ncbi.nlm.nih.gov/gene/?term=12808
Gm11758	Gm11758	predicted gene 11758	545637	https://www.ncbi.nlm.nih.gov/gene/?term=545637
Polr3e	Polr3e	polymerase (RNA) III (DNA directed) polypeptide E	26939	https://www.ncbi.nlm.nih.gov/gene/?term=26939

Biology Open • Supplementary information

Pde2a	Pde2a	phosphodiesterase 2A, cGMP-stimulated	207728	https://www.ncbi.nlm.nih.gov/gene/?term=207728
Kif3b	Kif3b	kinesin family member 3B	16569	https://www.ncbi.nlm.nih.gov/gene/?term=16569
Gm11236	Gm11236	predicted gene 11236	100862015	https://www.ncbi.nlm.nih.gov/gene/?term=100862
Twist1	Twist1	twist basic helix-loop-helix transcription factor 1	22160	https://www.ncbi.nlm.nih.gov/gene/?term=22160
Sox14	Sox14	SRY (sex determining region Y)-box 14	20669	https://www.ncbi.nlm.nih.gov/gene/?term=20669
Rnf165	Rnf165	ring finger protein 165	225743	https://www.ncbi.nlm.nih.gov/gene/?term=225743
Gm428	Gm428	predicted gene 428	242502	https://www.ncbi.nlm.nih.gov/gene/?term=242502
Cbln1	Cbln1	cerebellin 1 precursor protein	12404	https://www.ncbi.nlm.nih.gov/gene/?term=12404
Amz1	Amz1	archaelysin family metallopeptidase 1	231842	https://www.ncbi.nlm.nih.gov/gene/?term=231842

Mid1	Mid1	midline 1	17318	https://www.ncbi.nlm.nih.gov/gene/?term=17318
Gm38423	Gm38423	predicted gene, 38423	100502924	https://www.ncbi.nlm.nih.gov/gene/?term=100502
Gm11238	Gm11238	predicted gene 11238	100502896	https://www.ncbi.nlm.nih.gov/gene/?term=100502
Gm11237	Gm11237	predicted gene 11237	623197	https://www.ncbi.nlm.nih.gov/gene/?term=623197
Tmod3	Tmod3	tropomodulin 3	50875	https://www.ncbi.nlm.nih.gov/gene/?term=50875
Gm11239	Gm11239	predicted gene 11239	101055863	https://www.ncbi.nlm.nih.gov/gene/?term=101055
Ergic1	Ergic1	endoplasmic reticulum-golgi intermediate compartment (ERGIC) 1	67458	https://www.ncbi.nlm.nih.gov/gene/?term=67458
Cops8	Cops8	COP9 signalosome subunit 8	108679	https://www.ncbi.nlm.nih.gov/gene/?term=108679
Aff4	Aff4	AF4/FMR2 family, member 4	93736	https://www.ncbi.nlm.nih.gov/gene/?term=93736

Biology Open • Supplementary information

Lrit1	Lrit1	leucine-rich repeat, immunoglobulin-like and transmembrane domains 1	239037	https://www.ncbi.nlm.nih.gov/gene/?term=239037
Csf2rb	Csf2rb	colony stimulating factor 2 receptor, beta, low-affinity (granulocyte-macrophage)	12983	https://www.ncbi.nlm.nih.gov/gene/?term=12983

Biology Open • Supplementary information

Table S13. miRNA modulated by estradiol, progesterone, prolactin and oxytocin in T47D cells.

Estradiol	Progesterone	Prolactin	Oxytocin		
miR-1287-5p	miR-6867-5p	miR-4763-3p	miR-148b-3p	miR-1180-3p	miR-2110
miR-130b-5p	miR-4668-5p	let-7b-5p	let-7a-3p	miR-320b	miR-23b-5p
miR-181a-3p	miR-135b-3p	miR-16-5p	miR-197-3p	miR-320a-3p	miR-27a-3p
miR-181b-5p		miR-181a-5p	miR-1301-3p	miR-106b-3p	miR-296-3p
miR-194-3p		miR-186-5p	miR-941	miR-374a-5p	miR-29b-3p
miR-195-5p		miR-574-5p	miR-340-3p	miR-361-3p	miR-301b-3p
miR-205-5p		200c-3p	miR-1291	miR-99b-5p	miR-30e-3p
miR-23b-3p		miR-151a-3p	miR-449a	miR-429	miR-30e-5p
miR-25-3p		miR-342-5p	miR-126-3p	miR-449c-5p	miR-3129-3p
miR-27b-5p		miR-17-5p	miR-330-3p	miR-21-5p	miR-320c
miR-30b-5p		miR-92b-5p	miR-340-5p	miR-193a-5p	miR-423-3p
miR-30c-2-3p		miR-184	miR-200a-3p	let-7f-5p	miR-4326
miR-3124-5p		let-7d-5p	miR-1226-3p	miR-125a-5p	miR-4664-3p
miR-339-5p			miR-32-5p	miR-1299	miR-486-5p
miR-342-3p			miR-615-3p	miR-1307-3p	miR-501-3p
miR-3615			miR-141-3p	miR-141-5p	miR-505-5p
miR-374b-5p			miR-96-5p	miR-1910-5p	miR-6511a-3p
miR-378a-3p			miR-6880-5p	miR-192-5p	miR-671-3p
miR-455-5p				miR-200a-5p	
miR-4657					
miR-548ad-5p					
miR-548ae-5p					
miR-6787-3p					
miR-7-5p					
miR-92a-1-5p					
miR-99a-5p					

<table>
<tr><th>Estradiol | Progesterone</th><th>Estradiol | Prolactin</th><th>Estradiol | Oxytocin</th><th>Prolactin | Oxytocin</th><th>Estradiol | Prolactin Oxytocin</th></tr>
<tr><td>miR-27a-5p</td><td>miR-125b-5p</td><td>miR-101-3p</td><td>miR-365b-5p</td><td>miR-190b-5p</td></tr>
<tr><td></td><td>miR-193b-5p</td><td>miR-135a-5p</td><td>miR-877-5p</td><td></td></tr>
<tr><td></td><td>miR-30c-5p</td><td>miR-16-2-3p</td><td>miR-26b-5p</td><td></td></tr>
<tr><td></td><td>miR-365a-5p</td><td>miR-378i</td><td>miR-423-5p</td><td></td></tr>
<tr><td></td><td>miR-92b-3p</td><td>miR-92a-3p</td><td>miR-148a-3p</td><td></td></tr>
</table>

Biology Open • Supplementary information
